# *Eat a little and save a little*: A qualitative exploration of acceptability of a potential savings intervention to reduce HIV risk among female sex workers in Western Kenya

**DOI:** 10.1371/journal.pone.0310540

**Published:** 2024-12-19

**Authors:** Sophie Otticha, Jane Moraa, Jacob Onyango, Olivia Okumu, Marylyn Ochillo, Judith Ayallo, Phillip Owiti, Lillian Ouma, Nancy Ounda, Tobias Odwar, Shantana Carol Ogot, Kawango Agot

**Affiliations:** Impact Research and Development Organization, Kisumu, Kenya; United States Agency for International Development (USAID), NIGERIA

## Abstract

**Background:**

The burden of HIV among female sex workers (FSWs) remains higher in sub-Saharan Africa (SSA), with an estimated prevalence of 36.9%. In Kenya, HIV prevalence among FSWs is 29.3% compared to 6.6% among adult women in the general population. Economic disempowerment is a significant driver of HIV among FSWs, specifically manifested in engagement in higher-paying, high risk sex. Saving interventions to improve financial security have the potential to reduce HIV risk among FSWs.

**Methods:**

We conducted 24 focus group discussions (FGD) with each session involving 6–10 respondents. The FGD guide explored saving history and income sources, spending and loan-taking practices as factors associated with saving. Thematic analysis identified themes related to financial burden, loaning, saving and spending, sources of income, HIV risk behaviors in the context of sex work, and acceptability of the proposed saving intervention to reduce HIV risk.

**Results:**

We conducted 24 FGDs with 221 respondents, of whom 19.9% were married and 85.4% reported being heads of households. We identified the following key themes, that FSWs were: open to participating in a saving intervention being proposed to reduce their HIV risk; financially insecure, thus engaging in sexual practices that increase their HIV risk; living beyond their means leading to further financial insecurity; and desiring an intervention that equips them with knowledge and skills on how to balance earning and spending in order to save and how to take and repay loans without increasing their HIV risk.

**Conclusion:**

FSWs in western Kenya were receptive to the proposed savings intervention, believing that it would increase their financial stability and reduce the need to engage in risky sex when faced with emergency situations that require immediate cash.

## Introduction

HIV prevalence among FSWs in the sub-continent is significantly higher than in other geographic regions globally, with an estimated pooled prevalence of 36.9% in SSA compared to 10.9% in Eastern Europe, 6.1% in Latin America and Caribbean, 5.2% in Asia and 1.7% in Middle East and North Africa [1]. In Kenya, HIV prevalence among FSWs is 29.3% compared to 6.6% among adult women in the general population [2, 3]. In Kisumu and Siaya counties in western Kenya where the study was conducted, HIV prevalence among adult women is 17.7% and 19.3%, respectively [4], and women who are engaged in sex work in these settings are at higher HIV risk.

FSWs are a socially and economically marginalized population and studies in diverse settings indicate that financial insecurity undermines their ability to consistently practice safe sex [4–11]. The majority of FSWs live from hand to mouth [5] and have to balance competing financial demands like paying for food, housing, children’s expenses, medical expenses and supporting other dependents [4–7]. FSW’s inability to meet their financial obligations is made worse by the interaction of poverty [4], low levels of education, lack of formal employment skills, shortage of alternative or supplemental job opportunities, and low sex work pay [3, 8, 9]. When faced with financial stress, FSWs tend to engage in sexual behaviors that expose them to HIV risk, including anal sex and unprotected sex with clients of unknown HIV status just to get an income or to attract higher earnings [10].

While numerous HIV prevention initiatives concentrate solely on reducing sexual risk by changing the sexual behaviors of FSWs [11, 12], several studies have suggested a holistic approach to address the health needs of FSWs [3, 9, 10, 13]. Savings and money management are among the strategies that have been proposed to increase FSW’s financial security [5, 14]. Saving groups have the potential to promote individual agency to reduce sexual risk behaviors and foster community empowerment by addressing structural sources of HIV vulnerability and helping FSWs to achieve economic security and make safer sexual decisions [4, 15, 16]. According to a Ugandan study, FSW who invest to start earning money outside of sex work and have more access to finance are more likely to be empowered and have better economic standing, which lowers their risk of STIs and HIV [13]. Few studies that have reported economic empowerment interventions among FSWs that also mitigated against HIV risk, and the few that combined the outcomes of economic empowerment and HIV risk have not been evaluated [8, 17]. Therefore, including economic empowerment in programs involving FSWs can address some structural barriers to reducing HIV risk.

We conducted a formative study to inform the design of a planned intervention–known as *Jitegemee* (rely on yourself)–in which FSWs will be encouraged to save part of their earnings to build a financial reserve which they can call back in desperate situations to avert engaging in risky sex. Specifically, the study aimed to determine if the *Jitegemee* intervention would be acceptable to FSWs in Kisumu and Siaya counties, Kenya; establish the types of economic activities that FWS would engage in, in order to build a financial reserve; and, to identify intervention components to include in the package in order to appeal to FSWs and encourage them to save.

## Methods

### Design

We conducted a mixed methods formative study that included a quantitative survey and focus group discussions (FGD) with FSW in Kisumu and Siaya counties. This paper focuses on the qualitative component of the study, conducted in April and May, 2022. Written informed consent was obtained from each FSW willing to participate in the FGD. We explored how women navigate the financial shocks that may expose them to risky sexual behaviors, what they think about an intervention anchored on saving part of their own money, and how to make adjustments in their lives in order to save towards securing financial stability during sex work. The intervention was presented to the groups using a job aid that was embedded in the FGD guide (Box 1).

**Box 1. A brief description of the *Jitegemee* intervention**

**Moderator to read out loud:** *In the next section I want to get your views on an intervention we are thinking about that may help you or your peers to stay safe from HIV or to plan for life after sex work for those who may be thinking about leaving sex work in the near future*. *The intervention will primarily prepare sex workers to reduce their risk of getting infected with HIV through saving*. *The intervention is known as Jitegemee*, *which means rely or depend on yourself*. *The reason we are calling it Jitegemee is that sex workers who take part in it will use part of their own income to save towards some level of economic independence*. *They will save through MPesa directly into an account opened by the study*. *The saved amount by each sex worker will be available to her to call back in part or in full any time she needs it so that she does not have to engage in unsafe sex because she needs money urgently*. *With the savings*, *sex workers can say ‘No’ to unprotected sex or to certain clients if they want to*, *or to take a short break from sex work if they need to rest*. *This is because they have savings and cannot go hungry*, *for example*, *because they said ‘No’ to unsafe sex or took a break*. *Some sex workers may also want the savings to go towards long-term goals such as investing*, *educating children or even quitting sex work in the future*. *The intervention staff will help those enrolled in Jitegemee to set saving goals and timelines*, *work with them to plan their savings while being able to support their other needs*, *and help them to monitor achievement of the goals and address challenges that come along the way*. *The questions that follow will ask you what you think about such an intervention*, *whether it would work*, *what the intervention package should comprise of (i*.*e*., *components)*, *how we can implement it so it works well*, *and the challenges we may face and how to address them*.

This paper also sheds light on the views of peer educators (PEs) of FSWs obtained when we presented to them preliminary findings to help in interpretation [18, 19]. A PE of FSWs is a sex worker recognized as a leader and a role model by her peers and who conducts sessions with the peers to improve their knowledge, skills, and attitudes, hence reduce their HIV risk [20]. We engaged the PEs in recruitment of study participants and interpretation of study findings.

### Study setting and recruitment

Our field team identified and engaged 14 PEs from hotspots in Kisumu and Siaya counties and tasked them with mobilizing and referring their peers to the study. The 14 PEs were recruited by the field officers before commencement of the study and trained on the protocol, research ethics, and their role in the study as recruiters.

Six trained trilingual (English, Kiswahili and Dholuo) research assistants screened and consented potential respondents in the language of their choice and enrolled those eligible in the study. Eligibility for participation included being ≥18 years old, self-identifying as a sex worker (exchange sex for money, goods, services or favors), residing in or receiving HIV services in Kisumu or Siaya counties, not having participated in the quantitative phase of the study, and willing and competent to give written informed consent. Our eligibility was limited to women aged 18 years and older because adolescent girls below the age of 18 who engage in sex work are considered as sexually exploited minors and not as sex workers [20].

To represent the views of different sub-typologies of FSWs operating in Kenya [21], we engaged those who conduct sex work business at entertainment venues, streets, brothels, homes and beaches. We added to the guidelines two sub-typologies (beach and home) which were relevant to our setting. We considered place of work as a way of classifying FSWs because place designates where FSW practice sex work. Stratification by these sub-categories was relevant to ensure the study purposely enrolled FSW from the different categories in order to make the results applicable to all the FSW population in Kenya’s urban and rural settings [21]. The FGDs were stratified by county (12 FGDs in Kisumu County and 12 FGDs in Siaya county), age (≤30 years and >30 years) and by hotspot location (urban, peri-urban and rural). In each FGD session, we ensured that participants comprised of a mixture of two or more sub-typologies in order to gain insights from all the six sub-typologies in the two counties and to make the results applicable to FSWs elsewhere where similar sub-typologies operate, as well as to those younger or older and those who sell sex in urban, rural or peri-urban locations. All these considerations increased the number of FGDs we needed to conduct.

### Data collection

We conducted 24 FGD with 221 participants. Each focus group comprised of 6–10 FSWs. The FGD topics explored their spending patterns, financial burden, loaning habits, saving habits and thoughts about leaving sex work. The study also explored whether the Jitegemee intervention being proposed would be acceptable and the components that FSWs would wish to see in the intervention package. Each discussion session lasted for approximately 90 minutes and was conducted in participants’ language of choice by trained and experienced tri-lingual qualitative research assistants. Moderators assured confidentiality and no names or identifying information of participants were recorded. The discussion sessions were audio recorded and transcribed verbatim following a transcription protocol [20]. Upon reaching data saturation, as a team we decided that the sample size reached was sufficient to answer our study questions [22]. During the FGD sessions, process notes were taken in English by two skilled research assistants.

### Data analysis

The collaborative process used in analysis involved several steps: the research team first read through selected transcripts and independently generated possible themes [23]. We then met to discuss the themes and to develop codes which were both deductive codes (including categories drawn directly from FGD guides) and inductive codes (topics that emerged during group discussions). We developed a draft codebook for an initial round of coding [24]. Weekly meetings were held to discuss, reiterate and resolve interpretive differences and to build consensus to ensure inter-coder reliability throughout the coding process [24].

We conducted a thematic analysis [24, 25] of the following coded segments: earnings, expenditure, borrowing, living beyond means, savings, HIV risk, acceptability of the intervention, ethical consideration and components for inclusion in the intervention package.

PE were engaged in the dissemination session [19] and taken through a brief overview of the study and key findings of the qualitative component. They were asked for their thoughts about the findings (what they anticipated and what came as a surprise, and why), and why some themes came out repeatedly during their training but only a few times during the FGD sessions (S1 File).

### Ethical consideration

This study was approved by the Maseno University Ethics Review Committee (MSU/DRPI/MUERC/1033/21). All participants gave written informed consent for study participation.

## Results

We conducted 24 FGDs with 221 respondents, of whom 19.9% were married and 85.4% reported being heads of households. The FGDs were conducted by trained and experienced trilingual qualitative Research assistants in Dholuo (n = 13), Swahili (n = 6), and a mix of Dholuo and Swahili (n = 5); none was conducted in English. We identified the following key themes, that FSWs: (1) are financially insecure, (2) live beyond their means (3) are open to joining the proposed *Jitegemee* saving intervention if implemented, and (4) need an intervention that can equip them with knowledge and skills on how to balance earning, spending, saving, taking and repaying loans without increasing their HIV risk.

### Acceptability of the proposed *Jitegemee* saving intervention

Overall, in the narratives, the proposed *Jitegemee* intervention was deemed highly acceptable. Most FSWs agreed that they need to save in order to not have to depend on the male clients.

*“What can make me agree with it (the Jitegemee intervention) is to stop having sex with men. The little money that you save, you open a business with it and you depend on yourself. The things that you want, the time that you want them…. if you want to buy something that interests you, you struggle and save money then you buy it. So you don’t have to depend on someone”.* (FSW below 30 years—rural)

“*I can like it because it gives me self-dependence*. *I do not depend on someone else”* (FSW, below 30 years, urban).

“*Jitegemee would help me on how I can depend on myself without necessarily depending on these men”* (FSW below 30 years—rural)

Some were also willing to join the proposed *Jitegemee* intervention so that they would not have to do sex with people of unknown HIV status for money. A participant felt that with savings, sex workers would protect themselves from the risk of acquiring HIV.

*Like me say…I meet someone and we agree, maybe I don’t know his status well. So you know when we have gone and have done that thing [had sex], maybe I can get infected… If I go and test, it will force me to just leave it (sex work) so that I find a way to help myself with the money I save.* (FSW below 30 years—peri-urban)

Participants provided specific suggestions that would increase acceptability of the proposed *Jitegemee* saving intervention. There was an immense preference for having community sensitizers who are female sex workers to educate their peers about the intervention. According to the participants, those already sensitized would cascade the information to their peers so that more people can be reached with the intervention information from trusted sources. Additionally, sensitization could be done through the social media. Across all discussions, the participants stressed that to avoid stigmatizing sex workers, there is need to sensitize the community at large.

*You see to reach people currently is through the social media. That’s where you can reach them. Another thing is to educate people… it is not a must you have to look for the sex workers to educate them. You can reach ordinary people.* (Younger FSW, below 30 years, rural)

*As for someone like me (a sex worker) I will be an ambassador for the other person*, *I am going to tell her what I have heard here about Jitegemee*. *Jitegemee wants us to at least save*, *I will now explain to her and tell her more*. *If she is somebody whose understanding is good she will hear and then join*. (FSW above 30 years—urban).

To further remove the spotlight away from FSWs and boost acceptability of the proposed *Jitegemee* intervention, confidentiality was strongly discussed by participants who felt that some FSWs do not like to identify as sex workers. For this reason, they felt that the name of the intervention should not identify them as sex workers; rather the name and the identity of the group should be camouflaged. In addition, covert participation in the intervention would help address stigma issues around sex work.

*“They can give it a decent name, so that it’s just known that those are just some ladies but not… those who are doing this kind of work” (sex work).* (FSW above 30 years—urban).

Some participants recommended that the leadership of the proposed intervention should include sex workers.

*You ask them the way they would want to run their organization. Let us say of Jitegemee, then once that organization of Jitegemee has been formed, as sex workers ourselves, we should run it.* (FSW above 30 years—peri-urban).

When asked about the components of the proposed intervention, participants recommended the need for loan provision and reliable saving platform. They suggested saving in banks, because it is safe and they can access loans and interests. Some suggested saving on phones through Mpesa/Mshwari (popular online saving platforms in Kenya)—because many are used to those platforms of saving and their money will be safe because they are in control. A few suggested keeping their savings at home because going to the bank consumes a lot of time. Other suggested platforms for saving to include Savings and Credit Cooperative Organizations and table banking groups.

### Need for training

Importantly, participants emphasized the need to train sex workers on financial literacy in order to boost saving. There was a strong need for knowledge and skills on how to manage earning, spending, saving, taking and repaying loans without exacerbating HIV risk.

*Challenges that make them not to save, is lack of knowledge. You know when you are not taught about what savings do, I cannot save. I will just be spending all the money. For now, through Jitegemee I can start saving because I know that if I can eat a little and save a little it can help me in future when faced with problems. So, lack of knowledge also makes us not to save. If you don’t have anybody who has told you about the importance of saving, then you may not save.* (FSW above 30 years—peri-urban).

Furthermore, provision of other apprenticeship like tailoring, agriculture and hairdressing through the proposed *Jitegemee intervention* would help address financial instability among FSWs hence improve their ability to save frequently.

*Also if you show us some ways that we can do business … that can bring money quickly so that we can save quickly.* (FSW above 30 years—urban).

### Financial insecurity and HIV risk

In a majority of the groups (both young and older FSWs, across urban, peri-urban and rural areas), the discussants explained that they spend their earnings on primary needs (mainly clothes, food, rent for family residence, school fees and other school needs for their children) and secondary needs (rent for the rooms for sex work, makeup, drinks, among other expenses). Their financial constraints lead them to taking loans.

*…even today I came back with 500 shillings (1US$≈120 Kenya Shillings). When I came back the children began dealing with the 500 shillings. They are three children and I gave them 150 shillings for lunch… but didn’t have soap, didn’t have sugar, cooking oil. It is finished. I still don’t have what we will eat for supper.* (FSW, above 30 years, peri-urban).

Some FSWs who are not able to settle their loans reported resorting to having sex with the loan administrators to get their repayment terms relaxed or loans cancelled. A loaning system that has evolved and is becoming increasingly popular, is where hawkers (mostly male from a specific ethnic community in Kenya) sell items such as clothes, shoes, utensils, beddings, and other household goods and also lend money at some interest to their customers who are in financial need. This is illustrated by the conversations below:

*When I get stuck like it reaches the point where everything is stuck (so) I borrow money for rent and school fee from the [ethic community].* (FSW above 30 years—peri-urban).

Below is a discussion with young FSWs who are below 30 years from urban setting.

***Respondent 1:*** *… I have even seen others borrow money from the [ethic community] and if you are not able to pay, you pay back with your body [have sex with the lender]*

**Respondent 2**: Yes, it happens.

**Moderator**: They pay with their own body… now does the [ethic community] approach you?

***Respondent 1***: *No he does not approach you*. *If you are not able to pay it back*, *the [ethic community] have taken that advantage when they are lending*, *they lend to women not men*. *If you are not able to pay he comes and threatens you*. *So if he threatens you now as a sex worker you see it (having sex) as the only way to pay back*. *It is something I have seen happen*.

For some sex workers, when they are not able to pay their loans they opt to increase the number of sex clients so that they get enough money to pay the loans. For those do not have money for basic needs and cannot access financial loans, they reported having no choice but to sell sex even when they do not have the will to do sex work. Due to pressure to make more money, they are sometimes compelled to engage in unprotected sex, work for longer hours or increase the number of clients, so that they can earn more money for domestic use.

*I mean if I have shortage of something in the house and then I find someone who calls me that I may go to a certain location, I can go and have agreement with him and when he tells me that when he has sex with me, he can give me certain amount of money, I may ask him if he has a condom and he may tell me that “It’s not a must that we use a condom” so if I am in a situation whereby I have challenges (need money), I will accept and have sex without a condom and I charge him slightly more than what he ought to have paid. I will accept and we may have sex without a condom.* (FSW below 30 years—urban).

A discussion among FSW below 30 years from a peri-urban setting proceeded as indicated below

***Moderator:*** *Number 3, if income is low and expenditures are high, and you need to increase your income, what will you do as a sex worker?*

**Respondent 5:** Number 5, let us say that you were going for sex work only during the day and resting at night. It will force me to work both day and night. I will do it double.

**Moderator:** You do it double; number 9 do you have something to say? Okay is there anyone who would like to add?

***Respondent 9*:** *I increase partners*.

Our report from the dissemination meeting with PEs of FSWs indicated that when in desperate need of money, FSWs sometimes have sex with clients in places they term as “green lodges” (meaning in the bush or on the grass) which are usually convenient because they do not pay for the venue. In such cases, one would not know the potential sexual risks she is exposed to nor the dangers in the bushes. While this term featured prominently during training of PEs as mobilizers, it did not come out as strongly during data collection. When asked why they thought this happened, PEs reported that sex workers often feel ashamed to report that they engage in *‘green lodge’* sex because of the stigma and connotation attached to it as cheap and demeaning.

Some FSWs felt that savings would help them make safer choices that protect them from risking HIV infection through increasing number of partners, engaging in unprotected sex or in green lodge arrangements. When asked about their thoughts on the proposed *Jitegemee* saving intervention, a respondent said:

*“What comes into my mind is…this means that when you save, you might not experience the worst at the time that you are experiencing difficulties, whenever you are experiencing any difficulty you would think about the money you have somewhere (saving) that can help you out. But at the time that you don’t have anything and left the house with nothing, you feel that, that action (unsafe sex) is wrong but still, I have to do that because I have nothing”.* (FSW, above 30 years—urban).

Some participants reported that they engage in anal sex which yields a lot of earnings and would have enough to save. Below is what a FSW aged above 30 years from an urban setting said:

***Respondent:*** *Okay, and if I add, you know these things have two prices. In front (vaginal) has its price, behind (anal) has its price. So the day I will see that I have an (racial group), (racial group members) don’t like to eat fish on one side (have vaginal sex only). He eats the front of the fish (vaginal), he eats the back of the fish (anal). That day just know you have killed kenge (kenge is swahili for alligator, implying you have hit a jackpot). That one if you just agree to open the boot (‘car trunk’ = anus), ah you are just okay.*

**Moderator:** Opening the boot is going where?

**All Respondents:** Behind.

**Respondent 1:** Eating from behind (one participant laughing). Having sex in the behind

**Moderator:** Having anal sex?

**Respondent 1:** It has money. It has money.

### Female sex workers live beyond their means

Some of the FSWs reported that they live beyond their means by spending their earning on expensive purchases, many times exceeding their income. Besides the pressure to address various basic needs in their lives, such as paying school fees and rent, and buying food and clothing for their children, they also spend on expensive clothes, hair styles and beauty products to make them more competitive. Their coping strategies when they are not able to afford these expenses include having intimate relationship with sugar daddies (older men who are believed to have money and pay more for sex) or reducing their budget/ expenses.

*Sometimes the body oil that you use cost six hundred shillings, so you decide to buy body oil that costs three hundred shillings so that you balance (with income) and if you always plait your hair with three thousand shillings it will force you to spend two thousand or one thousand to plait your hair. (FSW below 30 years, Peri Urban)*

*If you don’t reduce your budget (live within your means)*, *it will force you to look for a sugar daddy to help you… fill the gap like number seven has said*… . *so it doesn’t have to be one but two or three sugar daddies because they are also looking for young girl’s not older women so you will look for one or two or more than two*. *(FSW below 30 years*, *Peri Urban)*

Others reported resorting to taking loans from banks, men, shops, close friends, *chama* (table banking groups) and higher education loan board for their children. Often, they are not able to pay their loans from their earnings and they resort to engaging in behaviors that predispose them to HIV infection, in order to generate money to repay the loans.

*I use more than I earn and I even go into debts, even recently I went into debts, I took some Mshwari (mobile bank) loan—you have to take.* (FSW below 30 years—peri-urban).

*The life I live is more expensive than my earnings because I am relied upon at (my rural) home*. *I am the bread winner of my children this side*. *That is why I like saving in KCB because sometimes I can request for a loan and they give me*. (FSW above 30 years—peri-urban).

## Discussion

Our study found high acceptability of the proposed *Jitegemee* intervention by FSWs. Peer involvement in the implementation and management of the intervention [16], confidentiality assurance, financial literacy coupled with management of loans, were recommended as important components of the proposed intervention that would improve its success. Similar to our study, there are studies that have recommended financial literacy as an essential element of effective savings-led or micro-lending programs aimed at FSW [26, 27].

As documented in other studies, stigma has played a significant role in inhibiting access to HIV prevention programs by FSWs [28, 29], and for optimum uptake of HIV interventions like the *Jitegemee* intervention, participants called for guaranteed confidentiality, meaningful involvement of their peers in leadership roles, and presenting the *Jitegemee* intervention without identifying it only with FSWs in order to not lock out those who practice transactional sex without acknowledging that they are sex workers. According to other literature, educating girls reduces their risk of early marriage and entry into sex work [30], therefore to achieve a holistic approach in reducing HIV risk, intervention programs should consider promoting education of girls to avoid entry into sex work.

Financial saving interventions have the potential to help FSWs reduce HIV risk by providing them with a financial safety net, thereby reducing their reliance on sex work [3, 26, 27, 31]. As established in various studies, reliance on sex work poses a serious risk for HIV transmission in the sense that many sex workers engage in high-risk activities including unprotected sexual intercourse, sex when under the influence of drugs/substances, sex with clients of unknown HIV status, anal sex, having sex in hazardous locations such as “green lodge” and other high-risk behaviors, to be able to get money to support themselves and their families financially [32]. Structural measures that address the financial vulnerabilities of FSWs have been shown to lower the risk of acquiring HIV [33]. Such measures include livelihood/microfinance programs or career training courses that can offer alternative sources of income’.

A study in India found that FSWs who took part in a financial literacy program and were given savings accounts were less likely to engage in risky sexual activities than those who did not; furthermore, those who saved money were more likely to engage in protected sex [34]. However, most of the structural interventions do not translate into meaningful or long-lasting economic empowerment of FSWs after the programs wind up [35, 36]. As a result, FSWs who are faced with emergency situations such as lack of food, sickness, school fees, death, or transport and have no financial reserve to fall back on are likely to resort to practices that increase their HIV risk. Our study documents how FSWs engage in HIV risk activities in order to address an emergent financial difficulty and to have a high standard of living. For instance, they work longer hours, increase the number of sex clients, or engage in unprotected vaginal or anal sex in order to increase their income and to manage their financial burdens or high cost of living [14, 32–36].

Studies have shown that poor saving habits among FSW may be due to unplanned expenditure [34, 37] and impulsive buying [38]. Participants in our study reported that they live beyond their means by spending a sizable proportion of their earnings on expensive personal and household items that they do not necessarily need. They emphasized the need to incorporate financial literacy and management as components of the proposed Jitegemee intervention. Training FSW on how to save and exposing them to financial literacy and management will ensure their buy-in and sustained economic empowerment. As observed by other scholars, FSW who are empowered economically can make important decisions that determine their lives, including their choice of work and their capacity to invest in alternative sources of income [34, 39]. Additionally, we hold the view that FSW saving part of their earnings–which is a core feature of Jitegemee–will make the intervention homegrown therefore more likely to be sustained compared to economic empowerment interventions that involve provision of incentives, start-up loans, or other forms of external support [4, 15, 40, 41].

Our study had limitations. The median age of our sample is older than that of FSWs in many settings, therefore these results may not be generalizable to younger FSWs in Kenya. The qualitative nature of the study restricts our ability to generalize our findings. However, this is a formative work that will inform a large scale implementation study that will test the impact of the *Jitegemee* intervention in reducing HIV risk behaviors. In addition, our sample was a purposive selection of FSWs population and from two of 47 counties in Kenya, therefore factors and practices unique to FSWs in the two counties may make our findings inapplicable to FSWs in other counties. Nevertheless, the inclusion of FSWs from different sub-typologies in our study minimized this limitation since the FSWs from other counties in Kenya would find their views relatable.

## Conclusion

Participants accepted the proposed *Jitegemee* intervention, with the majority feeling that the intervention would increase their financial stability and reduce the need to engage in risky sexual behaviors for income. Unfortunately, some participants would resort to riskier behaviors in order to raise money to save. While our findings suggest that the financial saving interventions have potential to improve the health of FSWs by reducing HIV risk exposure, care should be taken to ensure that the strategies to raise money to save do not increase their HIV risk. This study will provide critical insights for the development and implementation of the forthcoming intervention, utilizing the knowledge gained about its design and delivery mechanisms.

## Supporting information

S1 File Transcripts and dissemination notes. (ZIP)
