# Supplementary material for: Eat a little and save a little: A qualitative exploration of acceptability of a potential savings intervention to reduce HIV risk among female sex workers in Western Kenya
Source: PLoS One. 2024 Dec 19;19(12):e0310540. doi: 10.1371/journal.pone.0310540 (PMC11658496; doi:10.1371/journal.pone.0310540)
Supplement: S1 File — (ZIP) [file pone.0310540.s001.zip › Jitegemee Transcripts and Dissemination Notes for Journal/FGD B.docx]

**FGD ID: FGD_B**

**DATE OF FGD: 25/APR/2022**

**INTERVIEWER CODE: KSM003**

**NOTE TAKER: KSM006**

**TRANSCRIBER CODE: KSM006**

**VENUE: TYP**

**START TIME: 1213 HOURS**

**END TIME: 1504 HOURS**

**CATEGORY: BELOW 30, URBAN.**

**I: This is Jitegemee Study FGD conducted at Impact office the date today is 25/APR/2022 the moderator is [mentions name] and the note taker is [Mentions name]. The FGD number is Kisumu 0… Kisumu PB01 and the time is 12.13 and the participants are 07 in number. Okay now thank you so much for your time. We are going to start. Now from my brief description of Jitegemee, what is your initial reaction? The way I have explained what Jitegemee is you can just tell us what you feel about it, number 04.**

PB04: I speaking as number 04 Jitegemee is good because it cares about the lives of us the sex workers, because it explains how we can protect ourselves from getting diseases such as HIV and how we can better ourselves when we leave this work of sex work. So Jitegemee will help us a lot.

**I: Who else has something else?**

PB02: Okay I as number 02 according as the way my colleague here number 04 has said I can add by saying Jitegemee helps me personally, let me say personally let me say according to myself that when I become self-reliant no one can come to me and force come to do something that I am not willing of. Yes. No forcement there (NAO: laughter)

**I: Now you have talked that nobody can force you, force you to do what?**

PB02: Like I can say, me as a sex worker, being that am a sex worker, I am doing that work that work of sex … that job sex working job, no one can come and force me that I want to engage in sex with you or I want to have sex with you by force because I am doing that work.

**I: Any other with something to add?**

PB02: As number 03 I can say this Study of Jitegemee as a sex worker I did not know… it has at least opened by mind and I see that it’s possible to leave it and start doing something else apart from that.

**I: Another person, number 01.**

PB01: As number one Jitegemee is promising to us as sex workers because it is giving us a] economical value and it gives us an open mind of starting to think of doing something other than sex work and its giving us that platform of saving to start maybe a business apart from our normal work routine of sex work.

**I: Any other reaction.**

PB05: As number 05 I can say Jitegemee can help a lot, because once… one, we have been told about the Jitegemee it can make one see a way forward in life. You’ll realize this is not the only way you can get cash so you can decide to do other thing maybe start a business or do any other work apart from using your body to get cash.

**I: Any other person with any other reaction?**

PB07: I as number 07 I see anything that Jitegemee is going to teach us can help any other person out there who is going through the same things we are going through.

**I: Am I leaving anybody out? So, we will continue. What are the typical expenses that female sex workers make, and approximately how much do they cost? So first we shall discuss about the expenses then we shall discuss how much they cost. Do we all understand expenses, the daily expenses that we normally incur?**

P: Yes.

**I: So we shall talk about the various daily expenses going round until we finish then we shall talk about the cost. Who will start us off. Like we want to know your daily expenses and the estimated cost for each.**

PB04: As number 04 my daily expenses in cash is around 700 shillings.

PB02: As number 02 I can say my daily expense in cash it takes a little bit high because I have a kid. My daughter also wants to…At least you know a young child would want to be bought sweets and such, so I can say I can put it at a 1000/= per day.

PB03: As number 03 mine can also be around 900/=

**I: Another person?**

PB05: Number 05, my daily expenses are around 800/=

PB06: number 06, my daily expenses are around 1200/=

PB07: Number 07, my daily expense is round 500/=

PB01: Number one approximately… between approximately 500-700/=.

**I: Okay, now your daily expenses as you mentioned, can you break it down for us to see, this much goes into this and this much goes into this. Who is ready to start?**

PB07: Number 07, I spend 100/= to get diapers, 200/= I put into merry-go-round you put 200/= per day and get your cash after 5 days, the rest I put into airtime, because the only way I save is through the Chama.

**I: Another?**

PB05: Number 05, so like I have two kids both going to school. So, I have to give them, maybe pocket money when they are going to school and transport I also move from home to work I also use transport. Say lunch breakfast for my kids, I also need airtime, yeah.

**I: I want you to estimate the cost, like transport, lunch, pocket money and even sorry lunch. Can you just break the amounts?**

PB05: I use 200/= for my own transport and I use for my kid’s transport. 200/= in mine 100/= in my kids that is 300/= and breakfast in the morning I have to give my children a good breakfast maybe I can use about 200/= because I need milk and bread that is 500/=, supper… lunch is also there I have to buy them something to eat lunch can be out. And also supper… there is also supper I will need to buy something presentable for my kids.

**I: In total how much do you use for lunch and supper?**

PB05: For lunch, for my kids sometimes I use 70bob each that is around 150 there and for supper … supper I sometimes use 200.

**I: Another person?**

NT: Airtime?

PB05: Airtime I just use 50bob.

**I: Okay. Another person to give us a breakdown of her expenses?**

PB01: As number 01, I live with my brother, his transport to school 100 shillings, his lunch 100shillings, my transport 200 and then… because we leave the house early in the morning so we only cook supper sometimes we use between 100-200 depending on what we buy. And Airtime 100 shillings.

**I: Okay another person, daily expense?**

PB04: Number 04, in the morning I use 150/= for breakfast and 50bob the child’s fare, lunch 100/= that is 300/= my lunch also 100/=, that is 400/= in the evening supper 150/= then 100 my fare for the night and 50bob airtime.

**I: Who is has remained?**

PB03: As number 03, I see in the morning I may use like 200 bob for breakfast. Then because I have a child and the baby eats at around eleven we may use another 50/= this 250/=, when lunch reaches I may buy food because I am in the house during the day at around 200/= that is 450/= then his diapers at around 100 bob for the whole day, and that is about 550/= then supper. Supper may take about 200/= and then my transport 100/= for the night.

**I: Alright. Who is remaining, is there anybody remaining, any other who has not given us breakdown, number?**

PB02: Number 02, yes as number 02, breakfast 200/= for milk to buy milk and bread, then buying charcoal for cooking tea. Transport for the child to the daycare, I use transport of 50bob let’s say 100bob because it is 50bob going and 50 coming back. The diaper for 100 bob I have to buy some to last the child while there and then that is 300/=… 400, then the child’s lunch, it’s bought there not carried from home it bought at 50bob that is 450/= and then when the child comes back, she will need the snacking like the biscuits and sweets that costs about 50bob. Then Supper, it depends, sometimes I am not… I feel like treating myself so I can by some meat or fish 300/= then the night diaper is also there.

**I: Alright, no one has remained now. So, we want look at the expenses that we do once and the total cost. That which you do only once in a week. What are these expenses?**

PB07: If I spend 100bob to buy diapers it goes for 6days because he doesn’t use the diapers during the day. He uses it only at night.

**I: Another person?**

PB05: [inaudible] for me if I buy something like charcoal, I buy charcoal costing 200/= and I will use through to the end of the week.

**I: That is number 05 speaking, another person?**

PB01: Number 01 something like cooking oil or maize meal I just buy once that lasts through the week.

PB03: I can close my eyes and buy milk (meaning sacrificing) one carton box that will take us through the week.

**I: Number 02?**

PB02: As number 02 what I but once a week is charcoal and may be say water, some small stuff like water, sugar, yes.

**I: Now we want expenses that you do only once per week, you only buy it once.**

PB02: I only buy once… its charcoal and sugar.

**I: And sugar, and how much does it cost?**

PB02: 500/=

**I: Number 04**

PB04: As number 04 I usually buy 4kg of maize meal, but now calculating that cost is really hard because the price of maize meal has really gone up, I don’t know may be now it’s costing 13… 135/=

**P: 140/=**

PB04: Now 135/= by the 4kgs that’s around 500/= per week. Usually when I buy the 4kg it lasts me a week.

**I: Is there anybody who has not spoken?**

PB06: As number 06 let’s say I buy cooking oil that takes me through one week.

**I: Now thank you, still we are still talking about the expenses. Now we want to talk about the expenses that is done once or twice per month.**

PB06: As number 06 I can talk about house rent, I pay once a month.

**I: Who else?**

PB04: As number 04, gas I use 1500/= to refill and it lasts me a month.

**I: Another person?**

PB05: As number 04, my hair style (laughter- the participant has kept her hair short)

**I: How much?**

PB05: Just 100bob (laughter continues).

**I: One hundred bob**

PB02: As number 02 me mine is also hair style. Once in a month at 600 bob, it depends with the styling there are times instead of 600/= it costs up to 1200/=. It really depends on how the month is, if I decide this month I want to have this hair style another month this other hair style and another, another hair style. So it depends how the month is going, how you get your income and the styling you want to make.

PB07: As number 07 even for me is hair style, rent, gas yeah. So, for gas let’s say 1500/=, hair 1000/= even if it’s what type of hairstyle if it comes I just let it go. And then rent, then that number gets stuck there. So you have to get it.

**I: How much is the rent?**

PB07: its seven

**I: Seven what?**

PB07: K

**I: Seven**

PB07: Seven K (NAO: 7000/=)

**I: Number 01?**

PB01: As number one, is gas because mostly that is something that doesn’t regular refilling. Detergents, things like soap, omo because I do not do laundry every day so once I buy let’s say 1kg it’s enough for us for the whole month.

**I: Number 03 has not spoken.**

PB03: As number 03 I can say I agree with all that has been said by others, then what we are forgetting is buying pads. Pads I buy once a month yeah.

**I: For how much money?**

PB03: The one costing 250/=.

**I: Number 06 you said you pay rent, but you did not share the amount.**

PB06: 4500/=

**I: 4500. Okay, expenses done once a month, like how frequently for example the one done once per year once per school term. (Silence) Have we understood?**

Group: Yes.

PB05: The one for once per school term, that is for my kids. I must buy the uniforms maybe if they are torn. Then the socks, you cannot trust the small children with socks. It will force one to buy several pairs. So those are the things I spend on once per term.

**I: Now you say you but several socks, how many in particular?**

PB05: Not so many but let’s say 2 for the girls. Yeah. Two each, yeah to per each girl.

PB04: Number 04 if I buy shoes for the child it lasts for a term for 2000/=.

**I: Number 05 you said you buy socks is it two pairs or one pair?**

PB05: Two pairs.

**I: Two pairs for each so it’s four.**

PB05: Yeah

**I: Okay, at how much?**

PB05: At 80bob.

**I: 80bob. Another person, uniform how much the total cost?**

PB05: Uniforms, I don’t buy.

**I: You don’t buy.**

PB05: Unless may be a year but uniform unless it’s per year.

**I: Oh okay, per year. Who else?**

PB02: As number 02 per term I pay the daycare fee of 21… 2100/= per month that is per month. Uniform I put on uniforms, it’s a day care but it operates like a school, the age limit is below the school going so that is why they are placed in a day care but they have to put on uniform. You buy socks, shoes… for shoes I can say it’s once a year because he cannot out-grow shoes termly. But socks because you get two pairs and you wash them every day so it may wear out a lot so you have to… you can change for him but I can say it will depend with my assessment on the condition of the socks.

**I: Now tell us the total cost of the socks, uniform and the shoes.**

PB02: Shoes I took the second hand ones at 500/=, socks were 100/= a pair so 2 pairs are 200/= uniform… uniform I can’t say per term because he can’t outgrow all of them so uniform I can say it’s per year.

**I: And the cost?**

PB02: uniform cost 700/= a pair.

**I: A pair**

PB02: I got 2pairs

**I: Is there anybody who has remained? We have all shared? Okay, now we are moving on to the next question. We want to know the general spending profile of the female sex workers. Where do most female sex workers get money to spend? Where do you get money for spending?**

PB07: As number 07 aside from sex working I am a freelancer I do work online. I write articles. You may find an articles pays you about 5dollars. Sometimes you may be given the length of words required like about 1500 they will pay you about 100dollars per month. If you convert 100dollars to Kenyan shillings that is 10k. So that is where you can get some to have a little fun with.

PB06: As number 06 apart from sex working I do promotions. I would say it also depends, the commission you earn at the supermarket is about 1200/=. Promotions for alcohol you can be paid 1800-2k or about 2500/=. Then I can work in fast foods there you are paid per day between 300/=-400/= and other marupurupu. [Incentives]

**I: Marupurupu [incentives] from where?**

P: Tips.

**I: Oh tips, okay. Another person?**

PB05: Number 05 apart from sex working, I work in a club where by I earn 350/= per day plus the tips.

PB03: As number 03 apart from this job I sell second hand for someone who pays 200/= bob day.

PB01: As number 01 apart from sex work and promotions depending on when they come I normally do voluntary work cleaning an office. Just early in the morning around 8:00am I clean by 10:00amI leave. So 5000/= per month because I am not there fully.

PB02: As number 02, apart from sex work. I decided to open an Mpesa where I can spend my time at day. An Mpesa I can’t say the amount I am getting daily because Mpesa you just receive the commission once at end month.

**I: So how much commission… [interrupted by the participant]**

PB02: The commission is depending on how you have worked.

**I: But roughly how much do get in a month?**

PB02: 6000/= 7000/= there so it depends. If you have worked really well you can make up to 10000/= per month. Yes.

**I: Number 04 I want to hear your voice.**

PB04: As number 04, apart from that sex work I sell clothes.

**I: Alright, so mostly you have talked of, apart from sex work there is another source of income, isn’t?**

P: Yes.

**I: Now I want you to tell me from this sex work, how much money you make in a month. (Everyone speaking at the same time.)**

PB04: It depends with the work.

**I: One at a time, one at a time please.**

PB04: As number 04, that work… that work is difficult at the moment. The customers have no money they are taking children back to school. Sometimes you can go and come back empty handed sometimes you get… what you get by the morning is 500/=.

**I: Another person. Now this is 500/= daily or 500/= for the whole month?**

PB04: No, that is daily.

**I: Now approximately you can make how much in a month from sex work?**

PB04: Per month?

**I: Yes.**

PB04: It could be… 4000/=

**I: 4000 another person?**

PB06: As number 06 I can say when the business is good I mean when it booms well you can get even 20000/= to help you in somewhere. (Laughter)

**I: Just continue.**

PB06: It really depends sometimes you can get that 20000/= in one day another day you do not get anything and another you get 30….

**I: Now…**

PB06: Then the business goes down. Yes. You stay in the house planning other things.

**I: You said when the business booms, how does it boom?**

PB06: (Laughter) let’s say when the clients are many.

**I: Okay. Number 07 do you have something to say.**

PB07: Personally, I have specific people so how we agreed that’s what you stick to. So if we had agreed its 10k then you stick to the number or I do not show up.

**I: Okay so for you monthly you can make about how much?**

PB07: Around 30000/=

**I: Around 30. Okay, number 05.**

PB05: As for me end month is usually the good time. Yeah end month yeah, me I have my people not everybody, my people, potential people. So like 6000/= maybe in a day but in a month I can get around 40000/=.

**I: Number 01.**

PB01: Business is business. It has its ups and downs so there those days that you will be lucky. There days you can hit the wall even for the whole month. You can hit the wall seriously. But I believe as sex workers most of the time we have our target customers apart from the random customers who will just show up or they might not show up. So most of the time our hopes are on our target customers. There is this customer which you know when they hit [come] it it’s a good business, it’s a good business. Unlike the random customers that maybe you see them once and they are like they even want to give you even 200 shillings because they feel like they shouldn’t even be paying you.

**I: Now roughly you get how much money in a month?**

**Note-taker: Like your target, you mentioned about target, your target when you meet them how much do they give you?**

PB01: I wouldn’t say it’s consistent because the in terms of target they are people who do not live in town. Or rather even if they live in town they do not show up daily. It depends sometimes 10000/=…lets [inaudible] from 10 onwards depending on… how the market is.

**I: Alright, you also said you can hit a wall, what does that mean?**

PB01: Hitting the wall means that there are those days that go dry no client. Imagine leaving the house for business and going back without a single coin. Yeah, from your business, that is what is hitting the wall.

**I: Alright, there is someone who has remained right? Number 03.**

PB03: As number 03 what I can say before Corona things were good. I could get roughly in a month 35k and above, before Corona. But when corona came people were scared, people were so fearful of their lives. Now these days maybe around 15k.

**I: 15k, okay number 02**

PB02: As number 02 I can say roughly I can say just 20000/=. 20000/= yeah there. Because sometimes you do not go, sometimes just like you have been told, you hit the wall.

**I: Number 04.**

PB04: Oh, I share again, I had already shared.

**I: Oh sorry if you had shared then do not repeat. Okay,**

**Note-taker: There is ah… number 07 you mentioned about specific. Does it mean… Specific here means what, so that we get it clear.**

PB07: I do not go with random clients, I have people who if we had agreed prior then I just show up do the thing [engage in sex] and you give me my money.

**I: Alright, what are the typical reasons why female sex workers spend on what they do? Like what makes you spend the money that you get.**

PB01: Sorry may you come up again.

**I: What are the typical reasons why female sex workers spend on what they do?**

PB01: As in spend on our businesses.

**I: Yes.**

PB01: As number one I would say as sex workers we are also trying to get an income. And… this is business and we are trying to present a good package. To get a good profit get ah… good cash at the end of the day, you find you have to spend a lot on yourself to make a good package like on dress code, make up such like.

PB07: Well, as number 07 I would like to add on her point, when you know the kind of client, kind of customer you are dealing with you have to invest in yourself you look presentable because if the money he is giving you is a huge amount you ought to be worth the money.

**I: Another one?**

PB03: As number 03 there are clients who are also… there is something they want. There is a certain way they want you to show up. He can call you somewhere, somewhere fancy then there is a certain way he wants you to dress. So you have to dress up well.

**I: So how does he want you to dress up?**

PB03: As in you dress just great, because maybe his cash is also great.

**I: So I will ask the question again in Swahili, what are the typical reasons that make the female sex workers to spend on what they spend on? (Short silence)**

**Note-taker: One, is the presentation, the way you said. You guys use your money to look presentable so that the way you have packaged yourself you can attract better. From how I got, to attract better clients or well-paying clients. That is one way of spending. And what about the other, the other spending habits that you have.**

**PB02**: As number 02 I can say the other spending like I said you see you cannot just live because you are a sexual worker. May be you do not other people to know that you are a sex worker. You have to look smart you want to maintain a certain lifestyle you are not just there. If somebody doesn’t know what your occupation is but you can present yourself as, let me say you liken yourself as someone working in a bank or a health care provider. You want to be, how can I put this…I mean if one was to ask you, let me say you do not want to say what kind of job you do but how you dress up if one was to ask you “what kind of job are you doing” you can say “I am a health care provider”.

PB04: As number 04 that money of mine from that work of mine. I can use it to… as someone who sells clothes I can beef up my stock and sell so that I am busy and no one can tell what kind of job I am doing that is making my business do well. So I use it there as well to add stock.

**Note-taker: Again tell us “I use this amount to add stock…”**

**I: Like you who has said you add stock, how much do you use.**

PB04: From my work of sex work?

**I: Yes.**

PB04: Like on Saturdays I open bails. You may find I got about 5000 yes when I remove from my sex work.

**I: Okay, number 02 said she wants to dress so that when one looks at her they see a health care provider. How much do you spend for you to be seen as a doctor or someone who works at the bank.**

PB02: Let me say dressing code, I know how someone like a doctor, someone like…. Let me say you dress up in an elegant manner. You can’t tell me that the attire you are in is for 50bob or 100bob you will go for an attire that cost at least 1000 maybe it’s a long trouser 1500 approximately. The one that show off, it shows off. If it’s a dress, its dress that when one looks it draws attention.

**I: Draws, attention, now if it draws attention how much can it cost? Approximately.**

PB02: approximately 1500/=.

**I: So, we have spoken about the… there are personal expenses and for others, right?**

Group: Yes.

**I: Now let us now talk about… we have talked about self now what about the other expenses?**

PB01: [inaudible]

**I: Yes, those ones.**

PB01: As number 01, part of my money I save because I have my brother in college so yeah. Of course you cannot rely on help sometimes he gets sometimes he doesn’t. So for the Semesters he does not receive then we pay and then of course house needs, basic.

**I: How much do you pay in a semester?**

PB01: 45k

**I: 45k and housing?**

PB01: housing… I do shopping for 2k, depending on what we want and what we are lacking.

**I: Another person? [Inaudible] we are talking about other expenses we have already talked about self, right?**

Group: Yes.

**I: So, what are the other expenses.**

PB02: As for number 02 as for me I said I have a kid. I must buy the kid clothes, shoes and the household things you may want to buy. You may see something… “ei! I have seen that glass plate it has attracted me I want to get it.”

**I: Now how much money can you use approximately?**

PB02: I can use… I think there I cannot approximate because it depends with how much the thing costs, you know one can’t say “I am going to but this its 500/=.” At the moment the cost of stuff has gone up. You may go with 500/= and end up getting it costing more than or less than. And sometimes you want more stuff, now in that instance I can’t approximate.

**I: Roughly is the same as approximately, you can’t say that I will use 100, 000/= but when you think through how much can you use?**

PB02: When I think through maybe if I decide to do shopping I may carry with me 5000/=. I go to do shopping worth 5000/=.

**I: Okay, another person.**

PB05: As number 05. House shopping, I always target when I am shopping not to be more than 5000. And also my girls I have to buy them clothes, I buy them shoes and you know girl’s clothes are obviously cheap so there I can use about 2k to buy them clothes [inaudible] but once in a while I just like good thing, something expensive like I can buy a cute girl dress maybe worth 700. You have to be smart about it so there are those I spend 700 in a certain instance so that they look smart.

**I: Another person.**

PB07: Okay number 07, since once you are a sex worker even though you may have your daily clients, you do not depend on them completely. So the online job is what pays me rent so I must do because sometimes I have work for the entire day. So, that will pay the rent, so what I get from these other people I will spend about 2k for my clothes and the baby, I do my mother shopping for about 3000, my sister is in high school so I send her money most of the time about 1500.

**I: Who is being left out?**

PB04: Oh me?

**I: Yes**

PB04: [inaudible] from that money I also save. I want to buy a plot. So I usually save every day 500.

**I: Is there someone who has not shared. No, there is someone doing like this and she is the one who has not talked. Number 03**

PB03: As number 03 for all the other expenses I think I can roughly use about 5000 per month.

**I: Okay, now we want to talk about saving profile of female sex workers. Now do female sex workers typically save?**

PB06: As number 06 I can say I have my merry go rounds which I can say per day I put about 500. It depends, I save in my account as well like I can put a target of saving 5000 every month.

PB05: As number 05 like we have a family Chama, and you must pay 2000 to the member that is being visited. That cash also is where I can say my other cash is in.

PB02: As for number 02 I say yes we save. Because for me I save like… I do phone saving and I make sure I put there 500 per day. I make sure… my saving is not just for… it is just for emergency. There are times you may find you are sick and there is no money coming in. so the savings help you.

**I: Number 04?**

PB04: Yes.

**I: Do you typically save?**

PB04: as number 04, mh… I do not know if this is what is called saving or what is it called.

**I: Just say we shall see.**

PB04: It’s a Chama that we contribute everyday 200 shillings. And a person is given every month. So I don’t know how that is called. Because if its having an account I don’t.

**I: But this Chama one?**

PB04: I give 200/= daily

**I: Daily, then how much money do get when your turn of receiving reaches.**

PB04: When my turn reaches I get 8000/=.

**I: Who has not shared?**

PB07: Number 07. I am also in a Chama. We give out 200 daily and get your money after 5days and we are 15 in number. Because I can’t lie personally I can’t save on the phone because I will buy credit when it’s over or a will see something that I like and quickly I will remember I have cash in my phone and use Mpesa to purchase it and start regretting later. So I do not save up on the phone.

**I: But do you have savings?**

PB07: Yes.

**I: Number 01, do you save?**

PB01: I have a bank account and I also have my phone the KCB locked account. Yeah.

**I: Who has remained?**

PB03: as number 03, I the past I used to save easy, but nowadays, I save and use save and use. Whenever challenge comes you get all the money out and use.

**I: So currently you have no savings.**

PB03: Currently I do not have any.

**I: Okay, so you have said you save, how frequently, some have told me daily some monthly is that all, or do we have any weekly?**

PB07: Mine is weekly because its after 5 days you are given the money.

**I: Now how much can you approximately save in a week. As number 04 how much money can you set aside in a week.**

PB04: In a week, mh… 800/=

**I: Number 02**

PB02: 2500.I: Number 03

PB03: 1000/=

**I: Number 01**

PB01: From 1000 upwards depending with how much you get

**I: Number 05**

PB05: 200/=

I: 200 number 06.

PB06: 1000/=

**I: Number 07**

PB07: 500/=

**I: 500, okay are there specific characteristic of women who save? Are their certain characteristics of those who do sex work which if you look at you can conclude this one saves? Are there specific characteristics.**

Group: Yes

**I: Are there?**

Unidentified: there is.

**I: Okay tell me.**

PB01: (Laughter) you know when save you save with your own target so it motivates you. It gives you… it makes you look more empowered as in it makes you look in a manner that a client cannot despise you, you see there is this client whom you meet and he is like “I want you for the whole night for 200bob” (laughter). It also makes you not to lose your worth because at the end of the day, as much as you have a target to meet that you do not lose your worth in the process. Surely someone spends the whole night with you and gives 200 shillings only. That is like your transport to and fro. So what have you made?

Group: Nothing

PB01: Nothing, yes. (Laughter)

**I: Okay, someone else?**

PB04: As number 04, I don’t know if I got the question wrong… I understood it as How will you know by seeing that this person can save, or isn’t so?

**I: There are characteristics, when you look at them you can say this can save or this one cannot save, that is what we want to know if there are characteristics.**

04: yeah, there is, because there at the work place where we are you’ll find the kind of clients she goes with you’ll be like “no-no” [laughter] she may even be going to earn a 50bob and obviously 50 bob cannot be saved maybe that can only be fare. So when you look at such a person you can tell, this one cannot save a thing, from the clients she will go with, how she eats-there are things that are sold there at the streets. After a short while she has bought samosa and you can just tell such a person cannot save. (Laughter)

PB06: As number 06 I can say women do talk. Someone can start talking about how she does not save. She can ask her colleague “do you save” the other will tell her, “yes, I save”. So they will be in a discussion and she can tell the colleague “I do not save any money”. So automatically you’ll come to know that this one does not save any money. When she gets money she spends everything.

PB01: To add on to that, women like to trick each other. There is a person who may tell you that they are not saving but in real life they are saving. You know someone who saves you’ll even see their attitude. There is a certain attitude, there is a way I can share with you, “we are in a particular Chama would you be interested?”, “no” and there is some other person you’ll tell “we are in a particular Chama would you be interested?” and she ‘ll be like “how much money do you contribute”. You know, you see that attitude of someone wanting to save

Group: Yes.

**I: So let me ask this question again, are there specific characteristics of female sex workers who save?**

PB05: For me in my view for these women who are saving they are those when leaving work, they do not go to shopping. There are some people when they get money they go for shopping first and there is another they think twice, they first save they has something of their own. So you’ll see it from here, this person I heard they got such and such a client and it’s a client with money, then what next. What is her first stop after leaving the client, did she leave for shopping or for the bank. That is how you’ll know the characteristics of those who save.

PB07: Okay number 07, personally I think you can’t judge a person by just looking at them. Whether they save or not you can’t tell. Unless you are sure. As she said she may leave the clients place headed to shopping but maybe what she went to get are stuff she needed at that particular time. Like she had a need for. And there is this other who will get and won’t use it because maybe that money she is saving for something else somewhere. So you can’t tell. For me I can’t tell.

PB05: You know you can tell if anytime she gets cash she goes this one this particular one does not save.

**I: Another person? There is none. And now those with these particular characteristics who are they? Who are they, we have different places where these kind of people are found. We have street, we have brothel we have entertainment, we have beach. So different places. So those with these kind of characteristics of saving where are they mostly found?**

PB05: As number 05 I think they are found all over. It depends with your needs, you have a child somewhere you’ll be forced to save for that child. You’ll not say “because I operate from the street I cannot save I have left it for the beach ones” you cannot. You will save from wherever you work from because you want your child to have a better life just like your neighbor’s so it will force you to save too.

**I: Number 07 there was something you were emphasizing on saying “yes” can you explain it.**

PB07: (Laughter) I don’t think I have anything to say

**I: Are there specific characteristics of female sex workers who do not save?**

PB05: Yeah. Often they are those who do not take good care of their children, who do not care how their children look like. They don’t care what their children will wear their concern is their own dress code. They are not concerned how their children live, what they wear, she doesn’t even care whether she left lunch for her child. Those kind of people, those people they do not save.

**I: Another person? Let us talk so that we can finish**

PB01: As number one I can say those who do not save any client that come their way, they just slash and go their way. [Laughter]

**I: Kindly speak up you know we are recording.**

PB01: For those who do not save any client who come their way they just slash and go.

**I: They slash, meaning?**

PB02: (Laughter) If she sees any client who has come and has stopped his vehicle there she is the first one at his window.

PB01: Such a person will go to any client, is she sees any confused drunk who is from a club and he is willing to give you 50bob (snaps finger) (whispers).

**I: Another person?**

PB04: As number 04 its true there are others who do not save. Because I see… there is a neighbor, I know she is not here so we can gossip about her [laughter] this neighbor also does this kind of work. But that work of hers I doubt whether it’s similar to this one I do because hers is different. Because she leaves, the way she left at night she will come back the next day at 10am. She comes back dusty up to the knee, sometimes… she looks like someone who comes from disco matanga [ disco that are played when someone has been buried] but she is from that job. Her children did not eat the previous evening they slept hungry, the children do not have clothes but she has come from that job. That’s when she comes to ask for soap from you, for bathing and wants to leave again. But the children have not eaten… it’s the neighbors who look after her children. Obvious such a person does not save and does not eat well and the children are suffering. Such like people exist and they are many. (Whispers, laughter). So now I do not know how you can help such, how can such children be assisted?

PB07: Prayers (laughter, whispers)

**I: Number one you have something, I want to hear (laughter)**

PB01: definitely surely those are the ones I was saying previously, anybody passing along the way you just slash including the one who does not pay you. At the end of the day this job you do to get money so you come back dusty all over because he gave you nothing. You do not even have fare going back home it forces you to walk all the way from town to home. Then the package, this work is package, as in every business is how you package, even if you want to sell mangoes you must wash them nicely and package them well to attract your customers and they will feel worth the money you are asking. Now surely if you go to work in sandals, slippers that you use in the bathroom surely, who will approach you and tell you ‘I have 10k’ who? Only a 50bob worth client will come and quote even 20bob. Yeah so package is important.

PB04: If I add again on that issue of coming back home with nothing so making you not to save. Like this girl, when you ask her she tells you that whoever she spent with tells her “your place is close by you can go round the flyover” (laughter). for sure it is something for real [ true] You see someone has told you “just go round your place is nearby, just cut through here” You have arrived without anything. And then what brings about these problems that causes one not to save and be cheap, I call that being cheap, what I see with her it is not good discuss someone else status but it’s her who says she is spreading the disease. So for her she sees that that is the job she is doing and so she does not need money, so if you give her fine and if you don’t that still fine. That is what is hindering her from saving and she is putting the life of her children in danger too.

**Note-taker: I would like to know, that is just one case you have given us but there are several cases like those ones. What do you think will make someone decide to go with a person who would tell them “your place is nearby just go round through this way” we can all talk about that.**

PB07: That is maybe a person who is never satisfied. They do not have a man in the house, those people who are addicted to sex they cannot go a day without it [sex]. But if you are out there to make money you must look like the money. You cannot leave the house smelling sweat with bathroom slippers and you expect to earn 10k where will it come from. So such a person has gone because they just want to have sex and they do not have any other reason.

PB01: but I think… as number 01 sometimes it’s also self-esteem, amongst us sometimes we tend to lower our fellow self-esteem. Maybe because… you know we are not the same how we look, how we appear, our bodies not the same. Maybe someone has been body-shamed, we have been body-shamed amongst us. Some we have abused them because they do not get clients or maybe how they represent themselves or there are those they do this job but they are still fearful so sometimes they get a client and they decline and so it reaches appoint where you are doing it to prove your fellows that you can do this job but at the end of the day you are not getting anything. You are just going with anyone because you are trying to uplift yourself-esteem to prove a point to your fellow sex workers that “I can really do this job and I can get this clients”.

PB03: As number 03 for me I see there are those maybe they go for this job and they are being like someone has taken advantage of them, men take advantage of them. Like you go thinking he is going to pay you, he uses you and then he does not give you a thing and then you being a sex worker you have nowhere to report them to. You cannot tell the police that this guy forced you and yet you took yourself to do the job.

**I: Is there anything anybody wants to add on. Okay, now for female sex workers who save, why do they save?**

PB05 Okay, as number 05 like you can save. Let’s say that maybe you are single but you have a kid, and your child is approaching to finish primary school, you have to save for that child in order for her to go to high school. And then when she is already in high school you need to save so that she is not sent home for school fees. And also for her to be on the same level with other is she is in boarding school. You know boarding school has its own pressure you see people have things you do not have and it will make you feel bad. Others may say “I have gone through a hard life I must save for my child in order not to lead the same life that I did” So that their children experience a different life from what she lived yeah, so that is what encourages people to save.

PB04: Yeah, as number 04 in short sex workers or like me, I save this job that we do or I do, is my body … there will come a time when I will be sick and will not be able to go for work and that is my source of income so that is why I save. I can even get an accident out here now and I will not be able to go for work so that is why I save. So that is why I save, in case of emergency it can help me.

PB02: According to number 02, as she has said we also have a family, some of us want to help mum, dad they may need something and they know their daughter stays in =Kisumu= and working, despite them not knowing what kind of job their daughter does. So, they will call you “mummy I do not have this or that” because they know you are working you have to send money.

PB05: As… another thing is this kind of work you’ll not do it for the rest of your life and it does not have pension. So, it’s a trade that when you reach a certain age you are no longer marketable.

**I: What particular age?**

PB05: I can say … when you reach somewhere… like about 50. And who will be giving you the… who will be interested in you because they want young –young people. Yeah, so when you reach such an age, it’s like your job ends and you will not get that pension because it doesn’t have one. Yeah, so that too encourages one to save.

PB03: As number 03 as a sex worker I save because I want to be in the same level like other Kenyans because I see for example the government has provided NHIF for example so I save so that my child can have that same… can feel like the other people children, they too can be in the NHIF card just like the others.

**I: Another person, why do you save?**

PB07: Number 07 I also save for various reasons. Reason one I am a single parent, my child is a year old now and I am trying to prove to his dad (laughs) that I can do this alone and I have been doing it just fine from the beginning so I have defeated him. So number two my parents, after graduation it’s been hard to look for a job and to land on one, so they do not know what kind of job I do, they are aware I do online jobs and that’s how I pay rent which is true but they do not know I do this other thing. But that’s the only way I can help them because my dad is at the village currently and my mum operates a small business but it does not fetch her the cash she requires so I have to help. My sister is in school so sometimes I help to raise her fee.

**I: Number 06 do you have something to say?**

PB06: ah… a number 06 what I can say is, I save because I have a child and the child is supposed to go to school and I am the one to provide everything because the dad does not help. So, saving is good because I may be in need of something and I can’t get the money at that instant. So will get it from what I had put aside to help me cater for the need.

**I: Number 01, why do you save?**

PB01: Because I have needs to attend to, and I do not need to do this job for ever at some point want to stop and may be start my own business. The main goal of saving is to open up a business.

**I: Now what makes it easy for you people to save?**

PB01: Number one we have a motive and two money is coming in (soft laughter)

**I: So, if the money is flowing?**

PB01: someone saves, because you can’t save air, or what do you think? You save money so what it means when you are getting clients, good clients you are getting money and of course you will save.

**I: Another person, what makes it easy to save?**

PB07: number 07, I want to give my child the best he can get so one day I can sit and smile “it was worth it” and I have a lot of dreams, like I want to live in a big house have my own car, buy my mum a house. So when I remember such things then it makes it easier, yeah I keep saving anytime it comes.

PB05: As number 05, what makes me save is the life I have gone through. I went through high school, I don’t want my children to go through the same I want them to go… to experience a better life there. They feel at home when in high school.

**I: Another person? What makes it easy to save? If there is none we can continue.**

**P: Mmh…**

**I: What challenges may you face as you save?**

PB05: Okay, as number 05 you know in life there comes with a lot of challenges. Maybe in the process of saving your child can get sick or another problem may arise somewhere like death of a relative that necessitates family to contribute. Something of the sort. Or any other burden like for example something gets broken down in the house that requires to be replaced.so it will force you to get into your savings to sort out those things. Or maybe school have new levies or they want some other things. School expenses can be way too many until you decide “Ah! Let me break this thing and I will replace it” [house bank]. And then the next time when you want to replace another challenge comes up and the cycle continues. So those are the challenges that I see as myself.

**I: Another challenge? Challenges of saving which one can face?**

PB07: Number 07, I think adding to what number 05 said. We all go through the same things, because at some point, you find you are fed up with the house you live in and maybe the landlord has resulted to increasing the rent and it’s not worth it and you have to look for another house which will require rent and deposit, the process of moving may rent to some of your items breaking and you’ll need to buy new ones and all that. Then school and school fees and other small levies sometimes you get a phone call this or that is required and may be you do not have cash there and then and it forces you to get into your savings. Something like that. The sickness and all that.

**I: Is there any other thing one wants to say? (Silence) Nothing…**

PB04: … I want to add, so the challenges that we face, mostly as single parents, I will save and the baby gets sick the source is only one just me. So it forces me to get the money from the savings and sort out the issue.

**I: Now how can we may be address these challenges? Like for example the challenge like the child being sick, what can we do?**

PB05: Now in such a situation is where NHIF comes in. Because there is this one that you contribute 500/= per month. You see such will cater plus the children and yourself. When sickness come you will not need to break the cash you saved, like “I need to take this child to hospital you will use that NHIF of yours and it will cater for the medication. And maybe coming to family issues like funeral, personally I see fit to put aside some emergency funds don’t be like “whatever I have saved here can be for emergency” no. Just take some and put aside and say “this I put aside just in case something happens unexpectedly like a funeral at home, I can use this to cater for that.”

**I: Now how can we overcome the challenge of renovation? You said maybe something can get broken and need repair.so how will you overcome this?**

PB02: Number 02 like I said, that is why I spoke about putting extra, extra savings say you had decided to save 200/= and you go to work and earn more so you won’t be like, “Now I just want to save the 200/= that I usually save” I will add some more because I have earned more. Say I was getting saving 200/= before now I will add 300/= to make it 500/=. Now the 300 will go pending for that emergency. so now if I have something spoiling I can say “I have some saving I was putting aside and some extra I can take and use.”

**I: And school expenses, how can we address this challenge?**

PB05: For me with school my view is do not wait for the fees to pile up [accumulate]. If you hear there is money needed here, just try the much you can to pay it. If you say you’ll go to ask for permission for the child to be allowed to stay in school up to end month or some think like that or but the end of the term you say you’ll pay, and know you are sure “I can get 100bob per day to pay to pay for the stuff” the more you keep postponing another levy will be sent for maybe money for Saturday tuition, or exam fee. So for me I see if its school fee whatever little you get, if the child comes home and say “mum, we have been asked to take money for this or that”, just try and pay it. You know schools allow payments in instalment that happens. Pay some and next time you shall complete it but if you wait for the fee to pile to the end of the term it shall become burdensome.

**I: Anything anyone wants to add? Nothing. Now we want to know, for female sex workers who do not save, what are their reasons for not saving?**

PB06: Number 06, I can say maybe she has not saved because she doesn’t have, she could have gone to work and I could use the case of scenario like the number 04 said, she has gone to work but she has not been paid. I think those are the challenges that can make one not to save money.

**I: Another?**

PB05: As number 05, I see like I could say if I am alone, it’s me and myself I am independent I do not have anybody who depends on me. there is no need to save money, It’s me and me alone. What I get I will buy clothes eat great food and I treat myself and pamper myself and life continues. After all they do not think ahead, she doesn’t think “there is someone somewhere that I need to save for”. She looks at it as “this is my life, this is me I live however I feel like” she doesn’t think about saving. Why should she save, what for? Yeah.

PB02: And another one, as number 02, there are people who have given up on themselves, a person may not be in talking terms with her family sometimes and so she decides this is my life I want to live however I want. I want care whether that is my brother or my sibling. I just want to only care about my life.

PB05: And mostly girls say, “If I were to die this people will have to take my body and they will have to bury me. I don’t care. It’s not urgent who am I saving for”. She stays with friends and if she dies the family will look for her.

PB03: As number 03 there are those that are… nowadays there are sex workers… like not sex workers only but it cuts across all people depression can push someone to lead a life where they do not care. Yeah depression.

**I: Reasons they do not save. Why they do not save?**

PB05: Or maybe you may fail to save maybe… you know if you compete with another person, you are competing with a neighbor who is a health care provider, you live next to a health care provider and you want to lead a life like theirs. Yet you cannot make the kind of money that they make. You can’t manage. The little they make they will buy a woofer you also rush to buy one they buy another TV you also want that TV, they buy a fancy seat you want that too [claps] … [cross talk]

PB02: …you do not have the money.

**I: Is there anything you want to add? Alright, now what are the disadvantages of not saving?**

PB02: Me as number 02, I could say sickness, you know the body is a something… it’s not like a tree the body can get sick and when you are sick and you had not saved it will force you to… however sick you are, you have nothing to eat because you have no savings and you have nobody to borrow from. Now, when you get sick you now want to borrow and when you were of good health you never used to borrow or lend anybody. Do you think now the person you want to borrow from now will help you? They will not come through for you. So when you get sick and you have no savings it will somehow weigh you down.

PB07: Disadvantage of not saving, you will be forced to depend on others, and they will not help you all the time. You may tell them “kindly loan me some money” they will be like “oh… you should have told me earlier, I have already given someone, I have used to do this or that”. You see when you have your own money, when such things come, you just do your thing and you do away with debts.

**I: Another person, the disadvantages of not saving?**

PB03: Me I think as number 03 it may lead to like crime. There are people if you realize you do not have any savings and you have gone out to look for a job and have not found one. You end up getting into some gang so that you want to make some money because you are desperate.

**I: Any other, now we shall continue. What are the advantages of saving? What are the advantages of saving lets respond so that we can finish up. If we become silent we take too much time.**

PB07: Okay number 07 I feel like your question, the previous question we have answered [inaudible] question.

PB02: Because we were giving the problem and the solution at the same time.

**I: Aright we will continue. So, where do female sex workers typically save? Where do you typically save?**

PB06: As number 06 we mostly save in Chamas

All: Yeah.

**I: Just Chamas?**

P: From savings.

**I: What about the bank? (Laughter)**

P: They deduct people’s money

PB04: no this job does not have a lot of money. It has little cash.

**I: Mpesa?**

All: we will use on credit. (laughter) and bundles and meat and fuliza (laughter)

**I: MShwari?**

PB04: Maybe, one can save with mshwari because you can decide to save by locking it, yes so you can save in Mshwari.

PB07: but still you can withdraw from Mswari lock saving because they give you like 2 days and your money will be in your Mpesa. So still it will not help you in a way. It will depend if you are the type that can assume that money. Personally I am not able to (laughs)

**I: So where would you prefer to save your money?**

All: Chama.

**I: Chama? Now what are the reason for preferring Chama**

All: merry-go-rounds.

**I: So now why would you prefer Chamas?**

PB07: Because you get your money once in a lump sum and can do something with it. Most Chamas you get a lot of money, like may on the lowest could be around 8k. Because you can’t join a Chamas where you’ll get 5000/= after one month (smacks lips) that’s like wasting money because If it’s monthly your target should be 10, 15, 20. Yeah, that can pay rent, you can pay school fees and all that.

**I: That’s number 07, you are forgetting to mention your numbers. Who else has something to add, the reason you prefer to save with Chama’s?**

PB02: As number 02, maybe you have a target, by such and such a month I want to have done this particular thing. So you look at a Chama and analyze it, how many people does this Chama have, and how much money is contributed? For example, I am in a Chama that we contribute 100 shillings daily and we are 10people which is 30,000. So when you set a target, this month I want to accomplish this and you set the amount for that target so you take part in the Chama knowing that when such a such a month reaches I will be the one receiving the Chama money and I will use it on what I had planned to do.

PB07: Adding to that, as number 07, Chama in some way makes you to give out the money because in one way or another you will have to pay this money especially if you had received someone else’s, you know if you don’t pay that becomes a debt. Then no one will trust you to include you in another Chama. So you have to pay it. If forces, you.

**I: Another thing, is there anything anyone wants to add, on the reason why you prefer Chama?**

PB04: In addition, as number 04. Chama is good it doesn’t have all those complexities of saving like interest sometimes the =Equity= has been robbed and such like. In Chama if a person steals from you, you will follow that person (laughter)

**I: Let’s move on, now do female sex workers live typically beyond their means, I mean where they spend more than they earn? Number 07**

PB07: Well sometimes like when we finished school, you find your friends saying they want you guys to have a reunion and all that and you have this huge plans and they see you are doing well in life so they expect you to turn up. You see anything to do with parties one has to spend a lot. And you find the party has been planned to take place in =Nairobi= you have to go, the fare, where you’ll stay, you know putting up in someone’s house even if it’s for a week the host expects you to assist, you see staying in someone ‘s house even if it’s for just a week and you are not giving any contribution you feel like a burden, so you have to give something small like money for food and all that. So that’s like you will end up spending what you did not expect to. Then you really wanted to meet those people so you will have to show up.

**I: Who else has anything to add, do they live beyond their means like you spend more than what you earn? Is there a**nything? There is none So **you say you spend more when you want to…** **maybe to join the party the reunion and now you want to show them you also have money. Now do female sex workers bridge the gap between what they earn and what they spend, if they spend more than they earn. Like (peeping sound of the recorder) now I have asked how do FSW bridge the gap between what they earn and what they spend if they spend more than they earn. Like you earn less money and have spent more, how do you bridge that gap? Tell me how you will bridge that gap?**

PB03: Number 03 you will bridge the gap when you get a client who will pay well.

**I: Is there anything anybody wants to add? (Silence)**

**There is none**

**I: Okay and do female sex workers often borrow money or get into debts?**

PB06: Yes, what will make you get into debt is maybe you go to work and you do not get anything and maybe you have no food. Sometimes you’ll be forced to borrow so that the next day when you will have to pay.

**I: So, where do you borrow from?**

PB05: May be at the shops or from a closest friend.

P: You can ask them to lend you and pay them back when you get.

**I: Number 04 you are saying something.**

PB04: You can borrow from the money lenders, those who walk around. We must take debts, we cannot get every day.

PB05: Number 05 when it comes to borrowing I just depend on my Chama.

**I: Now this money that you borrow, what do you use it for?**

PB05: You know when we go out to borrow it means that there is something you lack. May be you may lack school fee, or rent you have not raised the money. The land lord is there and you have 3000/= and you require 4500/=. It will force you to borrow for you to fill that gap.

**I: What else do you do with the money you borrow?**

PB02: Number 02 like let me say for example my friend here has said she runs a cloth business, let’s say she goes to the market and finds the prices have gone up at the moment and when she looks at her account including the emergency kitty , that money will not be enough. Now she decided let me borrow from someone to feel this gap I take my Mutumba sell and I will return the money...

**I: Now you have taken the money, what do you do to pay it back?**

PB02: Number 02 like the way I have used the example of my friend here, she said her extra business is to sell Mutumba . She borrows money to add on it, obviously if you borrow money to use on something that is going to earn you profit. After selling it will make profit and you will get the money to pay back.

PB04: As number 04 I continue as number 04 you said we can talk about some else’s experience not necessarily about us.

**I: Yes. You can even give example…**

PB04: … I have even seen others borrow money from the Maasai and if you are not able to pay, you pay back with your body. [ in kind]

PB02: Yes, it happens.

**I: They pay with their own body… now does the Maasai approach you?**

PB04: no he does not approach you. If you are not able to pay it back, the Maasai have taken that advantage when they are lending, they lend to women not men. If you are not able to pay he comes and threatens you. So if he threatens you now as a sex worker you see it as the only way to pay back. It is something I have seen happen.

**I: Are you then one who decided to pay back like that or he is forces you to pay him in that manner?**

PB02: There are others who decide to pay in that manner because that is … there is nowhere to get the money from. That is the only thing you have to pay with in your body. [ in kind]

**I: Alright, which is the other way we pay back these debts?**

PB05: Okay, as number 05, most of the people who are here have another job besides this one. And this other job we get paid after a week and you will use this money to settle the debt.

**I: Okay, alright. Now what do female sex workers typically do to increase their income?**

PB04: As number 04. Doing side jobs like for example my mitumba business, yeah to increase my income.

PB07: This is not my experience but I used to hear people say before I started this thing [ sex work] I used to hear girls in school say that there are products the buy to wash down there [ the private part] so that the thing smells nice. Then you go sit in front of men and open your legs so they look at you and if there is one you notice is staring you go to him allow him to touch it and if it satisfies his fantasy then he pays you whatever amount you ask him to.

**I: When you say that thing, what do you mean?**

PB07: Vagina (laughs) it’s washed, they say there is how it’s washed and becomes clean with a great scent and becomes appetizing.

**I: What else can we do to increase our income?**

PB05: As number 05 you look attractive, put on smart clothes you should not dress like a member of the =Repentance= you dress smartly.

**I: How does a =Repentance= member dress?**

PB05: They put on very long dresses and a coat (laughter) in such you see a client will look at you and say “this one is saved” you are there for the job but he will look at you and think “this girl is born again” imagine he will bypass you and may even miss someone to serve him and you are standing there. You should dress attractively.

**I: Now, maybe apart from sex work, dressing attractively, using those many products you said. What else can we do to increase our income?**

PB03: As number 03, we can venture into business. Just business, selling we think of some small business little sales here and there.

**I: Is there anything anyone what to add? (Silence) nothing. So let’s say you don’t find a client, what do you do?**

PB04: I go to the savings. I will go back there to get something small, I cannot go hungry because I am saving money. I will remove from the savings for use.

**I: Number 07**

PB07: (Laughs) for me I some cause drama with those people. Because he usually tells you he is your monthly business and all of a sudden he cannot show up. “What are you saying?” I get so mad at that instant until he changes his mind and says “okay I will be there.”

**Note taker: Is this like mounting pressure on them?**

PB07: Yes, something like that, because he is aware that you need this money for other reasons and he tries to assume that. Why is he assuming, yeah you put pressure on him? Until he agrees to come.

**I: What else do you do when you do not get a client?**

PB03: As number 03 I am forced to get into debt again I go back to borrowing.

**I: How can you tell that you will not get a client?**

PB04: As number 04. You cannot tell, sometimes when you are not in the moods at all that is when you get a client and sometimes when you are like “today I don’t see like I will” that is when you get. When you have *chwadi* [sheng] [laughter] is when you do not get any client.

**I: Chwadi mean what?**

PB04: When you have taken your time to dress well and look attractive.

PB02: When you have the heart to go out and work and there is no work now

**I: So, if we say…at what time are you able to tell “today I have hit the wall”?**

PB06: I could say, as number 06 maybe… maybe… if you do sex work you do it online over the phone and your phone not ringing you will automatically know “today there is no business”

**I: At around what time?**

PB06: Let’s say you have been expecting his call since morning or in the evening.

**I: And he fails to call?**

PB06: Yes, he fails to call. So, automatically you know there will be no business.

PB07: Number 07 sometimes you may be seeing a married person and he is assuming you because of the wife. So you find sometimes he is around and sometimes he is not. But we do not care about that because we had an agreement and he has to stick to it.

PB04: As number 04 we were talking about the time, the time is about 11 or midnight. 11 at night.

**I: So from 11 you can now tell you have hit the wall. Okay. Now, how much money can one have in a debt at any given time? Debt, how much can you have as debt at any given debt.**

PB07: Number 07 like myself at the moment I have Fuliza of 500/= that I had to borrow.

**I: 500/=. Number 06?**

PB06: Do you mean the debt I have per month or… [cross talk]

**I: … No, in general any debt that you have can amount to how much?**

PB06: it can be about 7000/=thousand.

**I: Number 5**

PB05: 500/=

**I: 500, number 03.**

PB03: 2500/=

**I: 2500/=, number 02.**

PB02: Just a thousand.

**I: A thousand, number 04 the debt**

PB04: 26,000/= t

**I: 26000/=. Okay, now we want to talk about leaving sex work. We are about to finish do not grow tired. Do sex workers ever think of when they might leave sex work?**

P: Yeah

**I: Number 07**

PB07: I have thought of such a thing before but then again in our present Kenya getting employment is hard, even if you get one the pay is not equivalent to what you have been getting through this other thing you have been engaged in. so, like personally I do not see quitting right now or even in the next 2 years because the clients that I have pay me money that meets my needs. So, if I was to leave and the money I get in a day is what I will earn in a month as salary in another job, I’ll choose the one that pays me daily. So, not anytime soon.

**I: So, for now you have not thought of leaving?**

PB07: Not yet.

PB04: As number 04 mh… I think of leaving because of my children or the child. So as they grow it will be hard to hide it. And there are people not just neighbors but there those who are there to tell your child the truth (laughter). So those are the embarrassments I do not want to go through in the future

**I: You do not want such embarrassments.**

PB04: Yes.

**I: Someone else?**

PB05: As number 05, by 2026 I am not in this job. Yes.

**I: Why?**

PB05: Because I am targeting to get cash, I have some money I am saving in some place. I see opening a shop. So, by 2026 I do not want my children growing up asking “what kind of job does mummy do?”

**I: Someone else?**

PB02: As number 02, I think if you decide… maybe you decide you want to leave this field of work. Let’s say you get a caring husband. Say you get a man that you can say “this is a real man” and you discuss and agree. And find out “this one is working and earns a lot of money and can support me” so you decide “sincerely I have found a good husband with money and will be helping me to cater for some bills that I used to pay by myself”. So in such a case you can decide to leave sex work because you know you have someone to support you. You are not alone. Yes.

**I: Another person?**

PB03: As number 03, I have also desired to quit but I can’t… okay for now I cannot quit unless I get an alternative. So, mine is unless… if I get an alternative today even at this moment I will quit. Unless I was to get an employment or a business.

PB04: As number 4, to add to that I want to get a position at the church.

**I: So, if you get that position then what?**

PB04: Now I will have to leave this job.

**I: What kind of position do you want?**

PB02: You want to become a woman leader (laughter)

PB04: not a woman leader, but they want to appoint me as secretary. So secretary and this kind of job do not rhyme. So, I will have to leave in the near future.

**I: So, do you discuss among your peers about leaving this sex work?**

PB03: As number 03, I have friends and we discuss until we come to a conclusion of what to do next but you find what you want to do is business but you do not have the capital. You end up going back again to the streets (laughs)

PB04: As number 04 when this thing gets into your blood stream, (laughter) when it gets into the blood stream…[cross talk]

PB03: … it’s the lack of money, once you get broke you just go back.

PB04: in addition, it’s not good, because it makes one become lazy.

**I: How?**

PB04: Lazy as in you cannot do any other hard job and you are used to the job that brings you quick cash.

**I: Number 07?**

PB07: There was a time I was with someone and he was paying well but then I was expecting a call… he called me and I did not pick it. I called back and the wife picked it instead. And that is how I missed out on that huge pay (laughter). Because the guy was ready, he was a bank manager and was ready to pay very well as long as you do what he asks for. And you know when you are doing this job you end up doing whatever the client wants as long as you want that money, you do it.

**I: Now, whatever he wants may involve what?**

PB07: (Laughter) well, sex sometimes is very nasty there are those things you are uncomfortable with but you can try out…

**I: ... Like, tell me some example.**

**Note taker: It does not mean you have done them but maybe …[crosstalk]**

**I: No, example**

**Note taker: Not what you have, what you hear people say.**

PB07: Well some people are comfortable having sex without the foreplay and all that, sucking the dick and shit. But this one insists that that is the first thing you’ll do first. Like about 30minutes and it is disgusting. You will have to do it because he says he shall pay you 20 thousand and you can even see it there placed on the table “here it is”. You will have to do it and clean your mouth using a mouth wash later. And you do the thing get your money and leave. When you leave that place in the morning no one knows what you did for the money, you pay rest you consume the family bread by yourself (laughter) . So, yeah that how it is.

**I: Now what triggers this discussion of wanting to leave sex work?**

PB07: Personally at some point you feel like ai! No, this is too much I need to graduate to a wife. I should not be having sex with just anybody. But then you remember you may end up with a man who is… you know once you sleep with different people you get different types of satisfaction. So, you find a man who does not belong to you but he gives you what you need and the one that belongs to you does not give you.

**I: What doesn’t he give you?**

PB07: Does not know how to do it.

**I: He does not give you what?**

PB07: He doesn’t have any idea, some men are just out here to just have sex. They do not know how this sex is meant to reach… like the woman is meant to feel like her man has given her what she desires. But he just does it as long as he is satisfied. So, you will go out to get some more and get money on top of that too.

**I: Okay, anybody who wants to add anything? None. And usually do female sex workers leave or want to leave sex work for personal reasons?**

PB03: Yes, as number 03 I would want to leave because of my child. Yeah just that. Because they are growing and the moment they are of age it will not be a good picture.

PB07: As number 07, I have a problem, for me, I think for me my life revolves around social media because I see a lot of people who have made it through this kind of business. So things like the kid and such like are never bid deal to them. Because at the end of the day you have a target, you want to reach a certain level and whatever your child will think in the future will not stress you as such because now for me my child is little and does not understand a thing and does not know what I do. He only sees me in the house in the morning during the day he is with the nanny. So I want to give him that kind of life I want. For now, I do not mind what he will think when he reaches 5 years or there about but once I get the opportunity to leave this thing I will just leave it. But I won’t leave because of want him to think the mum is doing an honorable job or the mum does unworthy job, but I would have grown tired of doing it instead. Because I see those people in social media are just there. You get the car and you are good. You get the house and you are good. Whatever anybody thinks is not your problem.

**I: Are there personal reasons for wanting to leave sex work?**

**Note taker: So, you as number 07, that… because you’ve mentioned opportunity what are these opportunities that others want to have in order to leave as a personal thing?**

PB07: Every job comes with a promotion, there are those who do this job and are in a level we cannot reach however much we try. So Iike, somehow at some point I felt like there is no point of looking for any other thing when I can just work towards getting what these people get. Because there is… I had a friend in campus all she targeted were white guys and eventually she found one with a lot of money and she lives in a mansion at the present, but the guy is not the husband. She has a child and I mean they just live okay. So, I just felt like there is no need of doing any other thing while I can think how to reach that level because currently papers do not matter. I have studied and I have my degree but getting employment is so hard. It’s been 8 months as at now job hunting, you are in the field at night and during the day you are looking for a job that the parents will be proud of But you don’t get it This other one that they will not be proud of is what you land on. So this is what I am working towards right now.

PB04: As number 04, it’s about age.

**I: Age.**

PB04: Yes, just that.

**I: And the external reasons, to leave sex work are?**

PB04: How people perceive me, how will they perceive me? I have worked for a long time… like people currently when you leave for work you see how people look at you and you say now “this is the end” (laughter)

**I: Another external reason?**

PB07: As number 07, adding to her point, I think I understand her because she wants a position in church. And you know once you are walking in that path you have to look the part. But someone like myself I personally do not give a fuck about whatever you think. It will not be written at my back even if you say I am a sex worker of I am so and so. That’s your issue. You find the person speaking about you is sells vegetables when she leaves at work in the evening she has 200bob in her pocket because she has paid whatever she has paid for maybe her debts. In fact, even that 200 is a lot. That 200bob is what I get in a minute so I won’t mind what such a person says. Because whatever she says does not help me I know what really helps me. So, it depends with how you personally feel about whatever you do.

**I: External reasons that makes you want to leave sex work are?**

PB03: External?

**I: Yes, we initially talked about personal now, what are the other reasons that are not touching you, what are they?**

PB03: As number 03 I see like they will somehow touch me, getting married honorably.

**I: Getting married honorably how?**

PB02: You want to be faithful to your husband.

PB03: Yes, getting a good husband and staying in the marriage.

PB04: As number 04, what can be an external whatever, how you are treated there… how they treat you. There people who go and are ugly and they do not look attractive, and when they go there how those people mock them makes them consider leaving the job.

**I: Is there anything else?**

PB04: None.

**I: Is there something you plan to do after sex work?**

PB05: Yeah as number 05, like if you know a place you are gaining your source of income if you leave that source of income you will not come back. You will have to get another source of income so once you leave that work you go to another job, because you are used to get money obviously you have to go somewhere else you will earn money.

**I: Now, maybe asked the question wrongly. Like do you always leave when you plan, like if you say ‘like I want to leave sex work’ maybe like in your case you said you want to leave sex work by 2026, will it turn out like that or it may change?**

PB05: That how it will turn out. It will not change.

**I: Why will it not change?...**

PB05: … The reason why… okay the reason why it will not change, I care about my kids, I want… I do not want another person to ask her “what does your mother do?” I mean… obviously at home they do not know what I do, but here in town there is a person who must look for you just to find out “[name omitted] Stays in town, what kind of job does she do, why does she look in such a manner when she comes at home?” there is a person who will investigate you until he finds out where you are. I do not want my children to live… to ask themselves “what does mummy do, why does she always leave at night’. Because obviously if one asks… like in school there is a time I can remember in primary when we used to ask each other “what kind of job does your daddy do?”, “my daddy is a teacher and yours? “a doctor” “what about you” “my mummy leaves the house every night”. Obviously when you hear such you will conclude “Oh… my… this child’s mother is a prostitute”. Obvious (laughter). So, that is what pushes me, I will have to quit.

**I: Another person? When you decide to leave will you stop at the time you have planned to?**

PB07: number 07 I will not leave at the time I have planned. Because I have been thinking of what I want to do besides this and it is not forthcoming but I have always desired to own a club it has a lot of money. Considering this business of ours is a night business and the club works best at night so those who have no idea what I do, they will know I am busy with this club. Like my mum, she is the one person that I wouldn’t want to know what I do, the rest can think whatever they want to. So, how I have planned may not work as planned so will go with however things turns out. So once I save capital for starting such a thing then I will just stop. But I will have to save like forever. That will require I hook up with the white clients (laughter).

PB02: Milk

PB07: Milk, do you have ulcers?

PB02: Yes

**I: And now, female sex workers typically leave sex work at what age?**

All: we can…[inaudible]

**I: Lets speak one at a time.**

PB06: As number 06, when speaking of age, I can say around 50 or 40. When you reach 40 you will think no one will notice you. Those men want the young ladies, maybe about 40 you may present yourself well and dress in a smart manner and maybe you may attract some clients. But beyond 40 it may not be possible.

PB04: As number 04, when you reach menopause.

**I: Menopause comes at what age?**

P: 40-45

PB04: Those things [ menopause] reach about 50 nowadays. Because I still see some old ones still in the field. In fact, those are beyond 50 and they are old and you wonder what they are looking for

**I: Now at what age do you think one can stop?**

PB04: When they die.

PB07: Number 07, I think whoever wants to leave they leave having their own personal reason, because even those older ladies are there. And there are older men who want those experienced women, someone who looks a little like him. Not all men want the young ladies. So if you are at the age of 50 or 40 you will leave because you want to. Maybe even at that 50 you may feel like the money you get is good. You may be active but you are old.

**I: And…**

PB03… number 3 I can say if one wants to leave they will leave at any age.

**I: At any age.**

PB03: Yes.

**I: Any addition one wants to make? Now what do most sex workers do ones they leave sex work? (Silence) What do they do once they leave sex work?**

PB05: Like… the way I understand that is the kind of job they do.

**I: Let’s say I want to leave sex work, so after sex work what kind of job do I want to go for? Or what kind of thing do I want to be involved with?**

PB05: I feel there, it will depend with a person, once you leave something you leave because you have a plan, you have a plan B or you have pictured ahead and seen “this one won’t work, let me try this other one” it means you have laid down a plan already ‘I am leaving this job and getting into this other one’ so that depends with which other job you want to do.

**I: Now this other work you want to get into is what I want you to share with me, what is this other work?**

PB05: Like me if I leave, I said I would open a shop of which I am 100% sure I am going to do it. Yeah, [inaudible] so when I leave I move to my shop.

PB04: As number 04, those who have left the job, you know when they are in this business most of them I see they go to… I mean they used to apply makeup they used to dance, so when they get some money and say they want to leave most of the time I see them open bars, boutiques, those things that go hand in hand with the job she was in. and they do well because she understands the advantages and the disadvantages.

**I: Any other person who wants to add on what they do after sex work. When they leave the sex work. None. Now where do they go to do this job? If one wants to open a shop wants to open a bar, a boutique where will they go to?**

PB05: As number 05 I will have to relocate I cannot do that job here in Kisumu. I will relocate I look for another place where no one knows me. For me the problem is my children. I want a place where no person will feed my child any information. Even if you feed other people this information but my children I want the way they have no idea it remains like so. In future if they get to know they will have grown and I will explain everything. But now if I tell a child whose 12 years 13 years there is a way they will start viewing the mother. I will obviously relocate and go to a place where no one knows my children. Yes.

**I: What else will happen? Where will one start this business of hers?**

PB03: As number 03, if I leave this business maybe I open…will I get the money and open even a bar or a club. I will target where there are youths especially here in =Kisumu= where there are these colleges. Yes, because in such places you will get the business will flourish because the young people are many.

**I: Let’s say I leave sex work is there a way life becomes different?**

PB07 (Sighs) As number 07 I think it will be different because once you are a sex worker you are used to handling money daily. But if you get into another business, there will be profits and losses. At some point you will have money at some point you won’t have money. And then the money you will handle in a day will not be equivalent to the money were used to being given. Yeah I think I may be different. But at times you may get more money compared to what you are given out here.

PB05: As number 05, you know when you leave one job going to another adopting to the other can also be hard. You may say “Ei! I was doing something else this one is now it requires my commitment, I be at work, by this time I am awake, I do this…” it requires adapting.

**I: Adapting.**

PB04: Can I add? As number 04 it helps the body, the body goes back to its shape. It the body it makes the body to… it was used to working daily now it will relax.

**I: Now, do you know instances where female sex workers have left sex work and returned into it. Left and came back.**

PB02: Yes.

**I: Number 02**

PB02: As number 02, one leaves and goes to start up a business, she decides to leave yes and opens a business that does not do well. Then she starts to make losses, maybe she opens a bar and she realizes it’s going down in away. But since she has employed people working there she will go back to the field. Even though she has gone back, you may not see her at the field but she may have had clients who used to call her so those are the ones she will start looking for. Because she knows, “this one used to be a good client, if I call him he will be like… where are you? You are so lost pass by I get you something” and she will go and get some cash to come and boost the business.

PB05: As number 05, yeah there is a neighbor of ours who used to work in some place and that’s where he met the man and the man decided to marry her. He used to be her client and he was like “I feel like I want to start life with you”. But something else is, where did this person find you? Because of the place he found her later on in life he started telling her “you are a prostitute! Where I found you, you are a sex worker” But she had stopped, you see she had the plan of leaving and found this guy and the guy told her, “I just want you to be my wife, I do not care what kind of job you have been doing.” It reached a point where this guy does not give her any money, he has left her to fend for herself. If she asks her anything she is told, “other women go to do shamba work to get money” and remember you were never used to that kind of work he never met you where people do shamba work. It forced this lady to finally decide “I have to go back”. She decided to look for money where she initially used to. So that also happens.

**I: Is there someone who would like to add on to that? None. Now what are the negative things that happen when they return?**

PB07: I think maybe you will find someone else has taken your place. For the period you have been away you’ll find some other person has replaced you. She is the one on the seat. You reach there thinking you’ll be looked for like before then you take him… [Cross talk]

PB04: As number 04…

PB07: … Sorry just continue.

PB04: As number 04, I have… slightly, question. Read it again for me.

**I: Oh… I repeat the question. Let’s say I have left sex work, then I come back. What bad thing will happen to me when I come back?**

PB04: There, you see even when you are saved and you backslide, it becomes worse. One comes back… you left the job thinking the other side maybe the business or marriage will work. Then you realize it doesn’t work. They usually come back with all the… so it messes one’s life.

**I: How does it mess the life?**

PB04: Now, when she comes back she comes fully. Before it was strictly business that is what we were saying before that one comes with dust up to here (laughter). She has come back with her mind fully made up after being disappointed out there where she had gone to as she left work. And those are the things they go through.

**I: Is there anything anybody wants to add?**

PB03: I can add to that, as number 03. You know when she comes back she comes back bad intentions she will not even look at the time. If she used to start at 10 in the night till morning. She can now begin at 2 or 16 like she can meet different clients. She can meet so many clients in 24 hours.

PB05: To compensate for the time she was away.

**I: And what are the positive things that can happen when they return. Anything good that can happen on their return?**

PB04: Good? There are very few good things.

**I: Good things are few.**

PB04: As number 04 if she comes back maybe she may make it in life as she had planned before. May be.

**I: Positive things that can happen on their coming back?**

PB04: Or maybe she may get a good person finally. Who will get her out of this work for good now.

**I: Okay, what things do female sex workers would like to accomplish before they leave sex work? Number 06**

PB06: May be I would say she had a target, she was working while saving targeting that “I would do this and that”. So when she leaves the job she can still maintain herself, she will still go on with her life whether she leaves sex work or not.

**I: Another, is there a target, something you want to achieve before you leave sex work?**

PB07: Number 07, if I get money to open the club, I stop

**I: Number 05, do you have something to say? None. Number 03, number 02, number 04? You are really tired. Now, do you know those who have left sex work in the last 5-10 years?**

PB04: They are crying. Most of them are crying. There is one I know I can say about 2 years it about 2years…

**I: …5-10 years**

PB04: No that is like… yeah he is approaching 5. But she is crying, she left because of corona, the curfew and stuff and has gone to the casual labor. I think the money is not sufficient, although she has not shared with me but she is really crying. She is just suffering there is nothing good I have seen people experience. If you had not saved and start any sort of business you suffer.

PB02: Number 02, I know someone who left but she is living well. I can say she left in good luck. She left and found a man who took her and he is taking care of her and supports her. So leaving happens.

**I: Do you know anyone who has left in last 5-10 years? Okay, what made it easy for them to leave?**

PB02: As number 02. She left because she got a man. You see if you get one who has surely seen your state and he decides. There are men out here who desire women to live with. You see, so he saw… leaving is not just finding a man and there and then you have left your job. you get to know each other for long first and discuss and agree.

PB04: As number 04, corona

**I: Corona.**

PB04: The curfew.

**I: What did corona and curfew do? How did it make it easy?**

PB04: Like the one I know, the curfew made her leave and she has never come back.

**I: Is there any difficult they faced?...**

PB04… [cross talk] During the time?

**I: Yes.**

PB04: You see the time was limited, people cannot be outdoor. you will find them… The one I know, she was parking on the street in the cold, you can be canned if you are found standing in the street. So she stopped.

**I: She left. Now, I want us to talk about Jitegemee intervention.**

**Note taker: You said she is always crying and has a lot of problems, how is she coping?**

PB04: like at the moment, the one I know and was talking about is working as a house help, she does house work for someone. Like I told you earlier, in this field there is a lot of laziness. She was used to waiting for the night to apply makeup and go out there. In this one she has to wake up early in the morning to someone’s house and do all these chore… and she leaves in the evening by the way but it’s a lot of work, doing laundry washing dishes and all that I think that’s why she is crying. And the pay she receives is not the pay she used to get. I think that’s why she is crying.

**I: Now we want to talk about Jitegemee intervention. Earlier on I described Jitegemee intervention and I said the purpose is to ensure that sex workers have some savings that would allow them to say no to unsafe sex or to take a break from sex work if they want to rest. I also said it will entail female sex workers saving a small bit of their own money to use when there are no client or help them prepare for life after sex work…**

P: …Is it over?

**Note taker; this is the last one.**

**I: This is the last one, after sex work. So, Is Jitegemee something other female sex workers in Kenya would embrace?**

PB04: We have not gotten that clearly.

**I: Like we want this Jitegemee… we want to start up Jitegemee intervention, as we said we want female sex workers to save their own money and when they quit sex work at least they have some fall back. So, is this Jitegemeee something other female workers will like or how will it be?**

PB05: Okay as for me, it’s a new idea and some people may not have such an idea they are usually just there. So, if you bring such an idea they will be like “oh, so such saving exist, we can save money” maybe others are wondering “when I am done with this job, where will I go, when I leave this job because it will reach a point where by my age won’t allow me to continue” so you’ll find someone saying “so this thing when I am done with I can save money and when that time comes I can leave and still be fine”. So, for some people they do not have such an idea as for me I think it will be accepted.

**I: Another person with another reason?**

PB07: I think any open minded sex worker will accept it because like when I look at this Jitegemee intervention it gives people that chance and knowledge of knowing that the money you get can do a lot besides the things you are using it for. You can save and do a lot of stuff. So I think anyone who is open minded towards that can accept. Though they are stubborn.

**I: So, what type of sex worker can embrace it and what type of sex worker will not embrace it? Like which type of sex worker will embrace the Jitegemee idea? ...**

PB07: …I think, I think this age group right here because we have a lot to achieve. Those who are older have already gotten most of what they want and are just doing it because they have to. So I think this age group, a lot of them will embrace it.

**I: So, let’s say you have your 10 sex worker friends and you tell them about Jitegemee, how many among them do you think will accept it?**

PB04: As number 04, three quarter.

**I: Three quarter.**

PB05: For me is think it will depend, even all of them may accept. So it will depend on how you explain it to them. It explaining like “guys there is something here called Jitegemee that has come up and it goes like this and we ought to do this because we cannot stay here forever and we are hoping to have something in the future”. So it will depend with your patience and you explain to them how you understand it.

**I: But now we want to know how many among them will accept this Jitegemee.**

PB05: For me I see like they all will.

**I: All.**

PB02: I see, you know in this job it depends with the challenges that one faces, you can get 10 people and you wish to talk to them to change and leave this job. but if you get those who have not faced any challenges they will be like “this money is good.” However much you talk to them they will not leave. But if you get one who has faced challenges that have affected her so much if you talk to them they may leave.

**I: So, how many can leave?**

PB02: I can say if you find those who have gone through challenges, all will leave.

**I: All will leave. Who else?**

PB03: Number 3 I can say 8 out of 10

**I: 8 out of 10 will accept?**

PB03: Yes.

**I: Now number 04 had said three quarter of them will accept, roughly that will be 7 out of 10. So, why will the 3 not accept?**

PB04: Eh! (laughter) As number 4 you know people reason differently. Want will convince those three quarter to accept.

**I: the 3 you not the three quarter. You said three quarter will accept…**

PB04: …The three, the three it will not be easy for them to accept somehow even if you were 10 people there has to be that one who will not accept, however nice that thing is. That is their nature.

**I: And number three said 8 out of 10, the reason the 2 not accept?**

PB03: the 3 may refuse because as number 04 had said. There those once they get into business they become lazy. They do not want to work hard. The 2 you may find are those who don’t want to work extra hard. They want the job they just go and get their money [inaudible].

PB04: In addition, those 2 or 3 the business could be doing great for them. Even when you go to preach to them whatever you will find they are flopping (laughter).

**I: Okay, so what can we do to increase the acceptability of Jitegemee to as many female sex workers as possible. What can we do to reach many sex workers about this Jitegemee.**

P [inaudible]

**I: And they accept it.**

PB05: As number 05 you see to reach people currently is through social media. That’s where you can reach them, another thing is to educate people. Those who… it is not a must you have to look for the sex workers to educate them. You can use ordinary people. Like for example I am an ordinary people, and her she is a sex worker. I am an ordinary people but I have a plan to do what? To get in. you see I am not a member I was an ordinary person but I have a plan. If we are educated youths are called somewhere and educated “there is something called this and that” even if you are planning to do that thing or you are there already “this is what you want to do, do this and this” you will reach many people and you will make someone get in with a mindset “oh, so if you go there you can also save”.

**I: What else can we do to increase acceptability of Jitegemee? (silence) Nothing?**

P: Yes.

**I: What can we do to implement it so it is most effective. The Jitegemee (silence) we want to start it right?**

P: Yes

**I: What can we do so that it is effective?**

PB04: As number 04 people like money, for example myself. Now if these forums can be put so and when the training will be on going, you see the teachings sink well when there is something small you are leaving there with to go with in the house. So you will be eating your ugali as you say “ei yawa! Those people taught as well there, this thing in need to go about it like this or that”.

PB05: But there are people who come to the forum to just get the money but they will not change, they are who they are if they have decided “I am not interested and that is that.” They will be like “are people going?” when they hear that people are going… they are the first ones to put down their names there. Any other thing like for example “we were asked to do this or that, ah! That not now let it wait for the next meeting” Yeah, there are such kind of people.

**I: Is there anything you want to add to that?**

PB05: No.

**I: None. And now what should Jitegemee comprise of I mean like components to be acceptable. What should we have as Jitegemee people for you people to accept it.**

PB07: I think maybe you need to have a platform for saving money. Because if there is someone who will be interested in joining the group she may want to know “how do we save this money once we get it?”.so if you have that platform where people save that money they will see it as worth it.

**I: Another thing which we need to have?**

PB04: May be being conscious of time.

**I: Time.**

PB04: Time should be good. Because again if something is over stated then you leave the place tired but if it’s not overstated then it remains… it sticks in the mind.

**I: Is there anything else we ought to put in place?**

PB07: According to what number 07 has said. You can have something like Jitegemee savings account. If it’s something of the sort. Then that will work out.

**I: Why will it work?**

PB05: Because, if it can also be accessed via phone, you see someone will be like… ah! she has heard about it. It’s something maybe has gone to. or she has heard about it somewhere and she goes to her phone and finds the saving and jitegemee is there, she will be like “ooh this is the jitegemee” there is a way she will get encouraged and will be like “oh so this thing was for real.” She will feel like one of the founder members (laughter). They will join so fast.

PB04: it can be a Jitegemee but not affordable, so will be like “you are better of going to KCB”. So you need to make it affordable for us.

**Note taker: When you say affordable what do you mean?**

PB04: As in… this other whatever saving they have a very high interest. So, if you can put the interest less than others by some margin, it will be well.

**I: Okay.**

Note taker: Just for us to clarify Jitegemee does not have any interest you save your money the amount you want, when you want it, how you want and you withdraw it the same way. =Nancy= had explained this to us. And you withdraw same, how you want it when you want it.

PB04: If that’s the case then that’s good. (Laughter)

**Note taker: There is nothing like if you have not saved there is a fine charged.**

PB05: Then that is like savings account.

**I: Yes, it’s like savings account you can deposit anytime…**

PB05… and withdraw anytime. Then that is good.

**Note taker: There are no withdrawal charges nothing…**

PB04: … That I can join even tomorrow (laughter)

**I: Okay. Now what will they not like about Jitegemee, the female sex workers.**

PB05: How?

**I: Like we want to start this Jitegemee what will not interest them.**

P: We are not aware

PB05: Okay, I could say if they put an account like Jitegemeee savings account and then there are tax charged all the time. If they deduct even if it’s a coin in a month the person will notice and will be like “ala! ” these people are deducting our money and that is how they leave, just a bob deducted in a month even though the rest of her money is safely kept she will withdraw all her money and leave “laughter”.

**I: Another thing, what will they not like?**

PB04: Dishonesty (laughter).

**I: Like?**

PB04: Like the way you have explained nicely that there will be no deductions, then shortly you find a phone call “kindly come shortly” (laughter)

PB07: Abrupt changes.

PB04: yeah, that will not go well.

**I: What else will they not like? (silence) Okay, now will they like about Jitegemee.**

PB05: Like?

**I: Jitegemee.**

PB05: If it allows issuing of loans. Eish! That they will like! (laughter)

PB04: And they do not disturb me like =Mshwari=, sending messages in the morning evening. Send me a message one in a week. Yeah.

PB07: Do not be like =Tala= “you took our money we are coming for you!” (Laughter) Until you are afraid of picking the phone. You could be in the bathroom taking a shower thinking it’s your dad calling only to find its =Tala= on checking your phone. (laughter)

PB05: In my view if you have to take a loan, let it operate like =fuliza= when you deposit its deducted. If you deposit let it be like =fuliza= you deposit… someone sends you money and it’s deducted. Yeah.

PB04: To operate like so?

PB05: Yeah It will help you…

PB07: …it will help you to forget that issue. If you its paid that loan issue is finished…

**I: …Oh… it should operate like fuliza.**

PB05: Next time you can fuliza again.

PB04: No, I do not want to be like that (laughter).

Note taker: How will you pay it?

PB04: I will somehow pay it, when it’s time to pay I will pay back. Now if they deduct the little that have been sent for to restock my clothes…

PB02: That now will depend on you, you are able to tell on this line I used it for fuliza now do not send on this number (laughter).

PB05: You see what happens which is not good is, say I have taken some debt on that account from somewhere… =Mshwari= does not deduct right? They just send you messages. You know next time when you will be in need you cannot go to that =mshwari=, because you have not repaid the loan. But =fuliza, you will go back because you already paid the debt, you will go back there. So for me I see if it will be like an account let it be like =fuliza once you =fuliza= once you take money gets as its deducted…

PB03: … [cross talk]

**I: You see Jitegemee will not be giving loans.**

PB05: so that’s why we were saying…

PB03: …We are suggesting

**I: Oh… you are suggesting, it operates like… like =fuliza=…**

PB05… we were saying things that will attract people. So, what will attract people is where loans are given. Automatically it will attract people.

I: Okay, Alright.

PB04: And ten this money should be given individually, just one person no like “come 10 people”.

PB03: Like a group no.

**I: Alright, you have already told me what they will not like about Jitegemee, if there is dishonesty. Then what will be the ethical concerns among female sex workers about Jitegemee. Like concerns… ethical concerns are your rights as a female sex worker. So, are there any concerns about your rights if you join Jitegemee study?**

PB04: Say it in Swahili.

**I: (Papers shuffling) Could there be ethical concerns among female sex workers about Jitegemee? Like if you were to join in the Jitegemee will it infringe your rights in any way?**

PB03: Because, oh, Number 03, according to how you explained to us initially I think there is some privacy. It is private so I am not worried. Yeah.

**I: Another person, are your rights going to be infringed because you are in Jitegemee?**

P: No

**I: Why are you saying no, number 04?**

PB04: It’s alright because by the time I am… I am number 04, it’s alright because by the time I join in the whatever I would have read with you, you have explained for me and I have joined voluntarily.

**I: Alright. And now and what challenges do you think will we face with such an intervention? What are the challenged will we face when we start Jitegemee?**

PB05: When you start…

**I: Number?**

PB05: Number 05, Once you start something and when it is still new to the people there will be some will decline from the people. “Do this people want to con us with this thing?” so that will be the challenge. People and money, people value money, “this thing, this people, and this is a new thing…” they will start to compare it and do investigation. So that will be the challenge people will have fear about the money.

PB04: As number 04 I have not heard number 06. She has a good voice and she has not shared (laughing)

**I: Number six, tell us the challenge we can face.**

PB06: Like, Jitegemee will be something new to people so for those who do not know about it, we shall need to explain to them a lot more so that they understand Jitegemee so they do not be afraid of putting their money.

**I: Is there someone with another challenge that we may face?**

PB07: I think the number 05” idea Number 07. The idea of loaning may be challenging in a way because people will come with “eh! I save today and tomorrow I will access loan”. So, I think you will have to give people time and depending on how they save to determine if they are serious with what they want. But if not giving loans to whoever joins I think it will become a very big problem.

**I: Alright. You have given the challenges and solutions so we shall continue. So, how much will they be willing to save per week without interfering with basic needs. Like how much can you save for those who want to save per week without interfering with their basic needs? Like still you can have your basic need you can save and still you are having your basic needs. How much can one save in a week?**

PB04: As number 04 200/=

**I: 200, number 02**

PB02: 500/=

**I: Number 03**

PB03: 300/=

**I: Number 07, per week savings per week**

PB07: 500/=

**I: Number 06**

PB06: 500/=

**I: 500 number 05**

PB05: 500/=

**I: Now, if sex workers are not able to meet their set targets what other ways are they able to meet that? Like you have said you can save 200/= right? And you 500/=, the other 500/=, 500/=, 500=, 500/= right? Let’s say you have not met the target, what can you do? So as to achieve that target?**

PB06: As number 06, initially you said one needs to have side hustle that can boost you. Because you don’t get clients daily. So, from that side hustle I thing I can get 500/= and save.

**I: Another?**

PB02: As number 02 I think… let’s say in a week I have not achieved that goal of 500/=, that… maybe that week was not a good one the second week I may double so in case I double I will feel the gap that remained back there.

PB05: Okay as number 05, so, for me I see if you have … have my target like this is what I want. You usually are like “if I do not achieve to get this I want to get this. So you say in a week I just want to save that 500/=, right but you also tell yourself plan B if I don’t manage that 500 at least let it be 300/= but let the week not go by without me saving.”

**I: Is there anything we want to add on? So, how should the savings be kept where female sex workers can trust the safety? Where or how should we keep the money for you to…**

PB04: As number 04, bank.

PB07: As number 07 I have another option maybe if you can have a website and then everyone has their domain and there is a way I can access the web site and see how much money I have the money I deposited today has reflected.

**I: where else do you feel your money will be safety… safe. Apart from bank and the website.**

P: Savings account

**I: Savings account that’s in the bank still right?**

P: Bank.

**I: Is there something anybody wishes to add? (Silence) You know when you do like this the recorder does not capture that. (laughter) Nothing?**

Chorus: Yes

**I: Okay, those were the questions we had for you thank you very much for taking your time to participate in our discussion today and for sharing your views about Jitegemee. Thank you, thank you thank you and the interview has ended at 15.04**

P: Welcome, welcome, welcome.

End of interview
