# Supplementary material for: Eat a little and save a little: A qualitative exploration of acceptability of a potential savings intervention to reduce HIV risk among female sex workers in Western Kenya
Source: PLoS One. 2024 Dec 19;19(12):e0310540. doi: 10.1371/journal.pone.0310540 (PMC11658496; doi:10.1371/journal.pone.0310540)
Supplement: S1 File — (ZIP) [file pone.0310540.s001.zip › Jitegemee Transcripts and Dissemination Notes for Journal/FGD D.docx]

**DATE OF FGD: 25/APR/2019**

**INTERVIEWER CODE: KSM 006**

**NOTE TAKER CODE: KSM 004**

**FGD ID: FGD D**

**START TIME: 1615HOURS**

**VENUE: TYP**

**CATEGORY: ABOVE 30 YEARS, URBAN.**

**I: Thank you this is FGD D and we have started the audio recorder. The interviewer is Jane Moraa and the note taker is Lilian. The FGD is starting at 4:15pm. We are… the number of participants is 10 and it is done in Swahili. Thank you and welcome. Ok, from my short description of Jitegemee, what are your first thoughts?**

D1: Me as number 1, my first thoughts or my opinion, this work of sex work, yes, I trust myself I can leave it. But then it reaches a time when you think you can leave it but what will you eat if you have not relied on yourself properly? Because you know with us, like me I don’t have any other income. I depend on the 500 from there so that I can go and buy food. I can use 300 for food and 200 on books. Because I have children and due to that sex work is when my children are able to go to school. So, me I was just asking myself if this Jipange can help us as a woman who has decided to leave sex work, can you get capital to start a business so that you can sustain yourself in life from that point? Because if I have gone to meet a client, and the client has given me 500, this 500, 300 cannot be enough for me and then say that I save 200. I just usually feel that if I save that 200 the child will remain without going to school or maybe I will lack something. That is why we usually decide if we come back wit 500, we use that 500, because we have no other business.

**I: Thank you. Another opinion?**

D2: Me as number 2, I can also contribute saying, saving you can save yes. You can use 200 and save 300 or you can use 300 and save 300. But we have children in school. When you have saved the child is already sent for school fees. When you have saved, the child becomes sick. So, different problems come up. It can also happen that you are sick and you have not gone to your work, and it is just you. You are the mother and the father. So, this saving is difficult.

**I: Thank you.**

D10: And me as number 10, I will just contribute. It is a must… at first before COVID there was a lot of money in this sex work. Now the money has reduced. If we can be able to save, if you get someone who can explain to us how to save what we can save and the other to eat in the house. If you can help us, the way I have seen… I have read and seen some information there at the front, you help us so that if we can go to work and save a little, so that in 2 or 3 years we can leave sex work and use the savings that we…

D: Me as number…

**I: … you didn’t finish, the saving that?**

D10: Aah the saving that we… (laughs) sorry, the saving that we save to look if there is a business that someone does so that we stop sex work.

**I: Thank you.**

D9: Me as number 9, I feel that if you decide to rely on yourself, like me I can depend on myself but how do I depend on myself? I depend on myself if there is a way, I can meet my needs apart from dong that sex work. Because sex work also, honestly like now there is no money. When you go with someone, he wants to beat you, he has used you he wants to beat you. If you had agreed on the money, he doesn’t want to give you that money. So, there is… there are a lot of trials in that work also. By the time your children eat you have gone through what you did not expect in that work. You talk nicely with someone then eventually they disappoint you. Sometimes if you get… you want to leave that sex work with some reasonable amount of money, you use your brain. Maybe you have been bought for alcohol and they are more than 3 or 4 bottles, your work will be to hide them. So that you go and agree with the bar tender to and they give you the money. So that when you combine with the 500, when you get there, you can be able to even save. But if it is only 500 shillings... like me if I get only the 500, I cannot even put 100. Because starting from water it is money. I don’t live in a house where there is water or what, because even if you live in that house at the end of the month you will still pay water. You buy water, you buy matchbox, you can’t eat good food with that 500. You will have to take strong tea, you will have to eat vegetables so that you get that money to be enough for flour and children. So, when we find people like you and you can get us out of this work and help us, you see how you can get us out and give us money for business, even selling groundnuts, there are those who God has blessed them they are selling groundnuts and educating their children, so if we can find a way to leave this work… me if I get a way I will leave. There is nobody who enjoys this work. Yes.

**I: Thank you. Is there any other thought?**

D3: Me as number 3, for me personally, this work of ours is not easy. Sometimes you can get, sometimes you don’t get. So even when you leave your house, the children also, even to the neighbors where we live it is embarrassing. If the child disagrees with the neighbor’s child they will receive the abuse. Your mother does prostitution. So to someone like me, I would prefer that even if it is 2 or 3 years or whatever time it takes, help us at least, you can look for people like us, even 10 groups, if there is a way you can help us even with this small loans we can benefit because this business, it was good in the industry before, but now it has no benefit, it has no benefit at all. Because when you go with someone’s husband they will tell you that “eh, you know even my wife wants school fees.” You were also hoping to get your school fees from him. So, there is a big challenge there in this industry. That is all I want to say

**I: Thank you. Okay, let us move on. What is the usual expenditure that female sex workers usually do and approximately how much does each one cost? Expenses, which expanses do you usually make, what do you spend on typically and how much does each one cost?**

D1: Me as number 1, if I get to the side of food, the food only if I say breakfast, lunch and supper, I can use even 1000. Because maybe breakfast I want milk tea, egg, bread. Lunch time we will also eat vegetables, if we eat vegetables you have to have an accompaniment. Maybe at night I want to eat Chapati, you see, and the current world with a bad economy. When it comes to clothing I will see “this ones has a dress of 1000 will be the only one left behind with my secondhand worth 50bob. I also want that one of 1000”. I will put myself that my clothing I just put on for the 1000. Lotion, I can’t apply petroleum jelly, I want to apply Carolite, I want to apply Setrolite, and all that costs around 600. There is another one for 1000, but now you *scratch yourself according to the size you your hand* - cutting your cloth according to your size.

**I: I would like us to talk about daily expenses the way you have told me number 1, that you would like to eat good breakfast, how much does it cost you for breakfast?**

D1: Breakfast if it is just me and my children, I can use even 350 shillings.

**I: Lunch time?**

D1: Lunch time like now half a kg of meat is 200, flour 120, I have not added charcoal. If I add in charcoal, 70 or 100 shillings. Then I add onions and tomatoes.

**I: What other daily needs do you have apart from food?**

D1: Apart from food, no. Because if it is clothes, I have. I can’t buy clothes every day, I can’t buy lotion every day. The daily needs are just that, food.

**I: Who else wants to contribute in the daily expenses that they do?**

D: Me also… there in the needs also, there is a small challenge also.

**I: Number?**

D3: Number 3. Because here like us, for example like me I have children that go to school. You have not given them fare to go to school, you have not looked for tuition, you have not looked for books. When you look in your pocket, that five hundred is what you are left with. That breakfast, I don’t see enjoying it, because when I think of even eating that bread, I don’t have sugar. There is the price of sugar, there is the price of milk. My child has not reached school. So, it will find that many days or a lot of times I deny myself so that the needs of my household can be met. But it is still difficult.

**I: Thank you. Now you have talked about transport for the child to go to school, how much is it?**

D3: Let me say where she goes together with lunch is 100. And that is money that you make sure you have every day, Monday to Friday.

**I: And on food, how much do you spend?**

D3: Me on food, if I deny myself I can use 400 in a day. Which means in a day I can use 500.

**I: Thank you for your opinion number 3. Another person, daily expenses?**

D7: Me as number 7, transport for school I can budget for like 300. Breakfast 400, lunch 300, supper also 400.

**I: Thank you number 7. Is there anybody else with a different opinion that has not been mentioned on the daily expenses? Okay weekly expenses. Those expenses that you have once every week?**

D4: Me as number 4, weekly expenses, food you know is a must. You have to eat; you have to be clean. If you want to be clean you have to wash your clothes. You have to also buy your clothes. By the time you are buying yours you also buy for the children. You cannot be clean and the children are dirty, that is a must. You know how the economy at this time is difficult. If you are not careful in your life, even the money to buy soap will be a problem. Because now the soap that was 10 shillings is 35 or 40. So I feel that cost cause us to have to struggle with this work. Because if you just sit at the door there is nobody who will give you. And the children also like I have heard one of my colleagues say, number 3, said that they are abused by the neighbors. So, if you sit at the door let’s say today tomorrow I do not go to work, and the money that came with is spent, the children will sit and look at others like this. And you know now they will be abused even by the women when they go out. “How are you looking at us and your mother works, and every night she goes out. What kind of work does she do? You are looking at us… Get away from my door.” So, you see your child grows up already feeling embarrassed saying “what kind of life is this”. So I was personally feeling if there is a way to help us, it will be good. One of the way… it is not a must, if it is someone who can do business, there are people who are selling vegetables and they are living well, as long as they plan their lives well, if you can give us that money to start business it is okay. Or you can see, the way you are here, there are a lot of other work. You know this work is just forcing us. We just have to get some earnings. We have to earn a living. So even if there is other work you can just give us, I can leave that work. As long as I know that daily there is something I am getting.

**I: Thank you. I would like you to now tell me your weekly expenses.**

D4: In a week it is food.

**I: You buy food once a week?**

D4: I buy food every day.

**I: And the expense that you have once a week?**

D4: You have to do shopping in the house if you want to have an easier time. You have to do shopping if it is minimum, it is 3000. The things that you have to have in the house. So, you are just left with buying food every day. Apart from shopping, in a week like me in my house you have to buy something for the child. You can’t just sit that “eh the one that I bought last year is the one that they are with”

**I: What is something in this instance?**

D4: Things are many. Clothes, panties, if you have big girls.

**I: That are the weekly expense or monthly?**

D4: Weekly

**I: Every week you have to buy them?**

D4: Yes I buy them.

**I: You have to buy for everyone?**

D4: If you many of them then you cannot buy for all of them at once. You have ten children, you cannot buy for ten children monthly with that money, it is little money. So, you decide this week two, next week… like that like that.

**I: Aah, so every week you have set a side this week I buy for this child and the other week for another?**

D4: Yes, like that.

**I: And how much do you use?**

D4: If it is minimum in buying them things it is 1000.

**I: It is 1000?**

D4: Yes.

**I: Okay, thank you. Is there anybody else with an opinion that has not been talked about? Okay and about our monthly expenses that we have in general how much can it be? Average in total how much can it be?**

D10: Me as number 10, I can say I pay rent monthly and I live in a house of 3000. That month you have to know that you pay rent every month. Monthly expenses if I just do my calculations, eh it is a lot of money. Because if you calculate for breakfast, you calculate weekly, you calculate lunch you… calculate supper, ai! it is a lot of money.

**I: This breakfast you have done it once a month or?...**

D10: …By the way I don’t eat lunch. If I cook tea for breakfast what is left, they buy mandazi at lunch time, they eat. I save. Do you get me? I don’t go like this or this, I save. We have to save. If you don’t save, like COVID came the other day money has decreased. Now even if you get a client, he can’t give you 500. He will tell you to sit down short time 300, you take. You cannot leave it. So, you eat a little knowing that life is difficult you have a little. You can’t just eat all of it surely.

**I: That is why I am asking, the expenses that you have monthly. If I say monthly, I mean once then it waits until the month ends. How much have you told me?**

D10: 5000 every month.

**I: For rent?**

D10: For rent 3000, 2000 for food. You have already done your shopping for the house so [inaudible clause]

D4: Me as number 4, in a month, things are a lot. First rent you have to pay secondly at the end of the month they want school fees. I have to pay that one. In a month, maybe the shopping I had done was for weekly it was just a little for 3000. I have to do another shopping that will push me. You know sometimes you come back from there and there is no work, you have come back with even 200. So, if you had done shopping for the whole month or some things, at least you have an easier time that even if you have that money, you can put there. In a month maybe you have a child who goes to school and you have already agreed with a motorbike person that you will be paying monthly, the month has reached you have to pay.

**I: So if you put it together these your monthly needs at once**

D4: It reaches 10,000.

**I: Thank you so much. Let number 6 speak first.**

D6: Me as number 6, I can say my monthly needs, I pay electricity, water, house, school fees, transport and maybe lunch at school for the child, there are others that are written every month. So if you look at that, it can reach even 20,000.

**I: Thank you so much. Another person?**

D2: Me as number 2, I can also contribute. Because to save, saving is difficult and with the life of today saving is difficult. Like me what helps me, after coming back from that work, I go to the women’s chamaa, even if I come with that 400, I will contribute 100 and pay chamaa. So that day when I receive my money from the chamaa is what helps me to do shopping for the house, you will find that I have bought bar soap, I have bought 3kg of sugar, I have done a bit of shopping, I have bought tomatoes, I have paid rent. So, you come and find the money that you can use in a month is a lot of money.

**I: So, in a month the things that you do once, they total to how much? How much are they?**

D2: Those things that I can do in a month starting from 1^st^ to 30^th^, can take even 25,000. There are times it can even be 30,000 if you pay school fees for the children. If you buy school shoes. Like now in a month, we use a lot of money, and we don’t have right now. Even the children have gone to school they don’t have books, you just tell the child to go God will provide we will get.

**I: Thank you, number 2.**

D7: Me number 7,

**I: Oh, number 7**

D2: I am number 2.

**I: Sorry number 7**

D7: My monthly expense can be 30,000. Because if you put rent, let us say 5,000, school fees you put 5,000, transport you put there like 3,000, school lunch, electricity you put there 700 in a month, clothes also you have to put uniform, books. It costs around 30,000.

**I: Thank you so much. Now I would also like we talk about the expenses that we have once maybe in a year or after 3 months or half a year. Are those ones there?**

Chorus response: They are there.

**I: Like what?**

D2: Me as number 2, I can say I can maybe I don’t have mattress I can work hard and buy a mattress. You want to buy a nice cloth in the house. The things that you want. Maybe the children watch television at the neighbors, the neighbor chases them away. It will force you to work hard to buy the children TV so that they are happy in their house.

**I: If you look at those expenses, approximately how much do they cost?**

D2: It takes a lot of money it can take even 30,000.

D: Even 50.

**I: Thank you. Is there anybody who wants to contribute to the expenses? Okay thank you, thank you. Let us continue. Where do most female sex workers get the money that they spend?**

D1: Okay, as number 1, the money I get for using, when I have gone for sex, that client of mine the money that he has paid me is what I will use.

**I: Are there ones that you can say are primary sources, then there are those that are secondary, other sources, right? In English there is main hustle and side hustle, right? There is the main one that brings more money and there is that brings little money but it supports. I don’t know if I have explained myself well up to that point?**

D: Mmm

**I: Okay. I would like us to talk about it. Okay tell me we do this and this. That is the main hustle and this and this those side hustle or those that are secondary. Okay explain for me. Yes, number 5.**

D5: Me as number 5, main hustle is this sex work, then side hustle I have a small salon that boosts me if the other side is not doing well.

**I: Now like your main hustle can bring in how much in a month?**

D5: In a month?

**I: Yes**

D5: In a month if I calculate it can come to 27.

**I: 27 [this means 27 thousand KSM006]?**

D5: Yes. That is if work has been good.

**I: side hustle?**

D5: Side hustle, that one brings in 1000 per day.

**I: And how many days do you do it per month?**

D5: That one I do it Monday to Saturday. Sunday, I rest. You know even if we are doing this work we also go to church. So, Sundays I go to church.

**I: Thank you. now that is like 6000. times… 6 times 1000. That is like 6,000 in a week?**

D5: Yes

**I: Another person? Number 3**

D3: Me as number 3, the work that I trust myself with that I know when I go out, I will come back well is sex work first. Besides that, I cook chapatis in the morning, at least to get the 100 that tomorrow the child can use at school when I still don’t know what I will get in the evening. So, now its challenging as well because when you cook that chapati ingredients are expensive. You see like maybe I have gone at night and I have gotten even 1000. That 1000 when I come and calculate and invest it in this chapati business I feel that it is not enough. So, I feel on the other side, I’d rather go and preserver the cold there at night, I can get 200-200, 300-300 if I collect at the end of the night I have money.

**I: So, you have said that at night you go parking?**

D3: Yeah

**I: What is parking?**

D3: My main source.

**I: Parking is like sex work?**

D3: Yeah

**I: Then you have said the other one is for making chapatis?**

D3: Yes

**I: So, parking can give you only 1000.**

D3: Yeah

**I: And for making chapatis?**

D3: Making chapatis sometimes you can get you have only gotten 200.

**I: Per day?**

D3: Per day.

**I: Thank you. Is there anybody else who wants to contribute? Number 8 please (long pause)**

D8: I am tired

**I: Is there anyone else? We continue. What reasons make female sex workers spend on… oh sorry I have gone to another question. What reasons make female sex workers to spend money on the things they spend on every day? We have talked about spending that you do?**

Chorus response: Mmm

**I: Okay, the spending that you do. We would like to know what makes you spend the money especially on the things you spend on daily.**

D: That question… Repeat that question

**I: What reasons make female sex workers spend on the things that they buy? Some of us have said food, said certain foods, and there is someone who said she cannot use her money on this type of food I will use on this. Why? Have you understood me?**

D: Mmm

**I: Is it now understandable?**

D: Mmm

**I: If we choose like food, someone has said she spends on certain types of food and the other says a different type of food because she will not use on the food that the other has used. Like number 3 was different from the other. You remember when you gave your example?**

D: Mmm

**I: So, if you compare with that what makes you decide to spend on the things that you do?**

D1: Okay, me as number 1, I can maybe today I have gone to work and I get a good one, I will tell my body sorry. Maybe the work I have done, I have done difficult work and I get that I have received good payment and my heart longs for chicken, I will go and take that money, buy chicken and tell my body sorry. And my children who make me go out, I will also make them happy buy them rice, take them to the swimming pool because the work that mom has done today is just to tell her body sorry.

**I: Thank you. number 1 is saying the way she decides how to use money depends on the amount she has received. Another person?**

D7: Me as number 7, it gets to a point sometimes you have gone for a side hustle, you have gone and done your business you have not made any profit, so when you go for sex if you get something small you will have to use that money because you were not successful in the side hustle.

D4: Me as number 4, in using money it depends on how you get. You know, the way we are all here there is no one who would agree to live in that bad life. It is just that it depends with the amount you get. So, if I get a little that is what I will manage with. That is what I will see, today I have 200, what can 200 bring? Provided that I don’t remain without getting anything at all. Because today if I get 200 tomorrow, I get 1000, I will not eat vegetables again. I have to say my body today you have brought something let me tell you sorry the way one of us had said. So, we use based on the earnings.

**I: That’s on you ? Telling yourself.**

D4: Mmm

**I: And to others?**

D4: To others me as number 4, I cannot buy chicken and eat it alone (laughter). I will buy chicken and eat with the children. So, I feel that it depends with your earnings. Today if I get 200, I will say, I will get in the house and say that today only vegetable is available let us just eat what we have. Tomorrow if God allows and we earn well, we will eat chapati the way others are doing.

**I: Apart from food is there any other use that you do? Number 3**

D3: Me as number 3, sometimes I can be lucky and get a client, meaning you can’t say everyday is a Sunday. But if I get a good client, the first thing, I have a child who I pay school fees for, I will make sure I have cleared my child’s fees. This is because I don’t know how tomorrow will be. So there is where, if I get like that, because you will not get lucky every day. You may get lucky again even after three months. So, on my side if I luck out like that, the first thing I usually think is to complete my child’s fees first my mind to be relaxed.

**I: Number 6**

D6: On my side I see that I will even pay rent. You know you can pay rent… upfront by even 5 or 2 months, it will depend with the money that you get.

**I: Okay, thank you. Is there anybody else who wants to contribute? Okay let us continue. Do female sex workers usually save?**

D1: Like me number 1, saving is not easy. The reason I am saying it is not easy, I may have been lucky to make that 1,000 that house has no charcoal, no water, no vegetables, the children’s fare to school, you see, so I cannot save. You find that you have spent it all on food it is over that even any to get you from =Kaloleni= or =Manyatta= to take you to =Florida= is not there. It is not easy.

D9: Me number 9 here, also for me to save is difficult. It is very difficult because even if I come back with that 1,000 or 500. If I come back with 1,000 it will get finished, if I come back with 500 it will get finished. Based on how I will have planned myself. Even now my child, let us say, me my child has done exam, now if I get that money, I will rush it over to… the money that I had not paid in school so that at least she be given the paper to at least go to Secondary. She has done class eight exam. So now even if I get that 1,000, I will eat 500 and give the teacher 500. I try and talk to him, because she needs to pay tuition fee, there is a book she has lost, so now it is challenging.

**I: Thank you. number 6**

D6: I had an opinion saying, saving depends. You know some people can’t just put money, that today I have put one, two to the bank like that. Some use things like chamaa. Someone can come back with 500 and take 100 and put in the chamaa. So, the day she gets her money from the chamaa that is what she will say is her savings. That was her savings. So, there is where savings come from most of the time.

**I: So, like how much can you save in the chamaa in a month?**

D6: It depends. People have different chamaas. There are chamaas that people even get 100,000. There are chamaas for 5,000, there are for 200. Chamaa they are different. So, the one that is near you, that is pocket friendly to you is the one you join.

**I: So, usually for those people you know, they are in chamaa for how much per week?**

D6: Like for weekly, someone can get for 9,000, of 5,000, they are there.

**I: You pay 5,000 per week?**

D6: No. you pay 100 every day.

**I: Every day?**

D6: Yeah.

**I: Ooh. Is there anybody else with an opinion? So, to you a way of saving is through chamaa?**

D6: Chamaa

**I: Number 10**

D10: Number 10, I am saying in the beginning before COVID, this work was giving me money. True before COVID came this work had money. I would play in a chamaa you come and pay 200. You come and get number one or two you wake up and pay 400 in one day. In a day you get money you come and pay the second person. Then again tomorrow I will not pay, you have saved for yesterday and today. Then tomorrow again when you go to work you are lucky, when you come back 2 number. You see that it is not giving you work as long as you have put money somewhere in the chamaa. The day you get money you receive good money you go do your business. Now the way there is no money because COVID came and spoilt everything, so now you get a little. You don’t get the way you used to get in the beginning.

**I: So, I have a question, I would, like to know for those who are in chamaa, when it reaches the time to pay chamaa and you don’t have money what do you do? Number 2**

D2: Like me number 2, I have a neighbor who we get alone well, I go and borrow from her so that I pay that chamaa.

**I: Is there anything else that you do?**

D1: Like me number 1, I *fuliza [to borrow money via mobile phone app]*.

**I: You Fuliza?**

D1: Mmm

**I: Number 8**

D8: Even me I borrow. If I don’t pay… if I am late in paying, I take from the neighbor and pay. When I get it later, I pay back.

**I: Thank you. number 7**

D7: I usually go to the phone and get an MShwari loan or from KCB, I pay when I get I pay back.

D4: Me as number 4, it depends. Sometimes there in the phone loans it is dry (someone coughing) sometimes it is very dry. You may have already taken a phone loan, you have taken fuliza, you have borrowed from the neighbor until they are fed up with you. Me personally, if I have not received I just say “let it pass”. What you give is what you are given back right?

Chorus response: Mmm

D4: Eeh, I just say let it pass. I can’t force myself with something that I can’t.

**I: So, you they skip you?**

D4: Yes, if it is like that, I can even be number last. It is just that when my time reaches, whoever’s turn it was to receive the money that I did not contribute for she will also not give me. The ones I gave are the ones who will give.

D1: Me I have a question for my neighbor number 4. She has recieved the first number or even a middle number. Me I am following her. She has borrowed all those debts that she is saying, but she has already eaten and me I have not. So where will she get the one to give me. She has already fuliza, she has borrowed from the neighbor and she has eaten and it is my turn…

D8: And remember that behind her there are still 5 people remaining and she has already eaten and she has to pay these 5…

D4: …Me as number 4, I have already eaten her money and I have debts everywhere I can’t go and borrow again I go to the person whose turn it was and tell her “so and so I am overwhelmed, but I am requesting that you just leave me today and tomorrow I go look for it and bring to you” because work cannot be bad every day. Tomorrow I can go and get 300, I decide that I had already received from that person’s 100. I will have to take to her that 100.

D2: Me as number 2, there are those who don’t understand. Her if you ate her money, she will tell you “I want my money”. The way you received is the same way I want, my money I want to use.

**I: To female sex workers, are there some things that they can go… they go over and above that is they go beyond so that they can be able to pay this chamaa? Those are what I want to understand, are there things they do out there…**

D: … You sell the phone.

**I: You sell the phone, what else…**

D6: …If you have already bought some clothes…

**I: … number 6, mmm, number 3**

D3: Me as number 3, I don’t like being in a chamaa with neighbors because it will give me problems. I feel that I join a chamaa with those that I am with in the field because when you are in the field you know so and so has gotten a client, so and so has gotten a client, at least you understand each other as people in the field. So that is why when I join a chamaa, I don’t want to be in one that is so big that will strain me. I want to be in a chamaa with my fellow field mates, who know our earnings go like this and if you join a chamaa with a neighbor, it can give you problems some times. Me that is my opinion.

D: Okay, if I add, they say [?your things are what usually take you outside?]

**I: Number 3 you have spoken about field, when you say field what do you mean?**

D3: The people I work with at the parking know me very well. I also know them very well because we are doing one work and we love each other.

**I: Number 1, repeat what you have said**

D1: Me I am saying, what you own is what usually takes you outside. Me I have eaten your money maybe we will be playing *sindikiza [escorting].* You know there is a chamaa called *sindikiza* for carrying for each other. Maybe you have carried for me something meaningful and it is time for me to give it back to you and I don’t have. You know that TV of mine… and I will say let me take that TV and go and sell it so that I don’t do my partner wrong. I give her, her money because I know that one day if God opens the door, I will get a TV.

**I: Thank you. and for other female sex workers out there, are there other things they are doing apart from the ones that you have said? Your other colleagues. (Short pause) okay thank you, let us…**

D4: There are other things we do me as number 4. You know, now you are stranded you get a client, the client wants to give you 300 and yesterday you had money problems and you have debt everywhere, and he has come… his wallet has money, you will just have to steal it. (participants laughing) I will steal. There is no need for me to lie to you. I will steal because now he wants to be with me here and give me 300 and I am seeing 1,000 there and I have money problems, I will have to steal. And if he has a phone, I go with it and find a customer.

**I: Yes, number 3**

D3: Where my colleague has contributed, we do steal. Because that work is hard. You have agreed with someone 500. Me… for example me I have gone with someone’s trousers, yeah. Because he is asleep, he has put clothes over his head he has turned the pillow. So, for you to take the money, there is a strategy, there are drugs. I put for him. After I force him to drink it with the alcohol or even soda, I have already seen his wallet is good, my sister that day I have a wedding in my house.

D4: yes there is no need to lie to each other.

D1: It is true because even them when they decide to put us down, they put us down because they know that even us if we get them off guard, we will not give them space.

**I: Thank you. thank you for opening up and sharing your thoughts. I would like to know if there are certain behaviors of female sex workers who save? Are the women with certain characteristics that show these ones save? And they are sex workers?**

D10: They are many. Those of [?mucus?] are really clever. They come back from work, you, you had done [inaudible clause] she tells you ah give a small thing we save. You see here mucus, they have brains.

**I: Where is mucus?**

D: Here in town behind =octopus=

D: Okay, and if I add, you know these things have two prices. In front has its prize behind has its prize. So the day I will see that I have an Arab, Arabs don’t like to eat fish on one side. He eats the front of the fish, he eats the back of the fish. That day just know you have killed kenge. That one if you just agree to open the boot, ah you are just okay.

**I: Opening the boot is going where?**

Chorus response: Behind.

D: Eating from behind. (one participant laughing)

D: Having sex in the behind

**I: Having anal sex?**

D: It has money. It has money.

**I: Thank you. another one?**

D6: There is a challenge also we are facing. Like me there is a challenge I faced a certain year. I met an Indian, but it was good because I got what I wanted. But remember after that there is also treatment because Indians use a lot of chilies. So, when he releases, those chilies affect you. so, it will force you after they have affected you, you look for a doctor to treat you. I have the money but itching, ha!

**I: Okay. Thank you. women who save, do they have a certain behavior?**

D1: Yes. Like me I can say they have a certain behavior. They are like lesbians they do work how many times? Twice. There are us… for example like me, you know I don’t have the lesbian behaviour because I look at my earnings and that one I can’t. now this my colleague, she is a lesbian and has something.

**I: You have used her as an example?**

D1: Yes, I have used her as an example. She has something, you see. So, I will agree that she touches me, she sucks me, she puts her fingers in me but at least I get that thing from her.

**I: This thing is something to save or is it something to eat in the house? Because there is where I want us to understand each other well.**

D1: Okay. Now me I told you like me I cannot save. For now, I can’t due to the earnings that I receive. You know if I go everyday and get 10,000, 5,000, 4,000. That one I can save. But if that money is 1,000 or 500, ai! my friend I can’t.

**I: And you feel when you go to the lesbian?**

D1: If I go to the lesbian, lesbian can give me good money (laughing) yes, she can give me good money. The disadvantage of a lesbian, if I am with her, she will not want me to be with another person. She will just want me to be her only client. If she finds me with another there we will disagree. And me I will have to leave this man because the man might pay 1000 and I come to my fellow girl she might give me even 5 punch she does not give little money.

**I: What is punch?**

Chorus: 5000 (laughter)

**I: Thank you so much. Is there anybody else who wants to contribute? According to number 1, when she goes to a lesbian, for those women who have the behavior… she has sex with a lesbian and also anal make a lot of money so they are able to save. Have I gotten you right?**

D1: Yes.

**I: Okay. Now I would like to know, the women who don’t save, do they have certain characteristics and what are they?**

D1: We don’t save and our behavior also is stealing in the rooms.

D6: And borrowing

D1: And borrowing. Today I have borrowed from this person when I get this, I think about paying that debt. When will I save?

**I: I request number 6 you explain a bit about borrowing.**

D6: Borrowing?

**I: Yes**

D6: Today you don’t have right? You go to your colleague help me with 500 I will give it back when I get it. Just like that.

D10: And again, you find people like that, me as number 10 I am talking, you know that you are eating now tomorrow you will get. You don’t have that one that you will save. ah, today I have got 500 I will finish it because I know tomorrow, I will go and do what, I don’t think… you don’t think if tomorrow you will not get you can’t get every day. You have to open the mind to have something small even if it what. You save even 5… even that 5 shillings if you put it in the bank, when it comes to the end of the month it can be enough to pay rent. It is enough to pay rent, water you can pay with that five, five.

D4: You see me as number 4, saving sometimes is good. Even if you are saving ten shillings. Because you see not saving you will have problems. The problems are many…

D10… You ca not leave those things…

D4…Because now I have not saved. I knew very well I would get something. I don’t get, that day I don’t get completely. I will have to go there and borrow. They will know that you are a borrower. Because you can borrow until it reaches a point that they know [name omitted] is a borrower.

D8: They even give you a nickname.

D4: Number 10 is a borrower. You know now they avoid you. They will have to avoid you. You reach the neighbor, you will borrow there until you move away, and you move away with debt. Then =Kisumu= is small. You will move around =Manyatta=, =Kondele=, nyamasaria= it will reach a time that you will be found. So, I see that thing is embarrassing us. We would rather put a little with that chamaa that I was seeing number 3 was saying, that chamaa helps us. Because maybe it is a chamaa that they get the money in a week. So, let’s say that you were putting money in the chamaa, it reaches a time that maybe it is not my turn to get the money, but I know number 10 is going to receive, I go to number 10 and tell her my friend I am like this and this, I am pressed, give me this one of yours you will get during my turn because I know there are daily basics. The child wants to go to school, fare is a must every day. You have put that you must go for sex work to be given. And in sex work like now that they have closed school is difficult. Someone else husband will tell you “ me I have shopping, me I have I don’t know so and so…” many things. And you also expect him to do it for you. Now there is where they say women who do sex work are thieves.

D10: And now is the time to steal.

D6: (laughing) like yesterday I had nothing?

D10: Right now someone does not come to… right now someone does not come to the club with a lot of money. He can’t.

D4: He can’t

D1: But you kno …

D10:…Even alcohol he won’t buy for you more than five.

D1: You see like me number one, sometimes I can calculate and know that my neighbor her house is big. I have gotten a client, I tell her that money to go and book lodging, how much are you going to use? He will tell maybe for 1,000 shillings. You know the one thousand one has hot water for showering and it has its time. I will tell him, “if you don’t mind I will take care of all your needs. I will sell to you, and mine for sex you will give me. My hot water… you will just use but now for the room, we are going to my house.” Because I don’t have a husband, I don’t have a boyfriend who is just there that this is my boyfriend in the house, you see. He will give me the money for the room, he will give me my money the usual money, and you know there at my house I cannot play games with him. I cannot steal from him. Because if I steal from him at my place I will have brought problems, you see. So other times we usually defend ourselves. We think that “if I do this here, I will have spoiled for myself.” A place to put him until he is okay, take him to =Naselicca=. Give him what you give him. Put for him a drug in the alcohol and then take off with the wallet. You will have helped yourself there.

**I: Okay, thank you. Let us continue. For female sex workers who do not save, why do they not save? Or let us start with the ones who save. For female sex workers who save, why do they save?**

D4: Me as number 4, saving is good. There is an emergency, you have a child, the child becomes sick and you were not saving. You see there you will have a problem. That is why they said in every work that you do you have to open your mind because there are many things. You yourself can become sick and you had put that you don’t even save, these NHIF you don’t even bother. You are just there. You know… let me tell you something, there are people who go to work because they have seen others doing it. It is not that she is going because she has a problem. So someone like that, you know saving will be difficult because she sees so and so leaves with money, so I also have to do what, I have to go. So that I also get money the way she does. When she gets she celebrates money. She doesn’t know that the other one has good brains. When she gets money, she knows very well that today I have made 1,000, I have to save 200. Put it somewhere and use the 800. But now her when she gets, she wants… “number 4 wears a short”, that short is what she wants instead of using her mind. So I feel that we have to save. So that it helps us during emergencies.

**I: Okay. Number 10**

D10: I will contribute. You see saving is good. Sometimes you have been stranded you have nothing at all. That your saving does what… like now I was saving, during COVID we were getting money… the trailer people have money it’s no joke, even if you do it under the vehicle, you will get. It is just that now they are not there. They have become scarce. But I was saving and that saving is what is helping me now. Now there is no money. We have to do merry go round, we help each other, that is what will get us from this work. But if you say you cannot save, that you just go… today I have gotten this big one, tomorrow I will get a small one. Tomorrow I will get a circumcised one. Tomorrow I will get one that is uncircumcised. You will have blundered. Save a little.

**I: Thank you. Number 5**

D5: I will contribute we have to save because it helps us in emergencies.

**I: Thank you. What makes it easy to save? For those who are saving, what makes it easy to save? Number 7**

D7: Like... there is… let us say there is… to those who are doing… to other people who are doing sex work, there are agents who search for clients for us. So you know when you get a client you will get a lot of money. So after you get a lot of money you can save. But if you look for a client by yourself, you will get little money. So after you have gotten a lot of money is when you can save.

**I: Okay, alright. Number 10, do you want to say something?**

D4: Me as number 4, the thing that makes us save… you know in this our work, the earnings vary. Today is not like tomorrow. We had said clearly when we were renting the house. So if you don’t save, how will you pay rent? You can’t say that the way you get lucky, the way… I heard another client say today I might get 10,000 or I can steal and get 10,000 and go and pay school fees with it, and pay rent. You can’t put your mind like that. You work with what you get. So after you have saved, it is something that is just mentality, “every day I have to put my 100.” That is something that you decide. First before you start a business you have to say, “this business of has to pay.”

**I: Thank you. Number 8, what makes saving easy?**

D5: Okay. Me as number 5, if you want saving to be easy…

P: Number 8 was not understanding she thought you were saying number 9.

**I: Ooh, number 8. I say number 8. Let number 5 talk because she is ready and then number 8 to talk.**

D5: Okay me as number 5, if you want saving to be easy for you to do, you have to balance your things. You have to budget so that you find a way of saving. We must have save.

**I: Thank you. Number 8**

D8: Just continue I will just answer the next question. That one for saving I don’t have.

**I: Okay. Thank you. And what challenges do we go through during saving? What challenges?**

D4: Me as number 4, the challenge we go through… the way I had said, we have to put our mentality that we have to save. So you know maybe you have put yourself within a budget. That me my budget goes like this, that style is how you will get money. That now is difficult and let me tell you, I now can … what has passed today, getting it tomorrow is difficult. Because you have put yourself that you have to save 200 or 100. Today you have just gotten that 200 and you have children. They are going to school, and transport and they eat. They also have to bathe. You have got 200. So you know there, it will be a bit difficult. It will be difficult until that saving will pass. So if you are a person who uses her brains well, today it has passed. Tomorrow if you are lucky you double there.

D10: Eeh, that is why I said you double twice.

**I: Thank you.**

D4: Because whatever has its advantages has to have disadvantages.

**I: Who else wants to also talk about the challenges of saving? All of us go through them?**

Chorus response: Mmm

**I: We set targets. You save, this month I will save 5,000. Month end when you look at your savings, 1000 or less right?**

Chorus response: Mmm

**I: What challenge made it impossible to reach that 5000?**

D5: When you don’t get… oh me as number 5, if the work is little it is not easy to save.

**I: Number 7**

D7: If you don’t put money in a fixed account, let us say the child has been sent for school fees you will be forced to remove the money. And then you lack money for food, you will have to get money from there. So household expenses also.

**I: Expenses and school fees?**

D7: Mmm

**I: Mmh, number 6**

D6: Me as number 6, greed also.

**I: Explain greed.**

D4: That is what I wanted to say (laughing)

**I: Explain**

D6: So and so has bought, me I have not bought. It is a must. You take and go and buy. You have passed your budget.

**I: You have passed budget?**

D6: Mmm

**I: So what has so and so bought?**

D6: Maybe clothes, TV. Even jealousy, people have different types.

D1: Imagine even foundation.

D6: Even foundation.

D2: But me as number 2, I also like how she is saying greed. You know, someone you also have to see something good when you go to your friend, you will sit and see that this friend of mine has a certain thing. And also the other day, I want to look for money when I get I want to buy this thing. So I don’t see it as greed. I see it as if you are…

D10: …Progress

D2: Yes. It is like you are teaching yourself. When you see something good in someone’s place it will make you work hard to search for and then you also buy.

D4: Me as number 4, my colleague number 2 has spoken well. But where I want to correct her… or what I want to say personally based on what she has said, you know greed… I see so and so has bought a TV, I also want to buy TV. I don’t know how she has bought that TV. Maybe she had a jackpot and stole all of it. She just bought it once. So you know this greed will make me to go and remove my savings. Unfortunately I don’t know where she has bought it from. Greed is good but sometimes it can also be bad. She landed on a jackpot and got. Me I have not landed on a jackpot. It is just that my 200 that I was saving every day. That 200… you know I will have to remove it. Maybe it had already reached 5000. I will remove it and go and buy what I had seen in her house. And I was saving this money that at the end of the month I look at my account to see how much I have, I use it to buy my things. So you see I will have failed there. That is where you will get I walk counting my fingers. I walk talking to myself because I have done… because I don’t know how the other one gets.

**I: Okay, thank you. Now a challenge like this one of greed, what do we do with it?**

D6: Greed… me as number 6, there are two types of greed. There is greed to progress and there is greed like… I don’t know you are just born that way. You have a TV but you see another person has bought, you just have to go and buy. Just like that one.

Chorus response: That is jealousy.

D6: So there are two types of greed. One for progress and of jealousy.

**I: This one of jealousy what can we do?**

D1: Jealousy you know there is no time that that person will not be jealous. Even whatever she sees even if you buy that one of yours, she still wants it. So that one is jealousy. If you want someone like that you know you can just frustrate that person. If you see she wants chicken, you go and take chicken feathers and throw in the fire. It smells chicken. She will have to go and take the money because of greed and jealousy, she goes and buy chicken. At the end of the day she finds out that you were not even cooking that chicken.

D4: Or… me as number 4, jealousy… a person who is jealous… you know the one who is greedy for progress is good. She can come to me, “my client, tell me this thing, how have you gotten it?” you tell her and then she also starts planning. Not that she just goes immediately. But that one of jealousy, that she has seen number 4 bought for her child clothes she also wants to buy that clothe, if God can bless me with money I can do to her what she cannot. I can just buy something for 10,000. I know very well that when she gets 1000, she eats it right away. That jealousy will kill her.

**I: Another challenge number 7 said that makes us not to save, is household needs and school fees. What is the solution? How can we solve it so that we are able to at least save? Number 3**

D3: Me as number 3, if God can help me get a job that is not sex work that I depend on in my life, I get even a good business that gives me money, me I think those challenges can reduce.

**I: Is there anybody with another opinion? (short pause) number… 4**

D4: Me as number 4, you see those challenges that can make us not save, solving them is difficult. Because sometimes work is difficult like we had said and also by that time that work is difficult, is when diseases come in. problems now come in, money is also little. So me what I can say, it is just you personally to be organized. You just say… I had very well people saying that you tell your body sorry. Just tell your body sorry but if you just plan yourself properly, I want my household budget to be like this. Don’t put a budget that you cannot reach. That is… it is still going with the greed. You see others eating like this, every day, you also want that. Set it depending on your earnings.

**I: Thank you. For women who do sex work who do not save, why do you think they don’t save? Number 6…**

D10… Needs

D6: Me as number 6, I don’t save because I know tomorrow I will get. Why should I save and tomorrow I will get?

D7: Me as number 7, what makes me not save is because the cost of living is high and the income is low. So it forces me not to get that one to save.

**I: Anybody else who wants to contribute for those who do not save. What reasons make them not to save?**

D4: Me as number 4, what can cause you not to save are many. Problems can be many. Needs can be many. Like now the cost of living is very difficult. So you know you are already used to… earlier you were saving. Now you see that one thousand, is little. You cannot get… you cannot manage the way you were managing earlier…

D1: …Responsibilities, responsibilities

D4: Right now the responsibilities are many.

**I: Number 1, tell me a bit about responsibilities**

D1: That one because I am talking about it, me on my side as number 1, my responsibilities are many. You know once you are a single parent, if you get even 1000, it is not enough. You have three children, those children are supposed to go to school and they are supposed to go by motorbike, you give them lunch money, you also want to eat, you have oil that is supposed to come from there, I mean responsibilities are high. I cannot.

**I: What are the disadvantages of not saving? Number 6**

D1: Okay. Not saving also has its effects. You might be sleeping with your child and the child gets malaria at night, you don’t even have a shilling to buy Panadol. So there is where you will start saying if I knew I would have been- been saving. There already you are late. Because if you had been saving you would have saved this child of yours. Now tell me, at that time which neighbor will you be knocking on their door to help you even with Panadol, and you, you are doing your night work. They will ask you just the same way, “you mean you don’t have any savings?”

**I: That is number 1 who has spoken. Ehe.**

D6: Me as number 6, not saving is what will make you get into debt. You will start selling your property because you now have problems. What will you do? You have to borrow. If you don’t borrow you will sell things that you had bought expensively, you sell at a loss.

D8: Saving also depends with someone’s earnings. You see maybe every day you get 500. That 500 is what you have to eat, pay for children transport, you give them school lunch. There is no way you will now save. It depends also with the earnings.

**I: That is number 8 who has spoken. Number 8, thank you. Mmh, are there other disadvantages of not saving? (short pause) okay. Not saving… I see some of us have started moving in the seats. We will talk quickly now.**

D: Someone from me will be kicked out of work

**I: Okay. Is there a benefit of not saving? What are the advantages?**

D1: There is no benefit it is a loss.

**I: Okay. Where do female sex workers usually save? Where do they usually save?**

D6: Me as number 6, mostly we save money in the chamaa. That is where we put money then go and remove. During your turn you get and do with it your business.

D10: Me as number 10, there are those who save in the phone. You know when you save something, don’t put your mind there where you have saved that when I get something… you will not save because you will feel that when you get something small you want to do what- to remove it. Don’t put your mind on that saving. Just save but don’t put your mind there. When you put it there is when you will not have it at all. You will go and remove them.

**I: There is in MPesa or which one?**

D10: You can put it in MPesa, Mshwari. So long as you know that you have saved.

D2: Me as number 2, there is a game… there is a chamaa then there is a game called table banking. So that one is the one that saves me. When I go, I borrow and come and use it. I will look for it and find then pay back. After paying back then you borrow again.

I: Number 5 what were you saying?

D5: As number 5, I also save in the bank.

**I: In the bank?**

D5: Yeah.

**I: Why do you prefer saving in the areas that you save? Like number 2, why do you prefer table banking?**

D2: Because that is money that I know if I go when I have a problem I cannot miss I will get. They call that *“mchezo ya meza”*. It is just money on the table, you cannot miss.

**I: Number 5, why do you save where you save?**

D5: In the bank it is also just the same. Anytime I want it I can withdraw.

**I: Number 10?**

D10: Even in the phone it is just the same. When you want it you can get it, yes.

**I: Number 6, why do you save where you save?**

D6: Where I save… because when it reaches my turn I will get.

**I: When it is your turn you get it?**

D6: Yes.

**I: Mmh?**

D4: I am adding. You see where we save, why we like them, if it is for example in the phone, I can put it in Mshwari. It can reach a time that I borrow money and there now it is easy work because when you borrow money, you can pay school fees or pay rent then you are left with paying it bit by bit. Same to bank. You can also borrow from the bank. Table banking also you have to… if you are in a chamaa that has table banking it means there is a loan they give there in that chamaa.

**I: Okay, alright. Do female sex workers live beyond their means? Do they live beyond their means? Do they live beyond their means? Is that one understandable?**

D: Yes, it is understandable. (long pause)

**I: Are you leading life that is not yours…**

D9: … you cannot live more than your means because you have no way of helping yourself behind here...

D: …You don’t have an income.

D9: You don’t have enough income. So you cannot live a life beyond your means.

**I: What number are you?**

D9: Nine

**I: Number 9. Ehe, who has a different opinion?**

D4: Me as number 4, you cannot live beyond your life. It depends with your earnings. What we were just saying. You know there are some questions that keep repeating themselves. You find that this answer is the same with the one that you had said. Right now I can’t decide to live in a house worth 5,000 and my earnings can’t reach 5000 every month. It depends with my income.

**I: Now that is your personal opinion?**

D4: Me personally.

**I: And the others that are around you?**

D: Just like that

D: It’s the same.

**I: Okay. You believe that all of you and the other women out there, you live within your means?**

Chorus response: Yes

**I: Okay.**

D3: The ones that are better than us have men. I am number 3. And they have where they will be buried.

**I: No, I mean… not women who don’t do, I am saying all female sex workers, do they live within their means?**

Chorus response: Yes

D4: That is true.

**I: Okay thank you. Now… women who do sex work, how can they fill the gap between what they earn and what they need? And maybe they are struggling to get? If they are using more money?**

D1: Repeat the question

**I: Female sex workers… all of us have needs right? Maybe there is something you need here and it becomes a bit difficult to get it. So you have to do some things so that you can get, right? So I would like to know, you when you are here, what do you think they can do to fill this gap between need and earning? You see. The earnings are here but the needs are where?**

D1: They are high

**I: They are high. What can they do?**

D5: As number 5, that’s why we said we have side hustles. Yes, to fill that gap.

**I: Ooh. Is there anybody else?**

D: Just that one

**I: Only that?**

P: Mmm

**I: I know now we have started giving one answers because…**

D: We are tired.

D4: Not even tired, time. Time. Even its like my child has been rained on.

**I: Mmh, do women who do sex work borrow money or get into debt?**

: DYes

**I: We talked about debt?**

Chorus response: [inaudible segment]

D4: We can get into debt.

**I: And… I remember we had talked about these debts. Just a little maybe we touch on it, what reasons do people get into debt?**

D9: Because of lacking. You may want something or you get an emergency and you don’t have money. You have gone somewhere, you don’t get clients, so it forces you to just go and borrow.

**I: Where do you usually borrow from? Or where do they borrow from?**

D9: Someone like me, I can get any of my people, if he doesn’t give me, I go to the neighbor because it is not everybody who has.

P5: Me as number 5, I borrow from the shop.

**I: You borrow from the shop?**

D5: Yes, when I will get I will pay.

D4: Me as number 4, they say if you have a child you will have to steal. So that you don’t steal it will make you to go and borrow because the child will ask, “mom, what are we eating today in the house?” and you don’t even have a shilling, it will force you to borrow, right?

**I: And usually what do female sex workers do to increase their income? There are side hustles that you have told me. Other than the side hustles?**

D: Stealing

D: That one is stealing.

D: Just stealing.

D: [inaudible clause]

D: I put for him ten pills in the alcohol, in the chang’aa and give him to drink.

D: One by one

**I: I know we want us to answer quickly and finish, but let us give each other a chance for each one of us to answer. Then if you are talking say your number at least for the person taking notes to know. Okay?**

Chorus response: mmm

**I: So, sometimes maybe yesterday you had gone to look for clients and you have not gotten a client, what do you usually do?**

D8: Like me I wash for people.

**I: Number 8**

D8: Number 8, yes.

D1: Me as number 1, I can go even to cook. For example, the client is not there and it is on a Friday, if they want catering people, I am one of them.

**I: Another person?**

P3: Me as umber 3, if it becomes difficult, I am making my chapatis.

**I: DYou cook chapati?**

3: Yes.

**I: Another one? (Long pause) That is all?**

Chorus response: Yes

**I: Now, someone has said she cooks chapatis, another has said she washes, another has said catering. Is there any other thing… there was a time someone said others do anal sex, right? Maybe you have gone and the client insists that you have to do anal sex. Do you know of that type? Maybe we speak about it a bit more? There was one who said anal sex, right? So maybe let us speak about it, for those… we are not saying you or me, remember we are sharing thoughts or others’ experience out there. Those who do anal sex, or different types of sex, are they there? And what makes someone to get to that point of doing that?**

D4: Me as number 4, I cannot say that it is lack of money is what makes someone to do something like that. That thing someone does… you see we start slowly by slowly to do this sex work. It will reach a point that… they usually say peer group. I meet with another group and they say, “number 7, you know you are putting yourself here and you don’t know money is a lot in front. You go and try anal.” And there is where I will leave as number 4 and go and try. Once I have tried, I will find that money is a lot. So, I will see it was like I was wasting myself back there. So, I will decide to go on with that one of anal.

**I: So, do you think it puts them at which level of risk?**

D4: It is risky. It is risky because you heard them say clearly, “do that work and be ready to treat yourself.” So, it is risky.

**I: Is it risk of treatment or is there another risk?**

D4: Risk comes because once you have put yourself like that… you know if you are… now you are doing lesbianism work, there are challenges that you have to face. You will not be the way I am like this. You know when I do this sex work… the normal sex work that I do, you dress normally. But now that one… lesbianism let us say even if it is a man and a man, you have to dress now as a woman. You are supposed to force the hormones that you don’t normally have, to now be there. So that at least when you are caressed with a fellow woman, you have some feelings.

**I: Number 1, you had said that if you don’t have, you borrow right?**

D1: Mmm

**I: You can borrow from the neighbor or someone else? I would like to know; usually how do you pay this debt? How do you get to pay back?**

D1: Okay, like sex… I have told you even sex work is bad, I can go catering, you see. There in catering when I get the money, I will come and pay you your money and what is left I will use.

**I: Is there anybody who can share with us. After borrowing which methods do you use to pay back?**

D4: Borrowing… me as number 4, there are a lot of ways to pay back. You know debt is debt. Once you borrow you will have to pay back and you cannot pay the whole of it, maybe it was a lot. You can just talk with that person and tell them that you will be paying little by little. If work is bad today and even tomorrow, tomorrow can’t be bad again. So, if you had already borrowed and you get successful and get a little more, return… just go and reduce that debt because tomorrow you will also need help.

**I: There is a time you have said that when you go you don’t geta client, right?**

Chorus response: Mmm

**I: There was a time you talked about that. So, how do you know you will not get? Getting to this point I will not get?**

D1: Like me number 1, I can go somewhere… I can go into a pub, or a bar, I will ask for the first bottle and drink and you know the time is going? I will ask for the second one and drink and finish. Time is moving. When it gets to 2:00 or 3:00 and I see that there is nobody next to me, I just know that today is not a success.

**I: That is 2:00 or 3:00 of what time?**

D1: At night.

**I: At night. Ehe, number 5?**

D5: Yes, that is what I wanted to say. When the time is up, you just know that today is nt a success.

D10: Also, when you are a prostitute, let us just say that name. if somewhere is dry when you enter you just know that this place…

D1: … You feel

D10: Here there is nothing. You look and just know here there is nothing. You look for somewhere else because if you are a person who does that work, you sit somewhere and just know that here there is no work.

**I: Number 2**

D2: I just wanted to talk about that. Me as number 2. That one that she has said.

**I: Ooh, that one that number 10 has said?**

D2: Mmm

**I: Okay. Now, how much do Female sex workers usually have in debt? What is the amount of money usually? Number 10.**

D10: You can have a lot of debt. Sometimes it is bad. You can borrow… even in a day you can borrow from your friend you borrow 1000 and tell her you will repay even tomorrow. You know if you have your friend that you work with, she will understand you. When you get a client, she will know that this one has got a client, tomorrow she will get the money.

**I: What does roadie mean?**

D: Sex work

**I: Ooh, it has many names?**

Chorus response: Yes

**I: Dira, roadie, parking. (chuckles) okay thank you.**

D10: Yes, she gives you knowing that tomorrow she will see that you have got a client she knows you will pay. It is also good to borrow from someone you are with…

**I: Work with**

D10: Yes

**I: Aha, thank you. number 7, approximately how much debt?**

D7: Me… in a day?

**I: Even in a month,**

D7: 5000

**I: 5000. Number 6**

D6: Me as number 6, debt depends with the problem you have. Debt can even come to 50,000. Your father has taken you to the hospital and you are required with money. There will be debt.

**I: Number 8…**

D4: … me as number 4…

D8: Me I am afraid of debt.

**I: You do not want debt…**

D3: … Me as number 3, debt is debt. You…

D8: … I’d rather other people borrow from me. But me no.

D4: Me as number 4, we cannot say debt…

**I: … Number 3 has not finished.**

D3: Me as number 3, debt is debt. Because you will find I have debt starting from the club until the place I am living. Yeah.

**I: And in a month how much can it be?**

D3: Even 20,000.

**I: Thank you. Number 4**

D4: Debt is debt the way she has said. You can have debt even of 50,000. Because sometimes I have a problem today, I borrow 10,000. Then again, I have a problem. You know I will be afraid to borrow 10,000 from this person. Then I will go to another person. You can have debt even of ten people and it is just money. So, debt depends on your problem.

**I: Do female sex workers… we are** **close to finishing think about a time they can leave this work?**

D1: So, me as number 1…

**I: … do they think about the time when you will leave?**

D1: Me as number 1, there is a time you can go and find someone has carried *muchulbeng’* that you just say “oh God, spare me from this matter, it is not my wish me to do something like this” you see.

**Note taker: What is that?**

D: A big rod

D1: It reaches a time that you feel… you just pray to your God to spare you, you see. This is usually not to someone’s wish. It is just that poverty is what makes someone to get into some things. I am not lying to you, you can go with someone… you hear people say in the European countries that you agree with someone well, the money you negotiate is good and when you go, they give you a dog. The dog wants to have sex with you it wants to *kukukwangura*, you just come back and say “oh god, there is a day that I will wake up and find that you have removed such problems from me.”

**I: Another person? Number 3**

D3: Me also as number 3, that thing is a challenge. Because these things are not the same. There is a small one, there is a fat one, there is a short one but it is carrying weight. So, you will have to persevere in that weight. Maybe you have persevered… you know sometimes we pretend we are clever. Even us we get problems in the lodgings. Because you have met a man who also you know that drug, he had already put it in your glass first. He will milk you in debt. When you come to, even your clothes are missing. You come back you have no shilling but your body eh!

**I: So, in short you want to say, there are times that you think about leaving?**

D3: Yes

D4: Me as number 4, like I had said earlier, this work we are not ding it because we are happy, we are doing it so that we get… we see this is the way of getting our money.

D10: Quick money

D4: Yes, so that we get money. That is the way we see is easy to make money. But it is difficult. There are challenges. You get a drunkard, any style and he said 500. When it is time to pay, he tells you 200. But he had already done to you what he wanted. There is nothing you can do. Later when you sit down and think eh!

**I: Do you have this discussion among your peers? Number 3**

D3: In the field, we usually have something called meeting. On our own. If you have met in the evening, you have to sit down. You can’t come in that this one number 5 has come in number 3 has come in, no. we have a leader. So, this leader when it gets to the evening, it is like you have to sign because when you come to =Kondele= it has its name. there is another section also with its name. so, there is a prostitute who is known that this comes from this block, this one is from this block. In case even if something happens to our colleague, you know that this is from a certain block. So that is why we have to be with our leader. So that when we get to work, you start work knowing that so and so has come to work. That is why we like mostly when you get a client you be in that one building, if it is that club, it should be in one building, than getting a client who takes you away from there, and takes you to another building, or town or anywhere. That is usually a loss.

**I: Thank you. I would like to understand if among you… like you have said within that zone in the hotspot, when you talk as people in that hotspot, in those discussions are there times you talk “I want to leave or I want to leave,” do they come up?**

D: They come up

D: Yes

**I: And what specifically causes these discussions?**

D6: Me as number 6, mostly the things you go through, someone has not paid you, someone has used you badly. So, things like those are what makes you say I’d rather leave, one day I will leave, you see.

**I: Is there anybody else who wants to talk? (Short pause) okay. Normally, why do female sex workers leave or want to leave sex work?**

D10: As number 10, like she has told you, it is something that someone has gone through. So, she feels that it is better if she gets something else to do rather than that one. Because if you see anybody who wants to leave this work, there is an issue she has gone through.

D: There are difficulties.

**I: Is there anybody who wants to add?**

*Silence*

**I: Okay. Thank you. now… women who do sex work, usually leave this work at what age?**

Chorus response: That one has no age. It has no age. It has no age.

D1: Me as number 1, that one has no age. It has no age. Because even us where we are, the ones we found are still there and they are big, and they are old they are grandmothers. But they are still pushing their work.

D8: Some have even become rich from this work and until now they are going on.

**I: That is number 8 who has contributed. Mmh? Now we have talked about the struggles we go through, that is a personal reason why you would want to leave sex work. Is there another reason? Even if it is not personal**

D4: Me as number 4, you know now when you put yourself that you your work is sex work, you have no name. you have no respect. Even where you live. Because if they know that your work is leaving at night and going there, there is no respect and you can’t even sit where other women who have their homes sit and talk. If you start talking, they will start saying “what do you know? You only know sex work.” So, it is lack of respect.

**I: And do female sex workers leave sex work when they want? Or at the time they have planned?**

D10: Yes, there are people… me as number 10 I am talking… there is one who has left this work and she stays in her home. She is looking after the children. She has left this work.

D1: Okay. Me as number 1, you know you can find a client who is serious with you and tell you “Me I want to take you to be my wife and leave this work.” There is one I have seen. She has left this work and she is married with her client and she is married and she is staying and has given birth. It also means that it is the heart. You cannot be forced to leave and you cannot also just decide that today I am leaving and still you don’t have the will to leave.

**I: What do… after sex work, those who leave, what do they do?**

D: There are those who are married. There are those who are married in their homes they have stayed. And there are those who are doing, let us say business. Someone just decides to do her own business. She does not want to remember where she has come from. She has decided that cutting this Sukuma, it is me and Sukuma and the knife until the end.

D4: In fact, someone like that can do a meaningful business.

D10: When she remembers the life she has come from.

D9: Me as number 9, the sex worker I have seen who has left sex work of going to town parking, has come back to do sex work in the neighborhood.

D4: Now she is bothering wives…

D9: … A neighborhood sex worker. Now you don’t want her to see your husband. When your husband passes, I am with him. I am the one who seduces him by force. He goes and finishes for me my need. So long as he gives me money. Those are the neighborhood sex workers. She doesn’t want to see a man pass. That one of town was difficult for her. So, she is now in the neighborhood.

**I: Are there ways for those who leave sex work in which their life becomes different?**

Chorus response: Mmm

**I: Please let us answer one by one.**

D9: It is just like we have told you. me as number 9, it is like we have told you, when someone leaves there, one has gotten a man whose heart has decided to leave that work. Just like we have told you.

**I: How is her life different?**

D9: It is very different because she is now going to her home now. She is living with her one husband, she gives birth. The man helps her with everything that she wanted, she cannot go back to that work.

D1: And you see, me as number 1, you will find the one who has left that work, when she sees you, she feels pity for you until she comes and tells you “I am not forcing you to leave but I pray for you, there is a day you will also leave here.”

**I: Are there bad things that happen to those…**

D3: … Me as number 3, there are those who leave and even get into salvation. They are the ones who come back and preach “please, leave this work.”

**I: Are there bad things that happen… there are those who have left and bad things happened to them?**

D4: That one is there. Me as number 4, sometimes you have left and you get disease.

**I: Which disease?**

D4: Just this disease of HIV, because you can’t say malaria is a disease. Malaria you will be given Coartem and you get well. But that one of HIV maybe you have got it… and let me just tell you the truth there is no need to lie…

D: And STD

D4: These STDs, you itch that if you are in the midst of others you can’t sit. You can’t. even here smells. So, you know you are embarrassed you can’t go to your friend.

**I: Is there any other bad thing that has happened to them?**

D3: There are many. Like me number 3, there is a problem you might have when you leave that work, even when you sit, you feel like you cannot sit properly. It is like there is air coming out. You keep on polluting where you are seated.

D5: Number 5, problems are many. There are even those who come and take us you just hear they have been killed somewhere. They even get killed.

D1: Me number 1, here at =Kondele= there is a girl who was killed.

D5: They kill us.

**I: For those who have left, you leave then someone comes and kills you?**

D: No

**I: I am asking for those who have left**

D1: Yes, there is one you had done bad to them and you have ignored them for many years and has come and found you, eeh…

D: That one kills you

D4: And also, it does not mean that someone…

D: … For example…

D4: … takes a knife and stabs you, that he kills you. he infected you.

**I: That is number 4 who has spoken.**

D: For example, like me number 3, I had stolen from someone some money, the time I had stolen knowing that it has helped me. So, I have left and we have met even in a certain town. This person will finish me. He will finish me.

**I: Okay. Are there things they would like to accomplish before they leave sex work? for those who want to leave.**

D: Sorry?

**I: I am asking if there are things that female sex workers would like to accomplish before they leave? Mmh, number 1**

D1: Okay, me as number 1, maybe she was looking for money for a certain business, and the money she wanted has reached the target, she decides to leave sex work.

P3: Me as number 3, you may have done that work for a long time, you have even got a plot somewhere, then you will say now I can go and rest.

**I: So, for those who have plans like that of buying a plot, is it something that you have started working towards or you are just saying?**

D3: It is something that I have started working towards.

**I: So, when you look at it how many years do you see ahead of you?**

D3: Even three years before me.

**I: Okay. For the business, the one who wants to start her business?**

D1: It depends with the target of money that you wanted…

D4: …Me as number 4, if you want to do business you have to have a target. Because if it is for selling vegetables it is one thousand. One day and you have stopped it. So, it depends with the business.

**I: So, for business it depends with the business. Let us say it is business… like number 1, you had said someone may want to start her business that will make her… when she starts the business, she does not go back to sex work. Like that one, how long would you give yourself?**

D1: I can give myself two years r three years. I know in those 2 years I will have gotten 100,000 or 150,000, I will run to =Busia= and bring handbags and there is where I will start the capital.

**I: I would also like to understand those who have left sex work and come back. Are there?**

D3: Me as number 3, you can leave from this our work, you go and open even a stall. There is rain, Sukuma is for 100, you have not sold, tomatoes are rotting, you are making a loss. When you sit down you think “eh, better that business of mine, I go there and for two rounds, I have my 1000. I go back to the house.” They are many.

**I: Number 5. Are there those who have left and come back? (Short pause) you don’t know?**

D5: Yes

**I: Is there anybody else who wants to contribute there? I know we know people who have left and come back. Somebody else to talk. To give us another example of what happened so that we move quickly. (Short pause) Okay, thank you. now, do you know anybody who has left sex work between 5-10 years. Who has left between the last 5 to 10 years? Do you know anybody who has left from this work?**

D9: Me as number 9, let me say I have never seen anyone who has left and then came back. I have seen those who have left and they have left.

D7: Me as number 7, I have seen a woman who has left this work. Her children grew up and got their jobs, so they built her a house and she decided to leave. So, she is being supported by the children.

**I: Is there anybody else? (Short pause) we only have the one example?**

D3: Me as number 3, there is one who left. She found a man. He decided to go and build her a home. He gave her a good life. So, she is living and has forgotten this work.

**I: Are there who have left and experienced problems?**

D4: We have answered those things or which question is that?

**I: We just want an example. You see the way she has told us. There is one who has left and has been built a house. Yes, something like that.**

D4: Me as number 4, the reason I am saying we are repeating ourselves. We had a question like that and we said it well, someone can leave this work because she has gotten a husband. Someone can leave this work because her children have grown up, they have work they can decide to remove her.

**I: Okay. I want us o answer a few questions about this Jitegemee that has brought us here then we can leave. Earlier I had spoken about Jitegemee and I mentioned that it is a way that female sex workers can get out of sex work by saving some of their money that they make themselves over a period of time. Then if you decide you want to leave, you leave while having some savings. Or when you don’t want to do sex work, you don’t do because you have some money somewhere you can go and use and you eat, your children don’t go hungry and school fees is not a problem, right?**

D: Mmm

**I: This Jitegemee works in this way, you save when you can. Nobody follows you like the chamaa. You save when you can. When there is a lot of money, you save. When there is no money, it is not a must to save. Then it has no interest, you see. That when you go to withdraw your money, you are told that you will pay back with interest. You can remove all your money or half of your money or the little you need. When you go to work or the way people say roadie, and it does not go well for you, you cannot do the things a client can force you to do because you don’t have, right. But you come back home, you ask for your money maybe over the phone and you are sent and that day goes on. Maybe you are sick and you can’t go to work, you have your saving that you can do what, you can go for. So, this Jitegemee, do you think it is something that female sex workers would like?**

D10: Me as number 10, I feel they will like it very much. They can like it.

D3: Me as number 3, I feel you help us even very quickly. We are tired.

**I: Why do you think they will like it?**

D10: Like me number 10, because you know there, there are a lot of women who don’t know about saving. When they hear that there is something that they can save that will give them something, they will be happy. I am sure they will be happy.

**I: Is there something you think they will not like about this Jitegemee? Just the way I have explained to you. (Short pause) Anything?**

D: Nothing

**I: Nothing?**

D: Mmm

**I: And the female sex workers you know, what part… out of the ten that you know, how many can agree to join? Number 1?**

D1: The way we are seated here, me I see that the ten of us already we have entered in that box.

**I: And the ten that you know out there?**

Chorus response: They are there.

D4: If we tell them they will join.

D10: Me as number 10, you know the more someone sees that you are progressing with something is the more she wants and she will say “ah ah, I also have to do what, to join.” Something is to start. You see once you start something those who want are many they will want to join.

**I: Are there ones who will not join?**

D4: There is wanting. [?there is greed. One can refuse?]

**I: Are there ones you know that will not want to join?**

D4: Yes, I have said… me as number 4, you have heard me say *taama*, not greed, *taama*. When you cook dengu there are those that don’t cook. So, here also we will have to find those who will not agree.

D10: One who will not agree to join.

**I: Ooh. Do they have reasons?**

D1: Yes, they have their reasons...

D4: …Yes, they have their reasons...

D1: … They know themselves.

D4: They know themselves.

D3: Even they can say, she can save that money and not get it. That is also a challenge. I can save at the end of the day my money will get lost…

D4: …And then me as number 4, there are many things in the world. They will say maybe you are the people of illuminati that want to steal money from us.

**I: And what can we do so that most female sex workers will be willing to join Jitegemee?**

D1: You call them… me number 4, you call them, you have a meeting… ah I am number 1, we can call them the way you have called us like this, we have sat down and talked with us. You know someone else could have told us out there we would not have trusted them. We would have said that if they wanted us, they would have called us. But now like you have called us, you have explained to us gently, we have understood you. you know even us if we take this information back to the community, they will tell us no. we also want those big people who called you to also tell us clearly.

D10: And me as number 10, I can contribute by saying, even us who are going back to the ground, at least we take information and tell them this and this. You know even us when we sit with them even if they are not called here, they will have that information.

D3: Me as number 3, this agenda that you have started… us who have been reached, you would have started with us. When we see that the fruit is good, it is easy to call others.

**I: That is one way of doing it, right?**

D3: Yes.

**I: By starting with you. is there another way we can implement it?**

D4: It is just starting with us and we see the fruits so that when you tell somebody you say, “you see me I was doing this but now I have stopped, you see I am now like this. You also come.” I am telling you what has happened to me not tell you something that I believe.

**I: Are there things that Jitegemee should have so that you can accept it more? Or female sex workers to accept it more?**

D4: Yes, there are things…

D: …Number 3…

**I: … Yes, things like what number 3?**

D3: I have heard you say there clearly, you are supposed to do savings. Maybe I don’t have that savings. What help can you give me in the first place? I don’t have that savings and I wish to leave this work. What can you help me with as your client?

**I: So that you join Jitegemee?**

D3: Yes. Because you can use me, and when I leave here, I don’t see any help. I will say even where I am from, I have not received help…

D1: … let me continue with my work.

D3: Let me continue with my work. So, I feel like me number 3, if I get help, at least I would come out with something to announce out there.

**I: And this… female sex workers can like… um, sorry I think I have said that one. To female sex workers… you have said apart from that one… you leave here with help, you have been given something. Is there something in particular that you feel ai, this Jitegemee the way you have packaged it like this, this is what we will like the most. Particularly, what would they like the most?**

D4: Money.

**I: I have not said we will give money.**

D4: Money or work because we have gone there to look for money.

**I: This Jitegemee, I have not said we are giving work and I have not said we are giving money; it is true right? We have said it is saving…**

D3: … Me as number 3, advice is also something important. Your idea can help me somewhere. So, I can come and you give me that advice and when I go and sit down and think properly, there is a step I can make with it.

**I: Which idea have you especially got from Jitegemee?**

D3: It is that of saving, how to live, things like those.

**I: Number 7?**

D7: It is just assuring us that the money we will save we are sure will have security. Then also if you show us some ways that we can do business so that… that can bring money quickly so that we can save quickly.

**I: Thank you so much. Are there ethical concerns? Human rights issues, that you feel that maybe this Jitegemee comes with, that female sex workers will not like or maybe they will feel is infringing in their personal lives? Ethical concerns? Human rights.**

D10: Me as number 10, I would like that even if it is like that it has human rights. You know your rights personally.

**I: But the way I have explained, do you think it is infringing on your rights?**

D10: Me I feel it is okay.

D7: It is okay. It is not infringing.

**I: It is not infringing?**

D7: Mmm

**I: Number 7 you have said it does not infringe.**

D3: Me as number 3, I feel that it will help. Because there are challenges, we go through. And these challenges, there is no one you can run to. Let us say for example, someone has borrowed from me. If I harass her that I want my money, you know even if the police arrive and hears, “you what is it, what is it?” I will tell him “*Afande* [term used for police], this person I have given him a certain round, he has not given me my money.” He will say, “you fool, go and sleep.” You see. So, us we have been taken as the work we are doing is not work. But when we join this organization, it can help us.

**I: Thank you. Do you see if there are challenges people of Jitegemee will face when they come to the field to look for sex workers to join Jitegemee? What challenges can we face?**

D4: Challenges… me as number 4, you know what has an advantage has to have a disadvantage. You will come to convince me, I did not come to the meeting, I did not hear everything. So, it is my first time you want to talk with me. I will answer you rudely. “you woman what do you want? Or do you also want to do this work?” I will throw words at you. so, you know if you have a soft heart, you will feel that there is impossible.

**I: Is there another challenge apart from that one? Number two? What challenge do you think we might face?**

D4: It is just that one. You will be talked to badly like that.

**I: Number 4, you are not number 2.**

D4: Honestly madam, just saying the truth, we have spoiled things today.

**I: So, how can we solve this challenge. This one of being talked to badly?**

D4: You just be someone with a deep heart.

**I: We have a deep heart?**

D4: Mmm

**I: How much money can…**

D3: … I feel that thing you are [inaudible clause] ended in the industry. Now it is your time so that you find the problems people are facing out here. So, during your time, go outside, announce this thing for people to hear. Whoever wants to get help, will be helped, and the person who will feel ah, this is something that will not help me. Let her also not blame her colleague who has succeeded.

**I: Okay. As we finish, I would like to know, how much you as number 10, can you save per week with Jitegemee?**

D10: Per week if the job is good I can save 1500

**I: Without interfering with your needs?...**

D10: … Yes, if work is good. Because needs are many. I cannot say I will save a lot in a week. I can save 1,500 per week.

D3: Me as number 3, per week I can save even 300.

**I: Number 7?**

D7: I can save like 700, 100 daily.

**I: Number 6?**

D6: 500

**I: Number 5?**

D5: 500

**I: 500. Number 2, how much can you save?**

D1: Number 1, 500.

**I: Number 8?**

D8: Me it depends with the month. About 2 months… even now I still have school fees I am supposed to pay. Maybe even up to July is when I can start saving.

**I: So, like that July if you join Jitegemee…**

D8: … Maybe per day, 200

**I: Thank you. number 4?**

D4: It depends with how I have earned. Because today I can go and tomorrow, I don’t go. So, it can be 500.

**I: And that is the good thing about Jitegemee. You don’t have to save every day. I told you.**

Chorus response: Mmm

**I: Now, if this target that you want to reach, 500,500. What can you do to reach it per week? Is there something you can go over and beyond to reach your target per week? What is it?**

D3: You will have to over work. You will work during the day and work in the evening. That’s overworking.

D4: Me as number 4, there I can’t say overworking. It depends with how you will get work.

**I: Is there anybody else who wants to say… number 3 said overworking. Working day and night.**

D3: Overworking… I cook my chapatis in the morning, at night I am doing sex work.

D: Where is the toilet?

D: You should wait we finish then we go

**I: We are finishing. We are just left with one question. When we contribute on that just one more then we finish. Another person?**

D8: Washing. I will just wash. Just work hard. Because even washing gives me some income… eeh

**I: Number 2?**

D2: I also do domestic work. So, it helps me to save.

**I: Thank you. All of us have said?**

Chorus response: Mmm

**I: Okay. Where do you trust to save your money? Everybody to tell me. Me as this number I trust here, just like that.**

D5: Me as number 5, I trust the bank.

D2: Me as number 2, chamaa. I save in the chamaa.

D10: Me as number 10, my phone. I save in MPesa.

D7: Me as number 7, phone and bank.

**I: Now number… who has not said?**

D6: Number 6, bank.

D8: Me I save for myself in the house in my bag there is somewhere I have hidden it.

**I: That is number 8.**

D8: Even in the pant, in the pocket I hide it somewhere. I can’t lie in the phone, because in the phone I will use, I can’t lie. In the bank also ATM, I will use. Better that place… it is not easy to remove there. But in the phone for me it is a lie.

**I: Is there anybody else with a different …**

D3: … me I will save but if that Jijegemee comes, maybe I can save better.

**I: You can save better?**

D3: Mmm

**I: Okay. Alright.**

D3: Because it is not for every day…

D3: … me I can save in the morning and I remove it by evening.

**I: Number3, you had mentioned that you will do more work, you will cook more chapatis and you will work at night. When you say that at night you will go to work, what do you mean by more?**

D3: More if I go to work at night, I will not get one customer. Maybe the work I was going to get even 3, yeah. That is overworking.

**I: Okay. Is there a way… is there something you can do instead of going to overwork at night, you do something else?**

D3: Yeah. Me as number 3, if I save and I save well, and then get that saving when I am okay, I will stay in that my chapati now, while going on saving so that, that chapati also bears me another fruit.

**I: Thank you so much. Thank you all so much we are grateful. Thank you so much for being patient. I know that we have taken a lot of time more than we had said. But I thank so much for all your contribution and we have finished the discussion at 18:09pm.**

**END**
