# Supplementary material for: Eat a little and save a little: A qualitative exploration of acceptability of a potential savings intervention to reduce HIV risk among female sex workers in Western Kenya
Source: PLoS One. 2024 Dec 19;19(12):e0310540. doi: 10.1371/journal.pone.0310540 (PMC11658496; doi:10.1371/journal.pone.0310540)
Supplement: S1 File — (ZIP) [file pone.0310540.s001.zip › Jitegemee Transcripts and Dissemination Notes for Journal/FGD F.docx]

**FGD ID: FGD F**

**DATE OF FGD: 26/APR/2022**

**INTERVIEWER CODE: KSM 06**

**NOTE TAKER CODE: KSM04**

**CATEGORY: ABOVE 30 YEARS, PERI-URBAN**

**Transcript**

**I: Welcome to the interview and this is FGD F which is taking place at =Kibos =and the time is 11:54 am. This FGD the interviewer is (interviewer name) and note taker is (mentions name) and today date is 26th April 2022. The participants in this interview are 10 thank you and welcome. From my brief explanation about Jitegemee what are your thoughts? What comes first in your mind concerning Jitegemee? You say your number and then talk when you are ready.**

PF: Can you repeat the question.

**I: From the way I have explained about Jitegemee, what comes first in your mind, what do you think about it?**

PF08: As number 8 you have said jitegemee is about saving, if there is nothing you have saved can they give you something like a loan?

**I: That is a question that you have?**

PF08: Yes

**I: We will talk about it after the interview, it is good you have grasp something in Jitegemee it is saving on your own.**

PF07: As number 7 when I talk I have heard very well about jitegemee, you have said that jitegemee am supposed to save money meaning if I cease to work, I will get money even if I have an emergency like when am sick I can use this money.it is also good to save money so that when I have an emergency, I won’t have to suffer. It has taught me if I was not saving money then I am supposed to start saving.

**I: Thank you**

PF03: As number 3 I have a question, you have said that Jitegemee intervention am supposed to rely on my own and it has started isn’t it? Am asking you as workers of Jitegemee as a client, a participant, I have a problem like an emergency before I start saving or before it goes for a long period, is there a way you people can support me or that’s my question?

I: I have heard your question I first want people to say what they think all of them then we will talk about it.

PF03: Okay.

PF01: As number 1 I will talk about Jitegemee, I have heard about your teachings and it is good but to me I feel about savings we usually saving, I can have an emergency and from that savings you have you are going to use the all amount. And I was feeling that you know us as sex workers at least you put something that you can support the sex workers with. You will be of great help to us because we will be saving may not be enough and that is why I will still have to go back there [sex work] and am supposed not to go back there [sex work] at least you be of help to me.

**I: When you talk about going back there where do you mean?**

PF03: Going back to engage in sex work with men.

**I: Thank you.**

PF04: I have heard issues about saving, saving is not bad but am asking the issue about saving, as you people you can rut something like a group, for the sex workers can we put something small and you save for us or you want us to save in the house. It is something like an account where you can rut something small and use a little, to say the truth in the house if any emergency comes we will use it and you know savings should be some good amount that you focus the future with, it is not for sickness or death or I don’t know what. When we save as sex workers it should be something that will help you in the future even if you quit work you are focused because you can’t work and there is nothing you are saving even if you quit sex work there is no way you will be able to take care of yourself if you leave sex work, now I am asking if you can assist people to open another account so that people can put some little amount of money if even you are saving something small but still have something to use in the house for food, sickness and even death and in the future if you leave that work you can make another step that is the question.

**I: Thank you. Is there anyone else?**

PF09: Am number 9 the question I have as for me, I don’t know how to save, you know there are those who do not know how to save and those who know how to save, now what can I do so as to save?

**I: Okay**

PF10: You have talked very well and you have taught us very well, I am used to this my work and it is something that makes me put food on the table and if we are not there you will not earn and I took it positively and that is why I said I must come there because that is where we eat and you also eat from it and I agree fully. That is the work I do.

**I: You want to say something again?**

PF07: What I wanted to ask like me am experienced sex worker I don’t know whether you are getting me, now let us say that I have a partner [a friend] whom I want to introduce to this work. Let us say I have introduced her to this work and I was saving and she has a problem as workers of Jitegemee can you assist her? She didn’t save and have an emergency. Can we come to the office and you support her because you know me very well that am a sex worker an old one yes but I have come with my other partner I want to start training her and she has a problem will you assist her or you will leave her just to start working that is the question.

**I: I can see you have many questions.**

PF03: As number 3 I have a question, you will forgive me now where I work is not one place at times I go to *changaa* dens or at times I go “away “[outside where she resides] and gets my colleague there and I can tell her concerning Jitegemee intervention, it finds that in a hot spot we are six of us there are challenges there isn’t it

**I: Yes.**

PF03: and I felt that this one I can’t resolve on my own now let me call and hear about what they are doing, am asking if I call you people will you receive my calls and come and listen about the problem or?

**I: I like the way you are asking questions because those questions will help us when making Jitegemee we will know these are the things we need to address because as the field team these are the things we need. These questions are very good we can lack the responses now but we are telling you that Jitegemee is something in the offing all your questions are good because we are taking them to the office and if we start the program we will tell them these are the concerns and questions they have and they need feedback from Jitegemee.**

PF04: as number 4 am still asking about engaging in sex, for me I don’t engage in sex in one place, I can even go to =Busia= book a room and I can go to =Siaya= and book a room, now as you people, your colleagues, if I go and get into trouble can I call you people so that you can assist me when am there?

**I: What kind of problem is that?**

PF04: I can have a problem ,you know sex work they are different ,you can get a male client and you have negotiated well for you to go the room, and he comes with hidden motives like now mostly someone comes and tells you I have already booked the room let him come and maybe he comes with drinks and then rut something in the drink and then steals from you now that is a problem ,he steals everything from me, can I call you so that you can help me or I can just continue with my work or I leave.am asking you to kindly respond if you can assist somebody who has gone for away [ meaning she has gone for sex work in another place] since parking is not only here in town I can travel and book a room in =Nairobi= then encounter a problem I have my bosses who welcome us can I call them so that they can assist me in such a such way or they can advise me in any way.

**I: Thank you.**

PF05: As number 5, when you talk about savings, let’s say that I am the one who has been doing these work [sex work] and I am the one expected to do the savings, and I am sick and the money I had saved I have used the all of it in treatment can I come to your office you support me or I wait for me to heal so that I can go back to work because my savings have been drained?

PF04: As number 4 I have something to add whatever my sister has said, as a sex worker am talking about sickness as a sex work if am sick and I have nothing in my account do I have the right to come to your office and you treat me free of charge or?

**I: Jitegemee is not a hospital, it is a program for savings.**

PF04: That is why am saying I save you have heard that when she falls sick and have used all of her savings can you help her with treatment or she can just die like that?

**I: You are asking if we can assist her with money for treatment.**

PF04: Yes,

**I: I haven't said we will help her, I had already told you that all these questions.**

PF04: We have understood, can you help her even if it is not in the project these things we are talking about you forward them so that sex workers can be helped because those who do sex work pass through a lot. If I look around here am the experienced one but I have done this work for a very long time and I know the challenges, we are going through as sex workers. I am just emphasizing on what my fellows have said if she has depleted her savings and she is still sick that is why I said that if you can support am now talking on my behalf if you can help people to rut some savings even in the account and this can make us form a group even if one of us is sick we can... I can't see my friend whom we do one kind of job with is suffering now am requesting if we can put some little savings even if it has not been passed…

**I: Thank you we are moving on thank you for the questions it will help Jitegemee. What are the normal expenses that women who engages in sex for money usually do and how much is the cost per each item? Approximately how much? Let me explain now what are the expenses that you use with your money and how much is the expenses? I am requesting if you talk you tell me whether it’s a daily** **expense and the total amount, have you understood, total.**

P: Can you repeat the question in dholuo?

P: How much do you use daily in buying what and what, the money that she usually gets,

**NT: What things do you normally buy the money and how much is the cost that you normally spend on those item on a daily basis? (NAO: cross talk)**

PF03: As number 3 the way I have understood the question you have asked in a day how much can I use and this money what expenses does it cater for, isn’t it? As number 3the first thing is food in the house am the father and the mother in that house, secondly school fees and thirdly rent I mean to say everything like for me I have 4 children and for breakfast I can use 300/= and that I have exempted myself, lunch I can use 200/= and supper 500/= that is when the money I use is more excluding rent and schools fees

**I: Now in a day for food you have used 1000/=**

PF03: In a day I can use 800/= excluding water and whatever and my fare I have not included.

**I: How much?**

PF03: Something like 200/= since you don’t know what might happen and that is a must I have to go, you understand.

PF07: As number 7 I have three children and I usually leave 250/= for breakfast since when I come from work I go direct to the hotel and have a thick cup of tea and I send them 250/= for them to use at times lunch is 250/= and supper is 300/= that is 800/= at times I use motorbike as I go to work and I want to put I want to be on another level don’t want to use the vehicle that will take 20 or 30/= I just use the motorbike directly and he takes 150/= and when returning I at times use 200/= because it is at night and want the motorcyclist to take you to your doorstep so I usually use more money ,now when am there I don’t take 50/= or 200/= but if you [The client] have 1000/= or 2000/= you come since my budget is very high.

**I: So the clients ray 1000 and 2000/=**

PF03: At least it is 1000/= and if he doesn’t want he can be because I also save 2000/= the same and if you don’t have condom you bring 3000/= and you are sorted. (NAO: Participants chuckling)

**I: Another person, daily expenses? Number 9 do you want to talk?**

PF04: As for me I have one child, I usually use 700/= per day and I have not included water and soap and I have not also included the money for UBER that I use to town, secondly I have to use makeups because you can’t go to Njara [Meaning job] to work when you are not dressed well, thirdly it is a must when I go I have to go to the club, first of all I have to take two drinks and put them in front of me. Now …

**I: How much does it costs?**

PF04: It is something like 1500/= cause I take UBER for me to be there and it is a must I have to put on makeup, I can’t just go there like this, like today I was washing clothes, I have to use makeups, make my hair so that I can attract a man, now I get inside and take my two balee [Balozi] its almost 1500/= per day.

**I: What is Kabalee?**

P: It is a beer, balozi.

P: Where are you njiwa?

PF04: Okay when I go there you know we are looking for money and am not lying I go there and get someone for shots and for shots he wants to give 500/=I will not reduce that amount because I had already removed my savings and used in the house that is for shots and whoever has 1000/= I will also take ,and whoever wants sex without condom even if I take 3 or 4 thousand there is no harm there, I see it is good and there is no problem and maybe it was your day for the shotss and you get 7 clients where each one of them gives you 1000/= then you shall have gotten much.

P: And you know for shotss you don’t penetrate so much he just put the tip of the penis then removes it. You time him (participant chuckles) and when you know that he is done then you rush him away, his time is over. Give me the money.

**I: Okay now your expense is like 1500/=.**

PF04: 1500/=.

**I: In a day.**

PF07: As number 7 what I want to tell me colleagues as the people we work with in the same job, on my side if I go to work I usually tell my clients to deposit money in my phone before the engage in sex with me because there are some men who are not honest he will come and tells you after he has already had sex with you and tells you he is not giving you money what will you do? And already he has engaged in sex with you ,now on my side you have agreed let us just go but you will deposit the money in my phone and are you going not to use condom or it will be flesh to flesh , they may say it is sweet without condom, then he deposits the money I my phone, once he has deposited the money in my phone there is no way he will reverse , because it has taken time and he has already deposited the money and we go and he does the work even if he does it until I reject the money , but he will just engage in sex with me but already he had deposited the money in my phone now my fellow even if you go there try as much as you can they deposit money in your phone.

**I: For us not to divert from the topic so much remember we are discussing about the daily expenses.**

PF04: Daily expenses. I am number 04, we are still we will talk and we help each other, you came to help us and we also want you to help us I will respond in regard to what my friend has said, something like that had happened to me, I went to a lodging with somebody and he has done his work and put on his trouser and told me he is going to the toilet this man went away running, now in that situation how can you help us? We went and he satisfied his urge and he is dressed and I know I have a partner and he has said he is going to the latrine to urinate and he disappears what will you do to him? Surely how can you help us or what advice can you give to us? I feel that the advice that my sister has given is a good one. ‘*’Pesa kwa mkono kuma kwa kitanda*’’. [NAO: meaning you have to give her money before you engage in sex with them]

**I: That is number?**

PF03: *Number 3 Pesa Kwa mkono kuma kwa kitanda*. [NAO: meaning you have to give her money before you engage in sex with them]

**I: I am requesting those who are quiet can we talk, there is nothing wrong or good and all things are the same in this discussion. Just be open and say whatever you have. We continue I would like us now to talk about the weekly expenses, we have already talked about the daily expenses, now the weekly expenses that you do once in a week, they are like and how much do they cost?**

R: Something like oil,

**I: Number?**

PF08: As number 8 in a week something like oil, you have to buy because cooking oil you can’t use it every day it is something like after 3 to 4 days now soap price is high it is 200/=

**I: Are you talking about petroleum jelly.**

PF08: Its petroleum jelly because you have to go there when you are smart lip stick 150 and you have not bought body oil if you mix it comes to something like 500/=. So in a week you can buy lotion, soap and make ups and …

P: Food you have to eat.

PF08: Food is a daily affair we are now talking about weekly expenses.

**I: In a week all of us always do weekly expenses, kindly those who never contributed the other time to do so.**

P: Okay in a week I buy food, makeups there is this oil that we use in our private parts so that it is tight and that oil is 3000/= it is a must you have to have that oil , when you are going for sex work and after someone has done his work you use it so that it can be tight, so that when that person penetrates you now it will be tight and when he comes again tomorrow you are his client and you won’t miss that money , now in a week I can use 5000/=(Participant chuckles) that is apart from food and whatever it is 5000/=.

**I: So that 5000/= is the weekly expense?**

PF08: Yes.

PF07: As number 7 it is true that we normally eat every day and we had talked about it now in this our budget of one week, something like oil, soap cobra perfume I have not just sprayed it now you would all be running away, it is a powerful perfume and usually it is over by one week and it is 700/= and I must buy lubricant oil because you find that there you find men with big penises, now that lubricant when I buy it when I go there I control myself its use since I know the men am going to get and may be the man I get the penis is bigger and when I use the lubricant for him it won’t come to his mind to go and buy it and it is a must that I just have to buy and keep it in my purse for emergency I use my lubricant so that when someone else (another client) comes I don’t have any problem (she does not feel pain during sex), so that thing(the vagina) can’t be annoyed (does not get bruises)so in a week I can you that 5 if I buy those things.

**I: Can you clarify about annoyance?**

PF07: Meaning that if I get a bigger penis if I don’t have lubricant oil it will penetrating will be difficult, he will hurt me you know this lubricant oil if you use it will penetrate softly however big it is until you say waah! I have gotten my size [NAO: participant chuckles]

**I: Number 6 talk.**

P: Number 6 you can’t talk.

PF06: I have nothing to talk about.

P: Why don’t you talk, you talk at night but now you don’t want to talk.

PF06: No.

P: She still has something in her mind she will just talk.

**I: We continue the expenses that we do once a month and it is how much what are they let us start with number 2**

PF02: The monthly expenses that I do in a month, for me I have a child there is someone whom I leave the child with I have to pay her, and she doesn’t take less than… she takes 3500/= that is in a month, something like cooking oil I have to have extra 5 liters is 1500/= and some things like soap I have to have extra so in a month I use roughly 12 or 13.

**I: Hundreds or thousands**

PF02: Thousands.

**I: Thank you, number 2 is there anyone who has something to say? Do we normally have monthly expenses?**

P: Yes

PF08: As number 8 something like school fees, my child is in a private school, and we pay 4600/= monthly I have not included lunch which I pay monthly which is 1500/= I have not included fare, transport I pay in school that is also 1500/= it is something like 10,000/=.

**I: Is there anyone who wants to share? Okay we continue there are those expenses that we normally do** **once in a year or once in three months or 6 which ones are they and what is their cost?**

PF07: Let me not lie for me I normally do a weekly, a monthly budget because in a year those things can expire, I like one month because in a month if I use 20000/= for those things apart from fare that I usually go to work with roughly 20,000/=.

**I: Okay here we have the expenses that we do once after 6 months or let us move on now where do women who engage in sex for money do let me read it well first, women who engage in sex work where** **do they get their money from? Where do they usually get the money to spend?**

R: That is the reason we engage in sex.

**I: Number?**

PF03: Number 3 there is no where we rely on we only rely on sex work, ‘*’kuma kwa kitanda pesa kwa mkono*’’. Pesa kwa mkono kuma kitandani, [NAO: you have to pay first is when you will engage in sex with her] that is the business we engage in. that is where we get the money we spend.

**I: Is there anyone with different way of making money?**

R: No, that is the only way.

**I: Number 10 has something.**

PF10: The way my friend has said that is the livelihood.

P7: If I want to say something. when go to octopus the Indians normally give us their dogs to engage in sex with them and those dogs if we engage in sex with them for two hours you will be given 5000/=, and that is an advantage, if we get those dogs we engage in sex with them, we don’t want to know [NAO: they are not bothered].

**I: Okay thank you, amongst yourself there is no person who has another source of income apart from** **sex work**

P: It is the only source, the vagina is the source of income [we engage in sex] and it can’t get finished. And if I had God’s number I would call him and appreciate him.

**I: Thank you, number 2 is there something you have, now I want all of us to talk we have all agreed that we do sex work now I want each one of you to tell me how much they get in a week. The money** that **you get, we will start from here.**

PF02: Commercial sex work you can expect in a week am going to get 5000/= or 10000/= it depends with how it is, let us say in a week…you can be lucky and in a week you make something like 20000/= that if when you are lucky, and at times you are not lucky and you make around 5000/- so it depends you can’t explain well this is what you get in a week or month.

**I: Thank you now you have helped me to ask my question well, I want us to say if the week is good it is this much and when the week is not good it is this much? Will that help us now number 2.**

PF03: If the week is good I can get 10000/=and if the month is good now that one you have hit the jack pot, in a bad week you can get 5000/=.

**I: Thank you, let us continue like that you say your number and tell us when the week is good and bad.**

PF07: Those who are quiet ,can I say something , as number 7 in a week I make money because when I go there am very special since if I sit at a table my drinks are always there meaning they come for those who have money, I also dress smartly and that attracts customers [clients] now in a week I can even make 20000/= .and when I get that 20k I will say that this week I have made a little money I usually make money ,if I tell you without condom I make 5000/=, and I get like 4 men or 5 in a night or at times the way the schools were closed for two months I used to go even during the day so as to get that sonko [ rich man] who has a vehicle and I go with him then at night I also come back that is why I like using the lubricant oil it doesn’t harm me now in a week the least I can make is 20000/= and per month eeh! I have savings and about to buy piece of land.

PF01: Number 1 if I leave this place I have to visit the herbalist [dumba wa dumba] he gives me medicine and I bath with it when I want to leave, there is also another one that he gives me and I put it under my armpit and he tells me before I reach the place of work, I shouldn’t talk with anyone so when I reach at my place of work I will talk with my workmates and customers/clients just come they even leave the one who is nicely dressed and they only see me and when it reaches in the morning, I will have made even 30000/= now our problem is only one ,you will get 30000/= but when you reach home at times you used it in buying alcohol ,you ate chicken that money won’t help you in any way ,so we always remain the way we are just like that. And again tomorrow you go look for money and there is no money you just understand that is the problem, now at least the way you have come as Jitegemee, I want to at least help us…so that …. the way you have brought the matter of savings and find a way on how you can assist us and whatever amount of money we get we be saving but you don’t say that we save in the house that one you will be lying to us. We will consume all the money.

**I: Thank you we only have like 5 people who are talking, number 5 you are also doing this work let us just talk.**

PF05: Am coming up.

**I: Okay number 4.**

PF04: You don’t see me.

**I: Sorry number four the time is yours.**

PF04: As numbePF04 in a week if it good you can come out with 20000/= you know a prostitute is someone who likes good things, not only physical appearance. Just go to a prostitute house you won’t believe that is a prostitute’s house now that 20000/= if I get it that issue of saving if we put something small there and that saving will help us since there is no a prostitute can see a beautiful seven seater| chair she will just feel like going to sell the ones she has and take the seven seater, there is no money you can keep in the house, a prostitute is somebody who likes smart things and love to see her house to be smart even if she gets sonko who has UBER when she takes him to the house it is a BA, so in a week 20000/= if we find away on how we can save little by little, it will help us but if we save in the house it is not going to help us because…

**I: Thank you.**

PF04: Yes because a prostitute is somebody who likes beautiful things in her house that is around 20000/= is not bad.

**I: Thank you, now it is good you have talked about…. you have not finished?**

PF03: As number 3 I want to say something just as my colleague have said there are some people who like engaging in sex without using condoms, you know the one that no condom is used ,you have to increase the amount something like 5000/=, we have the female condom, you know if you insert it earlier that man will not know 5 if you have the condom, he will just know that he has engaged in sex without condom( flesh to flesh) now if you have not used condom may be you have gotten like 5 people ,or 7 and for that 5000/= that one I have made a lot of money but to save is a problem. I don’t want to lie to you, saving is a problem. I like when am coming from work and sees something smart, I want my children to be beautiful because the moment you decided to be a sex worker know that you have a bad name even within the plot. You find out that your child is being abused verbally, go away your mother is doing sex work,’’ now Át times I just want my child to be beautiful /smart even if she is a prostitute’s child but she is more beautiful than those who have both parents. I am just asking you teach us on how to save. That is my opinion.

**I: I like the way you have talked about the expenses because that was my next question, what makes you to spend the way you do? What makes you to spend your money on those expenses one person said that she wants to be seen beautiful number 4 you talked about that, you want your house to be smart that is one way and what other ways …You will finish first then we come to number 8.**

PF03: As number 3 I am still repeating, what makes us spend or I spend like that is once you are a prostitute you have a bad name, and you are not supposed to have a bad name and the work that I am doing makes no difference it’s supposed to be something that if you are stigmatized, you prostitute, you respond to her/him and tell him/her that’, ‘’you are a prostitute but just look at the way I have planned my things and you who is not a prostitute how do you look? That is the reason I spend money like that so that I can take care of myself, make my children be smart and also make my house smart and everything is neat even if it is in the church, or there is a sex worker who doesn’t go to church, you know that book in the church the one of 10% you are okay and you give out cheerfully it has to be high exceeding those who have wealth.

PF08: As number 8 when you come from work in the morning ,you have money and you have seen a smart trouser you can/t leave it you will have to buy it, you see nice shoes on your way to the house ,you see yourself that you have money, and it money that is controlling you ,that is what is harming us, if you see something nice on the road you buy, you are seated and you see a nice dress for a child and that shoes is how much and you are told if you look at your pocket you have money and you buy when you reach the house you find that your savings are low, because you have bought so many nice things on the way.

**I: Thank you is there anyone [cross talk]**

PF03: Let me add something small another thing that again that contributes I use my money like that because I know tomorrow I will still get and I see that I have not done too much work we have all enjoyed but for me I have gained at least have gotten money and say that tomorrow so there is no need of saving After all I will get money so there is no need of saving.

**I: Number 2.**

PF02: What drives me to use my money the way I do, most people say that sex workers have no life and I want to prove that prostitution I want to be above you, I dress well and my children more than your children so that perception drives me to buy good things, stay in a nice house, that is the reason why I spend the way I do.

**I: These are personal things can we talk about the things that we buy for others, as for you, you want to** **dress very well and your children to be seen smart,**

PF04: They eat well.

**I: They eat well, your house is smart and you have also told me that you want to prove to other people** **that his kind of work pays even if you talk am above you.**

PF04: I educate my child.

**I: That is an external reason, is there any other reason apart from that?**

P: Reason?

**I: Yes**

P10: Thank you, sex work .

PF04: Which number, say your number.

PF010: Number 10 they call us prostitutes and we are better than the working class by far because I have 3 children, you want to feed them, they go to school everything is your responsibilities, you have rented a house, your mother at home always calls you from home, my daughter send me money, you can tell your mother the kind of job you are doing so long us you have money, you will tell her mum I am sending.

**I: Thank you is there any reason**

R: Yes, there is a reason as you see us sex workers number one we are competing and the way are competing is like this if I see my fellow has a shop, others have cars, I feel I should be like so and we tend to live the life of others and I go on if I see someone else I want to be like her.

**I: Thank you.**

P: You have interest.

**I: Thank you so much, I like this discussion in normal situations do sex workers have some savings?**

P: Yes we do save.

**I: Let us start with number 8.**

PF08: As number 8, you know you have to keep some savings because you have rent to pay, school fees waiting for you, you have to save. Even if it is little but you have to save when it reaches month end you are sure of paying rent and school fees now it is a must we have to save. Even if we are taking alcohol, you know if you have received a lot of money you have to celebrate and there also you can’t go when you are sober

**I: Empty headed [meaning not drunk]**

P: You have to take something small, if you go there you twist your waist for someone for you to get money.

P: You be yourself.

P: Yes that one, you twist your waist (entice the client) each and every style.

**I: Ooh you do all styles and it is a must you have to?**

P: You will tell him, have you seen that did you know that it would be available.

**I: When you said that you have to put something what do you mean?**

P: You have to be tipsy a bit, you have to drink to ignite (take alcohol) or to start a vehicle has to have fuel. [Say what the participant meant by this]

**I: Thank you, number 9, number 9 is not talking do we save?**

R: It is okay she doesn’t want to talk.

**I: Amongst us there are those who take time and later on them talk and there are other who are very talkative.**

**I: Let her talk first.**

PF07: As number 7 we have to keep savings like me am away from home cause my mother is in those sides of =Homa bay= and I have come to =Kisumu= to work and in =Homa bay= they know very well that the daughter of so and so is working in =Kisumu=, I don’t want my mother to be stressed going to beg for salt, I want that when immediately she calls, halo my child am badly off, my mother doesn’t know that am a sex worker and it is a must that I will send her the money even if I go home ,they will say have you seen the daughter of so and so the way she is beautiful even if am smartly dressed and they be amazed where I work and say that she works in =Kisumu= yet they don’t know the type of job I do in =Kisumu= ,now the savings I need whenever she wants to pay her loan ,she likes borrowing loans when she tells me that she is badly off it forces me to take my savings and send her for her not to be auctioned by the loan people ,that is why savings has to be so as to help my mother to run her loan well , and also her children to have food on my account so I have to save.

PF09: Number 9, I do save the reason for me saving is I have two children, I stay with the one and the other one stays with my mum, maybe she can fall sick and she goes to school in our rural home. My mother can call me to tell me that the child is sick and I send her something and this one can also be sick and I also have a maid who takes care of the child, It is a must I keep some little money so that when it is end month there is something I can give her so it is a must I have to save, maybe that month I may not succeed like the others days I usually get, maybe money can reduce there are challenges like that. Now I have to save.

**I: Thank you so much, for those who will talk next I want you to talk about after saving how much do you save and how many times in a week or how many times in a month? And anyone who has something to add will do so we will start with number 1 as we continue like that I have seen your hands are up**

P: Can you repeat the question you have said.

**I: If you are talking about saving also talk about how much you save and how many times it is weekly or monthly? Or if you are not saving you can say.**

PF01: The money that I save, I save after every week, I mean I save weekly, I can save 2000/= in a week I don’t save much because at times I can get some problem and again go and use the savings. I don’t always want to use the savings because it’s for my sister and my child to join, now I know I will use the savings to take her school and my sister is also in class eight and she depends on me,

**I: Your child is joining?**

PF01: She is joining nursery and I am supposed to take her tomorrow, now I will use the savings to take her to school. The one who is class eight that is also my burden, I have also to pay for her school fees and weekly I normally save 2000/=, I am the one paying rent, there is no day that the land lord has come to my door that I haven’t paid him, that savings is what I give him when it is month end.

**I: Thank you, number 4.**

PF04: As number 4 in a week I can save 3000/= that 3000/= if I save I have a child who is going to school, I have my mother who is a widow and I have to carter for her since she is very old and I have to make our home smart even if one day I have a problem and the sex workers come to our home they say ooh it means [mentions name] was working and she had savings now I save 3000/= that 3000/= I n a month I can use it for school, I can save and send my mum.

**I: So are there a certain characteristics of those women who are saving are having? Women that do save.**

P: Yes, there is.

**I: You have certain characteristics; we have said that we are saving?**

P: Yes.

**I: Now I want each and every one who said she is saving she can tell me the characteristic s she has.**

P: I am not understanding about the characteristic.

**I: It is like characteristics… what is it that you have that is different from those who are not saving isn’t it? Now you understand.**

P: Yes.

PF08: You have saved , during end month you have paid rent, schools fees and other things and you still see you have remained with some money , you go to the club and tell your friends to come there is money for alcohol,

**I: Now one characteristic is that you know how to spend.**

P: I know how to spend, after I have paid rent, schools fees am now free next is alcohol.

**I: Be audible.**

P: We don’t like begging for alcohol from men that can you buy for me alcohol, I like it when I go there and the waiter serves me eeh! And I also enjoy as I want.

**I: Those are women who save, is there any character they are associated with, character doesn’t mean it is bad it can be good. How are they that when you look at them you will say surely this woman is saving?**

PF07: As number 7, what helps me to save is that when I go there and a client comes and he wants to pay 200/= I will refuse and tell him that 200/= is not enough because I have saved and I know I have money now I won’t take that 200/= let him go back and come when he has money, that is the importance of my saving.

**I: I want number 05… number 6 you have not talked.**

PF06: I haven’t gotten the question.

**I: Am asking what characteristic of women who save have you seen?**

PF06: That will make me now not to be somebody who keeps begging for things from people and my children are smart all the time and any time.

**I: What characteristic do women who save have that which you can talk about that those who look like this are the ones who are saving?**

P: The characteristics of smartness.

PF04: A person who saves like me after I have draft my budget I like taking my friends and we go to hippo point and buy Guinness worth 1500/=. and my friends whom we work we can go to hippo point and enjoy that is my problem with savings if I have money I just see hippo point and that I take my two friends we go and we eat, enjoy and I feel like somebody who has money but I don’t have anything now that is my problem.

P: Okay for those who are saving we are always proud ,you know there are some who came while they are still young and it is us who lecture them, as the older one we have a group like them and we want to go to Tanzania and make money , and there we meet white men and where we keep our savings one day when we get good money we cross and go to Uganda or Tanzania in those big hotels like Mombasa they do that , you find that they just go to the hotels and the managers of the hotels ,sex workers they have our contact and they call you when the white men arrives that is what we want to go and do in Tanzania when the white men are in Tanzania they call us and we go to Tanzania clients are available come on this such a such date, so you know the saving it helps us when they calls us we leave and go to meet them.

**I: Ooh because you have savings?**

R: Yes.

**I: For those who don’t save what are the characteristics?**

PF01: They beg continuously, team beggars

PF03: Those who are not saving they are in the team of begging if they see that you are smart they are jealous (cross talk)

**I: Remember we said that we talk one person at a time number 3 finish first.**

PF03: Those who don’t save are in team beggars, then they are jealous, if they see that you are shinning in terms of the way you dress they start saying look at her she has done this and that but her she is unkempt like charcoal.

**I: Number 10.**

R: 10: I am repeating what my fellow have said there are those who leave in the evening ,because we usually leave at 10;00pm or 7;00pm , if you pass someone she will say that she has left and she is going to park ( sex work) she is talking like that and she is seated and there is nothing she is doing , when you come back in the morning she sees you carrying your bag and they starting gossiping you see I told you this one is a sex worker and you surpass her by far if you go to her house there is nothing she has yet she is married and us we don’t have husbands .

**I: Remember we are talking about women who engage in sex for money who don’t save, what characteristic do they have number 2.**

PF02: You can find that no …those who are not saving in case they have an emergency she has gone to work and finds a client who wants to give her 200/= it will force her to engage in sex for 200/= and she doesn’t charge 200/= because she has no money but have an emergency

**I: That is what you talked about?**

PF02: Yes.

PF01: Whatever she has said is what I wanted to say, those who are not saving you will find a client’s comes at times he is somebody ,you know there is a way we spot these men ,you see one comes with limousine and he won’t have sex with you for 200/= ,he will give you 500/= but the one who is not saving will feel that 200/= that will help her but for me he can’t engage in sex with me for 200/=I will tell him 5000/= so those who are not saving are different from those who save.

PF04: Those who are not saving mostly they are always jealous, if they see that you are faring on well she doesn’t like your lifestyle.

**I: Who are they?**

R: Those who don’t save, and you are saving and she sees you have done your things smartly she is jealous with you.

**I: Thank you is there anyone who has something to add?**

R: It is minor, those who don’t save they have one problem of keeping on asking where you are and we are leaving at what time, those are the ones who don’t save since she wants to use one motor bike with you and they like to be parasite she wants to drain you, I want you to understand me very well

**I: Am getting you**

R: Today where are you and at what time are you leaving? That is someone who doesn’t save, she wants us to board the same motorbike (NAO: someone whispering) she has no fare.

**I: Thank you, we have discussed and said the reasons why we save Isn't it? Now I will not ask that question again but I will ask what makes saving to be easy to those who are saving?**

PF01: when you want to use your money you can do so , in case of any emergency there is somewhere you can fall back to ,when your child is sent away from school you can use your money and pay ,rent that is where you rely on,

**I: Is there any one with different opinion? We don’t repeat what have already been said. Number 4 then number 3.**

PF04: A person who saves is very happy and anything that crops up she can go to her account and withdraw and sort out her issues, that is someone who saves and if you are not saving even if you receive information that your sister is sick and you have been working you will really feel pain, (bad) it is not easy.

**I: Just say even in dholuo**

PF04: Now there no where you are going to get her money and when you are saving and she is sick it is easy for you to help her that is why savings are good.

**I: Number 3.**

PF03: The reason am saying that saving is good ,like me I play go away match ( going to another town for sex work) for example I left in the morning and I left the house just like someone going to the loo and I have been here till this time and I know that I have saved there and I have told my children if they see am late and it is time for lunch or supper they go to a certain place and take certain things, that is why I find it easy to save.

**I: Thank you, what are the challenges that we face when we are saving?**

R: One challenge is doing what someone is doing, you saved very well but I have now seen my fellow has done something and it forces me to take my savings and do as she did.

**I: Another challenge, number 3 is there any? It is one only okay this challenge of wanting to do what others do, how do we overcome this challenge?**

**I: What are the challenges we face while saving for those who are not saving?**

PF07: Having too many children , if you have many children there has to be a challenge because the budget will be high and you are overwhelmed .so it will force you that the money you are saving doesn’t last for a long time , we now need to do family planning do not just give birth.

R: Like it is an emergency that has arose. They have called from the school that your child has been hurt ,you will have to go and get the money from your savings to go get treatment this place where we work in =Kondele= you might think that you carried the lubricant oil but you forgot now you are already in bed and you think of slipping away go and use the lubricant but the client is not giving you that time, it will force you to go to the hospital because you are bleeding, it will force you to go get your savings and use.

**I: Thank you, these are the challenges we are facing, how can we overcome them for us to save? I say it in dholuo?**

**I: Okay she is saying what is it that we can do so that …the challenges we have talked about that can make, someone not to save, what can you do so as to save?**

**I: Number 8.**

PF08: As number 8 I feel that the way you have come with =Tuungane= it is good just open for us accounts let it be a fixed account, like you say let me put 200/= every week and you can’t withdraw, that is what I feel will help us.

**I: Is there a way we can overcome this? Number 3**

PF03: as number 3 am just requesting willing the staff of Jitegemee you have done well and we appreciate for you people coming and at least give us some advice, if even I open an account or even if there is another way of you advising us and see away of us saving it will be good.

**I: Thank you so much, now I would like to know the disadvantages of not saving? What are they .i think we have talked about them.**

R: Yes.

**I: I want us just to mention in brief, because these are questions and we might have discussed about them concerning the problems while we talking about other things because we need to read the questions the way they are ,you will just say that we said this and we go on.**

PF01: There is no way you will be able to deal with an emergency case.

PF09: If you don’t save you will be really stressed,

**I: Now I would like to know where you would want to save. Where would you prefer to save?**

PF07: If you fail to save you will yearn for other people things, I would like to save in account where I can get loan.

**I: Bank.**

PF07: Yes

**I: Another person?**

R: Mshwari.

**I: Mshwari number…6**

PF04: Equity it is a bank and we will decide in which bank

**I: Number 5.**

PF05: Bank

**I: Bank, thank you now I want us to talk …we still remain with like three section and we will try and be fast. First I want us to talk about Jitegemee I am sorry? (Some murmur in the background) what will help us is if somebody says something and you have nothing more to say don’t repeat**

R: How many sections are remain?

R: 3 sections

**I: Yes. Because when you repeat but we don’t also want you to stop before finishing whatever you are saying now number 1 will say something ,number 3 have also raised her hand ,you see it has consumed time now don’t repeat what your fellow have said that will help us. The way we talked about Jitegemee we said that it is a way whereby women engaging in sex for money can have some savings from the money they usually make if you are making 5000/= in a week and you have decided that 500/= that is 10%,I will save 500/= in Jitegemee , jitegemee is an account somewhere ,not your account in the house ,it’s an account somewhere where you save there, you will save when you want, when you have and when there is no money there is no work then you will not save, it will help you when you want to go for a leave( break) it will help you in times of emergency , it will help you when they is no work ,it will help you when you feel that you don’t want to engage in sex , and it will help you when you want to retire okay. What we have said now we want you to give us way forward the way you feel jitegemee should be do you think Jitegemee is something women engaging in sex for money will embrace? And when you say yes you also tell me the reason they would embrace it. If you agree you tell me the reason,**

**I: It means that jitegemee we are talking about do you think that women engaging in sex for money will like according to the way she has explained?**

R: It will help because.

**I: Number?**

PF08: As number 8 I feel it is good it is going to help us because it reaches a point when you don’t have money I mean you don’t have money completely, and you have a problem you know I can just come to you people and say check for me my shares is how much and say can you give me 5000/= so that I can use that thing is good, I support it.

PF07: Am number 7 on my side the women who engage in sex for money it is good because sex work is what takes me there am a mother who doesn’t know business and business will force you to have money so as to get the goods, you sell and maybe there is no profit you have gained, you are stuck but when I engage in sex today and got 1000/= or 2000/= there is no where I have used money to get the goods. I am advising those who don’t know business, this job is easy and there is no where I have gone to get the goods.

**Í: Thank you, what type of women who engage in sex for money will accept it? They will accept Jitegemee we have talked about the type of women who engage in sex work, now which ones will accept and why? Number 9**

PF09: It will force them to …because it reaches a point when it is difficult, now you just take them and you advise them again you are supposed to put something like loan that someone pays back with interest, more people will join

**I: If we have loan in jitegemee?**

PF09: Yes .loan and they pay back with interest (someone talking in the background, I am dying)

**I: Another person who wants to talk which people will accept.**

PF02: The people who will accept are women who engage in sex for money and they are saving. Those who like saving it will help them

PF08: As number 8 it will help those who don’t know how to save, in the house,

**I: The person who talked before number 8 is number 2.**

PF08: It will help those who don’t know how to save in the house.

**I: It will help those who don’t know how to save in the house, and which people would not accept it?**

R: pastors (participants chuckles)

**I: like us now remember Jitegemee is about us.**

PF01: women who don’t save, the people who are not saving will not accept. But those who are saving… even those who are not saving they will just accept. Because they will be able to see the way those people who are saving are developing so they will also long for them too to be like those who save.

**I: You are number.**

PF04: Saving in the house it is a challenge ,because saving , I have placed a tin am telling you if I see something in a fellow sex workers house and sees she has bought a fridge am going to destroy my saving bank and buy the fridge, and account will be good.

**I: Having account is important**

PF04: In the house it needs to have the one for sickness and …

**I: Now from 10 women you know not the ones who are here those who are not here how many do you think will join Jitegemee?**

R: Most of them (talking in unison)

I: If we have a group of 10 people out there who are sex workers you think out of 10 how many people **will accept to join.**

R: 8 of them.

R: Or 7 of them.

R: 20%.

**I: Number 10 and number 2 you have said.**

PF04: 100%.

**I: 100%**

R: 10% will join.

**I: All of them.**

R: Yes.

**I: Okay number 2 those 2% who will not join what are their reason for not joining?**

PF08: As number 8 sometimes they charge low price, to extent of having sex besides the lorry 50/=

**I: If they charge cheaply what does it do to them?**

PF04: You know if they charge cheaply they will be given 50- 100/= and when she goes to the house she will use all of it. What will she save?

R: There are those who don’t believe that they are sex workers and they don’t want to be identified with the other sex work they are doing sex work privately.

**I: Let number 4 to talk first.**

PF04: Still thinking.

**I: Number 10,**

PF04: I have remembered.

**I: Let number 4 finish first.**

PF04: some are saying that they have husbands and they want to be like us and they can’t, they want to join but it would be difficult because they have their husband.

**I: Now they won’t join jitegemee?**

R: Yes

**I: Number 4.**

PF04: Is somebody who if she gets money she cannot join this group… Is somebody who if she gets money ,you know somebody who likes eating because there is someone who can have 1000/= and doesn’t know how to use it ,when she gets 100/= she sees this and she doesn’t see the future she will find it difficult to save that money

PF01: to add something am number 1 is somebody who has no child, she has no plan of even buying land ,so she will feel like there is no need of saving since it is not going to help her it is better she gets the little she gets she feels she needs to use it all.

**I: What can we do so as jitegemee so that many women can join? Number 8**

PF08: as number 8 let this jitegemee be like a Chama, let it be like a merry go round

**I: Ooh merry go round. Number 4**

PF04: Secondly let us look for all of them because so that you to come for another meeting and let them hear on their own so that they know that it is true.

R: As for someone like me I will be an ambassador for the other person, I am going to tell her what I have heard here about jitegemee ,jitegemee wants us to at least save ,I will now explain to her and tell her more if she is somebody whose understanding is good she will hear and then join. She will ask (mentions name) where you went to. Or where did you hear about that information or the teachings? And I will tell her.

**I: Number 5 what can we do to make many women to join Jitegemee?**

PF05: Am hungry

**I: Eeh! Number 5, the recorder has not recorded the voice of number 5.**

P: There is too much hunger

**I: There is too much hunger**

P: Yes my client is going to leave me

**I: Okay let us speed up now, how can jitegemee be implemented how should it be? How do we implement it, how should we do it? There is one that you said that it be like a merry go round,**

PF03: As number 3 in my opinion I feel that if the group of jitegemee we can plan even twice in a month and see away on how we can meet at a certain place,

PF07: I feel that as the staff you go to the office and tell the manager let them have some savings that when one of us has a problem we can come there and you even be responsible for the hearse or coffin. Yes we can say that I this our job that we do we have a manager whom even if one person dies we approach her as friends of ( mentions name) we are responsible for the coffin, you are getting me ,go and tell the manager you form a …so that when one of us die we just come ,I am the chair o, I come with my secretary and vice and inform you that (mentions name) is dead ,what are you going to do for us, number 7 s dead now give us the coffin

**I: [mentions participant name] is not number 7**

R: Number one

PF04: We are used to 7 in the field, which is what you can take to them so that you tell them on how we can get support from them

**I: Okay thank you very much, are there components that jitegemee has to have for it to be mostly be accepted by women?**

R: Yes

**I: Like which ones?**

PF08: as number 8

**I: Number 2, let number 8 to talk, number 2 number 8 you will talk after number 2**

PF02: To make many people to accept it at least we see on how we can take insurance and if one of us is sick then the insurance will carter for treatment something like that

**I: If one of you has a problem then the insurance to intervene, number 8**

PF08: Yes

**I: What components must it comprise of for it to be acceptable?**

PF08: as number 8 just as my fellow had said we have things like hearse, you be responsible for the coffin things like that not only in death or you have encountered another problem and it is difficult and you are unable we come to you and help us.

**I: What thing should jitegemee have that the sex workers won’t like?**

P: They won’t like

**I: Yes, let us start with number 3, number 3 do you want to talk about this or the previous one?**

PF03: I wanted to say something about what we had talked about

**I: You can talk about that then this one,**

PF03: as number 3 in the field there are challenges as number 3 am telling you to go and say this to the office ,the two of you to take the message as number 3.let them stand with us as sex workers there are challenges in the field you can go and be arrested, and if you are arrested you don’t want your family to know , like for me I don’t want my people to know the kind of work I do you know it will force me to die on my own ,because when they come there if I call them and they come there they will ask why I have been arrested ,they will know the main reason and know the kind of work I do, now go and ask your office if they can stand with us during this period I feel the project will be okay.

P: I also suggesting at least they give us support you know money issues are always tough even if they say that sex workers monthly if they give us 3000/= you know we love money I will say that this week I will not go to work .I am waiting for that money that I am going to get something small from Jitegemee, and from that 3000/= I will save 2000/= or 1000/= and some will go to the budget, and some will be for the school now I feel that if there is support many people will join. The support will help us.

PF04: Am requesting you give us certificate.

**I: Why?**

PF04: Because if I go somewhere even if I go to Tanzania, I have the certificate and there is no one who will disturb me because they know I am working and have a certificate. I have my working certificate.

**I: Okay we have talked about what we would want Jitegemee to have for it to be acceptability, what is it that sex workers won’t like, is there anything?**

PF03: As number 3 what I see it is like you came and collected information from us our and then just disappear like that , next time if you come back as number 3 I will just say stop wasting your time there we are not going.

**I: Number …**

PF07: Number 7 what I feel should not be there as sex workers I want you to take this message to the office as number 7 am saying matters with security ,the police keep on arresting people , when I am by the roadside that time is over ,how arresting people and taking to the cell, they need to be talked to the way they are arresting people will make me if they arrest me the first day I will say that place is not easy to be there.at times there are no customers in the club and now I go to the streets I know they will arrest me and it will make me not to go there because they had arrested me and I gave them money. If it is free then the police will not arrest me and I will do my work .let them talk with the police if they are caring about us.

PF04: As number 4 it is the certificate that will help us because if I have my certificate and the police arrests me you will show him the certificate to read the way he can come and arrest me and he has the police tag as also we have sex workers tag. We need certificates and tags.

**I: Are there issues with the human rights that will affect you in Jitegemee, in jitegemee do you feel there are going to be issues of human right.**

**I: As per what they have read about jitegemee do you feel that your human rights, you know human rights**

P: Your rights

**I: It can affect your rights as a sex worker?**

P: No

**I: Number**

PF08: Number 8, I say there is none, it is okay with the human rights.

**I: There is none.**

R: Yes (participants responding in unison)

**I: What challenges do you think we will encounter as jitegemee people when we come to the field to work with sex workers?**

P: As number the challenges that you will encounter in the field when you are not trustworthy to sex workers you will have a challenge in the field. They are always talkative maybe I woke up with my issues its morning and I have not unlocked. And if you come you know we will disagree so you find a way on how you approach us.

PF01: The sex workers they are tough headed , whatever they have said that is what ,and when you say something you have to do as you said , then when you talk to them nicely that will make us understand each other when you come.

**I: Is there any?**

R: There are over.

**I: Now if you join jitegemee how much can you save?**

R: If you join, in the account or?

**I: If you join jitegemee.**

R: As number 3 I can’t tell because I can’t have a target because we haven’t seen the way we are progressing I mean growing.

**I: So for a start if you are joining Chama you will start with savings and they will ask you how much can you start saving.**

PF08: 100/=

**I: 100/= per day or week or monthly?**

R: Per week

PF03: Per week .for the start I can be weekly, I can save 200/= per week

**I: Number 6.**

PF06: Per week

**I: How much per week?**

PF06: 200/=

**I: Number 8 you said?**

PF08: It is that 200/=

PF010: In a week I can save 250/=

I: In a week you can save 250/=

PF04: Number 4 in a week 200/=

**I: In a week you can save 200/=**

PF01: Number , 200/= because we have too much wants, if we save much it will force as to go and be withdrawing now it won’t be savings .

**I: As sex workers they are targets that you want to meet or at the end of the day may be you have Chama where you take money or credit and you say that I have to leave with these from the field so as to meet your target, what are you doing till you meet your target?**

PF07: As number 7, the target I will be having is that for those who will want shots I will go with them.

**I: Elaborate?**

PF07: I have said that …

**I: What will you do to meet the target?**

PF07: The target for my money

**I: Yes**

PF07: that is why it will force me for those people who love shots time I will have sex with them so that I reach the target of the money I needed in the house I should wait for more money, it is less but I have it.

**I: Is there anything else?**

P: There is none

**I: There was a time that you said someone would want to engage in sex without using condoms. And you get a lot of money is it something you would want to do when she wants to reach the set target?**

PF08: when engaging in sex without condoms you get a lot of money, and it is something you are not sure of getting but it is just by luck.it normally comes once in a while.

P: That is why I have talked of shots time

**I: It means that when you engage in sex without condoms is also in the shots time?**

P: In the shots time you will expect the money you wanted but for the long one it will force you to want more with condom. But if I wanted to reach a target of 5000/= if somebody wants to engage in sex with me for 500/= it will force me to go for me to reach my target.

P: She will engage in shots sex because there are many people who want shots, than flesh to flesh.

**I: And this flesh to flesh what risks does it come with?**

P: Sickness.

**I: Which kind of disease?**

P: Like STI.

P: HIV.

**I: Number 8 is talking**

PF08: Like STI, HIV only those.

P: Pregnancy, unwanted pregnancy such like things. Us we don’t want children

**I: Is there anyone who wants to talk about this?**

P: They are over

**I: at times the target you want to meet because you have debts, or you have emergencies, isn’t it?**

R: Yes

**I: At times you want to meet this target and you have debt and you have emergency just think if you had something /somewhere you were saving this money you can go and withdraw and then you pay, will it be the same? You can go for that shots and I see you are doing this there is no body want to talk about it number 8**

PF08: it will force you to be proud because you have a backup, you will be proud because of the little money you have

PF01: you won’t have to be with the people who wants shots, you can wait for the people who you engage in sex with without condoms those who have a lot of money, and you won’t go for less money. You know you have a good backup, the savings are the backup.

**I: Thank you we are about to finish but I would like for the sex workers ,you had talked about this ,you said that if you see somebody has this thing you also want ,in normal circumstances are they living beyond their means?**

R: Yes

**I: Number 8**

PF08: It is true they are living beyond their means because at times you can come from the field with a lot of money and when you go shopping you pick an expensive item and keep and tomorrow you get 100/= we are living a life that is expensive with less income.

**I: Now what makes you to buy an expensive item?**

PF01: you have just gone to the supermarket and you have seen it to be good to you and you didn’t plan and you have seen someone has it (murmur in the background)

**I: We understand we have taken a lot of time, let us not repeat what has been said, you have said that someone can live life beyond their means?**

P: Yes

**I: For one to live a life according the money she gets what is she supposed to do? For you to balance your life what are you supposed to do? Do want to talk about it number 5? Number 4**

PF04: As number 4, I feel I just save, when I save and sees my life is below and if I save that is when my life will be above

**I: Thank you is there anyone who has something?**

P: There is none.

**I: Okay do sex workers beg for money? Do they borrow?**

P: Begging for money that is no

**I: Not asking for money but from the Chama, bank to borrow?**

P: We would like even if you start now as jitegemee.

PF07: We like so much

**I: where do you borrow from?**

P: Loans, Chama

**I: Now we have started talking all of us you have not said who is talking and this thing (the recorder) can’t know who spoke.**

PF07: number 7

**I: Number 7**

PF07: From the bank,

P: Soft loan

PF01: From SACCO and Chama.

**I: How much do you borrow and what is it for? Number 7**

PF07: I can borrow 80 or 100

**I: And what is it for?**

PF07: Pay the person taking care of my children, business meaning if I don’t get from sex work we will use this

**I: And how do pay back the money?**

PF07: Number 7 the profit I get from business I include the sex work money.

**I: Number 1 tell us where you usually borrow from, what is it for and how do you pay back?**

PF01: I borrow from Chama, it can be 20,000/= or 30,000/= ,I can pay for my children school fees ,pay rent and I pay back with sex work because I know if I go at night I will get 1000/= or 3000/= and take back there.(Chama)

**I: We said that all of us are doing sex work and there is no other source of income?**

R: Yes (respond in unison)

**I: And I heard you talk about business or number 7**

PF07: As number 7 I have talked about business because you have asked me if I borrow and I said that borrow money to pay the one taking care of my children meaning if I don’t get I will use that.

**I: So it is something you haven’t borrowed you thin k of what you can do?**

PF07: Yes

**I: I would like to know what you do to increase your income because it is sex work you engage in. what do you do to increase your income?**

PF03: As number 3 it is only sex work.

PF07: There is no other job.

**I: If you want to increase your income what do you do? Number 1**

PF01: We steal from the men in the bars.

**I: You steal, number 8.**

PF08: as number 8 your good work will make you to increase the amount, if you have appetizer he will add you money.

**I: Good work means?**

PF08: You have gone there and you have done it to your best, I mean you are come on top and he didn’t know you would do that, and he is satisfied ,that satisfaction will force him to add more money ,

PF07: We have something called odeng’o (tablet used to spike the drink) you put it in the drinks and after I have put it in his drink he will be too drank, we go to the room and if I get 100000/= or 50000/= I will take it all and go to another bar now that odeng’o is 500/= one tablet and there is also for 300/= and you can’t miss to have it.

PF07: number 7

**I: Is what you wanted to say?**

PF07: Yes

**I: Okay, if you go to the field and miss clients, how will you know that it has reached a point that you won’t get a client today? And what do you do? Number 3.**

PF03: The signs of rain are the clouds if you see that it wants to rain you start planning earlier.

**I: Now what are the signs here now?**

PF03: The signs of clients/customers when no one whistles to you, you will know there are no customers. Now you are alert and go

**I: Number 6.**

PF06: You will know by the time you go and get customer is coming for you.at times when you go and it takes time for the customers to come, you will know there are no customers.

**I: It takes time.**

P: Yes and there is no one who has called you.

PF07: As number 7 am mostly used to the accountants if I get a person who has money and buys me 4 or 5 beer I will talk with the accountant and take back 2 bottles of beer even if it was 400/= I tell him to take 100/= and he gives me 300/= so the day I will miss customers he knows that I usually give him and I will ask him to give me 500/= and I know how I will do and I go to my house.

**I: That is the time the clients are not available?**

PF07: Yes i: number 1

PF01: I am confused.

**I: Okay number 4 you wanted to … number 5 I have asked her but seemingly she is ….**

P: Even if she is given 50/= she accepts (NAO: participant chuckles)

**I: We said that we usually borrow how much we do borrow on normal situations**

P: I had already said 1000/=

**I: You said 100 and the other person said 30 and this person has not said.**

P: 20

**I: 20000/=**

P: Yes

**I: Okay, have we ever thought of retiring, quit or leave sex work?**

PF01: Number 1.

**I: You have thought of leaving this work? What are your reasons for saying that?**

PF01: It has paid me dearly and it has sustained me for long

**I: You are not thinking of quitting?**

PF02: The private part doesn’t get old (NAO: participants chuckles) and there is no day I am thinking of retiring.

PF01: There are even older people, we are not thinking of retiring, unless we find someone who will support us or remove us from sex work or find a pastor who will pray for you and you get saved and quit sex work.

PF04: we have sat for a long time

**I: We are almost finishing.**

PF04: I can’t leave parking( sex work)only death will make me leave.-dhako tedo nyaka tii [meaning a woman can be married at an age ] I will engage in sex till I die.

**I: There are those out there who wants to quit isn’t it.**

R: Yes

**I: Do you know them, amongst you there are your friends who want to leave what are their reasons for quitting?**

PF08: If she goes there and finds someone who has a big penis like the one for a donkey (participant chuckles) when she leaves that place she says that no am not going back.

PF01: They are those who started long time ago and they are now tired and us the young ones we tell them you old mamas go and rest and leave for us sex work, it is them who are tired.

PF04: The things that makes someone to leave parking mostly it is the men, there are some who approach you and they have bad intentions like it happened in =carwash estate = and he kills you, and someone says that in this job someone can kill me it is better I quit.

**I: What happened in car wash?**

P: A girl was killed and put in a bag, she was cut into pieces and put in a bag

P: You didn’t hear that?

**I: The one that happened there ooh I thought there was another incident that is why I wanted to know.**

P: There is another incident that happened recently, kona Legio, Joventure, her food was poisoned, and also another one was strangled who was buried the other side Nyaseme

**I: Now those are the things that make someone to quit.**

P: Yes (participants responding in chorus)

**I: Those who wants to quit or have quitted do they quit when they have made the decision?**

P: Some are leaving and they come back.

**I: Some are leaving and coming back?**

P: Yes (participants responding in chorus)

**I: When they come back what are their reasons for coming back?**

P: They leave and then finds that life is unbearable and it forces them to come back.

**I: And are there those who went and never came back.**

P: Yes (participants responding in chorus) it is enough

**I: what are their reasons for quitting?**

P: it seems they have gotten another source of income that is paying off.

**I: We had said that age is not a reason for retiring isn’t it?**

P: Yes.

**I: But you have said number 1 you said that there are some who are old and you tell them they are now old they leave.**

P: Yes they leave for others there are too many people.

PF03: what makes us to tell them like that them they are more experience than us and they can seduce your client even if you had gotten one, that is what make them get abused that she is too old let her go home it better you leave the field for us.

**I: Those who left and never came back.**

PF04: As number 4, the private part has not retirement

PF07: what make others to quit like me if am soft and I don’t have many friends. I have my partner (boyfriend) we are drinking and I tell my friend to take me to the toilet so my friend who was nearby takes my boyfriend and the other people whom she was with, they lock me inside the toilet the door and I stay there till morning and I have not gotten anything .it will make me feel disgusted and leave sex work and say am not coming back.

**I: It is over, those who have gone for good we are about to finish, am sorry, for those who have left for good how their life is?**

P: Those who…

**I: Those who have left sex work for good number 8**

PF08: As number 8, you find that someone has savings which is she leaves that place she starts a business. And she now runs the business and she can’t leave the business and she has decided to leave for good.

**I: Is there anyone who has anything about those who went and came back?**

P: There is none.

**I: For those who have come back, you said they come back what are the disadvantages of them coming back?**

PF07: You find that someone leaves and now wants to stay with one man and in the field she was even serving three people and she is not satisfied sexually it will force her to leave and come back, during that time she wasn’t married she was getting much and there she has only one man who doesn’t satisfies her

**I: She comes back.**

R: She is used to 7 or 8 men.

**I: What bad thing that happens to her when she comes back? May be you had forgotten about her and then all over sudden she comes back as an interviewer I go back to the field.**

P: It will force her to register. She still has no customers.

**I: Number 1 have said she has no customers.**

PF03: as number 3 it will force us to and lecture her for a while. Like since she has nothing we will give her even 100/= to start with.

**I: Is there any good thing that happens to her? You welcome her**

P: We have to abuse her.

P: That is obvious.

**I: You have said you don’t want to quit sex work isn’t it?**

P: Yes

**I: And for those who want to quit what is it that they are saying if they accomplish they will leave? Let number 10 talk then number 7**

PF010: As number 10 what they say they will leave is when they get find a man who will take care of her well she will leave.

**I: (interviewer chuckles)**

P: Is it over?

**I: Say another thing.**

PF07: I wanted to say am number 7, what makes them to leave is when they get a rich man, you know we are different people if you get someone who is smartly dressed and she is disciplined and if you arrive here you won’t know she is a sex worker, and she is fairing on well and finds a widower that feels he want to come to the field and get only one sex worker and if she accepts to be with me then I will stay with her. And they lively happily together. I have seen a tinted vehicle outside and then say this man wants me and he has money and tells me that the wife passed away I will go and leave sex work.

P: He has accepted you with all your responsibilities and he is ready to fend for you

**I: if you get someone who accepts you and will cater for everything**

P: You go

**I: And leave, is there anything?**

P: There is none

**I: Those who want to leave say that if I accomplish this I will leave?**

P: Some say if they reach their savings target they will leave.

**I: They reach their target.**

PF07: As number 7 if I get a rich man who sold a piece of land and he has 800000/= I will steal the all amount then go and invest and leave sex work.

P: You go to another county

P: You buy a piece of land

P: Or you start a business

P: Or build rental houses

P: then I go to church and get saved. And I now start putting on dera (long dress) and having head scarfs

P: you join choir

P: He will find when different, join SDA choir am

**I: The last question, do you know any sex worker who have left sex work from 5-10 years?**

P: Yes

**I: Is there any?**

P: Yes, she left because she got married, and he took her in with her five children built for her rental houses and they are staying. She leaves sex work completely.

I: It is a story that she went and she was built a house. What made it easy for her to leave?

P: What made it easy was, that person took her in with her five children that made it easy.

**I: Are there any challenges that she faced after leaving?**

P: No.

P: It is just that her private part is tired. (Participant chuckles)

**I: Is there anyone who left and things are not good?**

P: No, it is over now.

**I: I want to appreciate you all thank you so much, you have been a good group even if you were tired you still had some points to say, I am very grateful I know we have taken your time I want to be sorry unless there is a question,**

PF03: I have a message that I want you to pass to the office, as number 3 I hope the recorder will take my message as I say it, telling them that the sitting allowance was very little, as a sex worker that is amount that I can make within seconds, they can’t make us to be seated from 10; 00am up to it is almost 2pm because of 500/= surely tell them we are not happy. Let them make amend.

**I: Okay,**

R: And another thing like now I came from =Chiga= and the transport that I was supposed to use, I have eaten lunch and now want to walk.

**I: We are providing transport. Thank you [participant clapping] the interview has ended at 1:48pm.**

**END OF INTERVIEW**
