# Supplementary material for: Eat a little and save a little: A qualitative exploration of acceptability of a potential savings intervention to reduce HIV risk among female sex workers in Western Kenya
Source: PLoS One. 2024 Dec 19;19(12):e0310540. doi: 10.1371/journal.pone.0310540 (PMC11658496; doi:10.1371/journal.pone.0310540)
Supplement: S1 File — (ZIP) [file pone.0310540.s001.zip › Jitegemee Transcripts and Dissemination Notes for Journal/FGD G.docx]

**FGD ID: FGD G**

**INTERVIEWER: JUDITH**

**NOTE TAKER: NANCY**

**VENUE OF THE INTERVIEW: NYAMWARE**

**INTERVIEW DATE: NOT MENTIONED**

**TRANSCRIBER NAME: ALICE AKINYI**

**INTERVIEW LANGUAGE: DHOLUO**

**STARTING TIME: 4:20 PM**

**END TIME: 6:40 PM**

**TIME TAKEN: 2:20:22**

**CATEGORY: BELOW 30 YEARS, RURAL.**

**I: Okay. This is FGD G. The interviewer is [name omitted] and the note taker is [name omitted]. The interview… the discussion is being conducted at =Nyamware=. Starting time is 4:20pm. Okay. Following what I had already explained to you about Jitegemee, what comes to your mind first? (Birds chirping and motorcycle riding in the background) …what you think about Jitegemee the way we had said it before? ... (Birds chirping at the background) mmh! No.6?**

PG06: Me as number 6, my thinking as you had clarified to us about Jitegemee, it is how you can depend on yourself. You leave the issue of exchanging with money (the participant seems to be talking about exchange of sex for money) so you look for a way of living by yourself with your own money, that is how I know it.

**I: mmh! Another thought on Jitegemee as we had clarified? Number 1**

PG01: Ok I as number 1, what I understand about Jitegemee is that depending on yourself is how you can save by yourself so as to help yourself (people talking at the background, inaudible information at minute 1:48-1:49) and how you can help your household (family).

**I: Ok, mh! Another thought about Jitegemee? (birds chipping) number 3?**

PG03: Yes, I as number 3, my thinking, self-dependence is that whatever you get however little, you look for a way to save a little and you use a little. (people talking at the background) so you stop exchanging money for sex.

**I: Mh! (quiet laugh) number 8 another thought?**

PG08: I as number 8, what I understand about Jitegemee, is that you look for a way to save the money that you get so that even when a day comes you can use the one that you have saved even without going where you should go. Exchanging sex for money.

PG07: I as number 7, what I know about Jitegemee is how we can save the money that we get from our work, that we can save bit by bit so that when you don’t go to work, you can find a way to help yourself and the children and how you can also save a little. That is what I know about this.

**I Mh! Another thought? number 2**

PG02 Ok me as number 2, what I understand about Jitegemee is that it is… it is about saving. How you can get money, how you can save and how you can take care of yourself for the time being.

**I Mh! Number 4 (birds chipping in the background).**

PG04 Me as number 4, what I have heard about Jitegemee, is that you depend on yourself not that you depend on another person’s money. So that when you get a little you can save even if it is one hundred shillings you use fifty shillings and save fifty shillings, not that you depend on someone else’s money.

**I Mh! Number 5 (motorcycle riding at the background)**

PG05 I as number 5, what I know about Jitegemee, is that you can miss going to work let’s say you are sick, so the little that you had saved can help you somewhere even paying for school fees (birds chipping at the background).

**I Ok thank you. So for the money that women who do sex work get, what do they frequently buy every day? What and what can girls buy every day? Yes, number 6.**

PG06 Pads (sanitary towels)

**I Pads, you buy that every day?**

PG06 Everyday?

**I Yes.**

PG06 Food

**I Food, another thing?**

PG06 Rent

**I Aha, everyday?**

PG06 You can save for rent every day.

**I Ooh, you save every day. Ehe, something else that we can buy every day?**

PG01 Clothes

**I That’s number 1, she buys clothes daily?**

PG01 Yes, she can keep money to buy clothes daily

**I Oh, you mean saving?**

PG01 Yes

**I Now I am asking what you can buy every day without having saved money.**

PG01 Food

**I Food. Yes, number 2 another thing?**

PG02 Paying school fees for the child.

**I You pay daily or you save daily?**

PG02 You save and then when it is enough you pay for them.

**I Aha, I want something that you buy on a daily basis. Number 7, apart from food what else do ladies buy daily?**

PG03 Chips (laughter in the background)

**I Number 3 has said chips, ehe, something else?**

PG06 Alcohol

**I You buy alcohol every day?**

Chorus response Mmm

**I Mmh, that’s number 6. Something else? yes no.8**

PG08 Credit cards.

**I Credit cards every day; at how much do you buy?**

PG08 For ten shillings or twenty shillings

**I so how much do you buy most of the time?**

PG08 I usually buy for fifty shillings

**I You usually buy for fifty shillings every day?**

PG08 Yes

**I Okay. Someone else, what do you buy on a daily basis? Number 7, what do you buy on a daily basis?**

PG07 Food

**I only food?**

PG07 Things that the children go with to school.

**I Ok, number 5.**

PG05 I as number 5, what I buy daily is food

**I Food mh! Only?**

PG05 Is what I buy every day.

**I Okay, and in every week, what can girls buy without saving money, but a week cannot pass by without buying those things. Yes, number 4.**

PG04 In every week for girls especially those without children, they can buy clothes, she can set it that every week that she gets money, she can buy clothes. But for those with children, in a week she can say ah, in a day she can get three hundred shillings, she can save in a week and even if she has a child in nursery school who goes to school every day, she can pay for her motorbike transport to school or she can buy (coughing) something and put it in the house.

**I Ok uh! Number 8 what can u buy each and every week (soft laugh).**

PG08 Just clothes

**I Only clothes? Uh! Number 1 every week?**

PG01 Clothes

**I Clothes uh! Number 3?**

PG03 Just clothes

**I Uh! Number 6**

PG06 For me each and every week I can buy household stuff

**I Household staff mmh, number 4 has already said, I think number 5 has also said hers right?**

PG05 Not yet

**I In every week how much money do you use?**

PG05 In each and every week I can buy clothes for my child and me

**I Clothes eh! Number 2**

PG02 In every week I buy clothes, shoes and panties (laughter)

**I Okay. So for those who buy clothes every week, how much can you use in a week? like number 1 how much can you use in a week if you buy clothes?**

PG01 I can use more than five hundred shillings

**I Five hundred, ehe, number 2? panties (laughter) and clothes**

PG02 If I add all of them in total I can use *fifteen (*one thousand five hundred shillings)

**I *fifteen (*one thousand five hundred shillings) in each and every week?**

PG02 Yes

**I Mmh, number 3?**

PG03 One thousand shillings

**I One thousand every week eh! Number 4?**

PG04 I have a child, so I balance and I can use two thousand five hundred shillings

**I Twenty-five hundred every week?**

PG04 Yes

**I Okay. Ehe, number 5?**

PG05 I as number 5, in a week I use five hundred shillings.

**I Five hundred shillings ehe, Number 6**

PG06 I as number 6, I can use one thousand shillings

**I One thousand, that one is inclusive of food?**

PG06 Yes

**I Aha, number 7 in a week how much do you use? We can just guess**

PG07 In a week I can use fifteen (one thousand five hundred shillings) together with the child.

**I Ehe, number 8**

PG08 I can use more than one thousand, one thousand two hundred or one thousand five hundred shillings

**I One thousand five hundred, ok that’s fine. What can they buy in a month?**

(Respondents voice, at the end of the month or?). **Yes, at the end of the month provided it’s the end of the month. Yes, number 1**

PG01 Paying school fees

**I How much do you pay for school fees? Roughly. (Birds chipping)**

PG01 Roughly ten thousand shillings

**I Ten thousand ehe, something else?**

PG01 Pads (sanitary towels) (Birds chipping)

**I Pads, roughly? (Laughter) we are just placing it on an average**

PG01 One hundred (one hundred shillings)

**I One hundred, yes, something else?**

PG01 Only that.

**I Only that?**

PG01 and paying rent

**I and paying rent. Roughly how much do you pay for rent?**

PG01 Twenty-five (two thousand five hundred shillings)

**I Ok number 6**

PG06 I as number 6, per month I can pay rent three thousand shillings, food, school fees three thousand shillings per month.

**I Those are the things you do in a month? Ehe, someone else? I saw a hand somewhere here. yes, number 7**

PG07 I as number 7, when it reaches end month, I make sure that I’ve paid school fee for the child and for the house (house rent).

**I How much do you pay for school fees?**

PG07 I pay seven hundred shillings for school fees and the house I also pay seven hundred shillings. so in a month I think I can use around two thousand shillings inclusive of food.

**I Ok someone else who would like to tell us? (Slight laugh) we are estimating average. In every month number 5?**

PG05 I as number 5, in every month I use a lot of money. For school fees, I have two people, seven hundred, seven hundred, (she pays seven hundred shilling for each person) plus food and the house am staying in, (people talking at the background) of which we can say I use something like three *thao* (three thousand shillings)

**I in every month?**

PG05 Yes

**I Ok, alright. Number 2, how much can it reach?**

PG02 I as number 2, every month, if I pay school fees for the child I use two thousand shillings and then food together with rent can cost something like one thousand shillings, so in total I can use three thousand shillings

**I Mmh, number 3**

PG03 I as number 3, looking at it, rent, I use two thousand shillings, then school fees around one thousand two hundred shillings, only.

**I That is for every month?**

PG03 Yes

**I What of the things that someone can buy in a year? (Laughter) that by the end of the year, you do not fail to buy that thing. yes, number 6**

PG06 I as number 6, at the end of the year at least I have to buy something nice in the house.

**I Ehe, like what?**

PG06 Woofer, (laughter) chairs

**I So roughly how much can you use?**

PG06 In a year?

**I Yes**

PG06 I can use like five thousand shillings if I have saved well.

**I Mmh, someone else? number 2**

PG02 I as number 2, in a year if I work and if I have saved for that year, I can take that money and use it to buy a [*nyachecha]* goat then put it there (in the compound). In case I have a problem then I can use it to solve the problem.

**I You’ve said *nya?***

PG02 how else do you call it in Luo?

R Goat … [Cross talk] … goat

**I Ooh, goat. Ok, I didn’t hear it clearly. Yes, number 7?**

PG07 For me in a year, in a year I can use approximately four thousand shillings, because I buy for the child Christmas things, chair, in every year I normally buy 2 plastic chairs every year even those ones I usually buy. so every year I use four thousand shillings and something (she means that she uses four thousand shillings and more)

**I Four thousand and above?**

PG07 Yes

**I Yes, number 2 you said that you can buy a goat for emergency purpose, so if you buy the goat, how much will it cost?**

PG02 If I buy a goat, like last year I used three thousand six hundred shillings and I bought one

**I If you sell it, how much would you sell it for?**

PG02 If I sell it, (slight laugh) if the sale is good and I know it is good, I can sell it for five thousand five hundred shillings

**I Ok yes, number 8**

PG08 I as number 8, I can use more than three thousand shillings. If I add some, paying school fees for the child, buying clothes for the child and things like fabrics.

**I Meaning more than three thousand on clothes and fabrics. Yes, someone else? yes number 5**

PG05 I as number 5, what I love to buy that is a must to buy every year, I love to buy wallets, small house hold staff like utensils

**I Eh! Ok how much money can you use?**

PG05 I can use *two thao (*two thousand shillings)

**I Yes, number 4, the year does not end without you doing what?**

PG04 A year?

**I Yes**

PG04 I must pay for school fees, so in this year I can buy clothes for the child, then make my house by buying some small staff like a chair, woofer, you can buy chicken to treat yourself (laughter) so, I can use four thousand shillings.

**I You buy chicken and something to escort it with (coughing) right? (laughter). Okay. Where do most women who do sex work get the money that they use from? What is the source?**

R Repeat that question?

R I don’t understand

**I Where do most women who do sex work get the money they use from? Yes, number 4**

PG04 I as number 4, I can say that the money they get mostly comes from the man that she engages in sex with.

**I That’s a client or the man she has met with where?**

PG04 A man who… Let’s say girls like… who is a sex worker, you will not go and say you are going to your boyfriend, you can go to that place and you just meet with someone you don’t know, you agree on the price and go together. So, let me say mostly they get money form a person that they have just met.

**I Ok. Somewhere else where they get money from? yes number 6**

PG06 I as number 6, I can go to someone for example like the beach, I meet with someone who is from the lake, for me money is all that I want (laughter) I give him something and he gives me money.

**I Ehe, somewhere else that they get money from? (Short silence) yes number 8**

PG08 I as number 8, some people get money in places like bars, there are some who work in the bars and get money.

**I So, does she get money from people or from the work she does in the bar?**

PG08 From people she finds at the bar

**I Oh, Like a client?**

PG08 Yes

**I Number… who do I call (laughter) number 1 where do they get money from? (Music playing in the background)**

PG01 Most of the time they get money from places they work in, especially where they… a large number of them get money from parking [to mean sex work] where they work

**I Many people have said that they get money from parking, are there any different ways apart from parking or getting clients or finding someone to have sex with and they give you money? Apart from sex work, are there other places that they can get money? Yes, number 5**

PG05 I as number 5, let’s say if I don’t have money, I can go to the bar and work then I can get money which I can use to feed my children and me.

**I other places where they can get money? Number 8**

PG08 I as number 8, other places you can get money in this place is along the lake if you go and scale the fish. You can get a little to support yourself with.

**I And if you scale the fish how much money can you make?**

PG08 If you scale you can get even one hundred shillings.

**I per fish?**

PG08 Basin

**I What is the amount?**

PG08 basin

**I A full basin?**

R Yes

**I A small or big basin?**

**Chorus response: A big one**

**I A big one that can carry five liters of water?**

PG08 The ones that are being sold two hundred and fifty shillings

**I Ok**

PG08 It depends on how you clean the fish, if you clean 3 full basins you can get three hundred shillings

**I Oh! Basin? If you clean fish in 3 full basins?**

PG08 Yes

**I Meaning each basin is one hundred shillings?**

R Yes

**I Ok. Other places where they make money aside from sex work? Number 2 (laughter)**

PG02 I as number 2, what I can say is that many people go to the lakeside. I can go out as if am going to the lakeside, but am going to work for someone in a hotel and when am done the owner of the hotel gives me money which I will use for my needs.

**I If you assist in the hotel work, how much are you given on average?**

PG02 There are some who give you one hundred shillings, there are some who give you one hundred and fifty shillings

**I Ok! The whole day?**

PG02 Yes

**I Ok ehe, other places where they get money? Number 3**

PG03 I as number 3, in my opinion I see that mostly shops, you can work for someone in a shop and they give you money.

**I How much can they give you?**

PG03 Three hundred shillings in a day.

**I Oh! If you sell on her behalf?**

PG03 Yes

**I So, whether you sell well or do not sell well, provided you are in the shop?**

PG03 Yes, what you had already agreed on.

**I provided you stand in for her?**

PG03 Yes

**I Ok, other places where they can get money? Mmh, Number 7**

PG07 Other places where they can get money, they can find someone… I see mostly someone can come and she goes for other work and she can leave you their baby and she tells you to look after the child, you will talk later in the evening. Like teachers who go to school. If she leaves you the child to look after, she knows you can look after until evening then she can give you two hundred and fifty shillings. That is also a way of getting money.

**I Oh! If you babysit their children?**

PG07 Yes

**I Ok. There is somewhere we said that many women who do sex work like to buy clothes, right? Shoes. Why do they use money to buy these things? Number 5**

PG05 You know like us girls, you have to dress well. Even if you find a man and you go with him to the house, you know you can’t go looking unkempt, I first have to make myself up, (laughter) is when you can get his money.

**I So that you look attractive to your client?**

PG05 Yes

**I Ehe, another reason as to why they buy the things that they buy? (short pause) Let me come back to you number 5, if you buy clothes or things that make you look good how much can you use?**

PG05 I can use around two thousand shillings (coughing)

**I Two thousand, that is in a month?**

PG05 Yes

**I That you use to look good for work?**

PG05 Yes

**I Someone else? Yes, number 3**

PG03 I as number 3, the reason as to why girls buy clothes or shoes is so that they look attractive so as to be seen by a client.

**I Ok something else they can use their money on other than clothes? Yes, number 7**

PG07 I as number 7, what I can use money on aside from clothes, I have to buy body oil which I can use on my body, that you can apply on the face (laughter)

**I Which ones are those?**

PG07 Facial makeup that you use to look good, you buy nail polish, things like that

**I if you buy makeup, how much money can you use?**

PG07 I can use approximately four hundred or four hundred and fifty shillings

**I In a month?**

PG07 Yes

**I Ok, number 2**

PG02 I as number 2, what I can buy with money is first of all I can buy food, because I have to eat very well so that if I go there, I have energy (laughter)

**I So, what are these good foods?**

PG02 *Ugali,* I have to eat *ugali* properly.

**I Ugali with what?**

PG02 *Ugali* with beef or chicken then after I finish I also take some soda, even if I go there I have enough energy.

**I You do that every day or monthly?**

PG02 I can do it… I can do it… if I can I can do it every day.

**I How much can you use to treat yourself that way?**

PG02 Everyday? Right now flour is expensive. So, I can use… if every day I can use three hundred shillings or three fifty shillings.

**I For treating yourself? (Making yourself feel good)**

PG02 Yes

**I Ok. yes, number 6**

PG06 I as number 6, I use money to plait my hair, buy lotion, makeup, food. Yes, those are what I see that per month I have to buy.

**I When you buy, how much money do you use for all those things?**

PG06 All those things they cost something like three thousand shillings.

**I Three thousand, mmh, Ok. Who has not told us what they buy, number 4, (laughter) what do they buy? We are just talking generally.**

PG04 What they buy, for me I plait hair and after making my hair I’ll go buy makeup. you can’t apply that makeup if you haven’t dressed well. You first have to buy clothes. So, because plaiting I can use around one thousand, two hundred shillings, so in a month I can use four thousand shillings.

**I Ok. On your body?**

PG04 On my body because I make my hair, I buy makeup, I have to change clothes every day so I have to spend.

**I Ok, alright. Thank you. So, moving on to the next question, do those women who do sex work save? Do they save money? Yes, number 7**

PG07 I as number 7, savings, we have to save because you don’t know how tomorrow will be. There are times the field [to mean where they meet clients] may be bad. So sometimes you have gotten three hundred or someone has given you around five hundred, you make sure that in your Mpesa you put even two hundred shillings, that can help you during emergencies or for tomorrow. That is my thinking.

**I So, if you save, why are you saving? What are the reasons for saving?**

PG07 The reason as to why I save is because I have children and I might miss going to work but they will find a way of helping themselves because of the saving I was doing.

**I and if you are saving, how much can you save? How many times can you save in a week or in a month? If you are saving.**

PG07 in a week, if I say in a week… if work goes well for me who works in a bar, if I work well in a week, I can even save three hundred shillings. By the end of the week then I have something good.

**I How many times can you save in a week? We have seven days in a week**

PG07 Those days I make sure that when I leave with some money, I have to save something small each and every day.

**I So, you do not have specific days, or is it every day?**

PG07 I save even if it is fifty shillings or one hundred shillings, but I make sure that I save daily.

**I Ok. Ehe, someone else? Yes, number 6**

PG06 I as number 6, I usually save. If I leave, every time I get a customer I save a little. So, it depends with how we had agreed and what he has given me.

**I why do you save?**

PG06 I save because per month I have to pay rent, school fees per month. so that is why I save so that my child can go to school, and pay the owner of the house.

**I Ok, and if you are save, how much can you save? You have said how much can you save when you are saving?**

PG06 Ok. if I get even one thousand shillings, I can save three hundred shillings

**I ok. Someone else? (short pause) even if you do not save, you can think of why other ladies save money, what is the reason as to why they save? Mmh, number 8 (laughter)**

PG08 Some ladies who save can do it so that if tomorrow… or when the next day she goes and the fied is bad that she gets little money, so if she adds the other that she has saved she can be able to help herself.

**I Do you know any of them who usually saves?**

PG08 Yes, I have a friend who saves. I sometimes also save little by little.

**I if you save, how much can you save?**

PG08 Sometimes it gets to eight hundred or five hundred shillings

**I That one you save at once?**

PG08 I save little by little even one hundred shillings

**I every week or in a month?**

PG08 It’s not known at times I don’t get sometimes when I get I save.

**I oh when you get?**

PG08 Yes

**I Aha, number 5**

PG05 As number 5, I have to save because I have a child who needs to eat, what she will wear, how she will bathe. The child can also fall sick. You cannot tell when diseases will come. The little money I have saved I can use it to take the child to the hospital if the child falls ill.

**I When you save, how much can you save? Each time you save, how much can you put into your savings?**

PG05 The little I can save is even one hundred shillings, it depends on how I get it. The least I put is one hundred.

**I Yes, number 1**

PG01 I as number 1, I save so that I can help myself with the money that I have been saving because I have children and they have to dress, they should eat they should bathe.

**I So, when you help yourself, in what way do you do so? What do you do with the money you save?**

PG01 I pay rent, I pay school fees, I buy food for the children and also use it for my needs.

**I Ok, when you save, how much can you save? When you get some money how much can you set aside?**

PG01 sometimes when I get it, if it is good then… if I get when work is good I can even save five hundred shillings

**I once?**

PG01 Yes, if it is bad, I can even save fifty shillings.

**I Ok that’s fine. These women who save money what is their behavior like? (Laughter), these women who save money when you look at them can you tell that this person saves? If you look at someone?**

R No

**I You cannot tell?**

Chorus response: Mmm

**I For those who save money, what is their behavior like? Number 3**

PG03 Yes, as number 3, those who save, even you want to do something, I mean you want to eat something, at that time you balance, if you wanted to buy it for fifty shillings, at that time you buy for twenty shillings, so that you can save the other thirty shillings.

**I Ok, so they use a little money?**

PG03 little by little

**I Ok, and if they buy what do they buy for twenty shillings?**

PG03 let me say you wanted to buy food, you can buy food for one hundred shillings right? You usually buy for one hundred shillings, so in order for you to save you will have to buy for fifty shillings then remain with fifty shillings

**I Another behavior number 4? that those who save have?**

PG04 You can save in many ways.

**I like?**

PG04 You can save in *chamas (*saving groups) or you can save money by yourself. For those who save, it depends with how they save, because you can find that maybe she has gone to work, and the field was good. You find someone that you negotiated with and agreed and you get even two thousand shillings. Like me I can save five hundred shillings, the rest I use to help myself.

**I Ok someone else? How do those people who save look like? Number 2**

PG02 Those who save look like… let me say like me, I go out to work and after going to work I agree with that person, he gives me something like one thousand shillings. When I get back I take a little and put it aside in my Mpesa, I put a little in the chamaa, the rest I use it to buy food.

**I These ones we are talking about those who save, right?**

PG02 Yes

**I And for those who don’t save, what is their behavior like? Yes, number 6 (laughter)**

PG06 I as number 6, they do not have value for money, so they just walk around, they eat, they branch here, they buy this, they see nice things, they don’t know what to do with money.

**I so they just pick anything they see?**

PG06 Yes

**I Ehe, another characteristic? Number 7**

PG07 As number 7, the behavior which those who don’t save have, is that when they get money, they misuse this money. She can even go to drink alcohol with the money and that’s what she can use her money for. But me who saves, I know that I should use fifty and keep fifty. So their behavior is that they don’t save… they misuse money.

**I so apart from alcohol what else do they…**

PG07 … they like eating chips (French fries), bananas and some other small things, that they end up not having any money that they are left with.

**I ehe, number 6**

PG06 I as number 6, someone who doesn’t save has a lot of misfortunes. (Laughter) because sometimes this person falls ill she was not saving; how will she help herself? That’s when you find out that this person was not doing what, was not saving. Things happen abruptly, those unplanned things that happen can happen to her and money is needed and sometimes she has none.

**I Yes number 4**

PG04 someone who doesn’t save has problems, the reason as to why the person has problems is because when she gets money today she uses all of it. When tomorrow comes she starts begging.

**I That’s the behavior that most of them have?**

PG04 Yes

**I Do they beg all the time or how often do they do that?**

PG04 It forces her to beg all the time because she doesn’t have. She made some yesterday but she used all of it.

**I Ok yes number 5**

PG05 I as number 5, those who do not save have problems. Sometimes you find that she makes good money and she wants to spend it all in one day, and she has children. She uses all the money without knowing what tomorrow she will eat.

**I she uses all the money she has, yes, number 8**

PG08 I as number 8, someone who doesn’t save is someone who has no development because you find that maybe someone has money, and she uses that money in the wrong way, so there is nothing good that she can buy and say that I own this thing, such and such.

**I Ok there’s nothing to show that she has been working,**

PG08 Yes

**I Ok, yes**

PG06 I as number 6. Someone who doesn’t save can be detected by the sweet things she eats. When she gets money she thinks of buying meat, she wants to buy chicken and chips. She has no time for saving money because she knows that she will get the following day. Sometimes tomorrow she is unable to make money, she has failed, right? Yes.

**I Another thought, how do those who don’t save look like? (short pause) (slight laugh) This corner is quiet. number 3 let’s hear your opinion**

PG03 Those who do not save are those who like eating small things. She finds this she eats; she gets this she eats. So you know she cannot have money. So she cannot save because she is someone with a sweet tooth.

**I so what are these little things?**

PG03 Chips, let me say sweets, if she sees sweets she eats. That is things that… I don’t know what to say.

PG06 can I assist her?

**I Assist her**

PG06 I number 6 would like to assist my fellow number 3, that is sweet things, if she sees this she eats, if she gets that she will eat. When she passes where bananas are sold she will pick, where mangoes are sold she will pick, she also eats chips so can she really save money? She can’t

**I Ok that’s fine. We had already said that there are different reasons why people save money, right? We already said if a child falls ill, paying school fees, paying rent. For those who save money so as to find a way to support themselves in future, what make it easy for them to save money? Yes no. 1**

PG01 what makes it easy for them to save money is, for them to save money they have to focus ahead. Either she opens up something in their phone or open up a bank account.

**I Something else that makes it easy for them to save money? number 2**

PG02 I as number 2 the reason as to why I will save is that when am faced with a certain problem in future, I can find a way to help myself. I can open an M-pesa account or I can place it in another *chama* so that when something comes up, I find a way to help myself.

**I Even if you save money, what encourages you to save? What motivates you to save? Number 1**

PG01 what motivates me is as I said earlier that I have children, is that I have to remember that I have to pay school fees for them, buy them clothes and buy them food (birds chipping)

**I Yes number 2**

PG02 I as number 2 the main reason as to why I can save money is that I have a child and we can never tell when the child might fall ill. Sometimes the child can fall ill at night or maybe the child can be sick in the morning. So the money that I had saved I have to use it, if the child becomes ill then I will take it and rush to the hospital as soon as possible.

**I mmh, number 6**

PG06 I as number 6 what motivates me to save is that I am aware I have to pay school fees and am not employed, so I have to use my brains and take bit by bit and do what- save it somewhere so that I can get a place to get money per month.

**I mmh, number 5**

PG05 I as number 5 what will make me save is I may have a problem at any time, any type of problem. Like I can be sick a nd the one that I have saved can help me when I am sick.

**I so when you think of falling ill that motivates you to save?**

PG05 There’s a lot that motivates me. I am a person who lives alone, maybe rent is due. If you have not saved can you pay, it is not possible to pay. So things like that, I also have a child, I have to buy clothes. So that is what will make me save little by little.

**I Ok. Yes, number 4**

PG04 what encourages me to save is just the child

**I what has the child done?**

PG04 There’s nothing the child has done, but you have to save when you have a child because you cannot know when a problem will arise, a problem can arise when… (music in background) [inaudible clause] so when you have saved, that is where you will have to go and get it from because you can say I don’t save because so and so is available she can help me with it. If you don’t save when you go to that person maybe she also doesn’t have. So you just have to save.

**I Ok. number 8**

PG08 as number 8, what can encourage me to save is the same as my partner number 4 has said on the side of the child. You have to save so that you can find a way to buy clothes for the child or you can even pay school fees.

**I Ok are there any difficulties that those who save can experience? Is there any difficulty, challenge? Yes, number 4**

PG04 You have to have a challenge, following someone who lives alone and is hustling. So the challenge can be maybe you have made money but it is little. Maybe there is nothing in the house. So there is no way that you can save. Maybe you have saved today and tomorrow you have not. That is the challenge.

**I Yes number 7**

PG07 Speaking as number 7, the difficulties that I may have in regards to saving money is that maybe I have gone to work and find that it is bad, so when I get back the house also has needs. So the little that I have made is going to force me not to save it because I am the bread winner in the house, so I cannot save it. So, I can look if tomorrow there is a chance… that is maybe I can get money tomorrow then I save. But at that time sometimes there is none and there is also nothing in the house, it will force me not to save it so I am just going to use it.

**I Yes number 3**

PG03 Yes, as number 3, the challenge that I may have is that maybe I want to buy something and I have come from work with let me say five hundred shillings, and with this five hundred what I wanted to buy is also worth five hundred shillings. So what will make it difficult for me is that I will not buy it because I have five hundred shillings and I also want to save part of it, and the remaining part I use to buy things in the house. So it will force me not to buy that thing. That is the challenge that I will have.

**I Number 6**

PG06 As number 6, the challenge that I may have is that at times I go to work and I don’t get. I come back to the one that I had saved in the house, I use it to buy food. That is a challenge right? Then the second thing, maybe my house was broken into and the money was stolen aren’t I left with nothing? (laughs)

**I Has that happened?**

PG06 Yes. So that is a challenge.

**I Ok, another thought? Another challenge that those who save may face? (Silence) are they over? For the challenges you’ve mentioned how can we solve them? Like number 6 has said her house can be broken into, or she has not made any and she has used the one that she has saved. What can we do as people who save or people who want to save, so that this does not happen? Yes, number 1.**

PG01 What I can do to prevent that is, for me to continue saving, is that I can save in the house… like I had said earlier I can open a bank account or I can save through phone or in the house. Like I had said I can save in the house, I can save some through mobile banking and some through bank.

**I Ok another way that we can use to help? number 2**

PG02 I as number 2, I just feel I can save through Mpesa. Because if I save it in the house and go to work I can find that house was broken into and all the money is stolen, there will be no way to help myself. So, I can just save through Mpesa.

**I Yes number 6**

PG06 Like me I save with KCB Mpesa

**I so that will help with that challenge?**

PG06 Yes

**I someone else with a thought?**

PG08 I would just like to support that point of Mpesa because Mpesa is good and easy to use.

**I yes number 8 supports Mpesa. Someone else?**

PG03 I as number 3, I can save through bank

**I In a bank so that that doesn’t happen?**

PG03 Yes

**I Ok, and if you make five hundred and you want to buy something worth five hundred?**

PG03 If I have five hundred shillings and want to buy something worth five hundred, I will have to not buy it because up to… I should use some for the house also and I want to save, and I want to save every day. So I will not buy this thing and I have to use some for the house and save less.

**I Ok, for those who do not save, why do you think they don’t save? Yes, number 6**

PG06 (Birds chipping) I as number 6, I think that maybe their earnings might be low so there is no way they can save and sometimes the little they have made is spent on food so they can’t… or maybe they depend on someone else

**I Is there anything bad about not saving?**

PG06 There is

**I Which one? like which one?**

PG06 If you fail to save money, just as I said earlier, unplanned things can happen. Like let me say, unplanned things are emergency right? So like sickness, you know sickness just comes abruptly. It just comes when you are alone there is nobody to help you. There is no way to help herself. If she had her money, then she goes to do what, she goes to solve that her problem.

**I Number 2 did you raise your hand?**

PG02 No, number 7 is the one who lifted her hand (laughter)

**I Ok those who do not save money, what is the reason as to why they do not save? Just what you think. Whatever you think. Number 5**

PG05 I as number 5, I just feel that sometimes the earning is low. The house budget sometimes is more than the money you have. So it will force you to use all of it.

**I Apart from what number 2 and 6 have said… number 5 and 6 have said, sorry, is there any bad thing that can happen if you don’t save? The disadvantage of not saving money? Let’s start with the person by my side number 8**

PG08 They are the ones that have been mentioned

**I The bad part of not saving money?**

PG08 The bad part in not saving money, is like when you are faced with an emergency, there is no way that you will be able to help yourself. Sometimes the one you were depending on you don’t get from them or maybe you had planned to go borrow from someone and you don’t get from that person, so that will make you cry I wish I knew I would have saved. So, maybe it is a serious illness, maybe it is you or the child. So that will make you feel that you should be saving because when emergency happens you should be taking care of it quickly.

**I And what is the advantage of not saving money?**

Chorus response: the good thing of not saving?

**I Yes, the advantages of not saving? (long pause) Yes?**

(Whispers)

**I If you are saying something, say it loudly so that we can hear you. The advantages of not saving? Yes, number two speak (Laughter) Yes, speak number 2 (silence follows)**

PG02 The best part of not saving money, I still don’t understand that part

**I Is there any benefit of not saving money?**

PG04 There is nothing good

**I There is nothing good that’s number 4 who has responded. Has everyone agreed with what number 4 has said?**

PG02 I support her

**I Only number 2 has supported her, meaning the rest feel that there is an advantage? (laughter)**

PG07 I support her

**I Number 7 also supports her. So the remaining people to tell me the benefits of not saving money (laughter)**

R We all support her

**I All of you support her. What is the advantage of saving money? yes number 2?**

PG02 The benefit of saving money I as number 2 what I think, maybe I was in a *chama* and when I was in the *chama* I have gone and taken a small loan, and I have gone and used this loan in a bad way, and when it is time to pay it back, maybe it finds that I don’t have this money at that time for me to pay it back, it will force me to go back to my savings so that I can take it and pay it so that they don’t penalize me.

**I Another benefit of saving money? yes number 6**

PG06 The advantage of saving money is that you may get a visitor, visitors. You go back to your pocket where you had saved. You will now welcome visitors in a good way, right?

**I yes, number 4**

PG04 The advantage of saving money is that me as a sex worker, I can to work and meet with someone, you come to an agreement, you don’t go to the lodging but you go with him to the house. So when you get to his house, maybe the house was far you will be forced to use fare. So, if you have not saved and you have gone with him after agreeing, and he has left you there, if you have not saved money, or you have saved, you will have to use your money to get back, right?

**I Is there any other benefit of saving money? yes number 2**

PG02 The benefit of saving money in how I know it, is that maybe me as number 2, I’ve planned and the time for paying rent has already come, maybe I do not have the money to pay it and I do not want the landlord to lock my house because I don’t have the money to pay at that time, it will force me to use the money I have been saving so that I can see how to sort it out.

**I And if you save money, what places do female sex works like to save in? what sort of places do they trust? Number 5**

PG05 I as number 5, I save in my *M-shwari*. [A mobile loans and savings app]

**I M-shwari yes, number 4**

PG04 I also save money in M-shwari that is locked that I know when such a date reaches, then I take it out. Because if you don’t lock it, you can use it at any time. Any time that you have a problem, you can just take it out because you are able to see it. But if you put it in a locked account until the date that you have set, you cannot get it.

**I That’s why you prefer M-shwari?**

PG04 Yes

**I And number 5 why do you prefer M-shwari? Saving in M-shwari**

PG05 I usually save with them, sometimes I can be late, I honestly don’t have money, and I take a loan.

**I Ok you can request for a loan?**

PG05 Yes

**I Ok number 6 where do they like to save?**

PG06 Me I have a certain container (laughter) so I place money and then place it under the bed

**I In the house?**

PG06 Yes

**I Mmh, why do you prefer saving in the house?**

PG06 Because it is safe, I am the only one who knows. I live alone with my child.

**I You are not concerned that the house can be broken into? (Laughter)**

PG06 Yes

**I Ok number 3 where do they like to save in?**

PG03 Yes, as number 3 I like to save in M-pesa.

**I M-pesa, why?**

PG03 Because it gives me ease because I don’t use it all the time. I only use it for emergency cases.

**I Yes, number 1 where do you like to save?**

PG01 I prefer to save in KCB bank

**I In KCB bank, why do you prefer bank?**

PG01 I like it because for me to go and take it out will be difficult for me. So that is why I prefer it.

**I ok, you are tired of going to get it that is why you prefer the bank?**

PG01 Yes

**I You can’t find it easily?**

PG01 I prefer it because if you use M-pesa, you know with Mpesa it is easy to use the money so I usually prefer the bank.

**I Ok number 8**

PG08 I as number 8 I like to save in M-pesa

**I In M-pesa why so?**

PG08 This is because for M-pesa it’s a little bit far, let’s say you keep money in the house and you can see it, you will have a lot of yearning because you may see clothes and you have some money close to you. It will force you to take that money to buy clothes with. So, I feel that Mpesa is a bit far.

**I Ok number 7, where?**

PG07 I as number 7 I like to save in M-pesa

**I why is that so?**

PG07 The reason why I like saving in M-pesa, is maybe I have gone to work and I find that work was bad. I only make enough to use in the house or maybe the child was sent home from school, so it makes it easy for me to get it so that I can pay school fees for the child.

**I mmh, number 2**

PG02 I as number 2 I like saving money in M-pesa account.

**I mmh, why so?**

PG02 The reason why I like using M-pesa is because, maybe my child has been sent home for school fees and I don’t have money, it will force me to go to the nearest M-pesa shop to withdraw the money so that they go back.

**I ok that’s fine thank you all. I want us to put our minds… back then we talked about Jitegemee, right? We can all remember we talked a little bit about Jitegemee, and I clarified that its being done so as to see that female sex workers have money they have set aside that can allow them to refuse having sex without a condoms or they can take a break from engaging in sex work when they want to rest. I also said that it is about female sex workers saving part of their money so that they can use during the times that there are no clients or help them prepare for life after sex work. We remember that right? It is something we want women to prepare for life after leaving because you cannot do this work until you are old and are walking with a stick, right? If it reaches a time that you want to leave, how prepared are you, right? Do you think that this is something that female sex workers can accept? A program like this? (short pause) or you have not understood the question?**

R That question is not clear, can you repeat.

**I It is not clear?**

Chorus response: Yes

**I You say. If it is not clear you say, alright? Like we had said what Jitegemee is, Jitegemee is the money that you have, you save a little, right? That you personally know how you can save and what you want to do with it, right? The way we have arranged it, the way we are thinking of doing it, do you think it is something that female sex workers can agree with? Can they accept it and become one of the people in Jitegemee? What you think.**

R Are you talking about women who have husbands, that live with them?

**I No, that do sex work. We are talking to sex workers, yes.**

PG06 I as number 6 as a sex worker I can accept it.

R I support her

R I also support her

**I why so? Why would you accept it?**

PG06 I can agree with it because that work is work where you risk your life. You can be infected with a disease at any time. So if I agree and stop it, I have saved my life. I have saved my life and also my future life has greatly changed.

**I for those who support number 6 that some women can accept it, what kind of women can agree to be in Jitegemee or be part of Jitegemee? (silence) or are we tired? (laughter) opinions? (silence) what kind of women will agree, because Jitegemee is just the money you have is what you save, that is it. Your money is what you divide a little and save. So, what type of female sex workers will accept Jitegemee or being one of those in Jitegemee? (Silence) there is no wrong or right answer, any thoughts are acceptable. yes, number 6**

PG06 I as number 6, it depends on how you make your choices. So, it is a woman who makes right choices can agree to do so.

**I If you say making the right choices what do you mean?**

PG06 If I say making the right choices, so like me, I feel… like now I have learned, I feel that no, if I go ahead with this thing I will face some consequences. If I do this, I can get something good. So, it is my decision. So maybe my decision is important.

I Mmh, another thought? Number 1? (short pause) what kind of women will accept Jitegemee? (Silence, children playing at the background) or let’s start with...

PG02 …let me just try mine I am not sure if it is right.

**I Yes number 2**

PG02 I as number 6,

**I Number 2 (laughter)**

PG02 Oh, number 2, in my own thoughts I can say they are part of those who do *parking (*sex workers). The reason why they will have to leave is because let us say that maybe, like me I had gone there and I meet someone and we agree, maybe I don’t know his status well. So, you know when we have gone and done that thing [meaning had sex] maybe I can be affected. So that is what will help me to go… if I go and test it will force me to just leave it so that I find a way to help myself with the money I had saved.

**I Ok, like in Jitegemee we are not telling you to stop. Like right now you are still going on, right? You are still, let us say you are still going on with sex work. But we still want that even if you are still going on with it, you think about saving while you are still doing sex work, right?**

Chorus response: Mmm

**I Are there girls who will agree to be part of such a program? (short pause) we are not saying people to leave sex work.**

R People to leave sex work?

**I We are not telling people to leave it (laughter) stop sleeping (laughter)**

R No, I am asking you are not telling people to leave sex work?

**I Yes, I am not saying, because the study is trying to find out if they can reduce the chance of getting HIV. So, even while you still continue with sex work how can you protect yourself so as not to get HIV? That is what the research is trying to look at. Now what type of female sex workers will agree to join a study like this one? That’s the question. number 3. I want to hear your voice, even if you don’t know just say you don’t know but at least we hear your voice. (slight laugh)**

PG03 Girls that… sometimes… (laughter) sometimes she has stopped sex work and joined the church.

**I Those are the ones who will agree to be part of Jitegemee?**

PG03 yes

**I Which ones will not agree? (short pause)**

R That will not agree?

**I Yes, that will not agree to join Jitegemee**

R I have not understood that

**I Yes, number 6**

PG06 I as number 6, let me say that those who will not agree… to accept… for them to accept is going to be difficult, you see. For them to accept will be difficult for them but in the end they will accept. That is what I know.

**I What makes it difficult for them?**

PG06 Because they were used to that work (sex work), may be the way they were getting money, the way they were using the money, so if they start saving, they might feel that there is a problem.

**I Number 5, what type of girls will not agree to be part of Jitegemee? That is like Jitegemee?**

PG05 I as number 5, I still don’t know that one, number 4 can assist me (laughter)

**I Number 4 assist her**

PG04 ladies who will not agree?

**I Yes**

PG04 The ladies who will not agree to join Jitegemee…

**I How are they like? Or what is their behavior like. If I say how they look like, I do not mean they are brown or black, no. their behavior, what type of behavior do they have?**

**NT or their character**

**I Yes, or how is their character? (Silence, slight laugh) mmh, everyone must say**

PG08 I can say that they are ladies who love that work

**I what work?**

PG08 Sex work

**I Sex work?**

PG08 yes. She is already addicted.

**I Number 7**

PG07 I as number 7, I feel that the girls who will not agree to join Jitegemee, are those who had already been infected with that HIV, so they feel that there is nothing that can make them leave this.

**I Number 1 (silence)**

PG01 I don’t know

**I Number 1 does not know. Ok. From ten girls that you know, how many can agree to join Jitegemee? let’s start with you, number 1. From the ten girls you know who do sex work how many will agree to join Jitegemee?**

PG01 Few ladies

**I how many are the few ladies 1 to 10, out of 10 how many?**

PG01 One

**I One, ehe number 3, out of ten**

PG03 Three

**I Number 6 out of ten**

PG06 Seven

**I Seven, number 4 out of ten**

PG04 Five

**I Five, number 5 out of ten**

PG05 Two

**I Two, number 2 out of ten**

PG02 Six

**I Six, number 7 out of ten**

PG07 Two

**I Two, yes, number 8**

PG08 Four

**I Four, ok. Like of the six left who will not agree to participate in the study, what is the reason why they will not agree?**

PG08 It depends, sometimes she depends on sex work. So if you tell her to stop, she will find it a little difficult.

**I But we are not telling her to stop. We are just telling her to save money, right? That is what Jitegemee is. If you want to continue you can just do so, we are not telling you to stop, ok? So for those who will refuse to join number 7, what is the reason they will refuse?**

PG07 They will not join depending on that is the life they were used to that if you go and tell them another thing they will not agree with you.

**I Meaning that they would not want to hear anything about saving money?**

PG07 There are those who their thoughts do not rhyme with saving money, that’s why she can’t agree.

**I Number 2, how many did you say will agree?**

PG02 I said six

**I what of the remaining four?**

PG02 The remaining four, the reason why they will not join Jitegemee, is because she is already addicted so for her to join Jitegemee is difficult.

**I why is it going to be difficult?**

PG02 maybe she is already… she has… what is it called in dholuo

**I Just say it even in Swahili**

PG02 She has already been addicted, and she knows that that is the only way she can get money to save.

**I Ok, we are not telling her to leave sex work. She just goes on with sex work. Number 5, why will the eight people that refuse?**

PG05 I as number 8

**I Number 5 yes, sorry**

PG05 I as number 5, the reason as to why the eight will not join Jitegemee, the income might differ, one may go but the income maybe is different. There are some who get more and some get little, and you know every house has their own budget. So maybe they can get little that can prevent them.

**I Number 4, for the remaining ones, why would they not want to join?**

PG04 The reason why they cannot join depends on the challenges that they get.

**I Like which challenges?**

PG04 The challenges is like maybe you can tell someone, the remaining five. You can go to someone and tell her that there is something called Jitegemee, we can come together and save money little by little, and she will tell you… sometimes someone has both parents, so she will tell you that even if she does not save money, my parents can help me or even if I don’t save I have someone else who can help me. So that is the reason why some people will not join.

**I Number 6 the remaining people**

PG06 I as number 6, what I can say on the remaining three people, like now in the field, maybe they make little money. So this money gets used up on their budget, so there is no way for them to save.

**I Ok number 3 the remaining people?**

PG03 I as number 3, the remaining people maybe… if she does not go for that work, she cannot feel… her body will not feel that she is okay. Another way is that some cannot save because she has already loved sex work, so that one she feels even if she does not save she is going to get some money from there because it is a must for her to go.

**I Ok number 1**

PG01 The remaining people it’s going to be difficult based on the way they were used to doing sex work. that is why they cannot leave it easily or they cannot agree to stop.

**I They are still going on with sex work. what will make them not agree to join Jitegemee? Because work is still going on. Sex work is still going on. We have not told her to stop. And she has refused to join. Why is that? (Silence) the nine people that you know who have refused to join Jitegemee. (Silence) it has disappeared?**

R the response has disappeared

**I The response has disappeared? ok let’s continue. Is there anything that we can do so that female sex workers can accept Jitegemee? Is there anything that we can do so that those who do not want to join can join, can accept it? Number 1**

PG01 Advising them.

**I On what?**

PG01 On the benefits of that Jitegemee.

**I Number 6 you have something to say?**

PG06 I would like to support my partner number 1

**I what do you support her on?**

PG06 I support her on sitting down with your colleague, you talk with her, the good things you have experienced in this so that she can also start and see that benefit.

**I Number 4**

PG04 As for me I have some advice

**I what kind of advice?**

PG04 when she leaves work, even when she comes back from the field, I would advise her that when she gets some money, she should save some. If she is someone who can listen, then she will do so.

**I Number 5**

PG05 I as number 5, there are people, let me say like if you teach them they don’t learn. So I can buy something nice and I show off to her (laughter) then tell her that I have bought it with the money I used to save

**I Because you are in Jitegemee?**

PG05 Yes

**I Ok another thought? something else that we can do so that they can agree to be part of Jitegemee? number 8**

PG08 I can only give my colleague some advice on the advantage of saving money

**I Ok, even if it is advice, what sort of advice should it be? Or what kind of things should Jitegemee have? (silence)**

R look like, as in?

**NT what things should it have so that you agree to join Jitegemee? (silence) what is acceptable for Jitegemee to have so that you accept this issue of Jitegemee? (silence)**

**I Is it tough, even if it is just an advice, advice about what?**

PG01 Even if it is advice, you can give a positive advice on… maybe to open a bank account, you can give her advice through a way that you tell her the benefits of saving money in the bank.

**I Only the bank? We tell her the benefit of saving in the bank only?**

PG01 Even if she agrees to save money in M-pesa or in the house then it’s ok

**I So benefit of saving money?**

PG01 Yes

**I Yes, anyone else?**

PG06 I as number 6, if I give advice I’ll tell her about the benefits that Jitegemee has done for me, right? I tell her what I was doing, how I was saving and what I have done what- I have bought. Maybe I have bought something that she can see or I have done something that she has heard or I have the proof of what that thing has done. So, I can tell her that and it can change her mind to make the right decision.

**I ok, another thought? (slight laugh) number 2**

PG02 I as number 2 the advice I can give her; I can give her advice through how I have seen the benefit of this Jitegemee. Because maybe I depend on myself and have saved something, and I buy something for myself like I had said I bought myself a goat. So, you know if I go and advise her and tell her that Jitegemee has helped me with such and such a thing, if she can listen then she will listen.

**I Number 7 you have been quiet for long.**

PG07 I as number 7, what I can only say is the advice that I give to my colleague, I can tell her about how Jitegemee has… that is how we were taught and joined Jitegemee and got some benefits and bought something in the house that she can see, then she’ll say that this thing is truly good, then I will tell her the reason why I bought it and how I bought it.

**I Are there things that they would not like to see in Jitegemee? (short pause) because we have said different advices that are good if Jitegemee has them, right? Are there things that they would not like to see in Jitegemee? That if someone hears that this thing is in Jitegemee, it can discourage her. number 3 the one you are thinking of.**

PG03 what can make her loose interest is that, she is already used to doing sex work and this will force her to depend on herself and it will force her to stop sex work yet she does not want to stop that work. It is something that she is used to.

**I You remember that Jitegemee is not asking you to stop sex work. jitegemee only helps you to think about saving while you are still doing sex work, right?**

Chorus response: Mmm

**I That even if it reaches a time that you cannot do sex work or the clients are not around, there are times that there are no clients isn’t it?**

Chorus response: Mmm

**I Or are they available every day here?**

R There are times that they are not available.

**I There are times they are not there, isn’t it?**

Chorus response: Mmm

**I So even if they are not available or you are tired, then you don’t go hungry. The children don’t go hungry, you don’t miss paying rent, right? But not that you have stopped sex work. so, when you are still going on with sex work, what should not be in Jitegemee? (silence) you are the voice of all the other girls, and if you don’t speak then…**

R I haven’t understood that question well

**NT That is, she is asking, we want to start an activity called Jitegemee, right? we already said what we want to do. we want to reduce the chances of those who do sex work, reduce their chances of getting HIV, right? Then we want to see that those who join Jitegemee study, will save their own money, right? Not that we are the ones giving you money. Your money that you have saved, you can withdraw it at any time that you want it and if you like you can withdraw all of it or you withdraw a little and leave a little, and where you have saved it, there is no interest that it will get, right?**

Chorus response: Mmm

**NT so, what things will you not like about Jitegemee? (silence) Is the question clear?**

R It is clear

**NT Now answer**

**I Or has it passed? (laughter) yes, if it is understood, your thoughts? Yes, number 6 or you have not raised your hand?**

PG06 I haven’t raised (laughter)

**I Number 5**

PG05 I as number 5 depending on oneself is good. I would not like it when let’s say that sex work can be closed down, because it is the only way we have set in our minds to make money.

**I Someone else with a different opinion? (silence) number 7**

PG07 I do not know how to respond on that

**I You don’t know? Number 4**

PG01 I support her

**I how do you support her?**

PG01 I support her in a way that if that work has been shut down, maybe that is the way we were making money so that we can help ourselves or we save so that we can support ourselves. So if it is closed there is no way that we can support ourselves.

**NT Let me clarify something to you there,**

**I It hasn’t been shut down**

**NT you see Jitegemee, we have said that Jitegemee wants to reduce your chances of getting HIV, right? And this you can only do so if you have your own money, your own money that you can do with whatever you want when you want. You do not rely on someone else, right? We are not telling you to stop sex work, but we are trying to encourage you to save the money that you get from sex work, if you got 100, you use five and save five, right? Meaning you were using fifty and saving fifty, right?**

Chorus response: Mmm

**NT so one day, there will be a time that you will not do this sex work forever. So, at the time that you have stopped, how will you survive? So what things will you not like about Jitegemee? (Silence)**

**I You are saving your money. There is no money we are giving you, right?**

Chorus response: Mmm

**I That one is understood? You are also going on with sex work. we meet with different people, right?**

Chorus response: Mmm

**I do we know all their status?**

R No

**I There are those that we don’t know right?**

Chorus response: Mmm

**I There are those who don’t want to use condoms, right? Or do all of them agree using condoms?**

R No

**I There are those that refuse, right? So, Jitegemee is trying to open our minds to see the future so that if it reaches a time that the client tells you to have unprotected sex, you can refuse because you have some money that you have kept. But you know if you don’t have that money it will force you, right? And you don’t know this person’s status. You can get infected. Those are some of the things we are thinking of preventing. So, in a study like this one, what don’t you want to see in such a study? Let me put it that way. Or are we confusing you more? You know if we talk a lot maybe it is getting… number 5**

PG05 I as number 5 what… like me, I have protected myself. When you go there you have to protect yourself, right? So, sometimes someone comes and wants to have sex with you, you have talked and you go, then he tells you to have unprotected sex. I can tell him that I have my own condom and he tells me that he doesn’t want a condom. Like me I can use PrEP and it can protect me.

**I Ok. So if you join Jitegemee, what should it not have?**

PG05 If I join Jitegemee… (Laughter)

**I Ok, like we had asked is there something that you will not like in Jiteegemee? (Silence)**

**NT If there is none you can also tell us that there is none and we will continue**

**I Yes, don’t just keep quiet**

**NT Yes**

PG01 I don’t know

**I That’s number 1 who does not know, meaning the rest of you know. Number 5**

PG05 I don’t know

**I You also don’t know? So even if Jitegemee starts you will agree with it just the way it is? the way it comes is the way it will be?**

R yes

**I Ok. If all of you do not know let us continue. What is it that we can do to increase female sex workers… Sorry let me repeat. What is the main thing that female sex workers will like about Jitegemee and why? Since there is nothing that you will not like, you have not told us. meaning you like it just the way we had said it, right? They can… what do you like the way we had said it? Or will other girls like about Jitegemee? About what?**

PG04 I can love it because I am saving my own money.

**I ehe, that’s number 4, number 6**

PG06 I can like it because it gives me self-dependence I do not depend on someone else

**I That’s number 6. another thought? Why would you like Jitegemee? Let’s be a little fast so that the rains don’t catch up with us**

PG01 I like it because it makes me depend on myself and don’t depend on someone else

**I That’s number 1’s thought, mh! Number 2**

PG02 I can love it because I depend on myself and do not depend on someone else as I depend on myself

**I NumbePG07**

PG07 I as number 7 I can say that I depend on myself as in it helps me that’s why I can join it

**I When you say it helps you, in what way does it help you?**

PG07 First of all the information it has given us and how we are going to save and how we are going to save to support ourselves without relying on other people.

**I Number 8**

PG08 I can love it based on how I now depend on myself. It’s not a must that I beg someone so as to support myself.

**I Ok. Are there things about Jitegemee that female sex workers will feel infringe on their rights? Rights are *haki (*rights). Jitegemee, how we have heard about it. Do you think other women will feel that their rights are being infringed? Yes, Number 4**

PG04 I as number 4 my thoughts are that she will not see that her rights are being infringed because she saves if she likes and if she likes she does not save

**I That’s number 4’s thought number 6**

PG06 I can see that some women feel that their rights are being infringed because as we know in the house, the man is the head and you do not have that husband, and when you go there for sex work, that person will act as the husband. so if you stay alone, though you still go for sex work, it will infringe on the rights of that man in the house.

**I It has infringed his rights in what way? For that man in what way?**

PG06 For the woman not the man’s rights

**I Oh, the woman in which way?**

PG06 It has infringed in that, like the way… let me say that the things that she was supposed to get that the man has… she is the one providing for herself.

**I So for that she has a husband or she is alone?**

PG06 She’s alone

**I She is alone, so how does it infringe her rights?**

**NT I want to clarify that question to you, if you join the Jitegemee study will it infringe your rights in any way, you who is here, will it infringe your rights in any way? (Silence, a child crying at the background)**

**I Number 4**

PG04 The way I see it, there is no way it is going to infringe on my rights because I depend on myself. If I like I can save and if I like I won’t save, so there is no way it will infringe my rights

**I Number 3, then 6**

PG03 I as number 3 there is no way it will infringe on my rights, because I am the one who knows how I save money, at times I save at times I don’t save. So there is no way it is infringing on my rights since it is something I do if I like.

**I Number 6**

PG06 I also support what my fellows have said

**I you have changed your mind (laughter)**

PG06 I didn’t understand the question well

**I Oh, Ok someone else with a different thought? those who are quiet**

R I support

**I who do you support?**

R number 4

R Number 4 (laughter)

**I ok that’s fine. So what do you think are the challenges that we might come across when we will be introducing Jitegemee? Like what we have said what Jitegemee is, yes, number 4**

PG04 There must be challenges, because there are some of us who will not agree to save money looking at the difficulties she has in her house. so there must be challenges

**I So some people will not agree to save money?**

PG04 Yes, there are some who will not agree

**I Another challenge that we may come across when we introduce Jitegemee? Each and every one must give me a challenge. number 5 let’s start with you**

PG05 I as number 5, the challenge that you may face is that at times you may keep money, maybe you use it in the wrong way, then there are people who don’t want to save, right? Maybe she is truly your friend, maybe you have saved but it will force me to share with her a little so that she can help herself when she has a problem.

**I Number 2**

PG02 The challenge that I see is that maybe I depend on myself, and I didn’t go to the field or I didn’t get money, it will force me to go take that money which I saved

**I Number 7 the challenge that we may come across when we introduce Jitegemee? (a child crying at the background)**

PG07 The challenge that we may come across?

**I That us who will introduce Jitegemee may face**

PG07 how I see it, challenges may happen depending on us who are saving in the house, I mean I am at home. Because being that I don’t have a husband, so the way I can save will reduce because maybe my earnings are going to be little. So I may not save money.

**I That’s the challenge that you may face as a participant in Jitegemee? (silence) number 7 (silence, chairs dragging) that is the challenge that you as a participant in Jitegemee may have? Am asking us who are starting Jitegemee, what challenges will we come across? Let’s say we were introducing Jitegemee today right, we have come to talk to girls, right? What challenges will we face?**

R ok the challenge that I may face is that…

**I The challenge that we may face, us who bring the study**

PG01 challenges that we will face is that when we introduce it, it’s going to be difficult for me in my way of saving because when I was going for work, my earning was good, so when you introduce it, it is going to be low because when I get it I will put it in the account or where I am saving. So for me to find a way to use it will be difficult.

**I Number 6**

PG06 I as number 6 the challenge that you may face when you bring it, you may find someone who maybe cannot speak, so how you can explain to them something, they can’t understand it well.

**I So she doesn’t hear clearly or she doesn’t understand?**

PG06 She doesn’t understand and she may not understand what you are saying.

**I Number 4**

PG04 The challenge that you may face, is that you can bring it as a group where everyone can save their own money, and maybe you have saved it today, it can be set that people meet once a week. Maybe she saves money today and when it reaches the following week when people are supposed to meet, she gets a problem, she comes to take her money and maybe you have kept that money in a locked account, maybe people have an agreement that people get the money after three months. Her when it gets to a new week she wants money. That will be a challenge.

**I Ok that’s fine. Is there something that we can do so that we overcome those challenges so that they stop being a hindrance? like number 4 has given us 2 points right? Of getting those who want to save, was it that one?**

PG04 Yes, getting those who want to save

**I How can we address it? How can we overcome that challenge so that girls agree to save? That sort of challenge. Anybody can respond. That is not a question for number 4 only. That is the challenge she mentioned, so how can we overcome that challenge? Number 3**

PG03 Through advising them. You first give them the benefit of depending on themselves.

**I Another way that we can overcome that challenge? (Silence) so that people can save, if any? or helping those who do not understand clearly, how can we overcome that challenge? Number 4**

PG04 How we can overcome that challenge, so you know you are going to get like a group. You cannot tell someone that you want them to save money every day, you cannot tell her that maybe you want her to save five hundred, maybe she cannot get that five hundred. So that you overcome this challenge, if it is saving you tell her to save what she can. It is not a must to save that five hundred. So that we overcome this challenge, you tell her, you can only save the money that you have.

**I Ok for those who do not understand well, how can we overcome that challenge? Yes, number 6**

PG06 You try to be humble with them. You explain to her clearly so that she can understand.

**I So in what way, what can we do? example of what we can do?**

PG04 I want to assist her

**I Yes, assist her number 4**

PG04 Those who do not understand, you know you cannot talk with someone once about something that she has not seen with her own eyes and she understands. So, you know you have to talk with her several times that you can even come back three times in order for her to understand what you were saying. That is how it can be solved.

**I Number 8**

PG08 I can only say that you teach her, you don’t get annoyed. Even if you come to her today, another day you just keep on coming until the day she will agree to be part of Jitegemee.

**I Ok so we keep on repeating?**

PG08 Yes

**I That’s fine. In every week, if you join Jitegemee, number 8, how much money can you save every week? You don’t burden yourself. You just spare a little, right? We have daily needs, right?**

Chorus response: Mmm

**I and you have joined Jitegemee where we want you to save a bit. In a week, how much can you save?**

PG08 I can save one thousand shillings

**I One thousand, that’s in a week. numbePG07**

PG07 I can save four

**I four?**

PG07 four hundred

**I Four hundred, yes number 2**

PG02 I can save five hundred

**I Five hundred, number 5**

PG05 Seven hundred

**I Seven hundred, number 4**

PG04 Five hundred

**I Five hundred, number 6**

PG06 Seven hundred

**I seven hundred, number 3**

PG03 Two hundred

**I Two hundred, number 1**

PG01 Five

**I Five hundred. So that is what you plan right?**

Chorus response: Mmm

**I That is just an example that we are using right? So if you are unable to get, like number 8 has said one thousand. If you are unable to get it, what can you do to get it?**

R If you are unable to get the money that we have said or what?

**I Yes, the money that you target to save. You have set a target, right? You would like to save that money every week, right? But you are unable to get it. What will you do so that you can get that money? number 1**

PG01 As I have set my target for five hundred shillings, so as to get it every week, in case I am unable to get it exact so that I can save it, I will try in all ways so that I can get it to save that five hundred.

**I So those are the ways that I want you to tell me, what are those ways?**

PG01 Like having another thing that gives me money that will enable me get that money to be able to save it.

**I If you do any other thing, what can this other thing can be?**

PG01 By… like going to assist someone in the house

**I Ok someone else? number 3 if you don’t get your target what can you do to get it?**

PG03 If I don’t meet my target, it will force me to go and help someone in any kind of work, any kind of work that can give me money so that I can be able to save it.

**I so any kind of job can be what kind of job?**

PG03 let’s say like babysitting in a day.

**I Number 4, yes**

PG04 I as number 4, what I can do to reach five hundred shillings, I work in a bar, maybe where I work am being paid one fifty shillings. So, this one fifty is paid every day and I have said five hundred. In a week we have 7 days. So, in these seven days, it will force me to use half of that money and save half, so that if it reaches… let us say that the meeting is set like on a Thursday, when it gets to Thursday, I have that five hundred. You know before that meeting; I save on my own so that on the day of the meeting I have the target that I had said.

**I Ok saving, number 2**

PG02 I as number 2, what I can say so as to meet my target that I had said five hundred, it will force me to go to the beach to clean for someone fish. So I’ll take some and save so that when it gets to a week, I will have saved enough of the money then I save.

**I That is cleaning fish?**

PG02 Yes

**I Number 7**

PG07 I as number 7, if I fail to reach the target that I have which is four hundred shillings, what I can do, which mostly I am doing, I can babysit someone’s child. There is a lady who works in a school, I babysit for her the child and in a day she can pay me even two hundred fifty at times two hundred. So I can add it to my money and send it.

**I That’s babysitting. yes, number 5 what can you do if you fail to meet your target?**

PG05 I as number 5, (someone coughing) if I fail to get the target I can work in a hotel and at the hotel they pay two hundred shillings or one fifty shillings. so if it reaches one week I can save 700 shillings per week

**I That’s working in a hotel. Yes, number 8**

PG08 I as number 8, so as to reach my target of one thousand shillings I can also just go to the beach to clean fish.

**I Cleaning fish. Who hasn’t told me, number 6?**

PG06 I as number 6, what I can do so as to reach the target that I had set, I will find someone to go with (have sex with) so as to make me fill it.

**I That’s sex work oh. you get more clients?**

PG06 Yes

**I Ok that’s fine. Apart from sex work is there something else that you can use**?

PG06 Yes, I can sell things for someone.

**I Selling, ok. Where is the best… or let us say that Jitegemee is starting and we should save, where is the best place to save in? Number 1**

PG01 In a bank

**I Bank, number 3**

PG03 Through bank

**I Bank, number 6**

PG06 Locked account

**I Locked account, that’s through phone?**

PG06 Yes

**I Number 4**

PG04 Table banking

**I Table banking, saving?**

PG04 Yes

**I Number 5**

PG05 M-shwari

**I M-shwari, number 2**

PG02 (laughs) M-pesa

**I M-pesa, number 7**

PG07 KCB

**I KCB Bank?**

PG07 Yes

**I Number 8**

PG08 M-pesa

**I M-pesa. Ok, don’t get too tired we are almost done. So I want… are there female sex workers who have a lifestyle that is above their means? Are there female sex workers who have a lifestyle that is above their income? You earn… example, you earn five thousand, but your lifestyle is ten thousand. Are their people who live like that? Number 4**

PG04 There are some who live like that. It depends. the reason she lives that way… someone goes to live in a rental of say five thousand, and she depends on sex work, I mean she depends on someone else’s money that she has not worked for, she has children at home, there is also school fees. So you know the money that she is depending on is going to be difficult.

**I Ok another thought? Those who live in a lifestyle that is above their means? (laughs) is there? Number 2**

PG02 They are there

**I What is the reason why they live that way or why do you say so?**

PG02 The reason why I am saying so or the reason why it is so, you find that someone goes… that is she eats good foods and then she has put her lifestyle to be very high. So you see that sometimes in the field, maybe she has gone but has not managed to make the money that she was supposed to.

PG06 I as number 6, that can happen if she was leading that life earlier and later her income reduced. So she gets some challenge.

**I Ok. And how do female sex workers fill up the gap? She is living in a lifestyle that is higher than her earnings, right? How does she fill the gap? Yes, number 4 (laughs)**

PG04 If someone is living a high life, and there is a gap, and she does sex work, it will force her in a day to go even with five clients so that she can fill the gap that is remaining.

**I So in that case she has to have many clients?**

PG04 Yes, she has to have many clients

**I Ok, number 7 you raised your hand**

PG07 I as number 7, I feel that how they can fill their money if it is less, I feel that she will have more sexual partners. If she goes with someone and makes a little money and she sees that is less, she goes with another person so that she can reach her target.

**I Number 6**

PG06 I feel that she can add with another job. So, if this one brings this and it is not enough then she adds with another job so that she can fill that gap.

**I Another job like which one?**

PG06 Like going to sell for someone, do laundry for people so as to fill the remaining part.

**I Ok, number 1, what can she do in order to fill the remaining part?**

PG01 I support what number 6 has said

**I What did she say?**

PG01 In order to fill the remaining part… so that she can fill the remaining part, she looks for another small job apart from the one she is doing. She looks for another job so that she can top up the remaining part.

**I Ok. Where do female sex workers borrow money from?**

PG02 *chama (*group banking)

**I *Chama,* that’s number 2. Number 6**

PG06 if at all they save in M-shwari

**I Number 6 M-shwari, yes, number 5**

PG05 She can save in M-shwari

**I M-shwari, number 8**

PG08 She can borrow from *chama* (group banking)

**I Chama, number 3**

PG03 Through M-pesa

**I M-pesa, number 7**

PG07 Through M-shwari

**I M-shwari, and if they borrow, why do they borrow? Normally number 5**

PG05 I as number 5, sometimes you have a problem, that is where you will think of first and think let me borrow from these people and sought out my problem.

**I Number 6**

PG06 Maybe she wants to start her own business, so she borrows to start it.

**I Ok number 1**

PG01 maybe the child has been sent home for school fees so as to pay fees in an easy way she will go and borrow.

**I School fees, number 2**

PG02 Sometimes I saw something nice that I wanted to buy and I didn’t have the money at that time. So I will have to borrow that money and go and buy it.

**I What do they do so as to pay the money that they have borrowed? What can they do?**

PG02 It will force her to go for sex work. I just go and do that work. so after I have worked then I will get that money and come and pay back.

**I Isn’t that the work that you are doing? So what can you do that is different from that?**

PG02 It will force me to go work for someone like a house girl, where I can go work in the morning and come back home in the evening. And when I return I come back with that money in my hand

**I Ok that’s number 2, number 4**

PG04 I can assist a little, the reason as to why you will look for that money, if that is the only work that you have, sex work, it will force you to go with different people so that you can get that money to pay back. Because that is the work that you depend on, you don’t have any other. It will force you to go with different people so that you can get that money.

**I Every day we have different people, right? Or are there…**

PG04 No, you know there is… there is a difference. Yes, we go with different people. Today you go with this person, tomorrow with the other person. So you know maybe you had borrowed money and it is being paid in a week. This week maybe you had borrowed ten thousand. Before that week you don’t have ten thousand. It will force you to… now maybe you usually go at night, it will force you to go night and day so that you can get that money.

**I Oh, so you do double shifts?**

PG04 Yes

**I Ok. Yes, number 6 (laughter)**

PG06 How I can respond to it, I start saving so as the days go by I save some, as the days go I save some so that when the day comes to repay it I take it and pay the loan.

**I You save?**

PG06 Yes

**I Another thought? The person who is more tired everyone else number 7 (laughter) we are about to finish, don’t get too tired ok? A thought, what can they do so that they pay back the money they borrowed?**

PG07 I feel what they can do, on my side you may get tired of doing sex work, so if you want another way you can go to someone and even fetch water for her, in my view, I will fetch water for someone and get paid so as to save money and pay back.

**I So the things we have just said are the things that we do to pay back the money we have borrowed, right? So, what can we do to increase our earnings? there is sex work that we do, right? What can we do so that if I usually get five hundred then I can start getting one thousand? Let us start with here.**

R If we are still doing sex work?

**I Yes, we are still doing sex work?**

R That is different from this one?

**I Even if it’s different or it is sex work, I don’t know (laughter) what can we do to increase our earnings? Yes, number 6**

PG06 I can look for something and sell and I am also doing sex work, so my earnings will keep on increasing.

**I If you are selling, what are you selling? Like what?**

PG06 Clothes

I Clothes,

PG06 Shoes

**I Aha, yes number 2**

PG02 I also support number 6, I can look for something to sell and if at all I look for it, I can set a place to make and sell chips and if I get some money I can save

**I So it will increase your earnings?**

PG02 Yes

**I Number 5**

PG05 I as number 5, I can add it with working in a hotel

**I Ok number 3**

PG03 I as number 3, I can add it with selling shop stuffs, selling for someone shop stuff

**I So if you say selling things in the shop are they household stuff or shop clothes or what are they?**

PG03 Am saying that I can go sell for someone in their shop

**I A shop that sells sugar or a shop that sells clothes?**

PG03 A shop that sells sugar

**I That sells sugar, ok number 4**

PG04 I as number 4, the way I said earlier that I can work in a bar, sometimes you know someone can come and buy you something. In order for the money to increase, you can sometimes if they buy you even a soda, you can return it back to the counter so that you get the money (laughter)

**I So you make a deal with the bar tender?**

PG04 with the accountant

**I Ok another way that you can increase your earnings? (Silence) are they over? Who hasn’t told me number 8?**

PG08 I can only add the one I said of cleaning fish

**I Of cleaning fish, just cleaning fish? What if there are no fish? (Laughter) what if there are no fish in the lake?**

Chorus response: *Omena* (sardines) cannot miss

**I There are Sardines? what if there are no sardines?**

**NT Number one is still remaining**

PG01 Number 1, I’ll mix with…

**I you mix with?**

PG01 I’ll mix it with selling vegetables

**I Selling vegetables that’s number 1, ok. And how do female sex workers know when there are no clients? (short pause) There are times that they are not available, right? (silence) or are they available every day these sides? (silence) yes, number 4**

PG04 I as number 4, how they will know, you know in this community you cannot do it. You will have to go to town at =Kondele=, that is where they are. So for you to discover that they are not available, because there are only few people who have come there, and they choose who they want. You if you are not chosen you remain. You will have to conclude that there is no work today.

**I someone else? how do you know that there are no clients? (Laughs) yes number 6**

PG06 The way I see it, like when you go to the beach, there is a lodging close to the beach, maybe you have gone to the bar there and those people have come but they don’t bother with you (laughs)

**I You have sat down looking good and no one minds you, ehe,**

PG06 You are just seated scrolling through your phone and nothing (slight laugh)

**I Ok, number 7 how do you know that clients are not available?**

PG07 How I’ll know that there are no clients, is that when you are in the bar and are seated, maybe you started sitting from morning and the time is almost reaching one o’clock and no one has even asked for your name (laughs), so that is what will make you know that work was bad.

**I Was bad, ok that’s fine. When female sex workers borrow money, how much do they usually borrow? If they borrow money how much can it be? Number 1**

PG01 On my side I can say ten thousand shillings

**I Ten thousand, yes, number 3**

PG03 I can say three thousand shillings

**I Three thousand, number 6**

PG06 About twenty

**I Twenty?**

PG06 Twenty thousand shillings

**I Twenty thousand, yes, number 4**

PG04 Ten thousand shillings

**I Ten thousand, number 5**

PG05 Five thousand shillings

**I Five thousand, number 2**

PG02 Ten thousand shillings

**I Ten thousand, number 7**

PG07 Eight thousand shillings

**I Eight thousand, and number 8**

PG08 Ten thousand shillings

**I Ten thousand, ok. We are going into the last section, these two neighbors are getting very tired, (laughter) we are almost through ok? Do you think that sex workers have ever thought of leaving sex work? Are there those who have thought of leaving?**

R the ones who have thought to leave?

**I Yes, thought of leaving sex work. Number 1 yes**

PG01 On my side I can say that there is none because that’s where they get their earning which they use to support themselves in their houses.

**I There is none who you know that has thought of leaving sex work?**

PG01 None

**I There is none?**

PG01 Yes

**I Yes, number 6**

PG06 There is, it is possible

**I There is one who you know or you have heard them say that they want to leave?**

PG06 Yes

**I Number 4**

PG04 There are depending on the challenges they face there.

**I Those who want to leave, have you ever sat down as sex workers and discussed that you are thinking of leaving or… like number 6, how did you know that she wanted to leave?**

PG06 For me… she had already stopped and she told me the story

**I Ok, what is the reason why they stopped?**

PG06 The challenges that she was facing, maybe she has had sex with someone’s husband and the wife came and stabbed her with a knife

**I That’s the only challenge that she faced?**

PG06 Yes, they kept on fighting with other ladies over men

**I Number 4, the person that you know who wants to leave or has stopped sex work**?

PG04 There is, the reason why she wanted to leave is due to the challenges. Maybe you have an agreement with someone and you go together, so you know, in the field it is makeup, it is said that makeup is beauty, so when you go with him to the house he finds that the face that he chose that was beautiful to him in the light is different (laughter) it will force this person to run away at that night time and there is nowhere for you to go to. So, those are the challenges that they face. Sometimes you have gone and sat down without a shilling and nobody has also called you. Those are the challenges that they face.

**I Ok, number 6**

PG06 The challenges that you may also come across, you find someone and agree to go with him, after you have gone with this person, he removes his clothes and goes to the bathroom, he has gone to take a shower and if you look in his clothes and what he has carried you see knives (laughter) what will you do? you just decide that let me leave this work.

**I Number 3**

PG03 Ok, I as number 3, the reason as to why someone can stop, is that, maybe you truly go with someone and you have agreed on the price and after you are finished having sex he will not give you the amount you had agreed on.

**I Those are the things that can make someone want to stop?**

PG03 Yes

**I ok, number 5**

PG05 I as number 5, the reason as to why someone can stop this work, maybe you go, let me say I queue then go the first day and come back empty handed, the second time the same happens, the third time the same. You feel discouraged, right? So you stop.

**I NumbePG07**

PG07 I as number 7, I would like to say that there are some who have stopped and the reason why she stopped sex work she said is that what she is passing through, she feels that the things she is going through are not good because she said there is a day she went somewhere and she went and found blood under the seat,

**I She found what?** (Slight laugh)

PG07 She found blood in a basin

**I Oh, blood ehe,**

PG07 So she was suspicious that she might be slaughtered so when she came back she got saved and started praying

**I what did she find? Blood in a basin?**

PG07 Yes

**I Under the seat?**

PG07 Yes

**I Ok ehe, then what happened?**

PG07 So when she came back she said that she has stopped that work. she was like someone who escaped death and she said she has stopped sex work. that is the challenge she had.

**I Did she leave the work? Or she went back to it?**

PG07 She left

**I She left it for good, she didn’t go back to it?**

PG07 Yes

**I Alright, and if people talk about leaving sex work, is it something that people can talk about when they are seated together the way we are or is it something that someone thinks on their own? Yes, number 7**

PG07 I as number 7, there could be your fellow who you are close to that has come across such kind of things, she can come and tell you her secrets, what has happened what she has gone through. She can come and tell you then you know the problem that she has, because she trusts you and she can come and share with you. That is how you can know such things.

**I Yes number 6**

PG06 I can stop by myself depending on what I have seen.

**I Like what? (Laughter)**

PG06 Like at times I may meet a man and he gives me some alcohol, then he goes and has sex with me and leaves me there while I am drunk and he leaves. He has used me. Then I will just think that this- (laughter) this is difficult, let me just stop the sex work myself, because if someone teaches me that, it means that he does not want something good for my life.

**I Ok, number 2**

PG02 Like I see it, the reason why I can stop sex work, maybe I have gone with a client and after going with him I have sex with him. After finishing he doesn’t give me anything and I am left there.

**I What is this something? I think that you have already worked something with him.**

PG02 There are some who first have sex with you and they pay you later.

**I Ooh, Ok**

PG04 he will tell you to just go we’ll see each other (laughter)

PG02 Yes, just go or just go I will send you the money to your phone.

**I Then fails to send?**

PG02 He doesn’t send

PG06 I support number 2, or may be this person will pretend to give you money, then injects you and after injecting you, he will take that money and leave with it.

**I These are things that happen?**

Chorus response: Yes

**I That can make someone want to stop sex work?**

Chorus response: Yes

**I Ok, alright. Is there a certain age that when one gets to that they have to leave sex work? Number 7**

PG07 Ok there is, but with the current ladies they do not want to be old (laughter) you can find an elderly woman has worn makeup that she doesn’t look like an old person yet she has grandchildren. You will find that she has worn makeup so as to go for sex work and do that work, that’s how I see it

**I Yes, number 8**

PG08 I feel that there is a certain age that when someone gets to, she just has to leave sex work because you will find that maybe you are *faded* (very old) that you don’t look as young and attractive as before

**I So which ages, like how old?**

PG08 A person who is 50 years and above I feel will not be chosen a lot anymore.

**I She should stop sex work? yes, number 4 (laughter)**

PG04 With the Kenya we live in now, there is none because even if she gets to that 50 years and above, she will reach a point that she is called sugar mommy. She is the one buying not that someone is buying her. So there is none.

**I So there is no age in which one can stop sex work?**

PG04 There is no age

**I Number 6**

PG06 On my side I feel that there should be, because depending… if you have reached 40 to 50 years, your children are big. So, it should be that the money you were saving, you should start a business with it. One that helps you now that you are old, and your children can now help you, you stop doing that work.

**I Number 2 you carried your hand up?**

PG02 I feel there are some who should stop. The reason why they should stop is that you can find that now you are older, you have your daughter in law, some of your sons are married. So maybe you want to put on makeup you want to go out to go for sex work, you should be showing some respect to your daughters in law.

**I That’s about what age?**

PG02 From 50, 60 years.

**I 50, 60 years. yes, number 1, (silence) what does that mean? this doesn’t see you? You have to say it with your own voice**

PG01 none

**I none?**

PG01 Yes

**I Ok. Women who have stopped sex work, what can they do? What type of work?**

PG02 house help

**I That’s number 2 house help, number 5**

PG05 In a bar

**I In a bar, what does she do in the bar?**

PG05 Serving customers

**I Serving customers, that’s number 5. Yes, number 1**

PG01 Working for someone, like babysitting then you get paid

**I Number 6**

PG06 Open up a business to start life

**I Like which one?**

PG06 Selling things, she can sell things like basins, clothes

**I Ok, number 2**

PG02 Working in a hotel

**I Working in a hotel doing what?**

PG02 She can cook chapatis, she can cook ugali, serving customers

**I Cooking or service**

PG08 She can sell things in the shop for someone

**I Oh, I thought you were going to tell me something to do with fish, (laughter) apart from fish. Ok. Are there bad things that happen to those who have stopped sex work? Number 5 yes**

PG05 maybe the way I am in the village here, there are those who knew that I do sex work. there are those who will abuse you how you were doing that work. those who will criticize you will also be around.

**I Any other bad thing that happens? yes number 1**

PG01 Another bad thing that will happen is maybe you are taking drugs. So those drugs will work in your body and give you some effects in your body.

**I What medicine are they taking? There are a lot of drugs (motorbike riding at the background)**

PG06 Can I help her?

**I Yes number 6 assist her**

PG06 ARVs

**I ARVs ehe, your point?**

PG06 My point is that sometimes the way I was earning (a child crying at the background) is different from how the business that I will start will bring in, so that’s the big challenge

**I That’s the bad thing that can happen, another thing? (child crying at the background) that’s the remaining question left then we finish. Mmh, another bad thing that can happen? (silence) Do you know anyone who has gone and come back, who left sex work and came back?**

R Left and then came back?

**I Yes. Who had left sex work and has gone back to it? Each and every one. Do you know someone number 8?**

PG08 I do not know

**I Number 7**

PG07 I know of one who left and left for good

**I She didn’t come back?**

PG07 yes

**I number 2**

PG02 I knew of someone who left and went back to it again

**I What is the reason as to why she went back to it?**

PG02 The reason why she went back to it is that when she left, she went to work in a hotel. So, when she went to work at the hotel and you know with hotel work if you compare the money that you get at the hotel and the one that you get from sex work, the earnings from sex work is more than the hotel. So she had to return to it.

**I So she went back to it, ehe, number 5**

PG05 I as number 5, I have seen a lady who has stopped sex work. She left it then went back to it again.

**I Why did she come back to it?**

PG05 When she left, she was just working in the bar and when she compared what she made in the bar and the one she got from sex work, she said it was different for her. She was already used to sex work, so she felt she should come back to sex work and she is going on with it.

**I Number 4 do you know of anyone who left then went back to it?**

PG04 The reason as to why someone can leave and go back to it, if she compares what she used to get from sex work with what she gets, there is a difference.

**I There’s a difference like it has increased or reduced?**

PG04 Has reduced. When she leaves… even if she was working in a hotel she is going to be paid one hundred and fifty. So, when she compares that she can work in the hotel from morning to evening, even from six in the morning to six in the evening, and she wants to be given one hundred and fifty, and maybe where she was working you come to an agreement with a client to give you even six hundred for two shots, there is a difference.

**I Number 6**

PG06 I have seen. she used to do sex work and left then got married. so when she is in the house she is only with her husband. She felt that she has been kept captive and in that life she was free, that is why she went back to sex work.

**I She went back to it. Yes, number 3**

PG03 I haven’t seen

**I You haven’t seen?**

PG03 Yes

**I Yes, number 1**

PG01 Not yet

**I Not yet. now someone told me she knew of someone who left and left it for good it was number 7 right? What made it easier for them to leave it for good? What made it easy for them to leave?**

PG07 Because of what she saw

**I What she saw, oh, she is the one who saw blood in a basin?**

PG07 Yes, she said that she can lose her life so she stopped so as to take care of her children

**I someone else who knows someone who left and left for good? what made it easy for them to leave or anyone who has stopped? number 6**

PG06 There is someone who had left for good, because when she narrated for me she even showed me a certificate (markings she was left with from a previous occurrence). When she showed me, she pointed the marks on her body, she saw some knives, I mean some knife markings of where she was stubbed, so that’s why she left the sex work for good and never went back to it.

**I So it wasn’t as easy?**

PG06 Yes, sometimes she would want to go back but she remembers that

**I Ok, is there anything that female sex workers would like to do before leaving sex work? (silence) Like for example, we are all still doing sex work, is there something that you think that after a certain period of time I would like to stop right? Is there something that someone would like to do so that when she leaves she’s through with that thing? number 6**

PG06 I would like to buy a piece of land, buy my own things, buy animals so that even if I leave it I have somewhere.

**I That’s number 6’s thought, yes number 2**

PG02 My thinking is that I would also like to buy my own things. I would like to buy domestic animals so that in case I stop sex work I can take that animal and sell it and start up a certain business with the money

**I Number 7**

PG07 I support number 2

**I How do you support her?**

PG07 I support her in that as she has said she can buy an animal, I can also buy myself something, so that when I stop this work I can start with something

**I Count on something. Yes, number 1**

PG01 I can start a business in any case I stop that work, then I have something I have started.

**I This is something that you start before you stop sex work, right? we are talking of something that you can do before you stop so that when you stop, you would have already laid it on the ground. Yes, number 5**

PG05 I as number 5, I can use it to educate my children first, that’s when I can stop

**I Number 4**

PG04 I can use some and save some, so if I leave sex work I can still find money to start up some business (loud cough) because you cannot do two jobs at the same time. So if at all I leave it, then I can use the money I saved and if I want to relax, the money I saved let me start up a business with it.

**I Number 3**

PG03 I as number 3, I would love to buy land so in case I am leave sex work, I can find somewhere to live in as I do some business

**I Number 8**

PG08 I as number 8, I would love to buy a goat, so that when I stop sex work I can sell the goat and use the money

**I That’s fine. So for what we have said we would like to do before leaving sex work, is there anyone of you who has started preparing, has set a plan on the things… yes, number 6**

PG06 I have already started

**I You have started?**

PG06 I have a goat

**I How much did you purchase it for? How much did you buy it?**

PG06 I bought it a long time ago at three thousand two hundred shillings

**I So that one you have started slowly by slowly?**

PG06 Yes

**I Ehe, number 6 has started. number 2**

PG02 I also have a sheep, I bought this sheep for 36 (three thousand six hundred shillings)

**I Hundred?**

PG02 Yes, three thousand six hundred shillings

**I Number 2 has started. number 7**

PG07 I have also started I have 2 sheep with 2 lambs

**I The first one you bought at how much?**

PG07 The first one I bought it while the prices were still good, I bought it at 4

**I Four thousand shillings?**

PG07 Yes

**I Number 4**

PG04 I bought a chicken, and as for now they have multiplied to five. so I think by the time I leave this work, I think I would have bought a cow

**I You have started slowly by slowly with a chicken, right? Yes, number 1**

PG01 I also have a chicken

**I You have started with a chicken? One?**

PG01 Yes

**I Or has it already multiplied?**

PG01 It has multiplied

**I How many are they?**

PG01 Seven

**I (Slight laugh) number 3, have you started laying a plan?**

PG03 Yes, I have started. I have 5 chicken

**I 5 chicken. Yes, number 8**

PG08 I have also started

**I You started with?**

PG08 I have 3 chicken

**I 3 chicken. Who have I not asked? Number 5**

PG05 (laughter) I as number 5 I have started saving, I haven’t bought, but I plan to buy a goat

**I But you have started saving little by little?**

PG05 Yes

**I Ok. Good so we all have started with the plans that we have set, right?**

Chorus response: Yes

**I Ok. I think that as we finish with the last bit, is there any challenge that women who have stopped sex work face?**

R those who have stopped sex work?

**I Yes. Is there any challenge they face, you know when you stop you come back to the village, right? Is there any challenge? Yes, number 6**

PG06 Abuse you must be abused,

PG01 …you feel embarrassed…

PG06 …when you walk alone you have to hide your face

**I That’s number 1 who has supported number 6. Yes, number 5**

PG05 Let’s pretend that I had my parents and now am left with one parent, so when I leave work I come back home. Even if I am still working, the abuse is enough for my father even though he’s my father. That is why when I come back home… it will be abuse after abuse even if you slightly wronged someone, people can say that you are taking care of your father with sex work.

**I someone else? yes number 4**

PG04 There must be some challenge because in case you get married, and the sex work that you had been doing, those who might be interested in you, who can get your phone number, so that in case they want you they cannot call you any time and you go. So you know that when you have gotten married, your husband will not allow them to call you so that you can go there. So, there will be a challenge at that point. It can cause conflict.

**I In the house. Who has not said the final word? we are on the last words, we have finished. Yes, number 2**

PG02 The challenge that may arise is that I as a lady and am doing that work, it will reach that point in time that I will have to get my own place, so at my place there could be someone who wants to marry me and he hears that I was doing this work, it will be difficult for him to come and marry me because he will be told that I was doing that work.

**I So there are some stigma that comes with that work? sex work?**

PG02 Yes

**I Ok is there any question? (silence) the person sitting at the corner (laughter) ask me any question if any? question number 1?**

PG01 There is none

**I None, number 3 question?**

PG03 There is none

**I Number 6 question?**

PG06 None

**I Number 4?**

PG04 None

**I None. number 5?**

PG05 I have a question?

**I Ask**

PG05 Will you come back so that we get to learn?

**I As for now we will not come back because we said that this study we will do it only once as for now, because it has not yet started. It is something that has not started, we are just gathering thoughts so that when it is being formed, it will be made depending on the opinions that the participants have given us, right? But for today it is only once, we get to learn today and that’s it.**

PG05 Ok

**I Another question number 2?**

PG02 I have a question

**I Ask**

PG02 I also had a request, the question is can Jitegemee be made in a way that it can be as a group that can help young women? And the second one, I have a request, in my perspective I feel that it should be a group which can help people. It can be created as table banking so that if you are a lady and do not have, you can come and borrow some money

**I So if at all it is formed it should be created to look like table banking?**

PG02 Yes

**I That is a request you have made I cannot respond to your request, but we have captured it. Yes, number 7 question?**

PG07 I do not have

**I Number 8 question?**

PG08 None

**I You have no question. So if there are no other questions I want to say thank you so much right? I know that time you have taken to sit and those who are more tired than the others are many right? (Slight laugh), but I want to say thank you a lot. Ok so interview time ending at 6:40pm.**

**END**
