# Supplementary material for: Eat a little and save a little: A qualitative exploration of acceptability of a potential savings intervention to reduce HIV risk among female sex workers in Western Kenya
Source: PLoS One. 2024 Dec 19;19(12):e0310540. doi: 10.1371/journal.pone.0310540 (PMC11658496; doi:10.1371/journal.pone.0310540)
Supplement: S1 File — (ZIP) [file pone.0310540.s001.zip › Jitegemee Transcripts and Dissemination Notes for Journal/FGD H.docx]

**INTERVIEW DATE: 26^th^ APRIL 2022**

**FGD ID: FGD_H_ MAMBOLEO**

**NAME OF THE TRANSCRIBER: TRACY ONDU**

**INTERVIEWERS NAME: OLIVIA**

**NOTETAKERS NAME: PHILIP**

**TIME TAKEN: 2:18:08**

**CATEGORY: ABOVE 30 PERI-URBAN.**

**I: So this is FGDH, [posho mill sounds] the venue is Mamboleo, the date is 26^th^ April 2022 and the moderator is Olivia the note taker is Philip. So, as we begin I have already told you about *Jitegemee.* What comes to your mind after being told about that? *…* What can you say about *Jitegemee?* Number 5.**

PH05: I as number 5 I think that… when I hear about the word *Jitegemee,* it means that I don’t need anybody’s help, jitegemee already means that I myself. Yes, but now if it’s a group like we are in, Jitegemee as we have been told it’s supposed to… to help sex workers to save.

**I: Mhh, another person, what comes to your mind after hearing about *Jitegemee. Number 1.***

*PH01: A*s number 1 when I hear about the word jitegemee, I see it like being stable, uhh when we look at the financial sector mostly like us as sex workers must go (have sex) and depend on someone to come and pay us. But the way you have taught us, that jitegemee when we save for ourselves, everyone will have their own thing (their own money).

**I: Mhh another person with another opinion, number 7.**

PH07: I as number 7 I feel that this Jitegemee has come to help us, when you look at most of the girls that don’t have work to do, like a job we do and we get paid. So I feel it will help us a lot?

**I: Mhh okay, another person? Number 6.**

PH06: I as number 6 I think that according to the word *Jitegemee,* means that I will not depend on somebody to feed me, I will depend on myself to feed myself. Jitegemee the way It has come; it has come to help us as sex workers so that we learn how to save so that we stop depending on other people.

**I: Mhh, another person? … number, number 4.**

PH04: I as number 4, I’ve seen that, this depending on myself will help me of what I have gone through, that is why I have sat here. (that is why I have attended this).

**I: Okay, another person, number 2?**

PH02: I as number 2, I’ve seen that Jitegemee has taught me a lot of things: I should save and when God blesses us as we save we will develop ourselves and stop doing that job and then we will have opened our own accounts where we can save in them. So that they don’t misuse us a lot.

**I: Okay number 8.**

PH08: According to I as number 8, this Jitegemee I see it’s very important and it helps. So that we can stop doing sex work and depend on ourselves.

**I: Mhh, is there someone who wants to add on? [posho mill sounds] That posho mill is making a lot of noise so we will have to speak out louder so that it can record clearly.**

PH03: According to me as number 3, when I hear about the word Jitegemee, I see that this organization has come called jitegemee will help us as sexual workers so as to be able to get how to help ourselves.

**I: Okay. Is there anyone who would want to add on. Number 1.**

PH01: I as number 1, have sat in many of these meetings, different organizations, we have spoken a lot, but Jitegemee, I hope you will not be like the rest. You will help us to depend on ourselves. We are tired of sitting every day, we are given that stipend but the issues we say, we do not see where they are taken or what is being done. This jitegemee, if it will help us depend on ourselves then we will be very happy.

**I: Hmm… another person. … Anyone person who wants to speak. Is there an issue, number 9? Number 10? Okay. And the money sex workers get, when you work, let’s say the day before, you get that money, [coughing] what do you use that money for? Number 4.**

PH04: When we get that money, maybe you can go there [to do sex work], someone gives you even 500Ksh but if you go with it to the house, you may even buy food, you help the child that you have but it’s not by my liking that I go there. So, I have only gone there to look for what can help me. Mhh, so if you help us, we will be thankful.

**I: Okay. I want to stress on what I told you before, Jitegemee will only help to give ideas.**

NT: Opinions.

**I: Exactly, there’s no money you will be given, you will just be guided on how to start savings, you will be given information, be encouraged but any form of help regarding giving you money to save is not there**

R: We will save our own money.

**I: Exactly, ehe, number, who was the first to lift up their hands? Number 7.**

PH07: Okay, I as number 7, I was asking maybe sometimes you have been given that 500 Kshs, you depend on yourself in the house, how can I be saving from that 500 Ksh? Because you get that there is house rent that has to be paid, your child is going to school, eating is there, in that 500 Kshs, I should save how much?

**I: Mhhh, I will answer that later, for now, I just want you to talk about when you get your own money, how do you use it? Because sometimes the way you use it can prevent saving is that true?**

P: [All] Yes

**I: So, how do you use it (How do you use your money?). Number 4 has already explained to us, eh number 6.**

PH06: I as number 6, when I go to my sex work job, when I go back home, it first depends on how much work I managed to find on that day, if like that night I had gotten 1000 bob (1000Kshs), I will buy food, I will save for rent, I will take my children to school and the remaining one use it for my children’s upkeep bit by bit.

**I: Okay. So this (money), for food is for everyday or weekly or for how long?**

PH06: Food is for every day.

**I: Food is for everyday?**

PH06: Yes

**I: So averagely it can be like how much for food every day?**

PH06: Like for me in a day I use 500(Ksh).

**I: 500(Ksh) on food?**

PH06: Yeah

**I: Ehee. And that you were saying you save for rent?**

PH06: To save for rent, that’s why I have said it depends on the amount of money I earn that night. If I earn 1000 bob most likely I will save 300 (Ksh).

**I: Mhhm, 300Ksh for rent?**

PH06: Yeah, I will save 300 for rent and I will not keep it in my pockets. Either I will take it to a merry go round or I will put it in M-PESA as I wait to pay for rent.

**I: Okay. … Mhhh. Number 4, you spoke about food, but you did not tell us on food you use how, what amount of money you use on food?**

PH04: Okay, as for me food, you know breakfast the child must eat, lunch and at night (supper). Coz (because) now I can use something like 500 (Ksh), 6 (600 kshs), but getting money there is also hard. Maybe you have gone there and you have missed to get a client then you just come back.

**I: Mhh**

PH04: Maybe you have gone there and gotten even if it is that 500(kshs) of yours, when you come back with with it… it’s just, as in if you go there, it’s not a must we go there. That is what I was saying. Now it depends.

**I: Okay.**

PH04: The way you have earned.

**I: Soo, number 1.**

PH01: For the record, I just want to say this, 500 and 1000 (kshs) is not from one person, (one sex client) you can get that it is even from ten (sex clients) people who have paid 50, 50 (kshs). This money that we get, I as number 1, I want to say that, that money looks like it is from illuminati (cult). You go with it (the money) and it just gets lost. You even wonder what you did with it (the money). You can even get 3000 (Ksh), but when you reach the house you have paid the merry go round, you have brought food, like everything. You just here bills, bills bills. Everything just goes (all the money just goes). When it comes to food, I like buying what I don’t have, if its salt I buy the big pack that goes for 70 (kshs), I know that will take me for 4 months. I buy sugar, the one that is brought in portions. Like now as we speak what I don’t have is body oil and I know that by the time I leave work in the evening, I will come back with it.

**I: Mhh okay. So on average you have said that on food you use 500(Kshs).**

R: Yes

**I: And it is every day?**

R: Yes.

**I: And are there some things that you buy like weekly? Once a week?**

R: Yes

**I: Like what? [Cross talk]…**

PH01: Lubricant

**I: Number 1 is saying lubricant. Another one, once a week? (What else do you buy once a week). …**

PH10: Maize flour.

**I: Number 10 is saying flour, once a week, how much money do you use to buy flour?**

PH10: 300(Kshs).

**I: And lubricant?**

PH01: As for lubricant I buy a box worth (500kshs) per week that carries 10.

**I: Anything you buy weekly again? There is someone who wanted to speak.**

PH09: It is the flour

**I: Flour, you buy for the week? Number 9, and how much money do you use?**

PH09: 300(Ksh).

**I: 300(Ksh) for the week, ehee Number 2?**

PH02: Salad oil one litter.

**I: That’s enough for you for the whole week?**

PH02: Mhh.

**I: Okay, number 5?**

R: I can say that some are not the things we buy; they can be services I say we render. You have a child going to school, every day they need the fare. So everyday it’s on the budget let us say there is a day when we lack that money. We really get depressed over that. Here is a child who is supposed to go to school, he/ she did not apply for you give birth to them. They need lunch because lunch time finds them still at school, they need fare. So it is something that you have to do every day. And let me tell you there are days when work gets tough. There is no money.

**I: Okay. So averagely that 500(Kshs) may go up? Because you talked about food, you did not include information about your children.**

R: Yes

**I: Okay, so weekly you have already mentioned lubricant, flour, [crosstalk]**

P: Our children going to school.

**I: Is it also paid weekly? [Laugher from respondents] [Crosstalk].**

P: Snacks, fare, we groups these things with the daily needs

PH06: I as number 6, I pay for the fare of my children weekly.

**I: Oh, weekly fare for your children can be like how much?**

PH06: I pay like 2,300(Kshs).

**I: Mhh. And are there things you do monthly? That you buy monthly? [Crosstalk]**

P: [All} Yes.

**I: Like? [Cross talk].**

PH01 Rent, NHIF, merry go round’.

**I: That is number 1, when you mention rent, you say how much it is.**

PH01: I as number 1, rent I pay 6,500(Kshs), Wi-Fi 1,500 (Ksh), Merry go round 2000(Ksh), NHIF 1000(ksh), because I am paying for my old mother who is at home.

**I: Mhh, that is monthly, Number 6 wanted to speak.**

PH06: I as number 6, I pay my monthly rent at 5500(Kshs), NHIF 500, I do shopping monthly, I pay school fees monthly, the total school fees for my children when I calculate it, I pay 6800(kshs).

**I: Monthly?**

PH06: Yeah.

**I: And the monthly shopping?**

PH06: For the monthly shopping, I do for 4000(ksh).

**I: Okay. Ehe, number 4?**

PH04: I as number 4 I pay rent for 3000(ksh) and … how is it called, okay, I pay rent for 3000(ksh), and then I pay Go TV 800 (ksh).

**I: Mhh.**

PH04: Just like that.

**I: Okay, so monthly you have rent and? School fees you always pay.**

PH04: About school fees, I have a young child and a child in standard 8.

**I: Okay.**

PH04: Currently, the young one has not started school.

**I: Mhh. what about the older child?**

PH04: The older one is currently in class 8

**I: Is it you paying for him?**

PH04: I am the one who takes care of him.

**I: Okay, number 5?**

PH05: I as number 5, monthly I have house rent which is 6000(ksh), I have remedial which I pay to school every month, which is 1000(kshs), when it reaches end month, it finds the child is in school. I am still going back to the usual things we have the Childs fare to school food to eat in school. I usually prefer to do my shopping at end month, so if anything depletes here in the middle that’s when I will be able to buy. So I usually like to do shopping for let’s say 4500(Ksh).

**I: Mhh.**

PH05: So even when I keep these stock, the one that will be depleted in the middle of the month you just find a way of buying it then.

**I: Mhh, okay. Number 3 wanted to speak.**

PH03: I as number 3, I have two children and brothers, one is(brother) in form 3 I pay for him school fee like 3000(ksh) I pay per month, the other one in class 8 I pay for him 600(ksh). That is money for lunch. So, that’s like monthly I have pay 3600(ksh).

**I: Mhh**

PH03: Yeah.

**I: And daily?**

PH03: Daily I use 500(ksh).

**I: Okay.**

PH03: Yeah.

**I: Is there anyone who wants to add on that? … Yu have nothing more to say? … Okay. And this money? The money we use, where does it come from? [Sounds of a dog barking]. All of it comes from sex work or there is somewhere else we get it from? Number 2.**

PH02: I as number 2, to answer that, that money comes from sex work because there is no other work we can do. Because if you go to do work at a hotel, the money there is little, you cannot budget with in the house. Now when you go to sex work, u will earn even 500(ksh), sometimes 1000(ksh) which can help you.

**I: Mhh.**

PH02: Because that is what we depend on. We don’t have any other thing we depend on.

**I: Therefore, good money comes from sex work**

R: Yes

**I: And what of from these other small small jobs? Like you, you have said at times which one do you do?**

PH02: In a hotel

**I: At the hotel.**

PH02: And the money is little that it can’t help you

**I: So, the hotel always gives you (money) weekly or daily or monthly?**

PH02: Daily.

**I: Daily it’s how much)?**

PH02: 200 (ksh).

**I: Oh, 200(ksh) .**

PH02: Mhh.

**I: And you work for 30 days?**

PH02: Yes.

**I: Or you skip other days?**

PH02: Some days I skip like on Sunday’s, I can skip but you still get that the money is not sufficient enough.

**I: Okay. Another one, ehe, number 1?**

PH01: I opened a shop, all the women were not coming to my shop and they stopped their husbands from coming to my shop because I am a sex worker. So I stopped it, (I closed my shop) and ventured fully into sex work. Because I encountered a lot of losses: the bread expires, kdf(donuts) expires because they are not coming to buy, they say that you are going to steal their husbands.

**I: Mhh.**

R: Now, is it this sex worker?

**I: Okay.**

PH01: Then when you look at it, a shop has profit of only 2 shilling. And on this other side, you don’t even purchase. [Respondents laughing], [cross talk just put ‘’put your mouth hear’’]. As in you don’t even go to the whole sale to purchase. Already you have that river kunja (referring to the vagina). Now you, you just look for someone, you just call someone ‘pss pss’ and they come [referring to how they call a client for a sex]. [Respondents laughing] you don’t even experience any loss. Even if that day there is no one who has come, you don’t have any loss, you carry your property and return it home, the next day you take it there again [participants laughing.

**I: Okay, that was number 1, [respondents giggling in the background] another person? Number 6?**

PH06: I as number 6[coughing sounds], most of my bills are paid through sex work.

**I: Mhh.**

PH06: Even when I go the saloon (when I go to work at the saloon), salon you can even stay there a whole week without someone asking you if you are selling something, no one, and you are seated there the whole week.

**I: Mhh.**

PH06: Sometimes it is after two weeks that you can even get a client at the saloon. Now [ dog barking sounds]. Now mostly of the money I use comes from sex work.

**I: [Sounds of a dog barking] Okay. And do you own a saloon yourself?**

PH06: I work for someone.

**I: You work for someone. So how are you paid over there?**

PH06: Commission

**I: Oh, commission when you get a client?**

PH06: Yes.

**I: Okay. Averagely in a week, the saloon can give you how much money?**

PH06: Sometimes a week can end without me getting any money.

**I: okay.**

PH06: Or sometimes you may get 500(ksh) in a week.

**I: Mhh. [inaudible talk, murmuring and giggling in the background]. Ehee, another person … I had asked if this money you use to offset bills comes from sex work, was a chorus (to mean a unanimous response). It is like that, but you said there are these small small jobs. She has mentioned of a saloon, she has mentioned of a shop, which she left, she has mentioned of a hotel. Another person?**

PH04: I’m number 4?

**I: Eeh.**

PH04: I work at a bar here.

**I: Mhh.**

PH04: Now I said let me go and work at the bar, I get something small (meaning money), when it reaches my time for going to look for good money that can actually help me, I will go..

**I: Okay**

PH04: Now here at the bar, we are paid per day 200(ksh).

**I: Okay.**

PH04: Mhh.

**I: Mhh number 5?**

PH05: I as number 5, I can still speak on my side saying that this money from sex work is much better than these others, because it comes easy. Okay it is not easy, we cry. Let me not say easy per se, rather I can say it comes in bulk compared to these others. I have tried a few small small businesses’ like selling tomatoes, onions, you get that sometimes fine people will eat, but the little amount you get from there when you compare with this other one (to mean money from sex work). This one here is at least. You have heard the bills we have been mentioning, when you look averagely everyone has spoken of a bill of over 10,000(ksh). [Cough sounds]. There when we stand in the cold, we know that this cold of ours and what is written there just brings for us money. So these side ones (to mean other jobs aside from sex work) me I can say, I have tried one or two but this other one brings more money.

**I: Okay**

PH04: I have a question, [sounds of a dog barking],

**I: Mhh.**

PH04: Do you know I can pay rent with sex work?

**I: [Dog barking] Yes I know.**

PH04: You go to your landlord, you help him (to mean have sex with him) and then he helps you’’ (in exchange for rent). [Respondents laughing].

**I: Oh! Okay [respondents laughing] [cross talk]**

PH05: What about if it’s a female, how will she help you?

PH04: She has sons help you?’’ [Cross talk].

P: Only few of them are female

PH04: There are females (land ladies), but most of them are male. And when the landlord has already known that you do sex work then you are set. Your electricity can get damaged, or even any other thing, and he will come to fix it. And when he has fixed it, you also fix him (to mean have sex with him) [dog barking] just like that will have paid him.

**I: [Respondents laughing] Okay. So that way you pay him once (to mean you have sex with him once) and the will have been paid.**

R: [ALL} Yes”

**I: Or is it every time he comes? [Dog barking]**

R: Well you increase the price for him.

**I: Owh, okay. [Respondents giggling].**

PH04: For me since the time of CORONA heh!, life became so hard. We just sat down together with him (to mean the landlord) and I told him there is no need for us to go under the lorry, he should just come to my house where it’s not cold. [Respondents giggling in the background].

**I: Okay, alright, is there another person who wants to add on? Where does our money come from [dog barking], the money we use, where does it come from? You have already told me sex work is the major source, those others are small small.**

R: [All] Yes

**I: Mhh, number 9.**

PH07: It is to blind people’s eyes; me I can say it’s to blind people’s eyes so that they may not judge you. They judge us, its work. So, me I can say, this side one (to mean work other than sex work) is just so that someone can see oh! She always sells tomatoes and onions, but for us we know that the tomatoes and onions we sell are our own tomatoes and onions (to mean selling their bodies).

PH04: River kuja [their sexual organ].

PH07: That is it now.

**I: Okay, number 9, do you have a point?**

PH09: Okay me I can say as number 9.

**I: Explain even in Dholuo. [Respondents laughing].**

PH09: The earning from sex work is good, but it has its disadvantage.

**I: Mhh.**

PH09: His wife may get you and beat you. [Sounds of a cow mooing in the background].

**I: Mhh. It is good as in? It is good in what way?**

PH09: You may even be found here in =Mamboleo= or as even go to= Kondele= or you even go to town.

**I: Mhh [cow mooing]. Okay. Number 10 do you want to add on that?**

PH10: [cow mooing] ehh, I can add a little. I feel Sex work is good because sometimes you go, and someone has employed you at the hotel for 150(ksh) and if you compare with this sex work, you cannot compare because the money you earn from sex work is more than this one [What you are paid at the hotel].

**I: Okay.**

PH10: Because you may go (participant means you may go to sex work) and per day you even come back with 3000(ksh) or 2000(ksh). So you cannot compare with [cow mooing] with 150(ksh).

**I: Now, you have already told me where and what you use that money on, what are the reasons you use that money on the things you have mentioned? … [Respondents giggling] numbers, number 1 have you understood that question?**

R1: Eeh. [Crosstalk] [Participants walking at the same time] We have not understood.

**I: That money, now let me say, let me say it in both Kiswahili and Luo, the money you get, there is a way you use it. There are some who have to buy lubricant every week while some do not buy. Some have to buy lunch for their children while some do not buy. So there is a way that someone decides this (money) I am going to use on this and that? What reason makes you decide which one you are going to use where and when? Mhh number 5?**

PH05: I as number 5. I can say that (coughing), it is the responsibilities that push you, whether you want it or not. There are things you have to do monthly. We are human beings just like any other. The difference is that we are called sex workers. We are human beings just like others and we have, we have to eat, [coughs] we have to pay for water, pay for the house, pay for other things. That is why the money goes into those things, whether you want it or not.

**I: Okay. Ehe, number 1?**

PH01: I as number 1, I can say that most of us have children [coughs], and most of us are single parents and there are some of us who are taking care of our siblings not our children. Now if you do not step out and go and look for that job or our job of sex work. There is nobody who is going to come and pay for you that rent, because even if he pays you that rent even you will have to pay him. You cannot sit in the house, and your child is seated at home, and the child has not started school because you don’t have money. Now, people have attained education, in Kenya everyone is educated. The problem is getting a job. When you go for a job interview, you find everyone with their documents, but the person who will get that job is not even in that line. That is why with us, our bills you pay, you look at it like, from the most important you pay going down like that to the least important. Something like rent and school fee is on the top, food follows closely and then lubricants. Also clothes, you know you also have to be smart.

**I: Mhh.**

PH01: The way you see us like this, when you come those other times (to mean sex work hours), you will not even be able to recognize us [respondents laughing]. It is a must!

**I: I did not hear you telling me that you always apply make up? [Cross talk]**

P: [All] that is a must

PH01 That one is a must, you must not even be told about it [slight murmuring]

**I: I asked, this money, what do you use it for?**

PH04: We know that beauty is a must, there are people who will come to you and say, “No, today you are smelling bad.” After having sex you go and clean yourself even if it is brushing you brush before you go back and stand where you wait for your clients. There is someone who will also come and open your mouth to see how your mouth looks like.

**I: Okay, [posho mill sounds] there is a way you also spend money on makeup and you did not tell me.**

PH01: There are still so many things we have not told you about but we will tell you as continue.

**I: Okay, so averagely how much money do you use on make up? Number 1?**

PH01: like me, now you see I have this pimple here,[posho mill sounds] I tried, I cannot mention the name right? … [Slight giggling]

**I: Somebody’s name.**

PH01: A-ah the name of that product.

**I: You can just mention.**

PH01: Now you see last week we were called to a certain seminar similar to this one, and we were given makeup but we were paying like *3k 3k*(3000ksh) but they were worth over 10k (10,000ksh). In which that 3000(ksh) was just because the prices were reduced, they wanted to see the effects. That’s why were given. You see me it has caused a pimple to pop here? But they have said they will pay me. So in a week, like I buy, when it gets finished I buy, when it gets finished I buy a new set. But after 3 months I change everything even the lipstick, as in everything. I go to =Dingua= in Nairobi

**I: Mhh.**

PH01: So when I go there I can use like 3k (3000kshs), 3000 for transport, 1500(ksh) for travelling there 1500(ksh) when coming back. Then when I go for that shopping, it depends on which brands I would have bought.

**I: Mhh.**

PH01: You know there are some of our clients who know those brands, if you come with one that is *Chinese Chinese* like this [to mean low quality product] you lose them.

**I: Okay. So you go with a fare of 3000(ksh) and you buy with how much?**

PH01: Even 20(to mean 20, 000 Ksh). It depends

**I: 20,000 (Ksh).**

PH01: Eehh.

**I: And you use it for how long? [It to mean the makeup].**

R: About 3 months, when it is finished you dispose and get another one.

**I: Okay.**

R: Because it is that that brings money.

**I: Mhh. And number 6 you also wanted to speak?**

PH06: The point I wanted to say, she has already said. [Respondents laughing].

**I: Number 5 also wanted to speak. [Coughs sounds]**

PH05: Maybe I am even kind of confused, but I wanted to say that we do apply makeup, just the way she has said. You cannot stand there… our target is men let us not lie, men. You cannot go there looking like a man, one fact is that I have shaved my hair, so I have to pimp my face to look good. Because someone (to mean a sex client) will come and tell you ‘’where I am taking a fellow man with me.’’ You have to be… Ehh… and we always look smart. Just the way she has explained it to you. When you come [when we are at the hot spots], we are totally different people.

PH01: We have to look good so as to hide these pimples we were given for free.

**I: So she has told me [to mean respondent 5] the amount of money she spends on makeup. And it is after 3 months right? How about others.**

PH05: I as number 5 I can say that maybe I use the same products, and when it is over is when I can re-do it. You have to have deodorant; you have to smell nice. You have to do your face with a lot of things (to mean you have to make up your face with a lot of products). There are a lot of layers (of makeup) you put on your face, we have to use lipstick; I do not use the highest quality because it’s a bit more costly as compared to my other needs. If its lipstick, I use one that’s in the middle (that is affordable).

**I: Mhh.**

PH05: If it is matte (a brand of lipstick), they are sold at a fairly good price. I cannot buy like the one for *huddah (*lipstick brand name). There is *huddah* for us as common citizens, it is even branded as huddah, so when she uses *huddah* I also have my own fake *huddah. (Lipstick brand name).* We will all look the same, the difference is that mine (to mean the lipstick) will remain on the glass when I sip even if its wine. But the bottom line is so long as I look smart just like the other person who uses the other huddah.

**I: So averagely, [respondents giggling] how much do you spend?**

PH05: Averagely I have to have lipsticks which I can change shades; maybe four shades. Maybe I have applied purple today, tomorrow I have red, and I have maroon. Uhuh, one of my lipsticks costs around 250(ksh); so for the four I will spend about 1000(ksh) [interruption by interviewer].

**I: You use them for how long?**

PH05: To push me? I can’t really say maybe 2 to 4 months. Lipstick is something you apply only for that night, and you do not keep having to re-apply, so it can push me for about 4 months. Maybe I had forgotten number 5 just on the same, you get that people look so different; of cause like us… there are friends… the places where we are, we have friends to talk to. There isn’t any rivalry; business everyone will get depending on the day. You get that there are people who even put on these, there a lot of things. Maybe it may not even be us. Now I am no speaking on my behalf, there are people who put on these biker hips (to mean padded hip enhancers).

**I: Mhh.**

PH05: The motive is to attract. That is our motive in the market, to attract.

**I: Okay.**

PH05: You find someone in the padded biker shorts, the time she has gone with him [sex client]; he has already erected. Maybe he may not appreciate the after product but if the ‘ass’ is what he wanted then he will get that plastic one, we shall deal with it ahead.

**I: Mhh.**

PH05: Hmm. [respondents giggling}.

**I: When you compare the money you use for makeup with the money you use on your other needs you had mentioned, which one is higher? [Crosstalk ‘’makeup’’.] [Respondents laughing, inaudible information]. You say I as number 1. [Cross talk ‘’number 2’’. [Giggling in the background].**

PH02: I as number 1

P: You are number 1 [Laughs]

PH02: No number 2].

**I: Mhh.**

PH02: I can say that makeup is on the higher side because you see, if I do not use them, I will not get that money.

**I: Mhh.**

PH02: So I have to buy them even if they are expensive. I will just have to take them.

**I: Okay. Can you buy it [makeup] and miss to eat? Because the money is less**

PH02: Okay I will not fail to eat because I know that when I use it (make up) I will go there and get money; I have applied makeup I go there and get clients, I know that I will get money which I will go back with in the house.

**I: Mhh. Okay. Number 6?**

PH06: I as number 6 mostly I use makeup that is more expensive than other bills. why? There are different clients on the ground. When you go looking cheap, you get a cheap client. You look expensive you get an expensive client.

**I: Mhh. Okay. … Number 1 you want to add?**

PH01: No, I just want to say something fresh [giggling in the background]. I as number 1, in the past my makeup bills were more expensive than other bills, but currently other bills are higher than makeup costs because; make up I buy after 3 months but bills I pay monthly. Like right now I have a six-month old child, I take her to daycare; I take her at 6.00 am in the morning and I pick her up at 11.00 pm at night. I pay 2k (2000ksh).

**I: Mhh.**

PH01: It’s even as if it is like boarding, you can leave her (to mean her child) there and go for her the next day. But that 2k (2000ksh) is constant. It is located up here (the day care). Now, about clients there; clients, makeup and bills. Now about clients, like recently we attended a wedding of a governor’s child somewhere on the hills. You have to dress up! I had to hire a dress, you even hire a dress worth 5k (5000ksh). There are shoes, with makeup, you cannot just go the way you look and you have already worn a nice dress. You will just enter at =United mall= and get beaten on a face mask (to mean get professional makeup) and then you go. Between the time I leave the house and the time I leave the wedding, when you look at the money that I have used, it’s like 20k (20,000ksh). And there is no way it has even helped me. But when I go to the wedding, ‘’hi hi’’, (to mean greeting potential clients) like you try to interact with all those people. When asked who you are, you can even say you are the governor of Bomet’s daughter; just as long as you are given their phone number. [Respondents laughing] when you have already been given a phone number (to men when one of the men has given her his phone number) you go and sit down. You would have already established who and who is the target. Then you call them, [child crying in the background] the money that you used to go there comes back even 10 times.

**I: Okay.**

PH01: Because there is a friend of mine, a day before; she is not here, she is still there. She was called to be given a piece of land and told to choose which one she wants; this or that one? She said that one. Then he [the client] went and paid for it and now, she already has a title deed.

**I: Mhh.**

PH01: It now depend on which clients you go for.

**I: Mhh. [sounds of a child crying in the background]. And when you people think about it, do sex workers save money? … mhh number 5?**

PH05: I want to imagine in this forum we are supposed to be talking about us right? Now, I don’t know about anybody else out here, so me as number 5, I can say that savings is hard, needs, like the money is packed [has needs to sort] very very much and every day comes with its own issues. Everyday prices of things go up; everyday money enters as it goes out.

**I: Mhh**

PH05: The way we had said [coughs] it (money) enters as it goes out, there is nothing to save.

**I: There is nothing to save? Mhh. Are there some who save? Ehe number 4?**

PH04: I as number 4, when I go out there, and come back with that small thing of yours you have earned (to mean the little money you have gotten). When you go with in the house, there is nothing to save.

**I: Mhh.**

PH04: Because you will find that this thing is not there, that is not there also. When you look at it, there is no saving. [Coughing] there isn’t.

**I: Mhh. I have heard someone speaking on this side. … Number 6.**

PH06: I as number 6, in regards to how life is right now, sex workers cannot save. Because even those clients have *squeezed their pockets [to mean have reduced the money they give out].*

**I: Mhh.**

PH06: Because life is expensive. So the little that we get from there by end month there is nothing to save.

**I: Okay Number 1.**

PH01: I as number 1, I want to say that the way I said that this money looks like it’s from illuminati; it enters and leaves as in it just visits you. ‘’Money is a visitor, it visits every person, it visits you as it moves forward.” As in, you know, maybe today I have earned 2000(ksh) its fine, tomorrow I may go and get or miss it. It is like you are not sure whether you are going to get it or not, not like a scenario where you wait for like for example 1000(ksh) at the end of the month. Now like saving becomes a little bit hard. I as number 1,

**I: Mhh.**

PH01: I normally save in the merry go round. In that merry go round I only pay 200(ksh) per day.

**I: Mhh.**

PH01: That is the only savings I have and it goes the whole year, when it reaches December we hold a meeting and we are each given the money you have saved for the whole year. Now that way is easy, but the kind of savings in which you are just seated and you keep it in a savings box or in your bank account or even Mshwari (mobile money app); that is not there, it is really difficult.

**I: Now you have said that you are saving in a chama [saving group],**

PH01: Mhh.

**I: And it is 200bob (shillings) daily?**

PH01: Eeh.

**I: What do you intend to do with that money? The money you are saving.**

PH01: The one I am getting right now? I want to go to Konshens (musician) concert that will be in Mombasa. I will be going to look for clients there.

**I: Mhm. Who else is saving? Number 7.**

PH07: I as number 7, I don’t have a place to save.

**I: Mhh.**

PH07: And when I get money, it all goes to household. It all just gets finished.

**I: Mhm. Number 10 [coughing]. Do you usually save?**

PH10: No, I don’t usually save.

**I: Okay... But do you know of any of your fellow sex workers who saves?**

PH10: Eeh. [Mentions a name]

**I: Ooh! [Name mentioned] already told us.**

PH10: Well you had said no mentioning names? [Laughing]

**I: Eeh! No calling anyone by name. [Respondents laughing] So is there anyone who wants to speak? So here, to be honest there is not even one in a saving group? There is no one even in a chama [saving group… [Slight murmuring in the audience] Mhh number 1 is in a merry –go-round.**

PH04: Number 4, I am in a chama [saving group]

**I: Mhh number 4, is in a saving group, number 5?**

PH05: I am in a chama

**I: You are in a merry-go-round, number 2 is also in one. That saving groups are also a way to save, well its money you have set aside somewhere right?**

R; [All] Yes.

**I: It is what I am asking, this money that you have set aside in the chamas [saving groups] number 1 has said that she puts it daily (to mean she saves daily), Right? But it is for going to a concert in =Mombasa= to search for a client**

PH01: Mhh.

**I: what about the rest? Are you in a merry-go-round? Number 5?**

PH05: I am in a saving group. Let’s say like mine (to mean her money) give me till June, we said that this business is one that you may earn from or may not, May be today you go to work and you are in the cold and have not gotten anything. Let us say it is end month, and you had not gotten [inaudible information]. And most of the time you find that you get if not weekly then at the end of the month; leave alone the one for yearly. [Posho mill sounds]. Weekly or end month. When it reaches and you did not have rent what will you do? Isn’t that not going to be your rent? Then you will include shopping, remember we had told you we have monthly bills?

**I: Mhh.**

PH05: We have weekly bills, [coughing] so let us say with this money you wanted to save to even buy a plot; there is nowhere… it still goes back to these daily use.

**I: Okay. Do you often contribute daily (save money daily) or … [interruption by participant].**

PH05: We remove monthly, 2000(ksh).

**I: Mhh. Okay. Number 4 has also said she is in a saving group**

PH04: I am in a saving group. Mine is paid per week, 500 (ksh).

**I: Mhh.**

PH04: Now, every week when I get something like even 5000(kshs); this 5000(ksh) will go to household needs.

**I: Okay.it just goes back to consumption [interruption by respondents].**

PH06: Yes.

**I: Number 6?**

PH06: Okay I as number 6, I am in a saving group, it is weekly (to mean contributions are done weekly) but we distribute the money by end of the year. The purpose of joining a saving group, everyone has a different purpose for joining one. I joined that saving group with a certain target, at the end of the month I have specific target. Then in that group we can take loans, we have children we are taking; like now I have a child joining form 1. It forces me to go to that group, take a loa to take my child to school then start paying slowly by slowly. By the time it reaches end of the year, and I have not finished paying, I will be deducted a bit and then remain with a bit from my savings. That little amount I remain with then goes into other bills.

**I: Okay. Number 8{[coughs]**

PH08: I as number 8, I am in saving group where we pay 100(ksh) every day. And we are about 10 people, now when I get that 3000(ksh), it just goes into household needs.

**I: Okay, it just goes into household needs. And number 9?**

PH09: I as number 9, I do not have any saving group.

**I: Is there anywhere else you save in aside from a merry go round?**

PH09: I save in my stomach [respondents laughing].

**I: okay. Any other person again? … Number 10. You told me, you just know of someone that saves, [slight giggling].**

PH10: number 1

**I: Okay, so these people who save, I will ask again in Swahili; these people who save, usually have what kind of behavior? Those who save usually have what sort of characteristics? Mhh?**

PH10: Ask in Dholuo? [Respondents laughing],

**I: Those who save usually have which kind of characteristics? People who save or sex works who save? Hmm some people have spoken too much, that one is number? Number 3.**

PH03: Now I as number 3, those who save a lot, must be those who its very hard to see their money [they don’t spend their money easily] for you see it, you have licked them [you have pleaded]

**I: Mhh.**

PH03: For you to see their money, you may go and tell them “My friend I have a certain problem and I want you to help me with a certain amount of money.” He will tell you that he doesn’t have. He doesn’t have but she has because you may have worked with her the previous day, and she earned something and you didn’t. Then you tell her please lend me some money and when it reaches evening I will repay you She then tells you she does not have, just know that she used part of the money and saved the rest.

**I: Okay.**

PH03: So she just has to be a selfish person.

**I: Okay, someone who saves are people who are selfish. [Respondents laughing] Ehe number 5?**

PH05: Those who save, are lovers of good life. I can give an example number 1, you heard that she is saving because she wants to go where? To relax her body. That time that she has gone to konshens concert; as much as she wants to collect phone numbers, she is going to relax.

**I: Mmh.**

PH05: For me, when I am saving, I’m saving toward… I saw a nice fridge. It does not mean that as sex workers do not live a good life

**I: Ye…**

PH05: We try. Because we are just human beings, it (sex workers) is just a name. You have seen a nice fridge you would want to buy, but what usually goes wrong for us is that it usually finds when we still have bills to pay otherwise, we are lovers of good life.

**I: Those who save are lovers of good life. Ehe, number 6.**

PH06: Okay, I as number 6, those who save money or those who keep savings. Number one is someone who is an economist. She is an economist. She is someone who has done economics and accounts, anything he/she does they record.

**I: Mhh.**

PH06: Anything she/he does they record, anything, any amount that is used is recorded. So that when it reaches end of the week and he/she is doing their savings then they know this week I used this the other week I used that.

**I: Mmh.**

PH06: Secondly, somebody like me; I love good life, I love the high life I love a relaxed life. So I might save because I as number 6, I take all my clients to my house,

**I: Mhh.**

PH06: So when they come to my house and see how I live it forces them to enlarge their pockets [meaning to pay more]. So I save towards furnishing my house.

**I: Mhh Okay. Number 1 you have talked lot. I want somebody who has not talked. Number 7. [Posho mills sounds] those who save have behaviors like which ones?**

PH07: They are mean, when you borrow from them they do not give you, they want to save [respondents laughing].

**I: Number 10?**

PH10: The must be mean to give out. Even me you have taught me that I will also be hard towards giving and I save.

**I: Number 10 does not have anything, number 9? Those who save have what kind of behavior?**

PH09: Those who save are mean. [Respondents laughing] Sometimes you ask them for money and they want to go and save the money, so I will also start saving [respondents laughing].

**I: Mhh. Number 1?**

R1: I as number 1, as one of the people who save, I would like to say that those of us who save are people who have looked ahead and have already calculated. We have already started the process of depending on ourselves even before you came as *Jitegemee. We* are soft life ambassadors (to mean lovers of good life) the way they have said. It’s just that sometimes life hits us here and there but we try, just as number 5 has said.

**I: Okay number 8, those who save usually have what kind of characteristics? [Respondents giggling]. …**

PH08: It is just the way she has said.

**I: How about those who save? I mean those who do not save? They have what kind of characteristics? Number 7?**

PH07: They have a sweet mouth [Love eating sweet things][respondents laughing]

**I: Ehee, number 6?**

PH06: I as number 6, those who don’t save are people who do not have a focus in life, they have not looked ahead, they just see around where they are seated.

**I: Mhh. Number 2?**

PH02: I as number 2, those who do not save are those who have a sweet mouth [love eating sweet things], are people who don’t want to look ahead [are not focused] and have not passed through tough trials. So they just feel that it is better to just eat, they have not gone through the hurdles that we go through or even think ahead that there are children whom they will have to educate or they may have to do something else.

**I: Mhh. Number 5?**

PH05: Those who do not save are those people who leave with the mentality that ‘’we live for now’’ they forget there is tomorrow [giggling].

**I: Mhh. Number 3, those who do save are people whose characteristics look like what?**

PH03: They are people who love their stomachs.

**I: Mhh. Number 6 wants to add.**

PH06: I want to say something as number 6, those who don’t save are people who live the kind of life like thinking that they will also earn tomorrow, tomorrow I will earn..

**I: Mhh. Number 1**

PH01: I as number 1, I want to say that those who do not save are very very lazy and they love food; it is mostly food that finishes their money. Someone wants to eat pizza in the morning, to eat humbugger in the evening they do not even want to taste porridge and *githeri* (a local Kenyan food) they just want soft life and they do not see that they are supposed to save.

**I: Which kind of people are these?**

PH01: Those who do not save?

**I: There are like who?**

PH01: I do not want to mention the number [respondents giggling] it is in the recorder.

PH07: I as number 7, sometimes you can tell the person who does not save money mhh, and you people are saving. Like me when I came to =Kisumu= I joined a certain saving group, I am a Kisii (a Kenyan tribe) you people are Luo’s, but when it reaches a time when I want to withdraw all my cash, I do not get it all. So for me as number 7 I got tired of saving, so it is better to save in M-pesa, when I go broke, I again withdraw and eat. You don’t know a razor blade [Cross talk]

**I: Number 9.**

PH09: I as number 9, number 1 has told us that people who do not save love eating food. She also loves food, every one of us loves food. Sometimes you get so little that you find there is no way that you cannot save. But sometimes someone who don’t save, lives a better life than someone who saves.

**I: Alright, Number 4. [Coughing].**

PH04: I as number 4, [coughing] you know that people earn differently .so there is no way you can tell someone to save. Maybe there is no way she can save, she wants to eat, she wants to help someone, so there is no way to save.

**I: So number …**

PH04: number 4.

**I: But sex workers who save, how do they save? What makes it easy for them to save? Number 2.**

PH02: I as number 2, I save because I have children. I want to look at their future tomorrow. So, Incase my job gets stuck, and they want to go to school, I can now go into my account or to the saving group and ask for a loan and take them to school.

**I: Mhh. Number 4**

PH04: I as number 4, as per now, I have to save because next year I have a child joining form one. So I must deny myself and save to help my child.

**I: Okay that is number 4, number 6?**

PH06: Okay I as number 6, I save because; of one, my children going to school, number two; I will not do sex work forever, there is a point where my body will get tired and it will want me to do something else while seated.

**I: Mhh. Number 1?**

PH01: I as number one, I save because I love soft life and I cannot afford it at a once, so I have to deny myself bit by bit so that I can afford after sometimes. Then to add on to another point, not everyone that saves has extra (money) to save. So as to save you have to deny yourself; you say ehh” I really wanted a dress for 1000(ksh) but let me buy the one for 800(ksh) and save 200Ksh. Not that someone saves because they have a lot. If you have a lot, you cannot save. You use it because you have it.

**I: Mhh. What makes it easy to save? Number 8? … Even if it is just putting it in the saving group..**

PH08: I as number 8, I have a child so I have to save, so as to even get his/her; uniform, carter for his/her needs.

**I: Mhh.**

PH08: Even I myself, I may ran short of something sometimes and take from there and use.

**I: Okay. What makes savings become easy for those who save? Number 6.**

PH06: I as number 6, when it reaches 31^st^ December, I draw a plan for the next year. 31^st^ December at midnight. I draw a plan for the next year that is what makes me save. I set a target for the next year.

**I: Mhh. Number 1 told us, what makes savings become easy, is denying yourself. Number 2 says what makes savings become easy is to have a target. Ehe, who else. Number 5?**

PH05: In my life I can say that my biggest motivator is my child, that motivation to make sure that my child lives a good life is what makes me save.

**I: And what challenges do we have when saving? Challenges.**

PH01: Desire! As number 1, desire! Let us say you have gone to work and have come back with 400(ksh), per day you have to put 200 aside, you have already set aside this 200(khs). But when you just look like this, desire, you just start longing for something; let us say it’s even a phone cover or even screen protector. There is just something that as a human being you really long for. You just say that let me just not save today, let me buy this. And you know when you have already started missing one day, you have just already started failing; you will miss the first second day, third day just like that.

**I: Okay. Desire! Quite a big challenge, another one? Another challenge? Number 5.**

PH05: Less income than your need/responsibilities. Ah-ah, lower income than your responsibilities. You find that you have saved the money is there and there are bills. Who are you going to cry to? Because that is your money and these are your bills. Just like that you end up withdrawing (your savings) and you had a target, you end up withdrawing and it takes you back to step 1.Your door will not be shut and you have savings (due to failure to pay rent) and you have savings stored somewhere. Your child will not fail to go to school money for fare and lunch while you have savings somewhere.

**I: Now, what will we do about this desire issue so that we may be able to save?**

PH01: Me as number 1, I save alone, but if we are as group such as the 10 of us, we would have depended on this program to guide us on how we can save. Like I know it is not a must to remove 500(ksh) every Friday, but you know if you see that 10 people have contributed and it is only you that is remaining; there is a way you will be motivated to save.

**I: Mhh.**

PH01: Eeh.

**I: Mhm, and that other challenge you mentioned was which one?**

PH01: low income.

**I: Low income. There is also someone who mentioned to me a challenge here. Low income and responsibilities are higher; how can we go about that so that we are able to save? [Posho mill sounds].**

PH01: Maybe if just attempt to think, like this organization called *jitegemee, when* it just speaks to us, there is no financial support it can offer us? [Respondents giggling] just a bit so that we also fight not to bring down this j*itegemee.* Us as human beings love challenges but we also love support. Which form of support can you give us apart from just speaking to us? Yes, you are speaking to us, but what will we save by just speaking to us? Just begin with just a little (to mean financial support).

**I: Mmh, number 6?**

PH06: I as number 6 I think that people fail to save because of lack of knowledge of how to do the savings. If jitegemee can give us knowledge of how to save it will be better.

**I: Mmh. Number 1?**

PH01: I as number 1, that point concerning low income, as *jitegemee* the way you are talking us like this you can also speak to none sex workers so that they know that this is work just like any other. You know like now; I had told you I opened shop but things ended up expiring and getting spoilt in the shop. You know if they were taught so that they know this is just work like any other, but help this woman come out of that life so that she can come and live this life that you view as better. Nobody wants to suffer. Me I cannot suffer at the shop when I know that if I come this side (to mean sex work) I will get (money). You know if you teach them, that stigma reduces and then we can open other businesses’, then our income goes up the way as our bills go up.

**I: Mhh... And you have already told me why sex works do not save, there are even others who have said that they do not save and have given reason, and are there any disadvantages of not saving? What are the disadvantages of not saving?’ Number one has spoken too much, another 1 number 7?**

PH07: I as number 7, what can make you not to may be because you don’t have the capacity to. Maybe you may have it and you do not have it.

**I: Mhh. And what disadvantage may you get when you fail to save?**

PH07: Sometimes your child can get sick, and if you have not saved there is no way you can help it. Maybe for example there is something that happened somewhere if you have not saved there is no way you can help yourself.

**I: Okay number 10. What disadvantage can you encounter if do not save? Number 10[giggling] while number 10 is thinking, number 9? Disadvantages that you will encounter due to not saving?**

PH10: The disadvantages that can occur for me is that your child may fall sick just as number 7 had stated. Sometimes the landlord needs wants you to pay your rent.

**I: Mhh number 5?**

PH05: If you don’t save then you have not secured your future.

**I: Mhh. Number 3?**

PH03: If you don’t save then if a certain problem hits you, then there is nothing you can do. Since you have not saved, you do not have where you can get money from.

**I: Mhh. Number 4.**

PH04: Save, we just try so that must save, so that we can see how it can help us because saving helps. There is a time when savings can help but there are times it becomes so difficult until you don’t have what to save. But you have to really try hard so that you can even save 50(ksh).You can use it to sort out yourself.

**NT: if you do not save then what happens?**

PH04: if you do not save

**NT: What problems will you encounter?**

PH04: The disadvantages I will encounter are sometimes I have even desired to buy something and I do not have something [some saved money] I have put aside and I don’t have any other place I can get money; I must be stressed up.

**I: Okay. Number 2? The disadvantages one can encounter because they have not saved?**

PH02: I as number 2, the disadvantages one can encounter because they have not saved; sometimes it can happen that get stuck somewhere and you do not have money, you have not saved. Or your mother can call to tell you that my daughter, I am stuck somewhere and you do not have anywhere you have saved. So it is good when people save.

**I: Mhh. Number 6 you want to add something?**

PH06: If someone has not saved their money, it leads into debts, puts you into so many debts. You become someone who borrows a lot, someone who begs a lot.

I: **Mhh**.

PH06: [Interruption from participant] you look like Kenya, you have debts from East, right and center. (To mean you have debts everywhere). That is the disadvantage of failing to save.

**I: Mhh, number 1?**

PH01: I as number 1, I want to say that if someone has not saved, then you become a person who is alone, people leave you. Because in these groups, we save, so if you are not in a group or you are not saving, you are left all alone. Something like if a child is sick, you know if you pay insurance, it does not mean that your child is going to become sick today but that is saving. You can sacrifice 500(ksh) per month and pay. Even if the child is sick or has broken their leg and surgery is needed and the money is 100,000(ksh). With today’s world the way life is hard, you cannot go and borrow. You cannot go with a proformer that fill up for me like it used to happen earlier on. It is just that you depend on yourself, and if you have not saved you will find that people have abandoned you, your friend are also lying to me, I do not know what. They are not lying to you, you just have debts, you have stress, and when people see you they are even afraid of calling you. They know that you are just begging every time, you just keep on saying help me help me.

**I: Okay and number 8. Someone who has not saved can encounter which problems? …**

PH08: I as number 8, if you do not save you can find problems. Maybe you may have a child, maybe he/she wants something and you do not have where to get it from.

**I: Mhh.**

PH08: And if you have saved when they want you can take a portion from there I have finished.

**I: And the advantage those who do not save get are like which ones? Advantages of not saving are which ones? Number 10.**

PH10: I as number 10, there is no advantage one can have if they don’t save.

**I: Ooh! Okay.**

P: What is the question? [Respondents laughing].

**I: Advantages of not saving? Number 5**

PH05: Advantages of not saving, you don’t run into losses, like maybe you save and then you die; the people who are left behind are the ones who are going to enjoy your hard work. [Respondents laughing].

**I: Number 1.**

R1: Advantages of not saving? You will be fat. Like mostly those who have said they just eat (to mean those who said they do not save) you can see how their bodies look good. [Respondents laughing]. You will get fat.

**I: Eeh another 1. The advantage of not saving is? Number 3?**

PH03: If I have not saved then, maybe I have taken my money and kept it in the bank as it has been said here; sometimes your bank gets hacked and your money gets lost. For the person who did not save you have eaten it in your stomach (you have eaten all your money) [respondent laughing].

**I: Is there someone who wants to add on? Mhh number 6.**

PH06: I as number 6, the advantages of not saving; one of them is; your neighbor admires how you live.

**I: Mhh…**

PH06: The person you stay with (to mean close to) as your neighbor will even start competing with you and they do not know that you are not saving. [Respondents laughing]. As in if your neighbor sees you, he/she comes to borrow from you. You have friend who come to borrow from you.

P: Maybe you are denying yourself.

PH06: You are not denying yourself, you are eating but not saving [participants laughing].

**I: You have to say which number you are before you speak. Alright? And for those sex workers, who save where do they save from mostly? Number 2.**

PH02: I as number 2.

**I: Mmh.**

PH02: I always save in a saving group [chama]

**I: In the saving group mhh. Another one? Number 4?**

PH04: I as number 4, saving, you cannot save in the house because to be honest, if you take your money and keep it there at you house you cannot save. Even if you take your money and keep it in a bottle, you will just have to shake that bottle at some point until you remove. So, saving is good in a saving group [chama]

**I: Mhh.**

PH04: You take your 100(ksh) and take it to the group since you know very well you will not be given your money until some other day when your part reaches (to mean when your turn reaches).

**I: Mhh, number 8?**

PH08: I also cannot keep money in my phone ah. [Slight murmuring]

**I: Eeh. It is number 8 speaking, jus speak.**

PH08: I put it in the merry-go-round/saving group.

**I: In the merry-go-round mhh, number 6?**

PH06: There is part I keep in the merry-go-round and there is part [inaudible segment] … because nowadays there are agents everywhere, I put some in co-op (Co-operative bank).

**I: At the bank? Ehe number 3?**

PH03: I as number 3, mine comes and immediately It is transferred to M-shwari locked account.

**I: Locked savings. Number 9, where do you save from? [Respondents murmuring and laughing].**

PH09: It is when I have started saving, it’s now that I want to start [respondents laughing].

**I: Okay, okay. So she has said that she cannot save from the house because she will use it (it to mean money) right? She also said that she cannot save in the phone because she will use it. Is there any other reason for saving where you keep your money? Why do you put it in a locked savings? Why can it not just be M-shwari only?**

PH03: Because, let’s say I have 1000(ksh). I have come back with 1000 from working (sex work) from that 1000(ksh) I tell the agent I want you to deposit for me 500(ksh) because this 500 if I stay with it I will just use it. But if I put it in M-shwari, in the locked savings account, I will not withdraw it after some period of time; let us say 3 months. I will come to withdraw it. But if I save it in M-pesa a short while my bundles will be over and I will use that money to purchase, another short while credit (to mean airtime) is over I will buy, so it will not help me.

NT: You did mention the number.

PH03: Number 3. Number 1.

PH01: I as number one, I like to save in the Sacco, because I know that at the end of the year I will be given dividends and my money will not just stay there at the bank to make someone happy, it would have appreciated.

**I: Mhh. Number 6, you spoke of a bank. Why do you save money in a bank?**

PH06: As for me, I love the bank because there is something at the bank called fixed deposit, there in the fixed deposit account, there is interest after that period you have saved.

**I: Okay. Number 5 was telling us that there are times responsibilities become more than income [coughing]. In the normal life of a sex worker, do they always live their lives like hmm their income is low but they want to live a high end life?**

P: Yes [a collective respomnse]

**I: Why is that? [Posh mill sounds]. … ehe, number 3?**

PH03: I as number 3, I as a sex worker, I cannot accept to live a low class life because out here you find that there are people who identify you as a sex worker. So it is a must that you put yourself where they have placed you. Because they always say that those who do that work have money. So even if you do not have, you just have to put yourself in that class. That you have, even if you do not have.

**I: So, you will live a life that is higher than your income, so that people can?**

PH03: Can admire your life, they should not say that, “eeh, she always does this (does sex work) nowadays and why then is she like this?” Let them talk the way they want, but, there are some who will say that, “eeh!! She is living well even if she is doing that (sex work), she lives well.”

**I: Mhh.**

PH03: Eeh. Just like that.

**I: Number 7. Does a sex worker get less income but she lives a life that is, is high?**

PH07: That is higher than income

**I: Why?**

PH07: well, it will force you to live that way because that is how they identify you.

**I: Mhh … ehh, number, I will come there … number 2?**

PH02: I as number 2, us as sex workers, I myself do live a high class life; I also see to it that my children learn in expensive schools. So that those who can say that, “She goes out in the morning and comes back in the morning.” Why do their children go to those other schools? So I want a scenario when I go out in the evening and come back in the morning and my child is living well and is getting quality education and I also live a good life.

**I: Number … [crosstalk by respondent’ ’one’’]. Number 6.**

PH06: I as number 6, what will make me live a high class life is because I love to attract client who have money. I do want to attract clients who can only afford 50(ksh), [another respondent interrupts]

PH01: You know money attracts money, [respondents laughing]

PH06: You know money is like a spark [magnet]. When you live a high end life at up there It will attract people who live in that class. So I will just have to live a high end life, even if the bills are high, I will try to maintain.

**I: Ehe, number 1,**

PH01: I as number 1, I will force myself to live a high class life because I bring my clients to my house and you do not expect them to find that; the mattress is down [a small one], and it’s the one in color blue. When they come they should find that the mattress is high density that super big one. They find it on the bed well spread on.] Even the bed itself should look like an imported even when it is just made here at downtown. So that, when he he comes in [in the house], he finds that you have set the standards, he cannot tell you anything. He comes and does what he came to do, even if he was in the mood of giving you 5k(5000ksh), when he looks at the glass in which you have given him water in, what is 5000? He slaps you with 50000. You even say wow! I am not going to work this whole week. [Respondents laughing].

**I: And… we have agreed that we live a life in which, our income is low but you live a lavish life how do we bridge this gap? This money for topping up for our lifestyle to match our income because our earning is low, right? And life we live is high (high class). Now how do we get the money to bridge that gap? Number 2.**

PH02: I as number 2, getting money to top up there (coughing) depends on how you save. You see, you can save and then in future you realize that life is hard and you may go to your account to withdraw that money because you know in future you are going get some earning and be able to return it.

**I: Mhh, Number 6.**

PH06: I as number 6, that gap, I always bridge it by what I do during daytime. Daytime, you know as a sex worker, you should not just put your mind on just sex work only, during the day your mind should also be on other things because right now life is expensive. Now for me I usually bridge mine (my gap) through what I do during daytime.

**I: Now these things you do during the day time are like which ones?**

PH06: You get part time jobs.

**I: Mmh. Like which ones?**

PH06: like for instance, people were even going for catering, you are called to go. It is not that if you are doing sex work then when called on to these other jobs then you do not go. You do them, so that keep your standards.

**I: Okay. Ehe number 5. … [Respondent giggling], [inaudible information].**

PH05: I as number 5, uum, to kill the bridge (to mean the gap) between living a lavish life while having a low income can be through small mall jobs. Maybe you have bought few clothes you have hawked them a bit, a bit since maybe the price of renting a shop may cost you a lot. You have added a few shoes just as long as you have made a little money and the profit you have made. You see how you can fixed the portions of your budget that you messed up to that it balances up

**I: Mhh, number 4?**

PH04: I as number 4, you see the… to be down and may be you are better. Somewhere in between you have to pretend you go and search for a job even if it is just doing laundry, you can wash clothes for people but it is only you who knows what you are doing.

**I: Mhh.**

PH04: You even go and wash for someone and they pay you 150(ksh) and you come back. But as for savings (to top up your income), so that someone looks at you and they think you are well off. We will go to sex work where we usually go to get our money, we come back to buy our things. So that someone may see you and say, “This girl lives well.” But it us who knows where the shoes is squeezing our feet.

**I: Number 10, if your income is low but your lifestyle is high, where does money always come from to upgrade your life to be on top?**

PH10: I as number 10, If I go there where we meet out clients, now it depends on how much I will be able to attract from the client. I will talk to my customer (sex client) to give me good money. When he has already given me, I will go the house. When I come to my house during the day, I can even look for work to wash people’s clothes, and save all that money.

**I: Mhh. [posho mill sounds], number 7 you have a point?**

PH07: No.

PH01: I as number 1, I pretend, you will not know when I have and you will not know when I don’t have. But I have all the time. So, so as to bridge my gap of pretending; I have a man for rent, a man for food [coughing], I have one to pay for taxi, I have one to give money for plaiting my hair. You just make phone call ‘’ hi daddy’’ you fix your voice. Him he knows you are in a serious relationship; he does not know of this other work of yours. Now if you are in a serious with someone, there is a way a boyfriend takes care of a girlfriend and eeh. But now he does not know he is being helped, you know everything has to have something that supports it.

**I: Mmh.**

PH01: But he does not know that he is being supported by even six other people. [Respondents laughing].

**I: Okay, alright. So there are those who think that they are the only ones?**

PH01: Yes “daddy”, [respondents laughing].

**I: And do sex workers borrow money? Do you have debts?**

PH04: Debts have to be there.

**I: Yes.**

P: if Kenya itself has debts.

**I: Yes Number4**

PH04: I as number 4, debts have to be there. But we have to try hard so as to repay them so that someone does not say that one is a debtor. But the work we do, we do it but we try to hide so that someone does not say that, “that person who has a lot of loans, then why does she go to work? So we have to try very hard but debts are there.

**I: Mmhh.**

PH04: People must take loans.

**I: These loans, where do they come from**

PH04: Sometimes you have gone to shop to borrow… may be i do not have sugar, then I go to the shop and borrow sugar

**I: Okay, so you can borrow food and borrowing money? Loaning food items happens at the shop.**

P: [All] Yes

**I: What about borrowing money?**

P: [All] [Participant answering at the same time] from shop/ from saving groups/ friends.

PH07: From another person.

**I: Oh you take from another person?**

PH07: Yes

NT: that is number 7?

**I: Yes, that is number 7.**

PH01: I as number 1, I do not borrow from anyone, I know they will gossip about me. I go and take a loan from a Maasai [Masaais who give people money and you bare charged some interest] [respondents laughing]. Their interest is high but they do not know how to speak (to mean gossip) they will not go around telling people oh! It’s this person.

**I: Mhh.**

PH01: So if you take like 1000(ksh) you pay back with 1300(ksh), their interest is high but their money is readily available; you do not have to beg them to give you, they just give you.

**I: Generally, we have already said that in the shops; we take food and we use them at home.**

P: [All] Yes.

**I: But this other money we take from friends or Maasai what do we use it for?**

PH01: Rent. As number 1, when I get stuck like it reaches the point where everything is stuck I borrow money for rent and school fee from the Maasai.

**I: Okay. And what was number 7 saying?**

PH07: It is for food.

**I: Oh, it is for food. Number 3?**

PH03: I do not have anything to say.

**I: Number 4? These loans, what do we do with them? Where do we use them?**

PH04: When you go to borrow a loan, then you have gone to borrow so that this loan can help you somewhere. If you have failed to pay rent and there is nothing you can do. At times we have gone to our work [sex work] and have not gotten any money. So it will force you to borrow loans so that you can pay for that house (to mean pay your house rent).

**I: So how do you pay them back? You have borrowed from the shop; you have gotten from the Maasai.**

PH04: Through work. We know very well that the job we do. It is a job which you must at least get something small. If you don’t get today, then tomorrow you will not fail to get.

**I: That Is number 4.**

PH01: As number 1, in this job of ours, there is off peak and high peak.

**I: Mmh.**

PH01: You see during the week. Monday Tuesday, Wednesday, Thursday that is high peak. Because this man will leave the house and go to work then pass by your place and then go back to his home. The person he is with at his house (to mean he companion) does not know anything; so you get he is so free at that time and maybe it is end month, and it happens to fall on a holiday. On a holiday they have gone with their children to celebrate somewhere.

**I: Mhh.**

PH01: You have remained there with bills to pay but you do not have a customer, but once they come back when it is not a holiday there is nothing, those other small small weekend then you will be fine.

**I: So, we pay loans from our work or it comes from our work? Any other place? Where does it come from again? The money we use to pay debts? Mhh number 6.**

PH06: I as number 6, sometimes if I take a loan from the merry-go-round/saving group. I may take a loan while I have targeted a boyfriend who does not know the kind of work I do. Maybe he is a boyfriend whose pockets are not bad (who is financially well off). Maybe it is the governor’s child who has money. So I will take a loan at the merry-go–round/saving group and call to tell him that “I am really stuck here, can you send me even 10k (ksh)?” Then I use that 10k to pay back (the loan).

**I: Okay. Alright. And if our income is low, what do we do to try and raise it as sex workers? … Have you understood the question?**

P: [All] Yes.

**I: The Income is low, and we have already found out that our lifestyle has to be lavish, so what can we now do to increase our income as sex workers? I want people who are not speaking like number 9 to begin.**

PH09: Let me think about it a bit.

**I: You are still thinking? [Respondents giggling]. Number 6**

PH06: I as number 6, when the income is low, and I want to maintain a lavish lifestyle, I get other boyfriends. So I do not just keep one boyfriend.

**I: You add boyfriends?**

PH06: I add even four of them.

**I: Ehe. Number 1.**

PH01: I as number 1 when I began this work, like life was not as hard as it is right now. So I had like two clients per day, but now, the more the merrier. Even twenty (to mean 20 clients) so long as the money is there. So I had to increase my working hours. For me anytime, even now if I am called, you will just see me leaving. Because I have added my hours at work.

**I: You have increased your work hours and also added partners (sexual partners).**

PH01: Yes.

**I: And number 8, what do you do so that your income increases?**

PH08: I as number 8, I also look for a boyfriends. [Respondents laughing].

**I: Number 8 also adds a boyfriend, number 2?**

PH02: I as number 2, I will not wait for evening to reach, I will be working through out as long as I have gotten client who has called’ ‘’I am at such a place, can you come?’’ then I go.

**I: You work both day and night. Ehen number 3. What will you do so that your income goes up?**

PH03: I add boyfriends.

**I: You will add boyfriends?**

PH06: I as number 6, if my income has run low, and I have the phone number of some of my clients who moved/ are far away, I start flirting with them through the phone

**I: Mhh, number 1 you want to add?**

PH01: Eeh! I as number 1, the way life is moving like this and I have already added boyfriends, and also I have added clients and still, I see life becoming so hard. I see myself changing towns, you know if you are fresh in town, it is you that everyone wants. [Respondents laughing] Bomet here I come.

**I: Number 5 do you have point?**

PH05: No I am just listening.

**I: Okay, the way you have told me that sometimes, there are no clients. So what do you do as a sex worker, when there’s is no client? What do you do? Number 4.**

PH04: When you find that there is no client, you will just have to go back to your house. There are no clients, there is no way you can get anything small (any small money). That mean even if you are to walk on foot from where you are until your home, you will just walk.

**I: Mhh.**

PH04: Eh! …

**I: When you fail to get clients?**

PH04: You just go back to your house.

**I: Number 6?**

PH06: I as number 6, when I fail to get a client, I usually have connections with these ladies who work at the big clubs and hotels. So when I fail to get a client I always search for them through these ladies.” How is it there today?’’ I ask them, and I must get.

**I: Mhh.**

PH01: I as number 1 when I fail to get a client, because the market is dry like this, I on the other hand dot get dry. I take my phone and start asking my money back from the people I had lent money to. There are people we always lend loans, they pay you 1000(ksh) and remain with 1000(ksh). That 1000 you collect later and if they refuse to give you back your money, you blackmail them. He either pays you 10k (10,000) or you send his wife photos. [Cross talk from respondent] [Many respondents laughing]

**I: And can you know in advance that today there are no clients? Or you always just go and find that there are no clients? [Interruption from respondent]**

P: Today is a bad day.

**I: Do you know earlier or when you have just arrived? [Cross talk]**

PH07: After reaching there you just look and things and say, “Today things are not good at all.”

PH08: Time is just going and you see nobody saying, “Hi..

P: You have to mention your number.

**I: The one who has just spoke is number 7. Number 8 has also said something, eh what were you saying?**

PH08: I was saying, you do not see any sign (of a client); you do not even hear someone do “psss’’ [respondents laughing] you have to be prepared.

**I: Okay, number 6.**

PH06: I as umber 6, so as to know today the signs are not good. I know through my phone because most of my clients call me. That day if there is no call I have received, I know that it is bad and I start looking for clients from elsewhere

**I: So you can know in advance?**

PH06: Yes.

PH01: You see like during holidays, I as number 1, I’m saying that during holidays there are normally no clients. Because everyone wants to be lovey-dovey to their partners. So as we always left this other side dry [without clients]. Like during this Labor Day weekend, we already know it is going to be dry, like it is already in mind.

**I: There are some days you just know that this day is not ours?**

PH01: Like during valentines, on Valentine’s Day. Who do you expect to come to work on a valentine’s day? They have gone to be lovey-dovey, they come on 13^th^ or 15^th^.

**I: Mmh. What is the meaning of lovey-dovey?**

**PH04: They take their lovers outside [their families] [A participant laughs]**

**I: Okay;**

PH07: I as number 7 I’m begging; I am begging on behalf of all of you. I have a child who is coming from school and she has to go back to school, how can you help me?

**I: We are almost finishing.**

PH07: It is already afternoon and I have not cooked for her.

**I: We are almost finishing. We are continuing. We are moving on well. [Silence]**

PH04: My child is just 1-year-old, and hungry [respondent’s laughs].

**I: We are almost finishing. [cross talk: ‘’take her to day care”] and if you take loans … [respondents murmuring] how much is it? That we did not cover.**

PH02: I as number 2, when I go to the shop, or if I go borrow money from the Maasai, I takes if it is too much 2k or 3k.

**I: Mhh.**

PH02: Then when I loan at the shop if it is too much it is 1k. (1000 ksh).

**I: Mhh.Number 7**

PH07: I as number 7, I fear taking loans when I take, I take for 70 or 100 (ksh), [respondents laughing]

**I: Mmh number 6.**

PH06: I as number 6, when I go to the merry go round/saving group, I borrow 10,000(ksh), from the shop I have set a limit; my children know and the shopkeeper also knows it cannot pass 500(ksh).

**I: Per day or?**

PH06: No, per month.

**I: Oh! Okay uhuh number 5? Number 5 is already home (not paying attention) [respondents laughing].**

PH01: I as number 1, I just like to take loans from the Maasai, because they will not gossip about me. And at the shop, you will never find me borrowing from there.

**I: How much from the maasai?**

PH05: The more the merrier, even 20k as long as it is just from the maasai.

**I: Mhh.**

PH05: Do you know that sometimes you don’t have money to even count in your pocket? But you have done everything, you have paid all your bills but you don’t have any money in your pocket. Some pocket money in that when you are even called ‘’hello come to Acacia’’, you do not have money for taxi, you take a motorbike and reach there feeling as cold as axe that was left outside overnight. You just need to have some money to use.

**I: From the Maasai, and you have said it always how much?**

PH05: 10000-20000 (ksh).

**I: Okay and number 8? When you take a loan, how much is it?**

PH08: When I take, it does not exceed 500(ksh).

**I: Ooh! It does not exceed 500? Okay, alright, and. when you do sex work, do you think that someday you will quit it?**

P: [All] Yes.

**I: Number 4.**

PH04: I as number 4, I even wish I could quit it but now, I cannot quit because I have not yet gotten what can help me. So for now it will just force me to still go there.

**I: Mhh. When I asked that question, everybody has said “mhh”’. So let us that say.**

PH08: We will quit.

PH07: I as number 7, I saw a friend of mine doing that job, she was beaten here until I got afraid and wanted to quit. [Respondents laughing]

**I: Mhh. Mhm,**

PH07: Right now she is at the ward.

**I: Number 10…**

PH10: [Silent]

**I: Number 9.**

PH09: I as number 9 I see that in this work, sometimes you get someone who look mysterious. Sometimes you even fear. And he is also huge you can even think that he takes drugs

**I: So if you say that someone is mysterious, what does that imply?**

PH09: Sometimes his whole body is filled with scabies/rashes [respondents laughing].

PH04: So I as number 4, going (to do sex work) is not our wish, we just go because we are looking for something to eat, you can meet with something you have never met before. You can meet with someone who even when you look at him, as in you are having sex with someone and you are facing the other direction, just as long as he gives you the money you went to look for.

**I: Mhh.**

PH04: So it is not our wish. If we can save the savings can help us.

**I: Mhh.. Number 5.**

PH05: Even donkeys get exhausted [respondents laughing] donkeys get exhausted there are times I pray that someday I will quit.

**I: Okay, number 7.**

PH07: I as number 7, there is a man you get who is really tall and yourself you are too short. But when you touch his private part you find that it is small it is better for him to just give you money.

**I: Mhh… number 3?**

PH03: We will just quit [respondents laughing].

PH06: I as number 6, you know, I entered into sex work because of one, two, three things. And you know when you have a target, everyone… even those who have been employed even if it is in government; they enter and say within this 5 year I want to work for government and I will have captured this and that, I have succeeded in this and that even if I go back to sit at the door (if I retire), I can still eat.

**I: Mmh.**

PH06: You know I as number 6, I have a target of quitting this work (quitting sex work), but after I have accomplished everything that I want to, that is if age will allow me.

**I: Okay. Number 1**

PH01: [Respondent giggling] I as number 1, I really wish to quite this work. But if I remember the problems in my household, heh! I cannot quite not unless I have gotten something that helps me, so that I can quit. Because if I quit, who will pay for me my bills? And who will take my kids to school? Now is when they are growing (to mean her children) and you know as children grow then their needs increase.

**I: Mhh.**

PH01: So it becomes very hard for now and when I joined (sex work). I joined because I had my own problems. These problems have reduced but they have not completely ended. But when I quit I do not want to suffer, so I am still not quitting.

**I: And, this quitting, is it something you always talk about let us say in your hotspots where you work? Can you get people discussing that they will quit or that they are planning to quit tomorrow? Or every person thinks differently? Number 4.**

PH04: I as number 4, there are times after you leave, you just feel that you are tired. You don’t even feel like going there, but you are forced to go because you are going to search for money. Sometimes I even wish, I even say it in my heart that ehi! I am tired of this! (Sex work). I don’t even want to go there but it forces me to go. So I just speak to myself.

**I: Oh! It is not a conversation you can have in a group?**

P: We always talk about it but quitting is a personal choice, yours personally” you cannot tell the other person to quit”.

**I: So then, when you begin talking about quitting, what brings about such discussions?**

P: [Inaudible Segment]

**I: Number 7. What usually makes people start talking about quitting?**

PH07: Because of what they have seen, you know someone cannot just quit something all at once if they have not experienced something bad over there. So for me the cold I see is what I see that could make me quit.

**I: Number 3.**

PH03: I as number 3, you may go there and go with a client and then he beats you inside there (inside the room). There are some clients who go with intention of beating people (sex workers) [respondents laughing]. There is a friend of mine, [coughing] she is my cousin, she is also one of us (sex workers). She came and told me, sister, I have quit, I asked her why? The beating I’ve been beaten inside there (to emphasize on through beating the cousin got from a client) and I was not even paid. [Respondents laughing]. Ehh so at times they get beaten (sex workers) inside there, when you come out, ah! ‘’Ah… I have surrendered’’.

**I: Let number 7 add on.**

PH07: I as number 7, I have already seen my friend, there is a man who seduced her and took her to the room, that day he played for her *ohangla*( luo music genre*)* that Luo songs [respondents laughing] they did not have sex, they did not do anything but he told her to strip of all her clothes, she was left with her pant on. She danced until morning [respondents laughing] and she did not sleep.

**I: The question is, the topic of quitting sex work [interruption by respondent what brings it about? What brings it about so that people can discuss it...? [Respondents murmuring and laughing].**

PH01: I as number 1, I think that, it is frustration, the moment when someone becomes frustrated with a client is when they think about quitting. Even the ones that are beaten, you just here them saying that they would wish to quit but they cannot.

**I: Mhh.**

PH01: That is after they have been frustrated. But sometimes after they have gotten some good money, she has gotten someone who plays until it stand well*. ah!* There is no story of quitting, but when she has been beaten inside there and then she comes out she has been shaken up like milk shaken in a calabash gourd (to mean she has been ruffed up). They quit, they even sit down and say how they would want to quit.

**I: So, now when she has been shaken, does she come and tell her friends?**

P: [All] Yes

PH07: Maybe if He comes and wants to take another lady, she tells you, “this one! Don’t go with him,” With this one (this client] you will remove your clothes from morning and stay naked the whole day [slight giggling].

PH06: I as number six, you know we have different hotspots in which we stand. Let us say in our hotspots we have discussed that we want to quit because maybe sometimes we experience different types of things. We are frustrated by men, and we have tried to go to the police and they do not respond.

**I: Mhh.**

PH06: That is when we sit down and say ‘’we will just quit’’. A day will come when we will just quit .I

**I: Mhh**

PH06: If the police themselves cannot help us, they just take our money and they even arrest us. When the police arrest you, he does not take you to the cell (police cell), he takes you to his home. You go spend at his place, he beats you with a hammer until morning (has sex with you until morning) without a condom and then he tells you, I have already released you now.

**I: Okay. Number 2 did you have something to say?**

PH02: she has already said what I wanted to say.

**I: Number 5? Did you have a comment?**

PH05: No

**I: And… even this you have already mentioned but you can add on. Why do sex workers want to quit sex, sex work I mean? … You have already mentioned some right?**

P: [All] Yes.

**I: Why do they want to quit? But number 6 and number 1 want to add on, do not repeat what you had earlier said.**

PH06: I as number 6, there are some hotspots, where ladies are there and it is not a must for the men to have sex with them for them to get money, there is what they do so as to get money. And on our side, the men must have sex with us so that we can get money.so you find that men already have some of their feelings so they start to [inaudible word] sex workers.

**I: Mhh. You have said that there are some sex workers who do not have sex with men but they are paid. Ehe, what do they do?**

PH06: What they do we cannot know because us we here from the stories they come to tell us that “I did not even have sex with a man but I have come back with 50,000(ksh)” [respondents laughing].

**I: Number 4.**

PH04: I as number 4, I may go there, but I will not have sex with a man, a client may make you just touch… touch him but you will not have sex with him.

**I: Okay**

PH04: But you get the money.

**I: And those who quit, do they quit after planning or they just quit at once? Mhh?**

PH07: It just comes, like me as number 7 I can just say I have quit’’.

**I: Not that you plan that next year.**

PH07: You know you can say you have gotten… let us say me as number 7, I have gotten a job here, then if I see that this other job is not taking me well, I just quit it. And you just leave it like that.

**I: Mhh. Number 4.**

PH04: I as number 4, when you get a boyfriend, that boyfriend has told you, don’t do that job, I will provide you with everything, I will pay for you rent, and do what you want. But you know that money will not be enough for you, now he is helping you but you have to look for a job to get money to supplement the budget.

**I: Okay.**

PH04: So you add on to it, and leave this one [sex work].

**I: So the question is, those who quit, do they plan or they just quit? That is the question.**

PH06: Okay, no one plans to quit their job, someone just quits, I as number 6. Let’s say someone from Nigeria has come to the hotspot to pick me, the he asks me “why are you doing this work?” then I tell him I am doing this work because of such and such. And, we have stayed in the room the whole night. We have not had sex, we have only had some discussions. On that discussion he tells me then I want to take you officially and marry you, you will have to quit that work without telling anyone that you actually quit.

**I: Okay, alright.**

PH01: The final thing, I as number 1, you see this work of ours, you cannot quit. Like you take a break, and even if you take a break, you cannot tell your clients that you have stopped because they will also leave you. So save them for a rainy day. Now like, there is a time I got someone (a partner), we pretended that we were in love and started staying together. It then forced me to stop my work, I had taken a break it a bit. But I just spoke with my clients they ask me “where are you?’’ “Im in Mombasa, and so on, I have gotten work.” “When you come back to =Kisumu= tell me”. So when I came back, they all came back.

**I: Okay. And, sex workers quit sex work at what age?**

P There is none

PH01: As number 1, there is no age, *an old cat still drinks milk (a proverb)*. There are some who will come, Babyboy’s those ben ten (to mean young men) who want the older women. If there are no older women then he moves to another spot.

**I: There are women whom you know that have quit sex work right?**

P: Yes

**I: At what age did they quit? Number 6.**

PH06: I as number 6, people quit sex work at different ages. You will find; someone of 56 has quit, someone of 25(years) has quit, someone of 18(year) has quit, someone of 30(years) has quit it depends on how she personally feels.

PH08: Just the way we had said in the previous question; no one plans to quit. So you will find that… for example you get someone who is willing to change her life, just like that she went. Sometimes one is just old, so she will just quit because she is tired. So there is no age limit.

**I: Are there those who quit just because of age, age is the only determinant?**

P: No.

PH07: I as number 7, earlier on women would think about age… When they find out your age is when you can quit let us say 90 or 70 (years) you can quit. But for now, “heh! Even an old man who is 100 years old can park out here, and you get surprised.”

**I: Okay. [Respondents laughing] so age is not a determinant. And those who have quit, what kind of jobs do they do? When someone quits sex work, she has gone out, right? What kind of jobs? This side is too much? I want from this side (to mean a respondent from this side).**

PH01: You said you are starting with those who are willing.

**I: Number 9. Someone who has left sex work does which kind of jobs?**

PH07: I have not understood you.

**I: When someone quits this sex work, she has quit, she will have to look for another job out here so that she can find a way to help herself to get money. So what kind of jobs do they do? Number 7.**

PH07: I can sell chips or samosa.

**I: Ehe,**

PH07: So that I get my money.

**I: Ehe, number 3?**

PH03: I as number 3, when someone quits that work, she can even open a boutique,

**I: Mhh,**

PH03: Saloon or hotel. Because she had already made money.

**I: Mhh.**

PH03: She is seated eating it slowly (eating the money).

**I: Mhh. Number 6**

PH06: I as number 6, you know as sex workers we got used to a lot of money coming in very fast. I as number 6 if I quit sex work, I open up a bar or wines and spirits.

**I: You will open it anywhere or a specific place?**

PH06: Before I quit sex work, and it is in my mind that I want to quit, I start searching for locations in which I can open.

**I: Mhh. Number 1?**

R: I as number 1, a lot of sex workers whom I have seen have quit have gone to church (have gotten saved). Because, society has lived telling them of how they are sinners, I do not know what, they are doing a bad job. So you find that they dedicate their lives to God, so they are the ushers, and sisters (in church).

**I: Okay. [Respondents laughing] And, do you know of any who quit then went back?**

P: [All] Yes

**I: And why did they quit then went back? mhh number 5.**

PH05: There is no money as sweet as this one (money from sex work) [respondents laughing] there no money you can compare with this one [laughing]

**I: Okay. Mhh number 7?**

PH07: I as number 7, maybe you loved good life and now it has gone down, you will have to go back there.

**I Mhh, number 4?**

PH04: Like for me, I was getting my money I was doing my things, but there is a time when I get broke then I would have to go back (to sex work) to look for money.

**I: Number 2?**

PH02: I as number 2, If I quit that job then I go and open up my own business, and then I find that the business is not going well with me, the profit from it is little; and then there (in sex work) I get good money, I will just find myself going back there.

**I: Mhh. Number 6.**

PH06: I as number 6, I know of someone who had quit, and she had quit because she got married, in the process of getting married, the husband was not doing for her everything that she wanted. The first five years, she was being treated well, but when she got pregnant, she started being mistreated. So after she gave birth, she went back due to the mistreatment she went through.

**I: Mhh, number 1?’**

PH01: I as number 1, I had told you that I had quit before and opened up a shop, then it failed. I went back (to sex work) because of stigmatization. These people look down upon us they look at us like sex workers do not know how to open any other thing apart from their legs. Now, there is a way they have branded us in society. Even if you try whichever type of job, you just find yourself going back (to sex work). They take you back. They sent me back to sex work, if they had supported my shop, I would have even been having wholesale today. But they brought me back.

**I: Mhh. And positives things ah…, negative things that happen when someone goes back are like which ones? Bad things are like which ones that happen when someone goes back (when someone goes back to sex work)?**

PH06: I as number 6, I have already seen someone who has gone back. Probably when you go back you will have conflict [noise in the background] with your fellow sex workers because they see that you have comeback and you have started taking their clients.

**I: Mhh. Number 5.**

PH05: When you quit and then come back, maybe it took time. You get the people who are standing there, are not the same people who you were used too, so they feel like maybe that is just their own spot. Something of that sort. They feel like that spot it is them who have been there. By the time they accept you back, by the time you get used to… [to mean it takes time to get back on track]

**I: We are almost winding up, I see that two people are tired over here and one has already left. Im just requesting we sit here and finish up [coughing]. We are almost winding up. So those are the bad things that what will happen when one quits sex work and then goes back? And the good things? Any positive thing? … [Respondent giggling] number.**

PH06: When you go back to the field, probably you will get new clients, who were already used too. You will get fresh people [as in new clients]. It is like a visitor has arrived and you know people like something new.

**I: Mhh. Positive things. Another one?**

PH01: When you are like, ah number 1, when you have quit work, you will take time, your body will heal, [posho mill sounds] as in your body will heal. [Respondents laughing]. Then when you get one client he will want to come back, because you have healed [to heal meaning their private parts have healed from not engaging in sex work for some time]. You have taken time and then the second thing I, the things which you could not afford, now you can afford because money will be coming in.

**I: Now that part about healing, can you further elaborate? … [Respondents laughing] by saying. Number 1 can you explain what you mean by healing? [Respondents laughing]**

PH02: Okay, I as number 2, want to answer where she is stuck. [Respondents laughing]. It is like you are now a virgin again [respondents murmuring] you gone back primary, you become fresh..

**I: Okay number 8. Do you have a point?**

PH08: No

**I: Okay. And … number 6, is it number 6? She had already told us, there are things that made her join sex work, there are things that she wanted to accomplish right? And…which kind of things can make a sex worker say, let me accomplish this and that and then I will quit. Number 3.**

PH03: I as number 3, if I want to accomplish… things I have to do before quitting, I will make sure that my child has studied until he has cleared his studies.

**I: Mhh. And has he begun school?**

PH03: Yes he has.

**I: Okay.**

PH03: I will make sure he has studied and has everything he needs. I do this work because of my child.

**I: Mhh.**

PH03 But when he clears school, he will be old enough he will be able to fend for himself.

**I: Number 9. Are there things that your heart feels like you should achieve and then quit that job, if at all you had plans of quitting?**

PH09: Just like number 3 has said when I am done educating my child.

**I: Mhh. Number 7… there’s nothing? Number 10.**

PH10: My heart wishes to quit this work, when I have opened an account or,[ inaudible segment] so I am just okay, even when I quit I will not have any stress.

**I: Okay. Number 4, do you have any point?**

PH04: My points are over.

**I: Number 6**

PH06: I as number 6, I joined into sex work when I was a single mother. My plan was to buy land for my children and build a mansion.

**I: Mhh.**

PH06: My second plan was to make sure my kids are learned.

**I: Mhh,**

PH06: And they go to a good school, when those ones have been accomplished, [posh mill sounds], me and sex work done.

**I: So right now your children are going to school?**

PH06: Yes.

**I: And have you bought land and built a mansion?**

PH06: Not yet.

**I: So they are still in the process? [Posho mill sounds]**

PH06: Yes.

**I: Okay, number 1?**

PH01: I joined this thing (sex work) temporarily, I did not know it would be like this. Day one I joined because I did not have food.

**I: Mhh.**

PH01: And I had children, bit by bit the children grew up and joined school. Huh! I still do not have school fees! Then I continued to pay school fees. Now, I have to retire and the bills keep on increasing and needs are increasing and I have not yet gotten another job. So you get like …

**I: So there are things, you have something to say number 5?**

PH05: No, no.

**I: So there are things that we intend to achieve first before quitting sex work,**

P: [All] Yes

**I: There are those we have already started doing Others are by default because when a child reaches the age of going to school, you will take them, whether you like it or not. There are some, it is not you that decides… [Crosstalk]**

PH06: Like the mansion.

**I: … like that mansion or buying land. What would be the reason why we have not completed these others (to mean the other needs)? I also want this other side, number 4 tell me.**

PH04: [Posho mill sounds] it depends on your income; how much you get.

**I: Mhh… income? Mhh, number 5?**

PH05: Living conditions being high. Sometimes living conditions are high.

**I: Mhh, living conditions are high. Why is it that the things we had set out to accomplish first before quitting sex work, most of them we have not yet accomplished? Number 9?**

PH09: Just that low income.

**I: Just that low income. Number 7?**

PH07: low income [respondents laughing]

**I: Number 10?**

PH10: Inaudible information.

**I: mhh, okay. And …you have something number 1?**

PH01: Eeh. Russia is still at war [interruption; “huh?’’] It is so! You can’t see that the prices of commodities have gone up due to that. [Respondents laughing].

**I: Now when they are done fighting?**

PH01: Maybe life will go back. (To mean maybe the quality of life will go back to normal).

**I: Number 6.**

PH06: I as number 6, there are thing you cannot accomplish all at once, they come in a process, one by one. Let us say for instance, buying land or buying a car

**I: Mhh.**

PH06: You cannot wake up in the morning and then say that you want to buy land or even a car.

**I: Mhh.**

PH06: You have to set aside some savings; you have to start saving and then have the money.

**I: Mhh.**

PH06: So life has become expensive and we want to save and that is why we have not yet accomplished all our goals.

**I: Mhh. But you plan on accomplishing them?**

P: [all] Yeah

**I: In the last 5-10 years, do you know of any of the people who have quit sex work totally? … [Cross talk “mhh”] number 7 knows.**

PH07: I as number 7 already know of one who had quit.

**I: What made it easy for her to quit?**

PH07: Mhh she was raped by five men until her “thing” came out [respondents laughing], she then got born again and joined a church.

**I: What is her “thing?’’ [Respondent laughing]**

PH07: I do not know how I can I can put it [cross talk by respondents] the female sex organ came out.

**I: Oh! … [Inaudible information, respondents murmuring] number.**

PH08: For me the one whom I know has quit sex work, she joined sex work but she did not have a child. And when she joined it, she told me that she wants to buy; a car, she wants to live her life, she wants to buy land and build her own house. When she accomplished all that she then opened a boutique”. Mhh… “There in her village, and that is how she quit “. Okay. Number 1.

PH05: I thought you wanted to say that she got pregnant.

PH01: I have seen two who have quit. One of them quit because of illness, she had sex with a man who infected her with a disease (STI) and then that thing (her private parts) got blocked. And then another 1, got married. …

**I: And in… number 6 has told us that there is one who quit because she accomplished her goals [crosstalk] ehe, and another one also told us that there is one who quit because she was raped. … So… those who quit [interruption from participant]**

PH05: Number one also said that there is one who her sex organ blocked.

**I Eeh… she had quit because she had an STI that blocked her vagina. Okay. And after quitting, what difficulties did they find? When someone quits sex work, and quits the way those others had quit,**

PH08: For me my friend did not tell me of any difficulty because she does not pay rent because her boutique gives her food, she eats well everyday she goes to outing almost every day because she has a car, she also has a boyfriend who has money [respondents giggling] , so she did not have any difficulties”].

**I: Number 1?**

PH01: I have seen one who has tried quitting so many times and she still goes back. I had asked her, because she is the one who had introduced me to this field. And she told me that sex is addictive, not that she had gone back because of any other reason. She had gone back just because of sex.

**I: Mhh.**

PH01: When you become addicted to good sex you will just come back for more.

**I: Okay. Is there someone else with a point? There is none. Number 10, you have a point? [Respondents laughing]. Number 9.**

PH09: I do not have anything.

**I: Now we want to close but we want to talk to *jitegemee* a little. As I had already told you, *jitegemee* its main purpose is for sex workers to be able to save money so as to help them be at a lower risk of contracting HIV/AIDS. The reason why that is happening, we had already mentioned right? When you save money you can say ‘’ah ah I don’t want to have sex without a condom”, “ah ah today I am tired I want to rest” so… the money that they are (sex workers) going to save, is just their money. You are just helped in saving your money. So I would like to ask ‘’ this *jitegemee*, in the whole of Kenya, can sex workers embrace it?” would they like it?... huh? Number 6?**

PH06: I as number 6, if sex workers are taught, and they get to know the meaning of j*itegeme*e, they will embrace it.

**I: Why?**

PH06: Obviously when you get the knowledge, you know that sex work… most of sex workers are women, and us women love to listen. Whatever you have listened too, and put it to action, it must give you satisfaction.

**I: Okay. Number 1**

PH01: I as number, I feel that sex workers can like it (jitegemee). Because it is not the first time we have been called to attend and we came. We have been called by different organizations, we sit down and they speak to us. But there is nothing they do. But you see, we have never given up attending, we still do attend. Even if you people go (jitegemee goes), there are still some who will call us and we will go because we want to hear how they want to help us. We are ready to receive that help, you know that this is the first step, coming. To step out and identify as a sex worker. That is the first step.

**I: okay. So you are saying that they would embrace it?**

PH01: We will embrace it.

**I: Why?**

PH01: Because, we have challenges and these challenges, you have assured us that this jitegemee, you have assured us that if we save there is a way we can help ourselves. All these challenges we have mentioned here that we go through and all the benefits of saving plus all the disadvantages of saving, if we put them together; the advantages are more than the disadvantages of saving.

**I: The kind of sex workers who can embrace jitegemee are like which ones? Which kind of sex workers?**

P: Repeat please.

**I: Which type of sex workers would embrace jitegemee? Sex workers who would embrace jitegemee are like which ones? Mhh… number 5.**

PH05: Those who can save…

**I: Those who can save**

PH01: I as number 1, can say that sex workers, mostly those who stand by the road, not the one who take clients to their house. Those who stand by the roadside, and get hit by the cold, are likely to embrace it, because them they will see that change immediately. Because when they save, and open up their business, they will have to step out of that cold and go to attend to the business.

**I: Mhh.**

PH01: But that sex worker who brings clients to her home, it will be very hard to convince them to save and leave their houses and go do some business. And then there are some sex workers who work at brothels which the front part seems like a barber shop or even like these beauty shops. You are given a massage and then out go to the back and are given ‘happy ending’ (to mean sex). You see it will be very hard for you to convince to convince such a person to quit that job (sex work) and find another one.

**I: Mhh.**

PH05: But when you tell her to open her own, it is easier. Because she will still be going on with sex work.

**I: Okay. ... Is there someone who wants to add on? Number 2? If you know about ten sex workers and then you tell them about jitegemee, out of these 10, how many will embrace jitegemee? Number 7, how many will embrace it, if you have ten sex workers, and you tell them about jitegemee, out of these ten how many will embrace it?**

PH07: Let us say like around five, two or three.

**I: Five or two. Mhh number 10, how may ill embrace it?**

PH10: Ten

**I: Ten people? Number 9?**

PH09: 5.

**I: 5, Number…**

PH05: 2

**I: Number 5 two, number 3?**

PH03: Three.

**I: Number 4?**

PH04: About Three or four there.

**I: Three or four, number 2?**

PH02: Six

**I: Number 6?**

PH06: According to me, it will depend on how I will convince them.

**I: Mhh**

PH06: Maybe, ten can join, or three or four.

**I: Okay. Number 1?**

PH01: According to I as number 1, I see like 6 people, and those four people, will not join because convincing somebody to save when they have never saved is hard.

**I: Number 8. …**

PH08: I see that those I can convince are about three.

**I: And those one who will not have embraced it, why is that they will not embrace jitegemee. Number 2?**

PH02: Sometimes their income is low and budget is high.

**I: Mhh.**

PH02: So there is no way they can get into savings.

**I: Number 3, do you have a point? Number 6.**

PH06: I as number 6, some of them will not join because they have not understood the meaning of jitegemee.

**I: Mhh. They have not understood the meaning? Number 1**

PH01: I as number 1, I feel that the four that I know of, will not join because jitegemee does not give us money, it is just you saving our own money.

**I: Okay.**

PH01: And people join such initiatives because they are assured that there is a loan or something of sort, but with this one (jitegeme) it is our own money.

**I: Now what can we do so that many of the sex workers embrace jitegemee? Number 3?**

PH03: So that those others, I as number 3, for our fellows to see that it is (jitegemee) a legitimate thing, as we are seated here ten people, we can sit down and say that from a certain day, or when you get something, (some money) come let us put [pool] it together. Now let’s say you have again been called for a meeting of ten more people you have a sat down with them and tell them about jitegemee, they will listen. Because you already have something somewhere, if you tell someone about jitegemee they will ask you what you have kept.

**I: Mhh. Number 2.**

PH02: When I tell someone so that I can appease to them, in case you have a loan, at times I am stuck and say, “Let me go to Jitegemee to apply for a loan. Maybe I am stuck with school fee, and I go to apply for a loan then I am given. Then that other friend of mine will see that I applied for a loan and I was given, so let me also get into this group.

**I: Mhh, number 6?**

PH06: I as number 6, so that these other sex workers can accept jitegemee, like as we are here ten people, we start to speak about jitegemee, then we start calling the other people (sex workers), we gather like a group of twenty, thirty and we call the ones who came to educate us on jitegemee.

**I: Mhh,.**

PH06: To also teach them so that they can also understand.

**I: Mhh., … number 1.**

PH01: I as number 1, I see so as this idea about jitegemee to go on, uhuh, the founders, the pioneers [name mentioned] and those others members of the committee can adopt the findings, and then us we create groups of this Jitegemee of sex workers. So that we can depend on ourselves. For us to benefit they can in cooperate us in the youth fund or women’s club because most of us are youths. Most of us come from poor backgrounds, we do not have a way of sustaining ourselves. You know when we find someone who can hold our hands, and that is government money that people are given, you know it will really help us a lot and what we have spoken about here can be possible. Because it is not like we have some other money somewhere that you can say, now this is for jitegemee, we will put it there. Even if we begin right now, to save, for the ten people; each person to give 100(ksh), that will be 1000 ksh. That 1000 ksh with this Kenya the way it is currently, even if you start setting up a business, what type of business can you set up with 100ksh? You will just go back to the field [sex work]. But if we have like jitegemee group a, and jitegemee group b, we then come together, a government official tells us that they are from youth fund or somewhere of the sort; then he tells us conditions of getting a loan are such and such, we try to meet them, where we are stuck, we then stop.

**I: Okay, and you think that if it is built, what should it comprise of? Components? She has mentioned of loan. What else can we then put in place so that jitegemee can work well or be embraced? Number 3?**

PH03: I as number 3, here among us, we have those who are educated and if you bring the organization that we are speaking about [jitegemee], and say that we are teaching others apprenticeship work like tailoring, hairdressing or there are some who wish to go back to school. You see, they may quit that job and go back to school and learn. She will then come to say in future that that I was like this and that but this organization known as jitegemee has enabled me reach here.

**I: Okay. What can we do as jitegemee in regards to savings? What should be put in place so that it can be acceptable? Number 8? How should we create jitegemee to be like? …**

PH08: I as number 8 can say this, when we have already uplifted this… what is it called? jitegemee, [ some background noise of men speaking] even if you get some little money you just keep on saving. Sometime it will come to lift you up.

**I: Mhh. Jitegemee should be comprised of what? Number 5?**

PH05: Hmm. We get to be able to save, to borrow just such things, it should be like a Sacco or bank where you can borrow save and deposit.

**I: Number 9. How do we create this thing to be like so that many people can embrace it?**

PH09? If we save?

**I: Mmh.**

PH09: We Unite and save.

**I: You save together. Number 9, how should we create this project to be like, so that people may be able to accept it?**

PH09: we can sit down and agree if we can open up an account where we can save our money.

**I: Mhh.**

PH09: That is how we can do.

**I: Okay, number 3,**

PH03: we can have, I as number 3, we can have something known as table banking.

**I: Table banking.**

PH03: Yeah. Where we can borrow money. Like members of that group, we can come, save and then loan money and then return it with interest.

**I: Mhh,**

PH03: Eeh.

**I: Number 1.**

PH01: I as number 1, so that jitegemee can continue, it should not be a project that dies in the group we are in. You should take sex workers themselves and involve them in the decision making process.

**I: Mhh.**

PH01: You ask them the way they would want to run their organization let us say of jitegemee, then once that organization of jitegemee sex workers has been formed, As sex workers ourselves should run it because among us sex workers there are accountants, security personnel, there is everyone.

**I: Okay. And what would sex workers love the most about jitegemee?**

P: [All] Money.

**I: Which money?**

PH03: The money we borrow

**I: Number 7.**

PH07: I as number 7, the day when we come to meet up here, we should come with even 100ksh and then we divide among ourselves then we go back home with it. Because we want money, and save the rest.

**I: Mhh. Number 10. What would sex workers love about jitegemee?**

PH10: I am still thinking.

**I: You are still thinking. Number 9?**

PH09: [Inaudible segment] [respondents laughing].

**I: Number 4? [Coughing]**

PH04: If we have another meeting, others will hear that a group has been formed and you can go there and borrow some money which you can help yourselves with and then you will return because it is where you can get help.

**I: Mhh. Okay. And what would they not like? You still have a point on what they would like, number 1?**

PH01: I as number 1, sex workers are people who look like people who are seeking for help. [Inaudible segment] but if we get people who can hold our hands (guide us), teach us how we can save, teach us how we can invest after saving. Even if today they have brought us an agriculturalist who will educate us on how we can plant some kales where we are living, that we can like. But what we cannot like is being asked for money.

**I: Mhh.**

PH01: Like that one for musicians (lsk), I’m not even sure what those are. That we give money and then there is a way the money is distributed and you may not get it that we will not accept.

**I: Money that goes out. But jitegemee does not have money that goes out.**

PH01: Ah-ah! As in, you have asked what we would not like.

**I: In jitegemee.**

PH01: Eeh. So in case you have forgotten and you would want us to give out our money. We will not like that

**I: Oh, okay. From what we have spoken, do you want to add? From what we have spoken about, have you seen where jitegemee can violate someone’s rights? Violate your rights? Any ethical concern? Is there any?**

P: [All] None.

**I: None. And when it starts working, which challenges can it encounter? Jitegemee. Number 7? When it starts working, which challenges is it going to encounter?**

PH07: There is none

**I: Number 10**

PH10: It will just be fine.

**I: Mhh, number 9.**

PH09: It will be fine.

**I: It will be fine any, difficulties it may have?**

PH09: its just about the money

**I: Mhh, what is there about the money?**

PH09: Sometimes it is agreed that each person gives 500 shillings and may be someone don’t have the 500 shillings.

**I: Mhh. Number 5?**

PH05: It may just be difficult if… if…

**I: Challenge, Say it in Kiswahili.**

PH05: Eeh! Let me think first. Pass [laughs]

**I: Number 3. We are finishing up, this is the last part, second last part.**

PH03: This organization or program will run well, when we put in place measures.

**I: Mhh, any challenge that can arise, you do not see?**

PH03: The only challenge is when they start to say give a certain amount of money for us to forward them somewhere and the moneys are not given back. That one is not acceptable.

**I: Mhh. Number 4.**

PH04: The challenges we have there are just about money matters, you can get that some people save money, some people do not save. So they, mostly they are the first to want to take a loan.

**I: Mhh.**

R1: So there can be some difficulty there.

**I: Mhh. Number 6?**

PH06: Okay the challenge I can get, first, jitegemee can start well,

**I: Mhh.**

PH06: And there are people who will oppose it.

**I: Mhh.**

PH06: That you are bringing people who want to eat [steal] our money. There may be opposition. Number Two, jitegemee can fail if maybe the ten of us we join and begin to save but when we now want to withdraw our money when one has a challenge, then our money is not available. Number 3, jitegemee can start well, then after sometime, jitegemee moves from=Kisumu= and goes to =Nakuru=.

**I: Mhh.**

PH06: That will cause violence.

**I: So how can we fix that? The challenge that you have spoken about? You have told me three challenges right? The first one we can do what?**

PH06: First, we can educate people to understand what jitegemee is.

**I: Mhh.**

PH06: The second one, when people have saved, when they want withdrawal they should access the money very fast] if you move from one place to another.

**I: Mhh.**

PH06: You give a prior information before you move.

**I: Okay. Number 1.**

PH01: I as number 1, I feel that jitegemee will have a challenge of opposition from like, you know we are sex workers and this organization looks like it is for sex workers. I know it is a big group but its branch is for sex workers. You will see that we will face opposition from like religious groups, with human rights, we will get opposed by I do not know what else again. We will be by opposed child’s abuse department. So if this jitegemee is serious about us sex workers it should be prepared for stigma because even where they stand announcing about jitegemee; there is nowhere you can stand with a microphone announcing that ‘’we are looking for sex workers today” you have just to send someone to go and look for them.

**I: So, how can we address this challenge?**

PH01: This challenge about stigma is just through education, we have to educate the society and tell them that this is just work like any other.

**I: Okay. So as we finish the last question, and we will not repeat any other again, [respondents laughing]. If we were to save money, we can just mention all round about how much we can save. Number 7, how much money can you save if jitegemee was saving? In one week, how much money would you save?**

PH07: Monday to Friday?

**I: Yes**

PH07: I can save about 500.

**I: 500 ksh… number 10 how much would you save?**

PH10: 1500 (ksh).

**I: 1500(ksh) number 9?**

PH09: 500.

**I: 500 ksh, number 5?**

PH05: 100 ksh. Weekly?

**I: Ehh, number 5 can save weekly. Number 3.**

PH03: 250 ksh.

**I: 250ksh, weekly**

PH04: I can just save 200ksh only

P: Number 4 can save 200? Number 2

PH02: 300ksh

**I: Number 2, can save about 300 ksh. Number 6?**

PH06: 2,500 ksh.

**I: 2,500 ksh, number 1?**

PH01: 70

**I: 70? Bob (shillings). [Respondents laughing] 70 shillings.**

PH01: 10 bob per day, very comfortable.

**I: How much will you set apart in a week?**

PH08: If I get it is 100 ksh

**I: 100ksh in a week? Thank you, number 8. Thank you very much I have taken your time please pardon me. But I am very grateful for your participation. Okay?**

P: [All] Yes

**I: Thank you very much.**
