# Supplementary material for: Eat a little and save a little: A qualitative exploration of acceptability of a potential savings intervention to reduce HIV risk among female sex workers in Western Kenya
Source: PLoS One. 2024 Dec 19;19(12):e0310540. doi: 10.1371/journal.pone.0310540 (PMC11658496; doi:10.1371/journal.pone.0310540)
Supplement: S1 File — (ZIP) [file pone.0310540.s001.zip › Jitegemee Transcripts and Dissemination Notes for Journal/FGD I.docx]

**VENUE OF THE INTERVIEW: KISIAN**

**FGD ID: FGD I**

**INTERVIEW DATE: 27/APR/2022**

**MODERATOR: NANCY OUNDA**

**NOTE TAKER: OLIVIA OKUMU**

**TRANSCRIBER: WARDA**

**CATEGORY: ABOVE 30 YEARS, PERI-URBAN**

I: Okay, thank you. Thank you, this is FGD conducted at =Kisian= and the FGD number is FGD_Kisumu_I the date today is 27/APR/2022, moderator is Nancy and a note taker is Olivia. The interview is being conducted at Kisumu West Sub County. Okay, we are going to start. So according to how I have explained to you about Jitegemee, in short what has crossed your mind as at now? Like what I have said about Jitegemee, what can you say about it?

PI06: What I can say about Jitegemee,

**I: Mmh.**

PI06: The way you have tried to explain, I feel it is a good program.

**I: Uh huh!**

PI06: That can change our lives as sexual (sex) workers

**I: Mmh.**

PI06PI06: So that we can have a better life in the future than the one we have now. So the main thing that I can say is that we should give one another a chance so that we can put our ideas together and see how this study can help us and how it can help our lives in the future. That is what I have as number six.

**I: That is number six, uh huh, any other person with another idea? (Noise from the background) about Jitegemee, number**

PI01PI10: Number 10

**I: Uh huh!**

PI10: Jitegemee talks about self-independence.

**I: Mmh.**

PI10: In other words, it looks into how we can find something to do apart from the one we are currently doing so that we can see how our future can continue (be better) only if we change the work we are currently doing [Sex work] So it is good if we learn looking at 1, 2, 3, (something) that we can do so that we can be people who rely on their own apart from going out to engage in sex with different people.

**I: Uh huh! Any other person?**

PI02: Jitegemee has brought…it has brought the issue of, if you get money after you have had engaged in sex with someone and that person gives you money, you can say that this money, if he gives you five hundred shillings, I can keep (or save) two hundred and fifty shillings and the remaining two hundred and fifty shillings I can spend on my children because you also have children in the house. So you cannot take that five hundred shillings and keep (save), that is my view.

**I: Uh huh! Any other person with an answer? That is number two. Who has a response?**

PI08: We see Jitegemee as a program that can help us as sexual (sex) workers, so I feel that if we unite the way we are seated here we have different ideas. Like maybe you have sex with someone and maybe he has given you three hundred shillings or five hundred shillings you find a way of keeping something small (small amount of money) and spend small (amount of money), so that we don’t live this kind of life forever because you have children and these children are growing. So when they are adults and you are just that sexual (sex) worker, so at least when we are seated like this (someone clearing her voice) we have different ideas which can help people.

**I: Uh huh! Any other idea? (Silence) Another idea?**

PI07: Jitegemee teaches us that you should not rely on sexual worker (sex work) all the time

**I: Uh huh!**

PI07: Because it (Jitegemee) teaches us that we can do business as well and it also teaches us that if we do table banking we can find a way of eating (getting food) well, we can educate our children and we can have a smooth life and even if you are tired of going for sexual worker (sex work), you can eat when you …you can go to table banking and you borrow, borrowing and saving and you can eat even when you don’t go for sexual worker (sex work). Secondly it teaches us that we should have an open mind so that when there is a problem, our ideas can help you in future and it can make you to have…you can save and start even a big business and leave sexual worker (sex work).

**I: Uh huh! Another one if there is any. Uh huh, number ten.**

PI10: Number ten, I am emphasizing on something here, being that sex work is something that you meet with different partners, Jitegemee also teaches us on how we can leave that work and have one partner so that we can prevent ourselves from getting HIV/AIDS.

**I: Uh huh!**

PI01PI10: So that we can see how our future is apart from that work.

**I: Uh huh! Is there anyone with a different opinion apart from what they have said? (Silence) okay we are moving to the next question, there are those who have not talked. [Someone clears her voice] We are moving to the next question, now we are asking that what do sex workers normally do with their money, like things they buy on a daily basis with their money. Uh huh, number nine (crosstalk)**

PI09: Those who do sexual worker [sex work]

**I: Mmh.**

PI09: Clothes

**I: Uh huh!**

PI09: She wants to at least buy a clothes and it should be short so that those whom she wants to engage in sex with can see her, how she has put on a short cloth but it reveals the whole of her body, like shoes, just things like that, oil, she wants to apply make up nicely.

**I: Mmh.**

PI09: She does make-up, so she buys clothes, make-up, things like oil

**I: So clothes, shoes and make-up can cost how much if you add all?**

PI09: Very expensive, it can total to approximately two thousand shillings

**I: Uh huh.**

PI09: Mmh.

**I: Another person, what do you buy on a daily basis with the money you have? Uh huh, number four**

PPI04: Let me say, sometimes you are from work and you have come back with even five hundred shillings and you know that you don’t have food in the house, the kids should bathe and things you use like oil, you should also buy an attractive cloth such that when a person looks at you at least he can choose from a group of people.

**I: How much can it cost in total? The total cost of food, clothes, and make-up?**

PI04PI04: Two thousand shillings and above.

**I: Two thousand shillings and above, uh huh, any other person? (Silence) what do we buy on a daily basis**

PI0PI02: I must make sure my purse has trust (condom)

**I: Uh huh.**

PI0PI02: Or, nowadays we have female trust (condom)

**I: Mmh.**

PI02: There are those for male but mostly we use those for men [male condom] such that if you get someone you tell him that I am this way and I have carried this thing, but there are some people who don’t want

**I: Mmh.**

PI02: Yes, he wants to use you that way (have unprotected sex with you)

**I: Mmh.**

PI02: And after using you that way he wants, you don’t know how he is [status], so there are some drugs we are being given, we were in a study and we were given, it’s known as PrEP. So if you know you are going to meet someone who does not want to use a condom, you take that drug and then you just work, now you are safe.

**I: Uh huh, so apart from condom, is there any other thing you use which you use your money to buy on a daily basis?**

PI0PI02: P2 (emergency contraceptive pills)

**I: Mmh.**

PI02: P2

**I: P2, uh huh.**

PI02: It is a pill you take to prevent pregnancy.

**I: Mmh.**

PI02: Yes

**I: So how much can you use?**

PI02: Around three hundred shillings for P2.

**I: What about condom?**

PI02: Condom is two hundred and fifty shillings

**I: Uh huh, any other person? What do you use your money to buy?**

PI08: We have to eat because when you go and meet him, that is the time he can buy you food, but when you come back in the house, you have to look for what to eat in the house, this house where I am staying, rent you have to pay, small items for the house you also have to buy with the same money because that is the work you do.

**I: Uh huh.**

PI02: Mmh.

**I: So the total for rent, food and the small things which you have mentioned (crosstalk) how much can it cost you in total?**

PI02: In a day or in a month because

**I: In a day**

PI02: In a day

**I: Mmh.**

PI02: So in a day like rent we cannot pay in a day but now the little that I am given I have to save little amount so that I can find a way to pay for my rent and electricity, so in a day I should be spending two thousand shillings.

**I: Mmh.**

PI02: Yeah.

**I: Any other person? Everyone is going to tell me what she buys with her money (people laughing) with their prices, those who have talked will not talk. Those who have not talked, I want to hear from you.**

PI01: When you are from work sometimes you are tired, somethings…okay you have got money but you have to do calculation daily, the way you will leave the house and even when you come back in the evening because mostly, maybe you can reach the house and you get a phone call that you are needed again to go out, so being that you are looking for money, you will just have to go. You know that such a thing I should also leave in the house or such money or amount I have to leave in the house. We have not mentioned about hair because all of us here are making our hair, I have not seen people who…those who shave here are few, you have to go and make your hair so that at least when he sees you, he knows that this person whom I got…even if you quote your price, he sees the type of hair style you have (someone laughs), even if you tell him to give such amount of money, the money that I have used to look this way is such an amount mostly if he is a new person and not a regular person. Because there are visitors and regular people, so when you are quoting the price when he asks you how much it costs, you tell him to give you two thousand shillings or one thousand five hundred shillings if you want us to engage in sex. If you want you can give me but if you don’t want, so he can also bargain because they are also good at bargaining. He can bargain maybe you have quoted two thousand shillings and he takes you back to one thousand shillings, so you will have to agree that just give that one thousand provided I don’t go back to the house without anything.

**I: Mmh, so you have said that there is an amount of money that you leave in the house.**

PI02: You have to leave some money in the house.

**I: How much?**

PI02: You have to leave something, at least you should have some savings of even five hundred shillings in the house.

**I: Uh huh, then how much can you spend on making your hair?**

PI02: When you go to the salon it is about, when you want to weave your hair you must have one thousand five hundred shillings.

**I: Is okay, so is there someone who has something? I have realized that some people do not eat, (people laughing) there is nothing they do with their money. Meaning for them there is nothing.**

PI09: When you are going there you don’t walk, maybe it’s a (long) distance, you are going far, fare

**I: Uh huh.**

PI09: Maybe you are from here and you are going to town using a motorbike town because you are trying to get there early.

**I: Mmh.**

PI09: So that you can book a queue early, fare to and from (town) is roughly four hundred shillings.

**I: Mmh.**

PI09: Mmh.

**I: Uh huh.**

PI10: (Someone clears her throat) There is also nails, it needs nails that are attractive which when you put like this [demonstrating]

**I: Mmh.**

PI01PI10: He sees something that glitter which when he looks at it he is attracted to it. so we also spend money on our nails

**I: Mmh. Uh huh, another one**

PI05: I have not heard these people talk of earrings (respondent chuckles), you have to buy earring that suits your cloth well and a good shoe that suits your cloth

**I: Uh huh.**

PI05: You also have to eat well you go there when you are strong.

**I: Mmh.**

PI05: And you use fare.

**I: How much does all that you have mentioned cost?**

PI05: About four thousand (shillings)

**I: About four thousand (shillings), number three, I have not heard your voice (someone laughs)**

PI03: What I do with money, you can come from there and buy food (noise from the background), maybe you left a child in the house, you buy food or you buy clothes for the child. You have seen a good cloth you can take for him or her (buy for him or her) to make him or her happy, even if you will go out again and leave him or her, he or she will think that my mother has gone out and she will bring me another cloth. So when you leave him or her, she or he cannot cry, he or she becomes happy. So the money I can use to buy him or her a cloth is one thousand (shillings)

**I: It is okay. So we want to look at things which we buy once in a week, you say what you buy once a week and its price. Okay, who will start? Let’s go (do it) faster so that we can finish (crosstalk)**

R: Things like food or anything?

**I: Anything, something you buy once a week, (crosstalk) that is what I want you to tell me.**

PI09: Make-up kit.

**I: Uh huh, how much can it cost?**

PI09: Roughly two thousand (shillings)

**I: Two thousand (shillings)**

PI09: Yeah.

**I: Uh huh, another one?**

PI06: (Noise from the background) Like cloth, shoes, earrings, I can buy things used for making hair, and that can cost around four thousand shillings

**I: Uh huh, any other person?**

PI03: Hair, clothes, shoes and food.

**I: Total?**

PI03: Five thousand (shillings)

**I: Five thousand, another person?**

PI02: I buy, like I have to make sure that I have condom and P2, so condom and P2 is around six hundred.

**I: Uh huh.**

PI02: Because nowadays there is a condom that cost three hundred and it contains six sachets (vehicle hooting)

PI04: I am say that I don’t know whether these clothes we put on without under wears or pants (someone laughing). You must have a pant and a bra so that even when you undress before somebody, he sees that this person whom I got is neat we are no taking off sack (referring to pant). We have to put on a pant and you wear a pant matches with your bra.

**I: Uh huh.**

PI04: So that even if he looks at you he will say that he got a neat person.

**I: Mmh.**

PI04: A pant alone… a bra is five hundred (shillings), a pant alone is five hundred (shillings) that is (a total of) one thousand shillings.

**I: Mmh.**

PI04: Yes

**I: Okay, another one? What you buy once a week?**

PI08: I buy (body) oil which can cost one thousand five hundred shillings, you have to apply oil that has a good scent when going to meet him after bathing (noise from the background).

**I: Mmh.**

PI08: So even if you are walking round the smell that comes from you is nice

**I: Uh huh, is there anyone who wants to add?**

PI02: You can go to that work and you line up but you end up not getting a client, so that is a challenge that exist.

**I: We are going to talk about that later, there are questions here which some of you will (vehicle hooting) say that it is not easy (someone laughs). It’s when we want to start asking questions, we are still climate setting, right?**

R: Yes

**I: So, what do you buy using money once in a month and their prices?**

PI01: What we should make sure first, because people despise us because of the kind of work we do

**I: Uh huh.**

PI01: Maybe it’s your neighbor, maybe the neighbor knows that kind of work you are doing

**I: Mmh.**

PI01: First, what we normally do is that you clear rent, house rent, for me the house where I stay, I pay three thousand five hundred shillings

**I: Mmh.**

PI01: Even if I go to my work, the neighbor cannot despise me, he or she knows that I do not want the landlord to come and knocking at my door.

**I: Mmh.**

PI01: I clear with the landlord first, so the rest I will figure out later.

**I: Mmh.**

PI10: In a month, I make sure I save something (money) small then I go for small shopping (meaning buying a few things) which I leave the kids with. So within the month I move around doing my work but there are things that are necessary, those that are compulsory which I have just left in the house which I can use, so the child doesn’t have to borrow something like a soap, the child doesn’t have to go to the neighbor to borrow a soap those are the kind of things that I just leave (in the house) apart from daily food. So that one can cost three thousand shillings.

**I: Uh huh (crosstalk).**

PI05: I can save money for the children which I take to school, I go and pay for them. That money can be around four thousand (shillings)

**I: (Silence) is there anyone who wants to add?**

PI01: What I can add is that we must have savings in the house, such that when you are from work at least you can take two hundred shillings daily and put aside.

**I: Mmh.**

PI01: So that two hundred shillings is a must because sometimes the weather is, like it is now and you had planned (or) sometimes you are preparing to go out (or) maybe you didn’t get a customer who calls you, you have planned to go out and it starts to rain, so your two hundred shillings which you kept can help you

**I: Mmh. I have noticed that some people buy nothing once a month, okay, which ones can you buy once a year? As in you can buy it in what quantity? As in it can be bought in what quantities, it is something that you buy once a year or in one academic term?**

PI07: What I can buy once a year is school uniform for the children, text book, and I can pay money (school fees) once a year. And the things which I can buy in a year, okay when I refer to household items that I can buy once a year

**I: Mmh.**

PI07: (Vehicle hooting) I can buy even a sufuria [sauce pan] because you know for us we don’t eat mostly in the house.

**I: What is the total, all those things?**

PI07: Total, I can spend ten thousand

**I: So you have said you don’t eat mostly in the house, meaning?**

PI07: We, especially children… [Noise from the background] for us sometimes you have gone out and you come back late, maybe you have found that the children had eaten or maybe you ate outside [where you are coming from]

**I: Uh huh, another person [someone clears her throat]**

PI10: There is a way things…. There are items for kids like a cloth but it can repeat itself later it is not a must

**I: Mmh.**

PI10: There are classic clothes which you can buy for a child once a year and it is normally at the end of the year in December that is when I can buy for my child a classic cloth.

**I: Mmh.**

PI10: There are shoes that I can also buy which last for long such that I will not buy them again like wedges, high heels. Those ones I don’t buy those ones continuously but there are some which I add sometimes

**I: Mmh.**

PI10: Mmh.

**I: Total?**

PI10: It can cost even seven thousand if I save well.

**I: Mmh, another person? What do you buy once a year or per term, uh huh?**

PI04: What I can buy once a year is like paying school fee, sometimes there is a child whose school uniform is old and I can buy it for the child together with text book or incase some money is needed in urgently school I can

**I: Mmh.**

PI04: It is about three to four thousand.

**I: Uh huh, another person? If there is none, we will move to the next question. Is there anyone who wants to add? Let’s say most of women who engage in sex for payment normally get the money they use from where**

PI06: Mostly women to engage in sex for payment, all the money we that we always get for use come from men because we depend on those men, if we go and have sex with them that is when we can get money that we can use.

**I: Mmh.**

PI06: Because that is the kind of work we depend on, so that is where we get money (noise from the background).

**I: Another person?**

PI10: If I may emphasize, all this money just come from men but sometimes there are customers, we say (call them) customers or clients whom we are used to and you find that we have worked with them for long so you can ask him [for money]and he gives you without you going. So those are our sources of money.

**I: Uh huh.**

PI10: You can time when someone, like some working class you find that its end month and you call him and he supports you even if you don’t meet him, so they are our sources of finance [income]

**I: Is there anything different from what they have said or answered?**

P: There is none

**I: There is none, so apart from that… that is the main source of income, right? Is there any work you do to get money apart from that of having sex?**

PI02: For me sometimes a certain woman normally calls me to go and stand in for her in a job of frying potatoes. I help her and in the evening she gives me two hundred shillings which I take home, any work that I get be it helping with farm work, I go to the farm and help someone with farm work and he or she pays me 200/= that I use in the house.

**I: Uh huh.**

PI10: Apart from these men, there is way sometimes…although I go there to meet them (men) but sometimes it is not easy [there are no clients] another job that I normally do I go to a club and dance nicely then I realize that some small amount of money has been paid, sometimes I can also be called to offer services (bar attendant) if I offer services I am given some money.

**I: I am coming back to you, number two, you have said that you do help a certain woman to prepare samosa?**

PI02: Yes

**I: You can also do farm work, so you can always get money from these job.**

PI02: If I go and help her, in a day she gives me two hundred [shillings] and helping with farm work is also two hundred (shillings)

**I: So you get how much in a day?**

PI02: I a day I get four hundred [shillings]

**I: Okay, number ten has also said that if she goes to a club, she can dance and she gets something small and she can also offer services, so in total how much are you given?**

PI10: So in terms of dancing it varies, sometimes I have gone there and danced but there is nobody who…maybe during weekdays there are no people who can fund [pay] you well so you find that you are given even one hundred (shillings), things are not going on well it is Monday [people laughing)]

**I: Mmh.**

PI10: For offering services, you can be given only when there are many visitors (clients or customers), of which I will not finish so I go there with a goal that incase I get (a client) I won’t be able to continue with this job of yours, so if he or she tells me that it is okay you just work for me until that time I will pay you this, mostly they just give me two hundred (shillings)

**I: Mmh.**

PI10: Because I would work till morning, if I get I go.

**I: If you get what?**

PI10: If I get a customer, I go.

**I: It is okay, is there anyone who wants to add?**

PI08: Yes, sometimes you find that work (business) is not doing well, I will just echo what my fellow has said, we now go to clubs where music is played, we can dance and the way we have dressed even if someone does not want to talk to you, he will talk to you and make stories with you, he can send to the waiter and sometimes he tells you to keep change. So sometimes that is what we can get.

**I: Mmh, how much can you get?**

PI08: You can get five hundred or one thousand (shillings)

**I: Okay**

PI08: Yeah

**I: Is there any other answer?**

PI01: Apart from that, work is work, [not choosy on which work to do] it reaches a time you can feel that… during a season like this of rain, in fact this rain is really annoying me because when you want to go out, you are ready you want to go out and that is the moment it starts raining, will you go to the field when it’s raining? Sometimes when it starts raining, you will be forced to…or one of your neighbor ask you whether you can be for him or her clothes, if he or she ask you whether you can wash for him or her clothes and you tell him that you can, how much and you tell him or her to give you even three hundred shillings. How many clothes (quantity) and then you bargain and he or she gives you money and you wash for him or her clothes.

**I: Mmh.**

PI01: Okay other jobs we can go to like working on construction sites, like now that it has rained this evening and I had planned to go out, I want to go to =Nakuru= but I know it can start raining and I won’t go. So sometime if there is work at a construction site where work is going on somewhere we are out going (the construction site).

**I: Mmh.**

PI01: I am out going (there), I personally do go to work at construction sites, so when I work in those places I am give four hundred or even five hundred shillings which I take home.

**I: Mmh.**

PI01: Yes

**I: Uh huh, who wants to add something? (Silence) there is none?**

P: Yes

**I: Okay, why do women who engage in transactional sex normally buy those things that they always buy? As in why do you buy these things that you have mentioned? Why do you buy them? Uh huh.**

PI06: The main reason why buy make-up is for you to look smart because you cannot go there, you have lined up yet you have applied milking gel or oil, nobody will see you. You have to apply that milking gel or oil and even if you have applied milking gel or oil you also apply sexy (type of powder) and then your face will look nice, you also apply lipstick

**I: Mmh.**

PI06: Then you will at least look nice, maybe you can get an income (someone coughing)

**I: Mmh.**

PI06: So those thing that we try to apply or the things we try to buy so that we can look smart on the work that we normally do.

**I: Uh huh.**

PI06: Yes, that is it.

PI09: There is something that is out (noise from the background) that kienyeji girls [girl from the village and the digital, so you know for us sex workers, you have to dress nicely so that whoever wants to go and have sex with you sees the way you are smart, he knows that your price is high. So you say you use money to get money, so you just have to buy a nice cloth that fits you such that even if someone sees you, that person even if you quote five thousand shillings, I know that we are not going to ague and that is what he gives you because you have dressed nicely. So you use a lot of money to buy a cloth so that someone can at least give you good money.

**I: Mmh.**

PI09: Mmh.

**I: So that cloth can cost how much?**

PI09: It can be a ready-made (new) cloth or a nice second hand cloth at around five hundred to one thousand (shillings).

**I: And your make-up, how much can they cost?**

PI09: Make-up, in most cases we normally buy…if you buy them in a week, you buy the whole packet and it can cost one thousand five hundred shillings.

**I: Mmh.**

PI09: One thousand five hundred shillings, it can cost one thousand five hundred shillings

**I: Uh huh, another one?**

PI05: You know if you go there you must look smart, you have smartly done your hair and you have put on a bra plus and a pant that are also very smart, such that if you undress before him,

**I: Mmh.**

PI05: He has appetite Has the urge to engage in sex]

PI10: That is, I am only emphasizing

**I: Mmh.**

PI10: The main reason why we put on these things is to be attractive and in most cases we want to put on things that makes us half naked for that person to see the physical appearance, which is let’s say the mind

**I: Mmh.**

PI10: It’s not about issues a phone, if you are going for sex work you be half naked so that when he looks at you he has already seen what he wants. So the main thing is that we want to be attractive.

**I: Mmh.**

PI10: Mmh.

**I: When you become attractive, to what extent will you spend, how much money will you use?**

PI10: Okay, being half naked, it will depend with an individual

I: Mmh.

PI10: There is a time you go to= Kibuye= (market) and you know at that time you get clothes for kids, so you go and look into second hand clothes very well. So something that will be expensive and is upon you is shoes, now shoes plus these clothes can maybe cost up to one thousand five hundred shillings, if you put on nicely it can cost one thousand five hundred to two thousand five hundred shillings.

**I: Another person? (Silence) Why do we use our money to buy those items?**

PI01: Those items, first of all we must use them because we asked about items that we can have in the purse, because in our purses, when you are going for sex work you cannot go with one cloth, you can even go with two clothes. Maybe the cloth you wear from the house is different because I cannot come from the house with short clothes, no, in the presence of children, you also have to accord children respect in the house. The children also should not know that this is the kind of work that our mother is doing because you are giving a bad example to the children

**I: Mmh.**

PI01: So I go out dressed this way but I know very well that the cloth that I am going to put on at work place is there

**I: Mmh.**

PI01: I must have make-up in my purse, I also must have a perfume, meaning I have carried a purse I am travelling and after I shall have finished my work, I have gotten a customer and finished with him, I get into a bathroom again and take bathe. So these items that I carried which include oil, perfume and the rest, I have them in my bag, now I am starting again such that when I will be coming from that place, any smell…we had finished with this man, I don’t know him and he too does not know me, I am back to the field (sex work), I am new (clean)

**I: Mmh.**

PI01: Yes, so these items I must have them in my hand bag,

**I: Mmh.**

PI01: So if you include all these oil (body oil), perfume, all these things because you will not go and buy caro light (body cream), you want to smell caro light yet your fellows smell maybe rosemary, I don’t know what

**I: Mmh.**

PI01: You are also competing because competition is always there, people normally compete, the perfume she used it the same one I am going to look for

**I: Mmh.**

PI01: Now you are going to look for a similar one, that perfume alone can cost you one thousand shillings or eight hundred shillings because nowadays we have those one that can be refilled

**I: Mmh.**

PI01: So I must have them.

**I: Mmh.**

PI01: All these clothes if you put them together, you match the cloth that you put on with shoes and bra and also what you have put on inside (meaning pant), those things are uniform they match. All of them can cost thirty-five

**I: Uh huh, thirty-five what?**

PI01: Thirty-five hundred (three thousand five hundred shillings)

**I: Hundred, uh huh! So you have talked about things you buy and you on yourself (body), right?**

PI04: Yes

**I: Now, things that you buy for other people can be which ones? (Flipping of papers)**

PI04: In terms of food, children will have to eat and even you in case you have not gone to work, you will also need something to eat, and you will have to buy food to eat to sustain you

**I: Mmh.**

PI04: You have to pay rent because if you don’t pay rent, you will be locked outside. You have to pay fees also because children are going to school, if you don’t pay fee for children, they will be sent from school.

**I: Now we are asking about things that you buy for other people, we don’t buy rent, right?**

P: Yes

**I: We don’t buy school fees, things you can buy for someone, and it can be your child or another person, right?**

P: Yes

**I: They are things like?**

PI05: I can buy even a goat for my parent which he or she will remember me for (crosstalk)

**I: At how much?**

PI05: Three thousand shillings for the goat one thousand shilling for the chicken, a total of four thousand shillings.

**I: Uh huh.**

PI05: Yeah.

**I: Another one?**

PI08: I can buy a rapper for my grandmother, I can buy sugar and take to her, I can buy her shoes then I take to her (someone clears her voice) maybe someone is selling the items at your place, maybe she lacks money and you can send her.

**I: Total?**

PI08: In total it can cost me around five thousand shillings.

**I: Uh huh, number two**

PI02: I make a tendency that every month I must have four thousand shillings which I buy for my mother things then I take to her

**I: Mmh.**

PI02: Things that would last for a month and then until next month.

**I: Uh huh.**

PI02: Yes

**I: Number three do you have something?**

PI03: If I may emphasize

**I: Mmh.**

PI03: I can save money which I use to support my mother, I buy her things or I send her or even if I have a brother who is still in school, I can even pay his school fees.

**I: Mmh.**

PI03: Yes, per month it can be around five thousand shillings.

**I: It is okay, another person? (Silence) Should I continue?**

P: Yes.

**I: So do women who engage in sex for payment do save? Do you save?**

All: Yes

PI06: It is something hard and it is also easy to save, depending on

**I: Mmh.**

PI06: It would be nice if we save but again it is hard to save because sometimes in a week you even have sex with four people in a week and these four people who you have had sex with, maybe you have had sex with these four people but the needs you have in the house are many compared to the money you got, so how you want to help with these things is difficult. Meaning that money, you will end up that the month has ended but you have not saved anything not even a cent because maybe if your child is sick, you are the doctor (meaning she takes care of his or her treatment bill), sometimes school, you are the teacher you are the one who takes to the teacher (meaning she pays for anything needed in school). His or her clothes are old, you are the one to buy, so you find that saving…it is a month end and you have not saved anything not even a little depending on what you have in the house because you are the key and you are the padlock. [she is the one to provide everything]

**I: Mmh.**

PI06: So saving is difficult, it is little and we only save a little and sometimes you can save a little and it reaches a time that you withdraw (the savings) again and use it then you remain with nothing

**I: Uh huh.**

PI06: So saving is not easy

**I: Uh huh, another person? Are you saving?**

PI01PI10: As for me saving is not easy t

**I: Uh huh.**

PI01PI10: It is just from hand to mouth unless you acquire a lot amount of money suddenly then I take it and buy something with it but taking it and I cannot take it and save it, that one I cannot maintain. I can only save it for a short duration then I go and withdraw or take.

**I: Mmh.**

PI10: Because I cannot have it for long, so it depends, there are certain months which you can say you have saved and there are some which that savings cannot be there, so saving is difficult for me.

**I: You have mentioned that you acquire a lot of money suddenly, what does it mean?**

PI10: Meaning, how do I put it (people murmuring from the background), you get a rich man who gives you a lot of money.

**I: Mmh.**

PI08: When it comes to saving it is difficult because sometimes it is not good (the business is not doing well) and our needs are quite a number and when I am out, I go out after doing make-up nicely, but I go and sometimes come back with nothing, I am not able to get a customer. So maybe whatever you had however little it could be, you go and withdraw and eat it (use it to buy food) because you cannot stay hungry, you have to eat and get satisfied for you to be strong to go and play that game (have sex), you cannot go on an empty stomach. In terms of saving it is difficult.

**I: Mmh.**

PI08: Mmh.

**I: Another person? Do you save? (Someone whispering) you don’t save (someone chuckles)**

All: Yes

**I: Okay, now for those who save, why do you save? Why should you save?**

PI06: The reason why we can save, which is good if we save for an emergency that can come up

**I: Uh huh.**

PI06: Because sickness is not something that knocks, sickness is something that just comes up, so that is what savings can help us with

**I: Mmh.**

PI06: Yes.

**I: Uh huh.**

PI06: Or even any problem can come up, in most cases we can save for problems because it comes up without…it does not notify you that it is coming

**I: Mmh.**

PI06: So that is what the savings can help us with.

**I: Mmh.**

PI06: Yet saving is difficult for us (people laughing)

**I: Okay, uh huh. Now, in case you saving, how frequent would you save?**

P: In case we are saving

**I: Mmh.**

PI06: It would be nice because our work…if it is a good job, in a month when the business is doing well, you can go and after two days you get a customer, so it would be nice if we save. At the time you are going when you have found (a customer), you should save something little

**I: Mmh.**

PI06: Mmh, it is good if you have your small savings, so it would be nice if the savings…you should put aside something small in a week which you should save if your income is good.

**I: Another person?**

PI07: We save because…we should save because of school fees, we should save because, that is when you can buy yourself something. We should be saving then you buy even a goat

**I: So how frequent can you save? If you figure out how frequent can you save?**

PI07: The frequency depends because sometimes the business is doing well and sometimes the business is doing poorly

**I: Mmh.**

PI07: So it depends on how the weather is

**I: It’s alright.**

PI10: It is good if we save although it is difficult. If we are saving, it would be nice if the frequency at which someone saves, we should say that in a month you should have something in your account like roughly two thousand shillings.

**I: Mmh.**

PI10: Of which sometimes we do not achieve because there are different circumstances which you are supposed to attend to using money like the ones which…I am emphasizing my fellow’s point

**I: Mmh.**

PI10: You mentioned that sickness or accident because the rest are there, you can plan for them. So even if you can…suppose that work is continuous being that we deal with dressing code most of the time

**I: Mmh.**

PI10: It would be nice if we save so that if a person can open a boutique, it can boost her so that when she does not go to work, these things are here you can supply to your fellows because there are people who we know each other through that work (sex work)

**I: Mmh.**

PI10: So saving is good.

**I: So, in this case everyone is going to respond. In case you are saving, how much do you think you can save in a weekly basis? Number one.**

PI01: In a weekly basis, the money which I can save

**I: Mmh.**

PI01: If the business is doing very well for me such that I don’t lack a customer, I can save around five hundred shillings

**I: Uh huh, number two?**

PI02: Me I can save seven hundred shillings.

**I: Uh huh, number three?**

PI03: What I can save, I can save around eight hundred shillings.

**I: Uh huh, number four?**

PI04: I can save five hundred shillings.

**I: Number five?**

PI05: One thousand shillings.

**I: Number six?**

PI06: Five hundred shillings.

**I: Number seven?**

PI07: One thousand shillings.

**I: Number eight?**

PI08: Five hundred shillings.

**I: Number Nine?**

PI09: Five hundred shillings.

**I: Number ten?**

PI10: I have a good appetite I can only save two hundred shillings (people laughing)

**I: What do you mean by you have a good appetite?**

PI10: I have to eat healthy because that is work

**I: Mmh. I am also asking whether there is a character which is known that women who always have sex for payment and save have. I there a character which is known that women who have sex for payment have? Character, characteristics (silence). A known character?**

PI01PI10: There is. For us we don’t fear, how I can put it (crosstalk), we are empowered meaning we don’t care. If you find me at work, I do it to my perfection I don’t care who you are because I came to work.

**I: Uh huh.**

PI01PI10: So that is the character which I think most of us have unless you are new in this business. So that fear…we don’t fear (meaning they don’t care)

**I: Uh huh, you don’t fear. Another person?**

PI06: I am trying to emphasize what number ten is saying, the character which most of has have is that we don’t care

**I: Mmh.**

PI06: Because if you are there you must be a person who doesn’t care that is when you can get a customer but if you care or fear then you can go daily and get nothing. So the issue of not caring is the character have

**I: Mmh.**

PI06: Mmh.

**I: So the character I am asking about is that if you see a woman who have sex for payment, how will you know that this one is saving?**

PI09: You can find that after every month she must buy something in her house.

**I: Uh huh.**

PI09: So you will know that she is doing that work but she is smart, she is saving something (money), at the end of the month she buys something which will help her remember that she was doing a certain work and that is why she bought such a thing

**I: Uh huh, another person? How will you know that someone is saving? Number four**

PI04: She is someone whom even if you go to her house, you just see the kind of life she is leading, even how her kids are, their dressing code and everything, life style

**I: How is it?**

PI04: It is of high class.

**I: Mmh.**

PI04: Yes

**I: Uh huh, another person? Women who save, what is their character? How will you know?**

PI03: If I may emphasize on what my fellow has said, if you go to their houses, the way she has arranged the house will make you know that she is saving

**I: Mmh.**

PI03: Because of the way she has arranged her house, the kind of seats she bought, where she sleeps, the house she has rented, will make you know that she is saving.

**I: Mmh. Uh huh, another person?**

PI02: She don’t like begging so much

**I: What?**

PI02: Begging in that she goes to a neighbor to ask for something, she tries so hard to have that thing that she may go and ask from the neighbor

**I: Mmh. Uh huh, number one do you have something?**

PI01: Begging, me I do not beg because I get food from my work

**I: Mmh.**

PI01: And even people from the plot (neighbors) know that I do that kind of work

**I: Mmh.**

PI01: Don’t come to me because you have known that I do sex work, so you cannot ask something from a sex worker, how do you even ask, all my things smell sex work

**I: Mmh**

PI01: My things [vagina] smell sex work (people laughing)

**I: Smells what?**

PI01: Even if you come to ask for salt, it smells sex work because I got it from sex work. That is how they view us

PI01: Yes, so don’t ask me and I will not ask you too.

**I: Mmh.**

PI01: I have to try my level best that the kind of life you lead, you who has a husband, I am better that you by far

**I: Mmh.**

PI01: Yes, that is what we normally tell them. You and your husband who you stay with (someone laughing), I am better than you by far

**I: Mmh.**

PI01: I must have everything, even if I have gone shopping, is I carry them as I enter my house

**I: Mmh.**

PI01: So I have to save because we even have groups (we are members of self-help groups), even the way we are, we have groups as sex workers.

**I: Mmh.**

PI01: Those groups also there is an amount of money which we must save, in case of anything even is my fellow is having a headache such that she cannot get up from her bed, we can figure out on how we can support her. Maybe she is on her bed, she is very sick and she does not have money, so we decide that the little money we had saved, we can use it to take her to the hospital

**I: Mmh.**

PI01: So that is the meaning of saving, we do save. Even if it is 50/=

**I: Mmh. It is okay (crosstalk), and is there a character which is known that women who have sex** **for payment who do not save have? A character for those who do not save, what is their character?**

PI09: Those who do not save

**I: Mmh.**

PI09: They always beg, she mostly complains that she is suffering

**I: Uh huh.**

PI09: For how long will she suffer, so her life style is not good, in most cases she is begging.

**I: Uh huh, another person?**

PI02: You find that she stands at her door saying “to day I have not seen, I am suffering, today I don’t think I am okay.

**I: Mmh.**

PI02: She is standing by her door announcing to people (participant chuckles), today I am not okay, I am suffering here in =Kisumu= (people laughing)

**I: Another person?**

PI01PI10: Their character is that if they see someone’s customer, the want to highjack because they do not have

**I: Mmh.**

PI10: For example, me I can hike (highjack) someone’s client because I don’t have. I depend on that that is my hit point

**I: Mmh.**

PI10: So I have to try harder, if this person cannot see well, I have to try so that I would be the one to chip in so that I can have this man

**I: Mmh.**

PI10: And if I know that he was looking for someone, I would be the one going there because I don’t save

**I: Mmh.**

PI10: Mmh, so we do try, those people who do not save try to work hard. You can even bother someone by calling him so that you can meet him and then he gives you that little amount

**I: Mmh, is there anyone who would like to add? (Silence) is there anyone who would like to add?** **(Silence) there is none. Okay, I am also asking, for those women who have sex for payment and do save, why do they save? We had responded to that**.

**All: Yes**

**I: So, what makes it easier for them to save? (Someone whispering) Why is saving easier for them?**

PI10: You can find that those who save have part time business

**I: Mmh.**

PI10: Maybe they sell things, so they do not depend on that work (sex work) so much although they are doing it but they don’t depend on it so much

**I: Mmh.**

PI10: So if money from the other source (sex work) backfires or she does not get, she is in a good position because she has this other business, she goes and takes money from there and save in a bank, the money which she has made from sales, so she does not have to spend the whole of that money.

**I: Mmh.**

PI10: Even if she goes there and gets (a client), she can just spend that money and the one she gets from the business (part time business) she can save

**I: Uh huh.**

PI10: Yes

**I: Uh huh.**

PI03: What can make it easier for her to save, maybe she gets an urgent call from school that her child is sick, she will go and pick the child and the money which she had saved, and she can withdraw it and use it to take the child to the hospital.

**I: Mmh.**

PI03: Maybe the child has been sent (from school) and she does not have money, so the money she had saved, she can give it to the child to take to school

**I: Mmh. Uh huh, another person? What makes it easier for them to save? For them who save (silence). So, for those who don’t save, what challenges can we experience?**

PI08: I had mentioned that poor performance of the business is the reason why we cannot save, sometimes the business is doing badly, the money is also little and our needs are also many. Because mostly we are supposed to be clean, you are smart all the time

**I: Mmh.**

PI08: So you know for you to get money to save is a challenge.

**I: Uh huh.**

PI08: Yeah.

**I: Another person?**

PI02: Sometimes you have had sex with someone and he tells you that he will send you (money)

**I: Mmh.**

PI02: He lies to you yet you don’t know him, so you know you have lost that money like that, so you will not save, you will not get it

**I: Uh huh.**

PI10: In short most of the time we don’t save because sometimes, it depends on how you get these people

**I: Mmh.**

PI10: So, shortage of clients is what makes us not to save, but if you get frequently we can just save

**I: Mmh.**

PI10: The main reason is that there are few clients

**I: Uh huh.**

PI10: Mmh.

**I: Another one? (Silence) is there another one**

P: No

**I: Now, you have talked about shortage of clients, how can we overcome this challenge of shortage of clients? What can we do?**

**P**I04: So for example you have gone sex work and there are no customers and for part time job, you have a business or you have left someone runs the business for you somewhere or you have a salon

**I: Mmh.**

PI04: Can support you so you cannot rely on (sex work), you don’t have it in mind that today I did not get a customer, I do not have money, and you are just comfortable (because) after all I know I will buy whatever I want

**I: Mmh. Uh huh, another one?**

PI06: What we can do about the shortage of clients, is that we will be forced to have another business which we can rely on apart from that (sex work)

**I: Uh huh.**

PI06: Because when there is shortage of customers like now customers [clients] are few and we cannot lie to ourselves

**I: Mmh.**

PI06: Currently they are few and when they are few like now and we also have needs, if you have another business aside, it can help you. So you also check on that business too.

**I: Mmh.**

PI06: So it would be nice if we learn on how we can start saving and how we can start another business in future, so that if we are on this end, you do not fully depend on sex work because you have another source of income which you can rely on.

**I: Mmh.**

PI06: That is my view

**I: Is there anyone who would like to add? (Silence) is there anyone who would like to add? (Silence)** **there is none. Now we are saying, do women who have sex for payment who do not save, why do you** **think they don’t save?**

PI06: Just because of how we have talked, how we have said it

**I: Mmh.**

PI06: The reason why we don’t save is low income.

**I: Low income**

PI06: Low income is the reason we don’t save.

**I: Uh huh.**

PI06: Yes

**I: Another one? Number ten is talking besides me, I don’t know whatever she has said**

PI10: I am emphasizing on her point, it is few number of visitors (clients) and lack of being popular, there are those who started earlier, we are not in the same level.

**I: Mmh.**

PI10: There are those who started earlier, they are popular, there are those who know them when we go for sex work, there are those who know places where they stay

**I: Mmh.**

PI10: Some people also call them, so the reason why we cannot save is lack of being popular, how can I put it, the way you are not familiar (popular)

**I: Mmh.**

PI10: Familiarity hinders you from saving, you have little money as compare to those who save who got into that business earlier.

**I: Mmh.**

PI10: Mmh.

**I: Uh huh, is there anyone who would like to add?**

P: There is none

**I: Which challenges would you face in case you are not saving?**

PI06: Challenges which we face if we do not save are there

**I: Mmh.**

PI06: The challenge we face, one, we have families and how we support the families is a challenge. We also have our parents, how we support our parents is also a challenge

**I: Mmh.**

PI06: So we are forced to work extra hard for you to find a solution to the problem you are facing, so that is the challenge we face as a result of lack of saving

**I: Mmh.**

PI06: Mmh.

**I: Another one?**

PI01: The challenge we may face if we do not save, I can emphasize on what number six has said, it is true that you have a family that you feed in your house, you have not forgotten about the parents at home where they are, because the parent does not know the kind of work her child is doing, she knows that her child is just in =Kisian=, her child works in =Kisian=

**I: Mmh.**

PI01: She does not know the kind of work you do in =Kisian=. Maybe she calls over the phone, like me when it reached December 25th I knew that the market was good and I would make a lot of money because visitors (customers) are many in the village.

**I: Mmh.**

PI01: Then I receive a phone call at 4am in the morning that my mother or father is seriously sick and we want to rush her to the hospital

**I: Mmh.**

PI01: So you know if you didn’t have anything (money) that you had saved or put aside on phone or at hand, it is obvious that there is nowhere you will get it. So the two or three people who you know are the ones you will call and ask them to help you and then I will see what to do for you, because if at all I had that money at that time, I would have figured out how I could save that situation.

**I: Mmh.**

PI01: So that is the challenge I can face as a result of lack of saving and clients are also few, like now the business is doing badly and the economy is not good

**I: Mmh.**

PI01: Sometimes you take your phone to call him (client) (flipping of papers) because sometimes you are forced to call them (clients), you personally look for them because sometimes someone is quiet from morning to evening, he has not called you or look for you, so you ask yourself what you will do tomorrow when it dawns…breakfast, how I want to feed in the house, it will be a challenge

**I: Mmh.**

PI01: So you will just be force to look for them, you can find someone telling you that if you can accept three hundred shillings, just come so that we finish up with you and take three hundred shillings. You find that the three hundred shillings, if you go with it to the market, what will you use three hundred shillings to buy? They are just small items

**I: Mmh.**

PI01: So in that case you will not get money to save

**I: Mmh, so what are the advantages of saving?**

PI01: The importance of saving, if at all I could have

**I: Mmh.**

PI01: On my side (as for me), If at all the income was good, I can put a maximum of five hundred shillings in a day, I can save if I can get it in a day (vehicle hooting) that five hundred shillings is there as savings, savings cannot rot (‘hakiba haiozi’)

**I: Mmh.**

PI01: Because I know in case anything comes up, I hold on my five hundred shillings, I withdraw it and help me.

**I: Mmh.**

PI01: Because that five hundred shillings I cannot get it at that time and that is why I will not get (vehicle hooting) …because right now there are no…we do not have clients, you go there and stand on the streets and to you don’t get

**I: Mmh.**

PI01: Initially we were not going to town but nowadays we are forced to go to town, we personally look for them (clients), initially even those who pack trailers here, we were meeting them here

**I: Mmh.**

PI01: We finish up with them and go back to our houses but nowadays we have to go to town and meet them there in town.

**I: Mmh.**

PI01: So with that the challenge is that you have not saved, you have nothing you are supposed to eat, you should meet some needs in the house, kids are also in school like right now they have opened school and they are supposed to go to school. So you will be forced to try as much as you can to get money

**I: Mmh.**

PI01: The money is not there right now

**I: So number one, I am asking about saving (crosstalk)**

PI01: The importance of saving, if I have my own money which I have saved, it will give me easy time (noise from the background)

**I: Mmh.**

PI01: Because I know even if I don’t go to do that work, I know very well that I have my savings there which can help me

**I: Mmh.**

PI01: If anything comes up, the child is sick or anything because even us we always become sick

**I: Mmh.**

PI01: You can withdraw your savings and use it to support yourself

**I: Uh huh.**

PI01: Maybe you are tired because the body also gets tired to go to that work, maybe you got someone who is stronger than you, you go back to the house tired and decide that the work you did yesterday is enough and today I am not going

**I: Mmh.**

PI01: You have enough savings, you will not go again, and today let me give myself off so that my body can rejuvenate

**I: Mmh.**

PI01: But if you don’t have savings, you will have to go again which is hurting the body and overworking your body and hurting yourself.

**I: Mmh. It is okay, this question all of you will respond and those who are sleeping we will give** **you off. Where do women who have sex for payment do save? Where do you save your money?** **Everyone is going to tell me where she saves her money?**

PI02: Me I save in mshwari

**I: Mmh.**

PI02: Yes

**I: Uh huh, another person**

PI09: Chama.

PI08: Chama

PI07: Table banking

**I: Table banking**

PI06: Chama

PI05: Mpesa

**I: Number five mpesa, number three**

PI03: Mpesa

**I: Number four**

PI04: Mswari

**I: Number one**

PI01: Chama

PI10: KCB (Kenya Commercial Bank) bank, I put in mpesa then I send it there (KCB)

**I: Mmh.**

PI10: Mmh.

**I: it’s okay, the places where you save, why do you prefer saving there?**

PI01: The reason why I save my money in Chama (noise from the background)

**I: Mmh.**

PI01: In Chama…you have joined merry go round to get help, you have joined Chama to get help (noise from the background)

**I: Let’s continue**

PI01: The reason why I save my money in Chama is because sometimes there is a percentage which if you have achieved you can be given loan which you can use to attend to a certain issue that needs your attention somewhere

**I: Mmh.**

PI01: Once you are done, then you repay that loan in bits that is why I save my money in merry go round

**I: Mmh.**

PI02: The reason why I save on Mshwari, I can put it under locked saving for two months then I withdraw

**I: Uh huh, another person?**

PI10: For I save on KCB, KCB also have loans

**I: Mmh.**

PI10: So, for me if that money has accumulated to a certain amount, I can take a loan somewhere to use and pay in bits

**I: Uh huh, another person? (Crosstalk) number eight**

PI08: The reason why I am in a Chama is because when I have saved, I will not be given an opportunity to withdraw it unless it’s my turn

**I: Number four**

PI04: I save money on Mswari, in case something like sickness which can come urgently you cannot make to get a Chama to help you with that, so I can withdraw it and attend to the issue even if it is sickness, an emergency case or accident

**I: Mmh. Uh huh, another person?**

PI05: For me I save on mpesa because a have a sickling person in the house (noise from the background) so I save on mpesa and in any case she is sick I can withdraw it and help the person

**I: Uh huh.**

PI05: Yes

**I: Who is remaining?**

PI03: The reason why I save on mpesa, maybe I get a phone call that my mother is suffering and sometimes mpesa is nearer and I know the agent (number), I withdraw it direct and then I tell him or her that I have withdrawn such an amount of money and then he or she will ask me who I am, and I tell him or her that I am called so and so, then he or she gives it to me and I go

**I: Mmh.**

PI03: That is why save on mpesa

PI07: The reason why I go table banking, because I can borrow and I save my shares, as I borrow and save my shares as well

**I: Uh huh, is there anyone left? We are moving on, so do women who have sex for payment live a life** **above their standard? Meaning your income is small but your standard of living is high, right.**

All: Yes

**I: Give me responses**

PI06: For me who does that work, I can say that since I am staying in a plot, I lead a life above my standard

**I: Mmh.**

PI06: Because I do not want neighbors in the plot to gossip about me that this person is doing such a job and she live a life of lower standards or life of the same standard with us

**I: Mmh.**

PI06: So you try so hard to live a life of high standard like our client said that you live a life of high standard that a person who has a husband

**I: Mmh.**

PI06: So, we are trying so hard so that your living standard can be above that person with a husband so that your job can be respected

**I: Mmh.**

PI06: Yes

**I: Another one? Another person who would like to respond? (Silence)**

P: Pass

**I: I should pass**

P: Yes (people laughing)

**I: (Silence) you have responded to that. Now we are asking that how do women who have sex for payment top up insufficiency in a scenario that the money you get and the money you use, if they use money more than they get. Like in case there is an amount of money you used, right? And you have spent excess, how are you going to top up?**

PI02: I must make sure I get a client whom I charge good money and if he agrees then I take that money and use it to top up.

**I: Another person, what do you do?**

PI06: Sometimes you get a customer and get money which you did not expect and what you intended to use it for, because you should have another one (money) aside, it doesn’t have to be that from sex work only. You should have another one aside

**I: Mmh.**

PI06: So you will be forced to call those ones whom you have their contacts and lie to them that you are sick or even hungry

**I: Mmh.**

PI06: Yes, we know how to pretend, we know how to pretend, I am hungry (someone laughing), I am this way, he will have to give you eve one hundred shillings

**I: Mmh.**

PI06: If you put the money together it will help you to up the other one

**I: Uh huh.**

PI06: Yes

**I: Another person, what do you do? (Silence) another person? If there is none we will move on.**

All: Yes

**I: Okay, do women who have sex for payment like lend money or find themselves in other debts? (People murmuring) So you will respond one by one.**

PI01: As number one, I don’t borrow because if I come to you to lend, you will respond to me negatively

**I: Mmh.**

PI01: I don’t want someone to remind me of my job

**I: Mmh.**

PI01: Because that (job) is my husband and mother too, and that (job) is my father, so I do not borrow.

**I: Mmh.**

PI01: Mmh.

**I: So if you say that it is your husband, mother and father, what do you mean?**

PI01: I have said that with a reason (someone whispering)

**I: Mmh.**

PI01: You know if my mother was around, I would run to her for help, or father and if my husband was around, he too would have helped me somewhere, so being that they are not around so my job is all I have

**I: Mmh.**

PI01: Yes

**I: Uh huh.**

PI10: I lend look at the future, I can go and lend (taking on credit) a cloth which I have really longed for and then I put it on, it attracts me to customers

**I: Mmh.**

PI10: I get money and pay, I don’t over stay with someone’s debt.

**I: Uh huh.**

PI08: If I see a nice weave which I know that if I do (on my hair), I will make money… I also do my nails nicely and I have change style, I must make money and pay the debt

**I: Mmh, another person?**

P: It is just the same answers.

**I: It is okay. So, who do you borrow from? Where do you lend money from?**

PI07: We lend from those who sell the clothes, salonist

**I: Mmh.**

PI07: Those who sell shoes.

**I: Shopkeepers, where else?**

PI07: Shopkeepers, we must eat healthy, be it sugar you can buy there, even those things that you don’t have

**I: Mmh.**

PI07: When you wake up, you can take something nice for breakfast

**I: Mmh.**

PI07: When things are tough you can go to Mshwari (borrow from mshwari)

**I: You go to Mshwari, uh huh, where else?**

PI07: Fuliza,

**I: Fuliza, uh huh. Okay, so after lending, what do you do to pay that debt?**

PI03: If I get laundry work, I do that

P: you still do laundry work (someone laughing), if I get a customer…just like that any job that is available

**I: Mmh.**

PI10: There must be craftiness in this our job, we are also con women (thieves) here (someone chuckles), come rain come sunshine, they are people who I am used to and I also have their phone numbers

**I: Mmh.**

PI10: I will call them and lie to them then they send money, I can even lie to you that I will be coming in the evening, send me money for fare

**I: Mmh.**

PI10: That fare is gone like that, I pay debt (someone laughing) Am busy

P: You are busy (people laughing)

**I: Uh huh, another person, how do you pay debts? (Someone clears her throat) for those who lend, how do you pay back? (Participants murmuring)**

PI08: I can make a phone call that I am sick because sometimes you have their contacts

**I: Mmh.**

PI08: So you say that this brother will give me three thousand shillings, I am sick and I am at =Russia =or =St. Jairus =(hospital)

**I: Mmh.**

PI08: Babe how do you feel? I am feeling bad yet I am just in the house

**I: Mmh.**

PI08: So if he sends that money, I pay debt and eat well

**I: What if he says he want to come and see you at the hospital?**

PI08: I can tell him that where we are we are going to be tested, there is always a certain room that noise is not allowed there and phones should not ring as well

**I: Mmh.**

PI08: So we cannot be seen or visited right now, so when we will be given a chance I will let you know or I am just from the theater.

**I: Eeh.**

PI08: Eeh.

**I: You also need the money.**

PI08: Yes, it is need to be paid at the theater. So talking too much is not allowed, chatting is not allowed there

**I: Eeh.**

PI08: Yes

**I: It is okay, now, what do women who have sex form money usually do to increase their income? What do you do to increase your income?**

PI09: If you are someone who saves, you can start a business to increase your income.

**I: Mmh.**

PI09: Mmh.

**I: Uh huh.**

PI06: Ways of increasing our income, like myself

**I: Mmh.**

PI06: You have to apply make-up, I dress nicely so that I can look classic (nice) and be attractive, in that case I will get good money and my income will increase

**I: Mmh.**

PI06: So the class (level) I was in, I upgrade and move to the next class (level) when my income increases

**I: Uh huh.**

PI08: What I can do is…..sometime I use parking … thirty thousand, so maybe most of my clients (male partners) take alcohol there so they see that they have had sex with me so the next day I’m in =Ciala resort=, =Havannas=, nowadays =Havannas= does not exist, I’m in= Naselica=, I’m at =samba= club, I’m at =signature=, so I go to different places to meet with different clients to find money because when I go to one place daily, the clients get used to you because after all he had have sex with you. So I go to different clubs.

**I: Uh huh.**

PI08: Yeah.

**I: Mmh.**

PI07: (inaudible content) (people laughing)

PI10: Sometimes when the children have closed school, I take all of them home and my house becomes a place of work. [Homebased sex work]

**I: Okay**

PI10: So it is just making a phone call for them to come that is how I increase my income (someone laughing)

**I: Okay, you have said it well. So a phone call and he come, a phone call and he comes, when you have sex with these people do you use a condom or you do not use?**

PI10: It depends, there are those I have worked with for long so I don’t have to use, flesh with flesh, I have unprotected sex with them because they are people who must be deceived and that can also be a source of finance.

**I: Mmh.**

PI10: Because I can deceive you that I am pregnant and you take care of something that does not exist (people laughing) so not all of them I have sex with using a condom.

**I: Not all of them you have sex with using a condom (someone clears her throat), so you have mentioned that he takes care of something that does not exist**

PI10: I made a phone call and had unprotected sex with you, I have made a phone call that I am pregnant and someone who is pregnant must be taken care of

**I: Okay**

PI10: So I get money from that, I am vomiting terribly, I am sick

**I: Uh huh, is there anyone who would like to add?**

P: No pass.

**So I am asking, if those who have sex for payment do not get male partners who pay them, what will they do? (People laughing) if you don’t get a client, what will you do?**

PI06: Those are the challenges that we face at work, so what we normally do, we have a union. If you are in a club like this, you must have a union such that when one of use experiences a bad thing like that, she comes to the group (union) where we were and tells us whoever has done that, then we go and that person will have to pay.

**I: Mmh.**

PI06: Yes

**I: No, in this case I am saying that you have not gotten a client (crosstalk)**

PI06: If I lack a client, I have gone and I lack a client because lacking is existing, we do lack many times. You go but you lack, in that case our lies will work, that is the moment lies will help because we don’t have savings. So that is the moment lies will help because you want to sit in the house and the children are also looking at you, there is no food or you are sick or a child is sick or a child was sent for school fees, so that is the moment lies will come in because we don’t have, I went to work but I did not get

**I: Mmh. (crosstalk)**

PI07: It’s like that question is repeated

**I: We had not talked about it**

PI10: Maybe I have gone for sex work and lined up, my fellow has gone and I have remained

**I: Mmh.**

PI10: I have another job, I get in the club where people are and dance to get something (money) (noise from the background)

**I: Another person? Uh huh, what do you do if you don’t get a client?**

PI07: There is a time you enjoy yourself, if you get don’t get a client you can be happy (someone clears her throat), you can laugh, you dance, if you don’t get a client, you get into a club and dance, then you have to know each other so that is an opportunity to know one another

**I: Mmh.**

PI07: You go to your fellow and ask for 200 /= since she knows you didn’t get a client.

**I: Mmh.**

PI07: Mmh.

**I: So, at what point will someone know that clients are not there? When it reaches what time?**

PI01: As from 12 am

**I: 12 am**

PI06: Mmh.

**I: Uh huh (crosstalk)**

PI05: 3

**I: 3 am or pm?**

PI05: 3:00am

**I: Mmh, uh huh, another person?**

PI03: 12am

**I: 12, so how much debt can a woman who have sex for payment may have at any time? The debt that someone can claim from you, how much is it? Number one**

PI07: Even ten shilling someone can just claim, there are people who if you buy something and lack ten shillings, that ten shillings they claim it from you

**I: Mmh.**

PI07: Mmh.

**I: No, debts like we said that we can lend**

All: Yes

**I: How much can be claimed from you at any given time? (Crosstalk)**

R: Eight thousand shillings

**I: Eight thousand shillings**

PI07: They can claim ten thousand because plus rent and the rest.

**I: Uh huh.**

PI07: Mmh, number five

PI05: Ten thousand shillings

**I: Ten thousand shillings, number four**

PI04: Six thousand shillings

**I: Six thousand shillings, number three**

PI03: Five thousand shillings

**I: Five thousand shillings, number two**

PI02: Eight thousand shillings

**I: Number said that she does not lend money, (people laughing) number ten**

PI10: Fifteen

**I: Fifteen**

PI09: Five hundred shillings

**I: Six**

PI06: I have not responded

**I: Yes, not yet**

PI06: Five hundred shillings

**I: Yours is very little, we are almost finishing but at this point it is sweet, it is not bad, these questions are interesting. Someone said that we discuss, right? People who have sex for payment think of when they can stop having sex for payment, do you always think about that?**

PI06: I have that idea, like myself I have an idea of leaving, if I sit down thinking, I feel that it would be nice if I leave that job because my children have grown, I should leave it

**I: Mmh.**

PI06: If I sit down again and think if I look at myself I feel that if I leave it, there is no way I am going to get income but I am going to miss the sweetness and I am not of age to leave. (noise from the background)

**I: Let’s talk one by one, the recorder captures everything, if you are talking from the background, it** **captures, so you give your fellow a chance to talk first then you can…**

PI06: So, for me

**I: Wait, Number six can you say you point**

PI06: I was saying that I have been thinking of how I can leave that business because it would be nice if I leave it because my children are grown ups

**I: Mmh.**

PI06: But again if I look at it keenly, there is no way I can leave it because there is nothing else I am holding on my hands (meaning she does not have another source of income apart from sex work which can help me, so I feel it (sex work) is my source of help

**I: Mmh.**

PI06: That is what I am still stuck there, I cannot leave it easily

**I: Another person? (Silence) have you ever thought of leaving this job?**

PI08: We have thought of leaving it someday, it’s only that the game is sweet because we are in a world where there is no where I am going to purchase it from, it is just your body which you keep clean and it is the reason why you eat, so you know if you go and buy items for the business, the items do not sell, maybe you buy sardines and you don’t sell, you go and hawk in the sun while the other one if I am from there (sex work) you are clean. When I am from there (sex work) I bathe and when I go to the house, I relax and eat then I prepare again and leave, so I feel I do not make loses in the business because I have not bought any stock.

**I: Mmh.**

PI08: Yes, I clean my body then I leave

**I: Uh huh, another person? Have you thought of leaving this work?**

P: I have not thought about that [crosstalk]

**I: Number seven**

PI07: I have thought of leaving it

**I: Uh huh.**

PI07: It’s only that we don’t have enough things that we need.

**I: Mmh.**

PI07: Mmh.

**I: Uh huh, is there anyone who would like to add? When you think of leaving, is it something you share with your peers or you just think about it by yourself?**

PI07: Sometimes people do things like…when you get nothing you don’t get a client you have to think a lot. Maybe you went and got nothing (you didn’t get a client) (someone clears her throat) and you left nothing behind. So you say that you are tired of that thing (sex), maybe you have woken up when your body is aching and it is you have to work

**I: Mmh.**

PI07: So you say that in case I had something to do, I would have left this thing

**I: Mmh.**

PI07: Because the body that goes there is sick, that one must be there

PI02: Number two

**I: Uh huh.**

PI02: Sometimes you just wake up tired of such things, you don’t feel like

**I: Mmh.**

PI02: Because with those things (sex) you must have feelings, sometimes you don’t have feelings, you are out of moods (you are not in the mood)

**I: Mmh.**

PI02: Mmh.

**I: Uh huh.**

PI06: It’s not that the way we are we are the only friends, we must have friends from outside (someone clears her throat) like me I have friends outside, you can go and chat with you fellow because she knows the job that you are doing, she can advise you to leave that job considering your age and join a business of selling vegetable or tomatoes. Maybe that is the job she is doing and if you think about it when you go to your house, you feel that advice that help you and if you go and have a look at structure where she does her business, you will realize that she sells vegetable worth two hundred shillings for two days. You realize that cannot help you because you are used to going (for sex work), sometimes you go and come back with one thousand shillings or maybe you go and get five hundred shillings (someone clears her throat), you know you are better that her. So you decide to ignore her advice and continue with my work (sex work) if it can help me, so that is why you cannot leave that work easily.

**I: Mmh.**

PI06: Mmh.

**I: Uh huh, what leads you to such stories, when you have started thinking of leaving that work? What brings that in your mind?**

PI10: This thing, I think about it when sometimes this man has had sex with me thoroughly (someone chuckles) he has had sex with me in a way that I have never experienced.

**I: Mmh.**

PI10: There are drugs they use (so that) his money does not go at a waste, he is also in the mood but he feels his money will go at a waste

**I: Mmh.**

PI10: So when he takes that drug, he takes it but he hurts you but for him he is happy that he has worked nicely, so at that time even if he gives you that money, you do not enjoy. He injured you, so you think of what took you there, which is the moment you think of leaving

**I: Mmh.**

PI10: You can also think of leaving when you stay for one week, yet the people you are used to, none of them is calling you or if you try calling someone, he does not pick or some pick but gives you what you didn’t expect, you expect him to respond to you nicely by giving you money (someone clears her throat)

**I: Mmh.**

PI10: But he responds differently so at that time you lose hope and would like to leave

**I: Mmh, you have mentioned that they use drugs, which kind of drugs? (Silence)**

PI01: I can emphasize on what she has said and secondly we are not the only people who do that work, you will find that there are people who do that work in town centers and you go then you find out that something happened to one of you in the line of duty which discourages you, if this person can experience this where is my hope? In case I can get something to hold on my hands (meaning another source of income), I can leave this work

**I: Mmh.**

PI01: Maybe your fellow went (someone clears her throat) and she expected to go and serve one person yet those people ganged up, they are three men or four men and all these people wants to have sex with her twice and she is one person. These people who want to have sex with her have also used drugs which boost their libido

**I: Mmh.**

PI01: While you came from the house when you had eaten ugali and sardines or meat and that is all you have in your body

**I: Mmh.**

PI01: It is obvious that you will be discouraged and feel bad that such a thing has happened to that person, so this work, I should leave it

**I: Mmh.**

PI01: But being that there is nothing you can get hold of on your hands and say that I can go and do business for selling vegetable, but if you look at that person who sells vegetable we get discouraged (because) maybe she bought vegetable worth two hundred shillings but she won’t sell from morning till evening. So you say that if that business of selling vegetable is what I want to do yet this person does not sell from morning, vegetable worth two hundred shillings, then this work of ours it is better I continue with this one (sex work)

**I: Mmh.**

PI07: You can have sex with someone, upon reach your work station you can hear that someone had sex with someone and killed that person

**I: Mmh.**

PI07: That one always happens especially Kisumu there is a scenario that happen, a certain lady had sex with a man who killed her and put her body inside a bag, she was cut into pieces and put in a bag, such things also discourages people.

**I: Uh huh.**

PI09: Stigmatization by friends or family, like my colleague had mentioned that neighbors mostly say that you are a sex worker, so that can make you stop that job of sex work

**I: Mmh.**

PI02: Sometimes you can have sex with someone, when he comes he dressed nicely and he had buried his wife and he wants to have sex with you then you get to hear that this person’s health is deteriorating yet she did something which is a taboo. You know some people do go and look for ladies, they look for us (someone clears her throat) so you think this person is clean yet he is not clean

**I: Mmh.**

PI02: That is the biggest challenge…. because someone will not know your husband died unless you talk about it, you cannot talk about it

**I: Mmh.**

PI02: So when he comes he cannot tell you that his wife died, he has sex with you and leaves (vehicle hooting)

PI07: You can go with someone and when he gets to the room with you, you carried a condom because you told him that a condom must be used, when he gets to the room he does not want to hear something like that and as a result you get a disease,

**I: Mmh.**

PI07: So such things discourage people

**I: Okay, so when you plan to stop this work, does it happen how you have planned?**

PI07: If I want to stop something I don’t plan, I just stop abruptly

**I: Mmh.**

PI07: I develop laziness indoors and then I will start a business

**I: Mmh.**

PI07: I will go to table banking and lend (money) then I start a business

**I: Mmh.**

PI07: You cannot plan but you get discouraged gradually

**I: Uh huh, is there anyone who would like to add? There is none?**

P: Yes

**I: Now I am asking, women who have sex for payment always stop this work at what age?**

PI06: Mostly, those ones who I see always leave when they are forty-five years

**I: Forty-five, uh huh.**

PI08: They always leave they have reach fifty years

**I: Fifty, uh huh. Another person, apart from the ages that have been mentioned**

R: Sixty

**I: Sixty, uh huh**

P: Forty-two

**I: Forty-two, uh huh, another one? So is it only age that causes them to stop or there is another reason?**

PI08: The reason why she stops is that for you to reach fifty years it means you child is above twenty years, so this child is a grown up and you can go for sex work (vehicle hooting) and you meet someone whom your child had met, with [ engage in sex] so you feel that at your age you should leave that job (noise from the background) (someone clearing her throat), so you find that maybe your child has gotten something to do and she can support you

**I: Mmh.**

PI08: Because we don’t have savings and that is why we used a term that you have to reach fifty years, so for me to reach fifty years my child can support me, so I leave it.

**I: Uh huh.**

PI08: Yes.

**I: Apart from age is there….**

PI09: Maybe your body is tired, at that age your body is not strong as it was the you were at thirties or twenties, so you know if you reach fifty years your body is tired

**I: Mmh.**

PI08: Mmh.

**I: Uh huh.**

PI10: Apart from getting tired, I am emphasizing on this, there is a time that there is someone interested, you find that someone’s wife died and he wants someone whom he stays with and you have been together for long

**I: Mmh.**

PI01: Someone that you are used to and he is your customer and you know that this person supports me in such a way, so he has supported you for long and you are used to one another, he can decide to marry you

**I: Mmh.**

PI10: That can also make you to leave

**I: Uh huh, what do women who do a job of having sex for payment normally do after leaving this job?** **What do you do after leaving the job of having sex for payment? Jobs like which ones**

PI08: You can do a business which you can operate from where you are, where you are seated not hawking which can earn your money

**I: Uh huh, another person, what else can you do?**

PI09: Let’s say you are at old age and your children are grown ups, you can just be like a wife material at home so the children feed you, you are just resting

**I: Mmh.**

PI09: Mmh.

**I: Uh huh, another one?**

PI03: You start a business

**I: You start a business, uh huh, another person? What can you do after leaving this job? What can you** **do? You have left sex work and you want to do something, what is that you want to do?**

PI01: In most cases business is what we can involve ourselves in and do or you can say…. or all of us who are here you can find that there are those who had a misunderstanding with their husbands, so it is the misunderstanding which led them to this kind of work.

**I: Mmh.**

PI01: Or you can find that you have struggled with the children and the children also have been seeing how their mother has been struggling, so you can find that the children telling you to go back home (maternal home)

**I: Mmh.**

PI01: You stay at home and embark on farming or you can keep chicken (do poultry farming) at home

**I: Mmh, is there a different response? You can keep chicken (do poultry farming), you can start a business, you can do farming, you can just sit as the children feed you, and do you have any other?**

All: There is none

**I: So, if you want to start a business, you want to do farming or whatever you want to do, where are you going to do it?**

P: Me if I want to do farming, I go back home or if I don’t go back home, I can go to my maternal home or I can look for the father of my children in case he is available, I reconcile with him then I settle there and I start farming there

**I: Uh huh, for those who wanted to start a business (noise from the background), someone said that she can only start a business where she is living, what about number one, where can you start your** **business?**

PI01: (someone clears her throat) it’s at home that is where I can do farming

**I: Okay, it is okay. Is there a change you are seeing in your life on how you are living after leaving this job of having sex for payment?**

PI07: (Noise from the background) there could be a change because you feel cold outside but now you are going to relax in the house

**I: Uh huh, another person? (Someone clears her throat) A change in your life**

PI06: The change you are going to face in your life, you will find the change which you are going to face is that your veins are going to be a bit strong because when you were doing sex work, those who were having sex with you…your veins are weak because they those people have different strength, so you know if you go and sit down at home while you are being fed your veins will be strong.

**I: Mmh.**

PI06: Mmh.

**I: Is there anyone who would like to add? There is none. So do you know a time that a woman who have sex for payment has left this job and went back to it later? Is there anyone you know who knows a woman who left this job of having sex for payment then went back to it?**

PI10: There is a friend of mine who was doing that work, you know in most cases you find that long distance travelers, those who drive trailer, married her and after that I think she saw the challenge of being in the house then she got back to sex work.

**I: Mmh.**

PI10: So there is a way some problems that someone can face in marriage

**I: Mmh.**

PI10: Mmh.

**I: Uh huh, another person? (Silence) You left and got back to it, why?**

PI08: Maybe she got someone who has money, so money is being sent to you, so she decides not to go for sex work since I have someone who sends me money and this person goes to work and then…like trailer drivers who we get, maybe he goes to India or another place and disappears there and maybe there is no connection (communication), so you say that you had gotten used to feeding well now I will be forced to go back

**I: Mmh.**

PI08: Mmh.

**I: Is there anything that can make them to go back (someone clears her throat)**

PI08: To go back to work

**I: Yes**

PI08: They have to go back to work because the money which was being sent to them is no longer there

**I: You have mentioned that, is there any other?**

PI08: No

PI10: Right now we have our own freedom, you find that there are some restrictions which can make someone to go back because now she is being guided yet she was not being guided

**I: Okay**

PI01PI10: Mmh.

**I: So what are the negative thing that can happen if someone comes back, she had left and she comes** **back again? What challenges you would experience?**

PI09: She loses her customers

**I: Uh huh.**

PI09: Mmh.

**I: Another one? She left and came back, what challenges would she experience?**

PI10: You find that her veins are not used to work, she was used to one person and now we are moving to a group

**I: Mmh.**

PI10: Mmh.

**I: Another one?**

PI04: Stigma (someone clears her throat)

**I: How?**

PI04: Those who you were working with will start despise you, they will talk ill about you

**I: Mmh.**

PI04: Mmh.

**I: Uh huh, another person? What good thing could be there if I come back (noise from the background)?**

PI02: Those who we were in the same group with would be happy to see me

**I: Mmh.**

PI02: This person is back, maybe there is a way I was helping them or even in terms of money

**I: Mmh.**

PI02: Mmh.

**I: Is there any other good thing apart from your fellows being happy that you are back? (Someone** **whispering) should I continue?**

All: Yes

**I: Okay, I am asking, what is it that women who have sex for payment would like to do before they get that job? Things that you would like to accomplish before you leave that job of having sex, which ones are they?**

PI06: The things that I could like to accomplish, I would like to…. because I stay in a rental house, I would like to get a piece of land and build my house so that one day if I leave this job, I go straight to my house. So that is what I would like to do before I leave this job

**I: Uh huh.**

PI06: Mmh.

**I: Another one?**

PI01: I would like to educate my children so that they reach where I never reached, so that they can remove me from that job and put me somewhere else

**I: Uh huh, another person? What do you want to accomplish before you leave this job?**

PI10: I would like to do some investments so that even if I stop working, I sit down knowing what I am looking at

**I: Mmh.**

PI10: Like if I build rental houses or I have a business somewhere, so I just feed well if I stop working

**I: Uh huh, is there any other different thing? (Silence) now, those things you have mentioned that you want to accomplish before you leave this job, have started planning?**

PI06: I have a plan (noise from the background) but depending on the income, right now the income is small though I have plans

**I: Mmh.**

PI10: Mmh.

**I: The plans you have, what do you do to get that piece of land?**

PI10: I save little by little

**I: You save little by little, uh huh.**

PI08: What is the question?

**I: I have asked, what you would like to achieve before you leave this job. Some people have said they want a plot (rental houses), someone wants to invest in a business, so I am asking whether they have planned for doing that.**

PI08: You have plans, like we call it planning or what

**I: Yes, plan**

PI08: I plan but I still don’t have much for me to way that I want to start that business because I still don’t have money to start that business but once I get money, like I had planned that later I would leave that job but I have not gathered any money

**I: Mmh.**

PI08: Yes

**I: Okay, do you know anyone who was doing this job who stopped for five years to ten years?**

PI06: I know of a woman from our village who was doing that job and stopped, she has educated her children through that job, she bought a plot through that job and she built her house through that job. Right now she left that job and she leading a good life, she is feeds well and she is not doing any job, the things (business) she started outside generates income for her and her daughter also supports her.

**I: Mmh.**

PI06: So she is leading a good life, I have seen someone who has left

**I: Uh huh, number seven (noise from the background)**

PI06: I used to see a certain lady who used to stay in =Bandani=, she was a relative of one of my friends (noise from the background) (inaudible content)

**I: Uh huh, any other person who knows someone who had left for five or ten years? (Silence) you have said that one person got married and another one educated her children and nowadays her children support her, right?**

All: Yes

**I: Is there any problem they faced when they left? (Silence) Mmh.**

PI06: The changed they faced when they left, (noise from the background), the problem they faced when they left that job, in terms of feed and how she lives, there was no problem because she lived well and she could get good money. It is only that the problem she had was sex

**I: Mmh.**

PI06: She mentioned that she had that problem because she was used to meeting many people, so right now she wants to be alone, maybe she takes one week before getting someone (male partner), and she feels tired

**I: Mmh.**

PI06: Fatigue of the body

**I: Mmh.**

PI06: Mmh.

**I: Did yours experience any problem?**

PI08: Mine did not experience any problem, she was leading a good life

**I: It is okay, number six, the problem she was experiencing of missing sex, how did she overcome this?**

PI06: How she overcame, I would not know more about it

**I: Mmh.**

PI06: Yes

**I: It’s okay, so remaining part we are finishing, the questions talk about Jitegemee intervention, what we want to do in Jitegemee, so put your mind here so that we ask those questions then we finish. Initially I explained to you about Jitegemee study and I explained that it is being done so that women who have sex for payment have money that they save which can make them to refuse having sex without a condom or take a break for having sex for payment when they want to take a break. I also said that it is about women who have sex for payment saving part of their money by themselves so that they use when there are no male partners who make payments or to help them prepare for life after leaving the job of having sex for payment. So in Jitegemee we say that your money, even if you save it you earn no interest and you can withdraw it at any time, and the day you feel that you there is nothing you are saving, someone will not force you that you have to save something. The amount you save is what you can withdraw, you can withdraw part of it or you can withdraw the whole amount. Question, is Jitegemee something that some women who have sex for payment would like here in Kenya? Uh huh.**

PI10: The reason why it would be liked is because it is something that (participant clears her throat) you keep your money such that if you experience any problem, in case you get something urgently or something that has attracted you at that time and you don’t have cash money, you can go and withdraw and attend to that need.

**I: Mmh. Uh huh, another person, will other women like it? Will they like Jitegemee? Those who exchange sex for payment. (Silence) aah, we have what Jitegemee is all about, right?**

PI10: We can like it being that it is something which looks like encouragement because it removes us from sex work

**I: Mmh.**

PI10: The work we do is not a very difficult task, some situations are difficult and in some situations it is not very difficult

**I: Mmh.**

PI10: So Jitegemee wants us to start using our strength so that we can find a way that we can leave that life. So we can like it but only if we can join others who are not with us to teach us how we can (inaudible content)

**I: So, what kind of women who exchange sex for payment would like Jitegemee? (Silence) have you heard the question?**

P: Can you repeat the question

**I: What kind of women who exchange sex for payment would like Jitegemee? (Someone whispering) you have said that it is hard**

**P: Kind**

**I: Yes, kind, we have different people, right?**

All: Yes

**I: So which people would like it? Women who have sex, which people would like it?**

PI02: Women who like saving frequently such that if they get money they save

**I: Uh huh.**

PI02: Yes, they would like Jitegemee

**I: Uh huh, another person? (Silence) uh huh, is there any other response? There is none, and are there those who would not like? Are there those who would not like Jitegemee program?**

PI09: There are those who would not like because the way we are there are those who do not like hard work which can result to saving apart from where we go and lazy around

**I: Mmh.**

PI09: Yes, because those people seem they work hard for us and when they come…there are those who if you have sex with them, you enjoy

**I: Mmh.**

PI09: They had worked hard and when they come we enjoy their sweat because we are in the game, there are those who cannot accept to go and work hard to get their own money

**I: Mmh.**

PI09: Mmh.

**I: So, let’s say you have ten friends, right? And you tell them about Jitegemee, out of your friend how many do you think will accept Jitegemee? Everyone is responding to that question.**

PI02: They can be four people

**I: Four people**

PI02: Mmh.

**I: Uh huh.**

PI04: Six people

**I: Uh huh.**

PI05: Five people

**I: Uh huh.**

PI08: Two people

**I: Uh huh.**

PI03: Four people

**I: Uh huh.**

PI01PI10: Three people only with a strong convincing power

**I: uh huh.**

PI09: Five people.

**I: Uh huh.**

PI06: Two people

**I: Uh huh.**

PI01: Three people

**I: Uh huh.**

PI07: Four people

**I: Uh huh, who is remaining? (Silence) no one is remaining. So for those who mentioned that they can only tell four people, the remaining six why would they refuse? Why would they not agree with this thing?**

PI06: Because people’s understanding is different (someone coughing), those four people, their understanding could be easy and they can easily accept but the remaining six, their understanding could be difficult

**I: Mmh.**

PI06: Understanding is difficult

**I: Uh huh, someone said six people and four cannot accept, why?**

PI09: They can feel that it is an organization which does not want them to continue their work (someone coughing) and it is a job which they like

**I: Mmh.**

PI01: The reason why I would say that some people have different understanding, I want to emphasize what number six mentioned

**I: Mmh.**

PI01: There are some people who don’t like exposing themselves such that the job they are doing would be known, so when someone is out, you see her carrying her purse and she is going to work

**I: Mmh.**

PI01: For her to share with her fellows the kind of job she does, so it seems you want to expose her so that it would be known that does such kind of a job (someone coughing)

**I: Uh huh, the rest what about the numbers you mentioned, why? Are the reasons that have been mentioned the same or they are different?**

All: They are the same

**I: Same reasons**

PI07: Most people don’t like because of the reasons that have been mentioned

**I: Mmh.**

PI07: Mmh.

**I: What can we possibly do to increase the number of women who have sex for payment to accept Jitegemee? What should we do so that women who have sex to accept Jitegemee?**

PI02: We can make it to look like a club and teach people (birds chirping)

**I: Mmh.**

PI02: Yes.

**I: Uh huh, another person?**

PI10: By being an example how your situation in life, if you are a person who has joined Jitegemee, you tell them what Jitegemee is all about and you give yourself as an example, where you came from and understood what Jitegemee is and joined Jitegemee

**I: Mmh.**

PI10: That is when they can (noise from the background) (inaudible content)

**I: Mmh, meaning we must give them success stories**

PI10: Yes

**I: Uh huh, which other one? What can we do to increase the number of people who will like Jitegemee?**

PI08: Just like number two mentioned that we can have a club, so after forming the club we can do wonders, we do things in our club such that when others see, they can join us

**I: Mmh.**

PI08: Yes

**I: It is okay, what should Jitegemee have for people to accept it? What should we have for you to accept it?**

PI02: First, you should have someone who has volunteered to share where Jitegemee has taken her from, I was doing this job and this how it has helped me

**I: Uh huh, another one?**

PI02: Even if you would have some money to lend her to start a good business which generates income

**I: Mmh, uh huh. Another one? Another person? (Silence) are those two enough?**

P: Jitegemee as an organization should take one two three from these groups, like now you came to south west ward, we are at =Kisian=, you pick two people and take them to the organization and employ them, then they come back and teach these people

**I: Mmh.**

P: So we know how Jitegemee is good and through that the organization grows, so it picks people bit by bit and put them there. If those people are successful, they can have their own, so you know there are people who if they look at that job and feel it is tough

**I: Mmh.**

P: So how you would cope with this, so you know if someone could support you even if it is not working for the organization, Jitegemee should pick one or two and post somewhere to do away with this issue of sex work

**I: Uh huh, if you say one two three, is it one two three stones?**

R: It helps people, it can pick one person and take there, another one this way, so the more those people benefit from Jitegemee, where there go out and share how this thing (Jitegemee) has helped them which will encourage people and say that if it is this way then I should work hard so that I can get a job

**I: Mmh.**

P: Because there are people who have knowledge and they can join (work) in different places

**I: Mmh.**

P: Mmh.

**I: What will you not like or see in Jitegemee? What would you not like about Jitegemee? (Silence) what will not like about Jitegemee? We are left with three questions then we will be done, so speak up. What would you not like, if there is none you just say there is none and if there is any you can say?**

PI07: About Jitegemee, the name Jitegemee is …

**I: Mmh.**

PI07: Mmh.

**I: Why?**

PI07: She would feel that name is bad, it’s a sexual (sex) worker’s name

**I: No, have we written any sex worker name here?**

P: No

**I: Mmh, we have not written sex worker name here, we just call it Jitegemee meaning you do you things on your own so that you can support yourself and have your own money, right?**

All: Yes

**I: Yes**

PI09: Someone would not like to share her story here in Jitegemee then she is exposed

**I: Uh huh, another one? What would they not like? What would they not like? Is that all? (Silence) okay, we are moving on, what would you like about Jitegemee as we read? What did you like?**

PI08: (Noise from the background) what we like is that we can save and later leave the work we are doing

**I: Mmh.**

PI08: Yeah.

**I: Another one? What did we say Jitegemee is trying to do?**

PI10: Jitegemee is trying to help us to do away with the issue of getting HIV and be people who are faithful

**I: Mmh.**

PI10: Mmh.

**I: Uh huh, another one? (Someone coughing) That is all? Are there issues with Jitegemee that women who exchange sex for payment would feel that it violates their rights in any way? Yes, respond. If you join Jitegemee program, will it violate any of your rights?**

PI07: It will not violate, it is going to educate you so that you can move from your current life and move the life of high standard

**I: Uh huh.**

PI07: Mmh.

**I: Another one? Anyone with a different opinion? Will it violate your rights in any way? (Silence) can you respond so that we can move on.**

P: It will not violate

**I: It will not violate, it is okay. What challenges are we likely to face as Jitegemee if we want to start this program?**

PI01: How you will (inaudible content) if you come on the ground like you have done now and mobilize people like us, those who will disclose about their life, story and share like we are doing here now will be a challenge because someone maybe how you will explain to him about Jitegemee, all these things that are written here, her understanding is different from how we understand

**I: Mmh.**

PI01: Maybe someone would feel that you have your rules, maybe you have come to expose her life, her life history to the public, those are the challenge which I think you will face

**I: Mmh, another one? Which other challenge will we face?**

PI10: We can face a challenge of capital

**I: We people from Jitegemee**

PI10: We people from Jitegemee, we would face a challenge of acceptance

**I: Mmh.**

PI10: How someone will accept you to be one of them, because let’s say that you come from here to go and join a group of selling items in the market, someone would not accept you to be among them because you were not there initially

**I: Mmh.**

PI10: So they would feel that you have come to laugh at them but in real sense you want to change

**I: Mmh, another one? Which challenge would we face as people from Jitegemee? (Silence) there is none, we are about to finish, tell me the challenge we would face. We want to come on the ground (field) which challenge would we face? Uh huh, number four**

PI04: Insufficient capital, you know everything needs money in order to conduct them

**I: Mmh.**

PI04: Mmh.

**I: Insufficient capital, are we the ones with insufficient capital or you?**

PI04: You

**I: Uh huh, the challenges you have mentioned, how can we overcome them? Number one mentioned that some people would feel that we want to expose their life history, so this challenge, how can we overcome it?**

PI07: We lend them money then they pay back

**I: You said that we are going to expose their life history, so how will we overcome this challenge so that they don’t feel that we want to expose them?**

PI02: You can call four people and share with them your life history on how Jitegemee has helped you

**I: Mmh.**

PI02: You can tell them that I used to be this way, I used to do this job this way, I did this to get where I am, and you can share with her

**I: Mmh.**

PI02: Now you share with her your own story

**I: Mmh.**

PI07: (inaudible content)

**I: Mmh.**

PI01: (Someone coughing) change starts with me, I am the one to change so that I can leave the job I am doing to be where I want

**I: Mmh.**

PI01: If I can accept and change, for example Jitegemee has cause me to change, I will give them an example with myself. If I can find one person whom I can share with where Jitegemee has taken me from to where I am currently, that person will share with another person and the other person will also like that, so you will find that at least you have something on the ground

**I: Mmh.**

PI10: You create awareness about Jitegemee and close mobilization, now you are close to them, you tell them one two three

**I: Uh huh, now we want to start Jitegemee, right?**

All: Mmh.

**I: How much would you save on a weekly basis? (Silence)**

PI04: Five hundred shillings

**I: Five hundred shillings**

PI10: Seven hundred shillings

**I: Seven hundred shillings, uh huh.**

PI01: I support that of number ten

**I: You support five hundred shillings**

PI01: Seven hundred shillings

**I: Seven hundred shillings, uh huh, another person?**

PI08: Six hundred shillings

**I: It is okay, so where do you think you should save your money, this money you are talking about where you feel there will be no disadvantages?**

PI06: Bank

**I: Bank**

PI04: Mshwari

**I: Mshwari, number four, another person?**

PI01: In a club with a reason

**I: Mmh.**

PI01: When I save it there, it would not be easy to withdraw until the end of the year, so if I save seven hundred shillings weekly, if I multiply by fifty-two at least I find that at the end of the year what I have can help me somewhere.

**I: Uh huh, another person feels that where can she keep her money?**

P: Merry go round

PI10: I save at KCB bank with a reason via Mpesa, I mean phone, I can take a loan which runs for one month, so when I have taken a loan and save money which can allow me to take a good loan, I use this loan and the money which comes from the loan I find a way to use it to repay the loan within one month.

**I: Mmh, is there any other? (Silence) this is the last question, now I am asking that, you have said that you can save seven hundred shillings, right? Someone has said five hundred shillings, if at all you don’t reach the target that you were supposed to save, what will you do to get that money so that you can save?**

PI07: If in a week I save five hundred and I save four or three, the next week I will top up

**I: Mmh.**

PI07: Mmh.

**I: Uh huh, another person, what will you do to save? (Silence) she will top up, what will you do to save** **the money you said you will save?**

PI08: (Noise from the background) I said I will save six hundred shillings weekly and maybe I save three hundred shillings because maybe whatever I was doing I have not achieved for me to save that much (amount)

**I: Mmh.**

PI08: So when, since it is said that in business each day is different, maybe today it is good and tomorrow it is bad, so when it is good I will top up that money so that I can achieve my target

**I: Uh huh, is there any other idea?**

P: There is none

**I: There is none, do you have a question before we finish? Or any other thing that you want to say?**

PI06: I just appreciate

**I: Uh huh. (People laughing)**

PI10: Number ten

**I: Number six is still talking**

PI06: I would like to thank this organization for the opportunity you have taken to support people like us, for the opportunity you have taken to leave your families maybe there are those who have left little children and you are walking in the field to support us. So I would like to request you to continue having that heart and continue mobilizing other groups like ours and continue supporting them and stabilize that group and show us the right direction which can use to benefit our lives.

**I: Uh huh.**

PI06: That is all

**I: Another person?**

PI10: You asked if anyone has a question

**I: Yes, a question concerting what we have talked about before we finish.**

PI10: The way you are here with us, I am asking that (noise from the background), we you the way we are or? (People laughing)

I**: I would not lie, I am not the way you are, right? But I work with Impact Research and I just came to know about your ideas**

P: That is true

PI10: Secondly, I have decided, and I would like…. I took a social work course and I would like to join and also mobilize people the way you are doing and talk to them so that they can change because as I view it, I am a social worker and I have changed and I want to find something to do

**I: Mmh.**

PI10: Before I find something to do, (inaudible content) pick me so that I can be one of you so that I can change some people

**I: Uh huh, I have heard that. Uh huh, another person who has something before we finish? We want to finish**

PI01: I have a question, the way you are walking, indeed walking is difficult and mobilizing a congregation of women like us because many people discriminate us. In most cases you hear people say these are people who do the work of Sodom and Gomorrah

**I: Mmh.**

PI01: And you have the courage of sitting with us like this, you view us as people, we are also people’s children, it is the world that has molded us this way. So what plans do you have as an organization? Which plans do you have? Is there a way you can support people like us? Like me, let’s say for example there is a business I can do and I don’t have capital to do this business and I want to leave this job and change and do that my business, so that at least by the end of the day there is something I save which I can count on. Do you have a plan like that which you can support someone like me who wants to do a business but in lack money, is there a way you can give people money, like people like us, maybe you can tell them that we can give this amount of money to do this and by the end of the day you will figure out how you repay that money? Do you have such a plan?

**I: So number one, what brought us here is to get your views, we can get your views about this issue of Jitegemee, right?**

P: Mmh.

**I: You have heard what Jitegemee wants to do, as in you who do the job of having sex, the small amount of money you get, whether it is little or more, you spend ten and save how much, ten right. Meaning that the day you would want to leave, you will have money to start a business that you want to do, right? So I cannot lie to you that we have money to give out, we don’t have money, we are just giving you information. We are just sensitizing you so that you can focus on the future. Is there a question or [Mentions name] do you want to add something? So I thank you for the information you have given us, right?**

All: Mmh.

**I: The information you have given us will help other women, right? Thank you all.**

P: Thank you

**I: Okay.**

**END OF THE INTERVIEW**
