# Supplementary material for: Eat a little and save a little: A qualitative exploration of acceptability of a potential savings intervention to reduce HIV risk among female sex workers in Western Kenya
Source: PLoS One. 2024 Dec 19;19(12):e0310540. doi: 10.1371/journal.pone.0310540 (PMC11658496; doi:10.1371/journal.pone.0310540)
Supplement: S1 File — (ZIP) [file pone.0310540.s001.zip › Jitegemee Transcripts and Dissemination Notes for Journal/FGD J.docx]

**FGD TYPE: FGD J**

**NAME OF TRANSCRIBER: WINNIE**

**NAME OF INTERVIEWER: JUDITH**

**NAME OF NOTE TAKER: PHILIP**

**INTERVIEW DURATION: 2:14:52**

**CATEGORY: ABOVE 30, PERI-URBAN**

**I: Okay. This is FGD J the interviewer is [Interviewer name omitted] and the note taker is [Note taker name omitted], the discussion is being conducted at =Otonglo, Kisumu County=. Depending on what I discussed with you about Jitegemee, what is it that comes first to your mind?**

PJ08: Number 8, Jitegemee, I think how we can survive later when we stop sex work. Again at least when someone stops that work, how she can do a business, I think that’s what Jitegemee is trying to teach us.

**I: Any other thoughts?**

PJ07: Number 7. I think Jitegemee, is something good because there is a time your body is not active, you are sick and cannot go for sex work while sick because you do not even have feelings for a man. So if you are independent it can help you, that’s my thought.

**I: Any other thought?** **Number 1,**

PJ03: Number 3, I was saying that… it is true sometimes someone can be sick and there is no means of helping herself when she is sick. so the kids too are facing problems, those that are young and going to school cannot help themselves and you are also sick and it is sex work that helps you. So I was just asking Jitegemee to see how they can help us with that.

**I: That’s the thought of number 3, any other thought about Jitegemee, number 6? (*Silence*)**

PJ06: The thoughts that they have brought forward, I as number 6, they are good I agree with them.

**I: How do you agree with them?**

PJ06: I agree with number 7’s thought, for sure you can be sick, you have body pains, you do not feel you want a man.

**I: Any other thoughts, number 2?**

PJ02: I think as number 2, I agree with number 8’s thought, that for you to be independent you can start some business after stopping sex work.

**I: Any other thought? Number 5.**

PJ05: I as number 1, I support number 8…

**I: Number 5**

PJ05: Number 5, as number 8 has spoken, you can get tired, your body can become tired, you can have some business to help you in your coming life.

**I: Okay, number 4 any thoughts about Jitegemee?**

PJ04: Ok, as number 4, I can only join hands in what number 8 has said.

**I: What has she said?**

PJ04: If we can continue with this sex work, you don’t know what will happen tomorrow, if it happens that you had saved some from depending on yourself, at least you will start your business which can continue helping you after we have stopped this work.

**I: Okay, number 9, thoughts about Jitegemee?**

PJ09: As number 9, I support Jitegemee activities, because there are areas that it has encouraged us that we did not know.

**I: Like which areas?**

PJ09: You can have a problem, in this work that we do, so firstly, it is like a business, because you cannot get used to one type of work like sex work. You can have some other business that you can do which can help you other than sex work sometimes, because the body gets tired and that is something you cannot depend on, so this program has helped us in thinking about some areas that we did not know.

**I: Okay number 1, your thoughts about Jitegemee?**

PJ01: Thank you for all those who have spoken, what I can say apart from what you have said is that I can support Jitegemee, because the way I am, I think I cannot do that sex work every day and depending on the people I have, I may feel sorry for my children if I do not do that sex work. So you should have some business even just some kales to chop so that the children can get something they can depend on.

**I: Okay, thank you for your thoughts, and what are some of the things that female sex workers can buy every day? Things that they love buying every day.**

PJ01: I as number 1, things that those who do sex work can buy daily, just depending on how we have children food is something you have to buy every day, the children have to eat, so even if you have torn undergarment, but your children have to eat and that becomes a good thing.

**I: So in one day how much money can you use for food?**

PJ01: (*laughing)* In one day, it depends on which kind of foods you buy for your children, there are kids who do not like vegetables, there are kids who do not want meat, so it depends with the kind of kids one has and the food they eat. So, at least you can use 200/= or even 300/= per day.

**I: So that is per day?**

PJ01: Yes

**I: And what can you buy per week, like a week does not go by without you buying what?**

PJ01: (*laughing*) A week cannot end without me milling maize

**I: ooh, without you going milling. How much do you use for milling?**

PJ01: For milling as of now we get a 2kg tin at one hundred and forty shillings (140/=)

**I: And how many times do you mill per week?**

PJ01: (*Laughing*) I do milling twice a week

**I: So that is two hundred and eighty per week on only flour?**

PJ01: yeah, two eighty on only flour.

**I: Anything else that a week cannot go by without you buying?**

PJ01: A week cannot go by without me purchasing charcoal, but when I cannot afford I go fetching for firewood, as we are in the rural area you can fetch firewood and roast them something lightly.

**I: And in a month? (both laughing) what amount of money can you use in a month or what kind of things do you buy in a month?**

PJ01: In a month I can buy… let me say for example, depending on the kids you have, you can make sure that you buy inner garments for one of the kids, and another you can buy a blouse, another one too you must even a pair of sandals so in a month it seems the amount can be higher but it cannot be more than three thousand in a month (3000/=)

**I: Three thousand (3000/=) at least?**

PJ01: Yeah

**I: Okay those are the ideas of number 1, anyone else, what do female sex workers buy on a daily basis?**

PJ07: As number 7, I have different opinions from number 1, because as a sex worker you must have your own trust (condoms) in your bag and that is something she (PJ01) did not think of, that is my thought as number 7

**I: Do you buy that every day?**

PJ07: Yes, always wherever you go out that is something you do not miss having in your bag, because you can meet someone then he tells you he does not have his own condoms, for the time being there are diseases (that can be sexually transmitted) and you cannot risk your life knowing there are diseases just because you want money. So if he tells you he does not have any, you have a right to get yours that you had in your bag. That is my opinion as number 7.

**I: So how much do you buy condoms daily?**

PJ07: Nowadays they have increased the price, it is fifty shillings.

**I: It is fifty?**

PJ07: Yeah,

**I: Everyday fifty?**

PJ07: Yes, everyday fifty you must have it, sure bet

**I: A week cannot end without buying what?**

PJ07: Without buying it (condoms), that is something I make sure. I’d rather not having something to take tea with but have it in my bag.

**I: That is every day on condoms, and a week cannot end without buying what apart from trust?**

PJ07: A week cannot end without me buying a dress because I must go to work when am looking good that is when I can be attractive.

**I: If you buy a dress, how much does it cost?**

PJ07: Maybe one that I see which pleases me, it can be from second hand, it can be ready made, so the one that will please me.

**I: Average?**

PJ07: Any

**I: How much is any?** (*other participants talking at the background*)

PJ07: About seven hundred shillings.

**I: Seven at least?**

PJ07: Yeah

**I: And in a month? What is it that you cannot end a month without buying?**

PJ07: Without buying a dress that is what I like most

**I: So clothes are weekly and monthly?**

PJ07: Yes, you must get there (sex work venue) when you are clean that is when you get someone who gives you good amount of money, when you are dirty it depends and will not give you a good amount of money

**I: Mmmh, those are the thoughts of number 7**

PJ08: Now it is number 8, in fact what they have said I take it in two points, the first one when you are going to the field for sex work, you must know how you leave the kids in the house that is first thing before going out. You know kids, you must check that by the time you are leaving what have you left behind for the kids, you must also leave what they will eat. Another thing is trust that she (PJ07) has talked about. You must have condoms, she has talked about condoms that cost fifty shillings as for me I use condoms that cost one hundred shillings (*other* *participants laughing*) you know these things depends, condoms are different there are those that cost up to two hundred shillings (*other participants agrees to her comment*). So I must buy condoms of at least one hundred shillings, at least standardized, at least I feel that at least a little… so you must have, so in a week I can use like eight condoms.

**I: So that is like eight hundred shillings?**

PJ08: No, that is one thousand six hundred shillings, that is in a week, and for food I must make sure these kids have taken breakfast, for this breakfast on a lower side it means they have not eaten bread maybe they have just taken tea without bread but you bought milk. You must buy sugar daily because as you know sugar prices has increased you cannot buy it daily, that you are buying the bigger packet at once, at times that money is not available you have gone for the sex work and came back with (*participants laughing*) some little amount of money and you also use fare from there back home. So, you know that it becomes expensive in a way.

**I: So in a day roughly how much money?**

PJ08: So in a day roughly if I calculate breakfast, lunch and supper because mostly kids eat at the school. So when they eat at the school, I give each of them fifty shillings for two kids that is one hundred shillings. In the morning, am using like one hundred and fifty shillings that is what they have eaten and drank, that is one fifty per day, lunch is one hundred shillings when I leave in the evening I leave them with one hundred and fifty shillings for supper, so that is roughly four hundred shillings. So in a week, if I have just calculated breakfast and left those my other important things, that is four hundred times seven which is two thousand eight hundred shillings that is food alone in a week my other things are still left pending, so for my other things I calculate two hundred shillings, I calculate two hundred on Saturdays. (*participants laughing)* Stop laughing, I calculate two hundred times… you know that is per day and I buy them like eight per day and sometimes there are those that come carrying their own condoms, so that is one thousand six hundred shillings plus twenty-eight that is roughly how much? Thirteen, fourteen, that is roughly forty-four, right? That is around forty-four hundred (to mean four thousand four hundred shillings), remember I have not included fares my friend (*other participants* *laughing*)

**I: And you go to town daily?**

PJ08: Yes, I go to town daily so you know I also include fares, so it is roughly five thousand shillings.

**I: That’s a week?**

PJ08: Yes, that’s a week.

**I: What about a month?**

PJ08: So if you add five times four that is around…

Chorus response: Twenty

PJ08: Yeah at least twenty, that is twenty thousand per month but at times it does not go as planned (*other participants laughing*)

**I: Okay, that is the thoughts of number 8,**

PJ08: And you know you have to include oil and some perfumes.

**I: How much can the perfumes cost?**

PJ08: So for the perfumes, (*participant laughing in the background*) we nowadays use those that are being refilled, at times you can refill it at 200/=. This one for two hundred can last you for up to a week. So when you add that perfume there… Please don’t laugh, are you laughing at my thoughts? I have also not yet included soap, so if I add it is roughly twenty-two in a month.

**I: At least twenty-two thousand?**

PJ08: Yeah, at least twenty-two thousand in a month

**I: Any other thoughts number 6, you are laughing a lot at others tell us your thoughts. In one day how much can you spend?**

PJ06: I as number 6, I back up number 7

**I: How do you back her up are you staying together?**

PJ06: (*Laughs)* we are neighbors and we go for sex work to the same place.

**I: Do you eat the same food?**

PJ06: We eat different foods

**I: How much can you spend in one day?**

PJ06: On food

**I: How much can you spend in a day?**

PJ06: In one day I can use three hundred shillings

**I: In buying what?**

PJ06: When going to hustle I must take care of my body well. She said she will buy trust condoms of which I support but I also have to buy inner wears because am not Osogo Winyo (referring to a musician) to remove both trousers and inner wear together (*Other participants laughing*).

**I: Anything else you can buy in a week?**

PJ06: In a week, I have to leave my kids something to eat, so I must buy and leave them with food while going to hustle.

**I: So how much do you spend in a week?**

PJ06: In a week if I spend that money its almost approaching two thousand shillings

**I: And in a month, what is it that the month cannot end without you buying?**

PJ06: Something that the month does not go by without buying?

**I: Apart from food and trust.**

PJ06: (*silence) Dress*

**I: How much do you spend when you buy a dress?**

PJ06: Seven hundred shillings

**I: Is that per month?**

PJ06: Yes

**I: What else do you buy in a month?**

PJ06: Inner wears

**I: Mmh, of how much in a month?**

PJ06: one thousand five hundred shillings, those ones from =Busia=.

**I: Okay, any other thoughts?**

PJ09: I as number 9 I support number 7, because you know if you have to go to this job of ours, you must go when you are clean. She said you must have condoms, because for sure you can meet someone, and this person does not have that condom so you have to carry yours, so you know you have to carry yours of which you have to get money from your pocket to buy it with. Secondly you must be clean, you must buy body oil and make your hair and dress nicely and you must prepare inside here (*says while laughing*) you buy inner wears.

**I: So how much do you spend in buying clothes? roughly**

PJ09: For dress it depends, you can find a second hand clothe that pleases you, and you know the price of second hand clothes differs from readymade clothes, you can get a wonderful dress from second hand worth seven hundred or even five hundred shillings.

**I: Now roughly how much do you spend?**

PJ09: For clothing?

**I: Yes, for clothing**

PJ09: I can spend seven hundred shillings but at times you find a dress that impresses you that can cost up to two thousand shillings, fifteen.

**I: Is that every week or you buy it in a month?**

PJ09: That depends, you can even buy it once in a week

**I: So the week cannot end without you buying yourself something?**

PJ09: Being smart is a must, you must be smart because you cannot also walk like a donkey. You must look for a pair of shoes that makes you attractive, you also must wear some perfumes.

**I: Does that include cosmetics and other things?**

PJ09: Yes,

**I: So in total what amount of money can you spend in a week?**

PJ09: In a week, I can say it is about four thousand shillings

**I: Okay and what is it that the month cannot end without you buying?**

PJ09: What I cannot end the month without buying, you know you have to eat too, you cannot go to work without eating, you must eat. And then if you have some people that depend on you, you must leave something (money), and when going to work you must check on the situation you are leaving behind.

**I: So how much can you spend on food?**

PJ09: On food?

**I: Because food is daily**

PJ09: In a day or…

**I: … yes, even in a day**

PJ09: In a day I can use about six hundred shillings, because in the morning you must buy charcoal, sugar, what the kids will drink you must have them and remember I have not yet included lunch and supper, so it is something that can range to six hundred shillings.

**I: Six hundred in one day?**

PJ09: Yes

**I: Anyone else, number 3?**

PJ03: I as number 3, nowadays am a bit older, it is a must that I just have to hustle, I use five hundred shillings in a day, in the morning I use two something (to mean two hundred shillings and above), then I buy charcoal and soap so I will just have to spend five hundred shillings so by end month I use like fifteen thousand shillings.

**I: So in a month you use fifteen thousand shillings?**

PJ03: Yeah

**I: Does this include the things you use at home or on what sort of things?**

PJ03: Yeah, the things I use at home like food, soap, charcoal, like I have said because we do not have anywhere to fetch firewood, we also buy water and I have to make myself attractive while going to =Fanana= *[a night club]*, I must be clean *(other participants laughing*) so those are included. there is body oil and I have to dye my hair to be black.

**I: Ooh, so that they do not look white?**

PJ03: Yes, all that is money.

**I: Okay. Is there anyone with a different opinion on what female sex workers usually buy with the money that they receive?**

PJ04: I as number 4, I can support the things they have all said, but there is something that I feel was left out, so I can remind them. I think as a...

**I: Just say**

PJ04: I as a sex worker, I think when we went there we were being asked how many kids we have, and these children go to school, and I have not heard anyone talk about school fees, to mean the money that we use monthly school fees must be included. To mean roughly the amount we use, like me I can say I use around thirty something thousand shillings when I include school fees.

**I: Okay anything else? That you buy or use, the amount?**

PJ04: What I love most is beauty, I love buying clothes, I won’t have money in my pocket or have it in my M-pesa and pass a beautiful dress without buying it or even shoes, because if you are looking good and you are beautifully dressed, yes you can be beautiful but how you dress, your physical appearance, someone can be picked from your side whom there is possibility is your boyfriend too, he will pick someone from your side and leave you there because of physical appearance, you must be presentable, neatly make your hair, dress well and use makeup too.

**I: How much can you use on your body in a month or even a week? How much can it cost you to take care of yourself from your head to toe?**

PJ04: Okay, taking care of myself from head to toe, on my side I love ready-made clothes, I can buy a dress of not less than one thousand five hundred shillings, same to shoes one thousand five hundred shillings, making my hair alone takes a thousand shillings to one thousand three hundred shillings like for example the one I currently have is one thousand three hundred shillings (20:56-20:57 inaudible)

**I: How many times do you make your hair in a month?**

PJ04: Because my hair is soft textured, I make my hair twice a month.

**I: So on your hair you use roughly three thousand shillings?**

PJ04: Yes, three thousand shillings

**I: Okay, yes, number 2**

PJ02: (*participants laughing*) I as number 2, the amount I spend even in a week, I love cosmetics, I love taking care of myself because in this work you must take care of yourself, you have to look beautiful. I love cosmetics and I also buy food because I must go to work on a full stomach. So, in a week I can spend about one thousand five hundred shillings, am a short haired girl, I like shaving my head so in a month… I also love buying clothes, in a month I can spend about two thousand shillings.

**I: Is that on self-care alone? Eh and on other things?**

PJ02: I spend about two hundred shillings on food.

**I: Is that daily?**

PJ02: Yeah

**I: Anything else, what is it that the week cannot end without you buying?**

PJ02: A week cannot end without me buying shoes and making my nails, eeh

**I: So how much can you spend in buying shoes and making your nails?**

PJ02: I buy second hand shoes, I buy at three hundred shillings

**I: Okay, and what is it that a year does not end without someone doing or buying? Number 5**

PJ05: A year cannot end without someone buying food and she has to take care of herself*.*

**I: Those are daily activities, but there are those things that you must do that are not done daily. That by the time it gets to December without doing it you will still have to do them. What are they? Number 7**

PJ07: I have a question, when I got into this work I did not even have a bed I used to sleep on the floor on a mattress. So, I decided that in that year I had to buy a beautiful bed that even if someone decides to come and spend in my house, then it would make him happy. I struggled and God blessed me with the money till I bought a bed worth ten thousand shillings.

**I: So you did not lack a bed in your house?**

PJ07: I did not, this made me really happy. So, I pray that if God allows me another year, there is something am planning to buy, if I make that money, if the work will be favorable then I will buy it.

**I: Are you comfortable to tell us what that is?**

PJ07: Yeah

**I: what is it?**

PJ07: It is a refrigerator, so if I get a visitor he gets something cold in the fridge to relax with. (*other participants laughing*)

**I: Anything else that you want to own by the end of the year? that female sex workers wish to have, or wish to do**

PJ01: I’m number 1,

**I: Yes**

PJ01: What many ladies love, is cooking gas, they see that when someone comes it is something ready, they can warm something quickly and they eat. So, most of us sex workers do not want to stay without acquiring one. They feel the *Jiko [Stove that uses charcoal]* is slow.

**I: Those are number 1’s thoughts, any other thoughts, the year doesn’t end without someone buying? Number 4 (*music playing from the background*)**

PJ04: I as number 4, a year cannot end without me sending my mum money because she is back at home and she depends on me. She knows her daughter is in =Kisumu=, something like in the middle of the year, she knows I have to send her some money of which if it does not happen, she would call to enquire on what has happened. The year also cannot end, for my mum it is a must, that I have to send her money yearly. Something else that can come to my mind is that this year I want to pay for a certain television before the year ends, 42 inches, is what can keep the kids busy to reduce fear when am not around.

**I: So if sending your mum some money, how much can you send her?**

PJ04: For my mum, as a parent, I usually send her after every three months, I usually want to send her one thousand shillings.

**I: One thousand, that is number four. Yes, number 6, what do you have to do before the year ends?**

PJ06: The year does not end without me buying maize flour so that even when I get a client, I don’t have to go and borrow from the shop. I want the client to wake up and find the ugali is ready.

**I: Is that in a year?**

PJ: They are asking in a year what you buy

**I: Or something that you would like to buy in a year. Yes, number 8**

PJ08: Number 8. In fact, in a year, I look to raise something like twelve thousand to pay school fees when the child is going to form one. So you know if you don’t raise that school fees it becomes hectic. It becomes a problem to you, because you find that the child wants to go to school and you are everything. So, where you will start… maybe work is bad. So I think that saving in another angle can help us very much.

**I: That is the opinion of number 8. Number 2?**

PJ02: I as number 2, in a year I consider my mother. I have to send my mother something every year.

**I: When you send her something, how much can you send?**

PJ02: I send her even two thousand, five hundred.

**I: Number 9?**

PJ09: As number 9, the year cannot end without me sending my mother something.

**I: how much can you send her?**

PJ09: In a year I can even send her five thousand.

**I: Okay. Where do female sex workers get the money they use from? Mostly, number 7**

PJ07: Mostly we get it from clients. It depends with the client you get, how you deal with them so that they can give you good money. That is something I can tell you. If you are dull, he cannot give you the money you want. If you are dull he will not give you the money you want. That is why I said earlier that you have to go when you are looking good. Because when he sees you are looking good, even if he says he will give you two thousand shillings, he will see how you look and think if I give this girl two thousand it will not be good. So, my people who are here let us look good so that we can get more clients. That is my opinion.

**I: That is number 7’s opinion, from clients. Yes, number three, where do they mostly get money from?**

PJ03: Mostly it is just from clients.

**I: From clients. Number 5**

PJ05: Mostly they get from clients

**I: Clients. Yes, number 2?**

PJ02: From clients.

**I: Number 9?**

PJ09: Mostly it is just from those clients

**I: Clients. Number 4?**

PJ04: Clients

**I: Eight?**

PJ08: Obviously clients

**I: Clients. Six?**

PJ06: Just clients.

**I: One?**

PJ01: Yeah, those people.

**I: Who are those people?**

PJ01: Those clients.

**I: Okay. Apart from clients, we have all said that we get money from clients. Apart from clients where can they get money from?**

PJ04: Depending on themselves.

**I: in what way?**

PJ04: when we started we said through saving.

**I: That one we had said. But as for now, where do they get money from apart from clients?**

PJ01: I am number 1; I can say that they can get money from their business that you can rely on.

**I: So, business, that is number 1. What type of business?**

PJ01: Business like us we work in the hotel. So, during work is work and time for other work is just work.

**I: So, when you say work and other work, which is which?**

PJ01: They are two jobs. So, the one for the day, you can go and work in a hotel and during the night you go for sex work.

**I: That is number 1.**

PJ08: Number 8. Getting money, how we get money… you can find that at times we have gone for sex work and you know sometime you have an agreement with the person at the counter. So, sometimes you find that when you have been bought for drinks, there is a way that you calculate one or two because sometimes you find that you have been bought for four bottles and your house was not so good. You cannot drink all of it. You look for a way how you can hide a couple of bottles and take them back to the counter quickly when this person has gone to the toilet and you give the person at the counter. When you come back you return with empty bottles and put on the table. So, you find that we also have these challenges and we have to also lie so that at least we can achieve in life, because when you are straight forward sometimes you hurt yourself. Yes, like that.

**I: That is number 8, other sources. Anyone else? Number 3, where do they get money from apart from clients?**

PJ03: As number 3, before leaving going for sex work, I usually sell charcoal at my doorstep for my neighbors to buy. When it gets to evening, I close up and go for sex work.

**I: So, during the day you sell charcoal?**

PJ03: Yes

**I: Okay. Yes, number 5?**

PJ05: Like me, I can go… I have my work. I can cook chapatis and get some money before leaving.

**I: Ok. Number 9, where do they get money from, apart from clients?**

PJ09: Apart from clients, you know even if you have clients, you have to have a small business so that you can look after it… where I can get money from apart from them is just that small business that I do.

**I: Number 2**

PJ02: I as number 2, where I get money from before I go for sex work… during the day, let me say I can do service in the hotel that is combined with a club that is opened in the morning.

**I: Ok. Why do female sex workers buy the things that they buy? We had found out that people buy a lot of things right? There is someone who has said she has to buy clothes, make up. Those are examples, right? Why do they buy such things or the things that they buy? Even if someone buys a bed, why does she buy a bed?**

PJ04: Me as number 4, the reason why we have to buy those things, is just for attraction. If you are looking good, you will get a lot of clients. The bed that you have mentioned, maybe you have come with the client to your house and when he sees the bed itself, there is nowhere else that he will go to. He will just look for another friend of his, and tell you that the money you are going to use to book a room with, let me talk to that person so that you just go and use that bed.

PJ08: Number 8. The reason why you mostly find sex workers buy something, is because she does not look at that only. She looks at the future to come, where will she stay? That is one. Because you find that when she is buying these things, she is buying things for her house, she is not buying for anyone, she is buying things for her house. And when she is buying things for her house she has the idea that she will one day leave this work, because she will not do this work for life, that is one. So, you start looking in the future and in future if I do not buy this things, how will it be? Then secondly, the reason as to why we sex workers mostly buy things is because there are those clients that come to your house, and they are of different classes, there are those of higher class and those that are of low class, so there are those clients that when they come to your house and see how you have decorated your house, they will put you on some class, even if I usually pay this lady one thousand shillings it is little for her, let me add her. So, mostly that is why you find people buy these things.

**I: Number 2**

PJ02: I as number 2, her first point where she has talked about the future, you cannot just do this work in the name of working without planning on something to achieve you must achieve something. It is good to buy households items because one day when you stop this sex work and opened your business, you will see that it is better there was something you bought, so it is better you buy them.

**I: What kind of household items do you buy?**

PJ02: You can start with items like kitchen wares such as utensils, you buy chairs, bed, like you just make the house.

**I: Approximately how much does it cost you, if you can guess? Average, anyone or number 2**

PJ02: Someone can buy things worth two thousand. Let me say for example, something like a table, bed or even plastic carpets.

**I: Okay so at least two thousand shillings, anyone else with a different thought? Number 1**

PJ01: I support number 2 the things she has said are the most expensive that people usually like. mostly us sex workers do start with things like utensils and carpet or even a small mattress when you still don’t have a bed to start with.

**I: How much can you use?**

PJ01: I can say you can start with a mattress worth two thousand shillings, a carpet worth five thousand shillings, utensils can also be about five thousand shillings.

**I2: Why are you buying these things, that is the question we are asking.**

PJ01: We buy them because, even if the clients come to the house, he does not find that you live a careless life, because he might come and find you sleeping on plastic. Even if he wants to give you one thousand shillings, he will now give you two hundred or three hundred shillings. So that is the reason.

**I: Yes, number 5 why do they buy the things they buy?**

PJ05: They buy them to decorate the house so that when the client comes they will know you live a good life.

**I: Okay, when you are speaking speak a bit louder because of the music in the background. Okay are there any female sex workers you know who save money? Or do all the ones you know not save?**

PJ07: I as number 7, I have an answer to that. As sex workers we had started a merry go round amongst us but most it never used to work because you sometimes find the treasurer has disappeared with the money so that made us to stop. So if God blesses you with some amount of money, you can save some in the mpesa and use the rest because you don’t know what will happen tomorrow.

**I: So does everyone save on their own?**

PJ07: I don’t know about others but as for me when I get a good amount of money I do save some on the phone and use some because I have a child, he can be sick and what I saved will help me.

**I: Is that the reason why you save?**

PJ07: Yes, that is the reason I save.

**I: And when you save, how many times can you save in a week or even a month?**

PJ07: In a week the way I know myself, if I get there with good luck, I can get a good client who would give me even six thousand shillings or one who would promise to buy you something and sends you about twenty thousand shillings, so if I receive twenty thousand shillings I save fifteen thousand shillings and spend five thousand shillings.

**I: Okay, anyone else? Number 4 you had said something?**

PJ04: I as number 4, I was saying that saving is good, we have to save something because you don’t know about tomorrow. Even a step that you are at, you have to save.

**I: So, are you saving for the future?**

PJ04: Yeah

**I: And how many times can you save in a week?**

PJ04: Okay, I cannot say that I can save twice or even once every week, but it depends on how many times you have gone for sex work in a week. So, anytime you take a vehicle to go to town, when you come back you have to pass by the M-pesa shop to put some amount.

**I: If you put some money in m-pesa, how much do you put?**

PJ04: If I deposit on the lower side, I deposit five hundred shillings that is the amount I always want to deposit at least five hundred shillings anytime.

**I: Okay, yes, number 6? (short pause) we are talking about savings.**

PJ06: I just support what they have said.

**I: What have they said?**

PJ06: About savings.

**I: Who do you support?**

PJ06: I support number 7.

**I: What has she said about savings?**

PJ06: That if you receive money you can deposit it in m-pesa.

**I: Do you save? (*Silence)* let us be in one meeting please, if there is anywhere you did not hear well it is better if you please ask.**

PJ06: I did not hear it well.

**I: Yes, number 8**

**NT: She is asking that do female sex workers save? Do you save? Or other women that you know, do they save?**

PJ06: I have to save because I can get an emergency and if I don’t save I can have problems so I just have to save.

**I: Yes, number 8 you had something to say?**

PJ08: Yes, the question that the teacher asked I think she has asked if we have seen sex workers who save. On my side number 8, I have not seen ones who save. Maybe it will be the first time that I will meet a group that saves. Like for example in this discussion, this group called Jitegemee. So, you know if we can get it… on my side I have not seen any. Maybe now I am going to see. We can decide that we are going to start saving. But mostly you can find that most sex workers, you find them say that even tomorrow I will get. So, today if I get you find that maybe the needs I had today are more than the money I got. I will try to save that money but it will not be possible. So, in another angle, like this program that has come like for Jitegemee, I think we can decide the way we are here, we can decide to save. On my side that is my decision.

**I: Is there a reason why they save, for those that save is there a reason why they save? Yes, number 5.**

PJ05: As number 5, there is a reason why they save because you can get any problem and that saving can help you with the problem you have.

**I: Which type of problem?**

PJ05: The child can fall sick or even you can fall sick, and you can start with that which you had saved.

**I: Okay, number 3**

PJ03: In savings, we used to save but the interruption from COVID interfered with our group. It seriously interfered with the group and we had maintained saving that even if you needed financial help, you would go talk to the chairperson and you would be sorted. So since COVID spoilt things, savings stopped. however much we tried to save, you find that the treasurer would say so and so took some money and refused to pay back, so and so took money and refused to pay back. So, we felt that we should stop so that every person to save money on their own. So, mostly I save on the phone.

**I: Was there a reason why you were saving?**

PJ03: The reason why we started saving, as for me I have children that go to school. I would save so that when schools open or when anything is needed at the school we go and meet and they decide that let us give so and so some money to settle issues in school. That was the reason why we saved there can be sickness and even apart from sickness any problem can arise, so it won’t be an issue running around looking for money instead you talk to the chairperson who calls other members and they agree and you are given money. So, since COVID came that thing did not go well and business is also not going on well. So, the group divided that now everyone is saving on their phones.

**I: Okay, thank you, and is there a characteristic that women who save have? That if you look at someone you can know that this person usually saves?**

**NT: Do they have a certain behavior, a character that is different from someone who does not save?**

PJ08: Something I can say about those people who save or what those who save can do, I am number 8, many women face challenges wherever they are, for example when we had curfew hours and if it was past time you know the police will arrest you and if you are in a group that can help because you are going to call your leader and tell her that this and this happened, and there is money that is needed if you can help me with such an amount, this thing has caught me off guard. Another thing, us sex workers, you find that what happens is that sometimes women face challenges where we are. You can find that maybe someone called you and it was one person but when you get there you find that they are two people and they want to pay back and sometimes you can be hurt badly. So, you know at that point you have the opportunity of calling the chairlady so that she can look for a way that you can be helped.

**I: Any other character that the women who save have?**

PJ07: I as number 7, I have seen many women who save. You find that when they go to work at night they forget about what took them there, she can get up and decide that she is buying drinks y herself she is not working, so it makes the others think that so and so is boasting because she has money. So, she buys drinks by herself and says that whoever has not saved her money, now see what money is. She can buy a lot of drinks by herself. You find that by herself she buys even ten bottles of Guinness at once. So, that is the character I have seen with women who save.

**I: In women who save? Number 9, what character do women who save have?**

PJ09: I did not get the question well please pardon me

**I: Women that save what characteristic do they have? That if you look you can know that this woman saves.**

PJ09: I have never seen them show me any bad character that I can talk about.

**I: Do they have good characters or bad ones?**

PJ09: I cannot judge.

**I: So you cannot talk about their character?**

PJ09: Yeah I cannot talk about their character.

**I: Number 2**

PJ02: I as number 2, I am unable to respond to that question.

**I: What has happened?**

PJ02: I cannot answer.

**I: Okay, you cannot answer?**

PJ02: Yes

PJ01: Number 1, a great number of the women who save have disrespect. Just as number 7 has said, she can come somewhere and finds others, she wants to show you that she has something different from what you have. When you have come to look for something, her she already has. So, she can disrespect you in front of others. That is why women who save have bad behavior.

**NT: Okay, let me put it this way…**

PJ08: …Number 8 first. Women who save are confident. They are confident because as in… even if you don’t buy her anything she can buy that thing by herself, that is one. That is, they have courage. And women who save, cannot beg someone in another angle because if you like you give her, if you don’t like you don’t, because even if you don’t still I can do what- I can survive, because on another side she is saving. So she has confidence in herself. Yeah.

**NT: Okay, now we have heard about the characteristics of those who save, right? Meaning there are characteristics of those who do not save, right? What we are trying to find out is what do they do so that they have the ability to save? For those who save, what do they do so that they are able to save and others cannot save? How do they live to be able to save the little that they have?**

PJ07: I as number 7, my answer is this, there are those that if they get two thousand shillings, they want to misuse it all in a day. They do not see the need of saving, they will say that even tomorrow she will go back to work and get other money, and sex work is different. You can go there and not get anyone to buy you even the small soda of fifty shillings, and you come back home the way you left. So, that is the difference that is between a person…

**I: …So that is someone who does not save, right?**

PJ07: Yes.

**I: Any other character for those who do not save?**

PJ03: I as number 3, people who do not save have challenges when they go for sex work because when she gets there and takes a seat she only depends on that sex work. So, she wants to sit at the table without anything. She is seated looking at those coming in, she is seated looking at those coming in. So, you know these people who also come in if they see if you are a lady who cannot buy anything, because there are also men who you have to start with the first bottle is when he can give you the one that you want. So, where you are seated you only look at the people who are coming in. that is the bad thing about women who don’t save.

**I: That is a character of women who do not save. Any other characteristic? Number 5**

PJ05: Another characteristic?

**I: Yes. Speak a bit louder.**

PJ05: I will just support number 3 on what she has said.

**I: What did she say?**

PJ05: About women who do not save.

**I: What has she said?**

PJ05: She said that women who do not save, when you go to the bar you just look at people pass by, how people come in.

**I: Okay, number 6 what characters do women who do not save have?**

PJ06: I can only support what number 5 has said.

**I: What has she said?**

PJ06: If you do not save and you are at a bar, you keep looking at those coming in and you are seated just waiting for customers.

**I: Is there any other characteristic of women who do not save? And we have found out that there are those who save, right?**

All respondents: Yes

**I: What has made it easier for them to save?**

PJ07: As number 7, the problems they have faced. Like me a sex worker, you can find that your child is sick and you don’t have money. When you go to your friend, yes, she will help you and when you leave you will hear her say that so and so goes out every day and what is it that makes her go out? So, that is what you will hear. That is something that happened to me. So, you will feel that so and so helps me and talks about me. So, it does not give a good feeling, and you think to keep the little that you get. That is why I started saving.

**I: That encourages them to save?**

PJ07: Yes

**I: Anything else that encourages them?**

PJ03: I as number 3, the reason as to why am saving or the reason why other ladies are saving, at times you are coming from a humble background and you are the one people depend on. So, when you are going for sex work, you leave the house praying to God telling him to take care of where you are coming from. I am also living alone. I have also left the children with my mother. So, it forces this woman that the little she gets, she has to save a little while considering where she is from and how she lives. That is why it will force her to look good so that she gets customers and get the money to save.

**I: Number 9 what is it that encourages them to save?**

PJ09: I want to support number 3 the way she has said. You can find someone who truly comes from a family with a humble background and this is also your way of life. That is where you get your income from. So, you will just have to, no matter how little you get, you save half and use half to help yourself in the life tomorrow because you don’t know about tomorrow.

**I: So those are the things that encourages them to save, and is there any challenges they face? women that save do they face any challenges?**

PJ03: I as number 3, the challenges they face is if she goes for sex work and does not get a little to save, will be hurtful for her because she will think how she is and how her life looks like. There are women who go for this sex work because of difficulties and she has to save half and spend half. That if she does not get the money to divide, she will feel hurt or find it difficult.

**I: So sometimes there are no clients?**

PJ03: Yes, sometimes she does not get. So, she has to feel burdened.

**I: Okay, that is a challenge. Number 1, the challenge she can face?**

PJ01: I support number 3, challenges arise when you lack and you had saved, it will force you to spend that which you had saved, to assist your family and find a way to eat. This is the challenge to saving.

**I: Number 8 do you have any challenge that they can have?**

PJ08: Challenges that people who save can experience, challenges are there because you want to save, you must dress, you want to eat and do some other things and sometimes the money you get is less. So, challenges must be there because you want to save and spend and maybe it is less. So those are the challenges that people face.

**I: For the women who don’t save, is there a reason why they don’t save? Number 2**

PJ02: As number 2, women who don’t… repeat the question.

**I: Women who don’t save, why do you think they don’t save?**

PJ02: Sometimes if you go, like this our sex work, you go and find, you can’t know how work will be like. You go and you don’t make enough money. So, sometimes when you come back you will use all that money. You use it in all your budget. So that can make some people not to save. You get little money; budget is also big. So, you use the whole of it. So, sometimes you cannot save.

**I: Number 4 the reason as to why they don’t save?**

PJ04: Silence (*other* *participants* *laughing*)

**I: You seem to be far away, please come back we are moving at a good pace. What is the reason women don’t save?**

PJ04: Reason why women do not save is, I can support what number 2 has said. Sometimes, like this our work, I have gone to town and I use fare, isn’t it? When I come back I will also use fare. When I come back the child is depending on you, you also want to cook so that the children can eat. It will force you to use all the money that you had, and you are left with none. So, that has to be a challenge.

**I: That is challenge as to why they do not save, number 8, yes.**

PJ08: Challenges that make them not to save, is lack of knowledge, you know when you are not taught about what savings does, I cannot save I will just be spending all the money. For now, through Jitegemee I can start saving because I know that if I can save a little and spend a little it can help me in future when faced with problems. So, lack of knowledge also makes us not to save. If you don’t have anybody who has told you about the importance of saving, then you may not save.

**I: What is the advantage of not saving?**

PJ08: There is none, number 8

**I: That is number 8’s opinion, meaning the rest have advantages, give me the advantages of not saving.**

PJ01: There is no advantage of not saving because it is good when you get a little, you spend a little and save a little so there is no benefit in not saving.

**I: That is number 1, the rest?**

PJ03: There is no advantage in not saving.

**I: Number 3 says there are none. The rest any advantages of not saving?**

PJ02: None

**I: Number 2 says none. Number 5 advantages of not saving?**

PJ05: There is no advantage.

**I: There is no advantage, number 7?**

PJ07: I support number 8 there is no advantage of not saving.

**I: There is no advantage, what about disadvantages?**

PJ07: There is a lot of disrespect because you can find that… sorry am number 7 my thoughts are, not saving, you can find that if you rely on that you are going to get other work and you are not able to then you get locked out of the house, like us sex workers and you want to sleep in my house, you know I am also going to tell you that there is a relative of mine from the village who is resting here, because you go out every day and I also go out, I will not agree to that easily. So, that is the disadvantage of not having. People laugh at you. You look like a mad person in the midst of people.

**I: That is number 7’s opinion. Disadvantages of not saving?**

PJ08: Disadvantage of not saving, I am number 8, we have realized that there is nothing we gain in life. We live from hand to mouth. We spend the whole of it that if someone gets a problem, there is no way to help themselves. So, there is no good thing. It is just disadvantages.

**I: Someone who is starting to get tired, number 9 disadvantages of not saving?**

PJ09: Disadvantage of not saving, I am number 9, it is good because, because you can have a problem at any time, and you know when you have saved something a little, there is a way that can help you.

**I: Please just come back we are doing well, okay? Don’t drift off too much.**

PJ09: Okay

**I: Do not go so far, we are moving and where do female sex workers like to save?**

PJ04: I as number 4, women love saving on phone.

**I: On phone, number 9?**

PJ09: Phone.

**I: On phone, number 2?**

PJ02: Phone.

**I: Phone, when you say phone how do you mean? Number 4**

PJ09: Mpesa

**I: Number 9, Mpesa**

PJ04: Mshwari

**I: Number 4, Mshwari. Number 2?**

PJ02: Mpesa

**I: Number 5**

PJ05: There are some that save in banks.

**I: They prefer banks. Number 3**

PJ03: Mpesa

**I: Mpesa. Number 7**

PJ07: Bank

**I: Bank. Number 1**

PJ01: You can save even through a relative or even a neighbor, it is not a must that you go that far, because you see you can depend on that maybe I don’t have, let me go to so and so quickly, and when I don’t have fare when will I go to the bank. There is no way I will go. You can save even with a person you trust.

**I: So, you prefer a person because you can easily get access?**

PJ01: Yes, I can easily access them.

**I: Okay, number 6 where do you like saving?**

PJ06: Mpesa.

**I: Mpesa, number 8.**

PJ08: Mpesa.

**I: Mpesa, why have you chosen Mpesa?**

PJ08: Because most of the time I have Mpesa close by, so when I get any problem then I can just withdraw immediately.

**I: That is number 8’s opinion. Number 6 why do you prefer saving in Mpesa?**

PJ06: If I get a problem, I can withdraw easily and quickly.

PJ08: Another thing why I have chosen Mpesa, number 8, I have forgotten, my money cannot get lost in Mpesa. Yeah, I was forgetting that.

**I: Number one had told us why she prefers a neighbor, number 7 why Mpesa?**

PJ07: I said bank because if I save on Mpesa I misuse it because I am someone who uses these things you taste. So, when I see it I can start calling by myself the way I had said earlier. That is why I prefer the bank.

**I: What are this tasty things?**

PJ07: Things like alcohol.

**I: Number 3 why Mpesa?**

PJ03: The reason I prefer Mpesa, is because I have children who within a short time they want something and I just rush to the market and withdraw.

**I: Number 5 did you say bank or Mpesa, what was the one you said?**

PJ05: Bank

**I: Why bank?**

PJ05: Because I cannot misuse money when I have it saved there.

**I: Number 2 why Mpesa?**

PJ02: Mpesa helps you very fast.

**I: Very fast, how?**

PJ02: Let us say I want to buy something and that place it is written Lipa na Mpesa, then I can just pay it.

**I: Number 9 why Mpesa?**

PJ09: Reason why I save through Mpesa, you can have an emergency where you don’t even have transport to go to town, and you know with Mpesa if you have befriended an agent you can just send the child from your house to go to the agent and get it for you.

**I: Okay, number 4 why Mshwari?**

PJ04: I chose Mshwari because with Mpesa, the reason why I don’t like MPesa is, I like to google things we use a lot of bundles, I don’t use alcoholic drinks, you can find that you buy credit until all the money is spent. The second reason why I don’t support Mpesa, depending on my nature, you will not joke with me that [name omitted] buy me this thing and I don’t buy it for you if I have money in Mpesa. So it is better to use Mshwari than Mpesa.

**I: Okay, so there is a place we talked about Jitegemee do you remember that? Do you think it is something female sex workers would like?**

PJ01: I number 1 agree with it.

**I: You agree with it, what about the rest?**

PJ05: I as number 5 I too agree.

PJ06: I number 6 too I agree with that.

**I: Why do you think they will agree to it?**

PJ08: Number 8, they will agree with it because according to the teachings we have been taught, you know while we were here we were not just talking for the sake of talking, we were learning on how we can save then we have known the advantages and disadvantages of saving, so we can agree with saving.

**I: That is number 8’s opinion. Anyone else why do you think they will accept it? Yes, number 4.**

PJ04: We will accept it because we are planning for our future life.

**I: Number 2, why do you think they will accept it or not accept it?**

PJ02: They will accept it because you will find that someone might think that if she has done this work or… we have talked about savings, someone can do this work for up to five months and save and start a business, so she depends on that business. She has now stopped sex work.

**I: Okay, number 5, reason as to why they can accept or not accept Jitegemee?**

PJ05: They can agree because it has taught us about savings.

**I: About saving, and what type of women can agree to join Jitegemee or can accept its interventions? what type of women or those with what characteristics? Yes, number 1.**

PJ01: Character does not determine joining Jitegemee, that depends with your heart and those are the type of women who can join Jitegemee.

**I: What character do they possess?**

PJ01: They are just normal people like me without relying on the physical appearance but it will depend with your heart.

**I: So it is what someone thinks of?**

PJ01: Yeah what you put your thoughts in.

PJ08: Number 8, mostly single ladies can get a lot of help from Jitegemee, because you can meet a single lady who does not have means of surviving. Then secondly, we are sex workers and we can agree that Jitegemee can help us.

**I: We are talking about sex workers which type will accept Jitegemee? We have different characters isn’t it?**

Chorus response: Yes

**I: We cannot have similar characteristics.**

PJ07: I number 7, I can join because there is a day we lost a friend whose family did not have means, we were in the group that I had said broke up with this colleague. These ladies stood together with her and we collected funds that took us from here to =Ugenya=, everything was ours, and it reached a point that when we got there, we were called prostitutes. We were not afraid because that is our work. we were verbally abused till morning and it was raining. We also abused them and it was raining, and there was a time the guests who were there came to us and started flirting with us. So, others started saying that so those ones are also their fellow prostitutes. So, I can join because I have seen its benefits, a lot. And it also needs someone who is not afraid, because one of your own can pass away and there is someone who is afraid and say she does not want to go where there are other sex workers so that people will see me. So, once you commit yourself, you will not do this for life, God will one day give you a man to live with so do not be afraid love your work as it is.

**I: So, those are courageous ladies?**

PJ07: Yes, you are not afraid.

**I: Okay, any other? number 5,**

PJ05: Just as number 7 has mentioned I support her.

**I: Any different character from courage? What type of women can join Jitegemee? Number 9, what type of women can join Jitegemee?**

PJ09: I can choose to join because according to the teachings I have received it can be of help if I join, even if I have a problem my fellows here cannot abandon me they can stand and support me.

**I: Number 4 do you have something to say?**

PJ04: According to what number 4 said, the reasons why I can join Jitegemee, it is said that no matter how wealthy you are, you should not rely on your parents, you have to rely on yourself. Sometimes I am in =Kisumu= the way I am in =Kisumu=and our home is in =Homabay=. I encounter something here then my mother is being called from that other side. Before she helps you, she will talk badly, that is something that is true. She will say, why is she calling me and yet she is working in =Kisumu=. So, you have to have something small to take her to the hospital or something like that. So, that is why we have to be independent.

**I: Okay so those are ladies with responsibilities?**

PJ04: Yes

**I: Okay, out of ten women that you know, how many can agree to join Jitegemee?**

PJ03: As number 3, we have all gotten the lessons we were taught, so each and every person who knows they have any problem to follow the lessons we have gotten from the teachings. We can come together as a group to save something that if any of us is faced with a problem just like number 4 has said her home is far, we will see what can be done not keep disturbing the parents but at least from the little saving we shall have started something.

**I: So, from among the ten women you know how many do you think will accept?**

PJ03: I don’t know

**I: Not from here but those that you know.**

PJ03: For those that I know I will have to talk to them about how we have been taught so that those willing can join.

**I: How many? out of ten.**

PJ03: There are those if told can agree to join.

**I: How many?**

PJ03: You know we don’t have them here we just have to talk to them.

**I: Number 7 among ten women that you know how many do you think can join?**

PJ07: I can get at most thirty because I have those that are serious.

**I: Okay out of ten how many?**

PJ07: Out of ten I can get five that are serious.

**I: Five, number 1 out of ten?**

PJ01: Seven

**I: Seven, number 6, out of ten?**

PJ06: Four.

**I: Four, Eight?**

PJ08: Three.

**I: Three, Four?**

PJ04: Three are ready.

**I: Three, Nine?**

PJ09: Two

**I: Yes, number 2?**

PJ02: Three can be ready.

**I: How many, three?**

PJ02: Yes.

**I: Number 5 how many?**

PJ05: I can find five.

**I: Five, number 3 how many?**

PJ03: I can find two.

**I: Two. Okay, the remaining ones, why will they not agree to join Jitegemee?**

PJ04: I as number 4, what can prevent someone from joining Jitegemee one, it depends with someone’s behaviors then the second thing, if they just hear you start saying “a group of sex workers”, they will just tell you that [name omitted] the way you have started, just stop it from there.

**I: So if we mention the name sex workers that is what will prevent them from joining?**

PJ04: Yes

**I: That is number 4’s opinion, because I asked about ten women that each of you know right? So, seven out of ten that will refuse for [name omitted] sorry, for number 4, will refuse because of?**

PJ04: The name of the group.

**I: The group name. yes, number 9 why will the others refuse? The seven people remaining?**

PJ09: The reason as to why they will not agree, I know deep down they will want, but according to that name, I know they fear that name but in their hearts they are willing.

**I: Number 2 why do you think they will decline?**

PJ02: The reason they will refuse is introduction as sex worker.

**I: So, the name sex worker?**

PJ02: Yes, after it comes out like that then…

**I: Number 5 something different from the term sex worker, why will they decline?**

PJ05: They will decline because, sometimes she might not get a chance to attend those groups.

**I: The chance to attend, yes number 3?**

PJ03: I as number 3, the reason why they cannot come… I can just look for someone who am sure will want to join the group that I am in. The second thing is that when we are in a group, the person who can get little and save because most of the people now are bad when it comes to money matters. If she joins she will just join if we had stared it like table banking, after she gets her money then she will disappear. So, you just look at a person’s intention so that you can invite them to the group.

**I: Number 7 why do you think they will not come?**

PJ07: The reason why they will not come is this, there are those people who talk about those groups that after contributing up to around halfway they start complaining about the group and issues of money, that is why talking to people can be difficult. So someone feels she can save on her own.

**I: Number 1, why will they not agree?**

PJ01: They will not agree, just like the others have said, is the name, the sex worker name. so, the person who can be comfortable can just come. I can get those who are willing.

**I: Number 6, why do you think they will not agree?**

PJ06: They will not agree because of being busy. Being busy

**I: Ooh, they are busy?**

PJ06: Yes

**I: Number 8, why do you think they will not agree?**

PJ08: The first one is introduction, the second one is being busy, the third… teacher why are you laughing? (*other participants laughing*) the third one is about saving. You know you have to tell someone and eliminate her a little *(she meant educate her)* so that she can be at the level of Jitegemee, because I can have ten people and if I just go to them and tell them this and that, we have a group called Jitegemee can you come? I can find them. But it is going to be difficult if you had not taught her… if you had not taught me before I was to going to accept and it depends with if I am going to teach them how you have taught me, is when they can agree. So, it depends, there are those who will agree, there are those who will refuse.

**I: So for those who decline is because of those reasons?**

PJ08: Yes, those reasons.

**I: And the fourth reason? Or there is no fourth reason?**

PJ08: Fourth reason, (*laughing*) there is none.

PJ07: I can say the fourth reason. Reason number four, there are those that are doing sex work but they are not committed. If you are in town you are okay but when you meet here in =Otonglo= she is your neighbor, she does not know you. So, that is a problem that is in our midst. They don’t want to show that they are sex workers. But once you are in sex work, be free so that you can find a way to help you.

**I: That is number 7 okay. What can we do so that it can be accepted?**

PJ08: Number 8, you know first of all the name, introduction as sex worker, it is okay we are sex workers, but that introduction we get look for another name but inside the group we know each other we are sex workers, that is one.

**I: So we do not use the name sex workers?**

PJ08: Yes, then the second thing, we can ask them about the time that will favor them. We can also ask them if they can be available and if it is okay. I have those two points.

**I: Something else? Anyone with a different opinion? something that we can do for girls to accept it? To take it as their own.**

PJ09: I as number 9 I support number 8. we can look for a different name that we can have but among ourselves, like she has said, we just know what the group is for.

**I: So if you can give me an example, how can that other name be, so that even if we say it in public, the girls would still know that we are talking with them but maybe other people do not know what we are saying?**

PJ01: Number 1 I can say you can say a player that is the only option. A player.

PJ08: Number 8 I can say hard working girls, the reason am saying this, for someone to decide to join sex work it is not an easy task, to mean she has volunteered to do that kind of job in order to get the little to sustain herself that is why we can give that name. Or being determined sort of.

**I: What can we say if we remove the term sex worker?**

PJ06: I as number 6 I support number 8, about the name hard working girls I like it.

**I: What can we do in order for it to work better?**

PJ07: If we are hardworking then it will be better because if we are not hard working then it will not be good.

**I: What can we do in order for it to be much better when we are starting Jitegemee?**

PJ08: Commitment of time.

**I: Time commitment, something else?**

PJ: Absenteeism…

PJ04: … you should respect each other.

**I: Respect each other, something else?**

**NT: They are not saying their numbers**

**I: That is number 4 that has talked. Yes, number 8?**

PJ08: We try to omit absenteeism. So all of us come. We try and come all of us.

**I: Anything else that we can do for Jitegemee to be much better?**

PJ05: I as number 5, just to keep time.

**I: Are there things it should have in order for the girls to accept it, when we implement it?**

PJ06: I as number 6. We can have some tops that we put on while here.

**I: Number 7.**

PJ07: I as number 7, when we started ours for the sex workers, we would be meet in town, we had one of them who was assisting us as our leader, we started as only ten people, the number later increased to fifty of which if it were not for COVID we could be two hundred by now. So if we have one that encourages others, because you can tell someone, the level of dishonesty is higher in people nowadays, they can go the other way or the other way so that is my request.

**I: Something else it should have as Jitegemee? Something else, it is not chamaa because you are just taught by yourself and you do it by yourself. So what things should it have? Yes, number 8**

PJ08: Just the way she has said, you know for example now I am going to get even ten people and someone has left her work that she does during the day and at night she is somewhere else. So, that allowance will encourage some people. So, when that allowance is there, it should just be continuous. It will help a lot of people.

**I: Any other idea what it should have?**

PJ03: I as number 3, I support number 8 in what she has said that is what can make many people to attend.

**I: Number 2 any other thought what we can do that can make others accept it?**

PJ02: I as number 2, I support number 7. It is good if we have someone that encourages us. That can allow more people to join.

**I: Okay, like we had said that Jitegemee is a way of encouraging sex workers to have a means of saving money so as to have a chance of refusing unsafe sex or they can take leave. What do you think they can like about Jitegemee as we had said? Can I repeat number 4? Say it loudly, if you want me to repeat. Okay number 4 has not gotten it I will repeat. What do you think that female sex workers will like about Jitegemee?**

PJ07: First, you have to be people who understand each other so that you can give each other ideas to help yourselves because maybe if I don’t get along with number 8, I cannot sit together with her and talk. So, you have to be people who understand each other so that each person can encourage the other, because I may not be able to see my own fault if someone does not tell me. That is my opinion.

**I: Any other thoughts, about what they will like about Jitegemee? Number 1?**

PJ01: What they can like about Jitegemee, I am number 1, is coming together. Coming together is a wonderful thing. Number 7 has said it well that if they are not in good terms with number 8 you know there will be differences there that even if we go to work they will not agree. So, the thing for people to like about Jitegemee is (1:22:48-1:22:50 inaudible)

PJ02: Number 2

**I: Number 2, what they will like about Jitegemee**

PJ02: If they take it there, where they encourage each other, they get different ideas that can be of help to them.

**I: Number 5, what they will like about Jitegemee?**

PJ05: I as number 5, what they will like is the idea about saving they will like it.

**I: Is there anything that they will not like? something they will not like number 4?**

PJ04: What they will not like, the moment that they are together what I know they will not entertain nonsense.

**I: Nonsense, what are the examples of nonsense?**

PJ04: For example, as we are here, if we are talking and someone is (1:24:00-1:24:02 inaudible) am talking here and someone is talking there and what we are saying are different at the same time.

**I: That is an example of nonsense, yes number 9 what they will not like about Jitegemee?**

PJ09: I as number 9, what they will not like…

**I: …Talk loudly please**

PJ09: What they will not like is like disrespect and gossip, that what is said here, someone starts talking about it somewhere else and you also know when she shares it out there she will not say it the way it was said here.

**I: Number 2, what they will not like.**

PJ02: I just support number 9, issues with gossip people will not like.

**I: Number 6 what they will not like?**

PJ06: I support number 9, what you hear and spread that someone did not say

**I: Number 1, what they will not like?**

PJ01: Same, what they will not like is here and there. Carrying information here and there. Maybe you have done something when you are two people then it is going to become three people. That is something someone will not like.

**I: Do you think Jitegemee activities will bring issues of infringing someone rights?**

PJ03: I as number 3, it will not violate someone’s rights, because it was a nice teaching about savings, so anyone who feels it is good can just start saving as we have been taught.

**I: That is the number 3’s opinion, someone else? To the rest you feel that it will violate your rights. Why? Why do you think that way?**

PJ08: Number 8, it will not violate someone’s right because for you to join Jitegemee, first the teacher has taught you, the second we have sat in the class so since I agreed and signed, it means I had accepted and it is not violating anyone’s right I have already committed myself.

**I: That is number 8’s thoughts, the rest? How will it violate your rights?**

PJ07: Number 7, I support number 8 because if I was not interested I would not be here, I would have not signed that form but for me to sign it means I had read and understood what it is like, if I did not like it I would have not signed it so it does not violate my right.

**I: So it does not infringe your right, number 5?**

PJ05: I just support number 8 I as number 7, I am number 5.

**I: It will not violate your right, okay and do you think we can face any difficulty when bringing Jitegemee? Challenge, challenge that we may face.**

PJ08: Number 8, challenges are there because I might have committed myself to be in Jitegemee, then the next minute I relocate to =Obambo= fare that will ferry me from =Obambo= to here and it is exactly that time that am needed here and am from =Obambo= it means am going to face a challenge.

**I: Transport challenge?**

PJ08: Yeah, so if you can offer us transport it can be a good idea, because I might be staying here and am not a permanent resident so the next time I might relocate to =Mosco= So transport can be a challenge.

**I: So how do we make it?**

PJ08: I do not know how issues with transport can be facilitated, you know that goes back to the head office so you can sit down and know that what can you do about those who are offering transport. Myself I cannot talk about it that is upon you people.

**I: You do not have a suggestion?**

PJ08: Suggestion is if you can check and know how much we use for transport. Yeah, it can be of help.

**I: Any other challenge that we can face while implementing Jitegemee?**

PJ07: The challenge that we can face I as number 7, nowadays bar prostitution is being outdated, sometimes a client has called you and you have even spent in =Busia= You know you cannot keep saying you are not around, you are not going to make your fellows happy. Those are the challenges that I can have because for me to come from =Busia= to town it will take time, I might even find that the meeting is over, again next time I will say that am in =Kisii= that will make it not run smooth.

**I: So how can we solve that challenge?**

PJ07: That is what I do not know, because my thoughts might be low, so you can collect others thoughts too.

PJ08: The challenge that she has mentioned, what you can do as an office, you can be sending us messages earlier within three or four days in advance so everyone knows that on a certain day the teachers are coming so I should be prepared in such a way. Yeah

**I: Any other challenge?**

PJ09: I support number 8 in what she has said.

**I: What has she said, and about what?**

PJ09: About sending of messages, you can tell us in advance. Then we are aware, we are ready.

**I: Ooh any other challenge, number 2?**

PJ02: Challenge that people can.

**I: That we can have as Jitegemee during implementation… (*laughing)* has it disappeared?**

PJ02: I lost it

**I: You have lost it, number 5 the challenge that we can have?**

PJ05: Challenge... I do not have.

**I: You do not have any say, okay number 3, any challenge that we can have?**

PJ03: As number 3 I do not see any challenge you can have.

**I: Number 4, challenge that we can have?**

PJ04: Challenge that you can have, as number 4, a challenge that can be there, it is just what number 8 has said, time, let’s say when we are sent messages early even if we were to meet let’s say on Saturday, Saturday even if I was supposed to go somewhere I know that Saturday we are meeting, but if you wait till Saturday that is when you are calling saying that we are meeting today, it will not be possible.

**I: Number 1 challenge that we can have?**

PJ01: Challenges can come in ways like, us being in the *jua kali (*working in industrial areas) you would want to work a little during the day and a little at night and you can be scheduled during the day, so briefing can be just through phone.

**I: Number 6 challenges that we can have?**

PJ06: I do not have a say.

**I: You do not have any challenge? Okay and if you are saving in Jitegemee, how much can you contribute weekly? Without suffering, without feeling that you are suffering but we want you to save every week? number 4**

PJ04: Allow me to ask you a question before I answer you, what do you mean saving?

**I: Jitegemee is about savings, right?**

Chorus response: Yeah.

**I: So we want you to save, to save money**

PJ04: No do you mean I save mine or is there any other that I will save?

**I: No, Jitegemee is to be independent, don’t think too far just think here okay, we are almost. Jitegemee is to be independent meaning you divide what you have and save, so in a week when saving how much can you save?**

PJ04: I can save five hundred shillings.

**I: Five hundred shillings’ number 4, number 9?**

PJ09: I cannot say, because it depends.

**I: If you look at your responsibilities and how you earn, if you can save how much can you save?**

PJ09: I can save three hundred shillings.

**I: Three hundred, number 2?**

PJ02: Four hundred

**I: Four hundred, number 5?**

PJ05: I can save five hundred shillings.

**I: Five hundred, number 3**

PJ03: I as number 3 I save five hundred shillings twice a week.

**I: Five hundred?**

PJ03: Yes

**I: Number 7?**

PJ07: Eight hundred.

**I: Eight hundred, number 8, number 1?**

PJ01: (*laughing*) According to my earnings I can save four hundred per week

**I: Four hundred**

PJ01: Yes

**I: Per week? we are talking of per week.**

PJ01: Yes

**I: Number 6?**

PJ06: I as number 6 I can save three hundred.

**I: Three hundred, number 8?**

PJ08: I can save three hundred depending on the responsibilities.

**I: Let’s say that you have not gotten the money to save that week, you have targets in place just like we have said number 8 three hundred, three hundred in that order, right? We have talked about targets, it is you who knows what you earn and the responsibilities you have, and you have spared that which you have spared, right? if you don’t get, what can you do to get money that you can save?**

PJ06: I as number 6, I can sell something that I own, I can chop vegetables and sell and get something to keep.

**I: For you to meet your target?**

PJ06: Yes

**I: That is number 6, yes number 4?**

PJ04: I as number 4 if it happens that the week did not favor me, it will force me to multiply it in the next week.

**I: What do you multiply?**

PJ04: I multiply that five hundred by two, it depends during the day am somewhere and at night I also go somewhere so it cannot block me from multiplying.

**I: So what do you do in order to multiply is what I want you to tell me, what would you do to multiply in the following week?**

PJ04: Okay what I will do, okay daily work. In the past there is none that passed without getting something, so it will force me that from the daily money, I will be keeping one hundred shillings daily in order to cover up for the target of the last week and for this week I will save as usual.

**I: What is it that you do daily?**

PJ04: What did you find me doing there?

**I: I do not know.**

PJ08: Okay she works in a hotel

**I: What do you do?**

PJ04: I Work in the hotel.

**I: Number 9 what can you do in order to get money to reach your target? If you cannot achieve your target.**

PJ09: Something that I can do… I will just try.

**I: What will you try?**

PJ09: You know you should have a business that you are doing. There is possibility that in that week it did not go well with you, but it cannot be bad every day. So, it can be possible that the next eek you will be successful.

**I: So if you have a business, what kind of business is it?**

PJ09: Cooking chips

**I: She has been told by number 4, yes, number 2?**

PJ02: I as number 2… I work here

**I: Where do you work?**

PJ02: Bar it is a bar stroke hotel. So, we are being paid daily, so I have to save one hundred per day so that in a week I meet the target.

**I: So you reach that your target?**

PJ02: Yes.

**I: Okay number 5**

PJ05: I as number 5 someone can invite me for laundry and my daily target for the day has to be fifty shillings in order for me to achieve my weekly target.

**I: Number 3 what can you do if you have not reached the target?**

PJ03: If am defeated, I do sell charcoal, so with this charcoal I have to try harder to sell it in order to reach my target.

**I: Number 7?**

PJ07: I am a saloonist and in the salon you cannot go a whole week without getting some money, you must get, it is a sure bet.

**I: So you cannot miss your target?**

PJ07: I cannot miss.

**I: Number 1, if you lack money for your target what can you do?**

PJ01: Just the way number 5 has said, even laundry, you can do someone’s laundry and you cannot lack money for your target.

**I: Number 6, what can you do?**

PJ06: I told you I chop vegetables.

R: (*others laughing*) She is the one who started.

**I: Okay, number 8**

PJ08: I can get second hand clothes and sell them.

**I: Okay, I asked you because you are silent. Okay and if someone wants to save where can she save? A place that you trust where is it?**

PJ05: Number 5, I can save in the Mpesa

**I: Number 3?**

PJ04: Number 4, M-shwari.

**I: Number 4 M-shwari, number 3?**

PJ03: I as number 3 I love Mpesa

**I: Mpesa, number 7**

PJ07: I can have it in a locked saving account.

**I: That is a bank?**

PJ07: Yeah

**I: Number 1?**

PJ01: For me I said earlier that the person who is closer to me who can help me quickly without even boarding a vehicle.

**I: Someone you trust?**

PJ01: Yeah that I trust.

**I: Number 8,**

PJ08: You know that Mpesa has a locked account, if you don’t want to spend the amount you can save it there.

**I: Mpesa, number 2?**

PJ02: Mpesa

**I: Mpesa, number 9?**

PJ09: Mpesa,

**I: Mpesa, okay number 4 had already said. Okay, do female sex workers spend more than what they get?**

PJ08: Yeah

**I: Already number 8 has agreed (*others laughing*) number 8**

PJ08: Yes, there are.

**I: Why is it that way?**

PJ08: Because at times sex work does not favor you, and so it will force you to dig dipper, at times it you have to get second hand clothes to walk selling so that to top up on the money needed.

**I: Someone else? Someone whose lifestyle is higher than their income? Is there any?**

R: Yes, there is

**I: Yes**

PJ03: I number 3, I stay a lifestyle that is above my earnings from sex work that is why I have incorporated charcoal business in order to top up.

**I: And why do you stay in a lifestyle that is beyond your earnings?**

PJ03: It depends with the weight of responsibilities, you have school fees, you have people to feed.

**I: Someone else?**

PJ08: Number 8, the reason as to why someone can stay in a lifestyle that is above her earnings, when I started I stayed in a single room house and I have kids and at times these kids are older maybe above fifteen years, this is a child who we cannot share the same single room and am inviting someone here too. It will force to have another room where they can sleep, so this will force my earning to be higher depending on the way I had planned my life this means there is increase in expenses and when the expenses increase and your earnings are low, life has to be difficult.

**I: Number 2, why do they stay in a lifestyle that is above their earnings?**

PJ02: It depends sometimes with the family that you have, sometimes your family is big and the amount you earn is not enough, so it forces you to live a lifestyle that is higher than your income.

**I: A lifestyle that is bigger than her income, number 1 why do they live in a lifestyle that is higher than their income?**

PJ01: I support number 2 and number 3. It is the responsibility; you find that you have a big family but with little income that is why they live in a lifestyle that is higher than our income.

**I: We had seen that number 8 said if she lives a lifestyle that is higher than her income she would get a bale of second hand clothes in order to top up, right, so what else can someone do or that they do to top up the deficit they have?**

PJ04: I as number 4, I can fry french fries or even look for *jua kali* like working for someone at the hotel to help. Those are also there.

**I: That is number 4, we are almost, we are moving on, I can see you are tired.**

PJ01: I number 1, just as number 4 has said, you just be idle, you must be hard working in that even if you get laundry jobs or even hotel, use it to boost that.

**I: Any other thoughts, number 5?**

PJ05: Just the way they have said it.

**I: What have they said?**

PJ05: You can look for a casual job to add to your job.

**I: Number 7 what can they do to fill up the deficit?**

PJ07: In today’s world you must go to work because commodity prices have risen, so if you depend one place you cannot make it.

**I: Do sex workers frequently borrow a lot of money**

PJ07: Maybe if they are members of merry-go-round.

**I: If they are?**

PJ07: If they are members of a merry-go-round that is when you can borrow money, because you cannot just come out of nowhere that you want to borrow money from someone, even if you are in *chama*, even loans but for you to be eligible for loans you have to save with them in order to access loans you cannot just come from nowhere that I have come to borrow money they will not give you, you must save in order to access loans.

**I: That is number 7, you borrow from merry-go-rounds or *chama*?**

PJ07: Yes

**I: Anywhere else they can borrow money?**

PJ04: I as number 4. Previously we had talked about what causes problems if you do not save, it is not a must that you borrow money from the merry-go-rounds. I differ with number 7, I can be friends with number 9, I can approach her and tell her that I have got a certain problem and would request a certain amount from you, you can borrow from a friend but again with that a problem will also arise for sure borrowing is easy and repaying is a problem.

**I: You can borrow from a friend?**

PJ04: You can borrow from a friend.

**I: Anywhere else where they can borrow money? Number 2?**

PJ02: They can borrow money from…they can borrow money if they are members of a group like *chama.*

**I: Apart from *chama* and friends where else can they borrow money from? number 1, apart from *chama* and friends where else can they borrow money from?**

PJ08: If you have accounts with banks, number 8, if you have accounts with banks you can borrow money.

**I: What do they borrow money for? Why are they borrowing money, what for?**

PJ03: I as number 3, you can borrow money in accordance to a business thought that has cropped up apart from sex work, at times you have noticed you are tired so you think of getting a loan to start a business that is much better to support me, that is why they borrow money.

**I: Reason as to why they borrow money, number 9 again?**

PJ09: Reason why they borrow money. You can have a challenge and there is nowhere you can get the money so it will force you to just borrow the debt.

**I: Number 6 why are they borrowing money, and for what purpose?**

PJ06: To run a certain business.

**I: And what do they usually do in order to pay it back? If you borrow money hat can you do to be able to pay it back?**

PJ05: I can run a small business so I can find means of repaying that debt.

**I: Number 1?**

PJ01: It will force you to be hardworking for you to pay this debt. That is why.

**I: Number 3?**

PJ03: I as number 3, you will borrow it to do a serious business, after paying back in full you will borrow again to boost and continue with the business.

**I: And are there things that they do to boost their income while still in sex work, right? They perform sex work but they want that income to increase, let’s say you usually get five thousand you can now get ten thousand, what are the things they can do number 4?**

PJ04: What is it that they can do?

**I: The things they can do to boost their income.**

PJ04: Just the way we have said

**I: What have you said?**

PJ04: You be hardworking you must get some casual work.

**I: That one was to repay debts.**

PJ08: Number 8, what you can do, for example I can repay weekly, let’s say I borrowed ten thousand, for my account to be active I will pay in bits daily.

**I: That one you have paid debts.**

PJ08: I pay the loan so that my loan limit will increase.

**I: For your income to increase.**

PJ08: My income to increase?

**I: Let’s say you are working, and we are talking about sex work, you get five thousand and you want this five thousand to be ten thousand. What will you do so that this income will increase? I am writing it down as income not a debt, something that I earn that will reach that amount. What are the things that I will do?**

PJ08: Even if I go for sex work I must have a small business to add up so as to increase my income.

**I: You will add with a small business. Something else they can do for the income to increase?**

PJ09: Eeh (*others giggling and some whispering*)

PJ07: Number 7, you do not become someone with a sweet tooth, you cannot save to reach that, so you have to reduce the consumables too. Yeah

**I: To increase your income, what can you do?**

PJ09: You just have to be hardworking

**NT: Hardworking towards what?**

PJ09: You will have to double your effort in the business that you will do.

**I: What type of business?**

PJ09: The business can be even chopping of vegetables, frying french fries or even roasting maize.

**I: Number 2 what can they do to increase their income?**

PJ02: Just hardworking

**I: Hardworking in what way? I do not want to hear hardworking. What can you do to increase your income?**

**NT: Exact thing.**

**I: Say what you can do.**

PJ02: What I can do is just my daily work.

**I: What is your daily work?**

PJ02: Hotel work.

**I: What do you do in a hotel?**

PJ02: I serve customers.

**I: You serve customers, number 5 what can you do to increase income?**

PJ05: I can just maintain sex work to increase my income.

**I: What do you do when you go for sex work to increase your income? You do go for sex work daily.**

PJ05: Yes

**I: What will be different so that the income you get daily can increase, what will you do that will be different from what you do daily?**

PJ05: I can add a business

**I: You said going for sex work. What are you going to do that is different from what you usually do every day?**

PJ08: Number 8, if I get one thousand and used to spend eight hundred, it will force me to save and only spend five hundred so will use three hundred to increase my income.

**I: So you will reduce the expenses?**

PJ08: Yeah, I will reduce the expenses.

PJ01: For me I can even walk door to door doing laundry to increase my income.

**I: To add to your income, okay, and are there a times when women sex workers realize that there are no clients, or are the clients available every day? Are there times when there are no clients?**

PJ08: Number 8, yeah there are times when clients are not there. You go to work and return empty. There are times you go there and they just look at you and you come back empty handed. So, there are times they are not there.

**I: So they can just look at you?**

PJ08: Maybe they also don’t have money, so they just look at you.

**I: That is number 8’s opinion.**

PJ06: I as number 6, at times you might go and the environment is gloomy and you are told that it’s not going to be as usual.

**I: How will you know it is gloomy?**

PJ06: When you go there you will find it is full and the next time you go you will find empty seats

**I: Number 4, how will you know that there are no clients?**

PJ04: I as number 4, if you want to know that work is bad, no clients, signs of rains are the clouds even if you are running a certain business if you check the mood you will automatically know that today only God knows. So, that is why even if we go for sex work just the environment will make you detect that today we shall see what we will get out of it.

**I: When sex workers borrow money, roughly how much can they borrow? Number 8**

PJ08: Debt?

**I: Yeah, that you will pay that a sex worker can borrow?**

PJ08: It starts from thirty thousand backwards for a start.

**I: Thirty backwards, yes number 6?**

PJ06: It can be twenty-five

**I: It can be twenty-five, number 4**

**NT: Is it twenty-five hundred, or twenty-five thousand?**

PJ06: Twenty-five thousand

**I: Twenty-five thousand, number 4?**

PJ04: It can just be twenty-five.

**I: Twenty-five thousand, number 5**

PJ05: It can be twenty.

**I: Twenty thousand, number 9?**

PJ09: It can be fifteen

**I: Fifteen hundred or fifteen thousand?**

PJ09: Fifteen thousand.

**I: Fifteen thousand, number 1?**

PJ01: I as number 1, I step back because it depends with what am going to do with it first, then secondly it would be nice if you borrow ten.

**I: Ten**

PJ01: Yes

**I: Ten thousand, number 3?**

PJ03: Is that a debt for loans?

**I: That which you borrowed.**

PJ03: Is it what you borrow for business?

**I: I don’t know what you will do with it.**

**NT: That which you usually borrow.**

PJ03: Thirty

**I: Thirty thousand?**

PJ03: Yes

**I: Number 2?**

PJ02: Fifteen thousand if there is something am going to use it for.

**I: Number 7?**

PJ07: Ten

**I: Ten thousand. Okay and do female sex workers ever think about stopping sex work?**

PJ07: Yes

**I: Yes, number 7?**

PJ07: Reason as to why that thought comes, I as number 7, is because of the challenges that we go through. At times you go looking for money and clients dodge you and you do not have money. Some beat you and that is why when relaxing at home you think of even meeting someone who you can get married to and stop this job because that is what causes me problems.

**I: So a challenge like that one?**

PJ07: Yes

PJ08: Number 8, challenges are there. The reason why people think to stop, you find that mostly when the college students have closed school, at our age we get a lot of challenges because you find out that they are looking for the younger ones. So you just see that one day one time I will just drop from here. Those are the challenges that at times we face here.

**I: So the clients become?**

PJ08: They change

**I: They become selective?**

PJ08: Yes, they change. They tell you that we have been having you here every day. So you find that the college students come, those that have completed school come there and so when you are with them you are just scrap and they pick the other ones.

**I: Okay, something else? Any other reason why they think of stopping? Number 2?**

PJ02: The reason as to why they think of stopping. You can just work with a target because this sex work is tiresome. You can get tired of it, you can decide that you might do it for just a month, start your business and then stop.

**I: You are tired of sex work?**

PJ02: Yeah, you can get tired.

**I: You can get tired. Anything else that makes someone think of stopping? Number 3?**

PJ03: I as number 3, just the way number 8 has said

**I: What has she said, remind me.**

PJ03: When students close school, when school closes we have challenges

**I: Okay and is it something that is said in general or is it what someone thinks individually? stopping sex work.**

PJ05: It is something that a person thinks individually.

**I: You do not share it with others?**

PJ05: Yes

**I: Why?**

PJ05: You just see how your body looks and the way the world is evolving.

**NT: That is number 5**

PJ07: Yeah, most it is always cold where we go in town, you can go thrice and not get any work till you decide to stop and get someone who we can suffer together at home. That is my opinion as number 7.

PJ08: Number 8, you know, when doing sex work you must have a friend, and sometimes your friend is your age mate. So when the sex work has not favored you, two or three times you come and sit down and consult yourself, that until when will we be doing such a job? It is high time that we stop. We sit down and consult ourselves now that the schools have closed it is not going to be fair to us. When schools close it is very hard on us. At times we talk on our own we consult one another.

**I: So they are topics that you can talk about?**

PJ08: Yeah they are topics we can discuss.

**I: What is it that happens that can trigger such topics?**

PJ08: The reason that triggers that topic is the way we do not earn. You can even go to work for a whole week but there is no income, you can also find that the potential client that you always have, looks at you in a way you cannot move next to him. So you are left pending because he is having another one. So those are the challenges we face.

**I: Any other challenge that make someone to want to stop sex work? Are there any plans that someone can put in place to do before stopping sex work? number 4**

PJ04: I can say someone can have plans.

**I: Like which ones?**

PJ04: One, she has a child and is a single parent assuming both roles, I can try and immediately my child does his fourth form exams I stop sex work and I shall have my business running.

**I: Personally, is there something you would wish to do so that by the time you stop sex work you shall have finished doing it?**

PJ04: Just for the children.

**I: For the children. Number 9, plans that you have that you would want to finish by the time you stop sex work?**

PJ09: Something that I would want to do, I would want that by the time am stopping this work, I shall have saved some money to start a business that will support my life in future.

**I: Number 2?**

PJ02: I as number 2, my plans are that by the time am stopping sex work, I will have a big business and employ those who will be assisting me.

**I: Number 5**

PJ05: After getting something I will go and rest at home.

**I: What do you mean something?**

PJ05: Something like a job

**I: What kind of job?**

PJ05: Where I can employ someone.

**I: Who does what?**

PJ05: Who can help me even at home.

**I: Okay, number 3?**

PJ03: The way I have planned, even at now am getting tired, so the target is that if I can get a lump sum of money at once apart from the ones am saving, I can open a second hand cloth line, then I rest while selling next to town because I feel my body is getting tired.

**I: Do you have a plan of getting the money at once? Do you have a place to get the money at once?**

PJ03: I was wishing that if I could get it at once because the one am saving, every now and then school fees is needed or the child is sent home, so it keeps going down and I wish that I could find money at once. Anywhere I could find it at once, I will open a business.

**I: Do you have a place to get it from?**

PJ03: I don’t know. I don’t have anywhere; I still don’t know.

**I: You have not gotten?**

PJ03: Yes.

**I: Okay, number 7, plans?**

PJ07: I pray to God that if I leave this place, I stop sex work because we are also risking our lives. At times someone has taken you to his house and you do not know how he is. Some of us are hurt even in those houses. So if I can get my own money I enjoy my sweat rather than sex work.

**I: So, what would you wish to do and complete by the time you stop sex work?**

PJ07: I am a salonist so I pray to God if I can get a sponsor in my sex work activities if it is His wish I will be grateful.

**I: And if sponsor in not available?**

PJ07: Then I will work hard to get.

**I: Okay, number 1?**

PJ01: I number 1, in my opinion if I stop sex work I get an older man to build me a home, that is what I can say.

**I: Before leaving it, before leaving sex work?**

PJ01: When I retire from sex work I get a place to rest myself with Ajola’s grandchildren.

**I: Okay, number 6? Something you would like to do before you stop sex work?**

PJ06: Before leaving sex work?

**I: What to do so that by the time you stop sex work you have finished doing it?**

PJ06: Let it find me with even some money in my phone which can help me boost my business of chopping vegetables that I do.

**I: Starting a business?**

PJ06: Yes.

**I: Number 8?**

PJ08: Number 8, I have two things, the first one, is to have a stable job for example the second hand business where I find a good stock to sell of the second hand clothes. Another thing, if I can buy a land, so I have my personal land as I sell the bales and checks other things that I can do.

**I: Okay, we are almost number 1 don’t complain so much, okay?**

PJ01: Okay

**I: We are almost, and what types of jobs do female sex workers usually do after leaving sex work? What types of jobs can someone do?**

PJ03: I number 3, I have seen that female sex workers always like selling second hand clothes, they are really many there and salon work.

**I: Is that after stopping the sex work business?**

PJ03: Yes, after stopping.

**I: Okay, any other jobs that someone does?**

PJ01: Number 1, most of them like doing business just as number 3 has said.

**I: Which types of business apart from selling second hand clothes?**

PJ01: They are businesses like chopping vegetables, opening a hotel, those are the kind of business they like starting.

PJ08: Number 8, mostly if someone has good money she can open wine and spirit outlets and secondly, a hotel.

**I: Hotel?**

PJ08: Yes, that is combined with a bar.

**I: Number 2**

PJ02: Mostly someone opens these beauty and cosmetic shops.

**I: Beauty and cosmetics, okay and do you know of any sex workers who have stopped sex work in the past five to ten years? That has stopped? Number 8?**

PJ08: I know of one.

**I: Why did she stop?**

PJ08: Depending on her age and secondly, because the children she has are now grown up. That is why she stopped.

**I: Anyone else who knows someone who has stopped?**

PJ07: As number 7, I have also seen someone who stopped, when her former husband took her back to where she used to stay.

**I: She got back in to marriage?**

PJ07: Yes, but she went back when a bit older, so she has currently opened a baby care where she takes care of fellow sex workers children when they are away for work.

**I: Okay, number 4?**

PJ04: (*whispering*)

**I: Say it louder I cannot get you.**

PJ01: She is pointing at number 7.

**I: Stop pointing at number 7, say the words.**

PJ04: Number 7 has said my point.

**I: What was your point?**

PJ04: The one she has said.

**I: What has she said?**

PJ04: She has seen someone who stopped because the former husband decided to reconcile with her.

**I: And is there any that left and returned to sex work again? Is there… number 4, who stopped and returned to sex work again?**

PJ04: I as number 4, there is one that had stopped and returned again.

**I: Returned again, why did she return?**

PJ04: The challenges in marriage, she got married while doing sex work then she met a man who married her at once. You know when we stay here for long we use a certain slogan that we are not to be planned for. So she went there with the same mentality and she had to come back.

**I: She came back. Yes, number 5, one that you know who stopped but returned? If it has happened and you know.**

PJ05: I as number 5, I don’t know any.

**I: You don’t know any?**

PJ08: Number 8, there is one that I know who stopped but returned. Someone took her from where she was and married her. According to people, she did not prepare and when she got to the house she became a house wife. So most of the things she felt were challenging on her side. Like when she asked for money the man would say he does not have. So she decided that, that life she had alone was much better than the current life. So, she decided knowing that this life was much better just because she was not prepared, if she were prepared she would have found a job to do but because she was not prepared she returned.

**I: Number 1, anyone you know who came back? If there is any?**

PJ01: I know no one.

**I: There is no one you know? Number 2?**

PJ02: There is none that I know.

**I: You know none, number 9?**

PJ09: None

**I: None, number 3?**

PJ03: I know none.

**I: Okay, and are there any challenges that the ones who stopped met? Or that someone can encounter after they leave sex work?**

PJ07: Yes, I as number 7, you were used to getting money every day that is the first challenge, the second one, you were used to beautiful things and now when you stay there you are under someone’s money. Even if you tell him that you want to make your hair he will tell you that he still does not have money. So, if you sit alone you will regret saying if you were alone you would have done that by yourself that is why the ones who left come back.

**I: Any other challenge?**

PJ04: Another challenge, I as number 4, rules must be followed. When you are used to rule yourself and on the other side you are controlled.

**I: So, it is tough.**

PJ04: It is tough.

**I: Any other challenge that they meet after stopping?**

PJ03: I as number 3, when you left home to go somewhere, you keep looking at the watch that when it gets to a certain time I will encounter a fight. You were used to going home at your own time that is the challenge that is there.

**I: Okay, is it just age alone that leads to stopping sex work or are there other different reasons?**

PJ08: Number 8, age alone cannot make one stop sex work. But at times someone can just decide depending on the challenges encountered here. You will just decide for how long will I be doing this job, you know by the time you are deciding to stop you have already looked at your future but when I want to continue up to the age of seventy, one can look to the future and decide that even if am doing this job it should have a limit. You just don’t do it for the sake of it, but to build a place. So, that is why someone can stop, because of age I have done it for a long period of time but I cannot even see its benefit, let me find something even if it’s being independent and get something to do. That is why some stop.

**I: Anyone else with a different thought?**

PJ04: Number 4. Age alone cannot force you to stop sex work. Reason being, as I am, I am still young and can still go back to school up to latter years, but when you go home they gossip about you asking why has so and so’s daughter not getting married? Others daughters are getting married, and bringing us their husbands home but what about her. So you know such things make you feel embarrassed and you think that let me get someone to marry me.

**I: So it can make you to stop?**

PJ04: Yes, it is a challenge.

**I: Someone else?**

PJ05: I as number 5, someone can stop because she got raped which can make her lose feelings for the work.

**I: Number 1**

PJ01: I as number 1, you can stop it depending on the situation, such as you were once married and when you go to work you meet with your own child and at the same time you have met your child with someone that you had had sex with.

**I: Being together with her?**

PJ01: That is the reason some stop.

**I: Number 2?**

PJ02: Age cannot make you stop sex work. If you work for a long time it is true your body will get weary, then you just decide to stop. So, it is not just age alone.

**I: Number 9?**

PJ09: Age alone cannot make one stop sex work. You can still be young your body radiating heat but you can meet a situation in sex work that can make you lose hope.

**I: Number 6**

PJ06: I as number 6, the reason why you can decide to stop is you might encounter something unusual.

**I: Like what?**

PJ06: Like having sex with someone then they leave without paying you, you get disappointed and leave.

**I: Number 3?**

PJ03: I as number 3, the reason why I can stop is looking at my age, you just feel that you should stop and go do other things at home, have your own home to live in. When you realize that age is catching up with you.

**I: Okay, I say thank you. Is there any question?**

PJ02: Only those that are tired are many (*others laughing*) and hunger is killing us too.

PJ08: There is a question you said you will answer us, when I asked about interest and you said you will tackle that too. It was about getting interest when someone is saving money, you said you will reach there but you did not reply.

**I: About Jitegemee there is no interest. You will save money by yourself, the amount you are comfortable with even if it is fifty shillings, even if it is daily, weekly or even monthly, it is you who decides. There are no loans, no taking loans like in *chamas,* the money is completely yours.**

PJ08: The amount you saved is what you get?

**I: The amount you save is what you get. It is what you have, where you saved it is where you will get it as you saved it.**

PJ08: Okay

**I: Anything else? Thank you.**

**END OF INTERVIEW**
