# Supplementary material for: Eat a little and save a little: A qualitative exploration of acceptability of a potential savings intervention to reduce HIV risk among female sex workers in Western Kenya
Source: PLoS One. 2024 Dec 19;19(12):e0310540. doi: 10.1371/journal.pone.0310540 (PMC11658496; doi:10.1371/journal.pone.0310540)
Supplement: S1 File — (ZIP) [file pone.0310540.s001.zip › Jitegemee Transcripts and Dissemination Notes for Journal/FGD K.docx]

**DATE OF INTERVIEW: 27/APRIL/2022**

**MODERATOR: OLIVIA OKUMU**

**NOTE TAKER: NANCY OUNDA**

**FGD TYPE: FGD K**

**CATEGORY: ABOVE 30, RURAL**

Transcript:

**I: This is FGD K done in =Pipeline=. The date today is 27/APR/2022. The group is above 30. Now as we start, I would like to ask you that, as I have explained to you about Jitegemee in a simple way, what comes to your minds? Number 3**

PK03: As number 3, Jitegemee has, you have taught me something good about savings, I should not just be a sex worker, such that I do that work and take all the money, and I can use it without having any that I can save, so that one day if I decide, like me, I wish that one day five years coming (she meant five years to come), I should rest, because this thing (means sex) can destroy someone’s body, I should rest and sometimes relax my mind when I have something of my own which I can depend on, even if it is rentals, even if it is a business, then I can now do it to give me support, that’s my opinion number 3.

**I: Someone else, what comes to your mind after hearing things about Jitegemee?**

PK01: I as number 1, I feel that it is a good teaching because, maybe I was doing it and was feeling that it was something bad, but I have found out that it is something that can bring a development or something good in future, so I can do it knowing that, when I have some hope that I can do something good when I save, with this way of earning income that is this sex work, so I have seen that it is not a bad teaching, it is a good teaching, and I thank you for bringing to us such kind of teaching because, we were doing it without knowing, but right now we know wherever we are moving with it.

**I: Okay, someone else that wants to add, what comes to your mind when you hear something about Jitegemee that I have explained? (Movements, people talking in the back ground). Number 6 is moving as if she has an opinion.**

PK06: I as number 6, I am saying this, I can see that this job is a good one, because myself, I used to despise those who were doing this job, when we went to the clubs, we saw them parading there, and we used to despise them, so later on, I came to realize and said, that this job that these people are doing, it is a good job, it is even better that going to work at a hotel or doing any other job that is different from this one. So you can find someone that you go with (have sex with) and you will get a lot of money in one day or even those other times. So when I did this job, I found a way of helping myself with my children, how I can pay rent, how I can do what, I found out that this job was good, better than other jobs, only those.

**I: And about Jitegemee, what comes to your mind?**

PK06: My thought, I feel like I can continue with it, so I can save a little, and I use a little, so that I can look at how the future will be.

**I: Someone else?**

PK04: As number 4, when I started this job I with little interest, because you may find somebody, he goes with you (have sex), he tells you that he wants you to give him one shot (one round of sex), he wants to give you two hundred (shillings), if you tell him one thousand he feels that it’s difficult for him, so you say, today the field was bad (business is not good), let me just go, so when you go and come back this way you find when God has opened for you (done you a favor), you find someone that is serious, and he tells you, hi madam, when you go with him he tells you that, I want to be with you till morning, and you agree with him that, how much, three thousand, I agree, I sit, when morning comes I come back to the house, I take a little and keep it, I see how I balance this money, school fee, how these people are eating, how I also pay rent. But with my hope in the five years to come, I pray that if God allows then I will take a break first, take a rest first then I will one day start it again. [Cross talk]

**I: So I want you to**

PK06: I want to support number 4.

**I: That is number 6.**

PK06: I can say that like she has said, for the payment, I also went somewhere and after going somewhere then someone told me, before I knew about the shots (sex rounds), then I went somewhere and asked, what is a shot, so he said, if you don’t know a shot then wait for me first, so when he came back I told him that, I am used to, I can go with someone (have sex) and he gives me a large amount of money and not a small amount. So the meaning of shot is that you go with him (sleep with him) little by little (bit by bit), and he is going to give you a small amount of money. So I said that when you go with me you have sex with me till morning, three thousand shillings or two thousand if I reduce it for you, so that man left me there abruptly and went, and he said that is not the meaning of a shot for this place. (Laughter)

**I: Okay, thank you, so the question that I am asking is this one, I have explained Jitegemee, Jitegemee is about saving, you save the money that you have made. So this is what I was requesting you to tell me about, that, what comes to your mind depending on what I have explained about Jitegemee, number 8?**

PK08: As number 8, the teaching that I have got here, (someone coughs) if I get someone and he gives me a hundred shillings, I am supposed to use fifty and keep fifty. Even if he gives me five hundred, I am supposed to keep four hundred and use even a hundred, depending on how tomorrow will be.

**I: Mmh, someone else?**

PK02: As number 1

**I: Number 2**

PK02: Number 2 as I support number 8, yes, it is a business, and it is a good thing that has been helping us, and it has been helping me, but you must know what is coming in the future, because you have your family, and it is a kind of a job that you don’t even know what you will encounter, but a business is a business such that even when we were starting it or when I was starting it, you must find someone who is talking badly about you, someone who is talking nicely about you, all hustler are just hustle. So, what I was requesting is that even if someone is doing it the way we are doing it or if I am doing it according to my thoughts, because my thoughts won’t be the same as another person’s thoughts, a way of saving was good, because even if sometimes you come back and you are successful, because these things are different, the hearts of men are different in this town, and even in the beaches where we go to, they are different. There’s a person that feels that you have not come to do that thing because you like it, but you have come because you are lacking. He will go with you (have sex) and you will finish with him and he will give you something good. There’s a person who feels like, that is a business she is doing, whichever thing I will give her, it is something which is negotiable. So what I was requesting is, it is good to save, you use five and you save five to use it in the future.

**I: Is there someone else that wants to add, number 3?**

PK03: If I add, then I can continue encouraging, I can continue supporting sex worker’s community like me that truly the age is also catching up, there’s a certain age that I can attain such that even if you have to be twisted (sex) with even three men per a day, you just feel as if it’s now difficult. Sometimes I have reached menopaused (menopause) such that I cannot do that job, such that it is a must that I be at home, so this Jitegemee, I feel that it has enlightened me because the money that I was getting, I was getting money that that I felt that, I will still get them tomorrow, there’s nothing that I purchased, this is my thing and I didn’t purchase anything, so I just use money carelessly such that I don’t even know that tomorrow may come when maybe I am sick, I have children and there’s no way they can eat, so when I save, I feel that it will help a lot, so on that I have praised Jitegemeo (Jitegemee) for bringing us such a teaching, we didn’t have the issue of saving on our minds before, we just knew that in this thing (sex work) we were just going, when tomorrow comes I will still earn, just that.

**I: Number 9 wanted to talk.**

PK09: Me too as, me as number 9, I want support number 3 (birds chirping) depending on the teachings that we have had today, because me too, when I started this job, I used to go, and when I get two hundred, then I come and use the whole of it, because I know that tomorrow morning, tomorrow evening, I will go back and I will get another one, and depending on the teaching that you have brought, it has now made me know, that I am supposed to use a little and keep a little.

**I: Does number 5 have any concern?**

PK05: No, I don’t have.

**I: You don’t have a concern, number 10, do you have a concern? What comes to your mind about Jitegemee?**

PK010: For me, what comes to my mind is only how we can learn how to save. (Birds chirping)

**I: Okay, thank you. As we continue, this money that we get as sex workers, you go in the evening, you get the money right?**

P: Yes.

**I: When you come back, what are the things that you do with this money every day, things that, the daily activities, what are they, not for the week, the daily activities?**

PK03: I must eat

**I: That is number 3.**

PK03: I must bath

**I: Just give me a list that, I number 3, every day, this is what I do, I use this amount of money, and I buy one, two, three, four, and we move that way. (Birds chirping)**

PK03: Okay, for me number 3, after I come from my hard work, it is a must that treat myself, I must first bath myself and be clean and become smart, after bathing and I am clean, I cook tea because obviously those things are always done in the dark at night, after bathing I make my thick tea and drink it, that’s part of eating, my children go to school, they are children that go to school a bit far, I must pay for them a motorbike when they are going to school because they are children that are still a bit young, behind, so it helps me small ways more so on food, I can use it a lot.

**I: So in a day, how much money do you spend daily?**

PK03: Daily, in the morning, if I use a little money, it now depends on how the field was (sex work job), you know now you work with budget, you know that you must budget according to the money that you have in cash, sometimes I have two hundred, it will force me to now reduce my budget, but when the job is good, then I can even use five hundred per day.

**I: On food?**

PK03: Just on food.

**I: And on school?**

PK03: For school, fare I can pay, I pay fare weekly

**I: Okay that one you can say on the part of weekly. Daily, someone talked here, number, it was** number 6 or number?

PK06: 6

**I: Number 6, (someone clears her throat) in a day, after you have come from work, the money that you always use, how much can you say it is, that which you have to put separately, which you know is enough for a day, (birds chirping)?**

PK06: After I have come from work, then I can, let’s say that I have earned one thousand, God has blessed me with one thousand, and when I come with it, I make sure that the children have gone to school (birds chirping), even now that schools are closed, so I make sure that they eat (birds chirping), and they have bathed and they are clean.

**I: Mmh**

PK06: They are neat, and they have worn good clothes. The house that we have rented, also how we can pay rent, and also the money, I save put a little for savings such that in case there is sickness or anything that may come as an emergency then I can use it.

**I: Okay, so averagely in a day, how much money do you use?**

PK06: In a day I can use five hundred.

**I: Five hundred?**

PK06: Yes

**I: Mmh, number 9?**

PK09: I as number 9, when I come from work in the morning, for me let me not lie to you that I always take tea my fellows, because I always use these bitter things (alcohol) (laughter) I always take them. So when I come from work, I like ugali with meat. When I come back a light the gas and relax my body because you the body cannot be played with, the work that I went to do there is not an easy work, it’s a difficult work, so I must look for some meat, cook ugali, and I must also check how my children are doing, and mostly, I always like to use what my children have told me that they want, because I feel that I am going to work, and wherever I go for work, sometimes you may find someone, he has took you to eat, you have first eaten and then later you have gone to the room, and you didn’t leave the children well, so I always use what the child has said, when he tells me that, mom, today we want ugali and meat, that meat is what we start eating in the morning, is when tea will follow some other time, on my side.

**I: So on that, how much money do you use averagely?**

PK09: In a day, on my side I always use six hundred shillings the whole day.

**I: Six hundred?**

PK09: Yes, from morning to evening, so that is up to tomorrow again.

**I: Okay, someone else?**

PK01: For me number 1 as I support what number 9 has said, the body does not have a spare part, after you have gone and used it and you have come back then you must treat yourself. I have a child and he is at our home, so I must pay rent, and my mom has also put it that every week I must send something small that helps my child over there because you cannot just abandon a child, and he is also going to school. So after I have found some that I am using here in the house, that I use to relax my body, you are also applying oil, you are also making your body nicely so that you will find another customer, because if you are unkempt then when you reach there, because the field people can leave you (customers), that nowadays you don’t have market.

**I: Okay.**

PK01: So after you have done that, for me roughly I can use five hundred.

**I: This five hundred is food or?**

PK01: Food and I also save a bit that I can use on rent because I have put that I save even a hundred shillings, I put it in a certain pot such that when it reaches the time for rent then I remove it and pay with it.

**I: Okay, and what are the things you buy weekly?**

PK01: The things that I can buy weekly are food, and I also send to my child.

**I: Okay, so daily, it seems like a lot of money goes to the food.**

P: Yes.

**I: And as sex workers there are so many things we buy.**

P: Yes.

**I: That, I haven’t heard them in the list.**

P: Yes.

**I: So, when do you buy those, what are the things that are bought weekly, something that you buy maybe once a week, number 5?**

PK05: As number 5, I can, I always buy water, the water that I use in the house I usually buy daily, sometimes I always buy, these our CDs (condoms) I always have them.

**I: Every day? (Cross talk)**

PK05: We use them at the field, yes (background noise). Sometimes I had in the house and I have forgotten to carry it, it forces me to buy it every day.

**I: Okay.**

PK05: Yes. I also have a child at school, he always eats lunch at school, I give him the lunch money daily.

**I: Mmh, so if you add all the money in a day then how much can you use**  ?

PK05: In a day I can use even, if much then six hundred.

**I: Six hundred?**

PK05: Yes. If it covers all those things including food and sometimes I have even paid my electricity token.

**I: Okay.**

PK05: Yes.

**I: And the things that we buy every week, are they there that you know that if I buy this, it lasts** **me a whole week?**

P: Yes.

**I: What kinds of things are they?**

PK06: Sugar, you may buy sugar, flour, you can buy, soap.

**I: So I want to request that when you are talking, (laughter) then you say that, I number 6, I am saying this, okay?**

P: Yes.

**I: So number 6 has said sugar, and what else, what else do you buy that takes you a whole week?**

PK06: A week I can buy sugar, I can buy flour, I can buy, when the price for milk was fair, I buy milk.

**I: How much does it cost you averagely?**

PK06: Those?

**I: Yes.**

PK06: In the morning, three hundred (cross talk)

**P: In a week**

**I: In a week?**

PK06: In a week, five hundred, no not in a week (laughter)

**I: Do that calculation again then you will tell us later (laughter), number 8.**

PK08: As number 8, I can say that I use two thousand weekly.

**I: Two thousand, in buying what?**

PK08: Do the house shopping, food stuffs, my body oil, I also buy shopping that I take to my mother, for my child.

**I: So all those things are two thousand?**

PK08: Yes

**I: In a week?**

PK08: Yes

**I: Okay. So you are going to add it to the five hundred that you were saying in a day or you don’t do calculations in a day?**

PK08: I always spend three hundred in a day.

**I: Three hundred because in a week, you do shopping for the whole week?**

PK08: Yes

**I: Okay, number 9 you have another point?**

PK09: If I also add on what number 8 has said, for me in a week, I do shopping worth three thousand, because I am here with my people [ children], in a week I do shopping worth three thousand that includes milk, sugar, soap and my body oils that I apply.

**I: So you buy oils weekly?**

PK09: There are oils that I can buy weekly, and those that I can buy in a month.

I: Like which are the ones for a week?

PK09: Now (birds chirping), let’s say that, let’s pretend that, I always use Diprosone, so Diprosone is a certain tube, it’s a certain small cream that looks like this small Colgate, you cannot use it for a month, which means that you must buy it after one week, so you buy it and use it.

**I: Okay, so in a week (birds chirping), three thousand, that is on top of the five hundred you were saying per, how much money did you say per day?**

PK09: Per day I use six hundred shillings.

**I: Now six hundred is on top of, after you do your shopping worth three thousand, you will still use six hundred?**

PK09: I will still use six hundred per day.

**I: Okay, alright, number 3?**

PK03: If I may add there on weekly, what is putting me into a loss very much that I may say, is that I have children, I must do shopping for the whole week, different from the five hundred per day, per week I may go and do shopping worth three K (three thousand), that includes my bathing soap, laundry soap, Omo, small things, sugar, things for the house including flour, and there’s something here as KYJ, KYJ, I don’t know KY Jelly (lubricant), the one for applying down there, [ vagina] because when you are truly a sex worker, there’s a time when someone doesn’t even have the urge, if you are a woman when you are not producing the fluids then even a man are not being produced then even a male person won’t go there, because you meet different men, you have met this man right now, he wants you and he is ready, but for you, you have come from there such that you don’t even have the urge, so this KY Jelly also puts me into losses because I must buy it every week such that when I will be going there, I must use it a little bit there so that the place doesn’t become dry and so that someone doesn’t put for me (birds chirping)

**I: Saliva**

PK03: Saliva on my private (private part). So that also I budget for in that my three thousand.

**I: Okay**

PK03: It is being bought at the pharmacy

**I: Someone else, number 4 has a concern. Weekly.**

PK04: As number 4, if I emphasize on what number 9, has said, I do a shopping for two thousand five hundred weekly, and I buy water worth one hundred and fifty, (birds chirping) I buy small things for the house like soap, the oil that the children use, rice, things like sugar, those are the shopping I buy, that lasts for one week. Then I look again that, the time I always leave this place, how much fare do I use in a week, I always calculate that too. Then I look at what I remain with, for saving, and the ones I am keeping, even the ones I can send home to my mother on that weekly, I must send her some sugar after every week.

**I: So if you calculate all those things, how much money can they be, if you count the ones you send to your mother, for the fare, for the shopping?**

PK04: For fare I always use seven hundred in a week.

**I: Seven hundred, shopping two thousand five hundred?**

PK04: Yes, that is on my side if I don’t put my mother’s.

I: Mmh, for your mom is also there?

PK04: Yes

**I: That can be up to how much?**

PK04: One thousand

**I: One thousand?**

PK04: Yes

**I: Okay, and the ones we buy monthly, or the weekly ones have covered the monthly expenses?**

PK03: These weekly takes, as number 3, according to me, the weekly covers even the monthly, because monthly, I only know of one thing that always forces me to deal with monthly like the house. You will not pay someone’s house weekly, it will force you to keep this money once, even if it’s a house worth three thousand, I live in a house worth three K (three thousand), and I pay it per month, on the tenth you give the money to someone so [ landlord], that is why I squeeze my budget so that it may be weekly, so that when it reaches monthly then I give the money to the owner of the house, when I also have something that I can eat.

**I: Okay, someone else monthly, number 2, no number 10?**

PK010: For me what I use monthly that I feel can put me into losses so much is just school fees and rent, it’s the difficulty that I always get, monthly.

**I: Okay, so how much is rent?**

PK010: For rent I pay four thousand five hundred.

**I: Do you pay it once or in bits?**

PK010: I pay it once in a month.

**I: Okay, and school fees?**

PK010: On school fee I pay one thousand five hundred every month.

**I: In every month?**

PK010: Yes

**I: And I don’t hear you telling me the time when you buy make up, or you don’t use them? On your lists, (laughter) I have not heard it being mentioned on the weekly list, only KY Jelly has been mentioned, and also body oil that number 9 told me. So these other ones, do they come to you or you are the ones buying them [ they don’t buy or] (laughter)?**

P: For me if I support number 1 (birds chirping), (laughter), weekly, when you come from your work, truly, you must dress well there, the body must be kept well. Right now, life is expensive such that’ a panty, one panty is one fifty, sometimes I say, I pass in the morning, I see I stall where it is hanged and I say, today let me take this one, so that tomorrow even when I will be going back to the field (job), then when I remove it, then it can bring another customer, it brings another customer, so things like pants, you must buy them in a week, you can bump into them and take them. Weekly, you also have a child in school and sometimes he might come and tell you, mommy, my polish is finished, you go and buy it for him, sometimes you have seen your shoes, because us like this our job that we are doing, you must match, you want to match your shoes there that matches your cloth, you were coming and you say, let me take this pant from this shop (birds chirping), let me also take these slippers for myself for five hundred and go with them, that one too must be there in a week.

**I: She has said clothes, panty (birds chirping), the remaining people, are your clothes brought to you, or you buy them (laughter), (cross talk)**

PK08: As number 8, concerning the panty, on our side, wherever we are working we don’t wear panty, we are just that way free style, after you put on a beautiful and nice cloth (laughter)

P: You are just free.

PK08: You are just free now (laughter), because you are next to the job [ ready for sex work]

**I: Okay, that’s number 9 (cross talk).**

PK08: These makes up too.

P: Number 8.

PK08: We buy make up, like when I am going (birds chirping) to do shopping, when I see that my (birds chirping) powder is finished I can buy it (birds chirping).

**I: So the make-up is on the weekly list?**

PK08: Weekly.

**I: For everyone or there is someone that when she leaves, number 3**

PK03: According to me, I got a client who advised me on some issue, he is a man who has his wives and the wives are very keen, so he doesn’t want too much make up, he is my client that even if he meets you naked he doesn’t even ask you your name because I love natural, I am natural, I don’t do too much make up, the only thing I can apply is the nice and lovely lotion, one twenty (one twenty shillings), and it may take even two weeks, but make up where I apply on my lips and so on, he doesn’t want, and he says that the lips, sometimes you have kissed him, the lips (lip stick) have remained there, the wife will ask him, where have you come from with this lip stick, so I have reduced my make up (laughter).

**I: That is number 3, number 9?**

PK09: As number 9, in my opinion, for me I must do make up, I do make up, during the day like you must natural because you will not tell someone the work that you are doing, but meet me at seven o’clock going to, you cannot recognize me, I do make up such that I cannot lie that I do not do make up. So as number 9, because I don’t like, I don’t want something that can make me not to pay rent, because I step out every day, and I don’t want anything that can make me be at logger heads with the landlord (someone coughs) because there’s no where I am going to stay with the children, so I always try to buy things that can last me even two months, when I do my budget, even if it is hair food, I buy hair oil that can last me two months or three months

**I: Mmh**

PK09: Yes, and if it’s make up, lip stick is not something that gets finished like maize flour, it is something that you just apply, as long as you just apply it a little bit, it can last you (birds chirping) something like one month, and you use it, then you buy it again.

PK06: I am also saying this number 6, that at the time when I have left work even in the morning or what time, it can even be up to nine o’clock, so at that nine o’clock I will find a super market and get in, if not I even go to =K-City=, then I choose a beautiful dress that fits me well, even if it’s a trouser then a beautiful trouser such that when you dress up, you are just seen at the =Nyamasaria= fly over (laughter), such that someone’s son can say that, that is the one I have found. You cannot go with clothes like the ones I have worn here are the ones you go with, when you dress up well is when you become attraction (attractive) to the person who has seen you, even if they see you when passing with a vehicle, hey madam, wait (birds chirping) there a little bit. So you know we should be dressing up well, you put on your beautiful clothes, you become attraction (attractive), your face too, I don’t always apply make-up, after I take a little powder with some Gold Touch, and I have dressed well, I have also worn shoes that matches my clothes, I have succeeded.

**I: So the money that we use on these make-up is what you haven’t told me, dressing up smartly, you have said that you do, and the money for that, how much is it and are they being bought every week or monthly or any time (birds chirping)?**

PK06: It depends on how you are using it because sometimes, like me if I buy Gold Touch it is one fifty, and if I buy it, I can even use it for two weeks with it.

**I: Okay, and dressing?**

PK06: For dressing up I dress up normally, I dress up at the time when I am going, I just put on neat beautiful clothes.

**I: After how long are these clothes being bought (birds chirping)?**

PK06: For the clothes, if in any case God blesses me and I get my money and I see a beautiful one hanged there, then I go and point it, then I say give me this one so that I will use it here, on the field here (work place).

**I: Okay, so that is only the time when you see a good one?**

PK06: Yes.

**I: Number 9?**

PK09: As number 9 if I want to support what number 6 has said, (birds chirping), clothes, us as, I as number 9 personally, when I go to the field where we work, then you cannot go with one cloth every day, you become unattractive to and people will also call you the one with only one cloth, so you just try so that when you come back from work, I mean, the money for clothes, there’s no day you say that, for me such a day I am going to buy a cloth, a cloth is something that when you come and find it, you take it and come with it.

**I: Do you think the money that, not only the ones you use, you and other sex workers, the money for use, where is it coming from, how do they get it? (Birds chirping). The money that the sex workers use?**

P: The ones they give us?

**I: Sex workers are us here, number 8?**

PK08: As number 8 I feel that the money we use, we go to work.

**I: You go to work?**

PK08: Yes

**I: So just explain the work. (Laughter). Just explain the work, there are many jobs.**

PK08: Okay, the work that I do, I go to work when the evening comes at eight o’clock then I go and stand there, then I get, someone calls me that he wants a shot, he wants a little shot and he wants you to go and give him, then I take him, we go to the room and we agree on the price, then he does to me what he does to me they engage in sex], then he gives me, even if we agreed one thousand then he gives me the one thousand and I keep it in the pocket, isn’t that work that I have done?

**I: Okay, so the most of the money that you use comes from sex work or there’s another way that you do get money?**

PK08: There’s nowhere that my money comes from, only my work.

**I: That is number 8, number 8 works, the money that she uses comes from her job, like she has explained to us.**

PK01: As number 1, as I support number 8, the money that I also use on my things just comes from the sex work because, when I leave this place, like right now I am planning to go to the =Airport=, there’s a bar that is up there that is also good, and you go, but you don’t go when you don’t have something [ money] , you go with some money and after you have bought your soda or even one Guinness, and you have dressed well, and you look good, you know that from your eyes and how you look, we know, then someone from the far end can be attracted to you on this side, and he will tell you, madam, my table is free, then you will go and join him at that table because that person had already gone to work, you know that he works at the =Airport= and he had gotten his, so I am going to turn his to mine.

**I: Okay.**

PK01: Yes, so at the time I go there, his that he had worked for, at that time I have also gone to work, then I turn his into mine, and after we talk and finish then I carry his and come with it home, have I not dressed?

**I: Okay, so number 7?**

PK07: As number 7, (birds chirping) first of all I say thank you so much for coming to teach us how we can stay. The advice that you have brought us today, it’s a good advice. Truly we have been doing this our work, and we have not known where, what we can do with this money. There’s some time when we can get, a lot of money, someone can even give you ten thousand, thirty, and you know you don’t have in mind what you can do with this money. So the advice you have brought here, in my opinion it is good, so your money, these our people, as we are here in a group, with this our work that we are doing, we can leave here and have our group with our understanding and open an account or what, that is my question, you can reply me as people who have come to help us?

**I: Okay, your question (cross talk), that question is going to be answered (cross talk) later.**

PK07: Okay, then on the side of work, I am a sex worker that, I am not so much in =Kisumu= such that when I come back to =Kisumu=, this is where I live. Mostly I go to =Nairobi=, and right now I have two weeks since I came from =Nairobi=, I have not been in =Kisumu= for a sometime, so that is why I am happy that I have met with my fellows, it has been a bit long, and wherever I usually go, it is hard, are you getting me, if you are truly a real sex worker, that place, they start by looking at your cleanliness and your dressing, and how you dress, and also food, like you are a sex worker, you can sit on a table with your bottle or your soda that you are drinking, it’s when you get a customer that calls you, either you go to him and talk to him, and the way you talk to a customer is the way you continue getting a customer. In a day, like me I can say that in a day I can be with six different people, because there’s a shot person, there’s someone for the whole night, so the person for the whole night, I agree with him, if he is someone that I can see is well off, then there’s a price I will charge him. But mostly, the shot people, someone says five hundred, so that is what destroys us. Sometimes you find someone that tells you two hundred, so that destroys us, like that is the work that I do that I educate my child with and I pay the rent with here in =Kisumu=.

**I: Okay, so all the people who have talked are saying that all the money they get comes from sex work?**

P: Yes.

**I: Are there other sources that may be, other ways that brings us money that is different from sex work?**

P: There’s none.

**I: Amongst all of us, there’s, number 9?**

PK09: As for me, like sometimes I always go to =Kondele=, and in =Kondele= we have unity, for Maleche (Sex workers) in Dholuo. So when I joined this thing, I joined it with the help of my friend, because I did not know how it could be done so that I could know the price or know that one shot is this amount of money, so I used to ask her and she tells me. So she always has some drug, such that after you see someone’s pocket and you see that it is good (notice if someone has money), and you agree that you are going to have sex the whole night, so you just drink, but you don’t drink literally, you drink with your network high (be cautious), you keep on telling him to drink, and when you have taken enough you go to the washroom you make yourself throw up, you put your fingers into your throat and you vomit all the alcohol that you took so that you don’t go and sleep, it is something that you know that, today you are going to steal from this man, you are tired. So when he has taken alcohol such that is eyes have started feeling dizzy, he must go and ease himself, he won’t just drink twenty four seven, he must stand up, so at the time when you have seen him stand up, you take it then you drop it in the alcohol and you shake the glass and leave it there, so if he comes and takes it then he is just going to sleep, such that there’s nothing you are going to do, when morning comes, he finds when, sometimes he even tried one shot and he slept, after he slept then you just take what’s yours.[ steal from him]

**I: So you steal from him (cross talk)**

PK09: You steal from him, when he wakes up he finds that you are not there, you have even taken some seven (seven thousand) and gone with it, you are going to treat yourself.

**I: Okay.**

PK09: Yes

**I: So do you buy this drug or you are being given?**

PK09: This drug, that woman is the one who gives me, so we agree with her that if I succeed then I give her, I mean when I succeed wherever I was going, when I get good money then I give her one thousand.

**I: Okay**

PK09: That’s hers

**I: Okay.**

PK09: Yes

**I: So that is also through sex work that you get this money, right?**

PK09: It’s how I get the money

**I: Other ways that can be there that you can get the money apart from sex work?**

PK01: As number 1, you know that when you are doing sex work, you don’t even want your parents to know that that is the thing you are doing wherever you are, you don’t even want the neighbor to know what you are, so someone like me, I always pretend and go for some jobs here in town, the hotel work, you pretend and do it day time, and the owner of the hotel pays you some three hundred, the neighbor knows that you always go where, to town, and when you come back, you bath nicely and dress up and leave, the neighbor knows that you have gone out as usual, but you have just gone to your work, it’s when you have two sources, because you know that during the day, for the neighbor not to know my thoughts, I am there.

**I: Okay, so the one that you say you pretend with, is it higher than the money you get from sex** **work or it is lower?**

PK01: It is lower.

**I: It is lower?**

PK01: It is lower.

**I: Okay, someone else that has another source?**

PK09: As number 9, I know how to plait, I always plait people, so during the day I always pretend, there’s my friend I always spend with here at =Pipeline=, so during the day I go and spend there so that I can get market from there, because obviously the person who you always go with in the evening, even when you see him passing you just know that, that’s my man, that’s my market, where I will go and buy in the evening,[ client] so I always lie, I always go as if I am going to plait, and whether I plait or not, I just know what I am doing so that the landlord won’t see, you know that when you stay in someone’s house and he doesn’t see you leaving in the morning and coming back in the evening and you don’t have his debt, one day he will just say that you are a thief, you have stolen someone’s things, so you look for a way that you can pretend, so that he can know that, that lady is not a thief my fellows, that lady always goes to =Pipeline= to go and plait, so I always leave as if I am going to plait, but I don’t value that plaiting so much because in plating, when schools open like this is when, I mean when schools open, you can get school children on Saturday and Sunday when they come for you to plait them lines.

**I: That can give you how much in a day? (Someone coughs)**

PK09: The lines for a school child is only fifty, one child fifty, so it depends, sometimes you had good luck such that you plait four children, two hundred shillings, this two hundred shillings, you split it with the owner of the work, because that is not your place, you are employed.

**I: Okay.**

PK09: You split it, yes.

**I: You have told me the things that you always buy with, the way you always budget, weekly,** **monthly, daily, buying clothes, (birds chirping), why is it that the money you use to buy those things are used that way, and the moment you get money, do you split it that, this is for food, this are for** **clothes, these is for, that way?**

PK09: As number 9, (birds chirping), the way I always use my money when I get it, before, when you had not brought to us such a teaching, when I got my money, I did not look at it like I am keeping this one so that tomorrow I can buy clothes with it, because I know that (birds chirping), I use it now when calculating that I in the evening I will still be at the field (work), and tomorrow I will come with something.

**I: Mmh**

PK09: So I as number 9, the moment when I see something, when I see a cloth then that’s the time I buy it and come back with it, yes, because I know that tomorrow I will also go back.

**I: Number 8.**

PK08: As number 8, I sometimes try to save, if I get hundred, I keep fifty, so when I see a beautiful cloth I tell my customer, because I always have a customer that brings me clothes, I tell her that, this cloth, tomorrow when I go to work in the evening, please bring it for me because it is the one I will put on, then I take the fifty shillings that I had saved at the house, then I give it to her and she gives me.

**I: Okay**

PK08: Yes (birds chirping).

**I:** Why **do you spend on a cloth?**

PK08: At the time when I am going there, then a cloth, you know that when you dress well you are seen better than, even if you are ugly it can make someone come from far come to you.

**I: Yes.**

PK09: As number 9, (birds chirping), when supporting what number 8 has said, you see like myself, I work at =Kondele=, most of the time I am at =Kondele=, so for =Kondele= most when I leave this place, I don’t always wear the clothes that I use to stand there,[ parking] when I leave this place I just leave well wearing a nice trouser and a top, and I go, so after reaching that place, so there is a house where, we are in a…, at =Kondele= we are as a group, we are united, so in this unity, there’s a house that we have rented, and it is just for dressing up, so when you reach that place then the bag you carried is what you use to change your cloth even if you wanted to put on a small short, even if you wanted to put on a mini skirt, is when you put it on there. (Birds chirping),

**I: And is there money that you spend on yourself, (birds chirping), and there is money that you spend on other people, right [ mentions participant’s name]?**

PK03: Yes

**I: Sorry, number 3.**

PK03: Yes

**I: There is money that you have said you spend on the children, there is money that you spend on parents, and there is money that you spend on yourselves. Why do you spend the money that you spend on yourselves, she has told me one that when you are smart, then someone will see you from far, right?**

P: Yes

**I: Something else that makes us spend money on ourselves, we spend money on ourselves?**

PK01: As number 1, you know that when you are staying in a house then you must spend, yes so that is one of the things that I use money on, you must pay rent, you must also take care of the child to be clean, (birds chirping), you must also buy food. (Birds chirping)

PK06: As number 9, (someone whispers) as number 6, I can spend money on treatment, sometimes I can get sick. I can go there, sometimes I may find a job that is a rough case (rough sex) such that you may come back when you are sick, so when it finds when you didn’t save money, you may lose hope when you work, sometimes the money that you were given, you find that you are given money, and you have budgeted with it and the way that it has remained, you are also sick and you want to go for treatment, because, sometimes you might be sick such that you should be going to the hospital, not just for taking Panadol[painkiller].

**I: Okay**

PK06: Yes.

**I: She uses the money to treat herself when she is injured at work, someone else again, number 4?**

PK04: As number 4, I must spend money because I have a child in secondary, (birds chirping), you may find that you can even be called by the principal that, there is some money that is, I don’t want to send the child away, there is some money that is needed, so if it’s there then go to the bank or do this and this, so that is why I must spend money.

**I: Okay, number 10, is there money that you spend on yourself? (Birds chirping)**

PK010: Pass first.

**I: Okay, she has said that I should pass first, who and a ready answer so that we can move, number 3?**

PK03: As number 3, I must spend money on myself, like it has been said that someone should be smart, I must be smart, I must dress up, I must make my hair, because you cannot go there when your hair is smelly, I must have a spray that I spray on my hair such that even if I hug then my hair is fresh, so I spend money on myself so that I become neat, such that even if I am going where people are then I don’t be someone who is unkempt., that way, I must spend on my body.

**I: And do sex workers save?**

PK01: On saving, you are the one who has brought it to us today such that we have known that we are supposed to save but most of us, some of us save, and others don’t save, but someone like me, when I got it, and you know that as a sex worker you look like a fisherman, or a gold person that entered the hole (gold miner), you know that I have dipped myself there and tomorrow I will also deep myself in another place, and I will get out with something, like a fisherman too, when they go to the lake and fish and then comes back with five hundred shillings and it all finds work, he uses it on ugali, on the flour, and he knows that tomorrow he will go back to the lake, so some of us save and some of us do not.

**I: That is number 1, number 8, (someone coughs)?**

PK08: As number 8 the reason why I save, my child is at home, I can be called any time that he is sick, sometimes the money that I had, I have used the whole of it, it is when I will wait till evening to come so that I can go and get some money somewhere, sometimes my mother is sick, because I am the bread winner at home, so I must save fifty shillings, if I get a hundred I keep fifty for tomorrow, for emergency.

**I: So you always save every day or?**

PK08: Anytime I get it

**I: Anytime you get it?**

PK08: Yes

**I: So if you (cross talk)**

PK08: If I get a lot I save and if I get a little bit then I just use it. (Cross talk) But mostly I save.

**I: How much can you save in a week?**

PK08: I always put it, you know that at work you cannot get out that today you are going to get one thousand, sometimes you may leave that you are going to get one thousand and you are going to leave with one hundred.

**I: Okay**

PK08: So I cannot say that I save one thousand, when I get I always save one hundred per day.

**I: Okay, that is number 8, number 10?**

PK010: For me I always save, the reason why I always save is because I have a baby, sometimes the baby can be sick, and I can also get sick such that I don’t go to work, such that I only use the ones I saved, then I will go to work again and return it, I usually save.

**I: So you always save every day or once?**

PK010: I always save every day.

**I: How much?**

PK010: It depends with how I worked, if I worked well, I save well and if I didn’t work well, sometimes I can even save a hundred shillings.

**I: Okay, and the money that you save in a week, how much can it be if less?**

PK010: Sometimes I can save in a week until it even reaches one thousand two hundred.

**I: Okay.**

PK010: Yes.

**I: Number 4 wanted to talk, do sex workers save, and why do they save?**

PK04: As number 4, (someone coughs) I must save money because, you may not know, you might go to the field (work), and you find when the field is not good mostly a time when schools have been closed when the children are around, you hear them say right now the prices have gone down, that thing is fifty shillings, so you will go there and we will even take school children. You may go when you have your normal price, or you say that, for me if you want me to go and give you one shot, it is five hundred, he will tell you that he cannot give five hundred, he is going to give you two hundred. Sometimes you will only have two hundred until it reaches a time when you are going home, so when you come back home, it will force you to just use the whole of it (birds chirping), you cannot keep it, but when you just go when work is good, the field is good, and you come back with two thousand or three thousand, I must keep one thousand (birds chirping).

**I: And the habit of those who save, how would you know it, how will you know that so and so saves and so and so is not saving, if you look at someone and her habit?**

PK04: As number 4 as I answer that, if you want to know that so and so is saving or so and so doesn’t save, sometimes you have gone to the field (work), and you have found when it is not good, I mean the field is dry (no job), I mean when you call someone he just passes, when you signal someone he just passes, if you try sitting in the bar, there’s nothing, so you know that when you go back to the house tomorrow, your children will just eat, your life continues normally, that means that you are saving, and your fellow, even if you are neighbors and you know each other, there’s a way you know each other, you will find when she is in a fix, you will find that her children have not eaten or she will tell you that, so and so can you lend me even two hundred, yesterday the field (work) was not good, and if you have a good heart you just take it and give her, and you tell her that, you will return it to me.

**I: What character does the people who save have, that is number 4, who wants to add number 8?**

PK08: As number 8, when someone is saving, sometimes someone is passing with clothes, and it is when you have come back and truly you don’t have money, both of you don’t have money, and you see your fellow removing money and buying that cloth, sometimes it was an expensive cloth, you are just going to judge that this girl always saves money (birds chirping).

**I: Yes, number 10.**

PK010: If I give my opinion as number 10, when you want to know someone that saves and someone that doesn’t save, sometimes when the field (work) is not good, she might even need fifty shillings because she is going to sleep hungry, and a person that saves that knows that she has left some money behind, [ has some savings] she cannot take fifty shillings because she knows that she has left money at home, that she is going to start with.

**I: Okay, and how do you know people that do not save, you have explained to me some and the ones you can add again, someone who doesn’t save, for someone who doesn’t save, if there’s no client then her child sleeps hungry.**

P: Yes

PK08: As number 8, a person who doesn’t save will be known in that, sometimes you have both gone to the field (work) and your fellow comes to you that, so and so, please help me two hundred shillings so that I may return it to you tomorrow, and for that you will just judge that the money that she had earned yesterday, she used all of it, she did not keep some, the next day you will see that she goes to ask another person, because sometimes she has nothing and she didn’t save it, that is how you know her.

**I: Number 5**

PK05: As number 5, when you want to know someone who doesn’t save, in case she has an emergency, you will find that she will run and come to you, like in case she has been called like when the child is sick, so and so please help me with such an amount of money, I will return it to you at this time, meaning that she has nothing in her account, she doesn’t have anything, you will notice her very faster. And the way I am a sex worker, I have my iron sheet box for keeping money in the house, so when I come from my work I must put even a maximum of two hundred, such that in a week I want to have one thousand for emergency that I just have in the house.

**I: So you open the box weekly?**

PK05: No, now I know that I have it every week, I cannot remove it, it is just something that is there every week, I know I have my two thousand I cannot open it.

**I: Okay, number 9**

PK09: As number 9, when I want to talk on that issue then, someone who doesn’t save, you find that someone has come to this lady, and after coming to her, he has told her that, for one shot I have one hundred, and the way she saves, she will not accept, she will tell him, no, it’s five hundred, if you don’t want, go, and for me who doesn’t save, I will see that my children are going to sleep hungry, I will just go, I will just advertise myself and when he comes to me I will just go so that my children don’t sleep hungry, she will not leave the money, even fifty, she will not leave, she takes it.

**I: Number 3, someone who doesn’t save, what is her character?**

PK03: Someone who doesn’t save money, as number 3 as I have seen, first of all I have not been able to save, when I save money, the place where I kept it, today I will tell you that I thank you, I don’t know how to save, I know very well, I do this thing[ sex work] at =Pipeline=, for truck drivers you cannot miss one thousand daily, so it is something that I know that every day I must go and get a thousand, so I have relaxed such that I don’t bother, but a person who doesn’t save like me, sometimes I pass through hardships, you find that, I have children at school, and you find that I have been called, parent, such a thing is needed, school is somewhere that things can come as an emergency as a school, such a thing is needed, it becomes hard and I can even go to my fellow number 6, I can say, number 6, can you lend me five hundred” she can say that, the way you worked very hard, where did you take the money, and I can say, please just help me, we will see what we can do, so I can say that savings is good, so from today, I have changed, I will start saving.

I: Okay, and why does saving become easy to the people who save? To the people who save, what do **you think makes saving easy for them, number 8?**

PK08: The reason why saving becomes easy for me as number 8, it depends with how I am, my child is at home, I am being called all the time, that send this amount of money, and that child too, sometimes there is a certain food that he wanted, it forces you to send money. Secondly, sometimes you have gone to your fellow so that she can lend you some money, she cannot lend you, she tells you, did we not work with you, there are people who have bad hearts and are not helpful, so she will not tell you that, did we not work with you, so if you did not keep your money from yesterday, I am not giving you mine, so you will be angry sand say, I also want to start saving money the way she saves.

**I: What makes saving to become easy?**

P: The thing that makes saving to become easy is that, you can save money because you don’t know what might happen tomorrow, then secondly, you can save money because the child is going to school, any disease can strike a child for emergency, anything can happen, even at home, even away, sometimes you are being called, and you are needed somewhere urgently, even one of your partners that you were having a relationship with there, and you gave your number, he can tell you that, so and so, come to ==Bumala==, so if you did not save money then you will ask me for money, and for me I will not give someone my money that I have saved, that I give you so that you use it to go there, so we should be saving to make it easy for each and every one, when she finds a problem, or any difficulty between her and the place where he is living wherever she is.

**I: Is there anyone that wants to add there, the reason why savings becomes easy to the people who save, to the sex workers who save?**

PK09: As number 9, I want to add on that, I think that what makes those people to save, as number 8 has said, because, you know that someone like me, I have all my people here, I don’t even have parents that are going to ask me for money because I don’t have parents, I am just this way with these my people, so I feel that there’s no one that is going to ask me for money, so I will just use my money. Then something that came, the box that number 5 had mentioned, I feel that when you drop money in that thing little by little then the day when you remove it, you find that you have a lot of money, and we are different from a person who works per month, because a person who works per month she knows because she might use it in the middle, such that she must just save her money, and if she doesn’t save her money, then she might stay hungry, and for us, the thing that hinders us from saving money, it’s because you know very well that tomorrow you will still go there and you will find it, and if you miss it tomorrow, you will find it the day after tomorrow, you cannot miss on two consecutive days.

**I: And these people who save, what challenges do they go through, the challenges that they go through because they save, which ones are they? The people that save, which challenges do they go through, yes number 3?**

PK03:As people who save, you know that when you are saving money you should deny yourself, you must decrease your budget. You might find that a person who saves money, she can be, you know a malaya [ prostitute]in Dholuo, likes beautiful things, a prostitute wants her house to have a fun (someone coughs) that she uses sometimes when she is feeling hot, she wants to be at a cool place, so you know that if you are a person that saves, you are going to see something passing even if they are beautiful curtains and you are going to say that, when I buy this thing then my target that I should save every day that is two hundred, I am not going to buy it, so let me just leave it, so she can live it a life that she is forcing herself with that she must live there so that she can save. So a person that saves, it becomes squeezed (difficult) to buy her things sometimes, she feels that she is tight, she is very tight.

PK01: If I support number 8, with the Luo word that she has used, a prostitute, a prostitute is someone who, a person who is truly a prostitute, is someone who even if you enter her house, when you open the door and enter and she tells you that she is a prostitute then you will say that she is a prostitute, a prostitute is supposed to live well, the fun that she has said cools you, a T.V that is that size, such that when those clients come to your house, he respects you, and tomorrow he will again do what, he will say that, when I enter that house I sleep comfortably, the bed that you sleep on, you make sure that you spread it well such that it is seen as a neat bed such that when someone sleeps, and when he goes back to his house, he thinks that, let me finish one week and go back there. So a prostitute is someone who lives well, so if you save, then you must squeeze your budget, you know that you save such that you can’t even buy a bed sheet, you save such that a beautiful blanket cannot even do what, you save such that you can’t even stay in a beautiful house, because you are saving, so even if you go to =Nairobi=, sex workers in =Nairobi=, you even find that a sex worker is living in a house worth forty thousand, and her children are being well educated, and she also eats very well. Saving is not bad, people are saving, but after you have used a Luo word that I am a sex worker, a prostitute, a prostitute should live well. (Cross talk)

PK09: As number 9, if I want to support what number 1 has said, truly, the place where we stay should be good, such that even if you came with a client, then even if he wanted to give two hundred shillings, then he just feels ashamed because even the bed sheet that he sleeps on cannot be that price, it is better than that two hundred, it exceeded that and even passed it. So the way you live also makes someone’s pocket, makes someone to just judges whatever he is giving you, such that even if you said three thousand, he feels that truly, it matches that three thousand, there’s no bargaining, depending on the bed that you took him to, the chair that he sat on, the way he, I mean, the way he has been, it’s different.

I: Okay.

PK09: It makes the price that you agreed on, he does it how you agreed, there’s nothing that he bargains.

I: So the challenge is that when you save you are squeezed financially?

PK08: Yes, I also have

I: Yes, number 8?

PK08: As number 8 I feel that people who save like us are finding it very hard, sometimes the person who doesn’t save has come to you, she knows very well that you always save and you always have every day, she has come to you that she wants, when you don’t give it, she will be mad at you (birds chirping). When a blanket passes, you as a person who doesn’t save, you want your house to look beautiful, and I that saves, a fan passes, will I not but a fan and I have saved money so that one day I can buy something with it, and for you that didn’t have, is the fan not going to pass you and I am the one who is going to buy it? So we pass through difficulties as people who save, (laughter).

I: Mmh, (laughter), so these difficult things that the people who don’t save pass through, how can we help it, like the one for squeezing yourself so that you don’t buy things, is there a way that it can be addressed? (Birds chirping)

PK03: Yes

I: Yes, number 3?

PK03: I feel that I can address those who save, even me that wants to start saving, that when you are saving, it also has its benefits (someone coughs) because, once you are saving money and I wanted a seat worth thirty thousand, then it forces me to save so that it can be worth whatever I wanted so that I can do what, I can take it when I have the whole of it (money), and you know that someone who doesn’t save wants to buy her things bit by bit, and for someone that saves can just leave this right now, I just leave you like you my neighbor just within one hour and I just come back when I have changed and you will say, where has this woman got the money. So a person who saves gets money once and she goes and gets the thing that he wanted once, she perseveres but goes and gets whatever she wanted once and she comes with it, better that bit by bit for a person who doesn’t save, she goes and buys whatever she wants.

I: The challenges that people who save pass through, how can they be solved? Another person

PK01: The challenges that these people pass through as number 1, that we can pass through or that we are going to pass through as we are going to start saving now, because now I feel that this issue on saving is a good thing and it can also help us, so we are just praying that if God allows us, and if He makes for us the customers that we have, the we put prices that are a bit higher such that you can find a way that you can save, so that you don’t fear spending a lot, so if you find a person with a good heart and you charge her one thousand and he gives you that one thousand, it is obvious that you will just use six hundred and you save four hundred, that is understandable, and if someone gives you two hundred then there’s no way you can save. So it’s just our prayer that when the economy becomes good and things also becomes good, and our customers also have something good in their hands, our savings are also going to be good and there’s no any other difficulty.

**I: And for people who don’t save, is there any bad thing that they pass through because they don’t save? Number 3 has told us that sometimes there can be an emergency, and she gets stuck because she doesn’t have any money that she kept. Another one, any difficulty that can befall someone because she has saved, number 4?**

PK04: As number 4, a person who doesn’t save passes through a hard time because sometimes she might be late with the landlord’s money, and when she leaves, she will find when the landlord has closed the door for her, so when you ask, why has the landlord closed the door for you, then she says, I do not have the rent, then you ask, you don’t save even a little bit, then, I do not save, I always use the whole of it, so that is a problem that they go through.

**I: Another problem, number 3?**

PK03: The third problem that someone who doesn’t save passes through, you know here in sex work there’s also something that you can be sick, you can be sick and you be in the house because when you are sick you cannot go to the field (work) because you are sick, you won’t go to work and sometimes you have children, so if you didn’t truly save then your children can even end up sleeping hungry. Secondly it is work that may be so extreme until you become pregnant, and give birth, and when you just give birth and you have a small baby, you cannot go and do that job even in two days, even in one week even in one month and you have started going to do that work, you must first sit in the house and wait for the baby to be stronger, so that too can make you find it hard, when you were not saving.

**I: Another one, what are the advantages that can be there when someone doesn’t save, the goodness of not saving? Anything that has its advantages, has its disadvantages, and after you have said its disadvantages, even if only one advantage is got, there must be a little.**

PK09: As number 9, in my opinion, if I give my opinion on its advantages, the advantages that I see on not saving, it is that my things are doing well, I don’t see my things doing bad, yes because when I come with the money, sometimes you may have luck, you found someone, he has given you three thousand, you have come from the field (work) and you have gotten someone and he has given you three thousand, you have again stolen from someone, and he has not slept with you[ engage in sex ], and you have stolen from him, you have stolen from him even some five thousand and you have come back with your money. This my money, if I go and put it, let’s say I want a seat like this one, and I go for it and come and put it, (someone coughs) will I not just sit and my life goes on. When I come back I say, let me pass through here and eat pork, meat is very delicious to me, I carry my pork and come and fry it here in the morning when treating myself. And you know someone that saves will say, let me keep this, send this to the child and use this on this and this on this, then this two hundred, I will go and *buy omena* for two hundred then I eat. So I feel that my things are going on well.

**I: Another advantage for being that someone doesn’t save? (Background noise) Number 3 shook her head (laughter)**

PK03: I shook my head because I have seen the disadvantages of not saving, that is why I say that it has no advantages, because what you get you use it at that time and that’s how it ends like that, so I only see a lot of its disadvantages, I don’t see its advantage so much.

**I: Who sees its advantages that supports number 9, number 9 has seen one advantage there? Number 4**

PK04:As number 4, when I don’t save, when I go to the field (work), because at the time when you are going to the field you must do the sign of the cross, Lord help me, because even a thief prays, so at the time when you do the sign of the cross God is going to remember you, you can go and find, a man who is serious and you go and you buy your Guinness and you sit and you find someone who is serious, hi madam, what’s up, I want to go with you till morning, I want such an amount of money and he gives you even three thousand, and you go, you sleep till morning. So you know when I leave that place, I pass by Tuskys, and I say, I saw a smart cloth at (mentions name), let me look if the cloth is there, then I go and look if the cloth is still there and I take a trouser plus top plus shoes, then I do what, I take it and come with it, so you know that I have finished the chapter of that cloth. When I come to the house, I look at one, two, three that are not there, let me do this and this so you see your things just going on well.

**I: Okay, and these people that don’t save, why do you think they don’t save, why would someone decide not to save?**

PK01: As number 1, someone might decide not to save depending on the budget that she has in the house or, sometimes she has many children, and these children are going to school, they are eating, you also want to pay rent, so you know that when you come back with money form that place, then it may be difficult for you to save, depending on your budget at home, you may come with it and use it again until it is not sufficient

**I: Okay.**

PK01: So you know that it can be hard for you to save again.

**I: Someone else again, number 3.**

PK03: Secondly, what can make someone not to save, what can make?

**I: Someone not to save**

PK03: What can make someone not to save (laughter), what can make someone not to save money, (birds chirping), he may come, like us we, as I had said to you earlier that, someone doesn’t save when she sees that tomorrow she will still get it, and also lack of knowledge, lacking education like the one you have given us, like me, lack of knowledge made me not to have thoughts that I can do savings, when I get money I just use the whole of it, so lack of knowledge can also bring that.

**I: Someone else again, number 5, what can make someone, not someone, what can make sex workers not to (cross talk)**

PK05: Sex workers cannot save depending on times, sometimes the work is not good, the customers are less, and when she comes from that place then she comes with little money she uses the whole of it for budgeting such that there’s not none to save.

**I: Someone else again? And when the customers have reduced the way number 5 is saying, what do the sex workers do, there are no clients, what do the sex workers do? Number 8 you have a point.**

PK04:As number 4, when there are no clients, like I as a field worker (sex worker), in Dholuo I am malaya[a prostitute], when customers are not there like right now they are not there, because of the school issue, so I just try with the budget that I always have (birds chirping), and then I don’t use money as I waste, I use it when calculating, I say no, if the children wanted to eat meat, I tell them, today let’s fry kales with onions and oil, and eat, so I time with that and see how the field (work) is doing, the day when the field (work) is good, then they just eat the meat, so you must try and deny yourself, if you have two hundred, you can use that two hundred up to supper.

**I: Okay, when there are no clients, what do sex workers do?**

P: When there are no clients, I can just go and report and see if I can get any that I can get, and when it is impossible, then I use my money that I had saved, that is what I now use knowing that in the future I can go back, and tomorrow or the day after, God can bless me with another one, that is why saving is good, the time when there are no customers at all, so the one that you had kept at the saving pot, is what you open and use it to solve your little problems like you did here.

**I: Okay, number 9**

PK09: As number 9, when there are no clients, then as we had said earlier that you cannot stay in someone’s house without a job to cover up with, there are people who are covering up with hotel work, there’s one of us here who is covering up with hotel work, like me I am covering up with salon work, if in any case I went to the field (work) and I didn’t work today, I will still go back to my house and talk to God, God is also talked to as a human being, I just talk to Him and tell Him, God these are your children and I did not go to school and my school is not complete, so I left the field and I have left going to the salon, God let it not be that I have gone to this side and I have not gotten and I have gone to this side and I have not gotten, you will find when God has opened the way for you, if you didn’t get at the field, you must get on this side until you get a little that (someone coughs) can sustain you.

**I: So when there are no clients you go back to (cross talk)**

PK09: I go back to the hotel.

**I: The job that you cover up with?**

PK09: Yes, the job that I cover up with.

**I: Number 8.**

PK08: Yes, you can sometimes miss clients but they don’t all miss, there are some that we have their contacts such that they become customers such that when you wake up you can tell them, could you please send me something and I will pay you when you come, right now I am in a fix, then he sends you, they don’t just miss totally, you must take someone’s number, so that when they are all lost, then there is one that can hear your problem and help you.

**I: Number 2, what is done when there are no clients, what does a sex worker do?**

PK02: If I support number 8, it is only what she has said because truly, these people are not the same, their ability to give is also not the same, you must find someone that for him, he knows that you do that thing [you engage in sex work] but he listens to your problem, because we might love to save but it reaches a point when it is hard and you even use whatever you have saved because life is not easy. You must have your one or two that if you call him, he must send you money for vegetables and say, let me remember her, she also remembers me, this thing cannot be credited but there’s someone somewhere that can remember depending on how he helps you, this thing is never credited from way back.

PK03: I have a concern I want to support number 2.

**I: That is number 3**

PK03: Yes, I am supporting number 2 saying this, all these sex workers, we always have back up, we have men that, we even have three different men, that is special that, sometimes he doesn’t even know my job, he is just my special such that he knows that he is my friend, such that at the time when he needs me we can meet, so you know that he is not at the field, so at the time when I have a hard time then I must go to my backups, hello, I have a problem, can you do something for me, he must do it for you, because we also have terms that we speak in such that if you talk to someone’s husband then if he was sitting next to his wife then he must pretend on the phone like hello, hello, and when he goes out then he will listen to my report, so the backups always help us when there’s no work.

**I: So you have said that these backup are not met in the field, where are they met?**

PK03: You can meet them even in the bar. You can be with a sex worker like I had someone who we had communicated, you know that a sex worker gets anyone and not that they have met and they want to be friends, this is where we meet and this is where we do our business, and that is a person that met me at a wedding somewhere, and I attracted him, so we are friends with him, and sometimes he is in =Nairobi= (someone clears her throat), I always go to visit him, or he is in =Kisumu= town and I do parking wherever I am in =Pipeline=, so when he calls me to town he knows that I have gone as his friend, so those are backups that, sex workers should have backup, not that you must go and depend on those that you have found in the bar, or the people who have found you on the street.

**I: Okay, and do you think that it is something that is usually known in advance, do you always know that there are no clients, or you always just go and realize that there are no clients? Number 8.**

PK08: As number 8, we know that thing. After you have gone there you will hear your fellow ask you, so and so, today the field is very dry (there’s no work), are we really getting something, and I will tell her that truly today the field is dry, there is no hope, that is something that you know.

I: You know it after you have gone to the field or before?

PK08: After you have gone

**I: Okay, and is there one that you can know before going to the field?**

PK08: You have to go.

**I: And the sex workers that save, where do they save?**

PK04: As number 4, I save on the phone.

**I: On the phone, M-Pesa, M-Shwari?**

PK04: M-Shwari

**I: M-Shwari**

PK04: And I lock it

**I: lock?**

PK04: Yes

**I: Why do you prefer that one?**

PK04: So that, I can go to the field (work) and I miss, and when I come back I can say, let me withdraw this money, so you know that when you lock it you cannot withdraw it until it reaches the time that you indicated.

**I: That is number 4, she saves money on M-Shwari and locks it so that it doesn’t become easy for her to withdraw it, number 8?**

PK08: As number 8, I save my money under the mattress (someone laughs), yes, because I save it to save me at any time, it is an emergency so sometimes, so sometimes something was passing and I put money on the phone or I gave it to someone to keep it for me and he/she is not around, there’s no way it will help me and it was something that I had hoped to help me anytime, so I just pull it out under the mattress and say give me that thing, so I just save under the mattress.

**I: Number 8, under the mattress for her to access it easily, number 2?**

PK02: As number 2, I would like to put it in M-Pesa and the reason why I would like to put it in M-Pesa, sometimes I leave here, I am here in =Kisumu=, and I feel like, this week has not been good to me here in =Kisumu=, let me go to =Bondo=, I must spend fare to =Bondo=, so you reach that place that you were thinking is good such that you will even get the fare to come back with, and =Bondo= is just the same way you left =Kisumu=, so you will say, what will I do and it’s also night, I will not be stuck here, I will just go to my M-Pesa, and it will force me withdraw fare that can take me from =Bondo= up to =Kisumu=, and that is why I can like M-Pesa.

**I: Number 2 M-Pesa number 6?**

PK06: I support number 2, I can say I save on M-Pesa. I can even go to our job, and there’s no job, and how I can eat, there’s no one that can even buy you food, and I can withdraw money from my phone and buy food that I can eat when waiting for customers that can come later

**I: That is number 6, number 7 (someone coughs)?**

PK07: As number 7, I save on the phone on M-Pesa, because this M-Pesa, sometimes I can go to work and truly finds that there’s no job, sometimes I sat and ordered alcohol and I am drinking, and you know that I knew there was a client that was coming or even my friend that I was fond of, I talked to him and said, I am broke, can you pay this alcohol for me, and he said I don’t have anything, it forces me to go to my M-Pesa and pay this money and come back to the house, so on that, someone that saves has an easy time.

**I: That is number, 7 M-Pesa, number 9?**

PK09: As number 9, in my opinion I keep money in the house, the reason why I keep money in the house, is because I have children, anytime sometimes when I leave, then something happens to their fellow, so they will not wait so that the mother should come back with the money is when we will buy something, they know where I keep money, they just go and take it, it’s only that when I come back, they tell me that, mom, such a thing was not there and we took this amount of money, so my money, rarely, I put it on M-Pesa, most of the time I keep my money in the house.

**I: Okay, is there someone (clears her voice) with a different thought from M-Pesa and saving under the mattress that have been said to us?**

P: No

**I: There’s none?**

P: Yes

**I: Do you think sex workers live in… number 1 told us that when you open a sex worker’s door and get in the house, even before she does an introduction, you just know that this one, this is a sex worker, because you look how?**

P: Environment.

**I: She lives. Do you think that there are sex workers that have low income but have high living standards, they have low income but have high consumption? Number 3**

PK03: The reason why those are there, that is what brings the challenge of lacking savings, because your expectations are high and your earnings is low. So you just feel that the money that you have earned, you use it to make yourself beautiful like you want those beautiful things but now so you may lack some to save, because your income is low, you cannot want your beautiful things, and save some, so that is what brings the problem because her income is low and her expectations are high so it even hinders her from saving, that always happens.

**I: Someone else?**

PK01: As number 1, it depends with the environment where you do that work, sometimes you are doing it where there’s money, you will find that your income will be high, sometimes you are working where those people, their money, their rate is low, that will also make your earnings to be low, yes, so it depends with where you are working.

**I: And what if your income is low and you have a high life style, because a sex worker’s house must be made beautiful right?**

P: Yes

**I: So your earning is low, and your life style is high, the money that is to be used to increase your life style, where does it come from?**

PK01: For that you must just be clever.

**I: That is number one, how do you become wise?**

PK01: You must be clever because now you won’t have one, or two, or three you these people, you will have them such that even if you have twenty of them you just line them like this, and you just know that with so and so, when it reaches a certain day then I am going to get something like this from him. You can be with a person who you budget with that, for me I want a T.V, and if he has it, you find that he buys it for you and brings it, when he has brought it to you, you have just done prostitution is when you have got it. So it depends with the people you have, sometimes you have someone who you can tell, for me I want a chair, and you find that he has brought this chair with a good heart, and it’s not like he is the only one who will sit on this chair, even others will sit on it, because himself, he knows that he is the only one, and for you, when you list them you find that they are twenty eight people that you have just put there with telephone number, and you do not delete them, and you do not even put any on the black list, they are all on (going through). So you know that he has brought the chair, he doesn’t know that someone else brought a T.V and another person brought a tab….

**I: Table.**

PK01: And he finds when the house is smart, so this thing, it doesn’t depend on the money you have earned, it’s someone’s heart, you may find someone with a good heart and he can say, this your child, I am the one who is going to pay for him school fees until he is done with her/his education and he will pay for your child school fees until he finishes and he has a wife and the wife doesn’t know, until your child reaches university, because if you truly look at this sex work job, you find that someone has educated her children up to university level, not that you have educated your child up to university level in that you sleep with people all the time, it is just talking to your customers and you find one that you love and you know that this one, make him close to you such that you can tell him that do you know that you are marrying me next month, when next month comes you tell him that let’s postpone it, just postpone but what you want is what you want to get, so for you to make your house beautiful, it doesn’t depend with earnings, you may be truly earning eve five hundreds or one thousand per day, and you have made your house beautiful, you have made it beautiful with money from different people. (Laughter)

**I: The list is also long?**

PK01: Yes, the list is long.

**I: That is number one’s opinion, who has a different opinion, the money that we use to live in high** **lifestyles, where do we get it?**

PK04: As number 4, it depends with where you are working, sometimes the work is good sometimes the work is bad, sometimes you find someone who has money and he wants to give you a little money, and you have already known that this person has money, and you has already seen his M-Pesa pin, and I feel that tomorrow when I leave this place, I should buy myself a woofer, or go and buy my T.V, so I will keep this person close and when we go to the room, I will tell him that I will do anything that you will say I will do it for you, and when he sleeps deeply, I first test him and do this [wave my hand close to his eyes to see if he is seeing me], and when I see him not moving like this, you know that some, when you test them, you think that they are sleeping but (laughter), you think that he is sleeping and he is looking at you with one eye, a rabbit’s eye (laughter), so it seems like you are the one sleeping at the edge of the bed and he is sleeping the other side, he is testing you with his eye you know you are doing this, until you try pinching him , like babe, babe, babe and you see he is quiet, then okay, I wake up and take my cloth, I had known the pattern on his phone, (someone coughs), and I send how many thousands on my phone, and I also take the cash even how many thousands

**I: Five**

PK04: And I leave with ten thousand, I go easily and when I reach the gate, I give the soldier five hundred and I remain with nine thousand five hundred, have I not bought a T.V and put in the house, when you come to my house you as my fellow prostitute you ask, and the way it was hard you bought a T.V, I tell her leave it at that.

**I: That is number 4, someone else who has a different thought?**

PK09: As I want to, as number 9 as I want to support what number 4 has said, my fellows the work that we are doing is just skills, you must just use skills here, I always tell my children every day that there is knowledge that you are born with and there is knowledge that only the teacher can add you for you to be clever, so here, I just feel that it’s skills like I had said, before I also said that you can go and when you put a drug to someone, and they sleep, at the time when they sleep, and at the time when they are asleep, it is when you are going to take his money and it is what you are going to use to do those things, that you desired to have, and there’s also talking to someone well, there is one who even though you did not want to do bad to him, because we are also dying, wherever you are seeing us, we are dying, in this job that we have, we are being killed, because sometimes you have gone and stolen from someone who cannot be stolen from, you have stolen from him, and he cannot be stolen from, you can bark as a dog when he wants his money, you can bark and (laughs) sometimes you are barking and there is no one that wants to help you because you don’t know him and you just met yesterday, and you have stolen from him and you have parted ways, and wherever you can get him so that you stop barking, there’s nowhere, so there’s a way you can see someone’s heart and after you have seen that he is a good person, you sit down with him and tell him that, you wanted me to stay with you but it is only that I want you to do such a thing with me, and if you do that to me then we can be together, and he just does it to you.

**I: Okay, thank you, number 8 wants to add?**

PK08: Yes, as number 8 I can say that sometimes even if you don’t steal from someone but just talking. The rooms where we go to, sometimes someone tells you that I don’t want to sleep wherever you are taking me, let’s go and sleep somewhere else, then you go and sleep somewhere and you watch a T.V, and he says, put for me a certain channel, I like watching things like this, and he asks you, do you also like watching, and I say I to him that I don’t have a T.V in my house, you just talk to him well such that when tomorrow comes, the thing I told him that I don’t have a T.V in my house, there’s a way he is going to get it, such that tomorrow when he wants to leave then he tells me, take this and go and buy a T.V with it tomorrow, nowadays the world is digital and there’s no one without a T.V.

**I: Okay, and do you think sex workers always borrow money, do they have debts?**

PK01: Has she borrowed from the bank or from the Sacco or someone?

**I: Any where**

PK01: Yes

**I: Okay, that is number 1, they have debts after they borrow the money from where?**

PK01: Sometimes you might be a prostitute and you are in a certain Sacco, or even these merry-go-rounds for women, there’s a way you can even go there and take a loan (cross talk).

**I: Okay, Sacco, merry-go-round, meetings, are the meetings the merry-go-rounds?**

PK01: Yes, it’s the Chama

**I: Where else?**

P: Just the merry-go-round, the Micro-finance that lend people money (cross talk)

**I: Number 4?**

PK04: As number 4, we have merry-go-rounds, it may be hard for you, sometimes your child has been sent out of school and the job is also difficult, so you go to the merry-go-round and tell them my fellows, I want you to lend me even five hundred shillings, my child was sent out of school, so that I can go and pay, I will return it little by little, every week when we meet at the merry-go-round then I return it bit by bit, we must have debts.

**I: Number 1 has said Sacco, number 4 has said merry-go-round, number 3?**

PK03: Like me I am in a micro-finance where being loans deducted from people, I can go there and even take a school fee loan, I can take money, when you go there you are given any amount of money that you want.

**I: Micro-finance, number 5**

PK05: We must have debts, it depends with how the work is bad and it forces us to borrow vegetables from the vendors, and we can also borrow things from the shops.

**I: Okay, we can take vegetable debts from the vendors and we can take things from shops too, someone else again, you have said, what do you do with the money that you borrow from the Sacco?**

P: You can pay school fee, it may come a point when you want to pay rent, and it found when you are broke, so you can go and take this money and pay rent and you will return it, that way, when the baby is sick, you can go to that Sacco, help me, and other small things.

**I: And how do we get money to pay these debts at the payment time? I have lent at the Sacco, I have lent at the micro-finance, I have lent from the vendors, I have lent form the shops, I have lent from the merry-go-round, it has reached a time for payment, where is the money going to come from?**

PK01: Just from your normal work of sex work is where you can remove it.

PK08: I as number 8, I borrow money knowing that I am going to work.

**I: Okay.**

PK08: Yes, and when I come from work I do payments, they give me days, even if it is one week, in a week if I keep two hundred two hundred then their money is enough and I have given them.

**I: So, the money that is borrowed is paid with the money from work?**

PK08: Yes

**I: And when there is no client?**

PK08: The clients don’t miss.

PK09: As number 7, when I say, (cross talk), as number 7, when I say, number 9 (laughter), as number 9, as for me, someone like me, I don’t have one merry-go-round such that it can be said that this is the merry-go-round that I depend on, I have three merry-go-rounds, and I cannot have debts in all these merry-go-rounds, I can have two merry-go-rounds and when I see that this other one is going to take time before I get that one, then I go to another merry-go-round and borrow money and I clear this other merry-go-round, because I know that with mine, the work is still not good, so I play with them in these merry-go-rounds. I have three merry-go-rounds, after I borrow money from Kenya Women, then I know that there is money I am supposed to return every week, so I go to this other merry-go-round (mentions name), and take a loan and pay here, such that I know that after a week I am supposed to return it the other side, I again go to the merry-go-round and I borrow, that is how I can go round in merry-go-rounds up to the time when the work will be okay.

**I: Number 8 has said that when borrowing she knows she has a job, she will go to work and earn money** and come back and pay debts, number 9 says that she has merry-go-rounds, she can borrow money from this merry-go-round and go and pay debts for that merry-go-round and that is how the merry-go-rounds go round. Someone else again, numbePK010, where does the money for paying debts come from?

PK010: The money for paying debts just comes from work, you cannot borrow from someone and pay debts, it just forces you to go to work so that you can pay that debt

**I: And as a sex worker, as number 8 has said that we have work, when you leave you know that you are going to work right?**

P: Yes

**I: And sometimes you would like to increase your income there at work, so what does a sex worker do to increase her income? Is the question understood?**

P: Yes

**I: You want your income to**

P: Increase

**I: Increase as a sex worker, what is always done, what do you always do?**

PK04: As number 4, if I want to, I was recently getting two hundred, I now want to get five hundred, so I go when I am smart, I dressed up nicely, because I do not wear make-up, I am natural, I go, I have worn smart clothes, my top matches my shoes, I have dressed smartly, and when I see someone I just smile, and so when he comes to me, I get away from that two hundred, and I tell him that, just give me five hundred for a shot, you are my everyday customer, that, why don’t you just take the normal two hundred, and I say no, just look from my toe to my head, how do you see me, he says that you have said the truth, let me just go and give you that five hundred, let’s go so that you can give me what you are giving me so that I can go back and sleep at home.

**I: So you increase the price?**

PK04: Yes, so I increase the price, I come from two hundred and go to five hundred, and I will just increase that way until I even reach three thousand.

**I: Okay, that is number 4, number 1?**

PK01: As number 1, I can change the venue, because sometimes you are used to a place until everyone has known that it is the work she does what, she does, even if this beautiful lady said that, number

**I: Number 7.**

PK01: Number 7 said that, she is rarely in =Kisumu=, she always travels, I can change the venue, so that I can travel and go somewhere else, where I am new, you know that when you go somewhere and you are a visitor, then you must have a high value, so you will find that you have a high value, such that you find that you have a good earning, that place too, if you see that the value will go down, you had already disappeared in =Kisumu=, when you come back you are new again, you catch again[ get new catch].

**I: That is number 1, number 8?**

PK08: As number 8, sometimes there’s your fellow that you see that her income is very high until you ask her, so and so, can you tell me how you do it, and she will tell you that, I use drugs, so let me take you to a place so that you take drugs, so you do this and this, when that person comes, you do to him this and this so that he sees only you, and truly if she takes you to that place and gives you that drug, you can be with a customer the whole of that week until you ask, is God working or it is the drug that is working,

**I: So there’s also a drug that you can use so that you get a lot of customers?**

PK08: Yes

**I: We are doing well, we are not so far from finishing, (movements) number 3?**

PK03: If I support number 1, changing the venue also makes someone have a lot of clients, because you know that at that time you have changed the venue, you go and find, the new people see faces that they have never seen, men also like new things, they like these new catch, but when you get to the venue where you are new, you must be free with the ladies who you get there, because it may not be good to you, you may go there and take their customers who they are used to, so you just be free with them, sex workers also love each other, you just be free with them and say, I have just come to search here, they must give you a chance and I know that if you take a week there you will have some little change because you have gone to a new place.

**I: So number 3 says that sometimes you might go to a new venue, and an injury risk can occur because you are spoiling the market, right?**

P: Yes

**I: Any other risks that may occur at the time when we are looking to increase our customers so that the income becomes high, what else are they? You can be beaten at a place where you are a visitor, another one?**

P: You can be killed.

**I: Yes, you can be killed, where you are a visitor or?**

P: Yes, where I am a visitor.

**I: What of adding the price?**

PK04: On adding the price, as number 4, when you go to a place where you are a visitor, the old sex workers can gang up and beat you, and do something bad to you, they are going to feel that you will grab their customers.

**I: Okay.**

PK04: Yes

**I: Do sex workers borrow money, like here, how much money can it averagely be, number 1? How much can the money you lend be?**

PK01: You borrow according to your, problem that was at hand.

**I: Exactly, but how much money can it always be averagely?**

PK01: You may even borrow five thousand

**I: Five thousand?**

PK01: Yes

**I: And number 2?**

PK02: As I support number 1, it will depend with the things that you want to do because sometimes it is five thousand, sometimes it is four thousand so it will force you to borrow.

**I: Number 10?**

PK010: In borrowing you cannot say that I usually borrow such an amount, because sometimes I might be borrowing two thousand and I have a problem that exceeds that two thousand, it will force me to borrow five thousand or six thousand, as long as I can pay.

**I: Okay.**

PK010: Yes

**I: And the idea of stopping sex work, does it cross your mind, does it cross sex workers’ minds, number 3?**

PK03: Like me, one day, it crossed my mind, the reason why it crossed my mind, I found a man, the man is so handsome, a very handsome man, so after going with him and reaching the place where we are to sleep, then he tells that I should give him the number so that he will send me the money, (laughter) so you know he put me into a fight because a person that you are not fond of that is not your husband, you know you don’t know his capability, you know that for your husband you can try his capability and know that, my husband is weak or not, so that is a person who I don’t know his capability, and he put me into a fight, and I persevered, it’s always said that you persevere because you don’t know this man’s thoughts, he said to me, give me your number, he had really worked, then he said, give me your number so that I will send you the money, I told him that, do you think I have come to do your work here, you will give it to me, so what I did, I worked in a very simple way such that she didn’t know that I could do that, I told her please, I don’t have money and I have a sick person at home, and don’t think that I came here, I went to a new place, that is why I said that there is always a challenge when you go to a new place, and my fellow woman that was there that I could tell, I was not used to her, so when I reached there and he told me that, then I asked myself, this person is telling me that and the way he has worked on me this is now difficult, so I told him, what you should do, I left my bag, I only took my phone, my phone, I took what I knew had a great value, then I kept it, and told him, can you lend me your phone a little bit, I am calling someone because I don’t even have fare to go back, it forced me to escape with the phone, so that makes it hard, because when I escaped with the phone he even tracked it, after tracking me (laughs), when he got me I also told those people that it was not my wish to take money, it forced me to be free, and that is why I was set free, it was not my wish to take the phone, haven’t you seen that the phone was on and I didn’t even switch it off, (someone coughs) I just wanted him to give me my right, he did this and this to me and he did not give me money, so what I could take from him was this one, let him go and bring my bag wherever he left it and give it to me and I will just give him the phone, I was understood even by the police, he was told, pay this woman her money, because you went to have sex with this woman and you leave her without money (laughter), so that also happens.

**I: That is number 3, do sex workers have the thought to stop?**

PK08: Yes, as number 8 I just support number 3, sometimes you might get tired, sometimes you might find someone who lies to you from morning to evening he has lied to you, and when he leaves he tells you that he is going to send you money on your phone, and when you have a good heart you accept and say, yes, go and send it to me, and when you call him his wife is the one receiving his call, what do you want to tell his wife, don’t you just cry and pray to God that, God will I one day leave this job and have another one that is different from this one, so there are a lot of challenges in this job of ours.

**I: That is number (cross talk)**

PK01: Number 1, you may feel like stopping it, and you can cry, it’s not that you feel like stopping it and you are quiet, you feel like stopping it and you also go and cry at a place where you are not seen, and when you come back (laughs) you are quiet like someone who was not crying, and truly you can find a lover, and you find yourselves at a beautiful place and you take him home, he has slept on your bed, he ate your food, you know you are welcoming him well, and he has eaten well and you have served him and you have treated him well, and you have a T.V and he has watched until 12 ,11p.m, and he is also listening to the woofer by your side, he sleeps at peace, and when he will be leaving in the morning, (laughter) he tells you, madam, I have nothing, but there’s money that I am going to withdraw from the bank, and I am going to withdraw right now, two hours and I am sending you the money (laughter), my fellow woman you can wait until you ask yourself , were all these banks closed, such that there was not even one that someone could withdraw money from, it had happened to me, I felt like going to cry in the toilet, and I was approaching rent payment (interruption)

P: For that the shoes remains

PK01: And I didn’t think of holding his phone, because the way he dressed already and how he looked, had showed me that this man just has money, and that is why I took him and treated him that way, I cooked for him and even treated him with my money, because I imagined that what I am going to get from this person, I should just cook beef wet fry with cabbage, because whatever I am going to get here is big, (laughter) I felt like leaving that work.

**I: That is number 1, number 4?**

PK04: As number 4, that thing also happened to me, I went to =Eldoret= to a certain club called =Western=, so after reaching the club, I got my fellow sex workers there, they welcomed me, they were Kalenjins and they were mixed, so my fellow Luo lady did not like me but these Kalenjins became my friends just at the time when I reached there, so when I went there I interacted with someone, he came and bought alcohol there, when he stands up to go and ease himself, I pour it under the chair, he buys again, when he stands up to go and ease himself I pour mine under the chair, I just drink water, so it reached a time when we were negotiating, we negotiated on the price, three thousand shillings because I was also calculating the fare that I used from =Kisumu= to =Eldoret=, to and fro, so when I went with this person to the room, this person went and dealt with me ruthlessly , I told him to pay me upfront and he refused, I will give you money tomorrow, trust me, when it reached morning at six o’clock, so I wanted to leave early with the morning vehicle so that I could go back and rest, this man told me that, beautiful lady , just go, just go and I am going to send you the money, and he tells me, let’s go so that I can pay for your bus ticket, this man had notes arranged in his wallet, and he paid one thousand and he was given change, and he tells me just go, I am sending you money to your phone, just go, when you reach =Kisumu=, I know that in two hours or one hour and more, you will reach =Kisumu=, when I reached =Kisumu= and came to the house, I was still quiet and did not call him (birds chirping), and when it reached something like twelve noon (birds chirping), it was about four hours, so when I was calling this man, this man told me where I did you purchased that thing, where did you purchase that thing (someone coughs), I told him, send me my money, the vaginal debt is hard to claim, send me my money the vaginal debt is hard to claim, send me my money, I kept on calling him, kept on calling him, kept on calling him, then the last time it was now number busy (interruption).

P: I can’t leave =Kisumu= to go and go engage in sex for free somewhere else (laughs)

PK04: I cried, (laughter) I cried, my child was asking me, mom what are you crying, my head is hurting, can I bring you water so that you can put on your head, the child brings me water and a cloth, and he doesn’t know what I am crying (laughter)

**I: So the things we are going through, sometimes makes us feel like stopping (cross talk)**

P: To stop.

**I: To stop the things that, number 8 has an issue?**

PK08: Yes, I also have something to add on number 4’s issue, one day when I had gone to work well, and a vehicle stopped in front of me, and that man was telling me that he cannot go and sleep at those places, that we should go to his house here in =Kisumu=, I truly went with him but I did not dress up the way I would dress during the day, I dressed up in my job attire, truly I went and cooked at his house and we ate, when the next day came, I realized that it was morning, and there is no way I could walk with that dress in front of people, and he tells me that he has no fuel in his car, so he escorts you walking, after reaching the road he tells you, stand here at the road so that I withdraw money, no, I have forgotten the money at home, I am going to get it, the way he went to get the money at home, you also don’t know the way that you used because you are a visitor, and you have worn a dress that anyone that passes must turn when looking at you, you can cry in front of people, you sit in the vehicle, sometimes you had a little money, you are sitting in the vehicle and you are the one your neighbor (in the vehicle) is looking at your dress that you have worn, when you alight, you are the one people are looking at, when you reach home you will just remove that dress and will you not just cry and say, God will you one day get me out of this job, it is not easy .

**I: Yes, and the places where we work, don’t we have our hot spots?**

P: Yes.

**I: Does such discussions arise where work is done too?**

P: Yes.

**I: Yes, number 3.**

PK03:Such discussions as number 3, may occur, but you know that at that place, we are busy, because it’s hard to find a lot of time because sometimes you sit for a shot while you have already seen your client coming, and I won’t stay calm while making stories with you on how I passed through hard times, I am going to receive my partner that I am going to meet, so our discussion is always short, so if that discussion can be there then we can really improve because now someone explains the problem that she is passing through, so that we can stay knowing that, a sex worker, I carry money on my hands is when I get horny, I carry money on my hands like this is when work commences, but when I haven’t seen the money, I don’t have appetite, so that I don’t go through problems that may make me want to stop this job.

**I: Even if you leave wherever you are working, places where you meet, do you always make stories and talk about stopping sex work, number 9?**

PK09: As number 9 as I want to say that, (birds chirping) most of the times as we are, we don’t work at the same place, so we know each other as we are that this work that I am doing is what my fellow is also doing, but the area where we are living, there’s no one that knows the work that we are doing, so the time when we will meet, (birds chirping) we are just going to, we can only meet there at the field, and at that place there’s no time for us to make stories (birds chirping).

**I: Okay**

PK09: Yes, because we stay in different places, is what hinders us to.

PK01:If I may add on that, when you are doing this job it is supposed to be, like we are here, you have a friend who you are close, your fellow woman like us, you look for one person that you trust and she trusts you, that you become close to because the places where we go you may have a problem, but you must have your fellows number such that you can call and say, I am in such a place and I have encountered such a problem, and your fellow may find a way of coming to look for you wherever you are, so you find that you have someone that you are close to, and you go and share with her your problem, even during the day when you have woken up, because you go to work at night, and you go and bring your friend and you tell her, I went through this and this, and she can advise you (birds chirping).

**I: That is number 1, number 4 (cross talk)**

PK04: As number 4, I support what number 1 has said, for me inside here, there’s one of us who when I go, when I travel, I always travel up to =Mombasa=, when I travel far, I tell her, I tell her that, (birds chirping) today I feel that your =Kisumu= has bored me, I want to travel and go to another town, the reason why I am telling her is because I don’t know what I am going to meet there, so that in case the next day she calls me and she can’t reach me, then she must be worried and she can even take her transport to come and look for me that, what has happened.

**I: Okay, so you have told me what can bother you until you feel, I am tired with this job, (birds chirping), and (cars hooting), to those who have left, there are people who you know that have left, or to you people who have the thought of quitting the quitting, is it something that someone plans that, for me if it reaches 2023 on the second month, then I am quitting it, or it is something that someone just comes unexpectedly, number 3?**

PK03: Like me I have two girls that have stopped, they were really my friends, and they have stopped, and they just stopped unexpectedly, they didn’t plan, there’s someone who just stopped depending on how these men, behave to people, so it forced her to stop, and God had mercy on her and she became saved, she joined God’s work, and she got married and she is married and has children, so all along when she sees me she tells me, if God has still taken care of your life [ mentions her name], number 3, then stop this work and go back to God because this job is hard (someone coughs) and there’s nowhere it will take you, it is something with a lot of challenges, ever since I went back to my home, even the person who married me, I told him how I was and the things that I was doing, when I was saved and joined God’s work, I stopped that work and right now I am continuing with my life and doing business, so it is something that someone stops abruptly, she doesn’t plan.

**I: Okay, number 8?**

PK08: As number 8, quitting is also there, but there is a friend of mine, we are working, we used to work with her, she left and went, we had known that she had stopped work because she took five years without coming to work, we knew she had stopped because we even lost her number, so one day we unexpectedly saw her come back, that she had come to work, so leaving that job is also difficult.

PK04: As number 4, there is a friend of mine, she is the one who introduced me to this job, and taught me how to do everything, so there is a time she went with someone and stole from him, you just know this work for sex workers, if she sees that you have money there’s a lot of greed more that the money that you had agreed on, and she had stolen from this man and he was a Kamba, he turned her mad and when she was cured, (someone sneezes) she got saved and she stopped that job until now, so right now she is just doing her business. (Someone coughs)

**I: And number 8 talked about someone who stopped and came back, why would something like that happen in that someone stops and comes back again?**

PK01: Sometimes as number 1 you might stop while thinking that you have found someone and he has married you, until you have agreed with this man and you have stayed, later you will find that he doesn’t solve your problems, later on you find that you were good staying alone better that when you are with him, problems start coming up, your children don’t feed well now, he now knows that he has put you in the house, he leaves in the morning and gives you a hundred shillings, and you know that you used to leave and when you left you came back with one thousand shillings or five hundred shillings or some money that is different from that one, so you just feel like, this person is disturbing me, let me go back to my previous job (birds chirping).

**I: That is number 1, someone else, what can happen for a person to stop and then come back again?**

PK04: As number 4 as I support number 1, the reason why someone can stop that job and then come back again, sometimes she has found someone who has married her, they have stayed, so this person comes back and changes his mind, so she just says, let me just go back to the work that I was doing if this man has changed his mind, my life will still go on like it used to before, so it will force her to just go back to the sex work job.

**I: So after they come back, number 9?**

PK09:As number 9, it also depends with, as we had said earlier that there’s no parent that allows their child to do such a job, so it also depends with where your parent is, and sometimes your parent is harsh, and h/she comes and hears that that is the job that you do in town, h/she can get you out, like me, I had done it and stopped again, such that right now I just came back, I just came back, and I came back because, when my mother died I was left in the hands of my uncle, and my uncle was a police officer, and he was very harsh, such that even if he got you with even a dress, that you have put on a dress that was not acceptable, he doesn’t cane you, he doesn’t beat you himself, he takes you inside and you go and get beaten with those who are strong, and he says that you are stronger than him, so you get beaten by those who are strong, so it made me stop, I stopped because of the harshness that was shown to me, that is why I stopped, and when my uncle died it’s when I said, isn’t it the person who was harsh to me is who God has taken, I should just go back to my work and find what I can do, how I can survive with the children.

**I: The bad things that happen to someone when she comes back, what are they, when you had stopped and then came back again, what bad thing can happen to you, number 8, we are almost finishing? (Cross talk)**

PK08: When you come back, when you come back the way my friend came back, you find when her customers left her and we are the ones who are now with them, do you know that it brings chaos, between me and her, and she has come not the same way as she was, she has come back with changes in her body, she has reduced, and when she left she had a good body, such that even if the customers see her, they cannot accept that it is her, so you know that chaos must erupt, we fight with her there, we quarrel with her there.

**I: Another thing that can happen to someone when they have stopped and come back again?**

PK09: As I support number 8 on that issue, if it is good, it is supposed to be that if you left and came back, you are not supposed to go back where you left, if you know that, if you know that your body has, if your body has reduced, if you are skinny in Dholuo, then don’t go back to where you were before, because all the customers are not there, someone only loves you when, these people only love, the customers only love you when you are glowing and look beautiful, and they are the ones who are infecting us with the death, and when we have died (not attractive), then he feels (laughter) that you are sick, and he runs and leaves you, and he is the one who infected you with the disease, and he runs and leaves you, so for me I was in =Bondo= before, and when I came back to the line-up I came back to =Kisumu=, I changed the county, I am in =Kisumu=, but I can always go to =Bondo= once in a while, because you get used to wherever you stayed, I can always go once in a while because I changed my estate.

**I: That is number 9, number 7, what bad thing can happen to someone when she stopped sex work and she comes back again (noise)?**

PK07: When she comes back she might have lost a lot of customers (something bangs), then when she doesn’t get good teachings from her fellows, in that she goes to her fellows for teachings, then she can get sick.

**I: And what are the good things that can happen to someone when she stops sex work and she comes back again, (birds chirping) number 3?**

PK03: The benefits that if someone leaves, she comes and gets them again, because at the time when you left it there was a reason why you left it, so sometimes you might come back when you are cautious, you might come back when you are cautious such that you go when you know that, what has brought me back is this and this and this, and let me not just go back there that people are going, so someone who comes back, most of them come back when cautious, they don’t just come back.

**I: Number 5, benefit that someone who has left sex work goes and comes and gets, what is it? (Someone speaks in low tone) Number 5 is still thinking, number 10?**

PK010: The benefit that when she leaves and comes back, sometimes when she was leaving she washed someone (stole from someone) until it brought her down, so when she comes back she comes back to do what brought her, even if she gets fifty bob, that is what she goes home with.

**I: That is number 10, number 2?**

PK02: I am still thinking.

**I: Number 2 is still thinking, and these people who stop sex work, is there an age that when they reach is when they stop it or the age is not that a must, number 8?**

PK08: As number 8, I can say that the age is not that a must, that it is a must that you reach a certain age is when you stop, you can stop according to the problems that you go through, it makes you demoralize and leave /quit.

**I: And what’s the age that most of the people who stop, number 4?**

PK04: As number 4, the people that stop, like me now, I have an old child, a form two, so you know that as this child continues to grow, she will ask me, mother, what do you always do, such that when night comes you are out, and in the morning you come back. So it will force me to now stop this thing because my people are now old, I can get, and sometimes I can get a way of finding support from them so it will force me to stop that work.

**I: Okay**

PK04: So that they can even start a business for me to do, they have bought a land and even built a home for me so it will force me to stop that job.

**I: And what I would like to know, that these people who are stopping, you have said that age is not, number 8 has said that age doesn’t determine that someone should stop, but most of the people that stop, at what age do they stop, when she has reached what age, number 3?**

PK03:Most of the people who stop sex work are from forty years of age, people stop at forty because if you are someone that started giving birth at twenty to twenty two years, your children are also growing old, so every day at night this mother is not there, every day so even the time to explain to the child, do you go to night vigils every day because you know that a time reaches when she even lies to the children that, my fellow merry-go-round colleague, my friend, so this thing happens every day, so there’s this shame that the parent feels depending on her age and how her children are growing old, a female parent fears her children, she cannot do those things openly such that even when the children have reached a certain age she still goes out at night and sleeps, so at the age of forty, someone feels like stopping that work.

**I: Number 6, at what age do sex workers mostly stop that work?**

PK06: She can stop even when she is forty-five or fifty (birds chirping).

**I: Number 1?**

PK01: This thing is not always a matter age because sometimes you may be forty years and you gave birth late, sometimes you are forty-five and you did not give birth, so it just depends with how someone feels, because sometimes you can be forty-five, that’s when your sexual urge is high you just look for these young people, so you might be fifty, and you cannot still leave it, so if you can just leave it at sixty, then it is only good with forty something but it depends with someone’s heart.

**I: Okay.**

PK01: Yes.

**I: And these people that have left it, what kinds of jobs do they do, because you cannot quit and do nothing, there are still needs? So the sex workers that have stopped the sex work job, what kinds of jobs do they do?**

PK04: As number 4, I can start a hotel, I can sell kale's at my stall the side of the road, (birds chirping) and I sell kale's and get my money.

**I: Number 5?**

PK05: It depends, if you know how to plait like she had said, you can quit and open your salon or purchase mitumba clothes and sell around. (Noise)

**I: Number 10?**

PK010: I can support number 5 that you can stop it even purchase clothes and sell and get something every day for yourself.

**I: And where do the everyday sales, the people that do it? (Birds chirping)**

PK010: So it depends on where you are, you can even do it here in the community, you can even go and do it in town.

**I: Okay (birds chirping).**

PK010: Yes

**I: Number 9, (birds chirping) what kinds of jobs does someone who has stopped sex work do, wand** **where does she do them?**

PK09: In my opinion as number 9, I think that if it is good, someone who has stopped doing this job should be selling clothes

**I: She should be selling clothes?**

PK09: If it is good.

**I: Where should she sell them?**

PK09: If it is good she should sell it, it depends with (someone coughs), I just think that she should just go back and sell clothes wherever she was working, because that is where she is known, she is known at the place where she is working very well, such that when I bring you a cloth that you are going to stand with (standing while looking for customers as sex workers) then I know that when I take this cloth to so and so, then it is going to please her, because I know the work she is doing.

**I: Okay**

PK09: I just think that way

**I: There are people that have set their minds that they want to stop right?**

P: Yes

**I: But they feel that there is still one two three that they feel they are supposed to do is when they can stop, so these one two three, what are they such that someone can say, for me I will stop, but I want this and this and this to be accomplished. What is always this and this and this (birds chirping), you say faster and we pass, we are almost finishing.**

PK09: As number 9, I am doing this job because I want to buy my children land.

**I: Buying land.**

PK06: As number 6, I want to educate my children so that they can finish school.

PK04: As number 4, the hope that I have with this job, I want to work hard so that I can get a place so that one day my children can build there.

**I: Buying a land, number 1?**

PK01: I work very hard so that one day I can get my place, you that here in the field is where marriage is found, you may meet the right person, and he falls in love with you, so my prayer is that even if I work, then one day I should get a person who God has accepted, so that he can put me somewhere where I can build.

**I: So that you get married?**

PK01: Yes

**I: Number 2?**

PK02: In my opinion, I do this job so that one day if I stop it, I can find my place such that even (birds chirping) if I get a partner and stay with even if I get old, then I also have my business, that is why that saving is good.

**I: The things that we want to accomplish, have we started planning? Have you started planning to buy land number 9?**

PK09: As number 9, I have hope with it and I have started planning.

**I: What plans?**

PK09: How I am planning, that is why I have joined merry-go-rounds.

**I: Okay**

PK09: Yes, because I feel that when I get my money, then I should take a loan, then when I find a good land then I should take a loan and go and pay because a loan is something that is paid little by little, so when I have paid for that land, (birds chirping), then I can now pay for that loan

**I: That is number 9, number 6, what plan do you have so that whatever you are desiring should be accomplished?**

PK06: The plan that I have, I just feel like I should just work so that I can know how to save money, for my kids, I save in the bank so that one day, it may help me so that I can start my business while educating my children.

I: Okay, that’s on getting a land, that’s on buying a land, and on getting your own place, what do you **plan?**

PK06: On getting my place is just prayers, yes, you work for it and you pray to God, when one day I find someone with a good heart who wants a wife, because I can say that I want to save money to buy a land and I fail to buy it, but if I find that person that has a good heart that God has brought to me, you know that whatever you ask God is what He gives you, then that person gives me a land and I build on it.

**I: You had counted to me some of your friends that you know that stopped this work right?**

P: Yes

**I: And the things that made them stop, some of them, (birds chirping) is there something that you may** **add on what makes it easy for a sex worker to stop sex work? What makes, number 9?**

PK09: As number 9, (birds chirping) there are some men who can pick you and tell you that he is taking you to his house, like me I have a friend of mine who stopped this work, she stopped such that, she stopped such that if you go and tell her, my friend let’s go back to it, then she won’t accept, because there’s a customer that took her and told her that they were not going to the room, that he has a house that they are going to, so when he took her to his house around =Migosi=, and she told me that my sister when I reached that place the man asked me do you want money, and she said yes, tell me how much money you want, and when she looked at that man she saw that he had money, then she told him that, I want is, if we sleep up to morning then you will give me three thousand shillings, and that man told her I will add you two thousand so that it becomes five thousand, but it is only that I don’t want to sleep with you, I want to give you a job, and she accepted and said that all she wanted was a job if only it could give her money, and it was night, at =Lolwe= so she was taken to a room, after being taken to a room, she told me that when that person removed his clothes, that man had a big sore besides his tummy, a disgusting sore that she has never seen, then that person pointed the gun at her and told her, lick it, lick it and if you don’t do it then I am killing you, so she was also doing it because she didn’t want to die and leave her children young, she licked the sore, and when she left that place she came and told me that “I”, number 9, today I came from that place, and she was given thirty seven thousand shillings, that’s what she was given, so after coming with the thirty seven thousand shillings, that is how she stopped, she stopped because of what she saw, that is what discouraged her, such that when you tell her let’s go, she just vomits, it is when she has started vomiting depending on what she had licked, it made her loose interest, she can’t go.

**I: Okay.**

P: If I may also add, I had a friend that had gone to work, after going to work, the reason why she stopped, she found someone and that person told her that he was going with her and he was going to give her four hundred shillings, then the person booked a room for one thousand five hundred, then the gate man insisted, just go with this man, you are going to negotiate the price there, and after that person has told me that he is going to give me only four hundred, and the guest (guest house) is one thousand five hundred, how is he going to give me four hundred shillings and the guest is one thousand five hundred shillings, so she got angry and she didn’t go, (someone coughs) and she said no, leave him, let him go and find someone to sleep with because he is going to give me four hundred and he is going to pay one thousand five hundred for the guest house, that is how she gave up and stopped.

**I: Okay.**

P: Yes

**I: So the way I had told you about Jitegemee, this is little we have finished, this is very little, I can see that our faces show a lot of tiredness, just forgive me, these questions are many, we are almost finishing. I had told you what Jitegemee is, and its aim is to ensure that sex workers have saved, so that it can reduce their chances of getting HIV, okay?**

P: Yes

**I: They can say no to unsafe sex because she has saved. If someone doesn’t want to use a condom you can say, no, let us just stop it politely, I know how I have taken care of myself, or you are tired or sick, you can take a leave for one week, you just raise your leg on the other, you are watching T.V because you have your savings, you are tired, you want to rest, that only happens because you have save. So the things that I had explained to you about, on Jitegemee, do you think it is something that sex workers all over Kenya may like? Number 2, which kinds of sex workers can like Jitegemee, (someone coughs) number 3?**

PK03: As number 3, the sex workers that can like Jitegemee are sex workers that are trained the way you have taught us today. I can, I have known and I am going to leave this place knowing that I have to save money such that when I know that I want to relax my life or mind or even my body, then I save, so if you are a sex worker that has been trained the way you have done to us, (someone coughs), you can depend on yourself.

**I: Okay**

PK03: You save.

**I: And who are the sex workers cannot welcome Jitegemee, number 8, who are the sex workers that cannot welcome Jitegemee?**

PK08: As number 8, I feel that those who can’t welcome Jitegemee are those who do not know about savings, who have not sat down and thought that she is supposed to save.

**I: Okay, number 7, is Jitegemee something that sex workers can welcome, and which kind of sex workers are they?**

PK07: As number 7, this Jitegemee, how sex workers can welcome it, depending on someone that does not save, it is good with her, plus myself that has a plan of buying a land, that saving is suitable with me, I save my money until it is enough to buy a land.

**I: Okay, and when you have your ten friends number 4, and you tell them about Jitegemee, from these** **ten people, how many are going to agree to, are going to accept jitegemee?**

PK07: Three people can accept, the rest cannot accept.

**I: Why would these seven people not accept?**

PK07: Because they are used to getting and using everything, so when I told her, save even a little bit, even if you get a hundred, save even five and use ninety-five, then she will feel that it is hard for her because she is used to using the whole of it.

**I: Okay, someone else, number 4 says that three people are going to accept, number 1, how many people are going to accept?**

PK01: Many people can agree depending on how you have delivered the report to them, the teaching that you want to give them on Jitegemee, the way you want to tell them how those things are done, there are people who can join, there are people who are going to can refuse.

**I: Out of ten, how many people can accept?**

PK01: Depending on how you have taught them, then even seven.

**I: Seven, why can the remaining three refuse?**

PK01: The remaining three can refuse depending on earnings/income

**I: Depending on earnings?**

PK01: Yes

**I: Number 2, out of ten, how many people are going to welcome Jitegemee if you tell them about it?**

PK02: As number 2, it depends with on how an individual feel, you can be ten people, but like you have now educated us, we are ten people here, but there’s someone that can get out with that knowledge that, I have heard whatever I was taught here, and I have seen that this saving is good to me, so it depends wit how someone feels, so in ten people you may find the five people have come out with that knowledge.

**I: Okay, number 10, if you have ten people who are your friends, you have talked to them about Jitegemee, how many people will accept Jitegemee (background noise)?**

PK010: Out of ten people?

**I: Yes.**

PK010: People can accept it yes, but someone might accept but lack earnings that she can save.

**I: Out of ten people, how many people will accept it?**

PK010: Even five people can accept it.

**I: Five people will accept, five people will not accept?**

PK010: Because sometimes she might not get the earnings.

**I: Okay, and us as people who are making Jitegemee, how can we do it, what can we do so that people may continue accepting it, number 8?**

PK08: As number 8 I can only say that, the way you have educated us and we have listened, is the way you can teach other people, even us we can educate other people until they listen.

**I: Okay, someone else?**

PK04: As number 4, I have friends at the field (work), I can ask her, a woman from Europe (=Kisumu=), when you get out of here with a thousand shillings, do you always save even a little bit, or use five and save five, no I don’t save, so I tell her that from now, start saving, it will help you even if you have a problem or when there’s no work, it can help you, so you can try talking to people who you work with.

**I: When sex workers see Jitegemee, how should it entails or what and what can the sex workers like? Number 3, what is it that if put in Jitegemee then sex workers can like it?**

PK03: What sex workers can like when put, sex workers, depending on, we must have group discussions, how we had one here, you can even sit with your friend and you tell her so that the sex worker can listen, I am one of her fellow sex worker, if I teach her how to save, the benefits of savings, what this will benefit her with, it may make her listen faster depending on the education that I have received.

**I: Group discussion?**

PK03: Yes

**I: Another one, something that if put in Jitegemee then a sex worker will like, number 8?**

PK08: I can only support that number 3, when we are in a group, when we form a group then sex workers can like.

**I: What can we do in this group after we have made it?**

PK08: After making this group it is just for discussing our issues and keeping our money that we are saving, and we open an account.

**I: Okay, and anyone else who has a different opinion?**

P: The Jitegemee group can help sex workers with an idea such that even if they can be given some loan and they can see what they can do and they can make their group, so after making their group then they have a group chairlady, and they ask, what can we do and how much money do we need in our group, and I think many people can like that if Jitegemee can help sex workers with something like that.

I: And what would sex workers not like in Jitegemee, the things that I explained to you, do you see **anything that sex workers may not like?**

PK01: All the things you explained to us are good, in my opinion, I like all of them.

**I: That is number 1, someone else, what sex workers may not like, number 8?**

PK08: I also support number 1, on my side I like all of them, there’s none that I can say is bad.

**I: Number 4?**

PK04: I can continue supporting number 1, you have brought us good teachings, and you have given us a wise teaching, the way we used to do sex work and we didn’t know, so right now we know the way we are supposed to follow.

**I: And the things that I had explained to you, have you seen anywhere that this Jitegemee can interfere with someone’s rights?**

PK04: No, all those things are good

**I: There’s none, that’s number 4 saying there is none, number 3?**

PK03: There is none

**I: There’s none, number 1?**

PK01: There’s none.

**I: Number 7?**

PK07: There’s none.

**I: Number 8 here’s none, number 6?**

PK06: There’s none.

**I: There’s none, and if we start it and it’s something that has started working, when we were reading the first page we were being told that it is, it is, thoughts are still being gathered, it is something that is yet to be started, and if it starts well, what challenges can we have as people who want to start it? Number 3?**

PK03: As people who want to start savings, the challenges that we must face are not over spending, the spending a little that we were saying, I must try to squeeze my budget, my needs that are high, I must reduce them so that I can start saving.

**I: That is to the people who are going to save.**

PK03: That is to the people who are going to do it.

**I: And to us who are supposed to help you save, what challenges can we face, number 4?**

PK04: The way I see it, as number 4, (noise) if you want to save then you deny yourself, so if you were using five hundred, you now change your budget, you use three hundred, so that you may find a way of doing savings.

**I: Okay, (music in the background) the challenge that we might face, number 2 seemed to be having an issue (background noise). Like I had told you, Jitegemee doesn’t have a target like a merry-go-round, it is money that you can offer, that you have the ability to offer, you can withdraw it any time that you want, it has no profit that if you take, even if you had two thousand and you take one thousand that you can now return it with profit, no, it won’t be that way, so if it is the way I have explained it, who, I want all of you to tell me this, number 1, how much can you save in a week?**

PK01: In a week, if the work is good then I can save one thousand.

**I: One thousand that is number 1, number 2?**

PK02: It depends with work, if it is good I can save one thousand

**I: Number 10?**

PK010: Eight hundred

**I: Eight hundred, number 4?**

PK04: I can save five hundred

**I: Number 5?**

PK05: Even fifteen hundred if the work is good.

**I: Number 3?**

PK03: I can save one thousand.

**I: Number 9?**

PK09: When the work is good then I save one thousand.

**I: Number 7?**

PK07: When the work is good then I save one thousand

**I: Number 6?**

PK06: I can save two thousand.

**I: And where do we keep this money, number 1, where would you like to keep this money?**

P: I can keep it in M-Pesa.

**I: M-Pesa, number 2?**

PK02: Just M-Pesa.

**I: M-Pesa, number 10?**

PK010: I keep it in the house.

**I: In the house, number 4?**

PK04: M-Shwari, number 5?

PK05: My money box in the house.

**I: Money box, number 3?**

PK03: I prefer a bank account, and I have mobile banking, such that incase of any urgency then I can withdraw it on the phone even at night

**I: Number 9?**

PK09: I keep it at my house.

**I: In the house, number 7?**

PK07: I save it in the M-Pesa.

**I: M-Pesa, number 6?**

PK06: The M-Pesa is enough.

**I: Number 8, the money that is saved weekly, where would you like to save it?**

PK08: In the house.

**I: In the house?**

PK08: Yes

**I: Okay, (movements) us as people who want to start Jitegemee, are there challenges that you face as people who want save at Jitegemee, right?**

P: Yes

**I: And the challenges for us as people who want to run Jitegemee, what can they be, the challenges, that we might face when we want to start, (music at the background) does anyone have any challenge, number 3?**

PK03: Just as we had said, you must have a challenge, because like me, I had tried to explain that I don’t like to do savings, so right now as you have taught me, then I must start doing the savings, so for me to save that money, it is going to force me to reduce my wants, so that is a challenge that is going to be there such that even if I had a problem and I have to use money then it will force me to say, no, I want to save two hundred per day,

**I: Is there anyone that wants to add something before we finish? I know that you are missing the clients, your work time had passed a long time ago, right?**

P: A long time, and we haven’t cooked for the children.

**I: Yes?**

PK03:What I want to add, I want to say thank you for the education that you have brought to us today, I am happy, as number 3 I am very happy, teachings like this, I always know that we are people who are stigmatized, such that even if a person knows the work that you are doing, most of the people usually put us in a life that, they see that we are out of fashion or we are people who are not supposed to exist, and the idea that you have had and gathered courage and reached us, has showed us that we have people who are supportive of us, such that in case we may have a problem then there are people who can support us, so I am happy (noise) for you to know how our lives are fairing, for you to know the challenges that we are passing through (noise), the problems that we go through when doing savings and how savings becomes a problem for us, so I am happy, continue teaching other sex workers too, so that they should have the knowledge that I have left with here today, that I am supposed to do the work of a sex worker, and also have savings.

**I: Someone else again, (noise) who may have anything that they can say before we close? (Noise)**

PK01: As number 1, I am happy with the teachings that you have brought, because I know that even the president knows that sex workers are always here in Kenya, so I am happy because we are well known. (Noise)

**I: Someone else who would like to say, the people that are leaving should not leave because there is something that you are supposed to sign?**

P: Yes

**I: Someone else who has something that you may add?**

PK08: As number 8 I say thank you for the teachings that you have brought us (noise).

**I: Is there any other or we can now close?**

P: We are fine.

**I: Should we close?**

P: Yes.

**I: Thank you very much, I know that a lot of your time has been wasted, you have missed your clients, but you have taken time and listened to the things that we were saying, we say a big thank you.**

P: We also thank you (all)

**END OF INTERVIEW.**
