# Supplementary material for: Eat a little and save a little: A qualitative exploration of acceptability of a potential savings intervention to reduce HIV risk among female sex workers in Western Kenya
Source: PLoS One. 2024 Dec 19;19(12):e0310540. doi: 10.1371/journal.pone.0310540 (PMC11658496; doi:10.1371/journal.pone.0310540)
Supplement: S1 File — (ZIP) [file pone.0310540.s001.zip › Jitegemee Transcripts and Dissemination Notes for Journal/FGD L.docx]

**FACILITATOR: PHILIP OWITI ODOTE**

**NAME OF TRANSCRIBER: ESTHER OMINA**

**NOTE TAKER: JUDITH AYALLO**

**FGD ID: FGD L**

**TIME INTERVIEW STARTED: 1511HRS**

**CATEGORY: BELOW 30 YEARS, PERI-URBAN**

**I: Aaah, am this is FGD L done at koyango and the time is aah3.11pm okay**

**R:** Yes

**I: According to my explanation about Jitegemee, what comes in your mind first? What comes in your mind? About this program? (Papers raffling at the background) What is it (papers raffling) place these here others can read. Start answering. The way I have told you what comes into your mind.**

**PL03:** What comes into my mind?

**I: Mention your number.**

**PL03:** Number three. What comes in my mind? The way we can help ourselves, our lives, our family in our future. And the way we can save to continue having savings in our future.

**I: Another person?**

**PL02:** Number two, what comes in our mind, the way we can have our saving and the way we can save. Until it reaches such a time that we can stop this job that we are doing and depend on our own

**I: Another person**

**PL04:** Number four

**I: Mmh**

**PL04:** The way we can get good jobs and help our families

**I: I want you to be audible for this thing to capture**

**PL04:** Yes

**I: Another person? Who have a different opinion?**

**PL06:** According to my opinion, I can say like this?

**I: Number? Number six**

**PL06:** Number six, we are tired of this work, we are praying that we get how we can help ourselves in life

**I: Mmh. So if we talk about jitegemee what comes in your mind?**

**PL06:** We can find how we can save to stop doing the kind of work we are engaging in, even if we get a small amount of money to save.

**I: Okay**

**PL06:** Eeeh

**I: Okay [music paying at the background] What do sex work buy with their money and how much does each of you spend?**

P: [Silence] [noise at the background]

**I: Have you understood that question?**

P: Repeat that question

**I: Those women who do sex work. They normally use their money, they use it to buy what?**

PL01: Number one, the costs we incur and the challenge we face is to buy those protection [condoms]. Because you can reach there, because where you have gone you may be told that they are not available. We also need to protect ourselves it forces you to sacrifice, you see, and we also come from far. So you find that you have used so much on fare and also get disappointed when you go there. At some point again the costs that we incur at some point you go and agree, then after you are through with what you are to do, after you have done the job, and you have done a good job, and when it comes to payment you find that you are paid less than what you used from your house. Those are the costs we incur.

**I: That is number one, she has told us how she spends her money. So those are the expenses she incurs, how do others spend their money? I know each one of you spend money, is it?**

P: Yes

PL02: Number two, you can agree to meet with someone and use your fare the way my fellow has stated. He again tells you to buy drinks and he will give it back at once (participant meant money). You use your money and after you have spent you find that he runs away so you are left alone mmh…

PL03: Number three, you can go there with a guy and agree then he refuses to give you your money. When you ask him, he starts to argue a lot and even beats you and leaves you bored. The way you can help yourself after being beaten, is something that is there.

**I: Okay, now I want… There are some basic thing people use normally.**

P: Yes

**I: On what do you spend your money normally?**

PL03: Our normal spending … number three, is for payment of rent, to feed our children and us and also our dressing

**I: Rent, feeding children and your dressing also. Number six, I have not heard your voice. There are things you normally spend your money on daily basis.**

PL06: Yes

**I: How do you spend your money?**

PL06: My money …..

**I: That is number six**

PL06: I use my money, I have children who depend on me. Sometimes I have earned some small amount that what I use to buy food, there is also school. Sometimes you have come back with some small amount maybe three hundred shillings, and it can’t even help you.

**I: Eeeh, number four**

PL04: [Inaudible]

**I: Be audible a little bit**

PL04: The money that I get, I use to pay school fees for children, I spend it on breakfast and also rent.

**I: Okay I want you to tell me what you spend money on a daily basis.**

PL01: Daily spending, okay, number one, daily spending, I normally use approximately I spend around 700-800 Kenya shilling because for transport you spend 100/=, trust [Condom] 100/=, drink150/= before you get in you are told by the client that you have to pay to get access to the club. For entrance they may even ask you to pay punch, that is entrance fee only

**I: What is punch?**

PL01: Five hundred bob, for entrance only. All that you and you have not gotten a client. If you get a client, he pays you less amount. If you calculate the whole cost, if you calculate the whole cost, approximately I can say it is not 800 shillings. I can say one thousand and above,

**I: Mmmh…**

PL01: Something of sort

**I: Number five, whenever you get money what do you normally buy?**

PL05: Me what I normally do is to pay school fees for my child and food and rent and also the clothes you wear when going there

**I: So how much do you spend daily?**

PL05: I spend let’s say 1000 daily

**I: So what do you buy with it**

PL05: I normally buy food daily let’s say I use fare from my house to where I go. Then also when I get in I will have to use money.

**I: Number seven, if you get money, how do you spend?**

PL07: My money I use to buy food, buy for my children what is required of them to have in school and the remaining amount, I save a little to ensure I pay rent.

**I: So, you must save and those are daily expenses? Are they?**

PL07: Yes

**I: And there is something that someone must buy weekly. Tell us your number**

PL01: Yeah [participants laughing] Number one, weekly, make up must be there, secondly clothes must be there because you cannot go there you are untidy that one you will not get clients, third shoes

**I: How much do you use on make up? In a week**

PL01: Weekly make up you use 500/= because you must take eye lashes, extra eye lashes, mascara ponds then that… There are so many things you blend, provided it comes out fancy.

**I: Mmmh. And how much do you spend on clothes and shoes?**

PL01: Clothes approximately 1500-1200 kenya shillings

**I: Okay. Who else buys on a weekly basis? That must buy something weekly?**

PL03: Number three, I also use make up. And sometimes if I’m called upon for outings weekly. I can even be invited to maybe Kericho I hve to use clothes.

**I: Mmmh**

PL03: Of course clothes must be new, shoes and makeup

**I: So how much do you spend weekly**

PL03: Almost twelve [1200]

**I: Twelve hundred**

PL03: Yes

**I: Okay number seven, what do you buy weekly**

PL07: in a week I normally change my hairstyle

**I: Mmmh**

PL07: I braid and undo when it is tough (participant meant when she has no money) I just re-do what I had braided previously though that way you may not get a client the way you should.

**I: Okay. How much do you spend on braiding your hair?**

PL07: To braid hair?

**I: Yes**

PL07: Okay sometimes 1500 kenya shilling it depends on the style

**I: Mmh**

PL07: When it’s low it goes for 600 shillings the cheapest is 600 shillings

**I: Six hundred**

PL07: Yes

**I: Number two, you are quiet [participants laughing]**

PL02: Am listening because what they are saying normally happens to me and all are true

**I: And what do you buy on a weekly basis?**

PL02: Just those ones, I must braid my hair, must do makeup as usual and the cost of wig

**I: And there is nothing you buy for house hold consumption weekly**

PL02: Weekly shopping, shopping

**I: How much do you spend on shopping, say your number, eeh**

PL02: Number two, it depends with the amount of money you have. In a week you can do for 3000/=, 4000/= it depends with how much money you earn.

**I: mmh**

PL02: Yes

**I: Number six, you were saying something. Say your number first**

PL06: Number six, If earn money weekly, I do general shopping things like household items, clothes and also buy clothes for children not that I buy for myself and leave them

**I: Like how much do you spend?**

PL06: Approximately 1500 shillings

**I: 1500 shillings**

PL06: Yes

**I: Okay and there are things people do on monthly basis. Are there things people do monthly?**

P: (All) Yes

**I: I’m hearing everybody (participants laughing). We are starting with you P7, say your number first. Everyone to tell us her number first. Ehee**

PL07: Rent, water

**I: Eeh, how much do you spend on those?**

PL07: My rent is three thousand five hundred and water is 500/= sum up to four thousand shillings in total.

**I: Mmmh, another person?**

PL03: Number three, Rent, water, electricity and garbage collection.

**I: How much do you spend on each?**

PL03: I spend 5000 monthly, Rent I pay 2500/= all those things I pay five thousand.

**I: Okay, continue**

PL01: Number one, rent five thousand, tuition is 600 then I prefer doing my shopping monthly so that when it gets tough ahead I can find where to go back, then at least I save 100/= fifty bob that would have remained

**I: Mmmh… another person?**

PL04: Number four.

**I: Mmm…**

PL04: Rent 2500/= water 600/= tuition per month 400/=

**I: Mmmh… Anyone who have not talked can tell us**

PL02: Okay number two

**I: Mmmh…**

PL02: The major thing is rent, shopping for children, school bag their school fees

**I: Mmmh... Is there yearly shopping you normally do once or more than once a year**

PL03: There is

**I: Say your number then proceed. Mmh…**

PL03: Number three, there is a shopping I can do once in a year? Or once twice. When celebrating my child’s birthday I must do. When it happens I must do it

**I: Birthday?**

PL03: Yes I must do

**I: Another person? (Noise at the background) number seven there is nothing you normally spend money once in a year or more than once a year?**

PL07: Not that I plan for it but sometimes I get sick and you must spend.

**I: Mmh hospital bill. Okay. What is the source of income for those women who do sex work?**

PL02: Let me say most women get money from their clients

**I: Another one, I** am a hustler

**I: Mmmh**

PL01: Am a hustler I must bangaiza [to be smart], you can’t only depend on sex work.

**I: When you say “bangaiza”**

PL01: “Bangaiza” means to be smart, use your client to get something that you want apart from sex

**I: Mmmh**

R: there are these, let me say it is a luck, there are those you can get, you can call them to ask for a job. So when I get the casual jobs that is where I get a break through.

**I: Okay, others. The money you normally use, where do you get it from?**

PL07: Friends also do give me but in most cases you will find its male friends who demand for sex. So we exchange (participants laughing)

**I: Mmmh, so they are just the very same clients.**

**PL07: Yes.**

PL03: Number three money we get from doing some small jobs in the houses of those men. When you go there, you do cleaning, arranging the house they will see from what you are doing and you will get money from there.

**I: What can you tell that this is my main source of income?**

PL07: It’s about being smart and be social to people. You can be called to greet someone. I can say just talking nicely with people.

**I: What am asking, everyone told me the sources, but which source of income the most money every month. Like it is your main source of income.**

PL03: Sex

**I: Sex?**

PL03: Yeah

**I: That is P3, and others**

PL06: Sex is what gives me money.

PL02: Same.

PL04: Same

PL01: Same.

**I: What is same?**

P: [participants laughing] [All] Same is sex work

**I: Is there other sources of income**

PL03: There are other casual Jobs like you can do laundry for somebody, wash for a man maybe he took you to his house, you are forced to act.

PL01: Sometimes we are hired, do you know sometimes we get hired? Sometimes you are hired to be mistress there. And such occasions you don’t go until you get paid

**NT: That’s PL01 opinion,**

**I: You must say your number first P5, are there other sources of income apart from sexwork?**

PL05: Maybe if you get laundry job

**I: Mmmh**

PL05: Mmmh

**I: Okay when you are doing sex work, how much do you get paid? You have said is the main source of income?**

PL03: Okay PL03, it depends how many shots you will do. You can agree, there are those who you go with for a short time but he pays you money similar to what you could have been paid to spend till morning. So it depends how many shots you are going to take.

**I: Mmm**

PL03: A shot is “punch”

**I: What is “punch?”**

PL03: Punch is 500/=

**I: Mmh**

PL02: PL02 It depends with how you have agreed with the one. You can agree even for 500/= and you have excited him he decides to give you 1500/=

**I: Mmmh**

PL01: I don’t go for less than 1500/=

**I: Eehe another one**

PL07: PL07, the least I get 150/= and mostly I ensure I put effort to earn above 800/=

**I: Number six**

PL06: PL06 each shot is 200/= and when I go till morning its 700/= or 800/=

**I: And number four has not talked**

PL04: It depends with how you have agreed

**I: Mmh**

PL04: And what the person will give you

**I: I heard you saying washing clothes?**

PL03: Washing clothes also depends. You can find someone who has filled three basins for you to wash for him. It will depend how much you will tell him. For me one basin, a big one I will charge 300/=

**I: I also heard about washing clothes from her and her, how much do you charge? Say your number**

PL05: Number five it depends with the clothes he has, if there are so many, like 3 basins 500/= and if quantity is small its 300/=

**I: Mmmh**

PL04: number four

**I: mmh**

PL04: it depends with clothes if they are too dirty you charge a high amount

**I: You have said there are some things that make you spend. Why do you spend on such things?**

PL01: We spend to relax our bodies you can’t just work 24/7. Then, number two, we spend at least to blend and create bond with our children, not just going to work but do nothing. Number three, we spend to help other in our families. There are those who get stuck so if you get little money you can send them. According to me that’s how I spend

**I: mmh**

Pl02: Sometimes spending is a must because there are basic things we must use daily. Like a child depends on your everyday so you must get yourself spending.

**I: how much do you spend daily?**

PL02: let’s say when he is going to school per day 100 bob for lunch 50/= for break amm and let’s say what she/he will eat supper when he comes back. Let me say the least is about 300/=

**I: Aaah others? We have all said that we all spend**

R: (all) yes

**I: who is speaking next?**

PL03: I spend 400.

I: Why do you spend on those things that you buy?

PL03: I have three children, in the morning when they depart, they must use fares, and they must get something for breakfast, they must get something for lunch and when they come back they must use fare back and again at 4pm they must get evening tea.

**I: number six why do you always spend on things you spend.**

**PL06: [Silent]**

**I: When she is still thinking. Any opinion. Anyone with opinion (silence) okay naa… (Noise at the background) those who do sex work do they save?**

PL02:, according to me, it’s good to save, at least even if it small amount, it is good to save don’t just be there, because it can reach a point you are sick and you are required to go to the hospital, you can also find that your family, your children can also get stuck because you did not go to work. So must have some little savings that can sustain you. (Noise at the background)

I: Do sex workers save?

P: [All] yes.

PL01: p1 we save for emergencies

**I: mmh**

PL01: I save for, I save for at least through those savings, I try to always pay school fees, I clear school fees balance and also buy the school materials. Then the reason why I save is that, I want to better my life in three years to come. I want a better future for my kid. I don’t want to rely on sex work. In 2-3 years’ time I want a better life. At least in three years I want to have my own business that’s why I save

**I: you said mmhh (yes) tell us why do we save?**

PL03: we are saving to have a better future life and our children. In case any incidence occur you just use your savings

**I: Number seven it’s like you want to raise your hands [**participants laughing]

**I: Do you save or you don’t?**

PL07: I normally save. I save mostly to provide for my children. Mostly they will be seeing me going out and they know I am going to work and okay. They don’t know what am doing. And they know am going to work and at least I should have some money. So I have to save so that if they are in need of something am there for them.

**I: okay how many times do you save?**

PL01: it’s a daily thing

**I: say your number**

PL01: on my side I have made it a routine. Because it’s not every day you get a job. So per day roughly I save at least two thousand shilling

**I: okay**

PL01: yaah (yes)

PL02: I save weekly

**I: How much do you save Weekly?**

PL02: it depends with how that week was it can be 2000/= or 1500/=

I: P3 also had something.

PL03: also weekly it depends with what you got

I: Mostly how much do you save?

PL03: Let me say aboyt 1800, 2000 and above

**I: We have those who save weekly. And other? What opinion do you have about savings? Seven has said she saves (noise at the background) how many times do you save**

PL07: I normally save weekly

**I: How much do you save in a week?**

PL07: it depends. When things good it’s five thousand.

**I: Okay for those who save, they normally have certain characteristics that differentiate them from those who don’t save. What kind of life do they live that enable them to save?**

R: If you say life, what do you mean?

**I: Maybe lifestyle that promote savings? Say your number then…..**

PL03: We have chamas (merry go round) in our plot that help us. So weekly you can take your money, you do your savings

**I: So they love chamas(merry go round)**

PL03: eeeh

**I: the secondly**

PL01: me I do personal savings I don’t believe in chamas

**I: You have not said your no.**

PL01: P1

**I: mmh**

PL01: I do personal because these chamas (merry go round) can sometimes fail you. You can have an emergency and it is when you want to be told to go round from one person to another. I prefer personal savings even if it is in a locked account, if you get an emergency you know the time limit after which it can be sent to you even if you borrow you can pay back within 24 hours

**I: What is the other characters of those who save? Those who love savings? She has said they love chamas, self-denial**

PL02: Those who love saving are those who deny themselves. They don’t live a high standard life so that they save

**I: mmmh**

PL02: Yes (noise at the background)

**I: what are the characters of those who like saving? She has said they like chamas, other deny themselves and with her she does it personally and you?**

PL04: Sometimes people who don’t have money, if you get a little money you save because it can help you start a job.

**I: aah okay, are there specific characteristics for those who do sex work and don’t save?**

PL01: Mmm

**I: You have said mmm (yes) tell us (participants laughing) p1**

PL01: not in a bad way, characteristics of those whom we work with are those who fail us. Most of them are drunkards not all but you will get a greater percentage. It’s those who when they earn they go drinking alcohol. Alcohol and clubbing

**I: Mmh…**

PL01: But there are the chosen few who are different

**I: What’s the difference?**

PL01: The difference is they drink responsibly, they can drink but they have left something behind [left some money].

**I: Another one, I heard mmh and it was not from one person (silence) what is the characteristics of those who don’t save what is their lifestyle (silence)**

R: Like how is their behavior?

**I: how do they behave?**

PL02: You will get that most of them buy too much, they compete with their neighbors. Let’s say when her neighbor buys a flat screen TV, she also want to buy that not knowing how the other person get her money. That’s how I feel

PL03: There are those when they get money, they get involved in violence so most of their money is used to pay hospital bill

**I: For treatment after fights?**

PL03: Yes so they go after the violence

**I: Okay number four is smiling, do you have something or there is nothing**

PL03: okay

**I: number six, seven?**

PL07: Most of them are not disciplined, they are impulse buyers. They want to buy everything they see they want to buy. You have to be disciplined and buy only basic things

**I: For those sex workers who save, why do they save?**

PL02: We are saving towards our future because you never know how the future will be and you also have kids who depend on you so. At least use the short time to save so that if you decide to leave the job [sex work]. You can develop yourself through another business

**I: Mmh… for future life, another person (silence) many people have said they save (papers raffling at the background) number six**

Pl06: [silence] (papers raffling)

**I: Number seven**

PL07: For at least to be respected in the society, if you don’t save, you will be disrespected in the community.

**I: Okay. What makes it easy for those who save to save? What makes it easy for you to save?**

PL01: like for me I normally set a target. I always sit down and decide this month I want to do this and that. So, within no time I find it easy to save

**I: Are we together**

P: (all) yes

**I: okay. Let’s talk. What makes it easy for those who save to save?**

PL02: It is easy to save because you get the money so easily

**I: You get money easily. Another person? What makes it easy to save?**

PL07: Number seven, limit expense. If you limit your expenses and make it low, saving will be easy

**I: Do you encounter challenges to save?**

R: Mmm… [All] Yes

**I: what kind of challenges do you face I have heard you saying yes**

PL01: Challenges come when like right now commodities are high for example a child has been told to go and bring exam fee go and bring this, we also have money for lunch increased. So, you find that the budget you had planned, things has been disorganized a bit. Yeah so you find that you go to the shop that you normally go to, commodity prices have been raised with 5 shillings, 10 shilling. You know that reduces the budget you had planned. So, these small emergencies are the ones that bring about challenges.

**I: How can we address the challenge you have mentioned?**

PL01: like those are basic i.e education, when a child is sick. So, the money we should save we use to pay hospital bills. You know buying medicine, education need like school fees has been increased, and buying books you were not told. So you have a challenge because moment you come with a certain amount of money and the child brings you more. You find that by the end of the week you have saved less than the expected. Those are the challenges we encounter.

**I: other challenges? Number three you have been quiet, number three?**

PL03: The challenges I can mention, P1 has said even what I had to say.

**I: Which ones among what she said were yours?** [Participants laughing] Just say it briefly so that we move faster

PL03: I have forgotten [participants laughing]

**I: P4, what challenge do you get when saving**

PL04: You can be saving and all over sudden you are called that you have a funeral. So you have to pay the money you had saved.

**I: A funeral?**

**PL04: Yes.**

**I: Okay, number five?**

PL05: (inaudible)

**I: Raise your voice please**

PL05: The challenge we can face, let’s say you fall sick**, y**ou must use your savings

**I: okay. We are continuing well. For those ones who don’t save. Why do you think they don’t save? I know you had mentioned some but you can just list them**

PL01: General view, there are others who do sex work for fun. You find that she does it whether she’s paid or not, earning or not, she must use it. So most of them do it for fun that’s all I can say

**I: Mmh, another opinion** Neighbor, neighbor, okay, okay so, okay aah. Are there advantages of someone not saving? Which kind of advantages? Do you think someone who don’t save has advantages **Stop shaking, just say your number and tell me**

PL03: There are people who have that advantage for not saving.

**I: Advantage like which one**

PL03: She gets the money, she get gets a client during the day and the job is to drink alcohol and get other clients. Her job is to earn and to drink. She uses her money and that is the advantage

**I: Number two, number six you shook your head. That means that there is an advantage or not. Just be audible for us to capture (music at the background)**

Pl06: (silence)

I: **number seven**

PL07: For me there is no advantage because savings can help you when you are stuck. So I you don’t save you can get yourself into debts, (silence)

**I: what are the disadvantages of not saving?**

R: (silence)

**I: I know everyone has an answer, because all of you are smiling.**

PL01: Sometimes you have gone to work and you don’t find work. You have used fare costing about 800/= and there is no client. So, those are the disadvantages. So if you don’t save, how can you… this is when your savings can help you. At least you can use it to buy something and leave behind and also to leave the place where you have nothing.

**I: okay**

PL01: Yeaah

PL03: you can get a client and go with and you agree, when it reaches morning you ask for your pay and he refuses to pay you and instead beats you up and you don’t have any other option that is one disadvantage for not saving.

**I: number four, is it there (silence)**

PL04: Still thinking

**I: She is still thinking. Number five (noise at the background)**

PL05: disadvantage of not saving, if you have not saved maybe you have left your house and you have been called to go to a certain place to do the job and you ask for money and he refuses to pay you. Then it will force you to use you own fare back and you also used your fare to go mmh

**I: So that’s the disadvantage of not saving?**

PL05: mmm (yes)

**I: And where do they save?**

PL01: I normally save in m-shwari

PL02: For me I save in family bank

**I: I want you to tell me, why you are saving on m-shwari**

PL01: I save in mshawri. Because i think I love keeping things private. I save there because it gives me easy time to access. Anytime I have an emergency I can just pop in and withdraw, it only takes seconds and I am sorted.

**I: Okay that’s p1**

PL02: I save in family bank because (someone coughing at the background) If I save on phone I will be tempted to use even if I don’t want to use it. So I try saving somewhere so far from me.

PL03: I love saving in a chama [saving group] when I receive from there, I transfer to KCB, , so it gives me easy time

**I: Why do you transfer to KCB or why do you love Chama [saving group]**

PL03: I lime saving group because I want my money to grow and then I deposit there [in my KCB account] .

**I: Number four where do you like saving and why?**

PL04: (no response)

**I: number five**

R: I normally save on mshwari because something may happen and you want to access it, you will withdraw from there and use it. So for me mshwari is at least [is better]. If you want to withdraw you can access it and if you don’t want you can leave it there

**I: Number six where do you prefer saving?**

PL06: mshwari

**I: Why**

PL06: Because sometimes if you don’t have something in your account they can lend you and then you repay.

**I: Okay number seven**

PL07: I normally save in the house and accumulate the funds, is when I will go to cooperative bank to save at least it will have interest

**I: Why do you save in the house first?**

PL07: You know you can’t walk to the bank daily

**I: So you avoid walking every time?**

PL07: yes

**I: Those people who are doing sex work sometimes the money that they get don’t meet their need. Okay do women who do sex work live above their earnings? I mean living above what you earn. Do nyou think in a way sex workers live a life that is higher than their earnings**

P: That is there.

**I: Say your number then narrate to us**

PL03: You can get someone…. You can meet someone maybe a client and you go with him to the house and then he leaves you there. You look around and see there is something in there, because he may fail to pay you. You take the item without his knowledge and go with it home.

**I: I am saying the money you work and earn. Maybe the money you earn don’t meet your needs ,does it happen?**

PL03: it happens

**I: Each one of you to say her number and tell me**

PL02: You find that the money you get is not enough to meet basic needs**. You find that the daily needs** are more than your earnings and especially right now the cost of living is high. Initialy you could budget with 1000/= and that could take you for at least a week. But currently it is expensive. So you spend a lot

**I: The next person? Many people said yes**

PL01: What she said has covered everything

**I: eehe**

R: what they said, people have said almost everything

**I: I want to hear many people talking and when they talk of different points. Are there people who get less than they need? P1 wants to talk**

PL01: she has said

**I: she has said it, P3**

PL03: None

**I: Seven (noise at the background)**

PL07: okay. You know this our work, it is not like you are employed that you will receive your salary. It depends with the day and whoever you will meet. So there is a time you will meet a rich client and the day the business is not good, you will find that it does not match your expenses so you will get that your expenses are high and what you earn is low

**I: And in case it happens that way, how do you bridge the gap?**

PL01: that’s when your saving are helps you.

PL02: You just use your savings.

PL03: Your savings will lift you.

**I: There is nothing you people do to bridge the gap?**

PL07: To me…

**I: Your number**

PL07: Sometimes it forces you to travel far away it will force you to sacrifice and see if you will excel, if you try to get, change the environment and see if you will get better.

**I: To get extra money. What do people do to bridge the gap, they money you earn is not enough to pay your rent and pay other bills, what do you do to get the other amounts.**

PL04: You will check your savings to help you?

**I: Everyone just goes to the savings? Number six, what do you normally do?**

PL06: My savings helps me.

**I: Should I go back to the question about savings, I have realized that now people have savings, P5.**

PL05: I just use savings

**I: So everyone use savings?**

R: (all) yes

**I: Okay thank you so much. Women who do sex work, do they ask for money or do they normally get themselves into debts?**

PL01: I normally borrow. I normally ask for because I can’t suffer and am seeing future**. Myself I borrow, not that I put myself into debts. But I borrow.**

PL03: I also borrow

**I: Where do you ask from?**

PL03: I borrow from someone. I can even borrow from a client.

**I: How do you ask from a client?**

PL03: I just convince him, you can just sweet talk him.

**I: This one borrow from client, with you where do you borrow?**

PL01: me?

**I: yes**

PL01: I normally start with my siblings, friends and option B is client, the one I know it is a sure bet [Who I am sure I will get money from]

**I: And others, how do you people do it?**

PL02: I ask from family first and then also from the sponsors

**I: And if you ask for money, you ask for it for what purpose?**

P: it’s for sex.

I: What?

P: It’s just for sex.

**I: I mean if you ask for money. You said you ask from a client? You have asked him for money, “Please give me some more money” What do you do with the money you have borrowed?**

PL02: let’s say the money you want to fill the gap.

I: Which gap?

PL02: Let’s say you need around 5000/= to do your shopping and the whole of your budget and you have 3000/=. So, you will ask for 2000/=to add.

I: Number 1 left us

PL01: I normally ask for money when am broke in that there is no way to sustain myself with children. So, I go to an extent of borrowing

**I: mmh. P3 what do you do?**

PL03: I borrow, because I am an orphan. I don’t have a father or a mother so I normally go to client. I tell them, “How things are my gas is finished, help me this way or that way.”

**I: So you refill the gas, what else?**

PL03: I want to pay rent, my kids are back for school fees

**I: Number six, you have never asked for money**

PL06: I have

**I: What do you do with it?**

PL06: maybe I lack money to buy food. Maybe my child has been send for school fee

**I: Number seven, you have nothing to say?**

PL07: Just the same fees, when a child is sent for school fees

**I: Why don’t you say your number?**

PL07: Number seven (noise at the background)

**I: Okay, and when you ask for money there are some that are to be repaid, how do you repay back? Some are debts. Where do you get the money to repay?**

PL01: definitely after I have asked for money, I make sure I bangaiza [I am smart]. My formula is that I don’t borrow from someone within Kisumu. Because the moment you borrow the money disappear in your mouth to be safe I look for someone outside Kisumu.

**I: You ask from him then you bangaiza [be smart]. What do you mean?**

PL01: you can promise him that you will go and a certain day when that day come, I blacklist him but at least you have returned

**I: ooh, that’s what you mean by “bangaiza” Number 2 had begun talking**

PL02: Since I started asking for money I have never been asked to refund, they normally just help me

**I: Number five**

PL05: (silence) (noise at the background)

**I: after you have asked for. How do you repay back. Where do you get money to repay back? (Silence) number seven**

PL07: like there is a day I asked for money and I was not given and I had some chicken, when the owner came for it and he/she accepted, I paid her two chicken

**I: okay, you gave out chicken. What do Women who do sex work to increase their income? You have said that the income is low, what do you do to increase the income?**

PL01: For me I do organize for shows, I usually sing

**I: You are a musician?**

PL01: Yaah you find that when the business is down, I I get shows where I can sing because at least one show is 3500-4000 per show. If you get some shows and again at least you will get some clients from the shows. So it’s about balancing.

**I: it’s another way to get extra cash. What do others do?**

PL02: when business is down, you are forced to relax a bit. If there is none at all I can even go to an extent of looking for a job in a hotel so that you get some little cash.

PL03: I can go to sell tei (tei is a sheng word for alcohol)

**I: What do you mean by tei**

P: [All] [alcohol]

**I: And others what do you do? P7 what do you do for your income to increase?**

PL07: I used to have a business, I started and it collapsed because of corona but it used to help me when sex work was not paying. When sex work wasn’t booming I earned from it, but it collapsed because of corona

**I: For example, when you have not found client, what do you do? She has told me others also have to say. Number six**

PL06: I can just sell charcoal at my doorstep

**I: Number four.**

PL04: You can go and clean utensils in a hotel and they pay you.

**I: Number five**

PL05: You can do laundry to people, mop their houses and clean utensils and you get paid.

**I: How do you know that today there are no clients, how do you know that the day is going to be bad?**

P: [silence]

**I: Is there a way that you can notice that today there are no clients?**

PL02: when there is no sign, you will just see no one gets closer to you, no one talks to you, you will just see everyone who gets in has a partner. You will just know there is nothing

PL03: I normally target from Wednesday, Thursday, Friday, Saturday, Sunday. Monday and Tuesday I relax, I just go to look for small things, I sell alcohol. I have two days off.

**I: And how much money do you borrow? You said that you take debts, how much do you borrow**

PL03: I borrow money, about 1000/=

I: Let everyone tell me hers fast so that we move fast.

PL02: I don’t like borrowing huge debts. I normally borrow 200/= if it goes to the highest is 3000/=

**I: Number one.**

PL01: 1500/=

**I: Number five**

PL05: At most is 2000/=

**I: Number four**

PL04: The highest 1000/=

**I: Number six**

PL06: The highest 1500/=

**I: Number seven let me not leave you out**

PL07: I normally request for 2000/=

**I: Do sex workers think of leaving the job, do you think that “I will leave this job”?**

PL02: We think about it so much. I don’t think it is a good job that I wished I would do in my life. It is problems that made me to do it. I think a lot, that’s why I save so as one day I will leave it to start another business

I: Do others also think of when they will stop doing the job?

PL06: I have thought of it so much and I desire to leave this job

**I: mmmh , okay and others? You have never thought of leaving this kind of work? Alright, is itr something sex workers discuss in a group among the peers?**

PL01: It depends

**I: it depends with what? Number?**

PL01: it depends on the groups you are part of. Like the way we are familiar to each other here, we are free with each other. If something happens you can ask how it goes and give out your opinion. So, it depends on the group you associate with.

**I: For others, this is something you discuss in groups?**

R: [all yes

**I: Tell me?**

PL02: Mostly when someone has a problem, we come together to see how we can help. Even if it means contributing for her some money.

**I: Okay number six, you wanted to talk?**

PL06: Maybe if someone is sick, we discus in the group and each person gives a small contribution to help her.

**I: And right now we are discussing about leaving the job?**

PL06: we do discuss we normally discus, like everyone has her own dream like me in 2-3years to come, I want to be a business woman. I don’t want to be an artist or a sex worker. I want to be myself, to find peace within myself. Yaah

**I: Others do discuss or they don’t discuss? Number seven do you discuss about leaving?**

PL07: I normally discuss with them until I tell them if they can connect me for employment. If I get I will be in a position to leave sex work.

I: Number 6, Do you discus with your peers about leaving this job? **Just explain to me briefly**

PL07: We usually discuss because some of us have problems, some are killed so it is not a safe job

**I: So that is what trigger the discussion about leaving or stopping? What else trigger discussion?**

PL01: Okay what triggers the discussion mostly is disagreement. Because you can agree on payment but then you are not given what you agreed and you get out of there pissed off and then you need to share. We believe that if you can’t share that’s a stress you are giving yourself.

**I: What else makes you to start that discussion about leaving sex work? P3**

PL03: [silence]

**I: Number seven, what else trigger that discussion? She has said that you are thrown away, you have not been paid the way you agreed. What else bring such a topic of leaving that job?**

PL03: Maybe you are mistreated, not getting paid or sometimes even being beaten. That’s why many of us have a desire to leave that job.

I: Normally what causes FSWs to leave or to want to leave sex work?

PL02: Because they want to have an alternative life apart from that one [sex work] and to be a respected person in the society

**I: mmh**

PL07: You will find that in this kind of business we are at high risk of getting HIV and STI those are what make us develop thoughts of leaving this work**. Because** As long as you are in the field you are at risk

**I: okay, mm, number five (silence) number five has not spoken (participants laughing) number five do you have an opinion? (Silence) number four (silence) number six (silence) okay she has said that you just look at your life you want to live a different life. She has mentioned that there are risks of infection and those are personal reasons. Are there external reasons that can make someone desire to leave this job?**

PL01: sex work is not a decent work in the society, people perceive those who do sex work to be futureless. But it is situations that push us into it. Sometimes we want to stop doing it so that we can fit into the community. Then again to be respected, even if it’s in a small way it matters a lot to us.

**I: Mmh those who are planning to stop? Do they quit the season they plan to?**

PL07: it is like gambling. It is like alcohol addiction, you know with alcohol you can’t stop drinking at once. It’s something that comes slowly by slowly

**I: okay**

PL07: Yeah but for some if they get an activity that can keep them busy just once, they can leave at once.

**I: at once? Eeh others? If someone plan, “me, I want to leave.” Will she stop the day she planned to stop or how does it happen?**

PL02: someone can stop before that date or the day she had planned. Depending on. Let’s say you have planned to stop next year. And maybe the money that you have saved is enough to start a business, so you can decide to stop before next year

**I: At what age do they normally quit sex work?**

PL01: That depends with an individual

PL02: number two, it does not have age limit

**I: Why do you say it does not have age limits?**

PL02: it depends with your heart, the way you have figured out weather to stop it or not

**I: Mmh, number four, is there an age of stopping sex work? Just your opinion**

NT: We want to hear your voice.

**I: Do we have?**

PL04: No

**I: Why have you said there is none? How does it work?**

PL04: sometimes you wanted to leave, so you have to think first. You can think about how you can get another one first [job]. After getting another one you can just quit.

**I: Which is the other one?**

**PL04: Another job.**

**I: Number six, at what age do someone stop doing sex work?**

**PL06: [Shakes the head]**

**I: Just tell me if it’s not a factor you just tell me it is not.**

P: You can say it in luo that there is no restriction.

PL03: That thing does not have specific age. That thing is sweet even old women love that thing. It does not have a specific period.

**I: It has no specific age, P7 is there an age for leaving sex work?**

PL07: okay. There is no age limit, but again the more you age. This field also has competition because sometimes you go there and there are younger people who are more attractive than you. So there will be competition

**I: okay (cross talk) how are you? Sorry a minute we are proceeding on well. You have said that age is not a reason. So what do most women who do sex work do after leaving sex work? What kind of work do they do?**

PL01: It depends with what someone has decided you can start selling vegetable, you can start a different business from that you can get a job somewhere. So it depends with an individual

**I: It depends with an individual**

PL02: Others can go home and engage in agricultural activities.

**I: Agri-business and others? When you leave what you are doing. Each one of you said that you do this business (interviewer meant sex work)**

P: (all) yes.

**I: Each one of you to tell me, what are you planning to do if you leave sex work?**

PL03: Business

**I: what type of business?**

PL03: A stall [selling vegetables], selling clothes or I open my own pub.

**I: number seven**

PL07: If I get employed I will leave.

**I: Employment, and others**

R: I am desiring to leave and open a stall for vegetable.

**I: Okay**

PL06: bit by bit

**I: okay, do you know women who do sex work, stopped sex work and come again. She left sex work and come back, do you know? Anyone you know. All people are quiet now**

PL03: I was doing it and I stopped because I was married. I left because I was married when we disagreed, it forced me to come back again

**I: Mmmh and what happened when were back to it?**

PL03: Disagreeing with your husband and lack of job. I saw that it was the best option for me to move on.

**I: Is there any bad thing that happened?**

PL03: About…?

**I: Did you face any challenge when you came back?**

PL03: No

**I: you didn’t get a challenge. What about others, do you know anyone who left and came back?**

PL02: I have not seen because when someone decide she has reached a limit, and has decided to quit and it’s hard to come back

**I: And what are others saying, number seven have you seen?**

PL07: No, I normally see when someone is expectant when she wants to rest, she rest a bit and later come back.

**I: When they come back, what good things do they get? What good thing did you get Number three? if there is if there isn’t also you should not force.**

PL03: There were no good things as such

**I: What are the small things?**

PL03: The small good things I saw was just drinking alcohol and doing the work [sex work], at least to pay rent and feeding the children at least getting something [money]. And life went on.

**I: It brings in something (money)?**

**PL03: Yes**

**I: Alright, what kind of things do women who do sex work want to achieve before they stop or leave it. What kind of goals do you want to achieve before leaving sex work (silence). I know this one everyone has? Are there goals you want to achieve? Before leaving sex work. It must not be you**

PL02: My goal is to save and reach a certain amount of money

PL03Amount of money good enough to start a business

**I: aaha, others?**

PL01: I want to make sure I buy land before I leave sex work (noise at the background)

**I: P5 do you have goals**

PL05: To start my own work of selling second hand clothes then leave.

**I: mmh, have you started achieving these goods?**

R: [All] Yes.

**I: aaha. Okay if you have started achieving the way you have said… If you have achieved or not. And if not why have you not, why have you not started achieving?**

PL03: Our earnings are still low, what we get from the clients that is why we have not achieved the goals.

**I: Income is low, P1 you were talking**

PL01: The same

**I: The same? And others? P7**

PL07: That one, income is still low that it could have been better, if we had a sacco that could offer Small loans

**I: Small loans**

PL07: Just for a start then we are given a month’s allowance to boost the business we are doing. The following months we pay. It can help

**I: Do you know those who left in the last 5 years. Who knows someone who left sex work**

PL03: I know

**I: You know? What made it easy for them to leave?**

PL03: Nothing

**I: Do you normally ask each other?**

**PL03: Yes.**

**I: What did she tell you?**

PL03: she got a guy, and got married

**I: So she got married?**

PL03: Yes.

**I: Who knows someone who left?**

PL02: I know someone who left and started a business. She is her own boss

**I: Who knows someone who left? Number seven is smiling, you know someone?**

PL07: Yes, there is a friend of mine who got a job

**I: She got a job. Okay, did she encounter any challenges after quitting?**

PL07: Yes, because there are some client who are used to you. They will still be calling you

**I: So she is still receiving phone call. And the married person, did she have any challenges, did she share with you any challenge she encountered?**

PL03: She had but they solved.

I: They solved

PL02: The one I knew decided even to change her phone number.

**I: She changed even the telephone number.**

PL02: Yes.

I: The others, do you know those who left or you don’t?

P: [All] [Participants nods in agreement that they don’t know]

I: **You don’t know? Okay. You are nodding your heads. I want us to finish up. We are heading to the end. The last part. We told you that this is jitegemee and we said that jitegemee is not Chama [saving group]. In jutegemee you save money, it’s yours, you can access any time. Okay?**

R: (all) yes

**I: It is something we are thinking of it has not started, so we wanted to get your opinions about it. You can save your money, you can access it anytime. It is not a must to save when you don’t have because it is your money. For a chama [saving group] you have to save because you have received money in your round and you have also to pay those who had given you their money. You had already been given money, and you also have to give back the money that you were given.**

R: (all) yes

**I: So, when it is the day for chama there is always some stress that crops up. It’s the day for our chama meeting. But Jitegemee is not like a chama you save money the way you want and the money is yours, you can access anytime. Us we just want to encourage you to save because the money will personally help you. So that when you are in a problem it can help you. You were saying someone can get stuck somewhere. It can help you. Okay**

R: (all) yes

**I: Earlier, I had told you what jitegemee entails and stressed on that the goal is to make sure that those who are doing sex work can have their saving that enable them to refuse unsafe sex or to rest from sex work when they need a rest. I also said it will enable sex workers to save a section of their own money so that it will help them when there is no client or to help them prepare for life after sex work. Does women who do sex work in Kenya can accept jitegemee program?**

PL01: there you know not all people have same vision. And not everyone think about the future like others. So definitely there will be challenges there are those who will reject thinking that they will lose their money and there are those who will accept so, it depends with an individual

**I: mmh. How do you think? Do you think it will be accepted? The way we are planning jitegemee**

PL02: To me I think jitegemee can succeed because it is your money you are saving

PL07: It will succeed because most of us have financial strains and money is the motivator to do sex work. It is something that will work.

**I: Mmh number three**

PL03: it will succeed

**I: why**

PL03: it is money we have saved, we can withdraw anytime.

**I: mmh, number four I want each one of you to give one opinion**

PL04: Just as she said

**I: What has she said?**

PL04: it can work

**I: why**

PL04: Because everyone is saving her own money

**I: number five, is covering her face, is it something that will be accepted?**

PL05: It will.

**I: why**

PL05: Because what you are saving is your money and you can access it anytime.

**I: Because you can access it anytime.**

PL05: Yes

**I: Number six do you think it is acceptable.**

PL06: Yes

**I: Why?**

PL06: Because anytime you want you can take it

**I: You take:**

PL06: mmm (yes)

**I: What kind of women who do sex work will accept?**

PL01: Like us, if you have responsibility you must save to survive

**I: Those who have responsibilities**

PL01: yeah

**I: Which kind of people who do sex work will accept?**

R: Number six, for me I will accept

**I: why**

PL06: it will help me in future.

**I: How do you want it to help you, what are you thinking of?**

PL02: It will help because if you have children you will need a lot of money

**I: That is also responsibility. Is there a different opinion? Which kind of women will accept? Like those who do sex work? People practice sex work at different levels. Or they work in different places.**

R: (all) mmh

**I: That is all I am asking, which groups will accept it and who will reject?**

PL07: for the teenagers…

**I: Number?**

PL07: … some of them got into sex work because of influence. She sees some peers doing it then she also joins to her, it will not be a big deal to her. So she will not agree so much because she got there because of group influence

**I: okay is there a different opinion? (Silence) okay to women who do sex work, those whom you know. I know there are some that you know from ten that you know, how many can agree? (Silence) I will ask everyone that question, how many can accept it. Let’s start with number two.**

PL02: From ten that I know, let’s say seven of them will accept.

**I: Number one**

PL01: All will accept.

**I: Ten.**

PL01: Yes

**I: Number five**

R: I think about five only.

**I: Five**

PL05: yes

**I: let’s proceed**

PL04: Seven

**I: That is P4 eehe?**

PL03: Eight people

**I: Eight**

PL06: I think 10 people

**I: All**

PL06: Yes

**I: P7**

P7: More than five will accept

**I: more than five will accept. Why do the remaining section not agree? For those saying five, six, why is it that those four, five out of that 6 out of ten will not accept?**

PL02: You will find that some of them are young in age, so they don’t have children, they don’t have responsibility, so the money that she get, she uses for luxury like buying clothes, shoes. So, she sees it like something that has no benefits

PL0: The two that will not are people who Know us and our life so they will say, these people what they are doing is useless. It is not helpful to them. I better look for a husband to stay with that’s why I have not included the two there

**I: They have given up already. Those who don’t accept why will they not accept?**

PL05: sometimes they use their money to get drunk, buy clothes. Because there is nothing to do with their money

**I: They get drunk.**

PL05: Yes.

I: **Those who will not accept, why will they not accept?**

PL04: They have lust for shoes, clothes

**I: mmh, they are extravagant buyers. That is Number four, number six?**

PL06: Some will not accept. They love leisure, they don’t have responsibilities

**I: okay. Aah, we love … what can we do to make jitegemee acceptable to women who do sex work in large numbers as possible? What can we do to make it acceptable? (Silence) just your opinion. For it to be acceptable, what can we do?**

PL07: You can by educating them. You can organize training program so that they know the benefits without them knowing the details about it they can’t accept it.

**I: What are others saying? Number two has something to say but it is not coming out**

PL02: The way she has said

**I: Education?**

PL02: Yes

**I: How do you think a program that deals with savings, how should it be done for people to love it.**

PL01: the way it is said “seeing is believing” may be if we can do so practically so that they can see the chosen few succeed, they will join?

**I: Okay, what can we do to make them know that they are succeeding?**

PL01: You know there is always some dry spell outside here. You can wait for her when she comes to borrow the 50 shillings for transport. Then you will take her where the money is saved. Then you tell her, “I told you this, now see. Then just ensure that she sees you accessing money from there.

**I: How are others thinking on how we can do it for it to be acceptable? The program that will help you save money. How should it look like? (Silence) what should it entail? I normally hear people say “me I joined the program because of this.” What should a program that helps you save have for it to be acceptable?**

PL03: I think to mobilize people so that they get to know about it and come

**I: Yes.**

PL03: Mobilization

**I: mobilization?**

PL03: Yes

**I: Number four, you have something to say? Number five, number six. Okay it’s alright. Jitegemee is supposed to comprise of Aaah … that Kiswahili word is gone. What will FSWs like about jitegemee and why? What will sex workers love? (Silence) what can they like? What can you like in that program? (Silence) is there anything you can like that program?**

PL03: About jitegemee?

**I: Yes.**

**PL03: Yes.**

**I: Which one?**

PL03: We are looking for jobs, if we can get through this program what can benefit us we will leave this job.

**I: But it is not offering jobs, you only save your money**

PL05: That is it, those are the jobs we are looking for, even the sayings is a job because, you will leave the job and use the saving to start a business those are the jobs we will have received through the program.

**I: Now you are clear, thank you, another person, what will they like? Or what can you like even you in a program like that, if there is?**

PL02: What I can like from there is that your money you can access anytime even at night.

**I: Your money you can access anytime. P1 is there anything you like?**

PL01: What I can like, it’s like it works like mshwari. I don’t have a problem with that if I have emergencies

**I: Number five.**

PL05: I can like it because the money you save, you can get when you have not gone for job.

**I: Okay number seven, is there anything you like?**

PL07: I will like it because at least you will find that even our names… it is something that is shameful so, if we also do Jitegemee which is about savings it will lift us up and we will be recognized.

**I: Okay will there be ethical issues, or human rights issues in the program. Will it arouse ethical questions? Will there be ethical question about Jitegemee among the FSWs.**

PL03: There can be questions

**I: Like which one**

PL03: They will ask, why did we decide to be in this program?

**I: mmh**

PL03: Because when we will go to talk to other FSWs trying to tell them to join also. They will ask why we decide to join this program of jtegemee. And will explain to them, you should tell us the best ways to tell them so that they can also join.

**I: I am talking about your rights. Do you think it will conflict with your rights human rights? Do you think it will conflict? Number one**

PL01: Myself I don’t see

**I: You don’t see**

PL01: Unless it is something that will be exposed. If it is secure, I don’t see

**I: If it is confidential, you don’t see? Number two.**

PL02: Just what she said

**I: What did she have say? We are about to finish don’t worry**

PL07: To some of us, you will find that some of our family members don’t know what we are doing so when they come to know about it. It can conflict someone rights.

**I: So when it is not confidential**

PL07: Yes

**I: okay what kind of challenges do you think jitegemee can encounter. Are there challenges we can encounter?**

PL01: People know me as an artist, probability… if they get something like this, they will be shocked. Eeh so and so how has it been. So, the suggestion is you should do this thing privately through the phone your phone to be your secret. **I**nstead of giving out full thing like this. It is very tricky

**I: Consent?**

PL01: You can enter [go home] with such and the baby fails to go with it outside. Sometimes you just put it there and you are doing something else, you find that she has gone with it outside and has given it to the neighbor saying, “Mom just came back with this.” You know at some point it become known, it would have been more confidential if we were doing it through the phone, you just register through the phone and that is it. So it’s you and your phone.

**I: To be more confidential.**

PL01: To be more confidential. This one is a bit risky.

**I: A bit risky, that’s why we are saying if you are keeping this [consent copy] keep it somewhere no one will see it. In research it is recommended that you sign to show that you agreed. We are about to finish. Which challenge do you think we can encounter? You have not talked much about challenges. Are there challenges? The challenges we will face when implementing the program, do you think there will be challenges?**

PL02: there will be no challenges because it’s your money.

**I: We will have no challenges. Or you are the ones who will not have challenges**

P: (silence)

**I: Do you think we will have challenges (silence)**

PL07: The challenges that can there, okay the sex worker will listen better to someone, she is used to than the one she is not used to

**I: mmh so, how do you prefer we do it to curb that challenge how should we do? Anyone can answer. We are about to finish**

PL01: For me, I think we are using… use that sex worker to go and get another sex workers because not everybody will show up, there are people who are slippery.

**I: That is one, another one that we can encounter when we do such a program. Is there another one? P3, now people are tired p6? (Silence)**

NT: The persons who have not talked a lot to say number four and five give us challenges we can encounter when we ring jitegemee.

**I: Is it normally very easy to deal with sex workers, and the challenges can we face when dealing with sex workers are like which ones? The one she has said. We use sex workers and added that we use sex works to get others. It’s like we use peer educators. Any other challenge we can face with sex workers, number three there is something you want to say. Number four, number five (silence) okay lets proceed. How much can someone save weekly?**

R: okay number two, on their own the least should be 500

I: Number one, what do you think?

PL01: number two ideas is not bad at least it should be 500/=

**I: In a week**

PL01: Yes, in a week

**I: Number five**

PL05: Just that one, 500 is good enough.

**I: The other also have a similar thought or someone has a deferent opinion?**

PL03: 700/=

**I: Number four, yours**

PL04: 300/=

**I: 300/=?**

PL06: number six 500/=

PL07: number seven 500/=

**I: 500/= okay. And how do you think we should keep that savings? mmh? Let’s say sex workers… you have said your targets**

P: [All] Yes

**I: Let’s say there is a week you cannot achieve that target. Okay. What other ways would you use to achieve that target? What other ways can you use to achieve the target? Some said 300, 400, 500, 800, the week is dry [you don’t have money], you are not able to achieve your targets normally, which jobs will you do to reach your target?**

**PL02: There are so many casual jobs you can do like work in a hotel, work in someone’s farm, you can actually do anything provided you get the money**

**PL03: Doing laundry, you can go to your friends and tell them that you have no money and ask them if they can help you find some job to do, and you must get a casual job.,**

**I: Others, P5**

**PL05: Laundry**

**I: P4 what can you do to get 300, what else can you do**

PL04: I can go and ask for job to clean utensils in a hotel

**I: In hotels**

PL04: Yes

**I: Number six**

PL06: washing clothes at people’s doorstep, you can’t miss

PL07: Number seven babysitting

**I: Those are the things you can do or there are others you have not mentioned**

PL03:: There others we have not said, sometimes I can do waste collection, I can push that hand cart to get the money

**I: mmh**

PL03: That one too

**I: mm, and others who have means of getting it. When you do sex work in a normal way, you are not able to achieve the savings target. What can you do to achieve? Am seeing number one smiling**

PL01: Mine is weird obviously

**I: Just say the weird one that is what we want.**

PL01: I will rob Peter to pay Paul. Definitely I will con this side to pay it . [Participants laughing]

**I: So those are truly ways people can use, what will others do to achieve? The normal job you do, does not achieve that target. I have seeing P2 preparing until I thought she wants to talk. Okay. Let’s proceed (music) where do you trust that we can keep your saving? Most trusted place. Where do your money to be kept**

PL02: In a bank

**I: Number one**

PL01: My person account

**I: Where?**

PL01: In a phone

**I: Which account?**

PL01: We can just open a joint account. We can open a joint account and send money there

**I: And when you are doing personally**

**PL01: Personal account**

**I: Yes.**

PL01: it’s fine, so long as it’s registered.

I: Where should it be?

PL01: It is just a phone, because not everyone have time to go to the bank.

**I: In a phone, you open a bank accunt…**

PL01: you can open a bank account, and the bank account should be accessible through mobile phone. You must not go there.

**I: aayah. Mobile banking**

PL01: yes

**I: Where do you trust to keep your money?**

R: Bank account

**I: Which account in the bank?**

R: (silence)

**I: Number four**

PL04: Me I can save in my “changa box” (laughter) then I keep it somewhere

**I: You kee in a change box that has a hole, you drop slowly in the house?**

PL04: yes

**I: Number three**

PL03: Bank

**I: Bank eehe , number six**

PL06: bank at least you can get a loan and interest

**I: Why in a bank, some of us have said in the bank**

P: Because it is safe and you can get a loan, tht is the advantage

**I: Safety and loan?**

R: (all) mmm (yes)

**I: The one saving in a changa box? Why change Box?**

PL03:: a rat will escape with it [participants laughing]

**I: Let her tell us herself, number four**

PL04: am keeping in “change box” because of the issues of walking to the bank its time consuming and also waste of money that’s why I keep in changa box.

P: If someone steals at night?

**I: the last question, okay, is there someone with a question Or different opinion or any addition? No one has any question?**

PL02:, how long will it take to accomplish jitegemee?

**I: after we have done analysis, we will get the results. If your answers show that you want us to do jitegemee. That Is when our leaders will sit and plan, that’s why we have to come to get your opinion the way you will like it to be done? Any other question?**

Pl01: I have a question that, target should it be the one set or it can be more or less or its an opinion thing?

**I: it’s optional. The way I said even if you don’t have, you can assume, it’s not like chama. Yaah, because it’s your money you are saving. When you don’t have you don’t have you can also have an account and withdraw to help you but we want to make sure that you have an account to help you handle your challenges. Another question (music at the background). Thank so much for talking with me, am so glad and from what you have said I know what you have said will help us in jitegemee programming. Okay?**

R: (all) yes

**I: thank you for your time. I don’t always end without saying be blessed. Okay**

END
