# Supplementary material for: Eat a little and save a little: A qualitative exploration of acceptability of a potential savings intervention to reduce HIV risk among female sex workers in Western Kenya
Source: PLoS One. 2024 Dec 19;19(12):e0310540. doi: 10.1371/journal.pone.0310540 (PMC11658496; doi:10.1371/journal.pone.0310540)
Supplement: S1 File — (ZIP) [file pone.0310540.s001.zip › Jitegemee Transcripts and Dissemination Notes for Journal/FGD M.docx]

**VENUE OF THE INTERVIEW: UHEMBO**

**FGD ID: FGD M**

**INTERVIEW DATE: 28/APR/2022**

**MODERATOR: OLIVIA OKUMU**

**NOTE TAKER: NANCY OUNDA**

**CATEGORY: ABOVE 30 YEARS, RURAL.**

**I: This is FGDM being done in =Uhembo= the date is 28/APR/2022, the venue…the … moderator is Nancy and … (just continue- someone whispers to the interviewer from the background) the moderator is Olivia, the note taker is Nancy. So, now as we begin after explaining to you a little bit what *Jitegemee* is all about, what comes into your minds? I know that by the time I was speaking, some thoughts were in your minds, so each and every one of you to tell me what is in your mind? After explaining to you a little concerning *Jitegemee*, what comes into your mind? Or what do you think concerning *Jitegemee?* Number seven?**

PM07: For me what comes into my mind is how I can stop this job so that I can begin making my little savings so that I can start my business. Yes.

**I: Mmh, next person? Number five.**

PM05: For me what comes into my mind is that I also want to leave it a bit, I start… I make sure that I save some little money, so that l become independent.

**I: Okay next person? Number four?**

PM04: In *the Jitegemee* I understand it teaches us, it may teach you how you can quit sex worker to another job apart from sex worker.

**I: I would like to clarify something before we proceed, *Jitegemee* doesn’t want you to stop this job, or force anyone to stop but it require when you do it, you make sure that you don’t get infected with HIV, but to those who are ready to stop it are also encouraged to save so that when they stop they may find what to do. So it’s not that *Jitegemee* require that you stop sex work, no, is that okay?**

R: Mmh (cross talk, participants responds at once)

**I: Let’s continue, what comes in your mind? Number nine.**

PM09: For me what comes into my mind is that Jitegemee is not telling us to stop, because when we are told to stop, my mind is already thinking that if I may stop it by now and yet I haven’t saved … yes we have been told to save but maybe I haven’t saved enough amount of money as per my capacity… so what comes into my mind in relation to that is that, it’s good to save even though anything that my come my way soon and I dint expect to get it, and now for prevention purposes, we need to take precautions like the availability of drugs for PrEPs for prevention so I always know that because I use it, if I may find someone who says that he doesn’t want condom, he just want us to go without anything( go without anything she meant having unprotected sex) it’s that I hope and have faith that afterwards I will take PrEPs because I depend on that money and I cannot leave money ( to mean she cannot miss getting or earning money).

**I: That was number nine, another person? Number eight.**

PM08 I support what has been by number nine, that’s what I wanted to say.

**I: What did you want to say?**

PM08 I just wanted to say that I cannot stop. Just what has been said by this sister (participant meant respondent nine), I just feel like proceeding with it and therefore am the one to see how to proceed with it.

**I: Number two, what comes into your mind when you hear about *Jitegemee*?**

PM02: What comes into my mind is, are these, this means that when you save, you might not experience the worse at the time that you are experiencing difficulties ,whenever you are experiencing any difficulty you would think about the money you have somewhere that can help you out. But at the time that I don’t have anything and left the house with nothing, I feel that, that action is wrong but still I have to do that because I have nothing.

**I: Mmh, number three.**

PM03: I as number three, I do feel that when I save enough I use what I have saved is for me to start a business, I don’t continue with it (participant meant that she is not continuing with sex work)

**I: Okay, number one.**

PM01: Myself what I have seen in *Jitegemee* or what I have heard about or what I heard or what comes into my mind. I feel you can bring us teachings so that we may find on how we can prevent diseases like HIV and other STIs because during the time that you really don’t have something (participant meant money) and your customer (client) calls you, it will just force you to do just what he said for you to get how you will survive in the house with children. But when you have something that you had already saved, you will have… you will have a reason to refuse, because you have what to survive on even if you dint do his wish.

**I: Okay**

PM01: Now we can find how to prevent the diseases that are sex related.

**I: Okay, number six.**

PM06: (hitting sound is heard from the background, clears throat) For me, my opinion (flipping papers, I cannot undergo through problems, I may be…like accepting to…sometimes if I, if I, I mean if I have shortage of something in the house and then I find someone who make me a phone call that I may go to a certain area, I can go and have agreement with him. And when he tell me that when he has sex with me, he can give me certain amount of money. I may ask him if he has a condom and he may tell me that “It’s not a must that we use a condom” so if I am in a situation whereby I have challenges, I will accept and have sex without a condom and I charge him more slightly more than what he ought to have paid, I will accept and ewe may have sex without a condom.

**I: So the money that you get as “field team” (interviewer meant sex workers), what do we do with it? What do we spend this money on? (Flipping papers)**

PM06: I have an answer (respondent whispers)

**I: Yes, number six, you say I as number six.**

PM06: I as number six, I can…that amount of money that we are being given, if you have a school going child, sometimes school fees is needed for this child and you may be having a shortage of school fees on other small commodities that we lack.

**I: What are the things that you buy daily with this money? They are like which ones?**

PM06: I may buy pads, I may buy food with it, I may buy cloths, I may buy seeds that I may go and plant in my small piece of land.

**I: This amount of money that you get, there are things we spend it on, not so? The things that we buy every day, the things that we buy once a week and the things that we buy once a mount, so I was requesting that when we are giving comment she clarify/ explain to me that on a daily basis I mean I do use certain amount to buy one, two, three (to mean to be specific on the things that they buy) and once in a month we know the amount of money that we pay once in a month? So who is to telling me how she does it in a weekly basis, in a month, number four?**

PM03: The money that I buy in a month, I pay for Mary go round *“Chama”* and some I pay for school (school fees).

**I: Is that monthly basis?**

PM03: That’s for the month?

**I: (participant cough) Money that is meant for Mary go round can be how much?**

PM03: In the “*Chama* “I have Mary go round of three hundred shillings, and the one meant for the year, the one for the end month, I also have one which called “siri ya Jikoni” (This is a Chama which involves buying kitchen items in turns). And then weekly I can (clears throat) weekly I may buy…I can use money in making my hair, I buy even a panty, for the daily, every day is food.

**I: Now for the daily, averagely, how much do you use in a day?**

PM03: Averagely, it’s like five hundred.

**I: And weekly, the things that are done weekly, how much does it cost? (For the services and goods bought).**

PM03: For the things that are bought weekly cost one thousand and above.

**I: And for the monthly basis can be how much?**

PM03: Three thousand.

**I: Any other person, number two.**

PM02: As number two, you know when someone… it depends with how one’s day was, because even in the market you may go but in some day you may not make sales (clears throat). Sometimes in a day I may find some five hundred shillings. You know when you reach in the house with that five hundred shillings it will be over and get finish the same day. In a week fortunately or unfortunately you may get like two thousand or a thousand and you say “these two thousand shillings of mine I want to take like three hundred shillings I put somewhere (participant meant to save) one thousand I take to the side of school fees and I use the balance in making shopping that might push me to the next week. This is because each and every day you might not know about the income you might get. And a times you may be your sexual partners and may be…you know something also depends on how you relate with each other. You know when you are used to each other, it’s just like those who are staying together. One day you will be lucky to find one who is known to you, you know that those new people are the ones who can give you good amount of money (knock at the door is heard from the background). This is because the ones you are used to and meet most of the times may tell you “today, the daughter to my mother in law (Praise name is used) today I also dint have a good income” and in a month sometimes you may be lucky to find someone who might give you even five thousand. If you are not paying for the rent you might say “ah, let me save this five thousand and I use the remaining balance for fixing my small things(she meant doing shopping) that will push me to the other month. That is my opinion as number two.

**I: Number three. Your daily expenditure or even sometimes it might not be yours but generally as a sex worker, daily expenditure, it’s not a must, that you say your own expenditure, by the way, you can just talk as a sex worker like for example every day we buy this and that, weekly we buy this and that, monthly, in that manner.**

PM03: I as number three, my daily expenditure is five hundred, and if I get for the weekly, helps me buying cloths for the children, panty, and pads. For the monthly I use in doing my hair and paying rent and if I may get like five thousand, I save like three thousand, two thousand, or one thousand I use for shopping. One thousand I keep in my phone (save to M-pesa) for emergency purposes, because that three thousand that I save, would not want to use it (participant means she doesn’t want to use her saving, participant clears throat). My friend may also visit me and tells me that he or she has a problem so I may say that “these that are kept here let me help you with even two hundred”, Yes.

**I: What about the monthly basis, like how much?**

PM03: Monthly expenses is like, let me say it’s like one thousand five hundred.

**I: You do…usually what do you do once per month? Like which ones?**

PM03: The things that I do once a month are more and even I say I cannot finish now.

**I: Briefly, you will just say briefly.**

PM03 Like so if I may say once per month, I may say that it’s around that five thousand, I may say that I take like three thousand five hundred, I send to my mother. Something of the sought. Yes such like things.

**I: Okay (laugh from the background), so this area there is no payment of rent?**

PM03: But I pay.

**I: Is it monthly?**

PM03: No my rent is not monthly, it’s on a weekly basis, I would not afford monthly.

**I: Okay, so in a weekly basis, there is money you set aside for rent?**

PM03: Yes

**I: Okay, another person, the money that sex workers usually earn, how has it been used?**

PM05 I as number six (interrupted by interviewer and despondences, (cross talk) Number five.

**R I: Number five (Cross talk from participants and interviewer)**

PM05 If I get money even after one week. I may use it to buy pads, I may use it in doing my hair, a times may save a little because at any time, emergency may come at any time, and I buy my minor house hold items like cooking pot, cups, and plates.

**I: Cooking pot, cups, plates are bought after how long?**

PM05 You may even go to the market if you have some money in your hand (having some money) and you say “let me pick this cooking pot” And again you may go to the market after one week, after one week. Sometimes you may even go after two weeks and you may find something nice as a woman, something like a cup, you pick but you cannot buy it daily like any time you go to the market. You may go back after two weeks. Sometimes you go on a market day like Tuesday as in =Ugunja=, we usually go on Tuesdays, you go and find another cup, you pick but you cannot spend it all. You may just spend like some three hundred, even if you just save one day, God willing I will start my business.

**I: Okay, now generally, the use for… Some people have told me that they spend five hundred per day, is that so? What is it used for? What is it used to buy, number (Interrupted by a respondent).**

PM03: That five hundred (interrupted by interviewer)

**I: Number three.**

PM03: I can buy a soap, oil…

**I: Every day?**

PM03: No, when you get that five hundred, you can just do a small budget like buying a soap, oil, something like steel wool.

**I: Okay. And in a week, what do you usually do in a week (cough from the background) and you forget about the money you get. (Birds chipping from the background, silence) … seems that is difficult to talk about?**

PM03: I as number four, talking about that might be difficult because you know you might get money in a day like today and you use it and then tomorrow you don’t get money, the balance you remained with from that day you got money is what you can use in even buying food. It easy to budget with money daily or monthly.

**I: Okay**

PM03: So for the monthly you use it on saloon, make ups, you buy pads, you can pay school fees but the remaining balance is for the daily consumptions. You may…like now we are purchasing grains/ maize. You may take five hundred in purchasing maize like six kilograms, you buy cooking oil, make sure that the children have eaten. And again the next day when the work is nice, you again get some little (money), you budget little by little in that manner. The weekly one is not easy because you may not know a week may not go by before you get money so budgeting weekly is difficult ( not realistic).

**I: Okay, is there anyone who wants to add something (cough from the background) the money that we are spending that we have talked about as sex workers, (cough from the background) where does it come from? …have you understood that question?**

R: (All responded ‘Yes’ in choruses)

**I: Where are the money usually from? (Some whispers in the background).**

PM07: I as number seven, you receive the money after having sex with someone, that is when he pays you the mount you agreed on… is what he gives you.

**I: (birds chipping, knock at the door) is it someone you are used to (in a relationship with) or is he someone you meet the same day then after you are through with business (have sex) and depart?**

PM06:: I as number six (clears throat), I may say that maybe it’s someone you have been in relationship with and he might call you via a phone that “today I want you to a certain place so that we may meet and you go, so he tells you “we have been meeting but I have never treated you well and today, God has blessed me with something nice so I want to do for you something good of which I give you money so that you go when you are well sorted.’’ So you as an individual is the one who knows how you will spend it.

**I: Okay, so it’s from these… number six says that it is from those that we are in relationship with who usually communicate to us via phone call, and are there some people that you are not used to (not in a relationship) but you meet the same day, finish your business (have sex) and gives you money, do you meet such? (Cross talk by the participant)**

R: Yes they are there (non-identified participant)

**I: So the money that are from these people that we are in relationship with and those that we meet just once, which one is major? Those we are in relationship with or those that we meet once?**

R: The ones we meet once (Cross talk, from [participants)

**I: Okay… (Birds chirping) and have you counted for me what you use for this money, the things you buy using this money. I haven’t heard the list exhausted but you have talked about food, food and school fees. But these are the things that when you internalized well you as an individual, food you prepare for the children, school fees you pay for the children. But you as a sex worker, what do you buy for yourself and how much do you usually spend in buying it? Number seven.**

PM07: I may buy my cloths, I may buy panty, and sometimes I may even buy the “E” pills (emergency pills) I may spend something like five hundred shillings.

**I: In buying “E” pills?**

PM07: And the clothes or pads or (overlapping speech)

**I: Okay, this five hundred, you set five hundred aside for yourself after a period of how long?**

PM07: It’s like a week.

**I: Five hundred for yourself? Another person?**

PM06: I as number six I, can use that money to maintain myself so that I look nice using make ups (cosmetics) so that I be more attractive to them (Laughter’s from the background).

**I: Now the amount of money that you use in make ups, how much does it usually cost, like how much do you set aside for the cosmetics?**

PM06: I can spend five hundred.

**I: After how long?**

PM06: I can ,say that in a week when I want to buy make ups and beauty commodities, I may use one thousand five hundred because of oil, make ups ,weaves for making hair, or even a nice shoes together can be .one thousand five hundred.

**I: In a week?**

PM06: Yes in a week.

**I: Another person?**

PM09: I as number nine, part of the money that we get in a week from our customers, the first thing that we can’t lie to you about is that for you to find most customers, you must make yourself nice, and making your body beautiful is costly, so that when you reach there and giving out the price, like the place you have gone to sell at, that person would just feel that he must just do as you have said. So to me personally, for me to take note of my expenses as I am taking leave to leave to the job, I don’t receive less than two thousand shillings.

**I: In a week?**

PM09: Yes and I know I will come back with that money and may even double it or triple it. Of which a portion of it must be school fees I pay for my child where he is at school, myself I don’t pay rent for myself but I pay for him/her rent where he/she is in school. So in that house I have to make sure that there is school fees, food as part of that money.

**I: So there is amount of money that we set aside for cosmetics (cross talk while saying cosmetics, both participants and interviewer) for you to get a good amount of money (cross talk while saying a good amount of money by the participants) another person again?**

PM05 I as number five, from the money we make you can use like one thousand five hundred, you want to buy oil, You want to by those make ups, you buy another nice cloth of which when you leaf while dressed it and when you reach that place (the meeting point) he will say “ this lady is not affordable to me because of the things she is put on, they are expensive” So even if you leave that place taking in ,consideration that you had already bought make ups, you may buy a another cloth, your trouser, you may add another dress/cloth. You may also save some money. You even may sought any problem that may come your way. You may go and meet someone who tells you that he is giving you five thousand shillings but he doesn’t want to use a condom and you may say “Just leave it”. So when you get such la person, you can come back and take the money you had already saved to help you out while still looking for someone who may be ready to do your wish.

**I: So for buying the clothes, number nine has told us that two thousand is enough for her… no number six has said that one thousand five hundred, cosmetics for maintaining beauty in a week, one thousand five hundred is enough for her, Number nine, two thousand is enough for her, number five, for you, how much money is enough for you in a week in doing the same?**

PM05 One thousand five hundred.

**I: One thousand five hundred. Number four?**

PM03: I as number four, my view is two thousand shilling because right now taking into consideration of the current economy, everything has risen in price, so when I want to make myself beautiful, I start from body oil, you must buy shoes, you buy cloths, so if I am calculating that, I may even spend about two thousand.

**I: So the cloths that are meant for the “field” (sex work), are they bought weekly?**

R: No, it’s not weekly. (Participants are murmuring from the background)

PM03:: You may buy it weekly because you cannot put it on daily ( the same cloth), people would see you with the same one today, tomorrow, next day so you need to change them a times.

**I: Number nine was speaking,**

PM09: I as number nine, I cannot say that I can buy cloth for the “field” (sex work) every week because sex work found me already dressing. (Participant meant that she was dressing well even before she got engaged into sex work) of which now I am just adding them (the cloths) for me to be more beautiful. I may even put on one of my cloths that I bought sometimes back of which when you it on, you look smart. You go and attract the money and then keep it (The cloth). It’s not a must that you have to buy it weekly because a times you may get that money but then when you calculate, for you to buy a nice cloth, it’s about one thousand, you may be pressed somewhere of which you cannot buy it every week, this is because you want to fix different things, and so I may buy it twice a month, but I cannot focus on buying cloths weekly, No.

I: Number eight.

PM08 The money that I get may help me in paying school fees, making hair because the hair is about five hundred shillings, because you want it designed in the modern way, you buy oil, you buy soap, you buy biker, you buy panties the remaining balance I take to the Chama then the little that will remain I save. I save in my phone (Mpesa) may help me (the sound of mooing cow from the background) and may survive with.

**I: Do you do that every week, or every month or daily?**

PM08 (Child playing from the background, I do it in a month.

**I: (Cow mooing in the background), and now do the sex workers save money? I don’t know if =siaya= those… or is it understandable? What I have said, is it understandable? The sex workers in this area.**

PM06: I as number six ( birds singing), that money you may save of which you may get ( money) you may spend in purchasing piece of land of which one day when you meet your friend who usually boost (Support) you and he may tell you “ I have been, I mean we have been meeting (interviewer clears throat) with you and I felt like coming to see your residence” and honestly when you picture how that person have been supporting you and for what he has requested, you may accept “ For myself it’s my wish that you may come and see (visit) my residence” because you kwon when he come to you he will not let you bear handed (Participant meant that she will not be left without anything). He will let you sorted and he would realize that you are a person with a brain (Participant meant to be wise), of which you are not wasting money, you may… you save your money and you can even buy anything of your, of which, when he comes he will view you as a lady who may make something great.

**I: Do the sex workers save? Can a sex workers save?**

R: Yes (Cross talk from the participants)

PM03:: I as number three, Sex worker can save, she can even change responsible in that same work that she is doing, she may even make the partner responsible and for it to come to realization of people that she has been working as a sex worker, even people would not believe, this is because of how you take good care of yourself and the way you have saved, you can buy a piece of land and you build yourself a nice house, permanent. And you have nice car, you understand? And you also open a large business and you employ others.

**I: That was number three, number nine?**

PM09: Yes we can save as sex workers because the way we are given this money, like I go to job and come back with money, of which I had said that you have come back with money, you have come with five thousand, this five thousand you want to spend, it’s a must that you will remain with some balance. So how I do it personally as number nine, I have a Mary go round of which I go to weekly, I have Mary go round of fifteenth and end month. If I want to divide this money to both of them (Mary go round), that would mean that I have saved, even if I don’t save it on phone( mpesa) or not even go through bank account, but I have save it? Five hundred have been saved to that pool, on fifteenth one thousand in the other pool. I know how I divide my money to safe place of which I know that such a date, I will receive a certain amount of money and I will spend it in buying my major thing (commodity) that is attractive.

**I: Number four? Do the sex workers usually save?**

PM03: Sex worker may save in many ways, he may save in the way that number nine has said it. She may even save, she may even save in her business, she may get some money of her daily, day time if she is the one who works at night, day time she goes to her stall as she sells her items and when she gets her money, she saves. And again she goes to look for other money, and again when she gets, she use it in ,supporting her business and even save part of just as it was said by number nine.

**I: Another person, number one?**

PM01: I as number one I may say that I am supporting these people, when I get money, I go to Mary go round so it’s the Mary go round that I usually save in, when it reached December we share out, So at the time you share out, the budget that you had for the last year, you calculate and it will fit the money that you have been saving, so sex workers do save.

**I: Okay, now number nine has said that she can save twice a month, and you can…**

PM01: I save weekly and I save twice a week.

**I: Number seven, do the sex workers save?**

PM07: sex workers can save but in relation to greater inspiration ratio, at that time when you go to work you get small amount of money and that you may spend in food or school fees if you have, you pay rent, so you can save or you may not save.

**I: Okay but the money that we save, they are for what purpose?**

PM05 I as number six, five, and you may open your business, you may even open an Mpesa, and you can even build your personal house.

**I: Another person, number nine? The money that you worked for so hard every week, it’s for what purpose?**

PM09: It’s in ,the program, when I save, at the end of this year, I save next month, when it comes to the end of the year, I will buy something of my household hold that I planned to have in my house, of which at end ,month I know that I am going direct to buy certain thing ( commodity) of mine so that even if my friends comes and you feel like “ah, I as number nine, the health of this lady, what could be contributing to this lifestyle that she is leading , and the work that I do is just the same one. “

**I: Number two, the money that we save, what do we usually l do with it?**

PM02: The money that we save, we save it with a purpose, It’s in the program as in you may work and the job must have a retirement so you feel like “ ah, this my job even if I am performing it but as the time goes, I may want to save so that I may meet my target amount of money, and the day I will get it ,back, I may start my business of from which I will built myself a house and stay in it as I will be weary and tired of work, so I build myself an house to stay in as I have my Small shop around to be that environment, ;that why I use to save that amount of money.

**I: And the behavior of those who usually save, how would you know? Is it easy to identify those** **who save?**

PM05 I as number five, we may know by looking at the lifestyles she leads, she has prepared the lifestyles she leads well, so we just look at the lifestyle she lives for us to know if she is making savings.

**I: How is the lifestyles of someone who saves look like?**

PM05 They live in a good life style.

**I: Another person? How is the behavior of those who save?**

PM09: This…I as number nine, I may just support what has been said by number five because the person who saves cannot totally luck or let’s say that I may have a problem like let’s say that I’m sick and have not gone to job, and if I luck and my minds will click on somewhere, I cannot be stressed so much because I Know there is somewhere I will go and withdraw, and will help me fix the problem I had or help you out of that disease in you, so you just see it that so and so it’s like…that’s why it’s usually said that so and so has money, it’s because of savings, and it’s just because of savings bit by bit. So it’s something that is visible even in your forehead that it seems you are making savings.

**I: Mmh, another person, number eight, the behaviors of those that do saves are like which ones?**

**PM08: Just as number 06 has said**

**I: May you please tell me something different from what number 06 has said**

**Participant whispers-number six) tell me, something different from what they have said.**

PM08 (sharp sound in the background ) I as number eight, if you have a problem in the house, there is no way you would save or and if you are capable of saving and may be having some money that you want to survive on here in the house, for that reason you can get how to save. (Silence)

PM01: I as number one, savings helps us so that ,you may not be that disparate to the extreme, because when you don’t have savings you will always be disparate to the extent that when someone comes to you with even two hundred (Shillings) you .have already accepted because you want lunch, you don’t have anywhere to turn to, sometimes he comes to you with even two hundred, you ,you have just feel like you take that two hundred and ,your colleague had already rejected that five hundred somewhere but he will just come to you because he already know that you are disparate. And when you have savings, you don’t be “loose” (disparate).

**I: What do you mean by saying “loose?”**

PM01: You don’t be someone who is quick of which you are just too quick to take the money which is valueless.

**I: Mmh, that’s number one, number has raised her hand?**

PM05 Mmh (No)

**I: Okay number six, what is the behaviors of those who save?**

PM06: I as number six, the life of the person who saves money have changes, or even if you have… the person who saves money, you will see a change in her lifestyles and you cannot be someone who “has abandoned himself (is irresponsible against her body and the overall look out), you will find that you use that amount of money and you save bit by bit.

**I: A person who has “abandoned himself” is what kind of a person?**

PM06: This is someone who doesn’t take good care of her body.

**I: Mhhh**

PM06: Mhh

**I: I am still not getting it right.**

PM03: She unkempt, I as number three I may say that she is unkempt because when she gets a little… I am just trying to support what was said by number one. You know that when you don’t save, you will… you are just there (participant meant that the parson who doesn’t save cares less, he cannot even make a good decision, may also mean to be careless). And a times when she wants to leave she doesn’t feel like “Let me wash my legs and put on my cloths…, yes you are someone who doesn’t care. But when you are someone who saves her money, you must take good care of yourself so that you seem clean and smart, because you have already made the decision you want something big,

R: You be expensive (Non identified participant)

PM03: Yes, you become expensive.

**I: And those that doesn’t save has which kind of behaviors? You have already given me** some view … what kind of behaviors do those who don’t save have? Number seven?

PM07: It’s someone who can be easy going (disparate) to her when k anybody approaches her, she gives in so easily.

**I: Number nine? The person who doesn’t save has what kind of behaviors?**

PM09: The person who doesn’t save is someone who… I can just support what number seven has said. Because she honestly don’t have of which when she finds someone with two hundred shillings, she will; go, she goes (Have sex), because he has left the house without anything, and you know in this work that we are doing, for you to get anything having in mind that you left your children behind and you have to have a plan for everything, and even if you go there as a visitor you will see the children giving you food in the absence of the mother, this is because she planned it, what of if I dint have...I dint save that two hundred and the way our economy is right now, when somebody gives you two hundred, and the work he has done in you so big (to mean the action of sex), how is the money going to help you? That must be someone who doesn’t save, she will go for two hundred, or five hundred.

**I: Number four? The person who doesn’t save have what kind of behaviors?**

PM03:: The person who doesn’t save have selfish desire, she feels like she may go to the “field” (Sex work) for her to get even some two hundred shillings or even get one hundred as long as she gets money. Its, money provided that she gets money. She does not have a target or stand out for the amount; of money that she wanted, she don’t have a target of the amount. She just want what might help her for that moment, provided that she gets anything in, the name of money, even if it would be one hundred (shillings).

**I: Number three?**

PM03: That’s someone who doesn’t save.

**I: Those who don’t save, what is usually their behaviors?**

PM03: Those who don’t saves have selfish desires, I mean because they haven’t save, the need money that comes so quickly. (Noise from the back ground) Provided that she got someone that gives her fifty or one hundred, provided that she gets money, she doesn’t care about the amount. Those are the behavior of those who don’t save, but now those who save, they just know that today I may get like five hundred shillings, six hundred, one thousand, and from these, the may say “from these five hundred let me take two fifty and save somewhere, you may get that she can save even one thousands shillings in a day

**I: And to those that save, what makes it easier for them to save?**

PM03: What makes it easier for them to save, depends with the responsibilities that they have, You might go and luck any income and maybe you luck enough food in the house for the children, you are in the house with them and one may come and tells you “mother, I need money for the tuition or a book “or maybe you could have saved, so that’s the point that you will think about savings. You’ll ask yourself, I haven’t buy cloth or even books for the children and I have not even saved even one thousand anywhere. You may abruptly go to someone and she gives you that one hundred for you to take to school so that your child may not stay at home, because people know that you are working but they don’t know the type of work that they are doing. They just see you leaving at night and coming back in the morning or you leave in the morning hours and you come back at five evening or even at noon. So when they see your child staying at home they will just say that is like she doesn’t ,have money, but now you personally in your thinking you will be like “ I don’t want my child to be seen here (at home), I just want my child to stay in school”. So it forces you to save.

**I: Okay to those who keeps money, or those who save, what makes it easy for them to save? Number five?**

PM05: Savings is easy for them because, you can just think that you will need something in ,the feature or even any problem may come your way even if you dint know. So you must save so that when it happens that you have any problem, it will sort you out.

**I: To those that save, what makes it easier for them to save money? Number four?**

PM03: Saving money may be easier (interrupted by the interviewer)

**I: What makes it easy to save?**

PM03: When you are well planned, you already have it in plan that… maybe you have received even like two thousand, you said that this two thousand, I want to spend one thousand five hundred, five hundred I save it somewhere, the person who saves money savings is easy for her because, she has money most of the times, she can’t luck. Even when she leaves for the job and she doesn’t receive any income, she doesn’t feel stressed because she knows that she has save something. And the next day when she got (Income) she again adds (Participant meant add to the savings, she doesn’t spend it all. So she finds it easier because any time that she lucks something she finds where to turn to.

**I: What makes it easier for those who saves to save? Number two?**

PM02: She has been meditating and thinking about that, that she must be making savings. Money… You must be making savings because… like I as number two, in relation to this kind of job that we are in, sometimes you may leave for job but you get an accident on your way, You know when I have not been savings then it will cause some burden, but when you have been saving, it’s so easy, You shall have caused your fellow ones an “easy work” (caused them no burden)You know when doing this job you also have to be friends, so you cause your colleagues humble work like you will hear number two got an accident in such an area, she will then rush to you and you ask her “ what do we do? And she tells you that I personally, do this withdraw this amount of money from such a place and take me to the hospital with it. But when she comes, and find that you don’t usually save, and may be it might have caused you injury, you know she may also say that “To me so and so has abandoned herself, I also abandon her and leave there. So you must have the motive of saving as you as you look forward. The person who saves is the person who has the view of the feature, He is not only think of the thing that are right here.

**I: Number six.**

PM06:: I as number six I am just supporting what number two have just said because the person who saves money would not want his account to drained to zero, this is because problems can come in any time.

**I: Okay, and what are the challenges that those who save usually face? The challenges? Do those that save undergo through challenges? Challenges?**

PM03: What do you mean by that? In case you keep money, you save, is there any challenge you will save?

PM03: If you save?

**I: If you save.**

PM03:: Yes, I as number three, when you save you must go through challenges, the first challenge is that you personally can be sick and you feel that you dint want to spend what you, had already saved, so it will force you to withdraw and spend it settling your hospital bills. And may be there in the hospital you have not been given me enough drugs. They are just prescribed so it will force you to go and buy them from the chemistry. So you withdraw the money that you had already saved, you use it for the hospital bills and then, you spend some in buying the drugs. After recovery you may go for work, get some money and you repay back to your savings.

**I: Another away that caused us difficulty are like which ones?**

PM09: The challenge that you may face, I havened understanding that much because even the answer that number three has given out, you have asked me about the challenges or what makes it easy?

**I: The challenges.**

PM09: The challenges that we are facing. I as number nine, if you have been saving and I know that I have money in my account, you may be having challenges but it might not be of a burden to you that much because I Know, I am sick or maybe one of my children is sick, I just go to my account and withdraw whatever little that may help me out of this problem. So this challenge may not stress you so much because you have somewhere that you can turn to.

**I: The challenge that I am asking is the challenge that is related to money savings. When you are saving money, is there any challenge that you face because you are saving money? Challenge that anyone who saves may face. (Some participants’ mummer from the background) Or May I am not asking it in an understandable way? Is there anyone who gets it right?**

PM01: I am trying to understand it bit though (Interrupted by interviewer)

**I: Please clarify, that’s number one.**

R1 Yes, now the challenge that might be there is that you had already had a target of saving monkey and then the economy becomes a problem or the workload has gone down of which you may not be able to reach the target that you have been savings and therefore you must not meet the target or your savings may go down.

I: You want to add something? Number eight? Do you have any idea?

PM08 No

I: Number four?

PM04: Its ‘like number one said it is how it is sometimes challenge that ,might be faced by the person who save, because when you save, are focused to see that your money grow, and so it grows and as you save you have a target, maybe there is something you want to buy with it and you have one year, and then in the middle of the year, you are attacked by the disease, you are going to take ,a portion of that money for your treatment, but for you to recover well and go back to the work, it will take time. Meaning as the time goes, you’ll continue spending that money. That is also a challenge that you may encounter, so instead of the money to grow, it will be reducing.

**NT: Now let me clarify for you that part, if I want to save money, what are the things that might hinder me from saving money? What are the challenge that I may face that might hinder me from making saving? They are like which ones?**

PM09: I as number nine, the challenge that has hindered me from saving money; is something like (Cough from the background)) school fees, secondly sickness may come in or I may be having a funeral. Those are the things that might hinder me from saving money.

**I: And now if you are someone who saves and you faced such, how can it be mended? You have, your target that you want to maintain, disease comes in, or funeral comes in or those other one, what you do for you to maintain your target.**

PM09: Now when… like me, number nine, when we make something like group where we save in, because mostly we savings that are minor, now as group, there is agreements within it like now, so and so has a funeral, so and so is sick and there is collection of small amount of money, after overcoming this problem and for me to come back to the truck, I will sit down and make my own calculation and realized that I had already messed by certain amount, and what I am remaining with in my hand is this, now I will save the way I use to save, I send little in the account and gain, So, I have overcome the problem, So after recovering, I again go back to the work. And I will continue saving for me to reach the target that I had.

**I: Number one has told us that may be you have your target, the workload has gone down so there is no money for you to save, so how can this challenge be addressed? How can it be addressed?**

PM02: I as number two my view is this, the workload is too low and I dint save what I expected of me so one of the days that I would be lucky and received some good amount of money, I calculate from backwards the amount of money that I had to save, for example if I was to save three hundred, two hundred and fifty and maybe I was only able to save one hundred each time, so if I received a good amount of money I have to do my calculations, and if it’s enough and remain with some balance, I do my calculations and top up from backwards at the same moment and I will be aware that I have reached my target.

**I: Is there anybody else who wants to add something? And now those who usually save, you had already told me that there are those who save and those who don’t save? Those that don’t save, what could be the reason why they don’t save? (Silence) Even here in our midlist you may find that one of us doesn’t save, and she can tell us the reason why she is not saving while others save.**

PM03: What do you mean?

**I: To those that don’t save money, they don’t save, what could be the reason why they don’t save? (Interviewer Clears throat)**

PM03: What makes them not to save (Interrupted by interviewer?)

**I: Number Three.**

PM03: I am number three, what makes them not to save, maybe they get (Money) and they buy something else that can be seen, they know “even if don’t save, I have bought this, I have bought the cow, I have bought the chicken” or I am making payments to someone and I may take my “field” (Land). I build myself a three roomed (house). That’s the reason she doesn’t have time to save because whenever she gets, they go to other activities.

**I: And buying the land, saving little by little money for buy a land is that not saving? It’s savings.**

PM03: Its savings (cross talk from the participants) I mean, when you have the land and you want to sell it to me, so I cannot save for myself, I save to you.

**I: Yes that would mean, you are making savings.**

PM03: Yes, that means that I save to you so that the target that you wanted was two hundred and fifty thousand, I will… I mean like I bring to you then you make your records and I also make my records.

**I: Okay, but for me I am asking about those who don’t save, why are they not saving? Number six?**

PM06+: (Silence) I am still thinking about that.

**I: Number six is still thinking, number four?**

PM04: (Silence)

**I: Number nine?**

PM09: (someone in the group cleared her, throat) I as number nine, about someone doesn’t save, I can say that maybe the income is low, the children are there and where there is a child you cannot just feel like saving the little amount that you received. Don’t the child needs food, and what about what has been eaten? Is it saved or not? (Laugh from the background). What has been eaten is eaten and gone it can never come back

**I: It disappears.**

PM09: It won’t come back, so I can just say that sometimes it’s the low income that makes someone not to save.

**I: Anybody else who wants to add, or have anything else?**

PM02: As number two I add that it depends with ones desires, she would always feel that she don’t have time to keep things, or like the time of her death…she feels that when she saves , it is somebody else who will benefit of it after her death. And maybe it’s because of her low income which is not even enough to the people who depend on her. So they are such sought of things.

R: I also want to add that maybe, someone doesn’t have the knowledge of savings, so she is not aware. (Unidentified participant)

**I: And the disadvantages; that come as a result of one not making savings are which ones?**

PM06: I as number six, may be that person is a *Rahulwe* (participant meant a beggar)

**I: What’s the meaning of *Rahu…*?**

PM06: I don’t even know how to put it, *Ramifuadhi*. I mean this is a person who feels that she is poor every time.

**I: Okay (Cross talk, interviewer and the participants)**

PM06: So when she receives, she better eats and die with it (to mean to spend it all during her time before she dies). She is like spending everything before her death, so she can’t save.

**I: Okay**

R: I as number five, that is the person who doesn’t think of the feature, he is someone who is not aware that problems exist, that’s why she cannot save money.

**I: The disadvantages of not saving are like which ones? The person who doesn’t save, the negative effects of not saving are like which ones? Disadvantages of not saving?**

PM05 I as number five, you may face problem at any time. That why it’s bad when you don’t save.

**I: Another person?**

PM02: I as number two, when she doesn’t save and face a problem… because the problem don’t consider whether you are saving or not saving, but it creating burdens to those who are close to her when sorting her out of that problem because she has nothing. So she creates burden to those that are within her surroundings.

**I: The disadvantage of not saving are like which ones?**

PM03: The disadvantage of not saving (Interrupted by the interviewer)

**I: Number three?**

PM03: I as number three, you may be in a problem, and the problem you may be facing can be, you get the news that your relative/ friend has been admitted and you don’t have any amount of money, and you dint save even a penny shillings, So how will you survive, It will just force you to go and look for money even if it would mean borrowing, or you may call friends of which in case she also do the same work, you tell her your problems for her to help you with the money, you go, that’s the problem of not saving, That’s the negative effect you may undergo through.

**I: Number seven, disadvantage for not making savings are like which one?**

PM07: Disadvantage of that you may have a problem, you may face a challenge any time and if you have not saved anything, it will not be easy on you on where to begin.

**I: Number eight? The negative effects for being that you are not making savings are like which ones?**

PM07: You may have a problem, the child may be sent from school for you give him/her money and maybe there is no food in the house, at that time you are also thinking of doing your hair and when you had saved, can be of advantage.

**I: And what’s the advantage of not saving, advantage? Yes, number four? No, number two has an information.**

PM02: For that there is no advantage, I anticipate.

**I: Mm, number two has refused that there is no advantage, number four?**

PM03: I support number two.

**I: The advantage that comes as a result of you not making savings is like which one? Number** nine?

PM09: In that, I as number nine, there is no advantage of saving something, there is no advantage that comes when you don’t save anything.

**I: Normally, what has positive impacts must have negative effects. And even if the advantages are outstanding, the disantantages must be few or even when the disadvantages are more, the advantages would be few. Now these few, what is it? The few advantage that the person who doesn’t save has is which one? The person who does not save has what kind of advantage?**

R3 I as number three, I would say that the person who doesn’t save undergo stress, maybe he lucked food in the house and when she checked on her account its zero, She feels like “ why should I continue with this work and I don’t save anything, like what is my expectation? My expectation should be, I go work sand save and sought myself out, so that’s how the person who does not save usually feels depressed.

**I: Okay, and where do sex workers save, that money that they save, where do you save it? Where do you save your money? Number seven? In the Phone, Mpesa, Mshuari**

PM07: Mpesa, Mshuari

**I: Mpesa and Mshuari, number eight, where do you save your money?**

PM08: Mshuari, Mary go round.

**I: Mshuari, Mary go round.**

R: I myself, I save my money at Mshuari, Mary go round and Mpesa.

PM03: Mary go round.

**I: Number four is Mary go round, number two?**

PM02: Mary go round.

**I: Number three?**

PM03: K.C.B Mpesa

**I: K.C.B Mpesa, number one?**

PM01: Phone and Mary go round.

**I: No, phone must be K.C.B… (Interrupted by the participant)**

PM01: Mpesa

**I: Mpesa, number six?**

PM06: Mpesa.

**I: Mpesa, number five?**

PM05: Mshuari and Mary go round

**I: Mshuari and Mary go round, why do we like Mshuari, Mpesa-Phone? Why do we prefer Mshuari, Mpesa? Number nine?**

PM09: The reason why we like saving money on phone is because we save there and sometimes when you have money you may also take a loan from them.

**I: Number seven,**

PM07: I as number seven, I like the phone because sometimes you may find someone, you make agreement and he pays you through phone.

**I: Mm, Okay, number two.**

PM02: That’s the one way that you trust.

**I: Number four?**

PM03: It’s easy to withdraw, any time you have a problem you withdraw it.

**I: For you, you said Chama.**

PM03: Chama, is easy to withdraw… you can easily withdraw you may have a problem so you withdraw it, spend it and you repay it with interest because it does earn interest.

**I: Why do you like saving money at the K.C.B?**

PM03: It’s because, I as number three, what made me to open an account with K.C.B Mpesa was because it’s easy since many people send to me money via Mpesa and I ,transfer to K.C.B. account.

**I: Who are the many people you are talking about?**

PM03: The people I visits.

**I: Customers?**

PM03: Yes

**I: Okay, number six?**

PM06: The reason, I as number six the reason why we save money in Mpesa, sometimes someone may call, you may receive a phone call (Interrupted by interviewer) and maybe you do not have any cash to go and buy airtime, it will force you to withdraw some money from that account so that you buy airtime and call for you to get what he intended to tell you.

**I: Okay when you ware speaking I had you saying, Number nine told me that when she leaves food, she leaves enough food for the children so that even if a visitor would come, he will get food to eat. And the question I want to ask right now is that (Hitting sound) is there a time that a sex worker leads a high standards live while her income remain low. I mean that your income is so low but you lead a high standard life. Do that question understandable? (Participants murmur, yes from the background) Mmh… Is there any time that there is no money but you just increase your living standards, you make sure that the children eats well, they go to good schools for people to see that you live well. Something of the sort, number two have you understood that question? Do the sex workers live a life that is higher than her income?**

PM03: No.

**I: Number four has refused, number nine?**

PM09: No.

**I: Even you, you are refusing? Number seven?**

PM07: No

**I: Number five?**

PM05 Mmh (to mean yes)

**I: sex worker live a life that is of higher standards than her income? Mh, please say it, clarify it well.**

PM05 She may higher living standard when the income is low, so the little that she gets, she it all, because you may find that one has two thousand shillings and she seems dirty but you may find that one gets one thousand and there is a way in which she plans herself, do her business and at the end of the day she become cleaner than even the person that who had two thousand.

**I: Okay, Now, where… why does that happen?**

PM05 It depends on how you settle it by yourself.

**I: Okay, Could it be that a sex worker may earn low amount of money but she higher life style, life style, or her expenses are high? Number four has refused, number seven has refused, number nine too has refused, number six?**

PM06: I as number six, your life can be depending on how you have been saving money, save part of your money.

**I: Mmh, okay, but do we, do the sex workers borrow?**

R: Mmh

**I: Mmh, who has made that sound “Mmh”**

R: Borrowing what? Is it food or… (Unidentified participant)

**I: Depts.**

R: No

**I: You don’t even borrow from Mshuari, *Fuliza*? (Borrowing that is done throw Mpesa and the payment is done through deductions whenever any amount is deposited in an Mpesa account)**

R: We just dint understand the question as in we thought that borrowing in terms of our work or?

**I: Okay (Laughers from the background, by the participants) Sorry, Okay you thought that you are going to work on a debt?**

R: Yes (Cross talk, the participants)

**I: No, (Laughers by the participants) it’s not that you have gone to work on debt, that that person does not pay you work on debt, (interrupted by interviewer)**

R: Loan?

**I: Even if it’s not loan but even just that you own someone a debt, number seven? Where do people borrow from? Where do you borrow from or who do you receives these debts from?**

PM07: As for me, I borrow from, Mshuari.

**I: Mmh, number eight do you usually borrow?**

PM08 Yes

**I: Where do you usually borrow from?**

PM08 Mshuari and Chama.

**I: Mshuari and Chama, okay number nine?**

PM09: Even me I borrow from Mshuari and Chama. (Voice of people from the background)

**I: From Mary go round, number two? Do you usually borrow?**

PM02: Yes, though debt has more meaning, it could be that you dint have money, and I don’t want to spend the ones I have saved and I go and borrow something from the shop, that’s still, that’s still debt. I even see a nice dress from the customer, I dint want to use this money (saving) and I dint have money and therefore I borrow it. I therefore have debt, because I have borrowed the cloth. That is also debt.

**I: So you also borrow such?**

R: mmh (participants’ cross talk)

**I: Number three? Do sex workers usually have debt?**

PM03: I as number three, to have debt is a must.

**I: Debt from which source? What’s the source of that debt?**

PM03: From the phone, you may take a loan from K.C.B Mpesa. You may take a loan from there.

**I: Okay, number one?**

PM01: Even me I do take a loan from Mshuari, and even food, I go to the shop and I take on debt when I don’t have money and I will pay.

**I: Okay, apart from the debt from the shop (cough from the background) because at the shop we have debs for the food staffs like bread, or from stalls we borrow the sardine, tomatoes, is that not so? Those will be eaten, so the money that we borrow from the phone, they are always… what do we usually do with them?**

PM09: I as number nine, a times you go to work, the work is not good (No customers or the customers are view) it forces you to, the child needs money for the school fees, or you want to pay for rent somewhere, it forces you to go and borrow, you finish the business that you had, when you get you will pay back.

**I: Okay, so at the time you are returning, where will you get it from?**

PM09: For that am hoping for, for me to go and borrow, I am hoping for a target of which I know that if I succeed in it, then I will refund it.

**I: Okay, in this same job?**

PM09: Mmh

**I: Number seven? You mostly borrow money from the phone for which purpose?**

PM07: When I have an emergency like school fees.

**I: Okay, Number four? When you borrow money from Mary go round, what do you borrow it for?**

PM03:: When I borrow money from Chama, I may be having a patient, or maybe I have received a phone call that my brother has been admitted in the hospital, sometimes my mother is sick and has been rushed to the hospital, I borrow from Mary go round knowing that at the end of the month, I know I shall have gotten that money and I take it back.

**I: Where do you get it from?**

PM03: From the work I do.

I: And what do sex workers usually do for them to increase their income, mmh, so that they may increase their earnings? You know normally you may find, when you leave in the late evening and you know ;that a times ;you may find one thousand shillings and maybe you have a feeling that this one thousand is too low as compared to the nature of your job, you feel that with this your job, you want to increase it. What do sex workers usually do to (Interrupted by the participants) Cross Talk …by the participants, have you understood that question number three? You have a low income and you know, I mean you do get one thousand and you want it to increase (the income) what do sex workers usually do when they want their income to increase? Number nine was speaking.

PM09: Mh,

**I: Or who was speaking,**

PM04: I as number four? (Phone ringing) For my income to increase, I need to be clean and also have must so have to communicate well. I don’t be the Kind of a person who speaks annoying words for me to attract customers, you bee a clean person and also communicate nicely, so you are like the person who seduces someone.

**I: That’s number four, another person?**

PM09: I as number nine, for me to (phone ringing) for me to increase my earnings and for it reach appoint that if I came back with one thousand and tomorrow I want to come back with five thousand or three thousand, I may try and meet someone to give me even some thousands, I may also try and get another person, in a day I may also do it like how many times a night?

R: (Cross talked inaudible statement)

PM09: For me to get three thousand, then already I have increased my earnings form one thousand to three thousand.

**I: Okay and it could be that when we want out income to increase, do we put ourselves at high risks of acquiring HIV?**

PM02: Mmh.

I: Number two? In which way?

PM02: In a way that you have to have several customers even in one night. So you know when you have several customers, you are not aware of their HIV status, you don’t care who he is, what you care about is only that your earnings to increase from one thousand to three thousand five hundred. S you don’t know, you don’t care about yourself.

I: Okay.

PM01: I as number one as I add on something, when you want your earnings to be high and when someone comes to you that she don’t want to use a protection, you will just a accept.

I: Mmh, another person?

PM05 I as number five, when you go with several people (have sex with many), there are those that uses condom, there are others who also don’t want, so you will acquire HIV because you need money so as your income to increase.

**I: s when, there are no clients, there is a time that you may go but the clients are not there, what do you usually do? You have gone to the “field” but there are no customers, no one is available for you, any one that you give signal is busy, and what do sex workers usually do in such a time? Number two? So when you go to the work at night and you expect to get a client and they are not there (Laughers from the background), you call them but no one comes, there is no income. What do you usually do?**

PM03:: I as number three, when there are no clients, there is no need of going back, I will just stay, I won’t luck, may be when it reaches at around ten or around eleven at night I may get, but I know at twelve midnight or one at night I must get. You can’t luck, sex work you can’t luck.

**I: When it has gone to the extreme that there are no customers, you have already in the cold the** whole night (Participants are murmuring in the background) there are no customers?

PM02: I as number two, it’s not easy to luck completely, Circumstances of lucky completely is difficult, and it’s that bad you are not sent away from your residence you just come back.

**I: Okay**

PM02: Mmh

PM05 I as number five, like how it is that a times we usually go to =Ugunja=, when it’s that difficult, your boarder vehicle you go to = Kisumu=, you cannot luck in that area. (laughers and murmuring).

**I: The same night or the next, night? (Laughers)**

PM05 That very night, you take a motor bike to Siaya, in town you can’t luck?

**I: So you change the town?**

PM05 Mmh

**I: Other person again?**

PM02: I as number two, sometimes I may have even dressed so well that even a motorist that I am bordering, (participants laugh), we may not reach where we were to going.

**I: Number one?**

PM01:` A that time, even a phone can operate, you are already in the field and there are no customers but you always have some of your customers, you have to call them and convince them so that you may not go without (without a customer) that night.

**I: And is it something that you may know prier that today, the field is bad (not good, no customers) or it something that you just realize once you pare there?**

PM05 I as number five, I may not know if the field won’t be good.

**I: You may not know that this month the income will be low?**

PM05 Mmh, it’s something that just come without your knowledge?

**I: We had already said that we do borrow and is there amount of money that is placed on us as a limit that you cannot exceed. Number six? When taking a loan, is there a limit that you are supposed to take or you can take any amount or do you have a target that for me I can only take a certain amount of money or that you cannot exceed this amount.**

PM03: I as number three, ,my loan limit, in my K.C.B Mpesa, I am aloud to take up to ten thousand, so I do take that less like when my loan limit is ten thousand, I may even ,take like five hundred, one thousand, or even three hundred.

**I: Okay that is number three, she is not taking more than one thousand? Okay, number one?**

PM01: Even me I don’t always exceed three thousand.

**I: She don’t exceed three thousand. Number two?**

PM02 Its just like one may just wish to have a debt but I don’t usually exceed five hundred?

**I: you take up to five hundred?**

PM02: Mmh, five hundred up to one thousand

**I: Who usually feels like taking one thousand and above? Number seven?**

PM07: One thousand to two thousand?

**I: One thousand to two thousand, number eight?**

PM08 Two thousand.

**I: number five?**

PM05 Six

**I: Six thousand?**

PM05 Mh

**I: Number nine?**

PM09: I as number nine, for you to borrow, I think that you need to ask yourself first that this debt if I borrow even ten thousand or twenty thousand, which problem am I going to sort out, and I have to be sure of a certain target that I am sure that I may get ten thousand or twenty which will allow me to pay back the debt, so debt borrowing depends on how you can get it, you have to be aware of how you will repay it.

**I: And now, this our job of sex work, do we usually have a mindset that we may stop it? (Silence) is the question understood, do you have a mindset that this work I am doing but in the next five years or in the next ten years I feel that I may stop, is that kind of thinking usually clicks on your mind? You may stop sex work? Does that usually come to the minds of sex workers?**

R: Mmh (cross talk by the participants)

**I: Mmh has been made in chorus, whom should I start with? Number four, please clarify for me?**

PM03:: Yes, this is the thinking that comes across the minds of sex workers because even every work, it usually reaches the point that now you are tired, so the age that they have already reached, even you, you understands that you cannot continue with this job that I am doing, so the body needs a rest, so that’s where savings come in.

**I: Okay, it’s like number two has some information that is so major?**

PM02: Work, and everyone in all types of work that you do it is a must that one day you will retire. Like for this one you would wish that you retired but it’s also sweet and you may do it for so long and tear and weakness of the body is what may make you stop, though it’s a must that you will stop it.

**I: Okay, number eight?**

PM08: It depends with the age of someone, you may say “now I have to stop it” but when you still have the energy (feels strong) it’s difficult to stop (it’s not an easy decision to make). You would just continue with it.

**I: This work of sex work, do the sex workers usually think that one day they will stop it?**

PM03: I as number three, they usually stop.

**I: Mmh**

R3 When they reach the age of sixty years, they do stop, at that time you feel tired.

**I: Okay, and is it something that people would sit down and discuss or you just think about it wherever you are as an individual or there is a hot stop that you do meet, does such discussions takes place at the hot sport? Number nine, please tell me.**

PM09: The reason why, I as number nine, I do feel that there is, you join sex work because you want to educate the children, you want to… the husband abandoned me, ABCAD, (to mean and some more reasons) and so you have just made it in your mind like I myself when am talking for me personally, there is an extent that I am targeting that after reaching a certain level with my child, I personally I have seen that in my account, it can help me open something ( My business) so that I may sit down, but one day I am sure that I will stop. Yes, but before I accomplish my mission I cannot leave it because it helps me. Before I finish my missions< I still can’t stop it, though I hope that I will stop it.

**I: Number seven?**

PM07: I support what number seven has said.

**I: Mmh**

PM07: It’s not something which is permanent, you will work and stop of which there is a target that you can set for yourself that after I have done this and that then you will stop it.

**I: Mmh, number four?**

PM03: Just as number one, number nine has said it.

**I: And you told me that it’s something that sometimes, you usually talk about at the hotspots where you stand, what causes, what triggers such information to be started, how does it begin? Does someone just starts that “Waa? Me I can stop this work” or how does it began? When you are at the hot sport where you work, I am asking if that discussion for stopping sex work use to be, and if it do have it, what triggers it to be started? What usually happen for people to start taking about stopping sex work.**

PM03:: I as number three, what makes us to sit and have discussion is that one usually considers her age, when you already reach like the age of fifty years, there are those who get tired of standing at the road, and then that’s the time that I volunteer to speech with my colleague and when my other colleagues are still there, they may say “ Even us we are supporting you (supporting her decision) being that we are seeing you are tired, you rest a bit as you leave us to continue with this work.

PM07: I as number seven, I may say that someone may make decision to stop based that she is harassed. May be she got a customer who work with her inconsiderately (in a bad way), so this may make her loose hope.

**I: Mmh, so when you work, when the customer works with you inconsiderately, do you come back and share here in the group or you only go with it in your household.**

PM07: you may share it with a friend that you are used to.

**I: Okay, number two, do you have any information?**

PM02: No, I just wanted to support what number seven has said, because may be you just left for job as normally, and when you reach at the field you meat even some three people who have the intention of harming you, they are fully aware that, that is the place you normally do that “thing”( sex work), they had already planned it and all of them are pointing at you one person, they say that “ this lady we will do for her a certain thing”, so when you reach there before your colleagues reaches or maybe they had already reached and gotten work, you just find yourself being carried to somewhere where they can do to you something you dint expect. If it happens that you will still be alive, you would say that “even if this is the work that is helping me, I am leaving it”.

**I: Okay, You have already told me some of the reasons as to why one would stop it, you have been harass, not so? Client does to you a bad thing, what can also make someone to have the thinking of stopping sex work? Number thee has said that you have reached the old age? What else may make it possible for one to think that “I am tired or even feel discouraged and may want to stop?**

PM01: I as number one, something else that may make someone think that she now needs to stop work is that when you have children and your children has already grown up, when you think that when you go to the field and maybe someone may meat you and may even want to meet your older children (may have sex with you and again have sex with your older children.

**I: Mmh, okay, this means that stopping it is not something that one plan that in the year two thousand and twenty four I will stop it, or in two thousand and twenty three December, I will stop it, or it’s something that comes without planning,**

PM05: I as number five, people are different, there are those who think about it in that manner, there are some too who are energetic and will only stop it when they are already old, and there are some people who also just work until… so it depends with everyone opinion.

**I: Another person, is it something that is being planned or it’s something that you just find that you have already stopped it?**

PM09: I as number nine as I reply to that, Sometimes you may just find that you have stopped it depending that may be you met a client who don’t respect the agreement, there are some people who after finishing (Having sex) probably you agreed that he will give you even five thousand but he gives you something like three thousand and this would Couse disagreement and you may lose hope and you just stop it unexpectedly. (Cough from the background) And there is one of whom you may tell “For me, this job helps me and even that my younger child, after reaching a certain level, I will stop it” so you know it’s something that is in your mind, because you are aware your reasons for doing sex work…after my child finishes his education or even after finishing purchasing the plot that I have been buying then I would stop.

**I: Okay, number three has told us that there is an age that when someone reaches (participant interrupted).**

PM03: You stop.

**I: Those who stop, is it because of age or there are some other things (cough from the background) that would make them stop?**

PM03: I as number three, age also contributes to someone decision of stopping (sex work) and there are others things that may make you stop. You may find a client who you don’t reach agreement with, you may find that he is vigorous during sex and maybe you want the one who is soft, he may scratch you with a lot of energy and this may make you stop (sex work).

**I: Okay but in which age does most people stop (Sex work)? Those that stop. She has already said** **fifty, sixty?**

R: Mmh (Cross talk from the participants).

**I: Mmh, which age do you know of? Number four? I’m realizing that nothing, nothing is so common.**

PM05: (Laugh) I as number five, it depend, it depends, and you know the energy level also differs. Some stops at sixty, some at fifty and there are some who stops at forty, and therefore it depends with someone’s energy. So she stops because of her age or he is tired?

R: Age. (Participant who has not been identified)

PM05: She is tired, there are some that you will find to be energetic than forty, so she uses her body so the body gets weary, she would stop because she would feel weary in the body.

**I: That was number five, another person again, most people stop it at what age?**

PM03:: (Whispers from the back ground, fifty, sixty)I as number four, some people stop it at fifty, but normally, when you have already fifty years, you feel when your body, you cannot compare your body with someone who is thirty years, Mmh, you feel week physically, So it will force you to rest.

**I: Number one you have any other information?**

PM01: I don’t have any information.

PM03: So what I know is that when you have already reached the age of fifty, or even late forties, you may feel like “I’m already tired” because at this time you are not sexually active. Mmh.

**I: Mmh, and those that stops (sex work) what types of job do they usually do? We are about to finish, (participants laugh) you know there is away in which the eye communicates ( non-verbal),I am seeing your eyes, we are about to finish. So just be patient, which kind of jobs can the sex workers do? And where do they do them? Number seven? What kind of jobs can you do?**

PM07: When the jobs could be… like myself I am a sex worker, so you work and save and when you decide to stop, you can start a business with the money that you used to save.

**I: Business, Where does that business being performed?**

PM07: In any Centre that is next to you.

**I: Any Centre? Number Eight? What can a sex worker do?**

PM08: She may start a business with the money that she use to save, she may work in his farms< gardens

**I: Mmh, she may do farming, any other thing apart from business (Cough)**

PM08: another work that she may do, you can also take good care of your chicken using your money that you saved, you can do any self-work of which you will be self-employed.

**I: Mmh, number four?**

PM03: She can be in an advisor for the other sex workers.

**I: But will that earn her money that she may survive on?**

R: (None identified participant) and I wanted to ask her, when you will be an advisor for the other sex workers, will you be paid? (Participants laugh)

**I: Number two? Which kind of work will earn you money that you will be surviving on after stopping involving yourself in sex work is like which ones?**

PM02: Just as my colleagues have said, it can be a business, ;and may be among your children god had blessed one and he may have said that “ mother you have suffered for us for so long, so now rest and eat from my work” so you just rest and you are being brought for what you want.

**I: When someone stops working as a sex work, what kind of jobs can she do?**

PM03: Business.

**I: Business, number one?**

PM01: It’s just business.

**I: Business. Mmh, is there any change when they stop (Sex work) I mean the change that you may experience when you were doing sex work and you stop? And how do they cope with that change? For example how we were saying that when you go, you are going to get some money? So you stop it, you are now doing business, you purchase the vegetables to sell but you dint make any sale? I hope there is some slight change?**

R: Mmh (cross talk by the participants).

**I: What do them do, how do they cope? When someone stops sex work, you had already told me that in sex work ,people usually get… when you go for sex work, you may get money, but then you have told me that one can leave sex work, she can do business. So like I was asking this, you have ventured in business for the vegetables, you purchased the vegetables costing two hundred shillings, you come with it to the stall, its already late evening but you haven’t made any sale, I hope there is some changes?**

R: Mmh (cross talk, non-identified participants)

**I: So how do they cope? Because this is the money that you are sure you will get, but now this money is the money that you get in bit and sometimes you may luck, so how do someone copes, Number two?**

PM02: That’s why when you were working and was making savings, the money that you ware saving would help you, the day that the business did not do well, you have somewhere you can go and get something from and live continues.

PM05: I as number five, when you are already an old woman, you have already toped that job and have gone home, you need to build yourself a nice house that you stay in, you built a place for the chickens so you just do a business for the chickens, you do farming and these other little things.

**I: Mmh, and do you know some of the sex workers who have stopped this job and come back to it? Whispers from the background followed by some sounds, and then silence) you know those who were doing this job but stopped and then came back to it again? (Some people are talking from the background) We are about to finish, the remaining parts are few, just respond faster so that we may finish)**

PM03: I as number three, when someone stops that her age is fifty or sixty, she cannot come back to it, but in case she stops at the age of forty, thirty five, for that she may say that the work I was doing was better than even the vegetables I am seated with down here, but for the person who has already reached fifty or sixty cannot come back.

**I: Okay, what would hinder someone of fifty or sixty years from coming back?**

PM03: Their age would not allow them to do that kind of work, yes, their energy has reduced?

**I: What about thirty?**

PM03: Thirty to forty? She still have the energy, she would be thinking that that work was nice and I am sure that I would get (Money). It was something that when I would leave home to go for, it was a must that I come back with something and those days my lifestyle was good.

**I: Okay, another person? Are you aware of those who stopped the sex work and came back to it again? Why are they coming back? Number four?**

PM03: She may go and come back, if her age is between thirty to forty, she may just come back because she may reflect on her past and have a view of the life she lives, sometimes like selling, she would sit by the road side, the day goes without making sales, she comes back without money, though she was used to going to work and would not even luck five hundred, she comes back with some five hundred of which she would begin with the next day. But for now she has nothing, it will force her to go back to the “field” (Sex work).

**I: Mmh, anything else that would make someone to come back? Number nine? (Phone ringing in the background).**

PM09: I as number nine ,may be you started it when you ware thirty, thirty one, when you considered how you remained lonely and maybe there was a problem between you and the husband, husband maybe went to those his prostitutes< So you get into this work and may do it for even a period of five years, he again remembers you and coming back to you, and you were still young, you must stop, take a break, and you will be like he is back so I can’t go on with this my job. You stop even for one year or even two years and you learn that that problem has come back again, he leaves you again and go back again (to his prostitutes) what would hinder me from going back?(Cross talk ,participant and interviewer).

**I: So when you are back, what are the problems that you are likely to face? The negative things you would meet when you come back are like which ones?**

PM09: When you are back after the rest, the problems you may face is that, you don’t have customers and it would take you time to reach the level that you were in? So those are the challenges that we experience when one is back.

**I: Number two, the problems that you experience when you come back are like which ones? When you stop sex work, you take a break and then you come back again, you’ll have what types of problems?**

PM02: (noise from the back ground) the problem that would make me come back (Interrupted by the interviewer)

**I: Which kinds of problems you would experience when you came back?**

PM02: When you come back to that work?

**I: Yes.**

PM02: Because you have taken a lot of time, (Cough from the background) you have stayed for long and you have rested and now you are not used to it now.

**I: Okay, number two tell me the problems that you will face when you come back are like which ones? You had already stop as said by number nine, your husband comes back and you felt like taking a break mean while he would still be there, he again take his leave and go, you come back, what are the problems you would face?**

PM02: The problem she may face, one of them is that your customers that you use to known would not be available (Hitting sound) so you will start looking for customers a fresh. As you are looking for new customers, you may not know what may come your way. Because you are coming back while desperate because you come in a poverty state? So anything that you would meat, your mind may not be able to think, you may not be able to think that you may contract disease, you don’t think of that. What you care about is just that you are back again, you lower the price for the work to continue.

**I: And what about the good things that they may encounter when they are back are like which ones? You said you stopped and come back again. The positive things you would encounter are like which ones, number seven?**

PM07: The good things you may encounter are like, those that have never seen you may think that you are a new person so you are the one that most people would want.

**I: Because you are a fresher?**

PM07: Mmh.

**I: Okay number eight?**

PM08: Number eight, when you are back (Laugh)

**I: You are back to the field.**

PM08: I don’t think if you can luck getting what you want even if the customers that you had are lost (not available) when you go, you are a visitor. It’s like you may meet someone, and he gives (money) you ones than the one they you are in a relationship with. You just start little by little and you continues, and the life continues.

**I: Number six, the good things that the person who stopped this work, come to it, may get?**

PM06:: Just as number seven had said it.

**I: Okay, as we were communicating, some of you told me that this work is helping them of which they cannot stop it right now of which they said that would only stop after accomplishing some things that they still want to be accomplished. Not so?**

R: Mmh (Cross talk, the participants)

**I: Now when we leave alone the ones we had already said, other things that want to accomplish before we stop sex work are like which ones? (Murmuring at the background) Number seven? What are the things that you feel that “for me this and this and this, I must a accomplish because it’s this work that earns me money for sorting them out”**

PM07: I, It’s until my children finishes school?

**I: Taking the children to school, number eight?**

PM08: For me also it’s until my children finishes school.

**I: Number nine?**

PM09: Its school, and maybe I have a parcel of land which I am in the process of buying, the children must finish school and I must also accomplish buying that parcel of land.

**I: Mmh, you are making payments for the parcel of land, Number four?**

PM03: School fees.

**I: School fees, mmh, number two?**

PM02: I as number two it’s the school and making sure that after stopping this work and my future life… how am I going to survive? Yes that’s that calculation I am straggling with, the children to finish school and also when I shall stop this my work, how shall I live? Will I live just the way I used to leave or I shall live while suffering, yes, so I must calculate, I must stop after making calculations? (Participant meant working on savings).

**I: I am asking, you had already told me that there are things you still want to accomplish before you stop sex work, so you as an individual, what do you want to a accomplish before you stop sex work?**

PM03: I as number three, I want to stop sex work after my child has finished school and after buying a piece of land.

**I: Number nine has said that she has already started paying little by little for the land and those that are mentioning land, is it something that you had already stated paying or you are just expecting? Like you, number three?**

PM03: I as number three, what I am expecting so much is that Land and where I shall be doing my business, when I shall already saves some little.

**I: Number one?**

PM01: I too my children have not yet finishes school and I wanted them to finish first and then there was a plot of which I had already started building, I also want to finish it.

**I: We are almost coming to an end, for the last five years or last ten years, are you aware of those that have stopped sex work? She left for good?**

PM05: No

**I: Number five have not seen the person who stopped (sex work) number four?**

PM03: No

**I: Not yet, number nine?**

PM09: For me since I started I have never seen someone who had stopped.

**I: Mmh, it so sweet to be stopped? (Participants laugh)Number eight? Any one has stopped?**

PM08: I have never seen.

**I: Number seven?**

PM07: No

**I: Number three, since you began work, or for the last five years or ten, do you know of anyone who have stopped sex work?**

PM03: I as number three, I am not aware of such a person.

PM01: Number one? There is a friend of mine who stopped.

**I: What was the reason for her to stop or what made it easier for her to stop?**

PM01: She was harassed.

**I: Okay, she was harassed? Okay. Number five for you, you said you have never seen anyone who stopped? (Participant meant sex work Sex work)**

PM05: Yes.

**I: And now as I head explained to you *Jitegemee*, you can remember or the information that we have shared has erased them?**

R: I can remember, I can remember (Cross talk, non-identified participant)

**I: Mmh, now*, Jitegemee* is for savings, not so? Savings so that you may be able to refuse engaging in sex work that puts you at high risk of contracting HIV, Mmh (Sound of a moving vehicle). Saving so that when you are tired (noise at the background) or when you are sick you may take an off for you to rest. Mmh, the money that you are saving is yours and it’s not like that for the Mary go round that has a target. You are the one who decides on the money that you want to save, you are the one withdraws it any time that you want to withdraw it. And when you withdraw it there is nothing like interest charged or returning with a profit. That’s the difference that it has with Mary go round. So do you think that such a program… can sex workers love it? Start by saying I number… we are about to come to an end, Number four?**

PM03: I as number four, we may like it?

**I: What kind of sex workers would like it? What kind of sex workers would like *Jitegemee*? Number two?**

PM02: I as number two, I feel that everyone can like it.

**I: Mmh, everyone may like it and which kind of people may not like it? Number seven?**

PM07: I don’t feel that there are some people who might not like it.

**I: For you, you feel that everyone would like it? Number nine?**

PM09: Mmh, I feel that everyone will like it.

**I: This *Jitegemee* that you talk about, is there some sex workers that would not like it? (Heating sound from the background)**

PM03: There are those who would like it and those that would not,

**I: For those that would not like it, why would they not like it?**

PM03: Because they feel that this work very difficult to them, so they better do another job apart from sex work.

**I: Mmh, I am taking about *Jitegemee,* are there sex workers that would like *Jitegemee*? (People speaking from the background)**

PM03: Yes.

**I: Okay, number one, those that will *like Jitegemee*, Why would they like it?**

R1; Because it teaches them how they can be self-reliance, without depending on someone, It teaches you how you can save.

**I: Just continue?**

PM01: (sound in the background, participant clears throat) you get the knowledge of which you can save. (Silent)

**I: Number seven also said that every person would like it, what do you think would be the reason why people would like it?**

PM07: Because its money that when you want it you just take it and even when you take it, there is no interest that you are charged.

**I: Number nine, even you, you said that everyone would like it, what could be the reason for everyone to like it.**

PM09: I just support how number seven has said it, mmh.

**I: number four?**

PM09: Yes, people would like it because it teaches you how you can get your money, it also helps you how you can prevent yourself from getting diseases that are sex related.

**I: Number two why would people love it?**

PM02: People may like it because now you don’t contact more diseases, you know how to prevent yourself from them, the money that you take and you know how to take it back, you know how to save and even to return back.

**I: Mmh, so you, number ten, number seven, when you have friends, you tell them about *Jitegemee*, out of ten, how many people would accept?**

PM07: Around seven.

I: Seven? The three people that may not accept, what would they not accept?

PM07: Three people that would not accept are people that always when they are told about something, it’s usually not easy for them to accept.

**I: Mmh, number eight, you have ten of your friends, you told then, not only friends only, but your ten friends that are sex workers, you told them about *Jitegemee*, how many people will accept *jitegemee*.**

PM08: Eight.

**I: Eight the remaining two would not accept for what reason?**

PM08: There is fear and embarrassment.

**I: Why embarrassment?**

PM08: For her to be enrolled.

**I: What would cause embarrassment when she is enrolled?**

PM08: Some people usually fear being in place where people are.

**I: Mmh, okay, number nine, when you talk to ten people, how many would go to *Jitegemee*?**

PM09: Among ten people, I as number nine, if they are ten people that I have told about it and all of them are sex workers, I don’t feel anyone would refuse?

**I: They would all accept.**

PM09: They would all accept.

PM03: Number four, I support number nine?

**I: Mmh, number two?**

PM02: People don’t usually agrees at once, you may have ten of friends, and out of the 10 of them, five would accept while five will be sitting back just observing, so if they see that you are doing well and would say that “what number 02 was talking about was something that we ought to go to all of us.

**I: And why would they ask me again?**

PM02: You just know humans feelings.

**I: Okay, if you have ten friends, you explain to them about *Jitegemee* which is a bout savings, how many people would accept it?**

PM03: Three people.

**I: Those five that will refuse will have which reason?**

PM03: They will refuse because they would feel it’s difficult?

**I: What difficulty?**

PM03: For the *Jitegemee.*

**I: How would it be difficult?**

PM03: It means, like if I talk to five and the five have accepted *Jitegemee*, so these five may just say “okay, but I don’t trust”.

**I: They will just refuse without any reason?**

PM03: They would refuse because it may not be easy for them.

**I: Mmh, number one, out of ten?**

PM03: Out of ten that I talk with and are al sex workers, they will accept *Jitegemee*.

**I: Mmh, okay number six?**

PM06: For me I just had what number one have just said.

**I: You had the point that number one have said?**

PM06: The same point as illustrated by number one. (Murmuring and the voice of a child. in the background).

I: Okay, it’s okay, we are just about to end, and the remaining portion is very small (participants laugh), its true I am not lying, we are remaining with two, three questions and we finish. Yes, now *Jitegemee*, what can we do so that we would make sex workers to accept it? To accept it even more? We have said that, number nine have said that she feels that everyone is going to accept it, number six everyone will accept it, number one, but there are some of us who felt that some will accept it, others will not. Number five has told us really?

R: Number five have not told us (non-identified participant)

**I: Number five tell us.**

PM05: They will all accept.

**I: They will all accept, and if we need those who don’t accept to accept it too, what can we do for them to accept? (Participants talking from the background) Let’s come back and finish.**

PM01: I as number one, I feel that we teach each other information about saving money.

**I: Teaching, another person? (Silence) How would this teaching be done?**

PM01: We are going to teach them as we have been taught here.

**I: Mmh, another person?**

PM02: Mmh, I as number two, I am just supporting what number one has said because they had refused but when they saw when we were doing well, we also need to continue talking with them so that we may bring them here, they may just accept it and be enrolled.

**I: Talking with them, another person? Number five? What can we do (interrupted by the response)**

PM05: I as number five, You know someone who is a woman knows how to talk with a man and he just accept even if he had refused and she would just come back without being forced.

**I: But *Jitegemee* is just ours as women.**

PM05: But when she had already known and you tell her about *Jitegemee* she won’t refuse. She will just listen to you. (Murmuring in the background).

**I: Another person, what can we do so that we would make many to accept *Jitegemee?* Number eight?**

PM08: Mmh?

**I: Number eight has already reached home (Participant meant that number eight was absent minded, participants laugh)**

PM08: Another thing just as number one, is that ourselves, we must start putting in practice these things that we have been taught, So when they find them good to them, they will find ways to join.

**I: So for us who are going to plan the operations of *Jitegemee*, what do we need include in the programs of *Jitegemee* for it to work well for the sex workers? (Murmuring at the background).Mmh, let’s all answer this for us to finish faster. The programs for *Jitegemee*, us as the people who are Making them, how should we make it to operate so that the sex workers would accept it. Is that question understandable?**

R: Mh (participants cross talked)

**I: It’s understandable? (Murmuring from the background) I want to ask in English too. Mmh, what do *Jitegemee* comprise of for it to be acceptable? (Child crying in the background) What are the things that *Jitegemee* should comprise of for you to accept it? Is it understandable?**

R: Mmh (cross talk by the participants).

**I: So let’s do it in this manner, as we start, number six?**

R: Start from that side. (Laugh from the back ground)

**I: Who is ready? Number two? (Laugh by the participant). Just like when you go to discussions of** **religious groups, usually people do seat and set the rules that lets do it in this manner, not so? We are doing one, two, three, four, five, (Laugh from the background). Now for *Jitegemee*, how do we set it? How should we make it?**

PM02: I as my perception, at the time that you started teaching us you said that for it, there is no money that it helps us with?

**I: Yes.**

PM02: And for it to be something nice and attractive, It must have little income that we find when we go there. Yes, now you know when you go to tell someone that you got a certain thing, then he will ask you that “how does it help you”. And if you exaggerate it and say that when you go there, you are given five thousand each just for the beginning, it’s something that may lift you up.

**I: That’s number one, number two, number one also wanted to talk?**

PM01: What you have said is just what I wanted to say but I can add that if you may, *Jitegemee* may make a program for educating, so that after being taught even business skills or even how we’ve been saving and you give us something small that we may begin with for *Jitegemee* to continue. This would mean that there is something that unites us together. So we make it to be something like Chama so that even those who are not part of this would come and join us. I am asking, when we are making operations for *Jitegemee*, how would we make it to operate so that the people would accept it?

PM03: We, we are, I don’t know how to say it.

**I: She is still thinking, as she is still thinking, number four tell us, you want to say, (laugh from the background) please say so that we may finish. What do you say ( Murmuring from the background) number two has said that we put some money aside, number one has said that we teach people, What do you personally tell us to do?**

PM03: We may make it as, as sports.

**I: Mmh?**

PM03: We may even want to make group and do something that is entertaining, it makes people to long for coming back to the group.

**I: Mmh, number nine?**

PM09: I as number nine, for it to be made in a way that when you tell someone about it, she gets to have a moral of following you, but only if it has teachings combined with something small (money) of which when you will be done and wants to leave, you leave with.

**I: Mmh.**

PM09: Because the faith of human is too small, for you to accept that you are going to sit there up to what time, but what will sitting there gained you, just as the way other participants have talked.

**I: Anybody else who wants to add something again?**

PM06:: I as number six, I want to say that there are some people who are part of us and we may go and explain to them , they will be like at what time do you go there, how long did you take and at what time did you leave? You may go and tell her that for us we were taught well, and after the teachings there was something that we were given,( child crying), This is something that when she hears of, it will excites her and would want to join.

I: Okay, and how I had elaborated for you *Jitegemee*, what do you think sex workers are not going to like in the *Jitegemee?* (Child crying in the background), I hope you still remember it? What would they not like? (Silence) the house is silent, number two has some information. (Participants laugh), Mmh

PM02: Making saving.

I: Making your own savings (money), you may take it any time and if you take it you don’t return it with a profit. Yes, you can take it in the morning or at night, any time that you would want it, you would take it. That’s (Cough at the background) *jitegemee.* And there is nothing you are given to save. What will the sex workers not like about it? (Whispers at the background) number one? Mmh, someone was talking?

PM06: I as number six, we would not like that there would be money that goes out( expenditure) but no money would be coming in ( income). That is what they will not like. We would not like that, if she takes away little amount of money, there should be some little income to boost it.

I: Mmh, do you have appoint, can we answer these questions so that we may finish (murmuring at the background, Laugh). What don’t sex workers need in Jitegemee? (child crying, being beaten at the back ground}

PM07: I as number seven I don’t feel there is anything that they would not like? Because it’s your money that you save and take it at any time you would want it. It don’t even have an interest, I may take it in the morning and refund it in the late evening, and take it the following day again, I take it daily as I want.

I: And do you think that these things that we have in place can interfere with the rights of sex workers?

PM06: No

I: Number six is saying no. Number two?

PM02: No

I: She is also say no, number four?

PM03: I am still thinking.

I: You are still thinking, can *jitegemee* interfere with the rights of sexual workers? Number one?

PM01: I don’t think there is any way that it would interfere with their rights.

I: Okay and now us, as we are going to begin working as *Jitegemee* when we reach home (Office) so that we may look into how it would work, what are the challenges that we may encounter for us to start our work? (Children crying in the background). What challenges would we encounter, which challenge?

R: Nothing.

I: Number one?

PM01: The challenge is there because it not easy for the sex workers to just come out that “I am a sex worker”, so looking for them and find them the way you have found us is not going to be easy.

I: Mmh

PM01: Secondly, You don’t have funds that you give people, and you know whenever program would come, what goes around someone’s mind is “what are they helping us with” if there is nothing you are going to give them or there is some token or even allowance whenever you have an activity with them, it won’t be easy.

I: Okay, another challenge... that we may have, (children crying in the back ground), when we start *Jikinge,* people started running, starting the journey (beginning), somebody wants to add? Okay, now the last question and we are finishing, in case *Jitegemee* started work, number seven, for you, how much would you save in a week? How much would you like to, you save the money and still be able to eat, you dress and you go to the work?

PM07: One thousand.

I: One thousand, number eight?

PM08: One thousand five hundred.

I: One thousand five hundred? Number nine?

PM09: Two thousand.

I: Two thousand, number four?

PM03: One

I; One hundred?

PM03: One thousand.

I: One thousand< number two?

PM02: One thousand five hundred.

I: One thousand five hundred, when we starts working number three, you personally you will be able to save how much in a week?

PM03: In the *Jitegemee?*

I: Yes.

PM03: Two thousand.

I: Two thousand, number one?

PM01: at least one thousand?

I: One thousand, six?

PM06: one thousand.

I: one thousand, number five?

PM05: One thousand five hundred

I: One thousand five hundred. Okay, is there anyone with anything that you would like to add before we end? And this amount of money after you have collected it, where would you like to keep it? Number seven, where do you want your money to be kept for you?

PM07: That’s what I wanted to ask. After collecting these money, do we choose ourselves from the group here or how would we do it?

I: Is when we are going to come up with, so just tell me that your money, where would like it?

PM07: In the bank.

I: In the bank, Number eight?

PM08: Mpesa.

I: number nine?

PM09: In the bank. Number four?

R: Mshuari

I: Mshuari, number two.

PM02: Bank

I: In the bank, number three? You would like your money to be kept in? Where would you like your money to be kept at?

PM03: My money for the *Jitegemee*, I would like it to be kept at the bank?

I: In the bank, number one?

PM01: Bank

I: Number five?

PM05: M-shuari.

I: Okay any other question that you may add or what you may add? (Silence). Nothing?

R: Mmh (participant cross talked)

I: Okay, thank you very much for taking this opportunity, for taking your time and responding to all the questions. we appreciate you so much.

END OF INTERVIEW
