# Supplementary material for: Eat a little and save a little: A qualitative exploration of acceptability of a potential savings intervention to reduce HIV risk among female sex workers in Western Kenya
Source: PLoS One. 2024 Dec 19;19(12):e0310540. doi: 10.1371/journal.pone.0310540 (PMC11658496; doi:10.1371/journal.pone.0310540)
Supplement: S1 File — (ZIP) [file pone.0310540.s001.zip › Jitegemee Transcripts and Dissemination Notes for Journal/FGD N.docx]

**FGD ID: FGD N**

**AUDIO RECORDING LENGTH: 2 H: 00 M: 07 S**

**FACILITATOR NAME: PHILIP OWITI ODOTE**

**NOTE-TAKER NAME: JUDITH AYALLO**

**INTERVIEW LANGUAGE: LUO, ENGLISH.**

**FGD SITE: BORO, SIAYA COUNTY**

**CATEGORY: BELOW 30 YEARS, RURAL.**

**I: Thank you very much. As I have told you about Jitegemee, what came to your minds when I was talking about Jitegemee, [baby crying] anyone can say their numbers and respond. What came in your mind when I talked about Jitegemee?**

R: [Silence]

**I: Nothing has come to anyone’s mind when I talked of Jitegemee? If you hear the word Jitegemee, what comes in your mind?**

PN07: I am number seven, what comes to my mind is you depend on yourself and not on others. That is what came in to my mind.

**I: You depend on yourself, mmh…, another person, when I talk of Jitegemee what comes in your mind.**

PN01: As number one, what comes in my mind is how we should work hard, so that men don’t use us to get strange diseases from them.

**I: Mmh, Eeh….what are the rest saying?**

PN06: I as number six, I think saving is good, if you earn, I think you should save little and eat little, you divide 50/50 and it can bring help in the future.

**I: The rest of the people, when I talk of Jitegemee, What comes in your mind?**

PN04: I as number four, I think Jitegemee is a kind a self-employed job, you depend on yourself to help yourself out, because no one is there to help you out.

**I: So you help yourself out?**

PN04: Yes.

**I: Okay. Anyone else? Number three, when I say Jitegemee, what comes to your mind?**

PN02: I as number two, what I think of Jitegemee is, I look up to myself and I don’t wait for another person to come and help me. I do things by myself in my own way.

**I: Thank you. Number nine, when you hear Jitegemee what comes to your mind? According to how I explained it to you at first.**

PN05: I as number five, it encourages me to work hard even if I have a family, so that they don’t look up to someone else but look up to me.

**I: Okay I’m requesting you to speak loudly so that we can capture okay? Is there another opinion apart from that one? [Baby cooing] Okay, what do women who exchange sex for money buy with their money? When you have gotten the money, what do you buy? I know everyone buys something right?**

R: (All) Mmmh.

**I: So this one anyone can tell me, okay? You can say what you always buy. We can answer all round okay? You say your number then you answer. [Baby cooing].**

PN08: I can buy body oil, food and little things I don’t have.

**I: The little things are like which ones?**

PN08: Like food to eat or you can see something nice like clothes and buy from the market.

**I: Mmh. Clothes, food, what about the rest, number nine what do you always buy?**

PN09: I can pay house rent and pay school fees for your child.

**I: Mmmh.**

PN07: I can plait my hair.

**I: Plait hair?**

PN07: Mmh.

**I: That is the only thing you do with your money? [Participants laughing in the background] Or there are other things you do with your money?**

PN05: Sometimes if we get that money, when we are doing our sex work job, no one will like to undress an old dress, it will force me to go buy a new dress for myself.

**I: Mmh. Okay.**

PN01: It helps me buy a new phone.

**I: A phone?**

PN01: It also helps pay medical bills for my child and house rent.

**I: Okay.**

PN02: When I get the money I buy food to eat to give me strength so that I can play sex well [participants laughing in the background], secondly, I find a way to feed my children, cloth them and paying house rent.

**I: Mmh.**

PN04: I divide my money in half, one half I buy food and you can also buy scented body oil so that if you go for sex work, you don’t have bad smell on the armpit [participants laughing in the background], this can make you have more clients.

**I: Mmh. Number six has not talked.**

PN06: [Baby crying] The money can help in paying school fees, buying body oil, clothes and underwear so that I don’t go to sex work with torn underwear.

**I: Thank you. Now we have mentioned the things that we buy and these things have prices, someone said paying house rent. Do all of you pay house rent? I hear some don’t pay house rent, so tell me how much you pay for house rent and buy food.**

PN05: Currently if you go to the market, you find that food are quite expensive, sometimes I may want to go to work and I must see that the children eat well, I must also eat well. So I’ll be forced that this money… when I go there, because the budget will be high, also when I go to that partner and tell him that even if he does not give me, he should know how life is [understand how expensive life is].

**I: Mmmh.**

PN05: Yes. Also sometimes the house rent has been raised, it will force me to pay.

**I: In a week, how much do you spend on food?**

PN05: I can buy food that cost almost five hundred shillings. It also depends with the family that you have and how you also want to eat.

**I: Okay.**

PN05: Yes.

**I: I believe all of you buy food, right?**

R: All (yes).

**I: I don’t see your number…..number four, how much do you use for food on a daily basis?**

PN04: I use six hundred shillings.

**I: Mmh and what about the others? Answer quickly so that we finish early.**

P: Starting morning till supper. In a day I use seven hundred shillings.

**I: Mmh, number two.**

PN02: From breakfast, lunch till supper, I use eight hundred shillings.

**I: Eight hundred shillings?**

PN02: Yes.

**I: Number one.**

PN01: [Baby talking] from breakfast till lunch I use four hundred shillings because I live alone.

**I: Four hundred shillings?**

PN01: Yes.

**I: What about number three or number eight [participants laughing in the background], number three on a daily basis how much money do you spend on food?**

PN03: I use four hundred shillings.

**I: Four hundred shillings?**

PN03: Yes.

**I: Number eight?**

PN08: Currently I live alone, I have no kids and I use three hundred shillings.

**I: Are there things that you need to buy weekly? Is there anyone?**

PN05: [Baby crying in the background] I do buy airtime to call my clients.

**I: You buy weekly?**

PN05: No. daily.

**I: Is there anything else you buy on a weekly basis?**

PN05: Maybe maize flour and sugar.

**I: How much does it cost you to buy sugar on a weekly basis?**

PN05: I do buy 2kg of sugar on a weekly basis.

**I: What about maize flour?**

PN05: I grind two tins of 2kgs each.

**I: Two tins?**

PN05: Yes.

**I: Number three, what do you buy on a weekly basis if there is.**

PN03: [Laughing] I usually shop on a daily basis.

**I: Is there someone who has to shop on a weekly basis? What about monthly? Is there someone who usually shop monthly?**

PN06: We pay house rent.

**I: Number six must pay house rent, mmh.**

PN05: I pay school fees.

PN01: I do shopping for a whole month, pay house rent and also support my kid at home.

PN07: I must pay electricity bills, my kid’s lunch at the school.

**I: Number nine, is there anything you have to pay or buy on a monthly basis? Is there?**

PN09: I pay for chama (merry-go-round).

**I: What about the rest, you don’t pay for chama (merry-go-round) for monthly basis?**

PN05: I do pay. There is also the one that you pay on a daily basis.

**I: Is there one that you pay on a monthly basis?**

PN05: Yes.

**I: Okay, and are there things that you pay once or twice in a year? Things that you spend your money on a yearly basis?**

RPN07: Nothing. Just buying my child new clothes for Christmas.

**I: Only.**

R: Yes.

PN08: Yearly I buy my kids school uniform.

**I: A new uniform.**

PN08: Yes.

**I: What about the rest of you, is there something you do with your money yearly?**

PN01: I must take my daughter for photo sessions.

**I: What about the rest of the group? At least in a year you must have a new photo right?**

PN01: Yes.

**I: Okay….umm….the money that you use, where does it come from?**

R: Majority get it from their salaries, others from businesses and others from sex work.

**I: You are saying it on a low tone.**

R: (Laughing).

**I: She has said from salaries….you work somewhere?**

R: Yes.

**I: What about the rest of the group, the money that you use, where does it come from?**

PN06: Most of the money comes from sex work.

**I: Okay. What about the rest? There are no right or wrong answers. Don’t say that you colleague said a wrong answer, all of you have you own opinions. Number three, the money that you use, where does it come from?**

PN03: From businesses.

**I: Businesses?**

PN03: Yes.

**I: Okay. What of the rest?**

PN07: I don’t have a job so the money I use comes from sex work.

PN04: Sex work is my job so that’s where the money comes from.

**I: Some of you have not answered, number five, the money that you use where does it come from?**

PN05: The money that I use comes from businesses and sex work, also currently you cannot have one business because there is no money. So you add here and there and when I receive the money, I divide it into two for various usages.

**I: You have talked of other businesses and if they come close, if who comes close?**

PN05: [Laughs] we do other things apart from sex work. We also do sex work because the money we get is more than what we earn from business and you get it faster.

**I: What kind of business do you do?**

PN05: I am a photographer.

**I: You take photos? Wow that’s good. What about the others?**

PN02: I work in a bar and when it reaches end of the month, they pay me also when it’s around eleven o’clock I’ll have a partner to have sex with and he gives me money then the following day, I’m good.

**I: You’re good?**

PN02: Yes.

**I: Okay, those are ways to get money, number nine has not talked. The money that you use, where do you get it from?**

PN09: I get it from a business.

**I: What kind of business do you do?**

PN09: I work in a hotel.

**I: That is the only place you get money, or are there other places you get money?**

PN09: Those others also

**I: [Participants laughing in the background] which ones are those? Tell us so that we know, when you say those others what do you mean? Stop looking at number seven, she already spoke.**

PN07: Just answer.

**I: Just answer, do not fear. Do you agree for her to help you? When she says those others what does it mean?**

PN06: I won’t fear saying that sex work is bad. There is no sweet money than than the one given by a man, right? Is that true?

R: (All) Yes.

PN06: I also agree with that.

**I: Someone said that the one that comes from a man is better, so how much money are you always given in most times?**

PN07: It’s only better when it comes from one man or maybe you’re his wife because he also has his own needs. So yours and his are sweet and it’s only better if you have one man.

**I: Mmh**

PN07: Besides you cannot have one man and he does not support you in anything.

**I: Mmh.**

PN07: Mh. If you have something to do and depend on then you can have one man

**I: What if you don’t have?**

PN07: If you don’t have then you go and look for one, they are so many outside here [participants laughing in the background].

**I: Those that you look for, how much money do they give you if you meet with them?**

PN07: They don’t pay well.

**I: How much?**

PN06: Even if he has given little money, the money is still sweet that even if you have a business, even if he gives you some small amount, it can help you sort out some issues.

**I: Mmh. Okay, what about number eight?**

PN02: I get paid monthly and in sex work I can get the money on a daily basis. If I get a partner today who gives me five thousand shillings or two thousand shillings that is for a day as I wait for the monthly basis.

**I: She has answered the question very well, that she gets five thousand shillings or two thousand shillings and what do the others get?**

P: [All participants] laughing.

**I: I know it’s different, not everyone gets the two thousand shillings, it happens and we know it, most of the times how much do you receive averagely?**

PN04: [Baby crying] I always get five thousand shillings because someone cannot have sex with you, because you will need money to buy soap to shower, body oil to use after bathing, even the clothes to wear so it will force him to give me a good amount of money.

**I: Mmh….anyone with a different opinion? Or another thing she wants to talk about? Number one?**

PN01: Nothing.

**I: Okay. What do women who have transactional sex usually buy? What are the reasons you buy these things? You have said you buy things, what are the reasons you buy those things? People buy different things, I have not heard people saying they buy similar things, why do you use money for those things?**

PN06: Things like clothes, things for hair when you want to go to work you have to dress up nicely because someone only notices you when you’re clean, then you get clients [male partners] but when you go when you’re untidy the person will be unhappy with you, so you have to meet that person when you’re clean. So, you have to buy body oil, dressed nicely and plait your hair nicely, spend money to get money.

**I: Spend money to get money?**

PN06: Yes.

**I: What about others, what are you thinking?**

PN02: The reason as to why we should do shopping, I’ll have to buy new clothes to get more clients. So if I’m smart, people will notice me faster and I will get a client to get money. So I have to buy new clothes, be clean, make my hair and be smart.

**I: Mmh.**

PN02: Yes.

PN04: Men like those things that are attractive, if you go to them when you are decent and attractive and you are soft all the time that will make you get clients faster.

**I: That is your clients?**

PN04: Yes.

**I: You said a lot of things that you buy, right?**

R: Mmh.

**I: Some talked of paying house rent and others school fees, why………….**

PN01: We buy things to satisfy our needs.

**I: Mmmh.**

PN01: Yes.

**I: Satisfy your needs…..okay….number three, the question is, why do women who engage in sex work buy the things they buy?**

PN03: [Silent].

**I: Number eight?**

PN08: The reason we buy these things is to be decent, be clean, you buy food and eat to be healthy.

**I: Thank you. She buys food to eat and be healthy. Thank you, anyone else?**

PN02: We have to buy these things because we depend on ourselves and no one will buy them for you, so you have to buy them by yourself.

PN07: I pay school fees. If you have a girl child or a boy so that he/she to have a good future ahead, for him/her not live a life like yours or maybe you didn’t finish your schooling, so you try for him/her to finish schooling and have a good future.

**I: Okay…umm, number six, she was talking about buying clothes, right? For you if you buy clothes, how much do you use?**

PN06: It depends with the type of clothes that you have gone to buy, the clothe that you like, you can buy a cloth worth three thousand shillings or two thousand five hundred shillings but mostly boutique clothes starts at one thousand shillings.

**I: Number seven has talked about school fees, how much do you pay for the fees?**

PN07: Fee?

**I: Mmh.**

PN07: Per….term…per month, I pay more than three thousand shillings.

**I: Mmh.**

PN07: Mh.

**I: Is there any other person that pays school fees?**

R: All of us.

**I: How much do you pay, tell us your number before you speak.**

PN05: I prefer my child to go to a private school, not that when he/she comes from school and he/she doesn’t find me at home and starts to cry, when I come back I go and pick her up. So the fee is high and pay for school fees at four thousand five hundred shillings.

**I: That is a month or?**

PN05: Yes. A month.

**I: What about number six, how much do you pay for school fees? You said all of us will answer.**

PN06: The school fee that I pay?

**I: Yes.**

PN06: It depends because I have different kids in different classes so it depends how much I’ve been asked to pay.

**I: How much do you usually pay?**

PN06: The one that I pay in a month?

**I: Mmh.**

PN06: I pay six hundred shillings for one person.

**I: The rest who have kids in school how much do you pay?**

PN01: My child goes to playground school and I pay nine hundred shillings in a month.

**I: What about number three?**

PN03: (Chuckles).

**I: Someone talked about house rent, number three is still thinking…how do you pay for house rent?**

PN01: If I live in a single-room house that has electricity so I pay house rent for one thousand five hundred shillings.

PN02: I live in double-room house and I pay house rent for three thousand shillings in a month.

**I: Number two pays house rent for three thousand shillings.**

PN06: I also pay three thousand shillings.

**I: Number six pays house rent for three thousand shillings.**

PN05: I pay house rent for one thousand five hundred shillings.

**I: Eeh.**

PN07: I pay house rent for four thousand shillings because it has three rooms and I pay electricity bill (token) separately.

**I: How much do you pay for electricity bill (token)?**

PN07: In a week I buy for one hundred and fifty shillings.

**I: Eh! Number three, do you pay house rent?**

PN03: Mmh.

**I: How much?**

PN03: One thousand shillings.

**I: Mmh, what about number eight?**

PN08: One thousand shillings.

**I: Number nine, do you pay house rent?**

PN02: I pay two thousand five hundred shillings.

**I: Two thousand five hundred shillings, that is number two. Okay now that you exchange sex for money, do you save?**

PN07: No. I personally, I don’t save.

**I: Number seven does not save. Do the rest of you save?**

PN01: One must have a savings account, anything can happen to you or you can have an emergency or your daughter can be sick and you don’t have money, so one must save. You can save five hundred shillings or even two hundred shillings and you deposit it at the bank.

**I: Okay.**

PN06: I save little by little according to what I have got, I save daily and when its end month I get it.

**I: Mmh.**

PN06: I save it in a group…..like that.

**I: Chama (merry-go-round)?**

PN06: Yes.

**I: Do you think the rest save? Number five?**

PN05: I tried to save and when my children were going back to school, I used the money to take them back to school and I started saving again recently.

**I: So it means that people are saving and you’re saving for schooling?**

PN05: Yes.

**I: Eeh. Number three, did you raise your hand? Just continue, do they save or not?**

PN03: I save on a daily basis and get the money at the end of the month.

**I: Why are you saving? What are the reasons as to why you are saving?**

PN03: I save so that when then month ends I add the money with the business money, because I always use that business money.

**I: Ooh. Number eight, do you save?**

PN08: I also save on a daily basis and when it reaches end month, I send the money to my kids at home.

**I: Mmmh.**

PN08: Mmh.

**I: Number five do you really save? Ah! You already said that you save.**

PN05: Mmmh. Though I want to save again this time.

**I: Ooh.**

PN05: Yes.

**I: You already talked about school fees and that is the reason people save right?**

PN05: Yes.

**I: What about number nine?**

PN09: I am saving because you can have an emergency and lack a way to help yourself.

**I: Mmmh.**

PN09: Mh.

**I: So there are those who save daily right?**

P: (All) Yes.

**I: Are there people who save every week? How much do you save weekly?**

P: I save the moment I get the money.

**I: Mmh. Roughly in a week, how much do you save? How much do you always wish to save in a week?**

P: You know earnings are different, sometimes you can go without earning anything in a day.

**I: Mmh.**

P: Yes.

**I: Those who save, on a weekly basis how much do you save?**

P: Sometimes in a week you can save up to two hundred shillings.

**I: Two hundred in a week? Okay ‘haba na haba hujaza kibaba’ [little by little fills the pot]. Number five, how much money do you save in a week?**

PN05: (Laughs) I have a small saving box that I put twenty shillings on a daily basis.

**I: Mmh. Twenty shillings every day?**

PN05: Yes.

**I: Number one.**

PN01: I save one hundred shillings.

**I: On a weekly basis?**

PN01: Yes.

**I: Someone talked of chama (merry-go-round)?**

R: (All) Yes.

**I: Who was it?**

R: (All) Number six.

**I: How much money do you save in a week?**

PN06: I save two hundred shillings every day so that in a month I get six thousand shillings.

**I: Okay. Those who save money, do they have a character that makes them to save?**

PN06: It’s not that I have the ability to save but I have to deny myself to save sometimes you squeeze so much to save money and achieve my target.

**I: One of the things you have mentioned is denying yourself. Those who save what’s the ability to make you save?**

PN09: Sometimes you want to buy something, it will force you to deny yourself to buy what you want.

**I: Mmh that is number nine.**

PN02: I also save and I work in a bar. Sometimes I serve a customer and maybe he has a lot of money in his pocket and when I give him the balance he tells me to keep change.

**I: Mmh.**

PN02: Maybe he gives me one thousand shillings and I decide to use five hundred shillings and keep the remaining five hundred, so it depends.

**I: What are others saying? Number four.**

PN04: Sometimes you have a problem and a problem does not notify you, anything can happen and you can use those savings.

**I: So you save as you think of the future. So, that means that those who save are those who think of the future?**

P: (All) Yes.

**I: What are others saying? Those who save, what kind of characters do they have that gives them the ability to save?**

R: Silence.

**I: Why can’t these other people save? What prevents them from saving? What kind of characters do they have that prevents them from saving? I will start with number seven because she is the one who said that people don’t save [participants laughing in the background]. Are there characters whereby someone who doesn’t save has?**

PN07: Yes.

**I: Like which one.**

PN01: One that has impulse buying [participants laughing in the background], the person wants to buy everything she sees.

**I: Say your number first. That is number one who has talked. Number two, I heard you said something when number one was talking, kindly repeat what you were saying so that we hear. Those who are not saving, what are the reasons as to why they cannot save?**

PN06: I think that, that person who does not save, can think that saving is difficult or the amount of money she has is little but I would like to encourage her that the money she sees as little, she can save twenty shillings only, so if she saves this twenty shillings for a whole month, she will get six thousand shillings. So you can just try even if you think it’s hard.

**I: Meaning they are people who are find their money to be little?**

R: No.

PN06: I mean someone can feel that I have the money but planned for something so it will force you to cancel, like let me cancel this in order to do that so that when it reaches a certain time I shall do it better, so I can encourage her.

**I: The rest of the group, what do you think about those who do not save, do they have attitude? Do they have characters that makes them not to save? I heard number nine talking a while ago. Is there a way that they are, those who do not save? Number nine has not given an answer. Okay, number eight? Is there a way that they are?**

PN08: Mmh. Sometimes they get little and use.

**I: They only receive hand to mouth?**

PN08: Mmh.

**I: Okay, for those women who exchange sex for money, the ones who save? Why do they save? Those who said that they save, what are the reasons you save?**

PN06: I save because the money I get every day, it’s little that I cannot use it to do something just once. So I save it till the end of the month and give it to the landlord for house rent then I remain with little to buy the things I’ve always wanted to buy and forget about it. So I think saving helps me.

**I: Mmmh [someone clears throat] lets finish this part then continue. Those who save, why do they save?**

PN08: I save to use the money for something meaningful the money that I get every day, if I use it every day, it doesn’t help, so you must save a little.

**I: Mmmh.**

PN08: Mh.

PN04: You save because one day you might be tired with the sex work job and then decide to start your own business, so that money can help you even if you want to start a carwash business, you can start a carwash business with the money then you leave the sex work job.

**I: Mmh. So many of you talked of saving, I’m not hearing responses. Number three, why do you save?**

PN03: (Silence).

**I: Okay, why do they find saving easy? Number five, what are the reasons saving become easy?**

PN05: It can be easy for me depending on my income, how I earn money, when the money is not there I don’t save.

**I: Mmh.**

PN05: Mh.

**I: Okay. What are the difficulties they face when saving?**

PN04: The difficulties you can face is that sometimes you save the money up to twenty thousand shillings. Then suddenly your child becomes sick and when you take him/her to the hospital, the bill is more than the twenty thousand shillings. Now you will see the difficulty in saving and the money that you saved, you will use it to take the baby to the hospital.

**I: So you save a little and when something comes up you use it?**

PN04: Mmmh.

**I: Any other difficulties?**

R: The difficulties in saving is when we give our money to someone to keep. Sometimes it reaches December and the person disappears with your [participants laughing in the background] then travels home and when they come back and you ask them for your money back, they tell you that you’re boring them that they already used the money that if you like you can sue her. So if you sue her you’ll be asked how you agreed with the person you gave the money. Then you’ll be told to go and sort the issue among yourselves. Then you will never get your money back.

**I: The person already used the money.**

R: Yes.

**I: What other difficulties do you face in saving? Number seven, what are the difficulties in saving? You have been quiet for a long time [laughs].**

PN07: [Silence].

**I: Okay, number eight? What kind of difficulties that someone who saves can have?**

PN08: Only the days when you don’t earn money. You can fail to earn money even for a whole week and you don’t have money to save.

**I: Mmh. The days when there is no money, how can that be helped? I mean when you don’t earn money today, tomorrow, how do you help yourself? Number five, I can see you have something to say.**

PN05: The difficulty I face is that when I don’t save I have borrow money hoping that the next day I may get a client [male partner] and the client [male partner] does not come by. So, I’ll have difficulties then ill borrow from a shop and maybe I promised the shopkeeper that ill refund the money the following evening hoping that I’ll meet another client (male partner) then I don’t. So I end up being an enemy to the shopkeeper. I become a liar, that’s how I face difficulties, then I feel disappointed in myself and I feel it’s not good for my health and it stresses me.

**I: It stresses you?**

PN05: Yes.

**I: Okay and what difficulties do others find in saving? Do all of us find it easy to saving? Number……**

PN02: I find challenges in saving, sometimes I’ve save up to ten thousand shillings and it happens that I get a phone call that my mother is seriously sick and money is needed, so it will force me to withdraw the money I have saved to help her that is one of the challenges I get.

**I: For those who do not save, why do you think they don’t save? I’m just seeing everyone is quiet.**

R: (All) laughing.

**I: You are already feeling tired? Don’t feel tired.**

PN01: Saving depends on your income. Mh… Sometimes you don’t have money, so saving becomes hard because house hold needs also awaits you.

**I: Mh. Number one has said that savings depend on your income and sometimes you don’t have money, what are others saying? These people who do not save, what are the reasons as to why they don’t save?**

PN09: Sometimes you get little and use it all.

**I: That is number nine. So the income is less.**

PN09: Yes.

**I: Um, what are the rest saying? Number four.**

PN04: Some people do not save because they feel they can use the money because they will get more the next day from sex work.

**I: Mmmh.**

PN04: Mh.

**I: She has responded well, to use for today and tomorrow…….**

PN04: Earn more.

**I: Yes. What are the rest saying? Why don’t these people save? Do you think there are disadvantages that make people not to save? Mmh…? (Someone whispering in the background).**

PN02: There are disadvantages. The disadvantages of not saving is that you can have an emergency and have no help, so I see not saving as a bad thing, it’s good if people save.

**I: Mmh. Number eight? Are there disadvantages of someone not saving?**

PN08: Yes, there are disadvantages.

**I: Like which ones.**

PN08: Like us those who have given birth, you should save like ten shillings somewhere because you can receive a difficult information and it helps you.

PN04: Not saving is bad because you can say, “Let me just spend this one, I’ll save tomorrow,” and you just move on like that and maybe you have small children who have not started school then you decide that they are still young and you use the money. Then as the days will be passing by, you will lack money to take your children to school that is if you did not save. But if you saved you can withdraw the money you saved and manage to take them to school.

**I: Mh, mmh, number nine. Are there disadvantages for someone not saving? We have already got some disadvantages, are there some other disadvantages, number six, is there any?**

PN06: By not saving can give me problems because I always use it to pay house rent if I am not to depend on someone else. So if I don’t save the landlord can come to my house and that is my worst fear, so I feel I should save.

**I: On a daily basis people say that what has a bad side must have a good one. What are the advantages of someone who does not save? Number seven, what are the advantages of someone who does not save? Are there advantages for that person who does not save? Are there any advantages? Number four says no, why?**

PN04: [vehicle honking] It’s not good because sometimes if you saved and you go for sex work and you don’t get a client [male partner] or maybe oil is finished and your hair is unkempt, so there is no way you can help yourself but if you did save, the money could help you plait your hair or buy oil.

**I: So the fact that someone is not saving it does not have any benefits?**

R: (murmuring).

**I: Say your number then answer in a high tone so that we capture.**

PN04: It does not have benefits.

**I: What are the rest saying, does it has benefits or not?**

PN02: I don’t see its benefits.

**I: You don’t see its benefits?**

PN02: Yes.

**I: Number three.**

PN03: If you are someone who saves in group meetings, you can lack something and decide to go and loan at the group meetings which is a good thing but if you are not a member you cannot be given.

**I: So that person who does not save, what benefits does it have? That person who doesn’t save money is there any benefits she sees in that?**

PN09: None.

**I: That is number?**

PN09: Nine.

**I: Number nine says none. Eh! Okay. Where do women who exchange sex for money save? We started listing some when we were talking earlier, right? Someone talked of chama (merry-go-round), where else do you save from?**

PN09: On phone.

PN07: On home bank.

**I: Home bank?**

PN07: Mh.

**I: How does it look like?**

PN07: It’s a man-made steel box which you cannot open easily but a wooden one you can break whenever you have problems, so the steel one you make a small space for putting money. And it is welded well.

**I: Okay. I heard number nine saying t something but she did not say her name, what were you saying?**

PN09: You can save on M-pesa.

**I: M-pesa. Mmh. In the house, M-pesa, how are the rest saving?**

PN04: One can save in the bank. You can open a savings account at the bank and they will give you… you will tell them that you want to save up to fifty thousand shillings or twenty thousand and you will not withdraw it but the one in the house you will have the urge to open it and take it back to the person who welded it to open it for you. Then maybe a problem arises but the one at the bank you won’t go and tell them that you have an emergency for them to withdraw for you the money, it will have to reach the amount you targeted.

**I: Mmh.**

PN04: Yes.

**I: Umm number five.**

PN05: I save on the phone. Mattress account for emergency that is a home bank.

**I: Mmh, where do you save in your phone? There are so many places to save money in the phone, which is a good place where you save in your phone?**

PN05: I save in KCB bank.

**I: KCB M-pesa?**

PN05: Mh, KCB, M-pesa, Mshwari.

**I: You save you money on all these?**

PN05: Yes. I distribute little by little.

**I: Number six, where do you save?**

PN06: I can save through chama (merry-go-round) or bank. You can open account and save little that you have. So at the bank there is one that you can withdraw daily and there is another that you can only withdraw after some period like Hekima Savings that you can withdraw after three months.

**I: Why are you saving in chama [merry-go-round]?**

PN06: Chama……in the bank just like if I have decided to open an account that I can withdraw after three months, I cannot wait for three months then I go and pay for house rent. I save in chama [merry-go-round] because it helps me easily [it is easy to access].

**I: Mmh.**

PN06: If I save in chama [merry-go-round] and when end month comes I take it and pay for house rent then I’m done with the landlord…….mostly, then I think of other things.

**I: Number two.**

PN02: I save in chama [merry-go-round] and I have my own bank account.

**I: Why do you save in chama [merry-go-round]?**

PN02: I write down daily and when end month comes, you are given the money then I see how to pay house rent so that I I’m not stressed up.

**I: Okay. Number seven said that she saves in a box, why do you save in a box? Why is it good for you?**

PN07: I save in the box because I have less money that I cannot save on phone. Secondly I stay far from a centre (town) to go and deposit in my M-pesa, so the box gives me easy time even if I get ten or twenty shillings I put in or if I get fifty shillings.

**I: Number nine you said that you save on M-pesa?**

PN09: Yes.

**I: Why? Why do you prefer M-pesa?**

PN09: M-pesa is the easiest place for me to save in.

**I: Why is it easy for you and not the others?**

PN09: I’m used to it.

**I: You are just used to it?**

PN09: Yes.

**I: Number eight, do you save? Which one do you like and why do you like it?**

PN08: I save in KCB bank, the little I get even if it is fifty shillings I save there.

**I: Why do you like KCB bank?**

PN08: It a good place that saves my money.

**I: Good in what way?**

PN08: I don’t withdraw faster.

**I: You don’t withdraw faster?**

PN08: Yes.

**I: Okay. Thank you all very much. So….the women who exchange sex for money, do they live in a lifestyle that is more than the money they get? I mean the money you get is less than what you want?**

PN08: That is true.

**I: Mmh.**

PN08: The money is less than my budget.

**I: Mmmh.**

PN08: Mh.

**I: What are the rest saying?**

PN04: There is a saying that says ‘cut your coat according to your size’. You cannot want the best lifestyle and your income is low, so you must ratio them to be on the same level.

**I: So you always feel that the money you get, is the one you use, is enough for what you want?**

PN04: It’s not enough but you reduce your lifestyle a little bit so that that money should to be enough for what you want.

**I: What do the rest think? The women who exchange sex for money, do they live in a lifestyle that is more than the money they get? More than their income? Number three, give us your thought. Do you think……I’m not saying you, if you respond, you respond for many people okay? The answers you give does not mean it’s from you, you can respond for others too, right? The women who exchange sex for money, do they live in a lifestyle that is more than the money they get? Mmh, okay another person, number three, what do you think about that? Can you ask this question?**

P: [All Silent]

**I: Among the women who engage in sex workers who live in a lifestyle that is more than their income, what do they do to fill the remaining gap? The person is who is the most tired among us will be the one who answers the question, right? (Participants laughing in the background) Mmmh number one? What can they do to fill up the remaining part?**

PN04: You can buy things but you must be able to reduce the buying.

**I: Mmh.**

PN04: Yes. Sometimes the body oil that you use cost six hundred shillings, so you decide to buy body oil that costs three hundred shillings so that you balance and if you always plait your hair with three thousand shillings it will force you to spend two thousand or one thousand to plait your hair.

**I: That’s number four’s opinion, number one? What can they do to fill the gap?**

PN08: It will force us to… it will force you to reduce so that your money is enough for everything you want.

**I: Mmh.**

PN07: If your budget is higher than you income, it will force you to look for someone to help you, you can look for sponsor, nowadays we say that they are the ones who have money more than young men, so you can look for an old man who is the same age as your father, his pocket has more money than a young man.

**I: So the sponsor fills up for you?**

PN07: Yes, the sponsor fills the gap better than a young man.

**I: That is number seven’s thought, I’m coming back to you number one, what can you do to fill the remaining gap?**

PN01: You can reduce your budget to a lower price, if you are buying 1kg of sugar @ one hundred and twenty shillings, you can buy a half kg so that the remaining money you fill the gap.

**I: Mmmh, number three, what can you do to fill the remaining gap?**

PN03: [silence].

**I: If there is a gap what can you do to fill the remaining gap? Number three I’m on you. Mmh?**

PN03: [silence].

PN04: If you don’t reduce your budget, it will force you to look for a sugar daddy to help you reduce… you fill the gap like number seven has said.

**I: Okay, you don’t have sugar daddy, you’ve have reduced the budget but it’s still less [participants laughing in the background], what will you do?**

PN07: You find one sugar daddy, you reduce and it’s still less.

**I: Mmh.**

PN07: Everyone wants a good life, you also want to live in a house with electricity, cook using gas cooker like other people, not that you are cooking with firewood, you plait your hair and become beautiful.

**I: Mmh.**

PN07: It will force you to look for……so it doesn’t have to be one but two or three sugar daddy’s because they are also looking for young girl’s not older women so you will look for one or two or more than two.

**I: Okay. So are there places…umm…. Are there things…..women who have sex, do they borrow a lot of money, repeatedly?**

PN07: I agree that one can borrow money repeatedly.

**I: Mmh.**

PN07: Maybe you have a problem then you go and borrow somewhere and maybe it’s not sorted, sometimes life is hard or maybe your sugar daddy does not have money, you also don’t have and the current situation in life, so it will force you to go and lie to someone then borrow from them money one day when you get money, you return back. You can even borrow money three times repeatedly.

**I: Mmh, repeatedly, so if you borrow money, who do you borrow it from? What kind of relationship do you have?**

PN07? You can borrow from young men, you know they don’t give a lot. So, you can borrow from these young men but later you return by having sex with them. He is someone who has always desired you for a long time even if he is an old man, he always desire you even if you don’t want, so it comes to a point where you are stuck and you will go to him to borrow money then he will lend you the money knowing that you will remove your pant for him.

**I: So you will have sex with him?**

PN07: Yes.

**I: Okay number two, where can you borrow from?**

PN02: In most occasions I always borrow from saving groups, I can borrow from the group repeatedly, because I borrow and when I get a client, then when there is a group meeting again I pay the money, then I borrow again because I never miss a client (male partner).

**I: Mmh.**

PN02: Yes.

**I: If you borrow, what do you use it for?**

PN02: I can borrow it if I have some commitment with money, so the money I get on that day is not enough for what I want to do, so it will force me to add with the borrowed money from the group and use it for the thing that I wanted to buy.

**I: Mmh. Okay, number six if you borrow, where do you borrow it from?**

PN06: I can use loan……I can borrow from KCB bank when my expectations are not enough and I’m less with some money, just like I’m a business woman, I can borrow from KCB then I do my business and return slowly.

**I: So, on most occasions when you borrow money it is for business or there are other things you use the money for?**

PN06: Things like those meant for the shop you can go and take… things like sugar, or soap (laughs), you go and take, they are things that are not difficult to repay. So when doing business you go and take them then after getting the business money you deduct little then you’ll add later. So, I can take loans from KCB bank to promote my business.

**I: Number seven has told us that you pay by having sex, right? Are there other things one can do to pay the debts that they have? Apart from paying debts through sex, what else can you do to pay the debt?**

PN05: A woman who has children or has a child or should work hard at least she should rear even some chicken so that when things are hard and you have nowhere to rely on, she can sell it in the market or put it as food on the table.

**I: Mmh number four, what can you do to pay the debts apart from the one that have been talked about? What can you do to pay the debts that you have?**

PN04: The debts that I have? [Phone vibrating]

**I: Yes.**

PN04: Sometime the sex work is done at night?

**I: Yes.**

PN04: So you may find a small job in the village like weeding or trimming then you are paid, then you go every day and get paid, and then if it reaches the amount you took as debt then you repay.

**I: Number eight?**

PN08: If I have a debt, I can borrow from a group (baby coughing) then if I get I repay

**I: Okay. Are there things that women who have sex usually do to add in their income? Are we all tired or the questions are not clear.**

R: (All) repeat it.

**I: If it’s not clear you can say, right?**

R: (All) Yes.

**I: So there are things that women who have sex usually do to add to their income, when they are still at sex work, what can they do to increase their income?**

PN07: If you stay at home and you have not rented a house in the city, and you have a small farm, you can plant vegetables then go and sell and get money. Or you have a chicken then they reproduce and become many then you can sell and get money.

**I: Number three? What can they do to add their income? Do not go far, just sit closer, women who have sex, what do they usually do to add to their income?**

PN04: I can look for those who I can wash clothes for, I go and wash people’s clothes in the centres sometimes someone is tired and they only wish to find someone to wash for them, maybe you have washed for them the duvet and they give you five hundred shillings. Go to another person wash a carpet and they pay you one thousand shillings and when the day is over, you might have even three thousand shillings then at night you go and look for money from sex work, so it will help you.

**I: Mmh, number 3 I’m back to you, the point that you started talking about?**

PN03: Sometimes you have a business, so you can look for work that you can combine it with.

**I: What kind of work?**

PN03: The kind of work that you can find time and combine it with.

**I: So if you do another work, what can it look like?**

PN03: [Silence]

**I: Mmh, anyone can help, number one?**

PN06: I can add my income like this, I’m a farmer, I farm…..I go to the farm… I live in the centre [town] but I have leased a farm, so you plough and weed and when people will be buying cereals like this… or when it will come a time that people will be harvesting then you will also be eating yours from the farm.

**I: Mmh.**

PN06: Yes. So I do it that way and I add it with business because I’m a business woman……like selling fish, so I go to the farm, then I come back and fry my fish and go to the market. Yes, so when I’m done, I just rest, then I wait for my time….you know it (laughs) my time is…….all of these does not prevent me from doing that thing [doing sex work], you plan your work (kazi kwa mpango), everything has its own time, so you must be busy.

**I: Okay, so when women borrow money, how much can they borrow? Number four.**

PN04: Mmh?

**I: How much can they borrow, the women who have sex [sex workers], averagely how much can it be in a month or?**

PN04: You can borrow depending on the problem that you have (baby crying), sometimes if I borrow so much it can be five thousand shillings which I use it for a specific job then refund

**I: Mmh, five thousand shillings, number five, how much can someone borrow?**

PN05: Sometimes I see that I can get a client (male partner) who can give me some amount of money, so in most cases I spend money knowing I’ll get money later.

**I: If you borrow from loan, how much can it be?**

PN05: If I borrow money?

**I: Mmh even in a month?**

PN05: In a month I can borrow five thousand shillings knowing the future is good.

**I: Mmh five thousand, number six how much can it be?**

PN06: I have never borrowed a lot of money but if a lot it can be three thousand shillings.

**I: If it’s a lot can be three thousand shillings, eeh number two?**

PN02: I can borrow five thousand shillings.

**I: Five thousand shillings, number one?**

PN01: I can borrow two thousand shillings.

**I: Mmh number three?**

PN03: I can borrow one thousand five hundred which I add in my business, in a day I can save little and pay slowly.

**I: Mmh number eight?**

PN08: I can borrow one thousand shillings.

**I: Number nine?**

PN09: The little that I borrow is three thousand shillings.

**I: Three thousand shillings, number seven?**

PN07: If I borrow, the highest can be two thousand shillings.

**I: Mmh, okay. Thank you very much, women who have sex do they even think when they can leave that job? Is it something people think about?**

PN05: It is difficult to leave something that you are used to already.

**I: [Laughing] so people never think of stopping?**

PN05: They don’t think

**I: That is number five’s thought.**

PN01: Many people think of leaving the job because it’s a kind of job that tarnishes names. So people can find other jobs and leave that one.

**I: So those who think, do they talk together or is it something that one thinks by themselves?**

PN01: You can think by yourself. It depends how it helps you, you cannot tell someone to stop and it helps them in another way. You can decide to stop it yourself because you can annoy the other person.

**I: Okay, number seven?**

PN07: According to my thinking, people always want to stop that job because it’s not a good job, it is even a bad job in your family because when your children grow up….people are scared to come together as women or young girls to unite and look for something that can help them in life so that they can leave that job. They are also scared that if they stop that job, what will they eat, they have a lot of things they need, where to get food and how to help their children.

**I: Mmh, another person?**

PN04: I feel that people wish to stop… especially myself I would want to stop that job, because maybe you joined and it’s was not your wish, your had problems that’s why you joined that job. So the pain in your heart wants you to stop that job and one day God will open the door for you and you will stop that job.

**I: Mmh, number two?**

PN02: You can want to stop that job, you cannot do it till death, it might reach a point when I’m old that I cannot open the server [have sex] (participants laughing in the background) so it will force me to stop or you might want to leave but you have to save so that when it reaches a point that I’m tired and want to stop it, I will have another thing to do

**I: So that is when you are old and your body is tired?**

PN02: Not old…..I can want to stop it even now depending on how swollen my account is but you can find that I eat too much, so it will reach a point where I work and just eat and I don’t save anything and it comes to a point where I am tired and cannot have sex and I’ll stop because I have aged

**I: What other things may make one want to stop?**

PN01: Things that may make one to stop?

**I: Yes number one.**

PN01: Insults and apart from insults, it gives you a bad name.

**I: Mmh, another thing that may make one to stop having transactional sex.**

PN04: You might want to stop because you might get a client, you just get people who are interested in your body but has no money. You have sex with him but he does not pay you then you find another one have sex with him and he does not pay you. So you get tired and say that these people have sex with you and they don’t pay you then you get discouraged and decide to stop that work.

**I: Number eight, why would someone want to stop?**

PN08: You may want to stop if you feel that you have done it for a long time, you may want to stop……I specifically would want to stop and find something else to do.

**I: Mmh, okay and are they things that people discus?**

PN07: I say that people don’t discuss it because they fear. It can be that I know you, but I don’t know your inner thoughts so I can tell you, you can go elsewhere and say your own things.

**I: Mmh, okay, is there another person who has a different thought from number seven. All of you don’t talk to your friends? Everyone thinks on their own?**

PN05: You can have a friend and you are in a place and get along well with each other and you do this job. You are both bored with doing it then you can come to an agreement and say, “I always save and you can also bring your savings so that we start business for selling clothes.” Then you combine your names and open one shop or boutique with your names written on it and one day you will remember.

**I: So there are people who can talk and others cannot.**

PN05: Yes.

**I: Okay. At what age does someone think of stopping? Is there an age where one thinks that she can stop? Eeh, number five.**

PN05: Where I reach a stage when I’m supposed to retire, so I don’t see that I can open the server [have sex], I will not want it so I’ll look for another business.

**I: So what is the age of retirement?**

PN05: Around 45years.

**I: 45years?**

PN05: Yes.

**I: Mmh, another person? Can someone’s age make them to stop?**

PN07: I say that someone’s age cannot make them to stop that work, your heart can tell you to stop and the challenges you face but if you see its benefits you cannot stop.

**I: Mmh, number nine your thoughts?**

PN04: I want to support number seven, sex work has no age and you might find an old woman where you work, she is old and well kempt and still does sex work.

**I: Mmh, what kind of jobs do young girls who have sex usually do after leaving sex work?**

PN07: The jobs that people do……you can find that you joined sex work unwillingly, maybe there is someone who had a dream of becoming a salonist , maybe someone wanted to open a boutique, someone might want to find money and open a second hand clothe business or sell chips and sodas.

**I: Mmh number one?**

PN01: Most girls who do that work are found in salons, apart from salon some work in bars.

**I: Okay, number three, what kind of jobs can they do?**

PN03: Maybe there is a job that you studied and you have not gotten it, so you will have to do this one [sex work], after finding that job, you can leave sex work.

**I: Okay, for the people who have started businesses, do they start it where other people are or there are differences, they are going to start it somewhere different like markets or they just open them whether in the market?**

PN07: It depends on where you think the business can do well. You can start it in a market or in the village, shops you can start in a village, boutiques you can start in the centres, a place where you think a business can go well is where you start.

**I: Yes, another thought, or they differently?**

PN04: Maybe I want to open a carwash but where I am I see a lot of carwash so it will force me to start my carwash somewhere else in a village where there is no other carwash.

**I: Yes, so the way you and number seven are saying is that it depends with the location not that your sex work makes you to move to a place.**

R: No.

**I: Okay, is there anyone who knows a girl who has stopped sex work and started again? She stopped and started again. Is there anyone who knows?**

PN08: I saw a girl……I know someone who stopped and started again.

**I: What are the reasons she started again?**

PN08: During COVID, were not working and also the business was low. She stopped and started selling tomatoes and when things picked up she again resumed.

**I: Mmh, is there anything bad that happened to her when she resumed, resumed sex work?**

PN08: Nothing.

**I: Nothing.**

PN08: Yes.

**I: And any other good thing that happened to her when she returned to sex work? (Laughs)**

PN08: I don’t know.

**I: You don’t know any good thing that happened to her?**

PN08: Yes.

**I: Okay, anyone else who know someone who stopped and started again?**

PN04: I had a colleague who stopped, she did not find umm… she found a client (male partner) who told her that he wants to marry her. So you also agree and live with this person, but the marriage did not work out because he was a polygamist, he comes to you and he has a family but does not tell you, so it was all about violence and she became tired and she had already had two kids so she went back and by the time you are going back you are not the same young girl as before you got married. So there are little changes because you are now a woman. Even if you put on make-up but you are still a woman that is why she still went the other side.

**I: Is there anything bad that happened to her when she returned to sex work?**

PN04: She told me that she doesn’t find clients (male partners) like she used to.

**I: And anything good (laughs) if there is?**

PN04: (Silence).

**I: There is no good thing that happened to her when she returned to sex work?**

PN04: A good thing that happened to her later was that she had the problem immediately she came back and when she god used to the field [hot spot] she continue receiving more clients and some of them were paying well.

**I: Mmh, okay, another person, number six do you know anyone who stopped and returned again?**

PN06: I know someone who stopped and started again, she left because she found a client (male partner) who agreed to live with her, right?

**I: Mmh.**

PN06: She got pregnant and rested a while, then there were some disagreements and when the child grew up she stopped and returned. She tells me that, that work has good money that she cannot stop.

**I: So that is a good thing that happened to her when she returned.**

PN06: Yes, but there were some challenges that she faced.

**I: Like?**

PN06: Even if she went back, she just went back but there are challenges. You can meet a person who just wants to do bad things to you until you wonder how he is.

**I: Okay, are there things women who have sex would want to do before they stop? We are moving well number one, don’t get tired. Are there things that, let’s say there is someone who has thought of leaving sex work, are there things she would like to do before she stops so that when it comes to a point when she wants to stop then she has done the thing that she desires to do.**

PN01: Some have big hotels, some have rental houses, so when they stop sex work they have money from the rental houses.

**I: They have built somewhere?**

PN01: Yes they have built somewhere. So even if you relax in the house, you just receive money.

**I: Another thing that they would like to do or that you would like to do before you leave sex work, let me put it that way, number four.**

PN04: What I would like to do is teach the youths so that one day I can remember that… I teach them so that they should not also go through… join sex work. So I give them challenges, disadvantages and advantages that are there so the choice will be theirs, you teach them thoroughly so that they don’t join sex work.

**I: Mmh, number five, anything that you would like to do before you stop?**

PN05: (Silence).

**I: It has disappeared? Mmh, number eight, something that you would like to do before you stop?**

PN08: I think that I should look for something to do [business] that can bring me money so that even if I stop I have little income.

**I: What can that thing look like?**

PN08: Just business.

**I: What kind of business? That you think of now, what type of business?**

PN08: I can sell shoes.

**I: Shoes?**

PN08: Yes.

**I: Okay, mmh, number two something that you would like to do before you stop?**

PN02: (Silence).

**I: You have no plan? If you have no plan you just say, there is no plan?**

PN02: No, I have a plan, what I would like to do before stopping is to start a business, so that when I stop I can find something little.

**I: What kind of business?**

PN02: I would like to open a bookshop so that when I stop sex work I will be there selling to the public.

**I: Okay, number nine, something you would like to do before stopping? Do not sleep.**

PN09: I’m not sleeping.

**I: Mmh, or you have no plan?**

PN09: I have not thought of it.

**I: You have not thought of it?**

PN09: Mmh.

**I: Okay, number seven, something you would like to do before stopping?**

PN07: Something I would like to do?

**I: Mmh.**

PN07: I would like to teach my peers who have just joined sex work are still not deep in. I will tell them the good and bad things that are there. So before they get used to it they can decide to continue or stop, this is what I would like to do.

**I: Mmh, number three, something that you would like to do before you stop? (Baby crying)**

PN03: (Silence).

**I: Or you have no plan? You have not thought of it? Mmh.**

PN03: (Silence) (noise in the background) nothing.

**I: Number one, something you would like to do before stopping?**

PN01: I would like to start a small business.

**I: A business, for what?**

PN01: A business for selling clothes.

**I: Mmh, number six, something you would like to do before stopping?**

PN06: Something I would like to do before I stop……..as a business woman and also as a sex worker, someone may take you as a wife but there is a way they will view you, you will start well but later on he will view you differently so what I would like to do before I stop is that I can buy a piece of land and build a house then live with my children there rather than someone seeing you differently.

**I: Have you started doing that or it’s just a thought?**

PN06: [Laughs] I’m not yet capable but I have that plan.

**I: Okay, is there someone who knows any girl who has stopped having sex in the last five or ten years? Who has stopped completely? Is there? Number five do you have someone who has stopped?**

PN05: I had someone.

**I: What made it easy for her to stop?**

PN05: Her first husband frustrated her then she went back and when she went back, she found that, that life was different. She met a client (male partner) that she has never met before who wrong her then she left the job.

**I: So she left sex work completely?**

PN05: Mmh, she left completely.

**I: You have said she was wronged, (participants laughing in the background), how was she wronged?**

PN05: There is a way where by the person she met messed up with her rights a bit and it affected her.

**I: Okay, number nine, do you know anyone who has stopped?**

PN09: No.

**I: You don’t know?**

PN09: Yes.

**I: Mmh number eight?**

PN08: No.

**I: You don’t know?**

PN08: Yes.

**I: You are feeling hungry? (Laughs), we are almost, number three, do you know anyone who has stopped? If you shake your head…..say it, this does not capture that.**

PN03: No.

**I: Okay, number one, do you know anyone who has stopped?**

PN01: No.

**I: Uh, number two?**

PN02: I don’t know.

**I: You don’t know, number six?**

PN06: I don’t know because it is also difficult to stop it.

**I: Mmh (laughs).**

PN06: You find that someone does it and goes back. I can say that the girl who was doing it……she was doing it but there are things she experienced that when she tells you about it may make you to stop but she still had plans to go back and has also experienced some of them. so I think it is difficult to stop completely (baby crying).

**I: Mmh, number seven do you know anyone?**

PN07: No.

**I: You don’t know?**

PN07: No I don’t.

**I: Okay. So we already talked about Jitegemee earlier…uh…and I explained to you that it is done to see that young women who have transactional sex have money that they have saved that can make them refuse to have sex without a condom or rest from having transactional sex when they want to rest. I also said that it is about women who have transactional sex saving part of their money themselves so that they can use it when there are no male partners who pay them or to help them in future after leaving transactional sex. This is the last step, we are going to talk about Jitegemee as we explained earlier, okay?**

R: (All) yes.

**I: Lets cheer up, number three put down your phone for a minute, and we are at the last step. Do you think Jitegemee as we explained earlier is something that women who have sex can agree on?**

PN07: I can agree with it, about Jitegemee how it was explained to us.

**I: What can make you agree with it? (Baby crying)**

PN07: What can make me agree with it is to stop having sex with men, the little money that you save, you open a business with it and you depend on yourself, the things that you want, the time that you want them and the time that you want to make them or if you want to buy something that interests you, you struggle and sane money then you buy it. So you don’t have to depend on someone.

**I: Mmh that is number seven’s thought, what of the rest? Women who have sex, can they accept Jitegemee? Number nine, do you think they can accept to join jitegemee?**

PN09: Mmh.

**I: They can accept?**

PN09: Yes.

**I: Mmh number eight?**

PN08: They can agree.

**I: They can agree? (Baby mumbling).**

PN08: Yes.

**I: Mmh number three?**

PN03: They can agree and others cannot agree.

**I: So it’s 50/50, eeh number one? (Baby crying).**

PN01: They can agree.

**I: They can agree? (Baby continues crying).**

PN01: Yes.

**I: Mmh number two?**

PN02: They can agree.

**I: Mmh number six?**

PN06: I can agree because even as per now, I’m independent, I have rented a house and I pay the rent on my own. I don’t want to know that there is someone who can pay for me and I don’t have time to ask him, I don’t see the need of asking him. That is why I save so that when end month comes I go and withdraw and pay directly because when I’m independent it helps me. But when put my mind on another person, he can delay and the landlord might come to my door. So I feel that when I’m independent then it’s good so I’m not bothered by anyone, he can come and go but I still go on with my life.

**I: Mmh number five, can they accept or not?**

PN05: I think they can agree.

**I: They can agree?**

PN05: Yes.

**I: What kind of women or who have what type of characteristics can accept Jitegemee?**

PN07: Jitegemee has no attitude, it’s your heart that decides for you whether to join Jitegemee or not.

**I: Mmh, so someone’s heart.**

PN07: Mmh.

**I: That is number seven’s thought, anyone with a different thought? What kind of character or women who have what kind of attitude that will agree to be in Jitegemee? No idea? Number eight, what kind of women will agree to be in Jitegemee? Your thought? (Baby crying).**

PN08: Women like us.

**I: Okay, so of the 10 women that you know, how many will agree to be in Jitegemee? Number six, of the ten women that you know how many can agree to join Jitegemee?**

PN06: Six can agree and four can refuse.

**I: Mmh number two?**

PN02: Five can agree and five can refuse.

**I: Five, mmh number one?**

PN01: 2 can accept but 8 reject.

**I: Two? Only two will agree?**

PN01: Yes.

**I: Mmh number three?**

PN03: (Baby crying loudly) the ones you tell and are able to see its benefits can agree.

**I: How many, of the ten that you know, how many will agree?**

PN03: Five.

**I: Five, mmh number eight, how many will agree, of the ten that you know?**

PN08: Six people.

**I: Eeh six people number nine, of the ten that you know?**

PN09: Four.

**I: Number seven, of the ten that you know?**

PN07: Of the ten that I know, you cannot know, only one can know themselves, so one can decide if they want to agree or refuse.

**I: But they are your friends, you can’t guess which one’s can agree and which one can refuse?**

PN07: Among my ten friends, six can agree and four can refuse.

**I: Six, mmh number four, how many can agree to join Jitegemee of the ten that you know?**

PN04: Five can agree and five can refuse.

**I: Mmh number five?**

PN05: Six can agree and four can refuse.

**I: So we all have those who can refuse right?**

R: (All) yes.

**I: What can make them not to agree or refuse?**

PN07: What can make them refuse, he will find that stopping that job is difficult for her because she is used to it, she knows how she can get her income, so it can be difficult for her.

PN06: The ones who can refuse, she can feel that she is goes to work but don’t always purchase anything, she earns and uses the earnings, and still don’t need to keep money to replenish stocks. So she can feel that she can purchase something take it to the market but she is not able to sell it, two or three people passes without buying and she gets discouraged. He can even decide to go back home thinking that people don’t want to buy from her. It is a must that the business will fail and she will stop. Because she will feel that in the other business she purchases nothing but still earns.

**I: Number two, why will the rest refuse to join Jitegemee?**

PN02: It depends with how someone is if she wants to stop, because she can refuse to stop depending on how she gains on this other side, so she feels it’s hard for her and may want to stop.

**I: Okay but we don’t want to stop, we just want them to join Jitegemee while they are still in sex work, we have talked of ten people and everyone has said the numbers who will agree to join. Jitegemee is all about saving, you save your own money and you decide how much want to save and the time you will to save it right? So among the remaining eight people, number one (laughs) why will they refuse to join Jitegemee?**

PN01: Some have children to look after and to pay school fees for, so maybe you have opened a business to sell groundnuts, the money you make will not be enough to pay school fees, especially those in secondary school a lot of money is needed. So even if you get one thousand shillings or five thousand shillings, you take it to the school for school fees. So that job [sex work] helps them better than businesses. You know in businesses you can only get like twenty shillings, that one you can save up to five years to pay for school fees and that will be when then child has been sent home.

**I: Is there something we can do to add. What can we do to add if possible the number of women who have transactional sex to agree with Jitegemee. What can we do to make the women who have transactional sex agree to join Jitegemee?**

PN07: We can come together as Jitegemee group who know each other, then we sit down with them and teach them, we share with them our thoughts, we tell them the advantages and disadvantages. Always they do know the advantages and disadvantages so we teach them about Jitegemee and those who are willing will join Jitegemee.

**I: So if it is about teachings, what should it be about or what kind of teachings?**

PN07: It should be about how to save money and build your life and how we can be independent.

**I: Mmh another thought, what can we do to make women who have transactional sex to agree with Jitegemee?**

PN06: You can tell stories with your friends, even when you are walking on the road. You start telling them one or two things how you met in sex work, you can tell stories and tell her ‘this work we are doing is like this and the way I’m seeing it, you should also have another business. So you can take loan and start a small business because there are losses and profits, so when you incur losses in one then other you gain profits’.

**I: Okay. Mmh number four?**

PN04: (Silence).

**I: You are still thinking? Number three, what can we do so that women who have sex to agree with Jitegemee in matters of saving?**

PN03: You can agree with her to take a loan and start a business, then you will repay bit by bit.

**I: Okay number nine, what can we do so that women who have sex to agree with Jitegemee? Or what can we do so that you agree with Jitegemee, for you to agree to join Jitegemee, what should we have?**

PN09: [Silence].

**I: Nothing? Mmh.**

PN09: [Silence].

**I: If there is nothing you should say because when you are quiet I don’t know what you are thinking or are you still thinking? The point has disappeared?**

PN09: Yes.

**I: Okay, number nine’s point has disappeared, mmh another person, what should we have for girls to agree with it? I only want one point (laughs) and number two? Nothing? Mmh number one, any thought? What should we have so that…..even if it is you for you to agree to join Jitegemee, you can think it like that? What should we have?**

PN01: The thought I can have to leave…..

**I: For you to join (laughs).**

PN01: For me to join and leave the other side because the disadvantages I’ve seen the other side [sex work]. You might find a partner but he has an infection maybe they have infected you with sexually transmitted infections (STIs), so you use his money to get yourself treated. That is when you can decide to leave that job and be independent, because I am the one running loses. The money that he gave you will be used for treatment. You end up adding your own money to cater for treatment.

**I: Mmh.**

PN01: Someone can also insult you, harass you, maybe your family may not want you and you can even be chased away from home.

**I: Mmh. Even if you don’t leave it, what can we have so that you have a connection with Jitegemee and you still continue with your work? What should we have even if you don’t leave it, number eight?**

PN08: We should have a theme that can help us get income like a business, so that we get little that we can save.

**I: Okay, do you think Jitegemee can violate someone’s right if she joins?**

PN06: There is no day that Jitegemee will violate someone’s right.

**I: Mmh, that is number six thought, number four, do you think it will violate someone’s right?**

PN04: It cannot violate someone’s rights.

**I: Mmh, number five?**

PN05: It cannot violate someone’s rights.

**I: Mmh, number two?**

PN02: It cannot violate someone’s rights.

**I: Mmh, number one?**

PN01: It cannot violate someone’s rights, it can only help.

**I: How can it help?**

PN01: It can help in thoughts.

**I: Thoughts in what way?**

PN01: How you can increase your business.

**I: Okay, number three can it violate someone’s rights?**

PN03: It cannot violate.

**I: It cannot violate, mmh, number eight?**

PN08: It cannot violate.

**I: Mmh, number nine?**

PN09: It cannot violate.

**I: Mmh, number seven?**

PN07: It cannot violate, it can help someone.

**I: Mmh, in what way?**

PN07: It can help someone with ideas for her to be independent to come from sex work.

**I: Okay, is there any challenges that we can face when we want to bring Jitegemee program? Is there any challenges we can face? Challenge.**

PN06: There can be a challenge in Jitegemee depending on… sometimes you have bought things… I mean Jitegemee needs patience because you have started a business and it is not going well. So it needs patience not lack of patience, so it maybe that you bought things with a lot of money and remained with little. So you have to be fast even if it is small, just start with the small one then you will get more ahead.

**I: Okay, so like number seven told us that we can teach girls how to save such like things right? Those are the things that should be in Jitegemee right?**

R: (All) yes.

**I: The time that we want to come and teach the girls, what challenges can we face? Yes number one.**

PN01: There can be a lot of challenges, some girls can say that it does not help them. Some can refuse by saying they went but was not helped and will not return. Some can agree, some can incite their friends by telling them that what those people are talking about does not help, let’s not go, by this you can lose majority and remain with even two people and majority have left.

**I: Ooh, that is number one’s opinion, mmh, thank you. Another thought, another challenge we can face? Challenge, another challenge we can face? mmh, before I go to another person, number one and the challenge you have talked about, how can we avoid it?**

PN01: How it can be avoided?

**I: Mmh.**

PN01: You can tell the remaining two to talk to their friends and convince them to come back. So she can convince her fellow by telling them, “Let’s go there because you will get something small,” they will come back because people love money, they will come back a big number and you will end up having a good number.

**I: Mmh, thank you, another challenge we can face number six, is there challenges we can face when we want to bring Jitegemee to the girls?**

PN06: You are asking that if you are bringing Jitegemee you are removing them from…

**I: No. they don’t leave, Jitegemee is just about savings**

PN06: You just bring them…..I don’t see a challenge.

**I: No challenge, okay, another person, number nine your thought, what challenge can we face?**

PN09: No challenge.

**I: No challenge, number seven?**

PN07: There is no challenge because challenges can be there or not. Some people love development and have thoughts of development while others do not. So others can see that saving ...first they will want to know what kind of program Jitegemee is, how it helps and then we try and explain then she decides if she want to be part of it. If it’s a program… you know there are programs that lie to people and programs that are have been approved by the government. So she will see that it’s a program that has been approved by the government then she decides to participate then we continue.

**I: Mmh, those are people who have agreed, what of those who have refused the challenges we meet that makes them not to agree, what can we do for them to listen to the information we have?**

PN07: We can talk to them. Listening to something is just talking, you just talk to them and explain then they can decide.

**I: Okay, number four, is there a challenge we can face?**

PN04: There are no challenges. Us as women we love development.

**I: Mmh number two, any challenges we might face?**

PN02: There are no challenges.

**I: No challenges, okay. So when saving in Jitegemee, let’s say you have joined as a young girl who is having sex in Jitegemee, you should save your money right, all of you know that Jitegemee is all about saving, your money that you receive on a daily basis or weekly, you take little and save, okay?**

R: Mmh.

**I: So when someone joins Jitegemee, how much can they save on a daily basis with the responsibilities you have and without straining, how much can you save weekly if you join Jitegemee, number seven?**

PN07: I can save two hundred shillings weekly.

**I: Weekly two hundred shillings, number nine?**

PN09: Two hundred shillings.

**I: Two hundred shillings, number eight?**

PN08: Three hundred shillings.

**I: Three hundred shillings number three?**

PN03: One hundred and fifty shillings.

**I: One hundred and fifty shillings, number one?**

PN01: One hundred shillings.

**I: One hundred shillings, eeh number two?**

PN02: Three hundred shillings.

**I: Three hundred shillings, number six?**

PN06: One hundred and fifty shillings.

**I: One hundred and fifty shillings, number five?**

PN05: Two hundred shillings.

**I: Two hundred shillings, number four?**

PN04: Five hundred shillings.

**I: Five hundred shillings, okay so let’s say that that is the target everyone has set, right? According to how you know yourself and how you live.**

R: (All) yes.

**I: Let’s say that you have not achieved that target, this week you have not reached your target, what can you do to get that target? Number five.**

PN05: I can try and look for something that can bring that money that week even if it is sponsor or anything for me to get that money.

**I: So you can look for a sponsor if you have not reached the target?**

PN05: Yes.

**I: That is number five’s opinion, yes number six what can you do to reach the target?**

PN06: That target?

**I: Yes.**

PN06: If I have not reached that target, I can look for fifteen shillings or ten shillings to cover half of it.

**I: So you save daily, little by little.**

PN06: Yes, I have a target for it to reached one hundred and fifty shillings but I can’t, which means I had a plan to save twenty shillings and I can’t so I save ten shillings. So the little I get I will save.

**I: Okay, number two what can you do to reach the target?**

PN02: If I don’t achieve the target then the following week I’ll have to save double.

**I: What do you do?**

PN02: I save double. So I save three hundred this week and last week so I get six hundred, so that the week doesn’t end with me not saving.

**I: So what will you do to get the double? That is what I want to know (laughs).**

PN02: It depends, maybe I met a fat client (male partner) [client who pays well].

**I: Okay, so….**

PN02: He gave me a good amount of money.

**I: What if the fat client (male partner) is not there? (Laughs)**

PN02: (Silence).

PN05: If there is no fat client [client who pays well]. then this other side is not bad, maybe I have a chicken or I have planted sukumawiki beside my house, I can take to the market.

**I: So number five you can sell something that you have?**

PN05: Yes.

**I: Okay. So when you say a fat client what do you mean? Fat in terms of body size or fat?**

R: (All) who has money.

**I: Who has money, okay, number one what can you do if you don’t reach the target this week, what can you do?**

PN01: I will let it be.

**I: You can stop saving?**

PN01: I let it be then the next time I start a fresh, you cannot force things, and if it’s difficult it’s difficult.

**I: (Laughs) so there is nothing you can do, you let it be?**

PN01: Yes.

**I: Number three what can you do to reach your target if you haven’t? That is the last question.**

PN03: I can stop then start a fresh next time.

**I: Number one has whispered that to you, uh, number eight, what can you do to reach the target?**

PN08: I can try even if I reach half of it then the following week I will top up that week.

**I: So what do you do to reach that half, I want to know what you do?**

PN08: What I do to reach that half?

**I: Mmh.**

PN08: Someone can buy me……like where I work, someone can buy me soda and I don’t drink the soda instead I take the money and keep.

**I: So you make a deal with the person at the counter?**

PN08: Yes.

**I: Okay, eh number nine what can you do to reach the target?**

PN09: What I can do to reach the target, if I have known that this week won’t be a good one, so I start saving little by little so that when the day comes I shall have completed.

I: So you save little by little?

PN09: Yes.

**I: Okay number seven, when you have not reached the target?**

PN07: When I have not reached the target… if I am married and I have other partners outside, my husband can give me a hundred shillings and the other partner also, let me say they are four or two, they won’t lack all of them, one person can give me one hundred shillings and another can also give me one hundred shillings.

**I: Okay, those men are not there…..the fuel price has also gone high (laughs), they say let me leave these beautiful ladies and then the men are not there, what can you do?**

PN07: What I can do, I’m a farmer, I have planted maize and beans, like now people are buying and I don’t buy, I go and take my maize to the market to sell, currently 1kg is one hundred and twenty shillings, 2kg is two hundred and forty shillings, so I get two hundred and forty shillings so I save two hundred shillings and remain with forty shillings.

**I: Mmh you have reached your target. Number four, you have not reached the target, what do you do?**

PN04: If I have not reached that target and currently people are farming, so I decided to for people in their farms so that I don’t fail to reach the target that I set, so I’ll be doing something and fulfilling the target.

**I: Is there someone who has not responded or we have all responded?**

R: (All) we have all responded.

**I: So we want to save in Jitegemee right, we have joined Jitegemee, we want to save. Everyone has set their target, everyone has set how they want to save even if it is ten shillings or five shillings right, as long as you have your target, right? What is the best place to save, that you trust, number seven?**

PN07: A place that is good to save…….it’s good to save in an account, those who have KCB Mpesa.

**I: KCB.**

PN07: Equity bank these days has Mpesa right?

**I: Mmh. Number nine, where do you trust to do your saving?**

PN09: Saving depends with where you are used to, like for me, I’m used to saving in Mpesa.

**I: Mpesa? You won’t withdraw little by little?**

PN09: (Laughs) no (baby laughs).

**I: Okay number eight?**

PN08: KCB is good for me.

**I: Mmh KCB is where you trust.**

**NT: (Whispers) bank or Mpesa.**

**I: KCB bank or Mpesa? Number four?**

PN08: KCB Mpesa

**I: Number four**

PN04: I trust Coop bank.

**I: You trust the bank, uh number three?**

PN03: I can open lock savings account.

**I: Lock savings in Mpesa or KCB?**

PN03: Mpesa.

**I: Mpesa eh number one?**

PN01: Equity bank.

**I: Bank?**

PN01: Yes.

**I: Number two?**

PN02: Bank.

**I: Bank, any specific bank?**

PN02: Post bank.

**I: Post bank, mmh number six?**

PN06: Cooperative bank.

**I: Cooperative bank, number five?**

PN05: KCB bank.

**I: KCB bank not Mpesa, we have KCB Mpesa and KCB bank.**

PN05: Both.

**I: Both? (Laughs) okay, thank you so much for giving us this opportunity to talk to you and giving your ideas. Is there any question or any comment? Yes number six.**

PN06: I can comment by saying: I am impressed with your teachings. I find it good because if you have grouped people like this and maybe someone have something that was troubling them, so if we sit like this then what one talks about is different from what the other person thinks, so it sooths the heart. Thank you.

**I: I also say thank you, mmh another person? Any question or thought?**

PN07: I’m happy that you have grouped us in one place and to thank you for teaching us deeply like someone who dint save, now I will be able to save, we are happy and we say thank you.

**I: I also say thank you.**

PN01: I am asking that now that you are not giving us your contact information, how will we reach you if we have a problem?

**I: Our contact information are in the forms that we have given you.**

PN01: Okay.

PN04: I thank you very much. I have learned a lot, if I leave here I will go and teach my colleagues what I have learnt here today, I will share with them.

**I: Okay, thank you. There is no other person? Okay if there is none……number six?**

PN06: I have a question. Now that you have taught us and we are finishing, you cannot take our contact information to look for us? Or if we have a problem we are the ones to look for you?

**I: We will take your contact information later but on more we will just get you together like today, we will look for you.**

PN06: Okay.

PN07: I have a question, now you have taught us, will we meet you again to start saving, or is it just a teaching that you have to think for yourself or we will start something like a saving group?

**I: So the study we are doing is done once, so I cannot lie to you that we will come back tomorrow, we are doing it once because we are still collecting ideas from girls so that we see if the girls can welcome a program like this, before we come back. So if we have analysed the information that you have given us and we see that among the 500 girls or 499 have agreed and 1 has not agreed, so it’s a good thing because many have agreed compared to those who have refused. So according to your responses we will see a way forward but I cannot say, so for today is just today. So we hope that since you came today, you have open your minds right?**

R: (All) Yes.

**I: Or are you still the same as you came?**

R: (All) No.

**I: Okay, if there is no other thing, I say thank you for the opportunity you have given us. Otherwise I have nothing to say, okay?**

R: (All) yes.

**END OF SESSION**
