# Supplementary material for: Eat a little and save a little: A qualitative exploration of acceptability of a potential savings intervention to reduce HIV risk among female sex workers in Western Kenya
Source: PLoS One. 2024 Dec 19;19(12):e0310540. doi: 10.1371/journal.pone.0310540 (PMC11658496; doi:10.1371/journal.pone.0310540)
Supplement: S1 File — (ZIP) [file pone.0310540.s001.zip › Jitegemee Transcripts and Dissemination Notes for Journal/FGD O.docx]

**FACILITATOR NAME: LILIAN AKOTH**

**NOTE TAKERS’ NAME: MORAA ARASA**

**INTERVIEW LANGUAGE: KISWAHILI AND LUO**

**TIME INTERVIEW STARTED: 1319 HOURS**

**AUDIO RECORDING LENGTH: 2HOURS 10MINUTES 27SECONDS**

**FGD DATE: 28/APR/2022**

**FGD ID: FGD O**

**CATEGORY: BELOW 30 YEARS, RURAL.**

**I: Welcome, mmh. This is FGD study…FGD and this is Jitegemee Study FGD Number ‘’O’’ done within =Boro=. The date today is 28/Apr/2022, time started is 01:19 PM. My name is (Name of moderator) and the note taker is (Name of note taker). Okay so we want to start. My first question. According to the few details on Jitegemee, what are your first thoughts?**

R: My first thought

**I: Mention your number first. Your first thought according to jitegemee that I have just mentioned. Yes number 3**

PO03: My first thought, I think that is a very sensible thing to do

**I: You can maybe tell me the reason why?**

PO03: Because I see it good as a good thing, because after I have retired according to how jitemee is planning to carry out its program, I think that it is a very sensible thing that will give me benefits later after I have retired from my sex work.

**I: Mmh, another person again?**

PO06: Number six

**I: Number six**

PO06: For me, I am saying this on the issue of sex work, as for me it has really brought me from far and again it helps me a lot. I can now say, that I am giving my children education. So I am still proceeding with that job. For me I will not retire

**I: Okay. Thank you. Another person again? Another opinion again please.**

PO04: As for me I will retire

**I: Mmh, Number 4**

PO04: As for me number 4 I will retire soon because I have been saving, I have provided education to my children and some are still continuing and (laughs) I have started a business through this job and the business is stable. So, I am quitting it very soon

**I: Thank you, another person again**

PO07: Number seven

**I: Yes number seven**

PO07: Jitegemee has taught us very well. My children are still young and you know that job is really helping me in educating these children because they are still young and in lower classes, so, if I continue following the lessons from jitegemee I will still save and also when retirement reaches I will just retire.

**I: Mmh. Is there someone that wants to add? Mmh, number 5?**

R2: Number 2

**I: Ehe, Number 2**

R2: As for me I cannot retire because it helps me, I am using it to provide education to my children.

**I: Speak loudly so that your voice can be recorded**

R2: Loudly?

**I: Yes, I just say it. They will not…**

R2: I cannot retire because I still do not have that plan. That is my everything in the house.

**I: Thank you so much. So my other question is, what are the daily expenses that women who always do business carter for each one and how much does each one cost? When we start with our daily expenses. What do we always budget for daily? And how much does it cost us? Number 4**

PO04: The condom that we always use, we always carry it with us, we buy them.

**I: Everyday?**

PO04: Yes every day you must have it in your bags, soaps

**I: You buy soap every day?**

PO04: No, but when I buy it, that is also money. I count that has been spent and even the clothing.

**I: Mmh, so if we start with, we will come back to others later slowly. You start with that one of every day the one that…**

PO04: And airtime, the ones for the house or the ones for the body?

**I: Mmh! anything which you see, you spend on daily basis.**

PO04: Just the condom and airtime, the way we plan for our meeting. I always use a lot of airtime

**I: Participant four mentioned airtime and condoms. Another person again? The one that we spend on everyday**

PO06: I always spend on transport to go and meet with that my lover.

**I: Mmmh! Another person again, number one you can tell us something?**

PO01: I do not have an opinion

**I: That daily expenses that you have**

**PO04: No, everybody will have to speak. Everybody will have to speak**

**I: Ok we said that we won’t force but I think it is good for each one to just try and speak.**

PO01: It is good for everyone to speak, to say an opinion.

**I: That your daily expense, it is not like every day that you have to buy this and this, so what are the things that we buy every day in our lives?**

PO01: Number one, I have to buy food and eat well and then go there.

**I: Mmh! Another person again?**

PO04: Number 5 has not spoken, I am the prefect

**I: Mmh! Number 5**

PO05: It is just that of the condom and the airtime

PO04: You are copying me (laughs)

**I: Is there anything you can add again apart from these? Okay, she can also just spend on those ones, number two what are you saying?**

R2: It is just food.

**I: Food, thank you so much, so we have mentioned condom, airtime, so the condom, how much can the condom cost? How much is the airtime? Transport is how much? Food.**

PO06: The condom we need is of high quality like the ones with high standards. At least that one you are close but not one hundred percent but some percentage are a bit high because it is of high quality and cannot burst

**I: So how much can the condom cost you every day?**

PO04: Like two hundred shillings.

**I: Mmh! Thank you. And what about airtime?**

PO04: Two hundred shillings

**I: Daily?**

PO04: Yes

**I: Okay. There is somebody that mentioned transport, how much can it cost you every day?**

PO06: It depends with where you have gone to

**I: So if you can approximate.**

PO06: Two hundred shillings.

**I: Two hundred shillings per day**?

PO06: Yes

**I: Two hundred shillings per day, that is number six. She has said two hundred shillings. Mmh, number two what were you saying?**

R2: It depends with where you are going. Sometimes you are going to =Busia= and it is not two hundred

**I: Okay, so if you take a look what you can use daily on transport because you know that transport that you can use on a daily basis that this is the same transport that I can use on a daily basis.**

R2: You know this is our job, it depends. Sometimes you can go to town and then you use two hundred shillings then again someone contacts you because I am a client. You see, come to Kisumu that is also transport. You can spend one thousand in a day?

**I: In a day?**

R2: Yes

**I: There is someone who mentioned food, how much can food cost us on a daily basis?**

PO01: Two hundred and fifty shillings

**I: Two hundred and fifty shillings daily?**

PO01: Yes

**I: If you take a look at that, is it from breakfast to supper, two hundred and fifty shillings**

PO01: You will eat it your family and the children also. [People murmuring]

**I: Yes number one**

PO01: It cannot go beyond two hundred and fifty shillings

**I: Ehee! How much can it be?**

PO01: Up to three hundred shillings per day.

**I: Up to three hundred shillings per day?**

PO01: Per day.

**I: Mmh!**

PO01: Because I have children

**I: Mmh, okay. Thank you so much. So, that was for daily basis and what about that one that cost us to buy every week? Every week, once in a week, there are things that cost us every day, there are things we say that must cost us every week. So the ones that we buy every week.**

PO03: In every week, that is when I love to do this my work, Saturday.

**I: If you mention your job…**

PO03: That job of meeting with my friend [She meant sex work] or should I mention another name?

**I: Just feel free to mention it, just speak.**

PO03: That moment that I want to go for that work, I have to be smart. So that Saturday I have to dress well, even if it is a dress, a skirt or shoes, something new. So if you buy a dress it will cost something like eight hundred shillings, I will also go with shoes and it can cost five hundred shillings or eight hundred shillings before I include transport. So I will spend something like two thousand shillings in a week,

I: Like do you buy clothes or shoes every week.

PO03: Every week I have to look smart because I am going to make money.

**I: Thank you. Another person again. That one that cost you every week, everyone has her own opinion. Right? That thing that cost you every week [silence]**

PO04: Every week I must have three rolls of bhang, I must have three rolls of bhang every week.

**I: Bhang?**

PO04: Yes, because as for me I do not take alcohol and that is my steam

**I: How much can it cost you in a week?**

PO06: Per week?

**I: Yes**

PO06: Three hundred shillings for bhang

**I: Mmh! Another person again. Number 5 I can see you are just laughing. Can you give us your opinion?**

PO04: Talk [laughs]

**I: If we become active, that is when we will finish faster. Okay then, let’s leave that one because we are still thinking, if someone has thought about it, you can also tell me. So those expenses that someone can buy like twice a month or once a month. In a month you only buy them thrice or once or the way you spend your money once or twice a month.**

PO07: Number seven, hair style, if you want to plait your hair. One month I have maybe bought a braid then look for the money for plaiting so I the way the products price has gone up, so maybe it can cost one thousand shillings every month

**I: One thousand shillings per month?**

PO07: Yes

**I: Mmh, another thing again?**

PO04: Rent, make ups

**I: Mmmh! Number four.**

PO04: Rent and make up

**I: How much can rent cost you per month?**

PO04: Three thousand five hundred

**I: Another person, you have said make ups**

PO04: Yes

**I: Like, how much can make up cost you?**

PO04: Eight hundred shillings

**I: Eight hundred shillings?**

PO04: Yes

**I: Another person again? Yes PO06?**

PO06: For me, I always buy soap to wash that my thing [vagina], buy boy oil. I also need to crack that thing [laughs] because I have to crack that thing very well [laughs]. You just know your ‘kasarani’ [coded language] [laughs] I must crack it very well

**I: If you say your kasarani, what do you mean?**

PO06: My vagina. [Laughs]

**I: ooh!**

PO06: I have to crack it very well every month [people murmuring at the background] shave it

**I: shaving that part, that field [laughs]**

PO06: Yes [laughs]

**I: So like shaving that field every month, how much can that cost you per month?**

PO06: Per month?

**I: Yes**

PO06: It only costs me two hundred shillings per month

**I: Two hundred shillings per month?**

PO06: Yes.

**I: You have also talked about body oil**

PO06: Body oil that will cost me one thousand shillings. Because I am using a lot of body oil, on the hair, on the face and every where

**I: Speak it loudly so that this one [Meant the voice recorder] can understand, you know if you use gestures it will not know, just talk so that this understands.**

PO06: And also soap, I buy it and costs 100 shillings

**I: Per month?**

PO06: Per month

**I: Is there anyone that wants to add? Yes participant 2?**

R2: Panties

**I: Mmh! Panties?**

R2: panties are one thousand shillings

**I: Per month?**

R2: Yes

**I: Any other thing?**

R2: Only that one, so you change it if it’s good and when it reaches another month it is just also like that.

**I: Mmh! Is there anyone that wants to add?**

PO04: On number 4, in a month I have to save to help me on the school fees.

**I: So savings like, how much do you save per month?**

PO04: Three thousand shillings per month

**I: Three thousand shillings per month?**

PO04: Yes, two thousand five hundred shillings per month

**I: Okay, two thousand five hundred shillings or three thousand shillings?**

PO04: Yes

**I: Okay.**

PO04: And that moment when the business is stable I can even save five thousand shillings

**I: Okay, thank you so much. We will proceed and are there expenses that we can spend on in less than a month? Like there are those things that we can spend on in less than a month?**

R: Repeat again.

**I: Are there those that we can spend on in less than a month? Mmh! [silence] once in a month. Number 4**

PO04: Taking care of the parents at home.

**I: Taking care of your parents at home can cost you how much?**

PO04: Like one thousand five hundred shillings or two thousand shillings

**I: Mmh, one thousand five hundred shillings or two thousand shillings?**

PO04: Yes

**I: Okay, sorry. It was more than one month? Like every two months or one. In a year. Something like that.**

PO04: After every two months I have to visit my parents and when I have for visit, I need to have something at hand.

**I: Okay, number one, there is nothing? Remember if we do like this or like this [gestures] this device is not seeing [audio device] okay?**

PO03: What I can do in a month

**I: Mmh! In a year.**

PO03: In a month or even a year, it is something like the school fees, because school fees does not come in every week. It is something that comes, maybe after four months because a child in our surrounding schools you pay school fees every month. So you must have something a little to pay, one thousand shillings for food and school fees. Five hundred, five hundred, so now that is one thousand shillings.

**I: Mmh, like per month?**

PO03: Yes, so maybe if they are on holidays but when they are in school it is something that I am doing daily per month.

**I: Per month?**

PO03: Yes

**I: Is there someone that wants to add something again? Maybe once a year. Okay then, let’s proceed. The female sex workers, where do most of them get their money?**

PO04: From our clients

**I: Number 4**

PO04: From our male clients

**I: Another person?**

PO03: From those clients

**I: From those clients?**

PO03: Yes

**I: Another one again? Seems like number one wants to say something**

PO01: Those male partners are the ones that give us money

**I: Is there another source from those clients? It can be that we are getting money from…**

PO04: If the job is not doing well, I do take from my business

**I: Another one again? Okay, so from those clients maybe we … number four you had mentioned that you might be getting money from the clients and sometimes also from your business, like what is the main source, primary source.**

PO04: It is just from those men

**I: Okay. Is there anyone that has primary source of getting money apart from that? Number seven it seems like you want to…**

PO07: It is just from these men.

**I: Okay, so now, what are the specific reason that makes the female sex workers to use that thing they are using? [Silence] what are the reasons that makes the female sex workers to use money or spend on those things, they are spending on?**

PO04: For business to succeed.

**I: If you say, for business to succeed…**

PO04: For instance I cannot go when I am untidy, I have to buy soap, I have to groom myself and dress well.

**I: For other people.**

PO03: You must prepare yourself for you to get many clients and get money. You cannot go when you are untidy

**I: Number five can add us something [laughs]**

PO06: You people do not have clients [laughs] you are hiding [laughs]

**I: Why are we spending this money that we are using on these things that we are doing.**

PO03: In luo, there is a proverb that says, use money to get money

**I: Use money to get money. Another one**

PO04: Number two, you are very silent

**I: Why are we spending, what is it that make us use those money to buy or to spend on what we spend? What is it? The reason [silence] Mmh! In that, there is nothing? Number seven, just say it.**

R07: It is because you have to use money in a day to day basis, which is the reason why we have to make it and use it.

**I: Okay, and do female sex workers usually save?**

PO04: A lot

**I: That is number four.**

PO04: For me I have to save because I do not know about how tomorrow will be. Today the business might be good but tomorrow it might be bad. There are loses and profits.

PO06: Eat five and save five.

PO04: Yes

**I: That is participant number 6, you have said, ‘eat five, save five’**

PO06: You cannot eat it all

PO05: I have to save.

**I: Women that save, number three.**

PO03: Most of them do not save that money, when you think back, many do not save that money they take it and buy their needs and they eat, that money is just finished like that.

**I: Mmh!**

PO07: Sometimes saving is not easy

**I: Number seven**

PO07: Yes

**I: Just speak**

PO07: According to life situations, sometimes saving is not easy but it is good to save, the way we will learn here we will know how to save.

**I: Okay, number one, you wanted to say something**

PO01: They use it like that because they know they will get another one. Tomorrow …

**I: Mmh, thank you so much so those women that save money, why do they save money?**

RI: Some of them do not save because they know that tomorrow they will get another one.

**I: Thank you so much, so these women who save money, what are the reasons they save money? Apart from those that we had mentioned, yes number four?**

PO04: I am confused again. You have confused me.

**I: Why do they save?**

PO05: You save because you do not know how tomorrow will be, that is why they save.

**I: Mmh**

PO07: It is just the way she has said it. In case of emergency. You can get yourself in something, a child can be sick or even you can get sick [cross talk].

R04: The child can get sick and you can also get sick, that is number 04

**I: Let one person speak first, number seven had started first.**

R07: She has already said it. [Laughs] it is just the same things, in case of emergency, a child can get sick, so those are the reasons why someone can save.

**I: Thank you, number four, you were also saying something… okay for others, for the workers that save why do they save?**

PO01: They save because they have a child and you need to pay school fees, they need to pay rent, they should budget in the houses if you save then you can find how to help yourself.

**I: Mmh! Okay number two**

PO04: Number 4, if you save, even if you do not have a job, your day will just go well because the children will find food and everything will just move on smoothly.

**I: Mmh! Okay, like now, how frequently do they save, these female sex workers who save?**

PO04: Like for me, I always save monthly

**I: Monthly?**

PO04: Yes

**I: Another person again, number 3**

PO05: Number five

I: Number 5

PO05: That moment I am going to work, if I get something I will come and save.

**I: So that one, sometimes is it daily, weekly, or monthly?**

PO05: So it depends with how I will be invited, with clients, it is daily then I will save daily, if it is weekly then I will save weekly and if it is monthly then I will save monthly.

**I: Okay. Another person again, number six**

PO06: I do not have a story on that.

**I: You do not have a story on that. Like approximately how much do we save in a week? How much is it per month? Approximately how much?**

PO04: In a month, I always save two thousand shillings

**I: Mmh! Two thousand shillings per month, per month right?**

PO04: Yes and I save through women’s Mary-go-round.

**I: Another person again, for those women that save, approximately how much do they save in a week?**

PO03: In a week, I can save five hundred shillings because it is something that you have in mind and so in daily basis I have to save one hundred shillings and after a week I take it to another place because it’s enough I take it and put it somewhere else.

**I: Does somebody want to add or should we proceed? Is there any specific characteristic that female sex workers that save have? Is there any character that we can use to identify that those women are the ones saving? Number two [silence] are there any specific characteristics of female sex workers who save? Mmh, number 2**

R2: You can save because you want your life to change. You can do it then it reaches a moment you achieve something good and meaningful.

**I: Thank you. Another person? Are there specific characteristics of those female sex workers who save**?

PO07: We save because you may have started your business and you can add that money to expand your business

**I: Mmh! Those that want to expand their business.**

PO07: Yes

**I: Mmh. Any other opinion? Mmh, my eyes are on this side [laughs] please let’s be active opinion. Those female sex workers that save. Is there any specific characteristics that we can use to identify them that these are the type of women that save?**

R: Translate that statement in dholuo

**I: I should translate it in dholuo?**

R: Yes [laughs]

**I: Thank you, is there any specific character that female sex workers who save have? A character that we can say that the female sex workers who save have?**

PO04: They do not beg

**I: Say it, yes say it, number four**

PO04: They do not beg [laughs] they have that self-discipline

**I: She does not beg?**

PO01: Those that are in that business always have money so we do not beg.

**I: Mmh, okay. Thank you so much. And is there any specific character that female sex workers that do not save have?**

R: They always beg

**I: We mentioned that there are those that do not save, right?**

R: Yes

**I: Are there any specific characteristics of those female sex workers who do not save have?**

PO04: For those ones, they do not choose clients, even if they find that client of two hundred shillings they will just go for it but for those who save do not need those of two hundred shillings. At least they need a client that can give them reasonable money.

**I: They want clients that give them reasonable money?**

PO04: Yes

**I: I see, mmh, another person again?**

PO07: Those that do not save, maybe the clients can come to you but he is mean. Sometimes he has lied to you, you had sex with him and he did not pay you. Some clients have that attitude, he lies to you that he will give you mean.

**I: Another person again, number five**

PO05: No

**I: Those female sex workers that do not save, is there any specific character that they have?**

PO01: You will get that one of one hundred shillings, how much will you save? Nothing [participants laughs]

**I: Mmh, number 3.**

PO03: There are women that do that job or even girls and they do not save. Somebody like that you will find that she has been locked out of the house, and maybe she has a child, so she will request that you give her child accommodation for a night when she heads out for work because she does not save.

**I: Mmh! Okay. Thank you so much, so we have said that there are those women that save and those that do not save, right? So we have mentioned the reasons why those women that save always save right? And what makes it easy for those who save.**

R: What makes it…?

**I: Mmh**

R: Repeat it again

**I: What makes it easy for those who save to save?**

PO03: I think it helps her and that is why she saves for the use in the house.

**I: Mmh!**

PO04: The advantage

**I: Advantage, if you mention advantage…**

PO04: Advantages of saving that money

**I: Mmh! She looks at the advantage of saving that money.**

PO04: Yes, the way it will help her in future.

**I: Other people again? What is that makes it easy for female sex workers who save? The sex workers who save, what is it that makes it easy for sex workers to save?**

PO05: She saves because she is someone who have children and anything can happen to the child then you use that savings to help the child.

I: Mmh

PO04: For emergency purposes.

**I: Mmh! Okay**

PO04: Like sickness

**I: Thank you so much. Yes number 6**

PO06: She is saving so that she can pay for the rent.

**I: That is the reason why she is saving right?**

R: Yes

**I: And what is that make it easy for her to save for that? What makes it easy number seven you want to say something?**

PO07: What makes it easy for someone to save it is maybe the Mary-go-round. When I receive the money and put it in something else for it to help me in future. So what makes it easy is when I use it the way my fellow participant has mentioned through the Mary-go-round. So it will make it easy to save.

**I: Okay. And what are the challenges that those women who save go through.**

PO03: If you save I do not see any challenges.

**I: Mmh.**

PO03: There are no challenges because you save. If you have any problem you just use the savings and the problem is gone [laughs]

**I: Another person, what are the challenges that they face? Those women who save? The female sex workers who save?**

PO07: The challenges are that the moment you want to go and change your hairstyle you will find that it is also an expense and your savings will reduce. This is because maybe you want to plait Daniella weave Monsey chase style, so that is the challenge that we have in that process of saving or maybe you want to buy a new under pant of the latest design [laughs]

**I: So you will take that savings to buy it?**

PO07: Yes

**I: Okay. Another person again? Number two [laughs] okay.**

R: [laughs]

**I: Okay. We have mentioned challenges like… my fellow has said that there no challenges and another fellow has said that it will force them to go into their savings for them to look attractive because the clients also want them to look attractive.**

PO07: Yes, to look attractive because the clients want us to look attractive. Maybe he has invited you over and he gives you money, so you will have to change a style to look beautiful. You buy bra and change the hair style.

**I: So we have found the challenges that make them go into their savings. How can it be addressed so that they do not face those challenges?**

R: Repeat.

**I: This challenge that female sex workers who save face? How can we address the challenges?**

PO03: I think there should be an intervention like a Sacco for people like us because you cannot go there any time to take the money.

**I: If you say you cannot go there, where is there?**

PO03: If it is a bank or a Sacco you cannot just go and withdraw. So if you save it will be difficult to withdraw it.

**I: Any other number?**

PO07: So the way she has said, I support. You can open for us something like sex workers savings account, if we organize ourselves that will be a fixed savings account for us so that we will not spend money all the time.

**I: So that account should be a fixed account that do not allow them to withdraw anytime?**

PO07: Yes so that even if it is for a period of one year that is when you will take it.

**I: Any person again.**

PO04: If you have self-discipline you cannot just use the savings that you have decided to save. If you have self-discipline you cannot go for the savings all the time unless it is for emergency purposes.

**I: We had said that there are female sex workers that do not save, what do you think are the reasons why they do not save? What make them not to save?**

PO04: Maybe she is the bread winner and she is expected to provide for everything at home. Her personal family and even at her parents place. So it becomes difficult to save because sometimes the business reduces.

**I: Mmh! Another person again? For those women that do not save, what makes it difficult for them not to save?**

PO07: There are those that like spending. The moment they get that money she will just spend on expensive things because she thinks tomorrow she might not have a client.

**I: Speak loudly please so that the audio device can capture your voice. [Laughs]**

PO07: Some of us are spend thrift, so we spend on a lot of things even those that are not needed and that is the reason why the savings are less.

**I: Okay, number six you wanted to say something.**

PO06: Some people love food. When they get money they just focus on the shopping of food items

R: [Laughs]

PO06: She uses all that money on food, she cannot save.

**I: Another reason why female sex workers do not save?**

PO03: Giving birth indiscriminately.

**I: Meaning?**

PO03: You have so many children. If you come back from work, they will need food and if you have budgeted for food you will not have money to save because it has all gone on the house budget.

**I: Okay. Now what are the disadvantages of not saving? What are the disadvantages of not saving? [ Silence] Mmh, what are the disadvantages of not saving.**

R: Not saving?

**I: Not saving if someone has not save, what are the disadvantages of not saving?**

PO03: Because you are not aware when you will face the problems and you do not have anything and there is no one you can turn to. That is the moment you will experience problems but when you have savings you can go into the savings and handle the problems. So I think that is the main disadvantage

**I: Number two can you tell us something? Number five.**

PO05: If not for that, we are doing this job and you have to save because you have children. So in case the child has a problems or maybe there is no food that is when I will take from the savings so that is the reason we save.

**I: So that is why we save? Okay. Another person again, number one you want to say something. What are the disadvantages of not saving?**

PO01: There is a disadvantage because we do not depend on anyone. So if you do not save and you have a problem nobody will help you so you have to save even if you have received a little amount.

**I: Is there anyone who want to add? Okay. Sorry for that interruption. So we were saying that what are the disadvantages of not saving? Number two you are just quiet and you have put hands like this [showing with the hands] talk, please talk**

R2: Repeat

**I: What are the disadvantages of not saving? Just say it, even if it is in dholuo, just say it.**

R2: In case you have a problem you will feel shy. So it is good if you save

**I: Say it loudly so the audio can capture**

R2: It is good if you save money such that the landlord will not lock the house for you and the children should not be sent home for school fees.

**I: Thank you so much, the way I had mentioned, even if I have asked using a different language in Kiswahili and you want to express in different language please feel free and express it in that language.**

PO06: By not saving, your friend can come over and she was supposed to go somewhere far and she has invited you as a friend. You can transport to go such that you will not get something good that was there. So I think that is the disadvantage of not saving.

**I: So if there is an emergency you cannot handle it? Any other?**

PO07: Number seven I feel that if you do not save you may face some difficulties, so you may be trouble and be forced to borrow from people.

**I: You will be borrowing things here and there.**

PO06: Yes

**I: Mmh! I understand that.**

PO01: By lack of savings, you can find a client that is difficult to handle and you will not find your way back home. You will not ask him for transport.

**I: Mmh! If you feel that things are not right then you can walk out.**

PO01: Have you understood?

**I: Just let it be, this is capturing it and that is the reason why I want someone to speak loudly so that this one captures it.**

PO01: [Laughs]

**I: Eeh**

PO04: Uncle [client], it is always not easy with the uncles.

**I: That is number four. What are you saying?**

PO04: Uncle [client] clients that are difficult to handle

**I: How? [Laughs]**

PO04: He can even hit you at the back you have gone there and he has not given you anything and if you ask him for money he gets angry then you will not find a way to go back home. So it is good if you have some money with you such that in case there is an emergency you will find a way out of it.

**I: You run away even if you have not been given something?**

PO04: Yes

**I: Mmh! Okay.**

PO04: You can leave, you can run and even forget your underpants. Sometimes you are in Kisumu and you want to come back to =Siaya=. You can have money, buy new underpants, put it on and return home.

**I: Mmh!**

PO04: You buy it on your way back home faster

PO01: You buy one on your way home.

**I: Mmh, number one is saying that if it becomes difficult you leave him behind and buy a pant on your way back home.**

PO01**:** Underpants on your way back home

**I: Thank you**

PO04: It is said faster.

I: Mmh! [laughs] okay and now we are asking, I know we had mentioned the benefit of savings. Is there any advantage of not saving?

PO07: No

**I: Number 7 you are saying that there is none?**

PO07: Yes the advantages of not saving

**I: Number four**

PO04: There is no advantage

**I: Is there anyone with a different opinion?**

PO06: ‘There is no disease the son of [mentioned name]’

**I: Now I am lost [laughs]**

PO06: There is no advantage in short [laughs] you do not recognize this dollar Jabari’s song?

**I: Yes**

PO04: They were going to test him for any disease and there was no disease found.

**I: That is the reason why I want you people to teach me. [Laughs]**

PO06: Go and give the feedbacks first and then come back

**I: I should take back the reports and then come back to learn this things?**

PO06: Yes

**I: Okay. And where do female sex workers save their money?**

PO04: Some people save in banks, others on those Mary-go-round created by women.

**I: Mmh!**

PO01: Mpesa. I get back and immediately deposit it in mpesa

**I: Another person again.**

PO04: In mpesa it is very easy to withdraw

PO06: Home banks, number six.

PO05: In Mshwari

**I: Mmh! Number 7**

PO01: In Mpesa

**I: Number two, usually where do female sex workers save?**

R2: In Mpesa, banks

**I: Okay. Why do they love these sites to save? Those female sex workers that save in Mpesa?**

PO04: It is easy to withdraw from Mpesa [child crying at the background] and it also has security. If you save money there it is not easy for someone to steal it unless you have disclosed your detail

**I: If you say you…**

PO04: Those pin numbers, something like that.

**I: Thank you, what makes those female sex workers to save in Mpesa?**

PO07: Mpesa is so private

**I: If you say private, what do you mean?**

PO07: You are the only one who… mpesa is personal you are the one who can access your Mpesa

**I: Mmh, we have also mentioned bank. We have said that there are those female sex workers who save in banks. Why would they love to save in bank?**

PO04: I just see it similar to Mpesa

**I: Mmh, number 5, what is your opinion? Why do you think most female sex workers love to save in banks?**

PO05: I save in bank or Mpesa because of easy withdrawal, whenever I want to withdraw and go on my daily activities in case I do not have fare to where I want to go to.

**I: Mmh**

PO06: Maybe I have saved a lot of money that does not require me to save in the phone anymore and I should take it to the bank

**I: Mmh**

PO07: Accessibility of the bank. You might have gone to town and you have separated with the clients and you want to go there to fit the right time for the children. So it will be more accessible.

**I: Number seven I will request to come and sit here please.**

R: You speak with a low voice, are they seducing you? A girl come and sit here. The prefect is here

**I: Thank you so much [laughs] thank you eeh, eeh. Another person again. Okay**

PO04: Let them move. I am very comfortable. I am close to the audio device here. I want to sit close to the device you can cross over.

PO07: In a bank it is easy to withdraw money to buy food for the children.

**I: You can withdraw money to buy food for the children. Another person again? Why do some female sex workers love to save in bank?**

PO03: Because bank is good and that is the reason why some people love to save in banks.

**I: What is its’ benefits**

PO03: Because your money is safe there, that is bank and it is the reason why you save there.

**I: What about home bank, when I say home bank we all understand. Why do others love to save in house bank?**

PO04: That one has bank challenges. Someone can get in the house and find that home bank and take it which means you will lose all the money while in bank your money is there such that even if there is robbery at the bank you will just need your money.

**I: Mmh**

PO04: So in short it is security

**I: Security**

PO04: It is there at the bank

**I: And home bank? Why do some female s3ex workers love to save money in the house?**

R: You know we love each other. So if you love saving money in the house and maybe a certain friend of yours is a thief she will challenge you. Even the clients are sometimes bad, he has also come and steal from you.

**I: Steal from you if he has come to your house?**

R: Yes, he can steal from you, so I do not support home banking.

**I: Ooh! You do not support. Number one you had an opinion.**

R: Home bank.

**I: Why do some people love to save in home bank?**

PO01: Home bank is not safe in the house, you should save in Mpesa.

**I: Speak loudly please**

PO01: You should save in Mpesa

**I: Why don’t you like home banking?**

PO01: Because a client can come and time me when I have gone to bathe [laughs] the ones that have long hands [thief] so it can bring problems with my children when he is gone.

**I: Mmh! Thank you [laughs] we also mentioned Mshwari, why do some female sex workers prefer saving in Mshwari? Number two, I want you to help me there please.**

PO04: Number 2 is so quite

**I: She will try.**

PO04: She is not an active member

**I: Number five, do you have an answer?**

PO05: No, I do not have.

**I: I want us to give number two time because she is still thinking right? Another person?**

PO03: I think Mswari is the same as Mpesa they are similar.

**I: Mmh**

PO07: Mshwari and Mpesa has advantages because if you save you can request for a loan of five hundred, so I think it is an advantage

**I: Mmh**

PO06: Mshwari is good because a client can send you money and again reverse it [laughs] but the moment you save on the Mshwari he will not reverse

**I: Mmh**

R: Challenges…

**I: So Mshwari is the best because there is no reverse**

R: Yes

**I: Another one again, I can see we are just proceeding very well. (Participants murmuring) is there anyone who want to add. Mmh, okay. Do female sex workers live a life that is beyond their means? Do they live beyond their means?**

R: Please repeat

**I: Do the female sex workers live beyond their means?**

PO07: Yes, they have to be very smart, neat, dress well and decently. So they live…

**I: So they live…**

PO07: They have to live well

**I: Okay. Another person again. Do the female sex workers typically live beyond their means?**

PO05: I always live in the means of God.

**I: Mmh, like you find that your income is down [using gestures] but your expenses are up [using gestures] do that happen?**

PO05: Because, let’s say you have been invited to go and meet with a client, so you know, you must also look descent since someone can invite you and the moment he sees you, the way you look he will despise you. The business will not go well with you, so which means you have to live beyond your means.

**I: Okay. Another person? What do we think? Number 03**

PO03: No, on that one I do not have an answer

**I: Another person that can give me an opinion? Do we live beyond our means? [Silence] Mmh? They spend more than what they get?**

PO07: I have a clear answer, we spend more than what we get we have to maintain your job. So you have to spend a lot to retain the job. For the business to do well.

**I: For the business to?**

PO07: For the business to do well [laughs]

PO02: Number two wants to speak

**I: Mmh, number two say something. Allow number two to speak**

PO02: You must spend a lot of money because you respect your work

**I: You respect?**

PO02: You respect your work such that even you have a client he will also respect you

PO06: In short, you have to respect the office

**I: You have to respect the office.**

PO06: Because it’s the source of income.

**I: So that is what make you spend more than what you get? That is what we are saying right?**

PO06: Yes

**I: Another opinion, is there anything that makes us spend more than what we get?**

PO02: You must also be classic because the clients that will come are also classic and if you do not look descent even those of one hundred shillings can just come to you [laughs]

**I: Ooh, [laughs] you understand that?**

R: Yes [laughs]

**I: Okay. How can the female sex workers close the loop hole between what they get and what they spend in case they are using more than what they get so that they do not use more than what they get? What can be done on that?**

PO04: It depends with the stage you are in.

**I: Mmh, you can elaborate a little [laughs]**

PO06: It depends with where you go for that business which makes you to have a low income.

**I: If you say that it depends with where you go for that business, what does that mean?**

PO06: For example here in =Boro=. You know there are low levels of life, so clients will also come that has low levels of life and that will make you have a low life.

**I: For you not to go beyond your means, you should do your business where there is low life according to your means**

PO06: You should try so that you live a classic life

**I: That is why I am asking, for us to avoid the classic life that is beyond our income what can we do on that? Number seven**

PO07: On that you have to be wise such that even if you are in the sex work, you should also have another business like a side hustle business [laughs]

Po04: Side hustle.

**I: Number 4**

PO04: I have just mentioned side hustle

**I: Another person again, mmh [silence]. For us not to spend more than what we get what can we do so that we can handle this challenge such that we just live within our income?**

PO03: I think people should just live their lives on how they can manage it. There is no need of living a life that is beyond your means such that you only use the small income you get. There is no need of looking for something that is beyond you. It can even drive you to stealing

**I: Living beyond your means can lead to you to become a thief. Any other thing? [Silence] let us proceed. Do the female sex workers borrow money or get into debts? The female sex workers do they borrow money or get into debts?**

PO04: In our todays’ lives, you must have debts. Yes, because it can get hard and you can have debts.

**I: It can get hard until you have debts, is it a must?**

PO04: Yes, even Kenya has debts, it is a must.

**I: Mmh**

PO03: Human being must have debts, it is a must

**I: Another person who has an opinion? Mmh**

PO03: You people please speak, I have started feeling hungry time is not on our side

**I: Another opinion again? So that we proceed, there is another part that I want to read**

R: There is nothing to add on it

**I: There is nothing to add on it?**

R: So people must have debts?

**I: People must have debts?**

R: Yes

**I: Why**

R: It is only the mad person that do not have debts but normal person must have debts

**I: It is only the mad person that cannot have debts but a normal person must have?**

R: Yes

**I: Okay. And if it is a must for them to have debts, where do they borrow it from? There are those who do sex work and borrow money or have debts, where do they borrow money from?**

PO04: Some of us have business, so we look for small micro-finances to borrow money there.

**I: Any other?**

PO06: Sometimes when I do not have, I can borrow from my friend

**I: From your friend, another person again, let us speak, we are not doing bad the way I see it.**

PO05: I go to Mary-go-round then I borrow money from there

**I: Any other**

PO06: Sometimes when I do not have I can borrow from my friend

**I: From your friend, another person again, let’s speak we are not doing bad the way I see it**.

PO05: I go to Mary-g-round, then I borrow and they give me.

**I: Number one it seems like you want to say something**

PO01: No

I: Number 6

PO06: You can borrow from Mshwari

**I: You can borrow from Mshwari**

PO06: Yes

**I: And what are the main purpose of borrowing this money? What sort of jobs do we do with it?**

PO04: That is development

**I: When you talk of development, can you elaborate it a little**

PO04: I can purchase a land and construct a house

**I: Mmh, thank you. [Participant murmuring] She borrows for development, remember we said that it is not a must for it to be your experience but the experiences we know from a fellow sex worker. If they borrow money, why do they borrow money?**

PO03: People borrow money for different purposes. There are those that will borrow it for development, there are those who borrow it for food.

**I: For food?**

PO03: Yes so there is differences

**I: Another person again, which means that we can borrow for development to expand business or somebody else for consumption right?**

PO06: I can borrow it to pay school fees

**I: School fees. Any other reason why we can borrow this money. For those female sex workers who borrow money because you had mentioned that a normal human being must have debts. Why do they take this debts?**

PO07: You can borrow for personal needs, maybe you wanted to help your parents and you are stuck.

**I: You moved closer to the recorder. So please try speaking loud.**

PO07: You can borrow to help your parents or you might have a personal need and you are stuck.

**I: Okay**

PO06: I can borrow to expand my small business

**I: To expand your business, is there any different opinion?**

R: No

**I: Okay. And what do they routinely do to pay this money?**

PO04: Just from that our business that is what will pay back the money

**I: Just name the business, just call a spade a spade. I do not want to complete it for you.**

PO04: From sex work business

**I: Sex work business, you get clients and pay you right?**

PO04: Then I take it to other place.

**I: Any other person? What do we do routinely, the sex workers that borrow money for them to pay that money?**

PO03: I think there is nothing they can do apart from sex work because that is what she always do

**I: Another person again?**

PO03: Move to this side so that they can see your face [referring to a fellow participant]

**I: Another opinion again?**

PO01: It is the client that will bring the money to pay for that loan

**I: Clients? Is there any other idea apart from the clients? Or is it just that?**

R: Yes

**I: Okay. What do the female sex workers do to increase their income? Maybe like daily or monthly. Let us say like in a month you get five hundred or one thousand shillings. What do the female sex workers do to increase their income?**

PO04: If you have a business you will take from business or from sex work, so if you put it together, you will receive something good.

**I: Apart from business, is there any other thing we do to increase our income**

PO03: I do not see

**I: It is only from that business or you do not see because you do not have anything?**

PO03: No there is no any other job, so for my income to increase I have to engage in sex work

**I: And from this work, is there anything you do to increase your income? So that you get more money?**

PO07: You talk well with the client. You sweet talk a client because if you bring him close then the job will just be good.

**I: So the moment you sweet talk him, how do you do it?**

PO07: You ensure that you check on him even after work for him not to disappear

I: You make calls…

PO07: You make calls, so there will be love even if he is just a client.

**I: Thank you, so that can make him increase the amount of money another day?**

PO07: Yes

**I: Another person**

PO06: For me to hold that person very well, I will go for dust [witchcraft]

R: Rice [witchcraft]

**I: Ooh! You look for dust and then?**

R: To hold him strongly

**I: Ehe PO05**

PO05: I blow him the dust [witchcraft]

**I: You blow him dust?**

PO05: Yes, for me to hold him to avoid other people

PO06: To hold him very well

**I: Mmh, for him not to let you go? So how will that increase your daily income if you do that?**

PO06: After I have hold him, I will get my money and still find other clients. So I will find other clients but for him, I do not want anybody to be with him

**I: For him not to let go off you? Number 5**

R: Your husband will leave you. I can see he is being taken away. You are warned.

PO05: It depends with how to give him that food [vagina] if you deliver good services that will make you increase that income.

**I: Mmh, thank you, number you wanted to add something.**

PO01: No it has disappeared.

**I: [laughs] if it comes please just say it. Even if you have forgotten, okay?**

PO01: Let me just say it. I have remembered

**I: Okay, thank you.**

PO01: If you want to get money, you first look attractive. Put on your make up and then look for that other one. Which holds him, everything will just be smooth.

R: African chemistry

**I: What is that other one?**

PO01: The witchcraft

**I: Ehe**

PO04: The African chemistry

**I: That is African chemistry?**

PO04: African chemistry, yes [laughs] I am the strongest more than others here, that is me, I am so knowledgeable in this field more than others, they are my juniors. I am the prefect. You see if I point at someone, they stay quite. [Laughs]

**I: I have heard you.**

PO04: I have PhD in this job

**I: I have heard you. Okay, is there anything that someone wants to add? Mmh, and if female sex workers are getting clients, ooh, sorry, they do not get clients, if they have not received a client, what can you do?**

PO01: That day is not a good day. It was a bad day.

**I: So, what will you do? You have not received a client**

PO01: Nothing

PO02: It will force you to go back home

**I: It will force you to go back home? Even if you have not received anything?**

R2: [laughs] so what will you do?

PO01: That is the reason why it is good if you save because if you lack clients you will go to bank.

R: You withdraw the money that you want to care and tomorrow, you pray to God that if tomorrow reaches remember me.

**I: Strongly**

PO01: Yes strongly

I: Okay [laughs]

PO02: And by the way, we do pray before we leave for work, we do pray and even when you return back home you thank God.

I: Mmh

PO01: Because you do not know where you are going since it could be very difficult.

**I: Mmh, any other person, if we have not received a client, what do we do?**

PO06: If I lack a client, it will force me to go to a certain bar for someone to buy me a soda and see if I can exchange it into money to find something to go with in the house.

**I: You stay such that in case someone buy you a soda you change it to money**

PO06: Yes

**I: You do not drink it?**

PO06: I do not drink it so that I can get something that I can go home with.

**I: Thank you**

PO04: But if you have gone for a week and you do not succeed, then someone has bewitched you and jealous of you. So you just go back to that African chemistry [witchcraft]

I: So these African chemistry, how frequent can you go for it?

PO04: You have to go and check it every time so that its strength does not reduce.

PO04: This our job you cannot do it without witchcraft or normally, you have heard I always go and smoke bhang but if you have planned yourself with at least steam you cannot go naked. I have to smoke bhang for my head to work well… [Mentioned name] here on steam [laughs] [mentioned name] you feel you are high, how do you feel when you see me? You feel good?

**I: You have to add steam? [Laughs]**

PO04: Yes, I am not a sieve *okutaywa* [bhang], it has so many names. You know the police do not want it so it has its names

**I: That the police do not know? Okay, is there anything we can add on that? [Silence] there is nothing? And how will you know that now it’s this time and I have not received any client? How will you know that now I have not received a client?**

R2: You will just see the signs

**I: Mmh, which signs will show you that today you do not have a client?**

R2: You will just see how things are

PO03: You know the schedule of your office work, the moment you get in and the moment you start your work, when it reaches a certain time and you have not received a client, you will just know that today there is no job and then you leave

I: Mmh!

PO07: It also depends with the days of the month because in the middle of the month there are no clients

**I: How will you know that now it’s in the middle of the month and there are no clients? So how will you know that if it reaches this specific time, there are no clients, let me go back.**

PO05: It is always my job, so I know that if it reaches this time there are no clients and then I will go back home because it’s always my job and I know.

**I: Is there any time that we have set that incase it reaches that time we will know there are no clients?**

PO05: Yes, it reaches a time and I have not seen big man *bazu* [client] we always know that today we will just go home [participant murmuring]

**I: Ooh, [laughs] number… you were saying**

PO03: Number two has something to say

**I: Number 2 just say what you were saying, how will you know there are no clients or how will the female sex workers know that there are no clients today?**

PO02: That is your daily job so you will just know.

**I: So how will you know that now there are no clients?**

PO02: You know if today you have clients and tomorrow you do not sell it, maybe its 3.00 or 4.00 and the weather has changed.

**I: If you say 3.00 or 4.00, which hours do you mean?**

PO04: It depends, you know in sex work we do not have specific time even now I have received a message and I should leave [laughs] [mentioned name] you will leave this place.

PO04: She has received a message and she should be leaving [laughs]

**I: Let us move faster so that we do not interfere with the job. Okay how much can sex workers owe as debt anytime? How much can female sex workers who borrow money owe as a debt? Approximately how much?**

PO02: A debt that they can owe in a day or…?

**I: You can just tell me even if It’s on daily basis or monthly. Approximately how much can the female sex workers borrow as dept.?**

PO03: Everybody can owe a different size of debts. We cannot owe same debts. It could be that you do not owe anybody this month, maybe you just have a dept of two hundred fifty shillings or one thousand shillings or three thousand shillings or more, it depends with an individual.

**I: Mmh, okay, another person again. Approximately how much can female sex workers owe anytime mmh? [Silence] approximately how much do you feel female sex workers can owe anytime? Let’s say something on this then we move to something here. How much can someone borrow at a given time?**

PO06: People have different debts because maybe for me I borrowed money from someone and use it to pay school fees so maybe I have borrowed a lot of money like three thousand shillings and another person borrows five thousand and the other person does not have debts. So people owe different kind of debts depending on what people do with the money.

**I: Is there anything that someone want to add? Okay, we had said that what brought us here is to have an opinion according to the program of jitegemee, I should say it in Kiswahili, number five, I am sorry for that, you will forgive me. Now at the beginning we had talked about the jitegemee’ program its purpose to ensure that the female sex workers have savings that will make them decline unsafe sex or to take a break from sex work in case they need a break. I also mentioned that it will involve the female sex workers to save part of their money for use in case there will be no clients or help them plan for their times after the sex work. This is an intervention. Maybe it wants to educate female sex workers on how to save money that will help them in future, if she has decided to retire. People do not retire from everything right?**

R: Yes

**I: So maybe it has reached the moment she wants to retire, would you want to retire and go empty handed?**

R: No

**I: You would want that if you have retired from that job and you want to start a business at least you have something at hand, so something like that or maybe your clients are doing something that you do not like and you want to leave him and life has to move on**

R: Yes

**I: How will life move on if you do not have something at hand, so things like these are the things we say, remember that we have said that jitegemee will not give you money to save. It is your money that you get, it will educate and it will also be motivating you on how to save this money, right?**

R: Yes

**I: And with this money, also remember that with jitegemee there is nothing like if you want to take your money they deny you that there time is not over. Let us say, you have save your money for about two months and something has come and you want withdraw your money. You are permitted to take it without interest.**

PO03: Can I ask you a question?

**I: Yes**

PO03: Is this jitegemee a Sacco or a bank?

PO04: Is it a microfinance or…

**I: Jitegemee is the reason why we are here and that is a good question, so if you ask like that, such questions are what we are taking back to the office. Would you like it to be a Sacco, Mary-go-round or a bank? How would you like it to be? What are the things you would like it to have for you to accept it? Would you like it to be like a Sacco or like a bank?**

PO03: You know the way she is saying it, the way you save and the money you save it means that is something you have created even if it is a Sacco or a bank I cannot change it. So I would like to know if it’s a bank or a Sacco.

**I: Remember that it has not started by now and that is why I need your opinions**

R: But we just need to know.

**I: It is a good question, so we are saying that with jitegemee. If you have decided that you want to join jitegemee and you have decided to save your money, it will not tell you that you have to save here, you choose on your own where you want to save. If you want to withdraw it you will withdraw without any interest. For example if we say its women group, if you want to take a certain amount then you will have to return it with some interest?**

R: Yes

**I: In this one, it will be your money and you will not pay an interest. It will be your responsibility, it will be your hard work, the amount you want to save there. Do we understand each other?**

R: Yes

**I: Okay, you save the amount you are able to save. You save when you are able to even if you want to save daily, weekly or monthly. There is no interest in borrowing your money and you can take your money anytime you feel, okay?**

R: Yes

**I: So we have talked about jitegemee, I would like to ask some few questions on jitegemee then I have remained with few parts and we are through. So the way I have talked about jitegemee, what do you think? Is jitegemee something that female sex workers in Kenya can agree to join? This jitegemee**

PO03: I see it is a meaningful thing and they can accept it

**I: You can tell me briefly why you think it’s a meaningful thing**

PO03: Because if it is created like a Mary-g-round it can educate people other things so it is a meaningful thing if someone joins.

**I: Mmh, any other person? According to what I have said about jiotegemee intervention. How do you feel? Do you think female sex workers in Kenya can accept this jitegemee?**

PO04: If they educate people very well like you people, you can convince people to join. You are doing a good job.

**I: Thank you. Another person? We need your opinions, this is where we need your opinions. This is the main reason why we are here.**

PO07: I see it is educating well, so if people can agree to join those in sex workers because already your discussions has shown us how we can save even if do this our job and we save, it is very important to us.

**I: Mmh**

PO04: You have not discriminated us, you are dealing with different kind of people.

**I: Mmh, thank you, number 6, it seems like you want to say something? Number one, okay. What kind of female sex workers can accept jitegemee and why can they accept to join jitegemee? Which kind of female sex workers can accept?**

PO04: It depends with an individual

**I: Can you elaborate a little please what you mean by it depends with an individual.**

PO04: There is a way I will view it that another person will not view it like that. So we view it differently or we understand differently.

**I: What types of female sex workers, can accept to join Jitegemee according to how I have explained it. What types of female sex workers can accept to join? Number one**

PO01: The female sex workers that get clients who has a lot of money. Those that get at least a good money.

**I: Those that gets at least good money, what does that mean?**

PO01: There are those that do not good money so you know if you do not get enough money you will be forced to look for somewhere else so you can save.

**I: Okay, number three, you wanted to say something**

PO03: She has said it.

**I: She has said your parents [laughs]. Another opinion? What do you think? What types of female sex workers can accept to join this Jitegemee intervention? Mmh?**

PO03: I think all the female sex workers can accept to join this Jitegemee

**I: All female sex workers?**

PO03: Yes

**I: What makes you feel that way?**

PO03: There are no rules that it is for those who have children or do not have children, it’s something that helps so I think for those who know its benefits, they would like to join and those who do not see its’ benefits will also not join. You don’t force anyone.

**I: Mmh, we had said that it will not be something that you will be forced to join.**

PO03: Yes

**I: Is there anyone who would like to add something? Number two, would you like to add anything? [Silent]. What kind of female sex workers according to how I read about Jitegemee, how we want to put it, which type of sex workers would like to join. Sorry I have go you off gird but I know you will tell me another one. What kind of female sex workers will not agree to join Jitegemee and why? What types of female sex workers will not accept? Is there any women you think will not join the Jitegemee in intervention?**

PO02: They will not decline it?

**I: There are no female sex workers that will decline Jitegemee.**

PO04: That is something good they will all agree I have told you that you know how to educate and talk to people so that first approach always motivates people to give in?

**I: So, the approach that I have shown is what will make people to join or not to join**

R: Yes

PO03: I think the way you have come with Jipange it will

**I: Jitegemee**

PO02: Yes. Jitegemee it will make people to join because a lot of programs always come but it does not come to female sex workers, it discriminates us. So I think if this thing has come, and all people are educated on its importance they will join and they will like it.

**I: Mmh okay**

PO06: You will not know what a person thinks, you can see us all the way we are here thinking that we can join this Jitegemee but there is someone who thinks that she cannot join. So you cannot tell what a person thinks.

**I: Mmh, you cannot know? So it will just depend with how it has been implemented. So let’s say we have come and met a group of female sex workers, for example ten people, among these ten people how many percentage can accept to join the jitegemee intervention among those ten?**

PO03: What can make others not to join is because maybe this thing will be so public and people will know my job that is the reason why someone might not join. But if it will not expose my job then we will just join it.

**I: Thank you so much, any addition? Okay if someone remembers you can just say it. I should not leave out any opinion, okay? So we were talking about percentage, we have come and there is a group of ten and we have discussed about jitegemee intervention. How many people among these ten people can join Jitegemee intervention? Out of the ten, like how many can join?**

PO05: It is just like that my number, 5 people

**I: Your number, five people, that is 50 percent some people can join and some people cannot join. So on that I would like to ask, for those who would not join, why would they not want to join?**

PO05: Because people are not same, people are different, that is the reason why two to three people can join

**I: People are not same, people are different? Any other person? Out of the ten**

PO01: You cannot know what a person thinks

**I: Out of ten, how many can join?**

PO01: Five

**I: So you are also saying fifty fifty**

PO01: Yes

I: Your reason

PO01: Because you cannot tell what I am thinking and I cannot tell what you are thinking

**I: What is your main reason why you think others will not join**

PO01: Because you cannot tell what thinking and I cannot tell what you are thinking

**I: What is your main reason why you think others will not join**

PO01: You can view it as a good thing and I am not viewing it like that.

**I: Okay**

PO01: People can have different opinions

**I: Okay, another person again, number four what do you think?**

PO04: It is just fifty- fifty

**I: Fifty can join and fifty cannot join?**

PO04: Yes

**I: This five that will join, what do you think will make it hard for them to join?**

PO04: Let me think

**I: So I should give you few minutes to think?**

PO04: Yes

**I: Another person**

R: Sometimes, Jitegemee can become something like a women group and sometimes somebody doesn’t want her job to be exposed, so this can prevent her from joining Jitegemee.

**I: So, sometimes other people will not join?**

R: Yes

**I: So what percentage do you think can join out of this ten people?**

R: Just fifty-fifty

I: You are also saying the fifty -fifty, another person

PO04: I think if that name can be changed, that of sex work and replace it with maybe even ten sisters, something like that people can just join. That name is not favorable

**I: The name sex work can prevent someone from joining?**

R: Yes

**I: Thank you so much. Any other pinion?**

R: Ulcers is high

**I: Eeeh, this ulcers, I am trying to speed up. What else can prevent someone from joining? This Jitegemee, we would like that if someday it has been implemented then we would know which things to put in place to motivate people in joining because it’s something that will help people in future? So we want to know the things that will prevent someone from joining so that will prevent someone from joining so that we can amend those things that can prevent someone from joining. Do we understand each other? Number two has not just hold her head.**

PO04: We are on the wrong times, she had already received a message [laughs] she did not even take tea, she is hungry

**I: Okay, what can we do to increase the acceptance of Jitegemee on female sex workers to the highest number possible? For example, most of you had said fifty - fifty so you feel that fifty percent can join for us to increase the number of female sex workers who can join jitegemee intervention, what can we do?**

PO07: Just the way my fellow participant 4 mentioned, to change the name sex work

**I: To remove the name sex work?**

PO07: Because we just know it’s an intervention idea

**I: Thank you. Number 3, you wanted to say something**

PO03: TI is just a matter of talking with people and people will just change and join because the heading has just been written Jitegemee not sex work. I am not seeing anything wrong there.

PO04: You have not read that form very well that term is there.

PO03: Yes it’s there but not at the heading.

**I: That is her opinion [laughs] okay. Another person again, what do we think? What can we do to those, for example for the five people that will not accept joining, for them to join such that it only remains two people, what can we do?**

PO02: Just talking to them.

**I: Okay, just talking to them, is there anybody that will add on something? Let us now move faster –faster, eeh**

R: Nobody [participant laughs]

**I: And what are the things that Jitegemee are supposed to put together for it to be accepted? Jitegemee, what are the things it should put together for it to be accepted by the female sex workers? [Silence] should I read It in a different language? What can we put in place for people to accept joining jitegemee? What do you think if we do can make people to accept joining?**

PO07: Motivate the people

**I: How? Let he finish hers first. How do we motivate the knowledgeable ones?**

PO07: How will I put it?

**I: Just say it in any language, dholuo, Kiswahili or English.**

PO07: It is different to explain, somebody to help me

**I: Okay, umber three help us a bit.**

PO03: I think if they come with other projects that helps, people can make people join.

**I: If you say the programs that helps people, how?**

PO03: For example let us say our children has just seated for a natural exam [KCPE]. You know there are those that will not be able to raise school fees, to join form one, so the moment jitegemee is paying school fees for one student in a certain school, someone can join this thing knowing its importance and its support, that’s what I think.

**I: Another opinion again?**

PO04: We are grown-ups, so you should increase this sitting allowance

**I: So, that’s when there will be jitegemee because for today it was just to get your opinions**

PO04: It is small

**I: It is small**

PO04: Yes and we are grown ups

**I: Mmh another person? Number 6 and there is also no lunch**

PO06: No, I am just supporting my fellow

I: You were saying

PO06: I know even if it will be increased, it be increased if…

**I: That program has been implemented. We said there is no wrong opinion, we have accepted all the opinions, number one**

PO01: When will you come back?

**I: We still do not know because by now we are still collecting opinions, we were in Kisumu, in fact this is our first day in =Siaya county=. So for us to collect all the opinions and then after we are through with collecting the opinions, there will be something that will be done like the analysis to know that this are the things that are needed and again prepare a plan, it is something that takes a long process that I cannot tell you when I will be back**

PO04: That is good. Let us proceed, we are already through

**I: [Laughs] we were asking that what can be done for it to be accepted so that we proceed.**

PO02: For what to be accepted?

**I: Jitegemee. Is there any other opinion? Eey, this issue of message [participant murmuring]. According to what I read on Jitegemee what should we do what do you think female sex workers can like about Jitegemee and why? What do you think they would like to see on Jitegemee?**

PO03: Please respond to that question [laughs] I am not thinking anymore. [Participant murmuring]

R: You can also respond?

**I: If we can remember, we had said that Jitegemee is an intervention that we want use to educate the female sex workers on how to save and this savings will be your money. Jitegemee will not give it to you but it will just educate you on how to save even if it is daily, weekly or what… and anytime you want to take your money, you will be given the money without any restrictions that you have to take it after one month or after one year and when you take it, there are no interests. You just take your money if you want to take it tall or you want to quit, there will be no problem.**

PO01: Is it a must that only female sex workers are allowed to join Jitegemee?

**I: Thank you. That is also an opinion that we will write down and ask in the office. Is it a must, it will be you to decide. I cannot answer that myself. That has been wrote down, it is good that it has capture. How would you like it?**

PO01: So I would like because you came for our purpose right?

**I: That is the reason why I am asking you how you would like it to be. Should it be for the sex workers or mixed?**

PO01: It should only be for sex workers because if we mixed people, they will spread news about us

**I: According to what I have, what do you think female sex workers can like about Jitegemee? What is it that Jitegemee is planning to do that would interest them?**

PO03: That issue of savings is a good thing

**I: Mmh, can you explain bait? Why do you think that can interest them?**

PO03: It is helping us in saving money. That is why I am saying it is good

**I: Mmh**

PO07: Because you have educated us that if someone would want to retire, the way you have involved us on Jitegemee it will help us after retirement to get our money. She can retire to engage in something else.

**I: You can also compare it to Mary-go-round. We had mentioned that it is different from Mary-go-round, so if you look at Jitegemee, what do you think JItegemee can do that will make women to love it a lot according to what I was trying to say.**

PO03: Only the savings

**I: Only the savings, to help you in future?**

PO03: Yes

PO01: If you accept to give us loans any time we want without restrictions

**I: What do you mean by without any restriction?**

PO01: You do not ask for higher interest

**I: Thank you. Can somebody remember what we said about interest?**

PO04: There is no interest

I: Your money is your money. Even if you had ten thousand shillings and you have decided to take even five thousand out of the ten thousand. You will just take that money without any interest. If you want to give it back no one will request an interest from you. That is your money

PO07: That is why it’s good and we are happy about it

**I: Mmh, number seven you are saying**

PO07: It is good

**I: Why is it good?**

PO07: Because we save it, it will help us even if we save, you have said that it has no interest?

**I: Yes**

PO07: So we are happy about it

**I: Thank you, that is her opinion, number three, you had a question?**

PO03: Let us say I have been saving this money now that I am still working and it has reached a moment that I have retired and I need this money. So for example I have saved ten thousand, am I only going to be given the ten thousand or will I be added something on top it because when I save sometimes you people always use it

**I: The way you have save, that is your account**

PO03: You will just get that

**I: Yes, that exact amount. It is your money that you have save because you have plans with it**

PO04: Will there be need of kin because you can die. This our job is not a play, it can cut you short

PO02: Yes life is short [participant murmuring] even tomorrow [mentioned name] body will be removed from the mortuary

**I: So those are the opinions that you can tell us. What do you think?**

PO04: Next of kin is important

**I: It is good if there is next of kin?**

PO04: Yes

**I: Why?**

PO04: This our job, if I am not there who will take this money?

PO07: In case you die someone can take it

**I: In case you die, someone can take it?**

PO04: Ye, because it is risky.

**I: Thank you. Another opinion, what do you think according to what we have explained to do will make the female sex workers to have an interest in joining Jitegemee?**

PO03: You know you have said that Jitegemee want to educate people on how to save but the things you are doing deeply, I have not heard you mention them.

**I: Like which things**

PO03: Like something that you will do like this, is it just about savings?

**I: It is just about savings. They will start with that. Maybe if you have given us opinions, the ideas we want are from you. What else would you like it to do? We had asked something like that? Is there anything you would like us to do to motivate people to join?**

PO03: Talking to people well and after it has been implemented you can look how people have save and add something like loans. This will motivate people because it can help you when you have a problem. Since people go to places like banks because it can help them when they have a problem such that if you are there you can say that you are into something called Jitegemee, I have a problem with school fees so you can go there and they can help you with that and you can pay it back because everything is there. People will like it because it is something that helps

**I: Mmh! But according to what I have mentioned that if you take any money, you do not pay it back with an interest, you can save anytime even if it is daily, weekly or monthly. You can also take it anytime there are no restrictions. What do you think we want to do that will make the sex workers an interest of joining?**

PO03: I think it is good that will make people have interest if it does not require interest to pay it back. The way you have save money that is the amount you get, it is good because even in banks we save money and when you have gone to withdraw they have to deduct some amount. So if there is no deductions in this, I think it is good and people will like it.

**I: Thank you**

PO07: I just wanted to emphasize on next of kin because if something happens to you and you have a child that you can leave it to, so it is good and people will like it so you are the people to follow up.

**I: Number two you wanted to say something?**

R2: I am finished on the ground. Please try [meaning she is tired]

**I: You are finished on the ground. I am trying, I have remained with one session. Can there be any worry on value among the female sex workers on jitegemee? Can there be any worry on Jitegemee that female sex workers might think that violates their rights according to what we have read here? Is there anything you think can violate the rights of female sex workers?**

PO03: I am not seeing

**I: You are not seeing?**

PO03: Yes

**I: Number 2**

PO03: Speak, you will be removed from here [laughs]

PO02: I am also not seeing

**I: According to what I have read, do you think there is something that can violate the rights of female sex workers?**

PO02: No, I am not seeing

**I: You are not seeing**

PO02: Yes

**I: Is there any person with a different opinion? We have said that there is nothing that can violate the rights. And what are the challenges, do you think we can have if we implement Jitegemee and how can we manage them? Let us start with the challenges. What are the challenges that we can have if we implement jitegemee? Number 6, what is your opinion? Which challenges?**

PO06: I am still thinking, what do you mean by challenges?

PO02: Challenges how?

**I: The challenges that we can have if we implement jitegemee**

PO03: Shame

**I: Depending on?**

PO03: The name, so people may think that this group is just for certain specific people

**I: Mmh, specific people**

PO03: Yes, so that is the challenge, the challenges you think Jitegemee can have so that we see how we can handle these challenges. How can we handle these challenges such that it will not be there if people join Jitegemee? There are no challenges that we can experience? If we start Jitegemee? If we start Jitegemee? [Silence]

R: Please maintain the challenges

**I: Mmh, challenges, number one it seems you have an opinion, tell me**

PO07: There are challenges because sometimes you can save and you lose your money. [Participant laughs]

**I: You can save and sometimes you lose the money?**

PO07: Yes [laughs] even in this Mary-go-round you can save a lot and someone runs away with the money

**I: So what do you think we can do about that for us to solve such like a challenge?**

PO07: It is just encouragement and trusting the person in-charge of the money, so I don’t know how we can trust them.

**I: So it is trust right?**

PO07: Yes

**I: Ehe! Another challenge again? She has said her opinion?**

PO01: I have a question, you have said that you are going to implement this thing in =Siaya= and where?

**I: What is that? For now is all about getting the opinions. You know if research is being conducted, it cannot be conducted everywhere and after the research has been conducted it can be something that can be done… you see even the medicine we take, the research was done at a particular place, but now the medicine is used by all, right?**

PO01: Yes

**I: And now everybody is using it right?**

PO01: Yes

**I: So if Jitegemee will be implemented, I cannot tell where it will be implemented, okay? That is understandable?**

PO04: Yes, it can be anywhere

**I: Opinions, opinions, please we are finishing.**

PO04: You are not tired of standing?

**I: Me?**

PO04: Yes

**I: I am a teacher [laughs] … sitting will make it more difficult for me.**

PO07: A teacher does not sit

**I: Opinions, challenges please. My siblings, there are no challenges that will be there**

PO03: We have said it all.

**I: Okay, it is just that one from her?**

PO03: Yes

**I: And what amount can they be ready to save in a week without interfering with their daily needs? How much can sex workers save in a week without interfering with their daily needs? [Silence] how much? How much do you think female sex workers can save per week? If they do not interfere with their basic needs?**

PO02: Those are people that do this our job?

**I: Yes, the female sex workers**

PO02: Weekly

**I: Yes**

PO05: Five hundred shillings

**I: Five hundred per week, ehe number 3 what is your opinion?**

PO05: Five hundred shillings

**I: Five hundred shillings?**

PO05: Yes

**I: They can save without interfering with their daily needs?**

PO06: Three hundred shillings

**I: Three hundred shillings per week?**

PO06: Yes

**I: Number four what is your opinion?**

PO04: Just that five hundred

**I: Five hundred shillings?**

PO04: Yes

**I: And number 7?**

PO07: Five hundred shillings

**I: Five hundred shillings?**

PO07: Yes

**I: Okay. How can these savings be kept such that the female sex workers can trust its safety? How can these savings be done such that the female sex workers can believe their money is safe?**

PO03: In a bank, that is when someone can know that her money is safe

**I: If it is kept in a bank**

PO03: Yes

**I: Number 7**

PO07: If Jitegemee has been implemented I think it can be safe because they are the ones that educate us.

**I: If Jitegemee?**

PO07: It can be safe if jitegemee can help us to save? Jitegemee savings

**I: Mmh, if it can help? Another opinion? How can this money be kept safe such that the female sex workers can believe that their money is safe?**

PO07: If it’s something like a bank

**I: If it has been kept in a bank?**

PO07: Yes

**I: Okay, is there any other opinion?**

PO04: None [papers chuckling]

**I: So I am asking like this, do the female sex workers think of the moment they can retire from sex work? [Participants laugh]. You people have laughed at me or that question [laughter].**

PO04: Teacher, we are tired there is no one who is understanding anything here.

**I: No, number four talk, what is it?**

PO04: Responding is not easy. I am not understanding it completely because if I look at my people nobody is understanding anything. So they are looking at me to speak. Now I have spoken.

**I: Chairperson has spoken? [Laughs]**

PO04: Yes

**I: The chairperson has spoken but I would like you people to answer me on this. Do female sex workers think of when they can retire from sex work?**

PO03: You cannot sleep thinking on how you will retire from something because money is sweet every day, so for me I do not think those women always think of retirements. People are different so maybe others do think of that but for me I do not think I can retire soon.

**I: Mmh, you do not think?**

PO03: Yes

I: Mmh! Number two, give me your opinion, say something you also know these things. Is it something that female sex workers can think that it has reached a moment that I need to retire?

PO02: I don’t think so because I still do not have capital, so that is my business.

**I: Another opinion again?**

PO01: That is the job, so I will not know when to retire.

**I: Mmh**

PO06: What I know I only retire immediately after giving birth [participant laughs]

**I: Other female sex workers, do you think they think of retiring from that job.**

PO05: No, I cannot think like that because if that is like everything I depended on. You have heard that some people can only retire after they have stopped their monthly period? If they have come back from leave

**I: You have said that they do not mention that**

PO05: Yes

**I: Is there a way you people can sit down as female sex workers, sit down and have such discussions, is there a time the female sex workers can sit down and have such discussions?**

PO01: Another person again? (Laughs) that of quitting she has ever heard, another person again

PO05: Even me I have never heard them talk about quitting it, we will just beat around the bush.

PO06: Sometimes I can have a thought of quitting this job because I have gone to that work yesterday and met with something that is beyond my capability. (Laughs)

**I: What is that thing that is beyond your capability?**

PO06: *Omuiri*… I have met with unusual thing

**I: (Laughs) what is *Omuiri*?**

PO06: The big snake (penis) that is beyond my capability.

**I: Can you meet with a snake there?**

PO06: This one is always a snake… that is his penis (participants laughs) so I can discuss with my fellow that I am tired of this job, what I met with yesterday I just need a break

**I: Ehe (laughs) another person, is there any discussion for female sex workers**

R: Yes

**I: Like which ones**

PO04: Just the way that job was yesterday. Maybe you want to game and I was here in =kodiaga=, so how was your journey, eeh! My sister yesterdays’ job, I am even tired with this our job. I found a very difficult job or sometimes your fellow tells you that yesterday I did not even get anything I got a client that has nothing for work so I came back empty handed, so I have decided to look how it will be today. I have received money and I have also not done my job. You have not felt nice and you have not also received money. (Participants laugh)

**I: Number 4 is saying that you have not felt nice and you have not also received money**

PO04: So you came back without success, there is no money and there is no pleasure (laughs)

**I: Another person again? So you are saying that there is no discuss of when will you retire? What makes it difficult for people to discuss about that?**

PO01: Because we cannot think of resigning from a job how will we support ourselves?

PO05: What will we feed on if we resign? That is why we cannot resign.

**I: Thank you, number 2 has still refused to speak**

PO02: The message, that message, she is getting late

**I: Normally, why do female sex workers quit or want to quit the job? For those that want to quit, she has mentioned one. Is there someone that can add? We were asking normally why female sex workers want to quit sex work. She had already mentioned one.**

PO03: The challenge of such bad things is what will make some people to think of quitting

**I: Mmh, if you say bad things, what are the bad things**

PO03: Bad things are like maybe you have met a client that have an expensive car and he t6ells you that he does not want to take you to a lounge, he wants you to go to his house

**I: He wants to take to his…**

PO03: He wants you to go to his house, to his house and then you will go because he wants to pay you well, so you will just leave with him. So the moment you go with him, there is a different plan he has for you. Maybe there is something bad he wants to do to you or there is something of his that is wrong. So if you get there, instead of engaging in sex, *stop laughing*, so maybe he has put you there with his pet, so this pet is the reason why he paid you or even his dog or another different pet. So this is the thing you are put work with [to have sex with]. So you will just do it because maybe a gun has been pointed at you and if you do not do it, you are killed. So you will be forced to do it because you fear for your life. But after that you will go and look for God [salvation] and quit that job.

**I: You have understood that right? Number two I have seen you laughing, there is something that has touched you there, it seems like you want to say something, why do female sex workers quit this job or want to quit this job? What is it? What you have heard other people saying even if it is not you personally**

PO02: Of the people I know, I have not heard any of them saying that they want to quit. Maybe just to have a break.

**I: Then what can make someone to have a break?**

PO04: If the wife to that clients finds you and goes for you to those people

**I: If you say goes for you to those people, what do you mean?**

PO04: That African chemistry (witchcraft) that we had mentioned and then bewitch you with a disease that you cannot cure and our income is always low, you will decide to quit.

**I: Mmh, another opinion, what can make you quit this job? That makes you say aah, I want to quit. It does not have to be your personal experience.**

PO07: What I can say is that maybe you are tired of it and you want to run back to God because our creator is greater than the things of the world

**I: Thank you. Is there any other opinion? Okay, that was what we think can make others quit this job. Any idea why you think this is the reason why I can quit this job. Personally and it should be our secret the way we had said, what can make you personally quit?**

PO07: If I have saved and feel that I am stable with these my children then I can just quit and continue with my business.

**I: Thank you**

PO04: If I have fulfilled my target, for instance I wanted to do this thing to purchase somewhere to stay with my children.

**I: Mmh, just like that, let us just mention them, another person again (silence)**

PO03: I think quitting is difficult because even a teacher retires because the moment has reached for retirement has reached, she does not retire because she likes.

**I: Mmh**

PO01: You can quit and come back to it again (participant laughs)

**I: Tell me a bit, how does that happen?**

PO01: Because you can quit and start your business and then realize the income is low in your new job so you will say that let me go back.

**I: Thank you. Another opinion again, I am winding up, the female sex workers how old do they quit sex work? Age, which someone can…**

PO05: There is no issue there on age, it is up to you to know that you are tired and should quit. There is no age limits or even the way my fellow has mentioned, immediately you have stopped seeing your periods.

PO04: The moment you have reached your menopause

PO05: Yes, if your body is not heating up (you do not have the desire for sex)

**I: Another person, just those ones? But there is no quitting without a reason? (Participants laugh)**

PO05: Who should leave it for…

**I: Okay, is there any other reason that can make someone to quit sex work?**

PO05: No

**I: What do many female sex workers do after quitting sex work? What do they usually do when they quit sex work?**

PO07: Like a job?

**I: Yes, even like a job? What do they do? Just say it loudly**

PO07: They always do business, you can even start your business and the most important they look for salvation.

**I: Some of them get salvation?**

PO07: Yes

**I: They get Jesus**

PO07: Yes

**I: Thank you**

PO01: You can get lucky and find someone that will marry you. (Participant laughs)

**I: You can get lucky and get married, thank you.**

PO04: You are playing

**I: Number four, I heard you saying you are playing, can you elaborate for us a bit?**

PO04: I am just supporting that sweet statement (laughs)

**I: You have supported that someone can get a client that can marry her.**

PO04: Yes

**I: Ehe, number 6**

PO06: If I quit and can just relax because I was using that money to educate my children and they have succeed so they are giving me support when I am just seated and eating in the house

**I: If the money now, you have educated your children**

PO06: Yes, I just wanted to educate my children and God has helped them and they are supporting me while I am seated and just feeding I the house

**I: Mmh. Is there any opinion?**

R: You are not yet done?

**I: It is done, I have just remained with questions. I am through with these. Do you know instances where female sex workers has quit sex work and returned back to it latter? I have heard of an opinion**

PO04: That your opinion, number one

PO01: How?

**I: That is why we are saying she had mentioned, so another person. Do we know instances where someone quitted and returned? Apart from what she had said, is there another one that can make someone quit and return? (Silence) have we heard of such situations?**

PO03: If you quit a job, quit it because if you return you will meet with your death there. If you quit a job, quit.

**I: You have not heard of someone who left and has return?**

PO03: I have heard

**I: What makes people quit and return?**

PO04: Unless if she thought she has found a good one and then that person disappoints her not giving the support is gone and she just returns to that job again.

**I: Number three you are saying…**

PO03: She should go and prepare alcohol

**I: Ooh, to prepare alcohol.**

R: Yes (Participant murmuring)

**I: That is her opinion**

PO01: People do return

R: I have not seen

PO01: If you have not seen, let those people who have seen to speak

**I: What makes someone to return? What makes people to return? Give me an opinion, I am winding up**

PO04: I am loyal, I do not have an opinion

I: You have never heard that someone has quitted and returned

PO04: No, there is no energy, now I am not thinking anymore, I have reached the wall [she is exhausted]

**I: Sorry, now…**

PO04: Cut the remaining part?

**I: Yes, I am cutting it, and have said that the reason why they return, any negative things that happens to them, those that has quitted and returned. The negative thing that happens to them. Do we know any negative thing that happens to those that quitted and returned to sex work?**

PO01: You left and came back

**I: Any negative thing that might happen**

PO01: Nothing bad can happen to me because I have been doing that job.

**I: That number 1, another person, another opinion, what negative things can happen to those who quitted and returned?**

R: Those who returned please talk, there is no energy

**I: Yeah, there is no energy, Eiya! Sorry. Number 6, what things would most female sex workers like to accomplish before they leave sex work? What would they like to do before, for those that would want to quit, generally, what would they like to do before they quit this job?**

PO07: You would like to buy my piece of land.

**I: Something like a plot?**

PO07: To buy something like a plot or a house before you quit.

**I: Another person that is her opinion, another person.**

PO06: If you have children, you would like to give your children proper education.

**I: Educating children before quitting, another person again. I have remained with only one question, please just give me** opinions

PO06: I would like my family to have a good life.

**I: If you say they have a good life, what does it mean?**

PO06: To have a better life.

**I: So you would like to secure them before you quit this job. What can show you that now you have secured them very well that you can quit this job?**

PO06: Sometimes they are dead, they do not exist anymore (participants laughs)

**I: Securing them well**

PO03: I think giving them a better life. Maybe their house was in bad shape, so she has built them, some houses.

**I: Constructing for your parent a house?**

PO03: Yes

**I: Is there anything else? Number 2 please just give me an opinion. Please provide, okay. Do you recognize those who quitted sex work for a period of the last five to ten years? Are there those that we know? Number one has said no (participants’ laughs)**

R: Those that quitted?

**I: Yes**

PO07: I know them, people like [mentioned names]

**I: People like [mentioned names]? (Laughs)**

PO07: Yes, people from Mombasa (Laughs)

**I: Apart from [mentioned names]**

PO04: The ones I recognize got married, so even if she is still doing it, now that is what I am not aware

**I: She quitted and got married, so you do not know if she is still going on with it?**

PO04: Yes

**I: Another person? Is there any person we know?**

PO03: No

**I: What makes it easier for someone to quit this job? What makes it easier for someone to quit?**

PO05: For someone to quit sex work?

**I: Yes**

PO07: Maybe if she has saved and make something and have a good account

**I: Ehe! Another person again, what makes it easy for someone to quit sex work?**

PO06: What my fellow had mentioned that you have found someone and he took you to his house to have sex with his snake that makes it easy for you to quit sex work

**I: Number 7, you wanted to say something? Thank you. Are there any challenges they go through? Those that quitted (silence) are there challenges they face?**

PO07: No, those that quitted. Those are the ones I know of?

**I: Those that has quitted, are the people that know (laughs) okay. Otherwise thank you so much. That is like end of our discussion today. Thank you so much for giving us your time. We are done with our todays’ discussion and the time is 1533 HOURS.**

**END OF INTERVIEW**
