# Supplementary material for: Eat a little and save a little: A qualitative exploration of acceptability of a potential savings intervention to reduce HIV risk among female sex workers in Western Kenya
Source: PLoS One. 2024 Dec 19;19(12):e0310540. doi: 10.1371/journal.pone.0310540 (PMC11658496; doi:10.1371/journal.pone.0310540)
Supplement: S1 File — (ZIP) [file pone.0310540.s001.zip › Jitegemee Transcripts and Dissemination Notes for Journal/FGD P.docx]

**FACILITATOR NAME: LILLIAN AKOTH**

**NOTE TAKERS NAME: MORAA ARASA**

**FGD ID: FGD P**

**TIME: 1153 HOURS**

**DATE: 04/MAY/2022**

**TRANSCRIBERS NAME: OMBEWA DEPHINE ANYANGO**

**CATEGORY: BELOW 30 YEARS, RURAL.**

**I: Welcome, welcome once again, as I have said my name is [Name of moderator], I will be leading the session, my colleague here is called [Name of note taker] and she will be helping us in note taking. So this is FGD P, it’s done at =Kamito=, in this FGD we have nine (9) participants and the starting time is 1153 hours. Ok fine so we are going to start, now I am starting so let us relax there is nothing difficult. This not an examination that is going to make worry that you will be going to which marks okay! (Yes at the background) So my first question is, I have read a certain part according to jitegemee, following to what jitegemee is planning to do. So according to what I had explained to you about jitegemee in a short statement, what has come to your mind about jitegemee now according to what we have read. What comes to your thoughts (chicken clucks) Number 8 it’s like you have an opinion?**

PP08: Should I stand up?

**I: No relax**

PP08: Thank you (clearing throat) according what you have said, what come to my mind is that what you have read is good (chicken clucks) and according to this research we can do savings and loans.

**I: Just tell us something on Savings and…? Just say it aloud.**

PP08: We can do savings and loans isn’t it?

**I: You are saying savings and loans, as for me for me I want to hear your thoughts.**

PP08: (laughing at the background) you want my thought, my views are (Respondents laughing at the background)

**I: According to what you have read what comes to your mind?**

PP08: What come to my mind is that, it’s a good thing

**I: Mmmm, why are you saying that it’s a good thing**

PP08: The reason why am saying that it’s a good thing, you can learn how you can get money, you can do your small business even though you cannot do a small business maybe doing business is difficult. And if your husband died (pass away) you can go to outside countries like Angola” (laughing at the background) I think you know where Angola is (laughing at the background)

**I: People may be knowing it but as for me I don’t know, may you please tell me.**

PP08: The outside countries are many (inaudible…) we can go there, I have forgotten the name but its beautiful have you there?

**I: Am not aware, tell me**

PP08: It’s along (respondents laughing) stop laughing, it’s along =Reru=, it’s very beautiful, so I have visited there.

PP01: Hide out (respondents laughing)

PP08: yes it’s that one you got it

**I: Number 1 what have you said?**

PP01: Hide out hotel, I think some of her clients (male clients) are the ones who are there

**I: Oooooh**

PP08: So it’s there.

**I: So you said we can go there at outside countries and also hide out hotel so that what can we do**

PP08: So that I can find how I can wash my legs and feed well

**I: Okay, so that was her thought, according to what you have read about jitegemee what comes to your mind number 1?**

PP01: Me as respondent 1 I see jitegemee as a good thing because here in the field, it can reach a time where your clitorises gets tired (laughing at the background)

**I: What is getting tired, just tell us**

PP01: There might reach a time where you go for sex work and you get tired, your virgin gets tired, so you just sit there and say my virgin today is tired from sex and I don’t want to go to sex work again. If there is a group that you are in that you can get five hundred shillings or two hundred , three hundred shillings and you don’t use the all amount, so one hundred shillings according to jitegemee its going to help me tomorrow as I will retire from sex work because the years are also moving I will not be able to do “Dog style” when l turn 45 years it will force me to stop sex work because people always retire, since my body can set all the styles so there will reach a time when I will stop sex work and say I am tired of it, and I have grand children to sex work for. So jitegemee it will reach a time where it will help feed and will not be forced to concentrate on sex work field.

**I: Thank you anybody else, number 4, you want to tell us something?**

PP04: What I was trying to say as number 4, here in sex work we have two different ways. Where if one side is difficult for you another one. Truly in sex work you can get tired where you can go with Onyango, you can find Otieno is different from Ouma, Onyango is different from Otieno so they are very different. You will find that the size of the penis of this person is different from another person. Maybe the one from that person is longer than this person. So you will say that today I have gone but I found that there is a different, let me change, they always change, you can take a break to go and rest so that you can depend on yours and not another person.

**I: Thank you so much, anybody else, respondent put the numbers at a place where I can see them clearly. I want to see the numbers (respondents laugh at the background) stop hiding the number, I want to see your number. Number five (respondent laughs) what do you think about jitegemee, what comes to your mind number five still thinking number 3**

PP03 Me as number 3 I think jitegemee can help when you are doing sex work get your money (respondent coughs at the background) you can take a little to keep and the rest you can use them on your needs. Maybe sometimes when you have saved them a time will reach where you don’t have a customers, you will find yourself using the money that you saved.

**I: Thank you, mmh, anybody else, yes number nine**

PP09: Me as number nine, I can say that saving looks like something good, sometimes you have gone with somebody and you are given/paid 500 shillings you have to divide, use a little and save the remaining because you don’t know and we always don’t receive phone calls, you can become sick and you can’t go for ‘sex work’ field and the money you had saved can use a little when you are going to the hospital, that’s my thought.

**I: Thank you, is there any other person who wants to add on what she has just said, number 6**

PP06: Me as number 6, I can say that jitegemee is good because in sex work you won’t go and meet your clients when you are feeling sick, so if you saved some money, you can rest, the money you saved can support you, what is the rest thinking.

**I: Thank you, so we are going to another question, the question is, what do women who exchange sex for money buy with their money ones they have money, what do you buy, what must you buy daily**?

PP01: I must buy inner pants

**I: Do you buy pants on a daily basis?**

PP01: Ooh daily basis

**I: Let’s start with daily basis, the ones that we buy daily**

PP01: I must have condom in the house, because a times I might be having one condom in the house, I won’t depend on my partner to carry condom, some always come to have unprotected sex and maybe you don’t wish to have HIV/AIDS. You will say NO to sex without condom, you. You must protect yourself with condom without failure. So I must have a condom. Maybe if the ones we were given got finished then it will force me to buy some because am a sex worker, I work daily.

**I: So you buy condom on a daily basis, how much do you spend on condom on a daily basis**

PP01: So maybe let’s say today I have come to buy condom, a times I can buy it three times a week, it’s just like I bought it on a daily basis, Condom is one hundred and fifty shillings and in a day I must buy condom

**I: In a day you can use fifty shillings?**

PP01: For condom

**I: Okay, number 2, tell me something, how you see it, what can we buy with the money that get daily**

PP02: (Cleaning throat) me as number two

PP01: She is still thinking

**I: Number four**

PP04: Me as number four, I think the biggest thing here is food because when you want to go and visit your client, you must buy food to eat, you can’t go for sex work when you are hungry, sometime you can go there and you feel conscious, so I must eat healthy food since I know am going for war (sex) only that

**I: You are going for war (sex)**

PP04: I am going for sex worker (war)

**I: Thank you, food isn’t it so how mu h do you spend for food on daily basis**

PP04: Like 500 shillings, because it’s going in my stomach

**I: From morning to evening, five hundred shillings? Number nine you wanted to say something**

PP09: I wanted to say what number four has talked about, you must buy food t remain healthy because you can find some style which can’t handle when don’t eat properly ,so you must eat and stay healthy to gain more strength

**I: Mmmmh**

PP09: Yes

**I: Thank you, as or you when you budget for food, how much can you spend for food on a daily basis**

PP09: It is around five hundred to nine hundred shillings only

**I: Around nine hundred to five hundred shillings, respondent eight you wanted to say something**

PP08: Me as number eight food is important in human life, so you must eat well, because sometimes you can go to do sex work and finds client with big penis, you know there are different penis some are circumcise and some are not circumcised, so you find the ones which are circumcised will make bend and since I didn’t eat, I won’t be feeling well after sex and when am eating healthy even if am given eight hundred shillings and have eaten healthy, so when it comes to doing “chicken style” I will do t well because am satisfied (full)

**I: So, how much can you spend on food daily?**

PP08: I can use seven hundred shillings” chicken style” because you work hard to carry your legs up.

**I: Yes**

PP08: You have worked, having sex the whole night, working hard to get back the seven hundred you worked for

**I: Ooooh**

PP08: So, do you also buy condoms when you are going to work?

**I: Apart from food, do you also buy condoms**

PP08: Yes, also buy food

**I: SO how much can you spend to buy condom daily?**

PP08 I will just buy one packet and keep some for my stock, so when am on duty I will have enough until morning, since we had 3 rounds.

**I: Mmmh**

: The client must also buy soda, I can’t open my legs and he can’t afford to buy soda, so he must buy sodas and I make sure his underpants is pure white, the client should know that am not dump. So you should not go with clients whose inner pants are tone (has hole) you won’t get money from such client

**I: (Laughing) yes**

PP08: Your private part should remain clean, since its sweet and don’t want to have bad smile to my clients

**I: That is what number eight has said, PP07 tell us what do you buy with your money on a daily basis**

PP07: I want support those who have talked about food

**I: Mmmmh**

PP07: Because food brings strength to handle your clients

**I: Mmmh**

PP07: Mmmh

**I: So you think you send how much on buying food on daily basis**

PP07: I can spend six hundred shillings

**I: Six hundred shillings?**

PP07: Mmmh

**I: Number 5.3 it’s like you have something you want to say, number 3 has nothing, respondent 5**

PP05: Me as number 5, I also support my friends

**I: Mmmh**

PP05: Just as they have said, food is important

**I: Mmmh**

PP08: When you are hungry you cannot go and meet your clients

**I: Mmmh**

PP05: Yes, that thing is very difficult

**I: Thank you, so how much do think you can spend in buying food daily?**

PP05: You can spend eight hundred shillings in a day (silence, people murmuring at the background)

**I: I don’t want people to know our secrets, I didn’t know was sitting around there,(respondents laughing)Okay, so aah I was asking, was that for daily basis and what about every week, what must we buy every week, lets me start with number 6, on weekly basis**

PP06: Me as number 6, I think that food is the first priority

**I: Mmmh**

PP06: Because you must eat food

**I: Mmmh**

PP06: Mmmh

**I: So how much can you spend on food on a weekly basis?**

PP06: You can spend a lot on a weekly basis

**I: Like how much? (Silence)**

PP06: I can spend five hundred shilling on a day, on daily basis

**I: five hundred shillings on a day**

PP06: Yes

**I: So we divide it into a week:**

PP06: Yes

**I: So the mount is three thousand five hundred shillings?**

PP06: Yes

**I: Okay, number 6 have said that, is there any other person PP02?**

PP02: As for me, food is obvious, that we must eat, people are just speaking but I have not heard about soap, I do they take bath with water without soap, when they are there, (respondent laughing)

**I: We were just waiting for you to say it (respondent laughing)**

PP02: Have not heard you taking about soap because as for me, I must take bath, apply oil, I must apply perfume that’s when I go to work my fellow friends, I smell nice so that my client can’t see another person

**I: Mmmh**

PP02: The client will just say that he is satisfied with me

**I: Mmmh, so you have said soap, how much can you spend on a soap in a week?**

PP02: Soap

**I: Mmmh**

PP02: In a week, in a week when I take (silence) flamingo, I only use one hundred and fifty shillings

**I: YES**

PP02: It’s going to help me for the whole week

**I: Mmh, so in a week you spend one hundred and fifty shillings to buy soap**

PP02: Just for taking bath

**I: Number four you wanted to say something, how much money can you spend in the things we must buy in a week, a week can’t end without you buying them.**

PP04: What we are using or what we buy

**I: That ones that you must buy and you are going to use**

PP04: We have already said that food you know it is better to be satisfied and not untidy, but you are satisfied

**I: Mmmh**

PP04: Yes you cannot be tidy and hungry, food comes first

**I: Thank you so much, is there anyone who wants to add something on weekly basis, number 8**

PP08: Honestly I can’t be feeling full while my virgin is smelling

**I: Mmmh**

PP08: I must buy bathing and washing soap

**I: Mmmh**

R: That’s not true

PP08: I must also apply nail polish

**I: Mmmh**

PP08: But there is no number

**I: Mmmh**

R: You are destroying yourself (inaudible content)

PP08: But you are applying makeup so you must also have a soap

**I: Yes**

PP08: You use soap to wash cloths, for bathing its different and also for washing is also different.so even when am full and I know am going for sex work

**I: Mmmh**

PP08: I am now smelling nicely and I believe am going to full

**I: So you think a person can spend how much to buy soap?**

PP08: Soap

**I: Mmmh**

PP08: I can buy soap for one hundred and fifty shillings and soap for my laundry I always a half of it.

I: Mmmh

PP08: It’s going to help me for the whole week

**I: Okay that is for soap, how much can a half of bar soap cost?**

PP08: A half of bar soap cost a hundred and eighty shillings now.

PP02: Its eighty shillings

PP08: One hundred and eighty since you have not added washing powder and the ones with good aroma

PP02: Downy [Brand of washing liquids]

PP08: Yes you have not included it

**I: Mmmh, so you can use one hundred and eighty on soap every week**

PP08: Yes, when you are going for sex work you look beautiful, when you meet your clients, they will actually say that you look beautiful

**I: Anybody else who wants to add something on what you spend on a weekly basis, Okay let’s forget about weekly basis and what about monthly what are the things that we must spend on monthly basis, number four what are the things**?

PP04: Me as number four, I can say girl child has a lot of budget

**I: Mmmh**

PP04:When girl child goes out for shopping, she will always shop for her clothes, body lotions, also she shops for braid to plate her hair, so when she goes for sex work she will always looks charming and beautiful and smell nice

**I: Mmmh**

PP04: Mmmh

**I: So how much can you spend every month/**

PP04: At most you can spend like seven thousand shillings

**I: so you can spend seven thousand shillings in a month?**

PP04: Its depends on all your needs and budget

**I: Any other suggestions, number five?**

PP05: Me as number five, girl child attends her menstruation periods every month

**I: Yes**

PP05: So we also must buy sanitary towel

**I: Approximately how much can you spend?**

PP01: fifty shillings

**I: Number one have said fifty shillings, PP05 do you have another suggestion?**

PP05: One hundred shillings

**I: You spend one hundred shilling on a monthly basis?**

PP05: Yes

**I: Is there any other person who has something to add, how much can we spend every month which you have noted down and always happens, what are they?**

PP01: Me as number one, am a sex worker and here at sex work

**I: Mmmh**

PP01: I think for the clients to admire you, always you must change your hair style, braid my hair every month, and you know this away makes clients to gets confused. If you don’t look clean you won’t be able to get clients, and that’s when you will only request for twenty shillings to take home and feed your children

**I: Mmmh**

**PP01: According to me beautify yourself, changing your hair styles even if it means to shaving your hair, shave it, braid your hair every month, I change my hair style every month.**

**I: Mmmh**

PP01: So that when am going to do sex work when I look decent

PP01: For me to braid my hair it’s around one thousand five hundred only

**I: One thousand five hundred shillings?**

RI: Yes

**I: Every month, mmh, any other idea number 6**

PP06: Me as number six am supporting number five, we must buy sanitary towel/pads, when the month starts and ends

**I: Mmmh, how much does that cost?**

PP06: One hundred shillings monthly

**I: One hundred shillings for sanitary towels, is there any other idea that we must come up with every month (silence) number two, number eight?**

PP08: Every month I must change and buy clothes

**I: You must buy clothes every month?**

PP08: The clothes am putting when am to going meet my clients

**I: Mmmh**

PP08: You know it’s not one client we also meet, we meet different clients and we must put on different clothes

**I: Mmmh**

PP08: So I must buy more clothes

**I: Mmmh**

PP08: We call this high class

R: Which is not a good journey

PP08: I also put on high hill shoes, I look high on them so I must put them on (noise on the backgrou**nd)**

**I: Sorry let us continue, let’s start from where we had stopped, okay. We have discussed and talked about how spend on every month, weekly and daily basis, so what must we buy and spend every year( bird chipping) number four have you carried your hands up?**

PP04: I am still thinking

**I: When you are still thinking, number three**

PP03: Everything must be closed every year

**I: Mmmh**

PP03: Maybe your clothes, inner pants, trousers that’s fits you have become old and torn

**I: Mmmh**

PP03: You know you must apply make ups in order to always look smart

**I: Mmmh so here on make ups, how much can you spend every month?**

PP03: IN a year (silence) you know make ups are very expensive, we buy them like every time and it can approx. to ten thousand shillings

**I: Mmmh, you have also talked about clothes. How much can you spend on clothes every year?**

PP03: We have different type of fashions for clothes, when a new fashion comes it comes with different prices, some prices are high some are on the lower side

PP03: So in a year clothes can take like twenty thousand shillings, sometimes you can find girls spending three thousand or five thousands on shoes and clothes

**I: Mmmh**

PP03: That is on one clothe only

**I: Mmmh, have heard you even spend twenty thousand shillings, number nine**

PP09: I don’t know I start from where, I don’t know if I should start from backwards

**I: Just talk**

PP09: I think there are some of us who have children and should also buy for them clothes and look nice

**I: Mmmh**

PP04: We must also take our children to school

**I: Mmmh**

PP09: So you know we must keep our children clean and maintain them

**I: Mmmh**

PP09: So even if you are leaving you house to work, people may not talk about how you look smart and your children looks untidy

**I: So on daily, weekly, monthly basis and every year, you spend how much on your children?**

PP09: It’s on a monthly basis and a year, when you are doing your shopping, you must also shop for children

**I: So how much can you spend on a child?**

PP09: I can spend like five thousand shillings, since you are buying beautiful clothes and shoes for your child. So this is for each child the whole amount will be ten thousand shillings since you are buying beautiful clothes

**I: Any other ideas, number seven**

PP09: I want to add on what number nine have just said about children, you must shop for them clothes to look beautiful, pay for their school fees if a tall they go to school and how they feed with this your job of doing sex work

**I: Mmmh how much can spend on school fees, isn’t monthly or yearly:**

PP07: It’s termly, you can pay like two thousand seven hundred shillings per term

**I: Mmmh**

PP07: Mmmh, so there

**I: Number one you wanted to say something**

RI: I was saying that in terms of fees and again I can do sex but child does not like this sex work life

**I: Mmmh**

PP01: I am not saying that sex workers are not educated but I want one day he may learn, go to school, get educated, I will be forced to pay per term, others pay per month and others pay per term

**I: Mmmh**

PP01: Sometime you pay monthly fee, you can calculate it per term if the child is in secondary school, and there is particular amount to be paid

**I: Mmmh so you calculate this for a month**

PP01: Yeah

**I: Okay, is there any other person who have an idea, different opinion, we want to move on before the noise from generator start again .PP08 do you have any opinion?**

PP08: Relax a little bit

**I: Relax a little, okay what can we buy like mmh we had said in one year, okay where do women who exchange sex for money to earn their living and upkeep from? Where do you get the money from, number eight?**

PP08: What have you asked?

**I: Where do most women exchange sex for money get their upkeep?**

PP01: Let me be alert, let me sit comfortable

**I: Where does those women who exchange sex from money, get their money for upkeep?**

PP08: Most women who exchange sex for money

PP02: Prostitute or sex workers

**I: Mmmh**

PP08: Meaning where do they get their upkeep?

**I: Mmmh**

PP08: So should I answer you?

**I: Yes**

PP08: Where I get money something like that?

**I: Mmmh**

PP08: If I go for sex work and I also run my business, isn’t it?

**I: Mmmh**

**PP08: When I get money when visiting clients and should be ten clients**

**I: Mmmh**

**PP08: Out of this ten clients, am actually looking for the one who pays well**

**I: Mmmh**

**PP08: The three days I will make a lot of money, I will give a kiss**

**I: Mmmh**

**PP08: If clients pays good money, I will continue and my children will go to school, so women who do sex wok like us**

**I: Mmmh**

PP08: Money is there

**I: Thank you, number one**

PP02: Me as number one am a business woman a sex worker too

**I: Mmmh**

PP02: Sometimes I break from doing business and go back to it when I want to do sells

**I: Mmmh**

PP02: When I receive phone call

**I: Mmmh**

PP02: Anytime I can receive a phone call, calling baby where are you, I will be forced to leave my business and go and meet him.

**I: Mmmh**

PP02: Me the business that am doing is sex work

**I: Mmmh**

PP02: That’s the business am doing and when time come to leave, let’s say a day I will know that I had sex with the four clients and I will find how I will help myself. So when I want to settle down and start business, then that business is going to fail to me.

**I: Mmmh**

PP02: Sex work is the only business am going to do

**I: Mmmh**

PP02: I recognize myself as a sex worker

**I: Mmmh**

PP02: Yes, am going to do sex work

**I: So as for you the business that you are doing is sex work**

PP02: Yes, sex work

**I: I want to go back to PP08, you have just said that a person might be having a business and client. So in business and sex work, which one is the biggest business for women?**

PP08: Business, I mean sex work is the business that help sex workers

**I: Mmmh, so which one is the best way for those women who are exchanging sex for money always to get money often.**

PP08:You when you have decided to do sex work, I do it with my heart, When you happen to go for work and I find a client from Nairobi who is going to give me ten shillings, so I can venture back to my business

**I: Mmmh**

PP08: I have never be paid five hundred with client, so sex work business is better than small business

**I: Mmmh thank you, number one**

PP01: Me as number 1, what I can say is that sex work is the business what am doing

**I: Mmmh**

PP01:I am doing sardines business, selling sardines usually makes you smell terrible, So for how long do I have to smell sardines, because a client may call you and that time you are still sun drying sardines, so for me what brings me money is sex work.

**I: Mmmh**

PP01: Sex work gives me money, it can even make me fly to America (respondents laughing at the background)

**I: Mmmh thank you, how much can that give you?**

PP01: I can’t say the amount, just what God will bless with, maybe a client comes and offer only five hundred shillings for one day and he tell you, baby today let’s just eat fried meat and drink alcohol

**I: Mmmh**

PP01: And after taking alcohol, we retire to bed then after having sex my clients tells me baby I want to write you a cheque, that cheque I will not misuse the money.

**I: Mmmh**

PP01: I am going to start a boutique business and employee someone to help me run it, using the cheque

**I: Mmmh**

PP01: So for me sex work will continue supporting me

**I: Mmmh**

PP01: Sex work is good

**I: Thank you, number two you wanted to say something, sorry number four**

PP04: Me as number four

**I: Mmmh**

PP04: There are loses in business and I can do two type of business

**I: Mmmh**

PP04: A small business and sex workmen work brings out a large number of clients and small business is something that can fails you

**I: Mmmh**

PP04: Sex work cant fails you its permanent like appetite, so it’s a pleasure to be a sex worker

**I: Pleasure?**

PP04: Yes, it’s a place where you see people going, it’s just hitting the table by the sword in the world and you do it to your satisfactory

**I: Mmmh**

PP04: When am doing small business and happen to find client, I will leave my business and go for sex work when am looking beautiful, sex work is the business that is out there. There is no woman that is not doing sex work, every woman does it, and that’s why am saying that sex work can make you rich’

**I: How much can that give you?**

PP04: Business?

**I: Mmmh**

PP04: When have gone to meet my client, I know very well that am going for business, even if it’s bad, you know so well how you are playing your games. I can come out with something like ten thousand shillings, and that’s what I have gotten. So I will buy 1kg of maize, I will divide it for 30/ shillings, the 30 shillings will help me on which way if I sell it on kilograms. And you know with sex work ,you can make money, even my children are send for school fees, I can manage to pay for them five thousand school fees.

**I: Thank you, number five add can you add something (respondents laughing) where does women who exchange sex for money get their money mostly**

PP05: This other business is not working well

**I: What type of other business?**

PP05: Selling kales, is not working so when you are going for sex work to meet client, you must to dressed nicely

PP02: Me as number, those things are difficult you cannot know because at times I have gone to take some money at m-pesa

**I: Mmh**

PP02: When I take and give it to her will they know that I have gotten the money from m-pesa.it is difficult to know

**I: Mmh, women who save are which type of women and which type of girl are able to save.**

PP02: Girls who save

**I: What is their characteristics number 4?**

PP04: You cannot know the characteristic of a person who save and a person who do sex work. You cannot know a person characteristic

**I: Mmh**

PP04: There is difficult to know you can’t know

**I: Mmh, there is difficult to know**

PP04: At times she is doing sex work and saves, times she is not doing sex work and also saves

**I: Mmh so you can’t know the person who is saving and those who save don’t do them have a characteristic they are known with women who exchange there body for sex and they don’t save. They can make you know that this women is a sex worker and does not save money PP08**

PP08: Those who get money and don’t save, so that person eats soil

**I: Mmh**

PP08: It is soil because you cook and eat

R: You can know

R: Knowing you can know

**I: A behavior/characteristic that you can identify them with**

PP08: You cannot know because the little thing that, can you know a times you have your money and you have saved that is what I am trying to bring

**I: Mmh**

PP08:It is that am getting mine and eat it, this girl is getting and saving it, she has gone and buy a wall unit and you know me I want to eat mine I will go and spread rumors that, that person is a prostitute and as for me I am eating mine that person is above me

**I: Mmh**

PP08: Yes

**I: So you are saying those people who save buy something and keep them in the houses**

PP08: Yes

**I: Those who don’t save are gossipers they can be given sugar and sweets so that they should eat**

R: What should they be given?

PP08: Lollipop of five shillings

R: They should be given money to buy *madondo [a mixture of cooked chapatti and beans]* food

**I: Okay anybody else PP01**

PP01: I don’t have point

**I: you don’t have a point, who wants to give us another idea PP07, how do you see it people who don’t save**

PP07 :( silence)

**I: What characteristics do those who dot save here?**

PP07: They are borrowers,

**I: Anybody else mmh number**

PP03: Me as PP03 my fellow sex workers who don’t save, where a times it can reach a place where a place where a person is gossiping and they are just, have you seen how that person is conducting herself she is talking a lot there at the field

**I: Thanks you anybody else who can add on that**

PP08: They are those that are suffering

**I: That is PP08**

PP08: They are those that are suffering

**I: What do you mean by saying that they are suffering?**

**R**: They have an appetite

**I: They have an appetite?**

PP08: they like hand to mouth things, when she gets money and use it to buy food and ends up not saving it so she fill jealous to those who are saving(respondents laughing)

**I: Okay fine, women who exchange sex for money who save? What makes them to save? (Bird chirping)I know we have already said is there anyone who can add something? What makes those who save money to save PP08**

PP08: What can makes me to save?

**I: Mmmh**

PP08: I have a child, one day I will remember that there was a time I did a dog style and I build this story building

**R: Will you manage to go for a short call my fellow**

PP08: So one day when I will be tired with sex work you know this storey building will help my child. Maybe I have died, listen maybe I have died this storey building will help my child and even my grandchildren as for me I have already died

**I: Mmh**

PP08: I have built rentals houses, this rentals houses helps my children

**I: Mmh**

PP08: I have built a machine and it helps my children just from savings

**I: Mmh PP06, I see that what makes them so save, here to have sex work, there are some who have children. Mmh**

PP06: You must take your child to school

**I: Mmh**

PP08: So when you come from there you can save some and eat. So I think

**I: Mmh, you look at your child’s education isn’t it**

R: Mmh

**I: Okay for those who save, what make savings as an easy thing number 7, what makes these savings? What makes it easy for a parson to save number 5?**

PP05: Repeat the question

**I: What makes it easy for those who are saving so that they can save?**

PP02: What makes it easy?

**I: Mmh, what makes it easy for them to save**

PP05: It depends with your salary

**I: Mmh, you can elaborate more (silence)**

PP0I: I can help PP05

**I: That is number 1 mmh**

PP05: Here at salary, I can say that a person can get an easy way in saving, they get an easy way of saving according to their salary. A Pearson can get an easy way let’s say I have had a sex with a client and the client has given me enough money

**I: Mmh**

PP05: And when I am looking at it I have been given one hundred shilling and I have planned my budget and I find that this one hundred shilling will not reach the amount that I want to save.so what am try to say is that for you to save because you are doing sex work

**I: Mmh**

PP05: And also sex work has money, sex work bring money you can save same and use the remaining

**I: Mmh, so you are meaning that when you find enough money it makes it easy for you to save isn’t it**

PP01: Mmh

**I: So any other, what makes it easy PP09 for those who are saving, what makes it easy for them to save**

PP09: Me as PP09

**I: Mmh**

PP09: I think the easier way that can make me save money it is how you can get money

**I: Mmh**

PP09: Mmh

**I: You can elaborate for us a little**

PP09: So even if you have sex with a client, you can find how you can getmoney.so you will divide them, they one you can save and the one you can use. You don’t use all of it

**I: So the way you get your money is what makes it easy**

PP09: Mmh

**I: And when someone gets a little?**

PP09: When a person get a little you can divide even fifty shillings because even fifty shillings you can put in an m-pesa you don’t eat all of it, it’s like that

**I: Number 3, how do you see it, is there anything that you can add what can make it easy for a person to save to those who are saving**

PP03: Me as PP03, for those who are saving can make it easy because when you have started it, it brings a certain motivation that when you live it and a times you have planned what you want to do with that money after you have saved.

**I: Mmh**

PP03: So you know when you stop saving, what you had planned you will not do it and when you continue saving you will do what you had planned to do

**I: Any other thoughts number 2, what make it easy for a person to save, okay you don’t have any idea that can make a person to save and can make a person to save**

PP02: What makes it difficult for a person to save?

**I: Just say it loudly so that we can hear you**

PP02: Me as PP02, what makes it difficult for a person to save it depends with how you get your money also with your clients

**PP02: What will make it difficult for me to save, this two people one wants to give me one hundred shillings and the other wants to give me two hundred shillings so now I have three hundred shillings**

**I: Mmh**

PP02: This three hundred shillings I have children who need to feed

**I: Mmh**

PP02: How will I come up with a budget so that they can find how they can eat and for me also to save it is difficult?

**I: You need basic needs and how you can get money**

PP02: Yes it depends with how you get money

**I:Thaks you other thing, who else wants to add, what makes it difficult for a person, so that can make a person not to save what is it PP08**

PP08: What makes it difficult, it depends with money that you get and the budget, sometimes let’s say you get one thousand shillings or even five hundred shillings

**I: Mmh**

PP08: You take four hundred shillings, this four hundred shillings a times God gave you that one that you go to greet you and visits

**I: What does that mean?**

PP08: She has started monthly periods

**I: You go to greet and visit means**

PP08: It means am on monthly periods so you see here I have four hundred shillings want to eat

**I: Mmh**

PP08: So you know it is a little amount of money you cannot save and a times I live in a rental house and its end month, the land lord needs money

**I: Mmh**

PP08: And the client has given me a little money, I will leave it because of one hundred shilling it does not even wash my feelings the economy has also risen, and he had sex with me with just one hundred shillings.it is difficult you cant

**I: You can’t save**

PP08: You can’t save

**I: Number 1 add as something what makes it difficult, what difficulties can a Pearson have that makes them not save?**

PP01: Difficulties can be there , I am a sex worker and also I have a child who goes to school and maybe the money that am getting I take it to school fees it is not enough money to save. And also there are sickness that can make you use your money so you will force that times when you don’t save you will save it in hospital and also if you don’t save you will save it in school fee

**I: Mmh**

PP01 : Mmh that’s what I was saying

**I: Okay so meaning this difficulties how can we handle them so that this difficulties should not be there to hinder us save**

PP08: On how we can handle them.

**I: Mmmh**

PP08: How can we defeat them?

**I: That is PP08**

PP08: Is how you save money, five hundred shillings.

**I: Mmmh (birds chipping)**

PP08: It can help you.

**I: Mmmh.**

PP08: The story building that I had said, rent is there you have gone to take some, its end month you have gone to take some and client who gave me one hundred shillings I have left him (child cries at the background) I have to another one because they are not three even ten.

**I: Mmmh.**

PP08: I have gone for sex and the client has given two thousand shillings; I have gotten money.

**I: Mmmh**

PP08: The level of poverty has gone down and we are going higher.

**I: Mmmh so with savings?**

PP08: So, with savings we must put our thoughts their first. So, the things that brings money are many that is the hope that I have and also sex work.

**I: Anybody else, you said that needs, needs can make a person to have difficulties in saving, isn’t it?**

R: Mmmh

**I: How we handle it in terms of needs so that it cannot be difficult for a person (silence) PP09**

PP09: Me as PP09, repeat for me that question.

**I: Okay, I was saying for example I heard someone saying that needs that we have on daily basis and money that a person gets can make a person not save easily, isn’t it? So how can we handle it so that the needs should not be a difficulty that makes us not to save (silence, birds chipping?)**

PP09: Just a minute am answering.

**I: Anybody else who has an idea number 2, number 4 sorry (child crying at the background)**

PP04: It depends with the clients that we get, maybe you have two of them so you will be forced to add them to reach ten.

**I: Mmmh.**

PP04: So, these ten at times on daily basis when I go there, I have found four clients and have sex so when I come back.

**I: Mmmh.**

PP04: The economy has also risen because when I had first client even the client gives me five hundred shillings the other one gives me one thousand shillings another one just like that.

**I: Mmmh.**

PP04: So, then I put them together.

**I: Mmmh.**

PP04: I will see how I can help myself and a times I had two clients and economy has also risen so it can make it difficult for me to help myself, I will be forced to add them.

**I: Mmmh, okay.**

PP04: Mmmh

**I: Yes, tell me that bad things that can happen when a person is not saving (chicken cooks) PP02?**

PP02: Bad things that can happen when a person is not saving.

**I: Mmmh.**

PP02: Me as PP02 the bad thing that can happen when a person does not save, you might be in a problem at any time.

**I: Mmmh**

PP02: A times you get and eat, sickness comes, you will be forced how you will handle it.

**I: Mmmh**.

PP02: You will start struggling here and there a times the client that have got, he wants that when he has sex with me is when he can give me something.

**I: Mmmh**

PP02: Yes.

**I: Mmmh, any other idea, the bad thing that can happen when a person does not save, what is it PP08?**

PP08: The challenges that a person can have when they are not saving.

**I: Mmmh.**

PP08: As for me I don’t save, isn’t it?

**I: Mmmh.**

PP08: I am sick, I am bed ridden, I also don’t have money, you I cannot do anything.

**I: Mmmh.**

PP08: The child is sick; I don’t have a place where I can look for money so saving is good.

**I: Mmmh.**

PP08: When you don’t save you are suffering where you don’t have a place where you can rely on.

**I: Mmmh, where you don’t have a place, you can rely on when you don’t save isn’t it?**

PP02: You are suffering.

**I: Is there any other person who wants to add something there, which challenges can a person have when they don’t save (papers crackling) Okay, what good things are there when a person don’t save?**

R: (inaudible)

**I: The challenges are what I want number, what challenges are there when a person don’t save?**

PP06: Me as number, the challenges that are there you know you can be sick at any time and you don’t save, even the money that you can use to buy medicine you don’t have, you are sick that you can’t go for sex work so that you can get that money.

**I: Mmmh.**

**I: Is there anything good sorry bad that is there when a person doesn’t save?**

PP08: That is suffering.

**I: That is PP08.**

PP08: Yes, it is suffering.

**I: What do you mean by suffering?**

PP08: When I don’t save, I am sick and the child is sick where will I get the money to buy medicine?

**I: Mmmh**

PP08: When I take ten shillings and put it in the phone, I will tell you that please there is ten shillings in my phone go and withdraw it then buy medicine for me and when I don’t save where will I get money from, that is suffering.

**I: It is suffering.**

PP08: It is suffering.

**I: Mmmh PP07, what challenges are there when a person don’t save? (Chicken cocks)**

PP07: I am still thinking.

**I: Okay and what good things can happen when a person don’t save?**

PP07: I am still thinking.

**I: Yes (Chicken cocks) you also don’t have?**

PP07: Mmmh.

**I: Okay, fine is there anything else that a person wants to add? Let us continue,** **we should continue, okay I am asking (Phone rings, (Paper crackling). Okay where do women who exchange sex for money get confidence always save? Where do they save?**

PP08: Where they save?

**I: Mmmh (Paper crackling) number 04.**

PP04: Where we save after she has left there.

**I: What do you mean by there?**

R: She has come from sex.

PP08: After I have come from sex work, you carry a phone.

**I: A phone mmmh.**

PP08: I will take the money and put on the phone.

**I: Okay.**

PP08: Yes.

**I: PP09 you wanted to say something.**

PP09: I wanted to say that.

**I: You wanted to say that, about phone.**

PP09: Mmmh

**I: Another idea apart from phone, okay when we a phone we have Mshwari and others so it is which one here on phone.**

PP07: Mshwari.

PP09: Mpesa.

**I: PP07 you said something.**

PP07: Mshwari.

**I: Mmmh where else do people save.**

PP08: Even KCB are many

**I: KCB**

PP08: KBC or KCB they are many (respondents laughing)

R: Don’t be at a place where women have stand (respondent laughing)

PP02: And when someone say people laugh surely.

**I: Number 03, where do women who exchange sex for money save?**

PP03: Me as number 03 when you have come from sex work and you have found money when a client has given you. You can go to Mpesa and put it even in locked saving account so you lock it there.

**I: Number 05, give us your thought there apart from phone Mpesa and Mshwari where else do people can also save, we were told KBC but it is KBC**.

PP05: Me as number 05, you can also enter into merry go round so when you come from there and times you have entered a merry go round of five hundred a daily basis even two hundred shillings per day. So when you come from there you put it there when you pay day. So, when you come from there you put it there when you left with even three thousand shillings so when you pay for a week.

**I: Mmmh, thank you is there any other idea on where you can save either Mpesa, Mshwari, locked account bank we have marry-go-round, PP08 you have something to say?**

PP08: Marry-go-round is also there you can save your money there.

**I: Mmmh merry-go-round, isn’t it?**

PP08: They are different.

**I: Ohh (Respondents laughing) explain to me.**

PP08: In a merry-go-round it’s the one that you get every time but Chama you don’t get it daily. So, in Chama let’s say when save you will get it at end of the year and in merry go round for example number one, two, three so number three will be getting money that is how they do it. So, when you get a burial merry go round will not help you but in Chama when you get a burial, they will help you so they will help you.

**I: I have heard you (respondent laughing) PP02, (respondent laughing) PP04 you are laughing, okay fine PP02 do you have something that you want to say okay fine I am asking what makes women who save loves saving in Merry-go round.**

PP08: Now I could have gone for sex work.

**I: What?**

PP08: Now I could have gone for sex work I buy with is shoes for my child, I have plaited my hair (respondent laughing) I have done a lot of things.

**I: In a Merry-go-round?**

PP08: Yes, in a Merry-go-round

**I: How can merry-go-round make you do a lot of things in that merry-go-round?**

PP08: In the merry-go-round we are ten people everybody gives five hundred shillings with ten people it how much? You cannot plait your hair; you cannot buy shoes.

PP01: You can.

PP08: I can buy my life goes ahead.

**I: Thank you, another idea, she has said merry-go-round is there any time who wants to add any other idea about saving in merry-go-round, number 01?**

PP01: I am blank.

**I: You are blank, okay so tell me what has made people who save can love to save in phone save in Mpesa, Mshwari.**

PP01: Mshwari, Mpesa.

**I: Mmmh.**

PP01: What has made people to love it, you know your phone is yours and in merry-go-round people also shift, I gave you left to go to give business at times you have gone to buy a lot of things at a shop. When I have worked hard and gotten my money, I give it to you and you shift with it. So, you know when I put it on phone even if its Mshwari a time can reach when I want a loan and how I continue to pay very well is how they will give me a good loan.

**I: Mmmh.**

PP01: So there, it’s safe, my money is safe.

**I: Mmmh.**

PP01: At Mshwari there its safe than a times I have given a person number one and has shift with my money so when I put it in my telephone I cannot regret. Though in business it will not give me a profit but it’s safe.

**I: Mmmh.**

PP01: It is better than the merry-go-round, you found that a times a person has given you a loan. You have taken your money to the merry-go-round you have worked hard a person goes take loan.

**I: Mmmh.**

PP01: The person has gone with the benefits where she has gone with all the money. So, in a phone it is safe.

**I: Thank you.**

PP01: Okay.

**I: Is there any other idea that a person might have (birds chipping) okay she has supported telephone mmh, it has Mshwari and Mpesa in, isn’t it?**

R: Yes.

**I: What makes other people also love to save in Mshwari and Mpesa, PP06?**

PP06: What has made?

**I: A person to love saving in Mpesa, let’s say any way that you might love so that you can save in Mpesa its which one that has made reason that has made you to save in an Mpesa or Mshwari.**

PP06: The phone is yours.

**I: Mmmh.**

PP06: You have your money near you sometimes you have found an emergency and you need the money urgently and by that time you don’t have it you can withdraw. Sometimes you can go in a merry-go-round and maybe the day that you will need your money you cannot get a loan.

**I: Mmmh.**

PP06: Mmmh

**I: Okay I heard a person saying bank, what has made some people love to save in a bank. Somebody told me a bank PP03.**

PP03: Me as PP03 when you say you save your money it is safe and again there is good security there. And in a bank when thieves go and stole the insurance can pay and you will get your money that was stolen.

**I: Mmmh thank you, any other idea in Mshwari in banks in merry-go-round what makes a person to love saving there.**

PP01: I might use.

**I: PP01?**

PP01: Let’s say in terms of my telephone sometimes I have gone for sex work let’s say a card I live in =Kamito= and I have to do like sex work and I went, the money that I had is cash and the cash money I had has finished and I started to drink alcohol as am waiting for a client, so I don’t have means to go back home and by bad luck I did not get a client and truly there was no client I will be forced to look for a way that I can withdraw the money so that I should find means of going back, a times I had cash money and somebody took it and there is a way that I can look for a person.

**I: Mmmh.**

PP01: You know the Mpesa people there are some who go with their money in their house you can look for how you can withdraw meaning a telephone can make work easier.

**I: Is there any person who wants to add something there and women who exchange sex for money lives in a life that is above them than the one they are supposed to live, sorry the one that is above their salary meaning that they use more money than what they get?**

PP02: Does it happen?

PP08: Those are the ones who are working

PP07: Life that is above your salary.

**I: PP02, do women who exchange sex for money lives in life that is above their salary. Is there a way that they use their money above what they use?**

PP02: Me as PP02, how can use money above what I am getting, I will use money according to what I get.

**I: Meaning you cannot live in such a life?**

PP02: I cannot live in a life that is above the money that I get, how I get my money will make me live a luxurious life or leave a low life. Then it will depend with the client that I get.

**I: Thank you PP09, what is your view there just say, yes PP02, PP04 Sorry.**

PP04: Pas first.

**I: I should pass first, okay.**

PP08: I can support, you must live a luxurious life and you have a little money. You must live a life according to your money. I cannot put my life in a standard of ten thousand shillings.

**I: Mmmh.**

PP08: And I have five hundred shillings, you know I can’t, I must live a life of these five hundred shillings. So, what I see is how on what you have said it depends with amount of money that you get.

**I: Meaning a person lives according to how they earn.**

PP08: Yes.

**I: They can’t leave above what they earn.**

PP08: Mmmh

**I: Any other, PP03 how do you see it, those people who live women who exchange sex for money do they live a life according to what they get?**

PP03: Me as PP03 living a luxurious life and you earn a little money is impossible.

**I: Mmmh.**

PP03: Because when you have gotten the little you have got and the money you have got you must sit down and do your budget on how you can use your money.

**I: Mmmh.**

PP03: You know you can plan a higher budget and the money you have is a little you must reduce it and use it according to your salary.

**I: Mmmh, thank you, is there any different idea or we all have this one idea, give me your thoughts PP09 what do you say?**

PP09: Me as PP09, I cannot put my life in a high standard of life according to my salary it is not enough.

**I: Mmmh**

PP09: Sometimes truly I have a little money, you want to eat tasty food.

**I: Mmmh**

PP09: It is impossible.

**I: Mmmh.**

PP09: You can buy even kales so that you eat. Salary is different. You can get today a lot and tomorrow you get a little so how you get it is how you use it time you get it you use it.

**I: Mmmh, okay fine and women who exchange sex for money love taking loan or even they are in other loans, does it happen, PP07 Do they take loans?**

PP07: Pass I am still thinking (respondents laughing)

**I: Please PP07 just say yours, PP04.**

PP04: Let’s say now my body is in pain.

**I: Mmmh**

PP04: I have not gone for sex work, I will be forced to go and borrow

**I: Si where will you borrow?**

PP04: I will borrow from the savings that we said.

I: Mmmh.

PP04: Yes

**I: So let us say here on sickness**

PP04: Yes

**I: Any others person who wants to add on that, PP06 what do you say, do women exchanging sex for money love taking loan or they are also found in other loans.**

PP01: I want to say.

**I: PP01**

PP01: Let’s say a female sex worker is like any other human being, she is like any woman she might wish to do anything for example she did not find a good thing.

**I: Mmmh**

PP01: And maybe her child has been sent fees, she will be forced when she is saving in that we have among KCB you will be forced to go to Mshwari and take a loan.

**I: Mmmh**

PP01: So that the child should not stay at home it reaches a time that you are sick you have gone a times you don’t have NHIF and you have to go to the hospital and you have been discharged you don’t have enough money, this telephone a times you can say that as for me our merry-go-round it is like this and this you can give a person a loan and then you say a day for a merry-go-round I will go and pick money and return it.

**I: Mmmh**

PP01: So, a sex worker is just like any other person she can also have an emergency until she wishes to take a loan.

**I: Mmmh so let’s say you can take a loan when you have emergency or people take it in a merry-go-round and return it isn’t it?**

PP01: Yes

**I: That has made us to move to another step, any other idea, do we take loans, do we have debt that we loan PP07 have you got an idea?**

PP07: I still don’t have an idea.

**I: Okay PP05 give us your idea, PP07 where are you thinking, we are the ones who are here.**

PP05: Me as PP05, I must take a loan because when I have not gone for sex work and I don’t have a way forward I will be forced to go for a loan so next time when I go for sex work and get I will find how I can pay.

**I: Mmmh.** Thank you, yes I have heard two ideas that for a person to pay for a loan you will go in a merry-go-round and another one has said in sex work. Which other can we use to pay loans, ways that we can find money to pay our loans, number?

PP08: Ways that we pay our loans.

**I: Mmmh.**

PP08: I don’t live with my fellow woman.

**I: Mmmh.**

PP08: When I have hopes in merry-go-round, I will go and take a loan there that’s what I had.

**I: Just say louder I want this recorder to record.**

PP08: This thing touches me.

**I: Mmmh. (Respondents laughing)**

PP08: Where is this girl going (respondent laughing)

PP04: I am going for a short call (respondent laughing)

**I: Okay fine the recorder has recorded you (respondent laughing)**

PP08: You see how you are laughing

**I: Mmmh**

PP08: You see you have laughed, if you were to die today you have added some days respondent laughing)

PP07: This thing touches me.

**I: Mmmh okay, where can we find the money that we can use to pay our debts?**

PP08: I can even go in a Merry-go-round

**I: Mmmh**

PP08: I take some money, I go and borrow some money in a merry-go-round, sometimes I have their debts and the merry-g-round has gone at Mombasa and I have debts I can tell my client that I have a debt that is of this amount, I am lying because you cannot tell the exact amount the client can reduce. If the client gives me a little, I have gone to another client, you go with that client you know in those ten I know how their pocket is.

**I: Mmmh**

PP08: I am going to another client then I lie that my child is sick and the child is not even speaking you know in that situation I must be given the money.

**I: Mmmh**

PP08: But gives sex to the clients he wants.

**I; Mmmh**

PP08: And with all the amount of I have paid.

PP01: You have paid.

PP08: I have paid (respondents laughing)

PP09: I want also to add something on that as PP09.

**I: Mmmh**

PP09: I cannot lie to myself and mind that I am young to take the money at merry-go-round that is still a debt, I must go for sex work.

**I: Mmmh**

PP09: So that is why I should find the money that I will use to pay debts.

**I: Mmmh**

PP09: That is my thought.

**I: Mmmh, you are to find it sex work, any other idea PP06 tell us what**.

PP06: Me as number 06.

**I: Mmmh**

PP06: When I have debts and I know very well that I am sex worker in.

**I: Mmmh**

PP06: Sex work I will look for money that I will use to pay the debts.

**I: Mmmh, okay so how in sex work what do you do so that it can some money, so that you can find how you pay debts.**

PP04: What you do at sex work

**I: That one (noise at the background) PP04**

PP04: Me as PP04 if I can find a client who has money.

**I: Yes**

PP04: And I know that I am looking for money so I have put that today I want to eat a tasty food.

**I: Mmmh**

PP04: When I reach there even if I give, I will get two thousand shillings in two and add them to four.

**I: What two?**

PP04: The game that we will say.

**I: Mmmh**

PP04: Let me say in dholuo so that the client can have sex with me four rounds are you hearing me very well.

**I: Mmmh**

PP04: I must get the money to go with it back in the house.

**I: Thank you.**

PP04: I had prepared myself very well with things for war, I am doing sex and I will find the money that I will go with it back in the house.

**I: Yes, thank you, PP03 (respondents laughing) yes PP03, what else do they do in sex work so that they should find what else do they do in sex work so that they should find what they should add in their money to pay debts or anybody else who can tell us.**

PP03: Me as PP03

**I: PP03 yes**

PP03: I think what I do first is cleanliness after we have agreed well for sex work the perfume that I have smeared when I pass and a person has heard.

**I: Mmmh**

PP03: PP03 is what they want you know they can’t choose PP06 and PP09 because she has passed us I behave as if I want and truly the style that you will give, they will forget the others (respondents laughing) and after they have forgotten the client just do like this, other you put it anywhere (respondents laughing) you give it.

PP09: Nigeria style.

**I: PP09 you are saying that?**

PP09: Me as PP09 I am saying you put it with the head, Nigeria style that I always see they always put money on the head.

**I: Mmmh, so you put it Nigeria style it just enters?**

PP09: Mmmh

**I: Mmmh, anybody else who wants to add something, what you do so that you can add on that money you find the one that can help yourself with it and the one you pay your debts with it, PP01 you are blank there?**

PP01: I am blank.

**I: Yes**

PP04: Even if they had put a lock in it, I open it.

**I: What are you saying PP04?**

PP04: I am saying that even if it has been locked, I will open it.

**I: Where do you put a lock?**

PP04: When I have put a lock in the boot, I open it now.

**I: Mmmh.**

PP04: So that I should find how I can pay debts because I don’t have a way that I will help myself with.

**I: Mmmh**

PP04: And my hopes are there.

PP03: If your waist has stopped you add it.

**I: PP03 what are you saying, we want to hear what you are saying.**

PP03: If there is no waist you can continue shaking it maybe you are shaking it different with how PP05 is shaking (respondents laughing)

**I: Thank you, is there anything that a person wants to add there even those women who exchange sex for money always do so that they should pay their debts. PP08, you had something to say you have forgotten the idea. Don’t forget it.**

PP08: First of all.

**I: Mmmh.**

PP08: I want to go for sex work and I have eaten my stomach is full, I have showered I have done things that I can do on my body down there I have not won a torn inner pant and I have removed it, it is clean.

**I: What have you done?**

PP08: I have removed the hair on my vagina (respondents laughing) I have carried a condom so when I go there at 90 degrees, I will get the money (respondents laughing) I will then come and pay the debts.

I: You pay.

PP08: I will pay… every round five hundred shillings.

R: Can he pay?

PP08: We agree, will he come on top of me and we have not agreed.

PP01: ‘’Pesa e lwedo pier e kitanda’’ [Money at hand vagina on bed].

I: Mmmh, that is PP01 who has said (respondents laughing) PP02 does not want to speak. You are respondent 2 add us something what can we add so that we can pay our debts so that we should continue as usual.

PP02: I will answer another one.

**I: So, let us move to another question, what does women who exchange sex or money do so that they should add their salary apart from what we have said, what do they add on their salary.**

PP04: Love first.

**I: PP04: is saying love first, when you say love first what do you mean?**

PP04: I give him love, I give love first when you don’t show love a times you have gone there and you are quiet how will you laugh?

**I: Give love means what?**

PP04: Giving love, when I go with a client in a room, what has taken me there?

**I: I don’t know.**

PP04: I will start with a massage so we are moving ahead and it will where it reach (respondents laugh) .

PP08: You kiss the penis (respondents laugh)

**I: Number 8 have you said? Just say what you have said.**

PP08: Its love when you kiss the penis, you kiss his mouth you go to a native doctor to give you something.

**I: That is what she said, you take him to a native doctor.**

PP08: You don’t take him, you go there when he does not know, and when you take him there the love has ended.

R: He will know you love witchcraft.

PP08: Yes.

**I: Mmmh.**

PP08: You smear it on your face (respondents laugh) there is more love at first a man is insane that after you have touched him, you kiss him on the mouth, you touch his penis a little that person will love you more (respondents laughing) after you have prepared his penis like this, he has love you (respondents laughing).

R: You (respondents laugh)

**I: Number 7 add us something here, what do women let’s say sex workers can do so that they can add their salary (silence) just say your thought that is why we are here with you.**

PP07: Repeat so that I can say.

**I: What do sex workers do so that they add their salary?**

PP07: As for me what I can do so that I add my salary.

**I: Mmmh**

PP07: I can smear it for it to be tighten so that when he enters.

**I: Mmmh**

PP07: He will feel like am a virgin.

**I: Mmmh**

PP07: It will make him happy.

**I: Mmmh, thank you, she said what she can do so that the client can see that is tighten isn’t it? That it is tightened he is the first one to do it, it sis like that isn’t it number 7?**

PP07: Yes

**I: Mmmh, anybody else how can we increase our salary in sex work number 1 do you want to say something?**

PP01: No

**I: Please and the way you have your telephone on your hands what can we do, you want to add something?**

PP01: No.

**I: Number five, there is none, you don’t have any other thoughts**.

PP02: None, there is none.

**I: What if women who exchange sex for money did not find a client who pays them what can they do.**

PP02: Like

PP08: I can bite his penis.

**I: I want one person at a time.**

R: An what I

**I: Number 8.**

PP08: I will bite his penis.

**I: What will you do (respondents laughing?)**

PP08: What I can do I might leave him (respondents laughing)

**I: Mmmh**

R: (inaudible)

**I: The question is sharing what can women who exchange sex for money do sorry. What if women who exchange sex for money did not get a client who pays them what do they always do? When they don’t get clients who pays them.**

PP04: So, when I bring a client who pays.

**I: Mmmh**

PP04: We will find something like law, Jitegemee.

**I: Let’s live Jitegemee first**

PP04: Is what I am telling you. The normal life.

PP04: That is why I am telling you when I did not that you find he refused to pay you, you got nothing. So, no your savings will help you when you go for sex work and you did not find.

**I: Mmmh**

PP04: So, you go back to your savings.

**I: Mmmh**

PP04: We have client and has refused to pay you and you went for sex work and you did not get a client.

**I: Mmmh**

PP04: Like a times you can go to the market so that you can sell sardines and it did not work out well so you will be having what you have been saving, even the money that you were keeping you will that since sex work did not work out well, I will remove the money that I have been saving so that the child can eat.

**I: How do you see that now that time has reach its like I have not found a client how do you see it?**

PP04: So according to me its ten.

**I: Mmmh**

PP04: And the day of you know a times sports does not happen.

**I: Mmmh**

PP04: I have gone and I have not seen a client and you have sat there for like 11 hours.

**I: 11 HRS in the afternoon or at night?**

PP04: At night.

**I: Mmmh**

PP04: You have tried to sit there like five hours and daily you know the time that the client always picks you its 08.00 PM and as for you, you sat there until 11.00 PM it will inform you that sex work you have no client and you will have to look for another alternative on how you can help yourself.

**I: Mmmh thank you, I heard you saying that we have two things we have a client who pays you and a client who pays you and a client who does not pay you. When you find a client who does not pay you what will you do.**

PP04: When I find and he did not pay me we will agree to ourselves that we want to do this and this on a daily basis I always say that you put money on my hands first virgin on bed so that I should not regret because when I have undressed myself you will have sexual desire you will have sex with me and there will be nothing to help me.

PP08: They forget.

PP04: There will be nothing to help me because at that time there will be no police, I will be shy that the things I do in secret how will I involve police in a small thing like this. I will not tell him.

**I: Mmmh.**

PP04: So, for us to have sex we must negotiate first before we have sex.

**I: mmh**

PP04: So, for him to do those rounds with me he will put money first on my hands if he does half, I will not return it to him and if he does it full (audible)

**I: So, for all those rounds he gives you once.**

PP04: Yes

**I: When he does it half you don’t return it.**

PP04: Yes, I will not return (respondents laughing)

**I: Okay number two tell us something my question was**

R: (clearing throat)

**I: What if women who exchange sex for money, they don’t find a client who pays them what can they do (noise at the background) a sex worker I have gone somewhere and did not find a client who pays me what can I do (noise at the background)**

PP02: Me as number two.

**I: Mmmh**

PP02: (clearing throat) Me as number 2 if I did not find a client who pays me.

**I: Mmmh**

PP02: (silence, noise at the background) Mmmh

**I: She is still thinking, we will get back who can give us her thoughts number six do you have a thought when you don’t find a client who does not pay you what will you do?**

PP06: Me as number six, there is where what you have been saving might help you.

**I: Mmmh**

PP06: Because here at sex work you know sex workers always have those who they love, sometimes you can go with the client that you always love.

**I: Mmmh**

PP06: And did not give you, you will not force him to give you.

**I: Mmmh**

PP06: So, there is when you can go to your Mpesa and withdraw your money and go to use it.

**I: Mmmh, so how can you realize that your time, it’s like I cannot find a client who pays me, how do you know?**

PP06: As number one has already said, you might rely on the time that they give you and when your time has passed you will just know that

**I: When you say that time you have picked means?**

PP06: May be a times you can enter and get a customer sometimes you can sit for about an hour and you get a customer.

**I: Mmmh**

PP06: So, when it passes you, just know that the sex work will not work out so the money you have always been saving you can deposit it so you will use it at home.

**I: Mmmh thank you, number 9 you can add us something there.**

PP09: Mmmh

**I: Mmmh**

PP09: Me as PP09, you can find someone who truly goes with you and does not want to give you money and in terms of money I always get a lot of it.

**I: Mmmh**

PP09: The first thing that always ask, when I want to know that you don’t have money how will we eat in the morning, how will we handle ourselves in the morning and he will tell you that he has left the money in the house.

**I: Mmmh**

PP09: And what I will do with you, I will tell you that wait for me I am coming back, I have forgotten something or even I am going to wear sweater and we are just there. Migingo there is near us wait for me I am coming back. Then I take my telephone and put them on my ears and I say yes the network here is not working and the way I have left I will just left like that you will not see me.

**I: Mmmh**

PP09: Something is also here.

**I: Mmmh**

PP09: A person can have sex with you and tells you that he will send you the money. You cannot demand for sex work money and there is nothing that you can do so you must calculate with your mind. That is my thought as number 9.

**I: Mmmh that is when you have found a client and has refused to pay you isn’t it with that time?**

PP09: Yes you got it, what you should start to ask is that money, because it will help you (inaudible content)

**I: So, a person who did not find those who pay not that you find and has refused to pay you?**

PP09: Mmmh

**I: You did not find a client who can be with you so that he can pay you.**

PP09: In that case the little money that you have been saving will help you.

**I: Mmmh**

PP09: Yes

**I: How will you realize that this time is like I have not find a client now you will go?**

PP09: You will go and sit somewhere and when you realize the time is moving but there are no clients, will you continue to quit you will go back in the house, so when reaches tomorrow the money that is in your phone the one that you always save will be the ones that will help you.

**I: Thank you. Number 3 is there anything that you want to add there, number 5?**

PP05: No

**I: You don’t have any, so there is a place that I want to read here first before I go backwards (clearing throat) We have come here because of Jitegemee and let me read here Jitegemee before3 I explained that it’s a research of jitegemee again explained that it is done so that the women who exchange sex for money have money that they save that can make them reduce to have sex without condom so that they can relax that they don’t exchange sex for money the time that they want to rest, we also said that she is above women who exchange sex for money aah, so that they can save their money themselves they use it when there are no clients who pay and also to help them in preparation for the living the life of exchanging sex for money. So, I would like to know so what I would like to add in Jitegemee we have a merry-go-round, is there any way that Jitegemee is different from a merry-go-round a little. As programme for Jitegemee wants to come it is just your money. It will not give you the money that you can save.**

PP01: (clearing throat)

**I: That is number one JItegemee will not give you money to save there is a way it will teach you that the little amount of money you get that you get you are the one who knows anything that you get you can save some and use the remaining. Secondly Jitegemee it won’t give a limit that you are supposed to pay and the time that you are supposed to pay. You individually when you entered Jitegemee and you have said that please I want to save at Jitegemee and you like saving everyday and put your money there is a problem even if you put a specific amount that you will be saving every week there is no problem even if you put it monthly there is no problem. They won’t put a limit for you, the limit is yours because it is your money even if you would wish to live and you live, its not like merry-go-round that you will need your money is the time you will take it, there is no interest let’s say that you had emergency and you have come to pick your money that when you return it will have interest No there is no interest are we together.**

R: Mmmh

**I: There is no interaction that Jitegemee will bring to you it is you your money that you will come to pick. If you wish to take all at of it you just take all, if you wish to take half of it and you see how you will add on it, it is okay. Even if you see that you want to start saving maybe fifty shillings while you are seeing how its progressing then you see if you can add there is no problem. Your money is yours no one will touch them and we will not tell you that we want you to save at bank even Mpesa the decision is yours it’s just that there is a way that it will support you in your savings so that you become strong in saving, it might help you in future, are we together?**

R: Mmmh

**I: So, my question is according to how I have explained to you about Jitegemee. You see Jitegemee as a thing that other women who exchange sex for money who are sex workers can they agree?**

PP08: (inaudible)

**I: Number 8?**

PP08: Yes

**I: How do you see it?**

PP08: They can agree.

**I: Just say it loudly.**

PP08: They can agree to enter.

**I: Mmmmh.**

PP08: Because where you save ten shillings or five shillings you have hopes it can help you investing.

**I: Mmmh**

PP08: That is according to my thoughts, you can find where you can save.

**I: Mmmh**

PP08: That was my thoughts.

**I: Number 7 how do you see it?**

PP07: How I see it is just how my fellow has said.

**I: What has she said?**

PP07: Just the way she has said (respondents laughing)

**I: Yes, that’s why I want you to tell me what she has said. According to what I have read about Jitegemee. Do you see it as if sex workers who are here in Kenya can agree to join?**

PP04: Yes, they can agree.

**I: Why do you think so?**

PP04: When my fellow has seen how beautify myself and how I decorate my house.

**I: Mmmh**

PP04: She will also wish to join so that she can beautify herself just like me.

**I: Mmmh**

PP04: Mmmh

**I: Any other thoughts number one how do you see it?**

PP01: I see it is good if a sex worker joins Jitegemee because when you have enterd Jitegemee if you put even like ten shillings.

**I: Mmmh**

PP01: Those ten shillings will be many you will not forget that even if you are tired or not tired even if you feel like today, I will just be indoors sleeping because on daily basis there is no sleeping.

**I: Mmmh**

PP01: So, I am not going to work, you can find how you can help yourself a times when become sick you can find how to help yourself.

**I: Mmmh**

PP01: Mmmh

**I: That is why you are seeing that if something that a sex worker should join isn’t it?**

PP01: Yes

**I: Do you think it is something that you can join?**

PP05: Yes

**I: Mmmh**

PP05: Sex workers can also enter because it is something that helps.

**I: Mmmh, number 2 give us your thoughts how do you see it according to what I have read, how do you see it do you think sex workers can wish to join.**

PP02: Yes they can love to join.

**I: Mmmh, elaborate to me a little why do you think they can love to join?**

PP02: They can love, when you find your little money and you have saved even twenty shillings.

**I: Mmmh**

PP02: One day you don’t have it you can go and pick it there.

**I: Mmmh, thank you number 4 you are still thinking.**

PP04: You will just pass me.

**I: I should pass you little, PP03 how do you see it do you think Jitegemee is something women can love to join according to what I had read that I have told you.**

PP03: They can wish to join because saving money alone sometimes it is difficult, so when you find where it can be saved for you it can be very good.

**I: Mmmh, okay when we move to another question which characteristics do women who exchange sex for money have that can make them love to join. Which type of sex workers can wish to join Jitegemee?**

PP01: When I see it.

**I: Number one.**

PP01: I see Jitegemee, I think it has not put which type of sex workers are joining.

**I: Mmmh**

PP01: I think it is any sex worker so long as you can bring the little that you got, so I don’t think if they have put that you are supposed to get a huge amount and you must get a little like this I think it has allowed anybody to join Jitegemee.

**I: Thank you, number 8 did you want to say something which women who exchange sex for money can wish to join Jitegemee.**

PP08: Yes, it has put together all the sex worker that do sex work.

**I: Mmmh**

PP08: That can join there.

**I: That**

PP08: I am saying what you have is good and can bring together.

**I: Yes**

PP08: Yes, it has brought people together who can join Jitegemee it has not discriminated that the person can join and that person cannot join.

**I: Mmmh, okay maybe when we look at it, we have explained what Jitegemee wants and how Jitegemee will be, according to what I have told you what things do you see that won’t be easy also people will not love it (silence)**

PP08: How many did you explain?

**I: Mmmh**

PP08: How many did you explain?

**I: Number 8 you are asking if I explained to you? I said that Jitegemee is just an organization that wants to help those who exchange sex for money so that they can see how they can save their money by themselves, your money not that Jitegemee will come and give you money for you to save, no just your money secondly it is different with other merry-go-round that this amount of money is what you should pay per day, per week or per month. The amount of money is what you should pay per day, per week or per month. The amount of money that you love either fifty shillings, twenty shillings, ten shillings per day or per the week, the amount of money that will love, when you want to take it there is nothing that will be put like it should be taken after how long because it is your money.**

PP08: I have a question there.

**I: Mmmh**

PP08: Does it have interest?

**I: Mmmh, we said that it does not have interest, it just saves your money you will take it and if you want to start bringing you can still bring it there is no interest that they will ask you to bring. So according to what I have said is there anything that you see women cannot love maybe will join and will not love it even it will hinder women who exchange sex for money to join?**

PP01: What I can see.

**I: That is number one.**

PP01: My view is just agree that the time I will be saving a times I put even ten shillings and I wish to pick my money, I would love to pick a loan, that is what I wish after I took it as a loan even if it is one hundred shillings a times that one hundred shillings I wish to take that one hundred shillings is little will not help me solve my problem when you will put it as a loan at times I will need six hundred shillings after I have saved one hundred shillings I would even if I can take two hundred shillings so it will help me to solve my problems.

**I: Mmmh that is an idea that she has gave us, it will be heard there. Yes, anybody else who have another thought numbePP09 how do you see it?**

PP09: I am still thinking.

**I: Number 4 give us your thought, according to what I have said what things do you see this cannot love?**

PP04: Pass there.

**I: Number 2 I would love you to give me your thoughts because it will help us prepare on what we want to do on your future.**

PP01: I was trying.

**I: Number one.**

PP01: I was trying to say question that this Jitegemee when I want to save my money, who will keep them or how will they do it?

**I: They will tell you; they must explain to you very well as for now we want your thoughts as for you would you wish who to save them that is the thought that I want us to say.**

PP01: No will talk about it when the time reach.

**I: No, just say, you know when we say our thoughts right now, they will use it to prepare those things so that they should see how they can do it. As for you what is your thought. We said that there is no bad thought just remember it let us be free.**

PP01: Mmmh

**I: There is no bad thought we welcome all of them.**

PP01: According to us right now you know we choose one another it’s not that we have chosen ourselves we came when you have selected us. So, you said there joining when you feel like joining and if you don’t feel like joining you don’t join. So those people who will join it, it can disrupt our “kosalo”, I don’t know if you know “kosalo” is where you can take your money and they write, meaning you save your money and you go and pick it, yes. According to my thoughts as number one see that is something that will be there because we will not give money to person who is not with us here.

**I: Mmmh**

PP01: We will sit and choose one of us and it can be our secretary, treasurer, chairperson and other.

**I: Mmmh**

PP01: And they will save them that was my thought.

**I: Mmmh thank you that was her thoughts. Anybody else number 5 how do you see it? (Birds chipping) Number 9 remind me or you are still thinking, number 8?**

PP08: Repeat it for me I am still understanding you.

**I: You are not getting it Jitegemee**

PP08: I don’t know whether I am hungry.

**I: Mmmh**

PP08: Meaning you see where you have stopped, I dint understand you.

**I: Mmmh**

PP08: Yes, can you elaborate it for me?

**I: I don’t know what I should elaborate you see we are coming with research called Jitegetee is research that is coming so that it can help you depend on yourself when you don’t have a way forward even when maybe you did not get a client or it has brought you some restrictions and you say NO, I cannot manage that one and at least you have your own money somewhere that you can go and pick to help yourself with it. If there is something that some women want to do and you are not agreeing with them you can say no one because by that time you have your own money that you saved somewhere and you have a voice. You know when you don’t have your own money that you have not saved anywhere you can say that I can just pick it whatever they say or want to do I will just accept because I know I don’t have a place where I can go isn’t it?**

PP08: Yes.

**I: So Jitegemee will come with a programme that will help you save that you can see how you can help yourself in the future. Okay even if you want to stop sex work because sex work as we have agreed I heard someone saying that is something that can it is there forever and you also said that you want to stop it. So, the time that you will stop it maybe you have money that you saved. The money that you saved Jitegemee will not tell you that you must save this amount of money in this period of time. It is for you to look that according to my salary and my needs I can save how much money and will it be daily or weekly or monthly. The time that you want to pick it no one will put restrictions to you that the amount has not reached, you need a guarantor those things are not there it is your money you just pick it. If you wish you can pick half of it you can withdraw any time you wish because it is your money nobody will follow you for as long as you have been taught what it is.**

PP08: Can you save it on phone?

**I: All of those re the thoughts I want you to give us, it is just Jitegemee that will teach you what it is. If you wish you can put it on phone so that they can teach you how you can save it and so on. We will not choose for you where you can save**.

PP08: Mmmh

**I: Mmmh**

PP08: So according to my mind we will see how we can speak (respondent laughing)

**I: You see that now I just wanted your thoughts so that we can come up with this programmed. You see even a bank they have things that they put. Those things came up from other peoples thought that when you start our accounts should be like this so that we can help ourselves. So, your thoughts are what we want today**.

PP08: So, you want the ideas on how you can save the money?

**I: How you can save as you see things, I have explained to you are there things that women will not love that can hinder them to join.**

PP08: What will make people not to save?

**I: Mmmh**

PP08: Even women will love to save, let me start with women who will love.

**I: Just say**

PP08: Let’s assume that we are here we are at the merry-go-round, I don’t know its name because now I am hungry. I have taken the money. They give it to me let’s assume that I am the treasurer I have gone and used it, the day that they will need it don’t have it. You know another person will not join it because the person who do not use her brain will go there and start a fight and live it and when they save it on phone it is easy. You know when you use your phone these days …a sim pin can mislead because we can change that we put a person who has a pin differently with a person who has line that always lie to people because they can withdraw the money secretly without you knowing. So, as for me what I see they teach us and the money every person should save it in her phone.

**I: Mmmh**

PP08: That is my thought.

**I: Thank you that is her thought, anybody else number 3 how do you see it?**

PP03: Me as number 3 I see Jitegemee as a good thing and they should continue teaching us, they should teach us how we can save there and what can hinder other sex workers to join. There are some who can say that when I always save my money even Banks my interest always increases and here is no interest that is increasing.

**I: Okay thank you any other thought, anybody else who can give us another thought. There none, number 6 it’s like you have something to say, there is none, okay fine. I am asking those women who exchange sex for money meaning sex workers how many do you think can agree with it for example let’s say in a group of ten. You have already been taught about Jitegemee out of ten how many people can agree to join?**

PP08: About 5 per cent.

**I: Five percent**

PP08: A times all of them can join so it depends with someone’s heart, know what the heart of number one thinks I cannot know. Yes, so it depends with someone’s heart.

**I: Mmmh**

PP08: Yes

**I: So, we think?**

PP08: We just think.

**I: According to how we have explained it.**

PP08: Yes, a times everybody can say a times 5% can enter so it depends with someone’s heart.

**I: Mmmh thank you that is number eight, anybody else out of ten who are taught about Jitegemee how many people do you think can join Jitegemee, let’s do so that we can finish quickly, let’s talk number 7?**

PP07: Mmmh

**I: Number 4 out of ten when I talk about Jitegemee how many people can agree to join?**

PP04: 7%

**I: Number 7?**

PP04: Mmmh

**I: Seven percent when you say seven, seven out of ten?**

PP04: Mmmh

**I: That is seventy percent.**

PP04: Mmmh

**I: Mmmh anybody else, meaning three people, seven people can agree to join according to you seven people can agree to join and maybe will not agree, is it**?

PP04: Mmmh

**I: Anybody else, another thoughts number 2?**

PP02: Me as number 2 I think everybody can join.

**I: You think everybody can join?**

PP02: It depends with somebody heart.

**I: Mmmh**

PP02: Mmmh

**I: Okay number….**

PP02: It depends with somebody’s heart and the teachings they had.

**I: Okay number 4 you said that three people a times cannot join according to the teachings, is it?**

PP04: Mmmh

**I: You think those people what can hinder them not to join?**

PP04: The thing that can hinder them to join

**I: Mmmh what can make them refuse to join.**

PP04: According to how we were those are those people who are willing to join and those who are not willing to join

R: What is here I can add.

**I: Mmmh, let her finish let number finish, finish first.**

PP04: Me I don’t have anything to say I was saying those are those whose are willing to join and the ones who are not willing to join.

**I: Mmmh**

PP04: It depends

**I: It depends with?**

PP04: The people’s heart.

**I: Mmmh, okay fine numbers.**

PP01: I was trying to add that so that the seven should agree and the three to agree a times these times three look at their interest, sometimes they took their money there and the one that they took there is the one that they will get. A times they will see that they will put their money where there is no profit, the second one mmmh live it there.

**I: (Laughs) why have you refused to tell me the second one?**

PP01: No, I will repeat myself.

**I: You wanted to say what you already said? Yes, anybody else who wants to add, number 3 how do you see it? None okay, tell me,… what do you think we can do so that we add as many as possible the number of women who exchange sex for money so that they should agree to join Jitegemee. What can we do?**

PP03: What we can do so that we can add their number.

**I: So that it can add the number of people to join.**

PP03: So that they should join.

**I: Yes.**

PP03: You know (inaudible) you know truly when you do your business because sex work is a business that a person does so that they can help themselves with it. When you are doing your business, you go and save your money you know you will need your own interest that you grow with it so you see the goodness when you save and the interest also increases so that one day when you decide that you have been saving, I do with it certain business because people always get tired. After you have gone to take it you find it has increased.

**I: Mmmh thank you, number five, please number five I am going to finish when I have not heard your voice (noise at the background) how can we do it so that Jitegemee can be good, number six how can we do it we need ideas (noise at the background) Yes (silence) there is no idea, who wants to tell me, number three do you want to add something, do you want to add. Number nine, number eight.**

PP08: What we can do it can encourage you through teaching on its benefits.

**I: On its.**

PP08: On its benefits

**I: Mmmh**

PP08: Meaning teaching people the more you continue teaching people will increase and the things that are there on what we can do there.

**I: They teach the good things there are there.**

PP08: So, it can bring a lot of people.

**I: Thank you any other thought number 4 how do you see it what can we do so that.**

R: Can you repeat for me so that I should say.

**I: Yes**

R: Can you repeat for me so that I should say.

**I: Soo, this thing of Jitegemee, should I have it just the way it is?**

R: We forget.

**I: Mmmh, in nine people.**

R: But we are supporting until we lead.

**I: Number six tell me what we can do so that it can put it to be good Jitegemee.**

PP06: It is just teaching and the good things that are just as number eight has said.

**I: Mmmh okay so I want to move quickly what things are Jitegemee supposed to do so that people should accept it differently with what we have said. What should they do so that people should accept it, number two?**

PP02: I am still thinking what should.

**I: Anybody else number eight.**

PP08: What are people supposed to do?

**I: Those people who want to join?**

PP08: According to the truth that is there you know truthfulness is greater than anything. Yes, and when we save money and our money get lost what is the good thing in it, there is none. So is truthfulness and its teachings as that thing is supported, how it is committed is how it will encourage other people.

**I: Okay fine is there any person who can add something there, yes number nine?**

PP09: Me as number nine I am trying to support what they said, you see today the teaching that you are teaching I very well I will be forced to tell my fellow sex workers so that we can go together to hear the teachings that is what will make them to increase.

**I: Okay.**

PP09: That was my thought.

**I: Thank you and according to things that I have tried to elaborate about Jitegemee are their things about Jitegemee that women who exchange sex for money can see that it is destroying their work. According to what I have said is there anything that can destroy someone’s stand, number eight you are saying?**

PP08: There is none because when I have gone and come back (inaudible) it does not destroy.

**I: Mmmh, number one you wanted to say something.**

PP01: There is nothing I want to say.

**I: Any other thought does you see, according to what I have said about Jitegemee is there anything that you see that can destroy someone’s work, work of the sex work?**

PP05: There is none.

PP04: t does not destroy.

**I: Number four what you saying.**

PP04: It does not destroy.

**I: Why do you feel like that?**

PP04: I don’t see anything that can be destroyed there.

**I: Okay fine, what difficulties you think we can find from things that we are doing and which thoughts you can give us on how we can overcome those difficulties. Which difficulties do you think we can have in Jitegemee?**

R: Difficulties that looks like?

**I: Mmmh**

R: Difficulties that look like?

**I: That is what we are asking you, they are which difficulties when we come with Jitegemee, which difficulties can make us have challenges or that makes girls or even women to join Jitegemee. Which difficulties can we have?**

R: First of all is money, when you don’t have money you cannot join, so one among them is money when the saving starts, it is what is doing everything.

**I: So, what can they do about this money?**

R: So that you get it or?

**I: So that it can encourage people to join.**

R: So that it can encourage people to join.

**I: Mmmh**

R: It’s just according to what we have said that we are still heading backward. It just to tell us the truth that is there so the difficulties is just money. A times I might wish to join the research so that I should be empowered and I don’t have money so there is no way that I will join.

**I: Mmmh.**

R: Yes

**I: Any other thought, which difficulties do you think we can find in the time that we start this Jitegemee research number three how do you see it which difficulties can we get?**

PP03: The difficulties that we can find it depend on how you know when you want to save money in it there must be trust. You must be with people whom you trust you can take. You can go do sex work; you can say that today I will send this so that they will save it. So, it depends with the trust that is among them and the people that you are saying.

**I: Mmmh, thank you any other difficulties, okay and mmmh which amount of money do you think they can wish the sex worker can wish to save on weekly basic when it has not destroyed their plans at having things that they are important that they must do every work.**

PP08: Fifty shillings.

**I: Number 8 has said fifty shillings every week, yes anybody else here I want everybody to tell me. The amount that you think, number nine.**

PP09: 100/= shillings every week

**I: 100/= every week.**

PP01: 100/= shillings.

**I: That is one, number two what are you saying?**

PP02: 100/=

**I: 100/= every week**

PP04: 100/=

**I: That is number 4, any other thought number six?**

PP06: 100/=

**I: 100/= shillings, yes number seven?**

PP07: 100/= shillings (respondents laughing)

**I: Yes, number three how do you see it?**

PP03: Even 200/= shillings.

**I: Mmmh, every week, number 5?**

PP05: 200/= Shillings.

**I: Two hundred shillings every week, so how can we save this money where women who exchange sex for money sorry, how can we save this money where women who exchange sex for money can have trust that there is no bad thing that can happen. How can we save even, where can we save it? Where those people who are there can have trust that there is no any problem with the money. Mmmh number 3?**

PP03: How then can save it?

**I: Yes.**

PP03: There you know we have become as people from one family, we have treasurer, secretary and the chair person. So, you know our fellow sex workers cannot save the money in the house, that one will not agree so the money we can save it on phone there is Mshwari and others. So, there is the place where we can save our money.

**I: Mmmh, Mshwari so long as it is on phone, as long as there is nothing bad that can happen mmmh, any other thought how can they save this money the sex workers who join, join with trust that our money is not at a bad thing that will happen there, number two.**

PP08: I am supporting Equity bank.

**I: You have said phone and that is number eight you have said Equity bank.**

PP08: They are tired so let me help them.

**I: Yes**

PP08: Equity bank, Mshwari so they will choose which one is good for the Equity, KBC (respondent laughing) no you see how they are laughing so where I have passed, they are laughing so where I have passed, they say it. It is better than the ones which are saved on hands.

**I: When they save there what do you mean?**

PP08: KBC (respondent laughing)

PP02: My fellow I am tired.

PP08: Even Equity (respondents laughs)

PP02: I will have headache.

PP08: Even Equity it is good.

**I: Let’s say there a person who will go to steal there is none.**

PP08: There when you even go you can’t need a treasury, secretary and the chairperson must be there members also know. One member must also be there so that they should go there.

**I: Number one you were saying something.**

PP01: I was just laugh

**I: You are laughing in what you have not said, okay fine number three do you want to say something.**

PP03: Me I think they should open an account that you can put the money that it goes.

**I: Mmmh, okay let’s say sex workers did not get money that they agreed they should save what things can they do so that they should find that money so that they should save the way they wanted to save.**

PP08: (INAUDIBLE SEGMENT)

**I: Number eight say what you have said.**

R: Just repeat it louder.

PP08: I was saying that when I was supposed to save money and I did not find the money. I will go for sex work and other I will go for sex work I will go for

R: That is which work?

R: I don’t know.

PP08: I can go for farming

PP08: After you have gone to farm and you farm very well you will get the money.

**I: Mmmh**

PP08: Yes

**I: Thank you, number six, how do you see it (respondents laughing) when those people who exchange sex for money did not get the money that they agreed they should save. What things will they do for them to find that money so that the save it. I said that as for me I wanted to save as we have said a times we can save one hundred shillings in a week and reaches that week and you don’t have that money that one hundred shillings. What do you think we can do, even sex workers can do that they find this money per week?**

PP04: As for me.

**I: Number four.**

PP04: I will just go back to sex work like before so that I should find the money to save.

PP08: There is no any other thought.

**I: What do you say (respondent laughing)**

PP08: There is no any other thoughts (respondents laughing)

**I: Okay from all the things that we have mentioned that makes them in high chances of getting HIV, okay as from your own view Jitegemee if it will deal with HIV prevention do you see it as something that can help or it cannot help.**

PP08: What can it help?

PP03: It can help.

**I: It will help, number three, how?**

PP03: For help it can help because let me say that there is a partner who has HIV a times this Jitegemee can help you to get how you can protect yourself. They can also teach about HIV.

**I: So, like what do you think this Jitegemee can do so that it can reduces the chances at the sex workers that has joined they’re so that they should not get HIV.**

PP03: They can tell us how we can use PrEP I think it can protect even we can use contraceptives.

**I: Contraceptives, mmmh another thought number two what do you think Jitegemee can so that they can reduces the chances of getting HIV.**

PP02: Just as number three has said.

PP08: I have a question

**I: Mmmh**

PP08: Do you think women also have their condoms.

**I: Which people?**

PP08: Women.

PP04: They have

**I: Number 4 is answering say it.**

PP04: They have.

PP08: That is among the ones that can help.

**I: Those are the thoughts that we want so just say them.**

PP08: Condoms, prep that I have heard you saying and what else ‘’aboyunga’’

**I: “raboyunga” and condoms are one thing, isn’t it?**

R: Mmmh

PP08: Condoms for female.

**I: Okay when you say for girls.**

PP08: Condom

**I: Mmmh, females condom.**

PP08: Because of females I don’t know even how they look like.

**I: It is also a female condo, condom for female it is condom, yes, any other thoughts on how we can prevent HIV.**

PP08: I make sure he has worn it; you make sure he has worn it.

**I: Mmmh**

R: And when it burst?

PP08: He will not work; he will remove it and wear another one.

R: You are very keen so that you cannot be harmed my fellow.

PP08: Yes (respondents laughing)

**I: Number nine.**

PP09: Me as number nine, I see prep is good even condom is good but a times it can burst when you are there and the work continues, so you know prep can help you above all.

**I: So, this prep should be there.**

PP09: Yes

**I: Okay fine and now.**

PP08: I have a question; do you think when I have AIDs and I am a man no as in I am female, I have AIDs and am going to sleep with a person who doesn’t have it so how can you help me.

**I: Who can answer me there?**

PP01: Mmmh

**I: Number**

PP01: That is (inaudible content) I will answer you when I am her peer educator you have said that the partner has HIV.

PP08: When I am the one who have.

PP01: When you have it but your partner does not have it… you have heard something mention PrEP here.

PP08: Yes

PP01: When you don’t want to use condom, you can use it [PrEP] because condom is what you will use by that time, now when you want to go and you are HIV positive you want to visit someone the thing that you will use by that time is condom, do you hear me? And when you want to continue sitting there because you are people who have lived you will go and look for something that is prep.

PP08: He is a friend of mine from outside, I have it and I don’t want to disclose to that I have HIV because what I have said is difference from what you said. Am at sex work and I have AIDs I don’t have a friend who visits me does not have that disease.

PP01: Earlier you were saying that you are the one who have it.

PP08: Yes, I am the one who have it.

PP01: And the client does not have it.

PP08: Yes.

PP01: So, you want how you can help him.

PP08: How will I help him?

PP01: You will tell him the truth.

PP01: You will just trust.

**I: Okay you know that is another destruction we will talk about it later isn’t it.**

PP01: Yes

**I: We will look at it behind that white tent.**

PP08: As for me I just asked.

**I: Yes, there is no problem (respondents laughing) it is a good question so that we finish, I can see that we are already tired okay it is a good question because it is a question that helps us in our lives, isn’t it? So, I am asking so those people who have sex, the sex workers think that when should they stop sex. Do they think respondent seven? Do you have any thought?**

PP07: I cannot think maybe if I have become old.

**I: If you have become old.**

PP07: Mmmh.

**I: Mmmh, number four I can see you are laughing (respondents laughing)**

PP04: Me as number 4 there are sometimes that we always leave we get a break how I will say it because when you have given birth you cannot go to do sex work and then oldness there is a time that when you reach menopause you cannot do sex work so you it will force you to retire.

**I: Mmmh, thank you any other thought number two is there a certain time that sex workers think they stop sex work?**

PP08: (inaudible content)

**I: Number eight what are you saying?**

PP08: That thing looks like sweat (inaudible content)

PP01: I can add.

I: Number one?

PP01: I can say that maybe I am a girl and have joined sex work when I am not married and a certain man comes and he is a man who can take my challenge and he can also take a good care of me. You cannot… I will say that I will leave and I go to get married. Not everybody that is in sex work is married there are some who joined this work and they are not married. You will find others got married and it did not work out well. So they will say I just want to do sex work and with all the period of time you have not find a person, that now wants to marry her she will say that I want to do sex work, I want to settle.

I: Any other thought respondent four, there is no time that sex workers think that I want to stop this work?

PP04: They always think.

**I: Mmmh they always think what brings these thoughts?**

PP04: Where tired ness is there.

**I: Mmmmh**

PP04: Mmmh

**I: Tiredness according to what just explain to us a little so that we should move forward.**

PP04: Everything has an ending time you can I can just do sex work and at the end I just say that I see now it’s enough let me live it realize this so when you tell them not to. They will see that you are going this way and they go that way so you can say let me now live it for them so that they can also do it.

**I: So as for you personally have you think about it so that you can stop.**

PP04: For now, I cannot live it, with my age?

**I: Tell me**

PP04: With this age of mine, I cannot stop it maybe in future.

PP08: That thing looks like builders (respondents laugh)

**I: Number eight I am hearing you supporting that statement just say.**

PP08: I am just supporting that she should not stop it, she should stop it when she is.

R: What about you.

PP08: As for me know it is sweet it looks like ground nut (respondent laughing)

**I: Good it is good, that thought is what always comes to sex workers that now they should stop.**

PP08: Yes, everything has an end, starting a race has put you there so at the end it will just finish when you reach even sixty years or fifty years what are you still there you should stop.

R: At fifty is when you are active.

PP08: You don’t have many sexual urges you have them they end (inaudible content, respondent laughing)

**I: Okay fine number six tell us something there how do you see it, do you think sex workers always this that they will stop sex worker at which level, sex work.**

PP06: People always get tired.

**I: What brings this tiredness that makes a person to think that as for me now I am leaving this just say you have said people always get tired as for you have you ever thought that you should leave it?**

PP06: No

**I: You have not yet thought about it, why?**

PP06: I have not reached the age that I should stop.

**I: Like at what age can a person leave it, what do you think?**

R: Sixty years.

R: You might have many years and you still have energy, that you can still do with it sex worker.

**I: Mmmh ok number five how do you see it, do sex workers… is there a time that sex workers think they can leave sex work?**

PP06: Yes

**I: Mmmh, what brings this that makes them to think like that (noise at the background), what brings the thought.**

PP06: When you are tired.

**I: You have said tiredness, what brings the tiredness that can make a person thinks that now she should leave.**

PP06: What brings the tiredness maybe you are old.

**I: Mmmh maybe you are tired, what about you personally have you ever thought that you should leave it.**

PP06: Write now no.

**I: Mmmh, why have you said that you have not thought to leave?**

PP06: What has made me to say that way?

**I: Mmmh**

PP06: It is according to me with my age, and the way I am young I can’t leave it.

**I: (LAUGHS) Thank you, number three how do you see it, okay so I have come with a thought that you do sex workers always story tell, or they always story tell in general that we want to stop this work meaning what they do in sex work. Do sex workers sit down and story tell on what can make them to leave sex work number nine?**

PP09: Let me think.

**I: Number two it’s long since I heard your voice.**

PP02: You will just hear it.

**I: So give me your voice so that I should hear, do sex workers story tell like the time they can stop this work, things that can make them to leave.**

PP01: Me as number one sex workers always story tell a times number two is my friend and we work at different places so maybe she comes to me with other challenges one of them might be a person has had sex with her and did not pay her, so sometimes you here when she tells you that if I could be having something that I am doing I would leave this work because a times it depends with this and this so those things must be there.

**I: Okay any other thought number six is there stories that sometimes the sex workers story tell about sex work mmmh.**

PP06: Pass me first.

**I: I should pass you first, so I should go to who now number seven?**

PP07: Pass me.

**I: Number eight.**

PP08: The stories that the sex workers story tell truly we can story tell a times I have gone to work to my hustle another person com to me and tells me a story that I have gone with this person and I found a girl physically assaulted by weapons you know she will discourage me. I I was saying that if people are assaulted, I better leave it, I look for something that I can do. A times I personally I planned to go, I have dialed a number 07 and I found him ate this place and where we meet, he is with me another girl there will be a thunder, we will fight there. A times I have been physically assaulted or even I escape a knife when I return will I still go back so that is the story that we can tell that this sex work at times is hard so we should look for something that we can do.

**I: Mmmh.**

PP08: Yes

**I: Okay fine so what makes women who exchange sex money leave or might wish to leave the sex work according to what you have said I know you have said other things? What can make them have these thoughts of leaving sex number four?**

PP04: They might leave.

**I: That has made sex workers can think to leave sex work what is it?**

PP04: A times it depends with how you have walked in your life.

**I: When you say when they have walked means?**

PP04: A times when you have showered with sand, a times you can find that God has blessed you will find yourself saying to your heart that in this life I have run with this life, I should leave it I might change so one day you will story tell with your fellows that I did this thing and say that my fellows and a person can say that I have been drinking alcohol God has helped me to stop drinking it and I have stopped. Sometimes this work of ours a times you can go somewhere and say that eeh in this life I did this and this and God changed me and I stopped.

**I: Mmmh, thank you, any other thought, I am seeing that I have finished, I am finished it.**

PP01: Another thought.

**I: That is number one.**

PP01: Me as number one in age it will force you to retire even the challenges that you have been passing through at the sex work, the number three a times you want to settle you want to be called someone’s wife that one also.

**I: Is there any thought number three?**

PP03: Me as number three, it depends with the harassment that you have found a times a customer has come a times when a customer they only see you so your fellow sex workers will start saying that what has made a person of this numbers the one who is only seen. What has she done so they will start to harass you, so they will not need you there so you will look for a place a times something happened? You will say that at least I should leave it and start your life.

**I: Mmm, and so those people who were saying they want to leave they always leave a time that they have planned to leave.**

PP08: They leave slowly by slowly.

**I: Number 8?**

PP08: They leave slowly by slowly.

**I: They leave slowly by slowly what has made them not to leave the time that they are saying they want to leave?**

PP08: The time that I want to leave and I receive a phone call, I want you at this place how will I conduct myself, I will go, what I will find their times I have found her with a bra with a petty coat after that we will do one out of three and when I will go back, I will be discouraged and I will completely leave it.

**I: Mmmh any other thought do you think it is a thing that they always leave a time that they have planned to leave number five. When women let’s say sex workers have planned to leave sex work because we have said there are things that can make a person to leave isn’t it? So, when a person has planned to leave do they leave the time they have planned to leave?**

PP05: You cannot leave by the time because you have not found where you can stand firm so you know, they will leave it gradually by gradual when they think that when they leave what will they do. So, they cannot leave it by that time.

**I: They see that there is no any other way of earning.**

PP05: Mmmh

**I: Okay any other thought number two, I am finishing it, I am closing it now, I can leave it there (inaudible content) just give me your thoughts do women also think, the sex workers do they think that they should leave the sex work?**

PP02: Yes, thinking they are thinking but leaving it they cannot leave it. When you say that you want to leave it, I cannot leave it. When you say that you want to leave you will be disappointed slowly by slowly you will find that one day your heart will not regret.

**I: So number seven sex workers always leave sex work when they are how old?**

PP07: Sixty years.

**I: Mmmh, anybody else she has said sixty years.**

R: I think sex workers leave when they have reached menopause, as in when their menstruation period has stopped. So, you know by that time your breath.

**I: When she does not see menstruation period isn’t it?**

R: Yes, she will leave because she will feel nothing sex sweet when you.

PP08: When your body is hot.

**I: I have heard number eight supporting it that when your body is hot.**

PP04: What I know.

**I: Number four.**

PP04: What I know there when a woman who still see her menstruation periods don’t lie to us that you don’t have sex.

**I: Mmmh**

PP04: A woman who have her menstruation period I agree that woman who goes for menstruation periods now I am very sure that woman who sees her menstruation periods even if she is fifty years, she still has sex for the men is what I dint know which age he stops sex. Because an old man is still following a woman who is my size and still give birth with her. So, I don’t know a man stop having sex with a woman at what age?

**I: Okay let me say things about sex workers, m other question is what do women who leave sex work, what kind of job do they do after leaving sex work? Number one what do they always do?**

PP09: Repeat.

**I: What do women who exchange sex for money do after leaving sex work?**

PP09: It has evaporated.

**I: Number one.**

PP01: Number one?

**I: Yes, number one.**

PP01: I am saying when we started research with savings and loans you said that a times they can find money and save, another one said that a times they can build with it story building another one said she can save she will do with it a business so when you have decided that you’re leaving sex work when you don’t, you can find some people can find their own job, a person tells you that now I can give you a job of a nanny when you teach them even another job so when someone does sex work and save or keeps there is where her money can help her.

**I: So, she starts eating that money or?**

PP01: Now her money because these days she was not able to do business at the same time she goes for sex work. So, no she can choose a sardine’s business and do it.

**I: Thank you so some people when they leave sex work, they can decide that they can do sardine business isn’t it? Anything else that they can do?**

PP08: The business is deteriorating.

**I: Mmmh eight**

PP08: You can leave because at that time the market has deteriorated (inaudible content)

**I: So what can she do?**

PP08: She can meal, she can fetch firewood to make charcoal and she will find how she can help herself (inaudible content)

**I: Now I am not hearing what you have saying.**

PP08: I have said that by that time there is no market, because market also ends so when I save the period that I was getting money, when I don’t have money and in have some sucks I can meal and gives me a little money and by that time I can open another business so I can sit and sell so there is hope and there is no suffering. So, that can give you how you can feed.

**I: Thank you, number six which thought can you give us okay what you think and what you have seen those who have left sex worker which other type of work can they do that can buy?**

PP06: So, your money that you have been saving you can open with it a certain business.

**I: Other business like that you think they can open anybody else?**

PP06: Business like selling in stalls.

**I: Okay and which difference do they see in their life after they have left sex work. Which difference do sex workers have there any difference?**

PP08: It is there.

**I: Mmmh number eight.**

PP08: There is difference, this time I cannot beautify myself, you know I cannot beautify myself so that I cannot shower this time because what I was using that time, I cannot use it now because the level of life has decreased people always say that she is now tired, she is now going back where she came from so life must change the things that was eating those days it is different from now.

**I: Thank you, number one do you want to add something?**

PP01: I just wanted to say that life must change, a times it can change very well maybe it can change very bad. And when it changes badly maybe in a day you had good client you can live a good life, you can do your own things very well, you can pay your school fees very well and when a times you have retired in sex work and a times at one side it is good when you have left with other good things let’s say the sex work has a lot of clients they call you frequently. Client harasses you; they abuse you are a sex worker as in you pass through a lot of things at times people abuse you even in the society you are someone who has been branded. When you have retired you look like you are good.

**I: Mmmh thank you number three, I wanted to ask you that do you know anytime that the sex workers have left sex work and returned their again, she has returned again?**

PP03: A person who has left sex work, it depends with the challenge that you get outside here a times when you were there you were not saving or even a times you were saving and you used it badly you will go back to sex work to look for it so that you should be there that one only.

**I: Mmmh, number five add us something there is time that you have heard a person has left sex work and after some period of time they return?**

PP05: No, pas me a little.

**I: I should pass you a little.**

PP01: I was saying the time when you will stop it is when you have gone with a client condom burst you get a child. You will not do sex work with pregnancy you will be forced to sit down first until the child starts to walk is when you will go back to sex work.

**I: So, when a person sees a menstrual period she left, that one she has left or she has gone to rest. When we say a person who has left, we say she has stopped and then after a period of time they return.**

R: Oooh

**I: Number nine.**

PP09: Me as number nine I want to try explaining what number one has said you know getting pregnant it is something like an accident is it. When you wish to leave so you have gone to a person whom the condom busted you got pregnant there will you will be forced to leave because now you are pregnant you cannot do sex work even when you have given birth so when the child has grown you can again come back to it.

**I: That was for going to rest, is it?**

R: Yes.

**I: And when a person you will say that I have left this thing and later you will start seeing me coming back.**

R: You know you will have urges according to what you are doing, you have stopped using condom but according to what you have urge on that elderly is what making you leave you cannot wish to come back again.

**I: Number 9, number four you wanted to say something.**

PP04: As I said you can go back to it according to the life you are living when you are still there or when you have leave. So maybe the life that you have when you left it is difficult more than the life you had when you were still there so you will be forced to go back to the work that you were doing.

**I: What bad things always happen when a person returns, they have already left sex work then they return number seven. What bad things always happen to them?**

PP04: How?

**I: A person has left sex work she has been outside then after sometimes they return to sex work, what bad things always happen to them when someone has returned for sex work.**

PP04: I don’t know.

**I: You don’t know what might happen?**

PP08: (inaudible)

**I: Number 8.**

PP08: When I have already left sex work and I have completely left it, I have left to relax and I have started, when I have left you know my vagina has compressed and become tiny because when I was still sex work there is when you hear that a person has pain.

**I: A Person.**

PP08: There is what you here that a person is in pain, yes, because you found a snake (big penis). She will go and find a fat penis that is big and you know suffering is what has made her to go. You know when the client takes his penis and put it inside the girl’s vagina let’s say like they started around at 0900hrs until 0000hrs you know you can even find she has died because her vagina has already compressed like the one of a child so the client want to force his penis until it enters the vagina (laughs) she must see a problem.

**I: Thank you, what other bad problems always happen number six tell us you have never heard a thing that has happened, the bad thing that always happen, the bad things that always happen to those people who stopped sex work and they returned to it I am finishing, the bad things that always happen to those who have left and returned when they have returned number 2, number six is still thinking number two tell us what bad thing always happen to them.**

PP02: The bad thing that happen to them.

**I: Mmmh**

PP02: It is (silence)

**I: The bad things that always happen when we talk it’s just two minutes.**

PP08: Let me finish for you I am hungry.

**I: Mmmh**

PP08: First of all I have gone there and found another woman you will be physically harmed until you die.

**I: Mmmh**

PP08: You will be physically harmed you will die, you will be harassed that the problem you have had sex with a person and has not given you the money you have returned with nothing you can die on work according to the year.

**I: Mmmh**

PP08: So that is the problem now we have finished (respondent laughing)

**I: Thank you what good things happen to them number four, what good things always happens to them when they go back for sex work, a person who has left sex work and returned again what good things always happen to them when they have returned.**

R: Sex work is sweet.

**I: Mmmh number four, number three do you have something to say?**

PP03: Me as number three I think when you have left when you return you return when you have more sexual urge the clients will be after you, you will hear the clients saying go and try this number she is hot and you know the one that enters, enters a lot because now you are like a visitor and a customer knows that a visitor that they had not had sex with.

**I: Mmmh, any other thought number five what good things always happen to them, those people who leave sex work and returns?**

R: Mmmh

**I: There is none and mmmh, okay what do sex workers, a person can wish to leave sex work what do always want to achieve they want to do it first before they leave sex work number nine?**

PP09: Me as number nine when I want to leave prostitution, I must know the amount of money that I have.

**I: Sex work.**

PP09: So that when I relax, I know I will feed even if I am not going for sex work.

**I: Mmmh anybody else who can give their thoughts number two give us your thoughts what do sex workers love that they should do first before they leave sex work.**

PP02: Before they leave.

**I: Mmmh**

PP02: It is a must that you should look for what you can do.

**I: Mmmh like what?**

PP02: A business that you can do, before you do sex work a times you can live and you have no stable business so you will be forced to look for what you can do first.

**I: Mmmh, okay they should look for a business that they can do.**

PP02: Yes they should look for another business that they should do.

**I: Mmmh number six what do sex workers want to do first before they stop sex work.**

R: What do am hungry.

**I: As for me I am not hungry.**

PP02: You see when (inaudible) when she has stopped sex work as in I have this amount of money I can open with it this business or even another work that I can do.

**I: Mmmh we must have a certain work or business, which type of business.**

PP02: A business of selling clothes it might be that, your money can reach the amount that she can go and buy them the clothes and sell them there is a profit that they can get and buy with it another clothes.

**I: Okay is there any other thought and do you know sex workers that has left sex work for five or ten years ago is there anyone who know them?**

PP08: I can know them.

**I: Number eight.**

PP08: Should I mention their name?

**I: No, just number is there anyone who know them?**

PP08: Yes

R: Their number

PP08: You know (inaudible)

**I: Is there the ones that we know who left ten years or even five years ago is there anyone that you know.**

PP08: The ones that go and return.

**I: The ones that leave and return we have already said that they can leave and again return, the ones that has leave do you know the number?**

R: The one who has leave has already died.

**I: The one who has leave are those who have died?**

R: Mmmh (respondent laughing)

**I: Anybody else number five is there anyone that we know who has left five or ten years ago?**

PP05: I don’t know anyone.

**I: Number four do you know anyone who left?**

PP04: Five to ten years ago?

**I: Yes five to ten years ago.**

R: Those are people who have already died.

PP04: Three years and two years are there but five it is difficult.

**I: Two to three years ago these are people who, what difficulties has made them to stop.**

R: (INAUDIBLE)

**I: Mmmmh**

R: They got married.

**I: They found people who married them mmh number three is there any one that you know that has stopped sex work five to ten years ago?**

PP03: Those who have already left.

**I: Mmmh**

PP03: Those who have stopped I don’t know them.

**I: There is none, you don’t know them?**

PP03: I only know those who are still there.

**I: You only know those who are still there number seven what about you, is there anyone that you know?**

PP07: No.

**I: We don’t know anyone, okay that is the end of the questions that I had today. So, I don’t know if there is a thought that you want to give us you will ask questions later, thoughts that you want to give us before we start the research. Any other thought we have already finished. There is none, okay if there is no any other thought I am grateful for your time so that we can be together in these today’s session and we have hope that the thoughts that you have given us will help at least when we put them together with the other one that other people has given us it will help us to build this research so that we should love it because it will help us so that we can love it and it should be something that will work out.**

R: Mmmh

**I: Okay thank you we have finished our session at 02:47**

**END**
