# Supplementary material for: Eat a little and save a little: A qualitative exploration of acceptability of a potential savings intervention to reduce HIV risk among female sex workers in Western Kenya
Source: PLoS One. 2024 Dec 19;19(12):e0310540. doi: 10.1371/journal.pone.0310540 (PMC11658496; doi:10.1371/journal.pone.0310540)
Supplement: S1 File — (ZIP) [file pone.0310540.s001.zip › Jitegemee Transcripts and Dissemination Notes for Journal/FGD Q.docx]

**INTERVIEWER’S NAME: JUDITH**

**NOTE TAKER’S NAME: PHILIP**

**FGD ID: FGD Q**

**NAME OF TRANSCRIBER: ESTHER OKOTH**

**CATEGORY: BELOW 30 YEARS, RURAL.**

**I: Okay, it’s okay (baby crying in the background), okay, this is FGD Q, the interviewer is Judith and the note taker is Philip. The discussion is being conducted at Lwak centre. It’s okay, welcome all, following how we’ve talked about Jitegemee in short what comes into your mind right now, (silence) yes no 3**

PQ03: Thank you, how we started and this far we’ve reached. What has come into my mind is just joy because I feel that if we join this research deeply we will receive help and will know how we can save our own money so that we can use them and leave our current ways of earning which is sex work.

**I: Mmh that is participant number 3, any other person what comes into your mind about Jitegemee? (Silence)**

PQ05: thank you very much

**I: Participant number 5**

PQ05: Thank you very much for today, that you decided to come and visit us following how we started… according to how Jitegemee is, we should not focus so much in sex work. But we should also find something to do like business. This will also help us to change our thoughts about sex work.

**I: Thank you very much, don’t forget about saying your numbers whenever you want to talk**

All: Yes

**I: Any other opinion about Jitegemee?**

PQ09: Okay, as number 9, following how we started with the facilitator who has shared with us briefly. It has enlightened me that I should have a business running that when I don’t go for sex work, I can sell or operate it and get some money to use even if I don’t do sex work.

**I: Thank you very much number 9, any other opinion about Jitegemee, yes number 6**

PQ06: I as number 6, what I have gained in the teaching about Jitegemee, I have realized that I should always save some money, so that when I don’t go for work I can support myself with instead of depending on sex work only.

**I: Mmh that is number 6, eeh. Yes number 1, your thoughts about Jitegemee following how we have discussed,**

PQ01: As number one, I only want to thank you for today. How we have sat here and you helped us learn about saving. We can save so that we stop doing some things that can destroy our lives

**I: Mmh, Okay thank you very much number 1, Yes number 8, tell us more about Jitegemee**

PQ08: As number 8, what I think about the teaching, Is that I can save something small [some money] than depending on sex work. Yes, that’s my opinion.

**I: Okay,**

PQ07: Yes, as number 7, I want to thank you so much for the good teaching you have taught us about how we can take care of ourselves by having two alternatives, if I don’t go for sex work I can still save some money for my tomorrow.

**I: Mmh, thank you, number 7, yes number 4**

PQ04: As number 4, I was feeling that we need to work hard rather than depending on sex work to earn.

**I: Okay, thank you very much. And what are the items that sex workers buy, and how much do they each of them cost? The things that ladies like buying daily, what do they buy daily? What do they buy with their money? Yes number 5**

PQ05: As number 5, the things ladies like buying with their money. A lady wants that when she has money she has to keep her body neat. She should be clean and also eat well

**I: Does she do that daily or?**

PQ05: Anytime she gets money she has to keep her body neat so that she continue attracting people

**I: That’s daily?**

PQ05: Yeah, she wants to be neat.

**I: The things they buy daily, yes number 7**

PQ07: If she gets money she wants to buy clothes, shoes, make her hair and to be clean

**I: These are the things she buy daily**

PQ07: Yes, if she gets money

**I: Okay, and if she doesn’t get money**

PQ07: She can only buy food for her consumption.

**I: Mmh. Only food? If you buy food, how much do you use daily?**

PQ07: You can only buy for 200/=

**I: For 200/=, that’s daily on food?**

PQ07: Yes

**I: Mmh, any other thing that they buy daily, yes number 9**

PQ09: Okay for me as a sex worker what I love buying when I have money

**I: Mmh**

PQ09: Pants

ALL :( laughs)

PQ09e: Because if I go with this one, and then again go with this other one. Now when i am coming back to this other one, I don’t want his to see the same white pant I wore the other time, so for me I buy pants and also food.

**I: So do you buy pants daily?**

PQ09: Yes, I buy pants daily, if I want to leave home I buy

**I: So you buy pants at what price daily?**

PQ09: Pants?

**I: Yes**

PQ09: I buy for 100/= I don’t buy expensive pants because I only wear it once. If I am to go the other side the following day then I wear a different one.

**I: That’s daily**

PQ09: Yes

**I: How much do you spend on food daily?**

PQ09: I also need to feed well because I don’t want to lose weight (laughs)

**I: How much do you spend on the good food?**

PQ09: If I buy food for morning (breakfast) to evening (dinner) I can use 500/= now that life is very expensive.

**I: When you talk of good food, what type of food are they?**

PQ09: Meat, Fish (laughs)

All :( laughs)

**I: Okay, number 2**

PQ02: As number 2, as sex workers we use a lot of money. You can… If you get money, you want to buy clothes, buy food and if it reaches evening when you should meet your client you don’t have money and you need to buy Trust (condom) because you cannot be sure if the person you are meeting also bought Trust. So you have to buy yours to be safe, so we use a lot of money, sometimes the money you used in buying trust the person you met didn’t refund you back or the money he paid is less than what you spent. So we have to work hard. If people meet this way, it will help us because if we save and we will be able to have some money that we can use in buying some other things. Because if we use a lot of money to attract those people [clients] and you find that at the end you can’t repair/furnish the place where you live because you are using a lot of money to attract clients.

**I: So, that’s the money you use for buying clothes and food daily?**

PQ02: Yes, I have to buy them because I don’t have someone who can buy them for me. I have to spend my own money

**I: So if you buy clothes, how much can you spend daily on clothes?**

PQ02: For clothes, I only buy them when I get money like maybe once in a month but for food I have to buy daily,

**I: Like how much do you use in buying food daily? Or you are also feeding well like number 9**

ALL :( Laughs)

PQ02:(Laughs)no I cannot feed well daily, sometimes today I can feed well then tomorrow I take vegetables, I balance them that way because you cannot have money daily

**I: Okay**

PQ02: Yes

**I: So if you approximate how much do you spend on food daily**

PQ02: I spend approximately 400/= daily

**I: Eeh. That’s on food**

PQ02: Yes

**I: Mmh, any other person (silence) what do ladies buy daily?**

PQ03:Thank you, me as number 3, the things we buy daily are like food ,and again you won’t just be sited in the house hoping that you will receive a phone call. Somebody will call for you go with him, you must have some money that you can use to buy a bottle of soda and drink as you think of what next

**I: Mmh**

PQ03: Yes

**I: So when you buy food, how much do you use daily?**

PQ03: Like right now life is very expensive I can use approximately 350/= to 400/=. But for the drink if the income is low you can buy a bottle of soda worth 50/= but when you have money you can buy Guinness (beer) worth 200/= it depends.

**I: So soda or genius is it daily or?**

PQ03: Soda you buy mostly daily because in the evening you won’t just sit there as you wait for the clients. You must buy for yourself first

**I: Ooh, when you are waiting for the client, so that’s daily, but how about Guinness?**

PQ03: If you expect that the client you are meeting is going to pay you well, because most of them don’t pay well: So you can strain to buy Guinness and he only pay you 200/= and may be in your house you also left nothing, so it depends

**I: Mmh, weekly or monthly**

PQ03: Weekly

**I: at least in a week you buy some Guinness?**

PQ03: Yes

**I: Any other thing that you buy daily?**

PQ01: As number 1,

**I: Yes number 1.**

PQ01: I can be very happy to eat some nice meal when I get money. If I get a client who is atleast pays me good money. You can find one that do not give you anything substantial. So if I get a client who gives 1000/=, then the next day I can treat myself by buying myself chicken for the work I did. That I have to eat well.

**I: How many times do you treat yourself, do you do it daily or how frequently?**

PQ01: I can treat myself after 4 days

**I: Meaning in a week you can treat yourself**

PQ01: Yes

**I: And how much do you spend daily?**

PQ01: Daily I can use roughly 250/= that is breakfast. Lunch and breakfast roughly 300/=then supper 200 so it’s roughly 550/=in a day

**I: Every day 550/=?**

PQ01: Yes

**I: Thank you, another opinion? In a week?**

P: Another opinion is here?

**I: Eeh…**

P: A lady must have airtime that she can use to call her clients

ALL :( Laughs)

P: If you don’t have airtime in your phone, how will you call [name of client mentioned]. Because sometimes we are forced to look for them by ourselves when things are hard. Because I will not wait for him to look for me, I will have to call him. And for sure someone you love you have to call him sometimes, I must have some airtime.

**I: So how much credit do you load in your phone daily?**

P: I can load airtime worth 100-200 daily.

**I: Okay, so you have to ensure daily that you have credit of 100/= to 200/=**

P: Because I have to chat/text with him, when he replies I will inform him and he says, “I sending you some credit.” and he will add me more Yes. I will continue sending text until I get some clients.

**I: It’s okay, and in a week, what is it that ladies cannot miss buying with the money they have before the week ends? You have heard of participant number 1 who said she cannot take a week before treating herself with chicken is that right?**

ALL: [Laughs]

**I: Eeh apart from chicken**

PQ03: Every week I have to buy… You remember the clothes we talked about every week, because you cannot put on the clothes you wore last week again. You must keep some money so that you can buy a nice clothe. You know these people buy clothes every week.

**I: Mmh, clothes worth how much?**

PQ03: You will find it is a nice cloth and the seller agrees to give you at 500 or another offered another one at 1000 shillings

**I: So it begins from 500/=**

PQ03: Yes

**I: Okay thank you PQ03. What is it that ladies do not fail to buy before the end of every week?**

PQ01: As number one I cannot end the week before I change my hairstyle.

**I: Like how much do you spend on your hair?**

PQ01: I change hairstyle with 500 shillings weekly

**I: 500/= that’s for hair, Okay, yes, number 8**

PQ08: A week cannot end before I purchase a cloth and make up kit. Because you have to look beautiful you don’t just go looking ugly

**I: Mmh, so how much do you spend on clothes weekly?**

PQ08: In a week? It’s like 500/=

**I: Around 500/=, how about make up kit, how much?**

PQ08: Make up kit, like 600/= because they are variety

**I: Okay**

PQ08: Yes

**I: So in a week 600 goes for beauty and 500 on clothes**

PQ08: Yes

**I: Any other person, in a week what is it that ladies can’t miss buying in a week?**

PQ07: Shoes

**I: That is participant number 7,**

PQ07: I have to put on nice shoes

**I: How much are the shoes**

PQ07: From 500/=, and also clothes that you will put on

**I: How much are the clothes?**

PQ07:700 Or 850

**I: What else**

PQ07: Earrings also.

**I: How much are the earrings?**

PQ07: 150 [Laughs]

**I: So in a week at least 1000 to your body?**

PQ07: Yes

**I: Mmh that is number 7, any other opinion weekly? Yes,**

PQ05: As number 5

**I: Yes number 5**

PQ05: In a week I have to copy new walking style and how to smile also change of voice to attract costumers so that when I’m at =ndori= I have a new style

**I: Mmh**

PQ05: When I reach =ajigo=, I have another new style how I carry my self-changes weekly depending with places I visit.

**I: So meaning you use transport when going to these places?**

PQ05: You cannot walk to these places my friend .You will reach your destination when you are dusty and you should reach when you are clean. You in these places where we visit, like you go to a bar then some men don’t like ladies who are not settling down. But with you are seated somewhere and the guy decides to signal you to join him, I have also realized that this is a good thing where we work.

**I: Mmh, so for transport how much do you use weekly?**

PQ05: I use like 100 to =Ajigo= then to =Tin toler= I use 150.

**I: Mmh, that’s for a week, and do you visit different places in a week or if this week you go to =Ajigo= and then next week you go to =Tin to Ler=**

PQ05: I go to different places when I’m looking for greener pasture

**I: Daily you go to different places or?**

PQ05: I don’t maintain one place.

**I: So by the end of the week you use like how much on transport?**

PQ05: I go looking for a client there I can use like 300. Maybe a client called me there and I get the opportunity to get another one there depending on high I carry myself.

**I: It’s okay and now in a month, what is it that the ladies cannot fail to buy before the end of the month?**

PQ03: I cannot end a month without buying sanitary towel

**I: Sanitary towel**

PQ03: Yes, that I can use

**I: What amount is the sanitary towel?**

PQ03: 200/=

**I: 200/= yes, number 9**

PQ09: A month can’t pass without buying something to pleat my hair

**I: What?**

PQ09: What I can use to pleat my hair?

**I: How much do you spend?**

PQ09:1500

**I: That is monthly, that is PQ09, PQ02**

PQ02: I pleat my hair monthly and I buy lotions because if you use milking Jelly no one will see you. I you use the lotion your face becomes a bit soft. If you calculate you realize you use thousands.

**I: How much do you spend?**

PQ02: About 1000, currently one thousand will only give you a simple style, and also for the lotion you will spend up to 800 shillings to get quality lotion.

**I: Another thing in a month, PQ03**

PQ03: You have to pay rent, you can’t live in someone’s house and you fail to pay, you might come back and find the door closed.

**I: How much is your rent?**

PQ03: 1300-3000.

PQ05: I don’t end a month without asking for money from a boyfriend that is something I keep in my mind I have to?

**I: That is money you request from him, how about your own money that you use?**

PQ05: My own money I can use like 2000/=in a month because I struggle to get it it’s not easy, but the one I request for I know it’s easy to get

**I: So what do you buy with the 2000/=**

PQ05: If I’m given 2000/=? I buy myself things and buy things in the house to make it look beautiful

**I: The 2000/=from your own pocket**

PQ05: Yes, that’s 2000/= from my own pocket

**I: Okay, how about in a year? (laughs)what is it that ladies cannot miss to buy in a year we have seen those who buy clothes daily, food and those are done daily, though rent is paid monthly, those who buy clothes ,make their hair and buy shoes those are normal things ,so by the end of the year what does some people do?**

PQ03: I cannot end a year before I get a new boyfriend

**I: How do you use money, when changing boyfriend**

PQ03: My own money?

**I: I want something that you have to do with your own money**

**NT: …continues as an interviewer) what you have to do with the money that you get yearly?**

PQ03: With the money I get?

**I: Yes, yearly**

P: like for me I have a child who I have to ensure that he goes to school, he bath, feeds and even me I have to ensure that my personal needs are taken care of.

**I: Mmh, so on average how much do you use in a year?**

PQ03: In a whole year (laughs) money is used so much

**I: Just one thing that you think you used money on that is different with other normal days.**

PQ03: So for example like my child is going to school and he also bath that can be 20,000 in a year

**I: In a year**

P: Yes

**I: Okay**

PQ01: As for me as number 1, I have to ensure that before the year ends I have to buy something with my own money that I can see by the end of the year

**I: Mmh**

P: I can buy fridge that I can say this year I bought a fridge.

**I: So if you think of buying fridge, what amount you will spend on that**

P: I’m thinking of buying a fridge of 40000/=

**I: Okay, yes number 2**

PQ02:As for me number 2, like for us women who are hardworking I ensure that in a year I keep money which I use in paying school fee because he is in private school and so I have to pay school fees and you also know how expensive private school is?

**I: Mmh**

PQ02: So after paying the school fees is when I can say that the remaining things will be done bit by bit. Because school fees is something you can pay once in a year so even if you are focusing on your beauty you also need to look for his schooling very well

**I: Mmh, how much is school fee?**

PQ02: It’s huge amount of money in a year

**I: Yes, in a year**

PQ02: Its roughly 15k

**I: So you save it bit by bit?**

PQ02: I do save bit by bit, so at the end of the year I pay it ones

**I: Okay, yes number 6**

PQ06:As for me as number 6 ,I have to ensure that the children I have put on nice clothes that when Christmas is being celebrated they are also looking good and also I have to ensure that I save something good.

**I: So if you buy clothes for Christmas celebration how much can you use?**

PQ06: I can use like 7000/=

**I: 7000/=in a year**

PQ06: Yes

**I: Okay, thank you all, most of the women who are sex workers normally get that they use from where?**

P: Kindly repeat the question?

**I: Where do most sex workers get the money they use from? The money they use? Where does it come from? Yes number 3.**

PQ03: As number 3,

**I: Mmh**

PQ03: The money we use we get from those who we have sex with.

**I: That is the real partner.**

PQ03: Yes

**I: That is number 3**

PQ01: As number 1, the money we use we get from those who we have sex with

**I: That is number 1, any other different idea? We all get money from those we have sex with**

ALL: Yes

**I: Okay, but apart from clients where else can we get money from? Yes number 5**

PQ05: As number five the money I save I get from business that I do

**I: Businesses**

PQ03: As number 3 I’m supporting what number 5 has just said

**I: That is business**

PQ05: Yes

**I: Apart from business where else do we get money from? (Silence) say it loud number 6**

PQ06: As number 6, you have to get it from your business, it can’t come from any other place.

**I: So it’s just business no one have a different idea apart from business (laughs). it’s okay and if you are getting it from business then how much can you get from business? Participant number 2**

PQ02: It depends with an individual’s business, we don’t get same amount?

**I: Eeh**

PQ02; so it depends with the business someone is doing, you can do different business with that of number 6 and maybe she is getting a higher and I’m getting lower

**I: Eeh, so for you on average how much can you get?**

PQ02: (Laughs) so for me I do my totals every week not daily

**I: Mmh**

PQ02: So in a week if sales are good, because there are times when sales are not good. So, when sales are good, because also it cannot be good all the time or bad all the time so the medium amount I can save in a week with good or bad sales is roughly 3000/= besides capital for adding more stocks

**I: So the profit is 3000/=**

PQ02: Yes

**I: Any other person with a different opinion with that of participant 2**

PQ01:As number 1,I’m selling maize and when the sales are good in a day I can sell one sack of maize but when there’s poor sales I can only sell 3-4 tins of 2kg ,so whenever I sell I have to save like 100/=

**I: Okay, now if you do your calculations how much do you save?**

PQ01: The amount I get?

**I: Yes if you do your totals?**

PQ01: It’s around 4000/=

**I: That is in a week or a month**

PQ01: In a month

**I: Okay, any other person? (Silence) there’s none**

ALL: Yes

**I: And why is it that most of the sex workers use their money in buying the things they use on themselves? Those things that we had mentioned earlier like buying clothes, shoes, make up and food we have talked about those why would ladies use their money in these things? Yes number 7**

PQ07: As number 7

**I: Mmh…**

PQ07: They are using money to buy these things to look neat so that someone can see her

**I: Mmh, so that applies when she buys what?**

PQ07: If she buys oil and apply and also make up kit, clothes and shoes, she buys these things to make her look neat to attract men

**I: Okay, yes number 9**

PQ09: As number 9, we are sex workers and we engage in sex with different men we have known their minds because you can find that you are ugly but how you have dressed is what will attract him

**I: Okay**

PQ09: Yes and sometimes you were not blessed with behinds [you don’t have buttocks] so the kind of necklace, earrings and hairstyle is what will make him get attracted to you and he will come to you

**I: Thank you, yes number 4 why do they use money to buy the things they buy for themselves?**

PQ04: For them to be smart

**I: Mmh…**

PQ04: When they go to boyfriend you have to look smart

**I: Okay, and do sex workers save? Number 7 is saying yes**

PQ07: Yes, we have to save

**I: Do they save?**

ALL: Yes

**I: Why do they save? Yes number 3**

PQ03: We have to save because, me as number 3 because these people we meet they give us money but they will not give use money daily so we are saving so that if you lack tomorrow at least you have something if you get 1000/= you save 500/=and this will make our lives to continue even if we lack in future

**I: Thank you, number 3, and any other thing why we are saving?**

PQ05: As number 5

**I: Yes, number 5**

PQ05: The reason why we are saving is that when we go to sex work clients, we go there with a target you cannot just go there without a target so when you are given something you use five and keep five for buying groundnuts that you sell

**I: So the 500/= is for business**

PQ05: Yes, that’s the groundnuts I sell

**I: Okay, any other reason why they save? Yes number 2**

PQ02: As number 2, the reason why we have to save

**I: Mmh**

PQ02: It’s only that we are saving these money but they are not of help to us, because sometimes you get client then you carry all these savings you have because you cannot walk without money because you can receive phone call anytime because that’s the job you are doing.

**I: Mmh**

PQ02: So you have to use your own money as fare, you cannot tell him to send you fare because he won’t send. So, you have also to have some money to buy something to drink before he comes. So you have to use your own money because sometimes also the clients promise you to send you money later and getting transport to come back home becomes a problem. So, you have to use the money you saved

**I: So meaning you save so that when you are needed you can be able to avail yourself.**

PQ02: Yes

PQ01: As number 1

**I: Mmh**

PQ01: I have to save because, sometimes you can go to where you were supposed to meet with your client then he decides to switch off the phone and sometimes you have drunk like 3 bottles of Guinness so you know if you don’t pay for the bottles of Guinness you will be given work in that bar to do in return of your bill.

**I: Mmh**

PQ01: You have to save some little amount

**I: Mmh**

PQ09: For me as number 9,

**I: Mmh**

PQ09: The reason why I am saving is because some of these men after you have sex with them

**I: Mmh**

PQ09: Then you had agreed in payment

**I: Mmh**

PQ09: But ones you are done doing sex, he says that he has no money, that he will send via Mpesa then again we have to save because when you are with these men and you share with them about the idea of starting a business they won’t support you ,and that can bring disagreement

**I: Mmh**

PQ09: They are just people who want to waste your time with 500/=or 200/= and that’s all so we have noticed that. So the little that you get if you are given even 1000/= you can use 800/= if you had nothing in the house then you save 200/=. You just start your business alone and you don’t share with him the idea that you want to start a business let’s say for example for selling chips that you were requesting 2000/=, that one you will spoil the relationship

**I: Mmh**

PQ09: They don’t want to lift you

**I: Thank you, it’s okay and when saving how many times do you save? In a week how many times can you save?**

PQ01: As for me as number 1 I am a business lady,

**I: Mmh**

PQ01: In a day I ensure I save 50/= for a whole week

**I: Yes, that is number 1, yes number 9**

PQ09:After doing daily budget and I have remained with 60 or 20 that is after buying even the next day breakfast I can keep it somewhere so in a week you find that I have like 380/= or 200/= or 400/= it depends with how the week was.

**I: Mmh, so you budget the balance you keep**

PQ09: Yes

**I: Any other person, there is none, okay and what are the characteristics of women who save, that if you look at them you will know that this one saves?**

PQ02: Women who save they have to be those that denies themselves, sometimes it might be tough but you have to deny yourself to save you cannot be spending the same way daily. Because maybe it rained and sales are not good then you only manage to sell amount of 100/= and you have to save 50/= you will have to eat vegetables for you to save the 50/=.So you have to be mean to yourself sometimes

**I: So women who save are mean to themselves**

PQ02: Yes

I: So women who save have a habit of denying themselves.

PQ02: Yes

P: Again they also have a habit of not going beyond their budget, for example if she wanted to buy omena and she has 50 shillings, and she finds that the price of omena is higher like maybe 70/= or 80/= she will not buy omena but instead will buy vegetable within her budget

I: So she has a budget that she follows

P: Yes, so that her calculations goes right

I: Any other character, Yes number 8

PQ08: As for me as number 8, I have same thoughts as number 2

I: Mmh, in which way?

PQ08: They deny themselves.

I: Okay, thank you, any other person idea about the characters of women who are saving?

PQ01: As for me as participant 1 women who are saving are women who are strong

I: Strong in which way?

PQ01: They know how life is

I: How life is, in which way

PQ01: They know how they can save, deny themselves so that the little they save can be used in future when things are tough

I: Okay

PQ01: Yes

I: And how about the behaviours of those women who are not saving?

ALL: (Laughs)

I: Yes say it louder, that is participant number 1

PQ01: Those that don’t save they just spend and they buy anything when they get money they go buy chicken, meat just like how number 9 loves meat (laughs)she doesn’t think of tomorrow and she treats herself every time(laughs)

I: Any other habit?

PQ05: For me as number 5, a woman who save doesn’t joke around, she is serious with what she does she cannot misuse money, her money has a target.

I: That is for a woman that saves?

PQ05: Yes, that is for a woman that saves

I: How about the one who doesn’t save? P3

PQ03: As number 3 those women who doesn’t save you find that when they have emergencies they don’t have money to use and they don’t have anything to put their hands on.

I: Eeh. So they don’t know where to start, any other yes number 9

PQ09: A woman who doesn’t save you can easily identify her, because even when she is just walking around and may she sees fish mongers coming in with fish she will ask the price of fish and when told its 300/=immediately she says I need two. She doesn’t know how she is going to fry them what she knows is that she has bought fish

**I: Okay**

PQ09:Again she sees a dress she ask the price of the dress without knowing if the dress will fit her or not she buys dress at 1200/=she does not have a target. Even if she has 1000/= she can use it within 2 hours and it’s over. So if you try asking her about the 2000/= how she has spent it she will say she bought fish and a dress that is not worth the money. Just as participant 3 had said earlier, when she get problem she will ran to you for help yet you had been denying yourself in that when you see fish you say, “I had budgeted for omena, let me just take that.” So they are such kind of women.

**I: Okay, and we had talked about women who can save, is that right?**

P: Yes

**I: What makes it easier for women who save to save? (Baby shouting in the background) What makes it easy for them to save? Is there anything that encourages them to save? Yes number 9**

PQ09:As for me as number 9 what encourages me to save is that just as I had said earlier when I have a kid who is going to school. I have to prepare everything for him for the whole year all that are needed in school. I have to use about 20,000/=and pay him school fee. So, when I save for one year and I saw its benefit, the next year I didn’t hustle for money. This gives me courage me to save so that I can also do other things.

**I: Mmh, Okay any other opinion, what makes gives these women the courage to save**

PQ05: As for me as number 5

**I: Yes number 5**

PQ05: What gives women courage to continue saving is because if you save you see the benefits of saving but if you don’t save you will find theirs a big gap? So when you save it enables you to do something, you will have hope and be encouraged to do it again so that you can sort out another thing.

**I: Are there difficulties they encountered when saving? Any difficulty or challenges that they encounter when saving, yes number 5**

PQ05: Yes there are, I have saved and the challenge I had was that I had some tin where I was saving and somebody got where I kept it. Somebody found the home bank and took all my savings and that was the big challenge I encountered. So nowadays I save on phone [baby playing in the background].

**I: Ooh so you sorted it out through saving on phone**

PQ05: Yes

**I: Yes, number 9**

PQ09: As number 9, I also use to save in the home bank they are talking about. So one day when I went out I kept money in my purse, I kept the money in the purse so that after I am done with my partner then I can go shopping. So, when I went for a short call and left my purse with him I realised he had taken all my money and had left and that day I learnt that when I am going to see a boyfriend I only carry little money or if I have to carry huge amount I have to keep it in a different purse which I can keep on my chest where he can’t see or I will rather keep in my phone. So, that is the challenge I got with savings, it taught me a lot.

**I: So nowadays you don’t carry cash**

PQ05: I don’t

**I: So that is the way you handled that challenge**

PQ03: Yes

**I: Okay, any other challenge,**

PQ07: As number 7 the challenge I can find when saving is that let’s say for example you have 2000/=. Sometimes you don’t have what to eat for supper or lunch and maybe you have saved 2000 and you are looking forward to save 5000. I may not touch it because the time may be nearing and I am still far from the target. I will just not touch it so that I reach the target I would rather sleep hungry.

**I: Mmh, and now how have you solved the challenge so that you don’t miss a meal.**

PQ07: So the following day even if I get 50/= I can buy food with 30/=then save 20/=

**I: Okay, mmh… number 2**

PQ02: As number 2, saving is very good, there was a time I was saving, just as it’s not easy keeping money in the house, so I kept my money on phone, so I was with my client in a room then I excused myself and left him with my phone. As you know someone you are close with you can allow him to access your phone anytime. I left my phone there, so he had checked my Mpesa and confirmed the balance then that’s the time he tells you about a problem that he have and needs your support financially, I didn’t know he had seen the balance. “I have a problem and if you have some money, help me and I will refund the next week.” being that you love him you will just give him. There is a time I gave someone 3000/= out of the 5000/= I had and I was left with 2000/= and he promised me that he was going to refund the money before the end of the week. When I needed the money I could not reach him on phone and when you talk about money again it brings disagreement and that made me learn that I should not leave anyone with my phone and I will never trust anyone again and that money was never refunded. I realised that it took me back and I said, “You can make someone to be too close to you thinking he can’t do anything bad to you yet he has his bad thoughts.” So we relate to these people but sometimes they hurt us. . You have money that you can use on yourself but he decides to hurt you on one side. So that was the challenge I encountered

**I: How did you overcome that challenge?**

PQ02: I tried to follow up for the refund but it was not easy so I decided to leave him that money. I just let it go

**I: So what are you doing right now to avoid such in future?**

PQ02: Nobody get access to my phone

**I: Ooh**

PQ02: Yes

**I: Any other challenge? There is none and what are the disadvantages of not saving? Yes number 7**

PQ07: As number 7, someone who have not been saving may see something good but will not be able to buy it because she has not been saving. But if you save you can see a good thing and go for your money and buy.

**I: Mmh… that it disadvantage of not saving is that right?**

PQ07: Yes

**I: Any other disadvantage of not saving? Yes number 3**

PQ03: As number 3 the disadvantage of not saving is that if you have an emergency there is no way you can solve it

**I: Mmh, so for those who do not have the disadvantage should tell me the advantages oooh (laughs) you still have disadvantages**

All: Yes

PQ07: You can fail to save, then your partner take you somewhere then he fail to pay bills for the meals you take the money you saved and to pay the bills so that you are set free.

**I: Mmh, thank you number 7, for those who are quiet any opinion on the disadvantages of saving? (Laughs) the advantages of not saving**

PQ04: If you don’t save you can see a good thing but may not be able to buy it.

**I: Is that an advantage or a disadvantage.**

PQ04: Disadvantage.

**I: Another disadvantage?**

PD08: Another disadvantage

**I: Yes, for not saving.**

PQ08: The disadvantage of not saving is that you can get an emergency and you may not have a place to get some money that is the disadvantage of not saving.

**I: P6 Advantage of saving?**

PQ06: The disadvantage of not saving is that sometimes you have a child in boarding school and she can call you that she needs pocket money and you have not been saving. If you have been saving you can send him/her so fast.

**I: Is there any advantage of not saving?**

PQ03: As number 3 there is no advantages of not saving

**I: There’s no advantage? Okay that is opinion for number 3, meaning other people have advantages**

PQ01: As number 1, there’s no advantage s of not saving

**I: There is no advantage**

PQ01: Yes

**I: Okay, thank you, and where do sex workers save, or which places do they save their money?**

P: Women who are?

**I: Who are sex workers, where do they save? Yes number 3**

PQ03: Thank you, some of us have home bank. Some also save through Mpesa

**I: So for those saving on home bank why you do like saving in home bank.**

PQ03: Home bank for example, when you have an emergency let’s say for example I need to go to the hospital it is easy for me to take the money from the home bank but if I keep it in Mpesa at night I cannot find Mpesa agent to withdraw from. So that makes home bank to be better than Mpesa

**I: So, meaning you prefer home bank. Mmh that is number 3, yes number 9**

PQ09: As number 9, I like home bank because whatever amount you are left with you can save it there be it 10/= you just save. But with mpesa it does not accept 10/=

**I: Where do they like saving? Yes number 7**

PQ07: As number 7, the reason why home banking is good because I can visit somebody and after that he can check my phone and then after seeing the money in your account he might refuse to give you money. With home banking it is safe in the house as he will not see it. So if I go back to the house I will find it there and I will have also been given the money which I can use a little and the remaining I save in my home bank.

**I: Ooh, that is why you love home banking?**

PQ07: Yes

**I: Okay, apart from home bank is there any other places they prefer saving in? Yes number 6**

PQ06: As number 6 apart from home banking we also save on our phones

**I: Your phones**

PQ06: Yes

**I: Where exactly on your phones**

PQ06: You can save in Mpesa then again you can save your savings on Mshwari

**I: Mshwari? Why mshwari?**

PQ06: The reason why I like mshwari is because when you save there and you delete the message that alerts you of your savings no one can know the amount of the money you have saved because when you will be going to withdraw you will be alone.

**I: Ooh... Okay**

PQ01: As for me as number 1

**I: Eeh**

PQ01: I save on mshwari fixed deposit in which you can’t touch it. So, you can save it for a duration of like 4 months on a fixed account and I am not able to withdraw it, so I prefer mshwari

**I: Mmh, so I like Mshwari because you cannot withdraw the money every time**

PQ01: Yes I can’t withdraw it anytime.

PQ02: As number 2, I prefer mshwari.

**I: Mmh**

PQ02: Just after I had been conned through mpesa, nowadays I load the money on my mpesa then I transfer it to Mshwari, and again the reason why I like saving on mobile phone, the phones can help us everywhere anytime because with home bank we leave them in the house let’s say for example you you go to meet somebody in =Bondo=and after eating and other things, that is when she tells you that he has no money. It will force you to pay the bills but if you only have the home bank and you left it at home. So, it will force you to pay that bill to avoid the embarrassment from the people around you

**I: Mmh…**

PQ02:So that even when you have to quarrel with the person you do it when you are only two, because these men also sometimes they change their mind somebody can cheat you then you go to a restaurant order food then afterwards they say they don’t have money. So, that’s when I withdraw from mshwari then I transfer it to mpesa so that I clear the bill that is the reason why I like mobile banking

**I: Mmh… that is mobile banking**

PQ02: Yes

**I: And do sex workers live higher standards than their income? Let’s say for example that your income is here but your living standards is higher.**

ALL: (Laughs)

**I: Yes number 3**

PQ03: As number 3, we cannot live a life higher than our income

**I: Mmh, you live according to your income?**

PQ03: Yes, just within my income

**I: That is number 3, yes the remaining people, higher living standards than your income**

PQ01: I can live a higher standards depending on the person who I meet maybe for a day. My life can improve a bit, maybe he gave me a higher amount of money so my life will change a bit.

**I: Once in a while how about daily?**

PQ01: But for daily I will just take life normally how it should be

**I: Thank you, number 1, How about other girls?**

PQ05: As number 5

**I: Mmh**

PQ05: I like living in high standards it’s only that I have low income [laughs]

**I: Mmh**

PQ05: It’s only that my income is low but nothing is sweet like living life of high standards

**I: So what would make you to live life of high standards higher than your income?**

PQ05: Because I have so many clients.

**I: You have?**

PQ05: Many sex partners?

**I: So do they give you a lot of money?**

PQ05: Yes, it depends with what someone does, so if he gives you good money you will live comfortable and also do your things well

**I: Mmh, so apart from number 1 and number 3 who are living within their income, the remaining participants are living life of higher standards than their income?**

ALL: (Laughs)

PQ02: As number 2 mostly these sex workers they do live life of higher standards than their income because if you live a low life, he will come to your house and see the condition of your living. He will say, “So this is how you live,” so after he has left your house if you try reaching him through phone you will find him unreachable because he has known the life style you are living. So we have to try to stay well, dress well so that we attract. Even when he come to your house it should be the same way he sees you outside there. So, most people want to live a better life but their income doesn’t allow them that is why we will have to do sex work so that if this person gives me 200/= then the other person gives me 300/= that’s it.

**I: So for those who are living high standards of living what can they do to fill the gap that is between their income and their living standards? For those who are living life higher than their income it is only number 1 and 3 that are living life within their income**

**ALL: (Laughs)**

I: Is that true? So the remaining people what are you doing to fill the gap between your income and the living standards yes number 9

PQ09: So for this gap as number 9 how I will fill it

**I: Yes**

PQ09: That is where saving comes in

**I: Mmh**

PQ09:So I will save so that let’s say for example I visited number 5 and I found she has bought chairs I will be motivated to save so that I also live the life number 5 is living. So I save bit by bit so that after like 6 months I will have saved some money let’s say 50,000. It will be enough for me to buy a chair and maybe a table that I needed. So it is through saving that can help us fill the gap.

**I: Mmh, that is number 9, she fills the gap through saving, yes number 7 how do you fill the gap?**

PQ07: To fill the gap I can do business

**I: Mmh**

PQ07: So that I fill the gap

**I: What kind of business?**

PQ07: Selling things like tomatoes, vegetables do that I can get the money to fill that gap

**I: Mmh, number 8 what do you do to fill that gap?**

PQ08: As number 8

**I: Mmh**

PQ08: I can start a business then also can ensure I have more sex partners so that I increase my income

**I: Mmh, how about if the sex partners are not available**

PQ08: That one can’t be, we cannot miss clients

**I: So you don’t fail to get them sometimes?**

PQ08: We cannot fail to get them all the time

**I: Mmh**

PQ08: Yes

**I: So when they are there is when you work extra hard**

PQ08: Yes

**I: it’s okay, yes number 6 what can you do to fill the gap**

PQ06: The life I am living as number 6, I live a life within my income

**I: Mmh**

PQ06: Only if I make phone call and I receive some money, only that

**I: Okay, thank you very much**

PQ06: Yes

**I: Do sex workers also like borrowing money? Do they like borrowing?**

PQ05: As number 5 they don’t like borrowing money

**I: Mmh**

PQ05: I am also a sex worker but I don’t like borrowing money following my savings And also my business. I do it so that if I get profit I can do what I want I don’t need to borrow from somebody

**I: Mmh**

PQ05: Yes

**I: That is number 5, don’t like borrowing of money**

PQ01: As number 1

**I: Mmh**

PQ01: I also do not like borrowing money because the little I get I know how to use it

**I: Mmh, yes number 9**

PQ09: I don’t like borrowing money but sometimes it can force you, because sometimes you have gone out with somebody and you have done everything then he tells you that he is sending you money through m-pesa. He asks you if your M-pesa is working and you say yes and then he promises to send you. You will wait for the money and maybe he is gone already, maybe he has lied that he is going to a nearby agent to deposit for you money. That is when I will be forced to call number 5 or number 1.

**I: Mmh**

PQ09: Who are my friends to send me money so that I can come back? Because sometimes I don’t have money in my mpesa or maybe I saved in my Mshwari and sometimes you are told that you cannot access the account but when I am back and the Mshwari allows me to withdraw

**I: Mmh**

PQ09: I will just refund

**I: So when borrowing you borrow from your friends?**

PQ09: Yes one of my friends here.

**I: Okay, number 4 when borrowing where do you borrow from?**

PQ04: (Laughs) I don’t like borrowing I just work hard

**I: You never borrowed even just once?**

PQ04: No

**I: You have never borrowed?**

PQ04: Few times

**I: Mmh, when borrowing where do you borrow from?**

PQ04: My fellow business person

**I: Your fellow business person**

PQ04: Yes

**I: Why do you borrow? For what purpose?**

PQ04: So that I fill the gap

**I: For the business?**

PQ04: Yes

**I: Okay, it’s okay number 3 if you borrow money where do you borrow from?**

PQ03: Thank you very much as number 3 when borrowing money I can borrow from a certain group so that I can boost my business.

**I: So in that group, are you a member or they are just people you know? Or what kind of group is it?**

PQ03: I can take through number 1 if she is my friend and she is a member of a certain group or maybe her friend so I can take through her then when I get the money I return through her and she returns it to the group.

**I: So if she is in a group she borrows from her group then she lends you?**

PQ03: Yes

**I: It’s okay, and when you borrow, what is your purpose of borrowing?**

PQ03: Sometimes issues of school fees or emergency so it will force you to borrow then if you find the money you return it back

**I: You return the money**

PQ03: Yes

**I: And what do sex workers do to adjust on their income? Right now we are still doing sex work?**

ALL: Yes

**I: Let’s say so, what can you do to increase your income let’s say for example you always get 500/=what can you do so that it increases to 1000/=what are the things you can do?**

PQ05: As number 5

**I: Yes**

PQ05: This income will increase from 500/= to 1000/=I have to do business little by little because 1000/= I cannot get within a day

**I: Mmh**

PQ05: I will be selling and adding stock time by time and it will reach me my target

**I: Mmh**

PQ05: Yes

**I: Any other thing apart from business? Any other thing they can do to increase their income? Let’s not be tired we are almost (laughs) just continue number 5**

PQ05: As number5, Apart from business another way I can boost my business is that we also try to join groups, Let’s say for example like now we are here we come up with a group where we do saving and loaning You can save and take a loan to help you help you achieve what you want to achieve.

**I: Okay, forming of groups and business, eeh any other thing that they do to increase their income? Yes number 3**

PQ03: Okay as number 3 I just want to support what number 5 has just said

**I: Mmh**

PQ03: We can form groups

**I: Groups, that’s okay, and is there a way sex workers can predict when there are no clients? Like you can say today there are no clients.**

ALL: (Laughs) Yes

PQ03: Yes thank you as number 3 sometimes I do go to [name of place mentioned] and just a few minutes even before my bottle of soda is half way I get a client but sometimes you can go and sit there the whole day, whole night and you don’t see any client, everyone is coming with his fixed money or maybe they come with their sex partners. You know you will be forced to come back and remember you had used your fare in going there and again when coming back. That is when you will say that this week the business was bad

**I: Okay, number 2**

PQ02: As sex workers, there are times when we don’t have clients. Like right now schools are opening and form one intake is ongoing. Right now getting the clients is very difficult, all the money is used in school fees and you won’t get anyone sited where you go to wait for them but there are times when they are available like in December they are so many. You can find 3 clients at once, so they are not available everyday but just some time like the times of Covid-19

**I: Mmh**

PQ02: There were no clients For sure. So, they are not available all the time, they have their duration

**I: Mmh, and now you know when they are available and when they are not available**

PQ02: Yes,

**I:Or apart from during school, any other times when you can tell that they are not available, any other person (laughs)Yes number 3**

PQ03: Okay mostly mid-month when people have not receive their money/salary

**I: So? Yes number 9**

PQ09: I had same idea as number 3

**I: Okay, you had the same idea, any other person when you can tell (silence...) only those that you have said**

ALL: Yes

**I: Do we all agree or we are tired**

ALL: (Laughs)

**I: And is there limit for the money that sex worker can have as debt? Is there a limit when one borrows money, you can find that when girls borrow money like here we are 11 let’s not count him**

ALL: Yes

**I: Is there an average that most of us will have, or I am confusing you?**

PQ02: We don’t get it

**I: Do I say it in Kiswahili**

P: No just explain it in dholuo (laughs)

NT: (Continues as interviewer) Like for example when borrowing money how much money do you usually borrow? How much money do you usually borrow?

**I: The average**

NT: (Continuous as interviewer)you can do an individual average and say that the money I borrow I cannot have it more than a week (cross talk)another person will say the money I borrow I can return after 6 months ,now it depends with an individual so in total how much is it?

**I: If you calculate the total how much is it?**

PQ01: As number 1 I have debt

**I: Mmh, how much**

PQ01:5000/=

**I: Mmh, yes number 5**

PQ05: I have 30000/= for a year

**I: Yes, 30000/= for a year, yes number 8**

PQ08: I have 3000/=

**I: Yes number 9**

PQ09:25000/=

**I: 2500/= Yes number 4**

PQ04:1500/=

**I: 1500/= Yes number 3**

PQ03:10,000/= for a whole year

**I: 10.000/=?**

PQ03: Yes for a whole year

**I: Yes number 2**

PQ02:15 in a year

**I: 15000/= or 1500/= in a year**

PQ02:15000/=

**I: 15000/= in a year, yes number 7**

PQ07:2000/=

**I: 2000/=Yes number 6**

PQ06:10,000/=

**I: 10000/=Thank you all very much, that was just a by the way, and have sex workers think of leaving their job as sex work? (Somebody coughs in the background) is it something people have thought of? Have seen participant 2 nodding your head.**

ALL :( laughs)

**I: Yes say it,**

PQ02: That is something that crosses my mind everyday How I can stop that job. But I cannot stop because of source of income. I have low source of income and I have young kids. And you have nobody to support you so that is what will force you to start that work

**I: Mmh**

PQ02: Yes

**I: When thinking about it, do you think about it as an individual or do you sit together in a group of women who are also sex workers and discus it.**

PQ02: I think of it as an individual

**I: As an individual?**

PQ02: Yes, I keep asking myself for how long will I do this and how will I leave doing it?

**I: Mmh**

PQ02: That thing really disturbs me but I have not shared it with anyone, it disturbs me alone. There are some things that somebody don’t just do for leisure, you are forced to.

**I: Mmh, any other person, number 9**

PQ09: I have also been disturbed with that thing one day when I was out with one of my fellow sex worker and we went out, she was treated badly just like I was. We both did not earn anything when we come back we asked ourselves how we could leave this work. The idea we had was just that one that number 2 has shared, that if we stop how we will survive and the business we are doing cannot be enough to support us together with our children. So, we decided we cannot stop it because we still don’t have any means of support. So even if a client disappoints you, tomorrow you get another one you will just go

**I: Mmh, so what happened to you is what made you to start talking about that**

PQ09: Yes, we do share, so when she asked, “what did you do?’ we realized we had the same problem (laughs)

**I: Thank you very much, any other person, yes number 3**

PQ03: As number 3, we face many challenges, you know if someone promise you that he will send you the money through m-pesa do you know that it’s a debt that is very difficult to ask for. It’s hard to take your phone asking what happened. Maybe you went… sometimes you are told to use your own fare when going that you will be refunded once you reach and he ends up not returning it. You know when you come back you are worried because the money you have spent you had saved for some other reasons. And now you have withdrawn it and used it for that reason yet when you were going you also had your target. Like when I’m given 500/= I will do this and this, it is a challenge now there is nothing we can do, because I have used my saving just because somebody called me to go and I also went knowing I have nothing

**I: So such kind of challenges is what will make you think…**

PQ03: Think of quitting the sex work

**I: Quitting**

PQ03: Yes

**I:We have seen the challenges of participant 2 and 9 have encountered that have make them think of quitting sex work is that right? Even number 3 is in support of that. Are there more reasons that make you feel that you can’t quit because you have not attained your goal. Even from your friend what makes then to continue even when they decide they want to stop? What can stop you from quitting sex work? Yes number 3**

PQ03: Just as we had said earlier. You feel like quitting but you have not reached target you have not saved enough, now it is difficult. You know for me I can survive by starving but for the children if they don’t get breakfast they will ask, “Today there is no tea?” we wre not even eating? [Laughs].

**I: Is there a specific age that when a lady reaches then she should quit sex work? Do I say retiring from sex work is there an age for retiring?**

PQ01: As number one. If you are a grandmother you cannot do it because you are now old.

**I: What age is a grandmother? According to you?**

PQ01: About 60 years -67 years

**I: Mmh any other person?**

PQ05: As for me sex work has no retirement It’s your death that will make you quit.

**I: Mmh, it doesn’t have retirement?**

PQ05: It does not have retirement, because even those who are 70 years -90 years they enjoy it to the fullest

**I: Mmh any one with a different idea**

P: For me I feel there is retirement because even the children birthed will also grow and it reaches a time when you will stop according to your age and the kids and the way they have grown will not allow you to come.

**I: So because of the children**

P: Yes.

**I: Any other idea, yes number 7, can somebody quit sex work just because of age or there are other factors that can make her quit**

PQ07: You can quit, sometimes you didn’t have a husband that’s why you were doing it but sometimes you can get a husband amongst your clients. So he can tell you to quit that work because he is with you, then you decide to leave.

**I: Mmh, okay that is okay, and what kind of jobs can sex workers do after quitting sex work, I know number 5 has talked of sex work has no retirement but now we have decided to quit, what kind of job can someone do? What kind of job, yes number 4**

PQ04: She can do any job that can put food on the table for her

**I: Mmh, like which ones**

PQ04: She can do business or do small scale farming

**I: Mmh business or farming, so when she do business what can she sell in specific?**

PQ04: Food stuffs

**I: Mmh. She can sell food stuffs, another thing number 9**

PQ09: As number 9, the job a sex worker can do after quitting. I know there are those who are educated but they just joined sex work because of lack of job or because she couldn’t do business not everyone can do business, so these are people that their documents if they present to an organization like this they can find her job. So, such a person being that she has experience in this field she can be able to talk to young girls about her experience in sex work and share the challenges faced when she was a sex worker also the kind of life she lived when she was doing that job. So she has enough experience to be a counsellor to those who are in the field of sex work.

**I: she counsels those who are sex workers**

PQ09: Yes

**I: Mmh, any other person who wants to share with us the kind of jobs they can do? I want to look for somebody who is quiet than everyone here to answer this question**

ALL: (Laughs)

**I: It is okay, if none of you have another idea then let us continue, do you know of any lady who has quit doing sex work? Who has quit for good not who left and again went back on it, yes number 3**

PQ03: Thank you very much, as number 3, I had seen a woman when I was in =Kisumu=. God had blessed one of her children who changed her life, bought her a piece of land then built for her and was taken to stay at home as she get support from that her child.

**I: So her children are supporting her, how was her life after she quit**

PQ03: She is just okay

**I: There are no good or bad things that happened to her.**

PQ03: No, she is just okay she left the Job and God blessed her kids and she lives a life that when you look at her you can’t even know that she used to do such a job.

**I: Okay, number 2**

PQ02: As number 2, I also saw one she wasn’t an elderly woman but she was just as young as we are. What happened to her that made her to quit, she stayed in rental house and one day she had a date with two men in her house. The first client called her but she thought he was not going to come then another one also called her and she allowed him to come. so these two men met and they fought and at the end one lost his life and that lady swore that if that is the kind of work she can do to put food on her table then it’s better she quit. And that’s how she left and then again imagine such a thing happening to you where many people are living, it was embarrassing to her and she decide to quit. And now she is in business, so those are some of the things that can make you quit from this work and not only that ,there are some things that can happen to you in this field until you swear it’s not the only job

**I: So, that is what made her quit**

PQ02: Yes, she left

**I: Ooh, okay yes number 9**

PQ09: As number 9, so one day we were with another lady (clears throat) then another lady was passing and some people were talking negatively about her that she is a sex worker. The lady told us to stop talking negatively because that is also just a job. She was an educated lady who was pursuing bachelors of education. She had completed the course and was waiting to be employed so I asked her why she did not want those people to talk negatively about sex work. She told me that her mother paid her school fees up to university level through that job, she went to university and completed. So, it was in December and she was to start her employment as a Secondary teacher come January. She said that being that she is going to start her employment she had talked to her mother to sex work and the mother already stopped. So she was now going to allow her mother to rest, and totally stop doing the job so that she can support her mother because she has undergone many challenges when educating her, so she assured me that her mother was going to totally quit the job.

**I: Is there anything bad she has experience since she quit?**

PQ02: She called me one time and she told me that they were just doing great.

**I: Mmh, okay and do you know of anyone who had quit and again came back? May be she left for some time and resumed?**

PQ05: As number 5

**I: Yes number 5**

PQ05: I have seen those who left and resumed.

**I: What made them resume?**

PQ05: It’s very difficult to ask somebody why she came back, but she was somebody who had quit and went to church to confess her sins, but later she came back to sex work.

**I: So she did not share with you why she came back?**

PQ05: She cannot share with you.

**I: Mmh, Is there anything good or bad that have happened to her after coming back?**

PQ05: She is just okay we are with her in the same field.

**I: Any other person, yes number 6 anyone you know of who had quit and resumed?**

PQ06: I don’t know

**I: You don’t know eeeh, number 8**

PQ08: Nobody

**I: You don’t know of anyone who had quit and resumed?**

PQ08: Yes

**I: Mmh, number 4**

PQ04: [laughs] nobody

**I: It’s okay, thank you very much are there something you need to do before quitting sex work? You have plans in your mind that by the time I am quitting sex work I should have done this and this ,is there something as such that you plan, yes number 1 what you have planned to do so by the time you quit sex work you should have done it.**

PQ01: As number 1 I have a plan that when I quit sex work I should have a stable business that will sustain me my entire life

**I: Mmh What kind of business?**

PQ01: Opening a big shop with stock then I sell the goods

**I: What kind of goods?**

PQ01: Selling things like…..maybe opening a hardware shop.

**I: Hardware, okay thank you, yes number 6, what you would like to do before quitting?**

PQ06: I will only want that by the time I am quitting I have saved enough.

**I: Mmh, Okay number 9, what you would like to do before quitting?**

PQ09: Before I quit, I became pregnant when doing this job and I bore a son so I would like that before I quit I will have bought a piece of land then I build a house. So even if I do another business and leave sex work. I have my home where I line and even if God take my life, my son will have a place to live.

**I: Mmh, number 5**

PQ05: As number 5 I would wish that if quitting this job, I should have someone I call husband who I will be living with. Someone who will take care of me.

**I: How about if he is not available?**

PQ05: If husband is not available?

**I: Yes and you want to quit what will you do?**

PQ05: I will have to find a husband (laughs)

**I: (laughs) number 5 doesn’t want to think of if there is no husband, yes number 7 what you would want to do?**

PQ07: I can…I’m supporting what number 5 said (laughs)

**I: Supporting her in what way?**

PQ07: In that when I’m quitting it finds if I have already found someone to live [a husband] that is when I can quit.

**I: Mmh, and if you haven’t found anyone and you feel you need to quit? Is there anything you would want to do?**

PQ07: I can’t quit until I find one

**I: Until you find?**

PQ07: Yes.

PQ08: As number 8, what I would like to do before I quit I would like to find land just the way number 9 had said. I wish to acquire land then build my home so that I also have somewhere to call home. Then I also do some business

**I: Mmh, number 2,**

PQ02: As number 2, when I take my time thinking, I only do this so that I can find something to save. But if one day I can settle buy land and build a house then settle at my home then my child has also reached some level. They if I get some money I will boost my business then I become comfortable and relaxed Then I will just be okay, but as per now that I still have no money in my hands, I cannot quit, it just forces me to continue though I always feel that someday I get enough money, I can save and attain a good amount so that I can do the three things I desire to do.

**I: Mmh, number 3**

PQ03: As number 3, I have realized that my future dreams are the same as those of number 9 and number 2 (clears throat). It has always been my wish that, because this job has retirement and I would wish that by the time I will be quitting I should be comfortable a piece of land that I have bought and built.

**I: Okay yes number 4**

PQ04: Just as they have said,

**I: People have talked about so many things, so, which one are you saying?**

PQ04: I cannot quit this job without reaching my goal and also taking care of my children and their school fees

**I: Mmh so you would want your children to finish their education first?**

PQ04: Yes.

**I: Thank you very much, so is there anyone of you who have start putting plans into action ,I believe all of us here have shared about your plans?**

ALL: Yes

**I: Is there any one of you who have started putting plans to begin doing what they plan to do before quitting or those who have started working towards the plan? (Silence) we are quiet to mean there is none or they are just our future plans but there is no one who have started working towards it, yes number 9**

PQ09: As number nine, the thing is in my mind, But, I begin saving but when I lack money it forces me to withdraw from the savings

**I: Mmh, so again you remove from the savings**

PQ09: Yes

**I: Thank your number 9, yes number 2**

PQ02: As for me the plans as I have always tried to plan. I plan but it don’t work, I save but for the money to reach the target you realize that again you have to deduct some money. Even on the issue of building that I am talking about. I already bought a piece of land but the money I had wasn’t enough. So, for you to look for a place it shows that it is in your mind that even if you can get money immediately, you will just pay for it. So it is just something that even if you get 20,000/= you take there so that one day you also live like your fellow, so for me it’s a step I have made

**I: Okay, so for the rest of you still don’t have plans? It was just in our thoughts?**

ALL: Yes

**I: t’s okay, I’m very much grateful for being able to answer all the questions we are almost winding up ,so number 1 don’t be too tired (laughs) We are finishing, so I had explained to you about JItegemee what we read about at first about Jitegemee and I explained to you that it is done so that sex workers have their savings and this will make them deny men sex without condom and also so that they have rest from sex work when they want. There is an option of relaxing because you have some strength stored somewhere. I also said that sex workers will be saving some part of their money to use when there are no clients and also in preparation for future life after quitting sex work. The way we are thinking of implementing Jitegemee, do you think sex workers in Kenya will accept it? Do you think they will accept this? Yes number 3**

PQ03: Yes as number 3, I believe sex workers will accept it because it will help us move from one level to the next, I don’t think if they can reject it

**I: Mmh, they will accept it, that is number 3 any other idea from a different person,**

PQ01: As number 1, I can accept it

**I: Why?**

PQ01: So that I reach my target.

**I: Mmh, any other person, yes number 8**

PQ08: As number 8 I can accept it

**I: Why?**

PQ08: To help achieve the target I want

**I: Mmh, yes number 9.**

PQ09: As number 9, I would accept it because following the teaching I have gotten new knowledge because saving is something I should be doing.

**I: Mmh, Okay it’s okay**

PQ09: Yes

**I: And for those who will not accept it why do you think they will not accept it? So those who were quiet, yes number 4 why will you not accept it [laughs]. Not specifically you but even other women, those who will refuse to accept Jitegemee [baby crying in the background] or what will make them reject Jitegemee? Or what type of women will not accept Jitegemee. Yes number 2**

PQ02: Women who will not accept Jitegemee are those women who do not think of the future. But for a woman who is concerned about their future cannot fail to accept. People are different, but I believe majority will accept

**I: Mmh**

PQ02: Very few people may not accept it because she is concerned about today but do not know about tomorrow and maybe she has a child and you don’t know that if you die today, how will your child be the next day? So many people will accept it because women love their children

**I:So let’s say for example that we all know 10 sex workers, how many out of 10 that will accept Jitegemee, yes number 6,how many out of 10 that you know will accept Jitegemee.**

PQ06: The number?

**I: Out of 10 that you know, how many will accept to join Jitegemee?**

PQ06: I know of none.

**I: There’s no lady you know of? You only know of yourself?**

PQ06: Only if I talk to someone and ask.

**I: Mmh… any other person who knows 10 people, how many out of the 10 will agree to join Jitegemee?**

P: 8

**I: Eight, yes number 5**

PQ05: 10

**I: 10 eeh, number 8**

PQ08: 6

**I: 6 number 9**

PQ09: 10 or more

**I: We only need 10 (laughs) PQ09 has said 10**

PQ09:10

**I: PQ09 has said 10 eeh number 4**

PQ04:7 eeh number 3

PQ03: Out of the 10 I know 9 will accept

**I: Eeh 9 will accept, eeh number 2**

PQ02:7

**I: 7 will accept, eeh number 7**

PQ07:10

**I: Eeh number 6**

PQ06:5

**I: At least now you know some people [laughs]**

P: She did not know where we were headed to.

**I: She didn’t know where we are headed to, apart from number 9, 7 and 5, the rest of the people there are those who will refuse is that right? Like number 3, 2 people will refuse then number 2, three people will refuse what will make these people who will reject out of the girls that you know what will make them reject? What will make them reject it I believe you can perceive them in your mind? They are your friends and you know them.**

ALL: Yes

**I: Why would they refuse? Yes number 3**

PQ03: Those who can refuse are those who…just like people are stigmatized there are those who will still not agree to join a discussion like this one. She will say, “Eh… people will identify me as… [Sex worker]” so stigma can make one not to accept

**I: Mmh, so she is embarrassed being called sex worker?**

PQ03: Yes

**I: And that will make her refuse, yes number 6 you had raised your hand**

PQ06: Some may not join after hearing about the discussions that they hear we get from this group

**I: Mmh, okay because we discuss about saving so she won’t be interested in that?**

PQ06: Or about having several sex partners

**I: Ooh, any other person, the reason as to why they will refuse for those who had people who will refuse, yes number 8 why will those remaining refuse to join**

PQ08: As number 8, they can refuse to join following the embarrassment they will get because most people will know the kind of job they do

**I: Mmh, thank you, and what can we do to these girls so that they accept Jitegemee? What can we do yes number 3**

PQ03: You who will implement it

**I: Yes, we are the people who are bringing the Jitegemee, what can we do so that the girls accept it?**

PQ03: Okay, thank you as number 3, What you can do is just to encourage us about the savings so that when they see those who had saved their lives have changed then they will also accept to join because they will see the benefit and know that Jitegemee is a good thing.

**I: Okay, yes number 1**

PQ01: As number 1, they might have a feeling that, you know when something starts some people don’t always like it. So later on they might realize that it was a good thing then they will change their minds then the number will increase.

**I: Mmh, what can we do so that they find it of importance?**

P: It will depend with how we will be running the programme that is what will make them know that it was of importance

**I: Ooh, so those who had accepted will let them know the difference**

P: Yes

**I: Okay**

PQ07: What will make them have a feeling of joining, is that they will see what I have done with the money I was saving and that will make them to join.

**I: Mmh, and if it is about teaching/discussions what kind of discussions should we have to make the girls feel comfortable as Jitegemee, what kind of things should we have so that even when you come from there you can say, “I was in Jitegemee and you feel proud feeling proud,” Pride in dholuo has disappeared from my mind.**

ALL: Happy

**I: Yes you feel happy to say that you were one of the girls who have been in Jitegemee, what are the things we should have? Yes number 9**

PQ09: As number 9, I think what will make me proud to share with someone that I was in a discussion of saving in Jitegemee. I can go and the questions I may be asked like, like people were being asked the number of partners, I will not go into such. I will only share about saving, what you can do with your money after saving for one months or three months you can do with it.

**I: Mmh**

PQ09: So when I have discuss with her and I try to find her perception in that So that is how I will get to convince her because she is my fellow sex worker and I always see her tough so I will share with her that do you know that this time round I have saved money and bought this table so it is not a must that you keep that sex partner because he do give you 200/= but you have managed to save like 3000/=So that is what will make me convince her

**I: Mmh any other person with a different idea, what kind of things should we have, yes number 4 (silence) eeh, this garget is not a video just be audible [laughs] yes what are you saying**

PQ04: Nothing,

**I: Okay any other person, what should we have? If any?**

PQ05: As number 5

**I: Yes number 5**

PQ05:Through love amongst ourselves and team work ,because as sex workers we know ourselves you have to come close to her and get to know her thoughts find how to convince her easily so that you find her to be one of those 10 girls.

**I: Mmh… and are there things that they will not like in Jitegeme? Like we had read about Jitegemee, what we would want to do and how we will do it, is that right?**

ALL: Yes

**I: Are there things that they won’t like [baby crying in the background] yes number 3.**

PQ03: Thank you as number 3 I don’t think that there are things that they won’t like following the teachings we have gotten from here. Unless we are the people who share with them different information from what we have been taught today. But when we just share with them the same information that we have been given today then no one can refuse.

**I: Mmh… that is opinion from number 3, any other person with a different opinion? Number 8**

PQ08: Still have no idea.

**I: You have no idea, [laughs] are you with us really?**

PQ08: [laughs] yes.

**I: Do not think much we are almost winding up, let’s just be together, and yes number 7 a different idea if there is? (Silence) say it louder**

PQ07: I am still thinking

**I: You are still thinking? For now you have no idea**

PQ07: Yes

**I: And for the things that we are thinking of doing, do you think it will violate the rights of the girls? Will the girls feel that they have been denied their rights?**

PQ05: As number 5

**I: Yes number 5**

PQ05: The girls will not have any feeling that their rights have been violated with Jitegemee. Because Jitegeme is also bringing their programme so that they help us from making us improve from 1 to the next level 2. So, for someone who is mature I don’t think if they can refuse.

**I: Okay, yes number 9**

PQ09: As number nine I don’t think if their rights will be violated because when we were starting you told us that… you didn’t tell us that we should stop doing this thing, but we should just continue with it having in mind that we should save meaning we depend on ourselves. So, if you explain to someone very clearly then she won’t feel that her rights are violated

**I: Mmh, thank you, number 1**

PQ01: As number 1 I don’t think if it can violet somebody’s right

**I: You can’t see.**

PQ01: Yes

**I: It’s okay, and what are some of the difficulties we will have when we start Jitegemee? Today we are just collecting information is that right?**

ALL: Yes

**I: Now let us assume that we have gotten good information and the programme is about to start. is that right, and now we are giving the girls idea how we had read earlier about how we can save and many others, what will make it difficult for us when we want to start it? Number 6. What will make it difficult for us?**

PQ06: I haven’t gotten you clear

**I: When we want to start Jitegemee**

PQ06: Yes

**I: Right now we have not started, it’s only that we are gathering information is that right?**

PQ06: Yes

**I: So it is a programme that is yet to start, which challenge you think we can encounter if we want implement it.**

PQ06: I don’t see any challenge you can encounter.

**I: P7 the challenge we can meet**

PQ07: I don’t see any difficulty because you have taught us how it is going to work so I don’t see any difficulty

**I: No difficulty?**

PQ06: Yes

**I: Eeh number 2**

PQ02: If you have not been taught about something that is when you can have difficulty. But if you have been taught and you have known the direction how things are moving you can’t have difficulty because you know what you are going for and you will okay with it

**I: Mmh, so, we just come today and have not taught you is there any difficulty you will have? Before you are taught is there any difficulty you will encounter.**

ALL: There is none.

**I: There is none, yes number 3**

PQ03: Their will be no difficulty

PQ03: No difficulty, yes number 4

PQ04: There is no difficulty (laughs)

**I: Eeh number 9**

PQ09: There’s no difficulty

I: No difficulty, yes number 8

PQ08: No difficulty

**I: No difficulty, yes number 5**

PQ05: There is no difficulty

**I: No difficulty yes number 1**

PQ01: There is no difficulty

**I: There is no difficulty, and let’s say that you are in Jitegemee how much can you save weekly? You have your daily responsibilities and you also have your daily income and you have joined jitegemee, Jitegemee is about saving, how much can you save weekly? Yes number 1**

PQ01: As number 1 I can save 50/=

**I: You can save 50/= weekly is that right?**

PQ01: Yes

**I: Number 5**

PQ05: As number 5, I support number 1

**I: You talk as per yourself, everyone is living in her own house**

ALL: Yes

**I: And everyone has her own responsibilities so you need not to support what another person has said, you say your own [laughs]**

PQ05: Yes number 5

PQ05: Just like number 5 I can also save 50/= that is easier

**I: Number 5 50/= eeh yes number 8**

PQ08: As number 8, I can save 100/=weekly

**I: 100/= weekly, eeh number 9**

PQ09: I can only save 40/= in a week

**I: 40/=in a week. Eeh number 4**

PQ04: 30/=

**I: 30/= in a week eeh number 3**

PQ03:100/=

**I: 100/= in a week, eeh number 2**

PQ02: 50/=

**I: 50/= in a week, eeh number 7**

PQ07:100/=

**I: 100/= in a week yes number 6**

PQ06:50/=

**I: 50/= in a week, so those are the targets we have fixed. that you are comfortable and will not interfere with how you live, so how about if you find it difficult to reach that target, maybe this week it was so tough that you couldn’t reach your set target. What can you do so that you reach your set target so that you be able to save those amount that you have mentioned 30/=, 50/= and 100/= that you need to save what can you do? Let’s start on a reverse yes number 6. What can you do if you are not able to to raise the money that is your weekly target. What can you do differently to help you reach your target of maybe 50/= that you were to save?**

PQ06: If I cannot find the money to save so that I raise the 50/= I need in a week

**I: Mmh**

PQ06: I can ensure that I am saving at least 200/=

**I: How do you get the 200/= what will you do to get the 200/= you are saving? You are to save 50/= in a week**

PQ06:50/= in a week

**NT : The 50/= you mentioned was it for a week or a day?**

PQ06: For daily

**I: Ooh for a daily, so daily 50/= so meaning in a week meaning 50 by 3… 50 for P3 that is 350 in a week it.**

PQ06: Yes

**I: So meaning number 6 is saving 350/= in a week, then its difficulty for you to raise that target that you had set for yourself**

PQ06: Yes

**I: Okay, you are not able to reach your target, you have set a target for yourself and you know your means of income, the responsibilities you have but you would wish to save 350/= in a week is that right?**

PQ06: Yes

**I: Now, you are not able to raise the money, what will you do so that you get that 350/=?**

PQ06: I can’t fail to raise it I must get even just 200/= [laugh] I can’t miss to get

**I: So there are no days when you will miss to raise that amount**

PQ06: In a week I can’t fail to raise it

**I: You cannot fail to raise it**

PQ06: Yes

**I: Okay, number 7 if you are not able to raise it what can you do to reach the target you set?**

PQ07: I cannot have difficult

**I: Mmh**

PQ07: In fact I can even get something small to add on top even just 20/=then I add

**I: Where do you get that small amount from?**

PQ07: The small amount I’m talking of I will get it through, let’s say for example I have get a client who invite me to have a drink with him and he will maybe buy for me a bottle of soda or beer then after buying me the drink he might give me money to go buy food with, but I will not use it all in buying food instead I will keep 20/= so that I add it on top of my savings, then use the rest

**I: Mmh, number 2 when you have been defeated to raise what will you do to achieve your set target?**

PQ02: If I have known that tomorrow I’m going to work so that I can save that money, I know raising 50/= cannot be difficult to raise but when it becomes very tough I can call one of us, knowing very well that by evening i will have raised some money to refund her the money, so I am sure 50/= I cannot fail to get.

**I: So you call your fellow girl? Or who do you call?**

PQ02: I can call those my partners but if they don’t have then I can call one of my girlfriend who can give me.

**I: Mmh**

PQ02: So I will have to start with those people first because I know if you request for 50/= they cannot fail to give you. You can even pretend that you were requesting for credit but it is you who knows what you are going to do with the money.

**I: Mmh**

PQ02: That is why I’m sure I cannot lack 50/=

**I: Okay, now when you talk of those your friends who are you referring to?**

PQ02: Boyfriends

**I: The clients?**

PQ02: Yes

**I: Ooh I just want to understand because there are different types of friends, friend is a general term used, yes number 3, what will you do if raising your target becomes a challenge?**

PQ03: Thank you as number 3, if it becomes difficult for me I can borrow from number 1 or number 5 then I return to them

**I: Okay eeh… number 4, if you have a challenge**

PQ04: If I cannot raise it then the budget for food stuff is what I will have to reduce so that I reach my target

**I: Mmh, you will reduce your budget**

PQ04: Yes

**I: Will that not make you strain?**

PQ04: No

**I: Because we had said you put a target that makes you not to strain, is that right?**

ALL: Yes

**I: So if you reduce that one, will it not affect you?**

PQ04: No

**I: You are okay with it?**

PQ04: Yes

**I: Okay, number 9 what can you do to achieve your target, if it’s a challenge to you?**

PQ09: I can borrow from my friends

**I: Borrowing from friends?**

PQ09: Yes

**I: Which kind of friends’ men on women?**

PQ09: No, just women

**I: The girlfriends?**

PQ09: Yes

**I: Mmh, no 8**

PQ08: As number 8, I can also just borrow from my friend

**I: A male of a female?**

PQ08: Either a male or a female

**I: Mmh… (laughs) okay number 5**

PQ05: As number 5, I will try so that the money I raise daily in a week it reaches 50/= then I just save it there

**I: Okay number 1 what can you do?**

PQ01: I can borrow from someone

**I: Borrowing from who?**

PQ01: either number 4 or 5

**I: It is okay**

NT : Why do you borrow from your friends, we had mentioned earlier so many different places where one can borrow from, why do you just talk of borrowing from a friend?

ALL: It’s a friend who understands us

NT: [Laughs] there is no any other reason why you prefer borrowing from a friend.

ALL: Yes

**I: It’s okay, so when saving in Jitegemee where do you feel is good place or a place you can trust with your savings? Yes number 1**

PQ01: Phone

**I: When you mention phone we have different application on phones which one do you prefer?**

PQ01: Mshwari

**I: Mmh Mshwari eeh.. yes number 5**

PQ05: KCB

**I: KCB bank or Mpesa**

PQ05: M-banking

**I: M-banking eeh number 3**

PQ03: I prefer home bank

**I: Eeh home bank, number 9**

PQ09: KCB

**I: Mmh, KCB, bank or mpesa**

PQ09: Mpesa

**I: KCB Mpesa yes number 4**

PQ04: Phone

**I: Phone?**

PQ04: Mpesa

**I: Mpesa, eeh number 3**

PQ03: Mshwari

**I: Mshwari, yes number 2**

PQ02: Mshwari

**I: Mshwari, Yes number 7**

PQ07: KCB

**I: KCB, bank or mpesa**

PQ07: Mpesa

**I: Mpesa yes number 6**

PQ06: Mshwari

**I: Mshwari, okay thank you very much we are grateful, sorry number 3 for being too tired we have come to the end of our session and I am very much thankful is that okay/**

ALL: Yes

**I: I am also grateful for the information you have given us and also your responses towards the questions we have asked [phone ringing in the background] is that okay?**

ALL: Yes

**I: Do you have any question?**

P: For us as …

**I: Yes number 1 you can close the session**

PQ01: We are also grateful for meeting us today

**I: Mmh**

PQ01: And giving us good teachings, so we also know how to take care of ourselves

**I: Mmh, thank you very much, we are also grateful for welcoming us here and the responses you have given to us is that okay?**

ALL: Yes

**I: Okay thank you is there anyone with any question or concern? Thank you.**

**END OF INTERVIEW**
