# Supplementary material for: Eat a little and save a little: A qualitative exploration of acceptability of a potential savings intervention to reduce HIV risk among female sex workers in Western Kenya
Source: PLoS One. 2024 Dec 19;19(12):e0310540. doi: 10.1371/journal.pone.0310540 (PMC11658496; doi:10.1371/journal.pone.0310540)
Supplement: S1 File — (ZIP) [file pone.0310540.s001.zip › Jitegemee Transcripts and Dissemination Notes for Journal/FGD R.docx]

**DATE OF INTERVIEW:04/MAY/2022**

**MODERATOR: NANCY OUNDA**

**NOTE TAKER: OLIVIA OKUMU**

**FGD ID: FGD R**

**VENUE: MADIANY RARIEDA SUB -COUNTY**

**CATEGORY: ABOVE 30 YEARS, RURAL.**

**I: Okay we are going to start, now just as I had explained about Jitegemee what comes in your mind? What does Jitegemee want to do?**

PR03: just as I am when I hear about Jitegemee (birds chirping) what comes in my mind is as us ourselves [birds chirping] we take care of our self. What we have we can take care of our self on our own that how have heard it.

**I: Mmh**

PR03: Aah

**I: Another person?**

PR010: I support number 3 and I add saying that it is my help myself Jitegemee is something that I help myself I have something that can help myself without depending on my fellow human being. So, I depend on my own thing that can help me.

**I: Mmh, another person?**

PR01: Okay, as number one I can support what my fellows have said. Jitegemee as I have heard means that you want us to have your own thing that you can depend on like your business. If you are a sex worker , you depend on a man or men such things.so this jitegemee can be something good because you can have your own things that brings you income on your own if you leave other things like so and so to sleep [engage in sex ] with me so that he pays me. The body can get tired like now I am tired of engaging in sex ,so, you can find something that you can depend on at least am done for now.

**I: We are all going to talk since I had read what jitegemee is all about with your own opinion, what can you say for those who have not talked?**

PR07: Me as PR07 if I speak about jitegemee it is me that I must have something that can help me without depending on someone else so that he can help me. I have my own that I know if I do it this way it’s going to bring something like this and can help me.

**I: Mmh**

PR07: So, it is about me not another person that’s how I have heard it.

**I: Another person?**

PR06; Me as PR06

**I: Mmh**

PR06: I support PR07 as she has said because when I have my own thing and I feel my body is tired someone won’t force me and also I won’t think that if have not slept with someone then my children will sleep hungry even if I have not done that my children will still eat.

**I: Mmh. that is number 6 Mmh another person.**

PR05; In my opinion I support PR06

**I: Mmh**

PR05: Am supposed to fend for myself and putting my mind that it is not to engage in sex that’s when I can find how I can feed myself am supposed to fend for myself on my own.

**I: PR04.**

PR04: Me as PR04. If I hear about jitegemee; I see it taking me to if have my small business I put all my strength that I should not depend on or maybe another man that he must give me something so that I can help myself.

PR02: As number 2 I also support PR04 because if you have your own business, it’s good it helps you instead of depending on someone else money (bird chirping) because sex work .sex work is not easy Sometimes you have not gone to the lodging you have slept on the grass [green lodge] then something pierced my buttocks before the penis have been inserted so we have to work heard my fellow even if its business you must find a little you work hard to look for money.

**I: Mmh PR08**

PR08: If I hear Jitegemee I feel it’s like how I should be looking after myself without depending on someone I must have a business it is not that I must just engage in sex with someone . I should have even my fellow even if its business, business you must find a little you work hard to find money.

**I: Mmh is there anyone remaining? PR09 yes**

PR09: If I hear about Jitegemee I feel it brings something into my mind that it reaches a stage that a man who is sleeping with you, you can get tired with him (coded language) and if there is no way you can help yourself you have a problem in your house and also your children can also have problem because you are used to if you have not engaged in sex then there is nothing you will eat. If someone has not given you there is nothing you can eat. This sex work can reach a stage that the person whom you engages in sex with is no longer there and you are no longer marketable so this is what brings me the even if it reaches a point am no longer marketable I can get also what I can eat without depending on that my market that can help me [sex work]

**I; I want to thank you for the responses you have given. So again, I am asking that what do sex workers buy with their money, then when we are answering you tell me what you buy with your money and that amount.. They can be a lot of things. So, we want to talk about things that you buy day by day. We will start with that. Okay?**

P: Mmh

**I: Okay who is going to start?**

PR07: If I start am a woman, and I have lacked somethings and I have found someone who want to give me something and I must give him my body [engage in sex] so that he can give me what I want so.it will force me to go there when am smart or clean, because when am clean that’s when I can be attracted to him. By the time am going there when I am smart I will start with the innerwear, I have to have a clean one, I have wear a biker and it has to be clean then look for a bra it has to be clean, I am going to him but I don’t know his status and he doesn’t know my status too. He is a person who want to engage in sex with you and he must give you money meaning they usually say flesh to flesh [no condom use] and the fact that you want to use a cd is a waste of time for him. So he is someone who you are going to meet and if you didn’t have the female condom it will force you to go and buy because you are not going to him the male condom so you are going to buy the condom with your own money since you want to prevent any bad thing in him even if he has the HIV virus may be you may also have it and you don’t know and these things are different strain now you will find a way of preventing it so that you don’t get reinjected it will force you to go and look for the female condoms and in all this you find that when you want to go and meet with this person he can send you fare but sometimes you can even use your own money .it will force you to go and look for it the female condom so that you carry and all these things you find that when you want to go and meet with this person he can send you fare and sometimes you can even use your own fare, you can find that when you go and come back you find that you have used 1000/= in one day it is you who have looked for the money on your own and sometimes he can engage in sex with you the way he wants and the money he gives you is fare only that is enough to take you back and we feel we go through a lot of challenges because I don't have anything and I have gotten someone who has it will force me to engage in sex so that I get whatever he has to be mine.

I**: Number 7 you are right but you have said it right but you have not told us the price of the pant, the biker and the bra then even the condom you have not told us it is how much and even the fare you have said you can use 1000/= right and the money he has given you haven't told us now can you clarify those?**

PR07: Yes, I can say this, the bra can cost me 200/= pant 200 and the biker 350/=

**I: Condom?**

PR07: It is something you go and buy that of 70/= then if you add that amount it is almost the same amount with the transport that is taking you to wherever he is and if you relied that you are going to come back with it has to be over. Now if you add up all these things bra, biker, panty and transport that you have used you will find that you have used your own money and sometimes the vegetable that i am selling I have used part of the money and used as transport and when I am coming back he has given me less money that I had used then my business fails and it will force me to go and look for another client who is also going to misuse you the way the other person did, for me to cover/replace the money I had taken here.

**I: Another person when we are talking let us talk about the things that we buy with the** **money, the things that we can buy daily right she has stated hers another person?**

PR01: As number one I can support number 7

PR07: Yes, I can say bra I can buy that worth 200/=, inner pant I can buy that’s worth 200/=, when I go for biker, I can buy that worth 300/=.

**I: Mmh condom**

PR01: I can support PR07 because (cheering throughout) this my body for me to take care of it and take all expenses so that can be attractive I think the Swahili says *kibaya chajitembeza kizuri cha jiuza* [ Kiswahili saying meaning that whatever is bad will take long to be sold while a good thing will sell very fast ] on my body I don’t apply milking jelly or Vaseline, the oil /lotion that I use in my body I use three types of lotions, and they cost me 800/= and when I buy glycerin worth 50/= and add on it.

**I: Mmh**

PR01: That’s an expense for my body, my innerwear pant that I put on cost 150/= that I want to go and entice my customer, I will look for a clean bra not know the one that I went with on the farm , it is that bra that that can make me to get money. So, if I leave that my fellow, me I have short hair and it’s not that it’s very cheap after sharing it I apply dye even though I have not applied it now. You know that’s money. I use black gel that it’s not 50/= its 150/= I use expensive things, sometimes by bad lack after I have taken care of my body so that I go and meet my customer, unfortunately he says that he has not enough money so you just take this and he has really engaged in sex with you properly. Your body is tired you want to go and get a soap and some jelly and also your clothes have torn so on my side let me say it’s a hard thing for me because life nowadays in my life I don’t apply milking jelly even if I don’t have money so it will force me to go and have sex that I can get jelly that I can apply. So, all these things can cost 1000/= and over, I haven’t included food because you can’t work hungry even your farm you can’t go unless come out of the farm hungry and there is something you can drink to be full You must come out of there with something that when you are back you can eat, I can’t go to that kind of job and come back and take porridge with no sugar or eat *ugali* with vegetable I can’t I went for work. So, on my side its more expenses I support PR07 that’s true I must take specific expenses the time am going to make something that can give me income, that can make me take out this money a little you must have Chama that you can pay you must see how you can feed your children. So, on my side its expensive for me to go for customer for me to get sometimes you get sometimes you lose he got someone else I *don’t* have much to say that I can go overboard g.

**I: Okay first use Kiswahili saying that *Kibaya chajitembeza kizuri chajiuza [*** Kiswahili saying meaning that whatever is bad will take long to be sold while a good thing will sell very fast ] what is the **meaning?**

PR01: Its means that something that is good sells itself but a bad thing do not sell itself you have to advertise it for it to sell meaning sometimes I was just someone that don’t apply jelly on body and I want to buy glycerin to apply on anybody and I want to buy glycerin to apply on my body so that my body become soft even if this person sees me, he sees me like someone soft, I must be someone who can take something that can make my body shiny so that is I must advertise myself to sell myself.

**I: Another one you spoke about you must just eat what do you eat?**

**PR01:** What I must eat, I must eat a certain food that is heavy that can be full for a long time because I can’t go to work on an empty stomach I have to look for milk that I can drink that will provide me with energy that I can go and do my work either after coming back from that work I must find something that I can eat even if I am going while am hungry I must have something that I can eat and that is food ,even if it is *ugali* with something that can give me energy.

**I: This other thing it can’t be mentioned.**

PR01: Its *ugali* even with meat it’s not something (rough on the ground) I can eat *ugali* with meat because I worked very hard [sex work] it’s a must I have to eat.

**I: Another person (silent) you are silent because you don’t shop or do you shop?**

**PR03:** Those things that I use daily, first its food, this food I want to start eating it in the morning, lunchtime and supper. In the morning only I use 350/= lunchtime I use 250/= at night I can use 400/= (birds chirping) and also children are going to school. In the morning you find that a kid wants money that is a must she goes with to school and the least is 200/= [birds chirping] so it’s just the issue of school and food is what I see am using daily.

**I: So, what can be the amount in total?**

PR03: Total can be 2000/=.

**I: Mmh another person (bird chirping) PR06**

PR06: I support PR03 because what we start with is food, we must take breakfast, lunch we must eat, supper also 100/= the important thing that I will buy first is food. The children also are going back to school. One will also say they take want money. Sometimes that’s the money you will take and give her to take to school. Clothes are also a must I buy for myself and the children will also dress so its money.

**I: Total**

PR06: Total can be 1300/=

**I: 1300/= another person, another person.**

PR010: I support my fellows who have spoken the biggest thing I know is for someone to see you , he sees you have made yourself clean and seen how some men fear people, me I do check people. My fellows I do spend 1300/= on a wig to buy and to be plotted so that’s what I depend on in my life because it put my face look smart and =Uyoma =where we are we have some expense that burden us we have a problem of lack of water right now is okay because it is raining we have water problem so when I wake up am a woman I went to work at 0300hr I must have 200/= that am going to use to buy water even if its 5 jerry can when am back I will prepare tea for my children The tea in my house I have 5 children and 2 grandchildren when can take tea that coast 380/= lunch we can use 300/= for lunch if there is food and if there is no flour the season of buying maize, if you buy 2kg right now is 160/= you purchase and mill and buy food already that money will total to 350/= at night you will eat if you spend list amount you use it will be 500/= so I support my fellows and have just added water because we have water problem, you can have food and flour and there is no water and therefor Jitegemee can help us I can find my own money that I can tell even a *boda boda* person to go and fetch for me water at a certain place because I have energy I don’t depend my life on another person and just as my sister have said it can reach a point where you are tired of sex and it might reach a point that no one wants to visit you and when there is money your life will continue..

**I: Who wants to add on for those who are quiet it means that you don’t shop?**

PR02: I want to start with that life has become expensive, love, men don’t look for love it is in the blood (strong feeling) some men don’t even look for compatibility what will do when love is no longer there and life has gone up and food the price is up for you to take breakfast, eat lunch and supper you spend a lot of money.

**I: A lot of money is how much?**

PR02: Morning in my house I can spend 200/= to buy sugar, milk and bread or mandazi escort, lunch is 150/= when there is flour in the house then supper you spend 300/=.

**I: Mmh another person?**

PR04: And how the standard of living has gone up, right now what we consider to be important is flour like what am looking at mostly is flour, how children can feed after finding out on how they can feed I use 200/= in the morning and daytime I spend 300/= because am looking for flour and in the afternoon, I can use 100/= looking for food so when they are added with the ones for school in a day, I can spend 200/=.

**I: Number PR07**

PR07: Am PR07

**I: Sorry you had already talked PR05**

PR05: I don’t differ with my fellows right now life has gone up. Like my house in the morning, I spend 300/= for breakfast at lunch time 350/= to look for flour and lunch when it reaches at night, I spend 400/=how children go to school, sometimes the child going to you want to give some money like 20/= to go and buy lunch at school and after that he will want to see on how they are going to have shoes, clothes for you to buy clothes that is enough for both children you have spent 1000/= meaning the total amount that I can spend is 3000/=.

**I: Mmh PR08**

PR08:I don’t differ with my fellow who have spoken in our everyday life , when we wake up we first start with food me I can use 200/= for breakfast, lunch I can spend 300/= supper I can spend 350/= without including small items for school and school items are also a must a child has sometimes gone to school within no minutes she’s back that they want money sometimes it is 150/= or 200/= so I can’t say with the life we are as at now the amount I can use in a day is 2000/=.

**I: PR09 (cough at the background)**

PR09: In today’s life what I know is when I wake up in the morning we must take breakfast in my house that costs 300/= lunch time with this high standard of living it will force me because maybe there is no food , you can do business ,also right now business is not doing well life is not good, sometimes you can even go to the market and you don’t sell your goods, the people who are the clients sometimes they don’t also give you money, they will agree with you and misuse your body and they don’t give you something meaningful , it sometimes forces you not to prepare lunch in your house whereby you will find the children are also suffering, during lunch time you will find them taking tea that was prepared in the morning and at night it will force you to look for something good for the children to eat. You find in my house sometimes it will force me to spend 400/= for my children to eat well because in the morning they only took breakfast and with an escort so supper they must have something good so that when they wake up the next day they wake up strong and not weak. So, this makes life to be difficult and you find that it is unbearable and what I can tell you my fellow sex workers it is not easy because you depend on it and sometimes you get a client who will spend with you and he doesn’t give you anything [money ].

**I: Mmh. We are done with that. So, we what to look for the items the items that we buy in a week, we buy once in a week which ones are they and how much do they cost? (Birds chirping)** Once a week

P: Once a week

**I: Yes**

PR03: What I see that I mostly use once in a week is making my hair, I can do it once in a week and I can spend around 600/= (birds chirping)

**I: Are you still talking?**

PR03: No

**I: Okay another person. What you do once in a week, the items you spend money on once in a week. [Silent] if there is none you say there isn’t and we can continue. If you keep quiet you know am also waiting for a response.**

PR07: What I can use once in a week is something like soap, bar soap for washing.

**I: Mmh**

PR07: I buy 1kg of bar soap in a week, I want to wash with it. If I buy it on Saturday, I want to wash with it until next Saturday and its over that’s a week. And it force me to use it and this one bar soap only 1kg is 200/= and maybe I buy 4 tins of maize 8kg and take to posh mill one Saturday I want to use it for a week until when it reaches Saturday again I budget for maize flour and for me to buy 2kg and grind it cost 160/= for me to buy 8kg is 520/=it is around 520/=(birds chirping ) 720/= its 720/= those are the things that I must use in a week

**I: Mmh**

PR07: In a week if I add everything 200+720 is 920/= that forces me it’s a must and with business nowadays there is no business you can do and every week you use the money for those two items this is what will make you to go for sex work even if you are going to hurt yourself .

**I: Another person the items you buy once a week?**

PR06: I support PR07 because when I buy1 kg of sugar 1kg in my house It will take one week. If I buy soap I buy it is the same .so weekly per weekly you can’t say you can get money weekly sometimes another week has to pass but you can buy 1kg of sugar and it pushes you even on the part of food. I can eat some food but I can buy even fish or meat once a week I can’t eat them daily.

**I: So PR06 can you tell us the price of meat, food,sugar and soap can be how much?**

PR06: Sugar 1kg 140/=, soap 1kg 200/=, fish when I buy once a week, I can buy fish that costs 150/= it’s enough for me and my children I can buy half kg of meat which is 230/=

**I: Another person?**

**PR02:** I also use 2kg of sugar if I take 2kg I will use it until if I buy it Saturday, I use it until Saturday and bar of soap also I can use like that so sugar 2kg is 280/= soap 200/=

**I: Mmh another person?**

PR04: What I buy once in a week is charcoal and maize flour, charcoal I buy one debe which is 200/= and maize flour 6kg and 1kg is 160/= so I use 500/=

**I: Another person?**

PR01: I can buy cooking oil that is costing 240/=

**I: Be audible.**

**PR01:** Cooking oil of 240/= which I can use for the whole of the week. Starting from Monday to another Monday I can also one bar of soap which I will use for a week and charcoal also I use in a week which is one debe is 200/= and sugar I use in my house is 1 kg of sugar 1kg which can take me for one week.

**I: Mmh are we done? Okay now what do we buy once in a month? (Birds chirping)**

PR010: What I buy once in a month first when I get money I start with sanitary pads even if am back from a journey. This sanitary towel I buy because I use them during my monthly periods and pads right now there are those which cost 200/=. I usually buy the one for 200/= the heavy once so I can buy them if I get money, I can even buy 5 packets which is 1000/=, I buy like that because the periods can come unexpectedly and sometimes when I don’t have money. Its better when I buy once in a month, Now I can use I can make my hair once in a month that I use 1300/=.

**I: Okay another person?**

**PR05:** What I use once in a month, I can buy salt worth 70/= and petroleum jelly worth 350/= and soap I can buy 2 pieces of bar soap. So, all of them soap is 420/=, petroleum jelly 350/=, salt is 70/= and the total is 820/=.

**I: Another person, that thing that you buy once a month?**

PR08: First I plait my hair; I like plaiting my hair once a month.

**I: Mmh**

PR08: My plaiting doesn’t take a lot of money I use 800/= another thing that I can buy is body jelly. I use jelly that cost 400/=.the oil I use for my hair I don’t use it for my body, my hair should have a spray that is 200/= and a soap, washing soap. I like buying omo that costs 170/= and 2 bars of soap which costs 400/= total 1970/=

**I: Mmh another person?**

**P:** If I can also talk

**I: Mmh**

**R:** Most of us women we plait our hair, when I plait my hair and the way I have put Bandika hair style I can go with it for one month and for me to buy these braids I need 6 braids for my hair the time am going to plait I must go for blow-dry and be washed clean am going to use 150/= the 6 braids you find that I will use 350/= (birds chirping) there 450/= has already gone. I want to look for spray. I will take care of my hair well sometimes have gone with it to the market I cover so sometimes when I want to go somewhere or travel that is when I will uncover my hair and spray , spray I will buy 400/= you find you have spent 1000/= in one month that is something that I have not consumed i have only made my hair with It .Then I want to buy body oil I know if I buy jelly that cost 400/= that I apply in my body is enough for a month that is 1400/= have I have used in myself not on food

**I: Another person?**

**PR01:** I can buy 2 bars of soap for a month that will take me for the all month on the side of my hair doesn’t require much I shave box after that I go to the salon and apply chemical of 150/= and then I buy dye known as highlight which cost 250/= that pushes me for the whole month. So, it is like that.

**I: Is there someone who wants to add something, there is no one. So, the things you buy less than once a month example once a year or once a term?**

**PR010:** I can make an African outfit once (birds chirping) ooh shoes also if I find money I can buy shoes, because they are things I can’t buy daily. So I can buy once because when I make an African outfit one or two and the rest I buy second hand clothes I wear those and when it reaches another term and I get money I can buy shoes and inner pant and biker.so those are the things I can buy.

**I; Total?**

PR010: the total for kitenge [African Outfit] is 1500/=let me say 2000/= shoes 700/=, that is 2700/= biker 300/=, 3000/= inner pant 200/=a200 and bra 3400/=

**I: The items that you buy once a year?**

**P:** Once a year or once a term.

**I: For example even in a term or a year**

PR03: Once in a term I have 2 children who are in secondary school I use 2315000= for those two children.

**I: Another one?**

PR04: In one year I do try and buy my children even a pair of shoes or a dress now I can spend 2000/=.

**I: Another person?**

**PR09:** I do try because I have children who go to school, for school fees, I do try every term I use 8000/= for each one of them and for the two children I use 16000/=for my children to go to school. the way school fees is drains me I can make kitenge African outfit once in a year not every day so the clothes that I can make in a year and it costs 1500/= I can also buy shoes worth 800/= for my body what I apply it is obvious I don’t include it in my yearly budget but I do it on monthly budget.

**I: Another person?**

PR07: I use a lot of money in a year we have children who go to school some children are in boarding school. If you approximate in a year you find that you have spent 35000/= for one child, when this child is going every term when she comes back she comes back saying she has no panty sanitary pads and mother my games kit is old, my skirt doesn’t fit me needs to be enlarged. Sometimes you went and bought a new one you find you have spent 45000/= because some you also have other children in the house. The one in primary school his or her uniform in torn he she wants a new one and as a mother you want a clothes and shoes because the time you go to the farm you are also smart you find you have sent 45000/= in a year and those are a must you have to do so people spend differently but you will only talk about yourself.

**I: Another person? [Long pause] when you are quiet it means there is no one with a response. Yes?**

**P:** Mmh

**I: So, you have said that many women who have sex for payment do find money that they use from where? Everyone will respond let me stand.**

**P:** Repeat the question.

**I: Am asking the money you do use where does it come from? Yes number PR03**

PR03: The money we use is money we find the place we find men. Let me say in a bar sometimes have gone for parking and got a client whom I engage in sex with and then he gives me money that what I mostly spend.

**I: Another person?**

PR01: Me too mostly the money I spend I get from prostitution. I get a clients whom spends with me that’s how I can get the money that I use.

**I: Another person? Is there someone with a different response from the ones they have given?**

P: No

**I: There is no one now you want to say that the main source of income that you all engage in?**

PR07:As for me we had talked about jitegemee we will just go back on what we had talked I sell vegetables in the market and when am doing this I will have to get at least 100/= per day which am very sure of when I sell the vegetables and tomatoes I have to get 100/= apart from the one I had gotten .this 100/= since I depend on my own I am smart since am a person who sell food and I have to be clean and you have attracted a client and he tells you that I am in such a such place and I want you to come and meet me and how does he think I am going to meet him I saw you are beautiful and I want to maintain you or I want to boost your business it will force me to leave this place with my own money which means I have made my own money when I go to =Bondo= you know I haven’t received his money, now when I go to meet him it will force me sometimes you get a big penis that you are even afraid of engaging in sex with him but it will force you to do that since you want to get his money now it means I can also fend for myself even if he doesn’t give me and I can also go and I feel I am not impressed/attracted to him because I have my own money and I am proud I will tell him if even he doesn’t give me his money I have my own money. So that is the way it is we go through a lot because we want get.

**I: Mmh, another question that am asking are there other source of income apart from sex work?**

**P:** Yes

**I: I want you to tell me what are they? She has said that she has her business she is running, another person?**

PR06: I sell maize in the market. I can get something small from it.

**I: How much?**

**PR06:** It can give some 300/= daily even if I don’t receive a phone call from a client so as I get a way of buying tomatoes the money I get from selling maize can help me before I get the other one [ money from sex work] that I depend/ rely on.

**I: Another one?**

PR02: I also sell maize so I don’t depend on that one[ sex work] that I must engage in sex work so that I can come back and buy maize even if I don’t engage in sex work I do my business and find a way of putting food on the table. I get some little amount and buy food.

**I: How much is that little?**

PR02: I can get 200/=

**I: Another person?**

**PR03:** Fish

**I: Fish?**

PR03: I go to the lake come back and sell. I can get 600/= per day.

**I: Another person, that’s number 3**

PR04: I can buy banana for sale if I can purchase bananas worth 300/=, I know I will 200/= on top that’s what I get and add with the one I get from sex work

**I: PR05?**

**PR05:** I sell chips and chapati. So, in a day I can get 200/= which I can use in the house with my people.

**I: Mmh PR08**

PR08: I do sell vegetables and tomatoes, my vegetables and tomatoes if I purchase and sell in a day, I can get 300/= which I can add with the money I get from sex work.

**I: Okay PR09?**

PR09: I do sell omena sometimes when I sell it till evening, I will get 250/= which I will find a way on how my children will have something to eat.

**I: Mmh number PR01**

PR01: I sell mandazi and chapati, so by evening I shall have 100/=which I can use in buying maize flour at 70/= before I go to the bar.

**I: Number PR010, What do you do after apart from having sex?**

PR010: I have two sources of income, right now I sell cassava. and a sack of cassava is 650/= the one that is being eaten you can sell at 1100/= and that I can use in a day and my main source of income that these people know is I do sell sun king solar its money that its topped up on me. So, it means this cassava can help me during the day and the lamp if I sell you are paid on commission when you sell you will be given your money on Monday. Every Monday it is something that goes up to Monday ,so this week there is nothing I am going to get that I can feed my children now it means that every week I bring cassava that I use in the house the 300/= that I get I can go and buy maize flour and vegetable. So, the lamp when sell one lamp they are of different type there is one that is 500/= and if you sell it you will benefit 250/= so if you sell even 10 lamps when it reaches on Monday you can get 2000/= there is a period that I sold very well and on Monday I got 3000/= it means that if if I add the weekly and daily income already I will get something like 3500/=.

**I: Mmh. Okay so we have all said other source of income?**

P: Mmh

**I: So, what makes women who do sex for money [sex work] do buy what you do buy? (Bird chirping) what are the reasons you buy the things that you have said the items we had said we buy what are the reasons of you buying them? [ NAO: Noise from the background))**

PR07: The things that we have said that we do buy are things that we eat and things like clothes.

**I: Mmh**

PR07:You buy it the moment you get the money , this means that when you buy it and it is there even if you are supposed to travel urgently and you didn’t have cash to buy a dress or make your hair ,you had already made your hair and it is neat and your shoes are new and smart you find that you have easy time you will go and meet your people [ client without using a lot of money to buy this and that that you didn’t have had already bought them and you have it so it is better you buy it earlier when you have money so that you keep it clean .

**I: Another person?**

PR010: I can say that those are the things that we must have in our own life like human beings. We be ready earlier before something has reaches us. We are ready, meaning in our life a child must go to school, in our life we must eat, in our life must eat, in our life we must dress and these things we have to do them with the money we get.

**I: Mmh another person, PR05?**

PR05: I don’t differ from number 10 who has spoken, its good if you buy those things and keep for example clothes, even if you are to travel it is not that you are going to beg a fellow woman to help you with a dress that you want to go somewhere, now it is better you buy your dress and keep even if you buy your child dress if he or she wants to travel ,you have bought books even when the time comes when they are supposed to go back to school you are not troubled where are you going to get money so that you buy books it finds that you had already put a stock and you just give him/her that is the reason we buy these things to be available.

I: **Another person?**

PR09: The reason why we must buy these things be it food be it clothes. Number one I must have taken care of my body well by eating and having good health number two I have to have clothes even if they are nice ones since I am also marketing myself when am dirty who is going to be attracted to me if I don’t eat well my body will be weak since when you are working hard you have to eat when you are looking for market you have to be clean/smart now these are the things that we must I have I must make my hair ,I have to eat and I must have clothes that when I travel I just go I will not want so and so to know where I am going to. And I would not like to tell someone my movements, that people will be still asking where did I go to where I have gone is none of their business now I would like to have my own things not that someone is going to ask me wherever I am going to or what? So these things must happen in my life.

**I: So, all at your things that you have mentioned how much can they cost?**

PR09: So, when am buying my clothes, I have to buy my clothes, I don’t know how much they can cost the total

**I: Roughly, total, approximate. It can be exact but roughly.**

PR09: Roughly

**I: Mmh**

PR09: For me to have food. food can cost me a lot of money because it’s something I budget for weekly or even on daily basis , daily I will have enough food I must have 1000/=. Clothes is something that I do not purchase daily I must buy clothes and in a year I can buy clothes maybe twice and I will use 3000/=. My travels cost me transport because it’s not somewhere I will go without a motorbike there are places that forces me to go with motorbike. Motorbike right now the price of fuel has shoot I must use sometimes even 300/= or 200/= to and from so they consume a lot of my money.

**I: Another person?**

PR06: You must be someone who is always ready. Because apart from our journey that we go to it can be a very bad thing that when I am leaving I go and tell my other sister that help me with a dress and after I have been helped with a dress they will starting asking amongst themselves why is she borrowing a dress yet she has identified herself as a sex work why can‘t she buy clothes that she can always wear when she is traveling with the money she is getting from those men. We try to avoid those so it will force you to buy clothes, bag and also your children to have nice clothes and they also need to eat very well, you must also buy food because it’s a problem when your children are loitering around why can’t those her clients give her money that is enough to eat with her children. She is paid nothing so I avoid them.

**I: Total, how much money is it?**

PR06: Total the money can be 5000/=

**I: 5000/= there is something you have said *tiaruok…*.**

PR06: You identify yourself as a sex work.

**I: What is it?**

PR06: You identify yourself as a sex work.

**I: Ooh a sex worker. Now you have said the reasons you spend your money on personal things and some have included others too now which items do you usually buy for others apart from you which items are they and how much do they cost? (Birds chirping)**

PR03: Those things that I do buy for others are things like let me say the children who I leave with. I have five children I spend on them something like 3000/= per month when buying for them shoes and clothes.

**I: Mmh another person? Let’s say faster so we move on.**

PR08: The other people I do buy things for are my children the things I do buy for them are clothes, shoes and items for schooling I can spend 4000/=

**I: Mmh another person?**

PR02 It’s only food and clothes for my children I can spend 4000/=.

**I: Another person?**

PR05: My children are the ones I can buy for shoes and clothes and school items total I can spend 5000/=.

**I: Is there……...**

R: I support PR05 its only school shoes and food and hospital expenses is also there, you can a child to the hospital already if we add these things for children and hospital expenses it is at least 6000/=.

**I: Is there anyone remaining?**

R: No one.

**I: Nobody okay, we are asking do sex workers save? (Birds chirping) aah**

PR07: Myself I go for sex work because I had nothing and I went to look for money, engaging in sex you can’t do it today and get another person tomorrow, if I get this week it will force me to save whatever I get because I am spending it knowing very well that tomorrow there is nowhere am going to get it ,I get it today and tomorrow I won’t get. And I want to buy my things one two, I want to eat with the children. If I misuse it tomorrow, where will I get so, it’s a must I save. Sometimes my child is sick am married, and am even better off than him[ husband] as a sex worker even if I don’t have money still am the one he will rely on. And you know I just went for sex work and when I don’t know how to save the money that you I get from sex work where will I get it from, now we have to save

**I: Mmh another person?**

PR010: We must save because everything even the child is from school even if the father is there, for us who are not married we can say that they have to come to us directly because we have no husband, but when a child comes from school she goes directly to her mother even if the father is there she will just go direct to her mother So, we must save because we have children, sickness can come unexpectedly, anything can come up anytime..

**I: Anything like what?**

PR010: It can be sickness.

**I: Mmh**

PR010: Eeh even any problem even the lack of food so, you must save something even if its little at least you have saved something like 2000/=.

**I: Another person?**

PR06: I must save because if yesterday I got something good I don’t know whether I will get today or tomorrow. So, I must save a little that even if a child sick I can take to the hospital death can sometimes be unexpected and I have I received a phone call that my mum has died I won’t go for sex work for me to look for money to travel with I will take my savings and go so we have to save..

**I: Another person?**

PR09: I must save because the way I do small business like selling omena ,when I go to the lake it is something that sometimes I don’t have enough money. The weather is not favorable and omena was not well sundried So, you can find that my money that I had saved it’s a fall back for my omena until it goes up, that way it’s a must I use t small amount of money and another one I put in stock so that when I am down financially I find how I can boost it and it can also help me, sometimes a child have been sent back home from school I find something small to give the child to go back with because I don’t get it daily. I get something small I take her back and I go and talk with the teacher then again tomorrow if I get some money and I find a way of taking the child back to school.

**I: Mmh.**

PR03: I must save its only that the kind of saving I am doing might not be of great help to me because I can save weekly, and for example like today, they want that the money I have saved I wait till month end that is when I can take it , you find that I am saving but this month in case I have any problem this month it means that I am like someone who didn’t save because I can’t sort out the problem I wanted to so I have to wait for a month

**I: Mmh but you do save?**

PR03: Yes. I save.

**I: Is there someone adding? So, on top of the saving that you have said, all that you save, how many times do you save? It’s something that often happen how many times? If it’s monthly, weekly, is it daily or how is it?**

PR03: It’s saving like me PR03 on my side I do save weekly

**I: Mmh another person?**

PR02: I also save weekly.

**I: Weekly another person?**

P: I do save weekly.

**I: Another person?**

PR08: I do save weekly.

**I: Weekly another person?**

PR010: Weekly.

**I: Weekly.**

PR01: Weekly.

**I: Weekly Mmh the remaining people you said you save; you save after how many days or do you save?**

PR09: I also save weekly.

**I: Weekly.**

PR06: I do save weekly.

**I: Weekly.**

PR07: Weekly.

**I: Weekly. It seems everyone saves weekly, Okay?**

P: Yes

**I: So, can you tell me how much you do save weekly? We will start with here number PR01?**

PR01: Weekly, I want to collect my small amount of money for chapati and mandazi so that they total to 400/= I save weekly.

**I: Now how much do you save?**

PR01: I save 500/= weekly.

**I: 500/= per week, PR010?**

PR010: 1200/=

**I: 1200/= number 3?**

PR03: Per week, but it depends I can say I put 500/= and sometimes I don’t get that 500/= so any amount I find in that week I save.

**I: How much is it?**

PR03: Starting from 100/= to 500/=

**I: Mostly how much do you save?**

PR03:500/=

**I: Eeh PR04?**

PR04: I do save 200/=.

**I: 200/=, PR05?**

PR05: 300/=

**I: 300/=, PR02?**

PR02: I save 200/= per week.

**I: PR06?**

PR06: Me I do save 500/= week per week.

PR07: 700/= Weekly.

**I: PR08?**

PR08: 300/=

**I: PR09?**

PR09: I do save 250/=.

**I: Okay so am asking is their character that is known that women who have sex for payment that don’t save have? (Noise in the background) character that women who have sex those that don’t save is there any character that they do have that you know?**

PR07: There is a character

**I: Tell me.**

PR07: She get even 300/= per day and she can’t save even 50/=. If she gets 300/= she uses the all amount there is a difference in that. She’s a woman that even if a man comes to her and have 20/= she will give out her body (laughter in the background)

**I: Aah.**

PR07: She will give out her body because for her to get and she want to get daily without getting and saving so there must be a difference it’s been said that her daughter some even sleep with you without paying because you don’t have and you expect to find and also you have come out empty. So those are there showing out that you are not saving because someone saving is someone who has pride you give me don’t give me, I have, I have gone with or not I will find.

**I: Mmh another person attitude?**

PR03: Another attitude a person who doesn’t save always borrow. I: Always borrowing, another person?

PR09: A person who doesn’t save there is a time she can want a man by herself even if a man does not want her. Because for her to think what that person is going to give her that person is going to give her, that person is going to give her money.

**I: Mmh**

PR09: So that also happen on those who do not save because she does not have and she is thinking how she can get so she thinks when she jokes with that man for him to agree and engage in sex with her for free and they don’t give her anything.

**I: Mmh, is there someone who wants to add?**

PR02: I support all those words.

**I: You support all those worlds (participants laughs at the background)**

PR01: I also support all those words.

**I: Okay, and is there any character known for sex workers that do save have? That they do have character what is it? We have talked about those who don’t save and now we are talking about those who save what character do they have?**

PR07: (Participant cheering through) a woman who have sex and save its some who has pride. I got last week I have done shopping if I look my bucket there is flour, there is soap, sugar is there if I look milk carton there is milk and salt also, I have. Even if you follow up to my house thinking that I don’t have I will follow you for 50/= you find that that I save and I have something even if you don’t give me

**I: Mmh, PR05.**

PR05: I support PR07 who have talked. But I am adding by saying that you won’t have a desire that you desire even someone to go and engage in sex with you. Because you had what you had saved and it is going to help you.

**I: Mmh PR03 wants to talk (participants laugh)**

PR03: It has disappeared, the character that someone who save has is that she has a good plan even if she has not gone for sex work, she has not found client her children can still go to school because she has saved a little that she can take her children to school.

**I: Mmh. Number 9.**

PR09: A person who do save if she has something even those who want her, who want her anyhow want to misuse you. I don’t know how to put it, just wanting to engage in sex with you is not easy even if he wants to approach you that he wants you he will have to think twice when he is approaching you because you are somebody who is very choosy you are not somebody who is just approached by anybody then he engages in sex with you. He knows very well that it is not easy for him to approach you because he knows that you have even if he wants you, he must think fast. (Participants murmuring)

PR010: That people who save are those people who are looked up to. Another thing a person who saves is someone who people think that they are of high standard but you are just average ,but you are taking care of the little things you have bought very well ,saving is like the things we buy e.g. clothes or shoes for example you can buy shoes and dress at the same time with someone but you will find that the other person’s items get worn out very fast and yours that you have taken care of very well now they are people who people respect and even if someone wants to approach you they are afraid and it will force him to come when he is afraid.

**I: How are they looked up to?**

Pr10: (Participant murmuring)they have respect Is not that if you want go to so and so and so daughter you will find her , but for you they fear you they say [mentions name ] is someone who does not just respond to people is a woman who has pride will she hear you , we have value.

**I: Mmh Okay.**

PR01: Second one if I add on saving, the reason who you won’t be an easy going person for a man the way he wanted when he has come and finds you have everything and also when he come, he must tell you why he has come and how he wants it to be then you will look at him and look down upon him and you tell him your price. Because when he looks at you, he sees you are worth the price. You will tell him the price that he can’t afford it will demoralize him the he will leave you secondly you tell him the price then he feels like that he wants to but he is going to try very hard so that he goes and gets the amount you told him for him to get the way he wanted [sex] and that is when you will be full of pride because you have increased your saving because he has brought much and you also had more, now you continue looking down upon and despising him them as you move on.

**I: Okay, we have said those things that makes us save yes?**

P: Mmh

**I: We save so that when if a child is sick you can take to the hospital even if you are sick you can go to the hospital. You want to boost your business you want to pay school fees, you want to buy food. Yes?**

R: Mmh

**I: Now, what makes it easy for you to save?**

PR03: It’s easy for us because you know things that do come abruptly like emergencies, we can’t do them if have not save, eeh they help us in situations like emergencies.

**I: Mmh another person?**

PR07: The reason why I will save I have children, my child will go to school, I am called from the school my child is sick to go and pick him and take him to the hospital. When I reach the hospital, I find there are no medicine it will force me to go and buy. I had my money that I had saved secondly most of sex workers like us I am not afraid to say we are people who are on medication ARVs. Sometimes you go to the hospital, you are given drugs and when you reach home the pharmacist did not check the expiry date of the drugs he/she gave you and you realize this when you have reached the house,. It will force to go back to the hospital sometimes you are going =Kabondo= it will force to go since it is your health and in case you didn’t have some savings are you going to walk and maybe he is the one who realizes that the drug he gave you he was not supposed to give you know that is an emergency and if you had saved you shall overcome that, so that’s why we save.

**I: That’s the reason why you do save. But now what makes it easy for you to save? (Participants murmuring)**

PR010: It’s easy for me to save because there is something that I do [source of income]

**I: Mmh**

PR010: That I depend on that’s mine.

**I: Another person? What makes saving easy for you?**

PR08: Saving becomes easy for me if I have something that I can save at least on daily basis I can save.

**I: PR06**

PR06: Me too I don’t differ with PR08and PR06 the reason it will be easy for me because I get some little money daily so it’s easy for me to save a little by little in case there is something that has come up I can find where I can get before looking for another place.

**I: Mmh**

PR07: We had agreed daily we can save there are even people who can save 700/=, sells from my kales and I get 50/=, Onions I get 20/= tomatoes profit is 30/= and the total is 100/=, I have the money that am going to save daily I have it in my phone that’s where it is.

**I: And now is there any challenge that we can have when saving**?

PR07: I can have difficulty when I wanted daily to save 100/= and when I go to market there are no customers that even the 100/= I have got it’s only for that time budget and the one for saving is not there. You find sales are low the ones that I can only use and not save.

**I: Mmh another person? The difficulties we face when we are saving.**

PR05: The difficulty I can face, sometimes I expect to save 300/= weekly and now that there is no money, sometimes I get 50/=, so there is no way I can save that is the challenge I can have.

**I: Another person?**

PR03: When sex work is not booming you don’t get customers well that’s a challenge.

**I: Is there someone who want to add something?**

PR01: I am supporting what my fellow has said.

**I: What have they said? (Participant cough)**

PR01: How they have said? Or should I say mine?

**I: Even how they have said, what have they said?**

PR01: They have said sometimes the number of customers can go down and it can interfere with your savings. Sometimes even when you go to the market you think maybe you sell 100/= per day. Sometimes the market goes down and you find you leave 50/= it will force you to use that because there is no way you will save. So that makes saving difficult.

**I: Okay, so how can we overcome these issues? So that even if you got 50/= because you thought you were going to get 100/= and you get 50/= what can we do so that we overcome these issues? (Noise in the background bird chirping)**

P: Repeat the question.

**I: Am asking, you have said the challenges that you face to save now the challenges you have mentioned how can we overcome them?**

PR010: like now jitegemee has made us to be one, what can make us change this is when we unite and we can even have our group if today I sold and the market was not good but there is this 10/= that we collect and this collection can make me to have something to save that was depleted that is how I feel [.birds chirping]

**I: Mmh another person what do you want to do so that when I was expecting 100/= and I got 50/= what should do I do so that I get that 100/=**

PR07: Ooh Because I had already known that am someone who can rely on my own, it will force me to work very hard because the issue of buying maize flour is what makes me not to save ,it will force me to have a farm so that I can haves maize if buying maize would make me to spend 500/= which I was saying about buying maize there is a way how I can divide it and then save because I am not buying maize I also have a kitchen garden that I get vegetable from instead of what I sell goes to buying vegetable, omena or small fish, I have vegetable that I can eat and the money that I would have used in buying small fish I shall have saved it.

**I: PR06 Do you have something to say?**

PR06: (Bird chirping) what can help me, we can have small group that when it reaches in the evening everyone contributes 20/= and it is given to one person (noise in the background) and in case you had nothing to contribute then that day you have even received 300/= from your fellows and you take 100/= and buy food and then take 200/= and save so that way it can also help us.

**I: Okay, so we are done with this part?**

P: Mmh.

**I: So for sex workers who don’t save? What do you think are the reasons they are not saving? (Noise in the background)**

PR07: There is someone whose likes eating when she gets money today, she does not think about tomorrow. (Participant murmuring). I have it now, and this is what I lacked and I have gotten I want to eat and don’t think about tomorrow I don’t know the truth is that tomorrow I don’t know about tomorrow and sometimes she completely has nothing she eats a lot that whatever she gets it is finished .

**I: Mmh**

PR03: Taking alcohol.

**I: PR03**

PR03: So, when she finds she goes and take alcohol she drinks until she does not know the amount she got. A lot she saw was a lot before she took alcohol, but after taking alcohol she finds out she does not have she spend it all on alcohol. So, there is no a way that she can save.

**I: Mmh another person?**

PR05: If I add there

**I: Mmh.**

PR05: Sometimes she found someone who want to have to have sex with her and he had sex with her on the grass and he want to give her 50/=

**I: Mmh**

PR05: So, this 50/= also she is thinking what to do with it so what she will take and save is not there 50/= is what she is going to use at home.

**I: Mmh another person to add?**

PR09: Women who have sex for payment and sometimes what hinders them to save is that sometimes someone is like that and she has not looked at money that she is been given can help her. What she is busy with sex in that when she finds someone who can have sex with her.

**I: Mmh.**

PR010: Am adding there that lack of plan. When you are in a house, when you wake up in the mourning. Me myself you wake up knowing you have this amount of money if I go and find this amount what can I do? So, she’s someone who wakes up and does not have plan. (Birds chirping) if there is a plan you can’t lack saving.

**I: Mmh.**

PR010: So, she does not have plan she wakes up how she has wake up that’s how she will be.

**I: Mmh. So, what disadvantages are there when someone does not save? (Noise in the background)**

PR07: The disadvantage that are there if someone does not save is that you have problems all time you beg all time.

**I: Mmh.**

PR07: That even if someone’s home, the person says that borrow is what is taking you there and you did not go to borrow meaning you have been known as someone who always beg you don’t know how to save.

**I: Mmh, another disadvantage when someone does not save is what?**

R: Another disadvantage when someone does not save a child is sick how you want to start is not there (birds chirping) because there is no where you can get help. If will force you to go to your fellow and the child is not well for you to get help. It will force you to go to your fellow and the child is not well for you to get money the child can die.

**I: Mmh.**

R: So that’s also a disadvantage

**I: Another one? (Noise in the background)**

PR04: Another one if you don’t save and the child have been sent home from school. Even if he was a brave child and all time, he is at home other children are learning and he is at home because the money to take him back to school is not there so you find the child lagging behind every time.

**I: Mmh another one?**

PR09: Another disadvantage being that you don’t save you don’t have even a little that you save all that you don’t save you don’t have even a little that you save all that you get you spend, it does not bring development for you yourself even your home even your children because there is none even that you can pay for your child school fees. Everything that you can do that can develop you. You are always on you foots the way you were yesterday; is the way you are today and the way you are tomorrow. There is no development in you, you can’t go ahead you can’t make a step.

**I: Making a step, can you give me an example of making a step?**

PR09: Like yesterday you had one hen.

**I: Mmh.**

PR09: It’s supposed that this hens increase in number. Me if it was one, that one if something goes wrong [emergency] I take it to the market and sells it, so I will remain with nothing. And if I had something to lay my hands on I will leave my hen and it will lay 10 eggs and if it hatches, I will have 10 hens lay 10 eggs and if it hatches, I will have to hens plus its mum am having 11 hens.

**I: Mmh.**

PR09: In case of any problem I will sell one and remain with 10 hens so that means I don’t grow am just constant.

**I: Mmh. Is there someone who wants to add? None so what are the advantage that someone can have if she does not save?**

P: Nothing.

**I: No I want you to respond what are advantages someone can have if she doesn’t save? (Noise in the background)**

PR07: If you don’t save there is no advantage.

**I: Mmh**

PR07: How can you have advantage and you have nothing? And you are always saying that you don’t have so what advantage do I have there is none.

**I: Mmh.**

PR07: There is no advantage for someone who does not save.

**I: Mmh.**

PR07: Advantage that someone who does not save is poverty. (Participants laugh)

**I: Is poverty an advantage?**

PR07: We can say it’s an advantage to her.

**I: Mmh.**

PR07: We can say it’s an advantage to her.

**I: Mmh.**

PR07: Because she does not save (participants laugh)

**I: Ooh.**

PR07: The advantages for someone who does not save is that someone can’t come and auction her things that she borrowed money from her

**I: Mmh**

PR07: Because it’s a loaning and saving.

**I: Eeh**

PR07: It’s been saved and it’s been borrowed. So, after borrowing and I can’t pay.

**I: Mmh.**

PR07: So, she is good position me am being asked to pay me debt which I borrowed but her she is not being asked to pay her debt (participants laugh)

P: You have said the truth.

**I: Okay, you told me you do save weekly. Everyone will tell me where she does save, number 9 where do you usually save?**

PR09: I do save on my small group.

**I: That group is a Chama or?**

PR09: Is a Chama.

**I: Aah, PR08 where do you save?**

PR08: I save in Chama.it is a group meeting [kosalo]

**I: Group meeting number PR07?**

PR07: I save on my phone because group meetings also have made us not to understand you have saved and another person comes that does not save and yours that you have save, she takes and after taking you will not see her and she defeat you. So, I save mine on phone (participants laugh)

**I: Mmh PR06?**

PR06: I save in group meetings.

**I: Group meetings. PR02?**

PR02: I save in group meetings.

**I: Group meetings, PR05?**

PR05: Group meetings Chama

**I: Group meetings, Chama, PR04?**

PR04: Group meetings.

**I: Group meetings, PR03?**

PR03: M-shwari.

**I: M-shwari, PR010?**

PR010: I bought another line.

**I: You bought another line? (Participants laughing)**

PR010: my child knew my pin and he stole from me.

**I: Mmh.**

PR010: So, I want to buy another line, sim card because for large saving I save in the bank and small once for vegetable I put in my phone like the one I had. So, I want to change the line and pin.

**I: Mmh, PR01?**

PR01: I save in group meetings.

**I: Group meeting. All those that save in group meeting [kosalo], tell me the benefits of saving in group meeting number one.**

PR07: Me saving in group meeting I cannot say there are benefits. There are no benefits and there are benefits. Everything has advantages and disadvantages Because I can go to save money in group meeting until my money reaches 15000/= and after reaching 15000/= you find out that some who people did not pay their debts and my money will be less or sometimes I come out with nothing may be I can’t pay my debt and the money is deducted and am left with nothing and its advantage is that I saved well and people are sharing out after one year or after 6 months mine runs for a whole year if I saved well and got interest I can come out with something good [ reasonable amount] and make a step and do something else. I can’t say that group meetings are 100% good I have seen the disadvantages and the advantages.

**I: Mmh and that’s where you save?**

PR07: That’s where I save because there is nothing I can do. I have not found another group that I can join and have something we can do or have a new idea.

**I: Mmh**

PR07: Am just there because there is nothing I can do, I can’t save my money in the purse I just have to save it group of people.

**I: Mmh PR03. You save in group meeting?**

PR03: Mshwari

**I: Mmh, why?**

PR03: The advantage of saving on Mshwari is there is no one with my money.

**I: Mmh.**

PR03: I see my money every time even if my account has 200/=. It’s just that 200/=that am will be able to see (participants laughing) that’s the benefit it has. I don’t quarrel with people that so and so borrowed money and the money is going to be shared out and that money will I really get it?

**I: Mmh.**

PR03: That’s what I see is good for me. The time I want to borrow am allowed to borrow.

**I: Mmh.**

PR03: So, I see that’s easy.

**I: PR010 you said you save on Equity. Why do you save on Equity?**

PR010: Equity is good for me, at first, they gave us line and now is again change in a way of dialing *247 and I check what’s in account. Anytime I want it can work in Mpesa even if I want to forward for you. Money today I want to send [mention name] something I can send you and still know what has remained [Participants laugh]

**I: Okay PR04? Group meeting?**

PR04: Yes

**I: Mmh, why do you save there?**

PR04: That’s where I saw it fit I can save because I did not have a place to save.

**I: Mmh.**

PR04: So, the reason why I choose group meeting the time I still don’t have money that I had saved a little amount I can borrow or lend me 3000/= my child has been send back home for school fees they give me and I take back the child to school. And I will pay it back in instalment.

**I: Mmh PR05, you also save at group meeting, why?**

PR05: The benefit I see at group meeting.

**I: Mmh.**

PR05: I don’t differ with PR04 who has spoken.

**I: Mmh.**

PR05: It can reach a point you have got problem that time you go to your fellow and tell them to give certain amount of money to take my child to school and they will give me and I will pay later.

**I: Mmh PR02?**

PR02: Group meeting is good and bad.

**I: I want its benefits.**

PR02: Its benefits that I can say.

**I: Eeh.**

PR02: I support if you get problem, you can’t go to your fellow woman that time to give you a certain amount of money. And group meeting is something that if you go find its only that paying back is difficult for us that’s why it has disadvantage.

**I: I wanted to ask you, these group meetings I have saved, Okay?**

PR02: Mmh.

**I: If you have a loan can, they again give me another loan?**

R: No, they don’t give you.

PR02: If I had a loan of 3000/=

**I: Eeh.**

PR02: I take 3300/= and pay back and they give me 6000/= that I wanted.

**I: Ooh.**

R: If I had problem am going to solve.

**I: Now I want money, Okay?**

R: Mmh.

**I: And I don’t have this money, how are they going to help me?**

R: They won’t help you.

**I: They don’t help me.**

R: If you have no shares.

**I: Okay**

PR010: The way we do it, the reason why we go to the leader. I had a loan for the group meeting 10000/= and today I have woken up with problems in the house worth 15000/=. I will come to your house; my fellow woman just helps me with 10000/= am going to refund you right now am going to come back right now and give you. And you have given me that 10000/= and I remain with 5000/= this 15000/= is going to incur interest up to 30000/=

Participants laugh)

**I: That’s group meeting?**

R: Group meeting.

**I: Mmh PR06 where are you?**

PR06: Am in a group meeting.

**I: Mmh,** (participant murmuring)

PR06: That’s where I save because there is no where I can save.

**I: Mmh.**

PR06: I save there that it helps me sometimes maybe I want to plough it is time I don’t have a place to get it. I know on Tuesday am going to get money and Wednesday in the morning there is a tractor at the farm ploughing

**I: Mmh.**

PR06: Even if I had a loan of 5000/= I start looking for it the previous day so and so help me with 1000/= so and when it reaches on Tuesday that we are going for the group meeting I shall have gotten the 5500/= [Birds chirping) (Participants laughing). I go with it if I wanted even 800/= that tractor work with 3000/= I will raise my hand that I need 10000/= and I say have reduced the amount give me 8000/= the tractor will start ploughing tomorrow.

**I: Mmh**

PR06: So, the day for paying back it is only God knows [meaning she can’t say when she is going to pay back the money she borrowed] (Participants laughing)

**I: PR07?**

PR07: I said I save in my phone, my money is in my phone.

**I: Mmh.**

PR07: The reason why I choose my phone.

**I: Mmh.**

PR07: In case am not around I went home am from =Sakwa=, I went to =Sakwa= my child is sick, sometimes I have stayed for long or I did not know that I was not going to come back when I have money in this my phone for money to be in my phone it means that I save money in Mpesa. I also know the mpesa agent or I can call the mpesa person and tell her my child is coming and I have withdrawn the money my children will not go hungry Am in Uyoma and my mother is in =Sakwa=, she is sick, in the group meeting is that if I took money 8^th^ of this month, if we go to another month that’s when I can go back and get money meaning I have stayed with it a month. I take that money with the interest. The month has not yet reached and I have an emergency, I have my money in my phone it’s going to help me before the one for group meeting. So am here and I say if mother is sick let her prepare and go to the hospital am sending money. Am here and I have helped there so that’s why my phone is good for me than the group.

**I: Mmh, PR08 what’s good for you?**

PR08: Am in group meeting

**I: Mmh**

PR08: Because that my money I can’t save when I save it myself, I can easily access it and if I save it in group I can’t access it easily I will budget with it for future.

**I: Mmh.**

PR08: That’s why I save at group meeting.

**I: Mmh, PR09?**

PR09: Am saying that me am not in a group but in one that is known as Silk there we save every week ,every week this money I am not allowed to save more than the other members we contribute the same amount we contribute one week that is 5 days it is 250/= we contribute the same amount and there is no way of you being ahead of other members, they have allowed me to start saving the minimum amount being 50/= and if I save this 50/= they will calculate it with 2 when I want to withdraw, so it is upon me to be saving 250/= every week in case this week I have borrowed 500/= it is easy to pay the 500/= the following week so that I can borrow another 500/= now I am going to borrow 1000/= because they are going to multiple it by 2 so it puts me in a position of not borrowing a lot of money I borrow manageable amount and sort my problems I save and borrow manageable amount That’s why I joined this one, that people contribute the same amount that someone has taken a lot of money Me we are in group meeting limit of wanting money is 100/= and the maximum amount is 1000/= that’s someone who has contributed more and this person is going to contribute her 1000/= But for me I contribute 100/=every day when I go for the meeting I contribute 100/= and they have also allowed me that I can borrow a lot of this people’s money and in the end they if it reaches the time they check my share and it’s a little where am going to get money from so as to pay them back is also not there that’s what made me to leave group meeting[ kosalo]. I borrow manageable amount of money that is enough and use it on my business and I pay back weekly. (Birds chirping)

**I: So, this silk.**

PR09: Mmh.

**I: What is it in full?**

PR09: I don’t know how I can remember it.

**I: Mmh. You can’t remember.**

PR09: I just know it’s silk.

**I: Ooh you just know it’s silk.**

PR09: Mmh.

**I: Okay, another question is asking do sex workers live a life that is expensive than their income? The money I get is little okay?**

P: Mmh.

**I: And the life am living is where?**

P: At the top.

**I: Does this happens?**

PR08: It can’t.

**I: It can’t?**

PR08: Because there is no way my life will be expensive than my income.

**I: Mmh.**

PR08: My income will dictate the life I want to lead

**I: Mmh, the other people what do you think? That’s PR08 who have experienced that and others what have you seen?** (Participants murmuring)

PR010: I support her.

**I: You support her Mmh.**

PR01: I also support.

**I: You support as PR01, another person who has something different from what PR08 has said to tell us.**

PR07: I won’t differ with her; I will support her because the reason why my life will be expensive is not that am a sex worker or even if am sex worker there is no way my life will be expensive it will be in the same

**I: Mmh.**

PR07: With the income I get even if I have sex.

**I: Mmh.**

PR07: Mmh.

**I: Okay meaning you don’t live a life that is above your income right?**

R: Mmh.

**I: You just live on you’re what?**

R: Income.

**I: Your income, so am asking sex workers borrow money or find themselves on other debts? Do you borrow money? Is not that you are the one giving out the money but , you have gone and borrowed money somewhere if you want to use right?**

R: Mmh.

**I: Do you do that?**

R: We do.

**I: Okay you do, right?**

R: Mmh.

**I: The reasons for borrowing money? (Birds chirping) everyone is going to tell me the reason she borrows money. (Bird chirping)**

PR09: Sometimes I have shortage (Papers chirping) a child has been sent back from school.

**I: Mmh** (Birds chirping)

PR09: My sexual partner we have agreed he is going to get me money and it is not there, I have gone and borrowed money because he told me just go and borrow somewhere so that you take the child back to school, I will see what to do.

**I: Mmh.**

PR09: Because he is my hope, and the little amount that I get sometimes it just enough for the house. Expenses so, this makes me sometimes I must go and borrow.

**I: Mmh.**

PR09: Because sometimes my hope was in him and he assures me that I go and borrow and we will find a way on what to do.

**I: Mmh.**

**PR09:** He had agreed we borrow.

**PR09:** Sometimes my Merry go round he told me to go borrow.

**I: Mmh**.

**PR09:** I want and borrowed at the Merry-go-round.

**I: Mmh.**

**PR09:** So, for me to find out how am going to pay I have difficulties.

**I: Mmh, So, what do you do pay that debt?**

**PR09:** For me to pay the debt.

**I: Mmh.**

**PR09:** I am selling sardines. Now I will pay little by little until I clear that debt.

**I: Mmh, PR08 you borrow money to do what with it? Who do you borrow? And how will you pay it?**

PR08: If I borrow, let me say there is something I want to do that its cost much that I can’t get it once.

**I: Mmh.**

PR08: Depending on my income I must go let’s say mostly I borrow from Merry-go-round let’s say like here in =Uyoma=, here in =Uyoma= we buy soil for plastering house, it’s not somewhere you can go and scoop soil and go and plaster a house. You have built and you want to plaster the house. Soil a lorry is 6000/= and I need two lorries that is 12000/= I will go and borrow from the group and smear my house with the vegetable am selling and sometimes I get money from sex work and I will add them and pay little by little to pay the debt

**I: Mmh number 7 the way she has said you do the same where do you borrow money from, what do you want to do with it and how do you pay it back?**

PR07: Yes we do borrow I said that I don’t borrow from Kosalo I save my money in the phone when I am saving in my phone it is easy to access mshwari my business is not doing well and I need money to boost it and may be someone is sick and I had used the money so it will force me to borrow from Mshwari in order to boost my business. I know I am supposed to repay by the end of the month so it is upon me since I had put the money in the business it has to bring me profit I will still go back to my daily savings the 5000/ shillings that I borrowed I have it and I will able to pay back and take another loan.

**I: Mmh. number six**

**PR06:** As number six.I save in the group I can borrowfrom the group if only I have deficit like if I want plough, when a child has been sent out for school feesI will borrow from the group so the business that I run is the one that I will use to pay back the loan plus my main source of income [sex work] when I’m given some money, when I am in debt I don’t misuse the money but I will try to pay back the loan right.

**I: Mmh……. Number two.**

PR02: I also borrow from the group as I give out example, sometimes this business is down so I want to boost it , I will make sure I pay the loan bit by bit by working extra hard .

**I: In that business of yours, how do you work extra hard?**

PR02: So, I will be going to sell daily to get even a little amount and add on the one I have been given from job[ sex work] I case someone gives you 500/= or 1000/= you add with the one you had .

**I: Yes, number five**

**PR05**: I also don’t differ with my colleagues,I also borrow from the group when business goes down, and I want to boost my business I can borrow money from the group or when I’m in need of school fees. When I want to pay back the loan, I will take from my business and use and also from my clients I will try and get something small from them in order to find a way of paying back the loan.

**I: Yes, number four**

**PR04:** Even me I always borrow from kosalo when there is something I want to do and its costly I will go to the group and borrow loan. So, I will be selling my banana and paying back the loan.

**I:** **Number three**

**PR03**: When I want take a loan from Mshwari whereby when I’m in need of school fees, I borrow 15000/ which will go for one year. So, the little I will get from sex work and whatever little I get I pay back the so that I will borrowagain

**I: How will you pay back the loan?**

PR03: I will pay back using my savings there is a way of paying back to Mshwari.

**I: Mmh……number 10**

PR10: I borrow because of hunger when am hungry there are some people here that don’t borrow. But with when I’m hungry, I go and borrow something like five hundred and fifty. I can use this money this money to buy six kg of maize and do some 1, 2,3 in the house. So, when I take a loan today, the loaners will start taking it tomorrow. So, when you fail to pay like today, you can tell them that you don’t have so it’s upon you to pay 100/ in the following day. So, what drives more into borrowing is hunger, I really fear hunger because it can cause certain diseases and there are some people who like borrowing in the community in that if they realize you have, they come saying “So and so please help me. These people really annoy me. They will also be saying that you have money from loan so they want you to give them. Not knowing that if you borrow amount like 550/ you will repay for 10 days. When you are done but it is hectic but also it keeps you busy than borrowing a lot of money that I will not be able to pay back

**I: So, when I’m given five hundred what the interest?**

**PR010:** 60/ per day

**R:** Until I will complete the loan payment.

**PR010**: Yes. Until the day I take them the 500/=

PR10: 60/ shilling for ten days they give you 500/= and then you pay 600/=

**I: I was thinking that when I’m given 500/ I will be paying 60/= per day for 10 days**

PR010: Yes.

**I: Okay Number one you have not told us why you took loan, (laughing from background). We want to know what you did (how you spent the you borrowed.**

PR01: Even me, I have a different opinion with my colleagues who borrow, I taken loan from (kosalo), this where I can go and borrow money when I’m in difficulty situation, this is when I can borrow money. Like there is part of my house which was in bad condition, I can make renovate then I will be repaying back the loan bit by bit through my savings. When my savings are not paying back enough, I will go to the field. [Sex work] and when I take from Sacco that is the loan that that is not helpful,and it helping me I mean when my child is sick, I can go and take four or three thousand to help in paying medical bills. So, when the child recovers or not is not a problem the fact remains that I have a loan of a certain amount, this child is epileptic and she is a baby girl,now after borrowing the money I will pay little by little with my small business In case I have paid the loan and it is not enough I will do sex work until I pay that loan till it reaches a point I clear the amount I had taken.

**I: Okay, sorry for the sickness of your child.**

PR07: Mmh.

**I: Then I again ask what other jobs sex workers do to increase their income, tell me. Yes, number one**

PR09: Number 9 what we always do to increase our income. One of them is I have to be neat. You must keep your body clean. You should be that person who have what to eat, a person having food. Should be the person with airtime on mobile phone on mobile phone, I should be able to call or be called. So those are things we try to do in our life.

**I: You have said that you must eat well, dress well and you also have credit in your phone. How does all these increase your income?**

**PR09**: the areas that it increases my income is that I should dress well is for the customer to be attracted to me I must have credit in my phone in order to communicate with that person who is in need of me. I must eat well for my body shine and glow. You know if you do not eat well, you will lack some nutrients in your body. So you should be someone who is neat and eat a balance diet. Plus, credit for communication so that you can get what you want.

**I: Okay number another person, what do you do to increase your income?**

**PR07**: As number 07 for me to increase my income I should be having something in my hand every time. I’m a business woman and I prioritize my business to sex worker. Because sex work, you can’t do every day.but my business I do it daily. And again, I should be in a position to eat what I want not what I don’t want. Even if it is that I want to put on a certain dress it should be neat even when my customer wants to meet with me, I should dress neatly in that if I go to him, I am presentable not somebody who has to beg to be smartly dressed..

**I: You people have not understood me. What do other things do sex workers do increase their income, like somebody like said she always get only 100=/ now if I usually get 100/=.What do you do to if raise that 100/ to 200/ or 300/, that’s the question, so answer me.**

**Pr07:** I have now understood you for me to increase my income to be something big. In a week earlier on I said I can save 700/ shillings can go and purchase fruits like orange, banana, meaning this 700/= I have already invested in banana and orange, I will also purchase vegetable and onions. So, I will get profit like even 150/ per day or 200/= per day .

**I: Okay any other person, what do you do to increase your sales, Are you people tired?**

**PR05**: Like me as number five, like I always sell bajia and chapati, I have to increase what I have gotten from chapati so whatsoever profit I get from bajia and chapati I must add a little since I always save 300/ per day. So, I must get another amount can end up saving 500/ a day and also, I must develop a good business language to influence people to come and buy my products, by that I will increase my customers.

**I: The rest what do you do to increase your savings?**

**PR03:** As number three, like when I’m doing sex work, I should not concentrate on one thing, I should do another business that increase my set income. I should not only concentrate in sex work; I must venture into business.

**I: Which kind of business do you do?**

**PR05:** Then business I can do is selling fish, so when I go for sex work, I don’t only concentrate there I also sell fish.

**I; Okay is there any other person, what do you do to increase your income, number nine?**

**PR09:** What I always do to increase my income like when the year begins, I always do farming, when I plant after three months I harvest, and it gives me easy time because I have maize food. So, the way I always sell sardines and also do sex work, I find it easy to buy food since I don’t spend a lot of money. I always harvest in plenty maize. Beans and green grams and I can as well sell them and get money, by doing this makes get income.and gives me easy time to save

**I: Okay, now everyone wants to tell me the amount of money she is to refund. How much are you to pay**

PR05: We have huge loans.

**I: I also want to know these huge loans**

PR09: 5600/=

**I: number nine 5600/ next number eight**

PR08: 7000/=

**I: Number six**

PR06: 12000/=

**I: Number two**

PR02: 8800/=

**I: Number five**

PR05: 20,000/=

**I: Number four**

PR04: 11,000/=

**I:** **To number three**

PR03: 16000/=

**I: Or 16 goats’ number 10**

PR10: 1850/=

**I: When you say 1850 it means that one thousand eight and fifty**

PR010: 18500/=

**I: Number one how much do you owe?**

PR01: 15000/=

**I: We really have huge debts , we are continuing for sex workers, do you always think of quitting sex work and when are you planning to stop, what happens when you decide to stop. In short what drives your t quit sex work? (People laughing in the background)**

**PR010**: My people very bad things happen, somebody calls you and tells you to even borrow fare that he is going to refund you to go to Bondo and he tells you to take a motorbike and borrow fare from somebody he would refund you back when you reach. And when I have reached and tells him to pay the motorbike owner he says I pay him he will give you money after finishing and since I don’t want the motorbike guy to see him I will pay and we engage in sex till morning when its morning after spending the whole night with him, he tells you to call your motorbike to come back and take you saying that he will send you money when you get back home. When you get home, I am sending you money even the children you left behind are hungry.so when you reach home a message that someone else was sent money and withdrawn pops and when you check your phone you see a message thinking that it is mpesa and when you check your account balance there is nothing you had not paid the motorbike you went to work and came back empty handed it is not easy in my own opinion I feel that if I can get something to do [ source of income] I can leave for good[ sex work].

**I: Any other person?**

PR01: As number one eeh it is not easy that is why you have to laugh because whatever I want to say, as number 1 you can leave your house go somewhere like I can leave this place and even go to Asembo and after I have reached it is my money that I used saying that you are going to send me money and then he says madam you know I only get money end month I am a teacher and I get my salary from 5^th^ and there is no problem with that and you know I had taken advance we are not badly off just take your money and come by 6:00 pm you shall have reached I will be getting ready and I am smartly dressed and called the motorbike guy Then I tell these people that I’m going to church to praying knowing that really, I’m not going to churchsomething that is going to be prayed over , when reaching he asks what do you want to take today?am still contemplating I can take soda with an escort now is when I want to start working[ engage in sex] and he uses different sex positions for me I gave birth through CS and operation doesn’t want that you engage in sex all the time, after that you are waiting to be paid ,the way you were thinking that you would be given 1000 and above to go home with and then he tells you that he has received an urgent message and then you ask him what is it then he tells you that the person who was supposed to send him money, he hasn’t sent me the amount he was to send madam you know today I was supposed to give you 2000/= according to the way we are used to each other and then he gives you 400/=.and he has really worked on you until my new pant I am now tired since I had said that I was going to pray and I was supposed to be spiritual and I come back with Satan my body is tired when you are tired and then someone wants to give you 400/= coming from =Asembo =to= Uyoma= I am supposed to be coming back with some shopping So, with I’m tired with sex work in that if I come to get another job [ sex work]I can do with all my strength and forget about sex work, where I engage in sex without being paid.

**I: Anyone with a question or addition, (laughing from background) anyone with something to add ,you are allowed to laugh but not continuously anyone with something to add they have shared with us what bring such conversation that she can leave this work [sex work] right**

ALL: Yes

**I: And this conversation do you talk about it with your other peers?**

PR01: As number one I can’t go to the market and find four or five women and start narrating to them my experience.unless you don’t know me and I don’t know you then I can share with you even as we know each other and we greet each other and we go our ways and we can’t talk about that we can talk about that unless we are in a corridor where there nobody and be able to see whoever is coming, and even if we know each other we may not talk about sex work (laughing from background) a woman born with good body that people give respect what do I have say in the market [participants laugh].

**I: Only two people have given me their experience, what brings such discussions when you sit down you say today I need to quit sex work?**

PR07: As number 7 You have known a man and you have talked and known each other and he even have your contacts because there is a way you had talked, he knows you and you know him, you had met , then he tells to go and you ask him how you are going to reach there since you don’t have fare Since I know am doing my business and he knows the business am doing ,and he knows if he tells me to go he will tell me to use my money he is going to refund me because if I tell him I don’t have and I do sell vegetables, I will tell him that I am coming you know even if I don’t have I am going to borrow money from [ mentions name] am going somewhere I will give it back to you, you don’t tell her where you are going to and you also don’t want her to hear about it you only know the two of you ,you go and finds him you meet this kind of a person who wants to revenge first he would be calm ,let him undress and you also undress he is going to engage in sex with you ruthlessly the first round is just okay then the second round then the third round you hear him say I don’t have money I had spent this and that and now I am financially down you know he is talking meaning he is trying to tell you that whatever he had engaged in[ sex] it is a debt and he is not going to pay you, after finish he tell you that just go I will send you the money via Mpesa, then you tell him that there is no way you can leave yet you don’t have money give me so that I can go back and then he tells you I have given you only the fare just go by evening I am going to send you money you will wait ,he won’t pick your calls and there is nothing he will tell you and In the long run you come back and work tirelessly in your vegetable business may be I didn’t get money from [ mentions name] it was my money I used that when it reaches evening my children will be able to eat and finds something to save, you have nothing to save and even what the children are going to eat is not there and the business has also collapsed it is a job that when you look at it you are always running into loses and say that you won’t go back .you swear you won’t go back, whatever I was doing is the only thing that can help me and nobody should lie to me and whatever I had placed my hand on I should not play with it is not easy you can long to quit and if you can get something to do you can why you don’t do it, you pretend by saying it’s not your time it’s their time but in real sense the losses you made in sex work is what has made you to quit.

**I: Is there anyone who wants to add?**

**PR07**: I can add that if number three had my money, I can her that can you please give my 50/=, I tell her when this mama is getting me So, even if you call him you will pretend,that you were just checking on him how he is . So, claiming any money after engaging in sex is very difficult but at least when I sell you cabbage it easy for me to tell you to pay memy 10/= that I sold you vegetable .there is no problem even if they get to hear about it ,but this one there is no way you can ask someone to pay you So, it better you do a business and I sell you cabbage of 10/= which I can pressurize somebody to repay your money. (Laughing in the background) it is better you do a business where you can ask someone to pay her debt.

**I: Any other person I want to add?**

**P:** You can come to agreement with different persons and then agree with one person that you are going to charge him such price, sometimes you go to restaurant and order something to eat, thinking that he is going to pay after eating you ask yourself where is that person who made the order and you wait this person has disappeared. Then you are the one to pay or else you are beaten thoroughly.or you are asked to peel the potatoes since he is gone and these are things that when you think about it makes somebody to say that I will quit sex work.

**I: Is any person who wants to add, now there is none , is there any reason coming from you personally**

ALL: Yes

**I: Are there external reasons, the reasons you get from other people apart from personal reasons why you want to quit sex work?**

P: External reason?

**I: Yes that you get from other people.**

**P:** The reason that comes out is discrimination. When they find out that you are doing sex work, you will not know peace at all , even if you are going to pray, people won’t believe you are going to church. You find that you don’t have a say before people.

**I: Apart from that is there any? External reasons, is there any apart from stigma?**

**PR07:** You can find a person coming to you, let’s say he is a man he comes to you with intention of messing up with you, then when you refuse to talk to him she starts accusing you that we know you very well, you are a prostitute and also know where you always go to have sex .So here you are denying, sometimes you can have man[ married], I can lie to him that I’m going to see my mum is sick,or Chama so when I come back I find that the husband has already been alerted where I went.you went for sex work and came back and, you find yourself in a problem , you went to the field [sex work] and came back but there is no peace a home.

**I: Now you people you feel you want to leave this job, and have planned to quit?**

PR07: as number seven

**I: Let her first finish is when you talk**

PR07: As per now, even those we go to have sex with complain that there is no money, so sometimes a man can call you when he is broken to have sex with you but as per you going there is a problem, because he doesn’t have money. So, you can tell that you have charges your mind nowadays, you don’t do sex work. Even if there is someone I was used to, so, you can go direct to him and tell him there is a change. Now I want to quit this job, you tell him in a good note. You can even find a way of convincing him by telling him you are tight, take care of your home and I also take care of my home this is because he can’t help you, he doesn’t have money yet he wants to sleep with you.it is better you find another way of surviving

**I: Here is the question. Do you ever have plans to quit sex work and how often? Or does it come abruptly**

**PR03:** I always that one day if I will get any job to add on my business a part from sex work, I can do and sex work. Even if I can get a job which pays me 3000/= in a month I can leave sex work, more so when am going through violence and stigma,that leaving sex work is what clicks into my mind

**I: So, when you think you think you want leave it, do you plan, is it in your mind?**

**PR03:** What you plan is what you want to do, so always pray hard that if I get what to do. So, when I get what I can quit sex work.

**I: What if you don’t get what to do?**

**PR03:** If I don’t get, I just go on with sex work, I will just continue with it

**I: Yes, number ten**

**PR010:** I am supporting number three, with me I want to leave but poverty, poverty is what drives me to sex work, when a child is hungry you have to do it to bring food on the table. Like me when I’m broke, I’m always very harsh to the extend that I can kick anything lying before me, so have a boyfriend and he doesn’t have money, I see him as a waste, but when he has money, everything is just good. And you all know money is everything, but if I can be busy like you interviewers you come to work early in the morning go back in the evening, you mind will be busy you can’t even think of things like sex work. Same to us we become busy, we can’t think of having sex, poverty is the problem.

**I: So, doing sex work you leave it after planning or what do you do?**

**PR010:** It is sometimes you leave after you have planned well, there are some women whose husbands were dead like mine left sex work, let me assure you sex work is not easy to leave/quit. They were pregnant before being inherited; this is because they never meant their words by saying they have left sex work. So, with me you cannot leave sex at ones or completely due to the age we are having, what I know we can leave for four-five days but you can have one which if he gives you money or not your life continues. We do have 3 and more because of income, we can as well leave.

**I: Okay, now women who do sex work at what age do they leave/quit it?**

**PR06:** Me as number 6 I think if you are 60 years and below you are in there, is when you can leave sex work but when below 60 you can have as many men as you want.

**I: Number 10 is supporting that 60 years you can say whatever you want to say**

**PR07:** There is a guy we lived with for eight years, so there is something that happened we parted ways ‘So I wanted to leave sex work for good but it was difficult because I was also afraid that when he hears that I have another person he can harm him, and it is impossible to leave because your mind is still on it. Even if it has taken five months but impossible.

**PR01:** Even when you are from it, your mind must click on it’

**I: When coming from where? Give me full statement.**

**PR01:** When you are from monthly periods

**I: ooh how……….**

PR01: Yes, during that period, when a woman is almost to menstruate, she must have some feeling during wee hours in her mind.

**I: Which feelings?**

PR07: Her mind will not be at peace. (Laughing from back ground)

**I: Why is her mind not at peace, what disturbs her mind?**

**PR07:** In short, she will be need of a man, meaning she wants a man.

**I: Ooooh, mmm mmh. (People laughing from back ground). So, at 60 years is where a woman can leave her job.**

**P**: According to me, I don’t see it in that perspective. I see it differently, am saying this like we always do sex work because we lack jobs/ income in that if someone gets a job which can pay her, she can stop doing sex work even at 20 or 30 or 40. So I feel that if when we get income this can prevent us from going for sex work, so that’s what I see.

**I: So, you people see that is only a matter of age that can make somebody leave sex work? Are there something’s a part from age.**

**PR01:** According to me as number seven, when responding to this it is not a must that when I clock many years, is when I stop it and it should not be that I clock my age is still young. So, I force myself into a man just like other colleagues have said, I have my business which I own, I get good profit and also can feed my family well, my children’s health is good and also academically. It is not a must for me to look for a man too live with is when I have a good life. Because a woman who is ever busy, who goes for work and comes in the evening, you know you already a ware you are the bread winner even when your husband was dead, so you’ll come back from work when you are tired and also your mind is busy thinking of your business & the responsibilities in the family, so as to your body. So, you can decide that if I can be tired this extent, then you find a man wanted to have sex with me, then let me remove this an out of my life because I have everything a woman could have. If a baby is what can drive me to have a man, I already have. Money only is what I can think of having man, but already I have my money. So, you can decide to leave a man in your life even at 35 years and living alone a good life. So, it is not only age that can make you leave being with a man as your sex partnerso it is not only age that can make me leave sex work , I can leave him when I’m young.

**I: Ooh, is there any other? A part from from age, reason. When silent means there is none, now what do women do after living sex work, after quitting sex work,**

**PR07: Which job they can engage in after**

**I: After quitting sex work what job do women do? Everyone is going to talk**

**PR07**: According to me, after leaving sex work, I have decided that will do farming I will have vegetable, maize, beans. I will be busy doing my activities in the farm because I had said no more sex work again. I had engaged in sex work and left so, the little the energy I have, I will put in the farm to help me sustain my life not to go for sex work.

**I: Any other person what kind of job can you do?**

**PR01:** What I know when am tired and leave sex work. I can put my mind and soul in what can give me income, even if selling omena, the way I am doing now I can continue selling and again I can be a teacher to the other person by advising her challenges that we go through in this job[ sex worker] , I came through when doing sex work. If only she is a person who is free to me and has a listening ear. I can tell her that I had done it before and what I came through is not easy. So, from there I can I can be a teacher to those doing it with an aim to teach them and stop sex work. And as for me I can do my business with all my heart to prevent me going back to sex work, and also, I don’t want my child one day to be a sex work. Because what I saw that pushed me to leave it, it is only me who knows and I can’t not even tell somebody’s those are the things that may encourage me to do business.

**I: You have said you can be a teacher to another person**

PR09: Mmh

**I: From this is there any income that you can get from teaching others to leave sex work?**

**PR01:** Yes, you know when am a teacher to my child or co-wife, the thank you that I get from them is a support to me because God also has blessed me. Even in a society when I preach about stopping sex work. I would have supported them.

**I: Okay I see you have supported them, but is there any income you are going to get from that work?**:

**R:** No.

**I: there is none okay number eight, what job are you going to do after leaving sex work?**

PR08: When I live sex work I am going to boost my business. The time I was wasting when called to =Luanda= or =Mbita=, I will put it in my business not that after short time I’m seen closing the kiosk and by doing so when a customer comes and finds I’m not there, he/she won’t come back, but when I open every now and then the customers, that used to go somewhere will always find me and I will save their money. By doing that my income will increase.

**I: Okay, which job can you do? Number seven you had said yours, what of number six?**

PR06: The job I can do is to maintain my business. This is because the job which I was boosting my business with I have left. It is now this business that now I will make sure that feeds me and it is my business that I can go daily and maintain it.

**I: Number two?**

PR02: Even me it is business which I can boost and also do farming. I can plant crops and when they are ready I sell some and boost my business.

**I: Number five?**

PR05: I can invest in my business to help me because main job I was doing I have quitted

**I: Number four?**

PR04: Even is only business that can put all my energy into.

**I: Mmh number three?**

PR03: Before I leave sex work, I want to save well so as in near future when I leave sex work, I want to build rentals so as to help me when I leave this job [sex work so that it is what I have in plans.

**I: Number ten?**

PR010: I will pray.

**I: Job which will make you earn money, will praying bring you money? (People laughing**)

PR010: Ooh, sometimes I will put my energy on business and mind. This is so to divert my mind from sex work. I can try hard to buy cattle in that when my business goes down, I can change them to money to boost my business. I wanted to add on that like poultry farming is very good and they grow fast. You can even sell three of them to boost your business and rearing chicken is also easy. They can really help.

**I: So, this business where do you want to run this business?**

PR07: As number seven I live in a center, every market has its own market day like any market. Like on a Tuesday like am a person who has grains, I can take green grams and go with it like =Bondo= because I know when it sells it where I am it will be sold slowly. So, I can go to where it will be sold very quickly that is =Bondo=. So, I can even sell one sack. I would have gotten money that I need so I will increase my business. So, I will again sit down and calculate that after selling green grams I will go to =Busia=to purchase ground nuts and green grams and put in my stall. When market day for Aram comes that is Monday I can go to Aram and sell everything and again go back and purchase again and that’s how my business will grow. I will just be selling in Rarieda and my business will thrive here.

**I: So, you are that it means that you people are selling within? So, is there change you see in your life after leaving sex work when someone has left sex work is there any change?**

PR03: According to me there is a friend of mine who was in sex work, and she left she was doing it in =Nairobi= I can see a big difference in her. She was kind of a person who was fighting all the time and drunk most of the time but since she stopped sex work, she is sober and turned to be a good person because of the work she is doing now she has changed. It reached a time when she used even to walk around with a knife in her pocket for security purposes, and all these she no longer does them so there is a change in her.

**I: Any other person? Is there any change we see in our life?**

PR07: I have seen an aunt of mine whose sex work was main source of income in =Kisumu=and she used to say that she is a sex worker and has her own house where she used to pay rent and it is from sex work that her children and here at home and they are in school. At the end she fell sick to an extent that she was unable to go for sex work. She back home and her mother struggled with her until she recovered and swore that no more sex work. By the time she was doing sex work she had her business at =Kibuye= market, when evening approached, she leaves early and she could go to the parking zone for her sex work. Secondly she could even hire sell for her and later pay her so when she left doing sex work and concentrated in her business, she never wanted a man in her life or get married, because what happened to her in sex industry was hurting. She could say that some people had sex with her as if they were sleeping with a donkey. [You don’t get tired] So, all these hurt her and she stopped sex work. The bad name she holds turned to be a good name and people would talk is this so and so who used to be and now look at how beautiful she is now you have a say and you are accorded respect.

**I: Mmh, is there anyone with another response, now is there someone who left sex work but come back to it again. Eeh**

P: She left and came back?

**I: Yes, please respond.**

PR03: Like me there is a person who left sex work and came back and I asked her why have you come back and she told me that there was a way she had an income when she was doing sex work and she came back because she had a business and it failed/ collapsed so she had no source of income. So, there was nothing she could do and she decided to come back to sex work, she said that there was a way she was earning from sex work and she came back full swing.

**I: Any other person who knows someone who left sex work and came back?**

PR06: There is a sister of mine who was a sex worker she left and again came back and when we asked her why she came back, she said that she used to get a lot of money in sex work than now, because she had no money so she decided to go back to sex work. But, after going back she got sick and died.

**I: She died?**

PR06: Mmh

**I: Is there any other person who left and came back? If there is no, we will continue. Now she has said that her sister went back and died, apart from death is there anything bad that can happen to you when you go back to sex work?**

PR010: Sometimes at first, she left when free from HIV/AIDs but after going back she can get it.

**I: And the benefits she can get after coming back?**

PR01: Benefits she was just doing it and never used to farm and when she left she found out that there was nothing she had and she felt like I am going back but I have to do something, she went back and whatever little she gets she would save and open up a business.

**I: Okay, now are their things you want to do before leaving sex work?**

**Before leaving sex work**

**I: Yes, sex work eeh**

PR03: As for me I had said earlier, before I leave sex work I want to save and build some rentals in that when I leave sex work, I would be getting some cash at the end of the month.

**I: And have you started planning?**

PR03: Yes, I have started saving.

**I: Okay any other person what do want to do before you leave this work [sex work]**

PR07: We have seen that it is very difficult because it has no money as per now, after I have realized that I had said earlier that I have a small stall and the thing that I need are like cereals I will make sure I buy and put them in the stall to make the business to grow in that when I have decided to leave sex work, I will get income from vegetables and cereals and my life continues of which I had already started so I had planned to leave it.

**I: Mmh any other person?**

PR09: I used to purchase omena and sell and I had seen that this thing is hurting me [sex work] and time is catching up with us, it made me it is like a coincidence and I had thought of how I can put this business to bring forth good profit to enable my life to continue. This made me to come to agreement with my sister financial support that I will be sending her omena and after she has sold it she is to send me back the money and again purchase omena and send her. Because the one that I do sell where I am only person brings little profit which is enough to feed my family but you can’t do anything tangible with the money. It is too small so that is what I am thinking of.

**I: Ooh number one do you have something?**

PR01: I had planned a lot because people get tired like you can be saved from doing sins and start preaching the gospel of God. I had thought of buying cattle that can help me in one of the ways. Mostly I had realized to buy sheep which I have started to buy. You know sheep do give birth after a short period so even if it gives birth and my business is not doing well, I can sell one and boost my business w. So, that is my dream because you can be tired and also keeping chicken for my life to continue after leaving sex work.

**I: Is there any other person with a different opinion? There is none and do you know anyone who has left sex work in the past 5-10 years?**

PR07: As for me my aunt whom I was talking of this is her fourth year since she left sex work.

**I: Mmh, number three the person you knew how many years has she taken?** PR03: This her sixth year.

**I: What made it easy for her to leave this job?**

PR03: She went ahead and opened up more sources of income like bar and also built rentals and after building all these she left sex work.

**I: Is there any difficulty she came across when leaving sex work?**

PR03: I don’t see any difficulty she came through because she fetched money from her investment.

**I: Okay now pay attention we want to talk more about Jitegemee intervention, what Jitegemee wants to do. In Jitegemee we say that where you save your own money you can withdraw it anytime you want whether you withdraw all of it or half of the money Then its money that does not incur interest but it is also not a must to save. So, it is something you save when you have and when you luck what to save you don’t. So, no one will be upset with you. Listen to what I want to read. Earlier on I explained to you what Jitegemee is and also explained to you it is being done to make sure that sex workers have extra savings that can enable them refuse to engage to unprotected sex or take a break in sex work. I had also said it is more on women who do sex work save their money to use when there are no more men to pay them or to help them in future after leaving sex work. So, you have heard that it is your money which you save after saving this money and you have not got a customer you are free to take it and use it. Then it will put you at lower chances of getting HIV/AIDs. It will also help you in preparation i.e., like if you have your own money you can budget for it, here comes the question. Jitegemee can be embraced by sex workers in Kenya?**

ALL: We can embrace it.

**I: Which kind of sex workers can like Jitegemee?**

R: Those who save.

**I: Yes, women who save, another one?**

PR07: That woman who is hard working, a woman who wants her money even if she is not given by any person.

**I: Any other person?**

PR06: There are some who won’t like because you are a sex worker and you don’t save or you are a business woman and you don’t save, so what you will have to save is nothing because you get money and use it all at the same time.

**I: Okay, you have ten friends who are sex worker and you discuss with them would like Jitegemee program. I am starting here. Number one?**

PR01: With how I think I can’t specify how many will agree but where there is ten people around five of them shall agree and you know there are some people who are after sex in that if they don’t it they feel there is not well, so it depends with somebody’s attitude on how the program is going to help her. With me I think if I tell them and explain to them more on Jitegemee a group of ten, I am sure that five of them can accept to save.

**I: Okay number 10?**

PR010: From ten people I can get two.

**I: Okay number three?**

PR03: When I tell them more on Jitegemee and how to save and withdraw your money at any time I can manage to get seven of them.

**I: Number four?**

PR04: I can transform three people.

**I: Okay number five?**

PR05: I can get four people.

**I: Yes, four people, number two?**

PR02: Six people.

**I: Number six?**

PR06: I can convince five people.

**I: Number seven?**

PR07: Sex work is very difficult like me the way I have been trained and go out there to discuss with ten people more than Jitegemee, I think they can join Jitegemee because as I speak even me, I am tired of doing sex work so I think all these people can change.

**I: Number eight?**

PR08: Six people.

**I: Number nine.**

PR09: Seven people.

**I: I am again going back, am now asking number nine has said it is seven people that can agree with Jitegemee. The remaining three why won’t they agree with this information?**

PR09: These three people remaining, first you know there are some people whose understanding is very low. They are people who want to see what impact Jitegemee has brought into their lives. Some people also if you teach them, it is like they don’t understand you at all. The other person I can say they are Thomas’s or salamander who waits to see with their own eyes. So, you can teach and seven of them can understand and the three will want to see the impact those who entered Jitegemee will get.

**I: Okay, you have said salamander sees with her eyes? What do you mean?**

PR07: Meaning they want to see what you benefit from joining Jitegemee.

**I: Okay number eight, these four who won’t join why?**

PR05: These four are those who will not even let you explain. When you just begin telling her she will just say you are telling us nothing. There is also one who says first start then we will join after seeing how it’s running. That is when we will join you. Some also will ask how long you have been in there in that you have audacity to tell us its benefits. So, you won’t be able to transform the whole ten there must be some four resisting.

**I: Number six you said only five will agree… Why will the rest resist?**

PR06: You know it depends with how somebody’s understanding is even after mentioning the word money and maybe you have not yet finished, she will start saying that you are the one who will be given the money to save. So, you won’t be on the same page, so you will only get five only who are accepting.

**I: Yes, number two why won’t four people won’t join?**

PR02: These four shall disagree with you since they won’t understand what you say they will even go ahead to discourage others not to join.

**I: Number five, these six who won’t agree, what will you do and why?**

PR05: I think it’s a matter of to call, they want their colleague to try first is when they join. So, when you try to advise her on Jitegemee, it will take you a lot of your time and will really disturb you with talks.

**I: You said seven people won’t agree with this information?**

PR04: The reason why seven people won’t agree with is because there are some people who are used to sex work, they do say something which is being stolen is usually sweet so for you to invade somebody’s life and tell her to leave sex work is difficult. Eeeh.

**I: Eeeh number three, you said that three people won’t agree, why?**

PR03: This is because there are some programs which came before Jitegemee came and they again disappear. So, you hear them saying you have started very well, but where will you reach, meaning it something which won’t last for long and therefore it won’t be beneficial to her. Because some always come and people save but end you find that they disappear with people’s money. So, when it comes to saving you find that people are not ready to save.

**I: You know when you want to save you are the one to select where to save, like earlier on I had asked you where to save, like you told me you always save in chama. So, it’s you to decide where to save your money. We as Jitegemee will only support you like number three said she can save 700 per day, meaning in a month you should have 2800/=, so if we come will expect to find 2800/= but unfortunately find 1000/= only, so we shall ask you what happened that you end up saving 1000/= in a month rather than 2800/=. So, it will be you to choose the right place you shall be placing your money. Jitegemee won’t save your money, Jitegemee won’t give you money to save, and it’s upon you to save the money you get from sex work.**

PR03: This money which we keep what interest do we get? Some people can ask so. Because you find that when a sacco called kosalo came, many people joined it because you find that when somebody borrow 200/= she will get 20 shillings interest so she can ask you this Jitegemee what will it bring forth.

**I: Ooh Okay, number ten you said eight people will agree and two will not why?**

PR010: You know where people are, you can’t know what people think. They will say let me go and see like many things have happened to people more so robbery, people have been conned when paying school fees, so people are just curious about joining any group. So, you can explain to somebody something on a program but when you go back the follow day to go now with her to that she is busy. So, you can fail in your mission but the remaining number I can find some.

**I: Okay number five, you said you can manage five, why will rest not join.**

PR05: Human being is someone whose understanding is hard the way my colleagues have said, they are people who want to see with their own eyes. You can go and talk with them but in other terms she will ask you how far have you been in the group and what benefits have you got, and since you are going to save how is it not bringing forth interest? It should be that when you save, it must have an interest. So, answering such questions may be a problem. So, you have to convince this person so much for her to embrace it. And besides going to somebody and start telling her direct that sex work won’t help you is not a walk on the park. Sometimes she may have your past and ask you that since you start sex work what benefit have you got and what losses have you encounter. So, after being told this way, advising her becomes difficult. She will also unsupported you to an extent that if you were to advise her you end up lacking words to tell her.

**I: You have said the reasons why people won’t like. So, what should we do as Jitegemee to increase number of women who do sex work agree with this information?**

PR07: As number seven, these ten women I can talk to them to enter in Jitegemee, I will use the teachings I get from this training and make sure Jitegemee change my life because earlier on I said I will leave sex work and concentrate on my business and through that I can tell them I depend on myself so from that they will ask me which means I have used. I will go tell them that I was a sex work and realized I was gaining nothing from it. So, from there I started savings helped me in boosting my business. And from that savings helped me to be independent so they will want to be like me who saved money and left being sex worker and now is able to live her own life. So even me I want to leave sex work and start saving to live the life so and so has lived without somebody giving her money.

**I: That was you, so for us as those in Jitegemee what can we do for sex workers to accept Jitegemee.**

PR03: I never heard you saying the interest somebody can earn like when I save 200/= or borrow it what interest will it bring forth, because I heard you saying if you borrow 200/= it won’t earn interest.

**I: So, but still, what can we do as Jitegemee, what should we do for sex workers to accept Jitegemee?**

PR03: You are the one to see when I save 200/= interest should I earn.

**I: But is when you have completed your statement at first you were like asking me question (laughing from background) next person?**

PR03: Secondly what you should do is sustainability, it should run for long, it should not be like some other ones that come and go, it should be something strong for somebody to benefit from it. Not that when you want to get some benefit from it is when you will have issues like.

**I: Ooh is there anything you can add on.**

PR05: We are hungry.

**I: I know people are hungry, we are only left with four questions and we are complete. So, you should just answer the questions because when you are silent, I again become silent. You have said saving money without interest on it they won’t like; she would like if her money she saves and earns interest. Secondly, she has said that if the project is started it should not stop after short time, so what can we do for it to be better?**

PR07: as number seven you as people from Jitegemee you were to make us something like Sacco, so we will join this Sacco, we will be able to save in it and as well borrow loan. So in that case there is interest that money will earn like I know if I save 200/= I will be able to get 210/= or 220/=, I place my shares I borrow loan and pay back.

PR010: What can make people join it just like number three said it should not end before reaching it mission. So, from here we should get some people out there and discuss about it. The more we discuss in groups it will spread. Sometimes we can have a group and maybe some people may ask what are these people that sit here from there we can be proud by saying we are from Jitegemee so the more we sit in groups the more it will spread. So, we can be able to make Jitegemee known and many will join.

**I: You are now the one to go and spread the gospel of Jitegemee since you are the one who have said but to us what can we do as Jitegemee to make it known?**

PR010: Like earlier on you promised you will come and you have come that is very good. So, what is not good is a lie, being truthful is good and something can prosper. If you are not coming you just said [mentions name] we were to come but unfortunately we are not coming so go back and do your jobs but not telling us to sit waiting for you but at the end you don’t show up. Now even when our time is consuming but we are happy we are with you getting knowledge and you know this teaching I get here is going to help even my child. So being a truthful person is good.

**I: What else should we do as Jitegemee to make it be embraced?**

PR09: When we came here, we were very many and some were told to go back that they will be taught on another day. The way you had started with this group ensure you teach the other group also so as to make Jitegemee will know and liked. You know when you teach them and we also keep on teaching them people will have more knowledge and many people will make it be widespread.

**I: Okay, we should teach more people, number three you are laughing do you have an idea?**

PR03: What am trying to say is that at times when I want loan like sometimes, I want a loan according to the financial issues I have, you are told you won’t get it today may be after some days or a week so you find that what you wanted to do you don’t do it. So, am trying to say that in Jitegemee where I save whenever I want, I should get it not that am told wait for some week is when I am given.

**I: Who can respond to her, what did I say about Jitegemee?**

PR010: You said that bank you save in is yours Jitegemee does not chose for you.

**I: Then what else did I say?**

PR010: You are free to withdraw it at any time.

**I: Yes, nobody coerces you, you withdraw your money anytime you want. No one is controlling you, you control yourself Okay what should Jitegemee have for people to join it. Number seven said we should have Sacco why do you want us to have Sacco.**

PR07: Reason is, I can save in Sacco and borrow loans.

**I: Number nine said we should teach them? What methods should we use for people?**

PR07: Just like the way you communicate today; some people went back and was told to go back they shall be taught later. By that it will make Jitegemee spread by teaching them and by teaching them they will as well be teachers.

**I: Okay, now what are the things you don’t want to see in Jitegemee?**

PR03: What I don’t want to see is dishonesty.

**I: Mmh, another one?**

PR06: What I don’t want to see is gossip and lack of confidentiality.

**I: Mmh, next.**

PR07: When we say the meeting start at 9:00 we should be at the venue plus our teachers.

**I: Mmh any?**

PR01: They do say time is money for example that woman who always sells mboga by 12 o’clock her time will be wasted so when we become punctual in that will be wasted so when we become punctual in that we start at the exact time and end as the started we start at the exact time and end as started time, Jitegemee will run smoothly.

**I: Any person who wants to add, so what do sex worker can like about Jitegemee number five?**

PR05: Jitegemee has taught me about saving.

PR03: Jitegemee has taught me how I can depend on myself than depending on a man for support and also it has taught me how to save. It has taught me many things. Which I can’t say.

**I: Those many things are what I want to hear.**

PR02: I am hungry now (people laughing)

R: Jitegemee has taught us a lot not only a man should sleep with you even your child. You are just too much on your child that my son today I don’t have sugar. So, I can say you woman you should have money aside that even if your child does not give you, you don’t panic. It has also taught us a woman should be busy.

**I: And is there anything you won’t like about Jitegemee?**

R: We don’t like lies and we should be time keepers.

**I: And apart from lies, let’s say if you have joined Jitegemee can it interfere with your right in any way?**

PR07: When you set date that you want to meet us then when reaching you find there are no people, on asking him/her that mobiliser he will insist that he/she had mobilized is when he/she will start mobilizing people upon you reaching that is a challenge.

**I: Any other is there any person who feels that when she joins Jitegemee her rights are going to be violated?**

PR010: Like when you go to meet people upon asking them questions you will find they are mute, that’s another challenge.

**I: Any other.**

R: Another challenge is confidentiality like the way people were sent back they may not turn out where you call them again to come back.

**I: Any?**

PR06: We were told to be in the meeting by 8:00 am but you find someone coming at 11:00 am or 12, or somebody enters even now and maybe you wanted 10 people so you will wait for them to come in two, three or four.

**I: Is there any challenge? Okay that challenge you have said tell me how we will overcome it.**

PR07: Like me number seven you have talked to me you want a certain number (people laughing) and I become difficult. I can lie to you I have already assembled people but in real times there are no clients. To overcome that you should do a background check on me to find if I am an honest person.

**I: Okay, number six. the challenge you talked about give us a solution.**

PR06: Like I said people will come in different time, when I am called that I am needed at such time like at 8.00 am it’s upon me to plan myself even if it is waking up at 5:00 am, so it is my duty to come and wait for you not that come and wait for me.

**I: Okay number five.**

PR05: Just like you can be told there is in need of 30 people but at last you again change your mind you say you want only ten people. This may disappoint others some may not even agree to come back.

**I: So how can we overcome that challenge?**

PR05: So, for you to overcome it you should be from on your point, if it is ten let it be ten.

**I: Okay, number three.**

PR03: You should be honest if you say it is ten sticks to ten, if I were to get money let me get money.

**I: Okay number ten?**

PR010: For us to make this thing nice, we ourselves we should love whatever we are doing. You find that if you like doing something you can even wake up at 3:00 am to do it. So loving what you are doing is a very important.

**I: Now, what amount of money would you like to save in a week? Without disrupting your daily activities/expenses number nine.**

PR09: 250/=

**I: Number eight.**

PR08:

**I: Number seven.**

PR07: 700/=

**I: Number six.**

PR06: 500/=

**I: Number two.**

PR02: 250/=

**I: Number five.**

PR05: 100/=

**I: Number three.**

PR03: 200/=

**I: Number four.**

PR04: 200/=

**I: Number ten.**

PR010: 1200/=

**I: Ooh, Number one.**

PR01: 500/=

**I: Okay 500/=, so the money you have said you can save where do you want to save them? Without any problem?**

PR03: Where I want to save as at per now?

**I: Yes that 200/= of yours where do you want to save it. Can I start from this side number ten where do you want to save your money?**

PR010: Where I want to save now

I: The 200/= you said you wanted to save where do want to save it?

PR10: Equity.

**I: Yes equity.**

P: Mshwari lock.

**I: Yes, Mshwari lock and you?**

P: When I leave Sacco, I will go to Mshwari.

**I: In Mshwari and you?**

P: In phone.

**I: In phone through Mpesa or?**

P: Mpesa.

**I: Okay Mpesa and you?**

P: Mpesa.

**I: Mpesa and you?**

P: Mshwari.

**I: Mshwari and you?**

P: Mshwari

**I: Mshwari and you?**

P: KCB

**I: KCB and you?**

P: Mpesa

**I: Mpesa, now (laughing from background) so whoever has a question to ask with me am done.**

R: Me I have a question, when we decide we want to be in a group as Jitegemee, can’t we make One saving space/group.

**I: Okay that’s question you want us to make group?**

P: My spirit is telling me so.

**I: Okay but when we bring Jitegemee, we will bring everything in Jitegemee and decide if we can be as a group, any question?**

PR07: I have a question? The way Jitegemee has brought us teachings today how often would it be coming back for more teaching? Is it on a monthly basis weekly or how?

**I: Who can answer her, we said we are meeting?**

**P: Once.**

**I: Any other?**

N.T: It is one because this is a small study which will bring forth a big study and you shall be able to join the study.

**I: Is there any question? There is none and the interview has ended at 15:14**

**END**
