# Supplementary material for: Eat a little and save a little: A qualitative exploration of acceptability of a potential savings intervention to reduce HIV risk among female sex workers in Western Kenya
Source: PLoS One. 2024 Dec 19;19(12):e0310540. doi: 10.1371/journal.pone.0310540 (PMC11658496; doi:10.1371/journal.pone.0310540)
Supplement: S1 File — (ZIP) [file pone.0310540.s001.zip › Jitegemee Transcripts and Dissemination Notes for Journal/FGD S.docx]

**FACILITATOR: PHILIP OWITI ODOTE**

**NAME OF TRANSCRIBER: BERYL OBIERO**

**NOTE TAKER: JUDITH AYALLO**

**FGD ID: FGD S**

**CATEGORY: BELOW 30, PERI-URBAN**

**I: Thank you all once again, for joining this study. I have explained to you what Jitegemee is. According to how I have explained to you briefly about Jitegemee, what has immediately come to your mind? Let us start with number one**

PS01: As number one, on topic of Jitegemee, what comes to mind is, when we are doing sex work, we should have in mind that one day we should have… or we should find a way to save so that the children who are growing should not have the same behavior, or we can save the little we get so that we can help them in future. We save the little we have so that we can do what…help them in future. If we get little, you use your body to hustle, and when we get, we end up using it wrongly or miss using it?

**I: You have said carelessly. What do you mean?**

PS01: Misusing. You are misusing

**I: Okay, number 4 had raised her hand**

PS04: Yes. Me as number 4, just like you have told us about Jitegemee, I think that it is good, the reason why I think it is good, as sex workers, you can depend on yourself, and you can always hustle and save. Like me, I have no house at my rural home, I can do sex work and then save my money little by little, so that one day when I am tired, I can find a way to build my house.

PS05: Me as number 5, just like you have explained that thing well, now it is giving me energy, now that I exchange money for sex, many things… I also do not have a house at my rural place. I can save that money little by little, one day I’ll think and do something with it and one day I will remember that while I was in =Usenge=, I was exchanging money for sex. Now I save that money little by little and I can do something with it, something that I can see later. Now I cannot misuse that money, walking from one bar to another drinking alcohol, no. now I can use that money.

**I: Another person… the other people, what comes to your mind?**

PS02: I do not have much to say, as number two, I support what number one has said

I: What has she said?

PS02: She said that, according to the study you have brought us, we save, we can save and help the children growing after us, so that when they grow, then this thing we have done, they grow knowing that mum used to save for me, and I should not do what mum used to do. I should also look for something else to do

**I: Number one wanted to add something, I saw you raise your hand**

PS01: As number one, what I want to add is, when we continue doing sex work, you have brought us a big organization and that is very good, to help us. Because at times there is some money we are given, and if you bring us Jitegemee study, and teach us, and even add us a little thought. I am imagining that the money we get from there, after you have saved maybe 500 shilling or a thousand shillings, you can start a business, so that one day after your business has expanded, now you do not depend so much on sex work, but now you focus on your business. Because we save and depend on ourselves, after it has reached a point where we are depending on ourselves, it will force us as women who are not married to open up a business, or even women who are the head of the house, you have a husband but he is good for nothing [not helping], you have to be smart to get something. So we have to save to open up a business, so that we can forge ahead, that is what I wanted to add

**I: Thank you. The others, what do you think? Number three?**

PS03: As number three, I am also saying that there is time for sex work. Because it reaches a point whereby someone has grown old, or you are aged in that you cannot engage in sex work anymore, because maybe hormones have become weak and cannot work well. So, I think Jitegemee is good, because it reaches days whereby now you cannot use body to get money, so I think Jitegemee is very good.

**I: Another…**

PS06: As number six, I see that Jitegemee is good, you have taught us well, at least you have given us an insight that we can do this our work and save a little something. So we see that it is a good thing, because there are days that the body gets tired, so we can save.

PS07: As number seven, I would say thank you because, you have come and taught us things that we did not know or things we did not have in mind, now we have them in mind. Now, I see that whatever you have taught us is good, because I can start up a business, because previously I may have relied on doing sex work, I would use up all the money, but now I can say that I take ten and the rest of the ten I save one day I might use it to do something I can see.

**I: Thank you, yes…**

PS08: As number eight, I want to say thank you for bringing to us the study. I have seen that it is good and I am so happy. Because even though I am a sex worker, I would not depend so much on sex work, as days go by. I see that you have brought us a good study that if I start saving, then I can make my future better for my kids. I want to say thank you for the program

**I: okay, your day are going, meaning?**

PS08: Yes?

**I: She has said the days are going**

R: [All] Years, as in the years are moving

**I: Number three, did you raise your hand? You did not? Okay**

PS05: Am number five

I: Yes, just talk

PS05: As number five, I am going to teach the other sex workers, how to save. Because the program you have started today, there are things I used to do, and I did not know how to use money well. Now there are some of us who are sex workers and are not here, so I can sit them down and teach them. After someone has done sex work, there is a time that we use money knowing that tomorrow I will get more. But now, the economy is high, at times we go and we do not get good money. Like right now the money is not enough, so I have found something good, and I can go back and teach the others, ‘I went, today we were invited by impact, and they have come with a new program. My friends, when we are still doing sex work, let us do it and save little by little. We save because we depend on ourselves, so the money we have, it can help somewhere, so that our children… they know how we live but now we can change their life.’ So now I just want to say thank you for the good thing I have heard this morning. So I will go back and teach the others how you have taught us. As that even as we continue with this job [sex work], we should save

**I: Okay, thank you, I want us to continue. You have said that you work, and after you have worked and received money, what do you purchase with the money? The money you have earned, what do you buy with it? All of you have carried their hands, let me start with number one**

PS01: So with this money, first we buy food, many of us here pay rent and buy clothes…

**I: If you buy food, how much do you use in a day**?

PS01: When buying food…

**I: You cannot quote an exact amount, just approximate**

PS01: As number one, I have children, in a day I can use four hundred and fifty shillings

**I: Four hundred and fifty shillings…**

PS01: Yes

**I: Okay.**

R: I use three hundred.

**I: What do you buy?**

R: I buy food. And I can… from morning, because in the morning I must buy breakfast lunch and supper, so supper I can buy food, maize flour, the next day buy sugar, tea leaves and milk, yes

**I: So the three hundred is for a day or a week?**

R: A day.

**I: A day okay, ehee**

PS04: As number four, after doing sex work the money I get, I have to plan how my kid goes to school. She must also feed, she must also wear clothes, I also have to dress myself, am also paying rent, so the money I get, those are what I use it for.

**I: How much do you use in a day?**

PS04: One thousand shillings

**I: One thousand per day?**

PS04: Yes

**I: Okay.**

PS05: As number 5, I can use four hundred, per day.

**I: What do you buy per day?**

PS05: In the morning when I go to work, I leave one hundred shillings in the house. Also use one hundred at my workplace. So when I go back to the house in evening, I use two hundred shillings, I buy food, maize flour, charcoal, those small things

**I: Okay.**

PS02: As number two, my work (sex work), once I am done, I come with my money to the house, I buy food for breakfast, I want my kids to go to school, I do not want to see them sent home due to fee arrears. I buy food, I also have to be tidy, I cannot walk around dirty, I tidy up and look smart, so that even if I pass by, people must look at me and say, ‘today I must have this lady.’ I have to look neat, I cannot lie about that, I cannot just look untidy no, my children must also eat well, and because that is the work I am doing

**I: In a day, how much do you use?**

PS02: I n a day I use five hundred

**I: Five hundred…**

PS02: Yes

PS06: As number six, I can use around four hundred and fifty, in buying food. Because after you are back from work, you see that, ‘I have sweat, I have done this work, and this person has treated me well today.’ So back at my house I must make sure that day I take beef, or even chicken, I have to appreciate myself. My children must eat well, they dress properly when going to school, and I have paid my rent, now that is how I can appreciate myself that day.

PS03: As number three, I use five hundred shillings in a day. Because if my children are going to school in the morning, they cannot come back for lunch, or maybe they can come back and find that i have gone to work so they would not find me in the house. Now, I give them one hundred, fifty shillings each for taking lunch in school. At the house I must also take tea before going to work, so I’ll have to use another one hundred shillings that is two hundred shillings, remaining three hundred. This three hundred I use to buy supper after they are back from school. I buy food and some other small things.

**I: Thank you**

PS08: As number eight, I also use five hundred. Because at times, if I find a client and go, then when I come back, like number six said, I have to appreciate myself, with good food. I also have two school going kids, they do not find me in the house, like number three said, I also give them fifty shillings each while going to school, that is a hundred shillings. While in the house, I have to take good tea, and for that I would use around 150 shillings, that is sugar, milk and an accompaniment. I have used 150, in total is 250 shillings. The remaining, when the kids come from school, they find something to start with. I make good supper, they also celebrate their mothers work

**I: We have talked about what we buy daily. Are there other things that you buy weekly? As in a week cannot go by without you purchasing it? Even if it is once.**

PS06: Sugar

**I: State your number**

PS06: As number six, a week cannot go by without buying sugar, maize flour, charcoal, being that we are hustlers and maybe you ran out of gas long time ago and you have not planned how to refill it so, you find fifty shillings and buy charcoal instead

**I: You buy charcoal daily or weekly?**

PS06: You can buy a basin of charcoal, and that can take you up to a week.

**I: A week, how much is a basin?**

PS06: Two hundred shillings

**I: Two hundred?**

PS06: Yes

**I: The things you buy weekly? I heard you talk**

PS01: As number one, I buy clothes weekly

**I: Charcoal?**

PS01: Clothes

I: Clothes… how much do you use

PS01: Clothe? According to my line of work, it forces me to use 1500 shillings. Not this one this one you cannot use to because nobody will see me.

**I: Another clothe**

PS01: Another clothe, this is just one that I was I used to seek respect right now

**I: You buy clothes weekly?**

PS01: Yes

PS02: As number two, every week I have to make sleeping place well, so that even if a person gets in and see, they can come back again saying ‘that bed was made well.’ I have to change bedsheet, every week I have to come to =Usenge= to buy bed sheets. So that I can made good my sleeping place

I: How much do you use to buy the bedsheets?

PS02: I use three hundred shillings to buy a bedsheet.

**I: Three hundred?**

PS02: Yes

**I: Number three, you are nodding?**

PS03: I also support

**I: How do you support?**

PS03: I support, she has stated well that if you are a sex worker, if you do not make your sleeping place neatly, and a client comes and finds the place untidy. Do you think he might come back again? He won’t, he will go forever.

**I: You also buy bedsheets weekly?**

PS03: Yes, I have to maintain.

**I: How much do you use?**

PS03: I buy… there are bedsheets that go for three hundred shillings, just like she has said

**I: Okay**

PS03: So that is what we buy

**I: The others, what do you buy weekly, a week does not go by without buying? Is there something else?**

PS08: As number eight, every week I have to go to the market. I love mini skirt. I always have to look for a mini skirt, with an inner wear [pant], a nice one. That even if I take off my clothes I just see it is a good one. I have to buy that. I have to maintain to go to the market every week to purchase that.

**I: How much do you use to purchase them every week?**

PS08: I can buy a skirt of five hundred shillings, a pant I take of one hundred and fifty shillings

**I: The remaining people, those who have not talked?**

PS05: As number 5, every week I must go to =bondo=. I must spend around four hundred shillings. I must also buy liquor in =bondo= in a good bar, because I am a sex worker and there I can also meet a client that is not so bad. So that even if I come back the following day, I know I have around two thousand shillings, so I have to spend money weekly, I have to go to =bondo= [participants laughing].

**I: If you go to =Bondo=, what do you do with your money?**

PS05: If I go to =Bondo=, I have to use fare. I use a hundred shillings to reach =Bondo=. I use three hundred shillings to buy beer while checking out clients. I know that after what I have used, there must be a profit

**I: You must add a little something?**

PS05: Yes, so I must just go. Because at the center we are known these days so, we must just go outside

**I: Is there some things you do monthly with your money?**

R: Yes, monthly we also… (Participants laughs)

PS03: As number three, monthly I pay rent, I have to pay because if I fail to do so then… at times you think that… sometimes you have gone to do sex work outside, maybe you are someone who does sex work out of town. You might think that you have to do sex work and you left your kids in the house, when you come back you find that your kids have been thrown out and your door locked with a padlock. So I have to pay my rent on time, before my door is locked.

**I: I heard many people talk.**

PS07: As number seven, I use my money monthly, I have to go to a Mary-go-round. Because I must depend on the Mary-go-round, I save something small. I must also pay my rent. So that is how I use my money that I have personally worked for.

**I: To pay rent, to pay Mary-go-round. Another person?**

PS08: As number eight, every month I have something that I must do with money. I have a kid in high school that at the end of the month, I have to give the kid something in school. I also have to pay rent at the end of the month.

PS04: As number four, I support what number eight has said. My kid is in school so every month I have to make sure that I take something to the kid in school.

PS02: As number two, I support what number 4 and number 8 has said. You have to pay money for your kid in school. Another thing, I have to remember my mother, in that if I have money in a month, I have to send something to my mum. So that she knows that wherever her child is, her child is okay, she is doing a great job.

**I: Okay, you have talked about food and school fees and your mothers. Is there any other thing you purchase every month, apart from those? Okay, I see that you have shook your head. We said that instead of shaking your head you should just say none. [Laugh] okay, is there something you must do with your money, even if it is just once a year?**

PS03: As number three, what I must do with my money once a year, in church there is something known as tithe, so in a year a have to remove… it is usually collected in December so, I must make sure that I have the money by December, so I just give my tithe in church so that I receive blessings, even if I work, I become strong knowing that God is with me in my work.

**I: How much do you contribute in a year?**

PS03: In a year I contribute one thousand two hundred shillings

**I: The remaining people, is there something you do with your money yearly? A year cannot go by.**

PS08: As number eight. In a year I have to make sure I purchase something in the house. Be it sofa sets, coffee tables, I must buy something to put in the house. To know how I have worked the whole year, appreciate myself knowing that, ‘this year I have worked, and this is what I have for that.’

PS01: As number one, (inaudible) I have my mother, my sibling and my children. I usually make a big party for them, then we eat together as a family. I have no father, there is only mum who acts as both my father and mother. So I just sit with her, make a big party, so that my mum can see that we are not just this side but we care for her.

**I: How much do you use to make this party?**

PS01: Roughly, like we did it last year and I used twenty one thousand

**I: Twenty one thousand, okay others? What must you do yearly?**

PS05: As number 5, I support what number one has said. I also do not have parents but my elder brother is in =Suba=, and us his siblings are this side. So end year we go sit together, we contribute money, we celebrate remembering our parents, then we come back, and those who like remains. So we do that yearly.

PS06: As number six, the whole year is large, so when it comes to an end, I usually go home. We come together as a family, we see each other even those you have not seen in a long time. We use a lot of money because people are usually happy and are in celebration mode.

**I: Thank you all for the good answers. Now, women who do sex, where do you get your money you use?**

R: Repeat the question.

**I: The money you use, you have said what you do right?**

R: [All] Yes

**I: The money you use, where do you get them?**

PS01: As number one, I have an answer. The money we get, because we are sex workers, we use our bodies to get money. So it forces us to use our bodies to get money. So the money we use comes from sex work or these other places we hustle because… I have no job and the sex work is what sustains me. I cannot lie to myself that I will get 21,000 easily, where will I get it? I will have to get someone that I can talk to and agree, so that I can get the money.

PS07: As number eight…as number seven, I get my money through… because where I work I cannot depend on my salary, now I have to depend on my other ways. It forces me to go out, so when I go out and talk to someone, because in day if I weigh with my job, the money I get from my clients when I talk to five clients in a day. So if I talk to these clients, maybe I start with three and these three give me something. The remaining two give me something and the other three also gave me something. I just see that my earning is more than what I get at my normal workplace.

I: You have another job?

PS07: There is… as in I am employed, so I do not depend on that because it is a nanny job. Now this other one pays more daily

**I: In your daily, how much do you get roughly in a day?**

PS07: It depends because at times someone gives you five hundred shillings. At times a thousand shillings, sometimes someone sympathizers with you like, ‘this daughter of someone has worked well for me.’ And they give you even two thousand shillings.

**I: So averagely how much do you get?**

PS07: The highest amount reaches one thousand eight hundred shillings.

**I: One thousand eight hundred, okay.**

PS04: As number four, I get my money through sex work. I start… am in a bar I sit there. If someone comes and they want a shot, I give him, he gives me maybe five hundred shillings. I’ll sit there until late in the night, around 11-12pm at night.

**I: Okay**

PS05: As a sex worker, I already explained well am tired

**I: You are number?**

PS05: I had already explained well. That I can be in usenge and I receive a phone call that says, ‘hello, where are you, Dress up?’ I dress up, board a car and there I am. So in my work I have ten people, coming from different places. In Usenge, I only have one person, but most of my clients come from out of town. When am going to someone I must make sure that am going to someone who will pay well. I have a big budget, I have three people in secondary school, one person in primary. So for them to be where they are, I cannot go to someone who only gives me thirty shillings, or fifty shillings, or a hundred or two hundred, my clients give me from a thousand shillings, because I serve them well. So this work [sex work] is where I get my money and it pays well.

**I: Pays well. Okay.**

PS02: As number two, I support what number four is saying. As sex workers, where we work well is in a bar, someone comes and we talk, you go give him one shot then come back, you rest. You know there is… you will give even three people a shot each but then there is someone who would want to spend the whole night with you, so prices differ. If those who only have a shot give you five hundred each, then you have one thousand five hundred shillings, you keep. Then the person that wants to spend the whole night, you agree on the amount, he might ask, ‘how much do you want?’ then I say, ‘you can give me two thousand shillings,’ but if it is someone with a good heart, and he sees how you have spent with him, he might add something.

**I: Okay, they add money according to your work?**

PS02: Your good work is what pays you. (Laughs).

PS06: As number six, I support what number one said. We do sex work therefore you must just go out, and you need a good site that someone can find you.

I: How much do you get when you go out?

PS06: In a day I can make two thousand shillings

**I: Two thousand?**

PS06: Yes

I: Okay

PS03: As number three, I think that you should arrange your people. Maybe you have five people, maybe three of the people live close to each other. That if you look at it you will say, ‘if I follow there, one will see me, if I follow the other side, the other one will see me.” So you have to plan so that they do not clash when one sees you walking with another one. For you to get good money I can even arrange that I be transported using a motorbike while covering my face so that am not seen. Maybe that is where am going to get good money. I go covering my face because I know that I will earn good money.

**I: How much do you get in a day when you decide to go to work?**

PS03: Two thousand shillings

**I: Two thousand?**

PS03: Yes

PS08: As number eight, I work in a hotel. In that hotel, is where I get my clients? During the day… The following day I can plan my job and when it reaches daytime, maybe I meet someone, we go for a shot that my peers talked of. The day that the pub operates at night… I might make two thousand five hundred shillings or one thousand five hundred shillings. There are days that it becomes hard, especially the days that children break from school, there is totally no money at that time. You can just spend your time at the hotel and take the small you have made from the hotel. If you try so hard you might end up with only five hundred shillings

**I: Five hundred shillings? Okay, there are other jobs?**

R: Other job?

**I: Yes**

R: Like what?

**I: Apart from sex work?**

R: No

**I: Okay, why is it that sex workers buy what they are buying, you already listed what you buy, what do you… other people had stated the reasons. Number two said that she wants to dress so that if she passes by, someone must notice that number two is passing by. Right?**

PS02: Yes

**I: She also said that she buys bedsheet so that if someone visits, they think of going back, right?**

R: Yes

**I: What about the remaining people, the things you buy, why do you buy them?**

PS01: As number one, the clothes that I buy, I buy them for attraction. My grandmother would tell me, “Your legs are nice”’ Now I know that my legs are what, are nice in that mini skirt. So that if I wear and go out, just like number two said, those people will do what? They must like me. That is why I buy those things, I buy them to attract people’s eyes.

**I: Okay, and the people who… the things you said you use your money to buy, why do you use your money in those things, that is the question am asking. Number four smiled, do you have something to say**

PS04: The things we buy, we buy them to attract clients. The men are our customers. So if I buy a nice dress, a mini that is revealing my legs, that Is what the clients will see. I will attract him and he will come to me. So I must purchase a cloth that does not pass my knee going down.

**I: And there are other things you do with money apart from those that can attract the clients that you said. Why do you do them**?

PS04: As number four, I work in a hotel.

**I: Am saying the things you do with your money that you have made.**

PS04: Ooh

**I: There were different ones from buying clothes, buying bedsheets. Let us start saying them**

PS08: As number eight, the other thing I use my money on is like paying school fees. I pay fees so that my child may not have stress, in that she/he has been sent home for fees, because their mother has no money and they become stressed. I also pay rent so that my door is not locked by my landlord and I get the shame. I buy clothes to attract clients.

**I: Okay. Number five is trying to raise her hand. Do you have something to say? Nothing. Number one you were carrying your hand?**

PS01: Yes as number one, the things I buy that are not meant to attract the clients], I buy them to make my children happy. I can’t work hard yet children go to my neighbor to beg for food. I use my body so that my kids can eat, so I buy the other things so that my kids may not embarrass me with my neighbors. They know that their mother provides for them.

**I: I know you had already said those. I asked what you do with and you had already said, right?**

R: Yes

**I: Now we are moving to the next part. Do sex workers save?**

PS05: As number five, I have a savings group that I save. If at times I lack money, I can take some money from there and pay my kids school fee.

PS04: As number four, I support what number five has said. I have a savings group where I save my money. We go for meetings twice a month and we go with one thousand five hundred. So I try so that in the middle of the month I go with one thousand five hundred shillings, by end month I also have another one thousand five hundred shillings, in total I would have saved three thousand shillings in a month

**I: How often do you save in your saving group?**

R: Sorry?

**I: How often do you contribute in the savings group?**

R: We contribute five hundred shillings.

**I: That is number five. Okay.**

PS02: As number two, I have to… I have weekly meeting with my savings group. I have to carry seven hundred shillings with me weekly. This seven hundred, there is an amount I write under welfare, so that incase of any problem I have my group that can help me. So five hundred is for saving. That is money that I save so that incase I need a loan to help myself or my child, I just go they look at my shares and am given, then I can refund.

PS06: As number six, I also save weekly. On Wednesday we go for meetings, we go with around six hundred shillings. I save five hundred and then one hundred is for emergency.

PS01: I have nothing to say as number one.

PS03: As number three, we have a Mary-go-round. We contribute two hundred shillings each. We are fifty people, so two hundred shillings each and give to one person. So after that person I just wait for my number. So I just contribute so that when it reaches my number I am given my money.

**I: After how long do you contribute?**

PS03: It depends on the number

I: Is it weekly or daily

PS03: It is daily two hundred daily, so if we are forty people, then you are given eight thousand shillings

**I: Daily? Ooh. Is there any other person with something? The remaining people do not save? Okay. You have something to say. P8 Do you have something to say?**

PS08: Yes as number eight, I have a Mary-go-round that we contribute a hundred shillings daily. We are ten people. After ten days I know I have one thousand shillings, I can save that somewhere else. I also have a savings group that I attend weekly, we must contribute four hundred shillings. We save some, some we share on the table. The rest we can save so that you can take a loan. So mostly I can use around seven hundred shillings or one thousand shillings. That is the amount I carry to the meeting.

**I: You have no other ways of saving? It is only savings group. Is there someone who has another way of saving?**

PS01: As number one I use home bank.

**I: Home bank?**

PS01: But… I have kept it in a place you can’t really find even if you turn the house upside down [laughs].

**I: You have a box that you have hidden.**

PS01: No matter how much you try you wouldn’t find it. (Laughs)

**I: How much have you saved so far? Roughly**

PS01: When I broke it the last time, I was broke and my mum was hospitalized. I found seven thousand five hundred shillings

**I: Seven thousand five hundred shillings? That is a good sum of money. Okay, are there known characteristics of female sex workers who save? Are there some characteristics that if you look at a woman who saves you just notice, “this is someone who is saving.” The people who save, do they have some characteristics that if you look at them you can notice that they save? Have you understood that question?**

R: Yes

PS03: They economize. As number three they economize such that if this person buys something, they do not overspend. Let us say they want to buy bread and maybe she has two kids and she wants to buy five loaves. That way now looks like wasting. They buy bread that is enough for the family, if she has two kids she will buy one loaf of bread or even one family bread is enough.

**I: Okay, another person? These people who save, Is there a life they live that might tell you that this person is saving? This one I don’t think she is saving, do they have certain behaviors? [Long pause] now people are just quite. Number five do you have something to say.**

PS05: As number five I can say how I live, because I know how I get my money so even if I buy some things I do not overspend, I limit myself. I do not overspend knowing that I might lack in future. So even if I buy household things I buy them limiting myself so that I can remain with something for saving.

**I: Another behavior for people who save?**

PS03: As number three, there are those people that when they have money, you just know. When they coming towards you just look at them and know. They come maybe they are drunk, the whole road belongs to them and they say ‘nothing, nothing.’ They just say nothing and the person they are saying it to is not even there. The money he used to drink alcohol is what is leading to that. Meaning that you will just know they have money. There are people that even if they have money, you would not know. If they have or they don’t you wouldn’t know, they are just quite, they don’t show off.

**I: So there are those people that when they have money they take liquor?**

R: Yes

**I: Another thing? We are stating the behaviors of the people who save. And she has told one of the people who do not save, what about the people who save, she has told us one. Most of them buy extravagantly and they get drunk**

PS08: As number eight, how you can know that someone is saving. You can see them going to work and they work well. Sometimes they are your friends or even a sibling. If you go to borrow money from them, they always say they do not have money. That shows that they are saving.

**I: okay. She does not have because she is saving?**

R: Yes

**I: Okay**

PS05: As number five, I have a friend who is also a sex worker, you can go to hustle with her and we get good money but when you go to her house, there is no food, she buys a little food. Now we would just say this person is lying to us, she is keeping her money. Because she uses a little money, even if someone buys us a soda, she will drink some and the return the rest to the counter, she will be refunded that money while the rest of us we would drink up all of them. So we would talk behind her back and say this one wants to do something with her money, so that is what shows that they are saving.

**I: She is stingy while using her money?**

R: Yes

**I: Number seven now let us hear your voice.**

PS07: As number seven, from those people that save, you can find that someone has money but if they go to the market, if they want to buy something that costs two hundred shillings, you will find that they will bargain, ‘I have seventy shillings,’ and the seller would say, ‘no seventy is not enough.’ You will find that she will move to the next person. Meaning that she wants something that is cheaper while she has money, it’s not that she does not have money. So she calculates that the money she has, she must save most of it and use less. Even if you look at her kids you will see that the children are not in good shape. But she works hard. While her kid, if you look at their condition, you will just think even though she is working, she is suffering. Meaning that she is working but she is not getting enough money. She is the one who knows what she uses her money for.

**I: Okay we have talked about behaviors of those people that save, we have said that they do not misuse their money. You had used a term that I have forgotten when we started. [Laughs] now tell me, what are the characteristics of those people that do not save? How do they stay? How can they be identified that the way this person is behaving, she is not saving**

PS04: As number four, if you want to know a person that do not save. Even if she goes out, let’s say she is a sex worker, and she goes out and we get good money together, when she goes back, when you are going back to the house, she starts buying things, she makes sure that she has finished her money knowing that she will still get some. Even if it is beef, she will buy that of two kilograms, she will just buy food, and she does not have the mind to think that she should save. She will just use up that money knowing that she will still go out and get some. She is like a fisherman who eats everything.

**I: Tomorrow is still there**

R: Tomorrow is there

**I: Okay**

PS07: As number seven, those people that waste things. You find that you have gone out, you get same amount of money. They want to buy things worth fifty shillings while you have used five hundred shillings, she takes the rest of her money with her everywhere, she buys things, maybe she also likes food, if she passes by a Mandazi vendor she wants to buy, if she sees fries, she wants fries, she buys those small things, by the time she reaches home, she has no money left. That is when she thinks she thinks she should have saved at least fifty shillings, because she has used all her money.

I: The money must be fished for her to relax.

PS07: Yes. [Laughs]

**I: Another person. The characteristics of someone who do not save. Some of their behaviors that makes them not to save? Is there some or we have exhausted them?**

R: Those who do not save are people… you see that they show off. Even if you go somewhere, maybe a bar you will hear ‘give such and such a person alcohol, give them two, give the other people also.’ They are people that do not save, they just give out their money they are so generous.

**I: Alright, thank you very much. For the people who save, why is saving easy for them? What do… let me change it like this, what makes it easy for them to save?**

PS07: As number seven, I save because I do not know my tomorrow. So it forces me to work hard, telling myself that I cannot use all this money, because I do not know how tomorrow will be. Sometimes I may use all the money then the next day I fall sick, I sit down I do not know what to do next. But if I save even if I sit down for a week, I know how I can feed with my kids.

PS06: As number six, saving is easier for me according to the work I do, because if I go I know that I must come back with something [some money] so at least you are relieved, you use some and save some.

PS08: As number eight, saving is easier for me if I check my program that I may have, because at times I may want to do something and there is no one who can help, am alone. So I resort to saving because I know I can get enough money at once and do what I want to do the program I have.

PS03: As number three, saving is good because, at times you may lack money, you may not get money as you are used to, at times you may get a little money and other times you get more. So, it is good to save. The day you get little money, you use the one you had saved. That is why saving is good.

PS05: As number five, the reason why saving is good is because savings has helped me. I save a little to help myself. I can save to pay school fees, I can save to help myself so that I do not borrow all the time. After short while I am making phone calls. So whenever I go out to hustle, and I wanted to do something I do it with my money knowing that I work and I will get another money to save like that and it will help in other ways.

**I: Thank you. Is there Challenges you face when saving? Are there challenges while saving?**

PS01: As number one, the challenges I see when saving. If you are someone who saves on phone, in M-pesa, and you tell yourself let me withdraw two hundred shillings, right now life is hard that two hundred shillings is only for breakfast. So, if you withdraw the 200 to buy food for my children, two hundred is not enough, let me add fifty shillings,’ you will continue doing that until you remain with nothing. So you end up wondering, ‘this money, I had four thousand shillings, now it has reduced to two thousand, and you had targeted to save a certain amount of money. Sometimes you tell yourself let me take this money or sometimes you save money in bank, you have withdrawn until you remain with a little money, you try to withdraw, transaction fails, it has remained a little money that you cannot withdraw. So you know saving has its challenges, sometimes you have saved you realize you have saved a lot, you are remaining with a little money. Sometimes you save little you remain with excess, you end not spending it well.

**I: Okay. Other challenges we encounter with savings? I saw many of you raise their hands. They have disappeared?**

R: The challenges I see while saving is that when I want to save something comes up. But I have decided in my heart that when I want to save I will save no matter what. That day I rather stay hungry, I can even eat vegetables for three days in my house for me to save. So when saving, I go through a lot, but the money will also help in future things will be nice for me.

I: Okay, so the challenge of now people having to stay hungry, how do you handle it?

R: That time I persevere

**I: What about the kids?**

R: I just give them vegetables.

**I: They don’t go hungry totally? At least they eat something small?**

R: Because normally I give them good food, so when I give them daily they just know that am in a good job. So at times I must show them that sometimes I see challenges in my line of work, they must even eat vegetables at least for three days. The intelligent one says that Mom has money but has saved. So when saving they must face the consequences, my kids cry that they do not want to eat vegetables. But I turn a deaf ear, because I am trying to secure their future, mine is already gone.

**I: Are there any other challenges? Okay if there is none. FSWs who don’t save, what makes them not to save? We had already said some. Is there anything you can add?**

PS04: As number four, female sex workers that do not save are women that see that they have got a lot on that day, they spend all of it knowing that the next day they will still get more. That is why they do not save.

PS02: What I see for example, I have stayed with a friend of mine, we were with her in =mbita=, I had kids but the other three did not have kids. Now when we would go and get good money, the wanted to introduce me to their bad behaviors, we would stay in bars, we do not go back to our house, until we finish all our money. I later realized that these women were influencing me wrongly because they would do that, the next day they receive another money, while on my end I had to save because I have kids.

PS06: As number six at times life can get tough. Sometimes you have gone to work, you get some little money, you find that the budget is higher compared to the money you have. So you just decide to use that money, instead of keeping it and end up suffering.

PS03: As number three you know someone who has a child is different from someone who does not have. If you have a kid you cannot save, in that your kid lacks you cannot even buy them food to eat. It forces you to…even if you have a little money you cannot save it all, sometimes that money is so small that you cannot save and still buy food to eat. So you just decide to buy food, and you will save later, you must buy food though, at least for your child

**I: Okay. Now, we had talked about the disadvantages of not saving. Are there other disadvantages for not saving? Let us start this side**

PS06: As number six, one disadvantage is, you might have an emergency, there can be a severe disease attack even at night and if you did not save then there is nothing you can do. You think of what to do but there is nothing.

PS04: As number four, I support number six on what she has said because if you do not save, your kid might get sick even in the middle of the night, and you do not have any savings. If people ask you to give even the little you have, you don’t have even a shilling. So that is what I would say is a disadvantage for not saving.

**I: Number two?**

PS02: As number two, I think that failing to save… saving is good, because it may not be business as usual. You might go to work, and that day you get nothing. Because you might return home and find that… maybe you come home with something small, which might not be enough to sustain you till evening. So you decide that ‘if I save, I might take something from there so that the life of my kids goes on as usual’ Instead of not saving

**I: Okay. Is there any advantage to not saving? Any advantage**

R: An advantage of not saving?

**I: Yes**

PS01: There is no benefit. I am number one, I support that saving should be there because if you do not save there is nothing good you will see.

PS06: As sex workers most of us are not married, so the challenges that we have, I do sex work but I save, I might be better than a married woman, because my family is fairing on well. Am called at my kids school, they want fees, I go to my savings and sort it out

**I: For those who do not save, is there an advantage?**

PS06: So failing to save is not good, because if I do not save then there is nowhere that I will get that money.

I: You have said that?

PS06: That failing to save is bad…

**I: That is number six**

PS06: So I have to save

**I: Okay, so where do sex workers save? Where do you save?**

P: We save on phone

I: Phone, there are many ways of saving on phone, which one?

P: M-pesa

**I: M-pesa.**

PS04: As number four, I save in saving groups.

**I: Saving groups, okay.**

PS07: As number seven, I save in daily Mary-go-rounds.

**I: Okay**

PS01: As number one, I save in home bank.

**I: Okay. Why do you save in the said places, why do you love the places?**

PS04: As number four, I save in saving groups because I would not go take money there anytime I want. There are rules laid down that prevents one from doing so

**I: You cannot take money anyhow**

PS04: Yes you cannot

**I: What of home bank, why do you love saving in home bank?**

PS01: Me? As number I save in home bank because the home bank that I have is that which is welded, it is made in such a way that it is not easy to break. For you to break it, you have to return it to the welder. I do not have the tools to do break it, so home bank is safer compared to saving groups.

PS03: As number three I support number one, saving is good.

**I: You said M-shwari**

PS03: No I said M-pesa.

**I: Why do you love saving in M-pesa?**

PS03: I save in M-pesa because something can come up abruptly, and now I might want to withdraw my money, I just go and withdraw and sort my issue.

**I: Are there other places that you save? Apart from saving groups, M-pesa and home bank. People spoke of saving groups, and the saving groups…oh she has told us why she saves in saving groups. What she has said, is why the other people are also saving in saving groups?**

P: Yes.

**I: Do sex workers live in a life that is higher than their ability?**

P: No.

PS05: We live in life that matches our income.

PS02: As number two, I cannot live in a life that is way above me. I must live in a life that matches my earning. So that I know how I can help myself well. If I live in a life that is above my earnings, then I cannot survive.

**I: Okay, what about you, why do you live in a life that is affordable to you?**

PS05: As a sex worker

I: Your number?

PS05: As number five, with the type of work I do, there is just a life that I must live, according to my earning.

**I: What about other people?**

PS01: As number one, I cannot raise standards of my living while my earning is low. Do you know what happens, you cannot raise your standards when you earn little, the space that will be left, who will fill it? If I have an average earning, then my standards will just be average, so that I get to save that will help later. Instead of raising the standard of life and my kids might get into robbery. That is not good.

**I: Okay. The other people, do you live a life that is higher than your earnings or what type of life do you live?**

R: In the middle [whispers]

PS08: As number eight I just live a life that is in accordance to my earning.

PS04: As number four, I live a life of my choice. Just according to my earning. A life that matches my earning

**I: Why do you love to live a life that is equivalent to your earning?**

PS04: Because I cannot a high standard of life yet the money I get is small

**I: Okay, number seven?**

PS07: As number seven, I live an average life, where God has placed me, because I cannot raise or lower it. So where He has placed me is where I’ll stay.

**I: Do you love borrowing? Do you borrow, do you have debts?**

R: [All] Yes

**I: Okay, tell me in which way?**

PS03: We borrow money. Borrowing money is good but… as number three, I am number three, I borrow money, borrowing money is good, and when you borrow it could be of help. But disadvantage is when the owner wants their money back, now stress hits, you don’t know what to do, you even hide from the lender. Because they want the money anytime they see you, even when you don’t expect them, they come to your house. If she gets your kids she tells them, ‘tell your mother I want my money.’ So that is the disadvantage.

**I: Who do you borrow from?**

PS03: Me?

**I: Yes**

PS03: I borrow from my savings group. The people that I am with in that savings group.

**I: You Borrow from people or the savings group?**

PS03: I borrow from the people that we are with, in the group. Even if I was to receive maybe five hundred, I tell them to top it up to a thousand shillings I will refund.

**I: Why do you borrow?**

PS03: I buy food with it or maybe at times my kid does not have uniform I buy them uniform, or even paying school fees.

**I: All the people said they borrow, so I want an answer from everyone as we move. If you borrow, you borrow from where and how much?**

PS04: If I borrow, I borrow from my savings group. As number four, I borrow from my group where I save my money. I can borrow three thousand, take to my kid for school fees.

PS02: As number two, I can borrow because I might want money to go do something with it. There are people that I work with, I might borrow three thousand, and then I refund three hundred shillings every day for ten days, then am done.

PS06: I can borrow in my savings group as number six. At times I borrow from my phone in M-shwari to sort out my issues that I have.

**I: Okay.**

PS08: As number eight, I borrow from my phone that is Fuliza [Ability to transfer more money than what you have in your M-pesa Account] when am stuck, I may want to do something and the money is not enough, I take Fuliza. That is where I borrow.

**I: The remaining people where do you borrow?**

PS07: As number seven I also borrow savings group. Because that is where I save in that if am stuck and cannot pay I simply tell them that today I cannot, take from my savings to pay. So I borrow from my savings group.

**I: And how do you pay your debts?**

PS01: As number one, when I borrow money, when you borrow money… like pesa pal, they are people that understand, he won’t ask. You will just tell them just give me this amount, I will pay today, tomorrow do not come, just like that and the days will go by until one day you finish.

PS07: As number seven I borrow from my saving group. If you borrow from there, you are given a day to pay back. You might be told to refund after two or three months, so it depends with the amount of money you have borrowed.

**I: The person who borrow from Mshwari why do you borrow from mshwari?**

PS08: As number eight I borrow from Fuliza

I: Fuliza… yes fuliza

PS08: I borrow from Fuliza because I know Fuliza I will borrow and I will be given at that time that I want it. And from the next day, if I get something small, I deposit and they take it.

**I: The other people, how do you pay the money you borrow?**

PS05: As number five I borrow from my savings group.

**I: How do you pay it? Where do you get the money to pay it back?**

R: The money that I use to repay?

**I: Yes**

PS05: that comes from… now that depends with how with how I get it. If I go to work [sex work] and get maybe three hundred, if I go to our meetings I use that to pay.

I: You refund little by little?

PS05: Yes

**I: Okay what do sex workers do to increase their earning? What do you do to increase what you earn?**

PS06: As number six what I do to increase my earning is, I become clean. Tidy up my place of work so that clients can come in good numbers.

PS03: As number three I support what number six has said. That you must tidy up your place of work, so that the clients can see that you are a clean person. That Is where I will go, a clean place is where I will go

**I: So if you tidy up your place of work, do many people come or what happens**

PS03: You attract clients, the clients that come for your service.

**I: So does that make the increase in number or what happens?**

PS03: No, now your cleanliness, how you maintain it, that is what makes them say, ‘I just want that girl because she is clean.

**I: Okay.**

PS02: As number two, what I do to attract clients is, I must tidy up myself well, even my clothes, and I hear people say that I look good in shorts now I love shorts. I go buy a nice little short that if someone sees they say, she looks good. Because even if I wear my clothes, they just see that this place is good, am going to a good place.

PS04: As number four what I can do to attract clients is talking well to them. You might dress nicely but you don’t talk well with them, tomorrow no one will come back. You must serve them well.

**I: So to increase your earning, you must just talk well**

PS04: Talking well to the clients

**I: Are there other ways that you can do to increase your earning apart from attracting your clients? [Long pause] is there something else you do apart from…**

PS02: Apart from sex work or?

**I: Even apart from sex work**

PS02: Yea that is also there we do them.

**I: Okay?**

PS02: Like me…

**I: What is your number?**

PS02: I am number two. During the day am in the market, I sell vegetables to increase my earning. At night I do sex work.

**I: Let us talk generally. Okay?**

PS02: Yes

**I: Let us not dwell so much on sex work, we can also say the other things we do okay?**

R: Yes

**I: Okay, other people?**

PS04: As number four I have a hotel. The hotel is inside a bar, now that is where I hustle.

PS07: As number seven, I work in a bar, I work there and that is where I hustle.

PS03: As number three I have a cosmetics shop, so during the day I sell there but I use my phone to connect to my clients so if it reaches at night, we just meet.

PS08: As number eight I also have a hotel. That is where I work and earn and that is also where I meet my clients for my sex work.

**I: What do you do the day that you have not found a client? This lady said that there is a day she went to =Bondo=, but she did not find a client, now what do you do in such scenarios**

PS05: As number five, when I decide to go to =Bondo=, I only go if I have received a phone call

**I: Okay. Are there times you can go to work and find no clients?**

PS02: As number two if I go out for work, I find someone and they refuse to pay, I have the right to report him to authorities because that is my job. He must respect my job and pay me, if he does not pay me, then he will pay me at the police station.

**I: Is there a day that things are hard, you do not find clients**

PS03: As number three what I know is as a sex worker, we come into an agreement first. Before we go we must agree. We agree maybe we are in a bar, we take liquor and agree beforehand, if we go for two shots, it is such an amount of money, you give that to me first, when we go into a room, he gives me first, I keep it then we can continue. Not that after he has finished he tells me that he does not have money, you go home without anything. We will not agree.

PS05: As number five just like I had said, people are ten, and not all of them are here. There are some of my people who know that I have a family, we had already agreed on that, they know that. Before I make a step and go to them, I receive my money on Mpesa, so what took me there is what I do, get fare the next day am back.

**I: So there is no day someone can go to work and fail to find a client?**

R: At times we don’t find them

**I: The day you don’t get a client what do you do? That is what am asking.**

R: The day I don’t get a client, I use my savings, I go to mpesa and withdraw and if I had drunk something or maybe I want to go back to the house.

**I: So you use your savings?**

R: Yes I use my savings.

PS02: As number two, let us not lie to ourselves. You must have one person, that one person that you trust that knows that, ‘that lady…’ it is not a must that he must come around this place, I am just with him and he knows that, ‘that lady, I am with her and I have to do this and this for her.’ He must help me. He must know how I eat in that if I go to work, I know that he caters for how I eat.

**I: So there must be that one person that gives you…**

PS02: Supports me and my house

**I: Did you carry your hand number six?**

PS06: As number six sometimes you might find it hard at work. Now we have other business that sometimes we can get our money from.

**I: Is there someone else? Okay. We said that we have debts right? How much debt do we have? Let us just say, you say your number and then talk**

PS04: As number four, I have a loan in my savings group that sums up to ten thousand

PS05: As number five, I have a debt of nine thousand.

PS02: As number two I have a debt of one thousand five hundred shillings

PS06: As number six, I have twenty.

**I: Twenty shillings?**

PS06: Twenty thousand.

**I: Ooh twenty thousand…**

PS03: As number three I have fifteen

**I: Shillings?**

PS03: One thousand five hundred

I: Ooh fifteen hundred.

PS08: As number eight, I have three thousand

PS01: As number one I have one thousand five hundred shillings.

PS07: As number seven I have twenty five thousand

**I: Twenty five thousand…**

PS07: Yes

**I: Okay. Now let us talk about leaving sex work. Do sex workers ever think of when they can leave sex work? Does it come to mind?**

PS01: As number one that comes to mind all the time. Because I yearn for something I can do so that I can leave sex work. But there is nothing I can do, so it forces me to do sex work. If I had something else I would not have opted for this. So it forces me because there is no other job.

**I: Do other people think of leaving sex work?**

PS08: Yes as number eight I usually think that in the next five years, I can leave it because my kids would have reached another level. Maybe I have finished paying for their secondary school fees, they are in another level. So I plan to leave in five years’ time.

PS04: As number four I had not thought of leaving it, but according to what you have taught us today, it tell me that you can leave sex work, and start saving and become someone that do not do sex work, and find a way to help myself.

**I: Other people? Okay, is it something you talk about, when you are with your colleagues? Do you talk about leaving sex work? As in if you are with your friends, do you talk about it?**

PS01: As number one where I stay In =Imbo=, if you tell someone that you do sex work, they will start gossiping about you. So it forces us to talk in between ourselves, for example if I am used to number two, I will tell her ‘let us leave this work and find some other work.’ If number two does not like idea, I will go to number three or number eight, we make stories because if we tell an outsider they will talk about us with other people instead of teaching me.

**I: So I was talking about fellow sex workers**

PS01: These people?

I: Yes

PS01: I talk to them about it

**I: Do you talk about it?**

PS01: Yes we do especially with number two.

**I: What brings about such conversations? There must be something that leads to such a conversation, tell me**

PS01: What brings about such conversations…?

**I: Say your number**

PS01: As number one, what brings these conversations is because sometimes the field is not good [there are not clients]. If schools are closed, the field is bad, you can go to work, like me I go to work during the day because I have a spouse. So it forces me to do sex work so that I can succeed. Now I go to work during the day, no one will even signal you to go. The field is so bad, children have closed school. Even that fifty shillings you used to… sometimes they give only fifty shillings and they are done.

**I: The money has reduced (participants laugh)**

PS02: As number two when schools close the charges go down. Like during corona, the money was so small, we suffered. There was nothing [participants laughing]

**I: Did you think of what I am asking you.**

PS02: Yes. You know it reached a point that we almost quit I told number one, I told her that, now am thinking of leaving this work, because I was getting nothing.

PS06: As number six I thought about it so much. Because at times you go to get and no client comes now you tell your colleague, ‘what do we do, should we leave this thing… should we leave it there is nothing we can do.’ Now at times that thought comes that we should quit it.

PS08: As number eight what can bring such a thought to mind is that at times you get a client who has collected firewood [has a big penis], and he would work on you thoroughly, now you might think of leaving sex work.

**I: If you say he has collected real firewood what do you mean (participants laugh)**

PS01: As number one, he has a huge penis (Laughing)

PS08: He is carrying something serious [a very big penis].

**I: Ooh something serious?**

P: Yes (participants laughing)

**I: Okay now…so if he has collected the firewood you can think of leaving?**

PS03: As number three we had already talked that you might find the field unfavorable, so at times you might go and on the first day the field is bad, second day the same, third day the same, you now lose hope. Once you lose hope now automatically you decide to leave sex work.

**I: Okay so these are the things that can make you desire to leave that job?**

R: Yes

**I: Not getting client, big penis, you go to work severally but earns nothing. When someone has decided to leave the job, do they leave at the time they planned to leave? ‘I want to leave I have…’ number eight said after eight years, if someone has decided to leave this job, if the time comes do the leave it?**

PS01: As number one this job on one side is hard to leave on the other side it is easy to leave. Because you might leave and your earning will reduce. Like the business of number two is doing, at times you have onions and they end up spoiling that you don’t have money to pay the suppliers. At times she is given a crate of tomatoes they pay back after sales and the tomatoes end up spoiling, now you end up going back to sex work to pay back. That is a disadvantage of leaving sex work. Advantage is once you leave it…we are usually told that…they say that exchanging sex with different men end up spoiling your body, now the advantage is once you leave it your body goes back to its normal state. Now leaving sex work is easy and also hard.

**I: Do most people leave it at the time they had planned to leave it? Is it possible? I don’t want the nods I want you to say with your** mouth.

PS03: As number three I think someone cannot just leave sex work at once, they leave it slowly and they end up leaving it completely.

**I: Yes…?**

PS06: As number six, someone can leave sex work, if she has planned to leave.

PS08: As number eight I think that I can leave it if I had already planned. Because I can arrange that this year am leaving, and if it a house I don’t have, I build then the next year I save and if I do not have land for farming, I buy it. Then the next year I save for school fees and when my kids finish school, I think it will now be easier to leave sex work because I have something to do. I have a place of my own, land for farming and so I can now I can just leave sex work.

**I: People leave sex work at what age?**

PS01: As number one according to my thinking, I think that someone who has left sex work, is usually around forty years.

**I: Forty years… what do other people think? Do age…**

PS03: As number three what I know is, what I usually hear people say is once someone has stopped seeing their periods, she can leave.

PS06: As number six, I think age does not matter. It depends with how someone has saved, the good things you have from that job, you can now decide that now I can leave sex work and continue with my new life.

**I: You have said that age does not matter… number two do you have something to say?**

PS02: I have nothing to say.

**I: Number seven?**

PS07: As number seven I think that age does not matter because, you might find that someone is older in age but the body is still young, in that if someone tells that that person is fifty years you would disagree. Because the body is still young and If she dresses well and sits down, instead of looking at me, you would be looking at her. So I think age is not an issue.

**I: and…**

PS08: As number eight I think that age matters, the reason is when I reach forty years and maybe a have a girl child who has reached 18years. We might end up sharing the same man, so I think that by forty is a good time to retire.

**I: Okay number eight thinks that forty is good. What do sex workers do after leaving sex work? What do most of them do?**

PS01: As number one I can just say that a sex worker who has worked and has been paid. And that is the program she got involved in, one that enables her to save. it will reach a point that even if the woman leaves sex work, or if we leave sex work and the saving are invested into business

**I: What type of business?**

PS01: Like selling vegetables, open a small hotel, and life continues

PS03: As number three I think they can even start a business of brewing alcohol. That is ‘chang’aa’ (local brew)

**I: Yes chang’aa, what work do they do? Once they quit is there a change they experience in your opinion? According to you? Is there a difference in their life?**

PS03: As number three, I think that if they saved well, they would not have much challenges because at times you find that her business has promoted her until she becomes someone big and does not think of going back to sex work.

**I: Do you know any time that a sex worker retired and came back to sex work? Does anyone know of anyone?**

PS03: As number three maybe someone can return, maybe they thought the business they opened could expand but that does not happen and she continues having losses. So she decides that she should just return to sex work to earn the money she used to earn there.

**I: Do you know of someone who has left and returned? Number five…**

PS05: As number five there is someone I have seen with my own eyes. She did sex work in =Mombasa= and she returned to our village. So she returned after leaving the job for so long and she built a house for her mum, paid her little brothers school until he finished. Then I saw her going back. She has not returned since then.

**I: Is there something bad that happened to her because she left sex work?**

PS05: No

**I: Okay none. Is there something good that comes from returning to sex work? Is there something good that happens? There is no one here who has seen someone apart from number five. Okay, what would sex workers want to achieve before retiring? She already said building a house and paying kids fees, is there something you would want to do before you retire?**

PS08: As number eight I would want to buy a piece of land and build a plot, build rentals so that once I retire I can get money monthly. I would also want to build a posh mill that I can get some money daily so that if I decide to retire I know I am in good hands.

**I: Have you started working on buying a plot?**

PS08: I am planning on that

**I: Ooh you are still thinking. Is there something that one would want to do before they retire? The remaining people…**

PS05: As number five am thinking that… I want my first daughter to go to college, that is something that I think of, my son who is in form three to finish form four. After that I can retire and look for something else.

**I: Okay that is something that you are already doing?**

PS05: Yes

**I: Okay, another person? Something you pan to do before you retire? You say, ‘once I’d this, I will retire.’ Or I want to do this before I start thinking of retiring. Is there something that comes to your mind like that? Now people are quite, I want you people to speak. Okay there is none, okay. Do you know of any sex worker who has retired in the last five years? Just say your number and state yes or no**

PS04: As number four I have not seen.

PS05: As number five I have seen

PS02: As number two I have not seen

PS03: As number three I have seen

**I: What made them retire, or made it easier for them to retire?**

R: The one that retired they killed… being a sex worker, they killed someone because of her.

**I: That is why she retired?**

R: She retired

R: She retired because there was someone they were fighting over a client, so she decided to retire

PS01: As number one, the one who retired told me that, sex work is good and it pays well. But there is a time that it is not good in that if she does not plan well, there is nothing. So she retired because at times the money was not enough.

**I: Okay. Now we are on the last question. We are going back to jitegemee. I told you about Jitegemee study, I said that this study is being conducted to know that sex workers have money to save so that they can refuse unprotected sex or they can take a break from sex work when they want to rest. I also said that it is about sex workers saving their own money. You save the money you have so that they can use it when there are no clients or can help them in future after retiring from sex work, okay? Can sex workers in Kenya love Jitegemee? Is it something that people will love?**

PS06: As number six, people will love Jitegemee

**I: What do other people think?**

PS04: As number four, I also think that people will love this study

PS05: As number five people will love it

PS02: As number two I think it can be really good

**I: What type of sex workers would love it?**

R: Pardon

**I: What type of sex workers would love it? What do you think, character. What type of people would love it, would type of people would not love it?**

PS01: As number one I think that this study, the people that would love it are people that wish that one day they can find a way to leave this work, and find something they can do. Or they are people that have done this job for long and have not seen anything good about it, so they wish that this jitegemee can develop them so they must love it.

**I: Thank you for the input. Another person? Now am not seeing hands…**

PS08: As number eight, what I think is more the same as that of number one. That for people who have worked for long, or for people who now have their own family, would wish to love the program. I think for the people who are just starting they might not like the study but for people like us who have worked for long, we might love it.

**I: Why won’t new people love it?**

PS08: As number eight, people who are just starting might not love it because they have immersed themselves fully in sex work

**I: Their whole mind is in it, okay**

PS04: As number four people who are just beginning may not love to leave this job because they see that it is money that is coming in fast

PS03: As number three, people who just starting believe that maybe that is what can change their lives so they see that every day they will receive a lot of money and that would change their lives.

R: People who are starting would not love Jitegemee because, when you start jitegemee and you have good luck, you get a lot of money, so you think that every day will just be like that. So you think that even if you do not join jitegemee even if you do not save, tomorrow you will still get money.

**I: I know we all have friends, do you know of ten women who are sex workers, out of the ten… I want everyone to speak, out of the ten women you know, how many can accept Jitegemee?**

PS08: I know of two who can accept.

**I: What number?**

PS08: Oh am number eight

PS03: As number three I also have two people who can accept Jitegemee.

PS06: I can get one person

**I: One person?**

PS06: Yes

**I: I am not saying the people you are sure of. Am saying the people you think can accept. Number two?**

PS02: I know of three people

PS05: I have many people but I know five people can love it

**I: How many people can love it?**

PS04: As number five I know of two people who can love it.

PS07: As number seven I also have two people

**I: Out of ten people you know?**

PS07: Yes

PS01: As number one I have around five, six people who might love it if I shared with them

**I: Okay, for the women who would not love, why would they not love it? Out of the ten you only said 2, the remaining eight why would they not love it. Because I was asking out of ten women you know right?**

PS08: As number eight out of ten women I know, I said two because am only close with the two, the rest we are not so close so there is no way I can tell them.

PS03: As number three am thinking that they may not like it because they think that if they leave sex work, there is no way they will live. They see that if they depend on themselves the money they are used to getting every day they won’t get it. So they just want to remain in sex work.

**I: They only depend on themselves when they are receiving something, because when she is getting money that Is when they can say they are depending on themselves, that is jitegemee right? The people you are saying cannot join, why won’t they join?**

PS06: As number six they may not join because most of them started sex work recently. So at times they see that they are still earning good money.

**I: Ooh of the people you know?**

PS06: Yes

**I: let us continue**

PS02: As number two, the remaining people, the three people, someone can tell you … you are advising me that we should depend on ourselves but at least you have your own business. I do not have any business and I depend on sex work, now what do I do?’

**I: Okay what can we do for people to accept this study? What should we do so that people can accept it, what can we add?**

PS04: As number four I think that we can start a group of ten people. We save money, and when we have saved a little money, we can go and buy iron sheets for one of us, then the others can see. They would say, ‘the people that started that group they have reached somewhere they have even bought something for one of them.’ The next time you again buy for another person and they just see us buying things. It might want to leave that work and join us.

**I: Okay you have said that we create a group and buy things for the others to see, what else can we do so that women can accept Jitegemee?**

PS03: As number three I can convince the other women to retire, because at times you can tell them that work is just work, don’t choose. You can do some other work apart from sex work, even if you get a sweeping job, just sweep as long as you can get money to save. Or you can go cook for kids in school, you can convince them like that, any job they get they should not lose hope. Should just work as long as she is earning some money to save

**I: This study what can we do for sex workers to accept it? Another person?**

R: Repeat the question

**I: What can we do for many women to agree to join Jitegemee. What can we do in Jitegemee for other women to love it and join?**

PS06: As number six I think Jitegemee can plan their program and call women then teach them. They can increase peoples understanding

**NT: If they teach, what are they teaching about?**

PS06: About jitegemee

**NT: On what topics.**

PS06: About saving

**I: What should Jitegemee have for people to accept it? What should it have for people to accept it? Now people are quiet.**

**NT: We are almost finishing. (Participants laugh)**

PS03: As number three I think Jitegemee should have a group that they have formed so that they agree how to... maybe if they come contribute to individuals in turns so that everybody can get a way to maybe start a business

**I: Should have groups**

PS08: I think Jitegemee should have loans because women love loans, so that it can pull them.

**I: Yes loans, there are good thoughts here, another one?**

PS03: They should have Mary-go-rounds, as number three I think that after forming groups they should have Mary-go-rounds in that if one person is given this time then next time another person is also given. They decide a business they want then start it.

**I: Before number three, it was number eight that talked? Okay another person, it should have loans, groups, yes…? Is there another thing? Number three still wants to…**

PS03: As number three it should have the strength so that even if given a small money they can have something to add on their work. Not that after a few days that work has flopped.

**I: You know anything good we say must have something negative about it, right?**

R: Yes

**I: So what would you not want to see in Jitegemee? What don’t you want to see happening in Jitegemee? We have said that it is for saving right?**

R: Yes

**I: what don’t you want to see in this study?**

PS04: As number four what I would not like to see at jitegemee… as a group in here we can form a group so that we can be contributing small money then save, then you will find those people that came to teach us, go and we never see them again. Plus the money we have contributed get lost with them

**I: The money gets lost**

PS04: Yes

**I: number one you have something to say?**

PS01: No

**I: Number two?**

PS02: I have nothing to say.

**I: We should just do it like we explained it or there is something you don’t want to see done? Or there is you usually think that ‘that group collecting money should not have this?’ that is what we are asking, is there something it should not have? Number eight is there something to say?**

PS08: As number eight I don’t have anything to say

**I: You have nothing to say, okay what would sex workers love about Jitegemee? What would love about Jitegemee? We had already read that. The things I have been reading on jitegemee saving, being that you cannot… what would you love about it? [Silence] you love nothing about it?**

R: We do not understand that question

**I: Ooh, what would sex wokers love about Jitegemee. Let me read in english. What would female sex workers particularly like about Jitegemee, what can they love in jitegemee, just like I have read it?**

PS01: As number one, Jitegemee has taught us on how to save for future use. I love it because it can help me and take me somewhere, I save for tomorrows’ purpose

**I: So you can save for tomorrows’ purpose that is the future. What would one love in Jitegemee?**

PS01: As number one, I think Jitegemee would one day take me…one day lead me from being a sex worker because as long as it remains out, jitegemee would help me on how I can depend on myself without necessarily depending on these men.

**I: Okay, another person?**

PS03: As number three I think that when you depend on yourself you become proud, it depend on how you were saving, if you were saving well. Now just know that you will depend on yourself

**I: You have some joy…**

PS03: You don’t have that mind that someone else will give you.

**I: Okay. Are there things about Jitegemee that can violate female sex workers rights? Are there things about Jitegemee as I read it that you think will mess with your rights?**

PS06: As number six I do not see anything bad that can violate our rights

PS04: As number four, I support number six, I also do not see anything bad that can violate our rights

**I: The remaining people is there any way your rights can be violated somewhere?**

PS01: As number one there is no way you can violate our rights because it helps us

PS05: As number five I would say that there is nothing that can violate our rights

**I: There is nothing violating your rights?**

R: [All] Yes

**I: Okay. We are finishing. What challenges do you think we can we get because of what we are doing. And what advice can you give us to overcome them when we do Jitegemee? What challenges do you think we can get?**

PS04: As number four, I think the challenge we can get is you want people to sit together and you come after talking to them then find no one.

**I: We do not get people to join. Number seven will we get any challenge?**

PS07: None

**I: That is number seven.**

PS01: As number one there will be a challenge because at times you will talk to people like us but when you come back the next day, all these people will disappear, let them not lie to you that they will stick to this thing, all of them will Stop participating and you remain alone. Jitegemee is gone right? So let them not lie to you that there will be no disadvantage, there is.

**I: Okay another person who knows the challenges we can get? I said that there is no wrong answer, do not be afraid. I see number six raising her hand then lowering it again (laughs)**

PS06: As number six the challenge you can get is, you have come from far Kisumu, Siaya, you have used fuel while coming then you don’t find people, so that can be a bit hard.

**I: Number eight what challenge can we get while doing Jitegemee, we are finishing, we doing the last rounds.**

PS08: As number eight I do not see any challenge that you can get.

**I: We are just right. Number 2**

PS02: As number two you might have a challenge, because you come then you fail to find people that is a challenge. You are supposed to come then you find people and talk to.

**I: Okay, now the challenge of not finding people, how can we overcome that, what can we do to find these people? These people that we cannot find.**

PS01: As number one for you to avoid the challenge of people not to stop participating, early communication can help in everything.

**I: Early communication, another thing? That can make us find people. The challenge is just finding people.**

PS05: As number five I see that whatever you are doing now is good because, we have someone here who alerts us. That such and such a date we have visitors, so let us try and come out in numbers. So they remind us. So we just wait for you and the time you are coming. So you don’t fail to find us.

**I: Number three you had a challenge right? Do you have another challenge?**

PS03: No

**I: Okay, how much would you like to save weekly, that does not mess your program of getting your basic needs? How much can you save weekly? Number five, children are still eating, you can still save. How much can you save while the children can still eat and still go to school? How much can you save weekly?**

PS01: As number one I can save seven hundred

I: Seven hundred…

PS07: Five hundred, number seven.

**I: Yes number seven**

PS04: One hundred. Number four.

PS02: Number two, five hundred.

PS06: Two hundred number six.

PS03: Number three five hundred.

PS08: Number eight two hundred.

**I: Yes. Where can we save these money, where you are comfortable with because this is your money? Where can we keep them safely?**

PS03: As number I think in the bank

**I: Bank account?**

PS03: Yes

**I: Yes. Number five you can carried your hand? Where can we keep your money?**

PS01: As number one, I just support my home banking.

**I: Home banking. Everybody to keep their money in the house.**

PS02: As number two, I support my savings group that I save weekly.

**I: Save weekly in a savings group**

PS06: As number six, phone

**I: Phone, yes…**

PS05: As number five, I usually save in my savings group.

**I: You trust your savings group more?**

PS05: Yes

**I: the person that chose her phone, where do you save we have KCB, MPESA, MSHWARI, they are many.**

PS06: Mshwari or Mpesa

**I: Mshwari, yes…the other people where would you want your money kept?**

R: I usually save in KCB

**I: KCB, bank or mpesa?**

R: Mpesa

**I: Mpesa, okay. If you don’t reach the amount of money you want to save…you have said that you can save four hundred, seven hundred, and three hundred. You have not reached that amount, what can you do to reach the remaining balance. Like you say, ‘I will be saving five hundred shillings weekly,’ but you have not reached that amount, what do you do to get that money.**

PS02: As number two, you already asked a question on debts, those debts are used in that. If I do not reach that money, it forces me to borrow money to add on that then I refund later.

PS05: As number five I try to save a hundred shillings daily, so that by Friday I have that five hundred shillings.

**I: The day that you do not get that money, you know things happen. That day you don’t get even a shilling.**

PS05: I borrow it from someone.

**I: You borrow from someone. Okay, the other people what do you do? Number three, you are thinking so much (laughs). What do you usually do? Like you usually keep two hundred, that day you find that you only have a hundred shillings, what do you do to top up that money?**

PS03: As number three I can just borrow.

**I: You borrow. So we all borrow to fill the gap or there is something else you usually do? Tell me guys. Someone else who has something they usually do apart from borrowing?**

PS01: As number one, if I keep borrowing, then I do not have a way to refund, if I go to work then I find a little money, because at times you may not find much money, and my kid still needs pampers. I will use the money I got that day then the next day if I get good money, I can top up so that I can replace what I used the previous day.

**I: Okay, number 7? Is there something you do to top up your money?**

PS07: As number seven, I can go to my friend talk to her/him to help me, then I refund later

**I: Which friend?**

PS07: Be it a woman or a man, any that can help

**I: Anyone that can help.**

PS07: Yes

PS04: As number four I said one hundred shillings that I know from today, tomorrow and the next day I can keep ten shillings so that by that day I have a hundred shillings.

**I: Number two**

PS02: As number two, I must get someone that can help me. I must sweet talk someone. If I do not have money to top up I must sweet talk someone and he add me.

**I: They give you without doing the other thing or how does it happen?**

PS02: Yes, there is someone you can just be free with, it is not a must you do that thing with them.

**I: Okay. I see that we have finished. You have responded to the questions well, thank you for that. The responses you have given us will be used to form jitegemee study, okay?**

R: Yes

**I: Thank you for giving us your time, thank you for talking freely. The questions may have been hard but they are questions for our daily lives okay?**

R: Yes

**I: thank you very much may God bless you. Is there a question or something you can add?**

PS04: We also want to say thank you, if you get time, you can come back another day to visit so that we can talk.

**I: Thank you. Number three, I see you have raised your hand.**

PS03: As number three I only have a request, even though we have learned, I request that you do not forget about us once you go back. Remember us

**I: Thank you. Number five?**

PS05: I only want to say thank you because today we have learned the things we did not know. How we can live in future, we also know that. Now am just requesting that once go, do not forget about us, you can come back

**I: Thank you, number one?**

PS01: As number one I want to say thank you for teaching us what we did not know, so if something comes like jitegemee that if one day this work comes to an end we can work. So do not give up on us, don’t let it fail.

**I: Thank you very much may God bless you. Thank you.**
