# Supplementary material for: Eat a little and save a little: A qualitative exploration of acceptability of a potential savings intervention to reduce HIV risk among female sex workers in Western Kenya
Source: PLoS One. 2024 Dec 19;19(12):e0310540. doi: 10.1371/journal.pone.0310540 (PMC11658496; doi:10.1371/journal.pone.0310540)
Supplement: S1 File — (ZIP) [file pone.0310540.s001.zip › Jitegemee Transcripts and Dissemination Notes for Journal/FGD T.docx]

**FGD ID: FGD T**

**INTERVIEWER: OLIVIA**

**NOTE TAKER: LILLIAN**

**VENUE OF THE INTERVIEW: NYAMONYE**

**INTERVIEW DATE: 05/MAY/2022**

**TRANSCRIBER NAME: OLIVIA**

**INTERVIEW LANGUAGE: DHOLUO**

**TIME TAKEN: 2HR 54MINS**

**CATEGORY: ABOVE 30 YEARS, RURAL.**

**I: Now we are beginning. This is FGD T being done in =Nyamonye=. The date today is 5/MAY/2022 AND THE TIME IS 11:37am. Now after briefly describing to you about Jitegemee, what would you like to say or what comes to your mind now about Jitegemee? What comes to your mind? (Cock crows) Number 9.**

PTO9: I’m grateful for Jitegemee that has made us to gather here. It has made me think… as number nine I have thought about saving a little and using a little so that I can find help in future.

**I: Mmh, that is number nine, someone else?**

PTO6: As number six, I have listened to the discussion and Jitegemee is teaching us that we should have something we are doing and after doing it we save some money to help in case you find a problem, you can fall back there. And when we don’t save, we will have to be sex workers. So, this is trying to show us…Jitegemee is trying to show us that when you have some business, you keep some and save some to help you when you have a problem in future.

**I: Mhm, that is number 6, someone else. What comes to your mind after explaining to you about Jitegemee?**

PTO5: In my opinion, I feel with this sex work that I’m doing, I should work hard and save, so that when I’m tired of sex work, I have some business I’m doing, sex work is not the end.

**I: Mmh, that is number 5, number 1?**

PTO1: I’m grateful for this information brought here about Jitegemee. This thing has opened our eyes. it is trying to show us that if you can get some money like the way we are doing sex work, you can save some. Things are not so good nowadays; you can have a child and you don’t have a standing or direction. But when you are saving something and your child passes exams, you can find where to begin from as you take him to school. You can buy the requirements without difficulty. That is my opinion on what Jitegemee is trying ti tell us

**I: That is number 1. Someone else with a different opinion?**

PTO7: As number 7, I feel Jitegemee is giving us a good idea that makes us realize HIV is there. On one side they are giving us an idea that even though we are doing sex work, HIV is there. That is why they have brought to us savings, so you can save and leave sex work before you get infected. That is my opinion.

**I: Mmmh. Number 8 is smiling like she has something to say.**

PTO8: Thank you, as number 8, I feel that even though I’m a sex worker, my life has to continue and the life of my children has to continue. I feel that if I get 5, I can save 5. I mean I can consume half and save half, so that when one day I feel I’m tired with sex work, I can find something to fall back to. That is my opinion as number 8.

**I: Mmh. Okay. Thank you. Number 4? do you have anything?**

PTO4: I’m grateful for the information I got today. As number 4, I feel that as a sex worker, the idea I got today is that even though I’m a sex worker, I should use the money I earn there to expand my business to grow. This business will help my children who are still young to continue with their education, as I do sex work, my children are also going to school because my business is also continuing.

**I: That is number 4, number 2?**

PTO2: Thank you. As number 2, the information I have got from Jikinge… Jitegemee is that as a sex worker, I should also just do business like selling omena, it is good to get a little from sex work but I should concentrate more on my business so that I’m able to save from both side and don’t find it hard when I’m tired of sex work.

**I: Mmh. Number 3?**

PTO3: I’m grateful to r Jitegemee program. I feel Jitegemee is opening our eyes for tomorrow’s life... where we work, a client can call you to a far place, then you don’t agree. They want to have sex with you and not pay you. If you did not have anything you had saved, or somewhere you can run to, you can find it difficult. you may have used a room and he does not want to pay, you ate and he did not pay. He hides and leaves. you know if you have some money you will save yourself from trouble and shame. secondly, I’m grateful for opening my eyes now we can save. Even though every child has a father, nobody would agree to accept you with them. I can buy land somewhere to give my mother easy time one time when I die. My children will have somewhere they can live. Yes, just that, thank you for opening my eyes.

**I: Mmh. that is number 3, thank you very much, as we continue, there is how we use money as sex workers. Isn’t it?**

ALL: Mmm.

**I: First I would like…I know there is daily expenditure, and others after some time. I would like you to tell me how you spend the money you earn daily? Daily expenses.**

PTO2: As number 2 this is how I want to respond to that question. It depends on how much you earn in a day and what your budget in the house, how much you spend in the house. You can find people with two children, some people have one child, so you can sometimes say you will spend 150/= or 100/=. So, you can always know the amount you can use in a week. As you work, you will know what to save after you have earned a certain amount of money. You can’t have a high budget and you don’t know how life will be tomorrow.

**I: Okay. The budget that…. like you, what do you include in your daily budget and how much do they cost?**

PTO2: Like breakfast I use 150/=, because I have two children. I use 150/= for what they will use for breakfast and lunch. I use around 100/= for supper.

**I: Mmh.**

PTO2: Yes.

**I: Okay. That is food?**

PTO2: Mmh.

**I: Is there anything you buy daily apart from food?**

PTO2: Just impulse buying, when I see something being sold that I like but I don’t use more than 300/=.

**I: Okay.**

PTO2: Mmm.

**I: That is number 2. someone else? Number 9.**

PTO9: As number 9 I can say that when I’m going for sex work; I’m going to look for money an I’m open I’m going for sex work. I can get three clients in a day who give me 1000/=, 500/= and 500/= respectively. Now I have 2000/=, when I come back from sex work, I make a budget there. What I must buy daily is a pant because you can get the client I met yesterday; he should find me with a new pant to show him the money he gave me was used.

**I: Mmmh.**

PTO9: Yes, that is how iam 9.

**I: Mmh. Money...you have not given me the full list. One of the things you buy daily is a pant.**

PTO9: I must buy a pant every day when I come from work.

**I: Mmh, any other?**

PTO9: My expenses in the house, when I get good money, like 2000/=, if I had cooking oil I can buy3 packets of maize flour, that is enough for one week because I have two children. It can’t go for more than one week. When I buy breakfast, l buys a big bread, sugar 2kg, that is enough for two days, When I get money, I can buy stuff enough for a whole week. So that if I go to work the following day, I will the save the money I earned that day.

Maybe just buy food if there was no food in the house because things like maize flour, cooking oil and tomatoes I had already bought...

I: **Mmmh**.

PTO9: Yes.

**I: Mmh. how much do you use daily for breakfast? How much do you use for lunch?**

PTO9: Let me say when I come from work, I go to the shop, I buy bread, milk and sugar, and that is around 300/=. When I use 300/= for breakfast it will be enough for two days, so I don’t buy breakfast the following day. Supper will now vary; I can buy beef or fish though fish is expensive but I can’t use 3000/=.

I: **For supper?**

PTO9: Yes. so, in a day I can spend 600/=.

I: **Okay, that is number 9, number 5?**

PTO5: Thank you. As number 5, I work in a bar. I have different clients at the bar. We live 3 people in my house. I have somebody paying my rent, and somebody buying food for use in my house. So, most of the money I get from clients who are not regular, I always don’t like using. I save that money because my child is in secondary school, it helps me in paying school fees and make ups. I prefer buying make ups after I have paid school fees, I buy my make ups and nice things with the money because I know there is someone paying rent, and buying food I don’t have a problem buying food for the day.

**I: Mmh.**

PTO5: Yes, that is how I am.

**I: Okay. do you buy food daily or once in a while?**

PTO5: So long as I have made a call to my client, I will have food. I pay rent every 10^th^ day of the month, “Hallo baby, are you paying rent or not? I get rent there and then.

**I: Okay. He does not send weekly or…**

PTO5: The person who buys food just buys food every time I tell him. After buying breakfast and I still need supper I just call and he tells me just come for it.

**I: Okay.**

PTO5: I just bring and eat.

**I: Okay.**

PTO5: Yes. I don’t buy food myself.

**I: Mmmh.**

PTO5: Yes, it is brought.

**I: How much do you spend on make ups?**

PTO5: It is good money.

**I: Mmh.**

PTO5: Mmm, I have many clients, let me not lie. After I get my thousands, Isave some, take 700/= out of it tnad go to a beauty shop and select some and keep in my bag.i will just have them in the house.

**I: Mmh.**

PTO5: My work goes on.

**I: Okay.**

PTO5: Mmm.

**I: That is not something you do daily?**

PTO5: I don’t do it daily because I’m employed. I can’t do it during working hour so I time.

**I: Okay.**

PTO5: Yes.

**I: Yes, someone else? how do you use the money you earn daily? Number 8.**

PTO8: Thank you, as number 8, I use a lot of money.

**I: Mmm.**

PTO8: because I like eating sweet things.

**I: Mhhm.**

PTO8: I don’t take vegetables or sardine(wui). I pretend that when I eat kales, I have some heart burn but the truth is that I don’t like vegetables. I always buy fish worth 300/=.

**I: Mmh. Daily?**

PTO8: Yes, daily.

**I: Mmh.**

PTO8: I buy 500ml milk, bread and blue band in the morning. At night I can take good tea, because I live with three children and their life must go on well like those who are married.

**I: Mmh.**

PTO8: They also have to go to school. so, sex work can give me 1000/=, I save 500/= and use 500/= for ensuring we eat well with my children.

**I: Mmh.**

PTO8: I don’t use second hand clothes, I want a good cloth. I don’t like a skirt or a short. I like an expensive trouser.

**I: Mmh.**

PTO8: Yes, when you want to be a sex worker, you have to be neat. That is when someone will respect you. When you have sex with someone, you are clean and your hair is well done, when he tries to pay something like 300/= he thinks twice because your class is above 300/=.

**I: Mmh.**

PTO8: You are now neat, I’m trying to say that you should not dress carelessly. You should respect sex work, and ensure you are also progressing in life.

**I: Mmh. Okay. You have said you love sweet things; you don’t take vegetables and you don’t use second hand clothes. How much do you use in buying food daily?**

PTO8: buying food like lunch…let me start with breakfast. I buy 500ml milk’, it was 50/= but is now 70/=, I buy bread worth 60/=, and blue band worth 50/=.

**I: Mmh.**

PTO8: We take tea and during lunch time I can buy fresh fish, I like it boiled so I prepare it and cook. I’m saying so because at night my children will…I’m normally not around at night, so I can buy food and send to them.

**I: Mmm.**

PTO8: Yes. I always prefer making tea at night or they can make tea while I’m still doing sex work.

**I: Mmm.**

PTO8: Mmm.

**I: Okay. How much do you use on clothes? do you buy daily?**

PTO8: I buy weekly.

**I: Oooh, weekly. Okay.**

PTO8: Yes.

**I: Okay, how much do you use weekly on clothes?**

PTO8: On one cloth, I can buy a trouser worth 1000/=.

**I: Weekly?**

PTO8: Weekly.

**I: Mmh, number one, how do you use the money you earn daily?**

PTO1: I am grateful. My friends sex work is not easy. When you go for sex work, first of all you have to ready for anything and when you come from there you must shop well. If you find. because in a day I can get even four people.

**I: Mmm.**

PTO1: But sometimes I want to serve all my clients and when you come from there you know that you have worked and you must do shopping because you have really worked.

**I: Mmm.**

PTO1: In my house, everybody who knows he is my sexual partner, must ensure I have everything I need. Yes, even if…it must be there. When I come back, I must buy for my children something of value. You can’t just arrive at the hotspot and leave after a short while.

**I: Mmm.**

PTO1: you must converse a little bit. You are not a hen that just… you must converse and laugh a little as you prepare to have sex. Yes?

**I: Mmm.**

PTO1: (Laughs)That is why sex work is not an easy thing. In sex work… on clothes, I choose and put on only the clothes I like.

**I: Mmm.**

PTO1: Yes. I like ready-made clothes, not second hand clothes. And I must take good food. When I am from sex work, I go directly to the butcher first.

**I: Mmm.**

PTO1: And I take tomatoes, cooking oil, loaf of bread, milk and 1 kg margarine. You know it?

**I: Mmm.**

PTO1: Yes. Now when you have got some money you buy enough food that you can leave your family with. You must also eat well when you are going for sex work. When it is tea make it good tea with enough milk and bread with enough margarine.

**I: Mmm.**

PTO1: You also go for sex work when you are smart and putting on a nice-smelling perfume.

**I: Mmm.**

PTO1: So that when he sees you, you immediately get his attention.

**I: Mmm.**

PTO1: You are attracted to him and he feels he is going to have sex with you and you have not even accepted

**I: Mmm.**

PTO1: So as for me, I am a sex worker and all my things must go on well.

**I: Mmm.**

PTO1: So, in one day I can spend about 700/=.

**I: Mmm.**

PTO1: Mmm.

**I: Now this 700/= is for your clothes and food or it is for food only?**

PTO1: No, clothes are exclusive. if I include clothes in the 700/=, how much would that cost, 200/=? I don’t use clothes worth 200/=. So that is just for food, expenses in the house. I buy things that can take me for two days.

**I: You said you just point clothes?**

PTO1: I point clothes.

**I: What does that mean?**

PTO1: The cloth I need… when I show you this is the trouser, I need, though I normally wear skirts. This new skirt or top, the way it is matching, that is what I want to put on tomorrow as we go.

**I: Mmh.**

PTO1: The cloth must be bought even if it is a suit. Sex worker knows. My clients know this very well.

**I: Okay. So, you point it to someone?**

PTO1: It is bought. I don’t point it and leave it, i point it, and he is the one who will buy it after having sex with him.

**I: Ooh.**

PTO1: He is the one buying it, he will be my client the following day.

**I: Is the person buying your client?**

PTO1: Yes.

**I: Okay.**

PTO1: Yes.

**I: That is number1, number 7. Mmh, number 3? How do you spend the money you earn daily? the money you earn, how do you spend it daily?**

PTO3: We can’t say how we use money daily because sometime I get 2 or 3 clients.

**I: Mmm.**

PTO3: Maybe you get someone with good money, it normally depends. There are those who pay 500/=, some pay 1000/=, there are also those who when they call you know your rent is sorted. You can come back with some shopping, if he is someone who cares about your dressing….

**I: Mmm.**

PTO3: Sometimes there is someone who has come, maybe they have come to a funeral and among them are people who know us in the bar. You will hear them say, “look for me someone to relax with”. There are those who just want to relax.

**I: Mmm.**

PTO3: Yes. We have visitors who want to relax, we have regular clients who have maintained you but they have not married you, they have their families. They just want to satisfy their sexual urge with you.

**I: Mmm.**

PTO3: So, you can’t say how much you get in a day, but as sex workers, we can’t get less than 2000/= if you know what you want. Somebody like me, I have 3 children, they are living with my mother, I have employed for her a house help who I pay 4000/= monthly. My rent is 2500/=, before including the cost of buying food. My children are going to school and I must pay for them.

**I: Mmm.**

PTO3: I must buy for them uniform, they need shoes, they should have 3 pairs of uniform, and 3 pairs of socks each and this is all money. I must buy for them clothes. You may have a regular client who you feel free with and you can just tell them “I’m a sex worker, I have this number of children and this is how I’m’. So, it is good to be open with this person so that he is free. Tell me you go home and find your children putting on rugs and your regular partner you are free with normally maintains you, “my friend I’m not coming but let me send you some 3000/= to help you buy food.

**I: Mmm.**

PTO3: You have to take good care of your children. so, somebody like me uses ten thousand shillings in a week.

**I: Mmh.**

PTO3: I buy maize, food prices have gone up, I need to pay the motorbike that take my children to school, feed the house help, I must also support my mother. What helps me is that I have some business where I earn something small daily.

**I: Mmh.**

PTO3: Yes, sometimes you don’t have…there are days you can be tired and decide “let me just rest in my kiosk here I will not go to work”. So, I just time, at 5pm I leave for work. I work in a bar and we close by 11pm. during that time you can get a client who can pay you 500/=. Where I stay, I use about 700/= because I buy milk, sugar, things like rice I must have. There is a time I can decide to go and visit my children who have missed their mother. You can’t just arrive and leave, you must be with them for 2 or 3 days to see if the house help treats them, how they get along with your brothers. The child will tell you uncle so and so loves me, and so and so don’t love me. You know the child’s mind and they don’t miss you. So, when I’m away, I must ensure I buy food that I will leave for them, shopping to take home. I can’t just go home like someone going to the toilet.

**I: Mmh.**

PTO3: Because when we go to our hotspots, we don’t just go anyhow. We must have sanitary towels, , you buy condoms, carry some toilet paper, an extra pant, pant liners , and wipes , Because sometimes you get a client immediately you are through with another one and you can’t refuse and you need money and you know they are the people who have money around.

**I: Mmmh.**

PTO3: So, you must have wipes, you leave as if you are going for a short call then you wipe yourself well so that you don’t have bad smell during sex. So, you must buy pads and the rest of the things.

**I: Okay.**

PTO3: We must buy wipes; we must buy make-ups to use.

**I: Mmm.**

PTO3: Yes.

**I: So, you have said you use 10,000/= weekly?**

PTO3: in a week I use 10,000 for expenses in my house and at home.

**I: does that include wipes, pants, or…**

PTO3: Yes.

**I: Okay. Mmmh, and you use 700/= in a day**

PTO3: I use700/= daily.

**I: Okay. What of monthly?**

PTO3: In a month, I use 40,000/= and sometimes it is more because I have someone who helps me in my kiosk and I pay her. she also washes the house and clothes for me and I pay her daily.

**I: Does 10,000/=include school fees?**

PTO3: 10,000/= includes school fees also.

**I: Okay, thank you. That is number3. anybody else? how do we spend the money we earn? she has told us how she uses it daily, weekly and monthly. Do other people…you had told me about daily expenditure, isn’t it**?

ALL: Yes.

**I: Can you tell me how you use it weekly or monthly? Number nine is looking for me like she has something.**

PTO9: As number 9, I use 3000/= weekly not because I can manage to get 3000/=, but because I’m saving. So, when I get money, I don’t want my expenditure to be more than 3000/=. I’m saying so because my child is in PP2. Yes, I use 3000/= meaning I use more than 5000/=.

**I: Mmm.**

PTO9: So, I pay rent, I can give my mother support. Because I can’t support my mother daily, I can pay her a surprise visit, and give her 500/= or 200/=. So, in month I can use 5000/= .

**I: Okay. someone else? number…I can’t see some numbers, number 5, are you present?**

PTO5: I’m present.

**I: Yes number 5, tell me. You had told me daily expenditure, what of weekly or monthly?**

PTO5: Weekly, monthly I use money. I must use money because I pay school fees. The main reason I do sex work is to pay school fees. I did not have a problem feeding myself just went so that I could pay school fees. So, every end month, because he is in day school, I give him money as he goes to school. When they want other monies in between, we pay a lot of money it can be 3000/= in a month, can be 800/= in a week, because he is a day scholar, sometimes they want 400/=, I give him 250/= to go back, they also need money for remedial. So, the money I get from sex work helps me in paying school fees.

**I: Mmm.**

PTO5: Mmm.

**I: Okay.**

PTO5: I mostly use it on school fees.

**I: Mmm.**

PTO5: Mmm.

**I: Do you pay school fees monthly?**

PTO5: let me say just for the one who is still in primary school. The one in secondary school I save and pay per term or sometimes I’m able to pay all the fees at the middle of the term.

**I: Okay.**

PTO5: Yes.

**I: What can we be buying with the money we earn and we have not mentioned.**

PTO5: You can buy animals and keep somewhere. This include cows, goats or poultry.

**I: Mmm.**

PTO5: Yes, we don’t have homes. we just move around and stay in rental houses.

**I: Mmm.**

PTO5. Yes, for example somebody like me stays in a rental house. That is the only house you have. If you don’t plan, where will you go when you leave there? You have to save little by little; you may buy land somewhere and live there.

**I: Mmm. Okay. that is number 5. Anybody else with something else to add?**

PTO4: As number four, I have children who are going to school, so I have saved some money that I get from sex work, I have a plan of buying land to build there.

**I: Mmm.**

PTO4: Because I have children, I have someone paying his secondary school fees.

**I: Mmm.**

PTO4: The other one gives me fish but I don’t eat fish so because I handle fish.

**I: Mmm.**

PTO4: So, when he gives me fish, I sell and buy beef.

**I: Mmm.**

PTO4: Or I can buy chicken and eat with my children.

**I: Mmh.**

PTO4: The other child is in an academy, there is someone else who pays his fees direct to the school. Most of my money I’m planning of buying where I can live. So even when I’m doing sex work, I have a plot where my children will live on. When I shall be tired will rest.

**I: Okay.**

PTO4: Mmm.

**I: Is it something you have begun planning or it is still in your minds?**

PTO4: I have that in my mind.

**I: You have not started?**

PTO4: Mmm.

**I: Okay. Now we have talked about how we spend the money we earn. Number 6, do you have something you would like to add?**

PTO6: There isn’t.

**I: There isn’t?**

PTO6: Mmm.

**I: Number 7?**

PTO7: When I get this money, you can buy something in the house like a chair. You will one day remember that I bought this chair when I was like this and now, I’m like this. You can buy somethings in the house.

**I: Mmm.**

PTO7: Yes.

**I: You buy after how long?**

PTO7: I can work hard for three months them buy a chair, the work for another two months and buy a table

**I: Okay.**

PTO7: Yes.

**I: Number 1, have you said?**

PTO1: Yes.

**I: Okay. Where do we get the money we spend? Where do sex workers get money that they spend? Some of us said there is something you can do before they go for se work, isn’t it?**

ALL: Mmm.

**I: Yes, now these are the things we should say what they are money that we spend and help us a lot, where do we get them from? the money we top up with, where do we get it from? Number 9.**

PTO9: Before I go for sex work, I work in a salon.

**I: Mmm.**

PTO9: I go to the salon early in the morning, I work up to 5pm and go back to the house. I use the money I earned from the salon to buy foodstuff that I leave in the house before I go. Because it is normal to look for money which is never enough so it forces me to…sometimes at the salon, I can stay for a whole day without getting a customer. or I plaited for r 150/=, or a blow-dry for two people, so you have a total of 300/=. So, you are forced to go for sex work where you can get 2000/=. But I just maintain my salon though it is not sufficient. Because I pay rent where I do salon work.

**I: Mmm.**

PTO9: So, I have to supplement with sex work so that it can be enough.

**I: Mmm.**

PTO9: But I prefer my salon because sex work is exhausting. After having sex with three or four people, you are exhausted that you don’t even long to go back. So, you are forced to…you sleep and when it is time, you go to the salon, where you are the overall and nobody will ask you the time you went to work. We have to work there because it has to do well, that is where our hope is.

**I: Mmh**.

PTO9: We go for sex work but we know it is something temporal, you can quit at any time, but salon you can’t quit easily because you have the skill of doing it.

**I: Mmh.**

PTO9: Mmmh.

**I: Mmh, that is number 9, someone else? Number 6?**

PTO6: As number 6, how I survive before I go for sex work, first I wash clothes for people.

**I: Mmm.**

PTO6: Anybody with clothes that should be washed bring to my house for washing. After washing and there is how a charge per basin. when it is a big basin, I charge 500/=, it depends and a small basin is300/=. So, my other source of income is laundry work.

**I: Mmm.**

PTO6: I wash clothes before I go for sex work because sometimes you can go to the hotspot and be surprised.

**I: Mmm.**

PTO6: You can decide to just sit comforting yourself that you will go to the hotspot at 5pm and get good money. You can go to the hotspot and finds things are different from what you thought. So, I do laundry work. Second, I also survive with hair dressing. I do hair dressing just at my door, I have not set aside somewhere I do it

**I: Mmm.**

PTO6: I work in my house. When I find someone who wants hair dressing, I just do it in the house, if I don’t find anybody, the day just ends like that. Those are my other sources of income apart from sex work.

**I: Okay.**

PTO6: Yes.

**I: Do you wash for pay every day or how many times a week?**

PTO6: I can’t say I wash every day because it will depend on how people come. Sometimes someone comes today and another one tomorrow, sometimes a day passes without washing. So, I can’t say I can wash daily or not.

**I: Mmm.**

PTO6: the orders come at different times.

**I: And averagely how much can you earn from washing clothes in a week?**

PTO6: In a week? In a week it depends on how people come. I can earn around 2000/=.

**I: 2000/=?**

PTO6: Yes, in a week.

**I: Okay. What about hair dressing?**

PTO6: Hairdressing can earn me 2000/= because sometimes I do dreadlocks, it depends on how I charge. I also do pedicure.

**I: Mmm.**

PTO6: It depends, they earn more than washing clothes.

**I: Okay.**

PTO6: Yes.

**I: What about sex work, how much can it earn you in a week averagely?**

PTO6: Sometimes it is difficult, I can’t say it can be 2000/=, It depends on the client you have met.

**I: Mmm.**

PTO6: Sometimes you get a client who pays less. Not all clients pay good money.

**I: Okay.**

PTO6: Yes.

**I: That is number 6, number 8 was carrying her hand.**

PTO8: Thank you, as number 8, what I maintain first is my business. I have my own business that I can go anytime I want, but I must go from sex work during the day because I do roast maize and sell.

**I: Mmm.**

PTO8: You know maize is normally roasted in the evening when it is cold. I can leave at 9am for sex work at work up to 2pm, I shall have got some money, go back to the house to freshen up and go to my business.

**I: Mmm.**

PTO8: Yes, where I do business daily in the evening.

**I: Okay.**

PTO8: Yes.

**I: So, you do sex work during the day?**

PTO8: I do sex work during the day. It is good to do something during the day while you can see.

**I: You sell roasted maize in the evening?**

PTO8: Yes, I go in the evening.

**I: What profit do you get from roasting maize?**

PTO8: When I buy maize worth 1000/= at a cost of 10/= each, I sell each at 20/= I have a target of 2000/= in the evening after selling 100 cobs.

**I: You sell all of it?**

PTO8: I sell all of it.

**I: Okay. And when there are clients, when you go at 9am and work up to when you will be going to sell maize, how much do you normally earn from sex work?**

PTO8: Sex work is something you…You can get clients or sometimes you don’t get.

**I: Mmm.**

PTO8: Yes, there are some people who prefer day time and others don’t many people like darkness. Sometimes I can get two clients at the hotspot, and sometimes I can get one client who gives me 1000/=.

**I: Mmm.**

PTO8: Or you had luck and got a teacher who has earned his salary and gives you 2000/=.

**I: Mmm.**

PTO8: I like clients who have big bodies.

**I: Mmm.**

PTO8: Fat clients get tired so fast, they will not work on you a lot so you don’t get tired. You can’t manage have 3 slender clients.

**I: Mmm.**

PTO8: Yes. so, I prefer big bodied clients, they are not so thorough at sex so you find a lot of difficulty.

**I: Okay. That is number 8. Number 7 wanted to say something.**

PTO7: What I rely on mostly apart from sex work is business, I sell onions I first sell onions before I go for sex work. I can be at the market selling onions during the day then meet my client at night.

**I: Mmm.**

PTO7: Mostly I sell onions.

**I: How much do you earn from onions?**

PTO7: Daily or?

**I: Yes, daily.**

PTO7: When I buy a sack at 5,000/=, I can get 1000/= profit.

**I: You sell all of it in a day?**

PTO7: Yes.

**I: Okay. It seems number 6 and number 5 have something, can you tell us also? Or we just continue?**

PT: My neighbor has called me; she needs some key.

**I: We can give someone to take to her, give those ladies who are out there.**

PTO1: Let me also talk.

**I: Do you want to say how you get money, or it is about your child’s lunch?**

PTO1: I will have to go and open for him the door during lunch time, it is not far. I just stay round here.

**I: Okay, say where you get money you use from.**

PTO1: What I do mainly is selling charcoal.

**I: Mmm.**

PTO1: Charcoal does not sell well during the day; it is not so easy during the day. So, one may decide to use firewood during the day and buy charcoal at night.

**I: Mmm.**

PTO1: Before I go for sex work, I must first go for sex work then go to sell charcoal after coming back. Charcoal sells best at night.

**I: Mmm.**

PTO1: I’m free during the day, if you go to sell charcoal during the day, you will sit the whole day and wish you had another work to do. So, I’m free during the day.

**I: Mmm.**

PTO1: I’m free during the day and sell charcoal around 5 pm.

**I: Mmm.**

PTO1: So, I go for sex work during the day. I can even go and do it in Kisumu and still maintain my charcoal business. If I go to Kisumu, I will be back to sell charcoal by around 4:30pm.

**I: Mmm.**

PTO1: Yes.

**I: Okay.**

PTO1: Yes.

**I: How much profit do you get from charcoal business?**

PTO1: I buy some at 900/= which is a sack that is full, and others at 850/= which is a sack that is not so full. I can get a profit of 500/=.

**I: Mmh.**

PTO1: The one I buy at 900/=.

**I: Okay.**

PTO1: The one I buy at 850/= I can get 300/= or 250/=.

**I: What about sex work?**

PTO1: Sex work is not sometimes easy. You can go and after you have had sex with someone, he has many excuses which he did not tell you before you had sex. so may finish and there is no money, he may tell you he will send you later which he does not. You start wishing you went to your business you would be having something to carry to the house. You came from sex work empty handed. You are waiting for the money to be sent buy the phone is switched off. So, I have hope in my charcoal business.

**I: Mmm.**

PTO1: Yes.

**I: Okay, when we were talking and signing the consent forms, some people said money from sex work is more than other monies. Is that true or a lie?**

ALL: That is true.

**I: Mmh, the way I have heard you talk, sometimes you don’t get something from sex work. Let’s identify ourselves, don’t talk all of you at the same time. Yes, number 9, let number 9 begin.**

PTO9: As number 9, sex work is good. What I would like to tell my fellow sex workers, don’t do sex work before you agree on the charges, you may not get something. Sex work is about giving me as I give you and it all ends there. Take the money first and after taking it, give a fellow sex worker who has not got a client or keep in your brassier. He gives you and keep then you can now go. Sex work is good, somebody who does not know how to do it will say there is no money. When you go, every style has its charges, you charge and not say just give me 500/= all the time. Every style has its charges and that is why there is money in sex work someone who does not understand will say there is no money.

**I: That is number 9? Number 6 also wanted… Number 8 wanted to talk. No, it was not you, number5?**

PTO5: I disagree, we are not all the same, people are different but sex work is good. Especially me, working here at =Summary= here sex work is very good. You get in and you have noticed someone at the corner. You can see somebody who has money from the face.

**I: Mmm.**

PTO5: Yes, that is why I was telling you I don’t mostly buy food. My food is bought.

**I: Mmm.**

PTO5: Because sex work is promising, you check someone from where he is sitting and know he has good money. So, let us do sex work wisely so that it can continue.

**I: That is number 5, anybody who wants to add? Number 3?**

PTO3: Personally, I feel sex work has good money, because it is something that can bail you out at a time you did not expect. Maybe I rely on my kiosk business and my child has been sent away from school, they want money As I had said, I use 10,000/= in a week, I was not able to raise that money from the kiosk, You get fish to sell, the person helping you to sell steals from you. Sex work is good, there is how it saves us.

**I: Mmm.**

PTO3: Secondly, you have to also check class in sex work you can’t just accept every invitation, you have to show some reluctance first.

**I: Mmm.**

PTO3: How you dress will also play a role, if you just wear rugs, second hand clothes, somebody will not judge you well. They call you and tell you they have 500/= and currently, 500/= can’t do much. For me 500/= will just be for lunch and breakfast then I lack supper.

**I: Mmm.**

PTO3: sex work is helping us a lot.

**I: That is number 3. Number 1 wanted to say something.**

PTO1: Sex work is not bad. Sometimes you have a regular client that you don’t have to talk about charges first. There are new clients that you have to talk with about charges first. Regular clients can just send the money later but you can’t just leave empty handed even without money to buy soap for bathing. You should leave with some soap.

**I: That is number 1, number 8.**

PTO8: As number 8, as I talk about sex work, you should go there after using some good perfumed body lotion. You can get a client there who is a teacher who earns at the end of the month. he wants sex but he does not have money. You can agree with him and note somewhere that he did not pay, only gave transport. Let’s say he earns around 5^th^, you will just be hope full that he will send it when he gets money.

**I: Okay.**

PTO8: Sometimes you have had sex four times in a month, after you have agreed with him he will be paying 1500/= after having sex because he does not do it like he does with the wife, it can just be one round and that is enough You must have other clients also because even as he waits for month end to pay, I need to eat.

**I: Mmm.**

PTO8: He will be your regular client set aside, you just record somewhere and he pays at the end of the month. I can even go somewhere after dressing neatly, I buy a soda or other drinks where this friend of mine works, I can’t fail to get a client who will give some money.

**I: Mhm.**

PTO8: That is about me as a sex worker.

**I: That is number 8. You have told me what you normally buy. Can you tell me why you buy these things? Some people said make ups, some domestic animals, some food, clothes, people said several things. Isn’t it?**

ALL: Mmm.

**I: I there anything...Why do you do so? When you set aside money to buy something, what helps you decide?**

PTO1: So that one time when you retire, or you are tired, you are able to remember.

**I: Mhm, that is number 1, number 8?**

PTO8: As number 8, somebody can get sick any time. When I take money and my poultry, when im very sick or my child has malaria and I don’t have money at that time, I can take my poultry or goat, sell and get treatment for mu child and my child’s life continues. My work also continues, I do sex work, buy another gat to replace.

**I: Mhm, that is number 8. Number 9?**

PTO9: The reason we buy those things is, when you buy make-ups, you will look beautiful. clients will notice you. Second, when you buy an animal, you are not thinking of getting married the animal may even give birth to four others.

**I: Mmm.**

PTO9: May be you have saved some money, you have got half or quarter hectare you want to go and buy and may be the money you have saved is not enough, you will sell even two of the animals to and get enough money to buy land so that you child is able to build and you can also be able to buy materials and build. That is why we buy those things.

**I: Mmh. that is number 9, number 4?**

PTO4: As number 4 the reason I buy things is because I always want to prove to those who are married that even though I don’t have a husband, I can always buy things likechairs, domestic animals, my children can go to school. I buy to prove to them I can manage with sex work.

**I: Mmm.**

PTO4: Mmm.

**I: A woman is able?**

PTO4: A woman is able.

**I: Mmh, number 1, number 2. Mmh.**

PTO2: Personally, I can’t say I have bought a cow or a goat, the money I have now I save on my phone because my child is in class 8 so I have to plan for joining secondary, next year. I hope to have 70.000/= by December for joining form 1. sometimes you can buy cattle and you don’t have somewhere to keep it because you are renting. So, you can save money on your phone, when I get money, I use some and save some so that my lie and my child’s life can continue. When I feel he is doing well in school, I can now have a plan b, buy something that I can keep to help me acquire somewhere to stay.

**I: Mmh, number 3 had something.**

PTO3: We are buying these things so that we can be like others. You will find in schools where our children go, you find a child has something and when asked why they are never bought such things, they say it is because they don’t have a father. So, we must work hard, and let our children live a good life, they should have what they need. you try to have…you buy things in the house. You know we can be attacked any time, maybe you had someone’s husband and they found out, they come to attack you. You should make your house so that when she comes to attack, she finds it is not as she thought When I now tell them this is my house, you can’t scare me they tone down. And when they go back, they can say I can also have this and the other. We also try to open the eyes of those who are married Because when I buy a chair, they also buy. Second our clients can make them afraid, sometimes I have a daughter and they have come to visit me. My friend did not know they are coming because most of my clients come from out so somebody surprises you, they wat to come and see how you are living. They find your daughter and you have gone for sex work, there are people with bad intentions, they have had sex with you and they also want to have sex with your child If your child is well brought up, they will refuse. but when the child has no pocket money, they lack many things this man will convince her. So, they are having sex with you and with your daughter. So, we should save money for emergency So that when the child is sick, I can rely on myself. Even when my mother who stays with my children has a problem or dies, I have an idea where I can stay or where my children can live. That is why we buy these things, when there is a problem, you will be asked, “what do you have?”

**I: Mmm.**

PTO3: And sometime you have not got clients for a week, if you call them they say they are still far and because we don’t want clients who charge 300/= or 500/=, we feel that is little money so sometimes you call someone and tell them I want to see you, you are looking for clients because sometime it is difficult. You try to look for someone and they are not around how can you find help? If you have an animal you can sell to solve your problems, you can slaughter it in your mother’s, grandmother’s, uncles, father’s or child’s funeral. That is why we buy those things, for emergency, kata risk. Sometimes I go to the club, there is a fight and I’m hurt, I will leave my children without anything, there is only the plastic trolley, plastic stools and a table, then people will ask, she used to really dress well, what does she has? You have left your children behind. Things like that make us want to fix things. Maybe I was hurt and I died, or I survived and came back, how would I feed While you are still sick

**I: Mmm.**

PTO3: When I come back here in =Imbo= that I was hurt in =Busia=, I have come back to the house and you know clients from =Imbo= pay 500/=. You need to buy medicine worth 3000/= and you can’t force somebody to pay for you, because you were hurt in a way that is not acceptable isn’t it?

**I: Mmm.**

PTO3: So, I can sell something to get money for treatment.

**I: Mmh.**

PTO3: Yes, this is why we buy these things.

**I: We said there is money we spend on ourselves. isn’t it?**

ALL: Yes.

**I: Some money we spend other people like… the list had your mother or children, isn’t it?**

ALL: Mmm.

**I: Who else do we spend our money on?**

PTO: I can help my grandmother.

**I: Grandmother.**

PT: Brother.

**I: Why… you had told me but you can just add Why do you send for your mother or grandmother money or what you buy for your children. You had said why you buy for your children is so that they can be like other children. Is there any remaining that you would like to add? so that we can continue.**

PTO1: You have to send your mother money because when you are doing sex work, she is the one taking care of the children.

**I: Mhm.**

PTO1: so, you must send your mother money, she has your children.

**I: That is number 1, number 4**

PTO4: As number 4, the reason I must send money to my mother, at the village when there are girls who were married, she should not feel that if my daughter was married, this could have been done to me. So, I can do sex work very well to help her.

**I: Mhm, and others? Do, sex workers save?**

PTO3: Mmmh. We save and have accounts.

**I: That is number 3 she has said you save. Number 9 also accepts you save. What do you do with the money you save?**

PTO3: As number 3, I save money because my children are still in primary school.

**I: So, you save for school fees?**

PTO3: I save for school fees.

**I: Mmm.**

PTO3: I save to buy land. Both me and my mother don’t live well. So I desire to buy land and relocate my mother and her grandchildren that is why I save.

**I: Okay.**

PTO3: Yes.

**I: Someone else? Number 5.**

PTO5: Why I save as number 5…

**I: Before number 5 talks, number 3 has told us why she saves. How frequently do you save?**

PTO3: I ensure every time I come from sex work; I have money to save.

**I: Daily?**

PTO3: Yes, daily.

**I: Daily, so how much do you save in total?**

PTO3: Can be 1400/=, I save 200/= daily.

**I: That is number 3. Number 5?**

PTO5: Mmh, the reason I began saving is because I had hard time when I took my child to form one. I feel I should open an account for the one who is still in class seven, and the bank promised I can take loan after 3 months. I don’t need the loan because it will go straight to school. I want to save so that I don’t have hard time like the one I had.

**I: Mmmh.**

PTO5: Yes, I found it difficult and that opened my eyes that I should save something small from sex work.

**I: Mmmh.**

PTO5: so that when my child reaches form one, I will manage that opened my eyes, others sources will also be helpful but that account will help me in future.

**I: How frequently do you save?**

PTO5: I just send the money from my house, at least for… I send 3000/= or 4000/= by every month end.

**I: Every month?**

PTO5: Yes, when I send to the phone, at night when I’m quiet, it is just 200/=, it is mine

**I: Mmm.**

PTO5: Yes.

**I: Mhm, that is number 5. Number 7?**

PTO7: I’m saving because I have children in school, I have somebody in secondary school and in a private school also. So, when I save, I find something to take to school.

**I: Mmm. you save after how long?**

PTO7: three months then I withdraw and take to school.

**I: Oooh, do you save daily?**

PTO7: Yes, daily.

**I: how much do you save?**

PTO7: 300/=.

**I: 300/=. how much in a week in total?**

PTO7: 2100/= in a week.

**I: Mmh, that is number 7. Number 9, you have nodded your head you save, what do you do with the money you save?**

PTO9: As number 9, I save to avoid embarrassment in future when I have a problem, I’m also saving for the future of my children. By the time they reach class 8, it will not be class 8 because it is now CBC, when they reach class 6, they do, 7 and 8 then proceed to junior school. I should have money when they are joining junior secondary. Secondly, I want to save so that I can buy for my mother land to build a house in future. My mother stays with her grandmother in their home. So, God helping me, I look for money, buy land so that my mother can build so that my brothers can also build. So, I always don’t spend money that I earn from the salon, so if it is 1000/=, I save the whole of it, if it is 200/= I save 200/=. so sometimes I have 4000/= in the account, so I ensure it is not less than 3000/= in a month.

**I: Mmh. Do you save it daily or once?**

PTO9: I save it daily because I work at the salon daily. So when I get something small, , like 200/= , 100/=, 1000/= I send to the account.

**I: someone else? number 8 do you have something? why are you not seeing clearly?**

PTO8: I’m hungry. as number 8, I can save my mother has land, but her house is not good so we can team up with my brother, save something in the account to buy iron sheets, because we want our mother to also have a good life like other women.

**I: Mmm.**

PTO8: so, I can save 250/= in a day so that at the end of the week, I have 1750/=.

**I: Mmh, that is number 8, is there anybody who want to add? What are the characteristics of those who save mar jo ma save? There are those who save and those who don’t save. isn’t it?**

ALL: Mmm.

**I: How do you know the characteristics of those who save, number 1 looks like someone who save. or number 2 looks like someone who saves. Have you understood the question**?

ALL: Mmm.

**I: Mhm, give me answers, number 8 how does someone who save look like?**

PTO8: Thank you. As number 8, my friend number 5 here I can know she is saving because I’m is seeing some change in her life after one week, she can save money for a week and you will see some change that requires some good amount of money.

**I: Mmm.**

PTO8: She had been saving bit by bit, she may tell the amount she saves but from the look of things after one month, you see how life is doing, you conclude… may be let me say like I looked like someone struggling but after one month there is some change. That means I was saving.

**I: Mmm.**

PTO8: Yes.

**I: Which change it this that you see in somebody and know she is saving?**

PTO8: Change that I can think see is like when they normally buy fish worth 200/= but can now afford chicken worth 800/= from the market to go and eat with her children.

**I: Mmm.**

PTO8: That is change I see.

**I: Mmh. that is number 8. Number 9?**

PTO9: As number 9, change that can be seen on someone who saves, like my friend number 66 here, is saving, you know when someone has money, you will notice. even when they don’t tell you. Like you can say, we are in the same hotspot, why is she not progressing? After saving and withdrawing her saving, she will buy thing like glass table, TV stand, iron sheets to build her mother a house from =Bondo=. She will show them off for someone to see by leaving them at the bus stop. This a sign that someone is doing something.

**I: Mmh, that is number 9, number 3, what are the characteristics of someone who saves?**

PTO3: Characteristic of those who save, you will see from their actions. there are people who like denying themselves, there are people you will just here that they had done something, they will not tell you. So that is the characteristic of those who save. They don’t show off, they are very simple but they are working.

**I: Mmm.**

PTO3: She has plans with what to do with her money and she does her things as she has planned. there are also people who are stingy, they have money but they can’t dress or eat well, people will know she has money. They want to save and do something with it. There are people like. that.

**I: Okay.**

PTO3: Yes.

**I: Number 6, what characteristic do people who save have?**

PTO6: When I want to know number 9 saves, you will find that she is a sex worker, she does not do any business but does sex work.

**I: Mmm.**

PTO6: So if you want to know someone saves, you will find they have a kiosk, which they didn’t have previosly, that will open my eyes that she had some plan ahead as she was going to do sex work, the kiosk has good stock, their lives are different from how they were, that will show you your friend was saving.

**I: Mmh, what about people who don’t save? what characteristics do they have? those who don’t save. Number 4.**

PTO4: Thank you, as number, people who don’t save have no change. How you see them this Monday is the way you will see them the following Monday.

**I: Mmm.**

PTO4: and they want to have everything they see somebody with. they are not contented; they don’t want to save. They want to spend all they earn.

**I: Mmh, that is number 4, number 2?**

PTO2: As I answer that question, someone who does not save when you are with them and they see something being sold, and you buy, you will hear the say I will also buy something like that. The seller passes again and you buy but they are wish to have but not buy anything

**I: Mmh, that is number 2, someone else? How does someone who does not save look like number 9.**

PTO9: Thank you, someone who does not save is jealous. When I’m saving, I will buy something, you will hear them say “this one has taken money from people’s husbands and has bought a table. You see her passing” that is not someone saving, even when you buy a flask that you can do casual work in a farm for one day you will buy.” See, whose husband was she with today, she is carrying a flask, today she was bought for a flask”. They are jealous, they are not happy when someone is doing well.

**I: Mmm.**

PTO9: Mmm.

**I: Mmh, number 8?**

PTO8: As number 8, someone who does not save does not think about tomorrow they, are like fishermen. When fishermen get 5000/= they will spend all of it reasoning they will get some money tomorrow. When such a person gets money, they drink all of it with friends and spend it on leisure, when they get it tomorrow, they say that job is not helping them but they are not able to save.

**I: Mmm.**

PTO8: Yes.

**I: Mmh, that is number 8, someone else? What makes saving easy for sex workers. Number 5.**

PTO5: As number umber 5, saving become easy because it will help you. You are saving it because you one time you will remember what you were doing. So, it becomes easy when you find it, you don’t spend it because you know the pain you go through. Yes, we go through a lot.

**I: Mmm.**

PTO5: We go through bad experiences so that you get money from someone to use. You talk and change languages for you to get the money one day you will remember, that around this time, this is what I used to do. I have to massage someone’s husband to get 1000/=.

**I: Mmm.**

PTO5: You call him by names the wife has never called him with so that you get that money. You will save that money because you know how you got it through difficulty, you worked hard.

**I: Mmm.**

PTO5: Yes.

**I: Mmh, someone else, what makes saving money easy from sex workers. Number 1, number one is still thinking, number 3**

PTO3: I’m saving money so that one time I can have land, or be someone that can be respected, at the moment I have some appearance and I can talk where people are, but it will reach a time that I will not be able to do sex work or my age does not allow me . I want to go and scramble for clients with my daughter or be found where my sons meet their sexual partners. So, this time as I do sex work it is good to save so that I have good life tomorrow. I’m used to thEelifestyle of taking tea with bread, if I think of going back to taking tea with ugali that was cooked the previous day, I find that difficult. So, I feel I should save to enable me build a house and buy a vehicle to pack at my door the house.

**I: Mmm.**

PTO3: Yes, even these clients I have, sometimes they want to show you pictures of their houses, families, like this is my first born, this is my wife, we want this or the other. So, I also long to have those things but I don’t have a man who can provide then for me.

**I: Mmm.**

PTO3: So that makes me find it easy to save so that I can have what I see other people have.

**I: Mmh, that is number 3. why is saving money easy for sex workers. Number 8. No, let number 7 talk first**.

PTO7: The reason I save is because the way I’m today will not be the way I will be tomorrow. I may get sick tomorrow. My life should not come to an end because I’m sick and I don’t have anything to fall back to. At least I should have something to be able to eat, my children can bath and go to school even when I did not go for sex work. At least I have something, that is why I do save.

**I: Mmh, why is saving easy for sex workers, number 8.**

PTO8: Thank you. As number 8, what saving money help me with is that sometimes I can rest when I’m tired. I don’t feel like having sex that day, I can rest when I don’t want my body to worked on. I will not go without food because I did not go for sex work, I had saved money. I can rest even for 3 days without going for sex work, because I have something I saved, I can use to buy food.

**I: Mmm. Okay. What are some challenges people who save have? or saving has no challenges? are there challenges in saving? Number 8.**

PTO8: Challenges I can face sometimes is when I save money, buy a cow and it gets sick suddenly, and maybe I kept it at a relative’s home, I will just be called that the cow died. When a cow dies, and when a cow dies, the money got from the meat sold, mostly get lost so I will just be lamenting I saved my money on a cow and I have lost it.

**I: Mmm, that is number 8. Challenges people who save are which ones?**

PTO1: As number 1, challenge that you face when saving is that you save 40,000/= with a target of buying land to build for your children and when you go to withdraw, it you find it is not there.

**I: Mmm.**

PTO1: You are left wondering what could have happened, you realize what you could have done after this has happened and can’t be rescued.

**I: Mmm.**

PTO1: Yes.

**I: You lose the money?**

PTO1: You lose all the money.

**I: Mmm. What can make money be lost?**

PTO1: What can make you lose money is when you have someone close to you who know your passwords and you also don’t think he can do something like that. So, everything has been withdrawn.

**I: Okay. how can this be addressed? like losing money because the person close to you knows you in and out?**

PTO1: You will just know you have a bad friend and know it on your own.

**I: Okay. Number 3. Which challenges are faced by people who save?**

PTO3: Challenges are, you can lose money

**I: Mmh.**

PTO3: Or I can buy something with the money then it is stolen.

**I: Mmm.**

PT03: Sometimes I have saved and bought land somewhere, only to realize that land had been sold and you are the sixth buyer. You don’t have where to go; your money is just lost like that. Sometimes you gave your child money to pay school fees, someone opens her bag and steals the money, that is lost money and there is nowhere you can get it. Those are some challenges we face.

**I: Mmh, someone else number 9?**

PTO9: Challenges we face when saving, maybe you have saved 20,000/=, there are motorbikes taken on loan, you take and give someone believing you will also pay with income from sex work and save income from the motorbike the person riding the motorbike refuses to pay any money and you are also not able to refund the loan. Sometimes life is difficult, you are also sick and is not able to look for money and pay They come for their motorbike and that is how I have lost 20,000/=. Those are the challenges we face.

**I: How can that be addressed? Challenge of that kind.**

PTO9: I should just ensure the motorbike is functional so that I can get money to repay the loan. I can take the rider who is not bringing money to the police and record somewhere that in case he does not bring money to pay back the loan, he will pay me back theme money I used to purchase the motorbike.

**I: Yes, anybody with something they would like to add? Advantages of...those who don’t save, why are they not saving? Or everybody saves?**

ALL: No.

**I: Mmh. Why are people not saving? Number 3?**

PTO3: Your fellow sex worker may have a client, as number 3, I can’t take number 4’s client when he comes before my client comes even when you don’t get money that day. As I had told you, I save 200/= daily, sometimes when I don’t go for sex work, I will not save. So there are many challenges, we have merry go round to pay, I came from sex work with 500/=, I want to pay merry go round300/=, I want to eat, I will not be able to pay rent. The money I have is just from hand to mouth.

I: Mmm.

PTO3: Yes, now I can save Because I did not get enough money.

**I: Number 1, do you want to say the same thing or you have something different?**

PTO1: No, I want to say the reason why someone may not save is because you have taken the position of being a father.

**I: Mmm.**

PTO1: You are the one who does everything and may be back at home you are the first born. You get many calls of needs to be met, so you have money that you get and is all spent So will decide to send you mother money and conclude that you will save money later Yes, people can’t go without food because you are saving.

**I: That is number 1. Number 4, do you have something different from what they said?**

PTO5: The reason some people don’t save?

**I: That is number 5?**

PTO5: Yes, the reason fellow sex workers don’t save, you know it is money got daily. So, some sex workers don’t save because they feel it is money, they get daily. So, they just use it reasoning that they will go and get more, so they don’t save. So, this attitude that you will go back and get more makes you use all the money you get. That makes my fellow sex workers not save, they spend everything because they know they will get it daily.

**I: Mmh, that is number 5. anybody who wants to add? Number 9?**

PTO9: The reason why some people don’t save is because they don’t have responsibility, they are all alone. So, the money they get, they use on buying clothes and buying food for themselves, they go and order chips, chicken and soda, eat all of it and go to sleep. The landlord will even lock for them the door because they don’t have money to pay rent. They use everything they have because they have no responsibility, they don’t have any plan for tomorrow. They even go without protection, then they get pregnant, they don’t have where to begin from because they can’t go for sex work. You must have some money so that when things are difficult… and you are about to give birth, you are forgetting you said you are alone, you don’t have any responsibility, you are about to get somebody, you don’t know where to begin from. Somebody who does not save is somebody who feel they don’t have any responsibility

**I: Mmh.**

PTO9: Yes.

**I: Are there others different from the ones mentioned or we can continue?**

ALL: Lets continue.

**I: We continue. What are some disadvantages for those who don’t save? When they don’t save, what bad experiences can they have? Number 8, mmh, number 1.**

PTO1: You don’t have where to run to when you have a problem.

**I: Mmh, number 8?**

PTO8: Sometimes sickness has stuck you, you have no means to reach =Nyamonye=.

**I: Number 9?**

PTO9: Disadvantages of not saving, sometimes you have been called that your mother is sick. You don’t have transport to your home and which is only 50/=, maybe you wanted to reach there urgently so that if it is a matter of death, she should die in your presence. You don’t even have 20/= you can bargain with to a motorbike rider. You are empty handed.

**I: Mmh, number 4?**

PTO4: Disadvantage of not saving is that your door will be locked. Like me I rent a house, when I don’t save money, the landlord can lock the door at any time, my children will be looking miserable at the door then people will start laughing at me” she does sex work, where does she take the money”? At this time, I’m looking miserable with my children at the door. Sometimes your child goes to school and they are sent away, when I’m saving I will pay, when I don’t save my child will look miserable at my door then people start wondering “she does sex work, when does her children go to school, they are just at her door” so it is good to save.

**I: Number 5?**

PTO5: In sex work, you can be called, number 5 can go to =Kisumu=, your child who is in boarding school can be sent from school for fee, if you don’t have anything, there is no way you will save the situation. You will have to call and give instruction on what should be done so that the child goes back to school. You will not be called names, or be laughed at and your work will still continue.

**I: Mmh, number 3, what are the disadvantages of not saving?**

PTO3: Disadvantages of not saving, maybe you have a patient, you mother is sick and you want to blood for transfusion, sometimes she should be operated on, there is only some little money contributed by my brothers, . You are with the patient in the hospital and you only 6000/= needed cannot be raised. That thing makes us get sick sometimes, maybe you go for sex work and find someone who wants to accomplish some traditional rituals. They have been instructed to go to a certain bar, that made a fellow sex worker die.

**I: Okay.**

PTO3: Need for money made someone accomplish traditional rituals with her, and we found out when it was late., this is where we get infected, because it will force me to have sex with someone who HIV positive but is not taking drugs so that I get money to go and save something with.

**I: What about advantages of not saving, advantage that someone has because they don’t save, which ones are they? When I don’t save, which advantages do I have?**

PTO3: There is no advantage I get at all.

PTO2: There is no advantage, you will just live like a dog. You will be chased away anywhere you go because you have no say.

PTO5: There is no advantage.

**I: Who has any advantage?**

ALL: None.

**I: All of you don’t have?**

ALL: Yes.

**I: The money we save as sex workers, where do we save them? Just say where you save so that we can move faster.**

PTO1: I save on my phone.

**I: Phone? Specify if M-Shwari or M-Pesa.**

PTO1: I don’t use M-Shwari, I use M-Pesa, how I save on M-Shwari what do I do in sex work, I get money and put it where I should

**I: Mmh, so you save on M-pesa?**

PTO1: Yes.

**I: Number 3?**

PTO3: Bank.

**I: Which bank?**

PTO3:= Cooperative=.

**I: Cooperative bank. number 4 where do you save?**

PTO4: I save in =Equity=.

**I: Equity. Number 2?**

PTO2: M-pesa.

**I: M-pesa**.

PTO8: M-pesa.

PTO7: M-pesa.

PTO6: M-shwari.

**I: I have not heard anybody mention…. You don’t have Chama this side?**

ALL: crosstalk.

**I: The recorder does not pick choruses, identify with your numbers like number 3. talk.**

PTO3: As number 3, I can’t join Chamas because I don’t want issues with married women. They will call sex worker’s names and sometimes you have saved more money from sex work and this may make me end up at the police because I will beat them up

**I: Okay.**

PTO3: Yes.

**I: You said bank, why do you prefer bank?**

I PTO3: A prefer bank because can take a loan when stuck. I can change the money to shares, then I get loan to do some of my things.

**I: Number 1 save in M-pesa. Why do you prefer M-pesa?**

PTO1: It makes work easier, sometimes it somewhere that would require transport like in =Bondo= , when I want something to be done faster, just go and withdraw and do what I wanted to do .

**I: Number 4 where did you say you save in?**

PTO4: I save in the bank.

I: **Bank, what is the advantage of saving in the bank?**

PTO4: My money is safe and I can get it any time I want.

I: **Mmh. number 2?**

PTO2: I save with M-pesa because a can get it any time, I want it.

**I: Number 5?**

PTO5: I save with the bank because I had a target for school fees. So, I don’t want…when I put it on the phone, I will use it anytime I want. Im sure with it there because it will go straiaght to the school.

**I: Mmh, number 8**?

PTO8: The reason I save in M-pesa, as number 8, maybe I get something that needs my urgent attention, and I can’t find money from a friend or boyfriend, because I have my phone all the time, I will just withdraw and use then I will return the money later.

**I: Mmh, number 7?**

PTO7: I also save in M-pesa because I’m a business lady.. Sometimes I want to go and buy thing, I just send money and all the items I sell are sent to me.

**I: Mmh, number 6?**

PTO6: The reason I save in M-shwari, when you want to begin saving with M-shwari, there are months for saving like 9 months, or 12 months. so, for the 12 months, you will just save them withdraw after12 months. When it is M-pesa, you may withdraw even 500/= and then keep withdrawing because you have access to the money. But M-shwari is locked and you will not be able to withdraw, that is why I use M-shwari.

I: **Mmm.**

PTO6: Mmm.

**I: Mmh, number 9?**

PTO9: I also like M-shwari because they lock savings for months.

**I: Mmm.**

PTO9: when I have a target to save for 6 months then the money, I need will be enough. I save for 6 months and withdraw when I have met my target. I can’t withdraw it now and I get it now, I will withdraw it and get it the following day.

**I: Okay.**

PTO9: Mmm.

**I: We had said that… some of us said we don’t use second hand clothes, what again did we say that we don’t do as sex workers?**

ALL: Vegetables.

**I: Sex workers don’t eat vegetables, you also said… and we also said…**

ALL: Sex worker does not take strong tea; they take tea with milk.

**I: So, what I want to ask is that can you have low income and you live a lifestyle beyond your income. Is it something that is possible?**

PTO3: It happens.

**I: Tell us more.**

PTO3: Like me now, my income is low but my lifestyle is high, I live a very unpredictable life.

**I: Mmm**.

PTO3: Sometimes my sponsor dies, I have 5 people I rely on; I have someone who when I call, they send something immediately. If they are not able, they will tell me to wait for a particular day. There are people I don’t have to have sex with to give me money.

**I: Mmm.**

PTO3: Yes, even when they have sex with me and they don’t pay, I know they will give me what I want. So sometime my lifestyle is high, I go do dance at =Vunduba= leaving =Summary=. Sometimes I want to go to =signal= that is for high class people who only speak English No food costs150/=, it is from 1000/= and the table is normally full. Yes, sometimes my lifestyle is high and my income is low.

**I: Why does that happen?**

PTO3: That depends on the class of people I walk with. I may have someone who drinks in =summary=, I can’t go to=Engineers=. At =Engineers= a room costs 2500/=, there is someone who wants a room costing 1000/=. There are lodgings with bedbags, there are lodgdings charged 300/= for one shot. There is somewhere for relaxing and for just finishing and leaving. There are things like that.

**I: Okay.**

PTO3: Yes.

**I: Can a sex worker have a high lifestyle and their income is low? There is no money but you can’t use second hand clothes, we have to get new clothes.**

PTO3: That is something that is there.

**I: Mmh, number 9.**

PTO9: The reason your lifestyle is high and there is no money is because sometimes you know…people like to show off number 9, I can lie to myself that that chicken I couldn’t eat when I had money I can eat now., I can’t do that. I leave my life even when I don’t have money. Secondly, I don’t wish my life to go lower because I get support from sex work and salon so these things are equal. When I realize that I’m about to fall, I just try to rise up again because you know as a sex worker, after you have raised your lifestyle, your neighbor is watching you. When you go low, “go to her house and see, she uses money from other people’s husbands, nobody even wants her. Go to her door and see how she looks miserable” I just apply oil on my body even when I don’t have clients, I don’t want my lifestyle to be low.

**I: Mmm.**

PTO9: Mmm.

**I: Mmh, do sex workers have lifestyle and their income is low? Who has something? Number 3 has talked. someone else? Number 5.**

PTO5: Thank you. My life will not stop because I had decided and I have also saved. My lifestyle will not be low because I lack. Considering that I work in a bar, a sex worker must just be neat and I will just raise it even when it is difficult, I’m the only one who will know that it is difficult, somebody else will not know because I chose that standard.

**I: Mmm.**

PTO5: So, I will not want mu standard to go down. I take strong tea but im very clean with mu lipsticks on, you will not know I took strong tea

**I: Mmm.**

PTO5: Yes, that is how I’m.

**I: Why do you raise the standard?**

PTO5: The reason I raise it?

**I: Mmm.**

PTO5: I had raised it and considering the title of sex worker, they just know that number 5 is a sex worker so when they will know when your lifestyle is low. So, you just maintain the standard. You become clean all the time, that is what makes it high, you maintain the title.

**I: Number 8.**

PTO8: Personally, why I will not accept that my life be low, when I have had enough of =Nyamonye=, I will move to =Usenge=. I must find clients there. I make my hair neatly, put on a tight trouser, I can’t miss to find a client.

**I: Mmm.**

PTO8: Yes.

**I: Okay. It is true that we must raise our standard because we have raised it. That is where we must maintain and our income is low. What do we normally do to bridge the gap? there is a gap here, low income high lifestyle, how do we bridge the gap?**

PTO2: You maintain the standard with the money you saved so that when you begin have s standing again, you return the money you took because you used it so that your lifestyle cannot go low.

**I: That is number 2. Someone else? Where do we get money to bridge the gap?**

PTO2: When income is low, there are sometime that…it will depend on income in the future. I cannot have a low lifestyle all the time, and not move higher all the time. When it is low you will have to adjust, when it is now high, you find a way of filling the gap that was there when it was low the money you saved is what you will use when life is difficult. What you earn in the future you use to return the money you took from your saving. Someone may not know you are in difficulty, but you press on with life.

**I: That is number 2. someone else?**

PTO9: As number 9, I can’t use my savings because I have a target. When I used to charge 500/=…, if sugar was 35/= and is now40/=, now I will not charge 500/= but 1000/=. it depends, it can’t be that “you just give me 500/= for one round, and let nobody know” no, if you find it hard, just leave but I know charges have gone higher. You only quench your thirst by taking water? You will just have to go =Nyamonye= school if that is where water is found and you are in =Nyamonye center. You must quench your thirst by drinking water Now the charges have to be increased.

**I: Mmh.**

PTO9: But the money I have saved I can’t touch.

**I: Mmh, that is number 9, she is saying she will bridge the gap by increasing charges for her clients, mmh, someone else? Number 3 how do you bridge the gap**?

PTO3: I can fill that gap…let me say I have gone to some club; we have some people who want to try you and know your life. They always see how you dress, how you behave, sometimes they find you taking some sodas nowadays there is ulcers, you will hear somebody say they don’t want to take sodas, they have ulcers, they only want Delmonte .This man comes and every time he come I must raise my standard.

**I: How?**

PTO3: if I was taking a drink worth 250/=, it must go to 300/=.

**I: Okay.**

PTO3: Maybe he wants to show you… I must have my money because I have saved, I will use money to get money you can be told go and book a room and put everything you want there and then pay. You can use 5000/= and you will get 5000/= more. I have somebody like that, he will tell you to just use your money he will pay back.

**I: Mmm**.

PTO3: You will find that he sends you double the money.

**I: That is number 3. that is money from clients, isn’t it?**

PTO3: Yes.

**I: Someone else, number 8?**

PTO8: When I’m low, and I don’t have money as number 8i can get a client then I tell him we go to =Summary= to drink. I have already known my stock is low, to be in a lass like he buys for me beer which is 200/= and he has ordered for me 5 bottles. Maybe he is in a hurry he wants to leave and pays for the beer at the counter, I will talk to the person at the counter to give me 1 bottle which I will take slowly, after he has gone I take the money the person at the counter and bridge the gap.

**I: Mmm.**

PTO8: Mmm.

**I: That is number 8. someone else? or we continue, lets continue.**

ALL: Mmm.

**I: Do we borrow as sex workers? Where do we borrow from? Number 9.**

PTO9: Maybe you went for sex work, got 2000/= and used all of it. You even forgot there was no cooking oil, I will go to the shop and take cooking oil and sugar. After borrowing… some people would borrow and just use those things without minding. I must borrow.

PTO3: We borrow when children are going back to school, I go to the boutique, borrow shoes if we agree.

**I: Borrowing shoes from a boutique for the children…**

PTO3: And uniforms also.

**I: Number one where do we borrow from? Who do we borrow from?**

PTO1: You can be stuck and you have a friend you know can not fail to help you. So, you can borrow from a friend.

**I: You borrow from a friend, number 6.**

PTO6: You can borrow from Chama.

**I: Chama, you told me earlier that you normally lose money that you save in Chama.**

PT: (Laughter).

**I: That is what number 6 has said. Number 6 says we can borrow from Chama. Number 7**

PTO7: We can borrow from Mpesa.

**I: We can borrow from Mpesa. Number 8.**

PTO8: You can borrow from a neighbor.

**I: You can borrow from a neighbor, number 5.**

PTO5: When I have a problem, I normally talk to the accountant to lend me money and deduct from salary at the end of the month.

**I: That is where you work?**

PTO5: Where I work.

**I: Number 2.**

PTO2: I can borrow money from someone who we do business with because I know I will be able to refund quickly after selling.

**I: Mmh, number 4.**

PTO4: I normally borrow from the bank to pay for my child’s school fees.

**I: Number 4 borrows from the bank to pay school fees, number 3 borrows shoes from a boutique when children are going back to school, number 6, did you say Mpesa?**

PTO6: Chama.

**I: Chama, where is the money borrowed from Chama spent?**

PTO6: Maybe your child was sent away from school for school fees.

**I: Mmh you pay school fees. Number 1.**

PTO1: I can borrow from a friend.

**I: What do you do with the money you borrow from a friend? What can you use the money you borrow from a friend for?**

PTO1: Maybe there is nothing in the house.

**I: You can use it for buying what you use in the house.**

PTO1: Mmm.

**I: Number 3.**

PTO3: I can borrow when I want to cultivate the land.

**I: Cultivate land, number 5.**

PTO5: I like borrowing money when I want to refill the gas, I don’t like using firewood and I don’t use charcoal.

**I: That is number 5, number 8.**

PT: (Laughter).

PTO8: I can borrow money when there is lack in the house or school fees.

**I: Okay, and how are they normally paid, these borrowed monies? I have gone to the shop and took cooking oil; I didn’t have cooking oil in the house. How do I look for money to pay it back with?**

PTO9: There are so many ways I can get money to pay back the cooking oil. Before I go for sex work, I go to the salon and I borrowed cooking oil worth 300/=, sugar worth 150/=, when I work in the salon up to evening, I have got money and is able to pay back the debt.

**I: That is number 9, how do we refund the monies? Number 8.**

PTO8: As number 8, personally when I borrow money, if I go for sex work and get 1000/= and the money I borrowed is 500/=, I go and pay that debt so that next time if I have problem, I can go back and borrow again. The remaining 500/=, I can keep.

**I: That is money from sex work?**

PTO8: That is money from sex work.

**I: Mmh, we can use money from sex work to pay debts. Someone else?**

PTO1: I can tell you to just lend me without fear because I know when I sell charcoal at night, I will have the money.

**I: You can pay from money got from selling charcoal. Number 4.**

PTO4: As number 4 the money I normally borrow from the bank, I pay with money from sex work. I know it is money for school fees and it is a lot. I can not pay it back with the little money from business. So, it is it is money from sex work that I use to repay the money to the bank.

**I: Okay, and what can sex workers do to increase their income? To increase their income, what can sex workers do to increase their income? Number 9.**

PTO9: Increase charges.

**I: Increase charges. Number 3, or she has said what you wanted to say?**

PT: Crosstalk.

**I: Number 9 has said what she wanted to say, mmh, number 9 wants to add.**

PTO9: Looking for more clients.

**I: Looking for more clients. Number 8.**

PTO8: Dressing neatly.

**I: Does dressing neatly income generating?**

PT: Crosstalk.

**I: Its number 9 talking… number 8 talking. Number 8, tell me.**

PTO8: When you dress neatly… as number 8, when I dress neatly… you know when I’m going for sex work, I can’t have poorly made hair, and the way I’m dressed, my breasts are hanging. You have to dress well and use a good bra that makes them look neat. Then you dress neatly and not like someone going to the garden.

**I: That is number 9… number 8, number 6.**

PTO6: Talking nicely.

**I: Mmh.**

PTO6: You have to know how to talk to your clients. You can’t just be rude all the time who wants everything by force. You find a way of getting to them, sweet talk them and get money.

**I: Mmh, someone else? Is there… Mmh, number 1.**

PTO1: I’m saying, after you have gone there and come back, you can use the money you got there to buy for yourself something.

**I: These things we do to increase our income, are there situations when they put us at high risk of HIV infection or violence? Do they put us at any risk? Number 9. Like number 9 said you increase charges or increase number of clients. Can that put you at a risk of violence or HIV infection?**

PTO9: You know when I go for sex work as number 9 and I’m standing in the darkness somewhere, and I see you come with your husband in some vehicle, I have seen you and your breasts are not neat and mine are very neat. I will go for your husband, and you know that time I have changed my walking style, I can’t use this kind of skirt at that time. It is in the darkness; you can even wear a biker and insist in calling him. There can’t miss war at such a place, there will be some fight. When I increase cost, I increase it because you have a problem. Maybe you get someone, have sex with him without a condom, if you do a test later, you may find you are sick because you did not know. Maybe you have increased the charges, you have booked a room and he insist that if you increase charges up to 2000/= I will give you but you don’t have condoms and he did not also carry.

**I: Someone else, number 5.**

PTO5: What can make us to be infected with HIV or experience violence is, sometimes I have someone I have maintained here at the bar. He knows that every time he comes, he will find me. So, one day he may just decide that we have been together for a long time, let us not use condoms. I can be infected out of that; he believes we have been together for a long time and I also reason he is my regular client. I may be infected. For me to be beaten, I have them with me here and I have taken money from this one, this one and the other. When it is time to go to the room, it is war, they all want me and I can give all of them time. That can lead to violence.

**I: The money we borrow and the debt we have can be how much? We all said we borrow money?**

PT: Chorus yes.

**I: We borrow school fees, we borrow from the shop, we borrow to purchase school requirements when children are going back to school, averagely, the amount we normally borrow can be how much?**

PTO1: 500/=

**I: Number 1 500/=, number 3.**

PTO3: 5000/=

**I: 5000/=, number 4.**

PTO4: 6000/=

**I: 6000/= number 2.**

PTO2: I can borrow 300/=.

**I: 300/=.**

PTO5: 4500/=.

**I: 4500/=, number 5. Number 8.**

PTO8: I can borrow 4000/= which I know I will be able to pay back faster.

**I: 4000/=, number 7.**

PTO7: 5000/=.

**I: 5000/=, number 6.**

PTO6: 1000/=.

**I: 1000/=, number 9.**

PTO9: 2000/=.

**I: Are there times when we think about leaving sex work?**

PT: Yes. (chorus)

**I: Does that come to our minds?**

PT: Yes. (chorus)

**I: Mmh.**

PTO5: The reason why we consider leaving sex work…

I: **That is number 5.**

PTO5: You have left your home, and all ladies would wish they were married. And you have decided to wear short clothes and move from place to place. You don’t even have a name where you came from. So, you can have a desire that “will I one time leave this work”? So, we have the desire.

I: **That is number 5, number 9.**

PTO9: That work can be left.

I: **Is it something…**

PTO9: As number 9, I can think of leaving sex work because I didn’t go training to do it. What you are trained to do is what you cannot leave because you know it well. But something I have just come across; I don’t think it would be wrong even before God. Personally, I would wish to leave it and do something else.

**I: Number 8.**

PTO8: in my opinion, it is something that leaves somebody vey exhausted. You can reach a certain age that you should not do sex to earn a living. You may even have some terminal effects on your body, or some diseases like sigete or rariw. So, you feel no…we have encounters with different men. You can find one with a short penis, you can find one with a long penis, you can find a difficult one who wants his own style know to him. Maybe you are used to lying on your back and he wants you to bend, and sometimes you are not comfortable bending, so you decide let me leave this thing. It has helped me achieve somethings. Let me go and do my work, so you decide to leave it.

**I: Mmh, leaving sex work, does this come to the minds of those doing it? Number 7, do you have something?**

PTO7: Thinking of?

**I: Thinking of leaving sex work.**

PTO7: The reason I can leave it is because of age. You may have reached a certain age; your children are grown. Your children are doing sex work and you are also doing sex work, so you just feel that at my age, I should just leave it.

**I: Mmh, number 4 also wanted to talk.**

PTO4: She has said some but I can add as number 4. Mostly I work at the beach. I feel I have to leave it because I may have sex with someone who has had sex with my daughter when she is grown.

**I: Mmh, number one.**

PTO1: I may feel I should leave sex work because sex work is just a title. You have a bad name even as you walk around. You can say…even when you have gone to school, they will say somebody who has not gone to school is better than you because they are married and you are doing sex work even though you have gone to school. Every evening you are at the hotspot welcoming clients. So, you can be tired with it so that your name…

**I: Does this kind of thought come to someone individually or it is sometimes you can converse when you are seated somewhere? Do you converse as peers or sex workers about this? Or it is something you sit down and decide that as number 1, or number 2, mmh.**

PTO3: there are some places we go and we risk and there are some things that happen to us. It is never easy, and you can see… you can go back to your house and as you sit down and wish that if I could have got such a thing… like me I started doing it because of problems or the life I’m leading with my mother. I’m not doing it because I like it, you see. You can find that it has not happened to me but has happened to you or number 9, maybe they went to the room she was mishandled and beaten and injured. We have some thugs… there are some men… somebody can make you… you are in the bar welcoming guests and he insists he is the only one who should have sex with you. If he finds out you had sex with someone else, they will gang up even three men and rape you. We refer to that as raping because 3 men will have sex with you at the same time then put a bottle in your vagina. So, these are things that when our colleagues are hurt, you are shocked and start thinking, do I leave? Then you again think that even if I leave it, where will I begin from. So, when we have support, it can be easy to leave, continue doing something else and quit.

**I: Is it a conversation we have among ourselves as sex workers or it is something somebody thinks about on their own? Number 5.**

PTO5: Like this friend of mine really like that conversation, we always talk with her. She normally asks “will we one day leave this thing” and I normally tell her it will happen; everything has its own time. So, when the time comes, we will just leave. Age begins at 40, it really becomes sweet.

PT: (Laughter)

**I: What is sweet at 40?**

PTO5: It is sweet, sex becomes so sweet. So, I don’t know when I will leave it.

**I: You have said you normally have a conversation with your friend, what normally trigger those discussions?**

PTO5: We just see how our children are now growing, you see how you and your neighbor are progressing. You just have to think about it.

PTO3: There is name calling there also. When your child disagrees with your neighbors’ child, your child is told “go and tell your mother to show you your father, you should also go and see your father, [mentions name] has his father here, who is your father?” Things like that are so painful as a parent, so you can decide to leave but our cry is how I will live. My lifestyle is high, I’m not saving, so I’m forced just to do it. But if someone can get support from somewhere, you just leave.

**I: That is number 3, why do sex workers leave sex work, number 9.**

PTO9: Personally, what can make me leave this work…you have gone to your parents and there is a cousin of yours who was married. You have been called to go and serve visitors, you still don’t know your target, you are busy serving visitors. Somebody has come with the husband and children driving, the children rush to hug their dad as your children watch, they don’t know… they are confused. “when will we have a father”. When you come back the child asks, why are others having a dad, why not us? So, after they have gone to sleep you are left thinking, if my child is asking me such a question, there is some life I should live. So that encourages you to leave and begin a new step.

**I: That is number 9, someone else.**

PTO1: As number one, what can make me leave this thing… you have gone to entertain guests, you are the only one they see. “call the daughter of so and so, she is the one who knows how she will entertain these guests very well”. Maybe they are guests who will be there the whole night, you are the one who entertains guests, you know how to open those things and when you get there, everybody becomes crazy. You have also been given a name here. That can make you feel let me leave this thing.

**I: That is number 1, mmh number 8.**

PTO8: As number 8, what can make me leave sex work, even where you did not pass, or a husband you were not with, you will hear people saying it is that sex worker who disappeared with so and so’s husband. So, you always have a bad name.

**I: Okay, why do sex workers leave sex work. Anybody who wants to respond, number 5, do you want to respond.**

PTO5: My colleagues have responded but I can still add. You will leave it depending on the age I was talking about. You will be forced to leave and check what you have put down, and how do you want to start. Age will force you to leave it.

**I: What age should someone reach then they quit?**

PTO5: I want to go beyond 40 years; I feel it is too sweet at 40 years. So, I want to feel the sweetness first then… even at 45 years old, I go and do farming a home.

**I: Number 8, that is number 5.**

PTO8: I can’t pretend that I can leave at 4o years old. Because I see old men still living, so I can’t leave sex work because I’m tired but not because my child is grown. I can’t do sex work where I know my child can do sex work. I do sex work with my agemates.

**I: Okay, which age is it? Number 5 says it is too sweet to leave at 40 years old, you leave it at 45 years old. Someone else?**

PTO1: You leave it when you die.

**I: Number one says you can only leave it when you die, number 3.**

PTO3: You can leave because of sickness, maybe you have stroke, you can’t get clients. Sometimes the women we have their husbands go to witchdoctors, they can kill you or make you sick. Some men are also jealous who had sex with you and used some powers, so you can’t have another man so you are just there. So, such things happen.

**I: Age? number one says you leave when you die. Which age do you say number 9?**

PTO9: It is not now; I can’t leave it now. I still want to do sex work. I even think of doing it up to 50 years of age. I will know my daughter has also started and I leave for her the button and take a rest. I can’t leave it now.

**I: That is number 9.**

PTO3: I think the age to leave sex work is 80 years old.

**I: 80 years old?**

PTO3: Because if[inaudible] can give birth, what did they do?

**I: That is number 3, 80. What do you think, for someone to leave sex work, do they plan or it just happens? For someone to leave sex work. Number 8.**

PTO8: as number 8, it is in sex work that you can find someone to marry you and you leave sex work to become a wife.

**I: Here you will leave without planning?**

PTO8: You had planned that if you find someone who is willing to marry me, after I’m fed up with sex work, I can be a wife.

**I: Mmh, someone else? Do people who leave plan or they just find themselves leaving?**

PTO9: As number 9, I have to plan. That is why I said I’m saving; I’m saving because I have a target. That is why I also said I have no training in this, because I know I will leave it. I’m planning that even after 10 years, I can leave it because I have saved enough, and can do what I wanted to do.

**I: Someone else?**

PTO3: It is planned, every work is planned. Even teachers retire, so I can decide like mama [mentions name], When [mentions name] is in class eight, these days children mature first. I have a grandchild, I have a sister who gave birth at 20 years old, I just feel that when I reach a certain age, you know we have to be open. We are open, it is just that we don’t want children to know that is why they stay away from us so that they don’t find out the money we use, this is how our mother gets it. So, when my child begins to relate with girls, I will have to leave it and I will know what I will do after I leave it. I will have some business, so I can do sex work a bit but I have something. We can also not say we will quit completely. I’m used to it and I’m just trying to avoid it with time, I will leave it slowly by slowly.

**I: Okay, what do those who do sex work do? Number 8, what do people who leave sex work do?**

PTO8: After I decide to do sex work, I can now major on my work. Let’s say like I go to do my business in the evening, now I go at 2pm and maintain it because I have quit sex work.

**I: Do you do it where you were doing it or you change the place?**

PTO8: I just is where I used to do it but change time?

**I: You change time, number 5.**

PTO5: After leaving sex work, what I can do very well is look for a place and sell beauty products. I stock nice things to maintain people and make them look beautiful.

**I: That is number 5, anybody who wants to add? Which work would someone who has left sex work do and where can she do it?**

PTO4: I can leave sex work because I was saving, now I can use my money to expand my business.

**I: Someone else? Number 6.**

PTO6: If I leave sex work, I can leave sex work because I was saving, I can wash clothes at a fee or do hairdressing. So, when I leave sex work, I can leave doing hairdressing in my house and open somewhere at the market center and make my money. So, I will only have to go their everyday and maintain time.

**I: When you leave sex work, changes they have after leaving sex work? Number 8.**

PTO8: Changes that someone can see is when your dress code changes, you put on long clothes, your life has to change and leave the things I used to do.

**I: That is number 8, number 6.**

PTO6: When you decide to leave sex work, I think you will have a good name, and you will have respect, not that every time you are seen they say you are going for sex work. They will say you have a salon where you go to. You will have respect even where you stay.

**I: Mmh, number 5.**

PTO5: You will check the kind of friend you have and you may find you have many friends. people may not have liked you even your family. You will good friends and be a good person who even goes to church and find someone good.

**I: Number 7, change in the life of someone who leave sex work.**

PTO7: You have to change because your life has also changed. If you left at a particular time and people knew you are now going for sex work, you will just be at your business at this time. So, you will have a name.

**I: Number 1, do you have something?**

PTO1: The reason you have to change is because there is a difference. They used to say, “you are going to so and so’s place, will you find her? She had left long time ago”. So find there is change, you have long cloths like a grandmother and your are unkept. “how was she in the previously, have you seen how she looks like, go and see how her lips are dry, she is looking miserable.” You have to change because you were not prepared. Because previously you were getting money every time.

**I: How do those who leave sex work manage with the changes that take place? You said you will get a new name?**

ALL: Mmm.

**I: What did you say?**

PTO5: A new name and maintain the work I will be doing. So, when I’m doing that work, there must some change.

**I: And those who look miserable with dry lips, what do they do?**

PTO1: It will be difficult for them.

PTO5: They did not save.

PTO3: Sex also works on the body.

**I: Number 1 and 3, identify yourselves with your numbers. So, what were you saying number one?**

PTO3: I’m the one who was talking. As number 3, you will your body recovering, even if you were slender, you will add some weight...

**I: When you leave sex work?**

PTO3: …because sex work leaves the body in bad shape.

**I: Okay, do you sex workers who have left sex work and returned? Mmh, let me start with number 8.**

PTO8: thank you, I can say I have seen fellow sex workers who were married from the bar where they were working, they later found life difficult where they were married and…she got a husband from the bar and was married. She was feeling marriage life was difficult she could not manage; she came back to the bar.

**I: That is number 8, number3.**

PTO3: there are times like we said there are people who save…

**I: I’m asking do you know those who have left and came back?**

PTO3: That is where I’m coming, so someone may have felt comfortable and settled in their home. She took a loan or bought a plot then she is relocated from there and she becomes desperate without where to go, she will think about what she was doing that let me go back and look for some more money.

**I: Have you seen someone that experienced that?**

PTO3: It is so common; I have friends who left and returned.

**I: Do we know people who left and returned? Number 5 is smiling like she has something.**

PTO5: I don’t know.

**I: You don’t know?**

PTO5: Mmm.

**I: And those who leave and come back, what negative things can happen to them when they come back? You were doing sex work then you left and came back, what negative things can happen? Number 8.**

PTO8: As number 8, something negative that can happen to you is that maybe you left sex work without HIV. And when you come back you may find it.

**I: Get HIV?**

PTO8: Yes, get HIV.

**I: Mmh, number 1.**

PTO1: The reason you can come back, you may have left it to go and get married and it does not work out. So, you may feel you are wasting time, if I were doing sex work, I would have made this amount of money, let me leave this thing. You look back to where you came from and return.

**I: Number 9, negative things that can happen to someone who leaves and come back are like which ones?**

PTO9: it is always said that if you quit drinking alcohol and return, you will now only quit when you die. So even sex work when you leave and come back, you may get syphilis are people can get into a fight that leads to your death.

**I: And what positive things can happen when someone leaves and come back?**

PTO9: If it someone who had been in marriage and did not see anything good she achieved, the people she left have gone a step ahead, they will be encouraged to do what others are doing. Maybe she lived with unmet needs and then comes to inquire from someone “how is everything doing, what good do you experience here that makes you better than me” you can engage them in a conversation and tell her your achievements, if she can catch up from there and start saving money and buy some things because of the encouragements.

**I: That is number 9, someone else, anything positive that can happen to someone when they leave sex work and return? Number 8.**

PTO8: As number 8, in sex work I can meet a man who can buy for a vehicle and make live a good life.

**I: Anything good that can happen to someone who leaves and come back, number 5.**

PTO5: If I left in foolishness, I did not save, I did not buy anything, I may come back with a lot of anger and do it with a lot of zeal. I will have to do something.

**I: And you told me during the time I asked you whether you can leave sex work, that there are things you would like to achieve before you leave. What are they?**

PTO5: Pardon.

**I: What you would like to accomplish before leaving sex work, number 8.**

PTO8: First you would wish to buy a plot and build or build for your mother.

**I: Buy a plot or build a house for your mother, number 5.**

PTO5: My children to complete form four.

**I: Children must complete form four, number 3.**

PTO3: That is what I wanted to say.

**I: Your children’s education, number 9.**

PTO9: Build rental houses and complete.

**I: Build rental houses, are these things we have began doing or they are still ideas? I know you pay school fees, what about buying land, buying a plot, build for your mother, number 9.**

PTO9: This is something I have in my mind.

**I: It is still and idea, you have not started doing it?**

PTO9: I have not started but I’m planning.

**I: Mmh, number 8.**

PTO8: What I’m planning like buy land to build a house is still in my mind, I have not started doing them.

**I: What are you doing to ensure that happens?**

PTO8: The plan I have as number 8 is to save bit by bit.

**I: In the past 5-10 years, do you know those who left sex work and never returned? Sex work.**

PTO9: I know.

**I: Number 9 knows. What made it easy for her to leave?**

PTO9: We were doing with her sex work; she had left her children and she did not want to accept. So, her eldest daughter she left with her parents gave birth and there was nobody to take care of the child. She was also not saving because she would tell us she is has no responsibility and that is what we knew. She left without any money to go and take care of her grandchild. That is how she went and has never returned to date. She is with her parents.

**I: That is one case, number 1 do you know somebody who left?**

PTO1: Yes, there is a lady who followed her husband from =Nairobi= and found him in another lady’s house. She burned this lady’s face with acid.

**I: That is number 1, any other, who knows anybody who left, number 8.**

PTO8: I saw a fellow sex worker leave sex work because she was pregnant. She later got married to a man and left the man after giving birth and came back to sex work.

**I: Who else raised her hands, number 4, number 3.**

PTO3: I have seen someone who left sex work, her children made her leave. She educated her children with money from sex work, one is a police man, one a doctor and the other is a lawyer. She was doing sex work at home and her brothers were with her children. So she would just send support where her children used to live. When they were out of school, they told their mother they want her home and they want to know their father. She was afraid of telling them they had different fathers. She bought land with her children and built then she left sex work as is just at her home.

**I: What are the challenges that people who leave sex work face? Number 5.**

PTO5: They will challenge if they did not save. If you did not save you must have challenges because you are now not doing anything. You don’t have something to fall back to, you don’t have any poultry, you don’t have a goat, you don’t have anything. You must have a challenge because you don’t know where to begin from. People will start saying “How was she doing sex work, she has just come empty handed?” There is such challenge.

**I: That is number 5, number 8, do you want to talk?**

PTO8: The challenge you can face in sex work you can find…

**I: When you leave, you were doing sex work and you leave.**

PTO8: … you can leave and get a husband and you are unable to give birth. This can be a problem and you can not live with this person if he wants a child. So, you will decide to go back to sex work.

**I: I had explained to you earlier about Jitegemee study. I explained it is done to ensure female sex workers save so that they can say no to sex without condoms or take a break from sex work when they want to rest. I also said sex workers will save their own money, they will no money they will get from somewhere. They will use the money when there are no clients or to help them prepare for life after sex work. Just as I had explained to you, Jitegemee has no profit, it is not like Chama that will give you head ache that the day has arrived and you don’t have the target. You choose who you pay depending on what you have, when you want it, you are the one who decides what you withdraw, whether you will refund it or not. Another thing is that… I have said it is different from Chama. Out of the things I have explained, is it something women who do sex work will accept in =Kenya=? Number 5.**

PTO5: They can accept it because of the education it gives, you can leave or stay, it does not force you to leave sex work. It is also emphasizing on saving, so if you are given the information and you like it, you will join Jitegemee.

**I: Considering what I have explained to you and you have heard, is Jitegemee something that will be accepted in Kenya, by some people who are not here in =Nyamonye=? Number 9.**

PTO: As number 9 I can say they will accept it. You know something bad, is bad before you hear about it, or you have not been explained to what it is. But when they sit as we have sat, because you know even us, there were people who were not free with this, but now they have found out it is good, they understand it and have accepted it. So even those people if they get to understand about this, know it is good, they can accept it.

**I: And what kind of sex workers will accept it?**

PTO5: I think those who have families.

**I: Those with families will easily accept it. Number 2, what kind of sex workers will accept it?**

PTO2: it depends on… when they have a meeting and they are given information; you know information can be given and when people leave there, they follow their own ways. But those who understand it and accept it will find some help in the future.

**I: Why would people with children like it?**

PTO5: Why they would accept it is because there is information here, you learn about saving, you learn about preventing HIV, you learn how to avoid violence and how to avoid getting into trouble. There is information from here.

**I: That is number 5. The type of sex workers who will accept Jitegemee, number 9.**

PTO9: As number 9, I can say it will be accepted because you know it is the person who has been pinched that removes the shoes. Sometimes you are the one who knows where the shoe pinches, and you long to remove it. When you get this information, sometimes you used to get money and spend all of it, buy clothes and dress well. You don’t even know how to light a jiko. When you join, you will know you need charcoal, you can sometimes use firewood to cook, so that the money I was using to buy gas for cooking everything I save. There may be a problem somewhere that forces you to save to solve it.

**I: Who wants to add? Which type of sex workers will not accept Jitegemee?**

PTO3: Those who claim they don’t have any responsibility.

**I: Number 9 is saying…**

PTO9: They will not accept it “I’m all alone, I don’t have any responsibility, for feeding who, I’m all lone” they will not accept it.

**I: That is number 9, someone else? The type of sex workers who will not accept it. Number 8. I have surprised you. Number 3, the type of sex workers who will not accept it**

PTO3: Women who will not accept it are women who don’t want to work hard and like free things.

**I: Okay and if you have 10 friends who are sex workers, number 5, you have 10 friends who are sex workers and you tell them about Jitegemee, out of these 10, how many will accept Jitegemee? Number 1.**

PTO9: I’m sure if I have 10 people, 6 will accept, only 4 people will fail.

**I: That is number 9, why would the 4 fail?**

PTO9: Because they have no responsibility, the four of them.

**I: Number 6 if you have 10 friends and tell them about Jitegemee, how many will accept and how many will reject?**

PTO6: If I talk to them well, all of them will accept.

**I: All of them will accept, number 7, you have talked to 10 people about Jitegemee, how many will accept how many will reject?**

PTO7: If I talk to them well, all of them will accept.

**I: All of them will accept, number 8?**

PTO8: If I talk to them well only one will not accept.

**I: Why?**

PTO8: Because they have no responsibilities.

**I: Mmh, number 5.**

PTO5: Let someone talk.

**I: Number 2.**

PTO2: If I talk to them being that people have different understanding levels, only two people will not accept.

**I: Why will they accept?**

PTO2: They don’t have any responsibility and they know they can eat even when they don’t work.

**I: Mmh, number 4.**

PTO4: Only two people will fail because they don’t have any responsibility, they spend they get.

**I: 8 will accept, mmh number 3.**

PTO3: 3 people will reject and 7 people will accept.

**I: Why would the 3 not accept?**

PTO3: Because they only love leisure.

**I: Mmh, number 1.**

PTO1: Only one person will disappoint me, because they think about here and now.

**I: Okay, when Jitegemee is to be implemented, and we want it to work for you, what would you like to be included in how it runs, how should it work? What are your opinions on that? We are about to finish.**

PTO3: I have not understood, repeat again.

**I: When we are beginning Chama, we normally sit down and set rules that will govern it, this where we want save our money, this the amount of money we want to contribute. So, when we want to implement Jitegemee, how would we… let me read something here. What should Jitegemee have so that you accept it? What should it have it so that it acceptable to sex workers? Number 9.**

PTO9: support.

**I: In which way?**

PTO9: When you give us information and encourage us, you tell us and do follow up, you call us to meetings and talk and see our progress. Secondly as you said we can’t join it because you have not implemented it, so when it is implemented and we are able to meet… you know even when you want to start a Chama like we want to start one on Friday, somebody comes with sugar, another salt and the we see whether we can join. And that is when we can meet and talk and discuss. What we have discussed today if you call us one time, we may discuss that. You can call us to discuss something different.

**I: Do you feel we should meet and discuss?**

PTO9: We should meet and talk.

**I: Number 8 was raising her hand, what should we do so that people can accept it?**

PTO8: When want to implement it, we must have a secretary, treasurer.

**I: We should have an office.**

PTO8: We have an office.

**I: Number 5.**

PTO5: We should set a day for meeting and do elections. After elections, we will know the way forward, so that what we... People from out who want to know what we do, and we will tell them we are Jitegemee. What does Jitegemee do, where is it, when do they meet, you will tell people and that is how it will spread.

**I: Is there someone with a different opinion? Number 3.**

PTO3: Jitegemee should bring for us a Sacco. Second, we should have an insurance for our work because you can get sick out of it. Let me say the way you have brought it today I agree and register, I want to go treatment when something happens to me and I’m not able, so it is good if you can cover us. Jitegemee has now taken us and can handle. Sacco can lend us money from business and we refund.

**I: What will sex workers not like from the things I explained? Is there anything they will not like, number 9?**

PTO9: No.

**I: Number 6.**

PTO6: No.

**I: Number 7.**

PTO7: No.

**I: There isn’t. What will sex workers not like about Jitegemee?**

PTO3: What I know sex workers don’t like are restrictions but I have not seen any restrictions in Jitegemee. All the things we read, it was about your choice not you must, so there is no difficulty.

**I: What things will they like? Number 3.**

PTO3: There people who are doing it because they like it…

**I: What would they like about Jitegemee?**

PTO3: What they will like about Jitegemee, is that you have educated them they don’t have to do sex work to survive.

**I: That is number 3, what will sex workers like about Jitegemee? Number 5.**

PTO5: Saving.

**I: Saving, any other? Number 8.**

PTO8: Development.

**I: Number 1**.

PTO1: Information on saving.

**I: Number 9.**

PTO9: It has been an eye opener

**I: These things we have talked about, do you see where the rights of a sex worker can be violated? Is there somewhere Jitegemee can violet the rights of a sex worker?**

ALL: No.

**I: You have all said there isn’t or number 3 you have something?**

PTO3: No.

**I: Which challenges can we face as we implement it? We are getting your opinions on how you want it done.**

ALL: Mmm.

**I: After we got your opinions and we want to implement it, which challenges can we have as people implementing it? Number 3.**

PTO3: You can have 10 people and only 6 people accept and 4 don’t agree.

**I: Number 9.**

PTO9: Silence.

**I: Number 9 does not have anything to say, I know you are now tired.**

ALL: (Crosstalk)

PTO5: We did not understand that so well, help us.

**I: Ooh, we want to implement Jitegemee and we have got your opinions and it should now begin, which challenges can we have for us who are implementing it? Number 8.**

PTO8: As number 8, we can agree on where to meet and the days to meet.

**I: You have answered some question we asked earlier. Mmh, number 9.**

PTO9: When you are weeding a plant, you start from the root. So, when you are weeding and pruning, you know you will not feel the weight of the tree. As the way you have began talking to us, you should do it well, and change opinions that are not relevant to Jitegemee. If that happens, I can’t see any challenge you will face.

**I: Who can see any challenge that we can face when we implement Jitegemee?**

PTO3: Before I respond to that, is Jitegemee’s aim for sex workers to leave sex work or we can be given such information and support while we do sex work?

**I: Who can tell her what we said earlier? Number 5.**

PTO5: Jitegemee does not give any support. You save your own money; we will be given different information and know how to proceed.

PTO3: I still don’t understand.

**I: Can you answer her?**

PT07: Jitegemee does not force you to leave sex work, you can choose to leave or continue doing sex work.

PTO3: If that is the case, Jitegemee cannot face any opposition because you are leading a life of your choice. Jitegemee wants us change our attitudes so that we live good lives and not be buried in a bush somewhere when you die.

**I: As we finish, when Jitegemee starts, number one how much would you want to save in a week?**

PTO1: In a week?

**I: Yes, how much can you save?**

PTO1: 1000/=.

**I: 1000/= in a week and where would you save it, where would you like it saved?**

PTO1: =Equity=.

**I: Bank. Number 9, where would you prefer it be saved?**

PTO9: I can save in the bank.

**I: Bank, and how much would you save in a week?**

PTO9: I can save 1500/=.

**I: 1500/=, number 7?**

PTO7: I can save in M-pesa.

**I: How much in a week?**

PTO7: 500/=, number 8?

**I: M-pesa, how much in a week?**

PTO8: 750/=.

**I: 750/=. Number 5?**

PTO5: Bank.

**I: Bank, how much would you save?**

PTO5: 2000/=.

**I: 2000/=, number 2?**

PTO2: M-pesa.

**I: How much?**

PTO2: It depends on my income but it can be something around 700/=.

**I: 700/=in a week, number 4.**

PTO4: Bank.

**I: Number 3?**

PTO3: Bank.

**I: How much would you save?**

PTO3: 1200/=.

**I: Number 4?**

PTO4: 2000/=.

**I: 2000/=, Okay, we have taken too long. Thank you for sharing your opinions. Is there any body with a question before we finish?**

PTO5: When will you be back?

**I: You will be told when we will be back.**

PTO5: You are welcome.

**I: Thank you, is there any other question? There isn’t? Thank you all.**
