# Supplementary material for: Eat a little and save a little: A qualitative exploration of acceptability of a potential savings intervention to reduce HIV risk among female sex workers in Western Kenya
Source: PLoS One. 2024 Dec 19;19(12):e0310540. doi: 10.1371/journal.pone.0310540 (PMC11658496; doi:10.1371/journal.pone.0310540)
Supplement: S1 File — (ZIP) [file pone.0310540.s001.zip › Jitegemee Transcripts and Dissemination Notes for Journal/FGD U.docx]

**FGD ID: FGD U**

**VENUE: IMPACT RESEARCH AND DEVELOPMENT ORGANIZATION OFFICES IN BONDO**

**INTERVIEWER: JANE MORAA**

**NOTE TAKER: LILIAN**

**STARTNG TIME: 1613HRS**

**NO. OF PATICIPANTS: 10**

**CATEGORY: ABOVE 30 YEARS, URBAN**

**I: Okay, welcome and thank you for being here. The FGD will be done in Swahili, English and the participants can respond in whichever language that they are comfortable in so you are so much welcomed, from my brief description about Jitegemee what are your first reaction. Just the way I have explained about jitegemee, (noise at the background) is there anyone who is ready to speak? We have explained what Jitegemee is and the way it will be implemented…**

R: … Yes

**I: So what is the first thing that came into your mind when you heard about jitegemee, R2?**

R2: This is what I can say, I do not want to do sex work as a job forever and ever,

**I: Be audible because we want to record.**

R2: I do not want to do it forever and ever

**I: Ever**

R2: I only want to do it this year, if I get money I will start my own business, that’s it.

**I: Ooh, thank you, any other person, R5?**

R5: Let’s assume I have registered with jitegemee, let’s assume I have quit this job as from now and I have gone home, and I had registered with Jitegemee so while at home how will I be able to meet these jitegemee people?

**I: That’s a good question, remember there are some questions that I will not answer but we need those questions as well so as go we shall continue thinking about this jitegeme idea. When it starts, we will be able to answer those questions okay?**

R3: Yes, I wanted to add something on R5’s point,

**I: Okay.**

R3: She has said that if she retires and goes home, how will it be? So I am asking the point that you were saying that someone I saving obvious you have to tell us the benefits of saving with you guys, and after we save with you guys what development will you people give us as in maybe every month am like 30% I save because maybe I want to use it in the next one year as my retirement so how will this self-development be? Because that’s a point you talked about but didn’t deeply explain well. You just said ooh… we should not use all our money (participants laughing) but you didn’t explain how Jitegemee will be helping us and supporting us.

**I: Personally how would you like Jitegemee to support you?**

R3: Obviously, just as the name suggests ‘Jitegemee’ you are also supposed to depend on us. So it’s a 50-50 thing, right? I stand with you guys and then I get something good from you guy ang you guys are here for us, right? You help us with the knowledge that you have (clears thought) the same way what we don’t know, we can come running to you people right. I’m like do you see this like this that’s why jitegemee has been brought to =Bondo=. To help the ladies of =Bondo= and =Kisumu= and the other regions so if you tell us you have a good idea, like the one for helping us save with you guys, obviously you have to explain to us. Saving is not an issue but directly after saving, I mean how are you helping us. What are we getting from saving with...

**I: Jitegemee**

R3: That saving that Jitegemee I mean am saving this because I am going to get that, you see? Yaah… I am following this path because there is something a head…

**I: Okay, any other person that wants to add something, (someone talking at the background) R1?**

R1: Is there… my question is after saving …after saving with you guys, is there any percentage we will get on top of our saving? (someone talking at the background) okay

**I: I am not saying yes there is a percentage you get, I am saying yes I understand the question**.

R1: Yes (participants laughing)

**I: Because you may think that I agree (someone talking at the background)**

R: Excuse

**I: R1, R1**

R1: Am asking that is our savings, In case you have an emergency and you want to withdraw, can you withdraw anytime you want or there is a period of time that you have to reach so that you can withdraw.

**I: While reading I must have read that this is your money right?**

R: Yes

**I: If you go for sex work and you don’t get a client you can call it like… I think I used Mshwari, like you can call money that you have saved in Mshwari.**

R: Yes

**I: And you get it and it helps you at that moment. But again we want your opinion on how best it can be done or operate, Okay?** **R1.**

R1: Will there, will it be… will it… be having a limit?

**I: For saving?**

R1: For savings yeah, or you can save as much as you can

**I: Remember it a personal goal, do you remember we talked about personal goals**

R1: Yes

**I: And the goal could be I save 10,000 you see**

R1: Yes

**I: If you reach that 10,000 and you feel you have achieved it,**

R1: Yes

**I: That’s it because when you were saving you had a goal, “I reach 10,000 so that I start a business for second hand clothes or boutique, right? Another person may have a goal to reach 100,000, right so it’s a goal if you reach that goal and feel that you want to set a new goal, its up to you (someone talking at the background) that’s Jitgemee .**

R3: Okay R3 so is it a group work or an account something?

**I: That’s what we want you guys to tell us how you would want jitegemee to operate, how would you like it?**

R3: Obviously by bringing this topic to us. There is something that you have on your mind that you haven’t told us yet. So just tell us, so that we can also get it eeh… Like you can tell us, “we have come up with this and we fell it should be group work,” and we tell you that, “we feel that group will tricky for us since we are not at one place all the time lets have it as an account”

**I: Yes**

R3: So you tell us what you prefer then we tell you, “we can’t do it that way because we obviously stay in different places, so you may choose group work and I stay in Kisumu and another person is also in her home so we cannot come together for a meeting since we may choose five and it is not possible”. So it should be something that even if it is through the phone, we have to connect like today we have merry go round and so and so has put something in place that can bring us together. Because if it is like we have to meet like this, heeh that’s tricky.

**I: It’s tricky**

R3: Because we won’t be available.

**I: Okay, so as I hear your opinion group for you may not work?**

R3: If it is group and that group has a leader, right? So someone may not be around and that day they are supposed to save and they will be like “let me just send it to the account.” So, it runs up like in a whatsapp group so one can see “today was ‘chama’ and so and so were present, so and so was absent with apology because they had a certain problem and she couldn’t make it here.” Because that group can also help if someone has a problem and we’ll be like “so and so has a problem, her child is sick”. So we’ll decide that from our savings, we can contribute “rwabe” per person so that she is given to help her child

**I: How much is “rwabe”**

R3: 200 (participants laughing) she takes that 200/- from each person so that she can take the child to the hospital (baby crying) you get? Or we can also get different kind of problems so you see through the phone its easier but for in person meetings, we may meet today and not meet tomorrow. Because how I understand group, we always help one another today I will be the one with the problem and tomorrow it’s another person so it can’t be a daily meeting it can’t be a meeting thing

**I: R8 do you want to add something? Okay R1**

R1: R1, according to R1 I think we can have it as a cooperate thing (someone talking at the background)

**I: What do you mean?**

R1: Cooperate is like… we have a group then from there we register a group then we open accounts. So, it’s like each and every time you want to save, you link your phone to the account so you just pay directly to the account. And at the same time we must have a chair lady or a chair whatever who once you have paid to the account, she at least records.

**I: Okay, okay , thank you that’s a very good idea is there another person wo wants to add on to that again? Okay, that’s fine so I want us to talk about financial burden of female sex workers. What is the normal expenses that female sex workers do and how much is each expense. Which are the expenses the expenses that you people do and how much does it cost you? (Silence) we can begin from this side. Expenses… maybe when you decide to contribute to the discussion for example R7 you can say your daily, weekly and monthly expenses and each of these expense you tell us how much you use for each expense, understood?**

ALL: Yes

**I: Okay let’s move on, do we all do that?**

ALL: Yes

**I: That means almost all of us can talk, right?**

ALL: Yes

**I: Okay (silence) remember there is no right or wrong answer, okay.**

R3: The expense that is very tricky at the moment?

**I: R3**

R3: What I can say which is not working on our favour those of us who operate in the streets, for the people in streets, streets I am talking particularly street, the room hire there is not always good. Because if you have not paid you are written for a note not paid it, it keep increasing because he will not write just for tomorrow and the other days he doesn’t. He doesn’t care because you may be coming from outside and it is raining and you have to shelter inside. Obviously when you get some money you cannot pay for the room first and your child has nothing to eat. You will rush to get the child food first.

**I: To pay**

R3: Because yes you have been a client he will write not payed but the next day if you are being calculated the cost you will see its like will I pay this cause the economy right now is down. I see house

**I: House**

R3: The accommodation for the guest right now it’s the am seeing its high

**I: So when you talk of room,**

R3:Yes

**I: Do you mean the room where you carry out the business or room where you stay?**

R3: For work

**I: Room for work**

R3: Yes

**I: It’s a daily expense?**

R3: Very much

**I: How much do you pay in a day?**

R3: 300/-

**I: 300/-**

R3: Yes

**I: Another expense you do?**

R3: Other expense are normal once that everyone do

**I: Which ones are normal?**

R3: Normal obvious, basic

**I: Like?**

R3: Eating, dressing

**I: How much does it cost you to eat every day**

R3: You know I eat in a hotel. Obvious you can use 500/= in a day without knowing you have used 500 (people talking in the background) because if you go to ask for this food is 200/- you ask this so also the hotel. So it’s just like that.

**I: Okay any other**

R3: The others we are used to.

**I: Any other person who want to share their expenses you have (children playing in the background) R5.**

R5: These expenses, me like a sex worker who work in the street you know right now the weather is rainy, paying is hard the rain is raining you want to look for money at least tomorrow you pay for room, maybe you lacked even 3000/- you want to eat tomorrow The owner of the room wants money, So you see its too hard, you know if it starts raining at 2100hrs you go to sleep. Until the rain stop again you wake up you have sited and you are feeling cold and you persevere at least you get money the children are also at home

**I: So what’s your expense?**

R5: My expense is for room and money

**I: How much is the room?**

R5: The room is 300/-

**I: Okay**

R5: Yes the money for children’s food and others

**I: How much is for food**

R5: Me alone I cost 500/- I eat at the hotel the children I do send them 600/- everyday

**I: Daily?**

R5: Yes daily

**I: Alright the expenses you have spoken about are daily?**

R5: Yes

**I: Weekly**

R5: Weekly?

**I: Yes, what expenses do you do weekly?**

R5: Weekly… no is only that money and money for room

**I: Okay**

R5: Yes

**I: What do you always do once per week if you buy it you are done with it until the week ends?**

R5: Flour, sugar

**I: Okay**

R5: As in flour, sugar mostly… sugar I buy for the whole week

**I: For how much?**

R5: Sugar I do buy even 10kg

**I: How long does it last you?**

R5: It lasts me like two weeks, No like 3 weeks

**I: Alright, what expenses do you do once a month**

R5: Once a month?

**I: Yes**

R5: Cooking oil, the one for cooking food and right now the price has increased

**I: You do once a month.**

R5: Yes

**I: With approximately how much**

R5: I do buy 10kg

R: Liters

R5: 10 liters. Yes

**I: So if you put all these shopping for a month how much can it be**

R5: ten millions (participants laughing)

**I: Anything else you expense on once a month?**

R5: Once a month

**I: Yes**

R5: Mmh the money for school

**I: It’s like how much?**

R5: The child is in secondary

**I: Okay**

R5: You know it’s a lot of money

**I: So is that an expense of once a month or once a term?**

R5: Sometimes I do monthly, you know it depends on how you get money. So I went and spoke with the principle and I told him how am supposed to send money because me my self who look for its me. Okay

**I: Would you like to tell us per month for example, I put aside this amount for fees**

R5: Yes I do send 3000/-

**I: Okay thank you so much.**

R5: Okay

**I: Thank you a lot is there someone else who want to speak expenses R9**

R9: R1

**I: Yes**

R1: The expense that I see a lot, the expense for at least if you go to the club, like me I find my client in entertainment joints. You go to the club you sit there you relax and wait for a guy there at least to find someone to talk to you you must take a beer even if its two, three. There obvious you have used 600/= when seated just there. So see maybe daily you can go to the club, you have set a side 500/- or 600/= that’s only for drinking beer as you wait for a client.

**I: Alright**

R1: Yes

**I: Thank you, is there anyone who wants to tell as her other expenses?**

R3: You had asked about other monthly expenses

**I: Yes**

R3: Rent, now you know lodging is for work. Like R5 when her children come from school they go to the house, that house you pay it’s different expense from lodge expense, because you can’t work where tomorrow neighbour will abuse your child “a child of a prostitute”, you want you stay away, and obvious the money also how you send you can’t keep record of everything, let me just say the truth, you can’t record that this week I’m sending 10. You can’t, you see it’s something… God plans. Because you can say “this week I should just have 3000/=” cooking gas gets finished and you miss it, it forces you to start using charcoal. So I can’t say weekly you save 10k in this job is *pata potea* (one time you get another you don’t) one week can get and another you go dry. So we cant really say that weekly we spend 5000/- or 10000/= that’s a not possible. What I know is that every month you must pay rent. That rent you must pay. Because the people you have left there, those children don’t work, food and rent everything is from the same sourse, there are some people you have left children who can not do laundry. So, you must include the laundry lady’s cost there, she goes weekly to do cleaning. Because you stay here in the lodging even two months before you go there. So, monthly rent is there. That rent is different from guest, because guest if its 300/= per day in a month is 9000/= that’s only for a room you have not payed where you are

**I: Where you are roughly**

R3: in the house where the children are now

**I: Approximately how much?**

R3: It depends, there are those who God blessed they stay in houses that cost 10000/= there are those who are not blessed we are just there

**I: There is how much?**

R3: Just there.

**I: How much is just there?**

R3: It depends with a person. Let’s not go there so… (participants laughing) what I can say. Money we can’t predict money, there we will lie to you.

**I:Okay**

R3: We cant tell you that Friday I make 10,000/= that Friday you will sit there look at people until you are told you are a virgin there is no one who will come for you (participants laugh) you change clothes 5 times they don’t see you.

**I: Okay**

R3: And you will on a Monday, the day that supposedly has no money and you get. So we can’t tell weekly I use 10,000/=, Monthly 20. We just struggle, you struggle like when today you get let me just save this 100/= and eat sardine (omena) let me just save this the child tomorrow is being send back from school, it’s just struggling but we can’t tell you how much. Like R1 she does buy drinks, she goes with all her hopes “God willing I will get someone”, she goes and drinks her 600/= that she could have used for food in the house and may end up without a client. So it’s always half-half, you get or you don’t get. So it’s not direct that we can tell you… its something a must… Or its something that is there, is something that we look for and we don’t get.

**I: But maybe to clarify my question, there are those things somehow we will do, like the rent**

R3: That’s a must

**I: If you don’t pay, those are the expense you will say me I do look after rent a month and this is how much money, you see?**

R3: Sometimes it passes, three months you are being…

**I: But that is something you will have to pay.**

R3: Yes

**I: Is it true?**

R3: Yes

**I: Because its there**

R3: mmh

**I: So at least that’s an expenses that we know that is a must you meet alright?**

R: Okay

**I: What the other one that is a must?**

R3: Food

**I: Yes**

R3: Clothing

**I: Let’s talk about clothing, for example it’s something get after how long**

R1: Clothing you can’t say you say you work on after how long

**I: Okay**

R1: You have to compete in wearing like you want to wear good clothes, is like you have gone somewhere and you have seen a clothe you must buy, because you are seeing like this cloth when I wear it I will look smart and bla bla bla… is a must you will buy so something like clothing you can’t say you have expenses on it like is something that comes like a week you can find you have bought even 3 clothes

**I: For how much money? Do you have that sealing where once you reach you are like “past here no…” lot?**

R3: As long as

**I: Let her finish then you talk.**

R1: Clothing, there is no cheapest cloth like the cheapest cloth that you see you yourself when you wear or you go with it to our job you can get a client, it’s like you buy a dress, dress is 1000 a short ,short goes for 1200/=

**I: Okay**

R1: Trouser has its own price as well

**I: Alright R3 then we move on**

R3: (participants laughing) R1 has also spoken well. You must buy, the way she has said her dress. Obvious she’s someone who hustle in the club. Her clothes must be expensive so that she is seen but us in the street, there in the market those that cost 100/= we pick them because they are short. Clothes for hustle are not like what she is wearing today, something you can wear you and sit in a meeting you are called to somewhere. These clothes you only wear at night, because you are half naked you can’t wear it anywhere. So their prices are low, you just buy it here it doesn’t have a high cost. Hers she can wear she goes even she’s called by an important guest, you know that you will wear a cloth worth 1000/- it’s sexy, isn’t it? But for street you wear transparent that when he pass on the road he sees you direct, he must just say the white one (participants laughing) so you know we can’t wear that trouser that is high waist. We just wear those that are transparent that when you wash at 0900hrs at 1100hrs its dry. Because it’s for wearing at night. Yes

**I: Thank you so much, is there anyone am leaving out? Okay let’s continue. So I would like to know where do most female sex wokers get money to spend. Those women who do sex work where do they get money to spend? And this one am requesting those who have not talked lets also talk. R6, women who do sex work where do they get money to spend?**

R6: They don’t get anywhere, they depend on client. Because everybody has her client, there are those who call you, there are those who come where you are they just pick you.

**I: Thank you is there anyone else? You can also tell me my main source is client the way you have said but I also have side hustle and it’s this, alright? Something like that so thre is main source and this other one, R7?**

R7: Should I speak in luo

**I: Yes, speak in luo**

R7: Am saying like me I work in a hotel, the time I don’t get at sex work sometimes I can add with the hotel, because according to children, school fees is needed, food, house. Sometimes you have gone and you sit there and you don’t get anything, next day the children will go hungry if you have not gone to the club, sometimes you don’t have money to go to the club, like R1 was saying you go to the club you cannot just sit without drinking a soda. And you know if you go and remove that money you can use even 200/= one alcohol you cannot also put one drink on the table you must start even with two. That’s when you can get a client sometimes you don’t get and the next day the kid won’t get something to eat.

**I: Thank you so I would like to ask how much the job pay you?**

R7: That for hotel?

**I: Yes**

R7: 150 per day

**I: 150/- and club how much do you come out with?**

R7: In club if that day is good you can get even 1000/=, 500/= it depends how you agreed. Because clients are different.

**I: Thank you, R6 you told me you depend on client, so client for example in a day how much do you get or per week**

R6: Sometimes you know when client comes, there are those who can tell you they want short. And he wants we just don’t go, short is 300/= so it depends, sometimes you can come out with 2000/= or 2500/=

**I: In a day?**

R6: Yes so that you deduct 400/- for the room.

**I: Thank you. Is there anyone who wants to add, R9 tell us what your source is?**

R9: So like me my side hustle is saloon. I am a salonist. So sometimes you go to work you have not got work (salon) and you want to go to the club, sometimes you have gone to club you have not found a client, so that’s the challenge.

**I: So in the club for example in a week how much can you get?**

R9: It depends. Sometimes you get 1000/=, sometimes 2000/=

**I: Is that in a day or in a week?**

R9: It depends. Maybe per week maybe per day

**I: The way you have said it depends with the day**

R9: Yes

**I: And salon like how much does it pay you**

R9: For salon you can say… you can’t work daily

**I: Okay**

R9: Yes

**I: Alright anyone else who wants to share with us? R5**

R5: Mine is client

**I: Okay**

R5: So it depends how I will talk with my client, you know us we stand on the street. And when a client comes and chooses you, you talk with him aside. You agree on price, sometimes shot. Shot you have agreed is 300/=. So when you reach there another style comes up, “madam when you do me this style how much can I pay you?” as in dog style. You tell him “you know that thing eats me down here so you know you will give me 600/=”, “take 500/=” “No problem”. You remove your clothes and you bend (participants laughing) you know now other than the 300/- we agreed from outside, now I have got again 500/=. He sees he should add you again because the style I have given him (participants laughing). It makes him remove money (participants laugh) there again money comes out so, it depends how someone talks with customer or service, good service

**I: Alright, is there anyone again who wants to add? Yes R4**

R4: Like me I work in a club. So you can get Maybe there is a client you got and agreed, you provided the service and he paid you. Tomorrow he comes back to the club to drink, maybe he can give you a tip, he tells you, “from my bill drink like 200/=,” there at least you have saved, maybe sometimes he pays and he tells you, “you have drunk keep change,” tips and whatever you can come out with can even reach 3000/= or 2000/= in a day. Yeah

**I: Thank you, yeah R7?**

R7: Like me I work in a hotel, (child crying) in that hotel clients do come. Someone can tell you, “you are smart keep change”, he can take your contact and then you will meet outside there also you can make money. Or he can talk with the owner of the hotel, I want this girl, I stay in a certain place, when you go to meet him you make money.

**I: Thank you, thank you a lot now I would like to know why women who do sex work… what is the reason why they use their money where they use? For example the way we heard R1 telling us where she spend her money, R3 also told us, alright? And you too even if you have not shared with us you know where you spend yours. Why do you spend it, where do you spend it? R4**

R4: I like R4 am saying this, like she goes to the club. You know you must use money to get money, you can’t just go to the club and just sit that you are waiting someone to come and call you. That’s when he can’t even buy you a beer. No, you buy yours and then if another person comes he sees you drinking that’s when he can call you and say, “come sit near me” and then you start talking there he can give you work. But if you just sit lonely you are using nothing you can’t get a client.

**I: So you use money to attract client, another person R10 why do you use money where you use?**

R10: I must use money because, like me I stay in a room so when am there I must use money, in the house I do stay, food and things like body jelly. Okay

**I: And what’s the reason as to why you us money there?**

R10: Because You know in room you can’t just stay you must pay for it. Yes, so you must pay because after paying you will make money from using the room. Yes

**I: Thank you so those are our personal expenses alright?**

R: Yes

**I: What about the expenses on others? Like for example what we use on children, you have told me you us some expenses on children why?**

R3: R3

**I: And approximately how much**

R3: You know all of us here, no one likes this job. We are pushed to this job for the sake of those children that we do spend money on them daily. Because if we did not have those children or parent or sibling is in school it’s a must, you want her to learn so that she won’t be like you, you see , you must use money. We could have just sited at home get married. Because now you are calculating it’s a must you look for that money there is someone who is in form four that’s your brother, your child is also there. Your mum is old, your dad died you did not even see him, will you leave her to be abused out there and you are a girl? And you have something that brings money it does not stick it does not have off days. You will look for that money so that she is not abused. Or she is not stressed out and start thinking that if her husband was still alive she could be better. I am very sure if there was nobody among us who did not have responsibilities like children, parents or any other responsibility, we could have just gotten a boyfriend who pays our rent and we could have been comfortable. But these kind of situations in life, you have to make sure you find a way on how your child will eat and every other thing so that one day if you retire, you will proudly say that “I suffered for you and where you are right now I am happy since the kind of streets life that I lived, you won’t live”. You tell yourself that your child won’t live that kind of life. Let her/him build him/herself so that she can help others though mine is done but I have brought her from somewhere she can never be like me.

**I: So normally for those of us who support our parents, how much do we roughly use? I have noticed that’s a question when I ask, none of us wants to look at me on the face.**

R3: Because it’s unanswerable

**I: Why are you saying its unanswerable R3?**

R3: Do you know why I am saying it is unanswerable, because obviously if I eat chicken here, my mother must also eat chicken I can’t tell you the cost of that chicken and my mum must also look smart while going to church and people can say that’s my mother. You may also find that in your up country you were blessed with a big piece of land and there are workers who have to be paid so we can’t say that we use 10,000/=. Personally that I can’t tell because your mother may call you today that she need money for “chama”, there is a cow that needs injection, there is a goat that ran away , you know such things you always have to send without knowing or recording because it’s not a debt, it’s not money that will be refunded.

**I: Okay**

R3: Its money that you have given to your mother so she is the one to make the decision to do whatever she wants to do with it. Whether is to pay her debts, that’s hers, provided that she had told you to get her a certain amount of money probably 1000/= “there is something that I want to do”. So you can never record all these that my mom 1000/=, 200, 50/=

**I: Okay**

R3: You can’t

**I: Okay**

R3: Personally I can’t tell

**I: What you can say,**

R3: I am okay

**I: If you have understood my question is,**

R3: Yes (noise at the background)

**I: when sending money to your children maybe you can say “I send this much”**

R3:I can’t say anything there since for me I send money daily to my house

**I: Okay**

R3: Yes

**I: Approximately how much do you send on a daily basis or may be the least that you can send**

R3: I send at least 500/=

**I: Okay**

R3: And am also paying 300/= for my room

**I: Yes**

R3: Daily, I also pay rent where my children are staying 6000/= monthly

**I: Okay thank you, thank you. R8, what are your expenses, on whom do you spend and how much is it?**

R8: Am I speak in luo

**I: Yes you can.**

R8: What I can say is that I use money daily for children, house use and my personal needs, I pay rent monthly, I pay school fees for my children (inaudible) and sometimes when sex work is not enough I usually go to the lake and vice versa so generally it’s never enough and there are no jobs as well, sometimes you will get from 300/= sex work and you have ben out all night in the cold and you have only gotten 300/=. Do you know that this money won’t be of help in the house and you can’t say that this 300/- will be enough for your usage from morning to evening so that’s why we have to have them as two jobs, you go for sex work and you also do another job because things are many and maybe your mum has also requested you to send her some little money for her to buy sugar, you will be disturbed and decide to send her at least 500/= so it’s hard on us kindly assist us (participants laughing)

**I: Okay thank you so much we are moving on well so I would like to ask, I would like to ask that normally, do women who do sex work save, do they save?**

R8: Let me answer that, how will you save, how will you save yet your budget, your rent is 6000/=, you have a kid in high school and you also have to eat. Mmh, your kids also have to eat, you also have to dress well and you don’t have that money where will you get the money to save? Give us loan, we will pay (participants laughing)

**I: That’s R8’s idea, any other person with a different idea, female sex workers**

R1: R1

**I: Yes**

R1: Its is very difficult to save. First, you find that today you will get but tomorrow you will not. It will force you to use whatever you have saved to buy food or any other thing. Secondly, it is very difficult to save since we depend on daily jobs, its like you know that the day you have gotten you over spend since you know that come the next day you will get, yea. You know that, “the next day I will obviously get around 200/= I will sort certain things”. Yeah

**I: So you have said that there are somedays that you don’t get and you use what you had saved**

R1: Yeah

**I: So, that which you had saved, when do you save it, when do you get to save?**

R1: You know is like you may feel that today you have gotten enough you have gotten around 4000/= you decide that let me save 1000/=, let me send 1000/= home, 1000/= let me get myself at least body lotion. And then you realize that you have given out this money (someone whispering) then the next day you don’t get anything you go back home with around 100/= and maybe just as I have said that normally I have to get myself a beer as I wait for a client. You will find that obviously the money that I had saved, I have to use it again so there is nothing that I have saved, yeah.

**I: Do female sex workers typically save R8?**

R8: R8, I want to apologize, I want to rush to school so could you kindly allow me go out 1700hrs I want to rush to school so that I can take this thing.

**I: Lets continue R2 (noise at the background)**

R2: We also want to apologize because we also are going to work at night

**I: Yeah**

R2: And we left home without taking breakfast and we also haven’t taken lunch

**I: Okay**

R2: And it is almost 1700hrs

R3: Okay R3 the question about us saving

**I: Okay**

R3: We only save when the money is there, mostly we save in December during the festive season but for now we can’t save because the money we make we use it to budget for everything hoping that tomorrow you will get then you don’t get tomorrow so, I can’t say that saving is easy

**I: Okay, is there a certain behavior that female sex workers who save have?**

R2: R2, Saving money for FWS is difficult because at times you will see some nice clothes or shoes and you want to buy so for female sex workers saving money is difficult

**I: It’s hard**

R2: Sometimes you want to make your hair since you have to look good

**I: R4**

R4: What I can say is you can save if you are in a chama and at some point you may not be able to pay for that chama. So when there is money we save in chama

**I: Is there another person with an another opinion? Okay to those who save because you have said you only save when there is money, what makes it easy to save?(noise at the background)**

R3: R3, You have to save because there could be an emergency anytime since you have left children back at home and your neighbor may just call you that your child just got injured so you will send the money you have saved since you cant get there on time. Your child is rushed to the hospital as you follow later.

**I: Yes**

R3: So you have to save because when you away from your loved ones like your children, your parents, you may be called anytime and some of us… some have parents with HBP (high blood pressure) they just fall down you know, before you get there you have to send money so that they can be taken to the hospital.

**I: Okay**

R3: So you have to save for emergency, which is only used for emergency since anything can happen you can’t predict (noise at the background)

**I: Okay another person, what makes it easy for us to save apart from emergency (noise at the background) mmh R10**

R10: What makes us save?

**I: Yes**

R10: In sex work you are not certain of getting so if you have saved tomorrow if you don’t get you may use your savings

**I: Yes**

R10: (in audible)

**Note taker: I am requesting us to be audible since we are recording because if it doesn’t record I may not be able to capture everything and your opinions are what we want so if you are not audible enough it won’t record what you are saying so please be audible, Okay.**

R: Yes

**I: R9, what makes it easy to save, (noise at the background)**

R: speak (noise at the background)

R9: I can’t say a thing because this job of ours, this job of ours is like gambling, if you stand along the road you can’t get money every day. There are times you can get lucky and get a lot of money and at times you can go with nothing, let’s say on a particular day you get 2000/. You can save 500 on your phone and use the remaining 1500.

I**: What challenges do you go through when you try to save? R6 Challenge**

R6: You see when you save, when you save money like I said I work in a hotel and earn 150/=and you can’t save that whole amount in a month, you can save around 100/=, so at the end month you can pay rent and remain with some cash that you can use in the house and for the kids in case of an emergency like when the kid gets sick, she can use it for treatment. She can use the remaining amount to do her own things.

**I: What are the difficulties, we’ve talked about the ease of saving. The difficulties are the challenges that am asking, what are the challenges that we encounter that make saving very difficult**

R1: R1, sometimes you find that there are no clients, so savings becomes very difficult because the earning you get is from hand to mouth, whatever you get on that day you have to use and the next day there are no clients. The little you get people at home also need your support and you also need it, the landlord also need it so it becomes very difficult to save. Yeah since we are surviving from hand to mouth

**I: R7, R6**

R6: Saving is okay that’s why we are saving in chamas because when you save in chama when you eat (a chama member gives you money) somebody’s money you’ll have to pay. When you lack or when you have a problem you can always be loaned. And I will pay that’s why I cannot have an account since my earning are minimal that’s why I save in chamas

R3: Okay R3 Challenges are there, because the income is minimal but expenditure is high so that is the challenge that we mostly encounter. What we are earning is less but the budget is three times the income so obviously savings become difficult, because you have earned 1000/- and your rent is 6000/=

**I: So how can we solve this challenge of low income and high expenditure, how can we address it, this question goes to everyone, not just R3 how can we solve it anyone who is ready can respond R4.**

R4: So you just have to struggle.

**I: Okay.**

R4: You just struggle, if you manage to get 1000/= you spend a little and save a little so that you can use it to solve any problem that may come in the future.

**I: Okay, when you say you spend a little what do you mean?**

R4: For example if you earn 1000/=, spend 300/= on your needs, 300/= for your room and save 300/=

**I: Thank you that’s one of the solutions, lack of clients is one of the reasons we don’t save, how do we solve it? (noise at the background)**

R3: R3.

**I: Yes**

R3: You don’t give up, you try the next day (laughing).

**I: Anyone with anything to add, R5?**

R5: When there are no clients today you just try tomorrow, you do your best to get clients.

**I: Okay thank you all, to all those women who don’t save I know you have said saving is very difficult because the expenditure is high and there are no clients but at least for you, you are saving. Those who don’t save completely, some of you said that sex workers cannot save, so that type, why do you think they don’t save?**

R3: There is no woman who is not saving

**I: Okay**

R3: Even if it’s just 10/= do you know why women have good priorities because a woman has to find a way to save, you make time for the people you love, you have to find a way like R4 said its hard but you have to struggle so if you were using 2000/- for your hair, you have to use 500/- because you have to save for your kid who cannot go to work. So, for saving, there is no one who doesn’t have that desire it’s something that we desire, you go be with your kids to feel like you are there for them. So you have to save even if it’s as little as 10/= or if it’s even the balances you have when you do purchases. So, it forces you to lower your lifestyle standards, if you were eating meat you will now be eating ‘omena’ so that at least you save even the little you can. Because we are in this business to save and to grow ourselves, we are not in this to have fun because you can have fun even when you are not doing this. Even in the rural place there are ”disco matangas” that you can go and dance and have fun, so we are in this to grow ourselves we are not in this just to sit. So even when it’s very tough we will go out and find a way to earn. If you are caught by the police you stay in the cells for 1 week -because we are always arrested- at times you are arrested. Friends come to visit, they ask how much you have and you say you don’t have anything and behind your back they say that when they were out looking for money in the cold weather you were sleeping so you deserve to be there even for a week so that you can learn. So you must have your money so that when we come to visit you, when the police ask you for 2000/= you can say you have 1000/= and ask your friends to add you 1000/- so that when you are released you can go and hustle (work hard and pay back). But when you don’t even have a coin, they will just leave you to stay there for a week so that you can mop at least the cash bail can reduce so that your friends can come and bail you out with 500/= so you have to save .

**I: Okay, any other person, the disadvantages of not saving**

R: The disadvantages of?

**I: Not saving**

R1: Yeah R1 you know I have forgotten what I wanted to say

**I: Sorry when you remember we shall come back to it. R9, the disadvantages of not saving**

R9: I am still thinking

**I: Okay,R5**

R5: Let’s assume… the disadvantage of not saving, let’s say you were not saving and you receive a phone call that maybe your mum or your brother has passed away yet you don’t have anything and you also have no meeting that you usually go to like these groups that could help you. And you don’t have anything, what will you do? And your home is in =Migori= yet you don’t have the fare to take you there and this is because you were not saving anything. So for people like us, we are supposed to save for example if you get 100/=, spend 50/= and save 50/=. So that we save to help us

**I: Okay, R7**

R7: We have to save, we try and save so that if you have a child and your kid falls sick, you can also fall sick until it gets to a point that you cannot go to work so you have to save so that when worse comes to worse you can find a way to help yourself even when your friends come to visit you to help you can tell them that you had a certain amount of money it makes them be motivated to help you

**I: So what are the disadvantages of not saving?**

R7: When someone is not saving, when you don’t save for example as number 3 was saying, do you allow me to say that?

**I: Yes**

R7: You may be arrested because we always get arrested, so you are arrested and your friends come to visit you like for example as we are 10 people here, these 9 people when they come to visit you, they find that there is nothing that I saved mmh , there is nothing you saved, you know it demotivates them from helping me. To add, I must save at least 10/ or 20/= so you have to save so that you find your way out during hard times

**I: Okay**

R1: Okay number 1, if you don’t save at times its difficult like when COVID 19 broke out like for us who depend on bars , the bars are closed so there is nothing that you can now depend on. Maybe you call your few clients and find they are in their houses with their wives and don’t get the time to come and see you. So at least your savings can help you hoping that maybe when COVID will be over the bars will be reopened and you will find a place to depend on (inaudible)

**I: So as you have spoken I have noticed that you have talked about the advantages and the disadvantages is there anyone who wants to add on the advantages of saving again**

R3: Okay R3

**I: Yes**

R3: Just as everyone has said here, everyone’s point is home. We stay in guest rooms here, when you fall sick that 300/-, the owner made the guest rooms go bring him money and doesn’t care if you are unwell. You have been rushed to the hospital at 2.00am and the hospital bill is 6000/=do you think the owner of the guest rooms will pay for you, your client who give you 300/- will they be there? The money that they have been giving you and you have been saving, that’s what will help you, you get. Because your client will not come to visit you at the hospital since he doesn’t even know you so the money that you have been saving that’s what you will tell your friends to help you sort, “the bill is 6000/=, I have 4000/=” someone will say the she is giving you 500, they will be motivated because you have also helped yourself. You cannot be in the hospital yet you don’t have even a single cent you get. So you must start a journey so that you meet those who are going with you on that journey so if you don’t save, you will be forced to call your relatives who when they come and find the kind of work that you do-will you take them to the guest room where you stay? You can’t so these are your sisters, these are your mothers so you finish it here, just like that.

**I: Okay, thank you so where do female sex worker typically save, normally female sex workers,**

R3: Mpesa

**I: Where do you save R3 has said mpesa mmh**

R: Phone

**I: In the phone, what does in the phone mean, like where in the phone?**

R: Mpesa, mshwari , kcb

**I: Mpesa R3 you are speaking and you haven’t said your number**

R3: Okay R3 KCB I mean the phone particularly has mshwari, mpesa, kcb, lock savings , fixed account everyone have where they save some of us we don’t know how to spend money well so you will deposit the money in your mpesa then to mshwari then to lock savings , so even when you desire to eat chicken, you cannot. Because some people don’t know how to save money. You find that someone maybe she had 1000/= but when evening comes, she has spent it all and only has been left with 200/- that’s a disadvantage so that’s why you put it in lock saving you know for lock saving you can’t withdraw up to two days. By then the desire to eat chicken will have expired, you can’t get it.

**I: Okay thank you R6, where do you save,**

R6: I save in chama and Mshwari

**I: Okay now do you have a reason for saving in chama**

R6: Chama

**I: Why do you prefer chama,**

R6: I prefer chama because if I get a problem right now they will give me a loan.

**I: Okay so I want us to talk about Jitegemee, we have been talking about Jitegemee and I explained to you about Jitegemee so I want to read for you about Jitegemee and then we will answer the questions after that on Jitegemee we are not badly off R3 I can see you checking time on your phone**

R3: eeh

**I: So, previously, we talked about the intervention of Jitegemee and I made it clear that we are doing it to ensure that women who do sex work are saving in order help them say no to unsafe sex or to take time to rest from sex work. There is one of us who said sometimes you want to go and be with your kids for one month for you have really missed them. You will take your time from sex work if you want to rest I also said that it will comprise of women who do sex work to save a certain amount of money or a little amount of money to use when there are no clients we have said that at times there are no clients, right? Or to help them to prepare for life after sex work if you decide to retire you will have saved. So I want to let you know that jitegemee is not like chama where you we take turns receiving money in rounds, like today it’s me tomorrow it’s another person. Okay you personally save your money, when you need to and in whatever amount that you need to, okay. And then you can withdraw it anytime that you want, when there is an emergency, when there are no clients or you have taken a rest, you can withdraw it, okay. There is no interest that you may say if I take it I will need to pay plus interest and then, there is no transactional fees and once you have saved it there. So there is no cost that you incur for saving with Jitegemee understood.**

R: Yes.

**I: So I want you to picture Jitegemee like that. So do you think jitegemee is something that female sex worker in Kenya can accept?**

R: Kindly repeat the question

**I: Jitegemee just as I have explained is it something that female sex workers in kenya will accept**

R: (All) yes they can

**I: What type of... I know all of you have agreed to that I would like to request that when responding we respond one by one because the recorder doesn’t know which number is talking okay**

R5: R5, Yes it can

**I: Why are you saying it will be accepted?**

R5: Because its Impact Research so I think that we are doing, I don’t know how to say it

**I: Just say with the language that you feel will express it right**

R5: The things that we do like us, that which you are saying will be accepted it’s the same thing with us we are doing here

**I: Okay**

R5: Yes

R1: Its okay because you cannot do sex work for ever there will come a time that you will be doing sex work and your kids are also doing the same thing and there will also come a time you will feel tired I should go and rest. And at least you will be going to rest if you are sure of something that you have and you may find that you were saving at least that saving will help you buy land and maybe you may have had land now you will feel that you should build your home. But you may find that you are building you home just because you had saved. You go for your savings and use it to do something.

**I: Okay, thank you. So which female sex workers do you think may accept jitegemee and why**

R: okay R3, you have said very well that sex workers. Please show me a place where sex workers are respected if not for research like =Impact=, =LVCT=, =TUUNGANE= those are the only ones that represent us. Try going out here with a short clothing and see the way you will be discriminated. So there is no sex worker who will not want to believe and join Jitegemee because that’s the source that knows and respects you and it understands you. So there is no sex worker that will want to miss this opportunity.

**I: Okay thank you mmh is there anyone who wants to add on that which type of sex workers will accept is that question understood**

R: Yes

**I: Do we understand the question**

R3: The type of sex worker do you mean the ladies, gay or who

**I: Women who do sex work**

R3: Women so its es sex workers like I have said that there is no one who will hear about this and miss because you know us. Just as you have called us here and we have come.

**I: That’s R3s opinion and I want everyone’s opinion that’s why we are here as a group otherwise I could have called her individually and talked to R3 alone but we are in a group lets add or subtract from R3s opinion we agree, we disagree or we give out different ideas that’s why this is a group discussion and again we wanna finish up (someone talking in the background) R4 do you want to add something. R4 has denied by shaking her head, should we ask that question in luo**

R: Yes

**I: We ask it in luo so that we understand it well okay, let me read luo (silence) what type of women will like it and why? Participants laughing**

R: I haven’t even heard what you have said (participants laughing)

R: Give it to me so that I can read it which question was it? (noise at the background) which type of women who do sex work will like it and why. Heh whoever wrote this luo.. who is perfect in reading luo to read it for us?

**I: lilian is back, let her help us**

**I: Which type of female sex worker will like it and why, that would love jitegemee that means that if you take a look at jitegemee, which type of women will like it, which women will like it those who do sex work?**

R: R1 more so those with kids will like it because they see that if they save, at the end of the year at the end of the month at the end at the week at least they have resources somewhere.

R: R7 (inaudible) you may like it when you are married even when you are not married. You are not and you have kids, you know you can’t leave your kids so you have to send them money so it forces you to join so that your kids can eat and go to school and live well. So it forces us to save

R1: Each and every one of us have a dream so you may be doing sex work when you know at the end if sex work, I’ll have this in life (children playing in the background)so you may live savings you are seeing that in future you’ll have something to help you and only if you have savings

**I: Okay what type will not accept it and why R3 said that all the sex workers will accept it and I said that that’s her idea and I also said that everyone should have their opinion. Which type do you think will not accept it. Are there those who will not accept it**

R1: Yes we all differ so you may find that someone is doing sex work and she is married so she is here doing sex work and has left the husband at home taking care everything, she will never think of saying “let me save” because she knows that at the end of the day the husband will be with the children taking care of them. She spends yeah she will not have the mind of saving

**I: Okay that’s R1s opinion, another opinion**

R4: My opinion is, I don’t think there is a woman who can forget her child, you have to save some money and send to your neighbor for your kids upkeep even if you don’t get along with your husband,

**I: Another opinion, Number 1**

R1: There are those who have lost hope with their lives, for example the ones living with HIV so she thinks that after all she’ll die, then people mock her by saying “she’s living a bonus live” therefore she decides not to save because she’ll soon die.

R3: Back to Number 1’s point, everyone loses hope even if they are not HIV +ve, even me I have lost hope; if you lack money you’ll lose hope. You don’t have to be sick to lose hope, even lacking money can make you lose hope, especially when need arise and you don’t have money, for example rent, torn uniforms and shoes for kids, can make you tempt committing suicide. So anyone can lose hope, especially in female sex work. Therefore Jitegemee program will help us share with you our problems and we will be available all the time and ready to walk with you.

**I: Thank you for your opinion, and for those women who work as commercial sex workers that you know, which area do you think will welcome jitegemee?**

**I: Out of ten female sex workers you meet, how many do you think will join jitegemee?**

R10: I need to think.

**I: R9 How many do you think will join?**

R9: If they are sex workers, they will all join.

**I: If they are all sex worker they will join, Number 7? how many do you think will join?**

R7: All of them

R1: All of them, unless the one who does not have future dream.

**I: R 9**

R4: Maybe 7 of them will join, because some of them have low understanding.

**I: Any other opinion?**

R1: May be few will join, because some will say that they earn little and can’t afford saving.

**I: Out of ten how many?**

R1: I think 4 of them.

**I: What can we do to mobilize more sex workers to join Jitegemee?**

R 5: Adding funds.

**I: What do you mean?**

R1: If you add some percentage on the savings, so that the savings increases even if its weekly it will attract more sex workers. In that your savings earns you more interest.

**I: Number 2, what can we do to make more sex workers join Jitegemee?**

R 2: I have no opinion.

**I: Thank you, Number 3, what can we do to make more sex workers join Jitegemee?**

R1: You can do nothing; it depends on someone’s interest.

**I: What can we implement to make Jitegemee successful**

R3: Has jitegemee been there for long?

**I: Jitegemee has not been there, its new.**

R3: Then continue holding meetings! Which will enable us mobilize others, and with time you will get followers.

**I: Any other thing that we can do? number 1, is this meeting made for Numbers 1,3 and 5 because the rest of you are not participating and number 4 is also trying and I also want other numbers to try, I know you have suggestions.**

R1: I suggest that you do empowering women.

**I: Like?**

R 1: Grouping women in 4 or 5 then giving loans which are returned with low interest rates in order to make them save, that will attract many.

**I: R 6**

R6: no suggestion

**I: Ok, thank you.**

**I: What components should jitegemee have to be accepted by many**

R1: Savings with interests people will accept

**I: We’ve talked about that.**

R1: Women empowerment

**I: Also talk about things that will discourage them from joining , you know it’s from here that we are forming jitegemee, so when we bring it will be having your opinions and if you don’t talk, we won’t make as jitegemee. That’s why we are here**

R1: If you put target on savings

R3: What do you offer us in exchange of our commitment to jitegemee? For example in Tuungane we are given Lubricants, CDs Free survical screening etc and what about you?

**I: Do you have a specific thing in mind? Because that specific thing will help us to know whether Jitegemee is in a position to give this or not**

R3: Jitegemee should not be like the =LVCT= thing, you people should come up with something different from what we have, because in Tuungane, if we are Molested by our Clients they stand with us by giving us Lawyers and police, so you should come up with something different, that other organizations are not offering.

**I: And that’s why we are asking what that gap that Jitegemee can come and fill is?**

R1: Point of correction to Number3, that’s why I came up with a point of women empowerment, Loan of low interest, which will make it look unique.

**I: Thank you Number5**

R5: giving its members loans

**I: Thank you. What don’t commercial sex workers like about Jitegemee?**

R1: Jitegemee has given us only one reason, which is saving with them, so what help are we getting from them by saving with them? all we need your thoughts, that’s why we came you

R4: Jitegemee has only brought us the saving agenda but they are not telling us what we will get back after saving with them , because we used to save before they came for example in m-shwari, I save and they give me loans. Jitegemee is not like other groups that give loans, it’s your own money that you save and take, so its upon you to set your limit, you can save the way you want and withdraw the way you want.

R2: We were saving money even before jitegemee so when jutegemee comes I think you should give us a loan that can help us because initially we were saving however little it was we were still saving so if you could give us loans

**I: So lets go back there is a point that we want, there is a point that say that jitegemee will be different from chama. So jitegemee will be that your savings is what you will see on how to save weather you like saving daily,weekly, monthly so you are the one to decide and put your own target that’s first, second there will be nothing like you have to pay with an interest because its your money and and you are the one to budget with it since there is a thing that you are focusing on that’s why you are saving it right so you are the one to decide since its your money. You just save the way you want and you withdraw the way you want . so with your opinion tell us what do you think female sex worker not like about jitegemee**

R3: You as jitegemee you have given us only one reason for joining, but we want more than one reason to join you, which is about chama is it just about savings, what else have you told us that and we have denied, you have asked us if people can join we have told you people can join because you care for us so if you bring us this thing we will join so is there any other thing that you have told us, you haven’t so there is nothing that we can say that jitegemee since we don’t have options

**I: Give us another opinion**

R3: I don’t have any other opinion since you haven’t given us options

**I: We have said it’s when we are planning to start we haven’t started**

R3: We cannot say no to something we don’t know so you cant ask us what we would not like yet you haven’t told us, so you should tell us what you want to bring so that we can tell you if it will work or not. You have told us about chama and you have told us that there will be no interest like chama so you haven’t given us the next point so we can’t say that we don’t want something you haven’t told us

**I: Okay is there anyone who want to speak**

R: What I am saying is that we have talked about savings and you have asked us about

**I: We have taken 1hr 30 mins, and I have just restarted the recorder now, it was on pause**

R: Time is not on our side you will just have to take 1hr 30 minutes because we haven’t prepared ourselves

**I: In that case lets give our opinions faster so that by 1800hrs we are done**

R: So when we are talking about this you know there is no way we can answer you since we have no idea what it is yet we have already accepted because for people to join or for us to mobilize other people we will tell people that this thing has just started and this is what it entails so that they can join us so there is no way we can answer you because you haven’t told us what it is

**I: Okay**

R: Okay so that when I will be mobilizing people, I have something to tell them because we are many, these are not all of us. So when explaining to someone you have to know the details, because we have agreed that we save and that means that if we agree we can decide that monthly or weekly depending on our agreement so we just want to hear from you what it entails

**I: Okay we have heard that, meaning when you go to mobilize people we have to tell you what we want to do so that we can get to know if female sex workers will like it**

R: Mmh (yes)

**I: Okay so what do you think there could be human rights issues**,

R3: That’s a must

**I: Let me finish reading the question, things that will make people feel like “if I join Jitegemee my human rights will being abused with”. Maybe the way they say this is abusing my rights. Is it there? R3**

R3: Eeh

**I: How?**

R3: You know being a sex worker is a decisions and we made a decision and we a PHD so there is nothing new to us and there is no way you will abuse us and you want to help us, this whole time you have been educating us here you haven’t abused our rights. You are telling us how tomorrow will be because when we grow old the young ones take over so you guys are helping us so there is no way you will abuse us. In the near future I want to meet my fellows here in a better place not the same same state we are in no. so you are heling us and you haven’t discriminated us. So we don’t have any problem with you guys

**I: Another opinion, is there another opinion,**

R4: I am requesting that when you come back please make it a better time because this time is not good with us since we don’t sleep at night, we sleep during the day so if you could make it by around 1000hrs to 1300hrs will be better

**I: Okay we have heard you and we will work on that. Okay what are the challenges that we may encounter when we bring jitegemee to the female sex workers community. I think we all want to leave here so lets speak up.**

R: There is no answer

R3: You will not encounter any challenges because nobody care about us, so you are the people so there is no way you will have any challenge. Because the outside world will not know what we are doing, this thing is private right, why are you guys recording and there is no camera, its because it is private so there is no way it will get to the public and we get offended.

**I: Okay so what amount of money are female sex workers willing to save per week with jitegemee**

R3: I would like to take you back, we cannot predict that we will give 2000/= because we are different there is one who can get 10 and another 5 and there is 200/-so we cant predict since we are all different. There are those who hustle online and they earn good money like 30000/= and they save around 5000/= weekly and as for me in the streets I can only save 1000/= weekly so we are all different and whatever we get is what we will save .

**I: And that is the beauty of Jitegemee, which you can save according to your capability like we said and at the time that you can, no one will tell you that every week or every day. As a person we all know the shoes that fits us, so you will decide according to your shoe size, you know how much you can give.**

R1: As number one like I said earlier sometimes there is no work making it difficult to predict that am going to save 1000/= daily because sometimes you get 6000/= and you decide to save 1000/=, 1500/= 0r even 2000/=, you again do it tomorrow where the work is so dry until you tell a motorbike person to just carry you and you will pay him later so we can’t say that I will save this much a day or a week.

**I: Yes, now I would like you to tell me how you may want this money to be kept, in which way will women who do sex work believe the money is well kept that it is secured and safe?**

R1: I told you very well that we form a group and create an account because we can’t trust any person with our money that we have chosen Njoki and now Njoki is the one receiving our money.

**I: We don’t have anyone by the name Njoki here, right?**

R1: I said that as an example.

**I: Am asking so that the recorder records that we don’t have someone by the name.**

R: (jokingly) she is called Njoki (laughing in the background)

R: Sorry

**I: Okay number one continue.**

R1: Like I said that we can open a bank account because we can’t trust anyone with our money and some of us are mobile working in different places, like today she might be in Bondo, tomorrow she has been called in Siaya another day after tomorrow someone tells her that the sex work business is good in Busia and then she goes.

**I: Yes, number two this money, where do we keep it? A place where you will trust that “my money is safe”**

R2: You know what, you can’t save money because you might not get work for almost two days or what you get is just enough for room rent and food.

**I: Am asking, someone has kept some money or you get even a little, where would you prefer it to kept, somewhere where you will not feel like these people will steal the little money I have.**

R2: The little money that I get I can keep it in Mpesa.

**I: Yes, number four.**

R4: As number four this money we can open an account and save it there.

**I: Number five.**

R5: Open an account and keep it there.

R3: And account should be yours from Jitegemee but not amongst ourselves here. It should be open by Jitegemee staffs and I can access it with my secret pin but the account is in Jitegemee.

R: (All) Yes

**I: This are the things we wanted you to tell us.**

R3: I can only withdraw the money that has my name like let me withdraw even 200/= but the people having the account are Jitegemee staffs, we will not have doubts that someone is stealing from us. It’s just you Jitegemee.

**I: Yes, number five?**

R5: As number five the money I will keep in a bank account, let me ask, that if I want to withdraw the money does it have some savings on top or as Jitegemee are you adding us some money on top of what I have?

**I: Or how will it be?**

R5: Yes

**I: Now those are the thoughts I want you people to give us, those are suggestions we are going to think about.**

R1: As number one I would like you to open for us an account and it should be Tran Saharan trade, you understand if I say trans-Saharan trade? So that at least, the money is trading and we can get something on top of what we have saved.

**I: Okay now, I would like to understand for us women who do sex work we have our own savings goals right? What pushes us to make sure that we get to meet our savings goals because maybe you don’t see yourself meeting the goal? What do you always do to push and get that goal?**

R3: I have a goal and maybe I want to reach 30/= everything comes at its own time, even if you go working and give out the bend over style if you don’t have that luck, you will get nothing out of it. This risk we always say that it comes from God, you can plan to get 30 and maybe he planned to give you one million so it’s up to God to decide. Because we don’t get money due to our strength but through God so we can’t say that this month you can predict that you will get 30000/= and buy a dryer to open up a business, we just pray and let God give you life to work and get that money. I always see that it’s not by me but by him to bless you and make you get that 30000/= because if he decides not to give you no matter what you do even if you shaking you “bambam” (your bottoms) he will not give you.

**I: What do you mean by shake your “bambam”?**

R3: Shaking what your mother gave you.

**I: Eeeh (participants laughing) anyone who wants to add on something, you have a goal that you want to meet, what do you do? I don’t want to go with number three’s opinion only. You have a goal and you trying so hard to meet this goal of 50 but you are not reaching the goal, what do we always do, what do people normally do? Number three has mention that its Gods plan and it will happen at the right time and there is nothing you can do about it, other people?**

R4: Me as number four I support her because sometimes when we go to the field from 7.00 in the evening till 4.00 in the morning without getting anything or maybe from 7.00 in the evening up to 9.00 pm and you only get 2000/=. Mostly its according God’s plan if you “kismet”, you will get something that night if you are unlucky, you won’t get anything.

**I: What do you mean by “kismet”?**

R4: You have never had luck where if somebody comes you are just shining and its only you they will be picking all the time until other start complain that you go for a leave at least so they can get, that its only you being picked. That you leave for a while so that they can get clients.

**I: Eeeeh number two you want to add on something?**

R2: No.

**I: Or you are just supporting her point?**

R2: Yes

**I: Okay, we are doing so great and we are about to finish. Do women who do sex work typically live beyond their means?**

R: Living as in?

**I: Living beyond your means, you get less money and spend it more than, standard of living is high.**

R3: As number three, there is no sex worker who doesn’t love herself, even if she carries vegetables, she will hide it in carrier bag so that you don’t get to see and fry onions for you to smell. The sex work has made us become weak that even washing clothes is a problem and we only do for the sake of the children and they are yours. We are living the life of celebrities like Vera sidika and Akothe, Akothes living style.

**I: This Akothes lifestyle, how is it?**

R3: That life where clients are coming and taking us at Pride Hotel swimming where you forget your problems for a while and when you are back at =Pap-onditi= standing but when you get you make good use of the moment because it comes once. A sponsor comes and carry you at the front sit even if you don’t have a nice face to sit in front, you tie yourself well since you are taken to Pride hotel, being asked what to eat you have money the wallet is full. You must have fun since it is a one-time thing since they don’t always take numbers the one who ask for numbers are those that give 200 not for those who give a thousand and above. Sometimes they even give wrong numbers that if you call it doesn’t go through.

**I: Now I want to know...**

R1: Number one, as a sex worker you must just live a good life being that men grade according to how you carry out yourself. You find someone hitting on you and you start going out with them, there are some clients, before I gave birth to my child, I used to take clients to my house, you just look at a client and know that this can give you a lot of money. For him to give me money I decide to take him to my house so that he can see the kind of living standard I do live so that he can be surprised whether I am the girl he has come with from the club till he asks if this is really your house. Tomorrow if he wakes up and decide to live, he will give something meaningful depending on your living standard.

**I: Yes, is there someone with something to add on?**

R3: Another one is we are getting money through hardship…

**I: Number three.**

R3: Yes, number three, before you get a thousand shillings you must buy something and appreciate yourself even a soda because that 1000/= shillings no man will give just for singing a lullaby song for them, you will have to work hard for it. Sometimes we go blindly not knowing what the man has, we just go because of money, you have to live a good life.

**I: Aaaah, so is there……**

R1: Number one.

**I: Yes.**

R4: (participants laughing) Number three for those at the club you can go even at 11;00 or 12:00 but us who are at the field you have to be there by 7:00 in the evening, when it reaches that 11: 00 pm there is no client that is coming and everyone is at the bar. Kindly compete with time even if you are asking question please consider us, it’s getting late the rest we will continue later we will still come back.

**I: Let her say that last one so that we don’t cut her out.**

R: (participants complaining) This are people who are working in hotels, some of us have not taken anything since morning, others were called from the bed so please lets us finish.

**I: Okay then let us summaries very fast, we can’t leave it at half.**

R: Why can’t you leave others and continue next time?

**I: This is the only chance I have to work on it next time I will come with a different thing.**

R: You mean you are still flipping on another page?

**I: Its just this alone, and we are doing the next question.**

R: All that, please use the Pythagoras theorem?

**I: (All laughing) Okay then, now what can we do to better our lives so that the life we are living and the money we are getting be the same, what can we do to bridge the gap?**

R3: Like me number three we are used to such kind of life, standards are high and money is less but we survive because there is no one who doesn’t put on expensive clothes worth 3000/= we all do. No one here not using 2000/= on a hairstyle we all use and that money is not available. We know how we fix the money, even if you get nothing today, tomorrow and one week we will not miss going to the saloon that we know. The kids will have something to eat with the cooking oil they are used to. We can decrease that life because we are used to it. If there are people who are looked down upon are single ladies at the plots without a husband, you will not allow that, when you reach the house you open your music and play loud music by the name tera mos (take me slowly), we must live that life we can’t go down.

**I: Is there anyone with a different opinion or anything else to add on?**

R: None

**I: Okay women who do sex work, do you borrow money, take loans?**

R3: It’s a tick, we always borrow even from friends like hello can you lend me 1000/= I will refund it on weekend, sometimes you may run short of something or I may find number seven and ask her what is the form tell her kindly buy me lunch and then I will sort you. Even if they are not loans from the bank.

**I: What do you mean by the term form?**

R3: You know I am a ghetto child, it’s a sheng like what can we do? Sort me today so that we can see what to do tomorrow.

**I: Most of the time when you request for this money, what do you always do with it?**

R3: Sometimes I am hungry and I can’t work without money, maybe I got money and sent everything to kids, so I will tell number 10 to kindly sort me out and then I will refund in the evening. We always help each other leave alone issues with banks because we all lack that money even pastors do lack why not us.

**I: How do you pay it back?**

R3: We get clients who will give you a short “chapa njoti” and use that to refund it. (Participants laughing)

**I: Chapa njoti………. (Participants laughing)**

R3: Giving me one short

**I: Oooh Short…Most women on sex work what do they always do increase their income?**

R2: You give all types of styles, touching my breast is 500 and me touching his manhood of another is another different price 600/= or 300/=, yeah.

**I: Okay**

R2: When you get to the room...

R: We said you take a little time explaining.

**I: Just let her finish first.**

R2: IF you get into the room, the client has to pay first and after paying it’s when I remove my pant put on a condom and continue with our business. And if he is the room and he doesn’t want to go on to the business he will have to because he has been in my room.

**I:Okay, any other person, what else do you do to make money number four, number nine? (silence) we are talking about what else you are doing.**

R9: For me I have a saloon, when I don’t go to the bar, I can get a client and make their hair.

**I: What else are we doing, number five?**

R5: If I want to get money, I must talk to a client well because if you don’t talk well he won’t leave you with good money, so before I need to talk well then go the room and give him other styles so that he can give you enough money.

**I: Okay..**

R1: Number one.

**I: Let number seven speak first.**

R7: If you go with someone then before you go you have to agree first, then after the agreement he finds that your job is good, you have a style that he likes and you as well so he will be satisfied by you giving him what he likes making him to give you more money. You shaken him very well.

**I: Number one.**

R1: Number one, it is something obvious you have to make up and groom yourself because men like what is glowing.

**I: Yes, so you have to groom yourself?**

R1: Yes.

**I: Apart from sex work do we have other source of income? number nine said she is a saloonist, number seven said she is working in a hotel, the rest that is the only source and it’s the main one?**

R: (All) yes.

**I: Okay thank you all, what is the amount a sex worker may have in debt at any given time?**

R4: About debt you never say, you can find yourself in debts that you dint plan for worth 5000/= for clothes only.

R: (from the background) Billboard of Kenya

R4: Clothes and shoes.

**I: Yes, number two, debts.**

R2: I can’t talk about debts.

**I: Why?**

R2: I can have even a debt of 10000/=.

**I: Yes, number six?**

R6: Everyone must have debt because you can find you don’t have debt and someone brings you a nice thing and you tell them to just leave it with you so that you pay later. Things like clothes and shoes.

**I: Yes, at any given how much debt can you have?**

R6: 5000/ = OR 4000/=

**I: Okay 5000/= or 4000/=thank you very much, do you always think at one given time you will sex work, number four?**

R4: Like me number four am just praying one day God should remove me from this work because one, it destroys our body. (Laughing in the background)

R3: Me as number three I will support her, this job has destroyed my body, do you see those old women who walk with sticks while bending, we are the ones. Our body has been placed in so many positions and angles because of a thousand shillings, bending style like you are flying. This job we should retire, even if we say we retire it is okay because sometimes you are bending and you feel like your back is breaking. It affects the body and you get money but the money doesn’t help that much. Sometimes you get a very big manhood and you wonder that it’s going to get in your womanhood, so this work is tough. Second if you go home this work you can’t even tell your mother that you are doing this kind of work, you can’t even tell your sister, you can’t tell it to anyone that you are doing sex work. This work is hard especially staying in the cold it’s hard.

**I: Now do we talk about it at the field where we are together as sex worker?**

R7: Someone like me had an operation less than two years ago, in July that is when I have two years after operation, with someone who has undergone operation with this type of work it’s hard. You will get a client because you need money who will want to position you the way they want since they have no idea about your condition, you see am suffering and I would really love to leave sex work. If I can get any other help that would be a great thing for me.

R3: Adding on that as number three if I can add on what number seven has said, we always get assaulted and miss treated. Someone will just be smiling at you when he wants to do something that you don’t want like removing the condom and you don’t want, he will beat you. Obviously, a woman you can’t measure yourself with a man, men got strength no matter how thin they are so we get abused. We are just enduring all that so no one wants to remain in this sex work we all want to be out even if it is getting married. Men are scarce so even if we are five in one man we share.

**I: Is this the conversation you always have at the field where…..**

R3: Sometimes you people as number three you should come at night not during the day when we are all there and get the stories. Some of us are not giving the stories because they haven’t tasted a sip of alcohol

**I: What is always the source of this conversation that always makes you start thinking that I want stop doing this work?**

R3: Sometimes when we are broke that is when you cry asking yourself how will you leave the sex work job, getting into it is very great especially for the first and second week you are still fresh and the male clients really know how to identify the so called new stock until they have a full taste of you they will be just passing by from a distance and taking other new people. They always go with new people that is why we keep on migrating from one place to another.

**I: Okay, another person, what always brings that conversation, we are almost done (silence). Okay, for women who do sex work at what age do they leave sex work?**

R4: As number four this sex work you can leave when you are even at the age of 50 years, because we have others who are at 60 years but are still on sex work others are age 20 and doing sex work. Its according to someone to decide.

**I: We have talked about personal reasons like getting a big manhood, the back ache what are other reasons apart from personal reasons?**

R3: As number three, other reason why we leave sex work is because of the children when they have reached an age that you can’t go to the streets. You may go to the bar with a young person of your child’s age and you find your child with someone of the age of his/her father, how shameful can that be? It reaches a point where someone says their child is almost in form four, they decide to go sell small fish at the door so that you give your child the space to have his/her fun not that you get to meet them at the reception while you are going in the room. There is a certain age when your child gets there you just have to retire by force even if you don’t want to, the shame because you don’t want to show the child the kind of work you have been doing. Maybe he is a boy and he wants to walk all over the (chuoms) coners with his (mbogi) group mates and you cant let him find you there.

**I: What is mbogi?**

R3: Group.

**I: What is chuom?**

R3: Corners

**I: Oooh okay.**

R3: He will be coming back and seeing you standing on the streets the way we usually just stand there, even the vehicles passing you never know its your grandmother passing inside. You see your boy is now grown and he wants to go to jam session things like that, someone can literally tell him there is your mother and that will be a shame so whether you like it or not you will have to retire.

**I: Another reason, number nine? (silence) none?**

R: Yes,

**I: Okay, do people retire at the time they have decide to retire?**

R4: As number four you must look for money first before you retire, you can’t retire on an empty hand or a sponsor.

**I: Okay, does that have a time limit like a time span?**

R: Like we are in May you might decide that if you reach December, you stop sex work.

R3: It cannot be up to December maybe you find a serious person in August and that is how end up leaving, we also have those who get in sex work the same year they get a serious guy and that is how they leave sex work. We also have those who die on this job, those who get old so everyone with there own plans. We also have those who say they will leave when they have white hair meaning when they are old, we also have those who say they can’t be here till they reach 38 to 40 years so everyone with their own thoughts, they are always saving even 10- 10 and they will never tell you. You will one day just realize she left as the saying goes big minds don’t tell people unlike us who like talking, we will just be talking and others you cannot talk about.

**I: Okay number two add on something (silence) you were just agreeing with what she was saying?**

R2: Its like that.

**I: You agree, okay, after retiring from sex work, what do people normally do?**

R: We do eat stock, bonus (laughing) but do we really have stocks,

**I: Number four.**

R3: As number three who doesn’t…..

**I: Don’t say where you are coming from.**

R3: I can’t even say.

R: I will give you a piece of land.

R3: Let me tell you number four, there is nothing bad as retiring and you still have a landlord, its very tricky, when it reaches a weekend and you here some music, something is just telling you to carry some three condoms and go look for rent. (Laughing from the background)

R4: You try and build your own house even if it is a small one.

**I: Number ten your opinion.**

R4: You will just be saying dear God only today and then the devil gives you with a client who pays 10000/= then tomorrow you are back again with your heels then people will be like number4 we thought you left sex work, it’s a rewind, tuition. (Laughing from the background)

**I: Number four has told us that, if you see me standing it means we are almost done, number four has said that she will be rewind and tuition, number six five.**

R5: If I retire, I am going to do farming, plant vegetables that will be helping me.

**I: Another person (silence), those who have retired, what do they normally do? Number seven**

R7: We can continue with business.

**I: Business like which one?**

R7: Selling small fish or vegetables or even fry nuts or cook porridge and hawk together with sweet potatoes.

**I: Those who retire, where do they always go, where do they go to spend their retirement? Number four**.

R4: At home.

**I: Home.**

R4: If you retire you go home in the rural.

**I: At the rural, yeah.**

R3: Wait number four, for us who are not married, which home will we go to?

R4: It will force you to buy land and build your house.

R3: You got a point there.

**I: Number six, when someone retires, where do they always go?**

R6: That will depend if you have a business set aside.

**I: You can start the business there.**

R6: Because most of us people always say as in after getting that money, I build my own house, then next month or next year I move from here and open my bale of clothes and sell because no one will go and just sit home after retirement.

**I: Yes, so the bale, do you open while here or somewhere else?**

R6: You can even start from here; you just do your business and during the day you go and sell that is when you go.

**I: Yes, for those who have retired, how is different is their life, according to how they are right now?**

R3: As number three, there are two types of retirements, one we have the retirement where someone has truly retired and then there is another where a person is half retired, the person doesn’t miss being at the bar over the weekend, that is not retirement. Retirement is like my fellows have mentioned earlier, going home. You have nothing that you haven’t done yet, you have been to all cars including Prado, so you just go home and stay there and take care of your life. If God blesses you with a husband get married or if not let your children feed you because now you will end at the age of being a reject. Men do like young women, none of them would want an older woman, and there is an age that will force you to just not be in sex work again.

**I: Okay we have talked about those who retired and came back, I want you to tell us the positive this they always get when they come back.**

R3: Abuses, we will just abuse them.

**I: Is an abuse a positive thing?**

R3: Positive, I heard negative.

**I: Okay let’s talk about negatives then.**

R3: They are always discriminated, like you left thinking you will not comeback, you are now back why. In case some was in Bondo it will force you to go to Mombasa so that you don’t go to the same place you were because you already told people that you have left and no you are back since situation has forced you so you will have to change town.

**I: You change town to retire and then start a fresh in another town.**

R3: You must start in another town because even if you try selling clothes like number six has mentioned people from boda boda will be abusing you that a sex worker today you are selling clothes, you no longer get clients and it will force you to look for something else.

**I: Okay, women who do sex work, what would they want to fulfill before retiring?**

R3: As number three, everyone with what they want.

**I: Like you, what would you like to fulfill?**

R3: Me (laughing) that’s mine.

**I: You don’t want to say, what about any of your friends, what have they said they want to fulfill?**

R3: Everyone with there own goal, its always a secret, maybe am in sex work and I got a plot am building I can’t talk about it, everyone is here on their job if she has given birth, she is educating a child so that if the child is in university she can now retire. Everyone with their own plans and they can’t mention in public, its private.

**I: Mmmh, number six you don’t want to say, number five say something… Now my last question, do you know anyone who has left sex work in last five to ten years ago, do we know anyone who has retired from sex work in the last five years, number seven, from five to ten years, you don’t know?**

R: (inaudible)

**I: Number one do you know?**

R3: Wait, have you said a period of five years or even one, two…

**I: Yes just tell me whichever.**

R3: Yes there is.

**I: Tell me.**

R3: She is called Njoro.

**I: Don’t mention.**

R3: But she is no longer there, God willing she left.

**I: How is her life right now.**

R3: She went far away and no one knows where she is.

**I: Oooh.**

R4: But she built her house…..

R3: She bought land and hustle a little to get business money where she moved away completely and she has never called to ask how the field is doing.

**I: Are there challenges that they faced for leaving sex work?**

R3: She is well since she is not paying rent.

**I: Oooh she is okay, is there anyone that left and her life is different from hers, yes number one?**

R1: Yes, there is someone I know who left sex work and her life is different, when she left and returned home, she is just at home.

**I: How is her life?**

R4: As number four, there is one I know and her life isn’t better, she is thinking of coming back to sex work (participants laughing) I mean she is struggling in life.

R3: Her life downgraded yet we knew her as someone who was slaying meaning dresses and carry out herself well, she smells good after using her nice perfume.

**I: Are you talking about the same person number four is talking about?**

R4: She is helping me.

R3: Yes, I know her (all laughing).

**I: Thank you so much everyone, I can’t thank you enough, I know there are people here who should be at the streets by 7.00 in the evening working and they are here they haven’t taken a bath, they haven’t eaten. Others did not even eat even breakfast and you waited for us. That humbles us a lot and shows how you have respected us; we are so great full and we appreciate and thank you so much may God bless you. Is there anyone with an issue?**

R3: Am asking is there paying over time because I can see time has really gone.

**I: It has not yet passed that is why am finishing up, it’s the same 2hrs that we said.**

R: Its has passed with 6-7

**I: Thank you very much and may God bless you and you continue growing.**

R: Same to you may God bless you and you continue to grow.

**END**
