# Supplementary material for: Eat a little and save a little: A qualitative exploration of acceptability of a potential savings intervention to reduce HIV risk among female sex workers in Western Kenya
Source: PLoS One. 2024 Dec 19;19(12):e0310540. doi: 10.1371/journal.pone.0310540 (PMC11658496; doi:10.1371/journal.pone.0310540)
Supplement: S1 File — (ZIP) [file pone.0310540.s001.zip › Jitegemee Transcripts and Dissemination Notes for Journal/FGD V.docx]

**DATE OF INTERVIEW: 06/MAY/2022**

**MODERATOR: NANCY OUNDA**

**NOTE TAKER: OLIVIA OKUMU**

**FGD ID: FGD V**

**VENUE: UKWALA UGENYA SUB-COUNTY**

**CATEGORY: ABOVE 30 YEARS, PERI- URBAN**

**I: Okay this is FGD V done at done at =Ukwala= in =Ugenya= sub county the date today is 6^th^ May 2022, the moderator is [mentions name] and moderator [Mentions name] Okay depending on how we had talked about Jitegemee what comes in your mind? What comes in your mind we had talked the way I had explained about jitegemee what comes in your mind?**

PV03: Jitegemee is on how you can fend for yourself. Now Jitegemee is someone takes care of herself without relying on someone to give her money.

**I: That is number 3 another person?**

PV07: as number 7, I want to add saying that what comes into my mind is about saving..

**I: Saving, another person?**

PV05: As number 5, I understand Jitegemee like you can survive without having to engage in sex so as to be paid.

**I: Mmh, another person with a response? Number 8**

PV08: Kujitegemea is you depend on yourself you can rely on your own without getting support from any person, even if you had a partner and he wants to force you to engage in sex with him without protection, you know very well that he can’t force you because even if he doesn’t give you money you are still able to take care of yourself.

**I: Another person?**

PV05: As number 5 I want to add saying, jitegemee makes your chances of getting HIV to reduce so you can’t get it.

**I: Another person, number 4?**

PV04: As number 4 Jitegemee I feel it is good because you can’t be infected with HIV because you depend on your own and you don’t depend on someone, you can do your own things without looking for someone else. That’s my opinion.

**I: Mmh, number 3?**

PV03: As number 3 to add something kujitegemea can make me to reduce HIV incidences to be low now we don’t increase the number of HIV cases. We can reduce it by depending on our own.

**I: Who is remaining who has not given me her opinion?**

PV09: I am number 9

**I: Number 9**

PV09: Kujitegemea is being someone who does not depend on others in terms of her future.

**I: Number 1 and number 2 you have not spoken, what have you heard about Jitegemee number 2?**

PV02: It is what they have said

**I: What have they said?**

PV02: Doing your things on your own

**I: Number 1.**

PV01: jitegemee you do not need support from anyone you do your own things on your own

**I: Is there anyone with something to add?**

P: Yes.

**I: Aah**.

PV05: As number 5 to add when you are in jitegemee when you go to the field [sex work] and comes back empty handed your children will not lack food.

**I: Mmh,**

PV05: They will have food.

**I: Number 4 has something.**

PV04: Jitegemee the way I see it is good because even if you go to the field [sex work] there are some people who will tell you that they are going to send you money in your phone and end up not sending and now if you depend on your own since you had something in your account, you can withdraw the money and feed your children. I feel it is good.

**I: Okay we are moving on, we want to look at the things you buy with your money and the total cost, which means that we want to look at the daily expenses after that we will look at the expenses done once a week, once a month and once a year. Now each and everyone is going will tell me the daily expenses right?**

P: Mmh.

**I: You tell me it is this and the amount it cost, right**

P: Mmh.

**I: Who is starting? Number…**

PV09: number 9, it is food,

**I: Mmh.**

PV09: It forces me to look for money that is flour, I have to look for 150/= to get 1 tin of maize,

**I: Mmh and what else, what are the things that you buy daily?**

PV09: It is only food and also find a way on how my children can have breakfast.

**I: How much is the total?**

PV09: It can cost like 300/=

**I: 300/= number 8**

PV08: We are talking about foodstuff?

**I: No, anything that you use money to buy daily, not only foodstuff it includes everything that you buy. The things that you buy daily mmh.**

PV08: what I buy daily is soap, because you want to wash children’s clothes that is a must. You need sugar they have to take tea, you have to look things needed for breakfast that you have to buy for them by evening when you calculate the total you will that you have used around 500/= if I don’t keep this money then there is no way out so it forces me whatever little amount I get I save because you know the amount you spend from morning to evening.

**I: Mmh, another person?**

PV05: As number 5, what I buy daily is food, food from morning, lunch until supper, now in a day it depends at times I can come from the field when I have money and when you have money you say that these children have eaten *omena* for several days let them today eat meat but if you get enough money then you will have to squeeze your budget to fit your expenses for the day.so it depends

**I: So how much can you use in a day?**

PV09: In a day if I get good money I can spend 500/=

**I: 500/= another person? Yes number 7**

PV07: number 7 my daily budget is for food, from breakfast lunch and supper in a day I can spend 700/= because for breakfast I want to buy charcoal which I will use for cooking, I want to buy sugar, milk and what we are going to take tea with it can be bread, mandazi or chapati. Then for lunch I buy flour whatever that is used to eat this ugali salad you know how the price has increased and then supper also is just food. So in a day I could spend like 700/= depending with the number of family members that I have.

**I: Another person? Each and every person is going to tell me what she uses daily at home, number 4.**

PV04: As number 4 I feel it is food from breakfast till supper I use a lot of money, the way prices have increased mostly cooking oil and maize flour there prices have gone up and you find that if you are using them it is going to coat you a lot of money and in a day I use 500/=

**I: 500/= Mmh number 3 do you have something?**

PV06: As number 6 what we spend on daily is like food, you find that the way the prices of commodities have increased you want to buy cooking oil and so on, so in a day I can spend 500/= .

**I: 500/= number 2 what do you buy with your money?**

PV02: I buy food for the children from breakfast, lunch and supper so I can use 500/= in a day.

**I: Number 1**

PV01: As number one what I can buy during the day is breakfast, lunch and supper, I use more than 500/= in a day I can use 600/=

**I: 600/= number 6**

PV06: [Inaudible segment]

**I: Be audible.**

PV06: That money I use in buying food it is 500/= to buy breakfast, lunch and supper. [Child making noise]

**I: Okay what is it that we buy once in a week what are they what is it that you buy once a week? Number 10.**

PV10: What I buy once is charcoal, I buy one *debe* that costs 250/= I know it is going to last for one week for it to be over, I am going to buy 1 bar of soap which is 200/= now I know when I cut it into pieces when washing clothes for my children I know by the end of the week it is over and I start budgeting for it again and food is daily

**I: I need the ones for once a week you have talked of two things, is there any?**

PV10: There is none.

**I: There is none number 5**

PV05: As number 5, what I can buy once in a week is soap,

**I: Mmh, how much?**

PV05: I buy half bar of soap and it is costing 80/=

**I: Eeh**

PV05: It is now 80/= you know I usually wash once a week so if I buy it this week when I wash and when it reaches Saturday I know I will buy .

**I: Another person? Number 4.**

PV04: As number 4 what I can buy once a week is soap or charcoal .I buy soap at 200/= and it takes one week , charcoal I buy 1 debe and it lasts for one week the rest it is impossible to buy once a week I buy them daily

**I: Okay number 3.**

PV03: What I can buy in a week is soap, I buy one bar of soap and I know how am going to survive till it takes one week. It is only soap.

**I: Only soap number 2 what do you buy once in a week?**

PV02: Something like maize flour,

**I: Is what you buy once?**

PV02: Yes

**I: Is what you buy once how much?**

PV02: The one for 600/=

**I: 600/= Mmh number one what do you buy once in a week?**

PV01: Soap

**I: Soap**

PV01: I buy one bar of soap,

**I: Number 6 what do you buy once in a week?**

PV06: Soap

**I: Soap of how much?**

PV06: of 200/=

**I: 200/= Now we want to look at what do you buy once a month, once in a month what is it**

PV07: Tea leaves, salt,

**I: You are through?**

PV08: What I buy once in a month is a dress.

**I: Number 8 buys dress once in a month,**

PV08: Yes.

**I: Wait, if you buy tea leaves and salt how much is it?**

PV07: Tea leaves 50/= is and salt of 130/=

**I: Number the cloth you buy is how much?**

PV08: 800/=

**I: 800/= is there anything else that you buy once in a month?**

PV08: Cooking oil [salad] I can buy 5 liters once in a month

**I: How much is it?**

PV08: 3000/=

**I: Is it 5 or 10 liters?**

PV08: 5 liters cost...

PV07: 1500/=

PV08: 1500/=

**I: Mmh, 1500/= number10 what do you buy once in a month?**

PV10: What I can buy once in a month is match box and then I buy 2kg of salt ,a packet of match box now is 50/= and I will go buy 2 kg of salt which is 70/= I buy salad[cooking oil] of 300/= that is 2litres and a half. then I go and do a budget of maize flour which a packet is 150/= and I take 2 packets that is 4 kg and put in the house as I wait for the month to end.

**I: Mmh, number 5**

PV05: What I can buy once in a month it is something that is eaten but it is a sanitary towels

**I: There is no problem I had said that we buy different things**.

PV05: I don’t it buy daily, when I buy it is enough till the next month,

**I: How much it is?**

PV05: I buy two packets at 150/= each is costing 75/=

**I: Number 4**

PV04: For me what I can buy once in a month I refill gas and it takes one month, I will spend 1600/= that is once in a month, secondly I can buy pads, I buy two packets that I will take one month with it that is what I can buy then there is salt I buy 1 kg of salt and it can last up to one month and tea leaves it is something that you use in small quantity that I can also take one month

**I: What is the total cost?**

PV04: Gas is 1600/= salt I use the one for 100/= tea leaves of 50/= pads is 60/= each 2 packets is 120/=

**I: Okay, number 3**

PV03: What I can buy once in a month is pant, I can buy dress, I can refill the gas,

**I: Total cost?**

PV03: Gas is 1600/= I can buy a pant of 150/= and I can buy a dress of 800/=

**I: Okay number 2**

PV02: I can only buy a dress once in a month.

**I: How much?**

PV02: 500/=

**I: Number 1**

PV01: What i can buy in a month is to refill the gas which is 1600/= and I can also buy a dress, salt, tea leaves that will last for one month.

**I: Mmh total**?

PV01: Dress can be like 800/=, refilling gas is 1600/=

**I: Just say the amount even if you don’t add them up**

PV01: 1600/= for the gas, dress is 800/= salt of 50/= and tea leaves of 50/=

**I: Okay, number 6 what do you buy once in a month?**

PV06: Dress, salt and match box

**I: Okay what do you buy once in a month and how often do you buy it for example once in a year or once in one term of learning and others what are they?**

P: Kindly repeat the question.

**I: What is it that you buy once in a year?**

P: Mmh

**I: Or in a term.**

P: Mmh

**I: Right in a term or a year what are they? Number 10**

PV10: As number 10 what I buy once in a year or term is that I can go to the market and do the calculation of buying a dress I can even buy 5 dresses and see how much they cost then after one year is when I will think of buying a new dress .I can also budget for buying one sack of charcoal or two sacks of charcoal and put in the house and it should take one year depending on my situation and I also go and make the three cooking stone since I stay in the rural and find a way of looking for firewood and I use firewood to cook , now I will be using firewood to cook but when it rains I use charcoal want this charcoal to last for a year, the 4 dresses that I bought I also want them to take me for a year and come next year is when I start budgeting for it and the children’s uniform I buy once in a year.at the beginning of the term I make sure each child has a pair of uniform that will last a year.

**I: How much is the total?**

PV10: The total, charcoal at the moment it is 1000/=, 2 sacks of charcoal is 2000/=I sacrifice 2000/= and buy charcoal then I will go buy dresses of 500/=I can even buy six dresses that will cost 3000/= that is a total of 5000/=

**I: Mmh**

PV10: For fire wood I don’t spend money

**I: Uniforms**

PV10: Uniforms for the children is 500/= that is one uniform and they are two children and each has to have two pairs of uniform, that is 2000/=.

**I: Okay another person, number 5**

PV05: As number 5, what I can buy once a year is that I wait when the prices of maize has gone down let us say it is 50/= per gorogoro I can buy 2 sacks of maize, when I buy them it will take me for a whole year the same with the big size of petroleum jelly for the children, that I know if I buy it will last them a year or more now that is how I do it.

**I: Total?**

PV05: Total I can spend 4000/=

**I: 4000/= number 4**

PV04: Here my budget is very small I have two children, school uniform each child have two uniforms in every year and 1 piece of uniform is almost 600/= and each child it will cost 1200/= and the total amount will be 2400/= in a year.

**I: Mmh**

PV04: That’s my budget.

**I: You use 24 cows or goats**

PV04: No shillings [participant chuckles] 2400/=

**I: 2400/= right another person**

PV03: As number 3 what I can buy is uniform and school shoes, if I buy 1 uniform it is 500/= and if it is a pair it will be 1000/= that is for one child and school shoes if I add, the shoes can cost 1000/= and if they are 3 children and every child when I buy shoes and two pairs of uniforms it is very expensive.

**I: Okay number 2**

PV02: Uniform

**I: Uniform,**

PV02: That I buy for my child.

**I: How much?**

PV02: 500/=

**I: 500/= number 1**

PV01: As number 1 what I can buy is uniform.

**I: Mmh.**

PV01: I want to buy two pairs that includes games kit games kit one pair is 1550/= I have to buy bag, shoes and socks

**I: Roughly how much, you can’t say the exact amount but approximately how much can you use?**

PV04: Approximately I can use 15000/=

**I: I am here these people have not talked number 7**

PV07: In a year I buy uniforms for my children once and each child’s uniform can cost 2000/= uniforms for 3 children and I also buy shoes toughees which is 2000/= each that is 6000/= for one year and then things that are done termly I can buy a dress for myself termly .also lipstick the lotion I use also I buy termly my shoes and dress I buy termly I can’t buy every month because they last longer.

**I: Mmh**

PV07: So those are the things that I can do after a while.

**I: How much can you spend?**

PV07: I also pay fees termly now it is over 30000/=

**I: Mmh Number 6**

PV06: I buy 2pairs of uniform for the child shoes and bag.

**I: Total amount?**

PV06: It can reach 10000/=

**I: 10000/= number 8**

PV08: What I buy yearly first of all it is shoes for school for the three children if I buy toughees it can cost 6000/= then uniform, there is one whose uniform is 500/= and the other one is 600/= then there is also uniform that cost 1800/= and again what I can buy termly is paying school fees

**I: Mmh**

PV08: I also buy books termly.

**I: Okay total?**

PV08: Total is roughly 15000/=

**I: Mmh, number 10 Number 9?**

PV09: My budget for the term or a year is buying for the children books that they will use for a term and another term they are two children and for one child the books can cost 1200/= then I buy them uniform and it costs 550/= each for each one of them that will be 2350/= in a term.

**I: Mmh**

PV09: Yes.

**I: Only that**

PV09: Only that.

**I: There is no one who has something to add?**

P: Yes.

**I: Okay we will continue but I have found out that there is nobody who is using lotion, there is no one who is plaiting her hair**

P: As we have shaved.

**I: There is no problem that is your budget now am asking most sex workers where do they get the money they spend from?**

PV10: As for me I get money from the men.

**I: From the men mmh another person? Number 4.**

PV04: As number 4 I get money depending on how I got clients from sex work when I leave for sex work is when I can get money and if I don’t go then there is no money I will get.

**I: There is nothing okay another person? Number 5.**

PV05: As number 5, I get money from sex work.

**I: From sex work, number 3.**

PV03: As number 6.

**I: Number 3.**

PV03: Number 3 I get money when a client’s calls me asks me how am I today if I can meet him so I can go.

**I: Mmh**

PV03: Sometimes you can stay for two or three days without anyone calling you.

**I: Mmh**

PV03: Unless they call if I can meet them then I leave that is how I get my money.

**I: Okay number two where do you get your money from?**

PV02: I can be called and ask where am I and I tell them where I am and ask if i can come to such and such a place so that we meet.so I will borrow money to use as fare so that I can go that is how I get my money

**I: Number 1 where do you get your money from?**

PV01: I get money from the men.

**I: From men, number 9 where do you get your money from?**

PV09: I get it from sex work.

**I: When you go for sex work. Number 8**

PV08: number 8 as a sex worker I will only get money from sex work.

**I: From sex work mmh number 7**

PV07: From sexual partners.

**I: From sexual partners, number 6 where do you get money from?**

PV06: From sexual partners.

**I: Okay, now all of you are getting money from sexual what?**

ALL: Partners.

I**: Now is there any source of income apart from sex work? Number 6 apart from sex work do you have any source of income?**

PV06: There is none.

**I: There is none, okay number 7**

PV07: There is.

**I: Please tell me.**

PV07: Salon.

**I: Salon number 8.**

PV08: There is none.

**I: Number 9.**

PV09: If I go and do work for someone in the farm.

**I: When you work for someone in the farm you get money, number 10**

PV10: I cook and sell chips.

**I: Meaning you are selling chips?**

PV10: Yes

**I: Number 5**

PV05: Washing clothes for other people.

**I: Washing clothes mmh number 4**

PV04: There is none.

**I: None, number 3**

PV03: None.

**I: None number 2**

PV02: I work for somebody.

**I: You are working or if you work for somebody? You are working?**

PV02: Yes [working for someone]

**I: Number 1**

PV01: When I go and wash clothes for someone.

**I: Washing for someone okay, now what is the reason for sex workers spend on what they buy** **the things you had mentioned that you buy why do you buy them?**

PV09: For us not to be seen as we don’t have that much

**I: Be audible Number 9**

PV09: For us not to be seen like we don’t have so we be the same with those who have.

**I: Mmh, another person? Number 5**

PV05: As number 5 the things we buy the reason we are buying for example food it is something that is a must you have to eat because if we don't eat you will die

**I: Okay, number 3**

PV03: Number 3 I get money when a client’s calls me asks me how am I today if I can meet him so I can go.

**I: Mmh**

PV03: Sometimes you can stay for two or three days without anyone calling you.

**I: Mmh**

PV03: Unless they call if I can meet them then I go that is how I get my money.

**I: Okay number two where do you get your money from?**

PV02: I can be called and ask where am I and I tell them where I am and ask if i can come to such and such a place so that we meet.so I will borrow money to use as fare so that I can go that is how I get my money

**I: Number 1 where do you get your money from?**

PV01: I get money from the men

**I: From men, number 9 where do you get your money from?**

PV09: I get it from sex work

**I: When you go for sex work. Number 8**

PV08: number 8 as a sex worker I will only get money from sex work.

**I: From sex work mmh number 7**

PV07: From sexual partners

**I: From sexual partners, number 6 where do you get money from?**

PV06: From sexual partners.

**I: Okay, now all of you are getting money from sexual what?**

ALL: partners.

**I: Now is there any source of income apart from sex work? Number 6 apart from sex work do you have any source of income?**

PV06: There is none

**I: There is none, okay number 7.**

PV07: There is.

**I: There is please tell me.**

PV07: Salon

**I: Salon number 8.**

PV08: There is none

**I: Number 9**

PV09: I f I go and do work in the farm of someone

**I: When you do farm work, you get money, number 10**

PV10: I cook and sell chips.

**I: Meaning you are selling chips**

PV10: Yes

**I: Number 5**

PV05: Washing clothes for other people

**I: washing clothes mmh number 4**

PV04: There is none.

**I: None, number 3**

PV03: None

**I: None number 2**

PV02: I work for somebody

**I: You are working or if you work for somebody? You are working?**

PV02: Yes

**I: Number 1**

PV01: When I go and wash clothes for someone.

**I: Washing for someone okay, now what is the reason for sex workers spend on what they buy the things you had mentioned that you buy why do you buy them?**

PV09: For us not to be seen we as if we don’t have that much

**I: Be audible Number 9**

PV09: For us not to be seen as if we don’t have, so we be the same level with those who have.

**I: Mmh, another person? Number 5**

PV05: As number 5 the things we buy the reason we are buying for example food it is something that is a must you have to eat because if we don't eat you will die

**I: Okay, number 3**

PV03: As number 3, the reason we buy these things like make ups we have to be attractive to our clients so we have to be very smart that when they see us they also have the appetite of engaging in sex with us.

**I: Number 4**

PV04: As number 4 you have to buy food because for me my clients come to the house when he comes to the house and find food he is free to give me good amount of money, so that when he comes again the next day he finds that I have prepared the food more than it was the previous day.

**I: Mmh, number 2 I can see you are smiling [participants chuckles]**

PV02: for me to be smart, so that when I meet him he will say this time round I am not leaving her

**I: Number 1**

PV01: The things we buy like soap, we need to wash our clothes with soap so that they be neat and we are also clean.

**I: Mmh**

PV01: We also have to buy food for us to be healthy.

**I: Mmh**

PV02: When you eat you have a healthy body.

**I: Mmh who is remaining?**

PV08 Me

**I: Number 8**

PV08: We just have to eat well so when you go for service [sex work] you work very hard

**I: Number 7**

PV07: The things we buy, we buy them because they are the things we need and it is a must we have them and there are others that we buy because we were to be in our comfort zone

**I: You want good life**

PV07: For example if I buy TV that I want good life.

**I: Mmh okay**

PV07: Music system so that I can be entertained

**I: Mmh**

PV07: That is good life.

**I: Okay, number 6**

PV06: For us to have food in the house

**I: Okay we have talked about the things we buy for ourselves, what about others why do we spend on** **them?**

P: Mmh

**I: For the others somebody said that she is paying school fees, buys dress for the child, why do we spend on them? Number 7**

PV07: Because it is a responsibility that is upon me,

**I: Mmh**

PV07: When I don’t pay school fees nobody will do that, it is my responsibility.

**I: Mmh, number 5**

PV05: As number 5, like I am the father and the mother I have to provide for the children, because if I don’t provide there is no one who is going to provide for them. So that is something I have to.

**I: Number 3**

PV03: As number 3 there is no way I can take responsibility for myself yet I can’t take responsibility to my own child, now if I want my child to go to school and be neat like me it is a must I have to take care of her/him so that he/she be neat so that I take her /him responsibility as a parent.

**I: Mmh number 2**

PV02: [participant chuckles] I want my children to go to school so that they have a good future.so that in the future they can’t be as me [sex workers]

**I: Mmh, number 10**

PV10: Am educating my children with this my work because i want them to have a better life

**I: Mmh**

PV10: One day she will tell a story and remember how her mother suffered for her to put her in a better life.

**I: Mmh, Is there anyone who has something to add? Is there anything you want to add?**

P: There is none.

**I: None, we are moving on to the next question, I am asking that do sex workers save, do you have** **savings?**

PV05: Yes

**I: You can respond one by one, number 3**

PV03: As number 3 I am saying it is difficult to have some savings because you have gone to the field [sex work] then someone gives you 500/= how do you start using this 500/= and you have so many things that you want to buy with this money. Now if you get good money from the field then you can be able to save you know here in the rural sex work can’t be better because people lament that they don’t have money obviously it can be good the amount you can be given is 500/= coming back there is nothing in the house and there is no way you are going to save 500/=.

**I: That is the opinion of number 3, another person? Number 5.**

PV05: I don’t save because whatever you get you are going to spend the all of it .Sometimes it is little like 500/= you are going to use it all and it is not going to be enough you know when it is not enough then there is nothing you can save. Now I don’t save

**I: Number 4**

PV04: As number 4 saving is not easy the reason saving is not easy is that we get very little and when you have reached the child wants to tell you mama I was chased they want school fees, mama I don't have pencil/pen, I am going to use this money and tomorrow am going back to the field and sometimes the weather is bad and I didn't get I came back with nothing so the next day is when I can get something so saving is not easy.

**I: Number 10 are you saving?**

PV10: When I go to the field, I make sure I have saved 10/= So that the day that I miss .I have something known as home bank whatever the case or reason I have to save 10/= because you can go to the field and you don’t get anything and when I don't get I know that there is bank somewhere in the house I am one the who knows where it is and that day my children will not sleep hungry they will have to eat.

**I: Number 9 do you save?**

PV09: It is easy for me I can’t lie that I can save because whatever we get is very little and sometimes you went to the field and you didn't get anything so when you come back and whatever little you got you will see on how to help children to have a better life But saving is not easy for us, I cant

**I: Mmh say that saving is not easy for you not all,**

PV09: On my own I can’t save.

**I: Okay, number 8**

PV08: Saving is not easy.

**I: It is not easy.**

PV08: You can save but the next day when you are broke you find yourself using the savings.

**I: But you have saved**

PV08: You only save today and tomorrow it is not there.

**I: Ooh okay number 7**

PV07: I can’t

**I: You can't .number 6**

PV06: Saving is not easy.

**I: Saving is hard, Now [interrupted by participant]**

PV05: Let me add something

**I: Okay, number 5**

PV05: I had said that on my side it is not easy but now that you have brought us Jitegemee now I have an advantage

**I: There is an advantage.**

PV05: Yes

**I: The advantage of?**

PV05: That we can save at least you can save 100/=, 20/= so when you don’t get anything from the field there is somewhere you can get from.

**I: Now is there any reason one should be saving for?**

ALL: Yes

**I: What are the reasons? Number 7**

PV07: There are emergencies that might arise like sickness, the child is sick at night and you did not have money at that moment, I mean money at hand but when you think that there is somewhere you have been saving you can go and withdraw and treat the child.

**I: Another one**

PV07: Another reason is that a visitor can come and the food you prepared was for two children so it will force you to find another source so that you add food to be enough for all so those are some of the reasons someone can save.

**I: Another one**

PV04: As number saving is good , if I had something that I saved when I go to the field and came back with nothing my children will not sleep hungry because I had saved something , I will withdraw and use it with my children secondly when I have gone to look for something [ sex work] and I get a client who want us not to use condom then I have the right of saying no or denying him sex because I know I left them when they are not badly off ,I will deny him sex and since I don’t want I can leave [not engaging in sex] because I know I am not badly off and my children will eat.

**I: Number 9 you have something right?**

PV09: Yes, saving is good it can reach a time when school fees is making your life to be difficult because the standard of living is high ,and when you are saving little by little you will find a way of helping yourself and preventing your child from being sent away from school every now and then.

**I: Number 5**

PV05: Saving is good for example as women, we do have our monthly periods ,so when you are on your periods and you don't go for sex work you can using your savings and you help yourself with it and secondly you can use saving for emergency purposes for example sickness, I can use that.

**I: Okay, number 3**

PV03: Saving is good because your child can come from school that the want this and that in school and when you had saved you can get something to give her and she goes back to school.

**I: Mmh is there anyone with something to add apart from the thing they have mentioned? Is there anyone who wants to add? [Silence] there is none**

ALL: Yes

**I: Now all of you said that you are not saving right, saving is not easy now what amount of money can you save weekly if you want to start saving, how much money can you save weekly? Number 9**

PV09: 100/=

**I: 100/= number 10**

PV10: 200/=

**I: 200/= number 6**

PV06: 300/=

**I: 300/= number 7**

PV07: 300/=

**I: 300/= number 8**

PV08: 400/=

**I: 400/= number 5**

PV05: 400/=

**I: 400/=**

PV04: As number 4 I can save 200/=

PV03: 100

**I: 100/=**

PV01: 200/=

**I: 200/=**

PV02: 200/=

**I: 200/= okay are there characteristics of sex workers who save has?**

P: Character

**I: Yes characteristic *tabia* is there a character that sex workers who save has?**

P: I have never heard.

**I: There is none, okay and is there character that is known for sex workers who do not save have?**

P: There is none.

**I: Okay for sex workers who are saving why are they saving that one we had responded to right?**

ALL: Yes

**I: Now what makes it easy for someone to save number 3?**

PV03: The fact that you have earned a lot of money for example when you go to the field and you get a lot of money that makes it easy for someone to save.

**I: Mmh**

PV03: And when you go and get less money it is very difficult to save.

**I: What else makes it easy for you to save?**

PV05: As number 5, what makes it easy for me to save is that when you go and get 10/= you use 5/= and save 5/= so that can...

**I: Is there any, Number 7?**

PV07:What can make it easy to save is that when I see best practice from another person that saving has made her to do such a thing, that can motivate me that when I save I can also be where she has reached or do whatever she has done.

**I: Mmh another one, okay what are the challenges we face while saving that makes as not to save what are they? Number 7**

PV07: Responsibilities versus the money does not march

**I: Do not march, how?**

PV07: The responsibilities are too many exceeding the money you have.

**I: Mmh another person number 5**

PV05: The challenge we face is that you feel that whatever you are getting is very little and now you feel that when you save the remaining amount is not going to be enough for your needs.

**I: Number 3**

PV03: The challenge is just getting a little amount of money and the amount is little and the needs/ wants are many.

**I: Number 1 do you have something you can say concerning the challenges that you face that hinders you from saving what are they?**

PV01: It depends with the money I get and the standard of living is also high.

**I: Number 6 do you have something what makes you not to save, what makes you not to save?**

PV06: Too much budget [things needed] and things are also expensive

**I: Okay, is there anyone who has something to add? Okay you have said that the money is little and whatever that we want to do with the money is too much. [Budget is high]**

ALL: Yes

**I: What can we do to overcome these challenges? Number 3**

PV03: Only if we save

**I: If we save mmh, what else? You have said that the responsibilities are too much and the money is**

ALL: little not enough?

**I: Now what do you want to do so that you find a way of getting something number?**

PV01: If only we work very hard

**I: Only when you work hard, another person? Number 7**

PV07: Having another source of income.

**I: Having another source of income, right**

ALL: Yes

**I: There is something that i want from you, another one?**

PV01: we should not depend on one source of income that is sex work.

**I: That is having another source of income, another one? Too much responsibilities and money is…**

ALL: Not enough

**I: What do you want to do?**

PV05: You squeeze your budget

**I: Squeeze your budget so that is can be…**

P: Enough

**I: Number 3 you have said you do what?**

PV03: That is what I wanted to say.

**I: That is what you wanted to say, okay for sex workers who are not saving, what do you think makes** **them not to save you had responded that too much responsibilities and the money is**

ALL: Little and the prices of the commodities are also high.

**I: The prices and economy is**

ALL: High

**I: Okay and is there any disadvantage of not saving?**

ALL: Yes

**I: You can say them, Number**

PV07: To begin the disadvantage of not saving is that in case you have an emergency I am stranded if I go to [mentions a name] to assist me she will say that she is just the same as me I don't have .if I go to [ mentions a name] she will tell me that she had given someone who is yet to give it back [ participant chuckles] now I am stranded with my problem but if I had saved I won't have any worries .I can call and go there and withdraw and I sort out my problem.

**I: Another person number 5?**

PV05: The disadvantage of not saving is that when you have an emergency there is no where you can turn to.

**I: Mmh**

PV05: Again another disadvantage of not saving is that if you didn't go for work [sex work] this our work obviously you are going to sleep hungry. Your children are going to sleep hungry because there is nothing you left behind so they will sleep hungry.

**I: Disadvantages of not saving there are so many reasons number 4**

PV04: The disadvantage of not saving is that you went to the field and didn't get anything and when you have nothing what are you going to eat, that is the disadvantage.

**I: Mmh number 2 do you have something?**

PV02: None

**I: Number 3**

PV03: They have talked about it

**I: They have mentioned it**

PV01: I want to add

**I: Mmh let her talk first**

PV01: As number 1, when you go to the field and don't get something when you come back you have no money and the landlord has locked the door so you will be so desperate since there is no where you are going to get the money.

**I: Mmh**

PV01: If you had saved you could go and take.

**I: Mmh, Number 7**

PV07: I wanted to add that the disadvantage of not saving is that it can put me at risk of getting HIV because have met someone and he gives me a lot of money and he says that we are not using condom because I am desperate, I don't have another source of income and there is nothing I have left in the house now I will just accept with whatever he wants so as to get the money so that I can help myself. Now that puts me at risk of getting HIV. The risks are very high.

**I: Okay number, what are the disadvantages of not saving?**

PV06: you can have a problem and you will have no way to get the money so whatever you have saved will help you.

**I: Okay is there an advantage of not saving?**

ALL: no

**I: Eeh**

ALL: There is none

**I: There is none now where do sex workers save their money where do they usually save? number7**

PV07: Chama, we have Kenya Women .we have Mpesa, Bank.

P: Mswhari.

**I: Where do you they usually save?**

PV05: Merry go round.

**I: Merry go round.**

P: Yes

**I: Is there any difference between Merry go round and Chama?**

All: It is the same.

**I: They are the same.**

P: Yes

**I: [ a child making noise] that child we will send her out very fast[ participant chuckles] ,you have said where you have mentioned but you had said that you don't save what are the reasons people are saving in the places you have mentioned? You have said Mpesa, Mshwari, Chama, right?**

ALL: Yes

**I: Now what are the reasons of them saving in those places number 10?**

PV10: I am in a Chama that I save after one year, So when a year reaches, you know a child wants a dress and when you save in the Chama and you share out after one year you know that such and such a date you are going to share out the money, if you get that money you can buy the child whatever she wants and in case there is something you lack in the house or you have not paid rent you can take it and pay rent. That is why I save.

**I: Mmh, number 7**

PV07: I can save in Chama because they share out by the end of the year your children celebrates Christmas very well by eating and dressing well.

**I: Mmh**

PV07: And for Mshwari or bank I save there because, now I have the freedom whenever I need the money I can take it,

**I: Mmh**

PV07: If there is need.

**I: Are there other reasons apart from the ones they have mentioned?**

ALL: We are supporting.

**I: There is none, you support**

ALL: Mmh

**I: Okay, we are moving to the next question, which says do sex workers live life beyond their means for example the money you spend is more than what you get?**

All: Mmh

**I: Eeh**

All: Yes

**I: Okay number 7 you have agreed tell me the reasons.**

PV07: I can agree because the life that I live is against the money that I get because the standard of living is high and that is why there is shortage ,I have a shortage because my wants are more than my money I have that is why I can sometimes go as far as borrowing

**I: Eeh**

PV07: Mmh

**I: Mmh another person we live a life where it is [demonstrating] and the money is**

ALL: Low

**I: Low why does this happen? Number 5**

PV05: That usually happens when the life we live in is more than what we earn sometimes you find that you want to pay rent, you want to buy food the one I had mentioned and the money is very little until you take debts.

**I: Mmh 8**

PV08: I am saying that we live life beyond our means because you want to be neat to attract men and when you are doing this he is going to give you less money now your life is high yet the money is not enough.

**I: Mmh who has something to add? Is there anyone with something to add?**

P: None.

**I: There is none, you have said that the we spend more than we get/earn, now how are sex workers top up between the money they get and what they spend.**

P: For me I go for sex work at night from 6:00 pm and for me I cook chips I want to cook till 5:00 pm then I close the business go back and cook for the children after that I leave for sex work now the money I got from the field I will add them together with the money I got from the morning sales and top up.

**I: Mmh another person how do you top up?**

PV09: I feel that I squeeze my budget so that whatever little I got to fill the budget I had that can help me.

**I: Mmh number 5**

PV05: I fill the gap by washing for others

**I: Washing, number 4**

PV04: As number 4 I had said that I am a sex worker and that is the only source of income I have there is no other source that I supplement with now it forces me to reduce my budget if it was high then it forces me to ...even instead of buying half half now I buy quarter quarter so that it can be enough.

**I: Number 4 how do you bridge the gap?**

PV03: I just reduce my budget to be a little bit low

**I: Mmh number2**

PV02: I also reduce my budget.

**I: Reducing the budget, number 1**

PV01: I also reduce my budget.

**I: Ooh number 6**

PV06: I reduce my budget.

**I: You reduce the budget, okay, someone said that you usually borrow money,**

All: Mmh

**I: Now what are the reasons for borrowing, where do you borrow from and what for? When you are responding you respond to all the questions right? Like you have borrowed money, where have you borrowed from right?**

ALL: Mmh

**I: What is the reason for borrowing and what are you going to do to pay back the debt and when responding you respond at once right?**

ALL: Mmh

**I: Or you are going to forget?**

ALL: No.

**I: Or we start with the reasons why you borrow money right?**

P: Mmh

**I: Okay what are the reason for borrowing? Number 7**

PV07: I borrow when I have an emergency for example my child has been sent from school because of school fees ,and I still have money I will go to the bank where I usually save and I borrow, a certain amount will be deducted every month until I finish paying the money I had borrowed.

**I: Mmh number 8 do you borrow money?**

PV08: Yes

**I: Where do you borrow from?**

PV08: I borrow from Chama.

**I: What are your reasons for borrowing?**

PV08: If I have an emergency and I don't have some savings.

**I: Mmh**

PV08: So it will force me to borrow for me to sort out my emergency.

**I: Which emergency?**

PV08: For example sickness, I can fall sick and I don't have money.

**I: Mmh number 9 do you also borrow?**

PV09: I don't borrow

**I: You don't borrow okay number 10**

PV10: If I borrow money depending with the emergency that arises, I borrow money because the money I borrow I will pay back with the interest ,so you can borrow and pay back within a month and in a month if you borrow 1000/= the interest is 100/= now I can borrow 1200/= or 2000/= after borrowing since I said I do business in the morning and also at night [sex work] I will go and borrow then boost my morning business [ chips cooking] because at times you can have losses . Now when I am doing the daytime business I will find a way of paying back, there are people who are walking here when they give you money by evening at 4; 00pm you shall have paid it back. now it force you if you have borrowed you have to pay back with your business, when I borrow this money I know an emergency can arise like my child can be brought home from school that she is sick and I can take that money and take the child to the hospital and also find a way of paying back.

**I: Mmh number 5 do you anywhere you have borrowed from?**

PV05: As number 5 I can borrow money for example when it is month end and the landlord comes and I still don't have money that will force me to borrow money so that I give him and apart from that money you can also go and borrow food from the stalls where they sell.

**I: Mmh now the money that you borrow to give the landlord where do you borrow it from?**

PV05: I can borrow from friends or even my friends are not around being that I am not a member of the group she will go and borrow from her group then when i get the money I pay back with the interest.

**I: Number 4**

PV04: As number I can borrow money sometimes my child has been sent from school and I had not gotten money ,it will force me to go to my friend so that she can lend me money so that I can take my child back to school.

**I: Mmh and how are you going to pay back this money?**

PV04: The way I am going to pay back is that when I go for sex work and God leads me in my sex work if I get I will pay back .

**I: Mmh**

PV07: : I can borrow money from Mshwari, fuliza in case I get an emergency of school fees it will force me to borrow from the phone and find a way of paying back

**I: Mmh number 2**

PV02: I can borrow money when the child is sick or she is sent from school fees.

**I: How do you pay this money?**

PV02: I Wash

**I: Where do you borrow money?**

PV02: I can lend my friend

**I: Mmh**

PV02: Or she can borrow from someone then I find a way of giving it back to her so that she takes it back from where she borrowed.

**I: Number 1 where do you borrow from?**

PV01: I borrow from the Chama sometimes I can fall sick and this can hinder me from going to work even for a week so it will force me to go and when I come back and pay back the loan.

**I: Mmh number 6 where do you borrow from?**

PV06: Mshwari

**I: Mshwari, and how will you pay back?**

PV06: I will be paying monthly.

**I: You will be paying monthly and what are the reasons for you borrowing?**

PV06: Maybe the child has been sent home because of school fees

**I: Okay, as sex workers what can we do to increase our income what is it that we can do? Number 10**

PV10: As number 10 we can decide and start our own business so that we don't engage in one business [sex work]

**I: Mmh**

PV10: So that we increase our income.

**I: Mmh what else apart from the one she has mentioned, what can we do to increase our income? You talk when you are quiet I am also waiting for you people, what else can we do to increase our income number 5**

PV05: What we can do as number 5 what we can do to increase our income apart from sex work, even if it is business she has just talked about, or washing, cooking chips or making porridge it can boost us.

**I: Mmh, is there any other thing we can do to increase our income?**

PV07: We can increase the price of shots.

**I: You increase the price of shots, you increase the price from what price?**

PV07: If I was charging shot 500/= now I can increase it to be 700/=

**I: Mmh**

PV07: So by the end of the day I’ll be having a good amount of money.

**I: Eeh**

PV07: Now it can make my needs to be at par with what i earn.

**I: Mmh apart from increasing the shot price what else can we do to increase our income?**

PV04: If you having three clients in a day you can increase it to four or five

**I: Mmh you increase the number of clients, how are you going to increase?**

PV04: Now it depends, sometimes there was a client who wanted to be your friend way back but you refused, now it will force you to go back to him

I: How do you go back and yet you rejected him?

PV04: Now you look for him

**I: Ooh, you look for him by foot or?**

PV04: You look for him by calling him.

**I: By phone.**

PV04: You will tell him that whatever he had wanted today you are ready, but that time you told him you were not ready so that day you tell him you are ready and if he was an interested person he can come but if he was not interested he will just go like that.

**I: Mmh what can we do to increase our income?**

PV05: According to me I feel that when I am doing this work I also do business.

**I: Mmh**

PV05: That is according to me.

**I: Mmh, okay we have mentioned those things we do to increase our income, somebody talked about** **increasing the price of shots will you protection or you will not?**

PV07: I will use protection.

**I: Still he will agree?**

PV07: Yes, he just have to agree since it is a mutual agreement.

**I: Eeh**

PV07: For me to increase the price we had talked and he agreed.

**I: Ooh, okay, in case you don’t get a client, you had said that you can go to the field and come back with nothing right?**

PV05: Yes

**I: Now what will you do?**

**PV05: Do what?**

**I: I have gone to the field and find that there are no clients what will I do? Number 10.**

PV10: As number 10, I have gone to the field and there are no clients and there is one shop that I usually go to even if I go there to be given something he knows very well that the next day I must pay.so that i find a way on how my family can feed because I am the mother and the father because at times you go there and you didn’t get a client and it is work that is done per day, now it forces you when you come back you have a plan B and the plan B is just going you usually borrow from e.g. mama mboga or you go to the shop and be given sugar on credit and tell her you will pay the next day. That is what I do

**I: That’s number 10, number 5 what do you usually do if you don’t get a client?**

PV05: If I don’t get a client just as had said earlier on it will force me to go and borrow.

**I: You are going to wash**

P: Borrow.

**I: Number 3 what do you usually do?**

PV03: If I don't a client it will force me to borrow.

**I: Number 4**

PV04: Even me it will force me just borrow so that my children can have food to eat.

**I: Mmh, number 2**

PV02: It will force me to borrow.

**I: Number 1, what will you do?**

PV01: I will go and do manual work for other people

**I: Number 9 what are you going to do?**

PV09: I will go and look for someone who wants his or her farm to be weeded and I do some weeding and get 200/= which I will use to buy food for my family.

**I: Mmh, number 8 what do you do if you don’t get a client?**

PV08: I will look for another source of income or I can supplement with borrowing.

**I: Mmh, number 7**

PV07: Just borrowing.

**I: Mmh number 6 if you don’t get a client what do you usually do?**

PV06: It will just force me to borrow.

**I: You will borrow,**

PV0: Yes.

**I: Okay, how will you know that today there are no clients? [Participant chuckles] I have reached the field how will I know that the clients are not available? Number 10**

PV10: You will just see the signs, you just know when something is good then you will know, I will see the signs because I will go and you always know that 20 minutes can't pass without you getting a client, but when you go there and you take 30 minutes or 1 hour you will just know that the day is bad. You have started seeing the signs very fast so it forces you to leave.

PV04: As number 4 when I reach the place of work I first check if the visitors{ clients] are available that is when I enter the club ,if I find that the tables are empty then I cry within my heart because i know the visitors are not available but when I get for example here

**I: Yes**

PV04: The tables have been arranged and find this place is very busy. I will take my seat and say that today ...now it will depend on how I dance, I dance for a while then I sit down I dance for a while and sit down and then things will just be okay, when the clients are not available it is the first thing you will notice as you reach the place and say today the day is not good and I am going to come out with nothing.

**I: Now at what time will you know that there are no clients?**

PV04: When the time is 11:00pm I give up

**I: Ooh**

PV04: Just leave because most people are going to sleep

**I: Mmh number 10, at what time will you know that the clients are not available?**

PV10: Just as I had said that when I go there I can’t take even 20 minutes, I just know if I take 30 minutes not even one person has approached me I will go to the hotel and look around since you have a place where you are usually based and there is nobody you will know that the that is a bad sign.

**I: Number 3**

PV03: Since I get my clients via phone when it reaches in the afternoon when no one has called or texted me I just know that today is a bad day. And *siku njema pia huonekana asubuhi* when you wake up in the morning and then the phone rings meaning today you are going then you just know things are okay

**I: Mmh .number 2 how will you know at what time will you know that today there are no clients?**

PV02: When the time is 3:00 before I get a missed call then I know there is nothing and even when it reaches at 2 o’clock

**I: Number 1 how will you know that today the clients are not available when it reaches what time?**

PV01: 11pm

**I: 11:00 pm Mmh anyone with something to add?**

PV07: Even me when it reaches 11:00 pm and I am in the club I will just go and sit at a corner where I know someone will beckon me and I have not heard then I know that it is not easy [participant chuckles] you know you are at a certain corner timing the clients and waiting to be called upon. And sometimes you act as if you are asleep but you are awake.

**I: Number 6At what time will you know that the clients are not available**

PV06: If he has not texted or called you.

**I: Okay, each and everyone is going to tell me the amount of money she owes.**

PV07: Are you going to pay for us?

**I: No [participant chuckles] I just want to know how much debt you owe, at any given time how much debt can you have right ,even me I have debts that I have not paid,number1 How much debt can you have?**

PV01: 3000/=

**I: 3000/= Number 2**

PV02:2500/=

**I: 2500/= number3**

PV03: 3000/=

**I: 3000/= number 4**

PV04: I have 10000/=

**I: 1000/= number 5**

PV05: 5500/=

**I: 5500, number 8**

PV08: 5000

**I: 5000 number 9 how much debt do you have?**

PV09: For now I have so much debt because I borrowed when I was taking my child to school I have 20000/=

**I: 20000/=number 7**

PV07: 12000/=

**I: 12000/= number 6 how much do you owe them?**

PV06:4000/=

**I: 4000/= we are almost we are moving on well do sex workers even think of when they would leave sex work?**

PV07: Yes

**I: Okay number 7**

PV05: The baby has fallen down [child crying]

PV0: There is retirement, there is nothing that has a beginning and does not have an end, and anything that has a beginning must come to an end I always think that one day I will leave but leaving is not easy because we are not saving.

**I: Mmh**

PV07: Now when Jitegemee comes they have a strategy that can help us so as to save then I can save and start some business then I retire.

**I: Mmh do you ever think about this that there will reach a time you want to leave this job [sex work]?**

ALL: Yes we do think about it.

**I: Yes you have thought about it and is it something you talk about with your peers? Is it something you talk about?**

P: Yes we do talk even with our peers /sex workers.

**I: What do you always talk about? What brings about such discussions?**

PV05: Sometimes you find that ... [child crying in the background]

**I: What brings about such discussions, now what makes you think that now or decide that I now want to leave this kind of work/job? Number 10**

PV10: Sometimes it find that you are seated and you have done this work until your children are now grown-ups and you want your children not to know the kind of work you were doing in the past , or they should not even hear about it that their mother was doing such a such job and was feeding them with it will portray a bad picture, now you try very hard that when the children are grown ups and there is somewhere you have been saving and you put a target that when it reaches this amount or day I will leave this job and go and start a business by the time this child grows up and she is an adult now whether is a girl or a boy she or he will find that you have some business and know that it is business you have being doing is what has been used to take care of her or him .

**I: Mmh I have asked that what brings about such discussions that now want to quit this work. Number 5**

PV05: As number 5 what brought about this discussion is one day I met with my friend somewhere known as =Nyaolo = as we walking ,as we were walking there are some people who know us and they started saying that see these sex workers where are you going to prostitute today [ participant chuckles]

**I: Now that person was abusing you people.**

PV05: Yes, now we felt bad about it, by the way it was hurting.

**I: Okay**

PV05: He was saying that where are we going to prostitute today now as we were going we talked and said is there any day we will leave this job and rest.

**I: Mmh**

PV05: It is not our own making but it is lack of something but to give those who are behind you how they can support themselves.

**I: Is there anyone with something to add, what brings about such discussions?**

PV04: I have something to add as number 4, what can you think of quitting is that you had come to an agreement with a client until you agree on the amount he is going to pay you, and you go to the room there is a way this person wants to treat you until you regret the reason you went to the room, now it reaches a time when you have left that place you sit and contemplate is there any day I would leave this work depending on how that person treated me so after leaving that place you regret.

**I: Mmh**

PV07: let me say add something about that,

**I: Yes**

PV07: I think she is trying to say that one shot someone stays for very long time, he wants to take too much time until you are now tired until there is no sweetness you just feeling pain instead of feeling good and his money is also little/less.

**I: Mmh**

PV07: Secondly maybe someone has a big penis and now you just persevere for him to finish so that you get your money. Now such things when you remember you and you need to retire and do something else that doesn’t give me headache [hard time]

**I: Mmh, now you have told me the reasons someone may want to quit this job right you have talked about personal and external reasons, for example you said that when you were walking someone is asking you, where you are going to prostitute today. Then you have said that someone can treat you badly right?**

P: Yes

**I: Is there anything you want to add apart from those?**

P: No

**I: There is none, now do you have plans that you want to leave this work? Is it something you are planning that you want to leave or what do you do? Number 4**

PV04: As number 4 according to me I can’t lie I have not had plans of leaving. Am still in the business [sex work]

**I: You are still in business, aah number 5.**

PV05: As number 5 I have not planned of leaving because if I leave there is no where I am going to get source of income.

**I: Mmh number 3**

PV03: As number 3 I don't have plans of leaving but in case I start another business then I can leave it but for now that is my source of income /survival. So there is no way I can leave.

**I: Mmh is there additions?**

P: We have the same opinion we are still not leaving.

**I: You are not leaving, reasons?**

PV07: How are we going to survive?

**I: I had said we talk?**

P: One by one.

**I: One person at a time**

PV07: We have not got another alternative that is the reason we cannot quit.

**I: Mmh, okay at what age do you think we can quit sex work?**

PV07: In sex work there is no retirement.

**I: There is no retirement**

PV07: There is no retirement in sex work it is you who decides that you want to leave but there is no age limit even 80 years old is still in the field.

**I: Ooh number 4**

PV04: As number 4 ,according to me I feel that there is no retirement in the field it depends with the way your muscles/joints will be tired because it also depend on the way someone may have different sex styles and when you are tired with those styles you can decide I can’t coil myself or I can't do what , it will force me to leave /quit and if my body is still strong but am old but I am still very flexible I will continue with the work

**I: Mmh another person, number 9 is dying with laughter tell us what you are laughing [participant chuckles] number 9 talk.**

PV09: There is no retirement in this our job it is just we will continue up to the end but sickness can make us leave and only death is what can make us to leave this work [participant laughing]

**I: You have said that only death can make you to quit.**

PV09: It is only death that can make us to leave because we don't have another source of income, you know we will just go and be there

**I: So age doesn't matter?**

P: Yes

**I: Okay and what can sex workers engaging in after leaving sex work I mean what can you do after quitting sex work? What type of job can you do number 3**

PV03: As number 3 what I can do after leaving this work when I am too old that I can’t do sex work anymore then my child is the one who will feed me ,now there is nothing I can do next.

**I: There is none, number 4**

PV04: As number 4 what I can do after quitting when I am old and I can look for small small business at my doorstep now that is the work I will be doing.

**I: Mmh another person? Number 5**

PV05: As number 5 I don’t see what I can do maybe I will quit when I am old unless there is someone who comes to me and tells me that I want you to quit this job and I give you job if it is something that can bring money then I can leave but if there is none I will just continue.

**I: No, whatever you are going to do must be a source of income, now is there any work you can do after quitting sex work?**

PV05: I can only leave when I am old and when I am old there is no work that I can do.

**I: Okay, now all of you want to leave this work after getting old.**

P: What will do and there is nothing we can do, there is no otherwise.

**I: Is there anyone with addition the kind of work I can do after leaving this job [sex work] which one is it?**

PV07: For me I can rear chicken, and sell the eggs and also sell the hens when they have matured and I get money. Now I am resting and able to eat.

**I: Right, number 6 what type of work can you do after you have left sex work?**

PV06: I can start a business.

**I: Start a business, is there anyone with something to add?**

P: No.

**I: Okay, where can you start the business you want to do? Where can you start this business?**

PV06: Near the place I am living.

**I: Near the place you are living, where do you want and rear the chicken?**

PV07: Where I stay

**I: Ooh where you live, who else said that there is something she can do yes number 4**

PV04: The business I can do where I stay because Sex work will leave me when I am tired and can’t go to the markets [participant chuckles]

**I: Okay, now is there any difference in your life after quitting sex work, in case you leave this job is there any difference you can have in your life or how you live? Number 9**

PV09: There can be a difference because when I quit and I had not settled on how I can get another source of income I can find that my life will be low because I am suffering since I don’t have any source of income and I also lack food since there is no way I can eat well .

**I: Mmh**

PV09: Now I feel if I leave without have something that I can support myself with then I can suffer because I don't have good source of income.

**I: Another person number 7**

PV07: If I leave this job I feel that my lifestyle will change because we had discovered earlier on that my needs are more than the income I earn, now when I leave and still my needs are still more and now there is nothing I am doing do you see how difficult it is .Secondly I had different sexual partners and I taste this one and the other ones [Participant chuckles] and now I longer have sex with those men, now you see it is going to be difficult.

**I: Anyone with something to add, is there anyone with something to add?**

P: No

**I: There is none, now do you know any sex worker who has left this job and came back? Left this job and came back?**

Pv07: Yes, personally I think even if leave I would go back

**I: Personally you think when you leave you would still go back?**

PV07: Yes, I have seen someone who left and came back.

**I: And you have you seen?**

PV08: I think I can personally go back without anyone telling me to

**I: That is number 8, number 9 have seen anyone who has left this job and came back?**

PV09: I haven't seen but what I know that if you leave when you are not old it is going to make you come back and when you remember your past life and you will also be craving for sex.

**I: Number 10**

PV10: If I leave this work I would still go back

**I: Okay and you are going to give me the reasons you would still just go back, number 5.**

PV05: I have seen someone who has left this job and came back she used to be in =Kisumu= her children brought her back home saying that she was humiliating them ,she stayed at home for two months and decided to go back.

**I: You mean she went back?**

PV05: She went back even as we speak she is not around she went back.

**I: Okay, number 4.**

PV04: The witch craft you are used to you can’t change my friend, you can’t change your source of income for me I can’t change.

**I: Mmh**

PV04: Even if I leave I would just go back.

**I: You just go back now what do you mean by the witchcraft you are used to?**

PV04: This is my business, you know even you have a business and the day you don’t get any income you have it rough. Now this is my business that I am used to it and the day that I don’t have a client I can’t sleep comfortably.

**I: You can’t sleep comfortably, number 3**

PV03: This is something even if I leave I would go back because if you are used to eating different food you can’t lie to us that you can eat one type of food and be satisfied, you have to change the diet [ engaging in sex with different clients]

**I: How do you change the diet?**

PV03: At times you get vegetable so you have to get meat that you are going to eat it with, sometimes you get a small penis and you get another one that when you engage in sex with him you are satisfied sexually .[participant chuckles]

**I: Now how will I know the diet?**

PV03: Now it depends with the person you get. You can get someone who has small penis.

**I: How will you know this?**

PV03: It is you who knows or you want me to experiment/demonstrate for you [participant chuckles]

**I: How will you select him amongst people that he has a small penis?**

PV03:That one now you will select randomly it is not that you know you don’t have a big penis ,you know when you tell him that he will be disappointed and even go and commit suicide after leaving that place, so it is upon you to know how to deal with him if you know he doesn’t have a big penis you can tell that today I don’t feel like engaging in sex , but the one whom you know if you engage in sex with you are sexually satisfied that one even it is which date you just go.

**I: Ooh, okay number 2 do you know anyone who left this job and came back?**

PV02: There is no one.

**I: You don’t know, number 1?**

PV01: I have seen many people.

**I: Many people.?**

PV01: I have seen three people.

**I: And number 6 have you seen someone who left this work and came back?**

PV06: Yes

**I: What was her reasons for coming back?**

PV06: She is used to sex work and the person she got is not supporting her

**I: Now what were the reasons for those who left and came back?**

PV07: I think when someone leaves and then comes back it means she left without plan [ participant chuckles] maybe she didn’t save and she decided that she wanted to take a break for a while that she had some savings to use and when the savings are over you are back again in the field.

**I: Mmh number 10**

PV10: The reason that can make someone to leave and come back is maybe she is addicted to sex and wherever she is she is not feeling ok, that is why she goes back

**I: Mmh another person number 3**

PV03: She has said whatever I wanted to say.

**I: She has said what I wanted to say, now what are the bad things that can happen to someone when she comes back? You left and came back again what are the bad things that can happen to you? Number 10**

PV10: When someone leaves and comes back you find that she loses many customers.it would be difficult for you to catch up. Now it forces you to start from zero looking for new clients.

**I: you start from zero, number 3**

PV03: She goes back when she is too old turn this way do this and she can’t do that.[Participants chuckles] she is old.

**I: She is old,**

PV03: She has no otherwise of getting income but she is going back because she is going to get something but the styles that have come up where someone wants to turn you upside down she can’t manage.

**I: Is there anyone with something to add the bad things that can happen to you when you come back? Number 9**

PV09: The bad thing that can happen when she left and came back is that maybe she left when she was healthy [ not sick] and coming back can make her die or be infected with HIV of which when she left she had not acquired.

**I: Mmh, is there another one**

P: No.

**I: And the good thing that can happen when she goes back?**

PV07: The good thing is just the pleasure [participant chuckles]

**I: Pleasure only?**

P: Money.

**I: And money mmh number 5**

PV05: the advantage she can have is that when she left she had no constant flow of income and when she comes back at least she will have something.

**I: She was getting money, mmh each and every person is going to tell me what she wants to accomplish before leaving this work [sex work] right**

P: Mmh

**I: Okay you want to tell me what you want to accomplish before you leave this sex work? Number 6 what do you want to accomplish first for you leave this work?**

PV06: I must have to save enough money

**I: Number 7 what do you want to accomplish?**

PV07: If I am done with school fees and build my house known as my home I think I will be in a better place

**I: Mmh**

PV09: I can retire

**I: You can retire, number 8**

PV08: after I have completed paying school fees and now I can be dependent

**I: Mmh number 9**

PV09: According to me ,for me to leave I have a stable finance even if there is nothing I am doing but there is a way I can survive without looking back where I had come from [sex work]

**I: Mmh number 10 what do you want to accomplish when you leave this work it finds that you have accomplished?**

PV10: when I completely leave this work, you know I have educated all my children and some are in the university and personally I have bought a piece of land where my children will live and even build rentals where I can be paid rent every month that I will leave this work and settle down.

**I: Mmh number 5 what do you want to accomplish?**

PV05: As number 5 after I have educated my children and they have completed school and get a piece of land and build a house now I don’t struggle to pay rent.

**I: Number 4**

PV04: As number 4 I just feel that after I have gotten a piece of land, my children have completed school and then I start a small business that will make me occupied, I can leave but I can’t leave without having something to do because what will I eat. I have to put something.

**I: Mmh number 3**

PV03: I can only leave after I have finished paying school fees, bought a piece of land and build rentals and I also know have completed their education and they can always sending me something end month. I can leave.

**I: Mmh number 2 what do you want to accomplish for you to leave this work?**

PV02: I can leave after I have educated my children and now they have their own income and I also have my small house but before I accomplish these then I can’t leave.

**I: Mmh, okay number 1**

PV01: I can leave after I have saved some serious amount of money, I can use that in making something that can generate income , that will make me to lack something which will force me to go back to sex work. And I have also educated my children and they don’t lack anything.

**I: How much is this serious amount?**

PV01: I mean the amount can’t be mentioned [participants chuckles]

**I: It has no limit, it is so much that it can’t be said?**

PV01: Mmh

**I: Okay the things you have mentioned are there any you have started planning for? Number 8 you have said?**

PV08: It is only school fees that we pay as we go on.

**I: Mmh only school fees but the rest you have not started putting them into action?**

P: Mmh

**I: What are the reasons you have not started planning on how you want to accomplish number 3**

PV03: Because of low source of income that is why we have not started planning. But if we get somewhere we can save at least it can give us time to accomplish what we want to.

**I: Mmh number 4**

PV04: She has said what I wanted to say.

**I: she has said what I wanted to Number 1**

PV01: As number 1 I had planned but the fact that the economy is high it has made my plans not to be as I had planned.

**I: Mmh okay and do you know any sex worker who have left sex work for the last 5-10 years ago**

P: Yes

**I: Eeh number 10**

PV10: I know one, She was even lucky she left that job and a client who she used to meet with married her and they have children and they living very well and have a good life

**I: Mmh another person who knows anyone who has left this job for 5-10 years ago?**

PV07: I have also seen she left and got married and has her home to date.

**I: Another person number 4 is nodding her head, you haven’t seen any?**

PV04: I haven’t seen

**I: Another person, there is none, number 9 is also nodding her head and what made it easy for them to quit this work?**

PV07: What can make it easy for someone to leave this work any person who has approached that he wants to marry me how is his financial capability , can he take care of me ,can he cater for all my needs if he can then I give him a go ahead .that is why I can leave it.

**I: Number 10**.

PV10: As one of the three people I had said she narrated to me how that person approached him, he came to her three times and they came to an agreement was married and took her back home and the man left for the town, the reason she agreed is that this person was working and he could provide for her. That is why she agreed and they stay at home to date.

**I: Mmh, number 3 have you seen one who has left?**

PV03: I saw one who left but came back

**I: Ooh came back?**

PV03: Mmh

**I: Number 1**

PV01: My friend told me that she left because when she saw her friend whom she was working with in the same job [sex work] came back and got infected with HV and she passed away.

**I: Mmh**

PV01: That is why she was disgusted.

**I: And there challenges they faced for those who left whom you know?**

PV07: yes there are challenges that are there.

**I: Like which ones?**

PV07: The cases we had heard about people who have left because you find that someone comes for you and takes out for a date but he is a kidnapper, he engages in sex with you and then he detains you, after he has detained you he engages in sex with you but he doesn’t pay you, you can’t call because you don’t have the freedom until the day he feels to set you free, after you have gone through such a scenario you just leave sex work.

**I: In this question I am asking for the people you said left, is there any challenge they are facing? You said that there was a friend of yours who left and she got married right?**

P: Yes

**I: Is there any challenge she is facing?**

PV07: The challenge a person can face is that the society they view you as somebody who is immoral even if you had changed/reformed, you are just like any other woman but they still call you with that name

**I: Ooh, that is the only challenge that might arise**

P: Yes

**I: Now how can we overcome this challenge?**

P: Unless I am saved

**I: What?**

P: Even if you are saved they will still say that you are sleeping with the pastor [participants’ chuckles]

**I: You have said that there is no way we can overcome this challenge even if I am saved still they will say the way I am sleeping with the pastor**

P: Yes

**I: It is not easy, okay now that we want to talk about Jitegemee, after we are done with this questions …I can see we are doing well. Now I want to read a portion here which says that earlier on I had clarified to you about Jitegemee and I also clarified that the sex workers have their money that they save that can to refuse to engage in sex without protection or take a break from sex work, when they feel like taking a break, I had also said that it is upon sex workers to save part of their money to use when there are no clients or what they have put in place as they prepare for life after sex work in the future You have heard that?**

P: Yes

**I: Now we are saying that this money you can withdraw it any time, you can withdraw a little amount or you can withdraw it all and there is no one who is going to force to…**

P: Save

**I: You save when you want to right now is jitegemee something that the sex worker would embrace in Kenya?**

P: Repeat the question?

**I: I am asking that the program of Jitegemee is it something that the sex workers in Kenya will embrace?**

P: Yes.

**I: What are the reasons for them embracing it?**

PV10: As number 10 they can embrace it because you have found a place where you can keep your money and you can withdraw it any time and there is no amount that increases, it is the same amount that you saved, so it will make you to be determined to save that money there, so that one day when you think of quitting this work and when you go to check your savings you find that you have an account with some money in it.

**I: Mmh**

PV10: And we feel that Jitegemee has come with us from far.

**I: Mmh, another person?**

PV07: As number 7, I feel that jitegemee can make me happy because it is going to stop me from borrowing money, now I will have some knowledge that there is somewhere I am supposed to be saving every day or every week and if there is need I can go and withdraw.

**I: Mmh number 5?**

PV05: As number 5, I can agree with you because it can help me with emergency cases or when I go to work and didn’t get something, there is a place I can go and withdraw money then it is not a must for me to engage in sex so as to get money because I have an account and again it is going to reduce my chances of getting HIV.

**I: Another person who has something to add, [silence] there is none, now what characteristics do sex workers have who would embrace the Jitegemee?**

P: Mmh.

**I: What characteristics of sex workers would embrace Jitegemee?**

P: All of us

P: Character as in

**I: What characteristics must you have so as to join Jitegemee?**

P: Okay, there is none

**I: Okay, if you have 10 friends and you have told them about jitegemee how many do you think would accept Jitegemee?**

PV07: Which type of friends?

**I: Your friends who you …sorry women friends**

PV07: Sexual partners.

**I: No women who are your friends and also doing the work of the field [sex work] if you tell them about Jitegemee how many of them out of ten would agree with Jitegemee? We will start from here. Number 1**

PV01: All of them

**I: Number 2**

PV02: All of them

**I: Number 3**

PV03: all of them

**I: Number 4**

PV04: You know where people are there are some people whose understands very fast and some who have difficulties understanding you will find that if you ten people you will find 6 people agreeing with you

**I: 6 people number 5**

PV05: All of them because it will force me to tell them about it and once they have understood after me explaining to them they will agree.

**I: They will agree number 10**

PV10: 9%

I: 9% 9,9

PV10: 9 out of 10

**I: 9 out of 10, which is 90% Number 9.**

PV09: They can all agree after we explain to them very well so that they can also join, they can agree all of them.

PV07: 8

**I: Mmh Number 6**

PV06: All of them

**I: Now number 7 the two people who would not agree what are the reasons they would not agree about Jitegemee?**

PV07: The reason I think the 2 people cannot agree is because her budget is higher than the money she has and she had gotten used to any money she gets is hand to mouth.

**I: Number 8 you have said that that 8 people will agree and what is the reason for the 2 people not agreeing?**

PV08: Some people their understanding is not easy, you know not all people will go to heaven you have to find rebels even if you explain to them to which extent they won’t agree.

**I: Mmh**

PV08: Eeh.

**I: Then number 4 you said that 6 people would agree, what of the 4 people who would refuse what is the reasons?**

PV04: They can’t accept because it depends for me I want to do as you say right

**I: Mmh**

PV04: our income what we earn is so little, now you know to get many people who will sacrifice to save would not be easy she will get used to how she has been going and getting money and spending her life should just continue like that and she won’t think of the future., that is the reason why when we meet/gather there are some people who will agree with you and others won’t agree depending on their income.

**I: Mmh number 10 you have said one person won’t agree why?**

PV10: You will find that when you are in a group and talking about this, there are some people who don’t understand, she will say that whatever you have brought those people eat people’s money, we will save our money there and we will not get our money it will disappear, not all would agree.

**I: What do you think as Jitegemee what can we do so that many sex workers to accept Jitegemee? What can we do as Impact organization we have come with Jitegemee program, what can we do so that many sex workers to accept Jitegemee? Number3**

PV03: If they see that we saved and it has borne fruits or we start a business or she sees us we are rising in our life. You know someone can come and ask you how you have reached there you can explain to her then she goes and tells the other people and this will make many women to accept what is going to happen in Jitegemee.

**I: Another person, number 10**

PV10: If you save money in Jitegemee and you tell your friend the amount of money you have saved and she also sees you withdrawing she will see that it is real. She will be interested and join.

**I: She will join, number 9**

PV09: If we mobilize them and then they are taught just like we are here now, they sit and they be educated, I think they can follow us.

**I: Is there anyone with something to add now the question I am asking, you know you have talked about yourself on what you can do but now us what can we do so that many sex workers can accept Jitegemee?**

PV03: It is just if you mobilize people and tell them the benefits of Jitegemee and their benefits.so that they know that is when they can accept and join many people.

**I: What else can we do, we are about to finish, we are remaining with 3 to 4 questions and we end they are not many.**

P: I am hungry.

**I: I know you are hungry, now [participant chuckles] you have said that we tell them the benefits**

PV07: What you can do is just what we are doing now [have a FGD] in every village, so it is something that is spreading and you will get people even if we are 10 people and 8 people agree and you go to another village like this.

**I: Okay now what can we do for it to be better? Number 3.**

PV03: What you can for it to be better is to if we are saving then you boost us financially so people will have interest of saving or someone can be interested of engaging in some other business apart from sex work, only if you can boost us or find a way of supporting us with like drugs, condoms and you find a way of supporting us with those things but mostly with financial support. You find a way of supporting us financially apart from the work we are doing at least it can give us some morale.

**I: Okay**

PV07: To add for us to be intact like the Jitegemee people it is good if we form a what’s app group where we can air our opinion, views and concerns that we have so that can make us to be together as a family.

PV04: To add, at times I can get a client and the client harasses me I must have somewhere I can report my problem and you get a way of supporting/ helping me if you have a center where you can help us at least you can give us advice or someone can tell you that he wants to engage in sex with you using protection and after going into the room he harasses you and force you to engage in sex without using protection, you can’t run to the hospital because they are going to judge you so we need a place where if we get such problems we can run to since you are going to understand us and find a way of helping us

PV10: If I can say something, Jitegemee you can put a target that if we reach then you can give us loan

**I: That is your opinion**

PV10: That’s my opinion.

**I: Okay, what would you not want to in Jitegemee?**

PV07: What I would not want in Jitegemee is that when I save my money but when I need it then I can’t get it or the money is missing.

**I: Another one what would you not want in Jitegemee?**

P: only that which we have said

**I: Only that, what would you like about Jitegemee, what would make you happy about Jitegemee?** **Number 3**

PV03: What would please me is when I save and in case of emergency I can get my money again what would please me is that even if I don’t go for sex work still I will find a way of eating.it is not a must I have to engage in sex with someone so as to be able to eat.

**I: Mmh number 5**

PV05: What would please about Jitegemee, is that when we save in Jitegemee and the time I need my money I am sure I will get it that would please me.

**I: Another person? What would you like about Jitegemee? What would you like about Jitegemee what did we say that Jitegemee wants to do?**

PV07: What I will like about Jitegemee is that I can retire from sex work and do a different type of job because I have enough savings and I can also sort emergencies if need be thirdly Jitegemee is empowering me not to say no to sex when I am tired or somebody wants to force me with something that is not acceptable because I am after his money, now I am empowered and I can say no when I want to or not want now Jitegemee can help me.

**I: Mmh is there anyone with something to add? I see you are tired**

PV07: We are having hunger pangs

**I: We know, now [participant chuckles] and is there anything you would not like about Jitegemee?**

ALL: Nothing

**I: There is nothing**

PV07: For now there is nothing we wouldn’t like because we don’t know how it would be in the future

**I: Do you feel that the information in Jitegemee is going to affect the right of sex workers, Jitegemee program is it going to affect your rights in any way**

ALL: No

**I: Nothing, now what challenges do you think as Jitegemee will face as we want to implement this program, we want to implement Jitegemee what challenges would we face? Number 3**

PV10: For you convincing someone to accept Jitegemee would not be easy. That is one of them.

**I: That is one of them what else is it? The challenges we can encounter as Jitegemee people? Number 3**

PV03: Some people when you are explaining to them about saving they won’t think that it is them who are going to save but they will think that it is you to give for them the money, to convince someone to understand that she is supposed to be saving her own money it will be difficult.

**I: Mmm what challenges would we encounter ?number 7**

PV07: As a Jitegemee member the challenge I will have is how I am going to adjust my budget for me to get something to save at least it will take me time before I catch up

**I: Mmh another person**

P: There is none

**I: Now the challenges you have mentioned tell me how you want to overcome these challenges?**

PV07: It is just by reducing the budget if I was buying 1kg of sugar now I buy ¾ kg. if I was buying one bar of soap now I can reduce it to half a bar of soap, or when I used to buy 2 packets of always now I will now buy 1 packet ,it is that.

**I: Number 3 you have said that for you to explain to someone about Jitegemee and understands she will think that we are the once going to give her money how can we overcome this?**

PV03: As you are going to explain to her that in Jitegemee that she is going to save her own money that will help you in the future not me giving you money.so the money you have worked for is the one that you save this it is going to help you in the future.

**I: Mmh number 5 you said that it is going to be difficult for us to convince people about Jitegemee, what are we supposed to do so as to overcome that challenge?**

PV05: Let me think about it

**I: Okay, we will come back for you to respond, now we are about to finish,**

P: I have a question

**I: Let as finish first there is a section anyone with a question will ask, okay now what amount of money can you save weekly?**

P: You had asked that question

**I: No that one I asked you about debt that you can have at any given time?**

P: you had asked for savings and I said nil,

P: I said 300/=

P: I said 200/=

**I: Ooh I had asked about it?**

P: Yes

**I: Okay,now where can you save your money comfortably and it is safe?**

PV04: Chama where I can be given after one year but in the phone I can have an emergency and use the money saying that I will top up which I will not do and you know when I have gone for Chama it is something that they are going to borrow and I have to pay back and now when its end year I have something that I will come back with to the house and in the phone I can’t lie to myself maybe in Chama

**I: Mmh number 3**

PV03: According to me I feel that Chama some people always run away with people’s money it is better I save in MShwari locked account whereby I say that during this duration I am going to withdraw because I can depend on Chama and then that person disappears now it is me who has the lock account if I put a duration of 6 months and when 6 months reaches I withdraw and spend it.

**I: Mmh, number 2**

PV02: I save in Mpesa

**I: Number 3 where can you save your money?**

PV03: I have not been saving now that you have come up with that idea I will now save [participant chuckles]

**I: Number 5 where can you save your money?**

PV05: when you shall have introduced Jitegemee then I will start to save because I know in Jitegemee when I will need my money I will be able to get it without doubt.

**I: Mmh number 10**

PV10: I will save in Jitegemee after an account has been opened that is where I will save

**I: Mmh number 9**

PV09: I will also save there [Jitegemee]

**I: Number 8 where will you save?**

PV08: I will save in Mshwari

**I: Mshwari number 7**

PV07: Jitegemee bank account

**I: Jitegemee bank account, number 6 where can you save your money?**

PV06: Mshwari

**I: Mshwari, now in case you have said you want to save in Mshwari, Mpesa you want to save in Jitegemee right?**

P: Yes

**I: In case you don’t get the money you want to save what will you do? That is the last question what will you do number 5.**

PV05: According to me I know I can’t lack

**I: No the amount you had said that you would save**

P: Yes

**I: In case you don’t get that amount what would you do?**

PV05: In case I don’t get, the one I had set a target if I don’t get I would not continue saving

**I: It is not that you didn’t get the money you saved but it is meaning that you said that you are going to save 700/= and you didn’t get to save what will you do, number 10**

PV10: If I don’t get the limit I had placed I will save whatever little I get to secure my account.

**I: Okay another person, number 3 is nodding her head what do you want to say, we are done just tell me so we finish**

PV03: She has said

**I: Number 5 can you give us your response on how we can overcome the challenge so that we finish [Participants chuckles]**

PV05: Just ask the question

**I: You said that it would be difficult for us Jitegemee people to convince people about Jitegemee now how can we overcome the challenge**

PV03: That was not even my response

**I: Whose was it?**

PV05 It was number 10

**I: Sorry number 10 respond all along I thought it was you.**

PV10: It is just that those who have saved money even you as the staff you also have to save there and tell her how you have saved and you withdraw when she is seeing you and see that it is real.it is something she will agree to.

**I: Okay number 7 you had a question anyone with a question can now ask as per the discussion about Jitegemee right?**

**P: Yes**

PV07: my question is now that we have heard I heard that Jitegemee can help us to reduce the risk of HIV now does it mean that those who are in the study they are HIV negative?

**I: What is happening is that we said that when one is financially empowered, right**

P: Yes

**I: her chances of one engaging in sex with her without using protection will reduce right?**

P: Yes

**I: when this program starts we would not choose those who are positive will not join, those who are negative will not join anybody who will agree with the program of Jitegemee but for the sex workers those are the people who will join the program if implemented. It won’t mind about your status.**

PV04: I have a question what if I got a client, there are cases that you are killed by a client now when such situations occurs how will you help that person?

**I: Now when you are already dead how are we going to help you?**

PV04: Now how are you going to help the other people? The rest of my people or my partners how will you help them so that they don’t fall in such scenarios.

**I: Partners?**

PV04: Sex workers

**I: Your fellow sex workers?**

PV04: Yes, my fellow sex workers

**I: There is a group which fights for the rights of sex workers like KISWA if that is what has happened you report and action is taken for the perpetrator.so that it be a warning to other not to do the same.**

PV10: And what if that person runs away?

**I: Now the government arm is long or**

PV10: That thing happened here in =Sega= twice

**I: Now that means that the administration here in Sega is sleeping on their job, if a person can be killed again another person is killed and they are just quiet**

PV07: As the Representative of KISWA I posted that incident in the group and you have has done nothing so in case of anything I am here,

**I: Now you are done?**

ALL: Yes

**I: Thank you so much for your time, I thank you all so much and the interview has ended at 2:00pm**

**END OF INTERVIEW.**
