# Supplementary material for: Eat a little and save a little: A qualitative exploration of acceptability of a potential savings intervention to reduce HIV risk among female sex workers in Western Kenya
Source: PLoS One. 2024 Dec 19;19(12):e0310540. doi: 10.1371/journal.pone.0310540 (PMC11658496; doi:10.1371/journal.pone.0310540)
Supplement: S1 File — (ZIP) [file pone.0310540.s001.zip › Jitegemee Transcripts and Dissemination Notes for Journal/FGD W.docx]

**FGD ID: FGD W**

**FGD DURATION: 1HR 45MINS 23SECS**

**FACILITATORS NAME: JUDITH**

**NOTE TAKER: PHILIP**

**CATEGORY: ABOVE 30 YEARS, PERI-URBAN**

**I: Okay this is FGD W, being done at Sega. The interviewers name is [Interviewer name omitted] and the note taker is [Note taker name omitted]. Okay, welcome all. Like I had briefly explained, what comes to your mind about Jitegemee? Yes, number six**

PW06: As number six, after your elaboration, I have been motivated so much. I have known that me as a sex worker, having done for a year, I can depend on myself, and have savings because I had set my mid that I would do sex work all my life. And mostly the money that I get is from this business, I always know that I will eat that money and the next day I would get another client, have sex with him and get paid. But now I have been motivated that now I can save for myself, open an account so that I can depend on myself instead of being abused… at times we would be abused sexually. Now we can start a savings group as sex workers so that tomorrow we can be independent, stop sex work or even take a rest.

**I: Thank you. Another opinion about Jitegemee as we had said? Yes, number nine**

PW09: As number nine, that word Jitegemee, according to my understanding, I will not be depending on sex work, I can decide to do my own things, I eat in my house, pay my rent, so I can do my own things like eat on my own, dress on my own and it is not a must to sleep with a man.

**I: Thank you. Number three.**

PW03: As number three, the word Jitegemee tells me that I can depend on myself without depending on anyone. Sometimes you find that the men we depend on, you can depend on them then you find something… then you go with him and he fails to pay you and maybe you do not even have food. But if I know how to rely on myself, I have my money kept somewhere, I can withdraw that money and use.

**I: Thank you any other idea about Jitegemee? Number four**

PW04: Me as number four the word jitegemee, I see it like someone can save their money. Out here right now there are those people we call them ‘sponsors’, yes they will take you and buy you everything but they have their own rules. So like in Jitegemee you can… if you get that money you can save that money and help yourself, it is not a must to have those sponsors

**I: Thank you. Another opinion, like we had explained, briefly? Number two**

PW02: As number two, the issue of Jitegemee, sometimes you can find someone, you go have sex with him then he does not give you money and when you go back to the house you don’t know where to start from and when I have my little amount of money it is not a must for me to depend on a client or I have sex with a client I can do my own job so that even when they come and don’t give me money I can help myself with it.

**I: Thank you. Number seven**

PW07: Okay me as number seven, I will say that this jitegemee as for me it has taught me about savings, I did not know about savings also it was not on my mind. So like at least if I get something small, it has taught that not every day I will get money, I might get today and tomorrow I fail to get. And tomorrow I fail to get a client, at least today I can save and that saving can help tomorrow.

**I: Thank you. Number one**

PW01: Me as number one this jitegemee taught me that when I get money, I don’t use all of it. There is a portion I use and the rest I save, it can help me when I do not have any money.

**I: Number eight.**

PW08: Me as number eight, this Jitegemee has taught me a lot, I can get today and save a little that will help me tomorrow, it’s not a must that I should depend on a client. I can go with the client and he fails to pay, then remember that I have my own savings that I kept somewhere let me withdraw to help me do a certain thing.

**I: Number five what is your opinion about Jitegemee? You don’t have anything to say?**

PW05: I have nothing to say

**I: Okay, what are the typical expenses FSW make and approximately how much does each cost, what do the buy each day? Yes, number three**

PW03: Me as number three, the money that we get through sex work, most of us have children who are school going, so at times you want to pay school fees and sometimes you want to buy books then there is also food, it is a must that you will use this money to buy

**I: So like books and school fees you can pay like how many times? in a month or every day or every week?**

PW03: Okay like now… okay as number three I have a child who is in CBC. I don’t know that CBC and I even don’t understand it. You buy it every day. When a child comes from school in the evening they want something, it becomes hard because at times you don’t have the money to give them. So if you save, you can find it easier to help yourself.

**I: How much does it cost you to buy those school items in a day?**

PW03: In a day it can be like… right now in a CBC a child can come today that they want wheat flour, tomorrow they want… so in a day you can spend like if it less it is five hundred shillings because the things that they want are many.

**I: Those are just for school?**

PW03: For school

**I: And how much can you spend on food every day?**

PW03: Okay for food, let us say that breakfast can be two hundred shillings, lunch is one hundred and fifty shillings and for supper, you must use around three hundred and fifty shillings, because the supper depends on the breakfast that you had.

**I: Okay thank you, another thing that girls buy every day? yes number seven**

PW07: Okay me as number seven, you know those clients that come there and you go with them, you know they always meet you when you are dressed nicely so sometimes you will be using your money to buy clothes yes because you want to dress nicely, you smell nicely

**I: So if it is clothes, do you buy them every day or every week or every month?**

PW07: So clothes I can put like every week because it is not every day that you get clients, so at least in a week you have a cloth, because you also need body lotion, you must also make your hair. And let us also say you have a family, you would also want to cater for them.

**I: So how much can you spend on clothes in a week?**

PW07: Clothes in a week, like one cloth is one thousand five hundred shillings that is for a trouser and top that is minus shoes, perfume, yes.

**I: Do you buy shoes every week?**

PW07: Not every week, but clothes at least weekly.

**I: For the clothes it is at least every week?**

PW07: Yes

**I: Perfume?**

PW07: For the perfume, you don’t know the time you might run out of it, you know if it runs out I refill.

**I: How much do you spend to refill it?**

PW07: For refill it is one hundred and fifty shillings

**I: Number four tell us what is making you happy, every day you spend like how much?**

PW04: As number four the money that I use, I must look good, I must be smart, my hair, the clothes that I wear. Outside there you will not get a boy or a boyfriend when you don’t look smart. You must be smart.

**I: So when you dress smart, you do it every day, every week, every…**

PW04: I look good every day

**I: Every day you spend like how much?**

PW04: Fifteen

**I: Fifteen what?**

PW04: Fifteen hundred shillings on clothes, five hundred on shoes.

**I: On daily basis?**

PW04: Yes

**I: So at least on daily basis two thousand shillings on your body?**

PW04: Yes

**I: Okay, another person, what girls buy every day? They are over? Yes, number six**

PW06: Me as number six, what makes us to have multiple sex partners or a lot of clients. You know a lot of people always ask themselves outside there our expense is high than our pay and also you must lower prices for our clients so that you can compete with others. We can get a client even ten times a day, sex work is like a shop, if you sell to many people you will be happy but the day you fail to sell, you won’t be able to pay rent. So I don’t mind the height of the client, I just state my price. I have to buy an expensive inner wear, because I have to compete with the wife the client has left at home. My bra and clothes must be clean. I bring my clients to my house, that is my office, instead of paying lodging, the client just pays for my house. I have to pay school fees, food and some other things. If the client comes to my house, I have to surprise with good food, after targeting some amount of money from him, you pay price to get price, you must lure a bird with something, so maybe I buy fish of two hundred and fifty shillings, and maize flour of fifty shillings, right now the cost of maize flour is so high, I know he will not leave me with less than one thousand shillings. So it forces me… I also use time, do first around 30mins am done, go shower and change new fresh clothes, so you find that we spend so much money for us to get that money, depending on the competition of other sex workers.

**I: Okay, like you in a day, how much can you spend to buy those things that you use?**

PW06: When the market is good, some of us we serve in the morning they eat quickly and go to work. So the morning clients are good because they don’t take time. Maybe he is the neighbor’s or the next door neighbor, he just passes by like a normal neighbor, such instances you know they give good money, because he is afraid. So it will force me to make him tea, maybe the wife did not make good tea for him, the one called ‘*DORCAS’ [to mean tea that has little milk]*. So you will find that making breakfast for one client is approximately two hundred and fifty shillings. You cook him eggs and things that he does not get in the house. So if I find like five clients am good.

**I: If you say they eat fast what do you mean?**

PW06: It is called a shot, he does what has brought him.

**I: Okay. A week does not end before you buy what? Yes number nine.**

PW09: Me as number nine a week does not end before I buy a condom and P2

**I: So condom is how much?**

PW09: In a week?

**I: Yes**

PW09: So like in a week let us say three each is seventy shillings, that is two hundred and ten shillings. Then in a week it is how much? one thousand times seventy, then P2 like one is one hundred shillings. Fourteen seventy in condom then P2 one packet is one hundred shillings so in a week I buy like seven packets of P2 that is seven hundred shillings and in the whole week I have spent fourteen seventy plus seven hundred it becomes two thousand one hundred and seventy.

**I: At least two thousand shillings?**

PW09: Yes

**I: Okay, so any other person a week does not end when you have not bought what things? yes number two**

PW02: Me as number two a week does not end when I have not bought panties, brassieres

**I: If you buy how much?**

PW02: Two hundred

**I: So at least every week you buy? You use two hundred shillings to buy them?**

PW02: Yes

**I: A month does not end when a girl buys has not bought what, yes number three?**

PW03: Me as number three, a month does not end when I have not bought alcohol. That is a must

**I: So that is drinking every day or once.**

PW03: I can drink maybe today I miss tomorrow but if it is drinking it is drinking

**I: So you can spend like how much?**

PW03: If I have decided to drink in a day I can spend like four thousand shillings and above. Because I must buy an alcohol that is expensive so that I get a sponsor that has money

**I: Okay. Any other person, what do girls buy before the end of the month? Number one**

PW01: Me as number one, a month does not end when I have not bought panty, brassiere and also drinking as for me. I use a panty of three hundred and fifty shillings and above so in a month I want to buy seven of them or eight you know when you use a panty there are some that you leave and use the others so in drinking when you are going to drink it is not that you go there and play

**I: And when you drink, it costs you like how much?**

PW01: As for me I need a bottle of “mzinga”

**I: A whole bottle of “mzinga” is how much?**

PW01: Fifteen hundred

**I: Fifteen hundred.**

PW01: Fifteen hundred that one cannot make me active so I will need another one. So in a week I can drink three times

**I: So that “mzinga” whole bottle is how many mls?**

PW01: 750ML

**I: Okay fine, a year does not end when you have not bought which things?**

PW03: Me as number three a year does not end without buying clothes for me and my family. Food is a must I mean anything that I can see and makes me happy it attracts me I must buy it

**I: So far food is every week or there is a food that you are waiting end of the year?**

PW03: There is one for every day, there is one for monthly so let us say the end of the year I could have bought all of them.

**I: Is there anything that you have not eaten the whole year you are waiting for that specific time?**

PW03: It is there but now I repeat

**I: And on food you can spend like how much?**

PW03: In a year

**I: Or a month**

PW03: In a month it is a little it’s like fifteen thousand shillings

**I: And when you spend on clothes, you spend like how much?**

PW03: In a year

**I: Yes**

PW03: It is around thirty thousand shillings because I buy clothes

**I: Thirty thousand?**

PW03: Yes

**I: Number five**

PW05: So, me as number five, in this question when I answer a little, in a year I have a target with this job. I can say that this time I had nothing I want to work hard to buy something. When a year ends I buy a chair and put it in my house. It can be that I don’t have a mattress I can buy. According to savings, the way that the budget is it will force me to save a little by little and I say that when this year ends even me I should change when I have a mattress so that is the way I can answer you

**I: Yes, is there any other ideas a year does not end when you have not bought which things. Okay fine if there is none I thank you so much. And women who do sex work always get their money from where most of the time? Yes, number four**

PW04: From sponsors

**I: The sponsors is from number four, yes number six?**

PW06: Me as number six according to me, I target peoples’ husband who are in marriage because they are very desperate, today economy has risen, the second thing is that a house wife will be close to you the first months after giving birth the love goes back to the child and they leave the husbands. When it reaches evening she calls you father so and so. As for me we call them babe, the wife does not have time to massage and a man is a child he needs those petty things, petty love things. So when we sit down we discuss that this time how we are going to snatch people’s husbands in the name of when he tells you like me number six, ‘number six I love you’ I reply with ‘I love your money too’ he will just think I said ‘I love you too’ it his money that I want so I will babe him, massage him even bathe him but my target is his money. so as for me peoples husbands the work is little he have sex with me a little but the money is more but for youths the work is more and the pay is little, so me I will go with peoples husbands

**I: So from clients is where you get your money?**

PW06: Yes

**I: Okay. Number five**

PW05: From the question that you have asked, me as number five I am speaking on my behalf now I deal with those people who have wives that are mature. Do you know why? Youths, let’s say the motorcycle riders, they will come to you when the school is opened because when the school are closed they get little girls that eat chips and soda and they go. Tomorrow you are given two hundred shillings, you will buy a nail polish of twenty shillings a panty of thirty shillings and the remaining ten shillings is for PK you chew you go and the budget has ended. As for me I will need a person who is matured because I have to pay rent, I have children, I have my own life, so for me I depend on peoples’ husbands.

**I: Okay thank you, apart from the sponsors, where do they get their money? Apart from the sponsors, clients, where do they get the money they use?**

PW03: Okay me as number three more so myself I can get a customer and gives me money and then I can start a business so those little amount that I get I put them on my business and if it flops then I go back to the sponsor

**I: Okay, that is an opinion of number three thank you, number nine.**

PW09: Me as number nine there are people that we have kept aside, they are on the list though they are not there. So at time I can wake up and call one of them that am sick, he sends me two thousand shillings. Another person I can ask for one hundred shillings, those ones are aside it is not a must I have sex with them but they are also on the line

**I: Okay, so they are not clients**

PW09: They are not clients

**I: Okay they have their own duties**

PW09: Yes, ‘crush’

**I: Okay where do some get their money from? Number eight, apart from the clients where do girls get their money from?**

PW08: Okay me as number eight apart from the clients or sponsors there is a small business that I might have opened somewhere and I have put a person there to look after it so when I see the business is not working out well I go to my business take some little money it helps me.

**I: Okay**

PW08: Yeah

**I: Okay, we have heard that some people will buy shoes and clothes every day, every week and every month, why do girls spend their money on such things?**

R: Pardon?

**I: Why do girls buy the things that they buy, the reason they buy things that they buy? number five.**

PW05: They will have to buy because they are in the market, even you now when you open a business to buy kales you will be forced to wash them for that person to see so that they can buy. That is the reason why we are spending a lot

**I: Number one**

PW01: Me as number one girls love to buy clothes that can attract those men, when you dress nicely or when you wear another clothe and it looks good on you when you walk there, they will only see you, so they want to look smart so that they attract those clients.

**I: Okay apart from clothes, the other things that they buy why do they buy them**

PW03: Me as number three we buy those things especially the ones from second hand because there are sponsors who come to the house and you know the men competition your house should be clean when a person enters there he sees that it is smart another thing that attracts us to buy is that we put ourselves in a luxurious life that when a person sees they say that this person even though she is into prostitution her money works.

**I: And buying those luxurious things you can spend like how much?**

PW03: In a year or?

**I: Any time that you have bought it.**

PW03: You know it is not that you have got something like forty thousand shillings, you look for sponsors who can give you that amount of money. You know for us we don’t have stress of buying things, the sponsor and the other person will give that money you just go to them and call them ‘babes’ when you have already said that you know that the money will come you know for us we always don’t want a lot of stress.

**I: Okay and the women who do sex work do they save? yes number six**

PW06: Me as number six, let us say the truth, you are the first organization we have seen to come with that idea, and we congratulate you for that on behalf of the people who have not heard it. It is a unique idea so far. Mostly we are just told to use condom or PrEP, things like that. So after we have worn condom and used Prep, what about tomorrow, we have never been told that. So I appraise impact so much for coming up with such a research, we are willing and we have seen that we are just normal people and we can change our lives. Then we do not save money, back to your question, knowing that tomorrow, this our work is more of gambling, you know we have we have many clients, not just one, a wife will stress that the husband has not provided, but we hustle and get even two hundred shillings, tomorrow I get two thousand shillings and balance. So we do not save, why should we save when we have our bodies, I will just sell my body and get money again. So our work is to get the money eat, buy clothes, pay rent on time. We do not save, you people are the ones that will help us to save.

**I: Okay that is an idea of number six, are there others who save?**

PW03: Me as number three I cannot say that I always save because I don’t see the reason for saving and I myself I have carried a bank so I know when I go today in my bank I will withdraw the amount of money and when it reaches tomorrow when time reaches I will go deposit and again withdraw all of it so savings for us is not easy I have seen JItegemee that will help us at least it has brought for us an information that we did not have.

**I: That is number six’s opinion, others who save?**

PW03: Me as number three, I cannot say that I always save because I do not see the reason for saving and I myself I have carried a bank so I know when I go today in my bank I will withdraw all the amount of money and when it reaches tomorrow when the time reaches I will go deposit and again withdraw all of it so savings for us is not easy I have seen Jitegemee that will help us at least it has bought for us an information.

**I: Okay number five**

PW05: In that opinion, I don’t save I cannot lie to myself. You will get someone go with him and then he gives you three hundred shillings, there is no way you can save, because maybe you did not have everything In the house, you will be forced to use it up. Plus that money isn’t legit, because maybe you were given unwillingly, maybe he gave you the money while drunk. He gave you maybe two thousand shillings, you will just go. You end up not saving anything.

**I: Okay meaning that the people who are quiet always save?**

R: No

**I: Why do you save?**

R: We don’t save

**I: Who are you that don’t save?**

PW07: Me as number seven I don’t save.

**I: Okay number one why do you save?**

PW01: Me as number one I don’t save. You just find that the money has ended so we depend on tomorrow that we will get some

**I: Number eight why do you save?**

PW08: Me as number eight I cannot lie, I have never saved because when you say you save, there is no way it will help you because you will just find that it is finished. You find you are empty, tomorrow you will have some more. So there is no need to save. So now that you have come with this information, it will help us.

**I: Okay**

PW08: Yeah

**I: Okay number two why do you save, the reasons that you save?**

PW02: Me as number two I have never saved

**I: Okay nobody has saved, number ten why do you save**

PW10: Me as number ten I have never saved when I get it I use all of it

**I: So nobody is saving, okay what is the behavior of people who don’t save now that nobody is saving what are their behaviors?**

**NT: Here people do not have groups?**

R: What groups?

R: The… (inaudible)

**NT: Merry-go-round**

R: Merry-go-round?

PW03: Okay me as number three, we as sex workers we always don’t go to those Merry-go-round because mostly those Merry-go-round when it reaches December you are given your money and imagine for you, you were having sex, you take your money to the Merry-go-round and then another woman uses that money, so those groups we have never joined my friend

**I: All of us nobody is in a group?**

R: Yes

**I: Number five**

PW05: It is not that it is difficult to join, but most of them despise the sex workers. When you join their group and they hear the type of work that you do, they say that you are a prostitute we cannot stay with them, most of them refuse. Also we desire if we can build our own group the way we are here it can help us a little.

**I: And do you have your own group, there is none like that?**

R: There is none

PW06: Me as number six, I am very happy and, I will depend on myself. Now we know that… okay so far I have heard that commercial sex workers have certificates, in large towns and here at =Sega Siaya= we are the ones who hide ourselves. Me on my side I will volunteer to even mobilize my fellows to start a Merry-go-round like those other people through you. So that we can know today I have gone and got how many, we create our own Merry-go-round. From that chama now we can give one person, if it is a certain number. The problem is we do not trust each other because we are mobile. Today am I go to =siaya=, I will just pass through. At times we even leave our kids in the house, we leave money behind, we buy and leave things and leave for them because you do not know if the client you got today you can even go up to =Mombasa= or you sleep in =Siaya= or even =Kisumu=, you will find another person in =Kisumu=. So from the information you have given us, we are willing so much, so that we can also have a savings group for the prostitutes, and we can save and know that we can have an emergency, in case one of us gets a funeral, we can depend on ourselves because we are looked down upon.

**I: Okay, thank you and have you ever seen those people who save that we know, friends, yes number four, you know?**

PW04: Yes

**I: They have which behavior, which behavior do they have, those who save it’s like which one?**

PW06: Me as number six in sex work there are classes, it is like a business where there is wholesale, retail like that. I can give an example like, we also have lower class sex workers, there are those for *chang’aa, busaa, [local brews]* the ones of bar and the VIP. There is no way we can come from the bushes, we target trailer drivers, and go to Topsy with your lower class prostitution, and get someone there, it is not easy. So those who save, we fear them. Because they spend with people who have money. They have bought plots from prostitution, they have taxis some of them even have rentals. So they started long time ago, it is like they got an upper hand or someone gave them an idea. So for us we love what jitegemee has given us, so that we can also be like them. They are our role models we admire them but we don’t know how they got where they are. We have never had the idea that you have given us today.

**I: Okay thank you, when you say ‘Topsy’ where is it?**

PW06: It is amongst the best restaurants

**I: I thought it is a slang that I don’t understand, okay any other behaviors that they have, number four**

R: Those who, ooh sorry, number four

**I: Number four**

PW04: Me as number four I have never seen a person who saves

**I: Okay, yes number five**

PW05: Me as number five we have seen them but they are ahead, not just a little but so much. Even if one of the passes on, there is a way that their responsibilities is better than ours.

**I: Okay and yes number eight**

PW08: Okay those who save, me as number eight I have seen them like =Busia=, there are those who call themselves sex workers like on 3^rd^ of March they always have their own parties that they do for celebrating so I have seen them a lot they are of high standards.

**I: Yes and those who don’t save they have which types of behavior**

PW08: Like us

**I: Like you, what do you mean by that? Number nine?**

PW09: Us we are team of begging, team of selling, team eating.

**I: Okay that is number nine opinion, any other person? Number seven**

PW07: Me as number seven I don’t save because let us say the needs are many and then I want to beautify myself and the money that is remaining to save is not there.

**I: Okay, number five**

PW05: Me as number five we as the people who do not save, we know ourselves as those who don’t have progress, we cannot move to a step we will just remain there without saving

**I: Okay**

PW05: Yes

**I: Are there any challenges those who save pass through?**

PW03: Me as number three we don’t know because for us you have heard we did not know if there is a thing like savings, so I do not know what to say because we have never saved.

**I: And is there something that makes it easy for them to save?**

R: Maybe there is a thing that made them have the idea to save, maybe they saw somewhere and decided to save or maybe something happened to one of them and they decided that on a sacco when they save they can help themselves. So for us we are just doing it getting it and eating

**I: Okay number ten what makes it easy for them to save**

PW10: Me as number ten who have never saved because when I get it I use it immediately

**I: And those who save what makes it easy for them to save**

PW10: For them they have their ways up there.

**I: You have never spoken to one of them?**

PW10: Yeah

**I: Okay, and to those who do sex work who don’t save, number seven has already told us that the demands might be high. Is there any other challenge or are there any other things that makes them not to save apart from demand, what else? Yes number five**

PW05: Me as number five a thing that can make me not to save the money maybe a person gave you the money when they were drunk and when you look at this person you see that alcohol is what made him give that amount of money, I will be forced to use it faster so that when am asked tomorrow he finds that I don’t have it.

**I: Okay**

PW05: Yes

**I: Okay, number two**

PW02: Me as number two many times I have gone with a person and he gives you a little amount of money and you see that this I will go and buy food with it and become satisfied then it ends like, that you cannot get money to save

**I: Number four disadvantages of not saving?**

PW04: Disadvantages of?

**I: Not saving**

PW04: At times you go broke you have nothing

**I: Okay any other disadvantage? number one**

PW01: The disadvantages of not saving me as number one I see that when I don’t save there is a time that I will not have any money and I have no place that I can depend on so there, I will be forced to stay like that.

**I: Okay number seven**

PW07: Me as number seven the disadvantages of not saving let’s say you know every day, everything comes on its day. Let’s say there is an emergency that can happen, let us say you have not saved then you find that you did not get a client you are stranded

**I: Okay**

PW07: Yes

**I: Thank you, number five**

PW05: Me as number five that question that you have asked it’s like you have seen me somewhere. The previous day I did not know that not saving is a bad thing and the way that they needed rent, that is when I realized I should be saving at least 20 shillings. That day is the day I got to know the advantage of saving and the disadvantage of not saving.

**I: Okay**

PW05: Yes

**I: And you have said that you were?**

PW05: The rent was being needed

**I: Okay, sorry I did not hear you very well. Okay, thank you. Number nine? Disadvantage of not saving?**

PW09: I am just supporting number seven, about emergency. You may have a funeral, and there is no way you can help, so that emergency can occur at a wrong time and you don’t have anything

**I: Okay. Where do female sex workers typically save? Let us start with number four.**

PW04: Me as number four, not saving?

**I: Saving, where do they save?**

PW04: Me as a person, I do not save

**I: If you were to save, where would you save?**

PW04: There is Mpesa, locked account.

**I: Number seven**

PW07: Me as number seven, if I want to save like you have taught me, I can open an account, let us say equity bank.

**I: Okay, number one**

PW01: Me as number one, I see that we can form group and start saving. Because in the groups that have been registered, if you save you can be given a loan even with the government.

**I: So like a savings group?**

PW01: Yes

**I: Okay number eight**

PW08: Me as number eight If I were to save, I would open an account with co-operative bank, equity, family bank

**I: A bank?**

PW08: Yes

**I: Yes, number five**

PW05: I would save in Mpesa

**I: Mpesa, number two**

PW02: Me as number two, if I were to save I would save in a lock account

**I: Lock account?**

PW02: Yes

**I: Okay, number six**

PW06: Now that we as the sex workers, we usually do not know where each one of us come from. We just meet at the field. The time we come from work [inaudible]. So wherever your colleague will say, you go. So I think it is I save in MPesa on my own

**I: Okay, number three**

PW03: Me as number three, if I were to save, I don’t know what to say because even if I decide to save on my phone, I would still withdraw the money. So I think that the idea of Jitegemee can work for us, at least you have somewhere that you can save your money. You just set a duration that you can withdraw the money. That can help. Maybe we can start a sacco save.

**I: Sacco. Okay number 10**

PW010: Me as number ten, I can save in Mpesa, so that in case of any problem, I have somewhere I can get money

**I: Okay, number nine.**

PW09: In a bank

**I: In a bank. So why do we prefer the places we have said? Yes number seven**

PW07: Me as number seven, I prefer a bank, opening an account because let us say I save in mpesa, I may want credit, let us say I do not have a client and I want to consult my friend, I want credit, I want bundles, let us say I see something that has attracted me, I will withdraw that money. So I prefer a bank.

**I: Okay, number four**

PW04: Me as number four, I can say that I prefer saving in locked account because once you save money there. First if you go save money there, you will be told to set a period. So let us say you have set a period of one year, so if you have no money at all, you cannot withdraw. The period you set must expire before you access that money.

**I: Okay, another person? Number two why do you prefer the place you said?**

PW02: I support what number four said.

**I: Remind me what she said**

PW02: She said that locked account, you can withdraw money that first. You must just withdraw the money the date that you had initially set.

**I: Okay, number one**

PW01: Me as number one, I prefer saving in a group, it has to be a registered group so if it is registered it would be recognized by the government and there is a loan that is usually issued, we can apply as a group and we are given to use.

**I: Okay thank you. Do FSW live typically beyond their means? I mean where they spend more than they earn? Maybe you earn 3k but you live the life of a person earning 10k, are there people that live in such a life?**

PW04: Me as number four, I say like yes, those sponsors usually have money, so yes. Like me I live in higher life.

**I: Why do you live in high standard?**

PW04: Because sponsors have money. A sponsor cannot give you two hundred shillings, they will give 10k

**I: So you live beyond that 10k?**

PW04: Yes, I live higher, second floor

**I: Okay number five**

PW05: Me as number five can say you live according to your market and your target. Me as number five I cannot live higher than my earning. I cannot compare myself to someone who do not have a kid, because if I get a thousand shillings I will share it with my kids. If someone who does not have a kid gets a thousand shillings, it their budget on their own, so we cannot be the same.

**I: So you live a life that is in accordance to your earning?**

PW05: Yes

**I: Okay thank you. Number three**

PW03: Me as number three, I cannot live a life…as in the amount I earn, I cannot live in that standard. I must find a sponsor that is living a posh life, so that I can also live that high life. That is when I will enjoy life. So my earning is just mine but life, I must elevate it, I cannot lower my standards.

**I: Okay and let us say the sponsors are not there, something is not there, how do you cope? You have said you live a life that is beyond your means?**

R: Yes

**I: How do you cope?**

PW04: Me as number four, pardon…

**I: You live a life that is beyond your means right?**

PW04: Yes

**I: It means that you can have debts that is an example. So how do you cope with that?**

PW03: Let us say. Me as number three, let us say this month, this sponsor will not be around, I will calculate my next move. I will look for those people that can pay for that budget until it reaches 10k, this month I must just get that money. There is no day that I will lower my life. My budget is my budget I must boost it because I cannot live a low life. So if my sponsor will not be there… you know these sponsors are good people, they just send money to your mpesa so I do not see any stress. So if he is not there, you know you can also have another sponsor, you can mix, we do mix them.

**I: So number three mixes them. Another person, how do you fill that remaining space? Number eight? Nothing, number five?**

PW05: Me as number five, if I live a higher life and a sponsor belongs to someone. Remember that he will go and I will remain living my life. It will force me to [inaudible] it will force me to steal for me to live that life. Because even if it is a man, you will not get him today and convince him on a budget that he did not have I mind. Let us say you have been living in a house that costs 3k, your salary is 1500 shillings, and the man has left, and you have to pay for your rent, it will force to steal.

**I: Another thing someone can do to fil that space?**

R: Another thing is, I have tried and I have not managed. I know that if I go to a certain place people drink different drinks. There is also a story of drugs and pick pocketing.

**I: So for you to fill that space?**

R: Yes, I must just live that life.

**I: Okay. Do female sex workers borrow money? You borrow money.**

PW03: Me as number three, I must just borrow money because I know if I borrow… I tell them borrow me three thousand shillings today, if I know that today the field is not good and that money is used for emergency, and I don’t save so it would be used for emergency. Let me tell you, you see these debtors, they give sex workers money fast because they know that tomorrow that money would be refunded. So I borrow.

**I: Who do you borrow from?**

PW03: From mpesa. There are also these people that give out loans you pay daily. How are they called? So I just tell them ‘give me 3k I will refund with an interest’ so they have that morale that ‘if I give her money she will refund’

**I: If you borrow, you borrow to use it for what?**

PW03: In case of emergencies. Maybe today I have gone to work and I fail to get a client, my kid cannot sleep hungry, I cannot also sleep hungry and I cannot fail to dress myself, so I borrow and use.

**I: Okay, number five.**

PW05: Number five only had a question. I did not understand on the issue of borrowing, I borrow from someone…

**I: You as a person borrow, you borrow. Isn’t that borrowing right?**

R: Yes

**I: Not give out money, you borrow. Number two**

PW02: Me as number two, I also borrow money from people, I go to my local shop and tell them give me 500 shillings I will refund

**I: You borrow from who?**

PW02: Even from shops, hotels

**I: To use for what?**

PW02: If I go to the field and fail I must go and borrow, tomorrow I refund.

**I: Okay, number one.**

PW01: Me as number one I have a side hustle so if I fail to get money this other side, I can go borrow from there, if find I refund

**I: If you borrow, what do you do to refund?**

PW03: Me as number three, if I borrow three thousand today I make sure I get a sponsor by evening the next day. Even if it will be a shot of a thousand shillings each, I must refund that three thousand shillings. I borrow find a sponsor to pay that money. Why should I stress myself and I have an ATM.

**I: Yes, number four**

PW04: Me as number four, if I borrow money obviously I will pay because I am good at conning people

**I: You con? How do you con them?**

PW04: On my phone

**I: Okay, another thing that someone does to pay debt?**

R: I support what the others have said

**I: What have they said?**

R: Good at conning, [name omitted] here

R: Use numbers

R: Number three and five. They have said they look for sponsors

**I: Do not sleep too much okay? Number five**

PW05: Me as number five, you heard me say in the beginning that I cannot live a life that… I will borrow, let us say I earn 15k, I cannot borrow more than 15k, I will borrow in accordance to my salary. I cannot borrow then depend on someone else to come pay it off, I will borrow, use it the use my salary to pay.

**I: So if you get your salary you refund?**

PW05: Yes

**I: And if you borrow, why do you borrow?**

PW05: I can borrow to pay rent I can borrow maybe my kid has been sent home for school fees. I will borrow to pay for the school fees, or I can borrow when I do not have food in the house.

**I: Thank you. Are there things women do to increase their income? What things do they do to increase their income?**

PW03: Me as number three apart from sex work?

**I: Yes apart from that**.

PW03: Me as number three, at times you have to hustle to add on the money to boost your income.

**I: If you hustle, what do you do?**

PW03: You can go to the salon. Like me I usually go to the salon. If I go there and find a piece of fifty shillings, I go and sell it for one hundred shillings, with that I have a profit of 50 shillings

**I: Another thing they do to increase their income? Number five**

PW05: Me as number five, I am employed as a nanny. So what I can do, I usually do peoples laundry. Someone can call me that maybe they are moving into a new house, they call me, ‘number five come and mop my house’, with that I have two hundred shillings. Someone can call at the hotel that ‘come and wash these plates’ maybe you are employed at the hotel and you are tired you will come me, ‘number five are you free there, come and wash these plates.’ I go wash the plates and am paid.

**I: Per day if you had a debt, how much would you have? Number four**

PW04: Me as number four if I have a debt?

**I: Yes, like how much?**

PW04: Three thousand

**I: Okay number nine?**

PW09: in a month or?

**I: Any. Anytime you have a debt, how much is it?**

PW09: Six

**I: Six what, a hundred or?**

PW09: Six thousand

**I: Okay number 10, how much debt can you have?**

PW10: Me as number ten, I can have a debt of two thousand or one thousand five hundred shillings.

**I: Okay, number three**

PW03: Me as number three, I can have a debt of five thousand

**I: Okay, number seven?**

PW07: Me as number seven, I can have a debt of 4k or 3k

**I: Okay, number one?**

PW01: Minimal is 8k

I: Okay, number eight

PW08: The minimal is 6k

**I: Okay number 2**

PW02: The minimal amount is 2k

**I: Number five**

PW05: I will that of number two

**I: Which one**

PW05: Two thousand

**I: Okay thank you. Do sex workers ever think of when they might leave sex work?**

PW03: Me as number three, I think of that every morning when I wake up.

**I: What makes you think?**

PW03: Because I am tired, I think am getting tired of it. But the thing is even I leave it, I have no employment, it will force me to do it. But I will endure. The things we experience are not things that you can like, at times you go to this work it forces to, but what we go through can make… you can sit down and say I want to leave this work and maybe start my own business. There are many that have left ventured into business

**I: Is it something that you have thought of on your own or you talk about it with your friends**

PW03: I think about it on my own

**I: Okay, number five**

PW05: Me as number five, I have thought about it for long, because there are challenges. Like me, there are times there are no clients, you can find someone and they are stronger than you, and he gives you a small amount of money. And then again you can… like me the other day I sat with a friend with mine, she is also in this group, I told her ‘my kids are growing and they will mature, and they will see some picture that this woman is doing this to feed us’ so you know you cannot scold your kid, if you scold them they will tell you, ‘mum you are scolding and you also do the same thing.’ So I talked about that, even yesterday we talked, I said, this work even though we are doing it, you find someone that is the same age as your father, it will force to just go with him, but you might wish to give back that money. He will give you ‘’mzigo’’ that if you look at yourself, you just wish God would rescue from this work, and I get a business to sell even kales and feed my children. So it is something that I have thought about so many times.

**I: So your kids are the ones making you think of quitting?**

PW05: Going through challenges. I told you that you can get ‘’mzigo’’ that you might wish to refund that 2k

**I: What does ‘’mzigo’’ mean?**

PW05: A big penis (participants laugh)

**I: So size?**

PW05: Yes

**I: Another reason that can make someone quit sex work? Number nine**

PW09: Me as number nine, I have thought about quitting sex work because family… maybe you want to settle down and have a family. So that can make or it can discourage me do this work

**I: Number ten?**

PW10: Number ten I have forgotten…

**I: Number ten, stand up a little, we are moving on well**

PW10: Yes

**I: Number seven, what makes people think of quitting sex work?**

PW07: Me as number seven, I usually think of leaving it because… I’ll talk about challenges, the challenges that are there. At times I can say you have met someone, if you look at him, he has money, the money is there, but if you look at him age wise, you are like, ‘this is my fathers’ age mate’ but now you just have to go with him because of money but it is not good. So I can say the challenges out there are what are making us quit

**I: So the one that you are saying the client can be your fathers’ age mate, is it one challenge**

PW07: It is one of them. There are so many challenges. Let us say you have fallen sick and that is the only way you can get money, so that is also a challenge, because the body now… you are tired you cannot do anything. So let us say if you had that other of kiosk, you can go.

**I: Female sex workers typically leave sex work at what age? At what age?**

PW03: Me as number three, I don’t think people ever quit this work. Because at times if you go to work [inaudible] if you look at some people, you just know that this work has no age limit, we are mixed. Until you just tell yourself this thing has no age limit. It is not like government employment where there is retirement, here you work as long as you are active. Because maybe you have sat somewhere, you are looking at one two three, you just talk among your friends like, ‘this one looks like my grandmother’ but she has pimped herself she is just her own class. So I don’t think sex work has an age limit.

**I: Another person. Number eight. Is age a reason to quit sex work?**

PW08: Me as number eight, that thing does not have an age. Like they usually say an old cat also drinks milk. Because you can come from somewhere and you find someone that is your mothers’ or you grandmothers’ age mate, what will you say? So it does not have age limit

**I: Okay. What type of job do FSW do after quitting sex work? Number ten? The ones that have quit sex work, what business do they do?**

PW010: Me as number ten, I have not seen someone that has quit sex work and is doing business.

**I: Number four, what type of work can they do?**

PW04: Me as number four I do not know.

**I: Number seven?**

PW07: Me as number seven, according to the people that we have seen quit that job, I can say that like you know when you were in the field, you know you used to make up yourself. You know how to dress. You can find someone has quit and she had an idea, she has opened a cosmetic shop. She is into beauty, something like that.

**I: Yes, number five**

PW05: Me as number five, many of those who quit that I have seen, someone quits and if she had the urge to save like you have taught us, she can open a cafeteria, lodgings and her own bar. If she was smart, but if you do not save, you will just cut kales by the roadside, sell soda and water because you were not smart. I usually hear them say, the ones that have opened their businesses, I usually sit with them. I even sit with those that are older than me and they tell me, ‘number five, there are challenges in that field, you will get a disease that you will live with forever. So if you do not want to contract that disease, according to the type of work that you do, you open your mind, have savings, take drugs, you also have a kiosk that you can have a little income from. So while you take your drugs, your kiosk is also earning you a little money. And if you do not have the brain, you will die faster because you will go sell water and soda, have stress and you will tell yourself, ‘I did this work but I came out with nothing while my colleagues have shops, have big hotels.’ You just die of stress

**I: Okay number three?**

PW03: There are also some of them, let us say like for example they have quit sex work, you will see they live an expensive life, she has her house there, you will wonder. This is something I have seen from one of them. You will wonder why this person has pretty friends, so one day I decided I want to follow up. So it reaches a point where this person acts like they are sponsoring something. So if she has friends, she gives them the girls, like they give company to her friends. So if someone goes there, there is your share and the share the person that took you there.

**I: Okay so she is like a pimp?**

PW03: Yes

**I: Okay, thank you. Do you know a time that sex workers quit sex work? The ones that quit sex work, is there anyone here who knows someone that quit sex work?**

R: Yeah

**I: Alright…**

R: Can I mention their name?

**I: Do not mention name**

R: There is one that I know that has opened her hotel. She also employed me in that hotel.

**I: Why reason made her quit sex work?**

R: Those challenges that I had told you

**I: Okay. Can you remind me the challenges?**

R: Should I repeat them?

**I: Yes the challenges**

R: The challenges that I said?

**I: Yes the challenges that made her quit sex work**

R: I sat with her and she told me, we usually make stories. Because they say one finger cannot… you must take one finger to aid the other finger. So she told me ‘number five, what made me quit sex work is, but now she used to save. She told me she got a visitor, she went with the visitor. So the visitor wanted to have sex with her without protection, she refused. The man pointed a gun and knife at her, placed them on the bed. That man… she had to accept saying ‘instead of killing me just do what you want then go.’ When that man withdrew his penis, it had turned red, so he slept with her and gave her 5k telling her, ‘use two thousand, the remaining three thousand use that for your funeral arrangement. That is what made her quit.

**I: Another person that knows anyone that has quit sex work? Number nine**

PW09: There is someone that has quit and she has opened a salon. The reason why she quit that she was saying was that she had been conned. She went with a man, they finished their business then the man sent her money then reversed it. So it turned into a communication where she called Safaricom customer care, but her money was not refunded. So she said like she would be [inaudible] so she gave up.

**I: Number two**

PW02: Me as number two, there is a friend of mine that used to do that work. She started with a sponsor. That sponsor told her that he would give her ten thousand. So she thought that the sponsor would sleep with her but he did not. He went ahead and opened a friend that had a corpse inside. So she was told to clean the corpse and oil the body. From that day she said she would never do that work. That is how she quit.

**I: Okay number one?**

PW01: There is one that I know that quit. What made her quit was she used to bring sponsors to her house and one day the sponsors met. So when they met, they fought and one of them was cut at the back of the head, then the other one cut the other ones’ hand. So it forced her quit that work and she went and opened her own bar.

**I: So the sponsors met at her house?**

PW01: Yes, they met.

**I: Okay. Do we know the ones that quit and then came back?**

R: Yes

**I: Yes number three?**

PW03: I know of one. She told me ‘I quit you know this and this and this…’ then another day I met her and told me, ‘this work even if you quit, you just come back.’ So there many that quit and come back

**I: Their reasons for coming back?**

PW03: Being broke (participants laugh)

**I: Another person who knows one who quit and came back? Number five**

PW05: There are those that quit knowing that they have opened their businesses. But with business if you are not smart, it cannot go well. If it flops, she thinks of the way she got money and opened that business, then she goes back to be able stand on her feet again. That is what makes them come back.

**I: Her business flopped, okay. Number seven**

PW07: Me as number seven I have a friend that quit then came back. She said that out there, she just quit due to stress but she came back. She told me that she could get that money fast. Let us say in a day she can make a lot of money compared to what she is earning now.

**I: So the money that she is getting right now is not enough compared to the one she used to get during her sex work days?**

PW07: Yes, plus it is fast money.

**I: Okay. Is there something negative that happened when they came back? For those that know the ones that came back. Negative things**

PW07: That they got when out there or when they came back?

**I: The ones that came back**

PW07: Negative things… I do not see any. My friend that came back is even wiser now.

**I: That is number seven. Tee good things that happened when they came back**

PW07: Good things? Me as number seven I can say that there are good things because, let us say my friend that came back, when she quit and came back she got to know about PEP and PrEP. At least I usually see her use them saying that she cares about her life.

**I: Number five, a good thing that happened or bad? When she came** **back**

PW05: There is no good thing, what I can say is there are people that did not see her so she came back as a new person. So she got more money than the people she got there.

**I: Are there things that we would want to accomplish before we leave sex work? Are there target put in place before quitting sex work? Number five**

PW05: Now that is [inaudible] like me I have kids. So my target, by the time am quitting, I should have my piece of land. So that I have a place of my own with my kids. That is my target

**I: Have you placed your plans down already?**

PW05: Yes, I have already started doing them.

**I: For you to get to your target?**

PW05: Yeah

**I: Okay number nine, the target you have before quitting sex work?**

PW09: My target before I quit, I expect my life to be better than now. Like I can wake up in the morning and my kids can eat. I have paid my rent. So like I can move on with life.

**I: Have you started planning for that. Have placed down those plans?**

PW09: That is what I have just thought of

**I: You have thought of right now?**

PW09: Yes

**I: okay, number four? Your plans before quitting sex work? If you have any?**

PW04: I don’t have any

**I: Number four has no plans. Number seven, any plans you have?**

PW07: Me as number seven, according this program you have brought us today, Jitegemee, at least according to the questions and these things I have been thinking, at least I can have a plan in that before I retire I can… my life can change in that if I wake up in the morning with my kids, I just make a phone call and money is brought me, I have opened a business somewhere, I have employed people

**I: So you wish to have a business**

PW07: Yes

**I: Is it something you have started planning on or…**

PW07: I have just thought about it. When you came is when I thought of how my life can be

**I: Okay you have thought about it now, number ten, what you would wish to accomplish before quitting sex work?**

PW010: Me as number ten I just wish to get money, open up an Mpesa shop somewhere. If the money is enough I can buy a place like my friend there said.

**I: Have you started planning on that?**

PW010: Not yet. I want us to form a group so that we can start.

**I: Okay number two, any plans?**

PW02: Me as number two, I have a plan. I want us to go with my kids to my rural home, start farming or a business, so I just go there.

**I: Have you started planning towards that?**

PW02: Yes

**I: Okay number eight, any plans?**

PW08: Me as number eight, I have that plan, I had started planning on that. Before I started, I had opened up a business that can help me with my kids. So that even if we wake in the morning and my kid asks for something, I just make a phone call, if it is chocolate it is brought.

**I: You have started planning on that?**

PW08: Yes

**I: Okay, number one, any plan?**

PW01: Me as number one, already I have started my own business but I want it to expand in a way that it can help me with my kids

**I: You have started working towards its expansion?**

PW01: Yes

**I: Okay. Number three any plans before you quit sex work?**

PW03: Before I quit sex work?

**I: Yes**

PW03: I had a plan that before I quit sex work [inaudible] I should have my piece of land a car is a must. My kids must all be learned.

**I: Have you started planning towards that?**

PW03: I have not been saving so from today I will start saving so that at least my plans can go well.

**I: You have just thought about it?**

PW03: Yes, I have just thought that savings is good

**I: Okay thank you. We are going to the last section about jitegemee that we had talked about. Earlier on I had described jitegemee intervention and I emphasized that the purpose is to ensure that sex workers have some savings that would allow them to say no to unsafe sex or to take a break from sex work if they want yo rest. I also said it will entail FSW saving a small bit of their own money to use when there are no clients or help them prepare for life after sex work. So like we had said, do you think Jitegemee is something that female sex workers would embrace? Do you think it is something they would accept?**

PW07: Me as number seven. According to my understanding it is something that they would accept because it is something that would build you. The word JItegemee means depending on yourself, they will accept because they would know how to save, how they can prepare for life after sex work.

**I: Number eight do you think it is something they would accept?**

PW08: Me as number eight, the life of depending on yourself is something that will be accepted. Because it can help your life in future.

**I: Okay, and what type of women would accept it?**

PW03: Me as number three, you know like you asked us if we save then we told you that we do not save. Because we did not know the reason as to why we should save and how it can be of help. But for people like us who do not save, we have to accept at first because I will have to know if I save my money there, it is safe. If I save on phone I can use. Then again there is that idea, like there is something in impact that usually help FSW to save. Maybe if she was saving in another sacco, she would want to join that sacco because she has the knowledge of saving. So me I see that many sex workers would accept it.

**I: Yes number four?**

PW04: Me as number four, I see that not only the sex workers would accept it but also others like single mothers, eve those that are married. Because there is no way you request money for onion from your husband when you have money saved somewhere. You can withdraw it instead of begging.

**I: Okay number seven**

PW07: Kindly repeat that question

**I: What type of FSW would accept it?**

PW07: Me as number seven, according to me I see that those women that have themes of change, the ones that want to better their future, the ones that have the idea to change, those are the ones that can accept it. Because they have a reason to accept it

**I: Okay number three**

PW03: Okay me as number three if I add to that point of number four where said even the married women. You know in sex work, the married women do not expose themselves that they are married. So if they hear this idea of saving it can help them. Because they are married but they are doing sex work, but the partners do not know that they are sex workers, so if they hear this idea, at least it can help them save. Then for people like us who have sponsors, we cannot refuse to save

**I: Okay, and out of ten women, we all know at least ten girls here who are dong sex work?**

R: Yes

**I: How many out of the ten would accept to join jitegemee? Number nine? Out of the ten that you know**

PW09: Six

**I: Number ten?**

PW10: Six

**I: Number three**

PW03: I do not know. You can say even ten?

**I: Out of ten, if all of them can accept then that okay**

PW03: All of them will accept plus extra.

**I: Okay, number two**

PW02: Me as number ten I can say all of them

**I: Ten. You know all of them?**

PW02: Yes

**I: Okay, out of ten, the one you know would want to join Jitegemee? Number five**

PW05: According to me, I do not see many of them accepting. Because as we are sitting here we know each other, do not hide anything. We are just seated because we respect you, but if we disperse, this person can come and tell someone [inaudible] previously I told you there are some people in this group who are well off, I said that right? My answer is most of them will not accept. Because you will give me money, we will keep it as a group right? You will hear that I have given this person money but I do not trust her. After talking like that as number five, number six will hear then number seven will also hear. So many of them will not accept. What we can say is anyone who is willing can join. But as at now, we cannot know an individuals’ mind

**I: So for the ten that you know, how many can have that heart to join?**

PW05: Five to six

**I: Number eight?**

PW08: Five

**I: Number one?**

PW01: Eight

**I: Number seven**

PW07: Ten

**I: Number four**

PW04: Five

**I: Okay thank you. To those that would not accept, what would make them not accept it? Yes number three**

PW03: Me as number three, maybe we have disagreed with number five, like she is thinking that the money we are saving will be given to [name omitted]

R: Stop calling [name omitted]

PW03: But this saving is like a sacco, I think jitegemee is something like that? So this money there is somewhere we are channeling it, even if I save five hundred this person and this person will not know that I have saved. It is just me and my phone, I think something like that right?

**I: Yes**

PW03: So maybe she is thinking that it is like chama. So maybe or she is saying something that…

**I: It is okay, every ones’ opinion is okay. Opinions are welcomed**

PW03: So I think it is something like that but if it is whereby one person is keeping money for the rest of the group then many people will feel like they don’t … but if the money is being channeled somewhere maybe in some sacco, whereby I send the money I can withdraw and everything. Then that to me, even to those who want to save they will see it is good and save. Because as the person saving I can say let me withdraw some money, we are in the market and there are things am buying. So I think for those that see it hard to save, once they see those that save saving, they would want to join.

**I: It is not like a chama. We are thinking of launching in that it is not like chama, it is individualized. Where were we? The reason why the others would not accept, another reason apart from number three and five? For the ten that we talked about, others said five would accept meaning five would not accept. What would make them not accept? Yes number nine**

PW09: There is ignorance

**I: Ignorance as in?**

PW09: Like there are people that are not smart

**I: Okay. What can we do to increase the acceptability of Jitegemee? Yes, number three**

PW03: Me as number three, what I can do because we are about ten people in here, so I think if really impact and Jitegemee is going to help us. You can go for, there are these things we call training. You pass these idea to people because they do not know. So at least you can say like here if we come we can save fifty shillings, then they can spread the message to other people. So you see you are spreading the news. But if I go as an individual, my understanding with the others are not the same. But if you go as a group and tell them then many of them would want to join.

**I: And if it is training, what can we train them on? Or what topics can we talk about?**

PW03: Okay we can… it is about us as sex workers right?

**I: Yes**

PW03: So you come and tell them like… you want to get the idea from us right?

**I: Yeah**

PW03: ‘You as a sex worker how would you want your life to be?’ I have come with this idea I want you to say, ‘would you want to save or not.’ You can tell them the benefits of saving. You can come with an example of a person who has been saving. So if I see like today you have talked to number four, this number four has progressed. So I will say that I must do the same thing so that we are the same level of her or even surpass her. So if you tell them they can join. You know the sex work of this side, let us not lie to ourselves, we cannot compare it to that of town. Like in town someone just tells you what’s the big deal, but this other side you will find that someone is afraid. Yes she is a worker but she won’t easily accept that she is a sex worker. Someone would tell her, ‘you know this is just like any other work that someone can do.’ So if you train them they know it is something that is wrong. Apart from that, after they have saved, people will see that they have progressed. You know there are people that would [inaudible] they do not want to be like us. So you see this thing will be growing, because it will attract even those people that did not want to join

**I: Thank you. Another thing we can do for people to accept Jitegemee?**

R: If you can get people that have been in that job and they are successful, they can come talk to people

**I: Okay. Another we can do for it to be accepted? Number six welcome back, we were talking about Jitegemee. So we are thinking of things we can do so that girls can accept it. Yes, that is what we are talking about. Another thing that we can do for girls to accept? Sugar levels are low, number seven don’t worry we are almost finishing. Number five**

PW05: What we can do for it to be accepted, if you can encourage us and then we grow. So that the people that saw us when we started saving, can see through us

**I: Okay thank you. What should Jitegemee comprise of to be accepted? There are like number three already said training, what else should it have?**

R: Sitting allowance

**I: Okay. What else can make it to be accepted? As in motivate girls to accept it? Number three?**

PW03: Me as number three, what I can request is if jitegemee can make up a group and connect us to a bank for example equity. So if I save there you would not know. If I go to take a loan they will ask if I have a MOU or even a sacco, I can prefer this one because I know if you take a loan from the bank and you signed as a MOU and if you do not have something in the account, they can track you with the information you have given. So that can even boost. By the way we want to elevate our life, we live a life that at least people can view us as human. If it can have an MOU with any bank, it can make many people join. [Inaudible] if I go to bank I will give details that they can track because with bank you cannot go with their money.

**I: So the MOU should be on what? The understanding should be about?**

PW03: Maybe you may want a certain amount that you can be given, so if you are given they check in this sample, this person has saved this amount, so they have to confirm, they cannot give you this limit when there is this other one. So I save a lot knowing that if I go there I can be given something. So it boosts us

**I: So it will enable you take a loan?**

PW03: Yes

**I: Okay, number one?**

PW01: What I can say is, they should bring trainings. Because we are so many girls and also there are those that are shy, they do not want to be sex workers but they are. So if they attend these trainings at least can help

**I: If we train, what do we train them on?**

PW01: If you train

**I: On what topics, if you can help us with those topics**

PW01: on topics, we want to start on savings. That is what we can talk about and there is the story of sex work, we can teach them that. So like how someone can depend on themselves

**I: Okay thank you. Is there something that FSW would not like about jitegemee? The way we think of doing it, do you think there is something they might not like? Yes number six**

PW06: Me as number six, what we might not like is the duration of meeting us, our time. Because as sex workers, any time that passes that is money wasted. Like we are seated here, they are hurting but they won’t say. Their morning customers are going to other people, so at least you spend a minimal duration, even thirty mins or one hour, we know that we lost something small but we have gained something that will help us in future but if you overspend time, you will not get sex workers.

**I: another thing they will not like in jitegemee? Number ten**

PW10: [Inaudible]

**I: Speak up please**

PW03: Me as number three another thing is… we have said that jitegemee we can join groups after training. You must bring us trainings they will help us because we do not know how to save. So another thing is I will be saving, then there are these things we hear about network, these are the reasons why we do not save, so maybe I have an emergency and am trying to withdraw, but that money takes long, it tells you to wait 24hours when my emergency is that time. Maybe I am somewhere I do not have the fare to come back, I have to get it

**I: Okay. Do you think that this would bring ethical concerns? Or someone would think her rights have been tampered with if she joins jitegemee? Number six**

PW06: At first, if something new is introduced, there must be a big percentage of negativity, but once you get one or two role models from within us, it can motivate us. Because we are called prostitutes but we are sex workers, we fear. But once you have shown us, and we see that we can rest for a certain period of time, even if a man calls us I can decline. We usually do not decline. So if I decline because I have my money, then another person can admire that. ‘If number six can survive, she does not go to bars, she does not sell herself, I can also do the same.’ Then she will come to me as group… we are a group here that you have taught, she can ask ‘how did you manage that.’ She can also refer for the first she sees that she has tried on life. A client has come but she says try number two, ‘number two can you take a client?’ something that has never happened. Then you will find that you as people from jitegemee you are motivated and we are also motivated, then we will be an example to people that are in hiding. They are se workers but they are hiding. They will come out in numbers and see that we are many. So it will give you easier time to get clients.

**I: And if people join jitegemee, do you think that they would think their rights have been tampered with?**

PW06: No, if jitegemee comes with positivity. Not unless you have come to interfere with us, but we are grown up. If someone can have six or seven men, they can service them, then jitegemee comes with good thing, I think we will handle them well.

**I: So there will be no rights lost?**

PW06: No, if there is development.

**I: Okay number six. Number eight do you think they will think they have lost their rights?**

PW08: No

**I: Okay. Number two?**

PW02: No

**I: Number ten**

PW10: There is none

**I: Okay, what challenges will we meet? Number six has already told us one, getting people to talk to, right? Yes another challenge we might face if we want to bring jitegemee?**

R: Another challenge you can get is. If you people come to the ground and do not give people education. An education based on the reason why you are introducing it. We are just ten here, five can accept and the rest can refuse. So at least if you know the way you are going to do it, you will not have challenges. Because we will follow and say we want this, then you do this instead of doing our way you do it your way. But if you come and we share ideas like you want us to do this then we will say this is good, this is also good, so we on both sides we come to agreement.

**I: So that is how we can overcome that challenge?**

R: Yeah

**I: Okay, we talk to people first?**

R: Yes

**I: Okay, number…**

R: The discussion should be on the importance of saving, why you want us to save, our future, and things like that.

**I: Okay thank you. Number three…number five you had raised your hand? Oh number nine, yes?**

PW09: Another challenge can be on you because you will be travelling by car, use petrol. At times you come and you fail to find people. So you might have money problems

**I: We will have money problems?**

PW09: Yeah, because you spend a lot

**I: How can we work on that so that we don’t spend a lot?**

PW09: Make a background of money.

**I: How now?**

PW09: Get donors, NGOs groups and you also have other many supports.

**I: Okay, so we have other backups?**

PW09: Yes.

**I: Thank you, number nine, any other person with a challenge?**

R: Other challenges that you will get as Jitegemee as per your target, as an organization you must have your targeted group, maybe the organization requires you to meet a group of 10 or 30, you communicate and the find sex workers are already with clients which they can’t leave the client to come for this learning. That is so challenging meaning you have to communicate in time to know at what time we can be free or when do we not always have clients, like Monday it’s always so dry. Communication first between the sex workers and you as an organization.

**I: Okay, number eight any challenge we ca face?**

PW08: As number eight challenges are always there….

**I: They are no challenges?**

PW08: There are.

**I: Like which ones?**

PW08: Like number nine has mentioned, things like transport, paraffin, yes.

**I: How would you suggest we address these challenges differently from what number nine has sad?**

PW08: I will just support what she said.

**I: What has she said?**

PW08: Having backups.

**I: Okay having back up, number five.**

PW05: As number five challenges that we call can see, this our group as you can see the name not everyone can volunteer to do what you are doing and not everyone would like to sit with sex workers. There are those who will be against this group of ours and because you agreed to stand with us kindly just stand with us. We also have those who are sex workers and they will not join us, you will be in our group which you agreed, those are challenges you will face and then we have those who will be against the groups.

**I: Being against the group what does it mean, sex workers or being against the group how?**

PW05: The sex work, there is a way people see it at all, other knows it more than you do, let’s just say the truth.

**I: How can we solve this?**

PW05: Now that you agreed to stand with us, continue standing with us.

**I: We said Jitegemee is not like a marry-go-round, it is you to decide to save and how much are you saving, when and how many times. If you decide to join Jitegemee, how much would you be saving in a week? Number four while checking all your daily expenses, you all have responsibilities, right?**

R: (All) Yes

**I: But you have joined Jitegemee and we want you to save, in a week how much can you save?**

PW04: As number four in every week, maybe 1000/= or 500/=.

**I: So, between 1000/= and 500/=?**

PW04: 1000/=

**I: 1000/=. Yes, number seven?**

PW07: As number seven you can’t be sure approximate amount that you will save, like the money you get today and then the expenses are too much. Yes, you will have to reduce the expense to save, I can’t say approximately how much I will save.

**I: We are looking at your daily expenses and you know the exact amount that you always earn. By looking at what you can spare in a week, how much can it be, what can you spare without suffering?**

PW07: At least 25.

**I: 25 What?**

PW07: 2500/=

**I: 2500/= Number one?**

PW01: 800/=

**I: Number eight?**

PW08: 1500/=

**I: Number five.**

PW05: 500/=

**I: Number two.**

PW02: 500/=

**I: Number six.**

PW06: 500/=

**I: Number three.**

PW03: 1000/=

**I: Number ten.**

PW10: 500/=

**I: Number nine.**

PW09: 1000/=

**I:1000/= so those are what we think if we have that chance at least every week you have save right.**

R: (All) Yes.

**I: The target that you have set, in case you don’t meet your target, what are you going to do so that you meet the intended target of that week?**

PW06: As number six there is this thing called fuliza, mshwari and KCB, there are other clients who pay through mobile payment and immediately they pay they might reverse so we always transfer to either mshwari or KCB so in case we don’t meet the target we can loan from those phone sacco. For emergencies there is no where we take it since that is where we keep our money, once you get a client you can refund back.

**I: At least you can borrow and pay your target and then you will pay back?**

PW06: Yes, I will pay back once I get a client.

**I: Okay thank you, what else can someone do to meet their target, yes number seven?**

PW07: You can look for a side hustle.

**I: Side hustle like which one?**

PW07: Like let me say you don’t get a client and you have not met your target, for me I know how to plait hair. I can go look for a friend or a network and then I top up.

**I: Something else we can do, number one?**

PW01: For me if I don’t get to meet my target this week, I will try and set a high target next week to cover for the week before.

**I: Okay number five.**

PW05: I can go and borrow money or take advance from my boss and add it there then when I get something I refund them.

**I: Yes, number three?**

PW03: May be for example this week I don’t meet my target, maybe I have, am a drunkard I will go to the club and tell the waiter since they know how they help us play some tricks and exchange the drink that a client has bought for me with money. She will get me water and I will be drinking it because I exchanged the alcohol drink with money.

**I: Okay, let’s say we have joined Jitegemee, where would you prefer to keep your money and you trust that it is safe, number nine, where you trust so much to save.**

PW09: Bank

**I: Yes, number ten?**

PW010: Bank

**I: Bank, number three.**

PW03: Now we are members of…

**I: Members of jitegemee you have joined Jitegemee and you want to save, where do you trust to save, a place where you will be comfortable and not using it anyhow?**

PW03: For that part I don’t understand, will Jitegemee bring for us their own account or we will have…

**I: Jitegemee is not like a merry-go-round, everyone will be saving their own money where you trust it and you will not misuse it.**

PW03: Bank.

**I: Bank, number six?**

PW06: Bank

**I: Bank, number two?**

PW02: Saving account.

**I: Saving account, that is in the bank or on your mobile.**

PW02: Mobile.

**I: Mpesa or KBC**

PW02: KCB.

**I: KCB Mpesa, okay number five?**

PW05: Bank.

**I: Number eight?**

PW08: Bank

**I: Number one?**

PW01: Bank.

**I: Bank seven?**

PW07: Bank.

**I: Number nine?**

PW09: Lock savings account.

**I: Lock savings account, okay, am really thankful that was the last question, i wanted to hear everyone’s voice as we finish up, in case of any question you can ask or anything that somebody wants to say? We are done, number eight I was asking if any of you has something to say. Yes, number three.**

PW03: I have a question, now that you have taken information from us and you are going with that information, for us whom you have left us here we are willing to join Jikingee, no sorry Jitegemee so that it can help us, kindly don’t just come and take information from us without helping us. At least we are willing so that you can help us on how we can save and tell us how we can save for us to change our lives, you guys should do that.

**I: Okay, thank you.**

**END**
