# Supplementary material for: Eat a little and save a little: A qualitative exploration of acceptability of a potential savings intervention to reduce HIV risk among female sex workers in Western Kenya
Source: PLoS One. 2024 Dec 19;19(12):e0310540. doi: 10.1371/journal.pone.0310540 (PMC11658496; doi:10.1371/journal.pone.0310540)
Supplement: S1 File — (ZIP) [file pone.0310540.s001.zip › Jitegemee Transcripts and Dissemination Notes for Journal/FGD X.docx]

**INTERVIEW DATE: 06/MAY/2022**

**FGD ID: FGD X**

**AUDIO RECORDING LENGTH: 2:23:35**

**FACILTATORS NAME: LILIAN AKOTH**

**NOTETAKER: MORAA ARASA**

**TRANSCRIBER: ESTHER OMINA**

**INTERVIEW LANGUAGE: LUO**

**TIME INTERVIEW STARTED: 1450HRS**

**CATEGORY: ABOVE 30 URBAN**

**I: Thank you so much and welcome. As I remind you the way I have told you earlier am [Name of moderator] and my colleague is called [Name of note taker]. I am going to facilitate the second and my colleague will be note taking. So…this is Jitegemee study done within =Ugunja =and the FGD today is FGD number X, the date today is 06/MAY/2022. We have in this discussion group we have 10 participants and our discussion has started at 1450hrs. It’s alright. As begin I have explained about Jitegemee.**

R: Mmmh [In unison]

**I: According to what I have explained in brief what comes in your mind when I talk about Jitegemee?**

R: (silence) (noise in the background)

**I: Number eight.**

PX08: According to me in Jitegemee research as I see it us sex workers let’s not depend on sex work for survival (eat) let’s find a way to save bit by bit and move on with life without depending on sex work.

**I: Mmmh, thank you, another person? Number 2.**

PX02: How are you all?

R: (All) we are fine.

PX02: According to my view, we can start our Self-Help Group that each one of us contributes what she has to save so that we can leave sex work.

**I: Mmmh, another person number…...number four.**

PX04: How are you all?

R: (all) we are fine.

PX04: According to my view number four, we can have a small business. You can take tomatoes worth 200/= and have in your house and people will know that number four, four has tomatoes. So that even if you went to do sex work, when you are back you will find that your child has sold tomatoes worth 30/= that is enough for some vegetables.

R: (All) mmmh

**I: Is there anyone who wants to add something? Number seven has opinion.**

PX07: As number seven according to my thought.

**I: About Jitegemee.**

R: About Jitegemee, we should look up to our future life, we should not focus on today eeh, and at least when you are saving or you have your own business that can help you tomorrow or in the future. When I leave sex work tomorrow, what will I do in future? Will kids stop schooling or lack what to eat because I have stopped doing sex work? So, we should depend on other things.

**I: Thank you someone wants to add something?**

PX04: As number five my view is, as a sex worker when you have gotten a job and earning something small you can save so that if you encounter a problem abruptly you don’t have to go for sex work to get money, you can use what you have saved. You can always use fifty and save the remaining.

**I: Number three.**

PX03: Irrespective of sex work we must have plan B for everything that we do. So basically, this sex work and we are given money, we all have plans, so plan for that money work you things out and you are good to go.

**I: Thank you, is there anyone with opinion?**

R: (All) no.

**I: Or we proceed. Thank you. So, I wanted to ask what does women who do sex buy with their money on a daily basis, number seven.**

PX07: As sex workers what we buy most with our money. As women especially those who are doing that job you must be a neat person, you must have nice cloths, nice body oil and general appearance and those savings.

**I: We want to start with what we buy every day, number one?**

PX01: Number one sex workers as you have said, when I get money, I buy food and my partner I also feed because he doesn’t have.

**I: So, its food that you buy?**

PX01: Its food, when kids are in school, I buy books and pay school fees.

**I: You said food, books and fees. Which one do you buy every day?**

PX01: Food.

**I: How much do you spend on food every day?**

PX01: Every day?

**I: Mmmh**

PX01: I can spend 500 shillings.

**I: Five hundred shillings every day, number two?**

PX02: As sex worker number two when I get money, I first design my house I buy chairs, I can buy something like wall nets. Secondly, I buy clothes for my children, thirdly I can treat myself to be smart that even if am going to my sex work am presentable which can make me get clients.

**I: I have seen you have counted clothes, wall net, chairs, clothes which one do you buy daily? Do you buy wall net every day?**

PX02: It’s obvious when I get money, I must buy my food and that of my children.

**I: That you can buy every day?**

PX02: I buy every day.

**I: How much do you spend on buying food every day?**

PX02: My family is still small; I can spend 300/= every day.

**I: Number nine you wanted to talk.**

PX09: As a sex worker I must be neat I must consider where I stay, third to be presentable to be attractive so that I can get some clients and when I get clients I have to do some savings because sometimes I might not get what I expect I don’t get but if I had save back then I can know what to do. mmmmh.

**I: Mmmh**

PX09: Yes.

**I: So, you have said that you must be presentable.**

PX09: Yes, because if am careless on myself I cannot get clients.

**I: You have said taking care of yourself, what do you buy every day?**

PX09: What I buy every day is food first when I eat well is when I can get energy to do sex work okay?

R: (laughing at the background)

PX09: Second my appearance is what will make me get clients.

**I: Thank you, what is the cost of food per day?**

PX09: Food, for example I have one child it can cost 200/= I can eat.

**I: You have said you are taking care of your appearance.**

PX09: Appearance is every day and I must eat appearance I must bath.

R: (all laughing)

**I: Let’s, check on number six, number six?**

PX06: As sex worker number six in this work of ours when I want to go and do it what I need to buy first.

**I: Mmmh**

PX06: I should buy my protection that am going to use there that should be first the second is food.

**I: I have not understood, you can you tell us what this protection is?**

R: (participants laughing)

PX06: Protection is condom that keep in my wallet where I place my money before get out of the house. Then I buy food, how my children can go to school, body oils, clothes that I wear when am going there I must be neat, when am ugly no one will see me.

R: (laughter)

PX06: When he calls me and I arrive when am dirty he will be shocked.

R: (laughter)

PX06: That who is this who has come.

**I: I have heard you talking about condom, when you buy how much you do spend daily?**

PX06: Every day I can spend 50/=

**I: Every day?**

PX06: Yes.

**I: Thank you and what about clothes not clothes food?**

PX06: The way I have children I can spent 400/= from morning to evening.

**I: Two hundred? I heard you talking about clothes do you buy every day?**

PX02: No, I cannot buy every day, weekly or some days to two days to.

**I: Mmmh**

PX06: Yes, when I go, I exchange clothes.

**I: Before we proceed you said you buy weekly, how much does it cost you to buy clothes?**

PX06: Weekly it cost 1500/=

**I: Weekly, to buy clothe, number two there is something disturbing you?**

PX02: As a sex worker when I get money, I buy pants and bra because when I undress in front of this person, I have gone to he will feel good and even add money or even tell him “Someone son add something you have seen the way the food (meaning the sexual organ) is clean”.

R: (all laughing at the background)

**I: So, these pants you buy every day?**

PX02: You cannot buy every day but for example I can buy today for example when I have work well, I can buy even dozen.

**I: After how long do you buy dozen?**

PX02: After even two weeks so that I should not have something that have turned pant that has turned a color due to dirt or worn out) a clean.

**I: So, after two weeks how much can it cost Bra or pants?**

PX02: Pants like my size to reduce the budget because households’ budget I can buy one at 100/= and when I buy even 10 can really help me, mmmh.

**I: Thank you, weekly what do you see or think sex worker must spend buy weekly?**

PX03: Make up, beauty is all that a woman must have.

**I: So how much do you spend on makeup per week?**

R: 500/=

**I: Another person number ten?**

PX010: Food, food is a must.

**I: Food is weekly or daily?**

R: (All) daily.

**I: If you say food number ten how much does it cost daily?**

PX010: Daily when we are two its 200/=

**I: What about weekly now, what can you not go without buying in a week?**

R: Make up, you apply that when you arrived money just come or get out.

**I: How much does it cost weekly?**

R: Make up?

**I: Yes.**

R: You can spend 500/= or even 1000/= depending on which one you want because you cannot use what the other person is using. You use differently because sometimes you do pedicure you must dress neatly.

**I: Why do you use different?**

PX010: There are things like cutex, sometimes you use gel, stick-on

**I: Number seven you wanted to say something.**

PX07: Just as number ten has mentioned. It is make up that we buy weekly, what is used by number one is not what is used by number two, we use different makeup and also depend with prices.

**I: Approximately how much do you spend?**

PX07: What I can buy per week it can cost 600/= eeh

**I: Mmmh, number eight you wanted to say something?**

PX08: It has been said.

**I: If we leave alone weekly or daily, monthly cannot go without buying anything, if we leave alone food, cloths.**

PX02: Always because am a woman, there are things I must buy every month so every month I must buy pads.

**I: Mmmh, thank you I saw a hand up.**

R: In a month, things that I have to do are like house rent in a month as a home-based sex worker you must pay for that house, school fees for the kids, you have to budget for households’ items that are used.

**I: Mmmh. Rent how much does rent cost in a month?**

R: In a month like where I stay three thousand.

**I: How much do you use for the pads in a month?**

R: In a month pads 80/= or 100/=

**I: You have said school fees, this fee is monthly or?**

R: With private school kids its monthly.

**I: How much is monthly fees?**

R: One kid is 600/=

**I: Number eight you are saying something?**

R: (All laughing at the background)

**I: What you have to buy monthly.**

R: Issues with paying rent, pads, school fees.

**I: Is there anyone with different opinion that every month she must, number three?**

PX03: For us singles like me number three we must change suitcases, is not the same, dressing code is trend and it has to change.

**I: Do you do that monthly or?**

R: You go with trend, if you can’t go with it per week, you will go with the trend as it is.

**I: What about yearly, what you know even if it takes one year you must have it in every year, number six?**

PX06: House decoration.

**I: You say house decorations**

R: You don’t have chairs maybe is when you have settled you have plastic chairs and maybe you want to have butterfly next year like right now single ladies are now using L (meant sofa chair letter L) this time I want to buy a TV, I want to buy a home system (woofer) thing like that.

**I: Number four, I haven’t heard your voice for quite some time.**

PX04: I don’t have.

**I: Number one.**

PX01: As a worker, I must buy a cow yearly.

**I: Yearly?**

PX02: Every year

**I: How much does it cost yearly?**

PX01: It cost 15000/=

**I: Thank you so much, is there anyone who want to add something? Women who do sex work, where do they get money from where do they get money from?**

R: Pardon the question?

**I: Where do sex workers get their money from?**

R: (participants cross talking at the background)

**I: You say your number**

R: (laughter at the background)

**I: Number seven**

PX07: As number seven where we get our money, as we have said earlier, we don’t depend on sex work, you can go there and find out that you are not alone you are many sex workers, if you don’t have a client who specifically calls you and you are sure it’s you he will call if you don’t have other work. Sometimes there are those who work in salon, tailoring and there are those who do small business though our main source of income is sex work-our main job is (All) sex work.

**I: That is number sevens opinion, number four.**

R: (participants laughing)

PX04: As number four, as a sex worker, we might have even four men, there is a type of man who loves you and you love him because of his love for you, there is a type of a man even if he is in Nairobi, he knows that number four is a friend whom I love, he sends you money even if you have not had sex, and then we have a man who will call you somewhere and you will go when you are well groomed to impress him, my friend this person will make you drunk when you go in a room, he will do like four rounds and tell you that he loves you. So, we have men in three types. So, you will deal with a type two man who will send you money even if you have not met. So, you will move with this other just because you love them but the other one is the one helping them to love (referring to number two) so to mean this is not an easy thing, you have to weigh this men eeh. There is someone when he loves you and then the one who normally gives you money when you see him it’s alright because what he is going to give you is something heavy. R: (laughter at the background)

**I: Thank you, so where do we mostly get money from?**

R: (All) sex work.

**I: You have answered, so apart from sex work, are there other jobs where we do get money from, number two?**

PX02: As sex worker I have a kibanda (stall) where I do business. I have employed someone that when am not around, she can find me something that when am back it can help. So, I do sex work and also have kibanda that I depend on.

**I: Thank you, is there any opinion number nine?**

PX09: Am truly doing sex work but I cannot depend on it only, I do tailor. After tailoring I do sex work so tailoring also help me.

**I: So, which one is the main one?**

PX09: Its sex work.

**I: Is there anyone with different opinion? There are things you said you buy why do you buy these things, number one?**

PX01: As a sex worker this is the work I do; I must buy something that will remind me of the kind of work I do sex work.

**I: Number two, you had something to say, number five.**

PX04: We are buying these things because we have to improve appearance to attract customers, things like makeup we must buy, food is like basic need you must buy and use to gain energy to work.

**I: Is there any opinion different from those two ladies okay. Do women who do sex work do save? Do they save? Number nine.**

PX09: Yes, they save. The reason why you must save sometimes you get a client and like you expect you agree with him that he will give you 500/= but after you have finished the job maybe you don’t. When you get home must save because I didn’t get what I expected, eeh.

**I: Number seven?**

PX07: As number seven, the reason why we have to save is because you can go and maybe you are not the only sex worker the way I had said. You did not get a client because maybe yours was not today. You will not lack what to eat because you did not work. So, you must go to saving and take to get what to eat for that day. You will try in the morning but we cannot sleep hungry because we did not work.

**I: If I may ask something?**

PX07: Yes

**I: You have said you normally save?**

PX07: Yes

**I: How often do you save, is it daily or yearly?**

PX07: With this job of sex work, you cannot say, you can save daily or once. It depends with how you worked ehh. I can go looking presentable and someone gives me money me 5000/= or someone gives me 1000/= you see I will not use a whole 1000/= it will force me to save even if it is 600/= and use 400/= so that if I don’t get tomorrow from the 600/= I saved the previous day I can take 100/= to help me and save 500/=.

**I: Back to you number six, you said that you normally save, how often do you save?**

PX04: Saving for example I went to work and got 500/= I will not use it all because you never know about tomorrow. Every day is a new day so I will use 250/= and save the remaining 250/=

**I: Do you usually save 250/=**

PX09: Not daily, it depends.

**I: Okay number two.**

PX02: As sex worker number two, I normally do try to set my monthly target for saving, that monthly target I always want it to be that 1500/= for it to help me in my kibanda that I own.

**I: Mmmh, I saw a hand there, number five.**

PX04: The reason why we must save, for example if you have an emergency, sometimes a child has involved in an accident she/he is injured, maybe you were called from home. Some of us have parents they can call you anytime that you send them something. So, you must have something that you have saved. As for me weekends from Sunday at least it’s when the bars are full people are around. So, you set that on weekend when there are many visitors you can save 5000/= in a weekend but from Monday to Thursday the flow is low, there is no work.

R: (participants laughing)

**I: Is there anyone who wants to add on opinion, how do we save number three?**

PX03: It has been said.

**I: You have said you normally save?**

R: (All)

**I: What kind of character do someone who save possess that you can say this sex workers are saving?**

PX02: A sex worker who save.

**I: Number two.**

PX02: As sex worker number two, if you want to know this sex work is saving, she is smart and when you go to her house you find that she is not disorganized she is organized. For example, as sex worker number two I have a stall if you check my background, you will find that I save and use to stall my business I in goods and sell.

R: (participants laughing)

**I: Number seven I saw your hands up.**

PX07: As sex worker number seven what can show you that someone is saving is the way she dresses, even if her fellow encounters problem she comes in to help sometimes number five have experienced a problem, the way she will talk to her over the phone it will tell you that this number seven is saving. So that one shows and always the way she dresses and also if you are self-disciplined. The way it is said the discipline stay alone.

**I: The discipline stays?**

R: (All) Alone.

PX07: This tells that even when you are somewhere someone knows that number seven have something, number five, six always have money. The way you look you carry yourself is self-discipline. And the happiness within you.

R: (participants laughing)

**I: The person having money is humble?**

R: Yes. (Participants laughing)

R: She relaxes with cool things, she walks heavily, yes, she is clean, her clothes are neat, she is responsible.

**I: That’s someone who save?**

R: (all) Yes (participants laughing)

**I: Another opinion number five.**

PX04: As number five, if you want to know someone who save for example when you visit her house, where she lives, when you see her children will tell you that this is an organized person, she has arranged her house well. They have their self-help group where they save money. Is someone that work but she also saves.

**I: Number two tell me yours.**

PX02: As number two if I want to know if number three and seven has money, I can call them like come along am at Ugunja inn, come let us celebrate and feel sorry for our body. You can go and serve yourself some meat because she knows this person has money.

**I: What kind of character do women who do sex work have that shows that they don’t save? (Participants laughing) which type if characteristics do sex workers who don’t save possess.**

R: She is far from home.

**I: What do you mean by that?**

R: She doesn’t unit with other people. (Participants laughing)

**I: Mmmh lets give number three a chance.**

PX03: As number three, those who don’t save.

**I: Mmmh.**

PX03: They normally have many problems that don’t end, and then number two they are always tired, they are malnutrition, and they don’t know how to manage themselves. Someone is 25 of age and appears to be like 45 years. I just think mismanagement of oneself.

**I: Mmmh, number six.**

PX06: As a sex worker, a sex worker who don’t save, is a person who is just worried wherever she is walking around. She likes asking people for help. If someone… if people see her coming they hide from her, she becomes a person that [inaudible segments]. She looks tired, sometimes she is called and she can even miss to go to work. Because sometimes she is called to go to a place like =Madeya= but she has not fares to go there she will be walking round asking people to help her with a certain amount of money, so just such things.

**I: Thank you so much**

P: Another one you can also see her dressing style, because someone who saves must be smart. But if you are not saving, you will realize that someone is untidy. I mean, the way she is dressed, will make you think, “Does number three even save some money even to dress herself?”

**I: Thank you, there is one that is really worrying you, tell me?**

PX05: I feel that sex workers who don’t save, most of them live together 2 or 3 people. For them to be able to pay the house rent they have to pool the money together, she doesn’t live alone and plan her own live. She is always begging. She bothers people every short while if she has a problem she has to go to a fellow sex worker to help her.

**I: Thank you, PX08 you want to add.**

PX08: Yes, if you want to know the characters of sex workers who don’t save, she is always drunk 24/7. Whatever she earned by the time she comes back she is already drunk. She cannot save, she only drinks alcohol and can’t even dress herself.

**I: We had already said, why we are saving, are there some we can add on what makes those who save to save.**

PX07: As a sex worker, the reason why we must increase our savings is because as we had said earlier on we don’t know about tomorrow. Sometimes someone from your heart, you can do something but then give up. If you give up doing the job you are doing. What will you do afterwards so that you don’t engage in this job? How you will take care of yourself really depends on how you have saved money and when it reached a time when you are fed up with the job you leave knowing that the money you have saved you can use to start up a business. Or the money I have saved I am going to pay my college tuition and I can learn to do something using it. That is the reason why we are saving.

**I: Is there any other opinion about why we save, I know we had talked about it PX08.**

PX08: The reason why we have to save, the rate of inflation in Kenya is so high. Let us not lie to ourselves, if we don’t save, we don’t know how tomorrows life will be. You can just be surprised they announce that they have banned sex work. You will die your death [to mean you will struggle]. The economy is so bad.

**I: PX05**

PX05: Another reason why we have to save, I don’t know if it had already been said?

**I: Just say it.**

PX05: We have to save so that if we go for sex work, we can luck but we are able to use the money we have saved in another way that can help us.

**I: Thank you, what makes saving easy for the sex workers who save. What make it easy for them, P1? She is still thinking, Number 06**

PX06: The reason why saving can be easy for sex workers is because, it’s a job that if you are lucky you can earn throughout the week. It is not something you will wait to be paid at the end of the month.

PX08: It can be easy to save if you went to do sex work and were able to earn some money. You come back from work and budget they you say, “Ah… today I want to save this 200 and I can use the 100” that is when saving can be easy.

**I: PX04 what makes saving easy to those who save.**

PX04: It depends on… you know you can go to work and you don’t talk nicely with a client in a way he can understand. You know he will not pay you good money to go home with save a bit and buy maybe some vegetables. You have to talk nicely so that you earn well and when you go back home you can even save 100 and the other 100 you budget with. So we can have a challenge.

**I: Is there anyone who wants to add?**

PX03: Saving has become easy because of what I want for tomorrow. Today I am doing this job, but what about the next life, maybe I will be blessed with a baby. What about the child and the need for her at the end of the day we don’t know what tomorrow holds. Today we have tomorrow we don’t have. You have this child of yours she wants to go to school and has her personal needs. It will reach a point you will get tired, the body has no spare part you will just get tired. So, when you get tired, at least you will be having a starting point in life.

**I: PX02 you wanted to add something.**

PX02: Saving becomes easy for me as a sex worker because I personally I know sex work pays well. It pays well depending on how smart you are to earn from it. And the type…. I don’t know [laughs]

**I: Just say what you want to say, don’t be afraid.**

PX02: The type of…. The way you attract your clients. I feel that sex work pays well. That if you are doing it and you decide to save, you can easily save.

**I: So, you save because sex work pays?**

PX02: My opinion.

**I: PX05.**

PX05: Saving can be easy because you do sex work daily. It is done daily that even if you miss today, the next day you will get something. In a week you must find a way that you get good money that you can save. In sex work you must find clients it’s an area people spend in.

**I: PX07 Do you want to add something?**

PX07: What I want to add, we as sex workers, you do not have to go to work daily. You can have some days that you are hopeful about that you are sure you can’t miss to get something. But for you to save, you must be sharp. You must have some skills, you may earn 200 and when you budget you find that you have sugar and also every other thing and for me to have all these things I may need 100. This means you can save 100. Even if you will not be going daily, even if you will be going only for only 3 days. You can save something if you are skillful if you are sharp.

**I: What challenges do sex workers who save have?**

PX02: The challenge that we may have is, I may go to work and get a client and we have agreed that, “Now that I will be giving you something [have sex with you] you will pay me 1000” we go and have sex after that he may even pay me 200. You see now the calculation I had done, maybe I had decided to pay 100 per day so that I may have 1500 at the end of the month. I can’t save it because my budget of the day may have been more but I was paid small amount of money.

PX10: The way I view it, you may go out hoping that you will get some money for yourself. So, when you reach the other side you realize that you are not getting any business. The way you had hoped to save even 200 shillings. You have that you will not be able to save anything. Because you went to work and was not able to transact any business. You can reach there and you get no business and nobody notices you. You have failed to earn and thus have nothing to save, because the day was bad.

PX07: the reason why saving can be challenging sometimes is because, you know on the work there are different people. There are people who are arrogant, and there are also people who would want to do something bad to you. He has known what you want, he knows what you want you may have gone there and you have already had sex. But, he is just there and has not even given you what you agreed, he totally refuses, and he doesn’t give you. After refusing totally to give you, you will not fight him because he is a man, you will just leave it at that even though he has misused you and failed to pay you. Sometimes that is the main challenge with saving.

**I: Does anyone have any addition?**

PX02: I can add another challenge because in this job there are people who snatch from others, you can get and for sure you have gotten and you have and you find that someone can snatch from you.

**I: What can you get?**

PX02: You can get a man, after getting PX03 sees him and the wan she will come and behave in front of this person… you know after you got him you told yourself, “the way I see him, I will charge him this amount.” If the other person snatches you know you will not earn anything. You will have a challenge because you will earn nothing and won’t be able to save.

**I: Is there anyone who wants to add another challenge. You have talked about the challenges that you face now how can we overcome them? How can we overcome the challenges that are faced by those who save?**

PX06. The way to overcome it…. Like I am a sex worker who is contacted then I go. We can agree on the call before I set off, I just tell him to send me a certain amount of money before I go. That can curb the challenge

P: [Sounds of rain hitting the roof] you must have a target, if you want to do this job

**I: Talk loudly**

P: Having a daily or weekly target, maybe depending on how you have personally decided at least on one side its lighter than the [in audible]… we don’t know about tomorrow .

**I: Thank you, you can hear it’s raining a lot, its making it hard for this recorder to capture our voices. So try and talk a bit louder. I will be pushing it closer to you at times.**

PX02: What I can use to curb the challenges, I would love that sex workers work in unity wherever they are working. If we have unity its better… we can even avoid the issue of snatching men from others. We agree and say, “So and so after getting a client let the client just deal with you and no one should snatch him from you.” So it should be that when he [client] walks to PX03 or PX07 he is hers that there is no issue of adding anything.

**I: PX09 is supporting something there.**

PX09: The way I can curb the challenges is through unity. Because maybe the way I see you is not the same way I see another person. Sometimes people think differently or the way you can talk to someone, someone’s appearance. I mean someone’s mouth…. Talking nicely that can make you get many of these people because, sometimes [in audible segment] but you are snatching him from me that can bring about disagreement.

**I: Is there a way we can address the challenges, the challenges of saving? Is there any other person. It’s like there is none. Are there sex workers who don’t save?**

PX01: With me I save.

**I: But are there those who don’t save.**

PX01: there are sex workers who don’t save. From her job he goes and buy clothes with all the money and remains with nothing. And if she goes to food, she will.. Even if she earned 1000 shillings, she will go and buy 1 kg of meat and eats it all the next morning she will go looking for it again.

**I: What makes them not to save?**

P: The reason why they don’t save, it depend on how one thinks. In her thought she might think that when they do the job the main intention is to eat and dress. She might not know that saving may help her in another way.

**I: On what?**

P: In another way in her life.

**I: Thank you**

PX10: The reason why sometimes you may not save, you may find that you go to work and earn money. You just earn money well then it reaches a point where your needs become so much. May be you have needs here and at home also there are needs. You want to send some money home, feed the baby you want to pay rent. You will just find yourself using the whole amount of money and you remain with nothing to save.

PX07:What I can say, there are sex workers who don’t save, the reason why they don’t save it depends on where they reside, maybe one lives with the parents. I mean she does the job, but has not opened up that she does the job, but she does it knowing very well that she doesn’t pay rent, I don’t have a child to take care of, I am not paying any school fees. So, those are the people who don’t save. That even if somebody has sex with her and gives her 1000 you can find that… in fact even if she goes and finds 5000, she will just party. She will not save because she is seeing friends, she is not paying rent and such things. That can make other sex workers not to save.

**I: Number 6 want to add something.**

PX06: I just want to support PX07 how she talked about…. The reasons why sex workers don’t save depends on their back ground, where she comes from. She could be the fifth child, not educated, maybe she was tough headed and that is why she joined this our job. She doesn’t have needs, she has no responsibilities she is just there depending on the family.

I: Is there anyone who wants to add something? PX03.

PX03: Generally we like spending, we like [inaudible segment] they don’t see saving as a very important thing.

**I: Thank you, No, What are the disadvantages of not saving, disadvantages of not saving.**

PX08: The disadvantage of not saving is that sometimes she may get sick and she has no means out. If she was not saving she can even die in the house.

**I: Number 4**

PX04: As a sex worker, if you don’t save, you don’t even have 30 shillings kept in the house the next day you wake up and you don’t even have sugar, you don’t even have enough to prepare tea. You should find a way of saving sometimes.

PX07: What I can say about those who don’t save,

**I: What are the disadvantages?**

PX07: The disadvantages they may have is when they have a serious problem. When they have a serious problem she walks around, “So and so help me, you help me.” But if we talk about those who go into sex work yet they don’t know what they are doing there. There is this person that goes into sex work with a reason. May be there are responsibilities you have, there are those who depend on you. So, there are those who join but they don’t know what takes them there, and we have said that they are people who live with their parents. They are paid for rent then don’t have children.

**I: Thank you, PX02. If you want to add something on disadvantages for sex workers who do not save?**

PX02: The disadvantages to the sex workers who don’t save, the main reason is that when you get a problem. There is no way you will deal with the problem if you are not saving and you don’t have money. If you are not saving, when you get a problem you may not have a way of helping it out. I feel that is the main disadvantage

**I: Is there any advantage of not saving? PX02 is there an advantage of not saving.**

PX02: I don’t think there is an advantage for not saving.

**I: PX02 Says that there is no advantage for not saving, P1**

PX01: I support what my peer has said, a sex worker must save.

**I: Is there any advantage of not saving? PX06**

PX06: The advantage, as I said earlier on, it depends on where you are coming from. Then you go to live in town where your relatives don’t know. You are doing the job but they don’t know it. Even if you go back home they don’t know which job you are doing. You might go there and even lie that you are working in a certain office but they don’t know anything that can also be its advantage, because they don’t know you are doing the job. Then the second, the advantage… I a lost [laughs].

**I: Failing to save, PX02**

PX02: Another thing I can say is an advantage of not saving, it makes someone not to know who you are

**I: Meaning?**

PX02: That means that if you don’t save, I don’t know how to put it.

**I: Just put it in any language you feel like.**

PX02: It is lost [I have forgotten] [laughs]

**I: Tell us when you remember, PX09 is there any advantage for not saving**

PX09: Sometimes you may think that I have money, but deep inside I know I don’t have. Because eit depends on your appearance, how you walk, your lifestyle.

**I: Okay, is there anyone who want to add? Let’s proceed; you have said that most sex workers save, a I lying.**

PX04: We save.

**I: We said that, where do they save. At which places do sex workers save? P1**

PX01: M-pesa

**I: M-pesa hmm… PX02**

PX02: M-shwari, it can help me because it has loans. Sometimes when I have a problem they can offer me loans and it can help me sort out a problem. Or I can add it to some money I had saved in M-shwari and sort out some problem.

**I: Coming back to number 1, you said M-pesa.**

PX01: Yes

**I: Why do other people like saving in M-pesa?**

PX01: If I want I just withdraw immediately, I will not be running ah… when I want it I withdraw and get the money immediately

**I: You withdraw it immediately you need it**

PX01: And I get it immediately.

**I: Number 10**

PX10: I feel you can save through saving groups, you can join some saving group and save the money you earn there. It will give you easy time because if you save in the group you will not be able to withdraw it anytime. But what you save I M-pesa you can use it to buy credit, you can send it to someone. Someone can just ask you for money you just withdraw and give out. But the one you have saved in a group, you keep it there having a target that if it reaches a certain amount, you can withdraw and do a certain thing with it. It’s better than M-pesa or M-shware.

**I: Number 08**

PX08: If incase we are coming from one area, we can create a group. That if it reaches a certain date people mean and contribute money and you can borrow and it will later help you. When the day of repayment reaches it finds you ready and you just pay back and again borrow.

**I: Number 07.**

PX07: I love saving in a locked savings account, the reason why I like it is because when I say I want to save some money they will ask me for how long I want to save, I will choose even if its 2 months. I will have calculated with the job I do, as in how much I make within 2 months. If I save it in a locked savings account I can’t withdraw it till the end of the period.. So it helps me that even if I say within 2 months I wrote that I can save 5000, I can withdraw the 5000 from my locked savings account and use it to buy what I wanted.

**I: Thank you, do sex workers live a life that is higher than their earnings? Higher than their ability Number 02.**

PX02: With my sex work I live a higher life.

**I: More than your earnings.**

PX02: Greater than my earning because on one side the job that I have employed someone to help me do in the stall gives me earning and the sex work also pays. Don’t you realize that the stall can make me live a life that is higher than sex work?

**I: Another opinion, PX09 Do sex workers live a life that is higher than their earnings, You find that their earning is at this level [low] but the lifestyle they live is here [high].**

PX09: That is why I can’t only depend on sex work. I must have some other job that can boost me so, you may think that I am only doing sex work but, I am also doing some other business, you may think that I live a higher life than my earning.

**I: Another thought, PX08**

PX08: Sex work can lift your life depending on those people you get. Maybe you are a kind of a person who when you say, “Ah, the sex worker number 7 if I go to him he will pay e 1000, If I go to the other one he will pay me 2000.” It makes me rate my life at a certain level.

**I: Number 04 you wanted to say something.**

PX04: I just wanted to support what she has said

**I: What has she said?**

PX04: You can have a rich client, then you just get a call, “number 4 come to a certain place, and take these seats to the house.” He tells you. You just find that you are now living another life.

**I: Number 07, you are supporting something there [participant laughs]**

PX07: I am just supporting what my fellow sex worker has said. It’s true we can live a different life. You can find that if you met a client last time, you please him and he loves you. So when he is coming back he can even bring a very nice phone. Even that who knew that number 7 uses kabambe phone, “yesterday I saw her carrying, hey!!!” You are higher than what people think. So mostly sex workers live a life that is higher than their earning due to the gifts people bring you and also how you please someone.

PX08: How you serve him.

**I: Number 8 you said that ser…**

PX08: How you serve him.

**I: How you serve him?**

P: [All] Yes.

**I: If you say how you serve him, explain this to me [participants laugh] if you serve him in what way**

PX08: When you know… with us sex workers we….

**I: Say that thing**

PX08: You know, you just know that this person with be not cold not warm, I will also just do for him the lukewarm type the way he wants it. But that who will pay well you will have to open it all for him [give the best sex] [participants laughing]

**I: That is her opinion, thank you. PX02**

PX02: For me to live a life that is high I must be sharp. I must be sharp because… number 7 and number 4 said that someone can call you and bring you a seat. If you don’t take care of the seat well. Tightening you mind is having a mature mind. If you meet another person you need to get a way of making him give you money, you can sweet talk. Even if you are called somewhere you don’t reach there looking tired, you reach there and hug and kiss him and from that you will have been given something [participants laughing].

**I: Is there anyone who wants to add something on top of that, number 10**

PX10: You can do this, if you are someone who brings them to your house, and you have beautified your house He comes to your house you know he has not come to play around in your house. When he comes, he comes to make you earn some money. Because he has not come to sleep in your house free of charge. He has to pay you, because the way you have kept your money he don’t know where you get your money so he has to pay for the house.

**I: We have said that our earning may be low or average but the lifestyle that we live is up here. That means that there is a gap, what do we do to fill this gap so that we can just live well?**

PX05: For us to fill the gap, you must rate the clients. You have to rate them that there is one who takes care of makeups you don’t let one person the whole burden. Everyone has a role, there may be one who pays the rent, someone gives money for clothes, so you top them up, if you add all of them, this person gives this the other one gives that among the clients you rely on. After rating how they give you top up and live the life you wish to live.

**I: Number 02.**

PX02: This gap, I can say the way I save, you know when I am saving I will take my money, I can buy anything anytime I want. I say that today I am eating fish, I can just buy.

**I: I am asking this way, so sex workers, do they borrow money?**

PX02: I must borrow because we say that on earth no one has enough. It doesn’t matter even if you were earning a lot, there must be a time you want to do something that you have to borrow money to make your money to be enough to do what you wanted to do.

**I: May be if I can ask you more, when you borrow money as a sex worker, from where do you borrow the money?**

PX02: I can borrow from saving group, like for me M-Shwari. We can borrow from a bank. It depends on where you feel comfortable borrowing. Because you can’t just come from your house that you are going to borrow but you don’t know where you are going to borrow from.

**I: Thank you, another thought, do sex workers have debts, PX04**

PX04: We normally have debts, I can go to number 8, I have a problem my mother is sick at home, lend me 500 shillings when I travel well and come back, I will refund you, we must have debts.

PX07: Having debts is something that is there as number 2 said there is no one who has enough. You can have debts and you can have temporary debts. I mean you can save in a locked account and you have money, yes but you can’t withdraw it immediately, you must have a set time to withdraw it. I can go to my friend and tell her, help me with a certain thing so that I can deal with my issue and if the work becomes better, I will refund, or may be if the job doesn’t improve and the day my money matures and I can now withdraw it, I will withdraw and refund. Debts are things we will always have.

**I: Thank you I saw some hand lifted I don’t know if it was number 5 or number 6 [participants laughing] do we have debts. Number 4 you want to add**

PX04: You may have debts because sometime you may wake up and you don’t have money, you can go to the shop and borrow something. Or go to the market and borrow something, lend me fish I will repay you, that is still a debt, we must have debts, let’s not lie to each other.

**I: I know we have heard some of the reasons why we take debts, what else makes us take the debts**

P1: A friend calls me and I am hoping that he will give me money but he fails, I will be forced to borrow money.

**I: Someone called you, you hope that…**

PX01 He will pay me but he fails me.

**I: So, you are forced to take a debt.**

PX01: I have to borrow

**I: Number 8.**

PX08: You may have gone out to parade for sex work and may be things are not right [there are no clients], you come back with nothing. It will force you to go back home and get a plan B.

**I: Any other person, these that we take, what do we do with them?**

PX05: You may have an emergency and that week you have less clients and the money you have used is more so the emergency will find you with no money. So, you are forced to take a lone to deal with the problem.

**I: Number 2 you wanted to tell me something**

PX02: I can borrow to cushion me when have a problem. I mean, I take debts to sort out a problem when I am stuck.

**I: Thank you, number 3 any idea why we borrow?**

PX03: To save the day, we just borrow to save the day

**I: It’s okay, after we take the debts, we take them because there is something, we want to do with them is that right?**

P: [All] Yes

**I: What do we do or sex workers do to repay the debts they have taken? PX02**

PX02: What I do to repay the debt,, for example I took a debt today, the following morning I am forced to go very early to my work place so that I can get some money and repay where I borrowed so that the next time I go to borrow, he should not refuse to lend me.

**I: Thank you, another thought, I don’t want to leave any thought outside and that is why we came here. What do sex workers do to repay the debts that they have borrowed? PX06 [silence] okay, she is still thinking, PX04?**

PX04: If I have a friend who is close to me and I love him and I took some debts from number 7, I will tell him that, “I took money from a friend to sort out some problems I had, could you please help me.” If he truly loves me, he will have to send and I get to repay the money my peer lent me.

**I: Any other thing that we do as sex workers to repay the debts? PX07**

PX07: What we can do to repay debts is just what we had done, pleasing the client. There is a way you can please somebody; I have debt that I took from number5. I just know that I have 1000 shillings that I borrowed from number5 and I know that I need another one thousand for my personal needs. We agree with my client that he will pay me even 1500 and I still lack 500. So, I have pleased him, the way I have talked to him the way I have pleased him. He will tell me, “Imagine instead of the money we agreed I am offering you three thousand.” He will have helped me; I have repaid the debts.

**I: Thank you. Number 02 wants to add something.**

PX02: What I want to add is, what I do to repay debts is…. What I do to repay debts is savings. First of all, when I am going to borrow the debts, I will go and tell number 4, “Lend me this amount of money and I will repay on a certain date. When you are going to take a debt, you must ensure that you ask yourself when you are going to repay the debt. So, sometime you can borrow and say that you will repay at the end of the month. So, when I am saving, I will save then I will use it to repay the debt.

**I: Is there any other thought on what we do to repay the debts. Is there any.**

PX08: We try to pay these debts as sex workers to avoid the insults of bad names we can get.

**I: So, what do you do to repay the debts to avoid the insults?**

PX08: Savings

**I: Oh… savings, thank you the next question asks, what does the sex workers, what do they do to increase their earnings. If she was earning some lower amount, she does something to increase the earnings. Number 05**

PX05: If you want to increase your earnings as a sex worker, if you were not using make ups you have to add make ups at least you have to exaggerate. And if you get a client and you go to the room you have to give him some work, you can add with different styles, you open for him the whole server. That can make you get more cash

**I: You open for him the whole server what does that mean mom?**

PX05: You give him the whole of it so that he can eat it the way he feels like, you give him the whole dish.

PX01: You can cover for him this way [demonstrates] he gets the whole of it, that way I will have opened the whole fire for him. He will give out all the money [good amount]. I will do for him this way and he will enter the whole of it.

**I: The is number 1, Number 2**

PX02: What I do to please my clients… what I do to increase my earnings First of all I can seduce a client.

**I: Seduce?**

PX02: Yes, seducing in that I can see a client coming from a distance, I ca tell him, “Customer, welcome,” I can do a display turn this side so that he can see how it looks like, , I make these one this way [demonstrating the action] my boobs so that he can see how they look like. When we go into the room, I can ask him, “Son of my father, which style do you want, or how do you want it for I can give you the way you want it.” So as long ask we have agreed on the amount, he then pays me. He will say what he has in mind then I can receive higher payment just that way.

**I: In that way, okay Number 10**

10: Your earning depends on how you talk with someone. The way you will talk to him and bring his heart close, you know there is a way you can talk to someone and make him to be close to you. The way you talk, can make him add your money even more than you agree with him. How you talk to him, there is an agreement you can have when you are the two of you.

**I: Any other opinion, number 8.**

PX08: To add, the way you talk with boost you, your dressing, if you didn’t know how to dress you have to improve in sex work. Where you live also you have to consider your neighborhood. Because there is a client that you can get and he has a car that if you bring him where there is mud [mud houses] then he will not respect you. He will give you your payment in line with the muddy environment. But if you relocate from there and come to a place like here [permanent building]. He will say that, “this woman is expensive let me pay her more”

**I: Thank you, is there any other thought on how you can improve your earning, among the sex workers, what do they do to increase their earnings, Number 2 you want to add something?**

PX02: I can say that your tidiness I think it has been said, and the sex style you have I think we are all adults and there is no child here.

**I: Say it mom.**

PX02: The sex style, you will give him all [So much cross talk not so clear] for him to give out money as you wanted.

**I: Thank you, Number 5.**

PX05: As I conclude on this, the way you can increase your earning depends on the clients you target. You know these people are different, there are people that if you go with them you can just guess the amount you can get. For example, the bodaboda people, you know there is a target he can be able to pay you. You can target VIP, people who are at least, you can target a place that if you go you can get a client that can pay you well.

**I: Okay, is there anyone who wants to add something? Now, if sex workers don’t get a client to pay them, what do they normally do? You don’t get the paying partners, what do they normally do. Let’s say in a day he doesn’t get a client.**

PX02: If I don’t get a client to pay me, I can call another one for help or secondly, I can borrow money just as it was said.

**I: Thank you, another one is which one**

PX02: As a sex worker if you don’t get someone to pay you just as you hoped and you had prepared and set off for the work. You can go even into a bar; you are dressed well. You had already dressed well because you were set to go to work but you have failed to get one. If you sit there sipping a small bottle of soda at least you can’t fail to get one in a day. The whole day as a lady you can’t fail to get totally.

**I: And if it reaches a point that now you are sure there is none.**

PX02: If there is none totally then I will go to my savings that we talked about. You think back and say that it’s the savings that will save you.

**I: Thank you, another person, Number 3**

PX03: [Silent] [participants laughing] there must always be a backup plan, there must be. Those fathers, we must have at least one of them.

**I: You can’t totally miss, there is no day you can say, “Today, I have totally missed to get a client”**

PX03: God also sees us; He will just bring you that father.

**I: Another thought, number 2 wants to add something, just say it I don’t want to leave any thought out.**

PX02: Another thought on what can make us not to miss to earn is…

**I: I meant what a sex worker can do if they don’t get clients.**

PX02: What we can do if we don’t get clients, I cannot put all my hopes that when I go and be the other side [do sex work] that is when I can earn. I must have a partner that when I lack, he can help me.

**I: Okay, PX06**

PX06: If I go and fail to get, I had talked about a business that I do, my business will help me on such a day.

**I: Thank you, the business can help on such a day?**

P: [All] Yes.

**I: That means that there are times we can go and fail to get those who pay us is that true?**

P: [All] Yes.

**I: How do you notice that today there are no clients, how do you know, PX08**

PX08: It depends, like on a Friday, Fridays you just know thing are good but when it reaches a Monday, even if you go to work, you will just go to hang out but you just know that there is no money, so there peak days are the weekends.

**I: Okay, number 02**

PX02: How I can know that there is no job, I can reach the place where we work because I don’t work alone. As a parking girl, I can go and find that so many people have come, those who have come to do a similar job to mine. Sometimes I reach later and find that they had taken all the clients, so I can miss.

**I: You can miss?**

PX02: Yes.

**I: Because you can late, now you find that no one is coming, is there any other opinion? How much debts do sex workers usually owe? At any time, they have debts, it sums up to how much? PX03**

PX03: Basically 1000, it can’t pass that.

**I: It can’t pass 1000?**

PX03: Yes.

**I: That is for you**

PX07: I support that.

**I: You are supporting the 1000**

PX07: Yes.

**I: Another person, Number 8 is shaking the head can you say something.**

PX08: No

**I: Sex worker have debts worth how much any time they have debts?**

PX05: It’s just around 1000

**I: Number 1.**

PX01: I have children, its 2000.

**I: 2000, number 2**

PX02: I can oppose number 3 number 7 and Number 5, because… I can oppose them but, on my side, I can have debts greater than the 1000 because sometimes I can pass by the market as I walk to this my job and I see they have hanged a nice dress. Maybe the seller is a woman I know I will go and tell her, “Mom give me this dress and I will pay you such amount of money.” Sometime it could be an amount greater than the 1000. I will be having a debt greater than what they have said.

**I: Thank you, is there any other opinion? Number 4**

PX04: the thoughts have disappeared.

**I: The thought has disappeared, there is… number 9, I want to move to another part. We said that we came to talk about Jitegemee. I want to ask you some questions about Jitegemee, but let me first of all read something here. Earlier on I told you more about Jitegemee I told you that it is being done to ensure that Female sex workers have money they have saved , that can enable them to refuse having sex without using condoms or to have rest from sex work when they want. We also said that it’s about Female sex workers keeping part of their money, your own money is what you save so that you can use them when you don’t have clients. Or to help you prepare for life after leaving sex work. This meant that we want to come with Jitegemee program to enable you save your money that can help you in future. I heard other people saying amongst us that you can also retire from sex work, it’s that true?**

P: [all] yes.

**I: You can even rest from it, so we want to come up with a program that suppose you decide you want to rest or you have said that you don’t want to have sex with condom because you are a bit tired or you want to retire. Do you have some money? Even though I heard us saying that you save.**

P: [All] Yes

**I: That is good, we want to come and re-enforce the issue of saving. What we want to say is that Jitegemee is coming with it in a different way. It is different from a Mary go round or saving group. We know that if you join a Mary go round there is a limit that is set that you have to contribute even if it is per day, week, monthly. Is that true?**

P: [All] Yes.

**I: With Jitegemee there is a difference, it is your money because we earn differently. You said different figures?**

P: [All] Yes.

**I: As you earn your money, you will decide alone that, “myself I have this target after a certain period of time, so I want to save after a certain period of time maybe daily, or after 2 days or weekly, and the amount I want to save is this.” We will not set a target for you, you will set your own target. And again when Jitegemee comes. It will not tell you that there is a period of time you must take as you save before you are able to withdraw. You can withdraw it anytime you want. Or if you have a need and want to withdraw a portion of it you can just take. When you are bringing it back you will not be charged any interest because it is your money. We will not retain any of your money claiming that its interest you are charged. Is that okay?**

P: [All] Yes.

**I: You will manage it yourself. That is what Jitegemee is coming with. My first question is this, do you think, depending on what I have explained about Jitegemee. Do you think Female sex workers in Kenya would want to join the Jitegemee program?**

PX02: I feel women can like it, because for me it can help me. It can help me with some of my small issues.

**I: If you say small issues what do you mean?**

PX02: Things that you can spend money on, it can help me.

**I: Thank you. Number 8 you have raised your hand.**

PX08: I can really love joining Jitegemee because sometimes you can go to sex work. It is true that sometimes there is no work but if you go where you save, you will not spend angry.

**I: Thank you**

PX05: I feel that if it comes to Kenya women can love it because there are women who said that there are sex workers who don’t save. That can help them boost… it can ensure that its something all sex workers are doing. They can also decide now to save.

**I: Number 03.**

PX03: I see it’s a unique thing, you said it has no shares, women fear things that require payment of small, small amounts, we refuse [Please listen not sure] so it’s unique, you save and whenever you want it you can withdraw the way you wish.

**I: Number 7**

PX07: The name itself has pleased me, “Jitegemee” So, myself as long as I live I can depend on myself and I continue with Jitegemee. It is mine, it’s me it’s a must that we will love it.

**I: What type of sex workers can desire to join Jitegemee the way we have said about it?**

PX07: Those who may want to join are people who save just as we had said. Those who are thoughtful about the future. They think about tomorrow, after I leave sex work what will I depend on in future? In future I will not be working, I will not go to the bar, or go anywhere, how will I depend on myself

**I: Number six you want to say something?**

PX06: I want to support number7, the way she has said that it is about depending on yourself. So when you are independent you will be thinking about tomorrow. In case you leave sex work you can find a way or providing for yourself later.

**I: Thank you, is there anyone who wants to add something. I want us now to move faster it’s getting late. Is there anyone who want to add something? Which type of sex worker will not want to join Jitegemee?**

PX02: The type of sex workers that will not want to join Jitegemee, I can say they are sex workers who are not disorganized.

**I: Who are not organized or disorganized?**

PX02: Who are not organized, because for me Jitegemee is good, and I would love it if it is brought because it can help me

**I: Thank you, number9 do you want to add something.**

PX09: I can say I love it because even if I withdraw I will not be charged any interest so it’s good.

**I: You can love it.**

PX09: Yes.

**I: Which type of women or sex workers do you think will not like Jitegemee when it comes?**

PX09: You know like for example the way I earn is different from the way this other one earns. There is a way that when I earn [in audible segment]

PX10: There are people who might decide not to join Jitegemee. A person that feels like… she joined the job but for her it is just for pleasure. She just the work for pleasure but not that she have needs she want to meet.

**I: Thank you, is there any opinion I have left out? Let me proceed. We all have friends outside there, they are also sex workers they could be 10 or above. Consider the sex workers that you know let’s say about 10 you go and tell then or they get to hear about Jitegemee. Out of the 10 that you know, how many do you think can agree to join Jitegemee? Out of 10 number 2?**

PX02: I feel 8 of them can agree

**I: That means that 2 cannot.**

PX02: 2 may not accept it depending on how I you weigh and see them

**I: How you weigh and see them, what is it that you weigh and see?**

PX02: I see, I mean, they are people who don’t like saving. They are just there, “We have come to do sex work.” But they don’t have… can I say dreams or a thought about how the future will be like.

**I: Okay, thank you.**

PX05: As I see it from the 10 that I may tell around 7-8 can like it but the remaining 2 may not like it because most of them have gone in it with hopes. They are there to benefit from the money that if they hear about Jitegemee they can accept it. But just a small percentage have gone there for pleasure.

**I: This small percentage what do you think…. Oh you have said they have gone into it for pleasure.**

PX05: Yes.

**I: Okay, another person, of the 10 sex workers you know how many do you think can agree to join Jitegemee as we explained it.**

PX07: As you have explained it I just must have something to say, out of 10 who I can tell 80% can accept maybe 2 can reject.

**I: What do you think can make these 2 to reject it?**

PX07: Just as we had said that it depends with how they are living, the reason why she began doing the job [sex work].

**I: Thank you, is there any different opinion?**

P: [All] No.

**I: Now, my next question is, what can we do so that we increase… we increase however possible the number of women who can join jitegemee? Like we’ve said there is a percentage… those who can join and those who cannot join, isn’t it? So for us to increase let me say that if 7 people could join, for us to increase to that it may even be 9, what do you think we should do? Number 6?**

PX06: As sex worker number 6, what can make people to increase (background talk, laughter), what is… what can make the number of people to increase, happens… when we are in a group and we make our own stuff, some people feel that *“these people what do they do that they have managed to do 1, 2, 3.”* So, they will come and be together with us.

**I: Number 8**

PX08: As sex worker number 8, it depends on the limit that we shall put, yes. That will make someone work to be easy, if we put that we are saving ten, ten, ten then I know that many people would love to join.

**I: That you save ten, ten…**

PX08: I mean so that…

**I: Ten shillings or ten…?**

PX08: I mean ten shillings… I mean a limit that is comfortable that someone can manage.

**I: Thank you.**

PX08: Yes so that she can join the jitegemee group.

**I: That is a good thought. Remember you are the ones who said that there is no limit… jitegemee does not set for you a limit, personally you are the one who chooses. Because today maybe work** **was good so you will decide that let me save 100, tomorrow it’s bad so you decide that today I am saving 20. That way… that way… that way… there is no limit that is set, you are the one who chooses. Number 2?**

PX02: As sex worker number 2, what we can do so as to increase the percentage of people, is that the way you are sensitizing you continue doing it at different places so that we can continue getting people who’d like to join jitegemee.

**I: Any other person? Number 7. (Laughter) you’d not laughed.**

PX07: Personally as number 7, I can concur with my colleague sex worker number 2. This sensitization… you know to convince someone, even if it’s chamaa if you want a fellow woman to join; you must give her… you tell her the benefit of the chamaa. You can start that “the benefit of our chamaa is that once you’ve saved Kes 500 we can loan you Kes 800.” In this way you’ve told her the benefit. Now, the benefit of something is what can make one to join, that is what I can say.

**I: Thank you. Now, what are the things you think jitegemee should do when it becomes available to increase its full acceptability by FSWs apart from what we’ve mentioned? Number 7?**

PX07: Personally should jitegemee be available, it’s being availed because of us women who do not have steady jobs. We have this work we are doing but we don’t like it. There are some people who’ve joined it’s not that they like it, there are people there is a reason why they have joined, isn’t it?

ALL: Mmmh.

PX07: Now when they come they should let us… they even tell us that “if you save and you can be given a loan you can… this and that… anytime that (coughs) that you have a problem they can… I mean they can hear your appeal even if the duration… let’s say that time for repayment of loan has reached, the duration that you were given even if you were given 3 months. Within that 3 months you’ve not been able to raise which means you’ve not repaid; they don’t harass you… you know there are people when they come they take even the broom and the bed. So you when you go to the house you ask yourself “where will I sleep? I will have to look for their money and repay.” So they have to consider how we women stay and it also how with our work we can lack or get.

**I: Thank you. Number 2?**

PX02: Firstly I am requesting that you repeat the question so that I can respond to it. (Chuckles)

**I: Ooh! I repeat the question? What are the things that jitegemee should do or should have in the** **program so that FSW can accept to join?**

PX02: As sex worker number 2, I’d like that once jitegemee has done this… then it starts for us a savings group, and savings there is always something known as table banking where we can put our money and we go back and get loans to do our businesses.

**I: Now, which women or which FSWs who would not like to… sorry… what things following what I** **have mentioned here… what do you feel we might do as jitegemee that FSWs will not like? Number 2?**

PX02: I as sex worker number 2, I don’t see anything wrong that can make someone to not like the jitegemee program. I feel that its good. There is nothing that make someone not to like it.

**I: Another view? Let’s talk fast, I want to now move it quickly. Another view? Okay, alright. Do we all agree that there is no this thing… there is nothing wrong?**

ALL: Mmmh.

**I: The benefit… why haven’t everyone told me the benefits (background talk). Number 7.**

PX07: You are talking about benefit?

**I: No. I’ve asked that what are the things that FSWs would not like? Or you can tell me a benefit if there is.**

PX07: Yes that what I am telling you… I am responding on the benefit.

**I: Alright (chuckles)**

PX07: The benefit… you’ve explained so well that jitegemee has not set a limit on what someone can save (phone ringing). That is the best benefit. If today I get Kes 50 and save, I won’t be laughed at if tomorrow save 10 bob that “normally she saves 100, 100 but today what would have happened, she has only saved 10 bob. Yes, that is its best benefit.

**I: Thank you. Number 5?**

PX05: Now, what the sex workers wouldn’t like, is that when this program is availed… I mean how you address it. Isn’t it for sex… commercial sex workers whom you are going to deal with, aha? We can put it in a way that it’s not exposed. It’s not exposed that these are sex workers who join this group. Yes, so they put it in a way that it’s not exposed that these are sex workers.

**I: Personally, how do you think it can be made available without it being exposed? What you think.**

PX05: They can get it a decent name, so that it’s just know that those are just some ladies but not…

UNKNOWN: Sex workers.

PX05: Those who are doing this kind of work.

I: That does not identify them. Let’s say… let number 6 talk first and then we go to number 13.

PX06: I just want to concur with six… sex… sex worker number 5, when you asked what we can do so that it’s not know that it’s sex workers who are in the jitegemee group. It can be done over the phone so that no one knows what is going on, that this deals with such people. (background noise, rainfall)

**I: The rain wants to mess us up again. Now, number 7 you wanted to say something before I go to something else.**

PX07: Personally, I would say sorry for taking your work. What I am saying is that the name that it’s been given does not indicate any sex (chuckles). It’s been put as jitegemee. We have to look properly at the way it is written, “Jitegemee” no sex is indicated. “Sex worker jitegemee”… what is written there is jitegemee. Jitegemee does not mean… does not show that you are a sex worker. Jitegemee means that you depend on yourself, you should not depend on another person. I should not depend on another person but I should depend on myself, so I don’t see any problem there.

**I: Another view? Number 3? Number 3, I am asking that it there… according to the things that we’ve discussed about jitegemee. Is there any that you feel that may hinder your work? It ca**

PX03: Sema Kiswahili.

**I: Can you… I don’t know. In accordance to to what I have said that jitegemee wants to do, are there some that you think would risk your rights? Talk loudly, you know I can get you but that person over there has not heard you.**

PX03: There is none.

**I: There isn’t? Okay, she is saying that there isn’t. Number 2?**

PX02: As sex worker number 2, I feel that there is nothing that can jeopardize our right or my right in jitegemee group.

**I: Is there any other opinion? Okay and is there… number 1 has responded so fast (laughter). Number 1 the way you’ve responded so fast…**

PX01: Eeh.

I: I would wish that you respond to my question. What challenge if we as jitegemee we come with this program would we face? What challenge would we face?

PX01: Challenge? As we are sitted and you make a phone call to me but I feel that…

**I: When we start jitegemee intervention… we are coming with it later… today we just came to get opinions, isn’t it?**

ALL: Yes.

**I: Now we want to come out with it, we want people to join and they start to save and we learn different things about saving. Now, what challenge… what challenge would we face as the people who want to introduce it?**

**NOTE TAKER: The challenges that we will face when we want to introduce jitegemee to women.**

PX05: Like me, I come from Sidindi, I need fare so it might be difficult for me to reach here.

**I: Talk loudly, I want that thing to record.**

PX05: Like fare. (chuckles)

**I: Fare might be a challenge.**

PX05: fare might be challenge to come here.

**I: Okay. Number 2?**

PX02: Personally as sex worker number 2, the first challenge that you can get is how you can talk to people so that they may agree to join this group. Secondly, how you can get ideas that may improve this group.

**I: Now if I may ask you before I go to number 1. You’ve said that we can have a challenge on how we can talk with people to join jitegemee or how we can get ideas? Now, how can we… which advice would you give us so that we don’t face so much challenge of talking with people? Which advice would you give us?**

PX02: The advice that I can give you as sex worker number 2?

**I: Mmmh.**

PX02: Let me say you should not have things like abusive words…

**I: When you talk of abusive words, how are they my dear?**

PX02: I mean badly worded words, like you can talk to sex worker number 7 and you talk… I mean even if you don’t say it but you can talk to her in way that you feel that she is doing some useless work dirty.

**I: Thank you. Number 1?** **You’ve said that we can have challenges like that of fare. Like now you coming from Sidindi…**

PX01: Sidindi.

**I: Now here how can we… what can we do? What advice would you give us so that this does not become a challenge?**

**NOTE TAKER: I can we solve the challenge that you’ve told us.**

PX01: If there can be something like this or that.

**I: Okay, when it comes to…**

UNKNOWN: Fare…

**I: Fare can be a challenge to someone like you who comes from Sidindi and people are meeting here at…**

UNKNOWN: Ugunja.

**I: Ugunja. Now, how can we solve this issue on fare so that is does not become a challenge?**

PX01: You go to where someone is.

**I: We just go to where someone is, thank you. Number 2?**

PX02: As sex worker number 2, I can assist sex worker number 1 with the way you can sort the fare challenge. You can look for a way that you can get us something that can help. Even if it’s not fare… money that sort out the whole amount of fare but it can be at least half of it or even the amount that take her… I mean that can bring her but you can’t send her back so she can get on her own what can take her back.

**I: Thank you. Number 9.**

PX09: As sex worker number 9, how we can get this fare for example…

**I: Does it mean that you are also saying that fare can be a challenge?**

PX09: It can be a challenge but now how we can reduce it is for example today is 6^th^ and maybe we should be meeting on 10^th^, so it’s better… I know that between 6^th^ to 10^th^ that is something I know that I have to look for far… fare. So it’s good if you know earlier.

**I: So we give information about this meeting early?**

PX09: Early, yeah.

**I: Thank you. Another advice… (laughter) number 7 wants to laugh at me. Number 7 say something (laughter)**

PX07: I feel that…

**I: Another challenge?**

PX07: The challenge that we can get… the challenge that you may get as people who are introducing jitegemee, is that if you want to hold a meeting we have say it early so that we know. According the peoples phone numbers… let me say currently we are members, isn’t it?

**I: Mmmh.**

PX07: We are like members…

**I: For example.**

PX07: For example we are like members, we have to get messages… messages on our phones that can inform us on such a date we should be meeting at such a place. But you as officials let me say that you should try and work us members. S… so and so number 7, you live in Ugunja I want you to bring together about 10 sex worker within Ugunja; and then sex worker number 5 let’s say you stay at Ambira I want you to bring together about 10 people at Ambira. Which means when you come from Ugunja the people from Ambira don’t come to save the fare. Now, once you are done with my people at Ugunja then you go to Ambira. Ambira is not far. I mean you do this so that… you inform us about this early so that when I am going to talk to people I know how I am going to sweet talk them because sweeting a woman is not easy. You go to her and she informs you that she is going to the farm, my maize plants are ready for weeding. I won’t seat down yet I’ve not gone to weed. So I have to sweet talk her so that I know the time that she will come, I have to tell her the benefit of that thing, yes.

**I: Thank you. Is there any other challenge and how we can overcome it? okay, alright. I have another question that, if we start jitegemee, how much money do you think that as an individual… at** **this point I wish that each and every one to respond. You as an individual you’d wish to save per** **week if it doesn’t interfere with your program, your daily needs. How much is it? Number 3?**

PX07: Per week?

PX05: Kes 500.

**I: What? One person each.**

PX01: Per one week?

PX08: Yes, per week.

PX04: 7 days?

PX06: Yes, 7 days.

PX03: Kes 700.

**I: That is number 3, Kes 700 per week. Number 2.**

PX02: Personally as sex worker number 2, (coughing) I feel that I can save Kes 1000.

**I: Per week. Number 1?**

PX01: Kes 1500.

**I: Kes 1500 or Kes 15000?**

PX01: Mmmh.

**I: You’ve not said which is which.**

PX01: One thousand five hundred.

**I: One thousand five hundred per week, alright? (chuckles) number… number 5.**

PX05: As sex worker number 5, I feel that I can save Kes 1000.

**I: Kes 1000. Number 9.**

PX09: As sex worker number 9, I can save Kes 500.

**I: Kes 500 weekly?**

PX09: Yes.

**I: Number 6?**

Note taker: Number 10.

**I: Number 10, sorry.**

PX010: I can save Kes 500.

I: Weekly. Number 6?

PX06: Kes 1000, weekly.

**I: Kes 1000 weekly. Number 7?**

PX07: Mmmh? (chuckles) oooh number 7?

**I: Yes.**

PX07: Now as sex worker number 7 I can save Kes 500 per week.

**I: Kes 500 per week. Number 4?**

PX04: Kes 1000.

**I: Kes 1000 per week. Number…**

PX08: Number 8.

**I: Number 8 you were out shortly but the question that we asked was… if you** **were to join jitegemee and you wish to save your money in jitegemee. Personally what amount of** **money would you like to save without interfering with your daily needs?**

PX08: Daily?

**I: No, per week.**

Unknown: Per week.

PX08: Per week, Kes 500.

I: Kes 500 per week. Thank you so much. Now, how can this money be saved where… your money that you’re saving, where can it be saved where sex workers trust that they know that there is no risk… any risk that may be there. Number 7.

PX07: May be bank and officials are chosen. 2 or 3 people who can always go there and they should be people who are known so that in case of anything I can go there tell her that “*this money is at the bank and you are the one who knows the pin that be used to get this money from there. If not you then so and so. Take me to so and so and then so and so takes me to the third person so that they can tell me why this money is not there.”* I mean as a group… I am talking as a group, yeah. (background talks)

**I: Thank you. Number 6.**

PX06: If we don’t want to drag each other as a group, we save money… we know that we meet on this day or that day, even if it’s twice in a month we collect our money on 15^th^ and put it under lock account for 30^th^. So that when we meet on 30^th^ we get it safe. And again on 30^th^ we unlock it, collect some more then take all to lock account until the next meeting day.

**I: Okay, thank you. Number 2?**

PX02: Personally as sex worker number 2 I’ve rejected what sex worker number 7 has said, because the issue of bank you cannot trust a person with money. We will say that we will select 3 people that we know. We will select the treasurer and I don’t know the 2 signatories. And they will… there is something that you can do at the bank, you’ll take the money and deposit then they can withdraw but bring us only the deposit slip of the money deposited. This one I have rejected. Now it’s better… (laughter) no wait first.

PX05: I’ve just lifted my hand.

PX01: She has just lifted her hand.

**I: Just say what you need to say.**

PX02: Let me just finish saying what I have then you can talk. Now, we could have… I would prefer the issue of table banking whereby we contribute our money then it’s written down how much it is, then the person requesting for a loan gets it and we know that the day we are coming back for a meeting on such and such a date it will be paid back. Thus we see that our money is there and so and so has it and she is going to repay it.

**I: Thank you. Number 7 you wanted to say something. (Laughter).**

PX07: I as number 7, the reason why I was saying so, I was saying… you know currently let me say that we have started something… let us say the way we are…

ALL: Mmmh.

PX07: You’ve contributed 20, and you’ve contributed 50. Do a calculation that someone has contributed twenty, twenty, twenty each one of us, isn’t it?

ALL: Mmmh.

PX01: It’s two hundred.

PX07: It an amount that is not even Kes 1000. How can you put it on the table and divide it equally amongst those who are here, because everyone wants to borrow. Do you get me? Everyone wants to borrow, you have contributed… we have contributed twenty, twenty and placed it here now you want to divide it amongst the 15 people that are here or 13 people who are here. Do you think that money… I am going to get any money that can help me? I am not going to get money that is going to help me.

ALL: No.

PX07: The reason why I am saying this is because (giggles), you see the times when we are saving money… we are saving it, we know that we’ve said for one month or two months, this money has accumulated to Kes 5000. This person social… if sex worker number 5 wants Kes 2000 she can get that loan. Sex worker number 1 or number 2 if they want to borrow 500 or even 1500 they can get. But we cannot do something and we put on the table yet we are contributing something that cannot be of help to us.

**I: (Cross talk) Let number 8 say something.**

PX08: In my thinking I also reject the bank because you’ve said that our program is called jitegemee. It is money that can of help anytime that you are stuck, isn’t it?

ALL: Yes.

PX08: Why if… if… if we meet just the way we are then we choose one person and may be buy one line where we save our money thus any time you have a problem… because you’ve said that here it’s jitegemee in that you don’t want your money to be deducted, isn’t it?

ALL: Yes.

PX08: And at the bank it’s going to be deducted.

ALL: Deducted.

PX08: And it’s a higher rate when the money is a lot. Can’t we look for one person here who can be keeping the money, and we buy a line where money is saved so that when you have a problem then you are withdrawn for just the amount that you needed.

**I: Now, those are different views. Everyone has their own view, isn’t it?**

ALL: Yes.

**I: Alright. Any other?**

PX07: Let me tell you first talk (laughter)

**I: And then now we leave that topic. Number 2?**

PX02: Now, the reason why I talked of table banking… first I’ve heard each one asking what extra amount we can get. The extra amount that we can get… we’ve gotten roughly 30 people and we get that 50% of this people can manage Kes 1000; Kes 1000 per week. And then we say that we want to this thing of table banking. Do you think we can lack money that someone can borrow and it helps out? Anyone who can respond to this question do respond, I am ready to defend (laughter) chap chap.

**I: Those we will discuss… there will reach a time that we shall talk about those isn’t it?**

ALL: Yes.

**I: We are just giving our opinions, alright?**

ALL: Yes.

**I: Any other opinion may be someone wants to add. The place where you think the money that** **we’ve contributed can be kept that the sex worker feel that is safe and there is no risk that can harm it. is there anyone who can add another opinion there? Now I am asking another one that** **those people who engage in sexual intercourse like the sex workers have not raised enough money to save… the amount that you had planned to save may be per week as we had discussed.** **What do you think sex workers can do for them to get the money that they want to save?**

PX03: Kindly repeat for me the question.

**I: That… that if a sex worker has not managed to raise the amount of money she planned to save for example per week, the week has ended and she had planned to be saving even Kes 1000 but the week has ended and she finds that she has not managed to raise that money. What do you think that they might do so that they may get that money and save as they had planned? Number 2?**

PX02: Before we had said that we as sex workers we always save. Apart from this group that we want to start we always save. We can go and borrow something from our account or if there is a business that we run as sex workers, we do business different from sex; sex working. We can go there and it can help us or another way we can borrow.

**I: Thank you. Any other thing that you think sex workers may do to make her get the money that she had planned to save in case the time is up when she has planned to save yet she has not raised the money. What can she do to get that money to save as she had planned? Number 10? What do you think (baby whining)? I know the baby is also tired. Number 3? Nothing? (Chuckles). Now, do you think that the things that we are doing as jitegemee is going to bring back hope for women… for female sex workers? Can it bring back their hope in that if the join jitegemee they will never be at risk of getting HIV?**

PX07: Please repeat.

**I: That following how we want to come us jitegemee, we have different things we have saving and sensitization on lowering the risk of getting HIV and other things. Do you think that the sensitization that we will come with as jitegemee will bring hope to sex workers thus if the see jitegemee they feel that they are not at risk of getting HIV? Number 2? (laughter)**

PX02: As sex worker number 2, I think there is because once we have been sensitized…

**I: Just proceed.**

PX02: Once we’ve been sensitized, we will meet with different people and everyone will give their opinion that can help us to protect ourselves from getting HIV.

**I: Another opinion. Number 5?**

PX05: It’s going to bring us hope and we will see that there is an NGO that values sex workers because mostly… because mostly majority of the sex workers are stigmatized. So we are going to feel… its going to bring us hope and then following the sensitization that we are going to get just as she has said, can give us much information and open our ears deeply in getting HIV.

**I: Any other thought? I want to move to the last topic so that I can now end. Number 3 do you think that since jitegemee will come with information, the things we want to come with. Is this something that will bring back… will bring hope to the female sex workers that now they cannot… they will not get HIV? They are not going to be at risk of getting HIV.**

PX03: Yes, at least they will be safe (cross talk) but at least with their savings they will be broad minded to start small businesses to do.

**I: Any other? (laughter) number 7, I have stopped. Okay I am moving the next topic. I want us to now move fast so that we are not so late. Now, do the female… the sex workers think… what do you think they can… sorry. Do the they think that now they can stop sex work? Does this thought come to their minds?**

PX04: Of course.

**I: Number 4? Number 4 tell me first. (Laughter)**

PX04: As number 4, do you know talking to a man all the time it’s not some easy work to talk to him for him to give you something. Let’s not pretend that we have to have sex all the time. There are times that you just meet and talk and go separate ways. It reaches a time that the age has progressed, when the age progresses, your brain cell reduces and you are tired… you are even tired of bathing, and you even won’t be able to purchase those body oils. Yes, your body does not even change with the body oil applied. So we have to broaden our mindset we start jitegemee…

**I: What does your body not get affected with?**

PX04: Body oil. (Laughter) the body rejects, I am not lying to you. Even if you apply oil it doesn’t work, even if you try being smart someone will say that *this person is really trying but her body has now refused.* So you are removed from the group.

**I: So you think… you think that they can stop sex work?**

PX02: We can stop at any time and even right now it’s only this year and the next year we are not here.

**I: That’s her opinion. Someone else? Number… number 2.**

PX02: As sex worker number 2, I feel that there is hope that I can leave this sex work because there comes a time when as a woman I won’t want a man, yes. So when such a time comes I can stop but even if it’s not that time maybe the program that you’ve started can change my life and I stop sex work… yes I may be doing something else but I am still in the group and I am no longer doing sex work.

**I: Thank you. Number 5.**

PX05: Yes, we can have the thoughts that there might reach a time that we will stop that work because of the challenges that are there. Maybe you have children and they are growing up, so there might come a time that the child won’t really accept the work you are doing. Or there might reach a time that s/he is taking care of you that it’s not a must that you have to for sex work so as to fend for yourself. Yes, there might reach a time that you now start thinking. Sometimes you’ve gone and met a client that has seriously long thing that hurts you. So you know even if you leave the next day there will be something in your mind that there will come a time that I will stop this work.

**I: Thank you. (Cross talk) number ten make me laugh too. (Laughter) Number 10, is there a time that sex workers think that they will also leave this work? They will leave sex work.**

PX010: Yes. This thing tires the body thus you just have to stop it and once you have stopped it in case you have something in your mind, or if there is some money that you’d made and kept aside. It’s that money that you can take and start some business which you will do. Secondly, may be you have educated you child and s/he has grown up and gotten employment somewhere, that child of yours can also help you out, yes. You might get that you are doing this work and you may have a daughter who knows about these things thus she will feel that *“how come my mum embarrassing herself.”* So in order to respect your children you may stop this work, yes.

**I: Thank you. Number 6 do you want to add something?**

PX06: No.

**I: Number 3?**

PX03: No.

**I: Okay, now I’ve heard that there is an opinion that sex workers can stop this work of sex working, isn’t it?**

ALL: Mmmh.

**I: Now, when we sit together at one place do we always discuss that we can stop sex work?**

ALL: Yes.

**I: Number… number 4?**

PX04: We talk about it.

**I: Eeh?**

PX04: We talk about it. it tires the body it reaches a time that you don’t even want this thing. We talk about it that *“my fellow woman this time round we look for tomatoes so that we can stop.”*

**I: What brings about these discussions?**

PX04: The thing that brings these discussions is think… it’s the head, thoughts. You feel that you are tired of that thing so you start thinking that if I do this wouldn’t it be good. Yes, so the thoughts.

**I: Thank you. Number 2.**

PX02: As sex worker number 2, what can trigger this into our minds, is that sometimes you’ve gone to work and you meet a certain man… you know at work the people whom we meet each of them there is a way they treat you they don’t do it the same way. One might deal with you ruthlessly while the other… I mean… when you go you start talking with sex worker number 7 that *“such and such a thing placed me in such and such a situation”* so now that thought crops up that we can stop.

**I: Thank you. Number 5 you had some addition? Do you…**

PX05: Yes.

**I: Talk about it?**

PX05: These talks we can… we talk about them severally. Sometimes you’ve seen what a colleague of yours has passed thorough may be on the television, maybe over news what she is going thorough. So with this you know you can share with your friend. At times you are someone who has at least saved… you know those people in the big cities there are some who save and even manage building plots of houses thus there reaches a time that now her mind stops thinking about this thing because at least she has saved. So you can try to be together, you try advise your friend telling of what you’ve heard somewhere else.

**I: Number… number 3, what makes… (giggles) what can make sex workers to stop or can make the to think of stopping sex work?**

PX03: Number 1, experience. Today you went… by the way some men are very brutal so per your services they just make you to be mad. You will hurl abuses at him and he will also hurl some to you as you leave. So your experiences with those people because today you will stay in town from January to December as you explore.

**I: Experiences. Any other reason which can make someone to think of stopping sex work? Number 1.**

PX01: Maybe you’ve gone with him inside the room, and when he undresses his penis is so big that you say to yourself that *“all this is going to into my body, all this?”* (laughter) you know you’ve always seen the normal ones but this time it’s a big one, it has not reached you yet but its standing thus you feel that *“if it’s this way then let me go and rest”* (laughter).

PX08: What can also make us want to leave is that maybe you are a sex worker and sometimes you went and met a relative may be a cousin and s/he does not know what you always do. You know that… you’ve seen him/her before s/he sees you, you know that is what you are going to think first that *“it’s better I stop this work”* such things.

**I: Thank you. Number 7.**

PX07: Personally as sex worker number 7, what can make someone to long to stop are the challenges that she is going thorough. At times you were doing it before you get someone who will marry you. So maybe you’ve gotten a man who tells you that he wants to and build for you a house at home. So you won’t leave your home and your husband that you are going to do such work. It will force you to stop and when you stop it’s good if you’ve also made some decisions that *“this work I have done for 8 or 10 years and it has helped me sort out this, and I’ve managed to buy this because of this work.”* So getting married can make you stop this work. The children that you may have may also make you to stop this work you are tired. The third one is age, now you have reached 50 years you are wrinkled so much. Makeup is just dripping thus you can’t do this work.

**I: Thank you. Another thought, what can make someone to stop this thing… think of stopping sex work? Number 4? (Laughter) Okay let me move on. Women who exchange sex for money I mean the sex workers… at what age do they stop this work?**

PX02: Mmmh?

**I: The sex workers, at what age do they stop sex work?**

PX02: Eeh!

**I: Number 9?**

PX09: I don’t think there is a limit because at times you might be young and but old. You might be having a tiny body structure but you are old age wise.

ALL: Someone aged.

PX09: So there is no limit there.

**I: Number 6, say something.**

PX06: As sex worker number 6, I say there is age limit because like my grandmother back at home is someone who is using a cane thus cannot do such work. (Cough, background talks) it’s the age of about 70.

**I: They can leave it at 70?**

PX06: Yes, you can leave it at 70.

**I: Another thought, that’s her opinion. Number 2.**

PX02: As sex worker number 2, I feel that there is no age limit. This thing depends with your decision, yes. I can decide that today I don’t want this thing and I stop.

**I: Another opinion? Is there an age that now if it reaches this age then someone stops sex work?** **Number 2?**

PX02: It depends with…

**I: Number 4.**

PX04: It depends with how God created you, because… and then it depends with how you live and the type of food that you eat, do you get this. Yes, it might be that truly you live poorly that you eat kales on a daily basis, your body hormones my friend have to go down but if you are feeling good and laughing all the time; and making your body happy. You just see that life is good. It depends with how you live.

**I: Thank you. And what do sex workers always do… let me say that they have said that they have stopped sex work. What do they always do when they stop sex work? Number 8.**

PX08: If she had invested then she can just proceed and continue with that.

**I: Another opinion. What do they always do? Number 10. No idea. Number 2.**

PX010: I can also concur with sex worker number 8 that it’s just business. Once you’ve stopped and maybe you are now an older woman you might feel that let me get some vegetables and sell by the roadside so that I earn a living.

**I: Thank you. Number 1?**

PX01: I don’t have.

**I: Now, I am asking that these sex workers when they stop sex work, where do they go? Where do they go when they stop sex work? Number 3.**

PX03: Some get married while some decide to be single. Some are just there with us.

**I: When you say they are just there what do you mean (laughter)?**

PX03: They are there… they are just there.

**I: There is nowhere they will go?**

PX03: Now where will they go, will they go back to the village? Unless they go back home.

**I: Number 2. What?**

PX03: If they were living in the city then they go back to the village.

**I: They go back to the village. Another opinion, number… what?**

PX05: Some have died.

**I: That is number 8, some die. Apart from dying where do some go when they stop sex work?**

PX08: They make themselves busy.

**I: That is number 10, what does making themselves busy mean?**

PX010: As in just make yourself busy in your own way, maybe you are at the farm this and that, I mean your own businesses.

**I: Okay.**

PX010: To do.

**I: Okay. And now for those who are stopping sex work, what difference do they have in their lives when they stop sex work? Number 2.**

PX02: The difference that I am going to have in my life as sex worker number 2…

**I: How they live.**

PX02: How they live or their appearance?

**I: It can be the appearance, how they live provided that it’s after they leave sex work.**

PX02: I feel that as sex workers when we stop this work and time has elapsed, I mean you see that their body is so withered, yes.

**I: Number…**

PX07: Or the mind-set that she has when you are relaxing with her, you find that her opinions are much more clear compared to someone who has not done sex work, yes.

**I: When you say…**

PX07: Because she can tell you something and you think that she is joking yet she is telling you something that she knows.

PX05: Experience.

PX04: What she personally experienced, yes.

**I: Thank you.**

**NOTE TAKER: What do you mean by clear thoughts?**

PX04: Clear thoughts… it’s matured thinking… (background talks)

PX07: She has the experience with the work she was doing.

PX09: She is sure with what she faced.

**I: Number 9 has said that she has experience with the work she was doing. Number 2.**

PX02: Clear thoughts is let’s say there is a sex worker who has just started doing this work, she can go and ask some advice from her so as to get a way on how she can go and get something from a client.

PX04: Yes, it’s true. You can get a young person who is 15 years old…

**I: That is number 4?**

PX04: Yes, number 4. She has gotten a client but this person is playing her because he feels that she is young and she cannot tell him anything and he listens… even if he is loaded with money she cannot talk with him and he gives her something that can help this young girl, so he is playing this young girl. And this girl will learn that a certain person is this type of person thus she will come and ask me that *“number 4 recently I went to such a place to relax and someone bought me a bottle of soda and told me that we indulge in sex, and this person it’s always said that he has lots of money”* why didn’t he give this young girl? There is nothing this young girl tells him. So you find that this young girl is… so when she comes to number 4 who is experienced what sex work is, I will tell her that *“young girl stop this thing that you are going to, look for some business you do what… you do, you are wasting your life”.*

**I: Do we know anytime that sex workers have stopped sex work but came back to it later?**

PX04: As number 4 returning is like you’ve gone and tried your hand at business, you’ve tried it severally and you are unable because you were already used to that money. You go talk, maybe have a round of sex and get may be Kes 2000 and then you come back but you know with business its something that you must be very committed you don’t joke with your money; you are going to sell, how much will you get and at the same time you are also consuming it, isn’t it. so you feel that *“I am tired this thing is headache let me go back”* that is why we go back.

**I: Thank you…**

PX04: And there at times when… when you can do this work and earn some good amount of money then you will say that *“I have done this job and earned such an amount of money. Let me sit and use this money.”* You can use all the money until it’s over and once it’s over then your mind tells you to go back and do that work.

**I: Thank you another thought? (Chuckles) number 5. I see that we are now not concentrating, I am ending… I am ending… I am ending… the thinking is now going haywire because of the children. I am ending it my dear. Another thought? Are there sex workers that we know that had stopped sex work then later we see that they’ve come back? Number 7?**

PX07: The thing that can make someone to go back to that work is that may be just the way we’ve said that you’ll get someone who wants to marry you. He has married you and then you know the current life…

**I: What is wrong with it?**

PX07: You are slapped and then you say that *“you are slapping me and yet I knew how to live on my own”* I go on the reverse.

**I: Okay.**

PX07: So that can make you go back (background talk)

**I: Alright. And what challenges can happen to those who have left sex work then they come back? What will happen to them when they come back to do sex work? Number 2?**

PX02: As sex worker number 2, those who’ve stopped and gone back… you know may be you’d stopped then you can go back and something bad can come out for example like fighting for a man. Fighting over a man with sex worker number 7 who stabs you and you die. Yes, that is a challenge that can happen.

**I: Another challenge that can happen… number 10?**

PX010: It can happen this way, I mean you stopped and then you came back you get that you lose clients. Now you don’t have clients, you don’t earn anything. You see that your earning is also low that can also be a challenge.

**I: Number 9 (chuckles) she had still gone to Dubai. Number 1?**

PX01: I live in town, I take my tea comfortably, I fry my meat I cook it well then I go back to the village and it’s take a hoe you go farm, they cook vegetable without adding cooking oil so I throw in the towel and say *“no let me go back to my work, I was eating my fried meat and I control myself”* I have to go back.

**I: What challenge… what bad things happen to those who stop and then come back? What bad things… number 6.**

PX06: No I don’t have.

**I: You don’t have for now. Number 2 do you want to add something?**

PX02: The bad thing is that you can go back to it… you can stop it then you go back then you find that stopping it is now so difficult unlike when you stopped the first time.

**I: Thank you. Number 5 you wanted to say something?**

PX05: As number 5 I am saying that once you have stopped and then you come back, when you go back you might find that you are advanced in age or sometimes the girls… the chicks… they young girls have joined in the work so you’ve lost the clients that you had because they are now only seeing the young girls. You know you those people had used you. You are a face that they knew so the clients are just going to the new people who have come.

**I: Now there are people who’ve not told me anything that I heard. So it means that there are some good things that can happen to those who’ve left sex work and then came back, isn’t it? Now some people to tell me the bad things. Do tell me the good things that happen to them. There is none? (Laughter) tell me the good things in case they are there. What good things can happen to those who stopped sex work and then came back? Number 2.**

PX02: The good thing that can happen as number 2, you can get stop it and you go do other things and you can go back and you meet a good client who might even help you in a good way and give you some good money as you want.

**I: Another thought, number 8? No idea. Good things. Are there good things that can happen to someone who stopped sex work and then came back? Anything good? Okay, it’s like that no one has any thought about it but should anyone think of it then she can tell me as I ask my last question. What do sex workers want to do before they stop sex work? What are the targets sex workers might have? What do they always want to first do before they stop sex work? Number 2… number 10?**

PX010: You’ve started your own business and its operational. Now you know once you started your business and its operational then you can stop this work because you now have a place where you can earn.

**I: Thank you. Number 6?**

PX06: If you had a child that you were taking through school and that’s what made you join that work and the child is done with either college or university education then you can stop.

**I: Any other person, a different thought? Number 3.**

PX03: If you feel like, out of good will you can stop.

**I: If you feel like?**

PX03: If you feel like it, you can stop.

**I: And is there any target that they say that *“I have this target… before I stop sex work this is the target that I have”.***

PX03: Maybe she has reached her target.

**I: Like which one?**

PX03: Maybe it was good life, owning a vehicle and so on, so she is tired and she feels that she stops.

**I: Okay, thank you. Number 5.**

PX05: The target that we can have as sex workers is that you need to invest big because this is some very difficult work that at least when you stop you need to say that I can stand on my own. You can buy a cow or cows and keep so that when you stop you will be seeing them calving. You can also buy some piece of land when the business has boomed then at least you’ve bought some piece of land somewhere and built some residential houses. You have invested in something that should you stop work you will always remember it with.

**I: Alright. Now my last question is asking that are there sex workers that we know that had stopped sex work like 5 to 10 years ago? Number 2.**

PX02: As sex worker number 2, there is.

**I: You agree… why does someone stop?**

PX02: Just the way it has been said, you can stop because you are tired of it, you can stop because the thing that was making you do it you have…

PX05: Accomplished.

PX02: You’ve accomplished.

**I: What makes it easy for someone to stop this work? Number 5.**

PX05: There is someone whom I have seen who left some time back… there is a woman who left following what she met… faced. She met someone who’s thing… his tool of work was so long that she ran… she ran away and even left the money that had been paid for her at the counter. She climbed through the window and left naked as she was and stopped totally. So the experience of what she saw happening to her made her stop.

**I: Number 2.**

PX02: As sex worker number 2 I want to add something there that we need to be thinking at all times. That can make us stop… we can have salvation of God that He helps us (laughter).

**I: All that laughter, does it mean there can be no salvation?**

PX02: True God can help in that I say as sex worker number 2, I want to stop this work. I have done a lot so God let me walk with you, or have I said something that is wrong?

ALL: You have not said anything wrong.

**I: That is your thinking.**

PX02: As I add someone can stop sex work depending with if she finds a client who takes care of her well. (Baby crying) she is brought for everything so she does not think of the past.

**I: Thank you. Is there any other thought before we end? Okay thank you all. Those are the questions I had. I sincerely thank you for taking your time to come and have this discussion and we hope that the views you’ve given us will help us to see how to make this program better. Alright. Sorry for taking much of your time. We’ve ended the discussion at 1710 hours. Thank you so much.**

END OF INTERVIEW.
