# Supplementary material for: Eat a little and save a little: A qualitative exploration of acceptability of a potential savings intervention to reduce HIV risk among female sex workers in Western Kenya
Source: PLoS One. 2024 Dec 19;19(12):e0310540. doi: 10.1371/journal.pone.0310540 (PMC11658496; doi:10.1371/journal.pone.0310540)
Supplement: S1 File — (ZIP) [file pone.0310540.s001.zip › Jitegemee Transcripts and Dissemination Notes for Journal/Jitegemee Study Dissemination Notes.docx]

**Jitegemee Study Dissemination notes (Qualitative Results) .**

The Dissemination activity took place on the 19 April 2023. 20 Peer Educators (PEs) of the Female Sex Workers from Siaya and Kisumu counties congregated together with 9 study staff who were involved in the study activities. The purpose of the dissemination was to seek an understanding from the Peer educators regarding the FGD data analysis.

The major themes that emerged from the data analysis included: FSW expenditure, FSW sources of income, FSW saving and challenges with saving, FSW living beyond their means, loaning, leaving sex work, rebound to sex work, Jitegemee Intervention acceptability and feasibility and challenges of the intervention.

Generally, the PEs were in agreement with the data collected and analyzed under the themes FSW expenditure, sources of income, savings and challenges in savings. In addition to the **findings on living beyond means** the PEs said competition among peers was a reason the FSWs spent more than their earnings. To supplement their income, the FSW steal from the clients and in other instances will charge high. Both these ways were seen to have drawbacks. For example, charging higher would lead to one losing clients. They also said sometimes it’s the clients who steal from them by drugging them or sending money via mobile money and reversing it later or some clients would refuse to pay the agreed price once the service had been delivered. Some clients could give false promise of paying on a later date which they in turn do not honor.

Another issue that emerged during this discussion is the **challenge of price control** at the hot spot. Some FSW ‘spoil’ the sex price by agreeing to very little amounts during negotiations. This could be due to some being new/young and unaware of the price at the hotspot or lacking skills of price negotiation. The fear of losing a client to another FSW especially if one had not had any client on the day was also another reason for failure to negotiate for fair price. Some reported that once a client is used to a particular price having them to move upwards becomes difficult. Clients curiosity to sample new faces which was termed as ‘rubber stamping’ was reported as the reason why the clients sometimes preferred to go with the new faces at the hot spots.

**On loaning,** the PEs said that in addition to the lending sources mentioned in the FGDs, the ‘Hustler fund (a loaning system introduced by government for the low income earners) was another source of loaning. They however said its terms of lending were very unfavorable. Moreover, Maasai loaning was seen as exploitative because not only were the Maasai’s charging exorbitant interest rates but they also engaged unorthodox ways of recovering their loans which included gang raping the FSWs. It was however reported that in some instances the FSW proposed the paying with sex when they were unable to pay back the loan. Other than the money the Maasai also sell fabrics and sandals on credit to FSW. As a way of showing commitment for repayment National ID, phones and electronics were used as surety in other instances. FSW were against the idea of Jitegemee providing loans because FSW would take loans and disappear.

On **leaving sex work**, the PEs were in agreement of reasons that emerged in the FGDs for wanting to quit sex work. One PE stated that sexual violence especially gang raping which cause both severe physical and emotional injuries may push one to quit sex work. They believed failed marriages were the main reason for rebounds in sex work. The failed marriage could be due to sexual dissatisfaction or husband not being able to provide as expected which came up in FGDs as well. Husband’s attitude towards the children out of wedlock was also mentioned here as a reason for returning to sex work.

The PEs recommended training on the following areas in order to help those who plan to quit sex work sustain their departure: life skills such as entrepreneurship, long term investments such as real estate and educating their children, alcohol and sex addiction.

**Jitegemee Intervention:** The PEs, just like their peers who participated in the FGDs had a high acceptability of the intervention. However, they did not support the idea of the FSW being in charge of the intervention as recommended in the FGDs because of the general trust issues that exist among the FSWs.

Some of the concerns that came up included.

- Varied opinions regarding loaning. Most felt that the intervention, as it was structured was fine because the members would not worry of people relocating with their money as one would only be allowed to withdraw what they had saved. Others however felt that loaning would encourage members to save in Jitegemee.
- One PE felt that rules and regulations on saving should be stringent to discourage those FSW who have a long term saving goal from reaching out to the savings anyhow.
- Others proposed for the intervention to introduce loaning on personal savings
- They hailed the idea of the intervention for having no cost on the transactions

**Green Lodge** was defined as having sex at the bush, shrubs or in the grass. This is a concept that was not discussed as much during the FGDs but the PEs mentioned that it was widely practiced especially in the rural set ups where chang’aa dens exist. They said that it offered more privacy and it was cost effective as it did not involve room charges. Circumstances that lead to people using green lodge include

- Unavailability of rooms for example if one meets a client on the way where there are no buildings
- For confidentiality as people will not see you walk with a client to a guest house
- It does not require one to dress up and put on makeup it can happen even when one is coming from fetching water or fire wood
- When clients have no money to rent a room
- Some FSW proposed it to the clients who did not have enough money to pay for both the room and for sex

They however reported green lodge to be unsafe as one may be attacked by animals such as snake or the clients may take advantage of an FSW. They instead advocated for cheap rooms for safety.

Other places where the FSW exchange sex with their clients include unfinished building or ruins and under the heavy tracks

**Surprises Observed.**

1. There was a divided opinion on the issue of putting on make-up and dressing up to attract the clients. Some FSWs felt that it did not matter whether you had make up on or not because the clients were less attracted to that while others felt that make up and dressing in attractive attire had a way of pulling the clients. Some PEs said that some clients preferred light skinned ladies hence reason why they use make up to look lighter. Those belonging to this school of thought said it cost one more to earn more.
2. One PE did not agree with the findings that sex work is addictive and contributes to FSW rebound to sex work because of the satisfaction they get from having sex with several people.
