# Supplementary material for: Eat a little and save a little: A qualitative exploration of acceptability of a potential savings intervention to reduce HIV risk among female sex workers in Western Kenya
Source: PLoS One. 2024 Dec 19;19(12):e0310540. doi: 10.1371/journal.pone.0310540 (PMC11658496; doi:10.1371/journal.pone.0310540)
Supplement: S1 File — (ZIP) [file pone.0310540.s001.zip › Jitegemee Transcripts and Dissemination Notes for Journal/FGD A.doc]

**DATE: 25/APR/2022**

**MODERATOR: RA 002**

**NOTE TAKER: RA 005**

**FGD ID: FGD A**

**CATEGORY: BELOW 30, URBAN.**

**I: Now we are starting, we are beginning our discussion. Based on how I have explained to you about Jitegemee in short, what comes to your mind now? After explaining to you what we want to do in Jitegemee, the way we want to do it. What comes to your mind… what comes to your mind based on what I have said? Yes, number one.**

PA01: As number one, what comes to mind is that I feel Jitegemee is going to help us because like us … me as number one sometimes I get something small then I spend all of it, I could not save and you go to look for some the following day, so personally I think it can help me… it can help me if that account is opened.

**I: Okay, that is number one, yes number five.**

PA05: As number five, I feel Jitegemee is helping us in preventing the high rate of HIV infection, because if you go for that work for one week and you rest the next week, maybe you were going to get the infection that week that you rested. So I feel that it is really helpful, it reduces the chances of HIV infection.

**I: Okay, any other person with a different opinion rom number one or number five. What comes to your mind after hearing what Jitegemee is intended to do?**

PA06: Say it in Kiswahili.

**NT: Okay we are asking that based on the short information we have said about Jitegemee, what is your first reaction about it?**

PA06: The first one?

**NT: What you think about Jitegemee.**

PA06: Is to open our mind.

**NT: In what way?**

PA06: Such that if you meet even a client at the hotspot and he gives you even ksh.1000, you spend ksh 500 and save the other ksh 500.

**I: So are you saying that it has opened your mind now or it is something you have been…**

PA06: I used to spend everything. I go and buy meat and cook then I eat everything, the following day I go back.

**I: Okay. So you feel that it has opened your mind.**

PA06: Yes.

**I: That is number 06, any other person?**

PA08: As number eight I feel that that thing has opened my mind because… Jitegemee has opened my mind because I used to get and spend, I was not thinking of the future, so I can get it and save it because it will help me in the future.

**I: Any other person? Just feel free if you have any opinion do not hold it back, just speak it out.**

**NT: Even if you are not agreeing with it, just say.**

**I: Number 10 is smiling as if she has something to share.**

PA10: As number 10, I feel that Jitegemee has helped me because it is going to make people reduce their risk of HIV infection. So you may have a client and if that client demands, you can decline.

**I: Any other person.**

PA02: Me I have another option.

**I: That is number 02.**

PA02: Yes, as a sex worker, apart from sex work you need to have some side hustles so if you go for sex work today then tomorrow you go for the side hustle you save the money you get from sex work then save the one you get from side hustle so you use it for other things.

**I: So have you learned that one today or it is something that you have been doing?**

PA02: I have learned it today.

**I: Any other person?**

PA03: As number three I feel that Kujitegemea helps because like me when I received money from those clients, I could spend all, I don’t think of savings. It will help in savings; you spend half then the rest you save.

**I: Okay. Any other person, you can speak in English, dholuo or Kiswahili. Whichever language you have thought of.**

06: Number six like me again, I would like to enlighten those people on the ground, I want to enlighten them …they should do what…they should not just see alcohol and there is no money yet they have some responsibilities to attend to.

**I: Who are these people on the ground?**

R: The people like me who are doing sex work. For them once they have seen alcohol they change their minds they don’t think of money being in their pockets.

**I: So you think Jitegemee …**

PA06: Will help them.

**I: Will help the…**

PA06: I have even told them.

**I: Yes, any other person? (Long silence) if there is none then we can move on or is there any other person who would like to talk? Number seven do you have something to say?**

PA07: No.

**I: There is none. Number nine, number four you are okay?**

PA04: Yes.

**I: Nothing has crossed your mind, what you have thought of.**

PA04: No.

**I: Okay and … let me ask in dholuo then we will explain, is it okay number six?**

PA06: Yes.

**I: What do female sex workers buy with their own money? What do you always buy with your money? There is that money number six said she gets and all of it goes to the sufuria (meaning she spends everything) now what do you always buy?**

PA08: As number eight, I buy clothes, food and I also have to buy beauty products so that the clients can see me.

**I: Okay that is number 08, number two?**

PA08: For me I have to do some shopping for my child and rent and also shopping… my upkeep.

**I: Okay let me first go back to number 08, you have said that you buy?**

PA08: I buy… I use that money to buy clothing, food, rent and I also buy beauty products. I make myself beautiful so that I attract clients.

**I: And are you able to know how much you use in food, clothes and all the others that you have listed?**

PA08: I can use around ksh 200 per day on food.

**I: In a day?**

PA08: Let us say, not the whole day. Like in the morning, then you know during lunch the expenditure must increase so that is ksh 300 and even at night. So as from morning up to night I can use about ksh 1200.

**I: So that is on food.**

PA08: On food.

**I: And what about on beauty products?**

PA08: On beauty products I can use around ksh 1500.

**I: is it daily?**

PA08: No I buy beauty products for the month.

**I: So the ksh 1500 is for the month.**

PA08: That is for the whole month.

**I: What else did you say?**

PA08: Clothes, for the clothes I have to buy them daily. I not put on just one cloth daily and yet I am a sex worker. I have to put on different clothes on a daily basis.

**I: So you have to buy clothes on a daily basis?**

PA08: Every day.

**I: And how much is one cloth?**

PA08: A cloth is around ksh 500.

**I: That is every day?**

PA08: Every day. Is there anything we have left out?

**PA08: Rent. Obviously in Kisumu rent is rent is ksh 2000 upwards. So personally I stay in a house of ksh 3000. Rent.**

I: So that is monthly.

PA08: Monthly.

**I: Okay, number two also gave us a list, may you kindly remind us.**

PA02: The child’s upkeep let us say monthly we use ksh 2500, it is a monthly shopping which you cannot do daily. So the upkeep is ksh 2500 and then rent is ksh 2500.

**I: Per month?**

PA02: Yes, and then personally now maybe per day I use around ksh 500 which caters from morning to evening and lunch.

**I: So that is ksh 500 for food?**

PA02: Yes.

I: What of clothes?

PA02: Clothes are things I always have so I cannot buy them on a daily basis.

**I: Okay.**

PA07: As number seven, I always use… like I do not buy clothes every day, it is once in a while and with breakfast once I have prepared tea that is what we use up to lunch and I use ksh. 200. Then supper, I don’t use a lot of money on a daily basis. I may eat fingerlings today then tomorrow I eat fish. So you know it is not the same on a daily basis. Today I may use ksh 500 and tomorrow I may use ksh 200. For the beauty products I don’t buy them daily. When I buy once and they stay for long, it does not clear once so I can refill. If the powder is finished today, tomorrow I buy lipsticks. They don’t get finished at once so I just refill. When one is finished, I buy another one.

**I: And when you buy all those that you have mentioned, how much do you spend? The beauty products.**

PA02: Around ksh 1200.

**I: And how long does it take?**

PA02: It takes one month but it does not get finished.

**I: Ok so they get finished one by one then you top up.**

PA02: Yes.

**I: Okay and you have said that you do not buy clothes every day?**

PA02: No.

**I: How long do you take before you buy?**

PA02: After Sunday… every Sunday… at times on Sunday and at times I don’t buy. It can take even two Sundays is when I buy another one.

**I: And how much do you always use?**

PA02: On phone?

**I: On clothes.**

PA02: on clothes.

**I: Yes, on that Sunday when you leave to go and buy clothes how much do you use?**

PA02: I can even use ksh 500.

**I: Ksh 500. Ok and number two also told us that she does not buy clothes frequently but when you buy, how much do you spend and after how long?**

PA07: Ok there are two types of clothes, we have second hand and we have ready-made, so for the ready-made there are those that ranges between ksh 1000-1500 and above. For the second hand we have those for 200/=. So when you go to buy the second hand one you take even for 1500/=, maybe if I buy mine and for the baby. The ready-Made one starts with 1500/=

**I: Okay so averagely how much do you always use:**

PA07: Averagely let us say around 4500/= on clothes

**I: And how long does it take?**

PA02: It takes time; I always take two to three months.

**I: Two to three months, okay. Any other person who would like to add?**

PA01: As number one.

**I: Daily expense, start with the daily expense, that which you buy daily.**

PA01: The daily one is food. I can support number seven because even me when I prepare tea in the morning, that will take me until lunch time then Iook for supper so at night I may use 200/=. If I have flour … if I buy flour I can take even one month with the flour, if buy floor I can take one month with that floor so I can just buy food on a daily basis of which at night I can use 200/=, so day and night I may use 400/= for food.

**I: So that one you buy daily.**

PA01: Every day.

**I: And what do you buy per week?**

PA01: Personally there is nothing I buy weekly because if it is clothing, I take long before buying and when I buy I don’t buy it on a daily basis then when I buy I go for second hand since second hand is cheap.

**I: And how long does it take you before you buy clothes?**

PA01: I may take two months then buy second hand clothes worth 500/=. Then also for the beauty products, they stay for some time, I can even take even four months before they get over.

**I: Any other person who would want to add? I have not heard you talk about children, sickness, money you send to mother, airtime because don’t you call those clients? Yes, so you can also add that one in the list.**

PA05: As number five I may become sick and seek treatment using this money and if I get treated free, I can go and buy medicine. Same to the child, same to my parents or even relatives. I may make my hair; don’t we use money to make our hair…yes so I may make my hair. I will buy airtime, shoes and I can pay fees with that money.

**I: Okay so you have not told me if you always separate some money to go to the hospital or do you always have that money ready to go to the hospital when you are sick?**

PA01: I have to set aside, I may set aside …like if I get 1000/= I save 500 and save 500/=

**I: So how much do you always save for hospital?**

PA01: That one will depend on how I have worked/earned. If I get 1000/= I save 500/=

**I: Where do you save?**

PA01: From today, as in from today.

**I: Ok you were not saving before.**

PA01: Yes.

**I: It is when you want to start, you want to start. But now I was asking not what you are going to do, I am asking about what you have been doing… what you have been doing and the amount of money you get. Some people have already sais clothes, beauty products.**

PA01: making hair.

**I: Like how much do you always use	in making your hair?**

PA01: 1000/=

**I: After how long?**

PA01: One month.

**I: Okay, any other expense?**

PA07: As number seven, I don’t always save for medication, you know when you save money and you become sick, you will be forced to withdraw and use but money for children education and house rent which you know that every month I must have… that is a must because you cannot stay without paying rent and again your child cannot go to school without fees that is a must but with sickness you can never predict that you are going to be sick. You can stay… you can even survive without becoming sick because you cannot predict of sickness but when you save money you cannot continue saving it if you are sick, you must withdraw it.

**I: So you always save for rent and fees?**

PA07: Yes.

**I: And do you save daily or weekly or monthly?**

PA07: Every day after I have deducted the amount of money for food and I remain with some cash I save. You have to do your calculations because at the end of the month I have to pay rent and I also have to pay fees for the child.

**I: So do you always have a target or you save whatever you have?**

PA07: I save whatever I have.

**I: Ok any other person? The money you have received; how do you use it?**

PA03: Like me number three I can use … because there is also the saving issue, so I can balance the issue of rent and school fees and food for the children. Let me say I use around 400/= for food per day and then being that the school fees for the child is paid monthly, you can save even 1000/= per month you take to school and then there is some money to send to parents at home let me say like 1000/= and based on what I am doing let us say I get 10000/= so I divide it in the middle, as in I use 5000/= for my personal use then I save the other 5000/=. So I will use the 5000/= for personal use and the child then also send for parents back at home.

**I: Ok that is an opinion from number three. Any other person, number six.**

PA06: Number six, me I have different responsibilities. I have one client whose responsibility is to pay rent, another one responsible for buying food, and another one whose responsibility is to pay school fees. I just have them in line like that. The one for rent must pay rent, the one for fees must pay fees, even the one for fees must pay the fees at the end of the month, I have children. And I will need money, if the child is sick I will not use my money to take the child to the hospital, that is why I gave each one of them some responsibility Client responsible for medication must pay when the child is sick, when I get mine I save in my phone.

**I: So you have said there is baba…?**

PA06: Baba rent.

**I: So is that a separate client?**

PA06: A separate one, I have them in line like this.

**I: So each of them is given some responsibility?**

PA06: …responsibility. I cannot manage on my own truly speaking. The amount I get from sex work is also little, it cannot be enough.

**I: And have you given them a target or just whatever they send?**

PA06: No just whatever they will send though the one for rent will just have to pay rent. He will just have to pay the three thousand, he cannot send 1500/= where will I get the other part yet I even pretend I don’t do anything. Once he has left the house I also dress and leave for my hotspot and I enjoy myself to the maximum.

**I: So the one responsible for rent gives three thousand per month?**

PA06: Yes, and the one responsible for shopping gives 2500/=

**I: And for…**

PA06: The one for school fees, I just send him the playbill number then he sends direct to school I just receive message on phone.

**I: Like how much is it always?**

PA06: It changes, it goes up to 8000/=, 10000/= and at times it comes down to 5000/=.

**I: Ok and the baba fees [ client responsible for fees] pays faithfully.**

PA06: Yes, he pays faithfully so that whenever he comes …fish... I turn for him like fish. He paid my child’s school fees up to fourth form.

**I: What of food?**

PA06: The one for food is also there, as I have already told you that he gives 2500/=

**I: Ok that was number six, but now you have not told me concerning dressing, beauty products**

PA06: Let me tell you I have so many clothes, if I buy they will curse me. I have suitcases.

**I: So it is not a must that you buy.**

PA06: It is not a must that I buy clothes; let my children complete their schooling first.

**I: What of the beauty products?**

PA06: I buy the beauty products at 1000/= every month.

**I: They don’t get finished in the middle of the month?**

PA06: No, it is not something to apply all the time, you know these things you only use them after you bath then you wear a concentrated one [cross talk]

**I: Ok number four, how do you always use the money you get?**

**NT: I also wanted to hear her voice.**

PA04: Paying rent, educating the child and sending to parents at home.

**I: So on a daily basis there is some which you buy on a daily basis, which ones are they?**

PA04: Beauty products.

**I: Do you buy the beauty products on a daily basis?**

PA04: No, Food.

**I: Ok now you buy food on a daily basis, how much do you use?**

PA04: 300/=.

**I: 300/= is that enough for the day, breakfast, lunch, supper?**

PA04: Yes.

**I: And what about the ones you do weekly? In a month, you have talked of rent and school fees, how much do you pay for rent?**

PA04: 3000/=

**I: What about school feel? Or do you have baba school fees? You have one responsible for school fees? So does he pay direct to school or he sends to you?**

PA04: He sends to school.

**I: So there are those that we buy on our own are there are those that we delegate to the clients?**

PA04: Yes

**I: Ok, it is not only number 06 and number 04 who do that?**

PA02: You cannot manage it alone or having only one partner, you can’t.

**I: That is number two, may you now explain further.**

PA02: You have expenses, you want to pay rent, you want the child to school, you want to do shopping, you want to dress well, you want to make yourself beautiful so you if you have only one person, this one person also has his needs to meet, so you know you must have like five or six people (clients) who can support you when you tell him that you are in need somewhere, he sends money.

PA07: And again if you leave all the responsibilities to the men they will leave you so you must also plan.

**I: So there are those they do and there are others that you do?**

PA07: Yes, he cannot do everything.

**I: Ok and like number six said that there is someone responsible for rent, one responsible for school fees and one responsible for shopping, so this money that you get from sex work…**

PA06: It is here in the Mpesa… it is in the Mpesa. That one I don’t even talk about, it is mine.

**I: But what do you use it for?**

PA06: This is my emergency like funeral. If my mother ran’s out of food, I send her to buy food.

**I: Okay what of others who have persons responsible for school fees, for rent, when you get your money, what do you use it for? Number six has said that she sends it to the funeral, she sends to mother.**

PA07: When they (the clients) do not have money you use it but they will have to refund.

**I: Ok so if you use they will refund.**

PA07: Yes, I will say that I borrowed from my neighbor and my neighbor wants back the money, I cannot even walk outside because the neighbor wants back the money yet it is my money I want to take it back to my Mpesa.

**I: Ok so sex workers’ money is saved?**

PA07: Yes.

**I: Ok and this one which is being saved, where does it come from because there is that person who is responsible for fees, there is that who is responsible for rent…**

PA06: So that is rotation… rotation (cross talk) if you get someone who gives you sugar… after giving you the sugar he leaves, that is where you will get this money so it just goes like that.

PA07: If you keep on using your money he will get used to it, so you rather cheat him. He may pretend that he does not have yet he has so you rather cheat him that you have borrowed then he will refund. If he refunds you save.

**I: So the one you get from the hotspot is the one you have in the Mpesa?**

PA06: Yes, it is in the Mpesa.

**I: And these clients responsible for rent, responsible for school fees, where do you meet the?**

PA06: We meet them at the hotspot, because they don’t stay there, maybe he has gone to see the wife for the weekend then he goes, I will not give him space, I will immediately call him, he will send money.

**I: Ok and is there anyone who would want to add before we proceed? Is there any one I have… number nine… number nine you always get money, is it? Just talk even in dholuo, we understand all the languages.**

PA09: As number nine I support number six, you must eat, the child must eat and anything you may want and whatever you get you save. When you do not have something… whatever you will be having the child will say “mum I want this” will you decline, you will have to buy for her.

**I: So like how much do you use on the child? And is it on a daily basis or what times do you spend on the child? How much do you spend on the child?**

PA09: 500/=

**I: So what is that for?**

PA09: I use it to buy food.

**I: food for both of you or only him?**

PA09: Since they are two, that is just for the two of them.

**I: Ok it is just for them. Any other thing you spend on a daily basis? It is 500/= for the children, any other?**

PA09: Making my hair.

**I: Daily?**

PA09: No on a monthly basis.

**I: How much per month?**

PA09: 1200/= then I also buy shoes, there are those of 500/= but that is not on a daily basis.

**I: How frequently do you buy that one?**

PA09: Even after two weeks.

**I: Ok any other person, who else has not spoken, number 10 have you said how you spend?**

PA10: Just the way they have said it.

**I: Ok they have already spoken. So on this one you have already told me something but I request that you top up, where this money comes from.**

PA07: Money?

**I: You have already told me that there is hot spot where you work and there is this which is sent by the person responsible for rent, the person responsible for school fees and the person responsible for shopping. Where does the other one come from, the money you use? I remember number two had told me before that she has a clients and there is hustle.**

PA02: Part time job.

**I: Part-time job, may you please tell me more on that how you do it as sex workers, there is sex work, there is client and there is money received from the clients and there is hustle. How does it happen?**

PA02: You don’t have to get the clients all the time.

**I: That is number two.**

PA02: You don’t have to get the clients all the time, during this time that you do not have a client you have a part time job which you can do. So instead of sitting down waiting for a client you can do the part time job and you get money.

**I: Like which one?**

PA02: Ok like me I work in a salon.

**I: Ok so some money comes from the clients and some come from the salon. What of the other people? Where does the other part of this money come from?**

PA06: I sell “chalding’”, do you know chalding’, that is where they flow like water.

**I: That is number 06.**

PA06: Illicit brew. It has money but the money is still not enough.

**I: Okay, so that is side hustle.**

PA06: Yes.

PA05: Washing for people. Number five I can wash people’s clothes.

PA07: Working in a hotel.

**I: Number seven is saying she works in a hotel.**

PA07: Salon.

**I: Number nine is saying salonist. Number 04**

PA04: Bang’.

**I: Number four is saying she sells bang’, number three?**

PA03: Salon.

**I: Salon, number eight?**

PA08: I sell ‘muturaa’

**I: You sell muturaa, yes number 10 would want to talk.**

PA10: I sell chips.

**I: Chips, number one?**

PA01: At times I even go for construction work.

**I: Number one can even go for construction work.**

PA01: Yes, so long as it income generating.

**I: So earlier you said that there is that money which is saved in the Mpesa for emergencies like funerals, sending to your mother, now this money you get from side hustle, is it the one saved in the Mpesa or what do you do with it?**

PA06: Like me I have a girl who is already grown up but I still do not want her to have a boyfriend so what I do… she is seeing me doing this work, she knows that this work of sex work I am doing is not of my wish, …she may want something so I will give her something to buy what she wants and if she wants to go swimming she can just go and enjoy together with the brother. When they come back in the evening, I again give her some money to go and buy chips they enjoy together with the brother as I go to my hotspot looking for clients.

**I: That is number six, any other person, this money we get from side hustle, how is it used?**

PA02: Like me the salon work… if it is your own salon, then it pays well but the way I work for someone in a salon, maybe in a day you come out with four hundred and maybe you have some debts.…. Leave alone the one we receive in our Mpesa. So the one you get from the salon is what you use to settle your debts then you get your saving to buy food.

**I: That is number two, so you are saying that salon can give you around 400/=per day. And you said chang’li… how much can you get from it?**

PA06: It has money; you see this small ‘dasani’ bottle is three hundred.

**I: Is that profit or…?**

PA06: No that is just selling, if you sell one that is 300/=, maybe you have five liters. Five liters contains how may bottles?

PA07: six.

PA06: Six of them and maybe you get 1000/=, it has profit. I even feel I should leave this sex work because it is making me run at a loss, I rather sell illicit brew and get money.

**I: That is number six, so do you sell the whole of this in a day or for how long can you sell the five liters?**

PA06: Let us say on weekends like Fridays, Saturday and Sunday the whole of five liters.

**I: One five liter or?**

PA06: I sell the whole of it.

**I: But on week days?**

PA06: I sell bit by bit. [Cross talk] you can sell for 30/=, 50/=, 30/= just bit by bit then you go.

**I: So number seven is helping number six to contribute, does it mean you also do the same side hustle or which one is your side hustle?**

PA07: Mine is the bottled one ’waragi’

**I: Ok so how much do you get from waragi per day?**

PA07: 500/=

**I: That is your profit?**

PA07: Yes.

**I: Number 06 you have said that if you sell five liters…**

PA07: five liters I get 1500/=

**I: If it is weekend you get 1500/= per day… 1500/=**

PA06: Yes 1500/= if I sell the whole of it. That is on weekends but during weekdays I sell bit by bit, 30/=, 50/= as long as whenever a customer comes he does not miss.

**I: Okay, for others what is the side hustles?**

PA03: As number three I support number two, I don’t work in my own salon, I work for someone so per day you may come out with even 300/=. As you can see the three hundred is very little money so it can help me on these small things like sorting out debt if I had someone’s debt or if parent wants something at home , you send.

**I: And when you have a good client, do you still go to the salon or now you don’t go because the money from salon is little?**

PA02: Okay when you go for sex work, in the field it is just negotiation.

**I: That is number two.**

PA02: You must agree on a certain amount because the previous day you worked so the next day you can rest.

**I: So the day you get a good amount of money you don’t go to the salon?**

PA07: You do not go to sex work (dira) at night, you just go sleep at work then at night you go for sex work.

**I: Number seven does not choose, you go for sex work and the same time you go to work.**

PA07: Yes.

**I: What about the others, number four you have something to say.**

PA04: But I will go and wash for someone, I will go to work during the day and then at night I go for sex work. So I can use the money I get at work in buying sanitary towels or airtime.

**I: That is number five.**

PA06: I want to support number five, there is a time I went to wash for a certain man after washing, now he wanted sex. I came out with double pay, I came out with 1500/=. He has even called me some minutes ago, I have seen his missed call.

**I: So he again wants you to go and give…**

PA06: Yes, he wants it again, I will give her, this(vagina)is not soap which clears up, he will just fulfill his urge then it (vagina)will remain where it is.

**I: Okay that is good so we get money from sex work and we get money from side hustle. And is it possible for someone to just wait for clients without side hustle? (cross talk)**

PA07: Yes, there are those people who just depend on sex work without any side hustle.

**I: So how do they manage?**

PA06: So they are in need, will come knocking the door, I can really chase her away, she is waiting to go to that cold while she goes to sleep early, I go to that cold then she comes to me to give her even salt… I cannot … let her go the way I went.

PA07: There are those who are just waiting for the time, when they see it is nine they leave

PA06: I cannot help such a person.

PA07: They do not do any other thing.

**I: Okay, I can see we have forgotten about saying ‘as number 06, as number 07’… I am reminding you. So you are saying that there are those who just depend on sex work. But they come to you for help. Are they many or they are just but a few?**

PA06: They are many. There are those who are not yet exposed and even if you make them to be exposed they don’t.

PA07: Some of them are afraid.

**I: Thank you so much so when we want to speak, please I request that you just say, I as number 04, as number 06, do not forget. And there are those times when there are no clients, are there times when clients are not there? (yes- in chorus) and you have responsibilities. (Yes – in chorus), how do you survive?**

PA08: As number eight I survive with the side hustle. The little I get from the side hustle is what I use to take care of my needs.

**I: Okay, what of those who said that they work in salons, how do you know that today there are no clients so I can go to work in the salon?**

PA02: Like me there is mobile phone.

**I: Number two.**

PA02: Yes, like me number two, I do use mobile phone so most of the time if I stay in the house the whole day without seeing any phone call, it will force me to wake up and go to the salon the next day.

**I: Okay, so you have the ability to know that that there are no clients let me go to the salon, Another person.**

PA07: Me as numbed 07, even if I have many clients, if I call even one of them he cannot miss to send me even 500/=. It is not a must that you have sex is when he gives you money, even if you call them telling them that you are broke, immediately they send you money in your mpesa.

**I: Thank you for that, what I wanted to know when the clients are not there, what do you do? number 02 has already told me that you always have the ability to know like ‘because I have not received a call, things are bad can go to the salon’. Yes, number 01**

PA01: As number one if there are no clients and I don’t have anything I can borrow because I know that if I don’t get clients today I will not miss tomorrow.

**I: And how do you tell that there are no clients?**

PA01: But you can use phone to call them. If they have not called you then you can call them, so it is just a matter of asking.

**I: Ok, so maybe you have asked and they have told you that they are far away.**

PA01: Yes, so if you were badly off, you can borrow… maybe he will tell you that he will send you tomorrow or the next day.

**I: Any other person.**

PA03: As number 03, if the clients are not there you go to your savings so that is where now the savings will help you.

**M: Ok, and how do you always tell that the clients are not there.**

PA03: Just over the phone.

**I: Ok, on phone.**

PA03: If they have not called you, you just know that they are not there.

**I: Ok, any other person.**

PA05: Number 05, like you go every week, Monday, Tuesday, Wednesday, Thursday… you just see… in most cases there are usually no clients in the clubs on Monday, Tuesdays, Wednesdays. So as from Friday, you just know that you will just know there are clients because it is somewhere you go every day.

**I: So on Monday, Tuesday and Wednesday what do you do to survive?**

PA05: I just go for the side hustle.

**I: Now as we were talking you one by one there is something you told me, when you compare side hustle and the money you get from sex work, which one helps you the most or…?**

PA02: As number 02, sex work helps me the most.

**I: Number two has not yet mastered to say number 02**

PA02: As number 02, sex work helps me the most.

**I: Yes, what about the others,**

PA08: As number 08, I support number 02.

PA04: I support number 02.

**I: Number 04 is also supporting number 02.**

PA09: As number 09, I support number 02.

**I: Ok, number 06.**

PA06: Personally the illicit brew helps me the most because you know with the illicit brew a customer must come be it in the morning or at night you will just hear someone knocking the window then I wait to hear the name, once I have heard the name I wake up then I give him through the window even when it is dark but this one (sex work) …

PA07: Sex work is not for a daily basis.

PA06: Sex work is not a daily basis thing; you will raise alarm … you will sing…

**I: So you are saying that sex work does not help you so much?**

PA06: It does not help me so much and I don’t concentrate into it so much. I have already told you I have someone who one who represents house, fees …and everything so the illicit brew helps me push with my children.

**I: Okay.**

PA07: Can I correct something a bit, these clients ‘mababa’ is there a specific time you meet them?

PA06: When they are coming they have to call first.

PA07: Yes, I don’t dispute but do you meet?

PA06: Yes.

PA07: Yes, so they are helping you, sex work also helps you.

PA06: Sex work helps but what about that time when they are not around, when they are not there I work with the illicit brew clients who can even pay and they forget to take the balance, is that not profit on you. She gives you 1000/= and 400/= is yours because by that time he is drunk he does not understand himself. Because I have children, every day I put 200/= in mpesa.

**I: Ok and that is number 06 talking, and is there any other thing you do with your money? Somebody said she makes herself beautiful so that she can be seen by clients. Would you say like there is one person who said that on a weekly basis she must buy clothes, why?... the one who said she makes herself beautiful said that she does this because she wants to be seen by clients. Why should one buy clothes?**

PA06: For them to look smart.

PA01: There are those who loves being smart. As Number 01. There are some people who really love themselves, whenever they go to the market and they see a dress they feel that that dress is so smart so they must buy and put it on, that is she loves when she is told ‘you are smart’. When they are told daily that they are smart they feel so good, she wishes to buy clothes every day.

**I: So that they can look smart.**

PA05: As number 05, there are those who have grudge with others. If she sees you with this dress today she will go and buy the same type of dress tomorrow.

**I: Because she has seen you with it. Any other person?**

PA06: As number 06, within our hotspot we have a certain lady who is so beautiful, I am telling you she must get a client, she must… she is ever smart. So some people feel bad about it. There is a time she was beaten, her clothes were torn and she remained naked. That is a grudge. She was just given a leso which she used to cover herself as she went.

**I: Because she is smart.**

PA06: She is smart on a daily basis.

**I: Is there any other thing we are leaving behind on why we are using our money on what we spend on like you have said that you buy the beauty products so that you are attractive. Some people buy clothes for competition and also to look smart. Any other thing we are doing with our money? Number three do you have something to say?**

PA03: No.

**I: Now another question there are a few people who have told me about saving and not all of you told me. So do you think the sex workers save?**

PA06: A few of them.

**I: You remember to say ‘I am number…’**

PA06: I as number 06, a few of them are the ones who save.

**I: What makes you say that way?**

PA06: There are those who get clients who gives them money then she goes to drink alcohol using that money. She does not think there is phone savings and she has children.

**I: So some people drink all the money they get.**

PA06: We meet at the bar and that is where we agree to do everything and we negotiate. After negotiating here, you get out of this bar with your money and you go to another bar and you enjoy that side, you even forget the condition in which you left your house.

**I: So those ones don’t save.**

PA06: Those who do not save.

**I: And are there those who save? And why do they save? For those who save amongst us here, is there anybody who saves who can tell us why she saves, and how does she save? Or if you know of anyone who saves.**

PA08: Me as number 08, I save and I put it as locked account. I leave it for one year then I can withdraw it because there are so many emergencies that can come out in between, maybe the child is sick and you did not get a client so it will force you to go to that saving.

**I: So you have put your saving in such a way that you cannot withdraw it after one year?**

PA08: No it depends, if I save as from this year then it is after one year, next year I will not put it that way, I can say two months or one month**.**

**I: And why do you save… what is the purpose of saving?**

PA08: For emergency incase anything comes I may help using that one.

**I: Ok, and what if this emergency finds when it is still locked?**

PA08: I will borrow then… I will borrow then I will tell her then I will tell her if it is December that I will have some money in December and refund.

**I: And how frequently do you save? Is it daily, weekly, or monthly?**

PA08: I always save weekly.

**I: Weekly, okay. And are you free to tell us how much you save per week.**

PA08: I always save 1200/= per week.

**I: 1200/= okay, is there any other person who can tell us the way she does it or the way she knows the friend does savings?**

PA01: As number 01, when I save I save in the mpesa because there may be some emergency anytime, when I save money in the lock account there is no way I can help. If the child is sick and you have saved in the lock account, there is no way you can save the child, so I usually save in the mpesa so that anytime I get an emergency I can withdraw it.

**I: Ok, and how much do you save?**

PA01: Me I can save even 50/= per day, yes even 50/= I save in the mpesa.

**I: Okay. And number 08 you said that you save your money so that you can withdraw after one year or some months, why do you save it?**

PA08: In most cases I save it for emergency and then incase my mother wants some money, I just tell her that I currently don’t have but comes this month I will have it and I will give you.

**I: Ok. Is there anybody who would like to add?... and for those who save what is their behavior? You can… is there a behavior you can relate with those who save or how is it?**

PA08: As number 08, I can say that most of them are always secretive they always don’t want many people to know like personally this is where I have said it, nobody know that I save.

**I: Ok so they are always secretive with their saving.**

PA08: Most of them are always secretive with their savings, as in they do not want it to be known.

**I: And are you able to know why they don’t want it to be known?**

PA08: Somebody like me, it is because I don’t want anybody like a relative to know, you just know relatives are different, so there are those who will want to fake something so that you can help them, so I rather make it secret.

**I: Ok, yes they are always secretive, how again do they behave?**

PA05: Once someone becomes aware that you are saving, there is no way they can help you, in case you go to them they will not give you because they know that you have some money somewhere.

**I: So that is the reason why they become secretive.**

PA05: Yes.

**I: And which is the other behavior of sex workers who save?**

PA06: As number 06, friends are never good when they know that you are saving, they can even plan with other people to strangle you and snatch that phone from you and get your pin.

**I: So those who save don’t tell people that they are saving, how else are they, which behavior is also associated with them, or which characteristics are they associated with? Number one.**

PA01: Me as number one, those who save are mean. Whenever you ask them anything they will tell you that they don’t have, they feel that they should be saving whatever they are going to give you.

**I: So are those sex workers or who are those?**

PA01: Sex workers.

**I: So the sex workers who save, number 02…**

PA02: They don’t even take alcohol.

**I: Ok they do not drink alcohol, their lifestyle is also different. Ok, any other, number seven, which behaviors are associated with those who save?**

PA07: I cannot tell because I always save in the locked account. I just save in the mpesa.

**I: Ok number 09 do you have something to say?**

R009: No.

**I: Number 04, what are the behaviors of those who save?**

PA04: They don’t spend a lot.

**I: They don’t spend a lot, but is use of make up a must for the sex workers?**

PA06: No.

**I: Or that is…**

PA07: No they are mean to themselves, you don’t use all the money.

**I: What about those who don’t save, which characteristics do they have? Which behavior do they have? Those who don’t save, we have already been told one by number six that they drink a lot.**

PA07: They eat a lot, they eat, they buy food all the time and they eat a lot, she buys anything she comes around.

**I: They eat a lot; yes, number 01 you want to add?**

PA01: I just wanted to say that they don’t bargain whatever price someone tells them; they will just buy.

**I: They don’t bargain.**

PA01: They don’t bargain…they misuse money.

PA10: Number 10, they are always spendthrifts, whenever she gets money she spends the whole of it.

**I: Is there any other… and can you identify them? Can you identify that this one saves and this one does not save?**

PA06: Within my hotspot I know those who save and those who don’t save… their behavior… I always spend time with them, I know them.

**I: How do you identify them?**

PA06: I know them… you just see their behavior… when you see someone who drinks so much you just know that this person does not save.

**I: Ok, that is number six, number two you want to say something?**

PA02: As number 02, for the person who does not save … those people who save always don’t show off. All the time they say they don’t have money but this other person who has money always show off, they drink alcohol at times they abuse you.

PA06: she will always come home making noise that she has money.

PA02: You know today I have money.

**I: Someone was also talking here; she was number…? Number one.**

PA01: I wanted to say what number one has said.

**I: Ok, we are faring on well, thank you so much. For those who save, the sex workers who save, you have already told me the reason why they save, can we repeat why they save. Number 02**

PA02: The reason why they don’t save is that some of them have needs.

**I: Number 03.**

PA03: Because when they save it can help them somewhere.

**I: It can help them somewhere… somewhere like where?**

PA03: You may have an emergency or the parent tells you that something has happened home, that money can help.

**I: Yes, number 09. Those who save why do they save?**

PA10: I support number 03.

**I: You support number 03. How do you support her?**

PA10: On saving.

**I: Why those people… for those who save, why do they save? Why do they save?**

PA10: (inaudible segment)

**I: Number two is speaking.**

PA02: Ok mostly… most of the sex workers have children and because she is not married so she saves so that she can buy a land and build.

**I: Ok. Any other person. Number 08 you told me it is for emergency.**

PA08: Emergency cases.

**I: Would you want to add another one.**

PA08: I will just support number 02. Obvious the sex workers are maybe you are a total orphan, it will force you to fend for yourself and rely on your own, you go places, you fend for yourself so that in future you can buy even a piece of land and build your house.

**I: Number 10.**

PA10: As number 10, some people save when they want to buy something, she has to save her to get whatever she wants.

**I: Number 01.**

PA01: As a sex worker number 10… number one

**I: You are number 01**

PA01: As number 01, I can say we save… sex workers save because they feel that some people feel that when they save they can do wonders. As in if you have a child, you cannot say that you save to take the child to ECD [Early Childhood Development], you can save to take your child to secondary. You know in ECD you can hustle and pay school fees for your child but with secondary school you cannot say that you will hustle for that. And then there are some sex workers who feel that they cannot get married like me, so they save so that I get a land to stay with my children.

**I: Yes, number 07.**

PA07: As number 07, I save money at the end on the month so that I get money for school fees and… paying fees for my child because I cannot get the whole amount once to pay school fees and again pay rent. I must save.

**I: So that you are able to pay those bills.**

PA07: Yes.

PA02: Can I add.

**I: Yes, number two**

PA07: At the hotspot maybe you have had a fight with another sex worker so maybe you are jailed and the police wants you to pay a certain amount.

**I: So that money will save you. Number 06 do you have something to say or you had shared?**

PA06: I had said it is for sending for my mother at home.

**I: Number 05.**

PA05: You cannot do sex work every day, you will not do it the whole of your life. You save so that one day you have your own business. You have your own business even if you can start selling second hand clothes.

**I: So that you get money to start business. And what makes it easy to save? Before that question, number four why do people save.**

PA04: It can help me to buy land then I send money to my parents at home.

**I: And what makes it easy for sex workers to save?**

PA08: We have not understood that question.

**I: Mmm**

PA08: May you repeat that question.

**I: I repeat the question. What makes it easy to save? … what is it that makes it easy to save. It is only that.**

PA08: As number 08 it becomes easy for me because I know how much I spend in a day. So once I have found the amount I spend in a day then I get an extra one so the extra one is what I will save.

PA02: Budgeting if you know how to budget you just plan that this amount goes to this and this amount goes to savings.

**I: Ok, that is number two, any other person, what makes it easy to save, number eight is saying she just takes that money she uses daily let us say it is 500/= if she gets 100/= extra, that goes to savings. And then number two is saying budgeting, you know very well that I need this and this and this, once I have sorted out that one it becomes easy to save. Any other person, number 10 do you have something to say?**

PA10: No.

**I: I thought you were rising up your hand.**

PA05: Number 05. You cannot tell whether you will get clients, so it just forces you to save some money. Yes, maybe you go there today and you don’t get a client so whatever you saved is want will help you.

**I: Number 06 have you understood that one?**

PA06: No it has passed me.

**I: So she is saying that you can never tell how the field will be, if today you find the field good, you will use some and save part of it because you may go there tomorrow and you don’t get clients. Yes, what makes easy to save, number 04.**

PA04: I support number 5.

**I: How do you support her?**

PA04: You use part of it because you are never sure you will get clients.

**I: Okay and for those who save, what kind of challenges do they meet?**

PA08: As number 08, maybe you were depending on the client to give you some extra money to save but you end up getting less that you need for your expenses so you can’t save for that day. That is the disadvantage.

PA05: As number 05, you don’t borrow every time once you save.

**I: Ok, challenges, that those who save are facing… which ones are they? She said that one of them is; maybe you expected a client to give you more but he ends up giving the exact amount you use on a daily use so you cannot save.** Yes, number 02.

R002: Maybe he has given you less than what you expected, so it will force you to go and withdraw from what you saved so that you cover what you wanted to buy.

**I: Yes, any other person, number 01, what are the challenges that those who save face?**

PA01: As number one, the challenges that are faced by those who save are like you had a plan with some amount of money… I mean you expected some money from someone and you had plans with the money but the person doesn’t give you, meaning that day you are not going to save. Because you cannot say that you are going to withdraw from your savings for you to save. That means you will not have saved that day.

**I: Now how can we address that one… the challenge you have just said? How can we address that?**

PA01: If someone lets you down.

**I: If someone lets you down, now how can we address that one so that you are able to save even if someone fails you?**

PA01: That is now beyond you, you are not able to save because you have not gotten what you expected.

**I: Ok number 08 you said that you may expect the client to give you some money to save but the client comes with less money and you had hopes to save, how can you help that… how can you address that?**

PA08: As number 08 I can reduce my expenses for that day. If I were buying sugar let me say I was buying for 30/= I will now go for the 20/=. So if I have the 10/= remaining I put together with what will remain then I save.

**I: Any other challenge, which challenges are faced by those who save? Are they over… number 06.**

PA06: For example, the client calls you in a hotspot, you thought you were going to meet him there but you don’t find him.

**I: So there is no money that day.**

PA06: There is no money.

**I: And you had planned to save 100/=.**

PA06: Yes. You thought you were going to get money from there…

**I: How are you going to address that? What you expected has backfired.**

PA06: But I will just get another one… I will get another one there and there.

**I: Will you look for the other one or?**

PA06: I will look for another one there.

**I: Okay, and to those who don’t save, why do you think they are not saving, like number 06 was telling us that there are those who have children they have left home, they have got the money but they will end up drinking with all this money. Why don’t they save, number 01?**

PA01: As number 01, for those who don’t sane you find someone saying that because they do not know about tomorrow, so they spend all they have. They say that ‘’who are they going to leave for the money and they don’t know whether they will die the next day’’. So she thinks of self but not of those who are going to remain behind.

PA07: As number 07. You know there are some families… there are some families where you just feel comfortable when they are with your child, they say that because in their family people are rich, she rather enjoys her money alone.

**I: Number 10.**

PA10: As number 10, maybe whatever she has gotten is little so there is no way she can reduce her expenses so that she can have some remaining and save. So it will force her to spend everything.

**I: So some people do not save because what they get is not enough, yes any other person?**

PA08: As number 08, there are some who are under pressure from those other sex workers we work with, if she sees the other person with something, maybe the other person is working, she feels that she should spend and buy what she saw from the other person so that they can be of the same level.

**I: So that makes her not to save.**

PA08: She is under pressure; she wants to do what the other people ate doing.

**I: Is there anyone who would want to add? … And is there an advantage of not saving? What is the advantage of not saving? Mmm but everything has advantages and disadvantages, even if it is minimal but there must be.**

PA07: Advantages of not saving, nobody will use your wealth.

**I: Nobody will use your wealth.**

PA07: Nobody will start fight that so and so left some money in your account that is mine, none.

**I: Any other person, Advantages of not saving... and what about the disadvantages of not saving? The disadvantages of not saving?**

PA08: As number 08, maybe you have not saved then you find yourself at a very hard situation, you may even be jailed for life because you don’t have any money to pay the lawyer. So you will just die at Kodiaga forever.

**I: Yes, any other person, number 04.**

PA04: Maybe you have funeral and you don’t have money it will force you to borrow.

**I: Any other person, the disadvantages of not saving.**

PA01: As number 01, if you have not saved and you have a child you need to pay school fees you will not take him to school because you will not be able to pay the school fees.

**I: I: Yes, any other person, number 02.**

PA02: Maybe you do not have a medical cover and you are involved in a serious accident, money is needed…

**I: You have?**

PA02: You are involved in an accident and you don’t have that medical cover and or don’t have that cash so… and there is nobody who can help you.

**I: And where do sex workers save mostly?**

PA02: Mpesa.

**I: Number 02 is saying mpesa, number 08?**

PA08: Mpesa.

**I: Mpesa.**

PA06: Number 06 KCB.

**I: KCB. That is in the bank, any other person, number 10 where do the sex workers save?**

PA10: Mpesa.

PA02: Can I add?

**I: Yes.**

PA02: There are some people who entrust someone so whenever she gets money she gives them to save for her.

**I: So do they save in the house or somewhere else?**

PA02: They can give someone who they trust maybe at the shop.

**I: Ok shopkeeper.**

PA02: Yes, shopkeeper.

**I: Why do they prefer a shopkeeper? Or someone else to keep for them?**

PA02: Maybe she does not have a place to save or maybe she does not have a phone or maybe she does not have that idea to open an account.

**I: Okay, what of those people who save in the mpesa, why do they prefer mpesa?**

PA02: Okay mpesa is…

**I: That is number two.**

PA02: Yes, number 02, yes mpesa will not deduct you any money but if you save with the KCB interest rates they deduct.

**I: Number 06 why do you prefer KCB?**

PA06: On that part, whenever I want to withdraw some money, I have to think twice. I have to look for the ATM, you know the ATM?

**I: Mmm.**

PA06: You cannot withdraw without the ATM.

**I: So you save it there because it is not easy to withdraw?**

PA06: Yes, it is not so easy.

**I: Mmm number 04,**

PA04: I prefer mpesa.

**I: You prefer mpesa.**

PA05: Some people save in Chama.

**I: Some people save in Chama; why do they prefer Chama?**

PA05: Because Chama ‘has interest.

**I: Chama has interest, (laughter) does mpesa have interest. Does KCB have interest?**

PA06: No it does not have interest unless you take loans you will get some on top of it.

**I: Okay but Chama has interest. And sex workers you were telling me… when we started you told me how you spend your money.**

P: (Participants talk in unison) Yes.

**I: And does it reach a point when they can spend more that they get, The Sex workers?**

PA05: That they spend more than what they have?

**I: Yes, you get let us say 2000/= but your expenditure is 3000/=, does it always happen?**

PA04: That is a backup,

**I: Backup, what does it mean?**

PA04: You borrow some money from somewhere.

**I: Ok when you use more that you have gotten then it means you have backup from somewhere. So number 06 you are saying that there is no way… not you… I am talking of sex workers, there is no way sex workers can use more that they have?**

PAO6: No way.

PA07: You can use more that you get, you can even borrow.

**I: Why does that happen?**

P07: They are not contented.

PA06: As number 06 they are not contented with what they have, even if you have more you are not contented, you just want to have the same as number 02, that means you are not contented at all.

**I: Yes, another person. Can sex workers spend more than they earn, I mean you get a little but the expenses are high. (Silence) on that part I can see everyone is quite, that means you are all disciplined or are you tired.**

P: No (in unison).

**I: You are not tired, but I can see we are not badly of, let us just respond so that we can finish. And to those who spends more than they get… I mean your expenditure but their income is down. How can they do so that the balance can balance. Number 05 has told us ‘backup. Number 02**

PA02: You can borrow.

**I: You can borrow.**

PA08: As number 08 I utilize the little money I have.

**I: You will just use the little that you have.**

PA08: Just the little one.

**I: Number nine.**

PA09: You can ‘fuliza’ from your phone if you have it

**I: Fuliza, that it you take loan.**

PA09: Yes.

PA03: As number 03, that is where side hustling will help.

**I: Side hustling, if you feel that the expenditure is high and the income is low, side hustle. Yes, number 04.**

PA04: Side hustle can help.

**I: Side hustle, number 05 (silence) so number nine has told me that if you feel you have some problem but you have low income, you take loan through ‘fuliza’, is there any other place sex workers borrow money that make them have depts. Said fuliza, is there any other place. Number 01**

PA01: Mshwari

**I: Mshwari.**

PA02: Some people withdraw from the Chama if they are in Chama.

**I: Number two Chama, number seven.**

PA07: There are some maasai people who lend us.

**I: Mmmh?**

PA07: Maasai.

**I: The maasais also lend out. Any other person?**

PA05: From friends.

**I: Number 05 is saying from a friend. Number 04.**

PA05: Mshwari.

**I: From mshwari. And what do we use this money for? This money we get from fuliza, mshwari, friends.**

PA04: The cost of living is so high that is why we borrow.

PA07: Cost of living is so high.

PA04: The cost of living you were saying is so high and there is no money so it will force you to fuliza.

**I: Then after the fuliza, in most cases fuliza helps in doing what?**

PA05: For emergencies.

**I: That is number five, so you can tell us what type of emergencies these are.**

PA05: Let me say I had 2000/=, you wanted to make your hair and then you happen to see a certain dress you feel you want to buy, it will force you to fuliza so to you buy that dress.

**I: She is saying that fuliza may help you to make your hair and it may also help you buy clothes.**

PA04: You may use it for hospital bills.

**I: You may use it to go to the hospital, Number 01**

PA01: If I fuliza or if I borrow from the Mshari, maybe I had 1000/= I will have to borrow 500/= so that I can pay school fees.

PA05: Number 05, maybe I had the 2000/= which I had budgeted with and I have someone’s debt, it will force me to fuliza so that I pay that debt because someone is asking me for money and the money I had at that time I had budgeted with, it will force me to fuliza so that I can pay dept.

**I: Number 06.**

PA06: Maybe my sanitary towels are over; menses are flowing so it will force you to fuliza so that I buy the sanitary towels.

**I: That is for normal expenses like sanitary towels. Now after you have borrowed from the fuliza what will you do so as to pay it back. This money to pay back the fuliza, how do you get it?**

PA05: Side hustle.

**I: Number 05 is saying side hustle, like which one?**

PA05: Washing for someone for money.

**I: Washing for someone, number 09.**

PA09: Side hustle, if I put in the mpesa it will all be taken.

**I: You are saying that someone can send in your mpesa then all of it goes, this person sending it on your mpesa, did he know that you have fuliza or just by mistake… by chance?**

PA09: By mistake, maybe it’s a wrong number,

**I: Ok it is a wrong number. (Laughter) so you let a wrong number to send on your mpesa then it will offset for your fuliza account. So you just sit down waiting if a wrong number can send you money from a wrong number, Number 01.**

PA01: You can just tell a client that you have a fuliza so she/he can offset for you.

**I: Okay. Even clients can pay. Number 10.**

PA10: I am supporting number 01.

**I: Number 01, how do you support her?**

PA10: I am just supporting her answer. The client can give you then you pay.

**I: Okay, and if the sex worker wants to increase her income, what do they do, how do you do…we said that for example you were getting 2000/= and you find that the expense is high, you want it to increase, like sex worker, what do you do so that it can move from 2000/= to may be 5000/=? Have you understood the question, number 01?**

PA01: As number 01, I will charge that client more, I will increase the increase the price if he was giving me …

**I: Mmm.**

PA01: I will charge them more

**I: Ok you will charge more.**

PA07: As number 07, you know when we are getting ready to have sex, we agree how many shots he wants, so the more the shots the higher the charges.

**I: So you increase the number of shots.**

PA07: And I also increase the charges.

**I: Any other person,**

PA05: Any other person, if you had like four clients, it will force you to increase the number to around six or seven.

**I: So you increase the number of partners. Any other person number 08.**

PA08: I support number 05. It will force me to increase partners.

**I: Number 03, if income is low and expenditures are high, you are needed to increase your income. What will you do as a sex worker?**

PA05: Number 05, let us say that you were going for sex work only during the day and resting at night. It will force me to work both day and night. I do it double.

**I: You do it double, number nine do you have something to say? Okay is there anyone who would like to add?**

PA09: I increase partners.

**I: You increase partners. okay. And what if… number 05 has told us that you increase partners and another person has said you work double, another person said that you increase shots, if it were one shot you increase them to be three then the amount of money also increases. The other one also said you increase partners; you have more of them. And now all these do they put us at risk of getting HIV? May you kindly share with me.**

PA08: As number 08, maybe you have increased the number of partners; maybe the one you have added has declined to use condoms and maybe he has HIV, so if you have sex without using condoms you may be infected with HIV.

**I: Okay.**

PA05: As number 05 maybe you feel you are so much in love with the ones you added so it will force you to have sex without condoms and maybe amongst them there are those you don’t love so much and there may also be those who help you so much, it will force you to do what they want you to do so by that you will be at risk of getting HIV if he had HIV.

**I: Any other person… we have said that the income is low and expenses are high so we need to increase our income, is it?... then we said that we now need to increase partners, we increase the shots and so some of us are saying that this increases our risk of HIV infection; so we have been told about the partners who do not want to use condoms, what else will put us at high risk of getting HIV… some people do not want to use condoms. Which one else… is there any, number 02.**

PA02: Just condoms, them not using condoms.

**I: And how much do sex workers have as dept. if you borrow from fuliza or mshwari how much do you borrow. Like how many thousands or how many hundred thousand?**

PA07: It depends.

**I: It depends.**

PA06: As number 06, I usually get 30,000/=

**I: 30000/= number 06 usually receives 30,000/=. What about others?**

PA05: Five thousand.

**I: Five thousand. Number 09**

PA09: I can get like 10,000/=

**I: And if you are doing sex work, does it reach a time when you think of leaving sex work? Someone is saying mmm… number 05… number 04.**

PA04: A time reaches when you want to leave.

**I: So when you want to leave how does it happen; may you please tell me.**

PA01: As number 01 I always sit down then just think on my own that I need to leave this kind of job. Though I am not ready to leave it but it will reach a time that I will leave it.

PA06: As number 06. Days are never the same, one day you will be lucky to meet a very good client who is meant to be yours, he may take you to= Mombasa= for a very big event you will just have to adhere.

PA05: Based on this job you can meet several untreated diseases so you sit down and say ‘I should leave this job’.

**I: So is this something that someone sits down to think about on her own or do you discuss it somewhere?**

PA05: You can either think of it on your own or you decide to have a discussion with other people.

**I: And within the hotspots where you go, do you ever have such discussions?**

PA02: Most of the time there is none because if you share with someone, they will go and start spreading.

PA05: One person will discourage you but one person will discourage you.

PA02: So you rather keep it to yourself.

**I: That is number 02 and number 05. And when you are alone thinking about leaving that job, what is it that makes you start thinking about such a thing, what is it that triggers it? Number 04**

PA04: As number 04, an infection.

**I: An infection like which one?**

PA04: You may be infected by either a HIV or STIs.

**I: Okay, that is fear of getting the disease.**

PA04: Yes.

**I: Number 08**

PA08: Me as number 08, sometimes disagreement with the client, maybe you agreed but he did not fulfill the agreement, if the same thing again happens the same day you feel discouraged.

PA05: Number 05, maybe you were saving some money so you just decide ‘let me quite this job and do my own business.

**I: Number one when you sit down thinking about leaving this job, what triggers this to happen?**

PA03: If the client annoys me, I may feel like I should just leave that job then I look for another job to do. At times there is no money in sex work, at times there is no money so you just think you rather leave this work and look for another job or maybe the clients are few, maybe clients are not many as usual … maybe clients are many only in December, so you just sit down and think that ‘let me go and look for something instead of wasting time there.

PA02: Can I add?

**I: Number 02**

PA02: Maybe something meaningful can come up, something meaningful has come up or maybe a meaningful job has come so the just the way people identify you as a sex worker so this one can destroy your reputation.

**I: And you have said that it is difficult to talk about leaving sex work at the hotspot where you work.**

PA06: It depends, I am number 06, it depends on the way you relate within your hotspot. There are those who can accept to share and there are those who cannot accept, you just know difficult it is to deal with a drunkard.

**I: It is difficult to talk to some of them...**

PA06: How can you have a discussion with someone who is drunk?

**I: What about those who you always talk with?**

PA06: We just talk nicely within the group; we sit together as a group of six or seven people.

**I: And whenever you start talking maybe concerning if in future she can quit this job what is it that triggers the conversation about this topic?**

PA06: I always go there to start the topic, I always go there then I just tell them, ‘I am tired of doing sex work will one day God give me a good man.’ He will do me great things and I will leave you in this hotspot. And it is true if I get a good man I will quit the job.

**I: Now after you have said that one what happens?**

PA06: They start talking…they start pouring out their opinions.

**I: Okay, is there any other person who has ever talked with other sex workers about quitting this job? Yes, number 05.**

PA05: Number 05, at times you hear a story that a lady has gone in to the room but was stabbed or something happened. So when you hear of such a thing you say that you rather quit this job that dying.

**I: Mmh you have already told me that someone may quit sex work because a fellow sex worker has been stabbed or they have got a husband who does what number 6?**

PA06: A husband who loves you and you leave the hotspot and go.

**I: Any other thing that can make sex workers leave sex work?**

PA03: As number 03, that is where savings helps because when you are going for that job, you go because you do not have money, maybe you are also have children who go to school. You do not have money and if you were saving… like the way I am working for someone in a salon, so you say… so if I want to start my own salon and get my own money. I will just have to quit this job so that I start mine.

**I: Ok who else can tell me why sex workers always want to quit sex work. Number 08 do you have something to say, number…**

PAO1: As number 01 you may wish to quit sex work because maybe you had a client who refused to pay you then you fought because of money. So you just feel that instead of fighting with him because of money, he had sex with you but refused to pay then he starts fighting you because he owes you and he does not want to pay, so you feel you rather quit.

**I: And to those who plan to quit, do they always quit?**

PA06: Yes, me as number 06, like I know of one who quit, she got a wealthy man, he relocated her from where we are and took her to =Nairobi=.

**I: There are those who quit… I mean those who wish to leave then they leave. And are there those who wish to quit but they don’t quit?**

PA01: Yes.

**I: Reason…Number one.**

PA01: There are those who plan to quit but they don’t… maybe there are those who leave but they come back let me say that way, yes there are those who plan to leave but they don’t, there are those who leave but they come back, maybe she went and started a business but feels that the business is not doing well so they feel that they can go back to sex work because there is nothing they are doing.

**I: Number 05.**

PA05: Number 05, there are those people who are just addicted to sex work such that if they don’t have sex with two or three people they just feel that their life is not fine. So she must find people to have sex all the time.

**I: So it may be difficult to quit. Any other, there are those who plan to quit but they don’t quit.**

PA05: Money is good because if she was getting money, they are just forced not to leave because if they can compare with the other side jobs she feels that she is getting less money from these other jobs but sex work gives her a good money. Maybe someone have sex with her then gives her 1500/= and she can get even 5000/= at night.

**I: Ok so she feels like leaving but the money she is getting makes it difficult to leave. Is there anyone who would like to add? And to those who leave sex work how old are they? Number 06, how old are those who leave sex work?**

PA06: Like the one I was telling you had 38 years.

**I: That same person,**

PA06: That same person had 38 years.

**I: And in most cases they leave when they are of what age?**

PA06: Forty something.

**I: And number seven when they leave with this forty something, is it because of the age or is there something else that makes them leave it?**

PA07: The age, the age does not allow her.

PA02: But it is always rare, chances are so rare for sex workers leave sex work.

**I: those who are already forty something?**

PA02: Yes, the chances are rare.

PA01: Like me number one, on the age, some people leave because their children also joined sex work so you may meet your child there. So you just feel that you rather leave.

**I: So it is not the age that makes them leave, some have grown up children who also join that work so when they meet she feels embarrassed.**

PA02: Some reasons yes.

**I: And to those who leave it forever, what kind of jobs do they do?**

PA08: As number 08, there are those who start their own businesses like selling clothes ‘second hand’ or readymade clothes. There are those who have shops, some people have built; they were using the savings to build plots so the money they collect from rentals helps them.

**I: Okay and when they do their business do they do it where other people also do or they isolate themselves?**

PA02: They isolate themselves.

**I: They do not do business together with the others?**

PA06: They do… within the market?

**I: Yes.**

PA02: No in the market they just do their business together with others.

**I: So they sell second hand clothes, any other thing they do?... and those who go to sell the second hand clothes do they go for good or they do the business and still do sex work?**

PA07: They just go for good.

**I: They leave for good.**

PA02: But there are some who are in that business and also sex work at the same time.

**I: They are still not able to leave for good.**

PA02: It is difficult, there are those who are married but they are still not able to leave sex work. When their husbands go to work they also leave foe sex work.

**I: Okay that is number 02, is there any other person who would want to add, we are almost done, I can see number 06 is already home. Sugar level are really reduced. We are almost done, so number 08 is telling us that when some sex workers leave sex work, they sell clothes which they do in the market. What about the others?**

PA01: As number one, among the sex workers there are those who were trained on particular fields, some of them trained as tailors some are hair dressing, so maybe when they go back to tailoring or some go to salons.

PA08: As number 08, I support number 01, because there are some sex workers who cleared form four and graduated and because of lack of jobs, that is why they joined sex work. So if an opportunity comes they may feel to leave sex work and go to the new opportunity which may favor them.

**I: And for those who leave it at forty something, according to how number 06 was telling her how do they manage life outside sex work?**

PA08: Me as number 08, I feel that there are those who educated their children while they were still in sex work and now the children are successful in life so the child goes to her and tells her ‘mum come and sit down here I will provide fir you everything’. So she managed through their children.

**I: And how do others manage?**

PA06: As number 06 there are those who have decided to go home, they have decided to move to their up counties forever to do farming there, she is tired of sex work so she decided to move home? (laughter)

**I: Ad those who are tired of sex work, can they do farming?**

PA06: Yes, they can farm, after you have gone home… village life will make you do serious farming.

**I: Ok, and do you know of those who left sex work then came back to it?**

PA07: They are so many.

**I: Ok and what makes to happen that way?**

PA06: Maybe she had her partner the other side and that is in the town… she came to town but when she went back home the husband totally refuse, she will have to go back to her hotspot.

**I: That is number 06, any other person, what can make someone to leave sex work then go back?**

PA07: I support her…I support number 06 maybe she left and got another partner but that partner does not satisfy her, so she will have to go back to sex work.

**I: Another person, number 05.**

PA05: Maybe you got married but you found marriage life difficult, it will force you to go back to the kind of life you were in.

**I: The marriage life…**

PA05: Broke up.

**I: Broke up. Number 04, why should someone leave sex work and come back to sex work?**

PA04: Maybe she is broke.

**I: So there is no money, so she is coming back to look for money. Number five, why should one leave sex work then come back to it again? Do you know of anybody who left sex work then came back to it again? Number nine… why did that one happens?**

PA09: Maybe there are those who can leave … they leave then come back because they leave they will have to use all the money that they had saved. It will force her to go back to sex work.

**I: And there are some requirements for a sex worker. There is always a list, number six there is always a list that sex workers have where they say that I would want to accomplish this and this and that then I leave sex work. So what does the list contain? Someone may be having an idea that I am doing this kind of job but I will one day leave it but before I quit it, I would want to do this and this then leave sex work. Number 10.**

PA10: Educating the child.

**I: Mmm, educating the children, number 02?**

PA02: Maybe she has bought a land and she is now building so she is saving as she builds then once she will be through with building she quits sex work.

**I: Mmm any other person.**

PA05: Maybe if number 01 had some loans, so once she has cleared the loan she leaves sex work.

**I: Number 04.**

PA04: I support number 02.

**I: What did number 02 say?**

PA04: Maybe she wants to buy land.

**I: So once she has gotten the money and bought the land she leaves.**

PA04: Yes.

**I: Number 05**

PA05: Let me think of one, the answers are almost over.

**I: Number 06**

PA06: I am still thinking.

**I: Mmm.**

PA02: May you repeat that question please.

**I: There are always those things that the sex workers wish to accomplish before they leave sex work, somebody has said that from that list I would like to educate my children first they complete their education is when I can leave. The other one is saying ‘I took some loan so I would like to pay the loan, once I am done…**

PA02: Okay I have understood. May be you are doing sex work but you do not have a child, so you would want to get a child is when you go back. You first give birth; you take a rest then you go back. Then you can go back.

**I: Number seven, what do you have in your list. ‘I would like to do this and this and this then I leave sex work.**

PA07: As number seven I support number two.

**I: Mmm, what did number 02 say?**

PA07: After buying land and building, I leave it.

**I: Is there anybody who would like to add? Or we can continue. So if you want to educate children, buy land, construct a house, give birth. What do you do? Is there anything you first need to do is when you leave sex work?**

PA08: Yes.

**I: Like what?**

PA08: As number 08, that is now where we encourage savings because you can never do all those things without planning. You have to plan, so you will be saving, when you save and the money is enough to buy the land, you go and buy the land you get your title deed then you keep it. You just continue saving then as you save. You will be doing like that you save until you accomplish your ambition.

**I: So when we want to accomplish all these things, we always doing something so that they are accomplished or you can just have your list ‘I want to construct a house or buy a plot but there is nothing you have started doing?’**

PA02: That one you cannot make it.

**I: Mmm.**

PA02: That one you can’t make it. (cross talk) you have to plan.

**I: Now all of you have talked. You must have plan. … do you know those who have left? Number 06 had told us that she knows of someone who left sex work at 38 years, is it?**

PA: (In unison), yes

**I: And do you know of any.**

PA04: I know one who left because she got married.

**I: She left and got married.**

PA04: Yes.

**I: Ok and what makes it easy to leave sex work?**

PA02: What makes it easy to leave sex work? For example, if you…

**I: That is number two.**

PA02: For example, like number 06, she got someone to marry he, that is how it became easy for her to leave.

**I: Ok, any other person?**

PA08: As number 08, those who are around you they tell you to quit that job, they tell you that that place is not good so even to yourself deep in your heart when you sit down, you just tell yourself that this kind of job is not good you get discouraged so you just decide to leave it.

PA05: Number 05, maybe you went to it just because it is a place to get money but it is not good for your health. You just do it but your mind is not in it.

**I: So does that makes it easy to leave?**

PA05: Yes.

**I: Is there anybody who would like to add?**

PA07: As number 07, the advice you get from others, maybe you get an advice from the person who was once a sex worker she advices you by telling you that,’that place is not good, you can try and find another thing.’ So you find it easy to leave.

**I: And these people who always want to leave what are the challenges they face or what are some of the things that makes it difficult to leave?**

PA05: Number 05, based on how you get money… the payment. Maybe you always get more than 10,000/= so whenever you think of leaving it, you find it so difficult.

**I: ok. You get good money so when you think of leaving it, it becomes difficult. Mmm any other person?**

PA08: Number 08, you find that at times you want to quit that job but when you leave it you do not see your future so you find it difficult to leave so it forces you to continue with it.

**I: Ok we are almost finishing, I can see the sugar level has really reduced but we are almost through. Is there any other person who would want to add on what makes it difficult to leave or the challenges that those who wish to leave always face?**

PA01: Yes.

PA05: As number 05.

**I: Number 05 then number 01. Number 05?**

PA05: Like number 06 said that you have client responsible for food, you have someone who is responsible for rent, so when you just think to yourself that you want to quit it becomes difficult.

**I: Ok, and there before we talked of Jitegemee the way I told you about it, can you remember?**

PA: (In unison) yes.

**I: So I told I told you that it,s purpose is to make the sex workers have the ability to decline unprotected sex, take a break when they want and take leave if you wish. You say no to unsafe sex take leave if you wish and I also told you that it is a way of saving and the money you save is your own so that you be ready for life after sex work is it. So do you think people would like such an idea within Kenya?**

PA: (In unison) Yes.

**I: How?**

PA08: Me as number eight I know the sex workers will love it because you know like after sex work there is a life…a future they need to protect. So based on those saving they will use them in future it will help them so much.

**I: Ok while talking we said we should… number 01.**

PA01: I have not carried up my hands.

**I: We were saying that there are some sex workers who save, there are sex workers who don’t save and that you can identify them with their behaviors is it? So what type of sex workers will like Jitegemee?**

PA08: As number 08 I feel it is those who are saving because they know the benefit of saving.

**I: Number 01.**

PA01: As number 01 I feel those who do not save will save.

**I: They will like it.**

PA01: They will like it…to save.

**I: Is there anybody who would like to add, and are there those who will not like Jitegemee?**

PA06: I am number 06, the stubborn drunkards.

**I: Mmm, the drunkards.**

PA06: Yes, they hear with one side of the ear and the same information gets out with this other ear.

**I: Why will they not like it?**

PA06: If you tell them something they may think that you want to eat their money, ‘she will ask you where do you want to take her money?’ but it is hers and her emergencies.

**I: Ok, who else will not like Jitegemee? Number 04… and for the sex workers you know, for example number 06 has drunkard friends or the drunkard sex workers, you also know of some is it?**

PA: (In unison) Yes.

**I: If there are like 10 sex workers, then you tell them about Jitegemee, out of the 10 how many will accept? Number 08**

PA08: Out of 10, I am sure with five.

**I: 10 will accept, number 10 if you share it out with 10, how many of them will accept to join Jitegemee?**

PA10: I am sure of seven.

**I: Seven, number one,**

PA01: Three.

**I: Number seven?**

PA07: Five.

**I: Number 06. If you have 10 sex workers then you share with them about Jitegemee, how many of them will accept to join?**

PA06: Maybe eight,

**I: Eight… number 05**

PA05: None

**I: You feel that they will all decline. That is a personal information.**

PA02: Can I add?

**I: That is number 02.**

PA02: You know not all of them will accept it, someone may feel that maybe you want to take her money. Someone may feel that instead of saving that money if she was drinking, she should continue talking alcohol so it is difficult yes accepting will be very difficult.

**I: Number 04:**

PA04: Only two will accept.

**I: If you tell 10 people only two will accept.**

PA04: Yes.

**I: Yes, number 03.**

PA03: Five.

**I: five, number 09.**

PA09: One.

**I: Only one, who has not responded? We have all responded. Ok and those other ones why will they not accept Jitegemee? Yes, number 10?**

PA10: They will think that you want to take their money.

**I: They would think you want to take their money. Number one for those who will not accept why will they not accept?**

PA01: I support number 10 because they will not accept thinking that you want to take their money and they will also want to know where that money is going.

**I: Number 07.**

PA07: They will want to know who is keeping the money.

**I: Sorry that was number 02, Number 07 why will they not want to join Jitegemee?**

PA07: As number 07 they will think you want to take their money.

**I: And what can we do to make the sex workers embrace… to accept Jitegemee?**

PA02: May you repeat

**I: What can we do so that majority of the sex workers can accept JItegemee. Yes, number 01.**

PA01: As number 01, as you said in the beginning that Jitegemee will open an account, the sex workers should be called and be informed., after they have been informed how their money will be kept, I think so many of them will accept it.

**I: Ok number 10.**

PA10: Silence.

**I: That is what you wanted to say. Number 6**

PA06: Trusting one another.

**I: Now how can we bring this trust because like today we have met with you here. What do we do to bring this trust?**

PA06: But we know each other within our hotspot.

PA02: You are welcome

PA06: We know each other.

**I: Ok number 05 do you have a point?**

PA05: For them to know that this money is saved for their future use in case of anything

**I: How will they know?**

PA05: They will be told issues about saving.

**I: It is explained to them; ok do you have something? Number 08**

PA08: I support number 01, we must… like me I can call them within my hotspot so we educate each other, so we have the communication and also trust one another.

**I: Ok, number 03 do you have any point?**

PA03: No.

**I: Ok, and how should we make Jitegemee to be acceptable to the sex workers, how should it work? Like for Chama you can say; we will meet, we will do this and that, the rules are usually set to make Chama do well so how do you think we can make Jitegemee, what you feel like the sex workers. Number 06 how can we make it? Yes, number 01.**

PA01: It can be made like a Sacco, so it is like a Chama, the money you contribute is taken to the Sacco.

**I: That is number one’s opinion, any other person, how would you want it to be made? Number 03**PA03: We should have some meetings within our hotspots.

**I: Mmm?**

PA03: I am saying the meeting

**I: Ok the meetings at the hotspots?**

PA03: Yes.

**I: And then?**

PA03: Now everyone will give out their ideas.

**I: Ok people to have a meeting at the hotspots then they give out ideas. Mmh any other person? Number one said that it should be made like a sacco. Number 06 do you have an opinion?**

PA06: No.

**I: Number 07.**

PA06: **Nothing**

**I: Is there anyone who would like to add something. When Jitegemee begins to work, what would you want it to do? We already know it is about savings, is it?**

PA: (In unison) Yes.

**I: Now this savings, you as sex workers what would you want this …to include? Remember it is not a Chama which gives you headache when you don’t have money. You can save money when you want, the amount you want, you can withdraw at any time whether during the day or at night whenever you make a call you can get it anytime.**

PA06: As number 06, that money can help you do something if you have a problem. Yes, when you have problem, you can withdraw and sort out the problem.

**I: Ok, is there anyone who would like to add, we have just a few questions then we are done. …the way I told you about Jitegemee, what is it that the sex workers will love the most in Jitegemee?**

PA05: Savings, number 05.

**I: Number 05 is saying savings, another other person?**

PA08: Number 08 I support savings.

**I: Savings. Why would they like savings?**

PA08: Because they will be confident that if they save in future they will use it to quit sex work.

**I: Any other person**

PA05: Loaning.

**I: Loaning…we did not talk about loaning; we just said that you can withdraw your money. Like even if you say that you had saved the 10,000/= and you need it at that time, you will be given. There is nothing like …it does not have interest; it does not have loan. Let me ask this… it seems you have a point; I can see you are thinking so deeply number** **05.**

PA05: I am waiting for the question you want to ask.

**I: The way I had said or explained to you what Jitegemee is going to do, is there anywhere it is going to affect your rights? Number 10**

PA10: No.

**I: There is none, is there anyone who has realized where it can…**

**NT: Affect someone’s rights?...**

**I: There is none. And if it is something that has begun working like you said we should call people at the hotspot or we should make it be as a Sacco… what are the challenges you think we can face after it has been started? Number 06, the challenges we may face once it has started working, which ones are they?**

PA06: I have not understood it well.

**NT: The challenges (she translated in Kiswahili)**

PA06: Ok the challenges.

PA08: Number 08, as you start, as you are going to start talking to people, maybe majority will not accept it.

**I: Mmm, any other?**

PA05: Most people are not going to save, number 05.

**I: Most people are not going to save. The challenges we will face when we begin?**

PA08: Number 08, language barrier, maybe you are going talk in English and yet we have a =Kisii= lady who has never been exposed, she did not even attend school of which the only language she knows is aware of is Kiswahili so if you go there in English it may affect.

**I: Language barrier, though we always try, we do mix English Dholuo and Kiswahili. The challenges, the challenges, what challenges are we going to face… (Silence) and if you were to save some money per week, how much do you think you can save per week? Number 01, how much do you think you can save in a week?**

PA01: In a week?

**I: Yes, in a week.**

PA01: 2,000/=.

**I: Without interfering with your daily budget?**

PA01: 2,000/=.

**I: 2,000/= in a month, number 10.**

PA10: 500/=.

**I: 500/=, number 08.**

PA08:1500/=.

**I: 1500/=, number 09.**

PA09: 1000/=.

**I: 1000/=, number 03.**

PA03: 500/=.

**I: 500/=, number 02.**

PA02: 800/=.

**I: 800/=, number 04.**

PA04: 2000/=.

**I: 2000/=, number 05.**

PA05: 12,000/=.

**I: 500/=.**

PA05: 12,000/=.

**I: 12,000/=, number 06.**

PA06:14,000/=.

**I: 14,000/=, number 07.**

PA07: 1000/=.

**I: 1000/=, this is the amount you can save but you cannot sleep angry.**

PA: (In unison) yes.

**I: And how would you want this money to be saved? Where should it be saved?**

PA06: But it is saved on phone or where else can it be saved?

**I: Where should Jitegemee save your money?**

PA: (Cross talk) account.

**I: Phone account or bank account**

PA: (Cross talk) Bank.

**NT: Number 06 prefers bank.**

**I: Number 06 bank, number 04 mpesa, number 07…**

PA05: But you have said you will open your account.

**I: Account.**

PA05: Then that means it is bank.

**I: No an account can be in the Bank, Mpesa or it can be with the Sacco, it can be with the bank. So which one would you prefer?**

PA05: Bank.

**I: Number 07.**

PA07: Bank.

**I: Number 01**

PA01: Sacco.

**I: Sacco, number 10.**

PA10: Mpesa.

**I: Mpesa, number 08.**

PA08: Bank.

**I: Bank, number 09.**

PA09: Mpesa.

**I: mpesa, number 03.**

PA03: Bank.

**I: Number 02.**

PA02: Bank.

**I: Bank, that is number 02. Mmm those are the questions I had for you today. Is there any question**

PA07: No

**I: There is no question, thank you so much for your patience, we have taken a lot of time but we appreciate.**

**END**
