# Supplementary material for: Eat a little and save a little: A qualitative exploration of acceptability of a potential savings intervention to reduce HIV risk among female sex workers in Western Kenya
Source: PLoS One. 2024 Dec 19;19(12):e0310540. doi: 10.1371/journal.pone.0310540 (PMC11658496; doi:10.1371/journal.pone.0310540)
Supplement: S1 File — (ZIP) [file pone.0310540.s001.zip › Jitegemee Transcripts and Dissemination Notes for Journal/FGD C.doc]

**FGD ID: FGD C**

**INTERVIEWER: JUDITH**

**NOTE TAKER: OLIVIA**

**VENUE OF THE INTERVIEW: TYP**

**INTERVIEW DATE: 25/APR/2022**

**TRANSCRIBER NAME: OLIVIA**

**INTERVIEW LANGUAGE: DHOLUO**

**TIME TAKEN: 1 HR 48MINS**

**CATEGORY: ABOVE 30 YEARS, URBAN**

**I: This is FGD C. The interviewer is [name omitted] and the note taker is [name omitted]. As we begin I would like to ask you this first question, as I have briefly described to you about Jitegemee, what comes to your mind now? What we just read about Jitegemee, what comes to your mind? Yes, number one.**

PCO1: Saving, that is what comes to my mind.

**I: In which way?**

PCO1: Saving money for future use.

**I: Somebody else, number 3.**

PCO3: As number 3, what I have heard is that the way we do our work, if there is no more work or you have decided to leave, how much have you saved that at least can help you. Or maybe if a client does not pay you, maybe because you did not do what he wanted, so you know in that case you have not worked. So, it is how you will survive on the little you had saved.

**I: Okay, number 4.**

PCO4: What I have heard from Jitegemee program, you should be able to rely on yourself and not on sex work only. Like me number 4, I have children, I can’t rely on sex work only the way I have heard. I should save through the money I earn from sex work so that… because it will reach a time I will not do it all of my life, I will not have strength. So, I should save, begin some business so that these children… it will reach a time when the money I earn from sex work will not help them. I should begin some business so that before the five years are over, I begin saving something small. For me not to consume this money, I collect an amount even up to 3000/=, then send to fix deposit account. You know it’s not possible to get money from fixed deposit account even when you sleep hungry. I can use this money when I don’t have a fixed deposit account. so when it is fixed deposit I will have to borrow money to use when in need. I can save even for one year then withdraw it but now… I will not withdraw because I want to leave sex work because of my children. So I will rely on myself and save then by the time it gets to that five years, I’m able to start something.

**I: Okay, any other opinion?**

PCO6: I want to say, saving is sometimes difficult. The reason it is difficult is, maybe you have children who want to eat, bath and dress. They are also going to school and you are paying tuition fees. so, sometimes during menses, it can be difficult, you can take 3 days without going to work and when you don’t work, you can’t pluck money from the tree. You are forced to use what you had saved because you cannot do sex work during menses. You see, sometimes the child is sick, you are forced to use what you have. So, you know with saving it is good if you have someone who supports you but when you are only depending on money from sex work or if you have some business to supplement it, then it is possible but if it is only from this one source then it is difficult to save.

**I: Mmh, any other opinion number seven?**

PCO7: As number seven, I feel Jitegemee research, I feel that there is something good it is going to bring us who are doing sex work. The way I have understood it, at least you should have some business. After making money worth five thousand, you can even begin some cloth selling business. So you can sell clothes during the day and do sex work at night.

**I: That is number seven, any other opinion about Jitegemee?**

PCO5: As number 5, I support her.

**I: Who are you supporting?**

R5: Number 4, the way she has said she has children, I also have a child, and you can’t do that work all of your life. So I can decide if it is this week, I will go and if I earn 5000 or 2000, I take 1000 and save on a fixed account, then I use the balance in the house. Or another thing that comes to mind is business the way she said. I can decide in the mornings to get somewhere along the road, cook mandazis and sell. That is in the morning, then in the evening you can go for this other (refers to sex work).

**I: Yes, number 2.**

PCO2: As number 2, what I have read about Jitegemee, it is good. So, the more we do this work, we should save something because we experience so many challenges as we do sex work. You can even find a murderer as a client who can even kill you at night, you may get clients who want to do things to you that are not good like refusing to use condoms and they know they are infected, something like that. Then for many of us the more we get older we go through a lot. So I really long to be able to rely on myself, to save something good and reach somewhere we can change to do another type of work and not do this work forever.

**I: Any other opinion about Jitegemee? Yes, number 8.**

PCO8: As number 8, Jitegemee has a good idea but the request I have is for them to open for us something like an umbrella so that when I save, I don’t save in the house because when I save in the house, it is easy for me to use the money. So they should provide an umbrella so that as we save, and they tell us what we are supposed to do. So you know if you save it will be difficult for you to withdraw the money, you will just have to persevere. You can also find some small business to do during the day, at least this will also be helpful. So if you now go for sex work in the evening, you can use the income for paying school fees for your children or do any other thing. That is the request I have, if they can open for us an umbrella we can join as we save.

PCO6: Selling during the day and night has to have some challenges, because you cannot work during the night and not sleep during the day. We must also consider this. or is there someone who can work during the day and night without sleeping? This is something we have to consider.

PC: It depends on…

PCO6: …even if you set apart 8 hours for sleeping, you have to sleep.

PC: It depends on the business you are doing.

PCO6: Maybe you come from work in the morning, when you arrive, let’s say you arrive at 5am, you want to arrive and cook and maybe you have other clients who will call you around the time you reach home and they want you, what time are you going to sleep? So you know we have to balance.

**I: Okay, we were talking about opinions about Jitegemee. The rest we will talk about later.**

PCO7: As number 7, number 6’s reaction is not a good one. The way she is saying someone can’t work during the night and again do business during the day. There is how you set your time. You know you sleep at a particular time and go to work at a particular time. That is the reason I talked about selling clothes. You can buy clothes on Saturday being that it is a market day in =Kibuye=, you buy clothes on Saturday and distribute to customers, after distributing you come back to the house and sleep then collect money later in the day.

**I: Any other opinion about Jitegemee?**

PC: As I talk, I support number 8 because sometimes you may make little money like 500/= and I can’t deposit it on my phone, so I’m requesting if you can provide somewhere we can save, like if get even 50/= I can deposit, if it is 100/= I can deposit there so that when I lack there is a place where I can get money from.

**I: Okay, so if you get money… mostly what do female sex workers like buying with the money they earn? The money you get daily from sex work, what do most female sex workers like to buy with the money they get from sex work? Yes, number tw**o.

PC02: The way I normally use it; I rely on myself. When I come back, I can buy breakfast and items used for cleaning from that money. I think that is all.

**I: So, on a normal day, every day when you don’t have visitors, just a usual day, how much can you spend on a daily basis?**

PC02: Daily when I have visitors or...?

**I: No, just a usual day.**

PC02: Ooh, a normal day?

**I: Yes.**

PCO2: On a normal day from morning to evening I can spend about 500/=.

**I: That is on which expenses?**

PCO2: That is breakfast, lunch and supper.

**I: Is there anything you buy weekly apart from food? What else do you buy with the money you earn form sex work? Yes**

PC05: As number 5, expenditure exclusive of expenditure on visitors, supper, breakfast, in a week I should be smart. So I will buy a dress or a trouser because I should be smart when I go there. Yeah, something like that or I don’t have a phone, I want a smart phone so that I can... you understand.

**I: Do you buy a smart phone, weekly?**

PCO5: Not weekly, once. Maybe I don’t have it that week but I have the money I have earned.

**I: Is it something you change every time, or for how long do you use it before you buy another one?**

PCO5: I can use it for 3 years or 2 years before I buy another one.

All: Phones really get lost, it will depend. Phones get lost there all the time. You just know if you lose a phone you have to replace.

PCO5: You know this work that we do, with phone matters, you can go there and get drunk then leave your phone there and when you go another time maybe you have a phone then forget to take your phone or someone steals it. And you must have that phone because of communication.

**I: How much is that phone averagely?**

PCO5: It can't be more than 5000/= because you know there are also daily expenses in the house.

PCO3: It can be more than that because as a sex worker you must have a simple phone and a smart phone. You see the way I have carried mine? There is one I use away from the house and there is one I only use in the house. So, you know if I lose this one (points at the simple phone), it is easier to replace than the smart phone.

**I: So it is easy to move around with the simple phone?**

All: Yes

**I: Okay, everyone agrees with that?**

All: Yes

**I: Other expenses? What do girls buy with the money they earn? You have said clothes, food, phone mhm. Yes, number one.**

PCO1: Pay rent.

**I: Pay rent, mhm.**

PCO6: Buy cosmetics, make your hair.

**I: That is number 6. Do you buy that every day or...?**

PCO6: No, you know if you buy cosmetics you can use it for a month. It depends on the size you are using.

**I: Is that monthly expenses?**

PCO6: It depends with how you are. if you get money you can buy something that will last for one month. So it depends on the amount of money you get on a day.

**I: Yes, number 4.**

PCO4: As a parent and a sex worker, when I get money in this CBC era, I will ensure I educate my child.

**I: You will take care of school requirements?**

PCO4: I will take care of school requirements first.

**I: After taking care of school requirements?**

PCO4: After taking care of school requirements, I will make myself look beautiful, because this work is all about being beautiful. When you are not beautiful, clients will not look at you among others. They will just pass. You don’t get clients.

**I: Yes, number 7.**

PCO7: As number 7, I will first pay money for Chama, after paying Chama, the house I’m renting is 2000/=. so I normally divide it by 30 so I prefer to keep some money every time I come from work so that at the end of the month I just take the money from where I kept it and pay rent. I must also eat well. I cannot eat vegetables only because the work I do is hard work. It is beef, fish or chicken.

**I: Any other thing apart from eating well?**

PCO7: I must also dress smartly.

**I: Any other person? Number 10, what will you buy with your money?**

PC10: When I get money, I use part of it to save and another part I use to maintain myself. If you go for sex work without making yourself look good, nobody will choose you. You must go there when you are smart and attractive so that when you are meeting a client and you are bargaining, and you say you want a certain amount of money, you are able to get that money because of how you look.

**I: Roughly how much money do we use on cosmetics monthly or weekly?**

PCO7: Monthly?

**I: Yes**

PCO1: In a month if I calculate it roughly, as number one, I use about 20,000/= or 21,000/=.

**I: That is average. Anybody else? Number 9.**

PCO9: It depends on whether you got clients. It depends on the number of clients you got.

**I: How much can you use on cosmetics on a monthly basis?**

PCO9: It can be about 10,000/=.

**I: Any other opinion on how FSWs can spend the money they earn?**

P: Just making ourselves look beautiful.

**I: Mostly on beauty. Apart from making yourself beautiful, food, school fees and rent, and phone, is there something else they can buy?**

P: They can even buy alcohol; they are making money.

**I: You buy alcohol?**

P: You can buy alcohol.

**I: For entertaining friends or the top shelf?**

P: Just yours.

**I: Where do most female sex workers get their money from? Apart from sex work, where do they get some of their money from?**

PCO7: As number 7, sometime work is very difficult for me. I can even go for two days without getting clients. I normally call my brother to send me something small that I can help myself in the house.

**I: Someone else? Where do others get money from? Yes, number 2.**

PCO2: I can get money from Chama.

**I: Any other place we get money from? As we think of other places, number 7 when you borrow money from your brother, how much do you normally borrow from him?**

PCO7: You know when you borrow money from someone, you can’t force them to send you a particular amount. You will just receive whatever you are sent.

**I: So you don’t specify to them the standard amount to send you?**

PCO7: No, I only receive and appreciate whatever he has sent me.

**I: Number 2, how much can you borrow from the Chama?**

PCO2: There is chama I can borrow from and then there is the one you wait to receive a cumulative amount. So one can wait for their turn to receive the cumulative amount.

**I: Any other place we get money from?**

PCO6: There are also people who walk around who are referred to as Maasai, you can borrow from them. When you borrow from them where you save… if they lend to you let’s say even 1000/= they will take from you 120/= per day. This can also help you when you are in need. You can borrow from them and meet your needs.

**I: That is number 6's opinion. Yes, number 10.**

PCO10: As a sex worker, when I don’t have money, I have my regular clients. When they come to town I will definitely meet them, so sometimes I can meet them when the business is not good, and they will give me some little money and when it is good they will give me good money. So sometimes when I have some difficulty, they can sort me out.

**I: When they sort you out, how much is it normally?**

PC10: The least they can give me is 3000/=.

**I: Okay, why do they spend on what they do? Like we found out that some people will buy a phone. Why buy a phone? Or what is the reason they spend on a phone?**

PCO7: Who is spending on a phone?

**I: Sex worker. We are talking about sex workers.**

PCO3: As number 3, most of my clients I meet with over the phone, they call me and I meet them. So when I don’t have a phone, there is no way they will find me. So I have to buy a phone, I must have a phone because I mostly deal with a phone.

**I: Apart from buying a phone, meeting with clients, we also mentioned buying clothes to look smart. Any other reason they spend on what they buy? Any other reason? (long pause) Have you exhausted them or you are still thinking? Okay and do you think female sex workers normally save money, can they save money?**

P: They save.

**I: They save?**

P: Yes.

**I: Why do they save?**

PCO3: You know where you stay, you have to pay rent, you have to eat. If you have a family, they also have to eat. Like in my case my first born is going to school, I have to pay school fee. So I just have to.

**I: That is number 3, any other person? The reason why they save money.**

PCO8: As number 8, I save because sometimes I may be sick and find it possible to go to the hospital, pay for my child school fees, pay rent. I must also secure my future. I can’t just watch things happen, I must have something I own. That is the reason I can save.

**I: How much can you save?**

PCO8: In a month?

**I: Yes, how you save, even monthly.**

PCO8: It depends because sometimes I can go for sex work, and it turns out to be a good day or there were no clients. So it depends on what you make. If you get 2000/=, you can save 500/= and sort yourself out with 1500/=.

**I: Is this saving monthly, weekly or daily?**

PCO8: If I go for work every day and get something today, I will have to save something small from what I earned. So it depends on what I earn in a day.

**I: Number 3, whatever you save, how frequently do you do it or how much is it? Is it daily, weekly or monthly?**

PCO3: Mostly I don’t go to work every day. You know you have to wait for a phone call. It depends with… If you have regular clients, they always pay less. They complain how they don’t have money and you also want to maintain them because when they come they give you a little and you save. So, it depends, if you get new clients they pay well depending on how long you have maintained them. So it depends with the little you get.

**I: How much can you save per week?**

PCO3: In a week I can save at least 7000/=.

**I: 7000/= in a week?**

PCO3: Yes.

**I: Any other person who saves?**

PCO1: As number one, when I save I again use. I know on Friday, Saturday and Sunday I’m sure I will get something. I can get 5000/= on Friday and this is all used in the house. Saturday I can get 3000/= or 4000/= and this I spend on my children and food for the week. On Sunday the money I get is for drinking and the remaining amount is what I save, but after two days I will take all the money I saved and use. So I don’t save.

**I: How much do you save before you use it?**

PCO1: I can even save 7000/=, even 8000/= or 10000/= then I take all of it and use it for drinking alcohol and having fun with all of it then I begin again.

**I: Any other person who saves? Number 9.**

PCO9: As number 9, saving will depend on how much I get, it will depend on how much I get per day. May be I get 500/= per day, I will save even 100/= and use 400/=.

**I: Do people who save have certain characteristics? Do FSWs who save have some characteristics or is there a way they can be identified? Number 1.**

PCO1: Yes, those who save they rarely share with somebody what they have. They are always just ready to receive from others. They don’t even buy alcohol; they just want to share alcohol that someone has bought. They don’t want to spend; they can’t even buy maize (refers to roasted maize along the road).

**I: That is number one’s opinion, somebody else, what characteristics do people who save have?**

PCO3: You also have to show off, as number three, you have to show off. If the client has little money and wants to play around with you, it is not a must because you know you have something in your hands.

**I: So they have a characteristic of showing off?**

PCO3: Yes.

**I: Any other opinion, for those who save? Number 4 tell us.**

PCO4: Someone who saves is different from one that does not save.

**I: In what way?**

PCO4: You will not force your demands on them, you will do what they want because they know that when they don’t get anything, they will still be able to meet their needs. You have to boast because you have something, you are saving so nobody will force you to do what you don’t want.

**I: Number 6, how do people who save look like?**

PCO6: You know what, saving money is good but it depends on how your family is. You may have many people who are depending on you and don’t have an opportunity to save. You may have 2 or 3 children and be able to manage. So there is some difference. Someone may have a big family like 5 children, you want to feed them and take them to school, you also want to save and when you save someone becomes sick and you are forced to use your saving. So it depends on how somebody is and how she gets clients.

**I: We have said those who are saving some show off, they don’t like spending. Any other characteristic they have that is different from those who don’t save. Which characteristics do those who don’t save have?**

PCO3: As number three, those who don’t save have a characteristic that we sometimes see where we do sex work. We can sit down and agree on how we should charge one shot, and this is the amount charged for every shot. So that if this person goes to the next he will still hear the same amount. So for those who don’t save, it is like if we agree to charge 500/=, they will lower it to 200/= because they don’t have anything. They accept any amount even if it is less money. They have that characteristic those who don’t save.

**I: They don’t bargain?**

PCO3: They don’t bargain. They spoil the market price.

**I: Any other characteristic apart from spoiling the market?**

PCO1: A characteristic that they have, when they arrive you will know they don’t save. When they are around you will know they have arrived. They always want to buy for everybody alcohol. Then they are left without anything.

**I: Without what?**

PCO1: Without any money.

**I: Ooh, she buys a lot of alcohol. Any other characteristic they have? Number 2.**

PCO2: As number two, we have seen many, you will hear one saying “today I have not got any client, did you get any? I have not got any client since I came.” These are the people who don’t save talk like.

**I: We have seen there are those who save. What makes it easy for them to save? Because we got different reasons. There are those who save to take children to school, pay rent. The reasons are different. What makes it easy for those who are saving to be able to save? Yes, number 6.**

PCO6: The reason they are able to save depends on the income they get and they don’t have a lot of responsibility. So she is able to get the chance because maybe they earn 3000/=, and they want to spend 1000/=, so that she can save the rest.

**I: Did you say a small family? They have small families?**

PCO6: Yes, it depends on someone’s needs.

**I: Anybody else with a different opinion? Why it is easy for them to save?**

PCO4: As number 4, life is now difficult economically but as number 4 if I get something small, it is normally said consume 5 and save 5. It helps me to save, I find it easy to save when I think about tomorrow. You may not know what tomorrow holds for me. Maybe I get a client today, and the following day I don’t get any, I will use what I saved as transport to go back home. Even if I get the least amount, I try as number 4 to save something small.

**I: And what motivates you to save even that little?**

PCO4: May be I get 3000/=, and 3000/= is little considering the economy now. The challenge I face in saving is just what I said, when you have child. The child is innocent, you will have to save something small so that when the child asks for something like bhajia beside the road, you are able to provide. If I don’t save, who will buy for him/her. That is the challenge that motivates me to save even if it is a little.

**I: So it’s the challenge of being able to provide for the child? Any challenge someone may face, number 3?**

PCO3: As number 3, you have a target and you also have things you want to accomplish in a month. So you must have a target. That is what will encourage you to save, because you have a target.

**I: That makes it easy for them?**

PCO5: As number 5, I can save money because you know sickness can come at any time and then even death. So when you save when sickness strikes or there is an emergency, and you have saved, that will help. Another thing, I have a child who will need some things in future even though he/she is still young, s/he will still need it. So saving is a must, what comes to the mind is how this child will survive in the future.

**I: Are there challenges someone may face that can prevent them from saving? Yes, number 2.**

PCO2: As number 2, challenges are there, for these who understand, someone may offer to buy a lot of alcohol for others. That is one challenge.

**I: That is one challenge, any other?**

PCO5: Another challenge, as number 5, maybe I come from… I am in =Kisumu= and you are the first born in the village. So the challenge I can face is when my mother tells me she doesn't have supper or she has some problem. So it will force me to send her the money I earned from my work. instead of saving I will send to her.

PCO3: To add on what number 5 has said, she said you may be a first born. You know your siblings may know you are working in =Kisumu=, so when they are going to school, they call for assistance. So you must have something to give them. Sometimes you have a child who is sick and you have to spend money.

**I: How can those challenges be addressed so that they don’t prevent FSWs from saving?**

PCO6: That is where there is a problem.

**I: Yes, number 6.**

PCO6: You know what, saving is a problem because sometimes you have something and you know that you should be saving it, save even 2000/= and you know that if you save this money in a month you can have some good amount after some time. In the middle of the saving the devil knows you have some money and the money is good and you want to use it, this money will be diverted to treatment, or you are called there is some emergency at home or your brother has been sent away from school and needs school fees. Everyone at home knows you are working in =Kisumu=, your mother knows her daughter is working in =Kisumu= but they don’t know the kind of work you do. So, you are forced to send that money. You have forgotten about other things and send the money. So for you to save fully, you need to have some support. If you are alone, without any support it is difficult. On my side, it is difficult because sometimes after saving a total of 10000/= there is an emergency that you cannot avoid. If you try asking for help from others, they don’t have and others complain you like asking for help. So you will be force to take money from your savings.

**I: Number 5.**

PCO5: As number 5, it depends on the amount of money you have earned. Even when your mother or your family need some help, you don’t have to save the amount you have been saving before you had that problem. If you were saving 2500/= or 3000/= or 2000/=, you can reduce it. Send your mother 2000/= because that is what you can afford and save the remaining 1000/=. Someone like me can do that.

**I: What do think are the disadvantages of not saving?**

PCO6: It forces you to do something you should not do.

**I: Like what?**

PCO6: For example, when you go to work, there are good people you may meet. When you want to have unprotected sex, they allow you to do a HIV testing first and when the result is negative, you can have unprotected sex. But there are others who will want unprotected sex without testing, and when you try reasoning with him he feels that you are refusing, and you also need that money to help you fix something, so you are really risking.

**I: Any other disadvantage of not saving? Number 9, any disadvantage of not saving? (Silence) Number 2.**

PCO2: I saw some disadvantage of not saving with a lady who was killed here in =Car Wash=. She was shown money and she thought that the money will help her where she was stuck. She forgot to consider where she was going was like. She went there and was killed. Not saving also puts us at a risk.

**I: Any other disadvantage of not saving, number 10?**

PC10: When you have not saved money and you have an emergency, you don’t know where to start from, you are just stranded.

**I: And advantage of saving? Those who have not told me about disadvantage, any advantage of saving?**

PCO6: You freely drink alcohol as much as you want. You get drunk until you can only shout “there is nothing” You don’t even know what is not there but you keep shouting “there is nothing”. Drunkards must always say “there is nothing” and even when there is something. An educated drunk even counts water. They will even speak English that they were not taught in school.

**I: That is the advantage according to number six, somebody else, advantage of saving?**

PCO1: Advantage of saving is to have a large sum of money and you use it.

**I: What do you use it for?**

PCO1: I use it *kuchafua meza* (buying a lot alcohol).

**I: That is number 1.**

PCO3: Number 3, the advantage of saving is that you can pay fees for the term once or pay rent for two months once without stress.

**I: Paying in instalment?**

PCO3: Yes, paying by instalment.

I: **Yes, number 8.**

PCO8: The advantage of saving, your life improves and stress free because you know where you can find something… [crosstalk]

PCO6: …You have time to sleep and when a client calls, you tell him “stop bothering me, why are you bothering me and I’m sleeping, you can’t get someone else”. You know you have some stock and you don’t know about tomorrow.

**I: That is number 6, when you have stock you are not bothered? Where do female sex workers save? When you want to save where do you prefer to save?**

PCO6: I don’t go to the bank; I don’t know whether others go. But I prefer two places, phone and in the house.

PCO1: I support number 6...

P: …Mshwari.

**I: That is number 1, you support number six.**

PCO1: I support number 6.

**I: Someone else? Number 10.**

PCO10: We can easily save in Chamas, because you can save in the house but then you get some commitments you use the money saved, or you are saving on the phone and you get some financial commitment. When you are saving in the chama, you know you should contribute certain amount by Friday, and when it gets to Friday I get this amount, and you know at the end of the chama you get a particular amount. So the best place you can save money is Chama.

**I: Why do you prefer chama?**

PCO10: Because whether you have money or not you have to pay, so at the end of the month or at the end of the week you know the amount of money you will receive.

**I: Ok. You had raised your hand**

PCO3: Almost the same thing, as number 3, if you are in a Chama… if you keep money in the house you may be lazy, or even on the phone you can have a target to deposit on the phone in a week. When you are in a serious chama, you must look for your target and not spend it but when you have it your phone you become lazy and you end up spending much because you either have it in your phone or in the house.

**I: Number 6, why do you prefer the house or phone?**

PCO6: You know the phone has Mshwari and KCB, you can deposit it on your phone and when you want it urgently you just withdraw.

**I: Anybody else with a different opinion? Phone or Chama?**

PCO1: in my opinion, I cannot save money in a Chama because we know each other as sex workers. Maybe you are given 20000/= and you can see the 20000/= you will just go to =Kitale= that day and you are gone never to return because we have people who have children and those who don’t have children, their children are in the village. They are all alone, she has a plan to leave… you give them money from Chama, they will disappear with it and you will not find them. That is why I prefer a phone or in the house.

**I: You prefer a phone or in the house. And do female sex workers live beyond their means? Their lifestyle is higher than the money they earn.**

PCO3: It depends.

**I: Why do you say so?**

PCO3: As number 3, It depends. Some of my friends are fully away from their houses. They only have a mattress, a stove, two plates and a cooking pot in their houses…

PCO6: … and a carpet and mattress. They don’t even have seats and that is all what life is to them.

PCO3: …and a small radio for listening to news with and that is all.

**I: Do the rest agree with what number 3 has said? Any different opinion? Are there those who live beyond their earning?**

PC01: There are those who have one child only and they want to stay in a house where they are paying 10000, they want where they can use shower and dress well when they wake up but they are earning little. You can find them staying there for two months then relocate on the third month because they can’t afford.

**I: That is number one, any other person with a different opinion? Number 8.**

PC08: I support what number 3 said.

**I: What did she say?**

PC08: She said you can put a carpet on the floor, stay in a house you can afford to pay, put a carpet and a mattress, look for a small gas cooker and two cooking pans, life just continues.

**I: Those who live beyond their means, what can be done to bridge the gap so that they can live a life that is equal to their earning?**

PCO8: As number 8, there are those who like showing off but they can’t afford, they will live there for even two months or even one month only then to relocate somewhere else. She is someone who keeps relocating, they cannot live within their means. They just want to show off.

**I: Number 2.**

PCO2: As number 2, people like that should balance the money they have and the life they want to live. They should not live beyond their means.

**I: Those who live like that where do they get money to sustain the life they want?**

PCO6: There is some difference, there are those who find good clients who rent for them houses. So there’s a difference. Is that not true? There are clients who are bosses who would tell you “madam look for a house” and every month they send you something small. And when they are in Kisumu you have to serve them sexually. You know when it is a client that you like and he calls you, you will not go because you know today the boss is around. You rush there without looking back.

PCO1: The person who pays rent is around.

PCO3: You have the person paying rent around.

PCO6: Yes, and you have to be very careful so that no client knows this house. You tell them you stay in =Kibera= while in real sense you stay in =corner maji=. When they call you, you tell them to wait for you at the stage. Because you know the owner of the house can come at any time without giving notice and start fighting when they meet at the house. So you have to be very careful.

**I: That is number 6 opinion, any different opinion? Number 7.**

PCO7: I support what number 6 has said.

**I: What has she said?**

PCO7: She has said that people are different, there are those who get good people who pay for them rent. And when this client is coming, she cannot meet other clients and would not want these clients to know where this house another client is paying rent for is located.

**I: We have discovered that there are clients who pay rent. Where else can get money, take loan when they want?**

PCO1: In sex work, as number one, there are people who are thieves. They steal from their clients so you can’t really know where they get money from.

**I: Any other apart from stealing? Number 3.**

PCO3: As number 3, some people have money and you can’t know where they get money from. These things happen and let no one say I’m lying; you can agree with a client the amount to be paid for unprotected sex. There are those who accept unprotected sex and agree on the amount to be charged. Some go as far as piercing the condom and insist for the client to pay for unprotected sex. They would argue the agreed amount for unprotected sex is 1000/= and protected sex is 500/=, right? And she is the one who has pierced the condom and the semen has spilled on her. She will demand that 1000/=. If you get a good client who does not want conflict he will pay. He will pay peacefully.

**I: So this is what they can do to get more money? Any other thing they would do? Yes, number 8.**

PCO8: When you go to the club and find a client who buys you alcohol, you just lick one bottle and your client is counting the bottles. At the end you count the bottles and you are given your money. You strike a deal with the waiter.

PCO3: They can even bring for you water. In this bottle

PCO6: She can fill the alcohol bottle with water.

PCO3: It depends on your deal.

PCO8: So you just count the bottles, when you put water in the bottle you can’t know whether it is water or alcohol. It depends on your deal with her… [crosstalk]

PC07: … you pour in a coke of 50/=.

**I: Anything else? number seven has added something. Someone else or something they do to increase their income?**

PCO6: To increase their income through sex work, it depends on the sex style and how you do it, using different styles, even if it is using your hands to support the position. Stop laughing, why are you laughing? You know sizes are different and women are also created differently. Let’s stop laughing, I am just saying the truth, if we all undress here we are all created differently, it depends on how your *migingo* (*refers to the female’s private part*) was made by God. You may have a long, short, shallow or a deep vagina. It depends on the kind of sex style. You may have a style that you have to use your two hands to support your position that is so satisfying and makes a client not to leave you no matter what. He concludes that “I have to take your number I will call you tomorrow”, so when he is driving he just thinks about that.

PCO3: Now you are the one who will not forget

PCO6: Now he has forgotten, anytime he just remembers that there is a madam in =Kisumu= who really satisfies me.

**I: That is number 6. Any other person? What they do to increase your income?**

PCO2: As number 2, there is a lot going on where we do sex work. There is no sex worker who is good. The more you talk to a client, you hug him to confuse him.

PCO6: You give him warmth.

PCO2: yes, you warm him. The more you give him warmth, you pick pocket and the more you pick pockets your income increases.

**I: Do you think what they do to increase their income put them at risk?**

PC: So much.

**I: How?**

PCO6: It is not good to steal from someone. You can steal from someone who has protected himself, they can even shoot you because you don’t know how he is protected. They can shoot you especially those that we have said are hugged as you pick pocket, they can kill you. There are men who are educated and have met many people like us, they know when a female touches a particular place, there is something you are trying to do. A man knows the part that when you touch you need sex and there is somewhere you touch and they know you are after their money. So they are normally alert.

**I: That is number 6, any other person? How do the things they do increase risk? Yes, number 3.**

PCO3: Like the way I was saying about those who tear condoms, may be they are protecting themselves with PrEP, they and their friends tear condom knowing they are protecting themselves using PrEP not knowing that there are also STIs that they can get from the clients. So they are risking their health.

**I: Number 2.**

PCO2: I have talked about having a client and you steal from them, and you don’t know their reaction, that is why we are normally killed. You have stolen from them and they have found out it is you, they will kill you and leave you there.

**I: And when there are no clients, what do girls do to get money?**

PCO8: The clients you have saved their contacts on your phone, you will be forced to call them, you may find a few.

PCO6: There are also others who just keep you in their minds, they just send you something, you don’t have to meet with them first, they will just send you something small to help you. There are those who even when they are away, they remember and can send you something small.

**I: How do they keep you in their minds?**

PCO6: They just send you something on your phone. They can tell you “how are you” I’m fine but I’m broke, what do we do? Then they will say let me send you 1000/= to use. So it depends on how you deal with your clients.

**I: That is number 6, number 10.**

PCO10: I want to support what number 6 has said then add some points. You can have clients and at the same time they may fail to give you so the best thing, we should find a way of helping ourselves. You know you should help yourself for others to help you. You can even look for some business as number 2,3 and 4 said, you look for some business to do, like during the day I do business because you can’t go for sex work at 7pm. You go for sex work from 10pm or 11pm so you cannot sit in the house for all this time, you have to look for something you can do. So, I support looking for what we can do during the day before we go for sex work because sex work is never easy throughout, sometimes it is hard and other times it is easy. So when it is hard you can fall back on your business. When business is not doing well you come back to sex work because sex work does well from Wednesday, Friday and Saturday. So you just want to sit and keep waiting, you have to look for some business and to do like salon and selling cosmetics.

**I: Yes, number 3.**

PCO3: As number 3, I see my some of my sex work friends carry groundnuts, *simsim* (sesame seeds) so that when there are no clients, they just sell their ball gums and return to the house. Unlike when there are clients, they will not sell the groundnuts and go back with them home because they were busy but when there are no clients, they just sell their groundnuts and go back home.

**I: How do they know there are no clients?**

PCO6: Like now when schools have been closed, or I’m I lying?

PC: You are not lying.

PCO6: It is not easy, it is difficult. When schools are closed, people have problems. Students charge very low amounts. With them you know even if you give them 200/= she agrees because she has taken long without sex, so when they are able to do it, they want to do it a lot. So clients forget about regular sex workers will be considered later

PC: After students have gone back to school.

PCO6: So when schools are closed, we find it very difficult.

PCO3: Because students are given as low as 200/= or just taken for lunch, they just accept... [crosstalk]

PC: …They just charge 200/= for sex or a dress for sex. We go for sex work with a target…

PCO6: …They have no responsibility; they just look for money to buy body oil or sanitary towels. Just two things.

PCO3: After they have been bought for lunch, and transport, that is sufficient for them.

**I: Let’s not forget to mention our numbers when we talk. So when schools are closed, there are no clients?**

PC: There are no clients.

**I: Other times apart from when schools are closed? When again do you find there are no clients?**

PCO1: Monday, Tuesday, till Thursday, there are no clients. You can get even only one client, sometimes only your regular clients. There are no new clients, and it is the new clients who pay well.

**I: That is number 1, number 3.**

PCO3: As number 3, sometimes work is good. If you have not realized, there are clients during end months. There are clients during month ends if you compare with mid-month.

**I: How much money do sex workers borrow or have as debt?**

PCO6: Debt from the shop or grocery?

**I: Any debt.**

PCO6: All of us here know our budgets

**I: That is number 6 who have just spoken. Yes, number 1.**

PCO1: As number 1, I can only have a debt of 500 shillings, that is the only debt I can borrow.

**I: Number 9, how much can you borrow normally?**

PCO09: There has to be debt. When there are no clients and you don’t get anything and you have not saved, you will be forced to borrow.

**I: You can have a debt of how much, normal debt?**

PCO9: 300/=, 400/= or 500/=.

**I: Number 5., how much debt?**

PCO6: As number 6, I can’t say it can be 300/= because if we consider buying breakfast, you want to buy sugar, milk, tea leaves, what you want to take tea with, it is almost 500/=, you still have lunch and supper that you will need before you go to work. It is more than 300/=, it is just about 700/=.

PCO8: Even 1000/=.

**I: Number 8 is saying 1000/=. And have sex workers ever thought of leaving sex work? Or anybody you know about that ever thought of leaving sex work?**

PCO6: You have to think about that because when you are doing this work, there is a certain age that you reach and start thinking that if I could save some money or decide to open a salon and employ those braiding, doing blow dry for income and I rest, something like that. Someone must have that desire. But you know everything must normally have an end.

**I: Number 7.**

PCO7: As number 7, I’m now tired, I’m thinking of leaving sex work next year May.

**I: Is it something you have decided on your own or it is something you decided with other sex workers?**

PCO7: I have only told a friend of mine.

**I: Somebody else, number 3.**

PCO3: As number 3, there are some sex workers who long to have families. I have seen people who pose as clients but they have come to look for wives. If they take a female sex worker as a wife, that will be the end of sex work because they will settle down in their houses as wives.

**I: And when someone thinks of leaving sex work, is it something somebody thinks alone or it is a discussion in your work places?**

PCO3: Mostly it is an individual’s decision.

**I: Someone decides on her own?**

PCO7: Because you are the one who knows how you feel in your body.

PCO3: Someone may say I long to have a family and if I get someone to marry me, that is how I will leave. They are thinking of getting married.

**I: What makes somebody think so? What normally happens to make someone think they want to quit?**

PCO7: As number 7, what I have gone through that makes me decide to leave it this coming year, sometimes we meet challenges. You can find that the penis is too big that the money you have been paid is all used in treatment. That is what can discourage you with the work.

**I: Number 10.**

PCO10: I support number 7. The reason I may decide to leave this work is because of the risk we are exposed to. You can have sex with someone and agree on the amount to be paid but afterwards when he is done he does not want to pay you. Sometimes he even wants to kill you so you feel it is not worth it, let me look for something else to do apart from this, and leave sex work.

**I: Yes, number 3.**

PCO3: I support what number 5 has said. Number 10. You can agree on the amount to be paid and the client does not have cash. After having sex with you he wants to send the money to your phone. There are those who sometimes reverse the money, it is like you worked without any pay. They reverse money back to their phones. These are some of the things that can make you think of leaving this work.

**I: Number 8.**

PCO8: As number 8, you know it depends on the agreement you made. In sex work you may agree with a client the number of shots and charges but after having sex with him, what they want to do to you, the kind of sex they want to have with you, so you really regret that if you can find something else to do or find someone, I can leave sex work. It leaves you with pain all over your body.

PCO7: It is pain that even makes it difficult to sit down…

PC08: …Sometimes they are people who use drugs, you meet them after using the drugs…

PCO6: …It is painful that it makes you scream in pain when you are taking a bath or going to the toilet…

PC08: …Nothing… [crosstalk]

PCO6: …He rubs your vagina until when you go to the bathroom you are in terrible pain an dyou just clench your teeth…

PCO8: …So when you come back feeling that way, you really regret what searching for money has put you in…

PCO6: You are laughing? It is true, the friction leaves you in a lot of pain.

**I: Somebody else?**

PCO4: I support number 8 as number 4.

**I: How do you support her?**

PCO4: I support her on sex work. Sex work has challenges, you have met a client and from outside he looks small but his penis is big, you long to run away but being that you don’t have money, you persevere but feels you like leaving sex work.

PC05: May be you left your house with a problem, you will have to agree because if you don’t accept, how will your problems be solved. You just have to agree…

PC: …So you just have to agree even when you have to do it crying…

PCO6: You really persevere having sex.

PC05: Even when they don’t want to soothe you as you cry, just accept as long as he will give you the money.

**I: Those are other reasons that make others feel like leaving sex work. And what do you think make them not leave sex work after they have planned? You may have planned to leave after 2 years because of certain reasons, what normally prevents them from leaving?**

PCO5: As number 5, may be you get a job that you will be paid monthly and you are used to earning daily, you may start thinking salary at the end of the month is far. I’m used to earning daily, if not daily, I can skip a day like Tuesday, Friday, Saturday and Sunday you earn and salary comes after four weeks. So something like that can make you not to leave.

**I: Any other reason why they are not able to leave, those that plan to leave sex work? Number 3.**

PCO3: The only thing that can make you not to leave and you had planned to leave is when you had a target. As number 5 has said,

**I: Number 7.**

PCO3: Number 7. May be you have set a target that by a particular month, this year I should be having a specific amount to do some business then you are faced with a problem that forces you to use all that money.

**I: Number 6.**

PCO6: What may prevent you from leaving even when you want to leave, sometimes you may plan because of facing challenges in sex that are difficult. Leaving is a problem when you start thinking on how you will survive when you leave it. But when you have some money that you know you will use to do something when you leave, it will be easy to leave this work.

**I: Do you think when somebody wants to leave age is a determinant or are there other reasons that make somebody want to leave? Yes, number1.**

PCO1: As number 1, age cannot make you leave sex work but there are reasons. There are many challenges that would make you decide to leave. Like me I wanted to leave. I left but it did not take three days before I came back because of what I had gone through, what helped me was only God. I went as usual and found a built man who had money and we agreed. The only problem is that he told me to go to =Mamboleo=, so I left =Dunga= to =Mamboleo=. When I arrived in =Mamboleo=, what I faced! We had agreed that when I come to your house, the charges are 5000/= and he was okay with that. I told him to pay first, he gave me my 5000/= and I put it in my purse. When I went outside, I decided not to get into his car with the money, I took…there is someone who normally keeps money for us and we take later. You don’t know when you will lose the money when you are drunk. So when you get money, you give him and take the following day. So I gave him and boarded the vehicle and went to =Mamboleo=. What I went through in =Mamboleo=, he wanted to kill me. What helped me was some pepper I had carried. He held me and he was huge, he was the type that when he holds you, you can’t even move an inch. I got a little space and got pepper from my pocket and put on him and got a chance to run away but he followed me and if he would have caught me, I would not be alive today. That is the day I said I have left sex work and I will not go for sex work again. From that day I did not go and the following day, the third day I went. I just found myself there. So there are many challenges, you may want to leave but you can’t leave because all your hopes are there. Your children are eating from there, your children are going to school with money from there, you are paying rent, your children are dressing, you can’t do anything. There are challenges in leaving, you can’t leave.

**I: Those who are leaving sex work, they normally leave when they are what age? Majority of those who leave sex work?**

PCO1: The person I know who has left sex work completely was 46 years old. That is the only person I know who left. She left completely.

**I: Any other age? Age when they usually leave sex work, or it is 46? If someone knows anybody who left.**

PC: There are those who leave at 50 years…

PCO6: …It depends on a person’s body. How they look like.

**I: Yes, number 5.**

PCO5: Some leave even before they are 40 or 30 years, if you get a man who loves you and has money, they leave because they have found someone they can trust. They leave even before they are 30 years. It depends because you were doing the work for money and you have found someone with money. So you will decide to leave that work and continue with life.

**I: And those who have left, what kind of things do they do? What type of work do they do? Yes, number 3.**

PCO3: As number 3, someone I know who left sex work had separated with the husband and they reconciled. She is now just a house wife.

**I: She is not working anywhere?**

PC03: Yes, she is not doing any work.

**I: Any other person who knows someone who left sex work and what kind of work are they doing? That 46-year-old, what is she doing? Number 1.**

PCO1: She decided to leave =Kisumu= and went to her rural home, where she was married but she started doing sex work after her husband had died. So she is just in her home taking care of her home.

**I: Does someone else know anybody who has left and which kind of work they are doing? Yes, number 2.**

PCO2: Some leave after saving as we have been talking about here and started some business.

**I: And do you know cases where sex workers left sex work and returned? Or if you know anybody who left sex work and returned? They decided to leave and then returned.**

PCO1: I said I left and returned.

**I: Why did you return?**

PCO1: The reason I returned was because it is the only thing that can help me take care of my children, that is the only thing I can rely on in my life and my children easily.

**I: Any other person apart from number 1, anybody who left and returned? Yes, number 8.**

PCO8: I have a friend who found someone who took her and lived as married and was doing everything for her. They stayed for one year and the man abandoned her. There was nothing she could do and she had been used to doing sex work, so she just returned back to her work.

**I: Anybody else who knows someone who left and returned?**

PCO2: As number 2, someone can decide to get married because they are tired. After they have gone, I don’t understand how men reason. You can go then they begin playing with you, you begin to disagree. The more you stay, the more you experience disagreements [Inaudible clause].

**I: And what are some negative things that happen when they leave and return? What kind of negative things can happen?**

PCO5: Maybe in that marriage, when you left and got married, in that marriage, maybe your husband has threatened you “I will kill you, you were just a prostitute, I just picked you I don’t know from where. Something like that.

**I: Verbal abuse, any other negative thing that can happen when they leave and return, what other negative thing can happen? Let’s not be tired, we are about to finish.**

PCO6: Something negative that can happen to them?

**I: Yes.**

PCO6: There can be some form of disrespect because of the kind of work you were doing, there can be some form of disrespect from that family or that man. They can even give you a nick name that they use to call you and you may not like it that makes you feel if that is what marriage is, let me leave and go back to where I was working.

**I: That is number 6, anybody with a different opinion? Yes, number8.**

PCO8: May be he married you when you already had a child, you stay with him for some time then he begins to reject your child and you took care of the child before you met him. So you will see your child’s education and life are being affected and your child is not doing well, you will be forced to come back where you were for your child’s life to go on well.

**I: And for those who plan to leave sex work, there are things someone may want to accomplish before you leaving sex work, right? Maybe you want to accomplish one, two, three, so that when you leave sex work, you have done whatever you wanted to do, right? What can these things be like? Yes, number 8.**

PCO8: As number 8, with the work I do, what I would want to accomplish is to get some plot and buy, build some rental houses that me and my children would benefit from some day. That is the target I have.

**I: And have you started working towards that target or you are still thinking about it?**

PCO8: I have started.

**I: In what way?**

PCO8: I’m in a Chama and I opened an account so when I get money from Chama, I divide some to deposit in the account and use some in the house.

**I: Number 6.**

PCO6: She has said what I wanted to say.

**I: What is it that you wanted to say?**

PCO6: I wanted to say that when you feel tired, it is good to buy land because before you get somewhere you call your own or a husband, you are still not stable and you have children, you can’t just leave your children just like that. You should have a plot you have purchased so that when one time you die; your child is able to identify your plot. You can even build some rental houses, two doors, that can fetch for them some income in case they are still going to school.

**I: Okay, number 2.**

PCO2: As number 2, you know we cannot all buy plots. Some may even buy cattle as savings for the future.

**I: That is something you wish to accomplish?**

PCO2: Yeah.

**I: Have you began the journey toward that?**

PCO2: Yes, I have even put everything down.

**I: Yes, number 3.**

PCO3: As number 3, I have the same suggestion as hers, I have already begun with goats and poultry, I have them at home. So from there I can get something.

**I: Any different opinion? Who looks very tired, number 9? What are some of the things you can do?**

PCO9: You can do things with the future in mind, you cannot focus so much in the past because you don’t know what the future holds.

**I: So what do you wish to accomplish before you leave sex work?**

PCO9: You can buy a plot and build some rental houses.

**I: Apart from a plot, what else can they do?**

PCO9: You can buy a goat or cow.

**I: Apart from buying a goat or cow, what else can they do?**

PC: You can buy a motorbike.

PCO6: You can buy a vehicle for public service, you can earn something from that.

**I: That is number 6’s opinion. Number 2, why are you laughing?**

PCO3: As number 3, there is a suggestion that you can pay 150,000/= for a tuktuk and pay the rest of the money later from the money you earn from tuktuk.

PC: You open an account for it.

PCO3: …it can even buy a motorbike.

**I: We are getting different opinions. What would somebody do before leaving sex work so that you have something to fall back to? Apart from the ones mentioned, is there any other? Yes, number 3.**

PCO3: I have seen someone who is planning to leave, she has begun a Mpesa business, she has her business. When she leaves, she will be able to sustain herself.

**I: Do you know anyone who left sex work 5 or 10 years ago?**

PCO1: As number 1, I know one who left four years ago.

**I: What made it easy for her to leave or why did she leave?**

PCO1: I think because she did not tell me that she left because of her age.

**I: Number 10, do you know anybody who left? What do you mean, this does not see, it records?**

PC10: I don’t know.

**I: Number 2, do you have something to say?**

PCO2: Yes, I have seen someone who has left for 3 and a half years. She was longing for marriage, so she got married.

**I: And did they experience any difficulties? those you know who left, did they experience any difficulty? Number 2.**

PCO2: I can’t know very well but in sex work, there must be challenges.

**I: We had talked about Jitegemee earlier; this is the last section for those who are tired. We are just about to finish.**

PC: Our children are outside.

**I: They will be okay. We had talked about Jitegemee earlier and emphasized that Jitegemee wants to help female sex workers to save. So it is not like Chama. Chama has an amount you are supposed to pay and the time to pay it and it is a particular amount. Jitegemee is not like that, here you decide the amount to save, the time you want to save it and how long you want to save it. And you can withdraw it any time you want it without interest. If you save 5000/=, that is the amount you will get without interest. The money you save is what you will get and this is to help female sex workers to rely on themselves in future. According to what we read, do you think Jitegemee will be accepted by female sex workers? Is it something they can accept? Yes, number 1.**

PCO1: As number one, when they are given good advice, they can accept.

**I: What kind of advice?**

PCO1: Talk with them, sit them down and talk if it is something good, they will not reject, they will accept.

**I: And what type of female sex workers will accept? In sex work, we have… I don’t want to say categories. We are different people, yes.**

PCO3: As number 3, there are people who are focused, they do this thing but are very focused. There are also those who drink all their money and are not saving. So, the ones who are focused, we can talk to them until they accept.

**I: They can accept. Number 6, do you have something else?**

PCO6: No. she has already said.

**I: You are now tired. We are almost finishing. Out of 10 girls that each person knows, number 9, out of 10 girls how many will accept Jitegemee?**

PCO9: Out of 10 people?

**I: Yes, out of 10 girls that you know.**

PCO9: A maximum of 6 or 7 people.

**I: 6 or 7, choose one.**

PCO9: 7.

**I: 7, number 4, out of 10 girls how many will accept Jitegemee?**

PCO4: 6.

**I: Number 3.**

PCO3: Let me say 8.

**I: Number 8.**

PCO8: Also just that 8

**I: 8 of 10 girls you know, number 2.**

PCO2: I know of 5.

**I: You know 5, number 1.**

PCO1: Of the girls I know, they can be 4.

**I: 4, number 7.**

PCO7: 5

**I: Number 5.**

PCO5: Like 7

**I: 7, number 10.**

PCO10: 7.

**I: 7, number 6.**

PCO6: 5 because you can’t know someone’s mind.

**I: And the remaining ones like one has said 7, what will make the remaining 3 not accept in your opinion depending on how you know them? Number 9. Who is talking?**

PCO5: May be challenges.

**I: Which kind of challenges?**

PCO5: May be you can tell them if you leave this thing (refers to sex work) you can do this. If it is not income generating on a daily basis, then she will not accept. She will say no.

**I: That is number 5, yes number 10.**

PCO10: Okay, not all who come to this field have problems that people have gone through. Some have just come for entertainment like those who have just completed their form four, they don’t have a lot of responsibility. So, when you tell them to join this in order to save, it is like you are wasting for them time. They want to get money, rave, and use all the money and look for more from someone just like that. So not everyone does this work because they have problems.

**I: Okay, someone else, those remaining from the ones mentioned, what will make them not accept Jitegemee? (Silence) You look tired but we are almost finishing. Are there reasons or there is none?**

PCO1: There are reasons, there are those who are doing sex work but this job we are doing, we are mobile. Today I’m here, tomorrow I will be in =Eldoret= for one week, then I head to =Mombasa=. With this job, you can go anywhere because you can work anywhere. That is what will make some people not accept to save with… what is it called?

**I: Jitegemee.**

PCO1: Jitegemee.

**I: What can we do to make them accept? Somebody had said we talk to them and explain, what do we tell them when we talk to them? Yes, number 4.**

PCO4: Like those 6, I said I can talk to 6, the remaining 4, we can begin something that brings people together like Chama. In case one has a problem, we stand together with her, so the 4 will see the unity we have and join.

**I: Apart from bringing people together, anything we can do to make girls accept Jitegemee? Number 2**

PCO2: What can make them accept, you know life outside here is difficult so your friend can come to the house to visit and finds you have made tea with milk worth sixty shillings and will wonder that this lady does not have a husband, where does she get her money from? So it will make her also want to join it.

**I: And the way we talked about Jitegemee, what do you think they will like most, of the things we said Jitegemee will do or expecting to do?**

PCO1: What will make me like it so much is money.

**I: In what way?**

PCO1: When you tell me about money, I will accept because all my needs are about money.

**I: So we are not giving you money. Jitegemee is not giving you money. You will save your own money. Will it still be acceptable because it is your own money that you save? Number 6.**

PCO6: If it is your money that you are saving, I had said earlier that it depends on someone’s income. You may desire to save but it is impossible. So, you know that it is difficult, you must just have support.

**I: Are there ethical concerns among female sex workers about Jitegemee? Or can they feel their rights are violated as we had talked about Jitegemee?**

PCO6: There are no ethical concerns, but when you live alone you have to find a way to rely on yourself as you have said. And if there is someone who can support you, you can find a way of balancing.

**I: Number 9, which challenges will we face when we come to tell people about Jitegemee?**

PCO9: Challenges we would face?

**I: Mmm. Challenge. Yes, number 3.**

PCO3: Let me help her. The challenge we would face is that you know sex workers, some people are…they can even abuse you “this is my money” It will appear to them like you are controlling them on their money. It is like willing buyer willing seller. There are those who will buy your idea, there are those who, however much this thing will help them, they will not accept.

**I: Another challenge? Number 7, give us a challenge, we are almost finishing.**

PCO7: As number 7, there are sex workers who are difficult to deal with, some will not even listen to what you are telling them and there are those who will listen. So it will not be easy.

**I: What can we do so that they can listen before they start abusing us the way number 3 is saying? Anybody can respond.**

PCO6: First you must give them information and any advice that they can be given in order for that to happen. Secondly, they must get support from other places and thirdly if they consider the lifestyle of those in Jitegemee, they can be encouraged to take that opportunity.

**I: When you are saving in Jitegemee, number 9 how much will you prefer saving weekly? How much will you manage to save every week?**

PCO9: I can save 500/=

**I: 500/=, number 3?**

PCO3: I had said 7000/=.

**I: 7000/=, number 8.**

PCO8: I can try and save 1000/=.

**I: Number 2, without straining yourself. Number 2.**

PCO2: I can save 700/=

**I: 700/=, number 1.**

PCO1: I have a question concerning saving. Can I save for 3 days then withdraw?

**I: It is your money; you do with it what you want. So in a week…**

PCO1: I can save 3000/= in a week.

**I: 3000/=, number 7.**

PCO7: 2000/=.

**I: 2000/=, number 6.**

PCO6: 1400/=.

**I: 1400/=, Okay.**

PCO6: Weekly.

**I: Weekly, yes. So, in case there are female sex workers who cannot raise the money they have set as targets, we have said the targets we can set, so in case you cannot meet your target. Remember this is not like Chama, you do it on your own. So in case you are not able to meet your target for that week, what kind of things can they do so that they are able to save that target for the week? This is the last question. Yes, number 3, we have gone round each of us saying the amount that they can save per week, right? Let’s say you are unable to get your target for the week; what kind of things would they do to get that money they have set as the target for the week? Number 3**.

PCO3: As number three, as I had said earlier I have a friend who carries groundnuts, *simsim* and ball gum every time she leaves the house. That will help her to get money and meet her target.

**I: So that is doing something other than sex work?**

PCO3: Yes

**I: Number 6, you had something to say?**

PCO6: I was saying that what you can do in my opinion, in a week maybe if she gets little money, but in case she gets more money at once, she can consider and say today God has blessed me, let me save this amount so that I have something at the end of the week.

**I: Any other opinion, yes, number 7?**

PCO7: As number 7, if by Sunday… I have set my target, I can try to save double my target the following week.

**I: Any other person with a different opinion? Or if you are in a Chama, what can you do to get money you are supposed to save there. Chama is fixed like if it is a Chama where contributions are done weekly, you have to save it. What can you do to get this money on a weekly basis? We are starting, number 9. What can someone do?**

PCO9: You can have some business to help you save on Chama.

**I: Number 3, do you have a different opinion with…**

PCO3: No.

**I: Just that, selling?**

PC03: Yes

**I: Number 8, any other opinion?**

PCO8: I can buy clothes and sell to my customers. So I set a later date for collecting money from them. So on the collection day, I know I will get my target for saving.

**I: Number 2, what can you do to enable you get money to save in Chama?**

PCO8: I will look for my clients I have saved their numbers on my phone. If I call all of them I will not fail to get something from one of them.

**I: Number 1.**

PCO1: For me I had set a low target so that I don’t fail to save. So I don’t set high targets that I will not be able to get, I set low targets. So if I set a target of 3000/=, I’m sure I will get it in the week. If I get more, I will set it apart. So I don’t set high target that I will not manage then I begin struggling and looking for help.

**I: In case we begin Jitegemee, where would female sex workers prefer to save their money? Number 6.**

PCO6: Yes, what have you asked? I’m sleeping in class. What are you asking?

**I: If you join Jitegemee, where would female sex workers prefer to save their money?**

PCO6: When I want to be 100% I will not lose money; I will go for mobile banking like Mshwari or KCB. So if I use fixed account even for one year, I know very well the amount I will deposit every week. I will be sure nobody will disappear with my money because I know when it reaches a certain date, I will get it.

**I: Number 7, any different opinion? Where it is safe to save money?**

PCO7: I save in Mshwari.

**I: Mshwari.**

PCO7: Sometimes it has... I can lend money to some people and return it with some interest in 10 days.

**I: Mshwari. Number 4.**

PCO4: Where my money is safe is just the bank.

**I: Bank. Number 9.**

PCO9: I support bank.

**I: Bank. Number 2.**

PCO2: I have heard about locked… locked what?

PC: Fixed account.

PCO6: You can save in locked account or you can save in fixed account. They are two places. There is locked account that you can’t withdraw, for emergency.

**I: You also prefer locked account number 2?**

PCO2: Yes.

**I: Number 8.**

PCO8: I can open a fixed account and deposit 50/= everyday so that I have something at the end of the year and the interest the bank will give me.

**I: That is the bank?**

PCO8: Yes.

**I: Number 3.**

PCO3: I prefer the bank.

**I: Bank, Number 5.**

PCO5: Bank.

**I: Number 10.**

PC10: Chama and bank.

**I: Number one.**

PCO1: I will prefer bank but I may not get time to go to the bank.

PC05: You do the transactions on your phone, withdraw to your phone from your account.

**I: Any other question? Anybody with a question? Question? Yes, number 7.**

PCO7: You said this research will just give us information on savings and loans without something we will receive?

**I: Yes, we just give information on how you can save your money. From the money you earn, how much can you save without straining because there are daily needs also.**

PC07: I just use my own money?

**I: It’s just your money, there is none you will be given or added, you just save your money. Are we all okay? Is there any other question? (silence) Okay, thank you all.**

PC: Welcome.

**END**
