# Supplementary material for: Eat a little and save a little: A qualitative exploration of acceptability of a potential savings intervention to reduce HIV risk among female sex workers in Western Kenya
Source: PLoS One. 2024 Dec 19;19(12):e0310540. doi: 10.1371/journal.pone.0310540 (PMC11658496; doi:10.1371/journal.pone.0310540)
Supplement: S1 File — (ZIP) [file pone.0310540.s001.zip › Jitegemee Transcripts and Dissemination Notes for Journal/FGD E.doc]

**FGD ID: FGD E**

**MODERATOR: NANCY OUNDA**

**NOTE TAKER: JUDITH AYALLO**

**DATE: 26/APR/2022**

**CATEGORY: ABOVE 30 YEARS, PERI-URBAN.**

I: This is Jitegemee study the FGD number is KSME01 and the FGD is being conducted at =Nyamasaria= the date today is 26/APR/2022, the moderator is (Mentions name) and the note taker (Mentions name). Now are starting, thank you so much for your time, just as I have told you about jitegemee in short, what came in to your mind immediately?

PE09: In my mind I think jitegemee they want us as sex workers to have a voice, you know you might go somewhere and someone is aware that that is the job you do and he/she may look down upon you. Or when you go… mostly when you go to pay school fees, and maybe some children are aware that, “your mother does this or that.” (NAO: sex worker) You know your child must have being placed in a certain standard. So if we join Jitegemee it will give us a voice also. We will have a bank account, you don’t have to have sex with somebody to earn money, and you can save, start a business and do other things rather than having sex with someone.

**I: Who has anything to add, PE08?**

PE08: I want to say this, Jitegemee if it is said what comes into my mind is how someone can earn her money on her own apart from doing sex work. How she can get a way of earning so that she can do whatever she wants at the right time without doing it in the dark or using inappropriate means. That is what I think jitegemee is telling us how we can earn or what we can do. It also makes us, that if we find ourselves just like right now the 10 of us are together, we can join hands so that we are stronger so that we can forge a way forward on what we can do in the future other than what we are currently doing.

**I: PE06 do you have anything to say?**

PE06: To support what PE08 and PE09 just said, jitegemee tries to inform us that even if we are doing sex work for example you are from work in the morning you can open your shop and work there. So, someone might say, “Ah even if she is doing this but she has a stall and is doing something.” That is what jitegemee wants us to do so that we have self-dependence,

**I: PE01**

PE01: To me I think Jitegemee is a link to us as sex workers, we have been doing it and we have been looked down upon in the community where we live, we have been stigmatized just as PE09 said, to add just as we are here, we as sex workers , we come from this place I think we have some knowledge on what we can do that at least can lift us to a certain level, so, even if we do it, I know I am doing this job but how can I leave this job and move to another one. Because we have families here, some of us are single parents, if the jitegemee staff can give us a direction on how we can come together as sex workers, so as to give us way forward and continue educating us.

**I: Is there anyone with a different thought? PE07.**

PE07: I think the jitegemee program that you have brought is so good, it is good because as sex workers we go through hardships in this job. Maybe you have gone to work somewhere, and where you went you have not gotten anything, maybe you are going back to the house it’s still night and you meet thieves. They will attack you, personally as a sex worker I was attacked by thugs, I would like to join Jitegemee. I support this idea, I accept it

**I: Is there any one we are leaving out, PE10.**

PE010: I can say that, we should also be given a voice so that we can have a voice because wherever we are, people look down upon us that is what I can say.

**I: Am I closing out someone, PE03**

PE03: I am so happy with the idea and I am so encouraged in my heart, I want to support PE07, she talked some truth because as sex workers you can go somewhere come with no earning then your children celebrate, “Mama is back.” May be when you are coming there is something that you have brought him, but maybe you have come back with nothing. You may not know what to do with the children and you will be worried how to pay rent and do other things. So, I am so pleased with the idea and I support it.

**I: Another to talk last, there are two people who have not answered, PE02 infact 3 people. PE02.**

PE02: In my thoughts sex worker is always at risk, you don’t go there to enjoy, you go there because you lack. You have children and they are your responsibility, like for us we are single mothers. How you can pay rent, feed the children and pay school fees. I think the jitegemee organization if it can encourage/empower us well even if it can offer us capital so that we can leave this job… it is not… I mean you are always at risk because you go at night and maybe you don’t get a client you know you go back home but with a lot of pressure. Maybe you went far, for example I live in =Nyamasaria= and maybe I don’t have fare back, this will force me to walk even up to =Kondele=. You know that is a very dangerous place somebody can hurt you; many people have died. You are going to search for money, so, the way you have brought the organization, we would like to request us some help [NAO: capital] so that we can start some businesses apart from this one.

**I: PE04**

PE04:As number 4 I just support what my peers have talked about that jitegemee is a good thing that as a person it encourages me that as a sex worker, these people know us… I mean they know us as women who exist. For example, I am a single mother, maybe you go out… it’s just a risk just as PE04 said. It encourages me that as single mothers who are sex workers, we have a voice. That is where it encourages me because the more, we do sex work and we have children who are growing and becoming big people, maybe you have a grown up child and she is aware of the job the mother does, you know that lack of respect between you are your child. Jitegemee encourages me that as sex workers, they care about us and they can give us some support that someone can get a side business after leaving this job.

**I: PE05 do you have anything?**

PE05: I also support the idea of my peer, on my side I do it ,my children are well grown. They know that I work in a hotel. When I come back, they ask, “Mama you work in a hotel yet you don’t bring us what remains there, all your food gets finished?” I tell them, “one day I will find what to bring you.” So, I am in support that if you can offer us some help then we can support the idea.

**I: It’s okay we have answered that well.**

ALL: Mmmm…

**I: We also ask, what does sex workers buy with their money and are their individual prices? Now, we want to know what you buy, what you buy daily and we will all talk about it . We will also look at what you buy once a week, what you buy once a month, and what… the least amount of money once a month… sorry… how often for example once a year, once a term one for education and other and the prices for each. Let’s talk about your daily budget? In one day, who will start? What are the things that you have to buy daily, after talking about the daily you will tell us the cost, how much you use per day, PE08.**

PE08: As a sex worker what I have to buy first every day is credit.

**I: Credit…**

PE08: The smallest I buy is for 50 shillings, that is what will allow me to look for clients when I don’t have one nearby. I have to look for them, “Can you send me something if you have, can you do this for me.” So, airtime is my first priority, even this morning I bought and I have airtime in my phone. The next one are basic needs like tea, vegetable to eat and you must make sure you have all of them. The least I can use in a day is 300.

**I: Another person PE09**

PE09: What I buy daily… just like my peer said, I love surfing the internet, I connect with most of my customers on Whats App and others. So, I have to buy bundles… you have to buy airtime then buy the bundles. Another one is since I have a small child you have to buy milk. You have to buy milk and leave there where you want to set off from your home… you leave it with the nanny who will feed her with the milk. So, on food I can spend at least 500/= in a day.

**I: Another person, PE04**

PE04: Sometimes I might go to the road and just stand there as I wait for a client, I must have some condoms, mostly we are sometimes given freely but sometimes we buy them. One packet of 3’s is 50 shillings if I am not wrong. When I bought it last it was 50 shillings? Personally, when I go to the road side… sometimes I can go to the road side sometimes we may go to town. I must have condoms and must ensure that back at home they have something because I am not sure what I will get where I am going, I just have to budget even for the following day. The least I can use around 400/=.

**I: Another person, PE010**

PE010: What I feel is that I have a child and have to buy pampers every day, must buy for her milk, must know how the school going once how they will eat and also buy supper before I can leave, in a day I can use even 600/=

**I: PE07.**

PE07: What I have to do daily is paying the person I leave my children with. Before I leave for this job I must make sure I pay her. She somebody you pay to take care of the children and she must sleep in your house in a day I use around 600/= because I pay her 200/= just to sleep there, that is also expensive.

**I: What about other expenses?**

PE07: It’s heavy because I said I use 600/=.

**I: Oh... 600/=**

PE07: Within the 600/= there is milk, lunch sometimes they also come for supper and you have to give them.

**I: Okay, PE06**

PE06: To support what [mention name] has said, this thing is hard. Everything is expensive because sometimes, apart from airtime and everything you have children. You want that by the time you come out of the house, when to come in the morning you have to plan from supper up to breakfast. Because you may come back after they have gone to school. You must also give them their lunch in advance because by the time you will be back, they will have gone to school. Maybe you have gotten the airtime and everything and made the phone call, but you are told, “just go and pay for the room am coming, You can end up saying, I am going to be given some good money let me just do it( book the room) because sex workers you personally decided that you want it, so you can end up paying for the room on your own. You might end up spending even 1000/= in a day because of the money you spent to hire a room some rooms are expensive, so for you to tell him that you want the money, those are the challenges.

**I: What can you spend in a day?**

PE06: To meet all the expenses I can spend 1000/=.

**I: You have said that some rooms are expensive.?**

PE06: Some are cheap,

**I: How much are the expensive ones?**

PE06: It can be 500/= or 800/=

**I: What about the cheap ones?**

PE06: 200/= or 300/=

**I: Okay, another person.**

PE01: For me the first is airtime, condoms and sometime when I… when I don’t have you know I have to take it on credit from the shop, borrow from the stall’s person, even the person selling charcoal. I am friendly to these people, the child going to school must also be given lunch 50 shillings every day . In a week is its bad, I spend 800/=.

**I: In a day?**

PE01: In a day, in a day 800/=

**I: Okay**

PE01: Life is currently hard that you may not have in the morning and evening.

**I: Who have not responded, PE03.**

PE03: I support what my peers have said, first of all PE10 said that those with children face tough times. For us who have children, you only breast feed if you have eaten. You can’t breast feed the child when you are hungry.Sometimes you come back exhausted that you have no strength… maybe you came back with nothing. You don’t have the strength to breast feed and you also don’t have food and you have come with nothing the baby is also crying because he needs something. You should leave the babies pampers, milk and leave them food, I also have children going to school. You also need to think about the fact that you have to pay rent at the end of the month, you also have to buy water daily, you also have to buy soap to wash clothes. What I can say about it is that it’s really tough. For especially those who have young children like me, it’s hard.

**I: How much can you use?**

PE03: The amount of money I can use per day… I have not put my highest limit; I can use 500/=.

**I: 500/=, is there any one remaining who have not given us her break down?**

ALL: [Silence}

**I: Is there any one remaining?**

PE05: Me.

**I: Oh number 5.**

## PE05: What I can say about that, on my side the amount of money I can use is 600/= because all my children take lunch at school, how they eat when they come back. The amount of money I can use is 600/=. If I talk about… about… [NAO: clears throat] … I I can’t remember.

**I: It is lost**

PE05: It has escaped my mind.

**I: Okay, we have all responded.**

All: Yes.

**I: Now, we want to talk about what we buy with our money once a day and their cost. What we buy ones a month and their costs? You say it is this and the price so that we move fast? PE06 what do you buy?**

PE06: I want to call it condom.

**I: What you buy once a week and how much is it?**

PE06; Condom

**I: How much is it?**

PE06: 100.

**I: PE07**

PE07: Ones a week?

**I: Yes.**

PE07: Once a week… I can buy… as PE07 said, condom

**I: Condom?**

PE07: Yes.

**I: How much?**

PE07: 50 shillings.

**I: 50 shillings**

PE07: I can buy condoms because we use them a lot.

**I: Hmm…**

P…: Protection and pampers.

**I: Totals**

P..: 200.

**I: 200, PE10?**

PE010: I just buy pampers once a week.

**I: For how much?**

PE010: 220

**I: 220, PE05?**

PE05: I buy condoms because it helps us a lot.

**I: For how much.**

PE05: For 100.

**I: 100, PE04?**

PE04: Once a week if I have money in my M-pesa I load airtime enough for one week. That is for about 300.

**I: PE03.**

PE03: What I can buy once a week is pampers and condoms.

**I: How much in total?**

PE03: Added together it can reach 300/=.

**I: PE02**

PE02: What I can buy is condom and pampers because I have a baby, total is 320/=

**I: PE01**

PE01: Condoms, airtime, those are the things I buy every day, and I have to eat

**I: You have gotten it.**

PE01: I have to ea.

**I: I have realized that the others are fasting.**

PE01: I love eating, I have to eat.

**I: What do you budget for every week?**

PE01: In a week

**I: Once**

PE01: Once a week.

**I: Yes.**

PE01: It’s about 1500/=

**I: Okay.**

PE01: I have to eat

I: 1500/=, now we want to look at what you spend money on everything. How much do you on what you buy monthly? Lets begin from this side, PE01.

PE01: Once a month?

**I: Yes.**

PE01: Pass.

**I: PE02**

PE02: I refill my gas cylinder.

**I: How much do you spend on it?**

PE02: 3000… 3450.

**I: That’s the big cylinder?**

PE02: 1250

**I: Okay PE03.**

PE03: Just as PE02 said its gas I refill monthly spending 1400

**I: Hmm…**

PE04: When I get my money, I once a month do major shopping in the household buying food, I can’t lie that I use the gas throughout the month I also use jiko. Sometimes I refill gas after 2 months, but I have to buy food.

**I: How much?**

PE04: Around 4000/=

**I: 4000, PE05**

PE05: I also consider buying food stuff more, because I don’t have a gas cooker, I use charcoal. So, if I one day do my shopping, I do for one month for around 4000

PE010: On my side things like gas or and other items in the house can cos 5000

**I: That is PE10**

PE09: Rent, shopping in the house, child’s clothes I love buying them monthly the total I can use is about 10000/=.

**I: 10000 that is PE09. PE08? What do you buy once in a month?**

PE08:Every month I pay my rent monthly, that is something that is a must, I pay 3000 for my rent. I must have flour; I buy monthly after buying 10kgs and keeping there, I am sure that even if things are tough, I will just take kales from the stalls and my family will eat. I normally take 10kg and keep there. Right now, it cost over 700 shillings. That is when I can buy salad [cooking oil] for about 300 and keep, buy a big packet of salt, I buy sugar also enough for one month. In a month, the way there is inflation I also think its about 10000. I normally make sure that I have those items in a month.

**I: That is PE08**

PE07: I do shopping monthly, I refill my gas cylinder, another important thing I do is to make my hair a bit that is when I will be smart and that is when I will get customers with this job of ours. You can’t just go there when you are not tidy. You have to be smart, that is what I feel, I use around 10000/=

**I: 10,000, okay we are on you PE03**

PE06: It is hard.

**I: PE06.**

PE06: It is hard, because you have rent, school fees and ALSO shopping you also have to renew your body every month.

**I: Renew in what way?**

PE06: You have to beautify yourself, buy yourself some fashionable clothes and where it, it can even reach 10000/= because fees alone is 4000/= or 3000/= and also shopping.

**I: Okay, PE01.**

PE01: on my side if I refill gas using 3400/= I can use it for 2 months, I also have to take care of my body I have to consider which clothes I am wearing, I don’t want to put on one dress this weekend and wear it also the next weekend, food stuff is also there, I also must do shopping for a month. Calculating roughly… after refilling the gas, I also have a child in secondary school. I don’t have to pay… whatever small amount I get if I collect… because my child is in boarding school, his fees is above 50, 000. I can’t pay it once, so I pay it bit by bit, so if monthly shopping reaches around 15000, I also have to keep 5000/= for school fees the lowest… for the school fees. That is my budget. It’s around 25000, no, 15000/=

**I: Okay, now we want us to look at the expenses we do less than monthly… less than one, how many times, I could be once a year, once per school term and the estimated cost. You buy it and its less than monthly, it’s not for one month. What kind of things are they, what you buy that are less than one month? It’s something you buy once in a month but its less than one month.**

PE01: I must have sugar in the house, I must have sugar, and maize flour that can push me toward the end month or I didn’t get it?

**I: Ah... it’s just that way**

PE01: I must have sugar because you can come from outside, some milk, flour, I must have them. I have to retain my friendship with the stall’s person, because at the end of about 2 weeks I must go so that she gives me something. I have to take onions, tomatoes and such things. If I do a rough calculation it can be 3000/=.

**I: 3000, another person.**

PE09: Milk…

**I: PE09, you are forgetting your numbers.**

PE09: I must have milk, sugar, vegetables from the stalls, condoms the totals amount to 15000/=

**I: Do you buy these things daily or after how long?**

PE09: Milk after 2 weeks, protection after one week because I don’t buy many pieces, food stuff I buy daily around 2000/=

**I: Okay, another person.**

PE010: I buy rice and milk and keep even if in case I lack maize flour, provided I have sugar, rice and milk I will just cook and we eat. This one might cost 1000/=

**I: Another one.**

PE04: As number 4 when clients are not there it is different, maybe I didn’t do enough shopping the previous month. So the basic needs like sugar, milk I have to add some in between

**I: For how much?**

PE04: It can cost around 2000/=

**I: Another person**

PE07: I love buying milk because sometimes you go outside and things don’t work out, you will come back so that you can cook rice and tea if you have and take. I love rice and sugar. I roughly use 2000/=.

**I: You have said that you can go outside then things get hard, what does this mean?**

PE07: You went outside to look for money but you came back with no nothing. You have not earned anything, so the rice and sugar you bought is what you will use.

PE06: Generally, it’s an issue of splitting, before a month roughly I do a shopping of 2000/=.

**I: Is there any one remaining, PE08?**

PE08: Thank you, there are things that we must look for, us as women every month. Women attend monthly periods, it is a must that we buy always sanitary towels every month. It is a must that we buy always so that we can keep them. We buy and keep them, also as women sometime there are things, we can say that you get… If God blesses you with good money in that month. One thing that hits the mind of a woman is something she can keep in stock. For example, I like stocking omena, rice, dried fish one day if you go to work and comeback with nothing, you find somewhere you can pick things. A woman is a wise person, it’s in a month that if God blesses you and you are able to get it in that month, you use it that way so that you find a way of going through. So, anytime I get such monies I can even use 5000/= because I want to get the stocks and keep. So, I will just sit and relax. So, these moneys that can go to waste you can keep some in your Mpesa.

**I: Okay, is there any one remaining? There is none, is that right?**

All: Yes.

**I: So, I thank you so much for the responses that you have given, now I want us to look at the things that you use, things that you spend on like a sex worker. Where do most sex workers get the money they spend? PE04.**

PE04: On my side, most of the money I use come from this job.

**I: Which job?**

PE04: My job as a sex worker.

**I: PE08**

PE08: Thank you, my job of having sex with men. These monies I get them from the pockets of men. They give me so that I can spend them. Men pay me.( NAO: participant chuckles)

**I: Thank you, PE010?**

PE010: When I went to do my sex work job, what I am paid is what I spend.

**I: Another person PE07**

PE07: The money I spend come from men’s pockets when I do sex work. So, my money come from men.

**I: PE02**

PE02: The money that I use… I must have sex with someone because that is the job that I do. So it depends on the time.

**I: Time in what way?**

PE02: He can have sex with you for one hours, or 2 hours or till morning. Sometimes also due to the fact that you are at a risk you can charge 10000/= without trust (condoms). You know you are at a risk, if someone wants to give you 1000/= and you don’t have money you will just have sex with him. So he must have sex with me for me to have this money.

**I: Another one, PE05.**

PE05: I support PE02, the inflation is high that if you get someone who can pay you 10000/=, I will just give him and after he is through, I will wash it clean, then begin doing other things. That is how it is.

**I: That is how it is, who is remaining? PE01?**

PE01: I just exchange… PE07 just said, I exchange sex for money.

**I: PE03.**

PE03: How does Kiswahili put it, kuma kwa kitanda pesa mkononi( NAO: somebody gives you money before you engage in sex)

All: [Laughs]

**I: Clarify what you just said?**

All: [everyone continues with the laughter]

**I: Elaborate on what you just said, we want to hear it in dholuo.**

PE03: This means that somebody first gives me money before we have sex on the bed. You will not also give me a small amount because you can be given something that is not even enough for your fare back to my house. You pay me as I wish. If you have sex with me let’s say first round, I want may be 1000/= in my hands. The smallest amount if things are not right, I should be given is 500 so that my children can take tea. I support that issue that way.

**I: You have said it well, you have said the jobs that you do, your main source of income is that right.**

All: Ye.

**I: Apart from sex work which other job do we do apart from this one? PE05**

PE05: On my side I don’t do any other job, its only that.

I: PE010:

PE010: That is the only business that I know.

**I: PE09**

PE09: Even me that is what I know.

**I: PE08**

PE08: Depending on my age my friends, right now they bite me slowly. I also sell dried fish.

All: [Laughing]

**I: Wait first, you said they do what to you, they bite you slowly, meaning what?**

PE08: They have to treat me well ,not engage in vigorous sex.

**I: They take you slowly.**

PE08: When I am doing this, I also sell dried fish. These my people know it and they are my customers and the also buy the dried fish so that I may prepare for them. Then again, I tell them, “Just give me tomorrow I will see how to deal with you,’ They give me and I give them the dried fish. And when someone sends something to me through my phone and tells me, “Come at a certain place.” I will go. I will take whatever I get there then I come back.

**I: Okay, PE07.**

PE07: I have tried leaving but I am not able, I try leaving but I come back. I have even tried working as a salonist but still come back. My friends I can’t leave this is the job that I know.

**I: That is the job you know? Okay PE06.**

PE06: I used to sell second hand clothes but it didn’t do well. The sun hits your head and it turns bald than you will just say, “these are just wasting my time.’ I left.(NAO: stopped selling mitumba)

**I: What did you do?**

PE06: It wastes your time but the things are not moving, the sun also shines on you, the governor also wants taxes from you that you have to pay, you also have to eat but you have not sold even a cent. I will go and transact a business that is cash at hand.

**I: PE01**

PE01: I also support number PE06 because it’s a job, I am a addicted and I am not able to stop. At the same time I sell clothes and sometimes you can give someone clothes and you want to go and restock the following Saturday. You will not be able to. But with this one you just go and after you are through you just come back immediately, so, that is the job that I do.

**I: PE02, is there any other thing that you do other than what we are discussing?**

PE02: That is what I majorly do, apart from when I am called, I can wash clothes, dishes for someone

**I: PE03.**

PE03: the same as she has said it, but I depend on the other job. I used to wash for people but it was difficult /hard to me but when you are from there that if you go for sex work, your body aches that you don’t even want someone to touch you and you know that is where we eat from.(sex work)

**I: Thank you so much, now, we want to know this, most of you have said that the main job you do is sex work, how much do you earn from sex work?**

P..: Per day?

**I: The whole month, you just give the rough estimate because you can’t say the exact amount, PE010**

PE010: For myself let’s say that I go today and I fail to get. May be the following day I get only one shot and earn 200/= and that day ends. Let me say that in a month I can earn even about 12000/=.

**I: 12000/=, PE09**

PE09: This is not something that you go daily and you are able to get daily. Sometimes you go, sometimes you do… but you are not paid

**I: What do they do?**

PE09: They have sex with you but you are not paid, sometimes you are paid an amount that you didn’t agree on. So, if I can say it’s around 10000/=-15000/=

PE08: It’s hard, the reason why it’s hard, especially mid-month, at this stage you have to be tough.

**I: You must be tough in what way?**

PE08: This is the point that if you are not careful, this is where you will be told, “come let’s go in, do fast am giving you,” if you are not careful you will go with nothing. Then he tells you, “I am going to send you through Mpesa just right now.” He will be gone and you have nowhere to find it to ask for the repayment of the debt. My friend that is what makes things hard in the middle of the week, that’s when we practice give me, I give you. Give me before I give you, he gives it to you so that you keep it in your pockets first, because you might leave with nothing because he has nothing and he has the desire. That is the most difficult part, this job is normally good during end months, but very hard in the middle of the month. What we can make in a month is around 15-16 thousand.

**I: PE07:**

PE07: Right now, it’s hard. I have said it’s hard because the inflation rate is so high. Men are also crying, “I don’t have… I don’t have… I only have a small amount; can you take the little that I have.” You will just have to take because you have left children back at home, rent is also waiting for you. So, you will just have to take the little that he offers you.

**I: The small amount can be how much?**

PE07: Sometimes you might go and he tells you that he has nothing and pays you about 500/= you will just take the 500/=. Sometimes you go and someone tells you that, “I will send you through the Mpesa. You look at your phone waiting for an alert but it doesn’t come ‘’ng’ony onge gowi’’ meaning in sex work you receive money first then you offer the services.… is no… so we find it rough. So sometimes it can be 15000, that is where you get your rent and food.

**I: PE06.**

PE06: That thing is hard, these people nowadays lie to us.

**I: Who lies to you?**

PE06: These men, when you go you may go to someone’s husband you don’t know if he will pay you or he will not, but with you, you are insisting that you have to get. Sometimes you might go but someone just tells you, “I don’t have money.” Sometimes you might bargain the price and you are longing that he tells you the price that you have at heart but he doesn’t. You wish that he gives you 500/= and go back home then if one day God gives you . And you earn 2000/= I will sleep that day I will go nowhere. It can be roughly 15000/=

**I: 15000/=**

P..: For me roughly… it is hard… I have sex over the phone. On my side I feel it’s a bit easy though it’s hard because you can do it for him over the phone but he fails to pay you. Sometimes you do it for him and he pays you, don’t be surprised.

**I: I am not surprised; I just want to ask you something.**

P…: With the phone can I tell you what I do?

**I: Yes, that is what I wanted to know.**

P..: I can remove my clothes and remain naked, if he tells me to remove this side, I do what… I put. ( demonstrates the way she does it)He also removes his from the other side. So, mostly I deal with white people. For me if its bad, because after doing the video show, he can send me 10000. [in audible segment] 5 the people I use their house I can even give them 1000/=. For me if I work that way, if I do my calculations per month is 30000.

**I: 30000, is there anyone remaining, PE05**

PE05: Talking about the issue, the issue of having sex, sometime you can have sex and then you agree well with someone that he is going to send you money. He then sends, not that he doesn’t send. But after making 10 steps from you he does a reversal of the money. You remain with nothing and you have been fucked, you can’t push someone to pay you for sex, it’s a problem. How will you begin calling him to ask for your money, will you say, “Send me the money you were supposed to pay me for sex?” that is Impossible, that will be gone just that way, you will just leave it that way.

**I: In a month how much can you make?**

PE05: In a month maybe, you get 3 or 4 people who does the same thing to you. One week will have gone, in a month the least I can get is 7000/=.

**I: Okay is someone remaining PE04.**

PE04: Just as PE01 said, she reminded me of something I had done as she said. I worked just as she said. That person washed me [didn’t pay] over the phone I tried once but I was not paid. Because he is a white man you met on Facebook, immediately you get to messenger and exchange numbers and you begin doing these things on video call just that way. I tried once but I was not paid. So I just go to the local places and if I calculate it totals to around 15000.

**I: PE03, have you given us your response?**

PE03: I have talked.

**I: You had talked.**

Pe08: Let me repeat for them, I have realized that these people are still young. After becoming a sex worker be a sex worker because that is where you earn your money. Never again remove your clothes and somebody sees your inner wear before you see the money. Tell him, “No,” just tell him, “Just go, because I am struggling with life so much that you should not even try me, bring money.” Bargain with him because that is the business you earn from and take care of your children. Bargain with him so that you can reach an agreement first, he can tell you, “I don’t have this, I only have 400/=, I don’t have the 800/= you are seeking,” “Add something and make it even 600.” You just agree, “Okay I increasing it to 550.” “Give me the money.” Let him give you your money, if it is at night if you are close to your friend leave her the money. Ask, “How long are we going to take.” That is something you have to agree on that is why you are a sex worker. How long are we going to take? After agreeing go with him and check the time, let him not tell you that he is not done time will be over. That is why you are a sex worker. your time is over, that is it that is what I want to teach these my children, I have realized that the still young sex workers.

**I: PE010:**

PE010: On issues of the vagina you must use time because there is no way we can agree that we are going to have a shot and we are done. The shot should have some minutes may be 10 minutes. This is what you have to consider.

P…: Shot is twa twa and you get 400.

PE010: Yes, you just soak and leave [laughing]

**I: Wait, you are allowed to laugh but still we need to hear what she is saying. You said that you just soak, how is it soaked?**

PE010: After we agreed on 10 minutes and you still have the desire for money after the minutes you will be forced to add me more money. If you can’t add me I just leave at that . It’s good that way because you have already pocketed your money.

**I: We are through with that or is there any one that want to add something? There is none?**

All: Yes.

**I: We are asking, what makes female sex workers…, sorry… we already talked about that I almost repeated it. We are looking at it this way… it’s okay… we are looking at a question, what makes female sex workers buy what they buy. We are asking, what are the typical reasons why female sex workers spend on what they do? PE01**

PE01: Protection, I don’t leave make up, I use it to beautify a lot. Protection, when I am going to work, I need protection.

**I: So why do you buy protection?**

PE01: I buy protection to protect myself,

**I: How much do you spend?**

PE01: 100/=

**I: 100, another person what you buy and the reason you buy it? PE07**

PE07: What I buy first that I must have in my bad that even if you look inside my bag you will see is a condom, that is something I am not ashamed of. You have to buy a condom; I have to protect myself because you don’t know the persons HIV status. Maybe you are going to do sex work and you know your HIV status maybe you are HIV negative but you don’t know the status of the person you are going to meet. So, you must have the protection. That is the number one thing that you must have, walk with it in your bag because you never know at what time you will be called. You must have it in your bag, you might reach there then you are told that, “I don’t have” You ask him, “Do you have condom?” He says, “I don’t have condoms.” You can just give him the one you carried and ask him to use it.

**I: How much can you spend on condoms.**

PE07: I can spend 100/=

**I: 100, another person**

PE04: Sometimes I am called, sometimes I am contacted through the phone. Sometimes I am with somebody who [in audible segment] [too much noise on the back ground like a posho mill] I have to load credit. That is the first one, I also have to use protection roughly I spend 200/=.

**I: Another person**

PE09: We must have protection., we have sex with different people so you must have protection. You must find a way of…. That child that you left at home you need to push on with him. Don’t just go like… “I am a sex worker, I just go.” Look for ways of taking care of your child. So, you must have protection [condom]

**I: PE08**

PE08: Sometimes I use a lot of protection, but it reaches a time I also use PrEP when things are tough, I also use PrEP

**I: How much do you spend?**

PE08: I spend around 300/=

**I: Is there anyone with an addition different from what the others have said? There is none. Those you have bought for yourself, what do you buy for others? That you buy…. You said that you people have children?**

All: Yes.

**I: What are the things that you can buy even for your child and how much can they cost PE010**

PE010: This out job is tough, if at all I get some money even if it’s just 2000, I can tell myself, let me go and buy my children some clothes this can cost me even 2000. I have 3 children

**I: Another person?**

PE09: I have to buy my child some clothes, toys, food and that can cost me 15

**I: It can…?**

PE09: Cost 15

**I: 15 what?**

PE09: 1500/=

**I: Another person, PE02.**

PE02: After coming from work and when I get some time during the weekend I take my children for outing or even to the museum, you buy the a soda or chips and they eat, clothes, shoes. I must make my children enjoy as a single mother.

**I: How much can you use roughly?**

PE02: 3000/=

**I: 3000/=, another person, PE08**

PE08: I handle my house depending on how I earn money. If I don’t get money, I push the way I feel like , but If I get money, I can buy my children appropriate things at the appropriate time. I handle my house depending on my earning. I don’t put maximum amount I use my money the way I get them.

**I: Anyone remaining.**

PE01..: After working hard I have to tell my children sorry because they are the ones who open the door for me when I am back. I don’t have a house help, its them that have to open for me the door when I come back. A day when I have money, I take them to swim in that process I can buy them chips. When I am with them in the house, I can change for them the diet, I have to change the diet.

**I: How do you change the diet?**

PE01 Roughly I can spend 5000

**I: 5000/=**

PE01..: that’s the total, at least I give them something that they think that mom is doing what she can do.

**I: Hmm… We are moving to another question, do female sex workers save? Do you have savings PE08**

PE08: Where we are and how we have been operating, we have not done that. We have not even had the thought that we should save. But I save small amounts in my phone. In fact that it’s how I saved and began the dried fish business.

**I: Yes, so you save,**

PE08: For me I save

**I: Another person, PE06**

PE06: When I go out I make sure I save for school fee and rent, I just save in my phone.

**I: Another person,**

PE04: I have to save for emergency, sometimes my child can get sick. I have to save for emergency.

**I: PE09**

PE09: I also save in my phone.

**I: Who is remaining? Those who are remaining do you have savings? Do you have, you may keep it in your phone or anywhere and its money that you are sure you can get when you need it.**

PE10..: On my side what I can say is that when I get my money from chama [group]when I get it , it helps me, that is what I can say.

**I: How many times do you save? This money that you save, for how long do you keep it, is it something you do daily or how is it?**

PE01..: After one month.

**I: One month, PE04.**

PE04: It depends on the condition of the job, sometimes you go but there is no job I have to do my calculation the amount of money am going to use in a day. [background noise from the posho mill]. I can say that I can save… I cannot save daily.

**I: How many times do you save?**

PE04: In a month?

**I: Yes.**

PE04: In a month I can save for 10 days

**I: Okay PE010**

PE010: On my side it forces me to save daily, you can’t skip to pay chama, you have to pay. When I am from my work, I will use some money but it forces me to keep100/= so that if at all I don’t get some money that day then I can pay chama the following day, so, its daily.

**I: daily, PE09**

PE09: as number 9 I save weekly

**I: Weekly, PE07**

PE07: I can save weekly, and save it in a chama. When I get my chama money I pay my child's school fees, it can also help me in doing some shopping, I save weekly.

**I: PE06**

PE06: I save weekly.

**I: You also save weekly; it means that those who are silent do not save?**

PE02..: I save monthly

P..: For me the needs are too much for me and I can’t save. I depend on earning when someone have sex with me. I don’t have savings. Unless I save in Mpesa but again if I save in Mpesa I use it to buy Credit. So I don’t have savings.

**I: PE03**

PE03: You can’t gain from the money you earn from sex work, its money that goes easily, its money you get, sometimes you save it and may be when you don’t get client you will use what you have saved. I can’t lie that I am saving money. If I had a chama that is where can save and rely on the money.

**I: Are you a member of a chama?**

PE03: I have.

**I: That is a means of saving.**

PE03: Yes, that is what I can talk of.

**I: So, you save, there are people who said that the save daily some weekly is that true?**

P..: Some monthly it is just that I don't have an account

**I: Okay, are there specific characteristics of female sex workers who save, some character. Like specific characteristics that you can use to identify this sex worker is saving?**

PE01: There some that if you look at them, you just know this one is saving. There are some that if you look at you just know that now this one is not saving. There is a way you have to look at her.

**I: I want you to tell me what you have to see in her you will know that this one is saving and this other one is not saving?**

PE01: If I look around there are people that do this our job that you see them and just know. They are drunk throughout. She is always drunk, such a person, even if she gets 20 shillings, she will use it to buy alcohol. If you just look at her… sometimes you find her sleeping by the roadside drunk, can such a person save, she can’t save. Is she someone who can’t save?she is not saving.

**I: Another one, which is someone who can’t save. Let’s talk first about that person whom can’t save then we get back to someone who can save.**

PE09: On character of someone who is not saving her children don’t go to school. You are with the children at home, they don’t go to school because of school fees.

**I: Another one, if there is a character you can identify her with, if there is none we can move on. PE07**

PE07: They don’t take care of their children well, the children are always hungry and when they see you eating, they come running to you. That is someone who don’t save because instead of saving so that she can take good care of the children the children are not taken good care of. That is a sign of someone who is not saving.

**I: PE04**

PE04: Someone who is not saving is someone if I visit your house as a fellow sex worker… maybe I am come after some period of time but realizes that there is no development maybe you have bought nothing. Maybe you had a 24 inches TV you can upgrade to 43 inches. If you don’t have any small developments it shows that you are not saving and thus moving backward you are not progressing.

**I: There is a question I was forgetting to ask; how much do you save for those who save weekly. How much do you save, and for those who save daily how much do you save?**

PE010: Myself I save 100/=

**I: 100**

PE010: Even if I earn 500/= I have to save 100/=.

PE05: Monthly I save 200/=

**I: 200 that is PE05, PE09**

PE09: I save 350 weekly.

**I: PE04:**

PE04: On my side it depends when things are hard, I skip when things are good at work I can even save 30/= or 200/= so it depends on the job situation.

**I: Mostly how much do you save?**

PE04: Mostly I try so that I don’t save less than 200/=

**I: Another person, those who are saving, how much do you save?**

P..: In a week I can save… no, per day I save 150/= so that I can save over one thousand and above in a week so that I can pay my rent at the end of the month.

PE07: Weekly I save 500/=.

**I: 500, so we are moving on to the characteristics of those who save, how are they? How will you know that somebody is saving, PE06?**

PE06: If you want to know that someone is saving among those who do our job,( sex work) if you see the way… for example if you go to her house you find that she has furnished the house with different things. If you look at the children where they are walking you can say, “this woman with the job she does is taking good care of her children.” Even if you see the way she dresses, sex workers believe in wearing designers’ clothes. If she sees one, she will say next time I have to do this, that is someone who is saving.

**I: Another person.**

PE01…: Someone who saves if you look at her, she is educating some children some she has taken to college. These children are getting quality education and they live well. If you go in to her house you will say, “Its true this my peer is living well.”

**I: Is there anyone who want to add?**

PE04: I want to add, someone who saves as I have heard… for example I am a daughter in our home I have to think of how my mother is living, where does she get help, how does she dress. You know right now she is advanced in age and cannot go to work. So, she depends on me and live here in town but I have not told her what I do. I am just a sex worker but sometimes I lie to her that I am doing a small business. So I have to think of her, I can’t forget what is back there at home . You have to take care of the parents you have left back there, help them and even build them a house, that is someone who saves.

**1: These people who save, what kind of people are they, who are they? Who are they, who saves? PE01**

PE01: Those who are saving , I don’t think that its only us sex workers, other people also save in my view.

**I: Okay, let’s move to the next question that asks, those female sex workers who saves, what makes them to save? What are the reasons for saving? PE02**

PE02: For better future

**I: For better future.**

PE02: You can even buy a piece of land, you can educate your children and even if you don’t educate your children, you can educate even a cousin’s child who lacks school fees.

**I: Another one, reasons why we save, PE04**

PE04: Just as my peer has said, better future and for an emergency in case because you are a single mother and you never know. PE010

PE010: Like for me who have a small baby he can get sick yet I have no savings, it will force me to save bit by bit so that if he becomes sick then I can take her to the hospital.

**I: Another person, why do you save, PE08**

PE08: I save because of my house. I want to do the things that I have planned that maybe next month or next year I want to do this thing or the other one. After I determine in my heart what I want to do, I just begin saving. So, I save so that I can do good things at the right time in my house. Right now, I am saving because my child is studying at the polytechnic in =Mawego=. So, you have to save bit by bit then send it to him, jus that way.

**I: Another person PE09.**

PE09: I save so that my child can have a good future, then again, we should not forget that we also have parents and they depend on us. So, you save so that if your mother asks you for some shopping she can get. It should not be a situation where every day you are saying, “I don’t have, I don’t have’ then what are you doing in town.

**I: Is there anyone who is adding, if there is none let’s proceed. What makes it easy for you to save? What makes saving easy.**

PE01: It is easy for me because when I get… when I have gone for sex work , whatever little I get I save somewhere

**I: Another person PE06**

PE06: It has to be easy because you don’t live in your own house, you have to pay rent, pay school fees and you have to eat. You have to save to survive.

**I: Another person PE010**

PE010: Even if people at your home have money, they can’t help you do everything. you have to say to yourself, “let me save this money so that at the end of the month, I find a way of paying rent or even school fees.” So, it makes my work easy. Asking people for money every time is not good.

**I: Is there someone adding PE04**

PE04: It may reach a day that I am feeling tired I can say, “let me rest today.” I will just take the little that I had saved and use it to feed myself and the children”

**I: Are there challenges we may have when we are saving?**

PE01: You can have challenges, maybe in a week, I can go without earning, it can be a challenge to me because I will not save that week.

**I: Another person challenges that we have when we are saving, they are like which one?number PE04**

PE04: Just as PE01 said that is what I want to add because sometimes you might go but there are no clients so you will not have a means to save and feed your family.

PE09: Sometimes it’s hard because you may earn a small amount and use the whole of it, maybe you had planned to, sometime you might have an emergency like disease attack… and like I said that I save weekly, that week will end with me saving anything.

**I: Another person, what are the challenges that we have when saving.**

PE07: I might have some challenge when a relative die, you will not save because it’s expensive to manage a funeral. Maybe you are given something to buy, you may not be able to even pay the chama. You will be forced to attend the funeral first. That is another area I get challenged.

**I: Is any one adding, there is none?**

All: Yes.

**I: Out of the challenges that you have said how can we overcome them?**

PE06: Its by good luck that God has made a way in that Jitegemee has come to help us overcome these things. That organization, you have come, you already know that these people have responsibilities. You have also known that it’s not the desire of the sex workers, we are big women. We are not children; we will now depend on jitegemee because you have come to help us.

**I: Number PE09**

PE09: Now that Jitegemee has come it should help us so that we can start some business. You can do your business, you know even if I will not get a client, the business will pay me something. If you gave someone some credit you are sure that in the evening you will pick the money and do something with it.

I: **I: Number PE10**

PE010: On my side I have longed so much to start a business, but I am not able. So if this organization can help me so that I can get a way of starting my business I can stop doing this job, my age is advancing.

**I: PE02**

PE02: To support PE06, God has seen our cry and the way we have been struggling outside there and we are single mothers. Now that this organization has come, don’t just interview us only today, just proceed this that even if you can give every individual 50,000 so that we can start some business then we will leave this job. So, we will depend on the money you will give us and we will save and our jobs will continue

**I: Is there anyone with an addition?**

PE01: Jitegemee I can’t say that I am thanking you, at least as female sex workers we would like it if you open our eyes. Right now I am getting old very soon I will be having grandchildren. If I can move a step ahead, I come out of where I have been, move forward and find something I can do. Its better if I have a business I can do, I can then save from this business if I leave this other one. I should move forward only if you stand with us, and open our eyes as sex workers from different places.

**I: Thank you for the responses that you have given, for the female sex workers who do not save, why do you think they don’t save?**

P..: You already asked that.

**I: No, for women who do not save why are they not saving?**

P: They have a lot of burden.

**I: They are overburdened, another one, just say you are the one talking.**

PE03..: You can come back and the money you are coming with and the budget you have… currently its hard… more so cooking oil is very very expensive, the price of maize flour has also been increased. You know for you to save is really going to be hard.

**I: Another one?**

PE09: Sometimes you are from work and you meet thieves, they beat and steal your money. What will you save that day? Maybe you are in a chama and they took all your money, it will force you to borrow money and still have debts,

**I: Another person, PE05.**

PE05: Talking about saving, even today I came back with 500/=. When I came back the children began dealing with the 500/=. They are three children and I gave then 150/= for lunch. 150/= is for lunch but didn’t have soap, didn’t have sugar, cooking oil. It is finished, I still don’t have what we will eat for supper. So I can’t save.

**I: Is there any one with addition, is there any bad thing that can happen if we don’t have savings?**

PE07..: Failing to pay school fees.

**I: Failing to pay fee.**

PE10..: And rent, and also food that you eat those are the bad things you can get.

**I: Another one.**

PE10: A child can be… your child can be attacked by a disease and you may have nothing to do.

**I: PE04**

PE04: You can be called because of an emergency at home, maybe your mother is sick and you are to give something. In your house you have children that is the only problem.

**I: What other bad thing can we have if we are not saving?**

P.E04.: Maybe you are called from =Luanda= and you are supposed to use a vehicle to the place and you already finished the money you had, how will you go? You will miss to get that client.

**I: Is there anyone who is adding PE08**

PE08:I feel that if you are not saving, an emergency can come up, if that happens there is nowhere you can dip your hands. This is why it’s good to save, if you don’t save you will have nowhere to turn to if you have an emergency that comes abruptly. You can also get sick abruptly, and there is nowhere you can fall back to, how will you reach the hospital. That is the disadvantage is someone is not saving. A bad emergency can come yet you don’t have anywhere to fall back to.

**I: Anyone with an addition, PE09**

PE09: If you don’t save it’s easy to be thrown out of your house, you will be harassed. You may say that during end month organizing well with your clients for example us who do it online, you have planned with your customer and may be the deal gets spoilt. Now that you didn’t save will you not be thrown from the house.

**I: Okay, what are the benefits of saving, benefits of saving?**

PE01: If I save, my children will go to school and I will comfortably pay school feed, I will pay the rent well, I will eat well my children will clothe well.

**I: PE02 if you save what will happen, what benefits will you have?**

PE02: My life will be good, my children will eat, my parents will live well, I will be able to do so many things.

**I: Many things for example?**

PE02: If I save for so long, I can even buy a plot, I can even have my own car.

**I: PE04**

PE04: If I save sometimes, I can say that, “Let, me enjoy myself. Let me relax” Even for my mom… I may have some savings that I have accumulated for may be one year. My mom’s house maybe is mud walled, I can say, “Mom I want to plaster you house so that you are able to mop just like other women are mopping.” That is the benefit of saving.

**I: PE05, what are the benefits of savings?( noise from the poshmill)**

PE05: I can only support what the others have said. If you are saving you will be okay, nothing worries you. You have the money in your phone if anything comes up you just decide, “let me withdraw this amount to do this thing, I will get money are return.” If you don’t have, what will you do, that is a benefit of saving.

**I: PE010 what are the benefits of saving?**

PE010: The benefit of saving is that is at all I go to work and fail to get some money; I will withdraw the money and use for that particular day and when I get some money I just return.

**I: PE09**

PE09: The benefit of savings is that you can manage all the emergencies that come by. And then again you will have a voice. Maybe you are in a meeting, you have a voice, if they need some money you have a voice because you can do something.

**I: PE08**

PE08: there are so many benefits of saving is, when you are saving you can do all that you have planned to do as planned. When you are saving, for example myself, it is saving that made God to give me wisdom to start the business of dried fish with the money I had saved so that I could sell them. Before CORONA spoilt the businesses like that of dried fish, I was handling good money. However, when COVID came everything increased in price the business is also falling. But, I could rest from the business of walking in the cold, I could rest and I sat and you could find a customer coming to buy and I could get money in my hands just from my house. So, saving is good. Its only that currently businesses are not doing well with this COVID of yours. I am praying that I will again begin to save very soon.

**I: You have said that things have not been good for you because of our COVID>**

PE08: Our COVID, oh, it’s not yours it is ours, let me put it that way, you have opposed me. It is our COVID [laughing]

**I: Okay, PE03, what are the benefits of saving?**

PE03: Saving can help us in many ways, maybe you have a pressing issue somewhere, maybe you don’t have food, maybe lack of school fees, you child might fall sick or you can fall sick. So you can tell others, “Take my phone, go and withdraw some money so that we are sorted.” So saving is a good thing.

**I: Okay where do female sex workers save their money, where do you keep your saved money.**

PE06..: I save in my phone.

**I: In your phone**

PE04..: Mine sometimes when I am given money from chama, I will say, “I dint have this thing then I pay,or I have not pay my rent, let me pay.” Mostly I save in a chama.

**I: You save in a saving group? Another person?**

PE09: In my phone.

**I: In my phone that is PE09. Where do you save your money PE08?**

PE08: In my phone

**I: Phone, PE06**

PE06: Mshwari

PE07: Saving group (chama)

**I: PE04.**

PE04: I save in my phone and I also save at KCB bank

**I: Oh, also in KCB, where do you save?**

PE05…: In my phone.

**I: In your phone.**

PE03..: In a changa box

**I: Changa box, where do you keep your change box?**

PE03..: In my house under the bed

**I: In your house. Why do you prefer where you save? Why do you love saving in a change box?**

PE03..: The reason why I like saving in a change box, I want that immediately I enter the house I dip it there immediately because if I keep it in mpesa I will buy some credit. When I go somewhere and see some good thing, I will buy it using the saved money. With the other one you know when I leave the house, I will leave the change box at home because I can’t carry it.

**I: Those who said that they are saving in their phones, what are the benefits of saving in your phones.**

PE09: I save in the phone to avoid picking it… you know I will be seeing it there; I will just pick it from there after a short while.

PE06: When I save in the phone, I save in a locked saving account, so I can only withdraw after a specific period. So I like saving in the phone.

**I: PE07 you said that you are saving in your phone.**

PE07: I said that I save in a chama

**I: Chama sorry.**

PE07: I save in a chama because you may plan to have somethings, when you receive your money, will you not go straight to what you wanted. So, I save in a chama.

PE08: I save in my phone but, my phone has never lied to me, any time in get an emergency… I don’t have to look for fare to town, wait in a queue at the bank. No, I will just go straight to an agent and ask him, “help me withdraw 600, I want to give somebody just now,” I find it easy that is why I save in my phone.

**I: Lets proceed, of there is anyone with an addition?**

All: No.

**I: There is none?**

All: Yes

**I: Do female sex workers live beyond their means or their earning? You live a life that is more costly that your earnings, does that happen?**

All: Yes.

**I: Okay, elaborate, clarify**

All: [Silent]

**I: I want you to tell me how you live a life that is costly compared to your earning PE08?**

PE08: I live a more expensive live compared to my earning, why, immediately my child stepped in college . What was required and the amount that was needed, I just felt some headache that very day. Due to the fact that there is perseverance, I pushed hard till I ensured that the child joined college. However, the life is too high for me, I am just trying as a person. It forced us to look for HELB, it helped us in some areas. You still find that the life is above what you can manage. Even if you tried hard to make life manageable you still realize that it’s still hard and not manageable. This is because you want to divide your money for this thing, this one, this other one, you also want to divide it for the other thing. You will still find it hard even if you try. That is where it is hurting us.

**I: For example, we are saying that, you are using more money than you earn**

PE08: I use more than I earn and I even go into debts, even recently I went into debts, I took some Mshwari loan you have to take.

**I: Okay, another one**

P..: The life I live is more expensive than my earnings because, I am relied on at home. I am the bread winner of my children this side. That is why I like saving in KCB because sometimes I can request for a loan and they give me.

**I: Another one, is there any addition?**

All: No

**I: You have said why you do that is that right?**

All: Yes.

**I: How do female sex workers bridge the gap between the money they earn and the money they use if they you more money than what they earned? If at all I had money and I have used it, and it has not reached the level I wanted how do I fill the gap**

PE09: It now forces me to take a loan, I have to fuliza.

**I: You will be forced to Fuliza to fill the gap**

PE06..: Sometimes when you go to work you may get a good customer, may be the way you planned the job … you planned the job in a way that pleased him. So, even if you agreed on 500/= he may add you something on top of that. You can use the extra cash to bridge the gap

**I: You said that you can plan the job in a way that he is happy, what does that mean?**

PE01: You have opened the server [sexual engagement] well just as he expected, you have not limited him the way others do.

**I: How can it be limited?**

PE06:..: You don’t time him m, timing is not there.

PE01: If you don’t time him, you give him some freedom, he can turn you the way he wants, it will just increase

All: Yes [Laughs together].

**I: Okay, another one, I have only received responses from 2 people, how they bridge the gap.**

PE08: I told you that when it’s hard, like when you have planned with the money you have and it’s not enough,, it forced me even last Saturday I took mshwari loan. I took 2000/= to sort out a problem, anytime I get something I just repay. They gave me, you must look for a place to take a loan, so for me I borrowed from Mshwari, because I also save there.

**I: Is there any addition? Do female sex workers take loans or do they have money they have borrowed. Do they borrow from somewhere?**

PE08: There is.

**I: PE01.**

PE01: I have debts

**I: You have.**

PE01: Yes, I have for	food stuff, for the house, school fees

**I: Okay, PE08**

PE08: We have to take credit that is common with men, we take loans. I take credits a lot. I took a loan last Saturday.

**I: PE07**

PE07: I have taken loans that when I see a creditor coming, I just say,”Kipchoge” [runs away].

**I: What do you say?**

PE07: Kipchoge that is an athlete, you have to run away [laughing]. Nowadays people call me kipchoge. Even those who serve food in hotels.

PE01..: She even eats food in hotels then run away, kipchoge.

P..: You will run my friend.

**I: Do we have debts, tell me PE05 is laughing do you have something to say? PE010**

PE010: The life that has become so expensive is what makes us go into debts, someone pays me 500/= after engaging in sex with me for a short time. When I go back with the five hundred and calculate what I should be buying it’s not enough, I am forced to borrow some money

**I: PE04**

PE04: I even have debts for my rent. You have to be smart so that he doesn’t push you too much, but we have debts.

**I: Who are the people that you have their debts?**

P..: [mentions a name]

**I: Don’t mention her name, you can just say it as an example.**

PE01: I have a man’s debt

**I: A man’s, debt, how?**

PE01: If it becomes hard, I will give him the server [have sex with] so that he leaves me alone

**I: So, that is a man’s debt that you want to repay by giving him the server [having sex with him]**

PE01: It has defeated me and he is asking me to repay 2000, and I am not able to repay. I had already told him that we should have sex once in a while and reduce the amount bit by bit. The first time we did that and he reduced the amount. Right now, he is serious, I am forced to, he even told me that if it is not possible then I should go and live with him. But I can’t live with him [others laughing]

**I: Okay PE06**

PE06: Its hard sometimes you come from work with nothing, sometimes we sit telling stories and you see someone passing… some hoteliers walk around this place. You may just say that, “Let me eat ugali and chicken” and maybe you don’t have, money. Then she calls the hotel worker who asks, “What do you need,” “Ugali and chicken.” This girl can call three plates and they are brought without a problem. When now they come in the evening that is where Kipchoge now applies [laughs]. If she comes back the following day and ask, “where did you go.” We were called somewhere. Maybe 2 people are not there she finds only one person. The one person tell her that they had already paid the other people. When the other will be there and am not and maybe she comes back she will be asked, “You were not paid?” we left her. The day we will have money we will go there and pay saying, “we are sorry, we didn’t have, take your money, give us ugali and meat,”

**I: Where else do you borrow, who do you borrow from?**

PE08: I borrow from the shops, there is a shop called “Edu’s shop, anytime I lack money, I just go there and I am given, when I get money, I just take to him.

**I: You are through with that area is that right?**

All: Yes.

**I: What do we do with the debts we take?**

PE10..: I use them in my house

**I: Another person?**

PE10..: Just in my house

PE04.: I take debts when I have an emergency.

**I: PE03.**

PE03: I take debt to buy food in my household. Mostly for food but you can also need rent. The month maybe approaching the end but you don’t have money, like right now the month is nearing the end but I don’t have money, so, for rent and for food.

**I: Another one?**

PE08: I borrow to use in my house for the basic needs.

**I: PE09**

PE09: I borrow to pay the person who takes care of my child.

**I: What do sex workers to do to increase their earning. To increase your income what do you normally do? What do you do to increase your income PE05?**

PE05: Beautify yourself and dress well, that is what we do to earn more.

**I: Now, when I beautify and dress well, how can that increase my income?**

PE08: Customers will notice you.

PE05: That means that customers will see you, how you have beautified or how you are smartly dressed

**I: Another one how can I increase my income; how can I increase my income.**

PE08: I increase my income from what I charge, I harden my charges. So sometimes God helps you to get those people and they add you some money. So, the money increases a bit.

**I: You increase...?**

PE08: If I get… if I get a client who may be… if you look at him you can see that he has money. When you should charge 1000/= you will begin by charging 2500/=. So you can bargain to 2000/= and that is good

**I: Another one, [long silence] is there any addition. There is no addition? If the FSW fail to get clients, what do they do? If you don’t get a client what will you do?**

PE01: Maybe we had sex but he has not paid me.

**I: No, you don’t get him, what will you do?**

PE01: This where sometimes… when I stand… maybe I stood in the cold and failed to get a client. That where maybe you... some calls me [name of her son mentioned] mother. I can stand the way I am standing, if somebody passes, I will try and maybe he also tries, because you may get somebody. That you also get a partner and go with him. At the end you can go with him have sex but after that he can beat me and send me away without payment. He became violent, maybe I thought I had a partner but he was just a rogue. How can we call it, how can we put it?

**I: That is what I want you to tell me.**

PE01: Class help me, maybe I thought I got a client but I got a..

P: Street boy.

PE08: Terrorist

PE01: Terrorist.

**I: What I am asking is this, if I don’t get a client what do I do?**

PE06: If I go there and stand for long and tame is also running and there is no client, I decide to go back home.

PE08: You can go and fail to get a client; it will force you to come back home with nothing that is what now makes take the loans we were discussing. Because now I am back and I don’t have other means, it will force me to look at how things will move. That is what now takes me to Edu’s shop, to the stall so that my people get the daily basic needs. That is what will take me to take credit because I didn’t get a client.

**I: Another one, if there is, if there is none we will proceed. How will you know that you will now not get a client, at what time?**

PE08: If it reaches 3 am before you meet someone.

**I: Which 3?**

PE08: At night. That day there is none, people will be going to sleep there is none.

**I: How much debts to you owe others yourself at any time. Everyone will tell me the debts that they can have at any time. PE01 how much debts can you have?**

PE01: In a day or in a week?

**I: No, debts that you have and people are seeking repayment from you, how much can it be?**

PE01: 15000/=

**I: 15000, PE02**

PE02: 17

**I: 17 what?**

PE02: 17000/=, school fees is there rent, food from the stalls, 17000/=

**I: PE03**

PE03: If I calculate the totals, it can be 12000/=

**I: 12000, PE04**

PE04: I have around 10000/=

**I: Around 10000/=, PE05**

PE05: I also have around 10000 because school fees and rent

**I: PE010**

PE010: What is disturbing me is that I only have 15000/=, pampers and milk take my money, I also have to pay the person who takes care of my child, if I add with rent and what I eat, 15 thousand

**I: 15 what?**

PE010: thousand

**I: PE09**

PE09: I think it’s about 7000/=

**I: 7000, PE08**

PE08: 31185/=

**I: PE06 how much do you owe others**

PE06: 10000/=

**I: 10000, We want to talk about sex, do those who have sex think of when they can stop doing sex work?**

PE09: It’s a thought that I have had so much, I have thought that can I change this thing that is why I went to try the dried fish business. Again, things became bad and the business is falling, it forces you to go back. It’s hard but I long to leave.

**I: PE010**

PE010: My daughter is now grown; I will be doing this and other people may tell her that you know your mother feeds you from this job. I have really longed to leave this thing. So if I can get a business that I can do, I can leave this [sex work] to that I go ahead with the other one.

**I: PE04**

PE04: Just like PE010 has said, those people [the children] are becoming big, they are growing to become adults I have also longed to do business, but the main problem I have is getting capital. If I could get some capital, I would be doing some business that I can at least rely on.

**I: PE06 do you have anything to say?**

PE06: Yes, when you are coming from your house you know you pray that God may lead you so that you make money just enough for the day for children food. When you go to the field [hot spot] you again don’t get anything, when you come back you again you will pray to God, “Can you remove this thing from me and give me something else I can do,” I have also longed to stop doing it.

**I: Is there anyone who has an addition? There is none. Is it something you discus with peers as in is it something you talk about among yourself?**

P…: When we meet at our place where we rent, we can now talk as ladies, you say, “for me the period I have done this job I just feel I should leave it, I don’t just have another source of money, I don’t have any means of survival.” That is how I feel.

**I: PE01:**

PE01: We normally sit as sex workers, we have sat and desired that we can get something else we can do, more so, personally I long to leave this job. But I don’t have capital. If I could have capital that can make me leave this thing, it can make me to focus on the business I will be doing.

**I: Any one with more information, is it something you discuss with your peers?**

PE08: We talk and share stories, but what meaningful thing that somebody wants to do is our main challenge. Everyone thinks of what she can do. What can she do?

**I: Now, what bring about such topics, what happens before we start these discussions?**

PE04: Challenges, maybe you have gone there, maybe you are new, there is something we call bullying. Those people bully you, maybe they mistreat you. These are the things that brings about those thoughts, if I had some capital, I would have left this job and begin some business.

**I: PE09**

PE09: As number 9 sometimes you go to the hot spot, and some member of the hot spot come, new members come, you know those people [client] will leave you, they will not see you. So, you will think, “why can’t We leave this job and do other things.”

**I: PE09**

PE09.: You go there and you are beaten, you come back at night you are alone on the road and thieves attack you

**I: PE07.**

PE07: The challenges that we have is that maybe one person has gone with a client the following day you get the same client, it causes fight at the hot spot..Those are some challenges that exist that can make you desire to leave the job.

**I: PE010**

PE010: Such a thing once happened to me, I went to the base and those older base members are left and you are called and you go with the client. When you come the following day, the older members start a fight with you, “why are you taking visitor and they are the people who have taken longer at the base

**I: It’s okay, what makes FSWs leave or may desire to leave sex work, reasons why we might want to leave are like which ones. PE05.**

PE05: Reasons why we can leave.

**I: First of all, when you are speaking, speak about yourself then we will talk about other people.**

PE05: The reason why I can leave is because of what we go through. Maybe you go to a person’s place, sometimes someone takes you to a place like =Luanda=( noise from posho mill) and leaves you there and you don’t have any means to come back. So, you begin thinking that if you can get a means of leaving the job you can just leave. Since there is no way we can leave it because we don’t have capital to start some business. That is why I can’t still leave it because if you leave it then what will you eat. And if we can get a way of doing business because the challenges, we are experiencing is not good.

**I: The reason why you may want to leave sex work, PE04**

PE04: The reason why I may want to leave this job is for example the way it’s currently raining; it’s raining and the place I live is also a bad place sometimes you are forced to wade in water. I also have children and that needs the attention of a woman. Like right now schools are opened, day time they are in school and at night I am not with them. I may desire to leave this job so that I can create time for my children.

**I: Another person who may want to leave This job and why? PE09?**

PE09: I may want to leave this job so that I can bond with my child because you live him with someone else every night, you don’t know what is done to him,

**I: Is there any addition, why you would want to leave this job?**

PE08: I am not so pleased with this job because in this job you meet big penises and deal with them, you meet small penises and deal with them, you meet fat penises and deal with them, you meet thin penises and deal with them. Why are you doing this, it’s because you have personal needs in your house. I long to leave because I am tired of accommodating these penises.

**I: PE010**

PE010: For me even the morning cold has made me sick, sometimes you get real muscles one that is more than you can handle. You will be forced not to wear an inner wear even for three days because [participants laughing] that is what I have seen and it makes me want to leave.

**I: Apart from what we have said about ourselves, we now want to talk about external factors that may make us want to leave this job, what are they? [too much noise from a grinding posho mill]**

PE09: Most sex workers die, sometimes somebody forces you to go with him without protection and you acquire HIV and maybe he hadn’t told you. You have the disease and maybe you are afraid to go for the medicine [ARVs]…

**I: You fear….**

PE09: Going to take ARVs and you have HIV.

**I: Another one?**

PE08: I want to talk about other things that we encounter, one day I was with somebody, he was a CID, we were already in the room, I told him, “Give me money.” He didn’t want to give out the money, “Why don’t you want to give me money? Give me money.” The money wasn’t coming forth. My friends we really struggled in that room, he wanted to tear apart my inner wear but he had not bought. When he held my innerwear, I held the waist of his. I told him, “If you tear mine, I will also tear yours because you have not given me any of your money and I will also not” he said, “I will shoot you.? I replied, “Try shooting me right now and you will pay.” We struggled there and he told me, “You are a stupid woman, you are a dog and many other insults.” I told him, “You are also a dog that is why you took a dog and brought her here, stupid.” It forced me to open the door, if he finds a weak person will he beat you or not?

P: You are beaten

PE08:, and he will give you a serious beating. You are beaten for a right that you were supposed to be given. We also meet such outside here, if you are weak you will be beaten a lot… these our people get beaten. They are beaten if they refuse to remove their inner wears before they are paid because some of them are weak. They are beaten and insulted about the sex work; these are things that we encounter that can make you want to leave sex work. Don’t think that we only have good experiences.

**I: Is there any addition, is there a time that you have planned that you are leaving this job?**

PE06: I plan every day.

**I: You plan daily.**

P.E06.: you plan, can God bring victory to me today, money should come in tit… tit… and you just know that you are not going. But that does not come. It will force you to go and arrange the stall then leave for sex work. You just have to plan that, “To day I am leaving this thing’ But you can’t leave, it will force you just to go.

**I: Another one**

PE07:The money that we get from this job may be is cursed. I have planned to leave and I even began a business but it keeps on failing.

**I: How many businesses?**

P..: Two and they just fail

**I: Another one? Have you planned if you leave this job?**

PE08: I have a plan, it just reduced. I had started selling dried fish. This is what I am glued to, I just tried it and I realized that its good because. Because when I have it in the house, what I will be doing display it and take it back into the house. And when I have time, I walk with it, “buy this… buy this.” When I come back, I display it. So, I have the plan. I started it so that I can leave this job. I just don’t have someone to boost me.

**I: Have you planned?**

PE08: Yes.

**I: To leave?**

PE08: Yes, I have a plan to leave and that is why I have these those things.

**I: Another person who have plans?**

PE05: On my side I have plans that if I can get money even today, I will not go there because even its raining a lot currently. The cold bites that you comeback so cold just like a corpse.

**I: Another person?**

P: Even if I leave it, I am already sick, I have chest problems. I just wanted to leave it forever so that even if I will continue treating my chest… it is the one that gave me this chest problem. I have longed to leave it and look for something else to do.

**I: Okay, FSWs leave this job at what age?**

PE08: Leaving this job is not determined by age, it depends on something else that you will be doing that will make you forget sex work. It doesn’t look at age.

**I: It is not determined by age.**

PE08: It’s not determined by age, its caused buy something else that you will be doing that will provide for and how you will be doing it so that you earn and leave sex work. It is not age determined.

**I: Okay, which types of jobs can sex workers do after leaving sex work, which jobs are they.**

PE07.: Business.

**I: Say it loudly**

PE07..: Business

**I: Another one, PE08**

PE08: These our people are still young some of them have done form four and they have certificates. I you can find places you can place them they can just leave this job because they are sure of earning and will not do sex work. There are some who attended some seminars and they have certificates, like myself I have a certificate from MAP international. If I can get somewhere to work and have some earning, will I again go anywhere else to look for anything? I will just be waiting hopefully that my salary is coming. Even these our people if they can get other jobs to do, not only business, even those who want to do business if they are boosted, they will just do business and you will just see their work as sex workers just end that way.

**I: Is there anything we can do apart from business, PE06**

PE06 :I want to request jitegemee. This organization we can also do research. You can put us somewhere to do it. We are educated, it’s not like we are not educated, we are educated.

**I: Another one, another one, I want us to talk so that we end this, we are remaining with one part. Is there anything you are adding of there is none?**

All: None.

**I: Now if you want to start doing this your business, where can you do it, where would you want to do it.**

PE08: My business like this on of selling dried fish, I will just do it within =Nyamasaria= I will just arrange them in a basin and hawk around with them as people buy them. They are the ones who buy them. I just hawk them within =Nyamasaria=

PE010: I would want to sell at the market just like other fellow women. I should be there and sell my wares there. Stall business selling vegetables.

**I: PE09**

PE09: I think market center is good

**I: PE07**

PE07: I am well known in =Nyamasaria=I have been insulted here, I wish to change and move to … how is it called=Busia =where people don’t know me. I can do some business there, i am known here.

**I: PE06 where would you want to do your business**

PE06: I will just go back to my space at the Korona market.

**I: CORONA.**

P: Same to me

**I: If I leave sex work will my life change?**

PE08: Very well.

**I: What changes**

PE08: Walking in the cold at night where mosquitoes bite me, I am rained on that ends. Many diseases will have to go. So, I will not have to treat myself every now and then. When I get an alternative thing to do I will have more money will come in different way from how it was coming in in bits the way it was happening in sex work. God can do me well and I may be able to eat better than when I was a sex worker, that means that my life will have changed. My life can change in different ways when I leave the job.

**I: Another one?**

P: My life can change because I am tired with treating infections, I will now rest from treating infections. I will be peaceful, even if I am selling clothes so that every day, I can get what to go back with.

**I: You have talked of treating infections, which type of infections**

PE01: You know when you are going to urinate, the toilets are used by so many people, after you are drunk you don’t even know what you are doing, you will just go and squat there. The following day that place will be so painful something also pulls you. It’s painful you also have pain in the lower abdomen.

**I: Another one, PE010**

PE010: I will be resting from the hammer [sexual intercourse], I will only do it when I want to maybe if I have a boyfriend. I will rest from the daily sex

**I: Is there a time you can leave sex work then later come back, you leave and come back?**

**P: It is impossible.**

PE06: You want to leave it… if you leave it, where will you get money. Who will provide for you when you leave it? You can leave it and begin asking for help from your brothers or uncles but you will not get, you will just come back. You want to leave it… you must have some money you have kept somewhere.

**I: Is that happening I can leave it.**

PE01: You can leave it.

**I: I can again come back.**

PE07: Yes, you can come back.

**I: What makes me to come back?**

PE07: Lack of money you fail to get things that you need.

**I: That is PE07, Another one.**

PE04: Sometimes the friends that you have matters, for example if I still have my old friends they can make me go back.

**I: Another person, another.**

P: They are finished

**I: They are finished, You have said that your friends can draw you back is that right, what bad things can happen to you if you leave and then come back.**

PE01: If I was doing my business and may be its falling and I am not getting money and I am still there, I can say “Hey! This thing that I was told to do, why is it not working well for me.” I can get stuck and get back to what I was doing. That makes me go back but I was not to go back...

**I: Now, you have gone back, what bad things can happen to you if you had left and went back. What kind of things can happen to you?**

PE06: What can happen is that maybe I leave the job and get employed may be you works for somebody in a hotel. You will be given too much work, you go early and comeback late and still you are not paid on time. You will say “the job that I was doing I can go even for only one hour and I have 200 shillings, I rather go back to that my job.” Those are the kind of things that make people come back to sex work.

**I: After coming back what bad things can happen to me when I go back?**

P: When I go back there

**I: Yes. The bad things that may happen to you, are like which ones?**

PE06: What can happens is for example when I have taken long and go back there and find new people, you are again bullied and such things. It will take you time to be used to the trends again.

**I: Another one, any bad thing that can happen to you if you leave and go back?**

PE07: You may have left swearing to yourself that you will never do the job again, and you go back to the job. You can even die because of the job.

**I: Another one, is there any other one**

PE08: The peers that you left… you know there are word people throw to others, when they see you have come back, you will receive insults.

**I: Is there any good thing that can come up when you go back?**

PE08: There is no good things, because if it wasn’t there when you left where will it come from when you come back? You will just receive the small amounts you were receiving .

**I: Okay, what can FSWs, we already talked about that, the jobs that we can do, if that true**

P: Yes.

**I: Do you know those who have left sex work between the past 5-10 years? Is there anyone you know who have left this job.**

P: I know.

**I: Who are they?**

PE01: The ones that we know or PE01.

**I: The ones you know who have left this job in the last 5-10 years.**

PE08: There are some that have left, they are married and even have…

**I: Okay**

PE10: There is one that we were working with in 2016 she left and bought a piece of land and built a home in =Siaya= because he was now old enough so she left.

**I: She left.**

PE10: She relocated from =Kisumu=.

**I: Another person who knows someone who has left.**

PE08: There is one that was also working in a bar here in =Nyamasaria=, she decided to leave, god married and she even has a child, she gave birth.

**I: Another person, PE01.**

PE01: I have seen one who left and currently she is living in her home in =Uyoma=,

**I: PE02**

PE02: I have seen one who left after educating her children. After the children succeeded they completed school and graduated. She left. Her children are now working and can give her money.

**I: Another one PE04**

PE04: I have someone at home, I call her my mother. She left, we thought she would not live, she is alive to date. She lives well.

**I: PE03, have you seen one who left.**

PE03: I have seen one that left, she lives well.

**I: Another person who have seen a person who left.**

PE09: I have seen a friend who left and is currently married.

**I: The question that I said we have talked about, we have not talked about it. It asks are there things you want to do before you leave this job. Things you want to accomplish. You are saying, “I want to do this job, but after getting what I want then I will leave it.” Have you had such a thought?**

PE06: On my side I don’t have a dream to have a husband to live with. I would like to leave it because I have already built a home. I bought land and built a house because I have a son, when he grows up I want him to get a place to build.

**I: Another one**

PE08: I have also been concerned about the land issues, that if God can help me. That I add my things and my money to purchase land then I will be satisfied and the issue of sex work will end at that stage.

**I: Another person who has something she wants to do before leaving this thing?**

PE02: I want to leave this thing after educating my children

**I: Another one. The person who want to leave what does she want to do before leaving?. Have you began working towards getting what you want to get?**

PE08: We are struggling to do it

**I: You are struggling.**

PE08: Those are the things I am struggling with so much.

**I: What are you doing?**

PE08: That is why you can see I am trying to expand what I do for example sell dried fish so that if I add what I get here with what I get the other side I may see what I can save. It’s again hard to save because we again have school fees, this is one thing that again stops me from saving. I am struggling but still I am not seeing any progress. Again you cannot let the child not to go to school after he had begun. That you are just thinking about the land and the child is stuck. It forces me that whatever little I get…. If I get something small…. If will force me to again begin planning for the future after he is through with that level of education.

**I: PE04 you said that there is something you would want to do, have you began planning?**

PE04: I am planning but it delays because I am the bread winner. So when I am trying to save with these people, sometimes I feel that the more I save with them, they may give me some loan.

**I: Okay, you said that there are people who have left this job that you know, is that true?**

All: Yes.

**I: What made it easy for them to leave, what made it easy for them?**

P: The one who was my friend, the daughter gave birth after getting married. These children grew here in =Nyamasaria= and she found a husband, so this job was causing shame to her and she told the mother to go home so that you can be built a house so that you can leave this job. That is why she decided to leave.

**I: Another one, what made it easy for them to leave the job?**

PE08: That little girl just decide on her own because the man she met loved her and he was serious. He told her, “No, this thing that you are doing I don’t want you to continue doing it, I want you to decide to marry me.” She also loved the boy, she decided and she left and got married to the young man and they are together to date.

**I: Another one.**

PE01: There is a girl friends of mine, she knew she was working and she said, “welcome customer.” When she said, “welcome customer” by bad luck it’s the son who took her. After saying welcome customer and the way she was dressed, when the son looked at her, the woman fainted when she fainted her son thought bad about the mother . When the child knew that the mother work was…. That is how the woman went and her life deteriorated that way, she went back home and she died. Welcome customer, she didn’t know she was telling her son she didn’t know it was her son . People also meet such things outside there.

**I: So that is a challenge someone can encounter, how can we overcome such a challenge?**

P: I feel that if we can unite as sex workers, if we put our brains together and say that this is what we want to do. If we put our minds together that even if we are doing this job, one day we will have to go back to our houses, we have to reduce or leave the job forever. We should have a stand… because it’s true everything has its end because I will not do this job for life, I will have to leave it. So, when I leave it I should have an alternative thing to do. I will go back home and live there, we will leave this job.

**I: The challenges we are talking about are like the challenge where a mother told the child, Welcome customer, how can we deal with the challenge, what can we do to stop this thing from happening?**

PE01 You never know, because I am standing the way I am standing[ demonstrating how she stood] , you know when I am standing I can turn this side, may be do this way, is that okay, you know when I am facing this side I may not know who I am dealing with. Because you can come when you are passing, you know sometimes it could be your husband who may stop and lower the car windows and immediately you realize it’s your husband, you can either run away, or you just sit there and put on a stun face.

**I: We are almost through, only a few questions remaining, what can we do to avoid these?**

PE08: Thank you, because it’s your source of income, the only way we can avoid these things is when we get some support from any other members or people [ interruption ] who can give us support for us to come from there and seat here and wait. It forces that if we get support that is when you can decide, “where we meet with different people you find even your child in front of you, your husband or brother in law comes to you, let me leave this job so that I can do what is appropriate for me to do the job that I have been supported to do,” so you fully focus on it and leave sex work. But if you still don’t have an alternative there is no way you can come from this side [sex work]..

**I: Okay, initially I told you about Jitegemee study and I told you that it’s being done so that FSWs saves their money that can make them say no to sex without condoms, or to take a rest from transactional sex when they wish. I also said that it is about FSWs saving part of their own money to use when there are no clients or to help them prepare for life after sex work. What I am saying is that Jitegemee is about you saving your money personally. You save it and if it reaches a time that you need it you can withdraw it. When you don’t have something to save a must you must not save. If you want to withdraw a small amount you can withdraw. If you want to withdraw the whole amount you can withdraw, there are no restrictions and no interest the money will gain. Is Jitegemee something that sex workers in Kenya may like?**

PE08: We may like it, we can like it because it is what we are doing we save and become independent.

**I: Another one will they like it**

PE010: I like it.

**I: You like it, another person**

Pe06: We like Jitegemee because it is recognizing us.

**I: We are asking this way, what type of female sex workers will accept jitegemee? What type of FSWs will accept Jitegemee**

PE04: women who have longed to quit the job.

PE05: I support he.

**I: You support her, what did she say?**

PE05: You may want to leave the job.

**I: So those who want to leave are the ones who can support Jitegemee?**

PE01: Mmh

**I: Yes? Let me repeat the question, I am asking this way, as in… you have said that the FSWs will accept Jitegemee is that right**

All: Yes.

**I: Which type of FSWs will accept Jitegemee**

PE07: Those who can accept jitegemee are those who has gone through difficult situations in the sex work job.

PE08: Those who can accept such a thing are people are people who have been taught who have heard about it the way we are hearing and decide to implement those are the people who can accept. Before you are taught how will you know that its good to be independent so that you can leave the thing [sex work] and come this side. So, only those who are taught.

**I: Are there people who will not accept Jitegemee?**

PE08: Yes, we will get some who will not accept because they may also have a feeling that in a way sex work is helping them. So, there are some who may not like it.

**I: In jitegemee we are not telling you to leave sex work, you will just be doing your job and be saving.**

PE08: Some may like it some may not.

**I: Those who will not like it what are the reasons?**

P..: There is a difference because for us you have taught us, some people have not been taught that means that they don’t know about Jitegemee. You have to teach them for them to determine why you want them to leave sex work, for those who have not been taught. It’s easy to leave for us, you can’t know about Jitegemee before you are taught about it.

**I: You have 10 friends who are FSWs and you tell them about Jitegemee How many of them will accept Jitegemee, how many will accept PE06.**

PE06: [Silent]

**I: You have told them about Jitegemee and they are 10, how many will accept Jitegemee?**

P: People perceive things differently.

**I: No, I know people are different, but how many of them will accept**

PE06: One out of the 10

**I: One out of 10, PE07**

PE07: Due to the information you have provided to us, even if we didn’t like it we now like it because we are here. It means that all the 10 will accept.

**I: What about you?**

PE08: If we sit them down and teach them the way you have taught us then they can accept.

**I: How many can accept.**

PE08: All of them can agree just as we have all agreed here.

PE010: there is no way you can refuse something before you are taught. You have to be taught first before you are aware what you are going into. I feel that all of us can agree,

**I: All of them, PE05**

PE05: I think that is I talk to 10 six of them will agree

**I: 6, PE04**

PE04: I feel that even if we leave the ten that was taken even other friends who do this job that I do, many of them have longed to leave this job even if you leave, I feel that all of them may agree.

**I: They can all agree, for PE06, you said that only one can agree is that true?**

PE06: Yes.

**I: The remaining 9, why will they not agree with Jitegemee?**

PE06: It depends on individuals’ hearts, maybe there are some people who concentrate in that work and maybe they earn a lot of money daily. If you tell her to leave sex work and begin a new job through Jitegemee she will feel that sitting idling will consume much of her time.

**I: It’s okay, PE05 also said that 6 people can agree, what makes the four not to agree?**

PE05: You know if I have 10 people and they think differently, I feel that if they are 10 people and I explain to them about Jitegemee six of them might agree.

**I: Why will the four not agree?**

PE06: You must find some people who are not easy to convince.

**I: Okay, what do you think we should do for people to accept Jitegemee, for many of those who do sex work to accept it? What should we do?**

PE08: Just walking around as we tell people about Jitegemee now that we have heard about it. So, we should tell our peers and explain it to them the good thing that we have heard and see if they can also join

**I: Another one.**

PE010: It forces us to tell them first as we explain the merits and its demerits for them to agree

**I: Now, we are talking about us as Jitegemee staff, what should we do for sex workers to agree with the idea of Jitegemee? We are almost through talk so that we can disperse we are remaining with just a few questions. If you keep quiet will also just wait for you.**

PE07: I feel they can accept the idea for example just like you have come right now and you give to us some business, and may be someone realizes that it has developed you in a good way. It will be so easy for them to agree. Depending on what you will have done to us.

**I: Another one, PE04.**

PE04: I support PE07, if jitegemee considers us as sex workers and maybe gives us capital for a start, our peers will see it is a good thing.

**I: Another one, if there is any, there is none. How can we do Jitegemee in a way that it becomes a good thing.**

PE06: Just like you have taught us in =Nyamasaria= maybe you have agents, you look for one in =Nyamasaria=. The agents are the ones who will be teaching people when you have told us when you are coming then the agent can tell us in advance what will happen and then you come.

**I: Another one.**

PE08: Adding on what PE06 said, first of all we have to get agents from amongst us after that then we gather women then call you are the women are taught. We then should look for ways of launching for FSWs within =Nyamasaria= area as from =buoye=, =Molem=, up to= Kasawino=, all those places. Then we do launch then we are taught while we are together. It can make it become better.

**I: Another one is anyone having an addition?**

P: No.

**I: There is none, what things should Jitegemee have so that people can accept it?**

P: In audible.

**I: Just say it loudly, PE05 you said something, what should it have for people to accept it?**

PE05: What you should have for people to see that it is good, is that you should give us a bit of support that we can use to do business so even if you tell someone else they will quickly accept it, She may say, “call me when they are coming next time.”

**I: Another one PE08**

PE08: Thank you, I also support my peer that have just talked, she didn’t talk so badly but I feel if Jitegemee can boost those they have begun talking to so that other can see what they are doing. This can make us to begin to talk with others so that if they see a change then they will also join so that the changes can spread wider.

**I: How should we boost them?**

PE08: Boost them even in Business, give jobs to others, you see you will have benefits so much so that even when you are talking to others, they will just see the good things that are there,

**I: What the reasons that you feel that there is need that we give you financial support.**

PE08: I feel it should be so because for a truth what we get can’t meet all that we need. Because amongst us there are those who pay school fees, there are those who deal with the needs in their houses, she is the one who pays the rent. So, you realize that the little she earns is not enough. But if she gets support it will give her strength that when she is doing something, she will be strong. And when she talks with another peer saying, “Stop doing this thing [sex work] come this side so that we can join hands with these people. You will be registered and then we will do this thing together.” You will now find it so easy to talk.

**I: Okay, what can FSWs like in Jitegemee, what can you like in Jitegemee as we had read and what we said we will do?**

PE08: The first thing I like about jitegemee is the fact that they have taught us deeply. The main thing is the deep teachings that have fully entered into my mind. And again, they have come together with us when we are having bitter hearts, as they look at how we can come from where we are and move to a different better position. I have liked that with Jitegemee

**I: Any other thing that that you have liked about Jitegemee, PE04**

PE04: What I have liked about Jitegemee is the way we as sex workers we have not known these things. Now that Jitegemee has taught us [in audible segment] [coughs]

**I: Who has an addition?**

P: I want to add on what I liked; I like even the teacher they have brought us.

**I: Which teacher?**

P: You, depending on what you have taught us, it is good, they have found a good teacher.

**I: What else do you like, we are almost through, we are only remaining with three questions and we are through. We are done with that portion is that okay?**

P: Yes.

**I: What will they not like about Jitegemee?**

PE06: What we don’t like about Jitegemee organization, they will seek our time and we will be meeting like today, then they will recognize us as sex worker. We will… if it’s so we will leave.

**I: Another thing that they will not like? PE08**

PE08: What I have seen that sex workers will not like most of them have children, can’t you see that all those who had children are not with us here. We are taking too much time. We should move fast; you see others have gone to take care of their children and they have left us here.

**I: We are talking about Jitegemee when we will start it. What they will not like about Jitegemee.**

PE08: Time, we have to reconsider time.

**I: Yes we have to reconsider time it’s only that, the thing we are doing we can’t say it in one minute. Have you realized that I am trying to make us move fast? So for time, it’s just this one that has taken long, but in future we will take less time.**

All: Yes.

**I: Is there anything that they will not like?**

All: There is none.

**I: Now, Are there things in Jitegemee that FSWs think will infringe with their right in any way?**

PE08: There is none.

**I: There is none, PE05:**

PE05: None

**I: PE010**

PE010: None.

**I: PE04**

PE04: None

**I: PE02.**

PE02: None

**I: PE01.**

PE01. None

**I: PE06.**

PE06: None.

**I: PE07**

PE07: None.

**I: We are asking those who have joined this way the Jitegemee program do you think it willaffect your rights in any way? PE010.**

PE010: None.

**I: None, PE03.**

PE03: None.

**I: Okay, what challenges can we have when we want to begin, what challenges can we get as Jitegemee. The challenges we may have when we want to begin Jitegemee, they are like which ones? We want to start Jitegemee is that true?**

All: Mhhmm…

**I: What challenges can we face?**

P: I was used to going there for one hour and I already earned some good money. For this one to make one hundred that I save daily, it may take me even a full day.

**I: PE06.**

PE06: Another one is that for sex workers during the day we are the true us but during the night we’re different. If you tell someone that sex workers are needed to participate in a study there, that word “sex workers.

**I: The word sex workers stigmatization. Another one? Any other challenge we may have. We want to start Jitegemee, what challenges can we have?**

PE04: The challenge we may have is that you may not have that impressive voice to do business. May be you were used to getting money daily, when you are just starting a business, you still don’t have customers you know you will have so many challenges.

**I: Challenges will be many, is there any challenge we will face as people who are implementing Jitegemee when we will be starting it. Is there any? There is none.**

All: Yes.

**I: Now the challenge that you have talked about stigma that we should not call you sex workers, how can we deal with it? What can we do?**

P: What you can do [in audible]

**I: Like, she said that the challenge we can have is if we come and we want to meet you that we should not call you sex workers, it will be like we are stigmatizing you?**

All: Yes.

**I: What can we do to avoid stigma as Jitegemee what should we do to avoid the stigma?**

PE08: We had been given numbers as those who are seated here, we know each other through numbers, and PE06 can meet PE03 and tell her to tell PE05 or PE04 that people are meeting at a particular place. And if you meet PE01 you will tell her to inform PE08 and PE09[ participants clapping for number 8] that people should meet somewhere because they are needed. So you just use the numbers.

**I: Just number, it’s only the numbers that should be heard.**

PE08: The numbers should be used because we already know each other using the numbers.

**I: Another one, this sister said that, she is used to money that come after a short while but more. But now she will seat there and only earn 100, how can we overcome that challenge?**

PE08: It’s like this class was mine, I feel this way, due to the fact that you long to leave the other side though you are earning more from there and this side you are earning 100 and you were not used to 100.You need to know how to budget with the 100 and get used to it for your heart to come out of the other place totally. But if you still want to look the other side and you are thinking that this other one is small, the way you think this one is small even the bigger amount will reduce and become small Get used to the 100 and budget with it and it will be enough.

**I: Okay, we are now asking almost in the last question, we are asking, how much you would wish to save weekly without affecting you budget for basic commodities that you must use like how much can you save in a week?**

PE06: 700

**I: 700, you.**

PE08: 350.

**I: That is PE08**

PE09: 500

**I: PE010 how much can you save?**

PE010: daily 100 that means that it is 700 weekly.

**I: PE05**

PE05: 500

**I: 500 PE04**

PE04: I take 200 daily, I can save between 600-800 weekly.

**I: PE03, how much can you save?**

PE03: Daily I can save 50 that is 350 in a week.

**I: 350, PE02**

PE02: 1000

**I: 1000, let’s say you have placed a target of saving 700, 350,500, 700 out of the money you have said , if you can’t meet the target, what can you do to achieve the targe?**

P: If you don’t reach your target?

**I: Yes if you don’t reach your target, what will you do to reach the target?number 9.**

PE09: I will reduce spending on my daily needs.

**I: You will reduce?**

PE09: Yes.

**I: How?**

PE09: For example if I was buying 1 box of milk, I will be forced to buy half a box in order to save the remaining amount.

**I: So that you save the remaining amount. Another one? What will you do if you are not in a position to meet your target?**

PE10: Only if I reduce.

**I: What will you reduce?**

PE10: My budget.

**I: You reduce your budget, another one.**

PE04: I can try… may be the work has reduced, I can try hard to lift my job a bit higher for example by being smart so that you can get good customers. Even if I am paid 500 and I get 2 clients.

**R: You said you will do what to your job?**

PE04: I make the job.

**I: How do you make the job?**

PE04: You become smart and wear a business face, you should not put on a very serious face, you can even smile with a client. The way you also talk to the customer also matters.

**I: Another person, what can you do?**

PE08: If I have less money there is nothing you can do about it but you can try hard to squeeze you needs so that you can get the money. I should save 350 but I have 300 I have to look for the 50 from an alternative so that you get the right amount you are supposed to save.

**I: I want you to tell me about the alternative.**

PE08: I will have to reduce the needs in my house that if I was to buy 1kg of sugar, I will not by the 1kg but will buy ¼ kg or half.

**I: Okay, where do you think is the best place for us to save our money that you feel you trust and it is safe.**

Pe07: Bank.

**I: Bank**

PE08: Mpesa.

**I: Mpesa**

P: Bank

**I: Bank.**

PE10: For me I have never saved where people save, I get my money from the saving group.

**I: You can also just say that because it’s not a must for you to save in a bank you have heard each person saying what they like.**

PE10: Saving group

**I: PE05**

PE05: Phone

**I: Phone, PE04.**

PE04: Bank and Mshwari

P: Phone, through

P: Mpesa.

**I: Mpesa.**

P: Bank.

**I: Bank, PE04 you have said Bank and Mshwari choose one saving site that you prefer the most**

PE04: Bank

**I: My fellows that is the end of the questions we were asking. Is there anyone with something concerning what we have talked about?**

PE08: Now I want to talk while standing because things are hard, it’s always exciting towards the end

**I: It’s exciting towards the end.**

PE08. Thank you for the teachings today, first of all from the people who visited us here on behalf of Jitegemee. What we want to tell you or the question we want to ask you, these teachings that you brought us, what target did you have with them that you have for us as sex workers. What do you think what will you do to us or what are your plans for us. When you come with such good teaching you come because you have a target with us. These is the biggest question I am asking, when you came, and the target you have for us they you can see we are struggling with penises, someone puts you with a big penis, another a small one, I wasn’t you to look at that for me, let the others also ask their own questions.

**I: Is there anyone with a different question?**

P: I wanted to ask the questionPE08 asked.

**I: The one PE08 has asked, is there any other questions?**

PE08: Question?

**I: If there is a question they can just ask, and if there is no question, can I answer that one?**

PE10: : Answer.

**I: Okay, when you want to start anything you must have a road map. You can’t just set off on a journey to Nairobi but you don’t know the route to =Nairobi=. You must sketch the map and know which route you will take from =Kisumu=.**

P*: Nairobi road.

**I: =Nairobi= road, I will take the route to= Kachok= not the route to= Dunga=. What we are doing currently is this, we are just collecting your views about Jitegemee. Any time you want to do anything you must consider how many people will agree with what you are doing. If I talked to 3000 people but only 10 agreed is that something I will come with to people to implement.**

P: No.

**I: We will not bring it, but if I do it with 3000 people and they all agree, that means it a problem every one faces and we need to look for a way of solving this their problem. Is there any other question? Is there any questions.**

PE01: I have a question, I read this paper when I came, and there is a place there I don’t know if you saw it. Pager number 1, I read somewhere here, will I be given anything for participating in this study.

**I: Let me ask you, Is that your question? I want to ask you this way**

PE10: Remind them even if they know

**I: We said clearly that there is something we are going to give you.. Let us complete then we will pay you they you sign to show that we have paid you okay.**

P: That is good.

**I: You have gotten the teachings well is it?**

P: Yes.

**I: Thank you so much…..[interrupted by participant]**

PE08: I still have a small question, this organization you have come with, can you assistance our children or may be jobs. Some are here that it is not proper for them to do it. Can you take them in so that you work with them?.

**I: That is a question I may not be able to answer you for now.**

PE08: When you go back tell the m that we have asked the question.

**I: Okay, those are the questions we had for you. Thank you so much for taking your time to participate in today’s FGD and for giving us your thoughts about Jitegemee. I want to thank you so much.**

All: Thank you.

**I: And the interview has ended at 13:4.**

**END**
